# Functional screening identifies aryl hydrocarbon receptor as suppressor of lung cancer metastasis

**DOI:** 10.1038/s41389-020-00286-8

**Published:** 2020-11-19

**Authors:** Silke Nothdurft, Clotilde Thumser-Henner, Frank Breitenbücher, Ross A. Okimoto, Madeleine Dorsch, Christiane A. Opitz, Ahmed Sadik, Charlotte Esser, Michael Hölzel, Saurabh Asthana, Jan Forster, Daniela Beisser, Sophie Kalmbach, Barbara M. Grüner, Trever G. Bivona, Alexander Schramm, Martin Schuler

**Affiliations:** 1 Laboratory of Molecular Oncology, Department of Medical Oncology, West German Cancer Center, University Hospital Essen, University Duisburg-Essen, Essen, Germany; 2 Department of Medicine, University of California, San Francisco, CA USA; 3 Helen Diller Family Comprehensive Cancer Center, University of California, San Francisco, CA USA; 4 Laboratory of Molecular Tumor Pathology, Department of Medical Oncology, West German Cancer Center, University Hospital Essen, Essen, Germany; 5 DKTK Brain Cancer Metabolism Group, German Cancer Research Center (DKFZ), Heidelberg, Germany; 6 Neurology Clinic and National Center for Tumor Diseases, University Hospital of Heidelberg, Heidelberg, Germany; 7 Faculty of Bioscience, Heidelberg University, Heidelberg, Germany; 8 IUF-Leibniz Research Institute for Environmental Medicine, Düsseldorf, Germany; 9 Institute of Experimental Oncology, University Hospital Bonn, University of Bonn, Bonn, Germany; 10 German Cancer Consortium (DKTK), Partner Site University Hospital Essen, Essen, Germany; 11 German Cancer Research Center (DKFZ), Heidelberg, Germany; 12 Department of Biodiversity, University Duisburg-Essen, Essen, Germany

**Keywords:** Non-small-cell lung cancer, Cancer models

## Abstract

Lung cancer mortality largely results from metastasis. Despite curative surgery many patients with early-stage non-small cell lung cancer ultimately succumb to metastatic relapse. Current risk reduction strategies based on cytotoxic chemotherapy and radiation have only modest activity. Against this background, we functionally screened for novel metastasis modulators using a barcoded shRNA library and an orthotopic lung cancer model. We identified aryl hydrocarbon receptor (*AHR*), a sensor of xenobiotic chemicals and transcription factor, as suppressor of lung cancer metastasis. Knockdown of endogenous *AHR* induces epithelial–mesenchymal transition signatures, increases invasiveness of lung cancer cells in vitro and metastasis formation in vivo. Low intratumoral *AHR* expression associates with inferior outcome of patients with resected lung adenocarcinomas. Mechanistically, AHR triggers ATF4 signaling and represses matrix metalloproteinase activity, both counteracting metastatic programs. These findings link the xenobiotic defense system with control of lung cancer progression. AHR-regulated pathways are promising targets for innovative anti-metastatic strategies.

## Introduction

Lung cancer is the leading cancer fatality at a global level. Despite major improvements in the treatment of patients with advanced lung cancers, the highest impact on reduction of mortality can be expected from prevention and early detection programs. While smoking prevention is of utmost importance, patients already exposed to cigarette smoke require alternative strategies. Moreover, avoiding environmental exposure to carcinogenic particles, volatile substances, and xenobiotic chemicals is much more challenging, and its role in lung cancer prevention is less understood. Recently, lung cancer screening by low-dose CT scanning was shown to be effective for preventing deaths in high-risk populations^1,2^. With the imminent implementation of national screening programs an increase in lung cancers detected at early stages that are amenable to curative surgery is expected. While this will significantly contribute to reduction of lung cancer mortality, metastatic relapse will still occur in many of these patients. Current strategies for risk reduction of systemic relapse following lung cancer surgery have evolved by translating platinum-based chemotherapy from the metastatic setting to earlier disease stages^3–5^. In locally advanced lung cancer this is complemented by radiation therapy to prevent local relapse in high-risk anatomic regions^6,7^. Despite being clinically efficacious the overall impact of these approaches is modest. More recently, durvalumab, an immune checkpoint inhibitor, was shown to prevent relapse in some patients treated with curatively intended chemoradiotherapy^8^. However, its role in resected lung cancer currently is unknown. Hence, there is a high medical need for novel strategies to prevent metastatic relapse in lung cancer.

Molecular dissection of the complexities of the metastatic process can provide the basis for such approaches. From current understanding many steps are required to license tumor cells for survival and growth at distant sites, including local invasion, epithelial–mesenchymal transition (EMT), anchorage-independent survival, and adaptation to a foreign environment^9^. Still, only a handful of genes have been found causally involved in metastasis, and the role of their products may change during the course of the disease. A prime example is TGF-β, which is thought to exert tumor-suppressive activity in premalignant cells. However, in established tumors, TGF-β signaling and crosstalk with WNT, PI3K/AKT, and EGFR/RAS pathways is linked to aggressive phenotypes and metastasis^10^.

The identification of functionally relevant metastasis genes has been challenging due to the paucity of relevant preclinical models. Toward this end we have developed and validated an orthotopic murine lung cancer model which faithfully reproduces the metastatic spread that is observed in lung cancer patients^11^. With this in vivo model we undertook an unbiased functional genomics screen and identified aryl hydrocarbon receptor (AHR) as suppressor of metastatic spread in EGFR-driven lung cancer. Mechanistically, AHR counteracts metastasis formation by repressing EMT programs including matrix metalloproteinase activity and regulation of metabolic stress responses involving ATF4 and ASNS. Our findings suggest that modulation of AHR activity is a promising strategy to interfere with metastatic progression of lung cancer.

## Results

### An unbiased shRNA screen in an orthotopic mouse model of lung cancer reveals metastasis genes

**Fig. 1:**
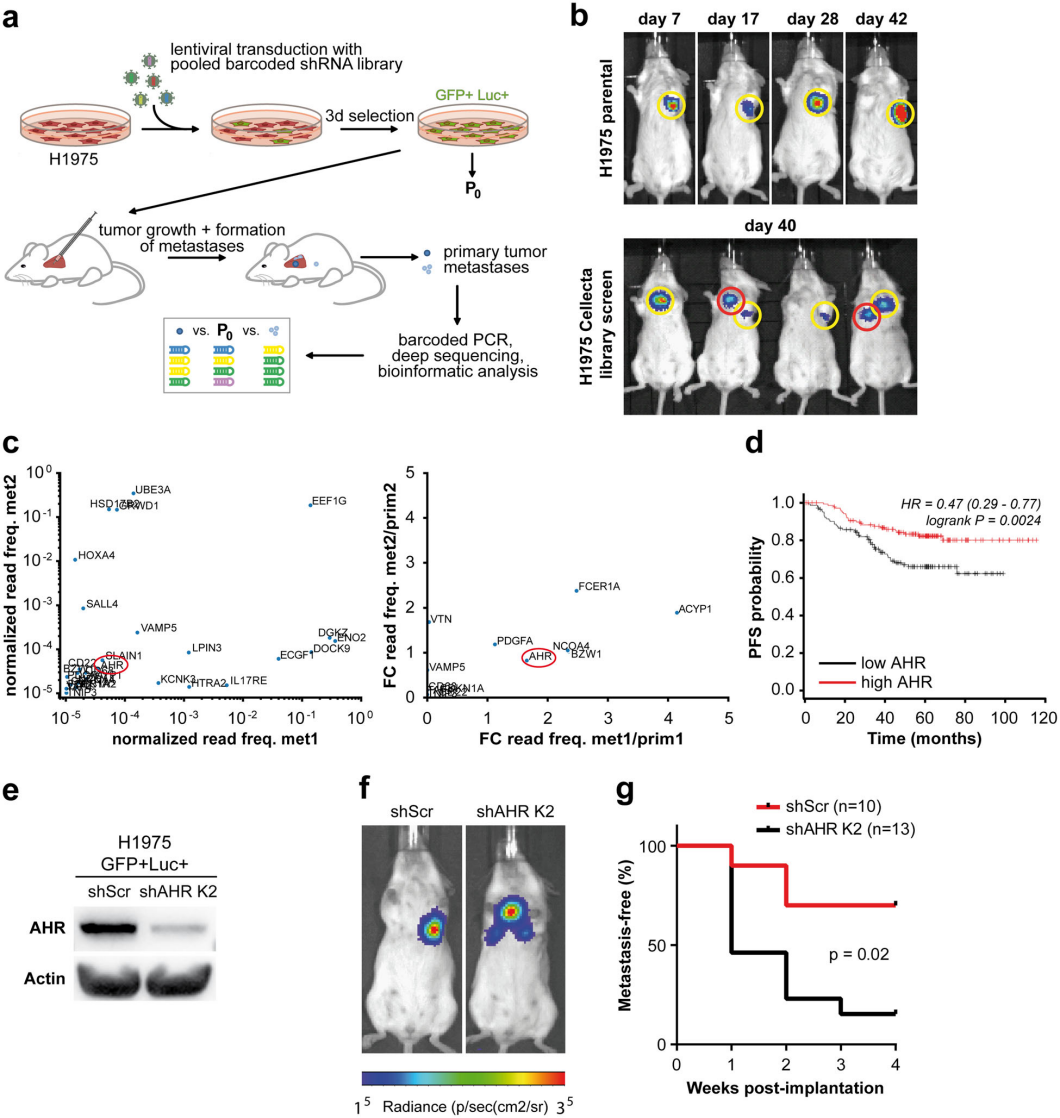
AHR functions as a suppressor of lung cancer metastasis in vivo and correlates with progression of early-stage lung cancer in resected patients. **a** Schematic overview of the experimental layout of an shRNA-library screen to uncover metastases-associated genes in an orthotopic mouse model of lung cancer (Luc = Luciferase, GFP = green fluorescent protein). **b** Bioluminescence imaging (BLI) of SCID CB.17 mice transplanted with GFP-Luc+ H1975 expressing the shRNA library (lower panel) or parental GFP-Luc+ H1975 cells (upper panel). **c** Differential representation of individual shRNAs with highest enrichment in metastatic tumors. Data were normalized to shRNA representation in the initial cell population. On the left side, dots representing genes that are targeted by these shRNAs are displayed as a fraction of read counts for individual genes relating to all read counts resulting from barcode sequencing of the samples from two different metastases obtained in two mice (met1, met2; ‘freq.’ = frequency). On the right side, these data are presented as fold change (FC) between metastatic and primary tumors from the same two mice (referred to as ‘met1’, ‘met2’, and ‘prim1’, ‘prim2’, respectively). In both graphs, genes close to the diagonal are expected to be consistently enriched in metastatic tumors. Therefore, aryl hydrocarbon receptor (*AHR*, red circle) was selected for further characterization. **d** Kaplan–Meier plots displaying progression-free survival (PFS) in patients with stage I lung adenocarcinomas with high or low *AHR* expression (20820_at)^45^. *AHR* expression was dichotomized at the median expression level. **e** Immunoblotting of AHR expression in GFP-Luc+ H1975 shAHR-K2 and GFP-Luc+ H1975 shScr. **f** Representative BLI images of SCID CB.17 mice transplanted with either GFP-Luc+ H1975 shAHR-K2 or GFP-Luc+ H1975 shScr. **g** Metastasis-free survival of SCID CB.17 mice bearing GFP-Luc+ H1975 expressing shAHR (K2) or shScr. *P*-value, determined by log-rank test.

To identify metastasis-modulating genes, we performed an unbiased shRNA screen in a model based on EGFR-driven H1975 human lung adenocarcinoma cells, which have low endogenous metastatic potential^11^. A barcoded shRNA library was lentivirally expressed under single hit conditions in H1975 cells, which were also engineered to express GFP and luciferase reporters (Fig. 1a). The library-expressing cell population was implanted in the left lung of immunocompromised mice following an established surgical protocol^12^. Six weeks post implantation the majority of mice receiving control cells displayed localized tumors. In contrast, bioluminescent in vivo imaging revealed metastatic spread in mice implanted with shRNA-library-transduced cell populations (Fig. 1b). Deep sequencing of barcoded shRNAs isolated from explanted primary tumors, metastases, and the initial cell population allowed identification of shRNAs enriched or depleted in metastases (Fig. 1c). We defined candidate metastasis suppressors by shRNAs enriched in metastases of two independent mice (Supplementary Table 1). While several candidates were identified with varying barcode representation levels, AHR was selected for validation (Fig. 1c) due to its comparable representation pattern in both metastatic tumors that were analyzed. Interestingly, re-analyses of publicly available RNA expression data from patient cohorts with early-stage and locally advanced lung adenocarcinoma revealed that time-to-first-progression and overall survival were significantly reduced in patients with tumors exhibiting low *AHR* expression (Fig. 1d, Supplementary Fig. 1a).

### Validation of AHR as a suppressor of lung cancer metastasis in vivo

To functionally validate a role of AHR in metastasis, shRNAs targeting endogenous *AHR* were stably expressed in H1975 cells. Suppression of *AHR* was confirmed by qRT-PCR and immunoblotting (Supplementary Fig. 1b-c) and did not affect cell proliferation in vitro (Supplementary Fig. 1d). Clonal H1975 populations with effective *AHR* knockdown (shAHR-K2) were engineered to express GFP and luciferase reporters (Fig. 1e). Notably, the sequence targeted by this shRNA was independent of those used in the primary screen. These shAHR-K2 cells and control cells were orthotopically implanted in the left lungs of immunocompromised mice. Luciferase-based in vivo imaging revealed significantly reduced metastasis-free survival in mice implanted with shAHR-K2 tumors (Fig. 1e, f, Supplementary Fig. 1e).

### Suppression of *AHR* enhances the invasive capacity and metabolic stress resistance of lung cancer cells

**Fig. 2:**
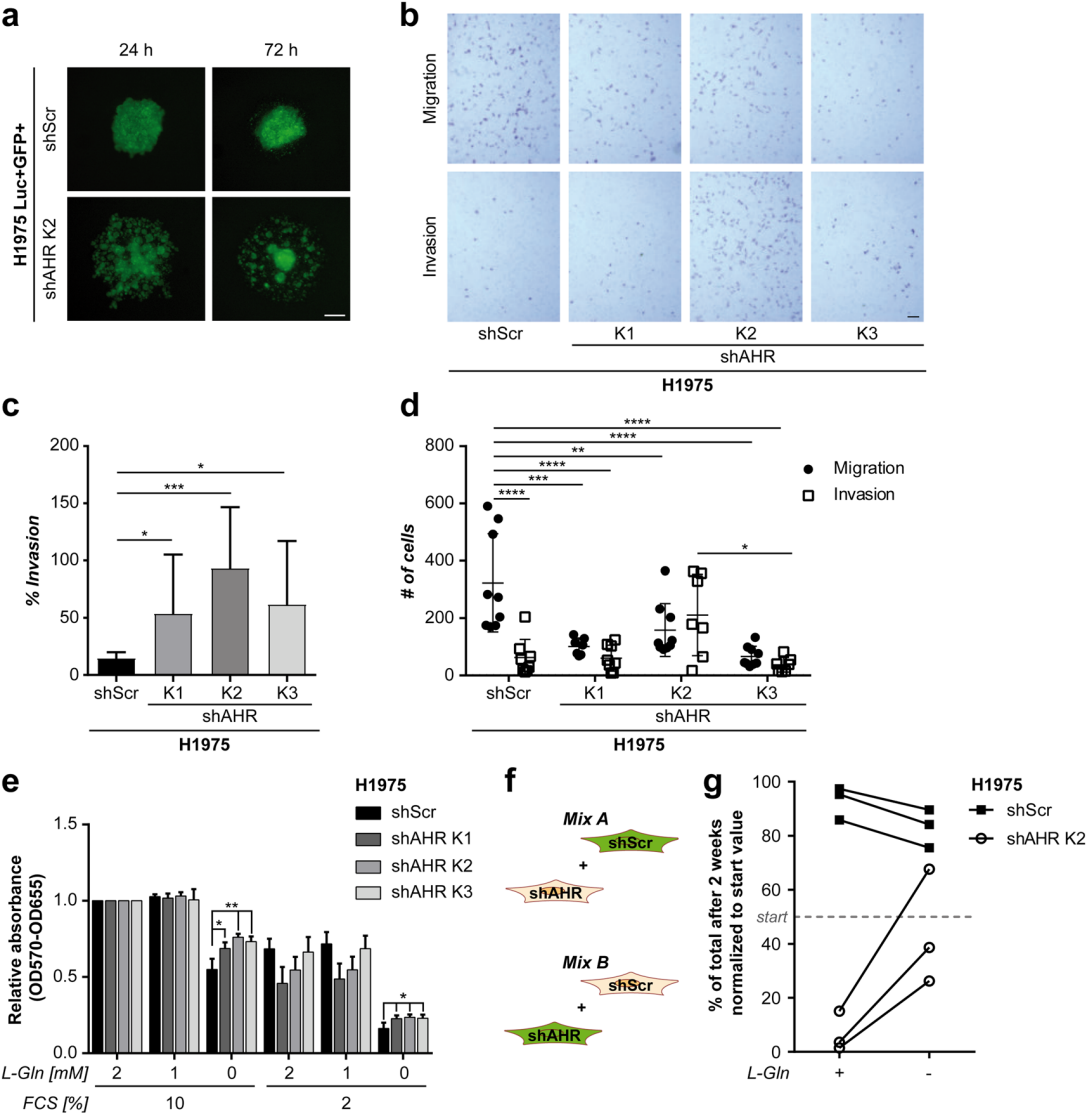
Suppression of endogenous AHR enhances invasive capacity and metabolic stress resistance of H1975 lung cancer cells. **a** Representative pictures taken from spheroids formed by GFP-Luc+ H1975 shAHR-K2 and H1975 shScr control cells. Scale bar, 200 µm. **b** Representative pictures from a combined migration/invasion assay using H1975 shAHR-K1, -K2, -K3, or H1975 shScr control cells. Scale bar, 200 µm. **c** Relative invasion was calculated by dividing the mean number of invading cells by the mean number of migrating cells. **d** Number of migrating and invading cells for the three different H1975 shAHR clones H1975 shScr controls. **e** Proliferation and metabolic viability of three independent clones of H1975 expressing shAHR (K1-K3) and H1975 shScr controls cultured at different FCS and L-glutamine (L-Gln) levels was studied using MTT assay. **f** Schematic representation of the cell competition assay in which H1975 cells with *AHR* knowckdown (shAHR) or control cells (shScr) expressing GFP were mixed with their unlabeled counterparts. **g** Ratios of GFP-Luc+ H1975 shScr and H1975 shAHR-K2 cells were assessed after two weeks of co-cultivation in glutamine-deprived or normal culture conditions using flow cytometry. *n* = 3. Data are shown as mean ± SD normalized to respective control. Significance was assessed using Student’s *t*-test (**c**) or one-way ANOVA (**d**, **e**).

To mechanistically dissect the modulation of metastasis by AHR we compared H1975 cells with maintained (shScr) and suppressed endogenous *AHR* in three independent H1975 shAHR cell clones (K2 and two additional clones, designated K1 and K3, respectively). *AHR* knockdown induced cell scattering in a spheroid formation assay, which indicates increased migratory and invasive capacity (displayed for shAHR-K2 in Fig. 2a). Concordantly, matrigel invasion was significantly increased in all three clones (Fig. 2b–d), whereas migration itself was reduced (Fig. 2d). Restoring endogenous AHR expression in shAHR-K2 cells by introducing an shRNA-resistant mutant *AHR* cDNA partially reversed this phenotype by reducing invasive capability while not effecting migration (Supplementary Fig. 2a-e).

Next, metabolic stresses encountered by extravasating and invading cancer cells were mimicked by limiting culture conditions. The proliferation of shAHR cell clones K1-K3 under glutamine starvation and serum starvation was significantly increased as compared to controls (Fig. 2e). Importantly, cells with suppressed AHR were positively selected in cell competition experiments with either fluorescently labeled H1975 shAHR-K2 cells or H1975 shScr control cells admixed with their unlabeled counterparts (Mix A and Mix B, respectively, Fig. 2f). When cultured under normal conditions for two weeks H1975 shAHR-K2 cells were outcompeted by AHR-proficient control cells. However, depletion of glutamine resulted in significant enrichment of H1975 shAHR-K2 cells in the mixed population (Fig. 2g).

Collectively, these findings indicate that suppression of AHR enhances the invasive capacity and adaptation to metabolic stress of lung adenocarcinoma cells.

### AHR activity impacts gene expression programs associated with stress response and epithelial–mesenchymal transition

**Fig. 3:**
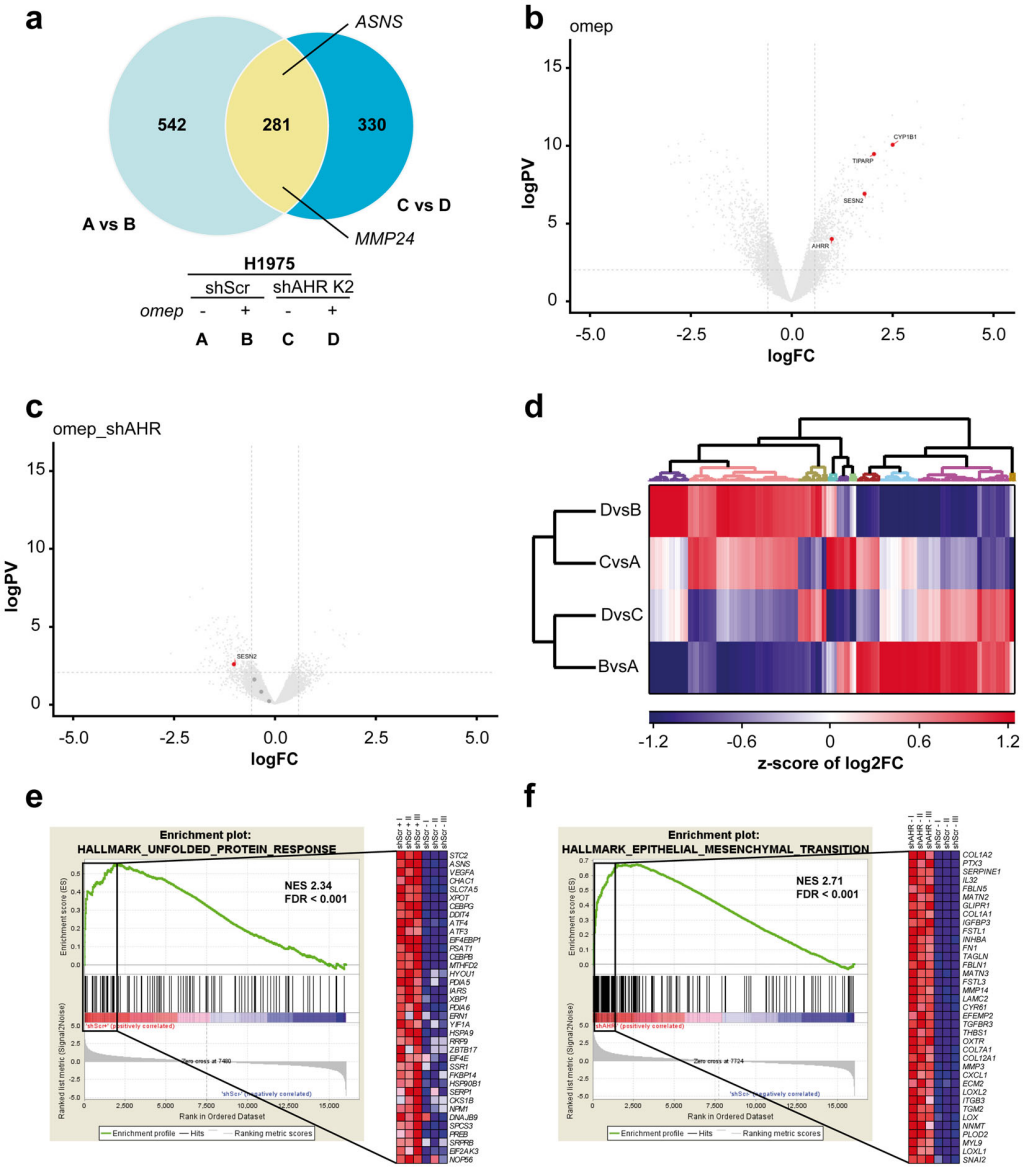
AHR-regulated constitutive and ligand-activated gene expression patterns reveal association with stress response and epithelial–mesenchymal transition. **a** Venn diagram of differential gene expression analysis of H1975 cells with (shAHR-K2) and without knockdown of *AHR* (shScr), treated (+) or untreated (−) with omeprazole (omep, 200 µM) for 48 h. **b** Volcano plot displaying differentially expressed genes between omeprazole-treated and control (DMSO-treated) H1975 shScr cells (‘omep’ effect, ‘log PV’ = log-converted *p*-value; ‘log FC’ = log-converted fold change). **c** Volcano plot indicating differentially expressed genes between omeprazole-treated H1975 cells with and without knockdown of *AHR* (‘omep+shAHR’ effect, ‘log PV’ = log-converted *p*-value; ‘log FC’ = log-converted fold change). **d** Euclidean clustering was used to generate a heat map displaying patterns of differentially expressed genes (*p* < 0.05) upon omeprazole treatment. Experimental groups A–D were chosen as defined in (**a**). **e** Gene-set enrichment analysis (GSEA) revealed significant correlation of genes annotated with ‘Unfolded Protein Response’ in omeprazole-treated H1975 cells expressing shScr compared to the DMSO-treated control. **f** GSEA plot indicating that genes annotated with “Epithelial–Mesenchymal-Transition” are enriched in H1975 cells with *AHR* knockdown as compared to the shScr/*AHR*-proficient cells.

AHR is a ligand-activated transcription factor which is guiding responses to xenobiotics and endogenous metabolites including kynurenine. AHR has also been linked to autoimmunity, metabolic imbalance, and inflammatory diseases (reviewed in ref. ^13^). To discriminate between intrinsic and ligand-induced AHR effects, we studied the impact of different AHR activators including kynurenine A (KynA), the tryptophan analog 6-formylindolo[3,2-b]carbazole (FICZ), biochanin A (BioA), and omeprazole on induction of the known AHR target, CYP1A1. We could demonstrate strong CYP1A1 mRNA induction by all ligands in H1975 cells that was impaired in the three shAHR clones. The magnitude of this effect was highest and became significant in omeprazole-treated cells. As omeprazole-mediated induction of AHR-dependent pathways had previously been linked to reduced metastasis in breast cancer models^14^, we set out to identify those AHR effectors involved in the modulation of metastatic processes. Therefore, we devised mRNA sequencing in H1975 cells with and without shRNA-mediated suppression of endogenous *AHR* (H1975 clone shAHR-K2 and H1975 shScr, respectively) in the presence or absence of omeprazole. Principal component analysis showed clear and robust separation of the four conditions (Supplementary Fig. 3b). A substantial number of known AHR-dependent genes was regulated exclusively either by omeprazole (*n* = 542) or by *AHR* knockdown (*n* = 330, Fig. 3a) using an adjusted *p*-value (p-adj) <0.05. In AHR-proficient cells, omeprazole induced *SESN2* and *CYP1B1* as well as known AHR repressors, *AHRR* and *TIPARP*, which are balancing AHR responses (Fig. 3b^15,16^). Induction of these transcriptomic programs by omeprazole was significantly blunted in shAHR-K2 cells (Fig. 3c), which is in line with our findings obtained for the AHR target, *CYP1A1* (Supplementary Fig. 3a). Of note, proliferation of AHR-proficient H1975 cells, but not of AHR knockdown cells, was significantly inhibited by treatment with omeprazole in a dose-dependent manner (Supplementary Fig. 3c). Still, global gene expression analyses revealed comparable gene expression patterns between AHR-proficient cells and shAHR-K2 cells upon omeprazole treatment, while the amplitude of target gene regulation was reduced in shAHR-K2 cells (Fig. 3d). Gene-set enrichment analyses (GSEA)^17,18^ indicated that AHR activation by omeprazole significantly correlated with the unfolded protein response and xenobiotic metabolism (Fig. 3e, Supplementary Fig. 3d). Interestingly, knockdown of *AHR* was significantly linked to increased expression of EMT genes including mediators of TGF-β signaling (Fig. 3f, Supplementary Fig. 3e-f). These events were partially enhanced in cells with *AHR* knockdown (Supplementary Fig. 3g). Importantly, the enhanced induction of TGF-β signaling in AHR-deficient H1975 cells resulted in further increase of their invasive capacity (Supplementary Fig. 3h-i).

### AHR regulates ASNS expression in an ATF4-dependent manner and impacts MMP expression and activity

**Fig. 4:**
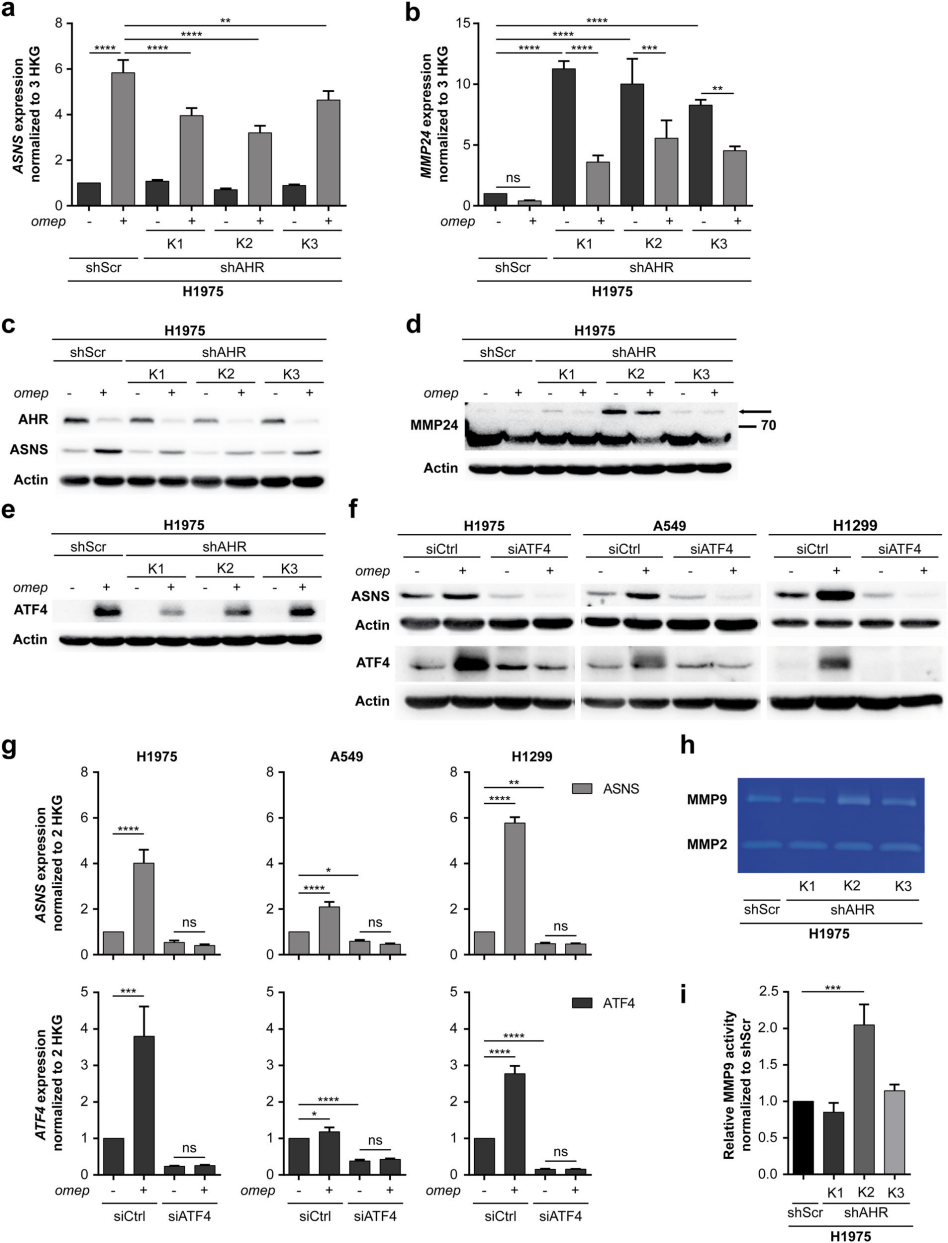
AHR regulates ASNS expression in an ATF4-dependent manner and affects MMP expression and activity. Target gene expression of three independent clones of H1975 expressing shAHR (K1-K3) and H1975 shScr control cells (shScr) was analyzed after treatment with omeprazole (omep, 200 µM, 48 h) or DMSO (−) by qRT-PCR (**a**, **b**) and immunoblotting (**c**–**e**). **a** qRT-PCR analysis of *ASNS* mRNA expression. **b** qRT-PCR analysis of *MMP24* mRNA expression. **c**–**e** Immunoblotting analysis of AHR, ASNS (**c**), MMP24 (**d**), and ATF4 (**e**) protein expression. **f** Prior to treatment with omeprazole (omep, 200 µM, 48 h), H1975, A549, and H1299 were transfected with siRNAs targeting *ATF4* or a non-targeting control (siCtrl). Expression of ASNS and ATF4 was assessed using immunoblotting. **g** qRT-PCR analysis of *ASNS* and *ATF4* mRNA levels relative to siCtrl control. Target gene expression was normalized to two (*ACTB*, *GAPDH*) or three (*ACTB*, *GAPDH*, *HPRT1*) housekeeping genes (HKG) as indicated. **h** Gelatine zymography of MMP2 and MMP9 activity. **i** Semiquantitative analysis of gelatinolytic bands from MMP9 (ImageJ). Data are shown as mean ± SD. Significance was assessed using one-way ANOVA.

Beyond EMT, evaluation of AHR-regulated expression and activity patterns revealed several targets implicated in cancer progression, including members of the matrix metalloprotease family (MMPs, *MMP9* and *MMP24*), asparagine synthetase (*ASNS*) and the *ATF4* transcription factor. We confirmed that mRNA and protein expression of ASNS was strongly induced by the AHR activator omeprazole (Fig. 4a, c). Induction of ASNS by omeprazole was significantly attenuated by *AHR* knockdown. In contrast, MMP24 was derepressed by *AHR* knockdown, but downregulated by omeprazole (Fig. 4b, d). ASNS has been suggested as direct target of ATF4 under nutritional stress conditions^19,20^. We further queried whether AHR-dependent regulation of *ASNS* was mediated by ATF4. Indeed, *AHR* knockdown blunted induction of ATF4 by omeprazole (Fig. 4e). Using a reporter construct, in which luciferase expression is dependent on ATF4 promoter activity, we confirmed ATF4 as a direct transcriptional target of activated AHR (Supplementary Fig. 4a). Moreover, ATF4 regulation by omeprazole was dose- and time-dependent, which was equally true for MMP24 and ASNS (Supplementary Fig. 4b-d). We next verified that the AHR activator omeprazole induced expression of ASNS and ATF4 in three different lung cancer cell lines, H1975, A549, and H1299 (Fig. 4f). Induction of ASNS by omeprazole was prevented by siRNA-mediated suppression of *ATF4* confirming this regulatory axis (Fig. 4f, g). In addition, gelatine zymography was performed to interrogate MMP2 and MMP9 activities, which are also linked to EMT phenotypes^21,22^. In line with our finding from RNA sequencing, H1975 shAHR-K2 cells displayed increased MMP9 activity, while MMP2 activity was not modulated by AHR expression levels (Fig. 4h-i). The significantly enhanced invasive capacity of H1975 shAHR-K2 cells could be reduced to levels observed in parental cells by addition of the MMP inhibitor, BB94 (Supplementary Fig. 4e). Re-expression of a shRNA-resistant *AHR* cDNA partially suppressed MMP9 activity H1975 shAHR-K2 cells (Supplementary Fig. 4f-g). Taken together, AHR modulates the expression and functional activity of MMPs, and regulates *ASNS* expression in an ATF4-dependent manner. Hence, AHR is a central regulator of multiple programs crucially involved in invasion and stress response during the metastatic process.

## Discussion

While targeted therapies are standard of care in subtypes of metastatic lung cancer, which are defined by dominant and druggable oncogenic drivers, currently no such molecularly targeted treatment is established in preventing metastatic relapse in early-stage lung cancer^23^. The CTONG1104 trial comparing gefitinib and chemotherapy following resection of EGFR-mutant lung cancer stages II and III A was formally positive but failed to demonstrate curative potential^24^. Similar studies with additional EGFR and ALK inhibitors are underway and results are awaited. While these important trials address the utility of targeting dominant oncogenic drivers in the curative setting, they do not study the modulation of specific metastasis factors. Against this background, we initiated a functional screening campaign to uncover genes involved in facilitating metastasis of orthotopically established lung tumors in mice. Using an unbiased shRNA-library based approach, we identified *AHR*, among others, as a significantly overrepresented shRNA target in metastases. However, it has to be noted that these findings are based on a single clone that was recovered from the screen, while several other shRNAs targeting AHR were not found to alter metastases.

Previously, AHR was mainly recognized as pro-tumorigenic, mostly by its ability to induce carcinogenesis upon sustained activation by its xenobiotic ligand 2,3,7,8-tetrachlorodibenzo-p-dioxin (TCDD) in different entities including lung cancer^25,26^. Lee and colleagues have linked AHR and TGF-β signaling to suggest a role for AHR in downregulating SMAD4 leading to impaired invasive capacity^27^. In a subsequent manuscript by Tsai et al. the same authors describe a correlation between AHR activation and autophagy in lung cancer^28^. They also show that AHR overexpression reduced homing of CL1-5 cells to the lungs. In a third study based on in vitro models, Li and colleagues have hypothesized that cytoplasmic AHR accelerates vimentin degradation and thus contributes to EMT and migration^29^. In malignant brain tumors, AHR suppressed anti-tumor immunity and promoted tumor cell survival and motility upon activation by its natural ligand kynurenine^30^. In additional models including Ewing sarcoma, however, autocrine AHR activation was repressed by oncogenic signaling, suggesting tumor-suppressive activity^31^. Further, kynurenine-mediated activation of AHR impaired tumor progression and metastasis in a neuroblastoma model^32^. A role of AHR in breast cancer metastasis has been controversially discussed with conflicting results obtained in different assays and model systems^14,33^. In lung cancer, in vitro studies indicated that AHR overexpression negatively regulates tumorigenesis by reducing lung cancer cell viability, growth and invasive capacity^27,34^. Collectively, these reports suggest entity- and stage-dependent functions of AHR in carcinogenesis and tumor progression.

In an orthotopic, spontaneously metastasizing lung cancer model, we here uncovered AHR-mediated regulation of several metastatic programs including EMT and MMP activity. Interestingly, AHR activation induced ASNS via ATF4 (Fig. 4c, e–g). Previously, oncogenic KRAS was described as regulator of ASNS induction by ATF4 in lung cancer models^35^. Our study involved several lung cancer models including H1975 (EGFR mutations), A549 (KRAS mutation), and H1299 (NRAS mutation). While glutamine deprivation induces ASNS in several cell types^36,37^, our data imply that the AHR-ATF4-ASNS axis is functional irrespective of the dominant oncogenic driver in lung cancer cells (Fig. 4f). ASNS activity is expected to increase the levels of asparagine at the cost of reducing aspartate, which is limiting cancer cell growth in the absence of the importer SLC1A3^38,39^. Suppression of endogenous *AHR* increased the metabolic stress resistance of lung cancer models, thus complementing invasiveness in a metastatic phenotype. Furthermore, invasive capacity itself is controlled by AHR through regulation of TGF-β signaling, which is derepressed in AHR knockdown cells. This is corroborated by the observation that TGF-β mediated invasion is significantly enhanced in shAHR cells (Supplementary Fig. 3h-i). Although these findings support target inhibition by the shRNA against AHR used in vitro, some of the less established AHR downstream effects await conformation by other methods, either by using AHR inhibitors or by shRNAs targeting different regions within the AHR gene. In summary, our findings strongly support a role of AHR as relevant suppressor of lung cancer metastasis. Intriguingly, AHR is a sensor and regulator of the endogenous defense system against xenobiotic chemicals which are implied in lung cancer carcinogenesis and progression. Here, we show that an AHR ligand also induces anti-metastatic programs in lung cancer models. This suggests that the host defense system against xenobiotics not only involves their decomposition and elimination, but also controls the phenotype of mutated, premalignant cells. Established lung cancers with suppressed AHR-dependent defense systems exhibit higher likelihood of metastasis and relapse in murine models and in patients (Fig. 1f, g).

Compounds reactivating suppressed AHR and/or AHR-regulated anti-metastatic programs may provide leads for the development of novel and specific pharmacologic strategies to prevent metastatic progression and increase survival in patients with early-stage lung cancer.

## Methods

### Cell culture and reagents

All cell lines were obtained from ATCC (Manassas, VA, USA), authenticated using STR analysis; absence of mycoplasma contamination was confirmed by regular testing. NCI-H1975, A549, and NCI-H1299 were cultured in RPMI 1640 (Gibco, Paisley, UK), and HEK293FT were cultured in DMEM (Gibco). All cell culture media were supplemented with 10% FBS (Biochrom, Berlin, Germany) and 2 mM L-glutamine (Gibco) unless otherwise indicated. Omeprazole was purchased from Sigma-Aldrich (St. Louis, MO, USA), recombinant human TGF-β1 was purchased from Peprotec (Rocky Hill, NJ, USA), and puromycin was purchased from Calbiochem (San Diego, CA, USA).

### Orthotopic lung cancer model

Orthotopic implantation of human adenocarcinoma cell lines in SCID CB.17 mice (Taconic, Germantown, NY, USA) was performed as described previously^11^. Briefly, following thoracotomy of anesthetized mice 10^6^ cells resuspended in Matrigel were injected into the left lung lobe. Mice were sutured and allowed to recover for 1 week prior to imaging.

### In vivo and ex vivo bioluminescence imaging

Tumor-bearing mice were imaged at the UCSF Preclinical Therapeutics Core with Xenogen IVIS 100 bioluminescent imaging as described previously^11^. For each in vivo imaging time point, mice were anesthetized and injected with 200 µl (150 mg/kg) D-Luciferin. Bioluminescence intensity (BLI) of tumors was monitored once weekly until week 5. After 5 weeks, D-Luciferin was injected and mice were sacrificed for ex vivo imaging.

### In vivo shRNA screen and identification of shRNAs positively selected in metastases

DECIPHER shRNA Library Human Module 1 (Cellecta Inc., Mountain View, CA) was used to generate a lentiviral pooled barcoded shRNA library following the manufacturer’s protocol. Briefly, HEK293FT cells were transfected with shRNA-library DNA using FuGENE (Promega, Madison, WI). Viral supernatants were collected 72 h post transfection. NCI-H1975 cells co-expressing GFP and luciferase from the EGFP-ffluc epHIV7 vector (kindly provided by Dr. Michael Jensen, Seattle) were transduced with the shRNA vector-containing supernatants. Transduction efficacy was set to 25% so that cell numbers exceeded the library’s complexity by 1.000-fold. After puromycin selection, cell populations were orthotopically implanted in the left lungs of SCID CB.17 mice (*n* = 20). Tumor growth and metastasis formation were monitored using bioluminescent in vivo imaging until metastases, as defined by infiltration of the contralateral lung, were detectable in 13/20 mice. Metastases and primary tumors of two mice were harvested and subjected to parallel sequencing of barcoded regions to identify representation of the library shRNAs as previously described^11^. Briefly, shRNA counts were grouped and summed up for the corresponding genes. The resulting values were normalized by dividing the gene-wise counts by the total read counts for each sample. A gene was considered enriched if its fraction of the total shRNA count was at least 10^−5^ in two independent mouse tumors, which corresponds to the 95th percentile of shRNA distribution in the metastatic samples.

### Gene suppression protocols

Lentiviral shRNAs targeting *AHR* and non-targeting control were obtained from Sigma-Aldrich (St. Louis, MO, USA). Following transduction, H1975 cell lines were selected with 0.5 µg/ml puromycin for 7 days. Clones were established from the transduced populations using limiting dilution. After lentiviral transduction of cells with EGFP-ffluc-epHIV7 vector, GFP-positive cells were isolated using fluorescence-activated cell sorting.

For transient knockdown of *ATF4*, specific MISSION® esiRNA (Sigma-Aldrich, Munich, Germany) and MISSION® siRNA Universal Negative Control were introduced by RNAiMAX (Thermo Fischer Scientific, Waltham, MA, USA) according to the manufacturer’s protocol.

For sequences of shRNAs and siRNAs see Supplementary Table 2.

### Immunoblotting and qRT-PCR

The following primary antibodies were used for immunoblotting: AHR (Cell Signaling Technology, #13790, 1:1000), ASNS (Thermo Fisher Scientific, # PA5-56113, 1:1000), ATF4 (Cell Signaling Technology, #11815, 1:1000), beta-Actin (MP Biomedical, #691002, 1:1000), E-cadherin (Cell Signaling Technology, #3195, 1:1000), MMP24 (Genetex, #GTX128246, 1:1000), SMAD2 (Cell Signaling Technology, #5339, 1:1000), pSMAD2 (Cell Signaling Technology, #3108, 1:1000). Secondary antibodies were HRP-conjugated (Pierce Antibodies, 1:4000). For qRT-PCR, RNA was isolated and purified using High Pure RNA Isolation Kit (Roche, Mannheim, Germany), and reversely transcribed by Transcriptor High Fidelity cDNA Synthesis Kit (Roche, Mannheim, Germany). *ACTB*, *AHR*, *ASNS*, *ATF4*, *CYP1A1*, *GAPDH*, *HPRT1*, *MMP9*, or *MMP24* expression (primers listed in Supplementary Table 3) was quantified in duplicates or triplicates on a LightCycler®480 System (Roche, Rotkreuz, Switzerland) using the 2-ΔΔCt method and *ACTB*, *GAPDH*, and *HPRT1* as internal controls.

### Luciferase reporter assay

Cells were co-transfected with 0.5 µg pGL4 (Luc2P/ATF4-RE/Hygro) and 0.1 µg pGL4.74 to express firefly luciferase from *Photinus pyralis* under the control of the ATF4 promoter and Renilla luciferase, respectively (both from Promega, kindly provided by Prof. Eric Metzen, University Hospital Essen). After treatment, cells were processed using Dual-Luciferase Assay (Promega) according to the manufacturer’s protocol. Both luciferase activities were sequentially recorded using a GloMax luminometer (Promega).

### Transwell migration and invasion assays

Migration and invasion were assessed using BioCoat© GFR Matrigel (Corning, Bedford, MA, USA) according to the manufacturer’s protocol. Briefly, 1.5 × 10^4^ cells/insert were seeded in the upper compartment after starvation using low-serum medium. MMP inhibition was achieved by addition of BB94 (5 µM) 4 h prior to seeding of the cells. Cells on the apical side were mechanically removed after 24 h. Migrated/invaded cells were fixed, stained, and counted on a KEYENCE BZ II analyser microscope (KEYENCE Corporation of America, Itasca, IL, USA).

### Gelatine zymography

Equal volumes of conditioned medium (CM) from cells adapted to serum-reduced medium were harvested after 16 h of incubation, centrifuged and separated by SDS-PAGE according to established protocols^40^. Enzymatic activity was revealed as non-stained areas after incubation in enzyme buffer for 18–48 h at 37 °C and staining with Coomassie blue.

### Spheroid formation assay

Spheroid formation was assessed by seeding 5 × 10^3^ cells/well in a 96-well plate with cell-repellent surface (Greiner Bio-One, Frickenhausen, Germany). Photomicrographs were taken after 24, 48, and 72 h of incubation at 37 °C and 5% CO_2_ in a humidified atmosphere.

### RNA sequencing

RNA was isolated using RNeasy Mini Kit (Qiagen, Hilden, Germany) following the manufacturer’s recommendations. RNA integrity was confirmed using the Screen Tape Kit (Agilent 5067-5576) and Screen Tape Station. 3’mRNA-Seq library preparation was performed as described previously and analyzed on an Illumina HiSeq2500^41^. Transcript-level quantification was performed using salmon 0.11 and Ensembl GRCh38 assembly as reference genome. Resulting count estimates were then merged and transformed to gene-level using tximport 0.16. Differential expression analysis was performed on the resulting gene-counts table using Deseq2 1.18. For displaying Volcano plots, count matrices were filtered for genes that had counts <20. Data were TMM normalized^42^, followed by modeling variance using voom^43^. Expression changes were considered significant with p-adj <0.05 and log2-fold change (log2FC) <−1 or <1. The adjusted *p*-values were generated using the DESeq2 implementation of Benjamini–Hochberg/FDR controlling. Principal component analysis and heat map clustering was performed using Perseus software (1.5.5.3^44^). Euclidean clustering was carried out for differential expression data of genes significantly altered between experimental groups. Gene-set enrichment analysis (GSEA) was performed using the curated hallmark gene-set collection and java-based GSEA software v3.0^17,18^. Parameters were set to 1000 permutations and gene-set permutation mode.

### Proliferation assays

Cell proliferation was analyzed using 3-(4,5-Dimethylthiazole-2-yl)-2,5-diphenyl-2H-tetrazolium bromide (MTT, Carl-Roth, Karlsruhe, Germany) in 96-well plates after 72 h. For L-glutamine and serum starvation studies, cells were directly seeded into different starvation media and incubated for 72 h before addition of MTT.

### Cell competition assays

H1975 cells expressing AHR-specific or control shRNAs were mixed in equal ratios and co-cultivated in culture medium with or without the addition of L-glutamine. H1975 control cells or H1975 shAHR-K2 expressing EGFP-ffluc were mixed with their non-labeled counterparts. The proportion of fluorescent cells was determined by flow cytometry.

### Lung cancer survival analysis

Survival curves were generated using the Kaplan–Meier Plotter database^45^. The following parameters were set for all analyses: ‘recommended’ AHR probe (20820_at), adenocarcinoma histology, stage I, and dichotomisation at median expression level. Analysis was performed regarding overall survival (*n* = 370) and time-to-first-progression (*n* = 283).

### Statistical analysis

Experimental data are presented as mean ± SD. Significance was assessed using one-way ANOVA or unpaired Student’s two-tailed *t*-test as indicated. For KM metastasis-free survival curves the log-rank test was used.

## Supplementary information

Supplemental Figure 1

Supplemental Figure 2

Supplemental Figure 3

Supplemental Figure 4

Supplementary Table 1

Legends to Supplementary Figures and Tables

Author list changes approval

## Data Availability

RNA-sequencing data generated in this study have been deposited in NCBI Sequence Read Archive with the primary accession code PRJNA576587.
