# Supplementary figures and images for: Functional screening identifies aryl hydrocarbon receptor as suppressor of lung cancer metastasis
Source: Oncogenesis. 2020 Nov 19;9(11):102. doi: 10.1038/s41389-020-00286-8 (PMC7677369; doi:10.1038/s41389-020-00286-8)

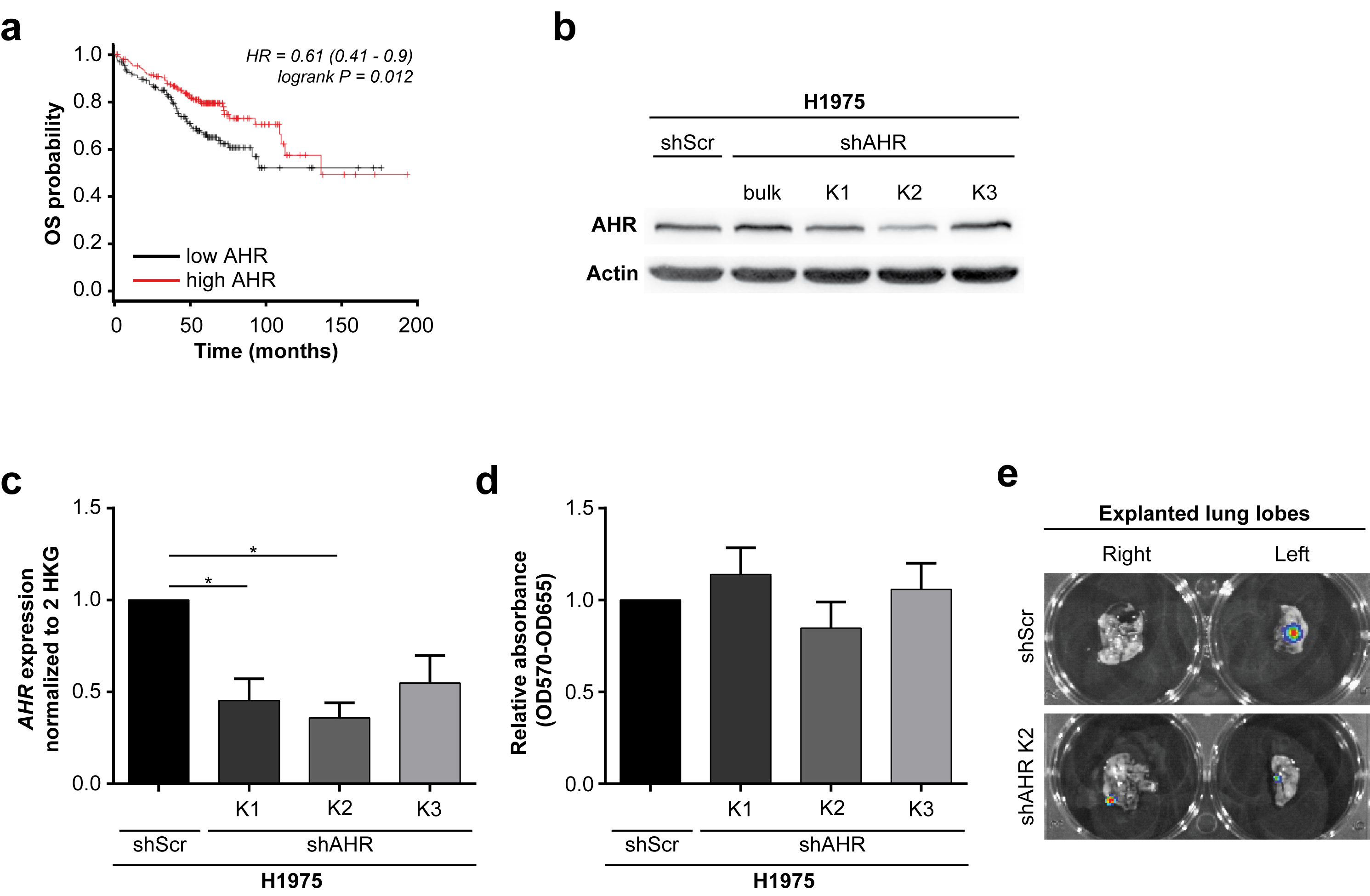

Supplement: Supplementary file 1 — Supplemental Figure 1 [file 41389_2020_286_MOESM1_ESM.png]

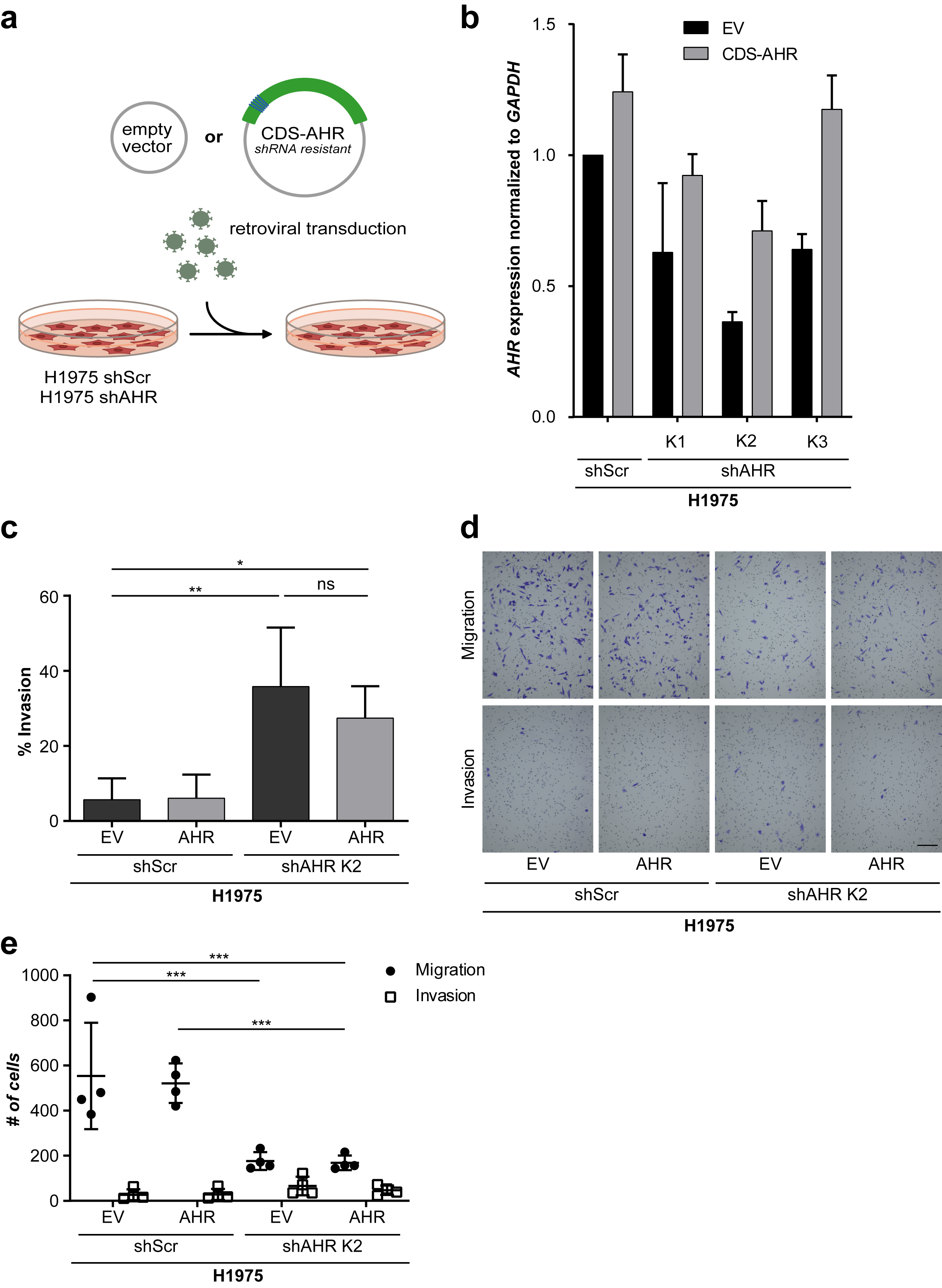

Supplement: Supplementary file 2 — Supplemental Figure 2 [file 41389_2020_286_MOESM2_ESM.png]

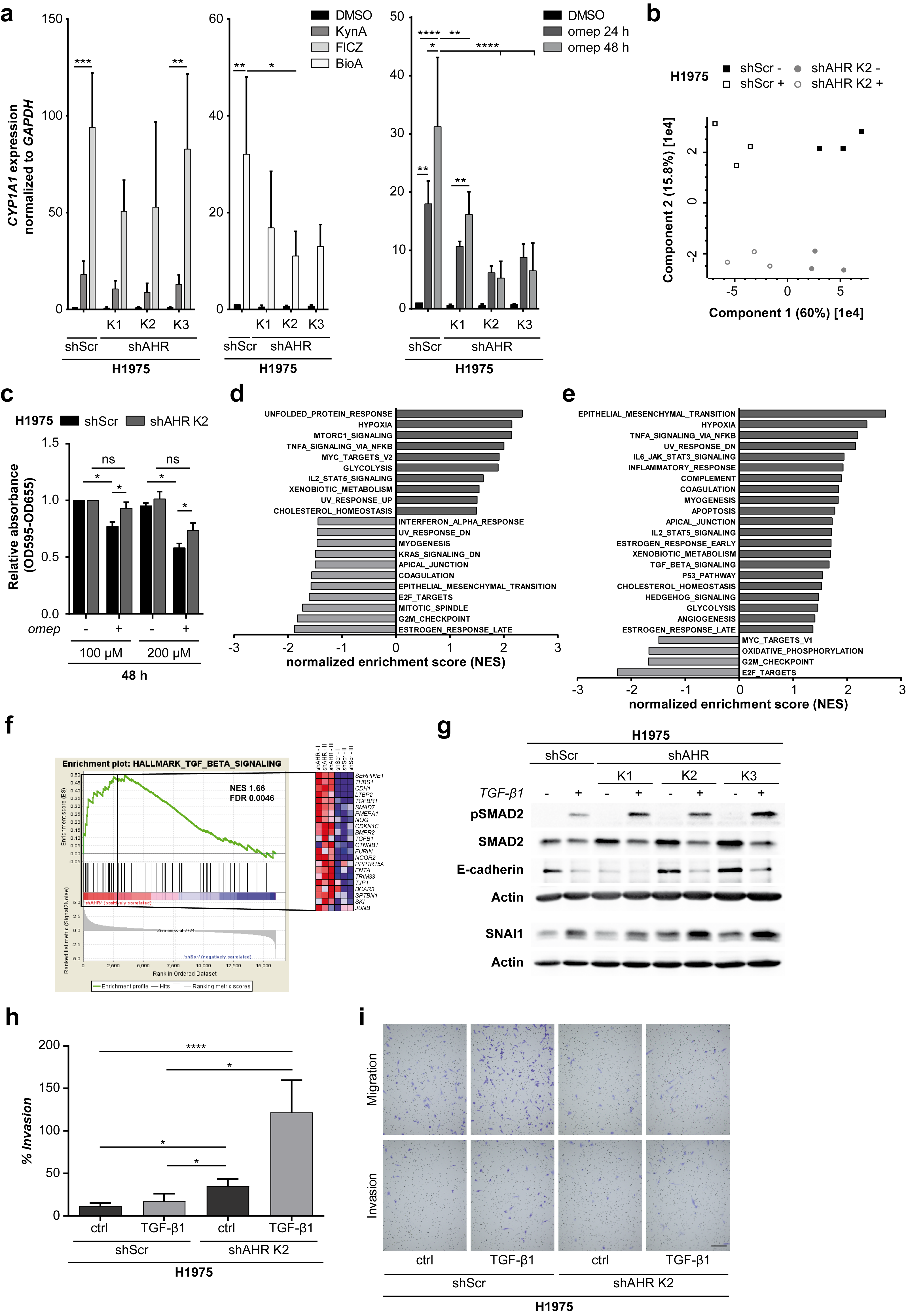

Supplement: Supplementary file 3 — Supplemental Figure 3 [file 41389_2020_286_MOESM3_ESM.png]

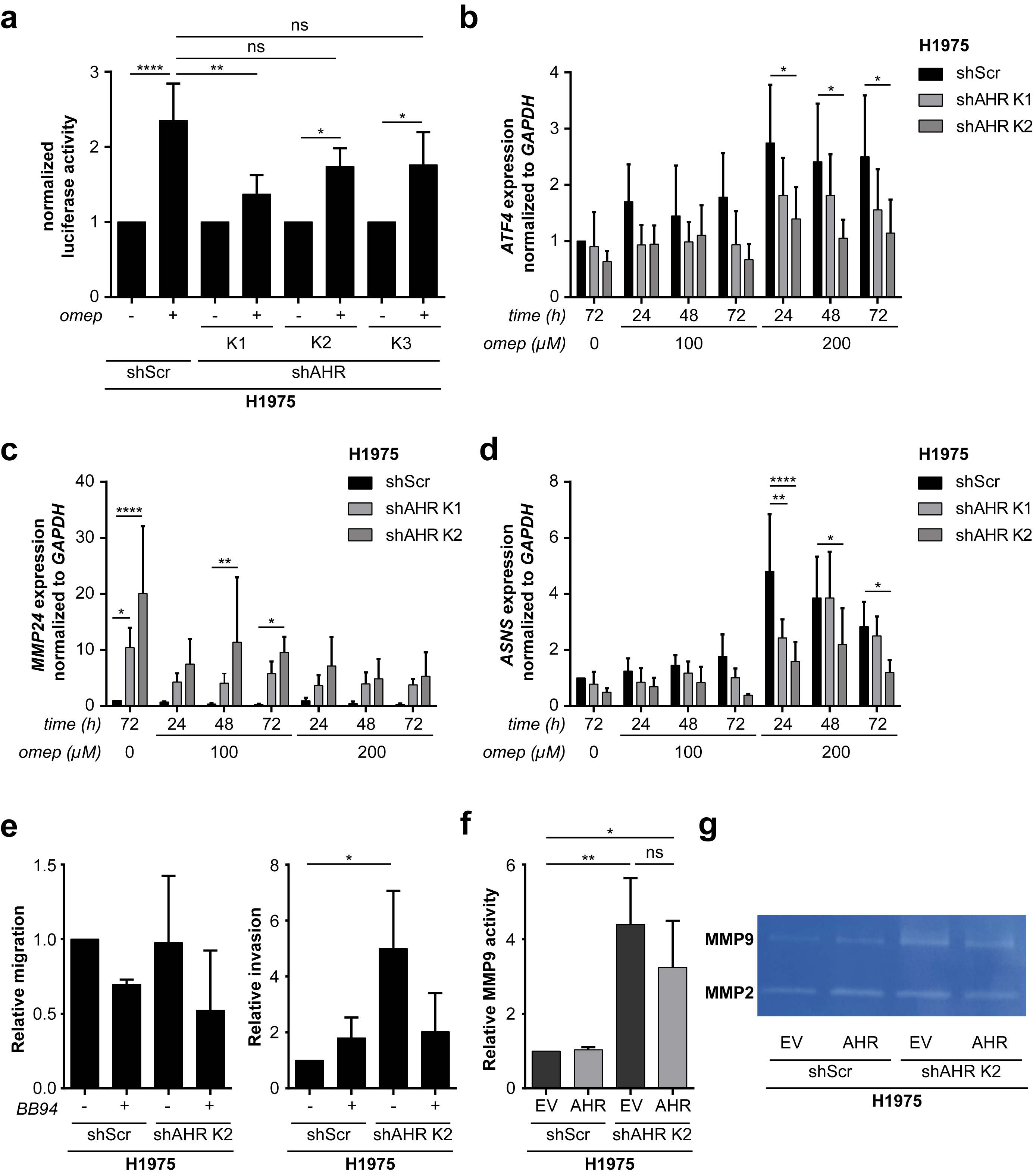

Supplement: Supplementary file 4 — Supplemental Figure 4 [file 41389_2020_286_MOESM4_ESM.png]
