# Supplementary material for: Functional screening identifies aryl hydrocarbon receptor as suppressor of lung cancer metastasis
Source: Oncogenesis. 2020 Nov 19;9(11):102. doi: 10.1038/s41389-020-00286-8 (PMC7677369; doi:10.1038/s41389-020-00286-8)
Supplement: Supplementary file 5 — Supplementary Table 1 [file 41389_2020_286_MOESM5_ESM.pdf]

gene target	barcode seq	h1975_t0.r1	h1975_t0.r2	h1975_primary.r1	h1975_primary.r2	h1975_met.r1	h1975_met.r2
A1BG	TGACGTCACACAGTACAC	30	57	1	0	0	0
A1BG	CAACTGTGGTGTCAACAC	106	31	1	0	0	0
A1BG	GTACTGTGGTGTCAACTG	109	132	0	0	0	0
A1BG	TGACTGTGGTGTCATGGT	185	177	0	0	0	3
A1BG	GTACTGTGGTGTCATGCA	217	250	1	0	0	5
A1BG	GTTGCAACCATGCATGGT	162	140	0	0	0	1
A2M	CAACGTGTGTTGACTGCA	115	205	1	0	0	1
A2M	TGACGTGTGTTGACTGAC	67	53	0	1	0	0
A2M	ACACGTGTGTTGACTGTG	246	196	0	0	0	0
A2M	GTACGTGTGTTGTGGTGT	656	549	0	0	0	3
A2M	CAACGTGTGTTGTGGTCA	301	427	4	0	0	12
A4GALT	ACACCACAACACGTTGTG	769	1021	9	1	4	64
A4GALT	CAACCACAACACGTTGCA	302	564	0	0	1	0
A4GALT	GTACCACAACACGTTGGT	267	195	1	0	0	1
A4GALT	TGACCACAACACGTTGAC	62	177	0	0	0	0
A4GALT	GTACCACAACACCAGTGT	189	200	1	0	0	0
A4GNT	TGACCACAACCATGCATG	128	88	0	0	0	0
A4GNT	ACACCACAACCATGACGT	386	677	0	0	0	1
A4GNT	TGACCACAACCATGACCA	531	437	1	0	0	0
A4GNT	CAACCACAACCATGACAC	71	215	0	0	0	1
A4GNT	GTACCACAACCATGACTG	384	301	0	1	0	0
A4GNT	CATGCACAACTGACACTG	550	288	0	0	0	2
AACS	TGACACGTCACACACAGT	69	97	0	0	0	0
AACS	GTACACGTCACACACACA	65	93	0	0	0	0
AACS	ACACACGTCACACACAAC	97	36	0	0	0	0
AACS	CAACACGTCACACACATG	335	351	1	0	0	0
AACS	GTACACGTCACACAACGT	69	92	0	0	0	0
AADAC	ACACTGTGCACACAGTTG	101	154	0	0	0	0
AADAC	CAACTGTGCACACACAGT	43	69	0	0	0	0
AADAC	ACACTGTGCACACACACA	57	67	0	0	0	0
AADAC	GTACTGTGCACACACAAC	21	24	0	0	0	0
AADAC	TGACTGTGCACACACATG	346	551	1	0	0	0
AADAT	GTACCACAACACCACAAC	58	28	0	0	1	0
AADAT	TGACCACAACACCACATG	223	367	1	0	1	0
AADAT	ACACCACAACACCAACGT	279	388	0	0	0	0
AADAT	TGACCACAACACCAACCA	359	223	1	0	0	0
AADAT	CAACCACAACACCAACAC	49	28	0	0	0	0
AANAT	GTACCACATGTGTGACGT	254	144	0	0	1	0
AANAT	CAACCACATGTGTGCATG	470	184	1	0	0	0
AANAT	CAACCACATGTGTGACCA	514	505	1	0	0	0
AANAT	TGACCACATGTGTGACAC	62	130	0	0	0	0
AANAT	ACACCACATGTGTGACTG	1030	692	3	1	0	0
AANAT	GTTGCACAGTCACAGTGT	999	537	0	1	0	2
AARS	TGACACGTCATGTGTGCA	133	200	0	0	0	4
AARS	CAACACGTCATGTGTGAC	631	125	0	0	0	1
AARS	GTACACGTCATGTGTGTG	275	333	0	1	0	0
AARS	ACACACGTACGTGTGTGT	141	129	1	0	0	0
AARS	TGTGCAGTCAGTCACACA	59	82	0	0	0	0
AARS2	CAACACGTCACAGTACGT	114	526	0	0	0	19
AARS2	ACACACGTCACAGTACCA	451	329	1	0	0	0
AARS2	GTACACGTCACAGTACAC	64	44	0	0	0	0
AARS2	TGTGCAGTCATGGTACCA	213	241	0	1	0	0
AARS2	CATGCAGTCATGGTACAC	154	117	0	1	0	0
AASDH	CAACCAGTGTACGTCAAC	42	121	0	0	0	0
AASDH	GTACCAGTGTACGTCATG	21	16	0	0	0	0
AASDH	TGACCAGTGTACGTACGT	35	30	0	0	0	0
AASDH	GTACCAGTGTACGTACCA	116	321	0	0	0	2
AASDH	ACACCAGTGTACGTACAC	63	169	0	0	0	0
AASDHPPT	GTACCAGTCATGCATGAC	252	144	0	0	1	19
AASDHPPT	CAACCAGTCATGCATGGT	365	473	0	0	0	0
AASDHPPT	ACACCAGTCATGCATGCA	348	298	0	0	0	28
AASDHPPT	TGACCAGTCATGCATGTG	99	127	0	0	0	0
AASDHPPT	CAACCAGTCATGACGTGT	2055	1887	5	2	0	375
AASDHPPT	CATGCAACCATGGTGTAC	28	35	0	0	0	0
AASS	TGTGCACATGACTGACAC	114	52	0	0	1	6
AASS	CAACACGTCATGTGGTTG	398	385	0	0	0	0
AASS	GTACACGTCATGTGCAGT	130	65	1	0	0	0
AASS	CAACACGTCATGTGCACA	290	487	1	0	0	0
AASS	TGACACGTCATGTGCAAC	17	17	0	0	0	0
AASS	ACACACGTCATGTGCATG	85	84	0	0	0	0
ABAT	GTACCACAACGTTGTGGT	262	172	0	1	0	0
ABAT	CAACCACAACGTTGTGCA	573	529	0	0	0	2
ABAT	TGACCACAACGTTGTGAC	12	38	0	1	0	0
ABAT	ACACCACAACGTTGTGTG	278	211	0	0	0	6

ABAT	TGACCACAACCAGTGTGT	376	227	1	0	0	0
ABCA1	CAACCAGTACGTACGTGT	221	293	0	1	1	0
ABCA1	GTTGCAACACCAGTTGGT	299	43	0	0	1	0
ABCA1	CAACCAGTACGTCATGGT	404	506	1	0	0	0
ABCA1	ACACCAGTACGTCATGCA	233	264	0	0	0	0
ABCA1	GTACCAGTACGTCATGAC	47	46	0	0	0	0
ABCA1	TGACCAGTACGTCATGTG	343	255	0	0	0	0
ABCA10	CAACTGCACAGTCAGTTG	281	266	0	314	1	1
ABCA10	TGACTGCACAGTCAGTGT	134	127	1	1	0	0
ABCA10	GTACTGCACAGTCAGTCA	120	142	0	0	0	0
ABCA10	ACACTGCACAGTCAGTAC	53	59	0	0	0	0
ABCA10	GTACTGCACAGTCACAGT	94	126	0	0	0	2
ABCA12	ACACTGCAGTTGTGCAAC	62	75	2	205	59	0
ABCA12	GTACTGCAGTTGTGCACA	190	80	1	0	0	0
ABCA12	CAACTGCAGTTGTGCATG	150	196	0	0	0	0
ABCA12	GTACTGCAGTTGTGACGT	98	119	1	0	0	0
ABCA12	CATGCAGTTGACTGCAAC	5	7	0	0	0	0
ABCA12	CATGCATGGTTGGTACCA	132	233	1	0	0	0
ABCA13	TGACTGCACACAACACGT	134	142	0	6	0	0
ABCA13	GTACTGCACACAACACCA	55	55	0	0	0	0
ABCA13	ACACTGCACACAACACAC	47	69	0	0	0	0
ABCA13	CAACTGCACACAACACTG	850	660	0	0	0	0
ABCA13	GTACTGCACACAACTGGT	324	207	0	0	0	12
ABCA13	CATGCATGCAGTTGGTAC	48	61	0	0	0	0
ABCA2	TGACTGCAGTTGGTACGT	241	364	0	0	1	0
ABCA2	CAACTGCAGTTGGTCAAC	23	24	0	1	0	0
ABCA2	GTACTGCAGTTGGTCATG	143	161	0	0	0	0
ABCA2	GTACTGCAGTTGGTACCA	504	504	1	22	0	1
ABCA2	CATGCAGTTGACTGGTGT	743	927	3	1	0	1
ABCA3	ACACTGCAGTTGGTTGTG	267	193	0	0	0	1
ABCA3	GTACTGCAGTTGCAGTGT	91	37	0	0	0	0
ABCA3	CAACTGCAGTTGCAGTCA	118	110	0	0	0	0
ABCA3	TGACTGCAGTTGCAGTAC	48	30	1	0	0	0
ABCA3	ACACTGCAGTTGCAGTTG	667	818	7	0	0	0
ABCA3	CATGCACAGTCACAGTCA	1750	2056	3	3	0	1
ABCA4	CAACTGCAGTTGACGTTG	210	145	0	0	0	0
ABCA4	GTACTGCAGTTGACCAGT	79	100	0	0	0	2
ABCA4	CAACTGCAGTTGACCACA	19	146	0	0	0	0
ABCA4	TGTGCAGTTGACACCAGT	352	136	0	0	0	1
ABCA4	GTTGCAGTTGACACCACA	521	253	0	2	0	3
ABCA5	GTACTGCACATGACACTG	566	626	1	0	0	0
ABCA5	TGACTGCACATGACTGGT	926	495	0	0	0	0
ABCA5	GTACTGCACATGACTGCA	178	172	0	0	0	0
ABCA5	ACACTGCACATGACTGAC	64	39	0	0	0	0
ABCA5	CAACTGCACATGACTGTG	416	423	1	0	0	0
ABCA5	TGTGCAACTGTGCATGTG	117	121	1	0	0	0
ABCA6	CAACTGCACATGACGTCA	67	79	0	0	0	0
ABCA6	TGACTGCACATGACGTAC	44	44	0	0	0	0
ABCA6	ACACTGCACATGACGTTG	318	242	2	0	0	0
ABCA6	CAACTGCACATGACCAGT	169	190	1	1	0	0
ABCA6	ACACTGCACATGACCACA	29	51	0	0	0	0
ABCA6	GTTGCAACTGTGTGGTGT	151	228	1	0	0	1
ABCA7	ACTGACACTGGTCACATG	1140	356	1	0	1	0
ABCA7	ACACTGCACAGTGTACGT	135	84	0	0	0	0
ABCA7	TGACTGCACAGTGTACCA	414	212	3	0	0	0
ABCA7	CAACTGCACAGTGTACAC	61	69	0	1	0	0
ABCA7	GTACTGCACAGTGTACTG	326	252	0	0	0	0
ABCA8	ACTGGTACCATGCAGTGT	1809	1181	1	4	1	1
ABCA8	CATGGTACCATGCAGTAC	38	18	0	0	1	0
ABCA8	CATGGTACCATGGTTGAC	50	20	0	0	0	0
ABCA8	GTTGGTACCATGGTTGTG	480	691	1	1	0	2
ABCA8	TGTGGTACCATGCAGTCA	194	231	1	0	0	0
ABCA9	CAACTGCACAGTCACACA	410	306	1	0	0	1
ABCA9	TGACTGCACAGTCACAAC	9	1	0	0	0	0
ABCA9	ACACTGCACAGTCACATG	505	191	0	0	0	0
ABCA9	CAACTGCACAGTCAACGT	283	236	0	0	0	1
ABCA9	ACACTGCACAGTCAACCA	660	727	2	0	0	0
ABCB1	TGACTGCACAGTTGACGT	1106	270	2	0	3	1
ABCB1	GTACTGCACAGTTGCATG	68	63	0	0	0	0
ABCB1	GTACTGCACAGTTGACCA	124	118	0	0	0	0
ABCB1	ACACTGCACAGTTGACAC	30	137	1	0	0	1
ABCB1	CAACTGCACAGTTGACTG	296	216	0	0	0	0
ABCB10	CAACTGCACAACTGCATG	286	150	3	1	1	10
ABCB10	ACACTGCACAACTGCAAC	16	23	0	0	0	0
ABCB10	GTACTGCACAACTGACGT	385	524	0	0	0	0

ABCB10	CAACTGCACAACTGACCA	105	170	0	0	0	0
ABCB10	ACTGGTTGTGGTGTTGGT	592	709	2	0	0	0
ABCB10	CATGCACATGCAGTACGT	115	97	0	0	0	0
ABCB11	TGACTGCAGTTGCAACCA	132	146	0	0	0	0
ABCB11	CAACTGCAGTTGCAACAC	16	6	0	0	0	0
ABCB11	GTACTGCAGTTGCAACTG	976	1736	698	0	0	4
ABCB11	TGACTGCAGTTGCATGGT	95	134	0	0	0	0
ABCB11	GTACTGCAGTTGCATGCA	148	196	0	0	0	0
ABCB11	CATGCAACACCAGTTGCA	86	72	0	0	0	0
ABCB4	ACACTGCACACAGTACCA	779	820	0	0	2	0
ABCB4	GTACTGCACACAGTACAC	16	18	0	0	0	0
ABCB4	TGACTGCACACAGTACTG	428	526	0	0	0	0
ABCB4	ACACTGCACACAGTTGGT	185	98	0	0	0	0
ABCB4	TGACTGCACACAGTTGCA	465	193	0	0	0	0
ABCB5	TGACTGCACAACTGTGTG	660	56	0	0	0	3
ABCB5	ACACTGCACATGGTGTGT	262	307	0	0	0	0
ABCB5	TGACTGCACATGGTGTCA	319	318	1	0	0	0
ABCB5	CAACTGCACATGGTGTAC	49	77	0	0	0	0
ABCB5	GTACTGCACATGGTGTTG	80	109	0	0	0	0
ABCB6	CAACTGCACATGTGACGT	167	107	0	0	1	0
ABCB6	CAACTGCACATGTGCACA	99	90	0	0	0	0
ABCB6	TGACTGCACATGTGCAAC	21	23	0	0	0	0
ABCB6	ACACTGCACATGTGCATG	244	207	0	0	0	0
ABCB6	ACACTGCACATGTGACCA	84	108	0	1	0	0
ABCB6	CATGCACATGCAGTCACA	229	264	0	0	0	0
ABCB7	CAACTGCAGTTGCACAGT	21	17	0	0	0	0
ABCB7	ACACTGCAGTTGCACACA	270	406	0	1	0	0
ABCB7	GTACTGCAGTTGCACAAC	38	24	1	0	0	0
ABCB7	TGACTGCAGTTGCACATG	591	401	0	0	0	0
ABCB7	ACACTGCAGTTGCAACGT	207	571	46	0	0	0
ABCB8	TGACTGCACAACGTCACA	180	226	0	0	0	0
ABCB8	CAACTGCACAACGTCAAC	59	74	0	0	0	0
ABCB8	GTACTGCACAACGTCATG	148	86	0	0	0	0
ABCB8	TGACTGCACAACGTACGT	136	223	0	1	0	0
ABCB8	GTACTGCACAACGTACCA	391	384	0	0	0	0
ABCB8	TGTGCACATGCAGTCAAC	26	15	0	0	0	0
ABCB9	TGACTGCACATGGTCAGT	163	181	0	0	0	0
ABCB9	GTACTGCACATGGTCACA	60	34	0	0	0	0
ABCB9	ACACTGCACATGGTCAAC	31	46	0	0	0	0
ABCB9	CAACTGCACATGGTCATG	284	410	0	0	0	0
ABCB9	TGTGCAGTCACACACATG	311	271	1	1	0	3
ABCC1	ACACTGCAGTTGCATGAC	64	64	0	0	0	0
ABCC1	CAACTGCAGTTGCATGTG	1118	711	0	1	0	0
ABCC1	TGACTGCAGTTGACGTGT	459	300	1	0	0	0
ABCC1	GTACTGCAGTTGACGTCA	136	262	0	0	0	1
ABCC1	ACACTGCAGTTGACGTAC	15	18	0	0	0	0
ABCC1	GTTGCACATGCAGTCAGT	1150	472	2	1	0	0
ABCC2	GTACTGCAGTTGGTGTAC	106	132	0	0	1	0
ABCC2	TGACTGCAGTTGGTCACA	344	123	1	1	1	0
ABCC2	TGACTGCAGTTGGTGTTG	227	341	1	1	0	0
ABCC2	ACACTGCAGTTGGTCAGT	84	73	0	0	0	0
ABCC2	TGTGCAGTCACAACTGCA	26	40	0	0	0	0
ABCC2	TGTGCACACACATGCAAC	18	30	0	0	0	0
ABCC3	TGACTGCAGTTGACTGCA	492	268	1	0	3	4
ABCC3	ACACTGCAGTTGACTGGT	807	672	5	0	1	893
ABCC3	GTACTGCAGTTGACACAC	19	21	0	0	0	0
ABCC3	TGACTGCAGTTGACACTG	427	503	37	21	0	0
ABCC3	TGTGCAGTTGACTGGTTG	394	322	0	0	0	0
ABCC3	ACTGCACATGCAGTCATG	192	182	0	0	0	0
ABCC4	CACATGTGACCAGTTGCA	250	283	0	0	0	0
ABCC4	TGCATGTGACCAGTTGAC	27	13	0	0	0	0
ABCC4	ACCATGTGACCAGTTGTG	101	149	1	0	0	3
ABCC4	GTCATGTGACCACAGTGT	1567	903	0	2	0	0
ABCC4	CACATGTGACCACAGTCA	211	161	0	0	0	0
ABCC5	TGACTGCACATGTGGTGT	639	364	0	0	0	0
ABCC5	GTACTGCACATGTGGTCA	459	253	1	0	0	0
ABCC5	ACACTGCACATGTGGTAC	108	118	0	0	0	0
ABCC5	CAACTGCACATGTGGTTG	745	614	3	1	0	0
ABCC5	GTACTGCACATGTGCAGT	81	92	0	0	0	0
ABCC6	GTTGACACCAACCAGTCA	141	144	0	0	0	0
ABCC6	ACTGACACCAACCAGTAC	86	52	0	0	0	0
ABCC6	CATGACACCAACCAGTTG	239	683	0	1	0	0
ABCC6	GTTGACACCAACCACAGT	192	179	0	0	0	0
ABCC6	CATGACACCAACCACACA	73	88	0	0	0	0
ABCC8	TGACTGCACACATGTGGT	632	933	1	1	1	1

ABCC8	GTACTGCACACATGTGCA	73	71	0	0	0	0
ABCC8	ACACTGCACACATGTGAC	23	79	0	0	0	0
ABCC8	CAACTGCACACATGTGTG	97	99	0	0	0	0
ABCC8	CAACTGCACAACGTGTGT	602	888	0	0	0	0
ABCD1	ACACTGCAGTTGTGGTGT	1005	1329	12	3	16	12
ABCD1	GTACTGCAGTTGTGGTTG	422	452	0	0	1	0
ABCD1	CATGCACAACACTGCACA	45	75	0	0	1	0
ABCD1	TGACTGCAGTTGTGGTCA	46	152	1	0	0	0
ABCD1	CAACTGCAGTTGTGGTAC	233	139	0	0	0	2
ABCD1	TGACTGCAGTTGTGCAGT	314	346	0	0	0	1
ABCD2	CAACTGCAGTTGTGACCA	126	153	1	0	0	0
ABCD2	TGACTGCAGTTGTGACAC	40	48	0	0	0	0
ABCD2	ACACTGCAGTTGTGACTG	206	233	0	1	0	0
ABCD2	CAACTGCAGTTGTGTGGT	209	234	0	0	0	0
ABCD2	ACACTGCAGTTGTGTGCA	44	65	1	0	0	0
ABCD2	TGTGCAACACCAGTTGAC	26	22	0	1	0	0
ABCD3	TGACTGCACACATGCATG	486	637	4	1	2	25
ABCD3	ACTGCACATGCATGGTTG	265	286	0	0	1	0
ABCD3	ACACTGCACACATGACGT	118	118	0	0	0	0
ABCD3	TGACTGCACACATGACCA	128	91	0	0	0	0
ABCD3	CAACTGCACACATGACAC	24	19	0	0	0	0
ABCD3	GTACTGCACACATGACTG	416	212	1	0	0	1
ABCG1	ACTGGTACACTGACCATG	215	164	0	3	1	0
ABCG1	CATGGTACACTGACACGT	39	29	0	0	0	0
ABCG1	ACTGGTACACTGACACCA	451	462	0	1	0	0
ABCG1	GTTGGTACACTGACACAC	235	125	0	0	0	0
ABCG1	TGTGGTACACTGACACTG	311	301	0	0	0	0
ABCG2	TGACTGCACACAACGTTG	730	678	1	0	1	0
ABCG2	ACACTGCACACAACCAGT	137	202	0	0	0	0
ABCG2	TGACTGCACACAACCACA	369	199	0	0	0	0
ABCG2	CAACTGCACACAACCAAC	83	60	0	0	0	1
ABCG2	GTACTGCACACAACCATG	209	243	0	0	0	0
ABCG5	TGTGCAACGTGTACGTTG	802	877	1	0	1	0
ABCG5	CAACTGCACAGTCATGAC	116	237	0	0	0	0
ABCG5	GTACTGCACAGTCATGTG	409	842	0	2	0	0
ABCG5	ACACTGCACAGTACGTGT	467	361	1	1	0	1
ABCG5	TGACTGCACAGTACGTCA	92	99	0	0	0	0
ABCG5	CAACTGCACAGTACGTAC	70	73	0	0	0	0
ABCG8	CAACTGCACAGTACCATG	662	385	0	1	0	0
ABCG8	GTACTGCACAGTACACGT	14	8	0	0	0	0
ABCG8	CAACTGCACAGTACACCA	174	159	0	0	0	0
ABCG8	TGACTGCACAGTACACAC	25	23	0	0	0	0
ABCG8	TGTGCAGTTGACTGACGT	312	86	0	0	0	0
ABI2	ACTGGTCACACACATGCA	715	826	116	2913	1	1
ABI2	ACTGCAGTCAGTTGCATG	178	115	0	0	1	0
ABI2	GTTGGTCACACACATGAC	107	74	0	0	0	0
ABI2	CATGGTTGGTACTGCACA	8	25	1	0	0	0
ABI2	CATGCAGTCAGTTGACGT	271	348	0	0	0	4
ABL1	ACCATGACGTGTCATGTG	109	152	0	1	0	0
ABL1	GTCATGACGTGTACGTGT	164	140	5	0	0	0
ABL1	CACATGACGTGTACGTCA	67	200	0	0	0	0
ABL1	TGCATGACGTGTACGTAC	42	51	0	0	0	0
ABL1	CAACCATGCAACGTCAAC	17	19	0	0	0	0
ABL2	TGCATGACGTTGCATGCA	91	87	0	0	0	0
ABL2	CACATGACGTTGCATGAC	50	76	0	0	0	0
ABL2	GTCATGACGTTGCATGTG	438	506	2	0	0	1
ABL2	ACCATGACGTTGACGTGT	468	447	0	0	0	0
ABL2	TGCATGACGTTGACGTCA	249	171	0	0	0	0
ABLIM1	TGACTGCATGCATGCACA	186	191	0	0	0	1
ABLIM1	CAACTGCATGCATGCAAC	5	15	0	0	0	0
ABLIM1	GTACTGCATGCATGCATG	402	106	0	0	0	0
ABLIM1	TGACTGCATGCATGACGT	74	128	0	0	0	0
ABLIM1	GTACTGCATGCATGACCA	255	293	0	0	0	0
ABLIM2	ACTGGTCATGTGCAACTG	1377	1344	0	8	2	3
ABLIM2	GTTGGTCATGTGCAACGT	162	120	0	0	0	0
ABLIM2	CATGGTCATGTGCAACCA	178	188	0	0	0	0
ABLIM2	TGTGGTCATGTGCAACAC	107	34	0	0	0	0
ABLIM2	GTTGACACTGCAGTCAGT	88	110	0	0	0	0
ABLIM3	ACACGTCAGTTGGTCACA	27	45	0	0	0	0
ABLIM3	GTACGTCAGTTGGTCAAC	40	12	0	0	0	0
ABLIM3	TGACGTCAGTTGGTCATG	28	18	0	0	0	0
ABLIM3	ACACGTCAGTTGGTACGT	86	89	3	0	0	109
ABLIM3	TGACGTCAGTTGGTACCA	787	775	1	0	0	0
ABO	TGACCACAACTGTGTGCA	533	219	1	0	0	0
ABO	CAACCACAACTGTGTGAC	89	46	0	0	0	0

ABO	GTACCACAACTGTGTGTG	208	260	0	0	0	0
ABO	TGACCACATGGTGTGTGT	518	699	2	0	0	13
ABO	GTACCACATGGTGTGTCA	635	829	0	1	0	0
ABP1	GTACACGTTGGTTGCACA	121	151	0	0	2	12
ABP1	GTACACGTTGGTTGGTTG	383	526	0	0	0	1
ABP1	TGACACGTTGGTTGCAGT	207	161	0	0	0	2
ABP1	ACACACGTTGGTTGCAAC	22	10	0	0	0	0
ABP1	TGTGACCACAACCACAGT	252	171	1	0	0	0
ACAA1	GTACCAACCAGTGTCAGT	363	347	0	0	2	3
ACAA1	CAACCAACCAGTGTCACA	97	172	0	0	0	0
ACAA1	TGACCAACCAGTGTCAAC	6	5	0	0	0	0
ACAA1	ACACCAACCAGTGTCATG	156	123	0	1	0	0
ACAA1	CAACCAACCAGTGTACGT	517	622	1	0	0	127
ACAA1	CATGCACAACACGTTGGT	53	303	0	0	0	0
ACAA2	GTACCAACCAGTCAACGT	400	819	9	0	0	0
ACAA2	CAACCAACCAGTCAACCA	256	390	1	0	0	3
ACAA2	TGACCAACCAGTCAACAC	98	69	3	0	0	9
ACAA2	ACACCAACCAGTCAACTG	368	413	5	2	0	2
ACAA2	CAACCAACCAGTCATGGT	527	267	0	0	0	0
ACAA2	ACTGCACAACGTACGTAC	75	112	0	0	0	0
ACACA	GTCATGTGCACAACTGGT	317	395	0	0	0	0
ACACA	CACATGTGCACAACTGCA	253	335	0	0	0	0
ACACA	TGCATGTGCACAACTGAC	96	38	0	0	0	0
ACACA	ACCATGTGCACAACTGTG	158	582	2	0	0	0
ACACA	GTCATGTGCACATGGTGT	118	212	0	0	0	0
ACACB	TGCATGACACCACACAAC	37	22	0	0	0	0
ACACB	ACCATGACACCACACATG	138	352	1	1	0	0
ACACB	CACATGACACCACAACGT	368	209	1	0	0	0
ACACB	GTACCAGTCATGTGTGCA	215	279	1	0	0	7
ACACB	ACACCAGTCATGTGTGAC	19	33	0	0	0	0
ACAD8	GTACGTTGGTGTACGTTG	107	57	0	0	0	0
ACAD8	TGACGTTGGTGTACCAGT	82	77	0	1	0	0
ACAD8	GTACGTTGGTGTACCACA	136	74	0	0	0	0
ACAD8	ACACGTTGGTGTACCAAC	27	19	0	0	0	0
ACAD8	CAACGTTGGTGTACCATG	247	141	0	0	0	0
ACAD8	ACTGCACAACACTGTGGT	459	326	0	0	0	1
ACAD9	GTACCAGTCACAGTCAAC	75	169	1	0	0	0
ACAD9	TGACCAGTCACAGTCATG	383	395	0	0	0	4
ACAD9	ACACCAGTCACAGTACGT	424	378	1	0	0	0
ACAD9	TGACCAGTCACAGTACCA	218	181	0	0	0	0
ACAD9	CAACCAGTCACAGTACAC	30	23	0	0	0	0
ACADL	ACACCAGTCAACACGTTG	902	851	2	0	0	0
ACADL	CAACCAGTCAACACCAGT	140	166	1	0	0	0
ACADL	ACACCAGTCAACACCACA	198	120	0	0	0	3
ACADL	GTACCAGTCAACACCAAC	21	26	0	0	0	3
ACADL	TGACCAGTCAACACCATG	392	57	0	0	0	0
ACADM	ACACCAGTCAACCAACAC	202	187	0	0	0	0
ACADM	CAACCAGTCAACCAACTG	845	682	0	0	0	6
ACADM	GTACCAGTCAACCATGGT	633	390	0	0	0	0
ACADM	CAACCAGTCAACCATGCA	701	495	0	2	0	0
ACADM	TGACCAGTCAACCATGAC	17	42	0	0	0	0
ACADM	GTTGCAACCATGTGTGTG	161	247	1	0	0	0
ACADS	GTACCAGTCAACGTTGAC	11	7	0	0	0	0
ACADS	TGACCAGTCAACGTTGTG	225	366	0	1	0	0
ACADS	CAACCAGTCAACCAGTGT	1261	1994	2	2	0	2
ACADS	ACACCAGTCAACCAGTCA	198	197	0	0	0	0
ACADS	GTACCAGTCAACACTGCA	15	9	0	0	0	0
ACADS	TGTGCACACACACACACA	254	194	41	0	0	0
ACADSB	CAACCAGTCAACGTACCA	464	411	0	0	0	1
ACADSB	TGACCAGTCAACGTACAC	5	10	0	0	0	0
ACADSB	ACACCAGTCAACGTACTG	68	41	0	0	0	0
ACADSB	CAACCAGTCAACGTTGGT	268	347	1	0	0	1
ACADSB	ACACCAGTCAACGTTGCA	278	256	1	0	0	0
ACADVL	GTACCAGTCAACGTCACA	183	351	0	5	1	0
ACADVL	ACACCAGTCAACGTCAAC	47	96	0	0	0	0
ACADVL	CAACCAGTCAACGTCATG	178	163	0	0	0	0
ACADVL	GTACCAGTCAACGTACGT	146	198	0	0	0	0
ACADVL	TGACCAGTCAACACTGGT	256	214	0	0	0	0
ACAN	GTTGGTTGCAGTCATGGT	283	200	1	0	1	0
ACAN	CATGGTTGCAGTCATGCA	352	217	1	0	1	0
ACAN	CAACTGACTGACCAGTAC	57	59	1	0	0	0
ACAN	CATGGTTGCAGTCAACTG	169	264	0	1	0	0
ACAN	TGTGGTTGCAGTCATGAC	209	200	0	0	0	1
ACAT1	ACACCAACGTGTGTGTGT	293	231	0	0	1	0
ACAT1	CAACCACATGTGTGTGGT	128	254	0	0	0	0

ACAT1	ACACCACATGTGTGTGCA	42	30	0	0	0	0
ACAT1	GTACCACATGTGTGTGAC	39	62	0	0	0	0
ACAT1	TGACCACATGTGTGTGTG	20	31	0	0	0	0
ACAT1	TGTGCATGCAGTACACTG	285	306	2	0	0	0
ACAT2	CATGCACAACGTCATGTG	245	425	0	0	1	76
ACAT2	CAACCAACGTGTCATGCA	215	363	2	0	0	1
ACAT2	TGACCAACGTGTCATGAC	35	59	0	0	0	0
ACAT2	ACACCAACGTGTCATGTG	186	190	0	0	0	0
ACAT2	GTACCAACGTGTACGTGT	684	541	1	1	0	1
ACAT2	CAACCAACGTGTACGTCA	41	41	1	0	0	0
ACCN1	CAACACGTGTACCACACA	75	54	0	0	0	2
ACCN1	TGACACGTGTACCACAAC	9	19	0	0	0	0
ACCN1	ACACACGTGTACCACATG	100	143	0	0	0	0
ACCN1	CAACACGTGTACCAACGT	105	401	0	0	0	0
ACCN1	ACACACGTGTACCAACCA	60	103	0	0	0	0
ACCN2	ACTGGTTGCAGTCATGTG	410	442	2	1	1	0
ACCN2	CATGGTTGCAGTACGTCA	330	283	0	0	1	0
ACCN2	GTTGGTTGCAGTACGTGT	204	190	1	0	0	0
ACCN2	TGTGGTTGCAGTACGTAC	21	247	0	0	0	1
ACCN2	ACTGGTTGCAGTACGTTG	275	218	0	0	0	0
ACCN2	ACTGCAACACCAGTACAC	29	9	0	0	0	0
ACCN3	ACTGGTTGACTGACGTTG	122	145	1	0	0	0
ACCN3	CATGGTTGACTGACCAGT	181	63	0	0	0	0
ACCN3	ACTGGTTGACTGACCACA	53	58	0	0	0	0
ACCN3	GTTGGTTGACTGACCAAC	4	7	0	0	0	0
ACCN3	TGTGGTTGACTGACCATG	176	164	0	1	0	0
ACCN3	CATGCACATGCAGTTGAC	14	6	0	0	0	0
ACCN4	ACTGGTACGTACTGACTG	192	332	0	0	0	0
ACCN4	CATGGTACGTACTGTGGT	397	337	0	0	0	0
ACCN4	ACTGGTACGTACTGTGCA	327	426	1	0	0	0
ACCN4	GTTGGTACGTACTGTGAC	56	29	0	0	0	0
ACCN4	TGTGGTACGTACTGTGTG	567	909	0	0	0	1
ACCN5	CATGGTACACCAGTGTAC	93	86	0	0	1	0
ACCN5	TGTGGTACACCAGTGTCA	124	68	0	0	0	0
ACCN5	GTTGGTACACCAGTGTTG	544	514	3	1	0	1
ACCN5	CATGACACGTACGTCACA	37	63	0	0	0	0
ACCN5	TGTGACACGTACGTCAAC	48	35	0	0	0	0
ACE	ACTGCAACTGGTGTTGTG	561	706	1	0	1	0
ACE	ACACCAGTTGTGCAGTAC	71	61	0	0	0	0
ACE	CAACACGTTGTGACTGCA	90	102	0	0	0	0
ACE	TGACACGTTGTGACTGAC	69	127	0	0	0	0
ACE	GTTGCAACTGGTCAGTGT	261	203	0	0	0	0
ACE	CATGCAACTGGTCAGTCA	192	188	0	0	0	1
ACE2	CAACCAGTTGTGCAGTTG	1300	1096	5	10	2	62
ACE2	TGACACGTTGTGTGTGGT	305	359	0	0	0	0
ACE2	GTACACGTTGTGTGTGCA	322	118	0	0	0	0
ACE2	ACACACGTTGTGTGTGAC	38	15	0	0	0	0
ACE2	CAACACGTTGTGTGTGTG	106	48	0	0	0	0
ACE2	GTTGCACATGACGTACCA	186	76	1	0	0	0
ACHE	TGACACCAGTGTACGTCA	416	500	64	1	0	0
ACHE	CAACACCAGTGTACGTAC	290	52	1	1	0	0
ACHE	GTACACCAGTGTACGTTG	156	204	1	0	0	0
ACHE	TGACACCAGTGTACCAGT	94	85	0	0	0	0
ACHE	TGTGCAGTTGGTACCACA	99	49	0	0	0	0
ACHE	GTTGCACAACTGTGACTG	401	370	1	1	0	0
ACIN1	CAACTGTGGTTGCATGGT	446	351	0	0	0	0
ACIN1	ACACTGTGGTTGCATGCA	176	94	0	0	0	0
ACIN1	GTACTGTGGTTGCATGAC	23	12	0	0	0	0
ACIN1	TGACTGTGGTTGCATGTG	542	510	0	1	0	0
ACIN1	CAACTGTGGTTGACGTGT	921	811	4	0	0	0
ACLY	GTACTGTGCATGCATGTG	64	75	0	0	0	0
ACLY	ACACTGTGCATGACGTGT	592	256	0	1	0	0
ACLY	TGACTGTGCATGACGTCA	178	330	0	1	0	0
ACLY	CAACTGTGCATGACGTAC	86	64	1	0	0	0
ACLY	GTTGCAGTCACAGTGTAC	240	347	4	0	0	0
ACMSD	GTACTGTGACGTTGCATG	359	303	1	0	1	0
ACMSD	TGACTGTGACGTTGGTTG	384	352	0	1	0	0
ACMSD	ACACTGTGACGTTGCAGT	372	279	1	0	0	7
ACMSD	TGACTGTGACGTTGCACA	147	55	0	0	0	0
ACMSD	CAACTGTGACGTTGCAAC	24	7	0	0	0	0
ACO1	ACACACTGCAACTGGTCA	606	545	0	1	0	4
ACO1	GTACACTGCAACTGGTAC	56	60	0	0	0	0
ACO1	TGACACTGCAACTGGTTG	414	591	1	0	0	0
ACO1	ACACACTGCAACTGCAGT	238	276	0	0	0	0
ACO1	TGACACTGCAACTGCACA	141	87	0	0	0	1

ACO1	GTTGCACATGGTCAGTCA	535	459	0	0	0	0
ACO2	CAACACTGCAACTGCAAC	31	34	0	0	0	0
ACO2	GTACACTGCAACTGCATG	449	104	1	0	0	0
ACO2	TGACACTGCAACTGACGT	85	112	0	0	0	0
ACO2	GTACACTGCAACTGACCA	66	47	0	0	0	0
ACO2	ACACACTGCAACTGACAC	51	45	0	0	0	0
ACOT11	TGACACCAACGTTGTGGT	117	185	0	0	0	0
ACOT11	GTACACCAACGTTGTGCA	634	320	0	0	0	0
ACOT11	ACACACCAACGTTGTGAC	44	42	0	0	0	0
ACOT11	CAACACCAACGTTGTGTG	201	255	0	0	0	6
ACOT11	ACACACCAACCAGTGTGT	698	137	1	2	0	0
ACOT11	CATGCAACTGACACTGTG	364	543	1	1	0	0
ACOT12	TGACACCACATGCAGTCA	520	443	1	0	1	0
ACOT12	CAACACCACATGGTTGAC	15	15	0	0	0	0
ACOT12	GTACACCACATGGTTGTG	293	521	0	1	0	0
ACOT12	ACACACCACATGCAGTGT	977	981	1	1	0	0
ACOT12	CAACACCACATGCAGTAC	66	79	0	0	0	0
ACOT12	TGTGCAACCAGTCAGTGT	691	604	1	1	0	3
ACOX1	ACACACGTTGCAGTCACA	139	170	0	0	0	0
ACOX1	GTACACGTTGCAGTCAAC	11	7	0	0	0	0
ACOX1	TGACACGTTGCAGTCATG	85	154	1	0	0	0
ACOX1	ACACACGTTGCAGTACGT	109	77	0	0	0	0
ACOX1	CATGCAGTTGGTCATGGT	976	702	4	0	0	8
ACOX2	TGACACGTTGCAGTACCA	182	131	0	0	0	0
ACOX2	CAACACGTTGCAGTACAC	18	25	0	0	0	0
ACOX2	GTACACGTTGCAGTACTG	515	195	0	1	0	2
ACOX2	TGACACGTTGCAGTTGGT	49	71	0	0	0	0
ACOX2	GTACACGTTGCAGTTGCA	113	117	0	0	0	0
ACOX3	CAACACGTTGGTACCACA	60	91	0	0	0	0
ACOX3	TGACACGTTGGTACCAAC	17	6	0	0	0	0
ACOX3	ACACACGTTGGTACCATG	214	380	0	0	0	1
ACOX3	CAACACGTTGGTACACGT	207	131	0	0	0	0
ACOX3	ACACACGTTGGTACACCA	163	159	0	0	0	0
ACOX3	TGTGCACAGTCACAGTAC	23	24	0	0	0	0
ACP1	CATGACCATGTGGTCAAC	33	25	0	0	0	0
ACP1	GTTGACCATGTGGTCATG	97	128	0	0	0	0
ACP1	TGTGACCATGTGGTACGT	498	136	0	0	0	0
ACP1	GTTGACCATGTGGTACCA	102	71	0	0	0	0
ACP1	ACTGACCATGTGGTACAC	26	36	0	0	0	0
ACP2	CAACACACCACAGTACTG	887	352	18	3	17	12
ACP2	TGTGCACACACACAGTTG	1348	1089	1	17	1	1
ACP2	GTACACACCACAGTACCA	323	580	1	1	0	0
ACP2	ACACACACCACAGTACAC	45	49	0	0	0	0
ACP2	GTACACACCACAGTTGGT	498	471	0	0	0	0
ACP2	ACTGCACACACACACAGT	659	614	1	1	0	0
ACP5	GTACACACGTCAGTTGCA	341	215	0	0	0	0
ACP5	ACACACACGTCAGTTGAC	50	166	0	0	0	0
ACP5	CAACACACGTCAGTTGTG	665	134	0	0	0	0
ACP5	TGACACACGTCACAGTGT	251	328	2	0	0	0
ACP5	GTACACACGTCACAGTCA	805	822	0	0	0	1
ACP5	TGTGCACAACCATGTGAC	40	30	0	0	0	0
ACP6	CAACACACTGCATGACGT	337	226	10	23	16	11969
ACP6	TGACACACTGCATGCAAC	140	158	0	0	0	0
ACP6	ACACACACTGCATGCATG	468	504	0	0	0	0
ACP6	ACACACACTGCATGACCA	676	610	1	1	0	1
ACP6	GTACACACTGCATGACAC	337	55	0	0	0	0
ACP6	GTTGCAACACGTGTACGT	68	43	0	0	0	0
ACPP	CACATGACCAACTGACTG	212	171	1	0	0	0
ACPP	GTCATGACCAACTGTGGT	151	262	0	0	0	4
ACPP	CACATGACCAACTGTGCA	274	294	0	0	0	0
ACPP	TGCATGACCAACTGTGAC	53	86	0	0	0	0
ACPP	CATGCAGTACGTACACCA	106	134	0	0	0	0
ACPT	CATGCAACGTTGTGTGGT	217	188	0	0	1	1
ACPT	ACACACACTGCATGGTAC	52	41	0	1	0	0
ACPT	CAACACACTGCATGGTTG	648	459	0	0	0	0
ACPT	ACTGCAACGTTGTGTGCA	123	157	0	0	0	0
ACPT	GTTGCAACGTTGTGTGAC	76	88	0	0	0	0
ACPT	TGTGCAACGTTGTGTGTG	407	447	1	0	0	0
ACSL1	CAACACGTCAACGTTGCA	88	230	1	0	0	0
ACSL1	TGACACGTCAACGTTGAC	50	65	0	0	0	0
ACSL1	ACACACGTCAACGTTGTG	305	535	1	0	0	0
ACSL1	GTACACGTCAACCAGTGT	1035	997	1	1	0	0
ACSL1	CAACACGTCAACCAGTCA	601	559	1	0	0	0
ACSL3	TGACACGTCAACCACATG	162	129	0	0	0	0
ACSL3	ACACACGTCAACCAACGT	489	506	0	0	0	1

ACSL3	TGACACGTCAACCAACCA	46	83	0	0	0	0
ACSL3	CAACACGTCAACCAACAC	63	82	0	1	0	0
ACSL3	GTACACGTCAACCAACTG	44	49	3	0	0	0
ACSL4	TGACGTCAACCACACAAC	37	90	0	0	0	0
ACSL4	GTACACGTCAACACGTCA	78	90	0	0	0	0
ACSL4	ACACACGTCAACACGTAC	62	67	0	0	0	0
ACSL4	CAACACGTCAACACGTTG	492	473	0	0	0	1
ACSL4	GTACACGTCAACACCAGT	32	54	0	0	0	0
ACSL4	GTTGCACATGACTGCACA	29	36	0	0	0	0
ACSL5	ACACACGTCAACACCATG	128	178	0	0	0	0
ACSL5	CAACACGTCAACACACGT	130	40	0	0	0	0
ACSL5	ACACACGTCAACACACCA	226	424	0	0	0	0
ACSL5	GTACACGTCAACACACAC	51	59	0	0	0	0
ACSL5	TGACACGTCAACACACTG	277	530	0	0	0	0
ACSL6	ACACACGTACACACACAC	12	22	0	0	0	0
ACSL6	CAACACGTACACACACTG	203	503	0	0	0	0
ACSL6	GTACACGTACACACTGGT	129	108	1	0	0	0
ACSL6	CAACACGTACACACTGCA	511	189	0	0	0	0
ACSL6	TGACACGTACACACTGAC	72	71	0	1	0	0
ACSM1	GTACACGTACGTGTCACA	120	22	0	0	0	0
ACSM1	ACACACGTACGTGTCAAC	8	6	0	0	0	2
ACSM1	CAACACGTACGTGTCATG	90	181	0	1	0	0
ACSM1	GTACACGTACGTGTACGT	47	50	0	0	0	0
ACSM1	CATGCAACGTACCACACA	50	165	0	0	0	0
ACSM1	TGTGCAACGTACCACAAC	22	22	0	0	0	0
ACSS1	TGACACGTCACAGTACTG	369	253	0	0	0	0
ACSS1	ACACACGTCACAGTTGGT	870	534	2	2	0	1
ACSS1	TGACACGTCACAGTTGCA	211	164	0	0	0	1
ACSS1	CAACACGTCACAGTTGAC	181	64	0	0	0	0
ACSS1	GTACACGTCACAGTTGTG	256	194	0	0	0	0
ACSS1	TGTGCATGGTCACAGTCA	378	402	4	0	0	0
ACSS2	GTACACGTCAACACTGTG	100	416	0	0	0	5
ACSS2	ACACACGTCAACTGGTGT	779	697	2	0	0	0
ACSS2	TGACACGTCAACTGGTCA	339	162	0	0	0	1
ACSS2	CAACACGTCAACTGGTAC	72	143	0	0	0	1
ACSS2	ACACTGTGCACAACGTAC	91	116	0	0	0	0
ACSS2	CATGCAACCATGACTGTG	202	430	1	0	0	0
ACTA1	GTTGGTGTTGACCATGCA	202	163	0	1	1	0
ACTA1	TGTGTGACACACTGTGTG	126	221	1	0	1	14
ACTA1	TGTGTGACACTGGTCAGT	149	212	0	0	1	0
ACTA1	CATGGTGTTGACCAACAC	132	101	0	0	0	0
ACTA1	GTTGGTGTTGACCAACTG	138	205	0	0	0	0
ACTA1	TGTGGTGTTGACCATGGT	163	260	0	0	0	0
ACTA1	ACTGGTGTTGACCATGAC	40	50	0	0	0	1
ACTA1	ACTGTGACACCAACTGTG	691	314	0	0	0	0
ACTA1	GTTGTGACACCATGGTGT	537	435	0	1	0	0
ACTA1	CATGTGACACCATGGTCA	213	469	1	0	0	1
ACTA1	TGTGTGACACACACCAAC	113	47	0	0	0	0
ACTA1	ACTGTGACACACACCATG	133	120	0	0	0	9
ACTA1	CATGTGACACACACACGT	47	105	1	0	0	0
ACTA1	ACTGTGACACTGGTGTGT	567	575	5	0	0	0
ACTA1	TGTGTGACACTGGTGTCA	94	244	0	0	0	0
ACTA1	GTTGTGACACTGGTCACA	68	29	0	0	0	0
ACTA1	ACTGTGACACTGGTCAAC	10	17	0	1	0	0
ACTA1	CATGTGACACTGGTACCA	370	282	1	1	0	0
ACTA1	TGTGTGACACTGGTACAC	27	17	0	0	0	0
ACTA1	ACTGTGACACTGGTACTG	457	406	1	0	0	1
ACTA1	GTTGTGACACTGGTTGAC	38	50	0	0	0	0
ACTA1	TGTGTGACACTGGTTGTG	375	231	0	0	0	0
ACTA1	CATGTGACACTGCAGTGT	574	462	1	1	0	0
ACTA1	TGTGTGACACTGCAGTTG	458	857	3	0	0	0
ACTA1	ACTGTGACACTGCACAGT	198	130	1	1	0	2
ACTA1	TGTGTGACACTGCACACA	16	29	0	0	0	0
ACTA2	GTTGGTGTTGGTTGTGGT	103	149	0	1	0	0
ACTA2	CATGGTGTTGGTTGTGCA	251	207	0	0	0	0
ACTA2	TGTGGTGTTGGTTGTGAC	8	7	0	0	0	0
ACTA2	ACTGGTGTTGGTTGTGTG	168	157	1	0	0	0
ACTA2	TGTGGTGTTGCAGTGTGT	441	767	1	1	0	0
ACTA2	ACTGCACAGTCACAGTTG	1638	1122	0	0	0	30
ACTB	GTACCAGTACTGCAACTG	703	771	0	1	1	3
ACTB	ACTGTGACACCATGTGAC	44	74	1	0	1	0
ACTB	ACTGTGTGGTCAGTTGTG	468	375	26	13284	1	3
ACTB	CAACCAGTACTGCAACAC	44	41	0	0	0	0
ACTB	TGACCAGTACTGCATGGT	1411	295	2	1	0	1
ACTB	GTACCAGTACTGCATGCA	373	458	1	0	0	0

ACTB	ACTGGTGTCAGTGTGTAC	76	85	0	1	0	0
ACTB	TGTGCACAACGTGTGTTG	91	198	0	0	0	0
ACTB	CATGTGTGGTCACAGTCA	477	575	1	0	0	0
ACTB	CATGTGTGGTCACACAGT	66	107	0	0	0	0
ACTB	ACTGTGTGGTCACACACA	36	35	0	0	0	3
ACTB	GTTGTGTGGTCACACAAC	1	0	0	0	0	0
ACTB	TGTGTGTGGTCACAACCA	113	87	0	0	0	0
ACTB	CATGTGTGGTCACAACAC	57	87	0	0	0	0
ACTB	GTTGTGTGGTCACAACTG	151	132	0	0	0	0
ACTB	CATGTGACACCATGCAGT	279	568	1	0	0	0
ACTB	ACTGTGACACCATGCACA	219	335	0	0	0	2
ACTB	GTTGTGACACCATGCAAC	14	28	0	0	0	0
ACTB	TGTGTGACACCATGACCA	189	326	0	0	0	0
ACTB	CATGTGACACCATGACAC	109	88	0	0	0	1
ACTB	GTTGTGACACCATGACTG	314	317	0	0	0	0
ACTB	CATGTGACACCATGTGTG	231	140	0	0	0	0
ACTB	CATGTGACACACGTGTGT	269	317	1	1	0	0
ACTB	CATGTGACTGGTCAACCA	141	201	0	0	0	0
ACTB	TGTGTGACTGGTCAACAC	18	25	0	0	0	0
ACTB	ACTGTGACTGGTCAACTG	328	261	1	2	0	7
ACTB	GTTGTGTGGTCACAGTGT	333	331	2	0	0	0
ACTG1	TGACCAGTTGGTGTCAGT	483	167	0	2	1	0
ACTG1	GTACCAGTTGGTGTGTTG	443	477	1	0	0	0
ACTG1	GTACCAGTTGGTGTCACA	29	39	1	0	0	0
ACTG1	ACACCAGTTGGTGTCAAC	3	2	0	0	0	0
ACTG1	CAACCAGTTGGTGTCATG	305	260	0	2	0	0
ACTG1	CATGCACATGCATGACAC	22	38	0	0	0	0
ACTG2	GTTGGTGTTGGTGTGTGT	621	889	1	0	1	1
ACTG2	TGTGGTGTACTGTGTGGT	95	167	0	0	0	0
ACTG2	GTTGGTGTACTGTGTGCA	201	190	0	1	0	10
ACTG2	ACTGGTGTACTGTGTGAC	48	40	0	0	0	0
ACTG2	CATGGTGTACTGTGTGTG	928	344	0	1	0	0
ACTN1	GTACACTGCAGTACCATG	218	166	2	0	0	0
ACTN1	TGACACTGCAGTACACGT	43	71	0	0	0	0
ACTN1	GTACACTGCAGTACACCA	125	265	0	0	0	1
ACTN1	ACACACTGCAGTACACAC	119	141	1	0	0	0
ACTN1	GTTGCAGTTGCATGCAGT	157	225	1	0	0	0
ACTN1	CATGCACATGACACGTTG	153	190	0	0	0	0
ACTN2	ACACACTGCAGTACTGTG	427	797	0	1	1	0
ACTN2	CAACACTGCAGTTGGTCA	362	305	1	0	1	0
ACTN2	GTACACTGCAGTTGGTGT	495	570	1	0	0	1
ACTN2	TGACACTGCAGTTGGTAC	161	78	0	0	0	0
ACTN2	ACACACTGCAGTTGGTTG	781	474	1	0	0	0
ACTN2	CATGCACAGTCACACAGT	166	440	1	0	0	0
ACTN3	GTACACTGCAGTTGACTG	795	453	0	0	1	1
ACTN3	ACACACTGCAGTTGACGT	216	269	0	0	0	0
ACTN3	TGACACTGCAGTTGACCA	147	144	1	0	0	0
ACTN3	CAACACTGCAGTTGACAC	21	15	0	0	0	0
ACTN3	TGACACTGCAGTTGTGGT	109	116	0	0	0	0
ACTN3	CATGCACACACACACAAC	77	143	0	0	0	0
ACTN4	CAACACTGCAGTCATGGT	778	1094	1	1	1	0
ACTN4	GTACACTGCAGTCAACGT	165	163	0	0	0	0
ACTN4	CAACACTGCAGTCAACCA	113	181	0	0	0	0
ACTN4	TGACACTGCAGTCAACAC	91	221	0	0	0	0
ACTN4	ACACACTGCAGTCAACTG	638	480	1	0	0	0
ACTR2	TGTGGTGTACCAGTCATG	467	226	0	0	3	0
ACTR2	ACTGGTGTACCAGTCACA	90	154	0	0	1	0
ACTR2	CATGGTGTACCAGTCAGT	365	249	2	0	0	1
ACTR2	GTTGGTGTACCAGTCAAC	15	11	0	0	0	0
ACTR2	ACTGGTGTACCAGTACGT	74	80	0	0	0	0
ACTR3	GTACCAGTACTGGTGTAC	101	120	0	0	0	5
ACTR3	TGACCAGTACTGGTGTTG	188	315	0	0	0	0
ACTR3	ACACCAGTACTGGTCAGT	244	481	0	0	0	10
ACTR3	TGACCAGTACTGGTCACA	33	48	0	0	0	0
ACTR3	CAACCAGTACTGGTCAAC	27	50	0	0	0	0
ACVR1	TGCATGCACATGCATGCA	4	73	0	0	0	0
ACVR1	CACATGCACATGCATGAC	41	92	0	0	0	0
ACVR1	GTCATGCACATGCATGTG	51	290	0	0	0	0
ACVR1	ACCATGCACATGACGTGT	504	802	0	1	0	5
ACVR1	TGCATGCACATGACGTCA	228	356	0	0	0	0
ACVR1	GTTGCACATGCAACCATG	331	47	1	0	0	0
ACVR1B	CACATGACGTACGTACGT	892	739	1	1	0	0
ACVR1B	ACCATGACGTACGTACCA	108	90	0	0	0	0
ACVR1B	GTCATGACGTACGTACAC	89	39	0	0	0	0
ACVR1B	TGCATGACGTACGTACTG	114	59	0	0	0	0

ACVR1B	ACCATGACGTACGTTGGT	425	178	1	0	0	0
ACVR1C	TGCATGACGTACGTTGCA	262	39	0	266	1	0
ACVR1C	CACATGACGTACGTTGAC	223	66	1	82	1	0
ACVR1C	GTCATGACGTACGTTGTG	154	495	0	0	0	0
ACVR1C	ACCATGACGTACCAGTGT	1157	393	0	0	0	0
ACVR1C	TGCATGACGTACCAGTCA	421	191	0	2	0	1
ACVR1C	GTTGCAACACCAACACAC	163	42	0	0	0	5
ACVR2A	GTCATGCAACCAGTGTCA	655	517	1	45	3	3
ACVR2A	ACCATGCAACCAGTGTAC	220	250	0	0	0	5
ACVR2A	CACATGCAACCAGTGTTG	341	240	0	1	0	0
ACVR2A	GTCATGCAACCAGTCAGT	438	275	2	0	0	3
ACVR2A	CACATGCAACCAGTCACA	57	74	0	0	0	0
ACVR2B	CACATGCACATGACGTAC	21	24	0	0	0	1
ACVR2B	GTCATGCACATGACGTTG	261	271	1	0	0	0
ACVR2B	TGCATGCACATGACCAGT	266	224	1	0	0	0
ACVR2B	GTCATGCACATGACCACA	240	119	1465	2	0	0
ACVR2B	ACCATGCACATGACCAAC	108	38	0	0	0	0
ACVRL1	GTCATGCACAACCACACA	207	232	0	0	0	0
ACVRL1	ACCATGCACAACCACAAC	28	33	0	0	0	0
ACVRL1	CACATGCACAACCACATG	446	444	0	0	0	8
ACVRL1	ACTGGTTGGTGTCAGTTG	468	468	3	1	0	0
ACVRL1	CATGGTTGGTGTCACAGT	223	165	0	0	0	0
ACVRL1	GTTGCACACACACACATG	119	111	0	0	0	0
ACY1	TGACACACACGTACTGTG	167	163	0	0	1	0
ACY1	ACACACACACGTACTGCA	165	100	0	0	0	0
ACY1	GTACACACACGTACTGAC	47	83	0	0	0	0
ACY1	CAACACACACGTTGGTGT	156	159	0	1	0	0
ACY1	CATGCAGTCAGTACTGTG	219	250	1	0	0	0
ACY1	ACTGCACAGTCACACACA	248	275	1	0	0	2
ACY3	ACACACCACATGACTGTG	354	222	1	0	1	0
ACY3	GTACACCACATGTGGTGT	263	171	0	0	1	19
ACY3	CAACACCACATGTGGTCA	885	887	11	5	1	14556
ACY3	CAACACCACATGACTGCA	116	239	0	0	0	0
ACY3	TGACACCACATGACTGAC	165	158	0	0	0	0
ACY3	ACTGCAACGTTGACCATG	99	75	0	0	0	0
ACYP1	GTACACACGTGTCAGTGT	301	273	40	29	92	90
ACYP1	ACACACACGTGTGTTGTG	237	203	0	0	0	0
ACYP1	CAACACACGTGTCAGTCA	132	183	0	1	0	0
ACYP1	TGACACACGTGTCAGTAC	125	81	0	0	0	0
ACYP1	ACACACACGTGTCAGTTG	326	167	0	0	0	13
ACYP2	TGACACACCACACACATG	220	217	1	0	0	0
ACYP2	ACACACACCACACAACGT	68	142	0	0	0	0
ACYP2	TGACACACCACACAACCA	493	262	2	0	0	14
ACYP2	CAACACACCACACAACAC	73	139	1	1	0	0
ACYP2	TGTGCAGTCAACACACGT	81	67	0	0	0	0
ADA	ACACACACCATGCATGGT	365	247	3	0	0	0
ADA	TGACACACCATGCATGCA	558	594	2	1	0	0
ADA	CAACACACCATGCATGAC	188	57	0	0	0	0
ADA	ACTGCAGTTGGTTGTGAC	67	106	0	0	0	0
ADA	CATGACGTCAGTACACGT	37	28	0	0	0	0
ADAM10	TGACGTGTGTTGTGACCA	392	291	3	0	0	3
ADAM10	CAACGTGTGTTGTGACAC	37	99	0	0	0	0
ADAM10	GTACGTGTGTTGTGACTG	321	279	0	0	0	0
ADAM10	TGACGTGTGTTGTGTGGT	688	1111	1	0	0	1
ADAM10	GTACGTGTGTTGTGTGCA	185	204	0	0	0	0
ADAM12	ACACACCAGTCAACTGTG	342	335	1	0	0	0
ADAM12	GTACACCAGTCATGGTGT	230	258	0	1	0	0
ADAM12	CAACACCAGTCATGGTCA	190	134	1	0	0	0
ADAM12	CATGACCACAACTGGTCA	42	46	0	0	0	0
ADAM12	TGTGACCACAACTGGTAC	69	111	0	0	0	0
ADAM17	TGACACACACTGGTGTCA	101	126	0	0	0	0
ADAM17	CAACACACACTGGTGTAC	259	167	0	0	0	0
ADAM17	GTACACACACTGGTGTTG	176	107	0	1	0	0
ADAM17	TGACACACACTGGTCAGT	264	465	1	0	0	2
ADAM17	ACTGGTGTGTTGACCAAC	60	18	0	0	0	0
ADAM8	ACACGTGTGTTGTGCACA	202	271	0	0	1	1
ADAM8	GTACGTGTGTTGTGCAAC	21	25	0	0	0	0
ADAM8	TGACGTGTGTTGTGCATG	329	207	2	0	0	1
ADAM8	ACACGTGTGTTGTGACGT	74	171	0	0	0	0
ADAM8	GTTGACCATGCAGTCACA	195	229	0	0	0	0
ADAM9	TGACACCAGTCAACGTTG	1005	444	1	0	1	0
ADAM9	CAACCAGTTGTGCAACGT	682	516	1	0	0	1
ADAM9	ACACACCAGTCAACGTCA	275	349	0	0	0	0
ADAM9	GTACACCAGTCAACGTAC	30	76	1	0	0	0
ADAM9	ACACACCAGTCAACCAGT	164	146	0	0	0	0

ADAMTS13	ACACACTGGTCATGCAAC	12	5	0	0	0	0
ADAMTS13	CAACACTGGTCATGCATG	35	67	0	1	0	0
ADAMTS13	GTACACTGGTCATGACGT	223	198	0	0	0	1
ADAMTS13	CAACACTGGTCATGACCA	492	231	0	2	0	0
ADAMTS13	TGACACTGGTCATGACAC	107	161	0	0	0	0
ADAR	CAACACACGTACCAACTG	243	352	0	0	1	0
ADAR	GTACACACGTACCATGGT	59	226	0	0	0	1
ADAR	CAACACACGTACCATGCA	106	126	0	0	0	0
ADAR	TGACACACGTACCATGAC	36	56	0	1	0	0
ADAR	ACACACACGTACCATGTG	316	506	0	0	0	0
ADARB1	TGACACACGTTGACTGAC	39	30	0	0	0	0
ADARB1	ACACACACGTTGACTGTG	903	505	1	1	0	30
ADARB1	GTACACACGTTGTGGTGT	269	280	0	0	0	0
ADARB1	CAACACACGTTGTGGTCA	247	131	1	0	0	1
ADARB1	TGACACACGTTGTGGTAC	37	38	0	0	0	0
ADC	ACACTGTGACCAACGTCA	287	380	0	0	0	0
ADC	GTACTGTGACCAACGTAC	122	100	0	0	0	0
ADC	TGACTGTGACCAACGTTG	1314	1170	1	1	0	0
ADC	ACACTGTGACCAACCAGT	234	388	1	0	0	0
ADC	TGACTGTGACCAACCACA	309	536	0	1	0	0
ADC	TGTGCATGCAGTCAGTAC	24	26	0	0	0	0
ADCY1	GTACTGTGCATGTGTGGT	278	264	0	0	1	0
ADCY1	CAACTGTGCATGTGTGCA	313	270	0	0	1	0
ADCY1	TGACTGTGCATGTGTGAC	7	37	0	0	0	0
ADCY1	ACACTGTGCATGTGTGTG	78	132	0	0	0	0
ADCY1	GTACTGTGACGTGTGTGT	697	817	2	0	0	1
ADCY1	CATGCATGGTTGCACAAC	35	28	0	0	0	0
ADCY2	ACACTGTGACGTGTGTTG	312	316	0	0	1	0
ADCY2	CAACTGTGACGTGTGTCA	430	806	0	0	0	68
ADCY2	TGACTGTGACGTGTGTAC	62	60	0	0	0	0
ADCY2	CAACTGTGACGTGTCAGT	65	64	0	0	0	0
ADCY2	ACACTGTGACGTGTCACA	44	41	0	0	0	0
ADCY3	GTACTGTGACGTGTCAAC	14	22	0	0	0	0
ADCY3	TGACTGTGACGTGTCATG	31	38	0	0	0	0
ADCY3	ACACTGTGACGTGTACGT	134	159	0	0	0	0
ADCY3	TGACTGTGACGTGTACCA	154	178	0	0	0	0
ADCY3	CAACTGTGACGTGTACAC	26	15	0	0	0	0
ADCY4	TGACTGTGACGTACCAGT	315	191	0	0	1	0
ADCY4	TGACTGTGACGTACGTCA	219	237	0	0	0	1
ADCY4	CAACTGTGACGTACGTAC	28	40	0	0	0	0
ADCY4	GTACTGTGACGTACGTTG	179	206	0	0	0	0
ADCY4	GTACTGTGACGTACCACA	109	51	0	0	0	0
ADCY4	CATGCAACTGACGTACCA	260	169	0	0	0	1
ADCY5	GTACTGTGACGTGTACTG	163	218	0	1	0	0
ADCY5	TGACTGTGACGTGTTGGT	203	377	0	1	0	0
ADCY5	GTACTGTGACGTGTTGCA	91	70	0	0	0	0
ADCY5	ACACTGTGACGTGTTGAC	17	30	0	0	0	0
ADCY5	CAACTGTGACGTGTTGTG	575	143	2	0	0	0
ADCY6	TGACTGTGACGTCAGTGT	316	466	0	0	0	0
ADCY6	GTACTGTGACGTCAGTCA	438	178	0	15	0	0
ADCY6	ACACTGTGACGTCAGTAC	40	37	0	1	0	1
ADCY6	CAACTGTGACGTCAGTTG	234	257	0	0	0	0
ADCY6	GTTGCAGTTGTGTGACGT	115	117	0	0	0	0
ADCY6	CATGCAACCATGCAACTG	474	513	1	0	0	0
ADCY7	GTACTGTGACGTCACAGT	48	28	0	0	1	0
ADCY7	CAACTGTGACGTCAACGT	983	467	1	1	1	0
ADCY7	CAACTGTGACGTCACACA	57	47	0	0	0	0
ADCY7	TGACTGTGACGTCACAAC	23	30	0	0	0	0
ADCY7	ACACTGTGACGTCACATG	289	186	0	1	0	0
ADCY8	ACACTGTGACGTCAACCA	235	280	0	0	0	0
ADCY8	GTACTGTGACGTCAACAC	55	130	0	0	0	0
ADCY8	TGACTGTGACGTCAACTG	91	95	0	0	0	0
ADCY8	ACACTGTGACGTCATGGT	131	272	0	0	0	0
ADCY8	CATGGTGTACCAACGTAC	40	33	0	0	0	0
ADCY8	TGTGCACACACACAACGT	192	457	0	0	0	1
ADCY9	TGACTGTGACGTCATGCA	64	109	0	0	0	0
ADCY9	CAACTGTGACGTCATGAC	37	39	0	0	0	0
ADCY9	GTACTGTGACGTCATGTG	228	180	0	0	0	0
ADCY9	ACACTGTGACGTACGTGT	216	188	0	1	0	0
ADCY9	ACTGCAGTCAACGTTGGT	477	256	0	0	0	1
ADCYAP1	ACACTGTGACGTACCAAC	57	46	0	0	0	0
ADCYAP1	CAACTGTGACGTACCATG	284	66	4	0	0	0
ADCYAP1	GTACTGTGACGTACACGT	62	57	0	0	0	0
ADCYAP1	CAACTGTGACGTACACCA	142	217	0	1	0	0
ADCYAP1	GTTGCAGTTGTGTGGTTG	515	489	0	0	0	0

ADCYAP1R1	GTACGTGTTGGTACCATG	189	133	0	0	0	0
ADCYAP1R1	TGACGTGTTGGTACACGT	79	164	0	0	0	0
ADCYAP1R1	GTACGTGTTGGTACACCA	44	28	0	0	0	0
ADCYAP1R1	ACACGTGTTGGTACACAC	18	26	0	0	0	0
ADCYAP1R1	CATGCATGACCAACGTCA	110	134	0	0	0	0
ADD1	CATGGTGTGTACACCAAC	8	30	0	0	0	0
ADD1	GTTGGTGTGTACACCATG	41	31	0	0	0	0
ADD1	TGTGGTGTGTACACACGT	20	18	0	0	0	0
ADD1	GTTGGTGTGTACACACCA	120	137	0	0	0	0
ADD1	ACTGGTGTGTACACACAC	11	7	0	0	0	0
ADD1	CATGCATGGTACACGTTG	209	198	0	1	0	0
ADFP	TGTGGTCACATGTGGTGT	1299	1188	4	1	1	1
ADFP	TGTGGTCACATGACTGGT	309	446	0	1	0	6
ADFP	GTTGGTCACATGACTGCA	311	152	0	0	0	0
ADFP	ACTGGTCACATGACTGAC	46	106	0	0	0	0
ADFP	CATGGTCACATGACTGTG	223	249	0	0	0	0
ADH1A	ACACCAGTCAACGTGTGT	1433	826	0	0	1	0
ADH1A	GTACCAGTCACATGGTAC	35	36	0	0	0	0
ADH1A	CAACCAGTCACATGTGCA	559	723	3	0	0	3
ADH1A	TGACCAGTCACATGTGAC	28	29	0	0	0	0
ADH1A	ACACCAGTCACATGTGTG	641	223	4	1	0	4
ADH1B	GTACCAGTCACAACTGAC	43	69	0	0	0	0
ADH1B	TGACCAGTCACAACTGTG	337	1022	0	0	0	0
ADH1B	CAACCAGTCACATGGTGT	1639	2043	2	2	0	1
ADH1B	ACACCAGTCACATGGTCA	389	469	0	1	0	0
ADH1B	CAACCAGTCAACACACAC	71	113	0	0	0	0
ADH1C	ACACCAGTCACAACCAAC	35	52	0	0	0	0
ADH1C	CAACCAGTCACAACCATG	453	337	0	0	0	0
ADH1C	GTACCAGTCACAACACGT	56	29	0	0	0	0
ADH1C	ACACCAGTCAACACACGT	50	72	0	0	0	0
ADH1C	TGACCAGTCAACACACCA	146	176	0	0	0	0
ADH4	ACACCAGTCACACAACCA	32	41	0	0	0	0
ADH4	GTACCAGTCACACAACAC	154	175	0	0	0	0
ADH4	TGACCAGTCACACAACTG	104	129	0	2	0	0
ADH4	ACACCAGTCACACATGGT	621	212	0	0	0	1
ADH4	CATGACCACACAACACGT	195	83	0	0	0	0
ADH5	ACACCAGTGTGTACCAAC	16	16	0	0	0	0
ADH5	CAACCAGTCACACACACA	238	359	1	0	0	0
ADH5	TGACCAGTCACACACAAC	122	21	1	0	0	0
ADH5	ACACCAGTCACACACATG	175	160	0	0	0	1
ADH5	CAACCAGTCACACAACGT	238	119	250	0	0	0
ADH6	TGACCAGTCACACAGTGT	1269	1189	3	3	3	7
ADH6	GTACCAGTCACACAGTCA	840	759	3	369	0	0
ADH6	ACACCAGTCACACAGTAC	175	154	0	0	0	0
ADH6	CAACCAGTCACACAGTTG	238	190	0	0	0	0
ADH6	GTACCAGTCACACACAGT	267	529	0	0	0	6
ADH6	ACTGCACATGCATGCACA	170	123	0	0	0	2
ADH7	CAACCAGTCACAGTGTCA	743	1001	0	0	0	0
ADH7	TGACCAGTCACAGTGTAC	214	161	0	0	0	0
ADH7	ACACCAGTCACAGTGTTG	269	364	0	1	0	1
ADH7	CAACCAGTCACAGTCAGT	292	484	0	0	0	1
ADH7	ACACCAGTCACAGTCACA	104	108	0	0	0	1
ADHFE1	TGACCAGTGTCATGCATG	503	276	0	0	0	0
ADHFE1	ACACCAGTGTCATGACGT	64	66	0	0	0	0
ADHFE1	TGACCAGTGTCATGACCA	133	110	0	0	0	0
ADHFE1	CAACCAGTGTCATGACAC	58	178	0	0	0	0
ADHFE1	GTACCAGTGTCATGACTG	142	240	0	0	0	0
ADIPOQ	ACTGGTCAGTCATGTGGT	802	1412	0	1	1	0
ADIPOQ	TGTGGTCAGTCATGTGCA	104	173	0	0	0	0
ADIPOQ	CATGGTCAGTCATGTGAC	44	83	0	0	0	0
ADIPOQ	GTTGGTCAGTCATGTGTG	309	402	2	0	0	0
ADIPOQ	GTTGGTCAGTACGTGTGT	446	754	1	0	0	11
ADIPOR1	CAACTGACGTACCAGTCA	144	214	0	0	0	7
ADIPOR1	TGACTGACGTACCAGTAC	36	51	0	1	0	0
ADIPOR1	ACACTGACGTACCAGTTG	698	647	1	0	0	24
ADIPOR1	CAACTGACGTACCACAGT	388	310	2	1	0	2
ADIPOR1	ACACTGACGTACCACACA	146	114	0	0	0	0
ADIPOR1	ACTGCAACACGTCAACAC	32	61	0	0	0	0
ADIPOR2	TGACTGCAACGTACACCA	135	443	1	2	1	3
ADIPOR2	ACACTGCAACGTACACGT	2	8	0	0	0	0
ADIPOR2	CAACTGCAACGTACACAC	210	75	8	0	0	0
ADIPOR2	GTACTGCAACGTACACTG	251	491	1	0	0	0
ADIPOR2	GTTGCAGTCAACACGTAC	14	5	0	0	0	0
ADK	ACACGTTGTGACCATGCA	170	77	0	0	0	0
ADK	GTACGTTGTGACCATGAC	74	110	0	1	0	0

ADK	GTTGCAGTCAACGTGTCA	126	129	1	0	0	0
ADK	ACTGCAGTCAACGTGTAC	92	187	0	0	0	0
ADK	CATGCAGTCATGACCATG	416	404	0	1	0	19
ADM	TGACTGTGTGCATGACTG	810	591	0	0	0	1
ADM	ACACTGTGTGCATGTGGT	261	131	0	0	0	0
ADM	TGACTGTGTGCATGTGCA	264	302	0	0	0	3
ADM	CATGCAGTGTTGGTACGT	38	71	0	0	0	0
ADM	ACTGCAGTGTTGGTACCA	227	169	0	0	0	0
ADM	GTTGCACAGTCACACAAC	18	19	0	0	0	0
ADORA1	CAACGTGTTGGTACACTG	240	273	2	0	0	0
ADORA1	GTACGTGTTGGTACTGGT	147	222	0	0	0	0
ADORA1	CAACGTGTTGGTACTGCA	178	336	0	0	0	2
ADORA1	TGACGTGTTGGTACTGAC	38	53	0	0	0	0
ADORA1	ACACGTGTTGGTACTGTG	243	260	2	0	0	1
ADORA2A	GTACGTGTTGGTTGGTGT	1309	475	0	1	1	2
ADORA2A	CAACGTGTTGGTTGGTCA	354	237	0	0	0	0
ADORA2A	TGACGTGTTGGTTGGTAC	45	106	118	0	0	1
ADORA2A	ACACGTGTTGGTTGGTTG	206	145	0	1	0	0
ADORA2A	CAACGTGTTGGTTGCAGT	29	62	0	0	0	0
ADORA2A	TGTGCAACGTTGGTCACA	79	62	1	0	0	0
ADORA2B	CAACTGACTGCACATGTG	349	414	2	0	0	0
ADORA2B	TGACTGACTGCAACGTGT	350	400	2	0	0	0
ADORA2B	GTACTGACTGCAACGTCA	128	124	2	0	0	0
ADORA2B	ACACTGACTGCAACGTAC	47	78	0	0	0	0
ADORA2B	CAACTGACTGCAACGTTG	414	401	2	0	0	0
ADORA2B	CATGCAACACTGACCATG	602	763	1	1	0	0
ADORA3	ACACGTGTTGGTTGCACA	271	247	0	0	0	0
ADORA3	GTACGTGTTGGTTGCAAC	7	20	0	0	0	0
ADORA3	TGACGTGTTGGTTGCATG	111	151	1	0	0	0
ADORA3	ACACGTGTTGGTTGACGT	139	115	0	0	0	0
ADORA3	TGACGTGTTGGTTGACCA	120	162	0	0	0	0
ADRA1A	CAACGTGTTGGTTGACAC	22	18	0	0	0	0
ADRA1A	GTACGTGTTGGTTGACTG	115	125	0	2	0	0
ADRA1A	TGACGTGTTGGTTGTGGT	731	305	1	0	0	0
ADRA1A	GTACGTGTTGGTTGTGCA	196	152	0	0	0	1
ADRA1A	ACTGCAACGTCAACTGAC	79	61	0	0	0	0
ADRA1A	CATGCAACGTCAACTGTG	287	304	0	0	0	0
ADRA1B	ACACGTGTTGGTTGTGAC	11	42	0	0	0	0
ADRA1B	CAACGTGTTGGTTGTGTG	432	1071	7	0	0	0
ADRA1B	ACACGTGTTGCAGTGTGT	638	491	1	0	0	1
ADRA1B	TGACGTGTTGCAGTGTCA	41	22	0	0	0	0
ADRA1B	CAACGTGTTGCAGTGTAC	48	53	0	0	0	0
ADRA1D	GTACGTGTTGCAGTGTTG	703	744	3	2	0	2
ADRA1D	TGACGTGTTGCAGTCAGT	263	251	0	0	0	0
ADRA1D	GTACGTGTTGCAGTCACA	274	420	1	1	0	4
ADRA1D	ACACGTGTTGCAGTCAAC	6	5	0	0	0	0
ADRA1D	GTTGACACACTGGTACAC	38	36	0	0	0	0
ADRA2A	GTTGCAACGTACGTGTGT	624	561	19	2	5	22
ADRA2A	GTTGCAACGTCATGTGTG	547	606	0	0	1	0
ADRA2A	CAACGTGTTGCAGTCATG	278	103	0	0	0	0
ADRA2A	GTACGTGTTGCAGTACGT	98	99	168	0	0	0
ADRA2A	CAACGTGTTGCAGTACCA	257	251	0	0	0	0
ADRA2A	TGACGTGTTGCAGTACAC	78	64	0	0	0	0
ADRA2B	TGACGTGTTGCAGTTGTG	119	152	0	1	1	0
ADRA2B	ACACGTGTTGCAGTACTG	481	436	1	0	0	0
ADRA2B	CAACGTGTTGCAGTTGGT	282	144	0	0	0	0
ADRA2B	ACACGTGTTGCAGTTGCA	383	367	0	0	0	2
ADRA2B	GTACGTGTTGCAGTTGAC	110	51	0	0	0	0
ADRA2C	CAACGTGTTGCACAGTGT	505	731	3	1	0	0
ADRA2C	ACACGTGTTGCACAGTCA	181	133	0	0	0	0
ADRA2C	ACTGACACGTACACCAGT	135	134	0	0	0	0
ADRA2C	TGTGACACGTACACCACA	92	100	0	1	0	0
ADRA2C	CATGACACGTACACCAAC	2	2	0	0	0	0
ADRB1	GTACGTGTTGCACAGTAC	15	36	0	0	0	0
ADRB1	TGACGTGTTGCACAGTTG	1059	639	1	3	0	0
ADRB1	ACACGTGTTGCACACAGT	306	400	1	0	0	5
ADRB1	TGACGTGTTGCACACACA	80	96	0	0	0	0
ADRB1	TGTGACACGTGTTGTGCA	440	187	0	0	0	1
ADRB2	CAACGTGTTGCACACAAC	4	6	0	0	0	0
ADRB2	GTACGTGTTGCACACATG	335	371	1	0	0	0
ADRB2	TGACGTGTTGCACAACGT	555	126	0	1	0	0
ADRB2	GTACGTGTTGCACAACCA	226	342	0	0	0	5
ADRB2	GTTGCAGTACCAACGTAC	60	65	0	0	0	0
ADRB3	ACACGTGTTGCACAACAC	46	17	0	0	0	0
ADRB3	CAACGTGTTGCACAACTG	726	464	1	0	0	1

ADRB3	GTACGTGTTGCACATGGT	168	180	0	0	0	0
ADRB3	CAACGTGTTGCACATGCA	486	457	2	0	0	1
ADRB3	TGACGTGTTGCACATGAC	17	19	0	0	0	0
ADRB3	GTTGCACACACACAACCA	452	281	0	1	0	1
ADRBK1	TGCATGCAACCAGTCAAC	18	24	0	0	0	0
ADRBK1	ACCATGCAACCAGTCATG	158	154	0	0	0	0
ADRBK1	CACATGCAACCAGTACGT	213	250	1	0	0	0
ADRBK1	ACCATGCAACCAGTACCA	276	123	1	0	0	0
ADRBK1	GTCATGCAACCAGTACAC	24	24	0	0	0	0
ADRBK1	ACTGCACAACCATGTGTG	366	451	0	0	0	0
ADRBK2	TGCATGACGTTGACCAGT	355	292	0	0	1	3
ADRBK2	CACATGACGTTGACGTAC	22	21	0	0	0	0
ADRBK2	GTCATGACGTTGACGTTG	134	128	0	0	0	0
ADRBK2	GTCATGACGTTGACCACA	174	95	0	0	0	0
ADRBK2	TGTGGTACTGACGTTGCA	49	47	0	0	0	0
ADSL	CATGCAGTTGTGTGGTAC	150	86	0	0	1	0
ADSL	ACACTGTGCATGCAGTAC	14	34	0	0	0	0
ADSL	CAACTGTGCATGCAGTTG	95	183	0	0	0	0
ADSL	GTACTGTGCATGCACAGT	85	79	0	0	0	0
ADSL	CAACTGTGCATGCACACA	51	61	0	0	0	0
ADSL	ACTGCACACACACAACAC	133	107	0	1	0	0
ADSS	ACACACGTCACATGTGAC	15	17	0	10	3	0
ADSS	CAACACGTCAACGTGTGT	432	1243	14	1	2	28
ADSS	TGACACGTCACATGTGGT	521	278	0	0	0	2
ADSS	GTACACGTCACATGTGCA	175	106	0	0	0	0
ADSS	CAACACGTCACATGTGTG	387	397	1	0	0	0
ADSSL1	CAACACGTACCAACCACA	369	188	0	0	0	0
ADSSL1	TGACACGTACCAACCAAC	28	18	0	0	0	0
ADSSL1	ACACACGTACCAACCATG	227	199	0	0	0	11
ADSSL1	CAACACGTACCAACACGT	21	30	0	0	0	0
ADSSL1	ACACACGTACCAACACCA	135	197	0	0	0	0
AES	GTACGTTGGTACTGGTTG	334	366	0	0	1	0
AES	TGACGTTGGTACTGCAGT	386	421	1	1	0	0
AES	GTACGTTGGTACTGCACA	174	74	0	0	0	0
AES	ACACGTTGGTACTGCAAC	28	18	0	0	0	1
AES	CAACGTTGGTACTGCATG	112	80	0	0	0	0
AFMID	GTACACCATGGTGTGTTG	486	865	11	7572	4756	6
AFMID	ACACACCATGGTGTGTGT	65	91	0	0	0	0
AFMID	TGACACCATGGTGTGTCA	256	202	0	0	0	4
AFMID	CAACACCATGGTGTGTAC	21	42	0	0	0	0
AFMID	TGACACCATGGTGTCAGT	22	40	0	0	0	0
AFP	ACACACTGACCACATGGT	307	494	1	1	1	0
AFP	TGTGCACAGTCACACATG	165	120	0	0	1	0
AFP	TGACACTGACCACAACTG	267	322	1	0	0	1
AFP	TGACACTGACCACATGCA	69	96	0	0	0	0
AFP	CAACACTGACCACATGAC	78	101	0	0	0	0
AFP	GTACACTGACCACATGTG	315	431	2	0	0	0
AGA	TGACACCAGTGTACACAC	136	133	0	0	0	0
AGA	ACACACCAGTGTACACTG	725	635	2	2	0	1
AGA	CAACACCAGTGTACTGGT	77	55	0	0	0	0
AGA	ACACACCAGTGTACTGCA	28	64	0	0	0	0
AGA	GTACACCAGTGTACTGAC	46	46	0	0	0	0
AGA	TGTGCATGCACAACGTCA	254	203	0	0	0	5
AGER	TGACTGACGTCAGTTGCA	63	81	0	0	0	0
AGER	CAACTGACGTCAGTTGAC	83	11	0	0	0	0
AGER	GTACTGACGTCAGTTGTG	196	201	0	0	0	1
AGER	ACACTGACGTCACAGTGT	381	143	0	0	0	0
AGER	TGACTGACGTCACAGTCA	130	107	0	0	0	0
AGER	GTTGCAACTGTGGTTGTG	719	453	7	0	0	0
AGK	GTTGGTACGTACCAGTGT	1169	1799	2	0	2	16
AGK	TGTGGTACGTACGTTGAC	71	68	0	0	0	0
AGK	ACTGGTACGTACGTTGTG	194	237	1	0	0	0
AGK	CATGGTACGTACCAGTCA	307	136	0	0	0	0
AGK	TGTGGTACGTACCAGTAC	114	135	0	0	0	0
AGK	CATGCAACACGTGTACCA	220	194	0	0	0	0
AGL	CAACCACAACACACCACA	76	76	0	0	0	0
AGL	TGACCACAACACACCAAC	108	57	0	0	0	0
AGL	ACACCACAACACACCATG	0	9	0	0	0	0
AGL	CAACCACAACACACACGT	26	21	0	0	0	0
AGL	ACACCACAACACACACCA	41	66	0	0	0	0
AGL	CATGCACACACACAACTG	433	835	1	1	0	0
AGMAT	GTACACCATGCATGTGAC	45	55	1	0	0	0
AGMAT	TGACACCATGCATGTGTG	414	393	1	0	0	3
AGMAT	TGACACCATGACGTGTGT	482	431	1	1	0	0
AGMAT	GTACACCATGACGTGTCA	305	381	0	0	0	0

AGMAT	ACACACCATGACGTGTAC	20	33	0	0	0	0
AGPAT1	GTACCACAACACACACAC	124	94	0	0	0	0
AGPAT1	TGACCACAACACACACTG	452	363	0	0	0	0
AGPAT1	ACACCACAACACACTGGT	444	416	1	1	0	0
AGPAT1	TGACCACAACACACTGCA	88	114	0	0	0	1
AGPAT1	CAACCACAACACACTGAC	45	36	0	0	0	0
AGPAT1	ACTGCAACTGTGCAGTGT	710	733	0	0	0	0
AGPAT2	CAACCACAACACTGACCA	564	517	38	1	1	4
AGPAT2	TGACCACAACACTGACAC	164	63	0	0	0	0
AGPAT2	ACACCACAACACTGACTG	773	1433	0	1	0	1
AGPAT2	CAACCACAACACTGTGGT	130	193	1	0	0	0
AGPAT2	TGTGCAGTGTACACTGGT	307	769	0	2	0	0
AGPAT3	CATGCAGTACTGCAACTG	1062	790	7	5	4	39
AGPAT3	CAACCACAACTGGTCATG	171	108	0	0	0	0
AGPAT3	GTACCACAACTGGTACGT	180	184	0	0	0	0
AGPAT3	CAACCACAACTGGTACCA	62	96	0	0	0	0
AGPAT3	TGACCACAACTGGTACAC	63	45	0	0	0	0
AGPAT4	CAACCACAACTGCAGTGT	1792	1637	1	2	0	0
AGPAT4	ACACCACAACTGCAGTCA	303	626	2	0	0	0
AGPAT4	GTACCACAACTGCAGTAC	37	30	0	0	0	0
AGPAT4	TGACCACAACTGCAGTTG	740	1151	1	1	0	11
AGPAT4	CATGCAGTACGTGTCAGT	171	400	1	1	0	0
AGPAT6	CATGCAGTACGTGTGTCA	211	248	0	0	1	5
AGPAT6	TGACCACATGGTCAACAC	25	19	0	0	0	0
AGPAT6	ACACCACATGGTCAACTG	223	384	2	0	0	0
AGPAT6	CAACCACATGGTCATGGT	515	572	1	1	0	0
AGPAT6	TGTGGTGTCACAGTGTCA	251	285	0	1	0	0
AGPAT6	GTTGCATGGTTGACTGCA	39	65	0	0	0	0
AGPS	GTACACGTACACTGTGCA	144	167	0	0	0	3
AGPS	ACACACGTACACTGTGAC	75	182	0	0	0	0
AGPS	CAACACGTACACTGTGTG	230	167	0	0	0	0
AGPS	GTACACGTACTGGTGTGT	1192	1055	2	0	0	1
AGPS	CAACACGTACTGGTGTCA	182	113	2	0	0	0
AGPS	ACTGCACAGTCACAACGT	348	298	0	1	0	0
AGRN	ACACACTGCAACCATGGT	960	1265	2	2	0	2
AGRN	TGACACTGCAACCATGCA	92	190	0	0	0	0
AGRN	TGTGCAGTTGCATGACTG	214	300	0	0	0	0
AGRN	ACTGCAGTTGCATGTGGT	529	226	1	0	0	0
AGRN	TGTGACGTACTGCACATG	111	130	0	0	0	0
AGRP	GTTGGTGTGTCATGTGAC	33	44	1	0	0	0
AGRP	CATGCACAGTGTGTGTAC	52	36	0	0	0	0
AGRP	GTTGCACAGTGTGTGTTG	217	152	0	0	0	0
AGRP	TGTGCACAGTGTGTCAGT	166	190	0	0	0	0
AGRP	TGTGCACAGTCACAACCA	429	303	2	2	0	0
AGRP	CATGCACAGTCACAACAC	43	41	102	0	0	0
AGT	ACACGTGTCAGTGTGTGT	507	516	0	0	1	0
AGT	ACACGTGTGTTGTGTGAC	9	25	0	0	0	0
AGT	CAACGTGTGTTGTGTGTG	282	283	0	0	0	1
AGT	TGACGTGTCAGTGTGTCA	161	189	2	0	0	0
AGT	CAACGTGTCAGTGTGTAC	88	108	0	0	0	0
AGTR1	ACACGTGTTGCACATGTG	214	196	0	0	0	0
AGTR1	GTACGTGTTGCAACGTGT	687	229	3	0	0	0
AGTR1	CAACGTGTTGCAACGTCA	132	140	0	0	0	0
AGTR1	TGACGTGTTGCAACGTAC	105	102	0	0	0	0
AGTR1	ACACGTGTTGCAACGTTG	131	133	0	0	0	0
AGTR2	CACATGACCACAACACAC	56	45	0	0	0	0
AGTR2	GTCATGACCACAACACTG	184	301	0	0	0	0
AGTR2	TGCATGACCACAACTGGT	113	129	0	0	0	0
AGTR2	GTCATGACCACAACTGCA	140	373	0	0	0	1
AGTR2	GTTGCAGTACGTACACGT	85	198	1	1	0	0
AGXT	ACACCACAACTGCACAGT	481	319	0	0	0	0
AGXT	TGACCACAACTGCACACA	100	251	0	0	0	0
AGXT	CAACCACAACTGCACAAC	11	6	0	0	0	0
AGXT	GTACCACAACTGCACATG	376	299	0	0	0	3
AGXT	TGACCACAACTGCAACGT	442	349	0	0	0	1
AGXT	GTTGCACACACACATGGT	852	486	3	0	0	1
AGXT2	CAACCACAACTGCAACTG	314	428	1	0	1	0
AGXT2	GTACCACAACTGCAACCA	224	225	0	0	0	0
AGXT2	ACACCACAACTGCAACAC	11	25	0	0	0	0
AGXT2	GTACCACAACTGCATGGT	531	378	0	1	0	0
AGXT2	CAACCACAACTGCATGCA	795	143	6	1	0	0
AGXT2	CATGCAACGTGTCATGGT	561	500	1	0	0	0
AGXT2L1	TGACCACACATGCACAAC	5	13	0	0	0	0
AGXT2L1	ACACCACACATGCACATG	206	174	2	0	0	6
AGXT2L1	CAACCACACATGCAACGT	588	1298	2	1	0	2

AGXT2L1	ACACCACACATGCAACCA	272	249	0	30	0	1
AGXT2L1	ACTGCAGTCATGGTCACA	70	134	0	0	0	1
AHCY	GTACACCATGACGTTGTG	407	220	0	0	0	0
AHCY	ACACACCATGACCAGTGT	351	452	3	1	0	0
AHCY	TGACACCATGACCAGTCA	173	330	0	0	0	1
AHCY	CAACACCATGACCAGTAC	95	189	1	0	0	0
AHCY	GTACACCATGACCAGTTG	1445	1953	5	0	0	456
AHCY	TGTGCACATGCAGTGTGT	324	201	0	1	0	0
AHR	GTACGTTGACGTACCATG	494	222	154	117	143	172
AHR	TGACGTTGACGTACCACA	74	97	0	0	0	0
AHR	CAACGTTGACGTACCAAC	18	13	1	0	0	0
AHR	TGACGTTGACGTACACGT	83	35	1	0	0	0
AHR	GTACGTTGACGTACACCA	148	177	0	0	0	0
AHSG	GTACGTGTCAGTGTGTTG	434	491	0	1	0	0
AHSG	TGACGTGTCAGTGTCAGT	320	132	2	1	0	3
AHSG	GTACGTGTCAGTGTCACA	390	106	1	0	0	1
AHSG	ACACGTGTCAGTGTCAAC	44	23	0	0	0	0
AHSG	ACTGCAGTACCACAGTGT	1542	1083	3	1	0	1
AHSG	GTTGCACAGTCACAACTG	357	305	0	0	0	48
AICDA	TGACACCATGTGACTGTG	988	659	1	2	1	0
AICDA	CAACACCATGTGACTGGT	507	787	2	0	0	0
AICDA	ACACACCATGTGACTGCA	89	58	0	0	0	0
AICDA	GTACACCATGTGACTGAC	37	45	0	0	0	0
AICDA	CAACACCATGTGTGGTGT	576	711	0	0	0	5
AICDA	GTTGCACATGCACAGTTG	932	410	0	0	0	0
AIFM1	CAACTGACACCACAACTG	571	511	1	0	0	0
AIFM1	GTACTGACACCACATGGT	363	175	0	0	0	0
AIFM1	CAACTGACACCACATGCA	202	316	1	0	0	0
AIFM1	GTTGGTTGTGGTCACACA	95	116	0	1	0	0
AIFM1	ACTGGTTGTGGTCACAAC	113	17	0	0	0	0
AIFM1	ACTGCAACACACACTGTG	733	254	3	2	0	1
AIRE	CAACGTACTGGTGTCAAC	44	44	0	0	0	0
AIRE	GTACGTACTGGTGTCATG	161	138	1	0	0	0
AIRE	CATGCACACACACATGCA	208	149	0	1	0	0
AIRE	TGTGCACACACACATGAC	58	60	0	0	0	0
AIRE	ACTGCACACACACATGTG	170	273	2	0	0	6
AIRE	GTTGCACACACAACGTGT	660	449	0	1	0	0
AK1	GTCATGTGTGACGTACGT	20	67	0	0	0	0
AK1	CACATGTGTGACGTACCA	65	101	0	0	0	3
AK1	TGCATGTGTGACGTACAC	247	40	0	0	0	0
AK1	ACCATGTGTGACGTACTG	110	170	0	0	0	0
AK1	ACCATGTGTGACTGGTAC	51	51	0	0	0	0
AK1	TGTGCACAGTCACATGGT	94	100	0	0	0	1
AK2	CACATGTGTGACGTTGGT	338	416	2	0	0	0
AK2	ACCATGTGTGACGTTGCA	82	60	0	0	0	0
AK2	CACATGTGTGACTGGTTG	547	193	0	0	0	1
AK2	GTCATGTGTGACTGCAGT	265	250	0	0	0	1
AK2	CACATGTGTGACTGCACA	76	48	0	0	0	2
AK3	GTCATGTGTGACGTTGAC	61	66	0	0	0	20
AK3	TGCATGTGTGACGTTGTG	211	122	0	0	0	0
AK3	CACATGTGTGACCAGTGT	259	285	0	0	0	0
AK3	ACCATGTGTGACCAGTCA	237	209	0	0	0	5
AK3	GTCATGTGTGACCAGTAC	42	40	1	0	0	0
AK3	TGTGCAACCACACATGTG	68	47	0	1	0	0
AK3L1	CAACCAACACACACTGTG	552	394	0	0	1	4
AK3L1	ACACCAACACACACTGAC	82	234	0	0	0	0
AK3L1	TGACCAACACACTGGTGT	163	189	1	0	0	0
AK3L1	GTACCAACACACTGGTCA	345	390	0	1	0	1
AK3L1	ACACCAACACACTGGTAC	68	43	0	0	0	1
AK5	TGTGCATGGTGTTGCACA	115	119	1	1	1	0
AK5	TGCATGTGTGACCAGTTG	1375	682	1	0	0	39
AK5	ACCATGTGTGACCACAGT	93	166	0	1	0	0
AK5	TGCATGTGTGACCACACA	143	99	0	0	0	0
AK5	CACATGTGTGACCACAAC	58	73	0	0	0	0
AK5	GTCATGTGTGACCACATG	166	73	0	0	0	1
AK7	TGACCAACTGGTACTGTG	912	705	1	1	1	1
AK7	ACACCAACTGGTACTGCA	376	141	0	0	0	0
AK7	GTACCAACTGGTACTGAC	45	55	0	0	0	0
AK7	CAACCAACTGGTTGGTGT	131	62	0	1	0	0
AK7	ACACCAACTGGTTGGTCA	257	353	0	0	0	0
AKAP1	GTACGTCAACACGTTGAC	7	25	0	0	0	0
AKAP1	TGACGTCAACACGTTGTG	122	128	0	0	0	0
AKAP1	CAACGTCAACACCAGTGT	506	808	1	1	0	0
AKAP1	GTTGCAGTACACGTTGGT	163	245	0	0	0	0
AKAP1	CATGCAACACGTTGACGT	448	313	0	0	0	0

AKAP1	ACTGCAACACGTTGACCA	126	227	1	0	0	0
AKAP10	CAACCAACTGCATGACAC	112	149	0	0	0	0
AKAP10	GTACCAACTGCATGACTG	218	255	0	0	0	0
AKAP10	TGACCAACTGCATGTGGT	191	567	0	0	0	1
AKAP10	GTACCAACTGCATGTGCA	209	237	1	0	0	19
AKAP10	ACACCAACTGCATGTGAC	51	92	0	0	0	0
AKAP10	ACTGCAACACGTTGCATG	91	112	0	1	0	0
AKAP11	ACCATGACGTACTGACGT	169	289	0	0	0	0
AKAP11	TGCATGACGTACTGACCA	54	127	0	0	0	0
AKAP11	CACATGACGTACTGACAC	41	58	0	0	0	0
AKAP11	GTCATGACGTACTGACTG	317	386	0	1	0	0
AKAP11	TGCATGACGTACTGTGGT	295	199	0	0	0	0
AKAP11	CATGCAACACGTTGCACA	38	78	0	0	0	0
AKAP12	GTACCAACTGCAACCATG	250	255	0	1	0	1
AKAP12	TGACCAACTGCAACACGT	160	134	0	0	0	0
AKAP12	GTACCAACTGCAACACCA	459	450	2	0	0	1
AKAP12	ACACCAACTGCAACACAC	30	37	0	0	0	0
AKAP12	CAACCAACTGCAACACTG	287	393	0	1	0	0
AKAP12	TGTGCAACACGTTGCAAC	11	18	0	0	0	0
AKAP13	TGACCAACTGCATGCATG	239	228	0	0	0	0
AKAP13	ACACCAACTGCATGACGT	564	571	1	0	0	1
AKAP13	TGACCAACTGCATGACCA	49	40	0	0	0	0
AKAP13	TGTGCAGTCACATGCAGT	23	51	0	0	0	0
AKAP13	GTTGCAGTCACATGCACA	112	56	0	0	0	0
AKAP13	CATGCATGCAGTACCACA	71	166	0	0	0	0
AKAP3	ACACCAACTGCATGGTTG	532	650	0	0	0	0
AKAP3	CAACCAACTGCATGCAGT	92	100	1	0	0	0
AKAP3	ACACCAACTGCATGCACA	72	128	1	0	0	0
AKAP3	GTACCAACTGCATGCAAC	19	9	0	0	0	0
AKAP3	TGTGCAGTACTGTGTGCA	325	455	0	0	0	0
AKAP3	TGTGCAACACGTTGACTG	564	436	1	0	0	0
AKAP4	GTACCAACTGCAGTTGTG	524	486	1	0	1	0
AKAP4	TGACCAACTGCAGTTGCA	461	109	0	0	0	0
AKAP4	CAACCAACTGCAGTTGAC	73	74	0	0	0	0
AKAP4	ACACCAACTGCACAGTGT	975	235	0	1	0	0
AKAP4	TGACCAACTGCACAGTCA	964	696	1	0	0	2
AKAP4	GTTGCAACACGTTGACAC	29	49	1	0	0	0
AKAP5	TGCATGACCAGTTGCATG	619	575	0	1	1	1
AKAP5	CACATGACCAGTTGCAGT	470	161	0	0	0	1
AKAP5	ACCATGACCAGTTGCACA	124	164	0	1	0	0
AKAP5	GTCATGACCAGTTGCAAC	23	19	0	0	0	0
AKAP5	GTTGGTTGTGCATGCAGT	742	191	0	0	0	0
AKAP5	ACTGCAACACGTTGTGGT	393	466	1	0	0	0
AKAP6	CAACCAACTGCAACGTGT	1740	1209	7	1	2	5616
AKAP6	TGACCAACTGCACATGTG	296	293	0	0	1	0
AKAP6	GTACCAACTGCACATGAC	19	15	0	0	0	0
AKAP6	ACACCAACTGCAACGTCA	110	130	0	0	0	0
AKAP6	GTACCAACTGCAACGTAC	27	19	0	0	0	0
AKAP6	TGTGCAACACGTTGTGCA	308	249	0	1	0	0
AKAP7	ACACCAACTGCACAACTG	805	835	0	1	2	11
AKAP7	TGACCAACTGCACAACAC	81	70	0	0	0	0
AKAP7	CAACCAACTGCACATGGT	259	159	0	0	0	0
AKAP7	ACACCAACTGCACATGCA	421	277	0	0	0	0
AKAP7	ACTGGTTGTGGTTGTGAC	66	70	1	0	0	0
AKAP7	ACTGCAACCAACACTGGT	769	609	1	1	0	0
AKAP8	GTACCAACTGCATGGTGT	587	1187	3	1	1	0
AKAP8	TGACCAACTGCAACTGAC	214	156	0	0	0	1
AKAP8	ACACCAACTGCAACTGTG	587	896	2	1	0	2
AKAP8	CAACCAACTGCATGGTCA	259	300	0	0	0	0
AKAP8	TGACCAACTGCATGGTAC	62	74	0	0	0	0
AKAP8	CATGCAACACGTTGTGAC	16	103	0	0	0	0
AKAP9	CATGGTTGTGGTTGTGTG	534	510	2	0	1	1
AKAP9	GTACCAACTGCAACTGGT	668	544	0	1	0	0
AKAP9	CAACCAACTGCAACTGCA	249	308	0	0	0	0
AKAP9	ACTGCAGTACTGTGTGGT	524	532	0	0	0	1
AKAP9	GTTGCAACACACTGTGCA	241	265	0	0	0	0
AKAP9	ACTGCAACACACTGTGAC	91	72	0	0	0	0
AKR1A1	GTACACGTTGACCAACCA	629	354	3	1	2	0
AKR1A1	TGACACGTTGACCAACGT	132	406	0	0	0	3
AKR1A1	ACACACGTTGACCAACAC	34	93	0	0	0	0
AKR1A1	CAACACGTTGACCAACTG	359	307	0	0	0	2
AKR1A1	GTACACGTTGACCATGGT	246	280	0	0	0	0
AKR1A1	TGTGCAACTGACGTCAGT	278	458	1	0	0	1
AKR1B1	TGACACGTTGACCAGTTG	357	637	0	2	0	0
AKR1B1	ACACACGTTGACCACAGT	143	247	0	0	0	0

AKR1B1	TGACACGTTGACCACACA	63	41	0	0	0	0
AKR1B1	CAACACGTTGACCACAAC	52	23	0	0	0	0
AKR1B1	GTACACGTTGACCACATG	130	166	0	0	0	0
AKR1B1	ACTGCAACTGACGTCAAC	13	63	0	0	0	0
AKR1B10	ACACACGTTGCATGGTCA	107	88	0	0	0	0
AKR1B10	GTACACGTTGCATGGTAC	38	49	0	0	0	0
AKR1B10	TGACACGTTGCATGGTTG	145	215	0	0	0	0
AKR1B10	ACACACGTTGCATGCAGT	186	159	0	0	0	0
AKR1B10	TGACACGTTGCATGCACA	201	150	0	0	0	62
AKR1B10	GTTGCAACTGACGTCACA	97	92	1	0	0	1
AKR1C1	ACACCAACACGTACCATG	114	94	0	0	0	0
AKR1C1	CAACCAACACGTACACGT	6	8	0	0	0	0
AKR1C1	ACACCAACACGTACACCA	224	376	1	0	0	0
AKR1C1	GTACCAACACGTACACAC	24	54	0	0	0	0
AKR1C1	TGACCAACACGTTGGTCA	19	37	0	0	0	0
AKR1C2	GTACCAACACGTACTGTG	52	54	0	0	0	0
AKR1C2	ACACCAACACGTTGGTGT	425	219	0	0	0	0
AKR1C2	TGTGACCAACTGACTGGT	490	102	1	0	0	1
AKR1C2	GTTGACCAACTGACTGCA	31	56	0	0	0	0
AKR1C2	ACTGACCAACTGACTGAC	105	78	0	0	0	0
AKR1C3	ACACCAGTCATGTGACGT	109	111	0	0	0	1
AKR1C3	TGACCAGTCATGTGACCA	193	105	0	0	0	0
AKR1C3	CAACCAGTCATGTGACAC	139	57	0	0	0	0
AKR1C3	GTACCAGTCATGTGACTG	254	294	0	1	0	1
AKR1C3	TGACCAGTCATGTGTGGT	703	631	0	1	0	1
AKR1C3	CATGCAACTGACGTCATG	100	172	2	1	0	1
AKR1C4	ACACCAACACGTACGTAC	70	129	1	0	0	0
AKR1C4	CAACCAACACGTACGTTG	360	222	1	0	0	0
AKR1C4	GTACCAACACGTACCAGT	55	91	0	0	0	0
AKR1C4	CAACCAACACGTACCACA	226	136	0	0	0	0
AKR1C4	TGACCAACACGTACCAAC	188	109	0	0	0	0
AKR1C4	GTTGCAACTGACGTACGT	58	126	1	0	0	0
AKR1D1	GTACCATGACCACATGCA	257	647	2	0	0	0
AKR1D1	TGACACGTTGACGTTGTG	118	160	0	0	0	0
AKR1D1	CAACACGTTGACCAGTGT	293	313	0	1	0	0
AKR1D1	ACACACGTTGACCAGTCA	709	256	0	0	0	0
AKR1D1	GTACACGTTGACCAGTAC	206	48	0	0	0	0
AKT1	ACACCATGCAGTTGACAC	131	112	4	1613	1183	0
AKT1	CAACCATGCAGTTGACTG	863	299	3	0	0	1
AKT1	GTACCATGCAGTTGTGGT	171	279	1	0	0	0
AKT1	CAACCATGCAGTTGTGCA	72	146	0	0	0	0
AKT1	TGACCATGCACACAACAC	82	54	0	0	0	0
AKT1S1	CATGGTTGCAGTACCAGT	167	243	0	0	0	0
AKT1S1	ACTGGTTGCAGTACCACA	16	32	0	0	0	0
AKT1S1	GTTGGTTGCAGTACCAAC	61	16	0	0	0	0
AKT1S1	TGTGGTTGCAGTACCATG	212	154	0	0	0	0
AKT1S1	ACTGGTTGCAGTACACGT	105	251	0	0	0	0
AKT2	TGCATGCAACCAGTACTG	403	429	27	36	9	47
AKT2	ACCATGCAACCAGTTGGT	292	258	0	1	0	0
AKT2	TGCATGCAACCAGTTGCA	94	100	1	0	0	0
AKT2	CACATGCAACCAGTTGAC	40	38	0	0	0	0
AKT2	GTCATGCAACCAGTTGTG	462	572	1	0	0	0
AKT3	GTCATGACGTGTTGTGTG	127	118	0	0	0	0
AKT3	CACATGACGTCAGTGTGT	199	237	0	1	0	3
AKT3	ACCATGACGTCAGTGTCA	489	328	1	1	0	76
AKT3	GTCATGACGTCAGTGTAC	218	108	0	0	0	1
AKT3	ACACCATGCAACGTGTCA	190	136	0	0	0	0
AKT3	TGTGCATGCACACATGCA	96	107	0	0	0	0
AKTIP	CACATGTGCACACATGGT	295	213	1	0	0	0
AKTIP	ACCATGTGCACACATGCA	396	168	0	0	0	0
AKTIP	GTCATGTGCACACATGAC	5	22	0	0	0	0
AKTIP	TGCATGTGCACACATGTG	118	195	0	0	0	0
AKTIP	CAACCAACACACACCAGT	154	196	0	1	0	0
ALAD	ACACACACTGGTCACAAC	54	62	0	0	1	0
ALAD	GTACACACTGGTCACACA	111	157	0	0	0	0
ALAD	CAACACACTGGTCACATG	392	396	0	0	0	0
ALAD	GTACACACTGGTCAACGT	183	155	0	1	0	1
ALAD	GTTGCAGTGTCAACTGAC	11	7	0	0	0	0
ALAS1	GTACACGTACGTACTGCA	108	365	0	0	1	0
ALAS1	TGACACGTACGTACACCA	346	326	0	1	0	0
ALAS1	CAACACGTACGTACACAC	30	18	0	0	0	0
ALAS1	GTACACGTACGTACACTG	341	106	0	0	0	0
ALAS1	TGACACGTACGTACTGGT	290	183	0	0	0	0
ALAS2	TGACACGTACGTTGTGCA	297	223	0	0	0	0
ALAS2	CAACACGTACGTTGTGAC	72	175	0	0	0	0

ALAS2	GTACACGTACGTTGTGTG	307	135	0	0	0	0
ALAS2	CAACACGTACCAGTGTGT	286	287	1	41	0	0
ALAS2	ACACACGTACCAGTGTCA	563	194	0	0	0	0
ALB	CAACTGCAGTTGACTGAC	6	22	0	0	0	0
ALB	GTACTGCAGTTGACTGTG	200	165	0	15	0	8
ALB	ACTGCACATGGTGTCACA	193	209	1	0	0	0
ALB	GTTGCACATGGTGTCAAC	7	0	0	0	0	0
ALB	TGTGCACATGGTGTCATG	271	258	0	0	0	0
ALB	ACTGCACATGGTGTACGT	113	303	0	0	0	0
ALCAM	CATGGTGTCAGTACGTGT	670	594	1	1	2	10
ALCAM	TGTGGTACACACACACAC	34	36	1	0	0	0
ALCAM	ACTGGTACACACACACTG	136	211	0	0	0	17
ALCAM	CATGGTACACACACTGGT	267	398	0	0	0	0
ALCAM	ACTGGTACACACACTGCA	412	403	13	0	0	0
ALDH18A1	GTTGACCAGTCACACAGT	275	260	0	0	1	0
ALDH18A1	TGACTGACACTGACACTG	113	105	0	1	0	0
ALDH18A1	ACACTGACACTGACTGGT	774	191	1	0	0	0
ALDH18A1	TGACTGACACTGACTGCA	210	248	0	0	0	0
ALDH18A1	CAACTGACACTGACTGAC	159	89	0	0	0	0
ALDH1A1	CAACCAGTCAGTTGCATG	125	49	0	0	0	0
ALDH1A1	GTACCAGTCAGTTGACGT	216	618	0	0	0	0
ALDH1A1	CAACCAGTCAGTTGACCA	198	261	0	1	0	0
ALDH1A1	TGACCAGTCAGTTGACAC	127	145	1	0	0	0
ALDH1A1	ACACCAGTCAGTTGACTG	275	376	0	0	0	0
ALDH1A1	ACTGCAACTGACTGGTAC	148	94	0	0	0	0
ALDH1A2	CATGCAACTGACTGGTTG	920	341	1	0	1	0
ALDH1A2	TGACGTTGTGTGCACAAC	29	25	1	0	0	0
ALDH1A2	ACACGTTGTGTGCACATG	946	394	1	0	0	0
ALDH1A2	CAACGTTGTGTGCAACGT	208	139	0	0	0	0
ALDH1A2	ACACGTTGTGTGCAACCA	389	695	2	0	0	0
ALDH1A2	GTACGTTGTGTGCAACAC	15	16	0	0	0	0
ALDH1A3	GTTGCACAGTCACATGCA	569	624	2	0	1	0
ALDH1A3	ACACCAGTCAGTCACACA	34	21	0	0	0	0
ALDH1A3	GTACCAGTCAGTCACAAC	24	38	0	0	0	0
ALDH1A3	TGACCAGTCAGTCACATG	378	331	0	1	0	0
ALDH1A3	ACACCAGTCAGTCAACGT	392	181	1	0	0	0
ALDH1A3	TGACCAGTCAGTCAACCA	122	142	0	0	0	0
ALDH1B1	CAACCAGTCAGTCATGTG	186	161	1	0	0	0
ALDH1B1	TGACCAGTCAGTACGTGT	260	415	2	0	0	0
ALDH1B1	GTACCAGTCAGTACGTCA	112	108	1	1	0	0
ALDH1B1	ACACCAGTCAGTACGTAC	64	59	0	0	0	0
ALDH1B1	GTTGCAGTGTTGGTACAC	20	38	3	2	0	3316
ALDH1B1	GTTGCAACTGACTGCAGT	85	54	0	0	0	0
ALDH1L1	CATGCAACACCACATGTG	325	386	0	0	1	0
ALDH1L1	ACACCAGTCAACCATGTG	608	313	0	1	0	0
ALDH1L1	GTACCAGTCAACACGTGT	528	600	0	0	0	0
ALDH1L1	CAACCAGTCAACACGTCA	395	67	0	0	0	0
ALDH1L1	TGACCAGTCAACACGTAC	193	146	0	1	0	0
ALDH1L1	ACACCAGTCAACACTGAC	164	115	0	0	0	0
ALDH2	CAACCAGTCAGTTGGTAC	27	61	0	0	0	0
ALDH2	GTACCAGTCAGTTGGTTG	536	475	0	0	0	4
ALDH2	TGACCAGTCAGTTGCAGT	112	227	0	1	0	3
ALDH2	GTACCAGTCAGTTGCACA	128	93	0	0	0	0
ALDH2	ACACCAGTCAGTTGCAAC	7	10	0	0	0	0
ALDH2	CATGCAACTGACTGCACA	275	236	1	0	0	8
ALDH3A1	ACACCAGTCAGTACACCA	272	179	1	0	1	0
ALDH3A1	CAACCAGTCAGTACACGT	80	27	0	0	0	0
ALDH3A1	GTACCAGTCAGTACACAC	29	36	0	0	0	0
ALDH3A1	TGACCAGTCAGTACACTG	762	514	1	1	0	0
ALDH3A1	ACACCAGTCAGTACTGGT	164	200	0	0	0	0
ALDH3A1	GTTGCAACACTGACACGT	104	92	0	0	0	0
ALDH3A2	GTACCAGTGTTGACACTG	538	225	1	2	0	7
ALDH3A2	TGACCAGTGTTGACTGGT	388	348	0	1	0	0
ALDH3A2	GTACCAGTGTTGACTGCA	43	59	0	0	0	0
ALDH3A2	ACACCAGTGTTGACTGAC	45	35	0	0	0	0
ALDH3A2	CAACCAGTGTTGACTGTG	265	260	0	0	0	0
ALDH3B1	GTACCAGTCAGTGTCATG	214	218	1	1	0	0
ALDH3B1	TGACCAGTCAGTGTACGT	209	321	1	1	0	0
ALDH3B1	GTACCAGTCAGTGTACCA	156	262	0	0	0	0
ALDH3B1	ACACCAGTCAGTGTACAC	26	23	1	0	0	1
ALDH3B1	ACTGCAGTACTGGTCAAC	13	7	0	0	0	0
ALDH3B2	GTACCAGTGTTGTGTGTG	1169	1505	2	3	2	6
ALDH3B2	CAACCAGTCAGTGTGTGT	1170	1574	4	2	0	1
ALDH3B2	ACACCAGTCAGTGTGTCA	1154	763	0	3	0	0
ALDH3B2	CATGACCACAACACCAAC	24	20	0	0	0	0

ALDH3B2	GTTGACCACAACACCATG	696	341	0	1	0	14
ALDH4A1	GTACGTTGTGTGCATGTG	1020	526	1	1	0	7
ALDH4A1	ACACGTTGTGTGACGTGT	128	197	0	1	0	5
ALDH4A1	TGACGTTGTGTGACGTCA	41	21	0	0	0	0
ALDH4A1	CAACGTTGTGTGACGTAC	53	71	0	0	0	0
ALDH4A1	GTACGTTGTGTGACGTTG	340	432	2	0	0	0
ALDH4A1	TGTGCAACTGACTGCAAC	109	125	0	0	0	0
ALDH5A1	TGACCAGTGTGTGTGTAC	17	36	0	0	0	0
ALDH5A1	ACACCAGTGTGTGTGTTG	1194	740	24	0	0	0
ALDH5A1	CAACCAGTGTGTGTCAGT	510	406	0	0	0	0
ALDH5A1	ACACCAGTGTGTGTCACA	8	19	0	0	0	0
ALDH5A1	GTACCAGTGTGTGTCAAC	18	15	1	0	0	0
ALDH5A1	GTTGCAACTGACTGGTCA	300	357	1	0	0	1
ALDH6A1	ACACCAGTCACAACACTG	633	1453	1	1	1	1
ALDH6A1	CAACCAGTCACAACACCA	344	284	1	0	0	0
ALDH6A1	TGACCAGTCACAACACAC	21	61	0	0	0	0
ALDH6A1	CAACCAGTCACAACTGGT	139	79	0	0	0	0
ALDH6A1	ACACCAGTCACAACTGCA	53	67	0	0	0	0
ALDH6A1	ACTGCAACTGACTGCATG	79	163	0	0	0	0
ALDH7A1	GTACCAGTGTTGACCAAC	232	80	0	1	0	0
ALDH7A1	TGACCAGTGTTGACCATG	503	1026	1	2	0	0
ALDH7A1	ACACCAGTGTTGACACGT	206	174	0	0	0	0
ALDH7A1	TGACCAGTGTTGACACCA	328	308	0	1	0	0
ALDH7A1	CAACCAGTGTTGACACAC	22	16	0	0	0	0
ALDH9A1	CAACCAGTGTTGTGCACA	178	191	3	1	0	0
ALDH9A1	TGACCAGTGTTGTGCAAC	20	66	0	0	0	0
ALDH9A1	ACACCAGTGTTGTGCATG	137	121	1	0	0	0
ALDH9A1	CAACCAGTGTTGTGACGT	236	279	1	0	0	3
ALDH9A1	ACACCAGTGTTGTGACCA	481	504	123	0	0	1
ALDOA	CAACACACTGGTTGCAGT	118	194	0	0	1	0
ALDOA	TGACACACTGGTTGGTAC	76	102	0	0	0	2
ALDOA	ACACACACTGGTTGGTTG	447	445	0	0	0	0
ALDOA	ACACACACTGGTTGCACA	80	117	1	0	0	0
ALDOA	ACTGGTGTACCAACCAAC	14	15	0	0	0	0
ALDOB	ACACACACTGGTTGACGT	147	326	0	2	1	0
ALDOB	GTACACACTGGTTGCAAC	33	45	0	0	0	0
ALDOB	TGACACACTGGTTGCATG	157	157	1	0	0	0
ALDOB	TGACACACTGGTTGACCA	167	297	0	1	0	0
ALDOB	CAACACACTGGTTGACAC	37	22	0	0	0	0
ALDOC	GTACACACTGGTTGACTG	170	144	0	0	0	0
ALDOC	TGACACACTGGTTGTGGT	271	248	0	0	0	0
ALDOC	GTACACACTGGTTGTGCA	169	137	1	0	0	0
ALDOC	ACACACACTGGTTGTGAC	19	14	0	0	0	0
ALDOC	CAACACACTGGTTGTGTG	141	78	0	2	0	0
ALG1	ACACCACAACTGGTGTGT	219	216	0	1	0	0
ALG1	TGACCACAACTGGTGTCA	408	184	0	1	0	0
ALG1	ACACCAACGTGTTGCATG	99	152	0	0	0	0
ALG1	CAACCAACGTGTTGACGT	51	30	0	0	0	0
ALG1	ACACCAACGTGTTGACCA	296	177	1	0	0	0
ALG10B	CAACCACACATGCATGAC	20	48	0	0	0	0
ALG10B	GTACCACACATGCATGTG	530	397	0	1	0	0
ALG10B	ACACCACACATGACGTGT	226	208	0	0	0	0
ALG10B	GTACCAACCAACACCACA	4	8	0	0	0	0
ALG10B	ACACCAACCAACACCAAC	13	23	0	0	0	0
ALG11	CATGGTGTCAGTCATGGT	223	104	2	1	0	0
ALG11	TGTGGTCATGCAGTGTAC	168	108	0	1	0	0
ALG11	CATGGTACACTGTGGTAC	58	93	0	1	0	1
ALG11	GTTGGTACACTGTGGTTG	274	454	1	0	0	0
ALG11	TGTGGTACACTGTGCAGT	112	309	1	0	0	0
ALG12	CAACCACATGACTGGTGT	1737	877	0	0	0	2
ALG12	ACACCACATGACTGGTCA	362	153	0	0	0	0
ALG12	GTACCACATGACTGGTAC	58	180	0	0	0	0
ALG12	TGACCACATGACTGGTTG	227	308	0	0	0	0
ALG12	TGTGCAGTACTGCATGAC	61	83	0	0	0	0
ALG13	CAACCACAACGTGTGTCA	597	365	0	0	0	1
ALG13	TGACCACAACGTGTGTAC	169	83	0	0	0	12
ALG13	ACACCACAACGTGTGTTG	811	526	1	0	0	0
ALG13	CAACCACAACGTGTCAGT	145	166	0	0	0	0
ALG13	ACACCACAACGTGTCACA	112	189	0	0	0	0
ALG13	CATGCAACCAACGTACTG	212	376	1	0	0	0
ALG14	GTTGGTTGACCAACACCA	91	120	0	0	0	0
ALG14	ACTGGTTGACCAACACAC	209	142	0	1	0	0
ALG14	CATGGTTGACCAACACTG	343	417	0	1	0	0
ALG14	GTTGGTTGACCAACTGGT	47	87	0	0	0	0
ALG14	CATGACGTGTGTGTACAC	100	106	0	0	0	0

ALG2	CAACCACATGTGGTCAAC	3	95	0	0	0	0
ALG2	GTACCACATGTGGTCATG	24	46	0	0	0	0
ALG2	TGACCACATGTGGTACGT	147	138	0	0	0	1
ALG2	GTACCACATGTGGTACCA	21	36	0	0	0	0
ALG2	ACACCACATGTGGTACAC	43	18	0	0	0	0
ALG3	CAACCACATGACCAACGT	378	120	2	1	0	0
ALG3	ACACCACATGACCAACCA	383	329	0	0	0	1
ALG3	GTACCACATGACCAACAC	264	180	0	0	0	0
ALG3	TGACCACATGACCAACTG	199	270	1	0	0	0
ALG3	ACTGCAGTGTGTACGTAC	41	32	0	1	0	0
ALG5	CAACCACATGCAGTGTAC	20	16	1	1	0	0
ALG5	GTACCACATGCAGTGTTG	203	222	0	0	0	1
ALG5	TGACCACATGCAGTCAGT	179	192	0	0	0	0
ALG5	GTACCACATGCAGTCACA	46	114	0	0	0	0
ALG5	ACACCACATGCAGTCAAC	45	34	1	0	0	0
ALG6	TGACCAACCAACTGACGT	100	92	0	0	0	0
ALG6	GTACCAACCAACTGACCA	371	778	1	0	0	0
ALG6	ACACCAACCAACTGACAC	101	64	0	0	0	0
ALG6	CAACCAACCAACTGACTG	260	294	0	0	0	0
ALG6	GTACCAACCAACTGTGGT	479	631	3	0	0	0
ALG8	TGACCACACAACACTGAC	46	25	0	0	0	0
ALG8	ACACCACACAACACTGTG	92	114	0	0	0	0
ALG8	GTACCACACAACTGGTGT	475	344	0	0	0	0
ALG8	CAACCACACAACTGGTCA	158	206	1	0	0	3
ALG8	TGACCACACAACTGGTAC	90	101	0	0	0	0
ALG9	CAACCACACATGGTCAGT	268	474	0	0	0	0
ALG9	ACACCACACATGGTCACA	20	41	0	0	0	0
ALG9	GTACCACACATGGTCAAC	41	54	0	0	0	0
ALG9	TGACCACACATGGTCATG	125	149	0	0	0	3
ALG9	ACACCACACATGGTACGT	99	176	0	0	0	0
ALK	CACATGCAACTGACCAGT	241	290	0	0	0	0
ALK	ACCATGCAACTGACCACA	322	142	1	0	0	0
ALK	GTCATGCAACTGACCAAC	14	41	0	0	0	0
ALK	TGCATGCAACTGACCATG	156	189	0	0	0	0
ALK	ACCATGCAACTGACACGT	14	25	0	0	0	0
ALK	ACTGCATGGTACGTGTCA	25	31	0	0	0	1
ALKBH2	TGACTGACACACCATGCA	124	256	1	0	0	0
ALKBH2	CAACTGACACACCATGAC	123	87	1	0	0	0
ALKBH2	GTACTGACACACCATGTG	268	300	1	0	0	4
ALKBH2	ACACTGACACACACGTGT	1185	733	0	0	0	0
ALKBH2	TGACTGACACACACGTCA	571	386	1	1	0	0
ALKBH3	ACACTGACACTGGTCAGT	198	258	1	0	0	0
ALKBH3	TGACTGACACTGGTCACA	13	26	0	0	0	0
ALKBH3	CAACTGACACTGGTCAAC	21	35	0	0	0	0
ALKBH3	GTACTGACACTGGTCATG	418	423	1	0	0	0
ALKBH3	TGACTGACACTGGTACGT	396	105	0	0	0	0
ALKBH3	TGTGCAACCATGCACACA	40	59	0	0	0	0
ALLC	TGACACACGTGTCATGGT	408	219	1	1	0	0
ALLC	GTACACACGTGTCATGCA	56	58	0	0	0	0
ALLC	CATGCATGTGACACACTG	148	213	0	0	0	0
ALLC	GTTGCATGTGACACTGGT	258	301	0	0	0	0
ALLC	CATGCATGTGACACTGCA	199	141	0	0	0	0
ALMS1	ACTGGTGTGTACACGTCA	209	278	1	0	0	0
ALMS1	GTTGGTGTGTACACGTAC	24	13	0	0	0	0
ALMS1	TGTGGTGTGTACACGTTG	205	217	1	1	0	0
ALMS1	ACTGGTGTGTACACCAGT	31	48	0	0	0	0
ALMS1	GTTGGTCAACTGTGGTAC	92	132	1	0	0	14
ALMS1	CATGCAACTGTGTGGTCA	507	686	2	0	0	1
ALOX12	CAACACTGCAACTGACTG	186	229	0	0	0	0
ALOX12	GTACACTGCAACTGTGGT	228	763	1	0	0	0
ALOX12	CAACACTGCAACTGTGCA	853	599	2	0	0	0
ALOX12	TGACACTGCAACTGTGAC	26	37	0	0	0	0
ALOX12	ACACACTGCAACTGTGTG	339	458	0	2	0	0
ALOX12B	ACTGCAGTTGCACACAGT	318	190	2	0	5	4
ALOX12B	TGACACTGCATGGTCACA	92	55	0	0	0	0
ALOX12B	CAACACTGCATGGTCAAC	37	45	0	0	0	0
ALOX12B	GTACACTGCATGGTCATG	307	282	0	0	0	0
ALOX12B	TGACACTGCATGGTACGT	98	119	0	0	0	0
ALOX15	GTACACTGCATGGTACCA	127	124	0	0	0	0
ALOX15	ACACACTGCATGGTACAC	97	91	0	0	0	0
ALOX15	CAACACTGCATGGTACTG	793	1436	90	2	0	0
ALOX15	GTACACTGCATGGTTGGT	479	448	1	0	0	0
ALOX15	TGTGCAGTTGCACACACA	84	87	0	0	0	0
ALOX15B	CAACACTGCATGGTTGCA	146	278	0	0	1	11
ALOX15B	TGACACTGCATGGTTGAC	34	85	0	1	0	0

ALOX15B	ACACACTGCATGGTTGTG	399	491	0	1	0	0
ALOX15B	GTTGACCAACACGTCATG	22	21	0	0	0	0
ALOX15B	TGTGACCAACACGTACGT	289	271	0	0	0	5
ALOX5	ACACACTGCATGGTGTCA	285	405	3	6	1	4
ALOX5	TGACACTGCATGGTGTTG	396	567	0	1	1	1
ALOX5	CAACACTGCATGGTGTGT	348	350	1	0	0	0
ALOX5	GTACACTGCATGGTGTAC	25	68	0	0	0	0
ALOX5	ACACACTGCATGGTCAGT	75	84	0	0	0	0
ALOX5AP	GTACTGTGCAACACACCA	130	138	0	0	0	0
ALOX5AP	ACACTGTGCAACACACAC	32	30	0	0	0	0
ALOX5AP	CAACTGTGCAACACACTG	502	472	1	0	0	2
ALOX5AP	GTACTGTGCAACACTGGT	551	369	0	0	0	1
ALOX5AP	CAACTGTGCAACACTGCA	83	136	0	0	0	0
ALOX5AP	CATGCAACGTACGTGTCA	347	396	0	14	0	0
ALPI	CAACACACCATGCAACGT	512	684	0	0	0	0
ALPI	ACACACACCATGCAACCA	95	172	0	0	0	0
ALPI	GTACACACCATGCAACAC	52	27	0	0	0	0
ALPI	TGACACACCATGCAACTG	342	630	1	1	0	0
ALPI	ACTGCAGTTGCAGTCAAC	12	0	0	0	0	0
ALPI	TGTGCAACGTGTGTTGCA	79	87	0	1	0	0
ALPL	TGACACACACACGTTGAC	25	16	0	0	0	0
ALPL	ACACACACACACGTTGTG	395	215	0	0	0	0
ALPL	GTACACACACACCAGTGT	41	35	0	0	0	0
ALPL	CAACACACACACCAGTCA	229	287	1	2	0	0
ALPL	ACTGCAGTTGCAGTGTGT	384	1017	1	1	0	1
ALPP	TGTGACCATGACTGGTTG	842	1608	4	1	2	140
ALPP	CAACACACGTCACAACGT	281	462	0	0	0	0
ALPP	ACTGACCATGACTGGTCA	152	79	1	0	0	0
ALPP	GTTGACCATGACTGGTAC	41	38	0	0	0	0
ALPP	ACTGACCATGACTGCAGT	167	121	0	0	0	0
ALPPL2	ACACACACGTCACACATG	368	575	0	0	0	0
ALPPL2	CAACACACCAGTCACAAC	83	44	0	0	0	0
ALPPL2	GTACACACCAGTCACATG	256	191	2721	4	0	0
ALPPL2	TGACACACCAGTCAACGT	267	357	1	0	0	0
ALPPL2	GTACACACCAGTCAACCA	627	585	640	0	0	0
ALPPL2	CATGCAACGTGTACGTGT	137	177	0	0	0	0
ALS2	GTACCATGACCACAACTG	300	234	1	0	0	0
ALS2	TGACCATGACCACATGGT	302	290	1	0	0	0
ALS2	CAACACCACAGTACACGT	70	68	1	0	0	0
ALS2	ACACACCACAGTACACCA	159	137	0	1	0	0
ALS2	GTACACCACAGTACACAC	25	38	0	0	0	0
AMD1	TGACTGTGACCAGTGTGT	152	219	0	1	0	0
AMD1	GTACTGTGACCAGTGTCA	839	508	1	13	0	0
AMD1	ACACTGTGACCAGTGTAC	16	25	0	0	0	0
AMD1	ACTGGTGTACCACATGGT	134	131	0	0	0	1
AMD1	GTTGACCACATGCACAGT	154	217	1	0	0	0
AMDHD1	ACTGCAACACTGCATGGT	31	48	0	0	1	0
AMDHD1	ACACACCAGTCACAGTGT	134	128	0	0	0	1
AMDHD1	TGACACCAGTCACAGTCA	566	448	0	0	0	0
AMDHD1	CAACACCAGTCACAGTAC	308	137	0	0	0	2
AMDHD1	GTACACCAGTCACAGTTG	387	451	1	0	0	3
AMDHD1	TGACACCAGTCACACAGT	99	141	1	0	0	0
AMDHD2	ACACACCAGTCAGTGTAC	29	75	3	1	1	0
AMDHD2	GTTGCAACACGTCAACCA	414	455	0	1	1	0
AMDHD2	ACACCAGTTGTGACCAAC	89	68	0	0	0	0
AMDHD2	CAACACCAGTCAGTGTTG	226	253	0	0	0	0
AMDHD2	GTACACCAGTCAGTCAGT	483	235	1	0	0	0
AMDHD2	CAACACCAGTCAGTCACA	278	472	0	0	0	1
AMH	TGACTGGTTGGTGTTGGT	485	463	9	1	2	9
AMH	CAACTGGTTGGTGTACAC	31	97	0	0	0	0
AMH	GTACTGGTTGGTGTACTG	209	240	0	1	0	0
AMH	CATGCAGTTGCATGTGAC	23	53	0	0	0	0
AMH	ACTGCACAACACGTGTGT	612	783	1	1	0	6
AMH	TGTGCACAACACGTGTCA	561	267	0	0	0	0
AMHR2	GTTGCACATGCACACACA	263	126	0	0	1	67
AMHR2	GTCATGACGTTGGTGTGT	1404	1194	0	0	0	7
AMHR2	CACATGACGTTGGTGTCA	553	368	0	2	0	0
AMHR2	TGCATGACGTTGGTGTAC	34	48	1	0	0	0
AMHR2	ACCATGACGTTGGTGTTG	1204	1303	1	2	0	36
AMHR2	ACTGCACATGCACACAAC	27	23	0	7	0	0
AMOTL1	CATGGTCAACACACCAAC	51	54	0	0	0	0
AMOTL1	GTTGGTCAACACACCATG	458	314	0	1	0	0
AMOTL1	TGTGGTCAACACACACGT	23	28	0	1	0	0
AMOTL1	GTTGGTCAACACACACCA	164	186	0	1	0	0
AMOTL1	ACTGGTCAACACACACAC	29	26	0	0	0	0

AMOTL1	ACTGCAACACACGTTGGT	106	134	0	0	0	1
AMPD1	ACACACCATGTGCATGGT	275	586	1	0	1	2
AMPD1	ACACACCATGTGCAACCA	224	154	0	0	0	0
AMPD1	GTACACCATGTGCAACAC	79	67	0	0	0	0
AMPD1	TGACACCATGTGCAACTG	140	245	0	0	0	1
AMPD1	TGACACCATGTGCATGCA	386	281	0	0	0	0
AMPD1	CATGCACACACAACGTCA	590	921	0	0	0	0
AMPD2	TGACACACACCAGTACTG	1607	1335	1	1	1	5
AMPD2	ACACACACACCAGTTGGT	103	118	0	0	0	0
AMPD2	TGTGCAGTTGTGACCAAC	76	89	0	0	0	0
AMPD2	TGTGACGTTGGTGTCAGT	150	154	1	0	0	0
AMPD2	GTTGACGTTGGTGTCACA	21	22	0	0	0	0
AMPD3	TGACACACACACGTCACA	49	68	1	0	0	0
AMPD3	CAACACACACACGTCAAC	73	15	0	0	0	0
AMPD3	GTACACACACACGTCATG	351	400	0	6	0	0
AMPD3	TGACACACACACGTACGT	274	524	1	0	0	0
AMPD3	GTACACACACACGTACCA	181	170	0	1	0	6
AMPH	GTTGCAGTACCATGGTGT	1188	1203	5	1	3	0
AMPH	ACACGTGTTGGTCAACTG	397	448	0	0	0	0
AMPH	CAACGTGTTGGTCATGGT	565	172	1	0	0	0
AMPH	ACACGTGTTGGTCATGCA	95	168	0	0	0	0
AMPH	GTACGTGTTGGTCATGAC	31	30	0	0	0	0
AMPH	GTTGCAACACCACAGTGT	649	826	2	0	0	0
AMT	GTACCACAACGTACGTTG	586	585	1	0	1	0
AMT	TGACCACAACGTACCAGT	156	538	0	1	0	11
AMT	GTACCACAACGTACCACA	38	39	0	0	0	0
AMT	GTTGGTTGTGCAGTGTTG	244	245	0	0	0	0
AMT	TGTGGTTGTGCAGTCAGT	60	110	0	0	0	0
AMY1A	CAACACTGCACAGTCATG	204	300	0	0	0	0
AMY1A	CAACACTGCACAGTACCA	97	79	0	0	0	0
AMY1A	TGACACTGCACACAGTTG	392	706	0	0	0	0
AMY1A	ACACACTGCACACACAGT	324	434	0	0	0	0
AMY1A	TGACACTGCACACATGAC	59	38	0	0	0	0
AMY1B	ACACACTGCACACATGTG	1323	1881	3	2	3	12
AMY1B	GTACACTGCACAACGTGT	116	90	0	0	0	0
AMY1B	CAACACTGCACAACGTCA	539	584	0	0	0	9
AMY1B	GTTGGTACTGCAGTCATG	358	579	0	1	0	7
AMY1B	TGTGGTACTGCAGTACGT	184	200	0	0	0	0
AMY1C	GTTGGTACTGCAGTACCA	194	234	1	0	0	0
AMY1C	ACTGGTACTGCAGTACAC	50	25	0	0	0	0
AMY1C	CATGGTACTGCAGTACTG	400	276	0	0	0	0
AMY1C	TGTGGTTGCAGTACACCA	390	474	0	0	0	0
AMY1C	CATGGTTGCAGTACACAC	41	64	0	0	0	0
AMY2A	CAACACTGCACACAGTGT	663	957	3	0	1	0
AMY2A	GTACACTGCACAGTACGT	118	165	0	0	0	0
AMY2A	TGACACTGCACAGTACAC	94	205	0	0	0	0
AMY2A	ACACACTGCACACAGTCA	253	408	0	0	0	9
AMY2A	GTACACTGCACACAGTAC	34	13	0	0	0	0
AMY2B	GTTGGTTGCAGTACACTG	465	595	0	0	0	0
AMY2B	TGTGGTTGCAGTACTGGT	643	280	1	11	0	261
AMY2B	GTTGGTTGCAGTACTGCA	50	46	1	0	0	0
AMY2B	ACTGCAGTGTGTTGGTGT	237	1047	0	1	0	1
AMY2B	TGTGCAGTGTGTTGGTCA	491	412	1	1	0	0
ANAPC1	ACTGGTTGCAGTACTGAC	58	51	0	0	0	0
ANAPC1	CATGGTTGCAGTACTGTG	54	82	0	0	0	0
ANAPC1	TGTGGTTGCAGTTGGTGT	1424	857	3	22	0	22
ANAPC1	GTTGACACGTACCAACGT	127	178	3	0	0	0
ANAPC1	CATGACACGTACCAACCA	146	285	0	1	0	1
ANAPC10	CACATGTGCAGTTGGTGT	422	255	0	0	0	0
ANAPC10	ACCATGTGCAGTTGGTCA	25	55	0	0	0	0
ANAPC10	GTCATGTGCAGTTGGTAC	61	76	1	131	0	0
ANAPC10	TGCATGTGCAGTTGGTTG	407	475	0	1	0	0
ANAPC10	CATGCAGTCATGTGGTGT	1094	947	2	0	0	1
ANAPC11	CAACCATGGTCATGTGAC	24	34	0	0	0	0
ANAPC11	GTACCATGGTCATGTGTG	264	313	0	1	0	0
ANAPC11	GTACCATGGTACGTGTGT	162	172	1	0	0	0
ANAPC11	CAACCATGGTACGTGTCA	417	411	0	0	0	4
ANAPC11	TGACCATGGTACGTGTAC	148	42	1	0	0	0
ANAPC2	TGCATGTGCAGTCAACTG	99	84	0	0	0	0
ANAPC2	ACCATGTGCAGTCATGGT	117	126	0	0	0	0
ANAPC2	TGCATGTGCAGTCATGCA	100	186	0	1	0	0
ANAPC2	CACATGTGCAGTCATGAC	89	89	0	0	0	0
ANAPC2	TGTGCAGTGTTGACCACA	75	45	0	0	0	0
ANAPC4	GTCATGTGCAGTCATGTG	125	141	0	0	0	0
ANAPC4	ACCATGTGCAGTACGTGT	32	78	0	0	0	0

ANAPC4	TGCATGTGCAGTACGTCA	77	71	0	0	0	0
ANAPC4	CACATGTGCAGTACGTAC	123	108	0	0	0	0
ANAPC4	GTCATGTGCAGTACGTTG	504	659	232	0	0	0
ANAPC5	CACATGTGGTTGTGGTAC	99	103	0	0	0	0
ANAPC5	GTCATGTGGTTGTGGTTG	577	831	0	1	0	0
ANAPC5	TGCATGTGGTTGTGCAGT	191	220	0	1	0	2
ANAPC5	GTCATGTGGTTGTGCACA	14	21	0	0	0	0
ANAPC5	ACCATGTGGTTGTGCAAC	29	78	0	0	0	0
ANAPC7	GTTGGTGTGTTGACTGAC	21	23	0	0	0	0
ANAPC7	GTTGGTTGCAGTTGGTCA	251	255	0	0	0	1
ANAPC7	ACTGGTTGCAGTTGGTAC	3	15	0	0	0	0
ANAPC7	CATGGTTGCAGTTGGTTG	803	712	0	0	0	0
ANAPC7	GTTGGTTGCAGTTGCAGT	114	147	1	0	0	0
ANAPC7	CATGCACAACTGACGTGT	1056	521	0	0	0	0
ANGPT1	ACACTGGTTGGTCACATG	275	266	0	0	0	104
ANGPT1	CAACTGGTTGGTCAACGT	728	517	0	0	0	54
ANGPT1	ACACTGGTTGGTCAACCA	200	153	0	0	0	0
ANGPT1	GTACTGGTTGGTCAACAC	107	78	0	0	0	0
ANGPT1	TGACTGACTGCATGCAGT	34	49	0	0	0	0
ANGPT1	CATGCAACACGTGTGTAC	20	24	0	0	0	0
ANGPT2	ACACTGCAGTACCATGGT	772	788	1	2	0	47
ANGPT2	TGACTGCAGTACCATGCA	185	338	0	0	0	0
ANGPT2	CAACTGCAGTACCATGAC	105	88	0	0	0	0
ANGPT2	GTACTGCAGTACCATGTG	244	142	1	0	0	0
ANGPT2	ACACTGCAGTACACGTGT	535	200	0	0	0	0
ANGPT2	TGTGCACACACAACGTAC	31	38	0	0	0	0
ANGPTL2	TGTGGTACTGCAACACTG	134	233	0	0	1	0
ANGPTL2	GTTGGTTGCAGTCACATG	425	589	1	1	0	0
ANGPTL2	TGTGGTTGCAGTCAACGT	187	695	0	0	0	0
ANGPTL2	GTTGGTTGCAGTCAACCA	178	131	0	0	0	0
ANGPTL2	ACTGGTTGCAGTCAACAC	21	24	0	0	0	0
ANGPTL3	TGTGCAGTGTGTGTCACA	90	145	0	0	0	0
ANGPTL3	CATGCAGTGTGTGTCAAC	20	27	0	0	0	0
ANGPTL3	GTTGCAGTGTGTGTCATG	267	388	0	0	0	0
ANGPTL3	TGTGCAGTGTGTGTACGT	183	153	0	0	0	1
ANGPTL3	TGTGCATGTGTGTGACGT	68	86	0	0	0	0
ANGPTL4	CATGGTACACGTTGCAGT	67	174	0	0	0	0
ANGPTL4	ACTGGTACACGTTGCACA	60	60	0	0	0	0
ANGPTL4	GTTGGTACACGTTGCAAC	28	20	0	0	0	0
ANGPTL4	TGTGACCATGGTTGCATG	165	169	0	0	0	0
ANGPTL4	ACTGACCATGGTTGACGT	133	204	1	2	0	4
ANGPTL6	CATGGTTGCAGTTGCACA	111	127	0	0	0	0
ANGPTL6	TGTGGTTGCAGTTGCAAC	6	27	0	0	0	0
ANGPTL6	ACTGGTTGCAGTTGCATG	63	59	0	0	0	0
ANGPTL6	CATGGTTGCAGTTGACGT	60	457	0	0	0	0
ANGPTL6	ACTGGTTGCAGTTGACCA	67	64	1	0	0	0
ANGPTL6	CATGCATGGTACACACGT	71	71	0	0	0	0
ANK2	CAACTGACACTGTGTGGT	982	881	2	0	1	37
ANK2	TGACTGACACTGTGACAC	56	162	0	0	0	0
ANK2	ACACTGACACTGTGACTG	82	85	0	0	0	0
ANK2	ACACTGACACTGTGTGCA	68	210	0	0	0	0
ANK2	GTACTGACACTGTGTGAC	97	87	0	0	0	0
ANKFY1	TGACTGCAGTGTTGGTAC	74	103	0	0	0	0
ANKFY1	ACACTGCAGTGTTGGTTG	398	679	0	0	0	0
ANKFY1	CAACTGCAGTGTTGCAGT	99	91	0	0	0	0
ANKFY1	ACACTGCAGTGTTGCACA	101	75	5	0	0	3
ANKFY1	GTACTGCAGTGTTGCAAC	18	6	0	0	0	0
ANKHD1	ACTGGTACACTGACTGGT	107	41	0	0	0	0
ANKHD1	TGTGGTACACTGACTGCA	18	23	0	0	0	0
ANKHD1	CATGGTACACTGACTGAC	23	47	0	0	0	0
ANKHD1	GTTGGTACACTGACTGTG	101	106	0	0	0	0
ANKHD1	ACTGGTACACTGTGGTGT	167	242	0	0	0	0
ANKRD1	ACTGGTCATGACTGTGGT	265	222	1	0	3	0
ANKRD1	CATGGTCATGACTGACGT	123	235	0	0	0	1
ANKRD1	ACTGGTCATGACTGACCA	51	66	0	1	0	0
ANKRD1	GTTGGTCATGACTGACAC	99	79	1	0	0	0
ANKRD1	TGTGGTCATGACTGACTG	141	233	0	0	0	0
ANKRD6	TGTGGTTGCAGTCAGTTG	659	507	3	0	5	1
ANKRD6	GTTGGTTGCAGTCAGTAC	551	389	1	0	0	0
ANKRD6	ACTGGTTGCAGTCACAGT	269	735	1	1	0	0
ANKRD6	TGTGGTTGCAGTCACACA	59	166	0	0	0	1
ANKRD6	CATGGTTGCAGTCACAAC	16	33	0	0	0	0
ANP32A	GTACGTGTCACAACCAGT	570	497	2	0	0	123
ANP32A	CAACGTGTCACAACCACA	54	66	0	0	0	0
ANP32A	TGACGTGTCACAACCAAC	42	38	0	0	0	0

ANP32A	ACACGTGTCACAACCATG	60	32	0	0	0	0
ANP32A	CAACGTGTCACAACACGT	37	7	0	0	0	0
ANP32A	CATGCAACCAACCAGTCA	147	161	0	1	0	0
ANP32B	GTACTGTGCACAACACAC	10	20	0	0	0	0
ANP32B	TGACTGTGCACATGCAGT	175	478	1	0	0	0
ANP32B	GTACTGTGCACATGCACA	30	160	0	0	0	2
ANP32B	ACACTGTGCACATGCAAC	26	32	0	0	0	0
ANP32B	CAACTGTGCACATGCATG	228	242	3	0	0	1
ANP32E	TGACTGTGCACAACACTG	922	841	1	1	0	0
ANP32E	ACACTGTGCACAACTGGT	245	281	0	0	0	0
ANP32E	GTACTGTGCACATGACGT	264	240	0	0	0	0
ANP32E	CAACTGTGCACATGACCA	560	467	57	1	0	0
ANP32E	TGACTGTGCACATGACAC	12	23	0	0	0	0
ANP32E	CATGCAACTGGTACCACA	93	71	1	0	0	0
ANPEP	GTACACCATGTGCACAGT	69	132	1	0	0	0
ANPEP	CAACACCATGTGCACACA	50	53	0	0	0	0
ANPEP	TGACACCATGTGCACAAC	23	59	0	0	0	0
ANPEP	ACACACCATGTGCACATG	144	133	1	0	0	0
ANPEP	CAACACCATGTGCAACGT	557	586	1	1	0	65
ANPEP	ACTGCACAGTCACATGAC	237	127	0	0	0	0
ANXA1	ACACACTGCACAACCACA	12	20	0	0	0	0
ANXA1	GTACACTGCACAACCAAC	53	11	0	0	0	0
ANXA1	TGACACTGCACAACCATG	98	119	1	0	0	0
ANXA1	ACACACTGCACAACACGT	207	174	0	0	0	0
ANXA1	TGTGCAGTCACATGACAC	107	78	1	1	0	0
ANXA1	CATGCACAGTCACATGTG	992	1085	1	0	0	0
ANXA2	GTACACTGCACAACACTG	1297	963	0	0	1	1
ANXA2	TGACACTGCACAACACCA	177	188	0	0	0	0
ANXA2	CAACACTGCACAACACAC	72	206	0	0	0	0
ANXA2	TGACACTGCACAACTGGT	395	421	0	0	0	0
ANXA2	CATGCAGTCATGGTCAGT	492	99	0	0	0	0
ANXA3	ACACACTGCACATGTGGT	809	1206	3	1	1	4
ANXA3	TGACACTGCACATGTGCA	240	256	0	0	0	0
ANXA3	CAACACTGCACATGTGAC	108	73	0	0	0	0
ANXA3	GTACACTGCACATGTGTG	222	301	0	0	0	25
ANXA3	GTACACTGCAACGTGTGT	1329	1050	1	3	0	0
ANXA4	CAACACTGCAACGTTGTG	598	193	1	0	1	0
ANXA4	GTACACTGCAACGTACTG	255	174	1	0	0	0
ANXA4	TGACACTGCAACGTTGGT	1586	570	1	201	0	2
ANXA4	GTACACTGCAACGTTGCA	254	222	0	0	0	0
ANXA4	ACACACTGCAACGTTGAC	42	89	0	10	0	0
ANXA4	ACTGCACACATGACGTAC	84	99	0	0	0	1
ANXA5	GTACCAGTACTGGTTGGT	188	205	0	0	0	0
ANXA5	CAACCAGTACTGGTTGCA	484	111	1	0	0	0
ANXA5	TGACCAGTACTGGTTGAC	63	31	0	0	0	0
ANXA5	ACACCAGTACTGGTTGTG	321	246	0	0	0	0
ANXA5	GTACCAGTACTGCAGTGT	797	553	0	1	0	0
ANXA5	CATGCACACATGACGTTG	1482	1114	0	0	0	3
ANXA6	ACTGCAGTACCAACACAC	84	80	0	1	1	0
ANXA6	TGACGTCAGTTGACTGTG	204	208	1	0	0	0
ANXA6	CAACGTCAGTTGTGGTGT	97	81	0	0	0	0
ANXA6	ACACGTCAGTTGTGGTCA	31	69	0	0	0	0
ANXA6	CATGCAGTACCAACACTG	1095	593	1	0	0	0
ANXA8	TGTGCACAGTCAACGTGT	482	236	0	0	1	0
ANXA8	CAACACTGCACAGTGTAC	211	104	0	0	0	1
ANXA8	GTACACTGCACAGTGTTG	626	1053	0	1	0	0
ANXA8	TGACACTGCACAGTCAGT	109	155	0	0	0	0
ANXA8	GTACACTGCACAGTCACA	82	78	0	1	0	0
ANXA8	ACACACTGCACAGTCAAC	42	31	0	0	0	0
AOC2	CAACACGTTGGTTGCATG	270	324	0	0	0	0
AOC2	GTACACGTTGGTTGACGT	474	857	2	1	0	0
AOC2	CAACACGTTGGTTGACCA	114	104	0	0	0	0
AOC2	TGACACGTTGGTTGACAC	42	30	0	0	0	0
AOC2	GTTGACGTTGTGCAACAC	15	8	0	0	0	0
AOC3	GTACACGTTGCAGTGTGT	454	307	1	0	0	2
AOC3	CAACACGTTGCAGTGTCA	386	246	0	0	0	0
AOC3	TGACACGTTGCAGTGTAC	58	105	0	0	0	0
AOC3	ACACACGTTGCAGTGTTG	157	121	0	1	0	0
AOC3	CAACACGTTGCAGTCAGT	152	118	0	0	0	0
AOC3	CATGCACAACACCAGTGT	181	210	0	0	0	0
AOX1	GTACACGTACTGTGTGGT	790	131	34	2	49	23
AOX1	TGACACGTACTGTGACGT	85	69	0	0	0	0
AOX1	GTACACGTACTGTGACCA	25	13	0	0	0	1
AOX1	ACACACGTACTGTGACAC	43	48	0	0	0	0
AOX1	CAACACGTACTGTGACTG	558	542	0	0	0	0

AP2A1	CAACTGACTGCAGTGTGT	1397	996	2	0	1	4
AP2A1	CAACTGACTGGTTGTGAC	53	71	0	2	0	0
AP2A1	GTACTGACTGGTTGTGTG	247	264	1	0	0	0
AP2A1	ACACTGACTGCAGTGTCA	164	170	0	0	0	0
AP2A1	GTACTGACTGCAGTGTAC	56	80	0	0	0	0
AP2A1	CATGCAACCACAGTACGT	122	98	0	0	0	0
AP2B1	CATGGTACACTGTGTGGT	868	562	0	0	0	0
AP2B1	ACTGGTACACTGTGTGCA	137	95	0	0	0	0
AP2B1	GTTGGTACACTGTGTGAC	54	63	4	0	0	0
AP2B1	TGTGGTACACTGTGTGTG	79	76	0	0	0	0
AP2B1	ACTGGTACTGGTGTGTGT	522	594	0	0	0	0
AP2M1	ACACTGCACATGCACAGT	658	440	0	1	1	4
AP2M1	GTACTGCACATGCAGTAC	8	153	0	0	0	0
AP2M1	TGACTGCACATGCAGTTG	132	128	0	0	0	0
AP2M1	TGACTGCACATGCACACA	42	30	0	0	0	3
AP2M1	CAACTGCACATGCACAAC	9	58	0	0	0	0
AP3B1	TGACTGACTGGTACTGGT	552	1094	2	1	0	18
AP3B1	GTACTGACTGGTACTGCA	82	130	1	0	0	3
AP3B1	ACACTGACTGGTACTGAC	301	96	0	1	0	0
AP3B1	CAACTGACTGGTACTGTG	912	530	0	0	0	1
AP3B1	TGACTGACTGGTTGGTGT	544	287	2	0	0	0
AP3M1	ACACTGACTGGTTGTGGT	762	2065	0	3	1	0
AP3M1	ACACTGACTGGTTGACCA	141	187	0	0	0	1
AP3M1	GTACTGACTGGTTGACAC	50	138	0	0	0	0
AP3M1	TGACTGACTGGTTGACTG	853	486	1	1	0	0
AP3M1	TGACTGACTGGTTGTGCA	393	430	1	0	0	0
APAF1	CAACGTGTCAGTGTCATG	217	269	0	0	0	0
APAF1	GTACGTGTCAGTGTACGT	217	223	0	0	0	0
APAF1	CAACGTGTCAGTGTACCA	157	152	0	0	0	20
APAF1	TGACGTGTCAGTGTACAC	37	32	0	0	0	0
APAF1	TGTGCATGCACATGCACA	8	16	0	0	0	0
APAF1	CATGCATGCACATGCAAC	60	68	0	0	0	0
APBA1	TGACTGACCACACATGCA	225	596	2	0	2	0
APBA1	GTTGCAACACACGTTGTG	72	93	1	898	2	2
APBA1	TGACTGACCACACAACTG	235	175	0	0	0	0
APBA1	ACACTGACCACACATGGT	281	622	1	1	0	0
APBA1	CAACTGACCACACATGAC	104	68	0	0	0	0
APBA1	TGTGCAGTTGTGCAGTAC	60	74	0	0	0	0
APBA2	ACACTGACCACAACGTGT	627	542	1	1	2	0
APBA2	ACTGCAACACACCAGTGT	1670	986	2	1	2	5
APBA2	GTACTGACCACAACGTTG	292	554	0	0	1	0
APBA2	GTACTGACCACACATGTG	430	513	0	1	0	0
APBA2	TGACTGACCACAACGTCA	201	120	0	1	0	0
APBA2	CAACTGACCACAACGTAC	73	38	0	1	0	0
APBA3	ACTGGTTGCAGTTGTGGT	199	207	0	0	1	0
APBA3	GTTGGTTGCAGTTGACAC	26	44	0	0	0	0
APBA3	TGTGGTTGCAGTTGACTG	776	717	1	1	0	0
APBA3	TGTGGTTGCAGTTGTGCA	92	124	2	0	0	0
APBA3	CATGGTTGCAGTTGTGAC	59	82	0	0	0	0
APBB1	ACACTGTGTGTGCAACGT	980	388	0	1	0	1
APBB1	TGACTGTGTGTGCAACCA	294	279	0	0	0	0
APBB1	CAACTGTGTGTGCAACAC	7	23	0	0	0	0
APBB1	GTACTGTGTGTGCAACTG	109	157	0	0	0	0
APBB1	TGACTGTGTGTGCATGGT	454	406	0	0	0	0
APBB1	TGTGCAACACACCAGTCA	287	261	0	0	0	0
APBB2	GTACGTACCATGTGACAC	240	39	0	0	0	0
APBB2	TGACGTACCATGTGACTG	371	593	0	0	0	1
APBB2	ACACGTACCATGTGTGGT	243	143	0	0	0	17
APBB2	TGACGTACCATGTGTGCA	304	272	1	0	0	1
APBB2	ACTGCAGTACACACACCA	80	56	0	0	0	0
APBB3	GTACTGCAACCAGTCATG	335	304	0	1	1	4
APBB3	TGACTGCAACCAGTACGT	695	692	4	0	0	0
APBB3	GTACTGCAACCAGTACCA	119	74	0	0	0	0
APBB3	ACACTGCAACCAGTACAC	46	52	0	0	0	0
APBB3	CAACTGCAACCAGTACTG	679	1220	1	1	0	0
APC	GTACCATGCATGACACTG	86	136	0	0	0	0
APC	TGACCATGCATGACTGGT	375	363	0	0	0	0
APC	GTACCATGCATGACTGCA	85	70	0	0	0	1
APC	ACACCATGCATGACTGAC	53	48	0	0	0	0
APC	CAACCATGCATGACTGTG	689	413	1	1	0	15
APC2	GTTGGTACACCACAGTAC	61	78	1	0	0	0
APC2	TGTGGTACACCACAGTTG	326	343	3	0	0	0
APC2	ACTGGTACACCACACAGT	168	120	0	0	0	0
APC2	TGTGGTACACCACACACA	7	19	0	0	0	0
APC2	CATGGTACACCACACAAC	194	106	0	0	0	0

APCS	CATGGTCAGTTGGTACTG	1083	960	2	0	1	2
APCS	TGTGGTCAGTTGGTACGT	473	708	2	1	0	6
APCS	GTTGGTCAGTTGGTACCA	133	40	0	0	0	0
APCS	ACTGGTCAGTTGGTACAC	46	37	0	0	0	0
APCS	CATGCACAGTGTACACAC	30	48	0	0	0	0
APCS	GTTGCAACCAGTGTGTGT	473	514	0	2	0	1
APEX1	ACACGTGTACTGTGACCA	386	479	0	0	0	14
APEX1	GTACGTGTACTGTGACAC	17	12	0	0	0	0
APEX1	TGACGTGTACTGTGACTG	562	787	0	1	0	42
APEX1	ACACGTGTACTGTGTGGT	241	206	0	0	0	0
APEX1	ACACACTGGTTGACTGCA	16	30	0	0	0	0
APEX1	TGTGCAACGTTGTGACAC	32	18	0	0	0	0
APEX2	ACTGCAACGTTGTGACTG	918	667	0	0	1	1
APEX2	ACACACCATGGTTGTGGT	131	208	1	0	0	9
APEX2	TGACACCATGGTTGTGCA	162	120	0	1	0	0
APEX2	CAACACCATGGTTGTGAC	22	45	0	0	0	0
APEX2	GTACACCATGGTTGTGTG	237	216	1	0	0	0
APEX2	ACTGCAGTTGGTTGACGT	88	97	0	0	0	0
APH1A	TGTGGTGTACGTTGTGTG	447	562	1	0	0	0
APH1A	GTTGGTGTACCAGTGTGT	298	360	1	0	0	0
APH1A	GTTGGTTGCAGTTGTGTG	316	327	0	0	0	1
APH1A	CATGGTTGCACAGTGTGT	1302	1364	4	0	0	0
APH1A	ACTGGTTGCACAGTGTCA	208	184	0	0	0	0
APH1B	GTTGGTCACAACTGACGT	244	299	0	0	0	0
APH1B	CATGGTCACAACTGACCA	88	267	0	0	0	0
APH1B	TGTGGTCACAACTGACAC	18	38	0	0	0	0
APH1B	TGTGACGTACGTGTGTGT	939	446	2	0	0	2
APH1B	GTTGACGTACGTGTGTCA	513	584	0	1	0	0
API5	ACACTGCAGTTGGTACAC	36	39	0	0	0	0
API5	CAACTGCAGTTGGTACTG	133	219	0	0	0	0
API5	GTACTGCAGTTGGTTGGT	87	127	0	0	0	0
API5	CAACTGCAGTTGGTTGCA	35	55	0	0	0	0
API5	TGACTGCAGTTGGTTGAC	352	118	0	0	0	0
APLN	TGTGGTACGTTGACACCA	138	154	0	0	0	0
APLN	CATGGTACGTTGACACAC	157	124	0	0	0	0
APLN	GTTGGTACGTTGACACTG	644	881	0	0	0	0
APLN	TGTGGTACGTTGACTGGT	160	252	0	0	0	0
APLN	GTTGGTACGTTGACTGCA	178	516	0	0	0	0
APLP1	CAACTGACCACAACCATG	226	341	0	1	1	0
APLP1	TGACTGACCACAACCAGT	165	158	0	5	0	0
APLP1	GTACTGACCACAACCACA	192	164	0	0	0	0
APLP1	ACACTGACCACAACCAAC	34	49	0	0	0	0
APLP1	TGTGCAGTCACATGGTCA	297	226	0	0	0	2
APLP2	ACACGTGTCAGTGTACTG	357	179	0	0	0	0
APLP2	CAACGTGTCAGTGTTGGT	379	807	0	0	0	1
APLP2	ACACGTGTCAGTGTTGCA	405	478	0	0	0	0
APLP2	GTACGTGTCAGTGTTGAC	35	215	0	0	0	0
APLP2	TGACGTGTCAGTGTTGTG	90	152	0	0	0	1
APLP2	GTTGCACAGTCAACGTCA	377	194	0	0	0	0
APOA1	CAACCAGTACGTACACTG	637	767	8	4	0	7
APOA1	GTACCAGTACGTACTGGT	221	261	0	0	0	0
APOA1	CAACCAGTACGTACTGCA	101	297	0	0	0	4
APOA1	TGACCAGTACGTACTGAC	19	13	0	0	0	0
APOA1	ACACCAGTACGTACTGTG	143	133	1	0	0	0
APOA1	ACTGCACACACAACGTTG	480	406	0	0	0	0
APOA2	ACACTGCATGACGTACTG	469	534	0	0	0	0
APOA2	CAACTGCATGACGTTGGT	310	451	1	1	0	0
APOA2	ACACTGCATGACGTTGCA	152	130	0	0	0	0
APOA2	GTACTGCATGACGTTGAC	15	15	0	0	0	0
APOA2	TGACTGCATGACGTTGTG	678	881	0	1	0	1
APOA2	ACTGCACAGTCAACGTAC	134	99	0	1	0	0
APOA4	CATGGTGTGTCAGTGTGT	870	1145	2	1	1	1
APOA4	GTTGGTGTGTGTTGTGTG	149	112	1	0	0	0
APOA4	ACTGGTGTGTCAGTGTCA	406	449	0	1	0	0
APOA4	GTTGGTGTGTCAGTGTAC	87	90	0	0	0	0
APOA4	GTTGCAGTTGTGTGTGAC	57	59	0	0	0	0
APOA5	TGTGGTGTGTCACATGGT	219	397	0	0	0	1
APOA5	GTTGGTGTGTCACATGCA	215	183	0	0	0	1
APOA5	ACTGGTGTGTCACATGAC	32	264	0	0	0	0
APOA5	CATGGTGTGTCACATGTG	202	85	0	0	0	0
APOA5	TGTGGTGTGTCAACGTGT	574	860	0	1	0	69
APOB	GTCATGACTGTGACCAAC	26	28	0	0	0	0
APOB	TGCATGACTGTGACCATG	12	128	0	0	0	0
APOB	ACCATGACTGTGACACGT	41	56	0	0	0	0
APOB	TGCATGACTGTGACACCA	327	121	1	0	0	0

APOB	CACATGACTGTGACACAC	65	39	0	0	0	0
APOBEC1	CAACACACGTCAGTGTCA	469	602	3	2	2	1
APOBEC1	TGACACACGTGTTGTGTG	326	404	0	1	0	0
APOBEC1	GTACACACGTCAGTGTGT	18	35	0	0	0	0
APOBEC1	TGACACACGTCAGTGTAC	2	13	0	0	0	0
APOBEC1	CATGACGTTGTGTGTGCA	259	206	0	0	0	0
APOBEC2	TGACACACCATGGTCATG	163	189	1	0	0	0
APOBEC2	ACACACACCATGGTACGT	142	130	0	0	0	0
APOBEC2	TGACACACCATGGTACCA	160	170	0	1	0	2
APOBEC2	CAACACACCATGGTACAC	89	77	0	0	0	0
APOBEC2	TGTGCAGTTGCAACGTAC	84	98	0	0	0	0
APOBEC3A	ACACACCATGCATGGTGT	485	445	1782	4	2	0
APOBEC3A	TGACACCATGCATGGTCA	534	565	0	1	0	0
APOBEC3A	CAACACCATGCATGGTAC	60	180	0	0	0	0
APOBEC3A	GTACACCATGCATGGTTG	227	240	0	0	0	0
APOBEC3A	GTTGGTTGCACAGTGTAC	81	60	0	0	0	0
APOBEC3A	GTTGCAACACTGACCACA	229	131	0	1	0	0
APOBEC3B	TGTGGTCAACTGACTGTG	674	708	0	0	1	552
APOBEC3B	ACTGGTCAACTGACTGCA	350	94	1	0	0	0
APOBEC3B	GTTGGTCAACTGACTGAC	0	7	0	0	0	0
APOBEC3B	CATGGTCAACTGTGGTGT	305	339	0	0	0	0
APOBEC3B	ACTGGTCAACTGTGGTCA	277	303	0	0	0	0
APOBEC3B	GTTGCAACACTGACGTTG	80	72	0	0	0	0
APOBEC3C	CAACACACACACACGTTG	384	193	0	0	1	0
APOBEC3C	TGACACACACACACGTGT	995	774	1	0	0	1
APOBEC3C	GTACACACACACACGTCA	453	368	0	0	0	0
APOBEC3C	ACACACACACACACGTAC	76	61	0	0	0	0
APOBEC3C	ACTGCAGTCAACACCAGT	295	503	0	0	0	1
APOBEC3C	ACTGCAACACTGACCAAC	46	73	0	0	0	0
APOBEC3F	TGTGACGTACTGCAACCA	82	103	0	0	0	0
APOBEC3F	CATGACGTACTGCAACAC	22	17	1	0	0	0
APOBEC3F	GTTGACGTACTGCAACTG	380	268	0	0	0	2
APOBEC3F	TGTGACGTACTGCATGGT	224	276	0	0	0	127
APOBEC3F	GTTGACGTACTGCATGCA	73	275	0	0	0	0
APOBEC3G	ACACACACACACACACCA	281	309	0	0	0	0
APOBEC3G	GTACACACACTGGTCACA	98	108	0	0	0	0
APOBEC3G	ACACACACACTGGTCAAC	68	86	1	0	0	0
APOBEC3G	CAACACACACTGGTCATG	643	149	0	1	0	0
APOBEC3G	GTACACACACTGGTACGT	163	181	0	0	0	0
APOBEC4	GTTGGTCAACACTGGTGT	1117	745	3	0	1	1
APOBEC4	CATGGTCAACACTGGTCA	367	395	9	11	1	15
APOBEC4	ACTGGTCAACACACTGTG	217	324	1	0	0	0
APOBEC4	TGTGGTCAACACTGGTAC	75	198	0	1	0	0
APOBEC4	ACTGGTCAACACTGGTTG	370	310	1	0	0	0
APOC1	ACTGGTTGCAGTGTTGCA	93	90	8	0	0	0
APOC1	GTTGGTTGCAGTGTTGAC	70	76	0	0	0	0
APOC1	TGTGGTTGCAGTGTTGTG	331	408	0	0	0	0
APOC1	CATGGTTGCAGTCAGTGT	489	520	0	0	0	5
APOC1	ACTGGTTGCAGTCAGTCA	243	298	1	0	0	6
APOC2	TGTGGTGTGTCATGACAC	125	67	0	0	0	0
APOC2	ACTGGTGTGTCATGACTG	336	244	0	0	0	0
APOC2	CATGGTGTGTCATGTGGT	1549	410	0	1	0	5
APOC2	ACTGGTGTGTCATGTGCA	289	376	0	0	0	1
APOC2	TGTGCATGCACAGTACCA	175	199	0	0	0	0
APOC2	CATGCATGCACAGTACAC	187	131	0	0	0	0
APOC3	GTTGGTGTGTGTGTCACA	72	91	0	0	1	0
APOC3	ACTGGTGTGTGTGTCAAC	10	17	5	0	0	0
APOC3	CATGGTGTGTGTGTCATG	271	238	0	0	0	0
APOC3	GTTGGTGTGTGTGTACGT	556	241	0	0	0	1
APOC3	CATGGTGTGTGTGTACCA	133	120	0	0	0	0
APOC3	CATGCACACACAACCAGT	209	226	0	0	0	1
APOD	GTACCAGTACTGGTCATG	131	129	0	0	0	0
APOD	TGACCAGTACTGGTACGT	90	265	0	0	0	0
APOD	GTACCAGTACTGGTACCA	84	109	1	0	0	0
APOD	ACACCAGTACTGGTACAC	20	38	0	0	0	0
APOD	CAACCAGTACTGGTACTG	368	380	1	0	0	0
APOE	CATGCAGTACCAGTGTTG	1144	487	3	0	1	0
APOE	ACCATGTGCATGCACAGT	87	187	0	0	0	0
APOE	TGCATGTGCATGCACACA	42	54	0	0	0	0
APOE	CACATGTGCATGCACAAC	80	62	0	0	0	0
APOE	CATGACGTCAACCAACTG	152	59	0	0	0	0
APOL2	CAACTGTGTGCACACAAC	73	99	0	0	0	0
APOL2	GTACTGTGTGCACACATG	157	202	0	0	0	0
APOL2	TGACTGTGTGCACAACGT	1110	632	0	185	0	1
APOL2	GTACTGTGTGCACAACCA	212	278	0	0	0	0

APOL2	ACACTGTGTGCACAACAC	76	14	0	0	0	0
APOL2	CATGCAACACACCATGGT	226	268	0	0	0	0
APP	CAACGTGTCAGTCAGTGT	1234	1746	4	0	0	1
APP	ACACGTGTCAGTCAGTCA	497	654	1	0	0	1
APP	GTACGTGTCAGTCAGTAC	37	108	0	0	0	0
APP	CATGCAGTACCAGTTGAC	256	62	0	0	0	0
APP	ACTGACGTGTGTACACTG	128	105	0	0	0	0
APPBP2	GTACTGACTGCAGTTGGT	889	1028	2	1	1	27
APPBP2	CAACTGACTGCAGTTGCA	369	406	1	0	0	0
APPBP2	TGACTGACTGCAGTTGAC	149	16	0	0	0	0
APPBP2	ACACTGACTGCAGTTGTG	116	105	0	0	0	0
APPBP2	GTACTGACTGCACAGTGT	808	592	7	3	0	55
APPBP2	TGTGCAACGTTGACACTG	119	173	0	0	0	0
APPL1	TGACGTGTGTCACACAAC	58	17	0	0	0	0
APPL1	ACACGTGTGTCACACATG	99	100	0	0	0	0
APPL1	CAACGTGTGTCACAACGT	118	149	0	0	0	0
APPL1	CATGCAGTACCACAGTAC	21	38	0	0	0	0
APPL1	ACTGACACACGTGTGTCA	109	119	0	0	0	0
APPL2	CATGGTACCAGTTGACCA	410	533	9	14	1	16
APPL2	TGTGGTACCAGTTGACAC	70	52	0	1	0	0
APPL2	ACTGGTACCAGTTGACTG	197	171	0	0	0	0
APPL2	CATGGTACCAGTTGTGGT	303	356	0	0	0	0
APPL2	ACTGGTACCAGTTGTGCA	134	154	0	0	0	0
APRT	GTACCACAACCATGTGCA	206	239	0	0	0	1
APRT	ACACCACAACCATGTGAC	30	74	0	0	0	0
APRT	CAACCACAACCATGTGTG	708	404	0	0	0	1
APRT	CAACCACAACACGTGTGT	593	606	0	1	0	0
APRT	ACACCACAACACGTGTCA	181	238	0	0	0	0
AQP1	CAACCATGTGACTGCACA	114	333	1	1	0	0
AQP1	TGACCATGTGACTGCAAC	61	26	0	0	0	0
AQP1	TGTGCAGTTGGTGTCAAC	16	17	0	0	0	0
AQP1	ACTGCAGTTGGTGTCATG	247	272	0	0	0	0
AQP1	CATGCATGACACTGACTG	384	365	0	0	0	0
AQP7	GTTGCACAGTCAACCAGT	286	538	0	0	1	0
AQP7	GTACTGCACAGTTGTGGT	1071	176	1	0	0	1
AQP7	CAACTGCACAGTTGTGCA	70	60	0	0	0	0
AQP7	TGACTGCACAGTTGTGAC	35	23	0	0	0	0
AQP7	ACACTGCACAGTTGTGTG	377	409	0	0	0	1
AQP7	CATGCACAGTCAACGTTG	901	950	2	3	0	0
AQR	CAACTGTGGTTGTGGTCA	641	698	1	0	0	3
AQR	TGACTGTGGTTGTGGTAC	193	75	0	1	0	0
AQR	ACACTGTGGTTGTGGTTG	537	319	1	0	0	0
AQR	CAACTGTGGTTGTGCAGT	67	99	1	0	0	0
AQR	ACACTGTGGTTGTGCACA	4	4	0	0	0	0
AR	CACATGACTGTGCAACTG	224	388	35	0	1	0
AR	TGCATGACTGTGCAACGT	205	107	0	1	0	0
AR	GTCATGACTGTGCAACCA	123	165	0	0	0	0
AR	ACCATGACTGTGCAACAC	54	49	0	1	0	0
AR	CATGACGTTGACTGTGTG	556	1195	0	1	0	7
ARAF	ACCATGCAACCACAGTGT	1243	839	0	1	1	1
ARAF	TGCATGCAACCACAGTCA	569	472	0	0	0	5
ARAF	CACATGCAACCACAGTAC	87	30	0	0	0	0
ARAF	GTCATGCAACCACAGTTG	825	812	2	0	0	2
ARAF	TGCATGCAACCACACAGT	119	162	1	0	0	0
ARAF	CATGCACAGTCAACCACA	63	117	0	0	0	0
ARD1A	TGACCACATGCATGACTG	152	156	0	0	0	5
ARD1A	ACACCACATGCATGTGGT	74	80	0	0	0	0
ARD1A	TGACCACATGCATGTGCA	429	247	1	0	0	0
ARD1A	CAACCACATGCATGTGAC	136	90	0	1	0	0
ARD1A	GTACCACATGCATGTGTG	92	116	0	0	0	0
AREG	GTTGGTCAGTGTGTGTCA	507	1040	0	0	1	11
AREG	TGTGCAACACACCATGTG	523	806	2	0	1	4
AREG	GTTGGTGTTGTGTGTGTG	26	43	0	0	0	0
AREG	TGTGGTCAGTGTGTGTGT	866	1072	0	1	0	17
AREG	ACTGGTCAGTGTGTGTAC	82	117	2	0	0	0
AREG	GTTGCACAGTGTACACTG	367	530	1	0	0	0
ARF1	GTACCATGCACATGCAAC	21	18	0	0	0	0
ARF1	TGACCATGCACATGCATG	181	132	0	0	0	1
ARF1	ACACCATGCACATGACGT	603	542	0	1	0	0
ARF1	CATGCAGTCACAGTTGCA	607	484	1	0	0	0
ARF1	TGTGCAGTCACAGTTGAC	86	55	0	0	0	0
ARF3	GTACACCACAGTGTTGGT	158	176	1	1	0	0
ARF3	CAACACCACAGTGTTGCA	192	162	0	0	0	0
ARF3	TGACACCACAGTGTTGAC	11	22	0	0	0	0
ARF3	ACACACCACAGTGTTGTG	314	327	0	0	0	0

ARF3	GTACACCACAGTCAGTGT	1265	1095	3	0	0	0
ARF3	TGTGCACAGTCAACCAAC	69	71	0	1	0	0
ARF4	GTTGCAGTCAGTCACATG	807	1412	1	2	1	1
ARF4	TGACACCACACAGTGTAC	116	114	0	0	0	0
ARF4	ACACACCACACAGTGTTG	212	159	1	0	0	0
ARF4	CAACACCACACAGTCAGT	427	704	0	0	0	0
ARF4	ACACACCACACAGTCACA	356	414	0	1	0	0
ARF4	CATGCACAACACACCAGT	222	129	1	1	0	0
ARF5	CAACACCACACAGTTGTG	141	100	0	0	0	0
ARF5	TGACACCACACACAGTGT	405	659	2	1	0	0
ARF5	GTACACCACACACAGTCA	556	556	0	0	0	0
ARF5	ACACACCACACACAGTAC	118	52	0	0	0	1
ARF5	CAACACCACACACAGTTG	746	1102	2	0	0	4
ARF5	ACTGCACAACACACCACA	219	266	0	0	0	0
ARF6	GTACACCACACACACAGT	201	236	0	0	0	0
ARF6	CAACACCACACACACACA	124	260	0	0	0	0
ARF6	TGACACCACACACACAAC	36	16	0	0	0	0
ARF6	ACACACCACACACACATG	429	438	0	1	0	8
ARF6	CAACACCACACACAACGT	1013	783	1	2	0	0
ARF6	GTTGCACAACACACCAAC	86	113	0	0	0	0
ARFGAP1	ACACACCAGTACTGGTGT	249	222	0	0	0	0
ARFGAP1	TGACACCAGTACTGGTCA	320	214	0	0	0	0
ARFGAP1	CAACACCAGTACTGGTAC	94	94	0	0	0	0
ARFGAP1	GTACACCAGTACTGGTTG	227	664	0	0	0	13
ARFGAP1	TGACACCAGTACTGCAGT	765	677	1	0	0	9
ARFGAP1	CATGCATGGTCAGTACGT	142	145	0	0	0	1
ARFGAP3	ACTGCATGGTCAGTACCA	79	94	0	0	1	2
ARFGAP3	GTACACCAGTACTGTGAC	40	76	0	1	0	0
ARFGAP3	TGACACCAGTACTGTGTG	61	67	0	0	0	0
ARFGAP3	ACACACCAGTTGGTGTGT	70	98	0	0	0	0
ARFGAP3	TGACACCAGTTGGTGTCA	125	71	1	0	0	7
ARFGAP3	CAACACCAGTTGGTGTAC	45	81	0	1	0	0
ARFGEF2	TGACACCAGTACACACTG	471	427	3	0	0	0
ARFGEF2	ACACACCAGTACACTGGT	526	463	0	0	0	0
ARFGEF2	TGACACCAGTACACTGCA	149	151	0	0	0	0
ARFGEF2	CAACACCAGTACACTGAC	145	129	0	1	0	0
ARFGEF2	GTACACCAGTACACTGTG	269	196	1	0	0	1
ARFIP2	ACACTGCATGCATGACAC	9	7	0	0	0	0
ARFIP2	CAACTGCATGCATGACTG	593	518	1	0	0	5
ARFIP2	GTACTGCATGCATGTGGT	130	83	0	0	0	0
ARFIP2	CAACTGCATGCATGTGCA	127	102	0	0	0	0
ARFIP2	TGACTGCATGCATGTGAC	33	118	0	0	0	0
ARG1	CAACACACGTACACTGTG	762	519	1	1	1	0
ARG1	GTACACACGTACACTGCA	264	109	0	0	0	0
ARG1	ACACACACGTACACTGAC	30	44	0	0	0	0
ARG1	TGACACACGTACTGGTGT	252	180	1	0	0	0
ARG1	GTACACACGTACTGGTCA	171	219	0	0	0	0
ARG1	CATGCACATGCATGCAGT	56	99	0	1	0	0
ARG2	ACACACACCAGTCAACAC	180	155	0	0	0	0
ARG2	CAACACACCAGTCAACTG	153	108	0	0	0	0
ARG2	GTACACACCAGTCATGGT	371	352	1	0	0	0
ARG2	CAACACACCAGTCATGCA	133	92	0	0	0	0
ARG2	TGACACACCAGTCATGAC	27	51	0	0	0	0
ARHGAP1	TGACACCAGTACACCAAC	47	34	0	0	0	0
ARHGAP1	ACACACCAGTACACCATG	162	395	0	0	0	0
ARHGAP1	CAACACCAGTACACACGT	52	34	0	1	0	0
ARHGAP1	ACACACCAGTACACACCA	289	253	1401	0	0	1
ARHGAP1	GTACACCAGTACACACAC	21	26	0	0	0	0
ARHGAP1	TGTGCAACCACACACAGT	105	136	0	1	0	0
ARHGAP4	ACACACCAGTACCACACA	85	67	0	0	0	0
ARHGAP4	GTACACCAGTACCACAAC	19	11	0	0	0	0
ARHGAP4	TGACACCAGTACCACATG	152	185	0	0	0	0
ARHGAP4	ACACACCAGTACCAACGT	252	196	2	0	0	0
ARHGAP4	TGACACCAGTACCAACCA	728	485	1	0	0	1
ARHGAP5	ACACGTGTGTACCAACAC	17	137	0	0	0	0
ARHGAP5	CAACGTGTGTACCAACTG	704	429	0	1	0	40
ARHGAP5	GTACGTGTGTACCATGGT	373	790	1	0	0	0
ARHGAP5	CAACGTGTGTACCATGCA	333	279	0	1	0	0
ARHGAP5	ACACGTCAACCATGCAGT	304	90	0	0	0	0
ARHGAP6	GTACACCAGTACCAGTGT	1499	2054	1	2	1	0
ARHGAP6	CAACACCAGTACGTTGCA	95	133	0	0	0	0
ARHGAP6	TGACACCAGTACGTTGAC	81	144	0	0	0	0
ARHGAP6	ACACACCAGTACGTTGTG	333	367	0	0	0	0
ARHGAP6	GTTGACCATGACTGTGGT	474	941	0	0	0	1
ARHGDIA	CAACGTGTTGGTCACATG	134	250	0	1	1	0

ARHGDIA	CAACGTGTTGGTCAACCA	481	645	1	0	1	0
ARHGDIA	GTACGTGTTGGTCAACGT	448	353	1	1	0	0
ARHGDIA	TGACGTGTTGGTCAACAC	27	17	0	0	0	0
ARHGDIA	TGTGCAGTACCATGCATG	99	113	0	1	0	0
ARHGDIB	GTACACCAGTTGCAGTAC	40	31	0	0	0	0
ARHGDIB	TGACACCAGTTGCAGTTG	932	643	6	0	0	0
ARHGDIB	ACACACCAGTTGCACAGT	151	217	0	0	0	0
ARHGDIB	TGACACCAGTTGCACACA	89	84	0	0	0	0
ARHGDIB	CAACACCAGTTGCACAAC	5	9	0	0	0	0
ARHGEF1	GTACCACAGTTGCATGAC	124	82	0	0	0	0
ARHGEF1	TGACCACAGTTGCATGTG	118	108	0	0	0	0
ARHGEF1	CAACCACAGTTGACGTGT	591	558	0	0	0	0
ARHGEF1	ACACCACAGTTGACGTCA	221	311	0	0	0	1
ARHGEF1	GTACCACAGTTGACGTAC	71	31	0	0	0	0
ARHGEF11	GTACACCAGTTGCAACCA	429	429	0	58	7	1
ARHGEF11	GTACACCAGTTGCACATG	426	203	0	0	0	0
ARHGEF11	TGACACCAGTTGCAACGT	190	186	0	0	0	1
ARHGEF11	ACACACCAGTTGCAACAC	40	122	0	0	0	0
ARHGEF11	GTTGCAGTCATGACACGT	12	91	0	0	0	0
ARHGEF12	CAACACCAGTTGCAACTG	369	282	0	0	0	0
ARHGEF12	GTACACCAGTTGCATGGT	1272	279	0	1	0	8
ARHGEF12	CAACACCAGTTGCATGCA	311	96	0	1	0	0
ARHGEF12	TGACACCAGTTGCATGAC	56	51	0	0	0	0
ARHGEF12	ACACACCAGTTGCATGTG	50	46	0	0	0	0
ARHGEF12	CATGCACAACTGCAGTAC	114	66	0	0	0	0
ARHGEF2	ACCATGACACACCACAGT	501	438	0	0	1	0
ARHGEF2	GTCATGACACACCACATG	132	644	0	0	1	0
ARHGEF2	TGCATGACACACCACACA	216	334	0	0	0	0
ARHGEF2	CACATGACACACCACAAC	22	14	0	0	0	0
ARHGEF2	TGTGCAGTACGTACTGTG	182	252	0	0	0	0
ARHGEF2	TGTGCAACGTCACACACA	44	40	0	0	0	0
ARHGEF4	CAACACCAGTTGTGACGT	118	61	0	0	0	0
ARHGEF4	ACACACCAGTTGTGACCA	141	224	5	0	0	0
ARHGEF4	GTACACCAGTTGTGACAC	66	50	0	0	0	2
ARHGEF4	TGACACCAGTTGTGACTG	276	515	1	0	0	0
ARHGEF4	ACACACCAGTTGTGTGGT	148	243	0	0	0	0
ARHGEF4	CATGCAACGTCACACAAC	16	15	0	0	0	0
ARHGEF5	ACACCAGTTGACACTGTG	184	643	1	0	0	1
ARHGEF5	GTACCAGTTGACTGGTGT	1090	1009	0	0	0	1
ARHGEF5	CAACCAGTTGACTGGTCA	224	221	1	2	0	0
ARHGEF5	TGACCAGTTGACTGGTAC	21	12	0	0	0	0
ARHGEF5	ACACCAGTTGACTGGTTG	145	196	0	0	0	0
ARHGEF6	GTACGTGTACGTTGTGGT	528	371	0	0	2	0
ARHGEF6	TGACGTGTACGTTGACGT	242	38	0	0	0	0
ARHGEF6	GTACGTGTACGTTGACCA	42	69	0	0	0	0
ARHGEF6	ACACGTGTACGTTGACAC	35	124	0	0	0	0
ARHGEF6	CAACGTGTACGTTGACTG	67	85	0	0	0	0
ARHGEF7	GTACACCACAGTGTGTAC	20	42	1	0	0	0
ARHGEF7	TGACACCACAGTGTGTTG	395	694	0	0	0	0
ARHGEF7	ACACACCACAGTGTCAGT	384	362	0	0	0	0
ARHGEF7	TGACACCACAGTGTCACA	58	73	0	0	0	0
ARHGEF7	CAACACCACAGTGTCAAC	63	29	0	0	0	0
ARHGEF7	TGTGCAACACACGTCAAC	31	33	0	0	0	0
ARL4D	CAACACCACACAACGTAC	415	279	1	1	0	53
ARL4D	GTACACCACACAACGTTG	871	362	3	1	0	30
ARL4D	TGACACCACACAACCAGT	198	257	0	0	0	0
ARL4D	GTACACCACACAACCACA	324	309	0	1	0	0
ARL4D	ACTGGTTGTGCAACTGAC	37	56	0	0	0	0
ARMETL1	GTTGGTCAACTGGTGTGT	671	631	2	0	2	2
ARMETL1	CATGGTCAACTGGTGTCA	1221	1043	0	0	1	1
ARMETL1	ACTGGTCAACACTGTGAC	45	70	0	0	0	1
ARMETL1	CATGGTCAACACTGTGTG	1007	739	1	0	0	0
ARMETL1	TGTGGTCAACTGGTGTAC	22	36	0	0	0	0
ARNT	TGCATGTGGTGTACCACA	47	95	0	0	0	0
ARNT	CACATGTGGTGTACCAAC	40	41	0	0	0	0
ARNT	GTCATGTGGTGTACCATG	225	109	0	0	0	1
ARNT	TGCATGTGGTGTACACGT	20	39	0	0	0	0
ARNT	GTCATGTGGTGTACACCA	312	381	2	0	0	1
ARNT	TGTGCATGGTTGGTACAC	5	4	0	0	0	0
ARNT2	TGACGTACTGCAACCAGT	368	401	1	1	1	0
ARNT2	GTACGTACTGCAACGTTG	202	208	0	0	0	0
ARNT2	GTACGTACTGCAACCACA	57	30	0	0	0	0
ARNT2	ACACGTACTGCAACCAAC	53	96	0	0	0	0
ARNT2	CAACGTACTGCAACCATG	142	280	0	1	0	1
ARNTL	TGACGTACTGCATGACGT	162	396	398535	429	27	56

ARNTL	CAACGTACTGCATGCAAC	126	99	2	48	1	1
ARNTL	TGACGTACTGCATGCACA	151	139	175	2044	0	0
ARNTL	GTACGTACTGCATGCATG	49	169	0	0	0	0
ARNTL	GTACGTACTGCATGACCA	152	259	0	0	0	0
ARPC1A	GTCATGTGACCATGGTTG	286	286	1	0	1	1
ARPC1A	CACATGTGACCATGGTAC	57	63	0	0	0	0
ARPC1A	TGCATGTGACCATGCAGT	152	170	0	0	0	1
ARPC1A	GTCATGTGACCATGCACA	81	104	0	0	0	0
ARPC1A	ACCATGTGACCATGCAAC	22	18	0	0	0	0
ARPC1A	CATGCAACACTGACACCA	591	462	0	1	0	9
ARPC1B	TGACCAACCATGTGTGTG	609	647	0	0	1	1
ARPC1B	GTACCAACCATGTGTGAC	6	7	0	0	0	0
ARPC1B	CAACCAACACGTGTGTGT	866	972	3	0	0	11
ARPC1B	ACACCAACACGTGTGTCA	409	445	0	0	0	1
ARPC1B	TGTGCAGTACTGACCATG	113	96	0	0	0	0
ARPC1B	TGTGCAACACTGACACAC	108	104	0	0	0	0
ARPC2	TGACCAACACGTGTCACA	166	159	1	3	1	4
ARPC2	GTACCAACACGTGTGTAC	51	82	0	0	0	0
ARPC2	TGACCAACACGTGTGTTG	367	342	0	1	0	0
ARPC2	ACACCAACACGTGTCAGT	90	64	1	0	0	0
ARPC2	ACTGCAGTACTGACACGT	6	18	0	0	0	0
ARPC2	CATGCAACTGGTGTTGCA	36	29	0	0	0	0
ARPC3	CAACCAACACGTGTCAAC	20	10	0	0	0	0
ARPC3	GTACCAACACGTGTCATG	125	134	0	0	0	0
ARPC3	TGACCAACACGTGTACGT	129	175	0	0	0	0
ARPC3	GTACCAACACGTGTACCA	223	172	0	0	0	2
ARPC3	ACACCAACACGTGTACAC	149	87	0	0	0	0
ARPC3	ACTGCAACTGGTACGTAC	34	32	0	0	0	0
ARPC4	CAACCAACACGTGTTGCA	538	529	2	0	1	1
ARPC4	CAACCAACACGTGTACTG	94	105	0	0	0	0
ARPC4	GTACCAACACGTGTTGGT	167	109	0	0	0	10
ARPC4	TGACCAACACGTGTTGAC	76	93	0	0	0	0
ARPC4	ACACCAACACGTGTTGTG	101	205	0	0	0	2
ARPC5	ACACCAACACGTCAGTTG	1298	1785	12	0	1	1
ARPC5	GTACCAACACGTCAGTGT	359	190	1	2	0	0
ARPC5	CAACCAACACGTCAGTCA	225	179	0	0	0	0
ARPC5	TGACCAACACGTCAGTAC	154	130	0	0	0	0
ARPC5	CAACCAACACGTCACAGT	175	181	2	0	0	0
ARPC5	TGTGCAACTGGTGTTGAC	21	36	0	0	0	0
ARPC5L	GTTGGTGTCACAGTACGT	394	495	1	0	0	0
ARPC5L	TGTGGTACGTCAGTCAAC	4	6	0	0	0	0
ARPC5L	ACTGGTACGTCAGTCATG	259	285	0	0	0	0
ARPC5L	CATGGTACGTCAGTACGT	765	426	2	9	0	1
ARPC5L	ACTGGTACGTCAGTACCA	50	119	0	0	0	0
ARPC5L	TGTGCACATGTGTGACTG	144	187	0	0	0	0
ARRB1	ACCATGTGACGTACGTTG	120	131	0	0	0	0
ARRB1	CACATGTGACGTACCAGT	278	319	0	0	0	1
ARRB1	ACCATGTGACGTACCACA	117	43	0	0	0	0
ARRB1	GTCATGTGACGTACCAAC	6	9	0	0	0	0
ARRB1	TGCATGTGACGTACCATG	41	57	0	0	0	0
ARRB2	CATGGTTGGTCAGTACAC	49	33	0	0	1	0
ARRB2	GTTGGTTGGTCAGTCAAC	21	20	0	0	0	0
ARRB2	TGTGGTTGGTCAGTCATG	627	295	0	0	0	0
ARRB2	ACTGGTTGGTCAGTACGT	63	40	1	0	0	0
ARRB2	TGTGGTTGGTCAGTACCA	100	61	0	0	0	0
ARSA	TGACACCATGACTGTGGT	1150	626	0	0	1	18
ARSA	ACACACCATGACTGTGAC	131	132	0	0	1	0
ARSA	GTACACCATGACTGACTG	467	424	1	0	0	0
ARSA	GTACACCATGACTGTGCA	264	650	1	0	0	0
ARSA	CAACACCATGACTGTGTG	344	282	1	1	0	0
ARSA	CATGCACAACACTGGTTG	2009	2039	2	0	0	2
ARSB	ACACCAGTACACTGTGTG	1007	499	0	0	0	0
ARSB	CAACCAGTACTGGTGTGT	1195	727	0	2	0	0
ARSB	ACACCAGTACTGGTGTCA	184	541	1	0	0	0
ARSB	TGTGCAGTCAACGTCAAC	11	16	0	0	0	0
ARSB	ACTGCAGTCAACGTCATG	213	188	1	0	0	0
ARSD	ACACACACACGTGTTGAC	62	49	0	0	0	0
ARSD	CAACACACACGTGTTGTG	133	181	0	1	0	0
ARSD	TGACACACACGTCAGTGT	525	448	1	0	0	0
ARSD	GTACACACACGTCAGTCA	557	419	0	0	0	0
ARSD	TGTGACCACACATGTGTG	168	261	0	0	0	0
ARSE	GTACACACCACATGACGT	149	65	0	0	1	0
ARSE	CAACACACCACATGCATG	147	155	0	0	0	0
ARSE	CAACACACCACATGACCA	532	310	0	0	0	2
ARSE	TGACACACCACATGACAC	30	56	0	0	0	0

ARSE	CATGCAGTCATGACGTAC	45	76	65	0	0	0
ARSE	ACTGCACAGTCAACCATG	358	413	0	0	0	1
ASAH1	CAACCAGTACTGCAGTCA	91	33	0	0	0	0
ASAH1	TGACCAGTACTGCAGTAC	44	55	0	0	0	0
ASAH1	ACACCAGTACTGCAGTTG	425	403	0	0	0	0
ASAH1	CAACCAGTACTGCACAGT	138	171	0	0	0	0
ASAH1	ACACCAGTACTGCACACA	176	237	0	0	0	0
ASAH3L	GTACACACGTCATGCATG	128	257	0	0	0	0
ASAH3L	TGACACACGTCATGACGT	127	142	0	0	0	0
ASAH3L	GTACACACGTCATGACCA	172	184	1	0	0	4
ASAH3L	ACACACACGTCATGACAC	72	138	0	0	0	0
ASAH3L	GTTGACCACACAGTTGGT	224	286	0	1	0	0
ASCC3	TGTGACCAACGTGTCATG	83	156	0	0	0	0
ASCC3	ACTGACCAACGTGTACGT	336	712	0	1	0	0
ASCC3	TGTGACCAACGTGTACCA	395	266	1	0	0	5
ASCC3	CATGACCAACGTGTACAC	24	41	0	0	0	0
ASCC3	GTTGACCAACGTGTACTG	649	579	3	0	0	0
ASCC3L1	ACACACACACCACACAAC	10	27	0	0	0	0
ASCC3L1	CAACACACACCACACATG	88	103	0	1	0	0
ASCC3L1	GTACACACACCACAACGT	27	22	0	0	0	3
ASCC3L1	CAACACACACCACAACCA	95	19	2	0	0	16
ASCC3L1	TGACACACACCACAACAC	39	26	0	0	0	0
ASGR2	TGACTGCATGGTGTGTTG	223	588	0	0	0	0
ASGR2	ACACTGCATGGTGTCAGT	679	470	1	9	0	0
ASGR2	TGACTGCATGGTGTCACA	180	202	0	0	0	0
ASGR2	CAACTGCATGGTGTCAAC	10	104	0	0	0	0
ASGR2	ACTGCAGTTGTGGTGTCA	254	45	0	0	0	0
ASH1L	GTACGTACCATGGTGTTG	443	961	0	0	0	1
ASH1L	TGACGTACCATGGTCAGT	212	181	0	0	0	0
ASH1L	GTACGTACCATGGTCACA	227	275	0	0	0	0
ASH1L	ACACGTACCATGGTCAAC	32	25	0	0	0	0
ASH1L	TGACTGTGACACACACTG	357	374	1	0	0	0
ASIP	ACTGGTGTGTCATGGTGT	1162	473	2	1	1	2
ASIP	TGTGGTGTGTCATGGTCA	546	608	0	0	0	0
ASIP	CATGGTGTGTCATGGTAC	93	359	0	0	0	0
ASIP	GTTGGTGTGTCATGGTTG	475	873	0	1	0	2
ASIP	TGTGGTGTGTCATGCAGT	330	319	30	0	0	1
ASIP	CATGCAACCATGTGTGAC	38	66	0	0	0	0
ASL	GTACTGTGCATGTGGTAC	12	49	0	0	0	0
ASL	TGACTGTGCATGTGGTTG	547	1000	0	0	0	0
ASL	ACACTGTGCATGTGCAGT	168	197	0	0	0	0
ASL	TGACTGTGCATGTGCACA	62	240	0	0	0	0
ASL	CAACTGTGCATGTGCAAC	45	50	0	0	0	0
ASMT	TGACCAACGTTGACCAGT	475	436	1	1	0	0
ASMT	GTACCAACGTTGACCACA	71	62	1	0	0	0
ASMT	ACACCAACGTTGACCAAC	41	42	0	0	0	0
ASMT	CAACCAACGTTGACCATG	52	86	0	0	0	0
ASMT	CATGGTACTGGTACTGTG	323	351	1	0	0	0
ASMT	TGTGCACACAACTGACGT	186	187	1	1	0	1
ASNA1	GTACCATGACACACGTAC	111	107	0	0	0	3
ASNA1	TGACCATGACACACGTTG	181	356	1	0	0	0
ASNA1	ACACCATGACACACCAGT	44	57	0	0	0	0
ASNA1	TGACCATGACACACCACA	167	193	1	0	0	1
ASNA1	CAACCATGACACACCAAC	44	55	0	0	0	0
ASNS	ACACACGTACACGTCATG	19	59	0	0	0	0
ASNS	CAACACGTACACGTACGT	285	283	0	0	0	0
ASNS	ACACACGTACACGTACCA	201	156	1	0	0	0
ASNS	GTACACGTACACGTACAC	32	28	0	0	0	0
ASNS	TGACACGTACACGTACTG	85	215	0	0	0	0
ASNS	TGTGCAACCAGTACTGTG	264	651	0	1	0	0
ASPA	GTACTGTGACACGTGTAC	14	23	0	0	0	0
ASPA	TGACTGTGACACGTGTTG	89	58	0	0	0	0
ASPA	ACACTGTGACACGTCAGT	195	167	0	0	0	0
ASPA	TGACTGTGACACGTCACA	65	77	0	0	0	0
ASPA	CAACTGTGACACGTCAAC	43	139	0	0	0	0
ASPH	TGTGACGTCACAGTACCA	389	398	0	0	1	1
ASPH	TGACACTGGTGTTGCAAC	16	16	0	0	0	0
ASPH	ACACACTGGTGTTGCATG	51	35	0	1	0	0
ASPH	ACTGACGTCACAGTACGT	193	184	0	0	0	0
ASPH	CATGACGTCACAGTACAC	136	66	0	0	0	0
ASRGL1	ACACACCAGTGTTGCAGT	455	248	0	0	1	3
ASRGL1	TGACACCAGTGTTGCACA	19	23	0	0	0	0
ASRGL1	CAACACCAGTGTTGCAAC	2	13	0	0	0	0
ASRGL1	GTACACCAGTGTTGCATG	276	284	0	1	0	0
ASRGL1	TGACACCAGTGTTGACGT	103	284	0	0	0	0

ASRGL1	TGTGCAACTGGTCACATG	111	155	0	0	0	0
ASS1	ACACACGTCAACACTGGT	196	310	0	0	0	0
ASS1	TGACACGTCAACACTGCA	63	115	0	0	0	0
ASS1	CAACACGTCAACACTGAC	187	85	0	0	0	0
ASS1	ACACTGTGCACACATGAC	86	74	0	0	0	0
ASS1	ACTGACACGTACGTTGGT	374	560	0	0	0	3
ATF1	TGACGTCAACTGACTGAC	63	63	1	0	0	0
ATF1	ACACGTCAACTGACTGTG	422	308	0	0	0	0
ATF1	GTACGTCAACTGTGGTGT	469	415	2	0	0	0
ATF1	CAACGTCAACTGTGGTCA	461	471	0	1	0	27
ATF1	TGACGTCAACTGTGGTAC	115	126	0	0	0	1
ATF2	GTACGTCATGGTTGCATG	407	105	1	0	0	0
ATF2	TGACGTCATGGTTGACGT	140	134	0	0	0	0
ATF2	GTACGTCATGGTTGACCA	345	191	0	0	0	0
ATF2	ACACGTCATGGTTGACAC	21	25	0	0	0	0
ATF2	CAACGTCATGGTTGACTG	402	220	1	0	0	0
ATF2	GTTGCAACACTGGTGTGT	725	442	1	1	0	1
ATF3	ACACGTCAACTGTGGTTG	1045	1842	2	2	0	0
ATF3	CAACGTCAACTGTGCAGT	127	148	0	0	0	0
ATF3	ACACGTCAACTGTGCACA	87	132	0	0	0	1
ATF3	GTACGTCAACTGTGCAAC	30	29	0	0	0	0
ATF3	TGACGTCAACTGTGCATG	35	34	0	0	0	0
ATF4	ACACGTCAACTGTGACGT	26	69	0	0	0	0
ATF4	TGACGTCAACTGTGACCA	606	487	1	0	0	3
ATF4	CAACGTCAACTGTGACAC	99	110	0	1	0	0
ATF4	GTACGTCAACTGTGACTG	679	849	0	0	0	0
ATF4	ACTGCAGTACACCAACGT	73	61	0	0	0	0
ATF5	CAACGTACACACCACATG	251	230	0	1	1	0
ATF5	TGACGTACACACCACAGT	163	87	0	0	0	12
ATF5	GTACGTACACACCACACA	57	59	0	0	0	0
ATF5	ACACGTACACACCACAAC	114	25	0	0	0	0
ATF5	ACTGACACACCATGACAC	68	67	0	0	0	0
ATF7IP	ACACGTTGCATGGTACCA	374	234	2	1	0	0
ATF7IP	GTACGTTGCATGGTACAC	33	22	1	0	0	0
ATF7IP	TGACGTTGCATGGTACTG	205	146	0	0	0	0
ATF7IP	ACACGTTGCATGGTTGGT	131	120	0	0	0	0
ATF7IP	TGACGTTGCATGGTTGCA	144	144	1	0	0	0
ATG10	CATGGTCAACACCATGGT	1057	586	5	0	0	6
ATG10	ACTGGTCAACACCATGCA	464	120	0	0	0	1
ATG10	GTTGGTCAACACCATGAC	21	24	0	0	0	0
ATG10	TGTGGTCAACACCATGTG	130	144	0	0	0	0
ATG10	CATGGTCAACACACGTGT	525	465	1	1	0	0
ATG12	TGACGTGTACGTTGGTTG	133	207	0	0	0	0
ATG12	ACACGTGTACGTTGCAGT	36	18	0	0	0	0
ATG12	TGACGTGTACGTTGCACA	148	179	1	0	0	2
ATG12	CAACGTGTACGTTGCAAC	27	21	0	0	0	0
ATG12	GTACGTGTACGTTGCATG	83	79	0	0	0	1
ATG16L1	ACTGGTCAACGTGTGTGT	469	611	1	2	0	0
ATG16L1	TGTGGTCAACGTGTGTCA	1147	695	428	1	0	3
ATG16L1	CATGGTCAACGTGTGTAC	27	37	0	0	0	0
ATG16L1	GTTGGTCAACGTGTGTTG	850	651	1	0	0	0
ATG16L1	GTTGACACCATGTGACGT	1000	397	1	10	0	2
ATG16L2	CATGGTCAACGTCAACTG	1008	874	0	1	0	0
ATG16L2	GTTGGTCAACGTCATGGT	210	375	1	1	0	0
ATG16L2	CATGGTCAACGTCATGCA	377	522	0	0	0	0
ATG16L2	TGTGGTCAACGTCATGAC	91	105	0	1	0	0
ATG16L2	ACTGGTCAACGTCATGTG	176	195	1	0	0	0
ATG2A	GTTGACGTTGACGTCAGT	150	133	1	1	1	0
ATG2A	TGTGGTTGACCAACCACA	27	29	0	0	0	0
ATG2A	CATGGTTGACCAACCAAC	42	83	0	0	0	0
ATG2A	GTTGGTTGACCAACCATG	100	79	0	0	0	0
ATG2A	TGTGGTTGACCAACACGT	25	34	0	0	0	0
ATG2B	GTTGGTCAACACGTGTCA	559	414	0	1	1	4
ATG2B	TGTGGTCAACACGTGTGT	966	460	1	0	0	0
ATG2B	ACTGGTCAACACGTGTAC	71	105	0	0	0	4
ATG2B	CATGGTCAACACGTGTTG	515	577	2	0	0	0
ATG2B	GTTGACACCACATGTGTG	181	451	0	0	0	0
ATG3	CATGGTACGTGTTGGTGT	158	170	1	0	0	0
ATG3	ACTGGTACGTGTTGGTCA	97	84	3	1	0	0
ATG3	GTTGGTACGTGTTGGTAC	49	47	0	0	0	0
ATG3	TGTGGTACGTGTTGGTTG	236	222	0	0	0	0
ATG3	ACTGGTACGTGTTGCAGT	109	72	0	0	0	1
ATG4A	CAACTGTGGTCAACCATG	180	448	1	13	1	0
ATG4A	GTACTGTGGTCAACGTTG	728	568	2	1	0	1
ATG4A	TGACTGTGGTCAACCAGT	344	434	0	0	0	0

ATG4A	GTACTGTGGTCAACCACA	52	58	0	0	0	0
ATG4A	ACACTGTGGTCAACCAAC	64	31	0	0	0	0
ATG4A	ACTGCATGGTTGGTTGCA	32	180	0	0	0	0
ATG4B	GTACTGTGGTCAACACGT	25	28	0	0	0	0
ATG4B	CAACTGTGGTCAACACCA	192	195	1	0	0	8
ATG4B	TGACTGTGGTCAACACAC	75	154	0	0	0	0
ATG4B	ACACTGTGGTCAACACTG	338	660	1	0	0	1
ATG4B	CAACTGTGGTCAACTGGT	308	308	0	0	0	0
ATG4C	TGACCAGTTGTGGTACCA	403	350	0	1	0	1
ATG4C	ACACTGTGGTCAACTGCA	104	71	0	0	0	0
ATG4C	GTACTGTGGTCAACTGAC	63	78	0	0	0	0
ATG4C	TGACTGTGGTCAACTGTG	712	178	1	0	0	0
ATG4C	CAACTGTGGTCATGGTGT	859	613	3	1	0	1
ATG4C	CATGCATGGTACTGACCA	61	26	0	0	0	0
ATG4D	TGACTGTGGTCATGCACA	98	147	0	1	1	0
ATG4D	ACACTGTGGTCATGGTCA	432	434	1	2	0	1
ATG4D	GTACTGTGGTCATGGTAC	226	272	0	2	0	0
ATG4D	TGACTGTGGTCATGGTTG	690	693	1	3	0	1
ATG4D	ACACTGTGGTCATGCAGT	117	67	0	0	0	0
ATG4D	GTTGCATGGTTGGTTGAC	176	50	0	0	0	0
ATG5	GTTGCACACAACTGACCA	320	324	10	0	2	1
ATG5	GTTGGTACGTTGTGTGTG	528	681	1	1	1	0
ATG5	GTTGGTGTCACAGTGTTG	1463	1817	4	0	0	0
ATG5	TGTGGTGTCACAGTCAGT	556	350	1	0	0	2
ATG5	CATGGTACGTTGTGTGAC	23	36	0	0	0	0
ATG5	CATGGTACCAGTGTGTGT	1163	715	3	2	0	0
ATG7	GTACGTGTACACGTTGGT	121	172	2	0	0	0
ATG7	CAACGTGTACACGTTGCA	104	216	0	0	0	0
ATG7	TGACGTGTACACGTTGAC	16	2	0	0	0	8
ATG7	ACACGTGTACACGTTGTG	169	267	0	0	0	0
ATG7	GTACGTGTACACCAGTGT	545	733	2	2	0	11
ATG7	GTTGCACAACGTTGGTTG	593	379	3	0	0	0
ATG9A	ACTGGTTGTGTGTGGTGT	415	319	0	0	0	0
ATG9A	TGTGGTTGTGTGTGGTCA	64	95	0	0	0	0
ATG9A	CATGGTTGTGTGTGGTAC	8	13	0	0	0	0
ATG9A	GTTGGTTGTGTGTGGTTG	510	1144	1	0	0	3
ATG9A	TGTGGTTGTGTGTGCAGT	430	373	1	1	0	0
ATG9B	TGTGGTTGCACAGTGTTG	828	1061	3	3	3	0
ATG9B	ACTGGTTGCACAGTCAGT	560	235	2	0	1	0
ATG9B	TGTGGTTGCACAGTCACA	95	123	0	0	0	0
ATG9B	CATGGTTGCACAGTCAAC	27	59	0	0	0	0
ATG9B	GTTGGTTGCACAGTCATG	180	238	0	0	0	0
ATIC	ACACCACAACCACATGCA	298	309	0	3	1	0
ATIC	TGACCACAACCACAACAC	36	31	0	0	0	0
ATIC	ACACCACAACCACAACTG	694	592	1	0	0	1
ATIC	CAACCACAACCACATGGT	198	192	0	0	0	1
ATIC	GTACCACAACCACATGAC	22	5	0	0	0	0
ATM	ACACCATGGTCAGTTGCA	319	205	0	0	0	0
ATM	GTACCATGGTCAGTTGAC	35	32	0	0	0	0
ATM	TGACCATGGTCAGTTGTG	275	365	0	0	0	0
ATM	GTACCATGCAACACGTCA	113	63	0	0	0	0
ATM	CATGGTACTGCATGGTAC	37	111	0	0	0	0
ATN1	CATGGTGTCATGACCACA	66	85	0	0	1	1
ATN1	TGTGGTGTCAGTACGTTG	269	244	0	0	0	0
ATN1	TGTGGTGTCATGACCAAC	81	144	0	0	0	4
ATN1	ACTGGTGTCATGACCATG	54	4	0	0	0	0
ATN1	CATGGTGTCATGACACGT	54	34	21	0	0	0
ATOH1	GTTGGTTGTGCACACATG	686	230	0	1	2	21
ATOH1	GTACGTCATGGTGTGTGT	813	1055	1	4	0	0
ATOH1	CAACGTCATGGTGTGTCA	446	368	2	0	0	1
ATOH1	TGACGTCATGGTGTGTAC	53	83	0	0	0	4
ATOH1	ACACGTCATGGTGTGTTG	214	241	0	1	0	0
ATOH1	ACTGCACACATGTGGTGT	394	239	1	1	0	0
ATP12A	ACACCATGACTGCAGTAC	196	90	0	0	0	1
ATP12A	CAACCATGACTGCAGTTG	1761	1872	3	2	0	0
ATP12A	GTACCATGACTGCACAGT	360	203	1	0	0	0
ATP12A	CAACCATGACTGCACACA	72	69	0	0	0	0
ATP12A	TGTGACCAACCACATGTG	238	258	0	1	0	0
ATP13A2	TGACACACGTGTGTGTTG	362	710	1	0	0	1
ATP13A2	ACACACACGTGTGTCAGT	202	110	1	0	0	0
ATP13A2	TGACACACGTGTGTCACA	71	75	0	0	0	0
ATP13A2	CAACACACGTGTGTCAAC	155	57	0	0	0	0
ATP13A2	GTACACACGTGTGTCATG	61	101	0	0	0	0
ATP13A2	TGTGCACATGTGACACCA	250	359	1	0	0	1
ATP1A1	CAACCATGACTGACGTAC	31	56	0	0	0	0

ATP1A1	GTACCATGACTGACGTTG	1788	1322	20	0	0	0
ATP1A1	TGACCATGACTGACCAGT	218	142	0	0	0	0
ATP1A1	GTACCATGACTGACCACA	123	59	0	0	0	0
ATP1A1	ACACCATGACTGACCAAC	34	66	0	0	0	0
ATP1A2	ACACCATGACTGACACTG	394	370	2	0	0	0
ATP1A2	CAACCATGACTGACTGGT	360	391	2	0	0	0
ATP1A2	ACACCATGACTGACTGCA	224	573	1	0	0	0
ATP1A2	GTACCATGACTGACTGAC	123	194	0	0	0	1
ATP1A2	CATGGTGTCACAGTTGGT	464	338	0	0	0	0
ATP1A3	GTACTGACTGTGCATGTG	429	507	0	1	0	1
ATP1A3	ACACTGACTGTGACGTGT	364	381	1	0	0	0
ATP1A3	TGACTGACTGTGACGTCA	104	101	0	0	0	0
ATP1A3	CAACTGACTGTGACGTAC	42	63	0	0	0	0
ATP1A3	GTACTGACTGTGACGTTG	521	906	0	3	0	0
ATP1A4	TGACCATGACTGTGTGAC	65	82	0	0	0	0
ATP1A4	ACACCATGACTGTGTGTG	263	275	5	0	0	6
ATP1A4	TGTGACGTCAACACCATG	312	159	0	0	0	0
ATP1A4	ACTGACGTCAACACACGT	23	29	1	0	0	0
ATP1A4	TGTGACGTCAACACACCA	912	218	0	0	0	2
ATP1B1	TGACCATGTGGTGTCACA	39	41	0	0	0	0
ATP1B1	CAACCATGTGGTGTCAAC	11	16	0	0	0	0
ATP1B1	GTACCATGTGGTGTCATG	334	132	0	0	0	0
ATP1B1	TGACCATGTGGTGTACGT	42	71	0	0	0	0
ATP1B1	GTACCATGTGGTGTACCA	297	132	1	0	0	0
ATP1B2	TGTGGTCATGTGACGTTG	434	680	2	0	1	1
ATP1B2	CATGGTGTACGTTGTGGT	321	330	1	0	0	0
ATP1B2	ACTGGTCATGTGACCAGT	93	103	0	1	0	0
ATP1B2	TGTGGTCATGTGACCACA	9	15	0	0	0	0
ATP1B2	TGTGCAGTCATGGTCATG	18	71	0	0	0	0
ATP1B3	CAACCATGTGGTCACAGT	130	130	0	0	0	0
ATP1B3	ACACCATGTGGTCACACA	66	83	0	0	0	0
ATP1B3	GTACCATGTGGTCACAAC	35	53	0	0	0	0
ATP1B3	TGACCATGTGGTCACATG	71	55	0	0	0	0
ATP1B3	GTTGACACGTACCATGAC	290	101	0	0	0	0
ATP2A1	TGACCATGGTGTCACAGT	31	75	0	0	0	0
ATP2A1	GTACCATGGTGTCACACA	227	269	0	33	0	0
ATP2A1	ACACCATGGTGTCACAAC	20	8	0	0	0	0
ATP2A1	CAACCATGGTGTCACATG	197	235	1	0	0	0
ATP2A1	GTACCATGGTGTCAACGT	295	262	0	1	0	0
ATP2A2	TGACCATGGTGTACACGT	8	19	0	0	0	0
ATP2A2	GTACCATGGTGTACACCA	26	59	0	0	0	0
ATP2A2	ACACCATGGTGTACACAC	32	53	0	0	0	0
ATP2A2	CAACCATGGTGTACACTG	179	167	0	0	0	0
ATP2A2	GTACCATGGTGTACTGGT	18	48	0	0	0	0
ATP2A2	CATGCATGGTGTCATGAC	49	53	0	0	0	0
ATP2A3	TGACCATGGTGTTGCATG	228	207	9	9854	4	1
ATP2A3	ACACCATGGTGTTGCACA	118	135	0	0	0	0
ATP2A3	GTACCATGGTGTTGCAAC	1	3	0	0	0	0
ATP2A3	ACACCATGGTGTTGACGT	96	92	0	0	0	0
ATP2A3	GTTGCATGGTCAGTCAGT	106	145	0	0	0	0
ATP2A3	CATGCATGGTCAGTCACA	57	66	0	0	0	0
ATP2B1	ACACCATGACACTGCACA	47	34	0	0	0	0
ATP2B1	GTACCATGACACTGCAAC	127	114	0	0	0	6
ATP2B1	TGACCATGACACTGCATG	110	289	0	0	0	0
ATP2B1	ACACCATGACACTGACGT	349	270	0	0	0	0
ATP2B1	TGACCATGACACTGACCA	616	496	1	0	0	0
ATP2B2	ACACCATGACTGGTGTTG	179	297	1	0	0	0
ATP2B2	CAACCATGACTGGTCAGT	133	189	0	0	0	0
ATP2B2	ACACCATGACTGGTCACA	120	114	0	0	0	0
ATP2B2	GTACCATGACTGGTCAAC	40	34	0	0	0	0
ATP2B2	ACTGACGTCACATGTGTG	37	45	0	0	0	0
ATP2B3	ACACCATGACTGGTTGAC	95	173	0	0	0	0
ATP2B3	CAACCATGACTGGTTGTG	127	154	0	0	0	0
ATP2B3	TGACCATGACTGCAGTGT	618	251	3	0	0	1
ATP2B3	GTACCATGACTGCAGTCA	314	525	0	1	0	0
ATP2B3	TGTGACGTCAACGTGTCA	272	327	377	0	0	0
ATP4A	TGACACACCAGTCACACA	132	208	14	0	2	3
ATP4A	TGTGACGTACCACAGTTG	1287	1746	2	1	1	1
ATP4A	TGACACACCAGTCAGTTG	122	101	2	0	0	0
ATP4A	ACACACACCAGTCACAGT	363	109	0	0	0	0
ATP4A	TGTGCAGTTGCAGTGTCA	111	84	1	0	0	1
ATP4B	ACTGGTACACCACATGTG	335	496	2	0	1	0
ATP4B	GTTGGTACACCACATGGT	490	615	1	0	0	75
ATP4B	CATGGTACACCACATGCA	356	504	0	0	0	0
ATP4B	TGTGGTACACCACATGAC	76	61	0	0	0	1

ATP4B	GTTGGTACACCAACGTGT	114	383	2	0	0	0
ATP5A1	CAACCATGTGGTCATGTG	1495	1102	1	0	1	0
ATP5A1	GTACCATGTGGTCAACTG	161	146	0	0	0	0
ATP5A1	TGACCATGTGGTCATGGT	212	255	0	0	0	2
ATP5A1	GTACCATGTGGTCATGCA	134	150	0	0	0	0
ATP5A1	ACACCATGTGGTCATGAC	12	9	0	1	0	0
ATP5B	CAACCATGTGGTACCACA	25	82	0	0	0	0
ATP5B	TGACCATGTGGTACCAAC	50	60	0	0	0	0
ATP5B	ACACCATGTGGTACCATG	174	154	0	0	0	1
ATP5B	CAACCATGTGGTACACGT	35	65	0	0	0	0
ATP5B	CAACTGTGCACAACGTTG	531	838	0	1	0	0
ATP5C1	ACACCATGTGGTACACCA	223	187	0	0	0	0
ATP5C1	GTACCATGTGGTACACAC	34	27	0	0	0	0
ATP5C1	TGACCATGTGGTACACTG	143	469	0	0	0	4
ATP5C1	ACACCATGTGGTACTGGT	98	116	1	0	0	1
ATP5C1	TGACCATGTGGTACTGCA	85	107	0	0	0	0
ATP5D	TGACCATGTGGTTGGTCA	124	202	0	0	0	0
ATP5D	CAACCATGTGGTTGGTAC	41	48	0	0	0	0
ATP5D	GTACCATGTGGTTGGTTG	382	386	0	1	0	0
ATP5D	TGACCATGTGGTTGCAGT	102	105	0	0	0	0
ATP5D	ACTGCAGTCATGTGACAC	111	112	0	0	0	0
ATP5F1	TGACCAGTACACTGCACA	42	45	0	0	0	0
ATP5F1	CAACCAGTACACTGCAAC	25	177	1	0	0	0
ATP5F1	GTACCAGTACACTGCATG	213	355	0	1	0	0
ATP5F1	TGACCAGTACACTGACGT	148	132	1	0	0	0
ATP5F1	GTACCAGTACACTGACCA	282	226	1	0	0	0
ATP5G1	GTACCATGTGGTTGCACA	46	48	0	0	0	1
ATP5G1	ACACCATGTGGTTGCAAC	32	41	0	0	0	0
ATP5G1	CAACCATGTGGTTGCATG	180	180	3	4	0	7
ATP5G1	GTACCATGTGGTTGACGT	351	73	0	1	0	0
ATP5G1	CAACCATGTGGTTGACCA	212	144	0	0	0	0
ATP5G2	TGACCATGTGGTTGACAC	39	51	0	0	0	0
ATP5G2	ACACCATGTGGTTGACTG	605	379	0	0	0	0
ATP5G2	CAACCATGTGGTTGTGGT	626	100	1	0	0	0
ATP5G2	ACACCATGTGGTTGTGCA	523	305	0	0	0	0
ATP5G2	GTACCATGTGGTTGTGAC	8	39	0	0	0	5
ATP5G3	TGACCATGTGCAGTACCA	165	193	0	1	0	0
ATP5G3	CAACCATGTGCAGTACAC	19	24	0	0	0	0
ATP5G3	GTACCATGTGCAGTACTG	684	153	2	0	0	0
ATP5G3	TGACCATGTGCAGTTGGT	408	350	1	0	0	0
ATP5G3	GTACCATGTGCAGTTGCA	122	68	0	0	0	0
ATP5H	ACACCAACTGTGACCACA	76	89	1	0	0	0
ATP5H	GTACCAACTGTGACCAAC	8	10	0	0	0	0
ATP5H	TGACCAACTGTGACCATG	157	138	0	0	0	1
ATP5H	ACTGCAGTCAGTACCACA	35	70	0	0	0	0
ATP5H	GTTGCAGTCAGTACCAAC	56	118	0	0	0	0
ATP5I	ACACCATGTGCACAGTAC	72	167	1	0	0	0
ATP5I	CAACCATGTGCACAGTTG	452	329	0	0	0	0
ATP5I	GTACCATGTGCACACAGT	144	109	3	0	0	0
ATP5I	CAACCATGTGCACACACA	151	135	0	0	0	1
ATP5I	TGACCATGTGCACACAAC	15	27	0	0	0	0
ATP5J	ACACCAACTGTGGTGTGT	247	176	0	0	0	1
ATP5J	TGACCAACTGTGGTGTCA	110	132	0	0	0	0
ATP5J	CAACCAACTGTGGTGTAC	135	172	0	0	0	0
ATP5J	GTACCAACTGTGGTGTTG	500	577	0	0	0	1
ATP5J	TGACCAACTGTGGTCAGT	145	149	0	0	0	2
ATP5J2	ACTGACGTACGTACTGTG	85	124	0	0	1	0
ATP5J2	CATGACGTACGTTGGTCA	947	413	0	0	1	0
ATP5J2	ACACCAACTGTGCACAGT	438	486	0	1	0	0
ATP5J2	TGACCAACTGTGCACACA	127	205	1	0	0	0
ATP5J2	GTTGACGTACGTTGGTGT	906	615	0	0	0	0
ATP5L	GTACCAACTGTGACTGCA	163	214	1	0	1	0
ATP5L	ACACCAACTGTGACTGAC	19	17	0	0	0	0
ATP5L	CAACCAACTGTGACTGTG	107	115	0	0	0	0
ATP5L	TGACCAACTGTGTGGTGT	618	521	0	0	0	1
ATP5L	ACTGCAGTGTGTACCATG	8	4	1	0	0	0
ATP5O	GTACCATGGTGTCATGAC	49	85	0	0	0	0
ATP5O	TGACCATGGTGTCATGTG	128	170	0	0	0	0
ATP5O	CAACCATGGTGTACGTGT	722	823	2	0	0	1
ATP5O	ACACCATGGTGTACGTCA	49	98	0	0	0	0
ATP5O	GTACCATGGTGTACGTAC	95	279	0	0	0	0
ATP6AP1	ACACCATGGTGTCAGTGT	349	735	0	0	0	1
ATP6AP1	TGACCATGGTGTCAGTCA	231	268	1	1	0	0
ATP6AP1	CAACCATGGTGTCAGTAC	95	121	0	0	0	0
ATP6AP1	GTACCATGGTGTCAGTTG	537	607	2	0	0	1

ATP6AP1	ACTGCAGTCATGGTACGT	351	183	1	0	0	2
ATP6V0A1	ACACCATGGTGTGTCATG	85	165	0	0	1	0
ATP6V0A1	TGACCATGGTGTGTCAAC	20	21	0	0	0	0
ATP6V0A1	CAACCATGGTGTGTACGT	64	140	0	0	0	0
ATP6V0A1	ACACCATGGTGTGTACCA	100	88	0	0	0	0
ATP6V0A1	GTACCATGGTGTGTACAC	138	31	0	0	0	0
ATP6V0A2	TGACCATGTGGTTGTGTG	445	471	1	0	0	0
ATP6V0A2	GTACCATGTGCAGTGTGT	805	350	1	0	0	0
ATP6V0A2	CAACCATGTGCAGTGTCA	407	365	0	0	0	0
ATP6V0A2	TGACCATGTGCAGTGTAC	156	20	0	0	0	0
ATP6V0A2	ACACCATGTGCAGTGTTG	291	256	0	1	0	0
ATP6V0A4	GTACCATGGTCAGTACGT	245	364	0	0	0	0
ATP6V0A4	CAACCATGGTCAGTACCA	371	614	1	0	0	1
ATP6V0A4	TGACCATGGTCAGTACAC	225	126	0	0	0	0
ATP6V0A4	ACACCATGGTCAGTACTG	321	274	0	0	0	0
ATP6V0A4	CAACCATGGTCAGTTGGT	218	305	0	0	0	0
ATP6V0B	GTACCAACTGTGTGACAC	26	25	0	0	0	1
ATP6V0B	TGACCAACTGTGTGACTG	222	281	0	0	0	0
ATP6V0B	ACACCAACTGTGTGTGGT	1097	1737	2	0	0	1
ATP6V0B	TGACCAACTGTGTGTGCA	109	123	0	0	0	0
ATP6V0B	GTTGGTGTCACAGTTGAC	36	22	1	0	0	0
ATP6V0C	CAACCAGTTGGTTGGTTG	201	182	1	0	0	0
ATP6V0C	GTACCAGTTGGTTGCAGT	59	84	0	0	0	0
ATP6V0C	CAACCAGTTGGTTGCACA	55	127	0	0	0	0
ATP6V0C	TGACCAGTTGGTTGCAAC	21	15	0	0	0	3
ATP6V0C	ACTGCAGTACGTCAGTAC	45	67	1	0	0	0
ATP6V0D1	CAACCATGGTGTACTGCA	74	88	0	0	0	0
ATP6V0D1	TGACCATGGTGTACTGAC	41	38	0	0	0	0
ATP6V0D1	ACACCATGGTGTACTGTG	66	97	1	0	0	10
ATP6V0D1	GTACCATGGTGTTGGTGT	589	589	2	0	0	3
ATP6V0D1	GTTGCAGTACTGTGACAC	19	45	0	0	0	1
ATP6V0D2	TGTGGTACACTGTGGTCA	320	384	0	1	1	0
ATP6V0D2	ACACCATGTGCAGTTGAC	34	41	0	0	0	0
ATP6V0D2	CAACCATGTGCAGTTGTG	696	925	2	0	0	0
ATP6V0D2	TGACCATGTGCACAGTGT	1830	906	0	2	0	1
ATP6V0D2	GTACCATGTGCACAGTCA	434	480	0	1	0	1
ATP6V0D2	ACTGCAACACTGACGTGT	229	258	0	0	0	0
ATP6V0E1	TGACCATGGTGTACGTTG	176	150	0	0	0	0
ATP6V0E1	ACACCATGGTGTACCAGT	340	499	1	0	0	0
ATP6V0E1	TGACCATGGTGTACCACA	53	186	0	0	0	0
ATP6V0E1	CAACCATGGTGTACCAAC	12	4	0	0	0	0
ATP6V0E1	GTACCATGGTGTACCATG	15	48	0	0	0	0
ATP6V1A	CAACCAGTTGGTACGTCA	640	200	4	0	0	0
ATP6V1A	TGACCAGTTGGTACGTAC	28	23	0	0	0	0
ATP6V1A	ACACCAGTTGGTACGTTG	389	237	1	1	0	0
ATP6V1A	CAACCAGTTGGTACCAGT	172	160	0	0	0	1
ATP6V1A	ACACCAGTTGGTACCACA	62	67	0	0	0	0
ATP6V1A	ACTGCAACCACAGTACCA	144	166	0	0	0	0
ATP6V1B1	GTACCAGTTGGTACACTG	260	339	0	0	1	0
ATP6V1B1	TGACCAGTTGGTACTGGT	264	561	0	0	0	0
ATP6V1B1	GTACCAGTTGGTACTGCA	66	72	3	0	0	7
ATP6V1B1	ACACCAGTTGGTACTGAC	179	48	0	0	0	0
ATP6V1B1	ACTGCAGTACTGGTTGCA	126	130	0	0	0	0
ATP6V1B2	GTACCAGTTGGTACCAAC	15	20	0	0	0	0
ATP6V1B2	TGACCAGTTGGTACCATG	248	247	0	0	0	0
ATP6V1B2	ACACCAGTTGGTACACGT	84	317	0	0	0	0
ATP6V1B2	TGACCAGTTGGTACACCA	205	360	0	0	0	0
ATP6V1B2	CAACCAGTTGGTACACAC	23	9	0	0	0	0
ATP6V1C1	ACACCAGTTGGTTGACCA	249	207	4	1	10	5
ATP6V1C1	ACACCAGTTGGTTGCATG	32	22	0	0	0	0
ATP6V1C1	CAACCAGTTGGTTGACGT	53	70	0	0	0	0
ATP6V1C1	GTACCAGTTGGTTGACAC	25	26	0	0	0	0
ATP6V1C1	ACTGGTGTCACAGTTGCA	196	296	0	0	0	0
ATP6V1C2	CAACCATGACTGGTACAC	202	122	0	0	0	0
ATP6V1C2	GTACCATGACTGGTACTG	228	83	0	0	0	0
ATP6V1C2	TGACCATGACTGGTTGGT	169	185	0	0	0	0
ATP6V1C2	GTACCATGACTGGTTGCA	339	115	0	0	0	1
ATP6V1C2	ACTGCACAGTGTCAGTCA	113	177	0	0	0	0
ATP6V1D	GTACCATGACACTGGTGT	1703	1725	10	2	2	15
ATP6V1D	TGACCATGACACACTGAC	377	29	5	1	1	0
ATP6V1D	GTACCATGACACACTGGT	264	493	0	0	0	1
ATP6V1D	CAACCATGACACACTGCA	33	222	0	0	0	0
ATP6V1D	ACACCATGACACACTGTG	312	346	2	0	0	0
ATP6V1D	GTTGCAACCACAGTACAC	66	98	0	0	0	0
ATP6V1E1	TGACCAGTTGGTTGGTGT	1085	818	3	0	1	0

ATP6V1E1	CAACCAGTTGGTACTGTG	291	476	0	0	0	0
ATP6V1E1	GTACCAGTTGGTTGGTCA	226	341	0	0	0	1
ATP6V1E1	ACACCAGTTGGTTGGTAC	31	46	0	0	0	0
ATP6V1E1	TGTGCAGTACGTCACAAC	4	6	0	0	0	0
ATP6V1E2	ACACCATGTGCAGTACGT	975	467	1	0	1	0
ATP6V1E2	CAACCATGTGCAGTCAGT	241	479	1	0	0	1
ATP6V1E2	ACACCATGTGCAGTCACA	92	134	0	0	0	0
ATP6V1E2	GTACCATGTGCAGTCAAC	11	24	0	0	0	0
ATP6V1E2	TGACCATGTGCAGTCATG	480	743	0	1	0	0
ATP6V1F	CAACCATGGTGTTGGTCA	254	237	1	1	0	0
ATP6V1F	TGACCATGGTGTTGGTAC	49	52	1	1	0	0
ATP6V1F	ACACCATGGTGTTGGTTG	312	317	1	1	0	11
ATP6V1F	CAACCATGGTGTTGCAGT	697	242	1	434	0	1
ATP6V1F	GTTGCAACCAACTGTGAC	56	47	0	0	0	0
ATP6V1F	TGTGCAACCAACTGTGTG	79	167	0	0	0	0
ATP6V1G1	GTACCATGGTGTTGACTG	449	670	1	0	2	1
ATP6V1G1	TGACCATGGTGTTGACCA	379	300	0	0	0	0
ATP6V1G1	CAACCATGGTGTTGACAC	9	12	1	0	0	0
ATP6V1G1	TGACCATGGTGTTGTGGT	450	469	1	0	0	0
ATP6V1G1	GTACCATGGTGTTGTGCA	17	26	0	0	0	0
ATP6V1G2	ACACCATGGTGTGTGTAC	87	41	0	0	0	0
ATP6V1G2	CAACCATGGTGTGTGTTG	292	373	0	0	0	0
ATP6V1G2	GTACCATGGTGTGTCAGT	237	300	0	2	0	0
ATP6V1G2	CAACCATGGTGTGTCACA	94	107	0	0	0	0
ATP6V1G2	CATGCAGTTGGTGTGTTG	753	758	0	0	0	7
ATP6V1G2	CATGCAACCAACTGTGGT	368	422	1	0	0	1
ATP6V1G3	CAACCATGACACTGTGTG	334	929	0	1	1	2
ATP6V1G3	ACTGCAACCAACTGTGCA	582	472	1	0	1	0
ATP6V1G3	ACACCATGACACTGTGAC	111	57	0	0	0	0
ATP6V1G3	GTACCATGACTGGTGTGT	1116	532	2	0	0	79
ATP6V1G3	CAACCATGACTGGTGTCA	155	227	0	0	0	0
ATP6V1G3	TGACCATGACTGGTGTAC	239	81	0	0	0	0
ATP6V1H	CAACCATGACACTGGTCA	169	193	0	0	0	0
ATP6V1H	TGACCATGACACTGGTAC	166	17	0	0	0	0
ATP6V1H	ACACCATGACACTGGTTG	439	355	1	0	0	0
ATP6V1H	CAACCATGACACTGCAGT	79	99	0	0	0	0
ATP6V1H	GTTGGTTGTGACGTACTG	239	405	1	0	0	3
ATP8A1	GTACCATGGTGTGTTGTG	477	516	0	1	1	2
ATP8A1	TGACCATGGTGTGTACTG	121	96	0	0	0	0
ATP8A1	ACACCATGGTGTGTTGGT	240	356	2	0	0	0
ATP8A1	TGACCATGGTGTGTTGCA	152	151	27	0	0	0
ATP8A1	CAACCATGGTGTGTTGAC	30	21	0	0	0	0
ATP8A1	GTTGCAACGTTGCAACTG	64	119	0	0	0	0
ATR	TGACGTTGTGACCAGTCA	88	74	0	0	0	0
ATR	CAACGTTGTGACCAGTAC	153	173	0	0	0	0
ATR	ACACCATGGTTGCAACGT	63	102	0	0	0	0
ATR	TGACCATGGTTGCAACCA	234	297	0	0	0	0
ATR	CAACCATGGTTGCAACAC	111	138	0	0	0	0
ATRX	CAACGTCATGGTGTCAGT	105	92	0	1	0	0
ATRX	ACACGTCATGGTGTCACA	39	104	0	0	0	0
ATRX	GTACGTCATGGTGTCAAC	105	19	1	0	0	0
ATRX	TGACGTCATGGTGTCATG	117	125	0	0	0	1
ATRX	CAACTGTGACACACACGT	5	13	0	0	0	0
ATRX	TGTGCAACCAACACTGCA	227	181	0	0	0	0
ATXN1	CACATGACTGTGACGTCA	201	187	0	1	0	0
ATXN1	TGCATGACTGTGACGTAC	15	20	3	0	0	0
ATXN1	ACCATGACTGTGACGTTG	171	298	0	1	0	0
ATXN1	CACATGACTGTGACCAGT	413	199	0	2	0	0
ATXN1	TGTGACGTACGTTGCATG	68	80	0	0	0	1
ATXN2	GTTGGTCAGTACTGCATG	165	181	0	0	0	0
ATXN2	TGTGGTCAGTACTGACGT	447	559	0	0	0	0
ATXN2	GTTGGTCAGTACTGACCA	155	95	0	0	0	0
ATXN2	ACTGGTCAGTACTGACAC	99	57	0	0	0	1
ATXN2	CATGGTCAGTACTGACTG	299	268	1	0	0	0
Aug-98	CAACTGCATGACTGACGT	378	513	0	0	1	0
Aug-98	ACACTGCATGACTGACCA	568	349	0	1	1	0
Aug-98	GTACTGCATGACTGACAC	30	27	0	0	0	0
Aug-98	TGACTGCATGACTGACTG	746	473	2	0	0	2
Aug-98	ACACTGCATGACTGTGGT	863	683	0	0	0	2
AUH	CAACACACTGGTCATGGT	2562	1202	1	17	1	1
AUH	CAACACACTGGTCAACCA	192	228	0	0	0	0
AUH	TGACACACTGGTCAACAC	21	16	0	0	0	0
AUH	ACACACACTGGTCAACTG	285	449	1	0	0	0
AUH	GTTGCAGTCACACAACTG	251	537	0	0	0	0
AUH	ACTGCACAGTCAACACCA	494	490	3	0	0	0

AURKA	GTCATGCAACACCACAAC	3	11	4	15	266	5
AURKA	TGCATGCAACACCACATG	98	499	0	0	0	0
AURKA	ACCATGCAACACCAACGT	428	462	0	1	0	1
AURKA	TGCATGCAACACCAACCA	68	212	1	0	0	0
AURKA	TGTGGTTGGTGTCAGTAC	51	83	0	0	0	0
AURKB	GTCATGCAACTGACGTGT	484	382	0	0	0	0
AURKB	CACATGCAACTGACGTCA	171	181	0	0	0	0
AURKB	TGCATGCAACTGACGTAC	33	41	0	0	0	0
AURKB	ACCATGCAACTGACGTTG	1579	1083	2	2	0	3
AURKB	CATGGTTGACACTGTGGT	222	305	0	0	0	0
AVEN	CATGGTCAACTGTGCAAC	35	32	2	3357	2	2
AVEN	ACTGGTCAACTGTGCAGT	109	230	0	1	1	0
AVEN	TGTGGTCAACTGTGGTTG	488	336	3	1	0	1
AVEN	TGTGGTCAACTGTGCACA	309	415	0	2	0	1
AVEN	GTTGGTCAACTGTGCATG	240	177	0	0	0	0
AVP	CATGGTGTGTGTTGGTTG	841	945	2	0	1	0
AVP	CATGGTGTGTGTTGCACA	14	17	0	0	1	0
AVP	TGTGCAGTTGTGTGTGTG	457	438	1	0	1	0
AVP	GTTGGTGTGTGTTGCAGT	104	122	0	0	0	0
AVP	CATGCAGTGTGTTGCATG	261	125	1	1	0	0
AVPR1A	CAACGTGTTGCAACCAGT	169	246	0	0	0	0
AVPR1A	ACACGTGTTGCAACCACA	186	145	0	0	0	0
AVPR1A	GTACGTGTTGCAACCAAC	10	10	0	0	0	0
AVPR1A	TGACGTGTTGCAACCATG	148	153	0	0	0	0
AVPR1A	CATGCAGTACCAACCAAC	40	59	0	0	0	0
AVPR1B	ACACGTGTTGCAACACGT	13	12	0	0	0	0
AVPR1B	TGACGTGTTGCAACACCA	182	210	0	0	0	0
AVPR1B	GTTGCAGTACCAACCATG	39	41	0	0	0	0
AVPR1B	TGTGCACATGACTGTGTG	603	1252	4	0	0	1
AVPR1B	ACTGCACATGTGGTGTGT	225	342	0	2	0	10
AVPR1B	TGTGCACATGTGGTGTCA	640	561	2	0	0	0
AVPR2	CAACGTGTTGCAACACAC	11	11	0	0	0	0
AVPR2	GTACGTGTTGCAACACTG	217	215	0	0	0	0
AVPR2	TGACGTGTTGCAACTGGT	192	606	0	0	0	0
AVPR2	GTACGTGTTGCAACTGCA	120	215	1	0	0	0
AVPR2	TGTGCAGTACCAACGTTG	476	467	2	0	0	0
AVPR2	ACTGCACAACGTGTGTCA	165	195	0	0	0	0
AXIN1	ACACTGCATGCACACATG	421	579	0	0	1	0
AXIN1	CAACTGCATGCACACACA	203	239	0	0	0	0
AXIN1	TGACTGCATGCACACAAC	28	77	0	0	0	1
AXIN1	CAACTGCATGCACAACGT	1297	1089	0	0	0	2
AXIN1	ACACTGCATGCACAACCA	598	651	2	0	0	6
AXIN1	ACTGCATGGTTGCACAGT	137	168	1	0	0	0
AXIN2	TGACTGTGACCATGCATG	732	348	0	0	1	4
AXIN2	ACACTGTGACCATGCACA	145	140	0	0	0	0
AXIN2	GTACTGTGACCATGCAAC	34	72	0	0	0	3
AXIN2	ACACTGTGACCATGACGT	227	194	0	0	0	0
AXIN2	GTTGGTTGACTGTGTGTG	512	800	0	1	0	0
AZGP1	ACACTGCAACTGCATGGT	763	608	1	1	0	0
AZGP1	TGACTGCAACTGCATGCA	142	113	1	0	0	0
AZGP1	CAACTGCAACTGCATGAC	120	98	0	0	0	0
AZGP1	GTACTGCAACTGCATGTG	304	354	2	0	0	0
AZGP1	ACACTGCAACTGACGTGT	212	195	0	0	0	0
AZI2	TGTGGTGTCATGGTCACA	36	37	0	0	0	0
AZI2	CATGGTGTCATGGTCAAC	36	62	0	0	0	0
AZI2	GTTGGTGTCATGGTCATG	311	240	0	0	0	0
AZI2	TGTGGTGTCATGGTACGT	140	522	0	0	0	0
AZI2	GTTGCACAGTGTCAACCA	38	58	0	0	0	0
AZIN1	TGACTGTGACCACATGTG	1041	245	0	1	1	0
AZIN1	CAACTGTGACCACATGGT	203	795	0	0	0	0
AZIN1	ACACTGTGACCACATGCA	455	559	4	0	0	0
AZIN1	GTACTGTGACCACATGAC	51	42	0	0	0	0
AZIN1	CAACTGTGACCAACGTGT	416	472	0	2	0	0
B2M	CAACACTGTGCAACTGCA	388	788	1	0	1	0
B2M	GTACACTGTGCAACTGGT	632	680	0	0	0	0
B2M	TGACACTGTGCAACTGAC	29	34	0	0	0	0
B2M	ACACACTGTGCAACTGTG	196	216	0	0	0	0
B2M	GTACACTGTGCATGGTGT	344	292	2	0	0	0
B3GALNT1	ACACCAACGTCAGTACAC	23	34	0	0	0	0
B3GALNT1	CAACCAACGTCAGTACTG	976	917	0	2	0	0
B3GALNT1	GTACCAACGTCAGTTGGT	18	43	0	0	0	16
B3GALNT1	CAACCAACGTCAGTTGCA	244	276	1	1	0	0
B3GALNT1	TGACCAACGTCAGTTGAC	26	48	0	0	0	0
B3GALT1	TGACGTACACTGCATGCA	708	621	0	0	2	2
B3GALT1	CAACCAACGTCACATGTG	202	237	1	9	0	0

B3GALT1	TGACCAACGTCAACGTGT	107	114	1	0	0	0
B3GALT1	GTACCAACGTCAACGTCA	515	388	1	1	0	0
B3GALT1	ACACCAACGTCAACGTAC	222	151	0	0	0	0
B3GALT1	ACTGCAACGTCATGGTAC	23	210	0	0	0	0
B3GALT2	CATGCAACGTCATGGTTG	776	816	0	0	1	1
B3GALT2	CAACCAACGTCACAACAC	58	199	0	0	0	0
B3GALT2	GTACCAACGTCACAACTG	889	480	0	0	0	0
B3GALT2	TGACCAACGTCACATGGT	120	214	0	0	0	0
B3GALT2	GTACCAACGTCACATGCA	47	270	0	1	0	0
B3GALT2	ACACCAACGTCACATGAC	40	58	0	0	0	0
B3GALT4	ACACCAACGTCACAGTTG	3289	2252	1	2	2	3
B3GALT4	GTACCAACGTCACAGTGT	511	633	1	0	1	0
B3GALT4	CAACCAACGTCACAGTCA	353	322	0	0	0	0
B3GALT4	TGACCAACGTCACAGTAC	49	59	0	0	0	0
B3GALT4	CAACCAACGTCACACAGT	216	320	0	0	0	0
B3GALT5	GTACCACACATGACTGAC	197	114	0	0	0	0
B3GALT5	TGACCACACATGACTGTG	9	9	0	0	0	0
B3GALT5	CAACCACACATGTGGTGT	438	404	0	1	0	8
B3GALT5	ACACCACACATGTGGTCA	204	198	1	0	0	1
B3GALT5	GTACCACACATGTGGTAC	29	25	0	0	0	0
B3GALT5	TGTGCACAACGTACGTGT	305	227	0	1	0	0
B3GALT6	ACACCAACGTGTGTCAAC	30	32	0	0	0	0
B3GALT6	CAACCAACGTGTGTCATG	75	98	1	0	0	0
B3GALT6	GTACCAACGTGTGTACGT	23	29	0	0	0	0
B3GALT6	CAACCAACGTGTGTACCA	602	487	2	0	0	0
B3GALT6	TGACCAACGTGTGTACAC	6	9	0	0	0	0
B3GAT1	CATGCAACGTACACTGGT	398	447	0	0	1	1
B3GAT1	GTTGGTACTGGTTGCAGT	722	273	1	0	0	1
B3GAT1	CATGGTACTGGTTGCACA	86	155	1	0	0	0
B3GAT1	TGTGGTACTGGTTGCAAC	29	28	0	0	0	0
B3GAT1	TGTGCAACGTACACACAC	44	28	0	0	0	0
B3GAT1	ACTGCAACGTACACACTG	35	70	0	0	0	0
B3GAT2	GTACCAACGTGTACCAAC	3	5	0	0	0	0
B3GAT2	TGACCAACGTGTACCATG	485	146	1	0	0	0
B3GAT2	ACACCAACGTGTACACGT	64	89	0	0	0	0
B3GAT2	TGACCAACGTGTACACCA	117	117	0	0	0	0
B3GAT2	GTTGCAGTACTGACGTGT	268	267	0	0	0	0
B3GAT3	CAACCAACGTGTCAGTGT	533	860	1	1	0	1
B3GAT3	ACACCAACGTGTCAGTCA	450	699	3	1	0	3
B3GAT3	GTACCAACGTGTCAGTAC	12	12	0	0	0	0
B3GAT3	TGACCAACGTGTCAGTTG	382	313	1	0	0	0
B3GAT3	ACACCAACGTGTCACAGT	28	63	0	0	0	0
B3GAT3	TGTGCACATGACACTGCA	229	472	0	0	0	0
B3GNT2	GTACCAACGTACCAGTTG	501	1424	0	1	0	0
B3GNT2	TGACCAACGTACCACAGT	82	121	0	1	0	0
B3GNT2	GTACCAACGTACCACACA	56	103	0	0	0	0
B3GNT2	ACACCAACGTACCACAAC	36	35	0	0	0	0
B3GNT2	CATGCAGTGTCAGTGTCA	104	85	0	1	0	0
B3GNT3	GTACCACACACAACGTCA	254	203	1	0	1	0
B3GNT3	CAACCACACACAACCACA	117	130	0	1	1	0
B3GNT3	ACACCACACACAACGTAC	40	39	0	0	0	0
B3GNT3	CAACCACACACAACGTTG	551	583	0	0	0	2
B3GNT3	GTACCACACACAACCAGT	275	353	0	0	0	1
B3GNT4	TGACCACATGGTTGACCA	395	336	1	0	1	0
B3GNT4	ACACCACATGGTTGACGT	81	92	0	0	0	0
B3GNT4	CAACCACATGGTTGACAC	66	39	1	0	0	0
B3GNT4	GTACCACATGGTTGACTG	77	101	0	0	0	0
B3GNT4	TGACCACATGGTTGTGGT	1048	392	3	1	0	0
B3GNT5	GTACCACAACGTGTGTGT	490	781	2	0	1	11
B3GNT5	GTACCACACATGTGTGGT	126	157	0	0	0	0
B3GNT5	CAACCACACATGTGTGCA	194	165	0	0	0	0
B3GNT5	TGACCACACATGTGTGAC	18	14	0	0	0	1
B3GNT5	ACACCACACATGTGTGTG	271	294	1	0	0	0
B3GNT6	TGACCAACGTACACCACA	317	166	2	0	0	0
B3GNT6	CAACCAACGTACACCAAC	41	32	0	0	0	0
B3GNT6	GTACCAACGTACACCATG	176	321	1	0	0	0
B3GNT6	TGACCAACGTACACACGT	143	42	0	0	0	0
B3GNT6	GTACCAACGTACACACCA	284	255	0	0	0	0
B3GNT6	ACTGCACAACCACAACCA	73	65	0	0	0	0
B3GNT7	ACACCACATGTGCAACGT	394	212	0	1	1	0
B3GNT7	ACACCACATGTGCACACA	193	248	0	0	0	117
B3GNT7	GTACCACATGTGCACAAC	80	38	0	0	0	0
B3GNT7	TGACCACATGTGCACATG	420	365	0	0	0	1
B3GNT7	TGACCACATGTGCAACCA	88	107	0	1	0	0
B3GNT7	TGTGCAACACCAACACTG	342	509	0	0	0	0

B4GALNT1	ACACCAACCAACTGCAGT	532	168	1	1	1	0
B4GALNT1	TGACCAACCAACTGCACA	110	121	0	0	0	0
B4GALNT1	CAACCAACCAACTGCAAC	19	16	0	0	0	2
B4GALNT1	GTACCAACCAACTGCATG	628	343	0	0	0	0
B4GALNT1	CATGCAGTACTGACCAGT	138	142	0	0	0	0
B4GALT1	GTACCACAACGTCAGTCA	1556	995	3	2	1	1
B4GALT1	CAACCACAACGTCAGTTG	1387	1672	8	3	1	16
B4GALT1	TGACCACAACGTCAGTGT	396	445	0	0	0	0
B4GALT1	ACACCACAACGTCAGTAC	27	56	0	0	0	0
B4GALT1	CATGCAGTCACAACCACA	191	150	0	0	0	0
B4GALT1	ACTGCAACGTGTCACAAC	41	16	0	0	0	0
B4GALT2	ACACCACAACGTACCAAC	18	27	0	0	0	0
B4GALT2	CAACCACAACGTACCATG	172	291	0	0	0	0
B4GALT2	GTACCACAACGTACACGT	22	9	0	0	0	0
B4GALT2	CAACCACAACGTACACCA	258	260	1	0	0	5
B4GALT2	TGACCACAACGTACACAC	110	35	0	0	0	0
B4GALT3	ACTGGTTGTGCAGTGTGT	265	205	1	0	1	3
B4GALT3	ACACCACATGGTCATGCA	137	125	0	0	0	0
B4GALT3	GTACCACATGGTCATGAC	37	34	0	0	0	1
B4GALT3	TGACCACATGGTCATGTG	88	216	1	0	0	0
B4GALT3	CAACCACATGGTACGTGT	99	74	1	0	0	0
B4GALT3	CATGCAACGTGTCACATG	104	132	0	1	0	0
B4GALT4	TGACGTACACTGCAACTG	140	143	0	0	0	0
B4GALT4	GTACCAACCAACCACAGT	179	108	0	0	0	0
B4GALT4	CAACCAACCAACCACACA	302	271	0	82	0	1
B4GALT4	TGACCAACCAACCACAAC	20	19	0	0	0	0
B4GALT4	ACACCAACCAACCACATG	295	154	0	0	0	0
B4GALT5	TGACCACATGCACAGTTG	1276	844	1	1	1	0
B4GALT5	ACACGTACACTGCATGGT	1534	785	2	0	0	0
B4GALT5	ACACCACATGCACAGTCA	956	915	1	0	0	3
B4GALT5	GTACCACATGCACAGTAC	87	123	0	0	0	0
B4GALT5	ACACCACATGCACACAGT	31	56	0	0	0	0
B4GALT5	GTTGCAACGTGTCAACGT	815	644	0	0	0	0
B4GALT6	CATGCAACGTGTCAACCA	130	141	0	0	1	0
B4GALT6	TGACCACATGGTACTGAC	55	56	0	0	0	0
B4GALT6	ACACCACATGGTACTGTG	340	233	1754	3	0	0
B4GALT6	GTACCACATGGTTGGTGT	270	330	0	0	0	0
B4GALT6	CAACCACATGGTTGGTCA	186	311	1	1	0	4
B4GALT6	TGACCACATGGTTGGTAC	51	9	0	0	0	0
B4GALT7	TGACCACATGTGTGGTCA	282	519	9143	7	1	5
B4GALT7	TGACCACATGTGACTGCA	91	126	1	1	0	0
B4GALT7	CAACCACATGTGACTGAC	97	132	0	1	0	0
B4GALT7	GTACCACATGTGACTGTG	541	476	2	0	0	0
B4GALT7	ACACCACATGTGTGGTGT	81	63	0	0	0	1
B4GALT7	TGTGCACAACCAACACAC	11	42	0	0	0	1
BAAT	TGACCACATGACACCAGT	267	148	0	0	1	0
BAAT	TGACCACATGACACGTCA	406	309	3	3451	0	1
BAAT	CAACCACATGACACGTAC	220	155	0	0	0	0
BAAT	GTACCACATGACACGTTG	746	535	3	0	0	2
BAAT	GTACCACATGACACCACA	115	260	0	0	0	0
BACE1	CACATGCACACATGCATG	46	44	0	0	0	0
BACE1	GTCATGCACACATGACGT	97	98	0	0	0	24
BACE1	CACATGCACACATGACCA	158	127	1	0	0	0
BACE1	TGCATGCACACATGACAC	16	28	0	0	0	0
BACE1	ACCATGCACACATGACTG	919	502	0	2	0	1
BACE2	ACACACACCAACCAACTG	1111	694	2	0	1	8
BACE2	CAACACACCAACCATGGT	140	245	0	0	0	0
BACE2	ACACACACCAACCATGCA	71	165	0	0	0	0
BACE2	GTACACACCAACCATGAC	46	54	0	0	0	0
BACE2	TGTGCAACCATGCAACGT	360	325	0	1	0	0
BACE2	GTTGCAACCATGCAACCA	88	75	0	0	0	1
BACH1	GTACGTCATGGTGTACTG	485	429	5138	67	23	79
BACH1	ACACGTCATGGTGTACGT	190	129	1	0	0	0
BACH1	TGACGTCATGGTGTACCA	153	148	0	0	0	0
BACH1	CAACGTCATGGTGTACAC	12	6	0	0	0	0
BACH1	TGACGTCATGGTGTTGGT	361	83	1	0	0	0
BAD	TGACCACAGTACCACACA	216	252	0	0	1	0
BAD	ACACCACAGTACCACAGT	87	122	0	1	0	0
BAD	CAACCACAGTACCACAAC	50	51	1	0	0	0
BAD	GTACCACAGTACCACATG	86	133	0	9	0	0
BAD	TGACCACAGTACCAACGT	919	656	0	0	0	0
BAD	ACTGCAACGTCAGTACTG	63	208	0	0	0	1
BAG1	CAACGTGTCATGTGACTG	454	278	0	1	1	0
BAG1	GTACGTGTCATGTGTGGT	795	472	4	0	1	1
BAG1	CAACGTGTCATGTGTGCA	181	238	0	0	0	0

BAG1	CATGCAGTACCACAACCA	324	272	0	0	0	0
BAG1	TGTGACACACGTGTGTTG	95	60	0	0	0	0
BAG3	CATGCAGTCAGTACGTCA	183	278	0	0	1	0
BAG3	GTACGTGTCATGTGCATG	88	87	0	0	0	0
BAG3	TGACGTGTCATGTGACGT	203	234	1	0	0	0
BAG3	GTACGTGTCATGTGACCA	131	355	0	0	0	0
BAG3	ACACGTGTCATGTGACAC	19	52	0	0	0	0
BAG4	GTACCACAGTTGACTGGT	217	226	19	15	12	43583
BAG4	TGACCACAGTTGACACGT	105	126	0	0	0	0
BAG4	GTACCACAGTTGACACCA	423	551	1	0	0	0
BAG4	ACACCACAGTTGACACAC	101	56	0	0	0	1
BAG4	CAACCACAGTTGACACTG	213	235	0	1	0	7
BAG4	TGTGCAACGTGTTGCATG	195	174	1	0	0	0
BAG5	TGACCACAGTTGACGTTG	87	117	0	0	0	0
BAG5	ACACCACAGTTGACCAGT	191	311	0	0	0	0
BAG5	TGACCACAGTTGACCACA	141	78	0	0	0	0
BAG5	CAACCACAGTTGACCAAC	13	130	0	0	0	0
BAG5	GTACCACAGTTGACCATG	92	69	0	0	0	0
BAI1	ACACGTGTTGCAACTGAC	9	10	0	0	0	0
BAI1	CAACGTGTTGCAACTGTG	90	165	0	0	0	0
BAI1	TGACGTGTTGCATGGTGT	508	458	0	0	0	41
BAI1	GTACGTGTTGCATGGTCA	119	218	0	0	0	0
BAI1	TGTGGTGTACGTTGGTCA	337	288	94	0	0	3
BAIAP2	GTACTGCAACCAACTGTG	480	1037	0	1	1	78
BAIAP2	CAACTGCAACCAACTGAC	15	94	0	0	0	1
BAIAP2	ACACTGCAACCATGGTGT	905	478	1	0	0	0
BAIAP2	ACTGCAGTCACATGACTG	166	368	0	0	0	0
BAIAP2	CATGCAGTCACATGTGGT	467	366	1	0	0	7
BAIAP2	TGTGCACATGGTACACAC	14	13	0	0	0	0
BAK1	CAACCACAGTACCAACTG	1234	710	0	0	2	0
BAK1	GTACCACAGTACCAACCA	135	238	1	0	0	0
BAK1	ACACCACAGTACCAACAC	26	31	0	0	0	0
BAK1	GTACCACAGTACCATGGT	318	355	1	1	0	0
BAK1	CAACCACAGTACCATGCA	275	369	0	1	0	0
BAP1	CAACGTGTACGTACACCA	82	100	0	0	0	10
BAP1	TGACGTGTACGTACACAC	10	1	0	0	0	0
BAP1	ACACGTGTACGTACACTG	171	146	0	0	0	0
BAP1	CAACGTGTACGTACTGGT	175	242	0	0	0	0
BAP1	ACACGTGTACGTACTGCA	169	196	0	0	0	0
BAP1	ACTGCAACCAGTACTGCA	84	62	1	0	0	0
BAX	TGACCACAGTACCATGAC	2	11	0	0	0	0
BAX	ACACCACAGTACCATGTG	385	175	2	0	0	0
BAX	GTACCACAGTACACGTGT	373	381	1	1	0	2
BAX	CATGCATGACCACAGTGT	642	365	4	0	0	0
BAX	ACTGCATGACCACAGTCA	84	77	0	0	0	0
BBC3	TGACCACAGTTGTGGTAC	57	44	0	0	0	0
BBC3	ACACCACAGTTGTGGTTG	398	447	0	0	0	0
BBC3	CAACCACAGTTGTGCAGT	188	184	0	1	0	2
BBC3	ACACCACAGTTGTGCACA	161	130	0	1	0	0
BBC3	GTACCACAGTTGTGCAAC	270	73	0	0	0	2
BBC3	ACTGCAACTGCAACTGCA	255	297	0	0	0	8
BBOX1	CAACTGACACACACTGGT	307	1109	0	0	0	0
BBOX1	ACACTGACACACACTGCA	242	254	1	0	0	1
BBOX1	GTACTGACACACACTGAC	28	131	0	0	0	0
BBOX1	TGACTGACACACACTGTG	149	188	0	1	0	0
BBOX1	CAACTGACACACTGGTGT	232	255	0	0	0	0
BBS1	CAACTGTGTGACGTCAGT	216	166	0	0	1	0
BBS1	TGACTGTGTGACGTGTAC	13	28	0	0	0	1
BBS1	ACACTGTGTGACGTGTTG	353	465	2	0	0	0
BBS1	ACACTGTGTGACGTCACA	57	30	1	0	0	0
BBS1	GTACTGTGTGACGTCAAC	56	33	0	0	0	0
BBS1	ACTGCATGGTCATGACGT	288	397	1	1	0	0
BBS2	TGACTGTGTGGTGTTGCA	276	65	0	0	1	0
BBS2	ACACTGTGTGGTGTTGGT	139	137	0	0	0	0
BBS2	CAACTGTGTGGTGTTGAC	39	19	0	0	0	0
BBS2	GTACTGTGTGGTGTTGTG	329	553	3	0	0	1
BBS2	ACACTGTGTGGTCAGTGT	1029	407	0	1	0	0
BBS2	TGTGCAACACACTGGTAC	56	62	1	0	0	0
BBS4	TGACGTGTCAGTCAACGT	639	193	0	0	1	0
BBS4	GTACGTGTCAGTCAACCA	85	56	0	0	0	0
BBS4	ACACGTGTCAGTCAACAC	257	109	0	0	0	0
BBS4	CAACGTGTCAGTCAACTG	188	208	0	0	0	0
BBS4	GTACGTGTCAGTCATGGT	171	203	1	0	0	0
BBS4	GTTGCAACTGACTGTGTG	313	666	0	0	0	0
BCAP29	ACACTGCAACGTGTTGCA	244	201	0	0	0	0

BCAP29	GTACTGCAACGTGTTGAC	23	14	0	0	0	0
BCAP29	TGACTGCAACGTGTTGTG	526	510	0	0	0	1
BCAP29	CAACTGCAACGTCAGTGT	1138	1528	2	0	0	0
BCAP29	ACTGACGTTGGTTGGTAC	83	280	0	0	0	0
BCAP31	TGTGCAGTGTGTGTTGAC	26	54	0	0	0	0
BCAP31	ACTGCAGTGTGTGTTGTG	255	340	2	0	0	2
BCAP31	GTTGCAGTGTGTCAGTGT	352	416	0	0	0	7
BCAP31	CATGCAGTGTGTCAGTCA	436	481	0	0	0	0
BCAP31	TGTGCAGTGTGTCAGTAC	82	75	0	0	0	1
BCAR1	TGTGGTGTACACCAGTTG	572	773	2	0	0	9
BCAR1	ACTGGTGTACACCACAGT	53	94	1	0	0	0
BCAR1	TGTGGTGTACACCACACA	27	89	0	0	0	0
BCAR1	CATGGTGTACACCACAAC	12	9	1	0	0	12
BCAR1	GTTGGTGTACACCACATG	218	163	0	1	0	1
BCAT1	GTCATGTGTGCAGTGTGT	728	866	1	1	1	0
BCAT1	TGTGCAGTACCACAGTCA	157	196	0	0	1	0
BCAT1	CACATGTGTGCAGTGTCA	176	335	0	0	0	0
BCAT1	TGCATGTGTGCAGTGTAC	62	43	0	0	0	0
BCAT1	ACCATGTGTGCAGTGTTG	1249	796	0	0	0	0
BCAT2	ACACCACAACACTGTGCA	452	366	0	0	0	2
BCAT2	GTACCACAACACTGTGAC	97	107	0	0	0	0
BCAT2	TGACCACAACACTGTGTG	105	146	0	0	0	33
BCAT2	TGTGCAGTCAGTCAACGT	1151	836	1	2	0	0
BCAT2	GTTGCAGTCAGTCAACCA	116	98	0	0	0	1
BCHE	CAACACCAGTGTTGTGCA	1110	990	1	1	0	0
BCHE	TGACACCAGTGTTGTGAC	28	58	0	0	0	0
BCHE	ACACACCAGTGTTGTGTG	446	247	0	0	0	0
BCHE	TGACACCAGTCAGTGTGT	790	677	0	1	0	0
BCHE	GTACACCAGTCAGTGTCA	412	313	0	0	0	0
BCHE	GTTGCACACACAACCAAC	46	46	0	0	0	0
BCKDHA	TGACCAGTGTTGCAACGT	930	964	4	0	0	1
BCKDHA	GTACCAGTGTTGCAACCA	41	61	0	0	0	0
BCKDHA	ACACCAGTGTTGCAACAC	162	22	0	0	0	0
BCKDHA	CAACCAGTGTTGCAACTG	538	431	2	0	0	0
BCKDHA	GTACCAGTGTTGCATGGT	90	134	2	0	0	0
BCKDHB	GTACCAGTGTTGGTTGAC	2	8	0	0	0	0
BCKDHB	TGACCAGTGTTGGTTGTG	301	284	1	0	0	1
BCKDHB	CAACCAGTGTTGCAGTGT	504	199	0	0	0	0
BCKDHB	ACACCAGTGTTGCAGTCA	247	258	1	2	0	2
BCKDHB	GTACCAGTGTTGCAGTAC	202	92	0	0	0	0
BCKDK	ACTGGTACTGACACTGTG	244	303	0	0	1	0
BCKDK	ACTGGTACTGACTGGTTG	168	322	0	0	1	0
BCKDK	GTTGGTACTGACTGGTGT	287	696	0	0	0	1
BCKDK	CATGGTACTGACTGGTCA	384	411	1	0	0	1
BCKDK	TGTGGTACTGACTGGTAC	43	76	0	0	0	0
BCKDK	TGTGCACAACGTGTCACA	74	60	0	0	0	0
BCL10	CAACCACAGTTGCAACCA	118	254	0	1	1	0
BCL10	ACACCACAGTTGCAACTG	825	681	1	0	1	0
BCL10	ACACCACAGTTGCATGCA	313	312	0	0	1	0
BCL10	TGACCACAGTTGCAACAC	48	44	0	0	0	0
BCL10	CAACCACAGTTGCATGGT	370	560	3	1	0	7
BCL11A	CAACCACAGTACCAGTGT	946	981	2	0	0	6
BCL11A	ACACCACAGTACCAGTCA	428	455	1	0	0	11
BCL11A	GTACCACAGTACCAGTAC	74	32	0	0	0	0
BCL11A	TGACCACAGTACCAGTTG	1665	2233	1	1	0	153
BCL11A	TGTGGTGTCAGTACACGT	53	183	0	0	0	0
BCL11A	ACTGCAACCAACTGCAAC	15	14	0	0	0	0
BCL11B	CAACCACAGTACACGTCA	516	825	2	0	0	0
BCL11B	TGACCACAGTACACGTAC	57	41	0	0	0	0
BCL11B	ACACCACAGTACACGTTG	846	877	0	2	0	2
BCL11B	CAACCACAGTACACCAGT	123	112	0	0	0	0
BCL11B	ACTGGTTGTGACCAACCA	58	84	0	0	0	0
BCL2	CACATGTGCACAACGTGT	137	130	0	0	0	0
BCL2	ACCATGTGCACAACGTCA	62	56	0	1	0	0
BCL2	GTACCATGCATGCATGGT	56	100	0	0	0	0
BCL2	CAACCATGCATGCATGCA	86	111	0	0	0	0
BCL2	TGACCATGCATGCATGAC	12	22	0	0	0	0
BCL2A1	CAACCACAGTACACACAC	34	46	1	0	0	0
BCL2A1	GTACCACAGTACACACTG	439	159	1	0	0	0
BCL2A1	TGACCACAGTACACTGGT	251	269	0	0	0	0
BCL2A1	GTACCACAGTACACTGCA	81	71	0	0	0	0
BCL2A1	GTTGCAGTGTCACAACAC	59	96	0	0	0	2
BCL2A1	ACTGCAACGTGTTGACGT	225	273	0	1	0	0
BCL2L1	GTCATGTGCACAACGTAC	3	11	0	0	0	0
BCL2L1	ACACCACAGTACACTGAC	11	21	0	0	0	0

BCL2L1	CAACCACAGTACACTGTG	244	175	2	0	0	0
BCL2L1	TGACCACAGTACTGGTGT	1081	964	3	0	0	13
BCL2L1	GTACCACAGTACTGGTCA	742	541	88	1	0	1
BCL2L1	GTTGCAACCAACTGACGT	71	137	0	0	0	1
BCL2L10	ACACCACAGTTGACTGTG	406	446	3	1	1	0
BCL2L10	GTACCACAGTTGTGGTGT	393	344	0	0	1	1
BCL2L10	CATGCAACCAACTGCATG	204	115	0	0	1	0
BCL2L10	CAACCACAGTTGACTGCA	759	519	1	1	0	46
BCL2L10	TGACCACAGTTGACTGAC	73	88	0	0	0	0
BCL2L10	CAACCACAGTTGTGGTCA	103	64	0	0	0	0
BCL2L11	CATGACACCACACAGTGT	226	240	0	0	0	0
BCL2L11	ACTGACACCACACAGTCA	234	215	0	0	0	0
BCL2L11	GTTGACACCACACAGTAC	103	73	14	0	0	0
BCL2L11	TGTGACACCACACAGTTG	1423	1737	7	1	0	1
BCL2L11	ACTGACACCACACACAGT	237	237	2	1	0	0
BCL2L14	ACACCACAGTACACCACA	241	118	1	0	0	0
BCL2L14	GTACCACAGTACACCAAC	80	31	0	0	0	0
BCL2L14	TGACCACAGTACACCATG	175	460	0	0	0	0
BCL2L14	ACACCACAGTACACACGT	35	20	0	0	0	0
BCL2L14	TGACCACAGTACACACCA	607	906	0	0	0	5
BCL2L14	GTTGCAACCATGCACATG	99	111	1	0	0	0
BCL2L2	ACACCACAGTACTGGTAC	37	22	0	0	0	0
BCL2L2	CAACCACAGTACTGGTTG	803	481	17	0	0	0
BCL2L2	GTACCACAGTACTGCAGT	624	413	0	1	0	0
BCL2L2	CAACCACAGTACTGCACA	169	454	0	1	0	1
BCL2L2	TGACCACAGTACTGCAAC	18	31	0	0	0	0
BCL2L2	TGTGCAACGTGTTGACCA	33	143	0	0	0	0
BCL3	ACACGTTGGTACTGACTG	98	116	0	0	0	0
BCL3	CAACGTTGGTACTGTGGT	464	535	1	0	0	0
BCL3	ACACGTTGGTACTGTGCA	142	279	1	1	0	0
BCL3	GTACGTTGGTACTGTGAC	24	10	0	0	0	0
BCL3	TGACGTTGGTACTGTGTG	443	396	2	1	0	1
BCL3	CATGCAACCAACTGACCA	170	189	0	0	0	1
BCL6	TGACGTCATGGTCAGTGT	581	813	0	8	1	0
BCL6	GTACGTCATGGTGTTGCA	139	121	0	1	0	3
BCL6	ACACGTCATGGTGTTGAC	34	54	0	0	0	0
BCL6	CAACGTCATGGTGTTGTG	591	396	0	0	0	0
BCL6	GTACGTCATGGTCAGTCA	681	343	1	1	0	1
BCL6	CATGCAACCATGCACAAC	7	1	0	0	0	0
BCL7A	ACACCACAGTACTGCATG	147	190	0	1	0	0
BCL7A	CAACCACAGTACTGACGT	108	251	0	0	0	0
BCL7A	ACACCACAGTACTGACCA	236	160	0	0	0	0
BCL7A	GTACCACAGTACTGACAC	16	13	2	0	0	0
BCL7A	TGACCACAGTACTGACTG	190	304	0	0	0	0
BCL9	ACCATGTGACACACGTCA	137	323	1	606	0	1
BCL9	GTCATGTGACACACGTAC	22	23	0	0	0	0
BCL9	TGCATGTGACACACGTTG	149	270	0	0	0	0
BCL9	ACCATGTGACACACCAGT	118	350	1	0	0	0
BCL9	TGCATGTGACACACCACA	254	196	1	0	0	0
BCMO1	CAACTGACACACTGCAAC	9	10	0	0	0	0
BCMO1	GTACTGACACACTGCATG	888	436	0	0	0	0
BCMO1	TGACTGACACACTGACGT	212	181	0	0	0	0
BCMO1	GTACTGACACACTGACCA	166	117	0	0	0	0
BCMO1	ACACTGACACACTGACAC	223	104	0	0	0	0
BCR	TGCATGCAACTGACACCA	1003	950	9710	11	3	8
BCR	TGCATGCAACTGACTGGT	1580	1010	3654	6	1	7
BCR	CACATGCAACTGACACAC	27	8	0	0	0	0
BCR	GTCATGCAACTGACACTG	64	90	0	0	0	0
BCR	GTTGGTTGGTGTACACAC	22	13	0	0	0	0
BCR	GTTGCACATGCATGACTG	592	828	0	3	0	0
BDH1	GTACCAGTGTACTGACGT	99	163	0	1	0	0
BDH1	CAACCAGTGTACTGACCA	328	209	1	0	0	0
BDH1	TGACCAGTGTACTGACAC	19	29	0	0	0	0
BDH1	ACACCAGTGTACTGACTG	403	509	1	0	0	0
BDH1	CAACCAGTGTACTGTGGT	229	332	0	0	0	0
BDH2	CAACCAACACCACATGAC	7	5	0	0	0	0
BDH2	GTACCAACACCACATGTG	887	769	2	2	0	9
BDH2	ACACCAACACCAACGTGT	978	1188	0	2	0	0
BDH2	ACACACGTTGTGGTGTAC	38	34	0	0	0	0
BDH2	CAACACGTTGTGGTGTTG	627	346	0	0	0	0
BDKRB1	ACACGTGTTGCATGGTAC	16	22	0	0	0	0
BDKRB1	CAACGTGTTGCATGGTTG	432	367	0	0	0	0
BDKRB1	GTACGTGTTGCATGCAGT	118	100	0	0	0	0
BDKRB1	CAACGTGTTGCATGCACA	85	71	0	1	0	0
BDKRB1	TGACGTGTTGCATGCAAC	29	33	0	1	0	0

BDKRB1	CATGCAACCATGGTACCA	205	261	0	0	0	1
BDKRB2	ACACGTGTTGCATGACCA	363	612	1	1	2	0
BDKRB2	ACACGTGTTGCATGCATG	137	94	0	0	0	7
BDKRB2	CAACGTGTTGCATGACGT	52	82	0	0	0	0
BDKRB2	GTACGTGTTGCATGACAC	67	77	0	0	0	0
BDKRB2	TGACGTGTTGCATGACTG	224	249	0	0	0	0
BDKRB2	CATGCAACGTTGGTTGCA	152	107	0	0	0	0
BDNF	CAACTGGTCATGACACGT	23	24	0	0	0	0
BDNF	ACACTGGTCATGACACCA	376	529	0	1	0	1
BDNF	GTACTGGTCATGACACAC	56	46	0	0	0	0
BDNF	TGACTGGTCATGACACTG	681	568	1	0	0	0
BDNF	ACACTGGTCATGACTGGT	47	114	0	1	0	0
BECN1	GTACCACAGTTGCAGTTG	832	623	1	1	1	0
BECN1	ACACCACAGTTGCAGTGT	1330	975	1	0	0	14
BECN1	TGACCACAGTTGCAGTCA	320	530	1	1	0	1
BECN1	CAACCACAGTTGCAGTAC	105	198	1	0	0	0
BECN1	TGACCACAGTTGCACAGT	56	61	0	0	0	0
BECN1	ACTGCAACCACAACACAC	57	42	0	0	0	0
BET1	CATGGTACACCAGTACCA	138	149	0	0	1	0
BET1	ACACTGACTGCATGACTG	47	111	0	0	0	0
BET1	TGTGGTACACCAGTACAC	9	3	0	0	0	0
BET1	ACTGGTACACCAGTACTG	276	275	0	0	0	0
BET1	CATGGTACACCAGTTGGT	330	247	1	0	0	0
BET1L	TGACCATGACTGACGTCA	185	203	3	1867	2118	0
BET1L	GTACCATGACTGCATGTG	795	1020	2	0	0	0
BET1L	ACACCATGACTGACGTGT	1246	653	0	1	0	0
BET1L	GTTGCAGTACTGACACTG	196	173	2	0	0	0
BET1L	TGTGCAGTACTGACTGGT	340	246	0	0	0	0
BFAR	ACACCACACAGTACGTTG	366	288	0	1	0	0
BFAR	CAACCACACAGTACCAGT	900	730	0	10	0	0
BFAR	ACACCACACAGTACCACA	222	199	0	0	0	0
BFAR	GTACCACACAGTACCAAC	32	96	0	0	0	0
BFAR	TGACCACACAGTACCATG	158	219	0	1	0	0
BFAR	CATGCACAACTGACTGCA	67	99	0	0	0	0
BGLAP	GTACGTTGGTTGGTTGAC	41	21	0	2	0	0
BGLAP	TGACGTTGGTTGGTTGTG	23	65	0	0	0	0
BGLAP	CAACGTTGGTTGCAGTGT	499	751	0	2	0	0
BGLAP	ACACGTTGGTTGCAGTCA	399	329	0	0	0	0
BGLAP	GTACGTTGGTTGCAGTAC	26	16	0	0	0	0
BGN	GTTGGTACCACAACTGAC	57	35	0	0	0	0
BGN	TGTGGTACCACAACTGTG	179	164	0	0	0	0
BGN	CATGGTACCACATGGTGT	465	456	0	60	0	0
BGN	ACTGGTACCACATGGTCA	315	873	1	0	0	0
BGN	GTTGGTACCACATGGTAC	6	17	0	0	0	0
BHLHB2	TGACGTCAACACTGGTGT	1331	1226	0	2	1	0
BHLHB2	ACACGTCAACACACTGAC	124	163	0	0	0	6
BHLHB2	CAACGTCAACACACTGTG	266	499	1	0	0	8
BHLHB2	GTACGTCAACACTGGTCA	189	524	0	0	0	0
BHLHB2	ACACGTCAACACTGGTAC	70	84	0	0	0	0
BHLHB2	ACTGCACAGTACCACAAC	33	29	0	0	0	0
BHLHB3	GTTGGTGTGTTGACGTTG	695	416	1	0	1	0
BHLHB3	TGACGTACACGTACACCA	136	182	0	0	0	0
BHLHB3	CAACGTACACGTACACAC	24	36	0	0	0	0
BHLHB3	GTACGTACACGTACACTG	134	58	0	0	0	0
BHLHB3	TGACGTACACGTACTGGT	79	76	0	0	0	0
BHLHB3	ACTGCACATGTGTGACCA	263	71	0	0	0	0
BHLHB8	CACATGTGACCAACACGT	8	8	0	0	0	0
BHLHB8	ACCATGTGACCAACACCA	566	435	0	0	0	3
BHLHB8	GTCATGTGACCAACACAC	52	85	0	0	0	0
BHLHB8	TGCATGTGACCAACACTG	1110	299	2	0	0	0
BHLHB8	ACCATGTGACCAACTGGT	610	568	0	0	0	0
BHLHB9	GTTGGTTGCACAGTTGGT	1156	1171	10	3	3	30
BHLHB9	TGTGGTTGCACAGTACGT	592	367	0	0	0	0
BHLHB9	GTTGGTTGCACAGTACCA	383	639	0	0	0	0
BHLHB9	ACTGGTTGCACAGTACAC	23	31	0	0	0	11
BHLHB9	CATGGTTGCACAGTACTG	234	250	2	0	0	0
BHMT	GTACCAACCAGTTGACTG	301	339	3	0	0	0
BHMT	TGACCAACCAGTTGTGGT	319	333	0	0	0	0
BHMT	GTACCAACCAGTTGTGCA	694	914	1	1	0	15
BHMT	ACACCAACCAGTTGTGAC	89	72	0	0	0	0
BHMT	CAACCAACCAGTTGTGTG	277	780	0	0	0	4
BHMT	GTTGCACAGTCAACACAC	10	35	0	0	0	0
BHMT2	ACACCAACCACAGTCAAC	11	7	0	0	0	0
BHMT2	CAACCAACCACAGTCATG	67	61	0	0	0	0
BHMT2	GTACCAACCACAGTACGT	177	103	0	0	0	0

BHMT2	CAACCAACCACAGTACCA	238	266	1	0	0	0
BHMT2	TGACCAACCACAGTACAC	132	105	0	0	0	0
BHMT2	TGTGCAACCATGGTACAC	27	27	0	0	0	0
BID	TGACTGACGTCACACAGT	353	244	2	1	0	0
BID	GTACTGACGTCACACACA	210	170	1	0	0	0
BID	ACACTGACGTCACACAAC	83	36	0	0	0	0
BID	CAACTGACGTCACACATG	96	112	0	0	0	0
BID	GTACTGACGTCACAACGT	105	49	0	0	0	0
BIK	GTACCACAGTACTGTGTG	739	1337	3	1	1	1
BIK	ACACCACAGTACTGTGGT	132	541	0	0	0	0
BIK	TGACCACAGTACTGTGCA	226	278	0	0	0	0
BIK	CAACCACAGTACTGTGAC	60	63	0	0	0	0
BIK	TGACCACAGTTGGTGTGT	120	153	1	0	0	0
BIK	CATGCAACACCACAGTCA	341	292	1	0	0	0
BIN1	TGTGGTCAGTGTGTACTG	949	978	3	1	0	0
BIN1	ACTGGTCAGTGTGTTGGT	144	279	0	2	0	2
BIN1	TGTGGTCAGTGTGTTGCA	290	285	0	0	0	0
BIN1	CATGGTCAGTGTGTTGAC	117	73	1	0	0	0
BIN1	GTTGGTCAGTGTGTTGTG	505	961	39	1	0	0
BIN1	ACTGCAACACCAGTTGTG	389	252	0	0	0	36
BIRC2	TGCATGTGGTGTGTCAAC	11	18	0	0	0	0
BIRC2	ACCATGTGGTGTGTCATG	86	110	0	0	0	0
BIRC2	CACATGTGGTGTGTACGT	93	107	0	16	0	0
BIRC2	ACCATGTGGTGTGTACCA	353	289	0	0	0	0
BIRC2	GTCATGTGGTGTGTACAC	33	33	0	0	0	0
BIRC3	GTCATGTGGTGTGTGTCA	580	287	2	0	0	0
BIRC3	ACCATGTGGTGTGTGTAC	145	29	0	1	0	0
BIRC3	CACATGTGGTGTGTGTTG	222	249	1	2	0	0
BIRC3	GTCATGTGGTGTGTCAGT	270	390	0	0	0	0
BIRC3	CACATGTGGTGTGTCACA	84	83	1	0	0	0
BIRC5	CAACTGTGGTCACAGTTG	841	847	60	1	1	0
BIRC5	TGACTGTGGTCACAGTGT	312	200	2	0	0	0
BIRC5	GTACTGTGGTCACAGTCA	218	236	1	0	0	0
BIRC5	ACACTGTGGTCACAGTAC	137	113	0	0	0	0
BIRC5	GTTGCAGTTGTGACACAC	84	83	12	0	0	0
BLK	TGTGGTACTGTGCACAAC	57	62	0	0	0	3
BLK	ACTGGTACTGTGCACATG	17	67	0	0	0	0
BLK	CATGGTACTGTGCAACGT	208	270	0	0	0	0
BLK	ACTGGTACTGTGCAACCA	67	72	1	0	0	0
BLK	ACTGCACAGTCAGTACAC	88	72	0	0	0	0
BLM	TGCATGTGCATGCAGTTG	875	1629	1	1	1	5
BLM	TGCATGTGCATGGTTGTG	183	188	1	0	0	0
BLM	CACATGTGCATGCAGTGT	572	388	2	0	0	0
BLM	ACCATGTGCATGCAGTCA	337	215	1	0	0	0
BLM	GTCATGTGCATGCAGTAC	18	14	0	0	0	0
BLMH	TGACACCATGTGGTACCA	96	122	0	0	1	0
BLMH	ACACACCATGTGGTACGT	347	210	1	1	0	0
BLMH	CAACACCATGTGGTACAC	59	49	0	0	0	0
BLMH	GTACACCATGTGGTACTG	701	639	0	1	0	0
BLMH	TGACACCATGTGGTTGGT	372	276	0	1	0	0
BLNK	GTACCAGTTGACCAGTTG	736	653	0	2	2	12
BLNK	ACACCAGTTGACCAGTGT	365	655	1	1	0	0
BLNK	TGACCAGTTGACCAGTCA	995	511	0	0	0	2
BLNK	CAACCAGTTGACCAGTAC	69	71	0	0	0	0
BLNK	TGACCAGTTGACCACAGT	371	168	0	0	0	0
BLR1	ACTGCAACGTGTTGTGAC	43	41	2	0	4	4
BLR1	ACACGTGTTGCATGTGGT	409	767	0	0	1	0
BLR1	TGACGTGTTGCATGTGCA	187	147	0	0	0	0
BLR1	CAACGTGTTGCATGTGAC	314	62	0	1	0	0
BLR1	GTACGTGTTGCATGTGTG	364	418	0	4	0	0
BLR1	GTTGCAACGTGTTGTGCA	52	70	0	0	0	0
BLVRA	GTACACGTTGACGTACGT	152	210	0	0	1	0
BLVRA	GTACACGTTGACGTCACA	64	79	1	0	0	0
BLVRA	ACACACGTTGACGTCAAC	12	19	0	0	0	0
BLVRA	CAACACGTTGACGTCATG	230	151	0	0	0	0
BLVRA	CAACACGTTGACGTACCA	261	328	0	1	0	0
BLVRB	TGTGCACAGTCAACACTG	759	813	3	1	1	0
BLVRB	ACACACGTTGACGTGTGT	57	46	0	0	0	0
BLVRB	TGACACGTTGACGTGTCA	104	52	0	0	0	0
BLVRB	CAACACGTTGACGTGTAC	94	63	0	0	0	0
BLVRB	GTACACGTTGACGTGTTG	210	231	0	0	0	0
BLVRB	TGACACGTTGACGTCAGT	125	300	0	1	0	0
BMI1	CAACGTCAACCATGGTGT	1090	1655	8	1	0	0
BMI1	GTACGTTGGTTGCATGGT	173	160	0	0	0	0
BMI1	CAACGTTGGTTGCATGCA	102	91	0	0	0	0

BMI1	TGACGTTGGTTGCATGAC	71	61	0	0	0	0
BMI1	ACACGTTGGTTGCATGTG	561	509	2	0	0	2
BMP1	ACCATGACTGCAGTGTAC	65	87	0	0	0	0
BMP1	CACATGACTGCAGTGTTG	2223	862	2	1	0	0
BMP1	GTCATGACTGCAGTCAGT	58	62	3	0	0	7
BMP1	CACATGACTGCAGTCACA	59	104	0	0	0	1
BMP1	TGCATGACTGCAGTCAAC	15	17	0	0	0	0
BMP1	TGTGCACAACCAACGTCA	41	51	0	0	0	0
BMP10	TGTGGTACGTCAACGTTG	584	662	1	0	1	0
BMP10	CATGGTACGTCAACGTGT	377	448	1	0	0	0
BMP10	ACTGGTACGTCAACGTCA	101	115	0	0	0	1
BMP10	GTTGGTACGTCAACGTAC	68	70	0	0	0	0
BMP10	TGTGCACAACACTGTGCA	255	102	1	0	0	0
BMP10	CATGCACAACACTGTGAC	42	42	0	0	0	0
BMP15	CATGCAGTTGACCACACA	271	149	0	0	1	17
BMP15	TGACTGGTACGTACCAAC	21	20	0	0	0	0
BMP15	ACACTGGTACGTACCATG	105	122	0	0	0	0
BMP15	CAACTGGTACGTACACGT	395	160	6	0	0	0
BMP15	ACACTGGTACGTACACCA	206	245	0	0	0	0
BMP15	TGTGCACACATGTGGTCA	47	69	1	0	0	0
BMP2	TGACTGGTCATGCAACCA	504	682	0	0	0	0
BMP2	CAACTGGTCATGCAACAC	73	62	0	0	0	0
BMP2	GTACTGGTCATGCAACTG	445	446	0	0	0	0
BMP2	TGACTGGTCATGCATGGT	731	644	4	3	0	13
BMP2	GTACTGGTCATGCATGCA	44	267	0	0	0	0
BMP3	ACACTGGTTGGTCAGTAC	212	52	0	0	0	0
BMP3	CAACTGGTTGGTCAGTTG	659	755	0	0	0	0
BMP3	GTACTGGTTGGTCACAGT	225	155	1	0	0	0
BMP3	CAACTGGTTGGTCACACA	309	164	0	1	0	0
BMP3	TGACTGGTTGGTCACAAC	20	13	0	0	0	0
BMP4	GTACGTTGTGTGGTGTGT	410	713	1	0	0	0
BMP4	GTACTGCAGTCAACCAAC	39	55	0	0	0	0
BMP4	TGACTGCAGTCAACCATG	42	90	0	0	0	0
BMP4	ACACTGCAGTCAACACGT	36	32	0	0	0	0
BMP4	TGACTGCAGTCAACACCA	508	780	2	0	0	1
BMP4	CATGCAACCAGTACCATG	80	155	0	0	0	12
BMP5	GTACTGGTACGTCAGTGT	164	244	0	0	0	0
BMP5	CAACTGGTACGTCAGTCA	467	156	0	1	0	0
BMP5	TGACTGGTACGTCAGTAC	74	138	0	0	0	0
BMP5	ACACTGGTACGTCAGTTG	198	259	0	0	0	0
BMP5	CAACTGGTACGTCACAGT	226	224	0	0	0	0
BMP5	GTTGCAACTGACCATGGT	185	241	0	0	0	0
BMP6	GTACTGGTTGGTGTTGCA	500	680	2	0	2	0
BMP6	ACACTGGTTGGTGTTGAC	11	21	0	0	0	0
BMP6	CAACTGGTTGGTGTTGTG	221	148	1	0	0	16
BMP6	TGACTGGTTGGTCAGTGT	1373	1185	1	0	0	0
BMP6	GTACTGGTTGGTCAGTCA	490	377	1	0	0	1
BMP7	ACACTGGTTGTGTGTGGT	223	509	0	0	0	0
BMP7	TGACTGGTTGTGTGTGCA	124	167	0	0	0	7
BMP7	CAACTGGTTGTGTGTGAC	8	11	0	0	0	0
BMP7	GTACTGGTTGTGTGTGTG	197	214	1	0	0	0
BMP7	TGACTGCAGTGTGTGTGT	769	992	1	1	0	1
BMP7	ACTGCACAGTCAACTGGT	765	524	0	0	0	0
BMP8A	ACACTGGTACGTCACACA	374	291	3	1	0	0
BMP8A	TGACTGGTACACTGGTGT	546	592	0	1	0	0
BMP8A	GTACTGGTACACTGGTCA	1072	954	0	0	0	0
BMP8A	ACTGCAGTTGACGTCACA	580	325	1	0	0	0
BMP8A	TGTGACACACACTGCAAC	24	26	0	0	0	0
BMP8B	TGACTGGTACGTCAACCA	705	589	1	1	3	1
BMP8B	GTACTGGTACGTCACAAC	20	7	0	0	0	8
BMP8B	TGACTGGTACGTCACATG	571	391	1	0	0	0
BMP8B	ACACTGGTACGTCAACGT	292	365	0	2	0	0
BMP8B	CAACTGGTACACACTGTG	295	209	0	0	0	0
BMPR1A	GTCATGCAACTGACTGCA	169	87	0	0	0	0
BMPR1A	ACCATGCAACTGACTGAC	22	8	0	0	0	0
BMPR1A	CACATGCAACTGACTGTG	579	339	0	0	0	0
BMPR1A	TGCATGCAACTGTGGTGT	827	407	0	0	0	3
BMPR1A	GTCATGCAACTGTGGTCA	113	123	1	0	0	0
BMPR1B	CACATGCACATGACCATG	208	190	0	0	0	0
BMPR1B	GTCATGCACATGACACGT	0	0	0	0	0	0
BMPR1B	CACATGCACATGACACCA	222	236	0	0	0	0
BMPR1B	TGCATGCACATGACACAC	23	11	0	0	0	0
BMPR1B	ACCATGCACATGACACTG	1190	1144	0	1	0	0
BMPR1B	TGTGCACAGTCAACTGCA	72	73	0	0	0	0
BMPR2	CACATGCACATGACTGGT	642	348	1	4	0	0

BMPR2	ACCATGCACATGACTGCA	145	253	1	0	0	0
BMPR2	GTCATGCACATGACTGAC	20	20	0	0	0	0
BMPR2	TGCATGCACATGACTGTG	133	216	1	0	0	0
BMPR2	CACATGCACATGTGGTGT	1718	839	648	2	0	0
BNIP1	GTACCACAGTTGGTGTCA	185	198	1	0	0	0
BNIP1	ACACCACAGTTGGTGTAC	7	20	0	0	0	0
BNIP1	CAACCACAGTTGGTGTTG	844	628	2	1	0	0
BNIP1	GTACCACAGTTGGTCAGT	69	41	0	0	0	0
BNIP1	CAACCACAGTTGGTCACA	116	207	0	0	0	0
BNIP1	CATGCACAACACTGACGT	64	74	0	0	0	0
BNIP2	TGACCACAGTTGGTCAAC	3	16	0	0	0	0
BNIP2	ACACCACAGTTGGTCATG	247	239	0	0	0	0
BNIP2	CAACCACAGTTGGTACGT	1110	1353	0	2	0	0
BNIP2	ACACCACAGTTGGTACCA	126	249	0	0	0	0
BNIP2	GTACCACAGTTGGTACAC	27	27	0	0	0	0
BNIP2	ACTGCACACAACTGACAC	99	61	0	0	0	0
BNIP3	GTTGCACAACTGCACACA	45	69	0	8	4	0
BNIP3	ACACGTGTACACTGGTGT	1150	558	0	0	1	0
BNIP3	TGACGTGTACACACTGCA	88	146	0	0	0	0
BNIP3	CAACGTGTACACACTGAC	43	68	0	0	0	0
BNIP3	GTACGTGTACACACTGTG	86	108	0	0	0	0
BNIP3	TGACGTGTACACTGGTCA	546	502	0	1	0	0
BNIP3L	ACACGTGTACACTGCAAC	33	19	0	0	0	0
BNIP3L	CAACGTGTACACTGCATG	53	64	1	1	0	0
BNIP3L	GTACGTGTACACTGACGT	146	130	0	0	0	1
BNIP3L	CAACGTGTACACTGACCA	266	229	0	0	0	0
BNIP3L	TGACGTCAACCACAGTGT	154	446	2	0	0	0
BOK	TGACCACAGTTGGTACTG	180	464	0	0	0	0
BOK	ACACCACAGTTGGTTGGT	514	456	1	0	0	0
BOK	TGACCACAGTTGGTTGCA	153	162	0	1	0	0
BOK	CAACCACAGTTGGTTGAC	99	22	0	0	0	0
BOK	GTACCACAGTTGGTTGTG	292	377	0	0	0	0
BP75	TGTGACCAACACGTGTTG	608	763	1	0	2	1
BP75	CATGACCAACACGTGTGT	283	412	0	0	1	0
BP75	GTTGACCAACACGTGTAC	64	52	0	0	1	0
BP75	ACTGACCAACACGTGTCA	245	200	1	0	0	0
BP75	ACTGACCAACACGTCAGT	69	105	0	0	0	0
BPNT1	GTACTGTGACACACCAGT	177	267	1	1	0	0
BPNT1	TGACTGTGACACTGCAGT	262	105	0	0	0	0
BPNT1	GTACTGTGACACTGCACA	216	156	5	0	0	0
BPNT1	ACACTGTGACACTGCAAC	16	14	0	0	0	1
BPNT1	CAACTGTGACACTGCATG	81	97	0	0	0	0
BPTF	TGACGTACACGTCAACGT	45	44	0	0	0	1
BPTF	GTACGTACACGTCAACCA	72	93	2	0	0	0
BPTF	ACACGTACACGTCAACAC	6	12	0	1	0	0
BPTF	CAACGTACACGTCAACTG	347	386	0	0	0	0
BPTF	GTACGTACACGTCATGGT	89	129	0	0	0	0
BRAF	TGACGTGTGTCAGTTGGT	1050	715	0	0	0	0
BRAF	GTACGTGTGTCAGTTGCA	260	238	0	2	0	0
BRAF	ACACGTGTGTCAGTTGAC	28	67	0	0	0	0
BRAF	CAACGTGTGTCAGTTGTG	64	70	0	0	0	0
BRAF	TGACGTGTGTCACAGTGT	304	240	0	0	0	0
BRCA1	GTACCATGCACACAGTTG	335	423	0	0	0	3
BRCA1	TGACCATGCACACACAGT	121	116	0	0	0	0
BRCA1	GTACCATGCACACACACA	141	110	0	0	0	0
BRCA1	TGACCATGCACACATGTG	430	546	0	0	0	0
BRCA1	CAACCATGCACAACGTGT	202	316	0	0	0	0
BRCA2	CAACCATGACGTACACAC	157	101	0	0	0	0
BRCA2	GTACCATGACGTACACTG	152	295	0	0	0	0
BRCA2	TGACCATGACGTACTGGT	92	170	0	1	0	1
BRCA2	GTACCATGACGTACTGCA	327	254	0	0	0	0
BRCA2	ACACCATGACGTACTGAC	27	23	0	0	0	0
BRD4	CATGCAACCAGTACACCA	668	667	0	0	1	0
BRD4	GTACGTTGACCAGTGTTG	524	418	1	0	0	0
BRD4	TGACGTTGACCAGTCAGT	130	100	1	0	0	0
BRD4	GTACGTTGACCAGTCACA	200	205	1	0	0	0
BRD4	ACACGTTGACCAGTCAAC	2	12	0	0	0	0
BRD4	CAACGTTGACCAGTCATG	206	67	0	0	0	0
BRS3	GTACGTGTTGACGTGTGT	630	1297	0	1	0	1
BRS3	CAACGTGTTGACGTGTCA	533	218	0	0	0	0
BRS3	TGACGTGTTGACGTGTAC	26	8	0	0	0	0
BRS3	ACACGTGTTGACGTGTTG	225	136	0	1	0	0
BRS3	CATGCACAGTCAACTGAC	53	197	1	0	0	0
BRS3	GTTGCACAGTCAACTGTG	184	56	0	0	0	0
BRUNOL4	ACACTGTGGTTGGTGTAC	23	4	0	0	0	0

BRUNOL4	CAACTGTGGTTGGTGTTG	398	300	0	0	0	0
BRUNOL4	GTACTGTGGTTGGTCAGT	38	51	0	0	0	13
BRUNOL4	CAACTGTGGTTGGTCACA	250	119	0	0	0	0
BRUNOL4	TGACTGTGGTTGGTCAAC	18	8	0	0	0	0
BST1	TGACACACACGTACGTCA	361	101	0	0	1	0
BST1	CAACACACACGTACGTAC	194	64	0	0	0	2
BST1	GTACACACACGTACGTTG	45	33	0	0	0	0
BST1	CATGCAGTTGCAACGTCA	178	222	2	0	0	0
BST1	CATGCACACAACTGACTG	119	438	0	0	0	0
BST1	GTTGCACACAACTGTGGT	280	261	1	1	0	0
BST2	GTTGGTGTCACATGTGTG	844	1147	0	0	0	0
BST2	GTTGGTGTCAACGTGTGT	453	585	0	0	0	0
BST2	CATGGTGTCAACGTGTCA	557	526	1	0	0	24
BST2	TGTGGTGTCAACGTGTAC	88	60	0	0	0	0
BST2	ACTGGTGTCAACGTGTTG	45	73	0	0	0	0
BST2	GTTGCACAACACTGCAGT	338	332	0	1	0	0
BTC	ACTGGTGTTGTGTGCATG	408	584	1	4	1	5
BTC	TGTGGTGTTGTGTGCAAC	21	26	0	0	0	0
BTC	CATGGTGTTGTGTGACGT	320	539	0	2	0	1
BTC	ACTGGTGTTGTGTGACCA	519	463	1	1	0	7
BTC	GTTGGTGTTGTGTGACAC	29	30	0	0	0	0
BTC	ACTGCACAGTCATGGTGT	151	209	1	1	0	0
BTD	ACACTGTGTGGTACCAGT	140	96	0	0	0	0
BTD	TGACTGTGTGGTACCACA	53	47	0	0	0	0
BTD	CAACTGTGTGGTACCAAC	58	195	0	0	0	0
BTD	GTACTGTGTGGTACCATG	386	313	22	0	0	1
BTD	TGACTGTGTGGTACACGT	77	142	0	1	0	0
BTG1	ACCATGTGGTGTACACAC	28	37	0	0	0	0
BTG1	CACATGTGGTGTACACTG	298	481	1	0	0	2
BTG1	GTCATGTGGTGTACTGGT	262	720	1	0	0	0
BTG1	CACATGTGGTGTACTGCA	229	107	0	0	0	0
BTG1	TGCATGTGGTGTACTGAC	84	99	0	0	0	0
BTG1	TGTGCACAGTCATGGTCA	50	84	0	0	0	0
BTG2	CAACGTGTACACCAGTCA	1074	645	2	1	1	1
BTG2	ACACGTGTACACCAGTTG	524	460	1	0	1	0
BTG2	TGACGTGTACACCAGTAC	51	37	0	0	0	0
BTG2	CAACGTGTACACCACAGT	105	167	0	0	0	0
BTG2	ACACGTGTACACCACACA	126	139	1	0	0	0
BTG2	TGTGCATGGTCAACACGT	55	66	0	1	0	0
BTG3	GTTGGTGTTGTGACGTGT	916	1512	2	0	0	2
BTG3	CATGGTGTTGTGACGTCA	255	299	0	0	0	0
BTG3	TGTGGTGTTGTGACGTAC	51	29	0	0	0	0
BTG3	ACTGGTGTTGTGACGTTG	21	54	0	0	0	4
BTG3	GTTGCAGTCACAACGTCA	191	389	1	0	0	0
BTG3	GTTGCATGGTCAACACCA	255	195	0	1	0	0
BTK	GTCATGCACAACCAACGT	786	450	0	0	0	0
BTK	CACATGCACAACCAACCA	547	596	0	1	0	0
BTK	TGCATGCACAACCAACAC	173	149	0	0	0	0
BTK	ACCATGCACAACCAACTG	871	799	1	1	0	6
BTK	CAACGTTGTGACGTTGAC	25	10	0	0	0	0
BTK	TGTGCACACACAACCATG	348	349	1	0	0	0
BTLA	ACTGGTCATGTGACTGTG	92	124	0	1	0	0
BTLA	GTTGGTCATGTGTGGTGT	605	1382	0	0	0	1
BTLA	CATGGTCATGTGTGGTCA	347	517	0	0	0	4
BTLA	TGTGGTCATGTGTGGTAC	37	39	0	0	0	0
BTLA	ACTGACACACACTGGTAC	27	26	0	0	0	0
BTRC	TGACGTGTGTTGGTGTGT	593	648	0	1	1	0
BTRC	CAACGTGTGTACTGTGAC	94	68	1	0	0	17
BTRC	GTACGTGTGTACTGTGTG	253	290	5	0	0	0
BTRC	GTACGTGTGTTGGTGTCA	479	193	0	0	0	0
BTRC	ACACGTGTGTTGGTGTAC	97	82	0	0	0	0
BUB1	CACATGCAACTGTGGTTG	524	418	0	0	2	2
BUB1	ACCATGCAACTGTGGTAC	94	49	0	0	0	0
BUB1	GTCATGCAACTGTGCAGT	575	405	3	0	0	0
BUB1	CACATGCAACTGTGCACA	169	167	1	0	0	5
BUB1	GTACCATGACGTCAGTAC	42	213	0	1	0	0
BUB1B	TGCATGCACATGTGGTTG	250	290	0	0	1	0
BUB1B	ACCATGCACATGTGGTCA	192	367	0	0	0	0
BUB1B	GTCATGCACATGTGGTAC	40	24	0	1	0	0
BUB1B	ACCATGCACATGTGCAGT	24	59	0	0	0	0
BUB1B	TGCATGCACATGTGCACA	69	76	0	0	0	0
BUB3	TGACTGTGGTTGGTTGCA	198	238	0	0	1	0
BUB3	TGACTGTGGTTGGTACTG	273	345	1	0	0	62
BUB3	ACACTGTGGTTGGTTGGT	190	195	2	0	0	6
BUB3	CAACTGTGGTTGGTTGAC	117	91	0	0	0	5

BUB3	GTACTGTGGTTGGTTGTG	259	40	0	0	0	0
BYSL	ACTGGTCACATGGTCAAC	33	19	0	2	3	193
BYSL	TGTGGTCACATGGTCAGT	279	347	0	0	0	0
BYSL	GTTGGTCACATGGTCACA	66	59	0	0	0	0
BYSL	CATGGTCACATGGTCATG	73	86	0	0	0	0
BYSL	GTTGGTCACATGGTACGT	165	117	0	0	0	0
BZW1	ACTGGTACGTGTCACATG	465	529	47	76	65	139
BZW1	CATGGTGTCAGTACACTG	390	459	1	0	0	0
BZW1	TGTGGTACGTGTCACAAC	12	14	0	0	0	0
BZW1	CATGGTACGTGTCAACGT	226	203	1	0	0	0
BZW1	ACTGGTACGTGTCAACCA	109	120	1	0	0	0
C10ORF110	GTTGCACATGGTACACGT	80	90	0	0	1	0
C10ORF110	CATGGTTGACCAACTGCA	405	248	0	0	0	0
C10ORF110	TGTGGTTGACCAACTGAC	78	106	0	0	0	0
C10ORF110	ACTGGTTGACCAACTGTG	92	100	0	1	0	0
C10ORF110	ACTGCACATGGTACCAAC	28	59	1	0	0	2
C10ORF110	CATGCACATGGTACCATG	67	60	0	1	0	0
C12ORF57	GTTGCAGTGTGTACCAGT	100	121	0	0	0	0
C12ORF57	GTTGCAGTCAACTGCAAC	19	19	0	0	0	0
C12ORF57	TGTGCAGTCAACTGCATG	82	89	0	0	0	0
C12ORF57	ACTGCAGTCAACTGACGT	292	168	0	0	0	0
C12ORF57	TGTGCAGTCAACTGACCA	246	151	0	0	0	59
C13ORF15	CATGGTACGTACGTGTGT	211	238	0	0	0	0
C13ORF15	ACTGGTACGTACGTGTCA	290	223	1	2	0	1
C13ORF15	GTTGGTACGTACGTGTAC	64	16	0	0	0	0
C13ORF15	TGTGGTACGTACGTGTTG	899	248	0	0	0	0
C13ORF15	ACTGGTACGTACGTCAGT	95	176	0	0	0	0
C14ORF166	GTACTGTGCAGTCAACCA	485	607	3424	2	1	0
C14ORF166	ACACTGTGCAGTCAACAC	79	75	0	0	0	0
C14ORF166	CAACTGTGCAGTCAACTG	1098	358	1	0	0	0
C14ORF166	GTACTGTGCAGTCATGGT	366	562	0	0	0	1
C14ORF166	CAACTGTGCAGTCATGCA	322	300	0	0	0	0
C14ORF166	ACTGCACAACTGACTGTG	644	243	0	1	0	0
C15ORF15	TGTGGTGTTGACTGACAC	29	43	0	0	0	0
C15ORF15	ACTGGTGTTGACTGACTG	545	265	1	1	0	0
C15ORF15	CATGGTGTTGACTGTGGT	655	891	0	1	0	4
C15ORF15	ACTGGTGTTGACTGTGCA	152	170	0	0	0	0
C15ORF15	GTTGGTTGACACCAGTGT	370	347	0	0	0	0
C15ORF15	CATGCAACCAGTGTTGTG	247	120	0	0	0	0
C19ORF40	ACTGCAACACTGCAACCA	47	66	0	1	1	0
C19ORF40	GTTGGTCACAACACCAGT	227	232	0	0	0	0
C19ORF40	CATGGTCACAACACCACA	71	80	1	0	0	0
C19ORF40	TGTGGTCACAACACCAAC	86	92	1	0	0	91
C19ORF40	ACTGGTCACAACACCATG	83	414	0	0	0	1
C19ORF40	CATGGTCACAACACACGT	64	178	0	0	0	1
C1GALT1	GTTGCACATGGTTGTGGT	1083	1145	2	1	1	1
C1GALT1	TGACCACATGCATGCAAC	11	1	0	0	0	0
C1GALT1	ACACCACATGCATGCATG	152	182	0	0	0	0
C1GALT1	CAACCACATGCATGACGT	235	161	0	0	0	0
C1GALT1	ACACCACATGCATGACCA	172	234	0	1	0	0
C1GALT1	GTACCACATGCATGACAC	85	34	0	0	0	0
C1GALT1C1	TGACCACAACCAACACGT	70	66	0	0	0	0
C1GALT1C1	GTACCACAACCAACACCA	313	597	0	1	0	0
C1GALT1C1	ACACCACAACCAACACAC	62	34	0	0	0	0
C1GALT1C1	CAACCACAACCAACACTG	49	55	0	0	0	0
C1GALT1C1	GTACCACAACCAACTGGT	182	214	0	1	0	0
C1ORF103	CAACTGACGTTGTGCACA	57	66	0	0	0	0
C1ORF103	TGACTGACGTTGTGCAAC	12	6	0	0	0	0
C1ORF103	ACACTGACGTTGTGCATG	163	126	0	1	0	0
C1ORF103	ACTGACGTACTGACACCA	38	34	0	0	0	0
C1ORF103	GTTGACGTACTGACACAC	41	49	0	0	0	0
C1ORF142	TGTGGTTGGTCATGACGT	202	240	0	0	0	0
C1ORF142	GTTGGTTGGTCATGACCA	94	119	0	0	0	0
C1ORF142	ACTGGTTGGTCATGACAC	59	188	0	0	0	0
C1ORF142	CATGGTTGGTCATGACTG	164	89	0	0	0	0
C1ORF142	GTTGGTTGGTCATGTGGT	481	1077	2	0	0	0
C1ORF142	ACTGCAACTGTGGTGTAC	350	83	1	0	0	0
C1QA	CAACACTGACTGTGACAC	43	28	0	0	0	1
C1QA	GTACACTGACTGTGACTG	258	218	0	0	0	0
C1QA	TGACACTGACTGTGTGGT	164	218	0	0	0	0
C1QA	GTACACTGACTGTGTGCA	166	185	0	0	0	1
C1QA	ACACACTGACTGTGTGAC	22	18	0	0	0	0
C1QA	GTTGCACAACTGACCATG	20	68	1	0	0	0
C1QB	ACTGCAGTTGCAGTTGCA	361	226	1	0	1	0
C1QB	GTACACTGTGGTCAACAC	67	68	0	0	0	3

C1QB	TGACACTGTGGTCAACTG	84	90	0	0	0	0
C1QB	ACACACTGTGGTCATGGT	132	139	1	0	0	1
C1QB	TGACACTGTGGTCATGCA	63	81	0	0	0	0
C1QBP	GTACACTGACTGACACCA	109	124	0	0	0	23
C1QBP	ACACACTGACTGACACAC	41	78	0	0	0	0
C1QBP	CAACACTGACTGACACTG	439	579	0	0	0	0
C1QBP	CATGCAGTTGCACACAAC	22	34	0	0	0	0
C1QBP	CATGCATGGTCATGCAGT	341	206	0	0	0	0
C1QBP	ACTGCATGGTCATGCACA	15	28	0	0	0	0
C1QC	GTACACTGTGCAGTCAGT	117	81	0	0	0	0
C1QC	CAACACTGTGCAGTCACA	40	15	0	0	0	0
C1QC	TGACACTGTGCAGTCAAC	62	103	0	0	0	0
C1QC	ACACACTGTGCAGTCATG	501	286	0	0	0	0
C1QC	CAACACTGTGCAGTACGT	452	538	0	1	0	1
C1QL1	TGTGGTGTACCACACAAC	28	13	0	0	0	0
C1QL1	GTTGGTACCATGACCATG	156	329	0	0	0	0
C1QL1	TGTGGTACCATGACACGT	237	129	0	0	0	0
C1QL1	GTTGGTACCATGACACCA	417	485	16	1	0	0
C1QL1	ACTGGTACCATGACACAC	161	123	0	0	0	0
C1QL2	ACTGGTACCACAGTACGT	146	229	2	1103	2	1
C1QL2	GTTGGTACCACAGTCAAC	50	66	0	0	0	0
C1QL2	TGTGGTACCACAGTCATG	157	209	2	0	0	0
C1QL2	TGTGGTACCACAGTACCA	53	94	0	0	0	0
C1QL2	CATGGTACCACAGTACAC	71	41	0	0	0	0
C1QL3	CATGGTACACTGTGCATG	157	139	0	1	1	0
C1QL3	GTTGGTACACTGTGACGT	137	172	0	0	0	0
C1QL3	CATGGTACACTGTGACCA	421	352	0	0	0	1
C1QL3	TGTGGTACACTGTGACAC	40	70	0	0	0	0
C1QL3	ACTGGTACACTGTGACTG	415	360	0	1	0	0
C1QL4	ACTGGTTGACCATGGTTG	598	404	1	0	0	0
C1QL4	CATGGTTGACCATGCAGT	59	64	0	1	0	0
C1QL4	ACTGGTTGACCATGCACA	193	252	0	1	0	3
C1QL4	GTTGGTTGACCATGCAAC	45	52	0	0	0	0
C1QL4	CATGACGTTGCAGTCAAC	39	36	0	0	0	0
C1R	TGCATGACTGCACATGTG	395	339	0	1	1	0
C1R	ACTGACCATGGTGTGTAC	44	73	0	0	0	0
C1R	CATGACCATGGTGTGTTG	984	1110	1	1	0	0
C1R	GTTGACCATGGTGTCAGT	126	62	0	0	0	0
C1R	CATGACCATGGTGTCACA	80	113	0	0	0	0
C1RL	GTTGCACAACTGACTGGT	455	515	0	12	1	0
C1RL	ACACTGTGCAGTTGCATG	80	251	1	0	0	0
C1RL	CAACTGTGCAGTTGACGT	275	108	1	0	0	0
C1RL	ACACTGTGCAGTTGACCA	151	267	0	0	0	0
C1RL	GTACTGTGCAGTTGACAC	201	125	0	0	0	0
C1RL	TGACTGTGCAGTTGACTG	591	395	1	0	0	0
C1S	CACATGACTGCAACGTGT	705	795	1	0	1	1
C1S	ACCATGACTGCAACGTCA	296	223	0	0	0	9
C1S	GTCATGACTGCAACGTAC	89	86	0	0	0	0
C1S	TGCATGACTGCAACGTTG	866	1065	0	0	0	0
C1S	ACCATGACTGCAACCAGT	247	234	1	0	0	0
C2	ACCATGACTGGTTGACAC	7	7	0	0	0	0
C2	CACATGACTGGTTGACTG	215	388	4	1	0	3
C2	GTCATGACTGGTTGTGGT	205	410	0	0	0	0
C2	CACATGACTGGTTGTGCA	547	278	0	0	0	0
C2	TGCATGACTGGTTGTGAC	85	117	1	1	0	0
C2	ACTGCAACCATGGTACTG	270	208	1	0	0	0
C22ORF28	ACACTGTGCAGTACGTTG	178	176	7	0	0	0
C22ORF28	CAACTGTGCAGTACCAGT	211	174	0	0	0	0
C22ORF28	ACACTGTGCAGTACCACA	146	42	1	0	0	0
C22ORF28	GTACTGTGCAGTACCAAC	29	17	0	0	0	0
C22ORF28	TGACTGTGCAGTACCATG	416	293	1	0	0	0
C3	TGACACTGTGGTGTACCA	435	277	8	0	0	0
C3	CAACACTGTGGTGTACAC	14	12	0	0	0	0
C3	GTACACTGTGGTGTACTG	71	93	0	0	0	0
C3	TGACACTGTGGTGTTGGT	751	557	0	1	0	5
C3	GTACACTGTGGTGTTGCA	74	87	0	0	0	0
C3	ACTGCACACACAACACGT	25	12	0	0	0	0
C3AR1	GTTGCAACCATGTGGTCA	289	345	1	0	3	3
C3AR1	CAACGTGTTGACGTCAGT	278	129	1	1	0	0
C3AR1	ACACGTGTTGACGTCACA	225	258	143	0	0	0
C3AR1	GTACGTGTTGACGTCAAC	25	16	0	0	0	0
C3AR1	TGACGTGTTGACGTCATG	2	24	0	0	0	0
C3AR1	TGTGCAACCATGTGGTGT	126	115	0	0	0	0
C4A	TGACACTGACTGTGCATG	65	121	0	1	1	3
C4A	GTACACTGACTGTGCAAC	9	14	0	0	0	0

C4A	ACACACTGACTGTGACGT	77	119	0	0	0	0
C4A	TGACACTGACTGTGACCA	543	225	0	0	0	0
C4A	ACACACTGTGGTGTTGAC	33	36	0	0	0	0
C4B	CAACACTGTGGTGTTGTG	89	82	0	0	0	0
C4B	TGACACTGTGGTCAGTGT	155	171	0	0	0	0
C4B	CATGGTTGCACAGTTGCA	137	126	0	1	0	1
C4B	TGTGGTTGCACAGTTGAC	143	39	0	0	0	0
C4B	ACTGGTTGCACAGTTGTG	201	218	1	0	0	0
C4BPA	TGTGCAGTTGCAGTTGTG	221	163	0	0	1	0
C4BPA	GTACACTGTGGTTGTGGT	686	502	1	0	0	1
C4BPA	CAACACTGTGGTTGTGCA	102	117	0	0	0	0
C4BPA	TGACACTGTGGTTGTGAC	72	44	0	0	0	0
C4BPA	CATGCAGTTGCACAGTGT	1229	487	3	0	0	0
C4BPB	CAACACTGTGGTCACACA	59	45	0	0	0	0
C4BPB	TGACACTGTGGTCACAAC	22	14	0	0	0	0
C4BPB	ACACACTGTGGTCACATG	141	179	0	1	0	0
C4BPB	CAACACTGTGGTCAACGT	352	299	0	0	0	0
C4BPB	ACACACTGTGGTCAACCA	594	517	1	0	0	0
C5	TGACACTGTGCAGTGTGT	234	331	0	0	1	0
C5	ACACACTGTGGTTGTGTG	354	298	0	0	0	0
C5	GTACACTGTGCAGTGTCA	229	265	0	0	0	0
C5	ACACACTGTGCAGTGTAC	40	29	0	0	0	0
C5	CAACACTGTGCAGTGTTG	240	272	1	0	0	0
C5AR1	ACACGTGTTGACGTACGT	412	139	0	0	0	1
C5AR1	TGACGTGTTGACGTACCA	774	438	1	1	0	0
C5AR1	CAACGTGTTGACGTACAC	54	33	0	0	0	15
C5AR1	GTACGTGTTGACGTACTG	569	409	1	1	0	0
C5AR1	TGACGTGTTGACGTTGGT	176	128	1	0	0	1
C6	GTACACTGTGGTACGTTG	1028	985	1	2	0	0
C6	TGACACTGTGGTACCAGT	137	149	0	0	0	0
C6	GTACACTGTGGTACCACA	194	157	0	0	0	0
C6	ACACACTGTGGTACCAAC	13	15	0	0	0	0
C6	CAACACTGTGGTACCATG	344	351	0	1	0	6
C6	GTTGCACACAACTGGTAC	94	60	0	0	0	0
C7	GTACACTGTGGTACACGT	121	93	0	0	0	0
C7	CAACACTGTGGTACACCA	196	139	0	0	0	0
C7	TGACACTGTGGTACACAC	116	18	0	0	0	1
C7	ACACACTGTGGTACACTG	225	181	1	0	0	0
C7	CAACACTGTGGTACTGGT	477	223	2	0	0	0
C7ORF16	TGCATGACGTACCAACAC	0	3	0	0	0	0
C7ORF16	ACCATGACGTACCAACTG	138	55	1	1	0	0
C7ORF16	CACATGACGTACCATGGT	489	110	2	0	0	0
C7ORF16	ACCATGACGTACCATGCA	98	130	0	0	0	0
C7ORF16	GTCATGACGTACCATGAC	18	119	0	0	0	0
C7ORF16	GTTGCATGGTTGTGTGTG	390	364	0	0	0	0
C8A	TGACACTGTGGTACTGTG	672	662	2	0	1	0
C8A	ACACACTGTGGTACTGCA	103	118	1	0	0	0
C8A	GTACACTGTGGTACTGAC	21	22	0	0	0	0
C8A	CAACACTGTGGTTGGTGT	207	163	0	0	0	0
C8A	ACACACTGTGGTTGGTCA	194	303	0	0	0	0
C8A	TGTGCACACACAACACCA	55	53	0	1	0	0
C8B	TGACACTGACTGACCACA	277	342	1	1	0	3
C8B	CAACACTGACTGACCAAC	52	54	0	0	0	0
C8B	GTACACTGACTGACCATG	182	244	2	0	0	0
C8B	TGACACTGACTGACACGT	39	42	0	0	0	0
C8B	GTTGACCACAACGTACAC	32	40	0	0	0	0
C8G	GTACACTGTGGTCAGTCA	586	214	17	0	15	10
C8G	ACACACTGTGGTCAGTAC	48	83	0	0	0	0
C8G	CAACACTGTGGTCAGTTG	2119	1338	4	1	0	6
C8G	GTACACTGTGGTCACAGT	270	245	1	0	0	0
C8G	GTTGCAGTTGCAGTTGAC	43	32	0	0	0	0
C8G	CATGCACACACAACACAC	28	14	0	0	0	0
C9	GTACACTGTGGTTGCATG	121	186	1	0	0	0
C9	TGACACTGTGGTTGACGT	208	652	0	0	0	1
C9	GTACACTGTGGTTGACCA	312	321	0	0	0	0
C9	ACACACTGTGGTTGACAC	30	37	0	0	0	0
C9	CAACACTGTGGTTGACTG	127	165	0	0	0	0
C9	CATGCATGCAGTGTGTGT	765	857	2	0	0	4
C9ORF47	TGTGGTTGACCATGCATG	664	510	1	1	0	1
C9ORF47	ACTGGTTGACCATGACGT	35	52	1	1	0	0
C9ORF47	TGTGGTTGACCATGACCA	43	39	1	0	0	0
C9ORF47	CATGGTTGACCATGACAC	25	22	0	0	0	0
C9ORF47	GTTGGTTGACCATGACTG	256	422	0	0	0	0
C9ORF95	ACTGGTACTGGTGTTGCA	240	281	0	15	1	0
C9ORF95	ACTGGTACTGGTGTACTG	334	51	0	0	0	0

C9ORF95	CATGGTACTGGTGTTGGT	336	214	0	0	0	0
C9ORF95	GTTGGTACTGGTGTTGAC	30	39	0	0	0	0
C9ORF95	TGTGGTACTGGTGTTGTG	477	464	1	0	0	1
C9ORF95	CATGCACATGGTCATGAC	49	128	0	0	0	0
C9ORF97	TGACACACCAGTTGACTG	752	869	1	1	1	2
C9ORF97	ACACACACCAGTTGTGGT	223	238	0	0	0	10
C9ORF97	TGACACACCAGTTGTGCA	506	260	0	0	0	0
C9ORF97	CAACACACCAGTTGTGAC	28	55	0	0	0	0
C9ORF97	GTACACACCAGTTGTGTG	169	123	0	1	0	0
CA1	GTTGCACAGTCATGGTTG	822	402	2	0	2	444
CA1	TGTGGTCAGTCAGTTGTG	225	308	0	1	0	1
CA1	CATGGTCAGTCACAGTGT	805	699	0	0	0	0
CA1	ACTGGTCAGTCACAGTCA	3	16	0	0	0	0
CA1	GTTGGTCAGTCACAGTAC	90	86	0	0	0	0
CA1	TGTGGTCAGTCACAGTTG	591	619	0	0	0	0
CA12	ACTGGTGTACACTGACCA	134	202	0	0	0	0
CA12	GTTGGTGTACACTGACAC	5	19	0	0	0	0
CA12	TGTGGTGTACACTGACTG	432	533	0	0	0	0
CA12	ACTGGTGTACACTGTGGT	164	168	1	0	0	0
CA12	GTTGCACAGTGTACCAAC	13	9	0	0	0	0
CA13	ACTGGTTGCACACAGTTG	964	672	2	0	2	41
CA13	CATGGTTGCACACACAGT	711	431	0	0	1	2
CA13	GTTGGTTGCACACAGTGT	359	458	0	0	0	0
CA13	CATGGTTGCACACAGTCA	991	1056	2	0	0	1
CA13	TGTGGTTGCACACAGTAC	19	126	0	0	0	0
CA14	GTTGCACAACACCAACCA	349	352	0	1	1	0
CA14	ACTGGTTGCACACACACA	104	119	1	1	0	0
CA14	GTTGGTTGCACACACAAC	16	22	0	0	0	0
CA14	TGTGGTTGCACACACATG	160	315	1	0	0	0
CA14	ACTGGTTGCACACAACGT	329	471	0	0	0	2
CA14	TGTGGTTGCACACAACCA	136	198	0	0	0	0
CA2	GTTGGTGTGTTGGTACTG	314	396	1	1	1	0
CA2	ACTGGTGTGTTGGTACGT	293	323	0	0	0	0
CA2	TGTGGTGTGTTGGTACCA	73	76	1	1	0	0
CA2	CATGGTGTGTTGGTACAC	46	80	0	0	0	0
CA2	GTTGCAGTCAGTTGCAGT	449	133	0	0	0	12
CA2	GTTGCACACACAACACTG	271	276	4	0	0	0
CA3	TGTGGTACCAGTACCAAC	54	48	0	0	0	0
CA3	ACTGGTACCAGTACCATG	268	376	0	1	0	0
CA3	CATGGTACCAGTACACGT	166	143	0	0	0	0
CA3	ACTGGTACCAGTACACCA	214	213	0	0	0	2
CA3	GTTGGTACCAGTACACAC	84	80	0	0	0	0
CA3	TGTGCACAACACACCATG	98	96	0	0	0	0
CA4	TGTGGTACACCAGTCAGT	244	233	0	0	0	0
CA4	GTTGGTACACCAGTCACA	92	138	0	0	0	0
CA4	ACTGGTACACCAGTCAAC	10	34	0	0	0	0
CA4	CATGGTACACCAGTCATG	141	153	0	0	0	0
CA4	GTTGGTACACCAGTACGT	675	436	1	0	0	0
CA4	ACTGCACATGCAGTGTAC	171	23	0	0	0	1
CA5A	GTACACACTGGTTGGTGT	624	711	4	0	0	0
CA5A	CAACACACTGGTTGGTCA	211	228	0	0	0	0
CA5A	TGTGGTTGGTGTTGTGTG	508	1559	0	1	0	0
CA5A	GTTGGTTGGTCAGTGTGT	750	907	1	0	0	0
CA5A	CATGGTTGGTCAGTGTCA	239	431	0	0	0	1
CA5A	TGTGCACAGTCATGCAGT	74	81	0	0	0	0
CA5B	CATGGTACCACACACACA	309	329	0	0	2	0
CA5B	TGTGGTACCACACACAAC	13	11	0	0	0	0
CA5B	ACTGGTACCACACACATG	155	111	1	0	0	0
CA5B	CATGGTACCACACAACGT	845	556	1	0	0	3
CA5B	ACTGGTACCACACAACCA	137	278	0	0	0	1
CA6	TGTGGTACACCATGTGCA	143	434	3	0	0	9
CA6	CATGGTACACCATGTGAC	33	17	0	0	0	0
CA6	GTTGGTACACCATGTGTG	221	207	1	0	0	0
CA6	GTTGGTACACACGTGTGT	958	304	1	0	0	0
CA6	CATGGTACACACGTGTCA	63	44	0	0	0	0
CA7	ACTGGTACCATGGTCATG	97	109	0	0	1	9
CA7	GTTGGTACCATGGTCAGT	456	458	0	3	0	0
CA7	CATGGTACCATGGTCACA	146	42	1	0	0	0
CA7	TGTGGTACCATGGTCAAC	15	8	0	0	0	0
CA7	CATGGTACCATGGTACGT	85	70	0	0	0	0
CA8	TGTGGTACACACTGGTTG	524	939	1	2	1	0
CA8	ACTGGTACACACTGCAGT	143	149	0	0	0	0
CA8	TGTGGTACACACTGCACA	290	188	0	0	0	0
CA8	CATGGTACACACTGCAAC	29	34	0	0	0	0
CA8	GTTGGTACACACTGCATG	167	231	0	0	0	0

CA9	CATGGTTGGTCACAACGT	120	108	0	0	0	3
CA9	ACTGGTTGGTCACAACCA	118	118	32	0	0	0
CA9	GTTGGTTGGTCACAACAC	18	29	0	0	0	0
CA9	TGTGGTTGGTCACAACTG	460	512	1	1	0	12
CA9	ACTGGTTGGTCACATGGT	885	431	2	1	0	0
CA9	CATGCACATGCAGTGTTG	162	105	0	0	0	0
CAB39	TGACCATGTGTGCATGTG	1445	909	3	0	1	1
CAB39	GTACCATGTGTGCATGAC	29	133	0	0	0	0
CAB39	CAACCATGTGTGACGTGT	161	192	0	0	0	0
CAB39	ACACCATGTGTGACGTCA	110	145	1	0	0	0
CAB39	CATGCAGTCAGTACACAC	51	66	0	0	0	0
CABIN1	TGTGGTCATGGTGTGTTG	476	650	1	9	4	5
CABIN1	CATGCAACACACGTGTTG	554	448	2	0	1	0
CABIN1	TGTGGTCACAACGTTGAC	65	199	0	0	0	0
CABIN1	ACTGGTCATGGTGTGTCA	1610	960	11	1	0	0
CABIN1	GTTGGTCATGGTGTGTAC	115	219	0	0	0	0
CABIN1	ACTGGTCATGGTGTCAGT	261	333	0	0	0	0
CACNA1A	ACACGTCATGGTCACATG	235	314	28	0	0	36
CACNA1A	CAACGTCATGGTCAACGT	788	833	0	1	0	1
CACNA1A	ACACGTCATGGTCAACCA	274	287	1	0	0	0
CACNA1A	GTACGTCATGGTCAACAC	31	24	0	0	0	0
CACNA1A	GTTGCAGTACACCAGTGT	431	359	0	0	0	0
CACNA1A	GTTGCACATGTGACCAAC	31	17	0	0	0	0
CACNA1B	GTACACGTGTCAACACTG	288	608	1	2	1	0
CACNA1B	CAACACGTGTCAACACAC	170	6	0	0	0	1
CACNA1B	TGACACGTGTCAACTGGT	93	455	0	0	0	0
CACNA1B	GTACACGTGTCAACTGCA	24	95	0	0	0	5
CACNA1B	ACACACGTGTCAACTGAC	87	6	0	0	0	0
CACNA1B	GTTGCACAGTCATGCACA	17	20	0	0	0	0
CACNA1C	CAACACGTGTCATGCACA	159	214	1	0	2	0
CACNA1C	CAACACGTGTCATGACGT	1419	467	1	2	1	0
CACNA1C	GTACACGTGTCATGCAGT	247	192	0	1	0	0
CACNA1C	TGACACGTGTCATGCAAC	10	24	0	0	0	0
CACNA1C	ACACACGTGTCATGCATG	48	77	0	0	0	0
CACNA1D	TGACACGTGTCATGTGCA	56	53	0	0	1	0
CACNA1D	ACACACGTGTCATGACCA	38	23	0	0	0	0
CACNA1D	GTACACGTGTCATGACAC	51	57	0	0	0	0
CACNA1D	TGACACGTGTCATGACTG	141	198	1	1	0	0
CACNA1D	ACACACGTGTCATGTGGT	33	51	0	0	0	0
CACNA1D	ACTGCACAGTCATGCAAC	27	53	0	0	0	0
CACNA1E	GTACACGTGTACGTGTGT	468	1087	1	1	1	0
CACNA1E	CAACACGTGTCATGTGAC	11	35	0	0	0	0
CACNA1E	GTACACGTGTCATGTGTG	178	143	0	0	0	0
CACNA1E	CAACACGTGTACGTGTCA	180	201	0	0	0	0
CACNA1E	CATGCAGTTGGTGTACGT	150	139	0	0	0	5
CACNA1F	ACACCATGTGCATGCAGT	131	124	3	0	0	0
CACNA1F	TGACCATGTGCATGCACA	99	45	0	0	0	0
CACNA1F	CAACCATGTGCATGCAAC	62	158	0	0	0	1
CACNA1F	GTACCATGTGCATGCATG	180	35	2	0	0	0
CACNA1F	TGACCATGTGCATGACGT	339	650	1	0	0	1
CACNA1G	CAACCATGTGTGCAACCA	229	189	0	0	0	0
CACNA1G	TGACCATGTGTGCAACAC	62	71	0	0	0	0
CACNA1G	ACACCATGTGTGCAACTG	564	609	2	1	0	0
CACNA1G	CAACCATGTGTGCATGGT	219	285	0	0	0	0
CACNA1G	ACACCATGTGTGCATGCA	81	113	0	0	0	0
CACNA1H	ACACCATGTGTGCAGTGT	256	186	0	0	0	3
CACNA1H	TGACCATGTGTGCAGTCA	483	195	0	1	0	11
CACNA1H	CAACCATGTGTGCAGTAC	17	87	1	0	0	0
CACNA1H	GTACCATGTGTGCAGTTG	873	507	1	0	0	0
CACNA1H	GTTGCAGTTGGTCACACA	120	180	0	0	0	154
CACNA1I	CAACCATGTGTGGTCACA	467	412	0	0	0	0
CACNA1I	TGACCATGTGTGGTCAAC	2	6	0	0	0	0
CACNA1I	ACACCATGTGTGGTCATG	126	172	0	0	0	0
CACNA1I	CAACCATGTGTGGTACGT	641	676	0	0	0	0
CACNA1I	TGTGCAGTTGGTCACAGT	102	79	0	0	0	0
CACNA1S	CAACCATGTGCATGTGCA	50	50	0	0	0	0
CACNA1S	TGACCATGTGCATGTGAC	43	120	1	0	0	0
CACNA1S	ACACCATGTGCATGTGTG	515	489	0	0	0	1
CACNA1S	ACACCATGTGACGTGTGT	484	565	0	1	0	0
CACNA1S	TGACCATGTGACGTGTCA	779	453	0	0	0	0
CACNA1S	TGTGCACACACAACTGGT	122	161	0	0	0	0
CACNA2D1	GTACCATGTGACGTACGT	215	186	0	0	1	0
CACNA2D1	ACACCATGTGACGTCAAC	124	58	0	0	0	0
CACNA2D1	CAACCATGTGACGTCATG	138	201	0	0	0	0
CACNA2D1	CAACCATGTGACGTACCA	71	47	0	0	0	0

CACNA2D1	TGACCATGTGACGTACAC	17	30	0	0	0	0
CACNA2D2	GTACACGTGTTGGTTGGT	292	685	1	0	1	16
CACNA2D2	TGACACGTGTTGGTACGT	23	38	0	1	0	0
CACNA2D2	GTACACGTGTTGGTACCA	126	144	0	0	0	0
CACNA2D2	ACACACGTGTTGGTACAC	10	35	0	0	0	0
CACNA2D2	CAACACGTGTTGGTACTG	155	90	0	0	0	0
CACNA2D3	TGACACGTGTTGCACATG	111	172	1	0	0	0
CACNA2D3	ACACACGTGTTGCAACGT	220	305	0	0	0	0
CACNA2D3	TGACACGTGTTGCAACCA	170	127	0	0	0	0
CACNA2D3	CAACACGTGTTGCAACAC	91	89	0	0	0	0
CACNA2D3	GTACACGTGTTGCAACTG	748	670	2	0	0	1
CACNA2D4	ACACACGTGTGTCAGTGT	770	641	1	0	0	2
CACNA2D4	TGACACGTGTGTCAGTCA	103	149	0	0	0	0
CACNA2D4	CAACACGTGTGTCAGTAC	58	71	0	0	0	0
CACNA2D4	GTACACGTGTGTCAGTTG	251	298	1	0	0	0
CACNA2D4	TGTGGTTGACTGTGTGCA	504	171	0	0	0	0
CACYBP	CATGGTCACAACCACAGT	983	858	1	2	0	1
CACYBP	ACTGGTCACAACCACACA	68	157	0	0	0	0
CACYBP	ACTGGTCAACACGTACCA	302	377	0	0	0	0
CACYBP	GTTGGTCAACACGTACAC	125	94	0	0	0	0
CACYBP	TGTGGTCAACACGTACTG	307	430	0	1	0	0
CAD	TGACACGTACACACCACA	38	23	0	0	0	0
CAD	CAACACGTACACACCAAC	42	20	0	0	0	0
CAD	GTACACGTACACACCATG	55	80	0	0	0	0
CAD	TGACACGTACACACACGT	146	48	0	0	0	0
CAD	GTACACGTACACACACCA	114	177	0	0	0	0
CADM1	ACTGGTCACAACGTTGTG	344	448	2	0	0	0
CADM1	GTTGGTCACAACCAGTGT	337	366	0	0	0	6
CADM1	CATGGTTGCATGGTCAGT	316	93	0	1	0	0
CADM1	ACTGGTTGCATGGTCACA	149	183	0	0	0	0
CADM1	GTTGGTTGCATGGTCAAC	21	81	0	0	0	0
CADM3	CAACACTGACTGACGTGT	600	311	0	0	0	0
CADM3	ACACACTGACTGACGTCA	279	305	0	1	0	0
CADM3	GTACACTGACTGACGTAC	38	50	0	0	0	0
CADM3	TGACACTGACTGACGTTG	382	409	0	1	0	0
CADM3	ACACACTGACTGACCAGT	125	141	1	0	0	0
CADM3	GTTGCAACACGTACCAAC	8	11	0	0	0	0
CALB1	GTACACTGGTCAACCAGT	326	348	1	0	0	0
CALB1	CAACACTGGTCAACCACA	92	106	1	0	0	0
CALB1	TGACACTGGTCAACCAAC	3	17	0	0	0	0
CALB1	ACACACTGGTCAACCATG	322	294	0	0	0	0
CALB1	CAACACTGGTCAACACGT	86	43	0	0	0	0
CALB1	CATGCACAACCAGTACAC	62	22	0	0	0	0
CALCA	CAACTGTGTGACTGTGCA	80	118	0	0	0	0
CALCA	TGACTGTGTGACTGTGAC	85	81	1	0	0	6
CALCA	ACACTGTGTGACTGTGTG	827	183	1	1	0	6
CALCA	CAACTGTGTGTGGTGTGT	796	687	2	910	0	0
CALCA	CATGGTTGCACACAACAC	65	66	0	1	0	0
CALCR	TGACGTGTTGACCAGTGT	714	587	2	136	1	0
CALCR	GTACGTGTTGACGTTGCA	144	170	1	0	0	0
CALCR	ACACGTGTTGACGTTGAC	8	9	0	0	0	0
CALCR	CAACGTGTTGACGTTGTG	115	96	0	0	0	0
CALCR	GTACGTGTTGACCAGTCA	356	368	1	0	0	0
CALCRL	ACACGTGTTGACCAGTAC	28	18	0	0	0	0
CALCRL	CAACGTGTTGACCAGTTG	567	746	2	0	0	0
CALCRL	GTACGTGTTGACCACAGT	492	115	1	2	0	0
CALCRL	CAACGTGTTGACCACACA	83	41	0	0	0	0
CALCRL	TGACGTGTTGACCACAAC	46	38	0	0	0	0
CALM1	TGACCAACACACTGACTG	1221	798	0	0	0	3
CALM1	ACACCAACACACTGTGGT	499	172	0	0	0	0
CALM1	TGACCAACACACTGTGCA	312	187	2	0	0	0
CALM1	CAACCAACACACTGTGAC	98	92	0	0	0	0
CALM1	GTACCAACACACTGTGTG	128	145	2	0	0	0
CALM2	CAACGTTGTGACCAACCA	466	286	0	0	0	0
CALM2	TGACGTTGTGACCAACAC	19	23	0	0	0	0
CALM2	ACACGTTGTGACCAACTG	13	64	0	0	0	0
CALM2	GTACACACTGCACAGTAC	12	13	0	0	0	0
CALM2	TGACACACTGCACAGTTG	284	365	1	0	0	0
CALM2	TGTGCAACCATGGTCAGT	144	183	1	0	0	0
CALM3	ACACGTTGTGACTGCACA	442	472	1	0	0	0
CALM3	GTACGTTGTGACTGCAAC	50	97	0	0	0	0
CALM3	CAACCAACTGCACACATG	589	204	13	1	0	3
CALM3	GTACCAACTGCACAACGT	59	50	0	1	0	2
CALM3	CAACCAACTGCACAACCA	221	407	1	0	0	231
CALML3	ACACACTGGTCAACACCA	109	160	0	0	0	0

CALML3	GTACACTGGTCAACACAC	210	126	0	0	0	0
CALML3	TGACACTGGTCAACACTG	143	95	0	0	0	0
CALML3	ACACACTGGTCAACTGGT	709	400	3	0	0	0
CALML3	ACTGCAGTTGTGACCATG	171	190	0	0	0	0
CALML5	TGACACTGGTTGGTTGGT	598	413	0	2	0	0
CALML5	GTACACTGGTTGGTTGCA	226	290	0	0	0	6
CALML5	ACACACTGGTTGGTTGAC	53	33	0	0	0	1
CALML5	CATGCAGTTGCAGTACCA	784	575	2	6	0	22
CALML5	TGTGCAGTTGCAGTACAC	34	39	0	0	0	0
CALML6	TGACACTGCAGTCACAGT	139	125	3	0	0	0
CALML6	GTACACTGCAGTCACACA	398	388	0	1	0	0
CALML6	ACACACTGCAGTCACAAC	17	9	0	0	0	0
CALML6	CAACACTGCAGTCACATG	417	626	1	1	0	0
CALML6	GTTGCAGTTGCATGACAC	37	20	0	0	0	8
CALR	CAACGTTGGTACCATGTG	587	111	0	0	0	0
CALR	TGACGTTGGTACACGTGT	129	190	1	0	0	0
CALR	GTACGTTGGTACACGTCA	153	155	0	1	0	0
CALR	ACACGTTGGTACACGTAC	97	167	0	0	0	0
CALR	CAACGTTGGTACACGTTG	89	179	0	0	0	0
CALR	CATGCACAACCAACGTAC	132	165	0	67	0	0
CAMK1	GTTGCAACACGTTGTGTG	425	661	0	1	1	0
CAMK1	GTCATGCAACACACGTCA	238	280	0	0	0	0
CAMK1	ACCATGCAACACACGTAC	18	29	0	0	0	0
CAMK1	CACATGCAACACACGTTG	753	851	3	1	0	1
CAMK1	GTCATGCAACACACCAGT	80	44	0	0	0	0
CAMK1	CACATGCAACACACCACA	18	46	0	0	0	0
CAMK1G	ACCATGACGTGTGTGTGT	485	694	1	0	1	2
CAMK1G	GTCATGCATGTGTGTGAC	40	12	0	0	0	0
CAMK1G	TGCATGCATGTGTGTGTG	113	143	0	0	0	0
CAMK1G	TGCATGACGTGTGTGTCA	129	133	1	0	0	0
CAMK1G	CACATGACGTGTGTGTAC	34	29	0	0	0	0
CAMK2A	GTTGGTACTGTGCAACAC	53	195	345	2	0	0
CAMK2A	TGTGGTACTGTGCAACTG	391	399	0	0	0	0
CAMK2A	ACTGGTACTGTGCATGGT	332	414	1	0	0	0
CAMK2A	TGTGGTACTGTGCATGCA	180	192	0	0	0	0
CAMK2A	CATGGTACTGTGCATGAC	34	38	0	0	0	0
CAMK2A	CATGCAACTGACTGTGAC	64	39	0	0	0	0
CAMK2B	GTCATGCACATGTGACCA	308	288	0	1	1	1
CAMK2B	CACATGCACATGTGCAAC	10	14	0	0	0	0
CAMK2B	GTCATGCACATGTGCATG	137	92	1	0	0	3
CAMK2B	TGCATGCACATGTGACGT	108	91	0	0	0	0
CAMK2B	ACTGGTTGACACCAACGT	214	167	3	0	0	55
CAMK2B	GTTGCAACTGTGGTCAGT	645	345	2	0	0	0
CAMK2D	ACCATGCACATGTGACAC	73	80	0	0	0	0
CAMK2D	CACATGCACATGTGACTG	564	430	0	0	0	0
CAMK2D	GTCATGCACATGTGTGGT	193	159	0	0	0	0
CAMK2D	CACATGCACATGTGTGCA	185	258	0	0	0	0
CAMK2D	TGCATGCACATGTGTGAC	251	113	0	0	0	0
CAMK2D	TGTGCAACTGTGGTTGCA	69	114	0	1	0	0
CAMK2G	ACCATGCACATGTGTGTG	278	216	0	0	0	2
CAMK2G	GTCATGCAACGTGTGTGT	495	510	1	0	0	0
CAMK2G	CACATGCAACGTGTGTCA	134	248	1	0	0	0
CAMK2G	TGCATGCAACGTGTGTAC	51	89	0	0	0	0
CAMK2G	ACCATGCAACGTGTGTTG	371	320	0	0	0	1
CAMK2G	CATGCAACTGTGGTTGAC	264	247	0	0	0	0
CAMK4	CACATGCAACCACAACCA	204	252	0	0	1	0
CAMK4	GTCATGCAACCACACACA	285	96	0	0	0	0
CAMK4	ACCATGCAACCACACAAC	15	25	0	0	0	0
CAMK4	CACATGCAACCACACATG	116	214	1	0	0	0
CAMK4	GTCATGCAACCACAACGT	199	195	0	0	0	0
CAMK4	ACTGCATGGTGTGTCACA	46	54	0	0	0	0
CAMKK1	CACATGACGTTGGTCAGT	344	97	0	0	1	0
CAMKK1	ACCATGACGTTGGTCACA	288	65	98	1	0	0
CAMKK1	GTCATGACGTTGGTCAAC	30	26	0	1	0	0
CAMKK1	TGCATGACGTTGGTCATG	163	530	0	41	0	1
CAMKK1	ACCATGACGTTGGTACGT	227	194	0	0	0	0
CAMKK2	GTCATGACCACAGTGTTG	427	493	1	0	0	1
CAMKK2	TGCATGACCACAGTCAGT	76	117	0	0	0	0
CAMKK2	GTCATGACCACAGTCACA	22	38	0	0	0	0
CAMKK2	ACTGGTTGGTGTACGTAC	31	28	0	0	0	0
CAMKK2	CATGGTTGGTGTACGTTG	57	52	0	0	0	0
CAMKK2	CATGCAACTGTGTGCAGT	340	192	0	0	0	0
CAMP	TGTGGTCAGTCACATGAC	48	65	0	0	0	0
CAMP	ACTGGTCAGTCACATGTG	39	78	0	0	0	0
CAMP	GTTGGTCAGTCAACGTGT	401	331	1	0	0	116

CAMP	CATGGTCAGTCAACGTCA	1081	209	1	0	0	0
CAMP	TGTGGTCAGTCAACGTAC	26	49	0	0	0	0
CAND1	CACATGACTGGTACGTAC	78	72	0	0	0	0
CAND1	GTCATGACTGGTACGTTG	1330	1423	1	1	0	0
CAND1	TGCATGACTGGTACCAGT	187	141	8	3	0	2
CAND1	GTCATGACTGGTACCACA	20	23	0	0	0	0
CAND1	ACCATGACTGGTACCAAC	60	122	0	0	0	0
CAND1	TGTGCAACACGTACGTAC	90	148	0	0	0	0
CANT1	CAACACACACCATGCAGT	712	698	0	0	1	9
CANT1	ACACACACACCATGCACA	309	333	0	0	1	0
CANT1	ACACACACACCATGGTTG	231	296	0	0	0	0
CANT1	ACTGCAGTTGCAACCACA	26	36	0	0	0	0
CANT1	GTTGCAGTTGCAACCAAC	41	32	0	0	0	1
CANT1	CATGCATGCAGTCATGTG	143	127	0	0	0	0
CANX	CAACCAGTACACTGGTGT	184	112	0	0	0	1
CANX	ACACCAGTACACTGGTCA	348	228	0	0	0	0
CANX	GTACCAGTACACTGGTAC	77	50	0	0	0	0
CANX	TGACCAGTACACTGGTTG	406	449	1	0	0	0
CANX	ACACCAGTACACTGCAGT	254	465	0	0	0	3
CAP1	ACACCAGTACACACACTG	526	519	0	0	0	0
CAP1	CAACCAGTACACACTGGT	191	41	1	0	0	0
CAP1	ACACCAGTACACACTGCA	152	171	0	0	0	0
CAP1	GTACCAGTACACACTGAC	12	37	0	0	0	0
CAP1	TGACCAGTACACACTGTG	206	327	0	0	0	0
CAP1	TGTGCACATGCATGGTAC	113	105	0	0	0	0
CAPN1	ACCATGACTGCATGCACA	244	230	1	0	2	0
CAPN1	ACCATGACTGCATGGTTG	240	128	0	0	0	1
CAPN1	CACATGACTGCATGCAGT	211	230	2	0	0	0
CAPN1	GTCATGACTGCATGCAAC	97	20	0	0	0	1
CAPN1	TGCATGACTGCATGCATG	48	62	0	0	0	0
CAPN1	CATGCACATGACACTGAC	13	4	0	0	0	0
CAPN10	CAACACACACACGTGTGT	567	174	0	0	1	1
CAPN10	CAACACACACCATGTGTG	156	149	0	0	0	0
CAPN10	ACACACACACACGTGTCA	151	115	1	0	0	25
CAPN10	GTACACACACACGTGTAC	22	36	0	0	0	0
CAPN10	GTTGCAGTGTCAACACGT	18	12	0	0	0	0
CAPN11	ACACACTGGTACCATGCA	532	252	0	0	0	1
CAPN11	GTACACTGGTACCATGAC	63	80	1	0	0	0
CAPN11	TGACACTGGTACCATGTG	136	217	0	0	0	0
CAPN11	CAACACTGGTACACGTGT	389	165	1	0	0	0
CAPN11	ACACACTGGTACACGTCA	76	206	1	1	0	0
CAPN2	TGCATGACTGCAACCACA	39	37	0	0	0	0
CAPN2	CACATGACTGCAACCAAC	43	53	0	1	0	0
CAPN2	GTCATGACTGCAACCATG	25	14	0	0	0	0
CAPN2	TGCATGACTGCAACACGT	33	36	0	0	0	0
CAPN2	GTCATGACTGCAACACCA	707	814	2	0	0	21
CAPN2	GTTGCACATGACACACAC	30	39	0	0	0	0
CAPN3	GTCATGACTGCAGTGTCA	383	287	5	1	2	3
CAPN3	CATGCATGGTGTCAGTTG	1275	1711	53	0	1	3
CAPN3	ACCATGACTGGTTGTGTG	22	26	0	0	0	0
CAPN3	TGCATGACTGCAGTGTGT	548	1057	2	0	0	0
CAPN3	GTTGCATGGTGTCACAGT	69	156	0	0	0	0
CAPN3	CATGCATGGTGTCACACA	90	206	0	0	0	1
CAPN5	TGCATGTGACCAGTGTTG	1222	1632	2	1	0	3
CAPN5	ACCATGTGACCAGTCAGT	75	85	0	0	0	0
CAPN5	TGCATGTGACCAGTCACA	45	26	0	0	0	0
CAPN5	CACATGTGACCAGTCAAC	13	27	0	0	0	1
CAPN5	GTCATGTGACCAGTCATG	26	56	0	1	0	0
CAPN7	TGACCAGTTGTGGTCATG	172	211	1	0	0	0
CAPN7	ACACCAGTTGTGGTACGT	244	230	0	0	0	0
CAPN7	ACACTGTGGTCAACGTGT	382	498	0	0	0	124
CAPN7	TGACTGTGGTCAACGTCA	75	104	0	0	0	1
CAPN7	CAACTGTGGTCAACGTAC	71	105	0	0	0	0
CAPN9	TGACACACCACAACACTG	1496	1298	6	40	9	0
CAPN9	ACACACACCACAACACCA	445	472	0	0	0	3
CAPN9	GTACACACCACAACACAC	45	40	0	0	0	0
CAPN9	ACACACACCACAACTGGT	328	353	0	0	0	0
CAPN9	TGACACACCACAACTGCA	238	238	0	0	0	11
CAPN9	ACTGCACAACTGACCAGT	47	81	0	2	0	0
CAPNS1	CACATGACTGCAACACTG	310	414	12	11	2	14
CAPNS1	ACCATGACTGCAACACAC	18	65	2	1	0	0
CAPNS1	GTCATGACTGCAACTGGT	156	268	0	0	0	0
CAPNS1	CACATGACTGCAACTGCA	441	80	0	0	0	0
CAPNS1	TGCATGACTGCAACTGAC	22	12	0	0	0	0
CAPNS2	GTACACTGCAACACACGT	97	71	0	0	0	0

CAPNS2	CAACACTGCAACACACCA	285	132	0	1	0	0
CAPNS2	TGACACTGCAACACACAC	28	39	0	0	0	0
CAPNS2	ACACACTGCAACACACTG	211	222	0	0	0	0
CAPNS2	CAACACTGCAACACTGGT	104	55	0	0	0	0
CAPNS2	TGTGCAACGTGTACACGT	27	4	0	0	0	0
CAPRIN2	GTTGGTACCACATGTGGT	372	383	3	0	1	0
CAPRIN2	CATGGTACCACATGACTG	331	520	2	0	0	1
CAPRIN2	CATGGTACCACATGTGCA	148	299	1	0	0	0
CAPRIN2	TGTGGTACCACATGTGAC	250	135	0	1	0	1
CAPRIN2	CATGACGTCATGACGTGT	416	532	0	0	0	0
CARD10	GTTGGTGTCATGGTACCA	153	153	0	0	0	0
CARD10	ACTGGTGTCATGGTACAC	75	35	0	0	0	1
CARD10	CATGGTGTCATGGTACTG	860	369	0	0	0	1
CARD10	GTTGGTGTCATGGTTGGT	130	178	0	1	0	0
CARD10	GTTGCACAGTGTCACATG	60	100	0	0	0	0
CARD11	TGTGGTGTCAACTGGTTG	2146	1373	1	2	3	15
CARD11	TGTGGTGTCAACTGCACA	64	73	3	0	1	0
CARD11	ACTGGTGTCAACTGCAGT	90	90	0	0	0	1
CARD11	CATGGTGTCAACTGCAAC	30	46	0	0	0	0
CARD11	GTTGGTGTCAACTGCATG	94	130	1	0	0	0
CARD11	ACTGCAACGTACCACATG	98	132	0	0	0	0
CARD14	ACTGCAACGTACCATGGT	833	501	1	0	1	1
CARD14	CATGCAACGTACCAACGT	162	101	0	0	0	3
CARD14	ACTGCAACGTACCAACCA	84	71	0	0	0	0
CARD14	GTTGCAACGTACCAACAC	23	11	0	0	0	0
CARD14	TGTGCAACGTACCAACTG	142	190	0	1	0	2
CARD14	TGTGCAACGTACCATGCA	116	166	0	0	0	0
CARD6	ACTGGTCAGTCAGTCAAC	3	139	0	0	0	0
CARD6	CATGGTCAGTCAGTCATG	157	139	0	0	0	0
CARD6	GTTGGTCAGTCAGTACGT	693	1102	0	1	0	8
CARD6	CATGGTCAGTCAGTACCA	178	354	0	0	0	0
CARD6	TGTGGTCAGTCAGTACAC	37	45	0	1	0	0
CARD6	CATGCAACGTACACGTAC	39	22	0	0	0	0
CARD9	CATGGTGTCATGGTGTGT	412	383	0	0	0	0
CARD9	ACTGGTGTCATGGTGTCA	574	434	0	0	0	0
CARD9	GTTGGTGTCATGGTGTAC	126	112	0	0	0	1
CARD9	TGTGGTGTCATGGTGTTG	1119	672	0	0	0	0
CARD9	ACTGGTGTCATGGTCAGT	155	126	0	0	0	0
CARM1	TGACGTGTCAACACACGT	17	13	0	0	0	0
CARM1	GTACGTGTCAACACACCA	91	77	0	0	0	0
CARM1	ACACGTGTCAACACACAC	98	121	0	0	0	0
CARM1	CAACGTGTCAACACACTG	151	196	0	0	0	0
CARM1	ACTGCAGTCAACTGGTTG	241	561	0	0	0	1
CARS	TGACACGTACGTTGACTG	252	425	0	0	1	0
CARS	CAACACGTACGTTGACGT	226	268	0	0	0	0
CARS	ACACACGTACGTTGACCA	111	152	0	0	0	17
CARS	GTACACGTACGTTGACAC	15	21	0	0	0	0
CARS	ACACACGTACGTTGTGGT	108	82	0	0	0	0
CARS2	CATGGTTGGTACGTACCA	121	130	1	0	1	0
CARS2	ACTGGTCATGCAACGTGT	263	420	0	0	0	1
CARS2	TGTGGTCATGCAACGTCA	336	354	0	1	0	0
CARS2	CATGGTCATGCAACGTAC	102	65	0	0	0	0
CARS2	GTTGGTCATGCAACGTTG	113	76	0	0	0	0
CARS2	CATGCACATGTGCATGCA	66	102	0	0	0	0
CARTPT	ACTGGTCAGTCATGGTAC	172	137	0	0	0	1
CARTPT	CATGGTCAGTCATGGTTG	519	698	1	0	0	0
CARTPT	GTTGGTCAGTCATGCAGT	175	292	2	0	0	1
CARTPT	CATGGTCAGTCATGCACA	422	271	1	0	0	1
CARTPT	TGTGGTCAGTCATGCAAC	14	18	0	0	0	0
CASK	ACCATGCAACACTGGTGT	393	393	1	0	1	0
CASK	TGCATGCAACACTGGTCA	102	132	90771	95	1	10
CASK	CACATGCAACACTGGTAC	292	141	0	0	0	0
CASK	GTCATGCAACACTGGTTG	421	479	0	0	0	0
CASK	TGCATGCAACACTGCAGT	250	166	4	0	0	0
CASK	CATGCACAGTCATGCATG	84	46	0	0	0	0
CASP1	ACCATGACTGCAGTCATG	46	59	0	0	0	0
CASP1	CACATGACTGCAGTACGT	231	247	0	0	0	0
CASP1	ACCATGACTGCAGTACCA	197	257	0	0	0	0
CASP1	CATGGTCAGTGTTGTGTG	282	254	0	0	0	0
CASP1	ACTGGTCAGTCAGTGTGT	176	546	1	2	0	0
CASP10	GTCATGACTGACGTACCA	116	162	19372	18	1	2
CASP10	TGCATGACTGACGTACGT	125	205	0	0	0	0
CASP10	ACCATGACTGACGTACAC	28	26	0	0	0	0
CASP10	CACATGACTGACGTACTG	293	370	0	0	0	4
CASP10	GTTGGTTGGTACACTGCA	125	159	0	0	0	0

CASP12	GTTGACACGTGTCACATG	124	70	0	0	0	0
CASP12	TGTGACACGTGTCAACGT	815	337	0	0	0	0
CASP12	GTTGACACGTGTCAACCA	4	6	0	0	0	0
CASP12	ACTGACACGTGTCAACAC	42	46	0	0	0	0
CASP12	CATGACACGTGTCAACTG	517	316	0	0	0	0
CASP2	GTCATGACTGCAGTACAC	37	37	0	0	0	0
CASP2	TGCATGACTGCAGTACTG	170	541	1	0	0	0
CASP2	CATGCAGTGTTGACCAAC	28	12	0	0	0	0
CASP2	GTTGCAGTGTTGACCATG	131	131	0	0	0	0
CASP2	GTTGCATGTGCAACACAC	2	3	0	0	0	0
CASP3	ACCATGACTGCAACTGTG	342	312	1	0	1	0
CASP3	CATGCAGTCAGTGTGTAC	75	102	0	0	1	0
CASP3	GTCATGACTGCATGGTGT	241	218	0	0	0	0
CASP3	CACATGACTGCATGGTCA	153	101	1	0	0	0
CASP3	TGCATGACTGCATGGTAC	114	100	0	0	0	0
CASP4	GTCATGACTGCAGTTGTG	821	874	1	2	3	3
CASP4	ACCATGACTGCAGTTGGT	74	116	0	0	0	0
CASP4	TGCATGACTGCAGTTGCA	53	92	0	0	0	3
CASP4	CACATGACTGCAGTTGAC	44	84	0	0	0	0
CASP4	ACCATGACTGCACAGTGT	532	493	0	0	0	0
CASP6	TGCATGACTGCACAGTCA	131	164	0	0	0	0
CASP6	CACATGACTGCACAGTAC	82	114	2	0	0	0
CASP6	GTCATGACTGCACAGTTG	207	272	1	0	0	0
CASP6	TGCATGACTGCACACAGT	180	179	0	0	0	0
CASP6	GTTGACCACAACCATGAC	2	9	0	0	0	0
CASP7	GTCATGACTGCACACACA	28	17	0	0	0	0
CASP7	ACCATGACTGCACACAAC	4	30	0	1	0	0
CASP7	CACATGACTGCACACATG	93	94	0	0	0	1
CASP7	GTCATGACTGCACAACGT	633	694	0	1	0	2
CASP7	CACATGACTGCACAACCA	273	258	0	0	0	1
CASP8	CACATGACTGCATGTGTG	280	289	1	0	0	0
CASP8	CACATGACTGACGTGTGT	413	747	0	0	0	0
CASP8	ACCATGACTGACGTGTCA	190	173	0	1	0	0
CASP8	GTCATGACTGACGTGTAC	173	47	0	0	0	0
CASP8	TGCATGACTGACGTGTTG	219	149	0	0	0	0
CASP8AP2	CAACTGCATGCAGTCAGT	136	160	0	0	0	0
CASP8AP2	ACACTGCATGCAGTCACA	164	117	1	0	0	0
CASP8AP2	GTACTGCATGCAGTCAAC	13	13	0	0	0	0
CASP8AP2	TGACTGCATGCAGTCATG	29	31	0	0	0	0
CASP8AP2	ACACTGCATGCAGTACGT	115	133	0	0	0	0
CASP8AP2	GTTGCAACGTACGTTGCA	33	35	1	0	0	0
CASP9	CATGCAACGTCACAGTGT	2141	1311	1	1	2	2
CASP9	ACCATGACTGACGTCAGT	292	217	0	0	0	1
CASP9	TGCATGACTGACGTCACA	79	167	1	0	0	0
CASP9	CACATGACTGACGTCAAC	35	17	0	0	0	0
CASP9	GTCATGACTGACGTCATG	103	32	4	0	0	0
CASP9	TGTGCAACGTCAGTTGTG	959	679	5	1	0	0
CASR	ACACGTGTTGACCACATG	208	234	3	991	0	1
CASR	CAACGTGTTGACCAACGT	14	67	0	0	0	0
CASR	ACACGTGTTGACCAACCA	633	397	0	0	0	0
CASR	GTACGTGTTGACCAACAC	27	20	0	0	0	0
CASR	TGACGTGTTGACCAACTG	245	185	0	0	0	0
CAST	TGACTGTGGTCAGTCATG	44	89	0	0	0	0
CAST	ACACTGTGGTCAGTACGT	99	165	0	0	0	0
CAST	TGACTGTGGTCAGTACCA	167	179	1	0	0	2
CAST	CAACTGTGGTCAGTACAC	0	11	0	0	0	0
CAST	TGTGCATGGTGTCACAAC	27	23	0	0	0	0
CAST	ACTGCATGGTGTCACATG	81	74	0	1	0	0
CAT	GTACTGCATGCAGTGTGT	399	288	0	0	0	0
CAT	CAACTGCATGCAGTGTCA	513	745	3	1	0	0
CAT	TGACTGCATGCAGTGTAC	5	19	0	0	0	0
CAT	ACACTGCATGCAGTGTTG	224	165	0	0	0	0
CAT	GTTGCAGTTGTGGTGTAC	188	212	0	0	0	0
CAV1	GTACGTGTTGGTCAGTTG	492	313	0	0	0	0
CAV1	TGACGTGTTGGTCACAGT	202	178	0	1	0	0
CAV1	GTACGTGTTGGTCACACA	292	133	0	1	0	0
CAV1	ACACGTGTTGGTCACAAC	50	38	0	0	0	0
CAV1	ACTGGTGTACGTTGACTG	360	18	1	0	0	0
CAV1	TGTGCAACGTCATGGTGT	106	40	0	0	0	0
CAV2	CATGGTCACAGTTGCAAC	45	31	2	0	0	0
CAV2	GTTGGTCACAGTTGCATG	372	327	1	0	0	0
CAV2	TGTGGTCACAGTTGACGT	87	141	0	1	0	0
CAV2	GTTGGTCACAGTTGACCA	187	281	0	2	0	5
CAV2	ACTGGTCACAGTTGACAC	27	33	0	0	0	0
CAV3	ACACTGTGTGGTGTCATG	31	43	0	0	0	0

CAV3	CAACTGTGTGGTGTACGT	252	450	0	1	0	0
CAV3	ACACTGTGTGGTGTACCA	274	219	0	0	0	0
CAV3	GTACTGTGTGGTGTACAC	39	45	0	0	0	0
CAV3	TGACTGTGTGGTGTACTG	532	592	0	2	0	1
CAV3	GTTGCAACGTCATGGTCA	10	51	0	0	0	0
CBL	CAACCATGCAGTCAACGT	765	726	1	0	1	0
CBL	CAACCATGCAGTCACACA	370	229	0	1	0	1
CBL	TGACCATGCAGTCACAAC	15	28	0	0	0	0
CBL	ACACCATGCAGTCACATG	286	445	0	0	0	2
CBL	ACACCATGCAGTCAACCA	705	627	0	0	0	0
CBLB	ACACGTGTACACACACCA	265	161	0	0	0	0
CBLB	GTACGTGTACACACACAC	24	67	0	0	0	0
CBLB	TGACGTGTACACACACTG	259	312	0	0	0	0
CBLB	ACACGTGTACACACTGGT	70	115	0	0	0	0
CBLB	ACTGCAGTACCAACGTCA	86	93	0	0	0	0
CBLC	ACCATGTGCAGTGTTGAC	82	75	0	0	0	0
CBLC	CACATGTGCAGTGTTGTG	466	353	0	0	0	1
CBLC	TGCATGTGCAGTCAGTGT	1817	992	5	1	0	1
CBLC	TGTGCAGTACCAGTGTGT	563	799	0	2	0	0
CBLC	GTTGCAGTACCAGTGTCA	878	831	0	1	0	0
CBLC	GTTGCAACCAACACGTCA	261	285	314	0	0	0
CBR1	CAACACGTTGCATGACTG	691	874	1	2	1	17
CBR1	GTACACGTTGCATGTGGT	148	226	1	0	0	0
CBR1	CAACACGTTGCATGTGCA	165	223	1	2	0	0
CBR1	TGACACGTTGCATGTGAC	40	94	0	0	0	0
CBR1	ACACACGTTGCATGTGTG	77	139	0	0	0	0
CBR3	CAACACGTTGCATGCAAC	34	68	0	0	0	0
CBR3	GTACACGTTGCATGCATG	96	112	0	0	0	0
CBR3	TGACACGTTGCATGACGT	130	183	0	0	0	0
CBR3	GTACACGTTGCATGACCA	149	170	0	0	0	0
CBR3	ACACACGTTGCATGACAC	49	61	0	0	0	1
CBS	GTACACGTCACAGTGTCA	161	151	0	0	0	0
CBS	ACACACGTCACAGTGTAC	118	103	0	0	0	0
CBS	CAACACGTCACAGTGTTG	157	187	0	0	0	0
CBS	GTACACGTCACAGTCAGT	453	742	0	0	0	1
CBS	TGACTGTGCACAACGTGT	149	175	0	1	0	0
CBS	GTTGCACACACAACTGCA	50	43	0	0	0	0
CBX2	CATGCATGCACACAGTTG	751	624	1	1	1	0
CBX2	GTACGTTGCAGTACCAGT	411	261	0	0	0	0
CBX2	CAACGTTGCAGTACCACA	173	172	0	0	0	0
CBX2	TGACGTTGCAGTACCAAC	17	4	0	0	0	0
CBX2	ACACGTTGCAGTACCATG	126	423	0	1	0	0
CBX2	CAACGTTGCAGTACACGT	36	54	0	0	0	0
CBX3	ACACGTTGACTGTGGTGT	536	583	0	0	0	2
CBX3	TGACGTTGACTGTGGTCA	204	276	1	0	0	0
CBX3	CAACGTTGACTGTGGTAC	111	96	0	0	0	0
CBX3	GTACGTTGACTGTGGTTG	659	285	0	1	0	0
CBX3	TGACGTTGACTGTGCAGT	129	163	1	0	0	0
CBX3	GTTGCAACCATGGTACGT	72	205	0	0	0	0
CBX4	ACCATGTGGTTGCAACGT	124	184	0	0	0	0
CBX4	TGCATGTGGTTGCAACCA	198	229	0	0	0	0
CBX4	CACATGTGGTTGCAACAC	70	87	0	0	0	0
CBX4	TGACTGTGCACAACTGCA	197	118	0	0	0	0
CBX4	CAACTGTGCACAACTGAC	236	80	0	0	0	0
CBX5	CAACTGCATGTGCAACCA	536	546	1	0	0	0
CBX5	TGACTGCATGTGCAACAC	50	64	0	0	0	0
CBX5	ACACTGCATGTGCAACTG	510	313	1	0	0	0
CBX5	CAACTGCATGTGCATGGT	726	596	2	2	0	0
CBX5	ACTGCACAACACCAACAC	105	108	0	0	0	0
CBX5	CATGCACAACACCAACTG	820	756	0	1	0	0
CBX7	ACACGTACTGCATGTGTG	114	377	1	0	0	1
CBX7	ACACGTACTGACGTGTGT	273	487	0	19	0	1
CBX7	TGACGTACTGACGTGTCA	115	145	0	0	0	0
CBX7	CAACGTACTGACGTGTAC	21	26	0	0	0	0
CBX7	GTACGTACTGACGTGTTG	803	655	4	0	0	0
CCBP2	ACACGTGTTGACCATGGT	358	335	0	1	0	0
CCBP2	TGACGTGTTGACCATGCA	348	242	0	0	0	1
CCBP2	CAACGTGTTGACCATGAC	61	84	0	0	0	0
CCBP2	GTACGTGTTGACCATGTG	376	268	1	0	0	0
CCBP2	ACACGTGTTGACACGTGT	90	100	0	0	0	0
CCDC6	CATGGTGTGTCATGACCA	328	389	0	2	1	0
CCDC6	GTTGGTGTGTCATGCACA	61	60	0	0	0	0
CCDC6	ACTGGTGTGTCATGCAAC	22	40	1	0	0	0
CCDC6	CATGGTGTGTCATGCATG	647	224	1	0	0	0
CCDC6	GTTGGTGTGTCATGACGT	24	62	0	0	0	0

CCK	GTACTGCATGACGTGTTG	668	341	1	2	0	0
CCK	TGACTGCATGACGTCAGT	312	243	0	0	0	5
CCK	GTACTGCATGACGTCACA	90	235	0	0	0	0
CCK	ACACTGCATGACGTCAAC	90	34	0	0	0	0
CCK	ACTGCAGTTGACTGTGTG	372	440	2	1	0	0
CCKAR	TGACGTGTTGACACGTCA	22	9	0	0	0	0
CCKAR	CAACGTGTTGACACGTAC	62	78	0	0	0	1
CCKAR	GTACGTGTTGACACGTTG	140	190	0	0	0	0
CCKAR	GTTGCAGTACCACAGTTG	631	747	2	1	0	0
CCKAR	TGTGACCAGTCATGGTTG	294	421	1	2	0	0
CCKBR	CACATGTGTGGTTGTGGT	156	696	0	0	0	2
CCKBR	ACCATGTGTGGTTGTGCA	144	143	0	0	0	0
CCKBR	GTCATGTGTGGTTGTGAC	23	30	0	0	0	0
CCKBR	TGCATGTGTGGTTGTGTG	696	493	0	0	0	0
CCKBR	GTTGCATGCAACTGGTCA	73	75	0	0	0	0
CCL1	CAACTGGTGTTGTGACTG	1487	401	3	0	1	0
CCL1	GTACTGGTGTTGTGTGGT	104	127	1	0	0	0
CCL1	CAACTGGTGTTGTGTGCA	135	162	0	0	0	0
CCL1	TGACTGGTGTTGTGTGAC	32	19	0	0	0	0
CCL1	ACACTGGTGTTGTGTGTG	37	66	1	0	0	0
CCL1	CATGCACACAGTACTGCA	251	239	1	0	0	0
CCL11	CAACTGGTCAGTGTCACA	59	20	0	0	0	0
CCL11	TGACTGGTCAGTGTCAAC	5	9	0	0	0	0
CCL11	ACACTGGTCAGTGTCATG	186	206	0	0	0	0
CCL11	CAACTGGTCAGTGTACGT	470	909	2	0	0	1
CCL11	ACACTGGTCAGTGTACCA	107	161	0	0	0	1
CCL11	CATGCAACACACTGTGTG	401	811	0	0	0	1
CCL13	GTACTGGTCAGTACTGGT	463	149	0	0	0	0
CCL13	CAACTGGTCAGTACTGCA	505	820	3	1	0	0
CCL13	TGACTGGTCAGTACTGAC	35	20	0	1	0	0
CCL13	ACACTGGTCAGTACTGTG	108	72	0	0	0	0
CCL13	GTACTGGTCAGTTGGTGT	337	300	1	0	0	0
CCL13	GTTGCAACACTGGTTGCA	221	92	1	0	0	0
CCL15	TGACTGGTGTTGTGACGT	112	191	0	1	0	0
CCL15	GTACTGGTGTTGTGACCA	410	364	1	0	0	0
CCL15	ACACTGGTGTTGTGACAC	48	128	0	0	0	0
CCL15	TGACTGGTCAGTCACAGT	109	323	0	1	0	0
CCL15	GTACTGGTCAGTCACACA	93	180	0	0	0	1
CCL16	TGACTGGTCAGTACCACA	150	160	0	0	153	0
CCL16	GTACTGGTCAGTACGTAC	240	196	0	0	0	0
CCL16	TGACTGGTCAGTACGTTG	444	538	0	2	0	1
CCL16	ACACTGGTCAGTACCAGT	122	227	1	0	0	0
CCL16	CAACTGGTCAGTACCAAC	30	13	0	0	0	0
CCL16	ACTGCAACACTGGTTGAC	17	42	0	0	0	0
CCL17	TGTGCAACACTGCAGTGT	872	865	3	0	1	0
CCL17	TGACTGGTCACATGCAAC	86	98	0	0	0	0
CCL17	ACACTGGTCACATGCATG	355	392	0	1	0	1
CCL17	CAACTGGTCACATGACGT	187	278	0	0	0	0
CCL17	ACACTGGTCACATGACCA	105	93	0	0	0	0
CCL17	CATGCAACACTGGTTGTG	190	230	0	0	0	0
CCL18	ACACTGGTCAGTTGGTTG	874	577	17	1	1	6
CCL18	CAACTGGTCAGTTGGTCA	582	292	0	1	0	0
CCL18	TGACTGGTCAGTTGGTAC	82	61	0	0	0	0
CCL18	CAACTGGTCAGTTGCAGT	144	86	0	0	0	0
CCL18	ACACTGGTCAGTTGCACA	263	211	1	1	0	1
CCL18	CATGCAACACTGCACACA	239	218	0	0	0	2
CCL19	CAACTGGTCACACAACTG	342	494	1	1	1	0
CCL19	ACACTGGTCACACAACAC	169	192	0	1	0	1
CCL19	GTACTGGTCACACATGGT	147	229	0	1	0	0
CCL19	CAACTGGTCACACATGCA	273	348	0	0	0	3
CCL19	CATGCAACACACACACTG	122	169	1	0	0	0
CCL19	GTTGCAACACACACTGGT	271	477	0	0	0	0
CCL2	TGACGTGTGTCAGTGTAC	87	194	0	0	0	0
CCL2	ACACGTGTGTCAGTGTTG	68	27	0	0	0	0
CCL2	CAACGTGTGTCAGTCAGT	427	557	1	0	0	0
CCL2	ACACGTGTGTCAGTCACA	29	23	0	0	0	0
CCL2	GTACGTGTGTCAGTCAAC	25	18	0	0	0	0
CCL20	GTACTGGTCAGTTGACTG	170	122	0	1	0	0
CCL20	TGACTGGTCAGTTGTGGT	909	879	2	1	0	0
CCL20	GTACTGGTCAGTTGTGCA	253	193	0	0	0	0
CCL20	ACACTGGTCAGTTGTGAC	15	18	0	0	0	0
CCL20	CAACTGGTCAGTTGTGTG	83	276	1	0	0	1
CCL20	GTTGCACACATGCAACTG	615	960	1	3	0	1
CCL22	CAACTGGTCACACAGTGT	610	712	0	1	1	0
CCL22	ACACTGGTCACACAGTCA	255	471	0	0	0	0

CCL22	GTACTGGTCACACAGTAC	17	151	0	0	0	0
CCL22	TGACTGGTCACACAGTTG	457	380	0	2	0	0
CCL22	ACACTGGTCACACACAGT	273	260	0	0	0	1
CCL22	GTTGCAACACTGCAGTCA	448	455	4	1	0	7
CCL28	GTACTGGTCACAGTACGT	402	148	1	1	1	0
CCL28	GTACTGGTCACAGTCACA	62	153	0	323	0	0
CCL28	ACACTGGTCACAGTCAAC	13	22	0	0	0	0
CCL28	CAACTGGTCACAGTCATG	65	54	0	0	0	0
CCL28	CAACTGGTCACAGTACCA	181	152	0	0	0	3
CCL3	GTACTGGTCAGTACCATG	96	107	0	0	0	0
CCL3	TGACTGGTCAGTACACGT	35	38	0	0	0	0
CCL3	GTACTGGTCAGTACACCA	94	30	0	0	0	0
CCL3	ACACTGGTCAGTACACAC	10	24	0	0	0	0
CCL3	CAACTGGTCAGTACACTG	279	366	0	0	0	0
CCL3L1	GTACTGGTCACATGCAGT	612	698	1	0	222	0
CCL3L1	CAACTGGTCACATGGTTG	1257	1581	2	0	2	0
CCL3L1	GTACTGGTCACATGGTCA	285	540	0	0	0	3
CCL3L1	ACACTGGTCACATGGTAC	42	36	0	0	0	0
CCL3L1	CAACTGGTCACATGCACA	185	255	0	0	0	1
CCL4	ACTGCACACAGTACTGTG	314	149	0	0	1	0
CCL4	ACACTGGTCAGTCACAAC	74	30	0	0	0	0
CCL4	CAACTGGTCAGTCACATG	288	339	0	0	0	0
CCL4	GTACTGGTCAGTCAACGT	426	669	0	0	0	0
CCL4	TGACTGGTCACAGTACAC	68	106	0	0	0	0
CCL4	ACACTGGTCACAGTACTG	596	1291	1	0	0	0
CCL5	TGACTGGTCACACACACA	99	51	0	0	0	0
CCL5	CAACTGGTCACACACAAC	60	63	7	868	0	0
CCL5	GTACTGGTCACACACATG	618	645	2	1	0	4
CCL5	TGACTGGTCACACAACGT	327	325	0	1	0	0
CCL5	GTACTGGTCACACAACCA	138	372	0	0	0	0
CCL5	ACTGCAACACTGCAGTAC	21	19	0	0	0	0
CCL7	CAACTGGTCAGTCAACCA	213	171	1	0	0	0
CCL7	TGACTGGTCAGTCAACAC	27	28	0	0	0	0
CCL7	ACACTGGTCAGTCAACTG	803	739	0	0	0	2
CCL7	CAACTGGTCAGTCATGGT	88	69	0	0	0	0
CCL7	ACACTGGTCAGTCATGCA	292	534	1	0	0	0
CCL7	GTTGCACATGTGTGGTCA	334	177	1	1	0	0
CCNA1	GTACACGTGTTGACGTCA	106	111	0	0	0	0
CCNA1	ACACACGTGTTGACGTAC	77	63	0	0	0	0
CCNA1	CAACACGTGTTGACGTTG	126	144	0	0	0	0
CCNA1	GTACACGTGTTGACCAGT	87	109	0	1	0	6
CCNA1	CAACACGTGTTGACCACA	41	73	1	0	0	0
CCNA1	ACTGCAACGTACGTTGAC	28	65	0	0	0	0
CCNA2	GTACACGTCAGTCAGTCA	721	1305	2	2	1	0
CCNA2	CAACACGTCAGTCAGTTG	802	1097	44	0	1	7
CCNA2	CAACACGTCAGTGTTGTG	60	53	0	0	0	0
CCNA2	TGACACGTCAGTCAGTGT	329	296	3	0	0	0
CCNA2	ACACACGTCAGTCAGTAC	68	49	0	0	0	0
CCNA2	TGTGCAACGTACTGCACA	20	21	0	0	0	0
CCNB1	GTACACGTCAGTCACAGT	185	189	0	0	0	0
CCNB1	CAACACGTCAGTCACACA	94	85	0	0	0	0
CCNB1	TGACACGTCAGTCACAAC	52	36	0	0	0	1
CCNB1	ACACACGTCAGTCACATG	150	182	0	0	0	0
CCNB1	CAACACGTCAGTCAACGT	986	159	0	0	0	1
CCNB2	GTTGCACATGCATGGTGT	726	1070	4	0	1	0
CCNB2	TGACACGTGTTGACCAAC	47	50	0	0	0	0
CCNB2	ACACACGTGTTGACCATG	119	217	0	0	0	0
CCNB2	CAACACGTGTTGACACGT	31	19	0	0	0	0
CCNB2	ACACACGTGTTGACACCA	31	12	0	0	0	0
CCNB2	GTACACGTGTTGACACAC	39	40	0	0	0	0
CCNB3	TGACGTGTGTACCAGTTG	75	97	0	0	0	0
CCNB3	ACACGTGTGTACCACAGT	1	6	0	0	0	0
CCNB3	TGACGTGTGTACCACACA	19	26	4	0	0	0
CCNB3	CAACGTGTGTACCACAAC	6	11	0	0	0	0
CCNB3	TGTGCAGTCATGACACAC	7	12	0	0	0	0
CCNC	ACACGTTGGTTGACCACA	30	27	0	0	0	0
CCNC	GTACGTTGGTTGACCAAC	80	39	0	0	0	0
CCNC	TGACGTTGGTTGACCATG	139	157	0	0	0	0
CCNC	ACACGTTGGTTGACACGT	157	204	0	0	0	0
CCNC	GTACTGTGCACAACTGTG	138	253	3	1	0	0
CCND1	CAACCATGCATGGTACCA	353	179	0	0	1	0
CCND1	ACACCATGCATGGTACTG	612	810	1	1	1	1
CCND1	GTACGTTGTGACTGACTG	533	314	29	1	0	6
CCND1	GTACCATGCATGGTACGT	112	78	0	0	0	0
CCND1	TGACCATGCATGGTACAC	306	109	0	0	0	0

CCND2	TGACACGTCAGTCATGCA	380	502	0	0	1	0
CCND2	ACACACGTCAGTCAACCA	543	448	2	1	0	0
CCND2	GTACACGTCAGTCAACAC	149	111	1	0	0	0
CCND2	TGACACGTCAGTCAACTG	123	72	0	0	0	0
CCND2	ACACACGTCAGTCATGGT	201	325	2	0	0	0
CCND2	CATGCAACGTACTGCAAC	30	23	0	0	0	0
CCND3	ACTGCAGTACACGTTGTG	414	475	0	1	2	0
CCND3	GTCATGTGGTGTTGGTGT	623	292	0	0	1	0
CCND3	ACCATGTGGTGTACTGTG	41	63	0	0	0	0
CCND3	CACATGTGGTGTTGGTCA	188	177	0	0	0	3
CCND3	TGCATGTGGTGTTGGTAC	65	208	0	1	0	0
CCND3	GTTGCAACGTACTGCATG	153	176	2	0	0	1
CCNE1	TGACACGTCAGTACGTCA	68	90	0	0	1	0
CCNE1	CAACACGTCAGTCATGAC	22	28	0	0	0	0
CCNE1	GTACACGTCAGTCATGTG	671	496	0	0	0	2
CCNE1	ACACACGTCAGTACGTGT	444	201	0	0	0	0
CCNE1	TGTGCAGTTGGTGTACTG	186	226	0	0	0	0
CCNE1	ACTGCAACGTTGGTACAC	11	15	0	0	0	0
CCNE2	TGACACGTGTTGACACTG	20	45	0	0	0	0
CCNE2	ACACACGTGTTGACTGGT	752	694	2	0	0	2
CCNE2	TGACACGTGTTGACTGCA	291	389	0	0	0	2
CCNE2	CAACACGTGTTGACTGAC	34	51	0	0	0	0
CCNE2	CATGCAACGTTGGTACTG	270	282	1	0	0	46
CCNE2	GTTGCAACGTTGGTTGGT	130	190	0	0	0	0
CCNG1	GTACACGTCAGTACGTTG	703	801	357410	381	24	41
CCNG1	CAACACGTCAGTACGTAC	43	21	0	0	0	0
CCNG1	TGACACGTCAGTACCAGT	72	51	0	0	0	0
CCNG1	GTACACGTCAGTACCACA	31	41	0	0	0	0
CCNG1	ACACACGTCAGTACCAAC	39	19	0	0	0	0
CCNG2	CAACACGTCAGTACCATG	101	62	141	1	0	0
CCNG2	GTACACGTCAGTACACGT	64	54	0	0	0	0
CCNG2	CAACACGTCAGTACACCA	334	284	1	0	0	1
CCNG2	TGACACGTCAGTACACAC	31	20	0	0	0	0
CCNG2	ACACACGTCAGTACACTG	466	530	0	0	0	0
CCNG2	TGTGCACACAACTGTGAC	87	79	0	0	0	0
CCNH	ACACGTTGGTTGTGCATG	214	267	0	0	1	0
CCNH	GTACGTTGGTTGTGCAGT	57	50	0	0	0	0
CCNH	CAACGTTGGTTGTGCACA	41	84	0	0	0	0
CCNH	TGACGTTGGTTGTGCAAC	20	14	0	0	0	0
CCNH	CAACGTTGGTTGTGACGT	189	161	1	0	0	28
CCNH	ACTGCAACGTTGCAGTTG	120	86	0	0	0	0
CCNI	GTACACGTGTTGTGGTTG	1179	785	0	82	9	1
CCNI	ACACACGTGTTGTGGTGT	416	435	0	1	1	0
CCNI	GTACACGTGTTGACTGTG	302	193	0	0	0	0
CCNI	TGACACGTGTTGTGGTCA	249	396	1	0	0	0
CCNI	CAACACGTGTTGTGGTAC	107	133	1	0	0	0
CCNI	CATGCAACGTTGCACAGT	95	102	0	0	0	0
CCNK	TGACACGTGTTGCATGGT	510	532	1	1	1	0
CCNK	TGACACGTGTTGACGTGT	748	810	0	0	1	0
CCNK	GTACACGTGTTGCATGCA	173	161	0	0	0	2
CCNK	ACACACGTGTTGCATGAC	22	35	0	0	0	0
CCNK	CAACACGTGTTGCATGTG	72	60	0	0	0	0
CCNT2	TGACGTCATGGTACACAC	67	91	1	29	1	0
CCNT2	ACACGTCATGGTACACTG	271	245	0	0	1	0
CCNT2	CAACGTCATGGTACCATG	67	46	0	0	0	0
CCNT2	GTACGTCATGGTACACGT	74	76	0	0	0	0
CCNT2	CAACGTCATGGTACACCA	20	10	0	0	0	0
CCNT2	CATGCAACGTTGCAACAC	54	55	0	0	0	0
CCR1	TGACGTGTTGACACCAGT	101	124	0	0	0	0
CCR1	GTACGTGTTGACACCACA	96	68	0	0	0	0
CCR1	ACACGTGTTGACACCAAC	38	73	0	0	0	0
CCR1	CAACGTGTTGACACCATG	109	61	1	0	0	6
CCR1	GTACGTGTTGACACACGT	78	59	0	0	0	0
CCR10	TGACGTCACAGTACGTTG	250	352	0	0	1	0
CCR10	CAACGTCACAGTACGTGT	428	294	1	0	0	0
CCR10	ACACGTCACAGTACGTCA	181	172	0	0	0	0
CCR10	GTACGTCACAGTACGTAC	12	153	0	1	0	0
CCR10	ACACGTCACAGTACCAGT	234	187	0	0	0	0
CCR10	GTTGCACAACTGACGTAC	201	21	0	0	0	0
CCR2	ACCATGACGTCACATGAC	48	84	0	0	0	0
CCR2	CACATGACGTCACATGTG	307	265	0	0	0	0
CCR2	TGCATGACGTCAACGTGT	487	198	1	0	0	0
CCR2	GTCATGACGTCAACGTCA	225	266	0	0	0	0
CCR2	ACCATGACGTCAACGTAC	33	33	1	0	0	0
CCR2	GTTGCAACGTCATGCAGT	102	108	0	0	0	0

CCR3	CAACGTGTTGACACACCA	207	287	0	0	0	0
CCR3	TGACGTGTTGACACACAC	31	83	0	0	0	0
CCR3	ACACGTGTTGACACACTG	728	521	1	1	0	2
CCR3	CAACGTGTTGACACTGGT	553	355	0	1	0	0
CCR3	ACACGTGTTGACACTGCA	572	213	1	1	0	1
CCR3	CATGCATGGTTGGTGTAC	204	28	0	0	0	0
CCR4	CAACGTGTTGACTGGTGT	576	855	24	11968	5	1
CCR4	GTACGTGTTGACACTGAC	350	45	0	1	0	0
CCR4	TGACGTGTTGACACTGTG	177	249	1	1	0	0
CCR4	ACACGTGTTGACTGGTCA	643	383	0	0	0	1
CCR4	GTACGTGTTGACTGGTAC	147	108	0	0	0	0
CCR5	TGACGTGTTGACTGGTTG	213	232	1	0	0	1
CCR5	ACACGTGTTGACTGCAGT	144	229	1	0	0	0
CCR5	TGACGTGTTGACTGCACA	84	82	0	0	0	0
CCR5	CAACGTGTTGACTGCAAC	20	15	1	0	0	0
CCR5	GTACGTGTTGACTGCATG	197	180	0	0	0	0
CCR6	TGACGTGTTGACTGACGT	107	96	0	0	0	0
CCR6	GTACGTGTTGACTGACCA	338	259	0	1	0	1
CCR6	ACACGTGTTGACTGACAC	41	121	0	0	0	0
CCR6	CAACGTGTTGACTGACTG	75	245	0	0	0	0
CCR6	GTACGTGTTGACTGTGGT	246	269	1	0	0	0
CCR7	CATGCATGGTTGGTCATG	420	370	1	1	2	1
CCR7	CAACGTGTTGACTGTGCA	255	282	67	0	0	1
CCR7	TGACGTGTTGACTGTGAC	91	180	0	0	0	0
CCR7	ACACGTGTTGACTGTGTG	191	232	0	0	0	0
CCR7	CAACGTGTTGTGGTGTGT	899	801	2	0	0	1
CCR7	ACACGTGTTGTGGTGTCA	178	211	0	1	0	0
CCR8	GTACGTGTTGTGGTGTAC	24	30	0	0	0	0
CCR8	TGACGTGTTGTGGTGTTG	58	93	0	0	0	0
CCR8	ACACGTGTTGTGGTCAGT	132	155	2	0	0	0
CCR8	TGACGTGTTGTGGTCACA	154	102	0	0	0	0
CCR8	CAACGTGTTGTGGTCAAC	27	29	0	0	0	0
CCR8	GTTGCAACGTGTCACACA	79	309	0	0	0	0
CCS	GTACACCAACACCAGTCA	394	707	0	2	1	0
CCS	ACACACCAACACGTTGAC	96	178	2	0	0	0
CCS	CAACACCAACACGTTGTG	192	249	0	2	0	0
CCS	TGACACCAACACCAGTGT	253	280	0	1	0	0
CCS	ACTGGTGTCACAGTACTG	551	583	29	0	0	0
CD14	CAACCAGTACGTCAGTAC	105	287	1	0	0	0
CD14	GTACCAGTACGTCAGTTG	302	487	0	0	0	0
CD14	TGACCAGTACGTCACAGT	119	105	0	1	0	3
CD14	GTACCAGTACGTCACACA	48	66	0	0	0	0
CD14	ACACCAGTACGTCACAAC	58	79	0	0	0	0
CD151	CAACTGGTGTCAACTGAC	99	106	0	0	1	0
CD151	GTACTGGTGTCAACTGTG	102	156	0	0	0	0
CD151	ACACTGGTGTCATGGTGT	160	170	0	0	0	0
CD151	TGACTGGTGTCATGGTCA	384	322	0	0	0	0
CD151	CAACTGGTGTCATGGTAC	23	3	0	0	0	0
CD160	ACACTGGTGTGTTGCATG	233	173	0	0	0	1
CD160	CAACTGGTGTGTTGACGT	320	323	0	1	0	7
CD160	ACACTGGTGTGTTGACCA	81	66	0	0	0	0
CD160	GTACTGGTGTGTTGACAC	22	26	0	0	0	0
CD160	TGACTGGTGTGTTGACTG	152	202	0	1	0	0
CD180	CAACTGCAACCACAGTCA	1415	1348	438	10	1	0
CD180	TGACTGCAACCACAGTAC	98	295	0	0	0	0
CD180	ACACTGCAACCACAGTTG	755	719	2	1	0	3
CD180	CAACTGCAACCACACAGT	1048	748	1	1	0	3
CD180	ACACTGCAACCACACACA	179	273	1	0	0	6
CD180	ACTGCACAACGTGTTGTG	117	196	0	0	0	0
CD19	TGACTGGTGTACCACAGT	447	269	1	0	0	0
CD19	GTACTGGTGTACCACACA	47	89	0	0	0	0
CD19	ACACTGGTGTACCACAAC	13	6	1	0	0	0
CD19	CAACTGGTGTACCACATG	282	469	0	0	0	0
CD19	GTACTGGTGTACCAACGT	37	57	0	0	0	0
CD1A	TGACTGGTGTCACACATG	191	108	0	0	0	0
CD1A	ACACTGGTGTCACAACGT	441	454	3	0	0	0
CD1A	TGACTGGTGTCACAACCA	134	143	0	0	0	0
CD1A	CAACTGGTGTCACAACAC	66	48	0	0	0	0
CD1A	CATGACACGTCACACAGT	106	76	0	0	0	1
CD1B	CAACTGGTGTACGTGTTG	859	753	2	2	0	1
CD1B	GTACTGGTGTACGTCAGT	278	73	0	0	0	1
CD1B	CAACTGGTGTACGTCACA	75	144	0	1	0	0
CD1B	TGACTGGTGTACGTCAAC	8	15	0	0	0	0
CD1B	GTTGCAGTTGACGTCAAC	44	49	0	0	0	0
CD1C	CAACTGGTGTGTCAACTG	1503	1431	3	0	1	5

CD1C	TGACTGGTGTGTCAACGT	370	442	0	0	0	0
CD1C	GTACTGGTGTGTCAACCA	60	131	0	0	0	0
CD1C	ACACTGGTGTGTCAACAC	29	29	0	0	0	0
CD1C	GTACTGGTGTGTCATGGT	822	897	5	0	0	4
CD1D	ACACTGGTGTCAACACCA	180	359	0	1	0	0
CD1D	GTACTGGTGTCAACACAC	32	30	0	0	0	0
CD1D	TGACTGGTGTCAACACTG	655	657	0	1	0	3
CD1D	ACACTGGTGTCAACTGGT	217	307	0	0	0	0
CD1D	TGACTGGTGTCAACTGCA	139	156	0	0	0	1
CD1E	TGACTGGTGTGTACCATG	161	184	0	0	0	0
CD1E	ACACTGGTGTGTACACGT	5	10	0	0	0	0
CD1E	TGACTGGTGTGTACACCA	45	34	0	0	0	0
CD1E	CAACTGGTGTGTACACAC	41	34	0	0	0	0
CD1E	GTACTGGTGTGTACACTG	52	68	0	0	0	0
CD2	ACTGCATGCAGTGTGTCA	351	447	0	0	1	1
CD2	GTACTGGTGTGTTGTGTG	368	369	0	1	0	0
CD2	CAACTGGTGTCAGTGTGT	129	317	0	0	0	0
CD2	ACACTGGTGTCAGTGTCA	101	83	0	0	0	3
CD2	GTACTGGTGTCAGTGTAC	70	110	0	0	0	0
CD2	TGACTGGTGTCAGTGTTG	265	334	0	0	0	0
CD200R1	GTTGACCACAGTGTCACA	116	100	9	21	3	7
CD200R1	ACACTGCAACACCAGTGT	402	744	0	1	0	0
CD200R1	TGACTGCAACACCAGTCA	169	74	0	0	0	0
CD200R1	CATGGTACTGCAGTCAAC	41	15	0	0	0	0
CD200R1	TGTGACCACAGTGTCAGT	168	128	0	0	0	0
CD207	GTACACTGTGTGACTGCA	228	306	0	0	0	1
CD207	ACACACTGTGTGACTGAC	6	17	0	0	0	0
CD207	CAACACTGTGTGACTGTG	167	205	0	0	0	0
CD207	TGACACTGTGTGTGGTGT	442	376	0	0	0	0
CD207	GTTGCAGTTGACCAACAC	90	145	0	0	0	0
CD209	TGACCAGTTGACGTCAAC	83	57	1	0	0	0
CD209	ACACCAGTTGACGTCATG	88	61	0	0	0	0
CD209	CAACCAGTTGACGTACGT	862	209	0	1	0	0
CD209	ACACCAGTTGACGTACCA	159	190	1	0	0	1
CD209	GTACCAGTTGACGTACAC	153	82	0	0	0	0
CD209	TGTGCAACACACACACGT	95	112	0	0	0	1
CD22	GTACTGGTGTCACAACTG	337	346	1191501	1318	68	159
CD22	TGACTGGTGTCACATGGT	193	202	0	0	1	0
CD22	ACACTGGTGTCACATGAC	43	34	0	0	1	0
CD22	GTACTGGTGTCACATGCA	190	357	2	0	0	6
CD22	TGTGCAGTTGACGTCATG	468	387	1	0	0	2
CD22	GTTGCACAGTCATGACGT	43	51	0	0	0	1
CD226	GTACTGGTGTCAACCAGT	38	63	0	0	0	0
CD226	CAACTGGTGTCAACCACA	50	66	1	0	0	0
CD226	TGACTGGTGTCAACCAAC	41	39	0	0	0	0
CD226	ACACTGGTGTCAACCATG	107	156	0	1	0	0
CD226	CAACTGGTGTCAACACGT	37	45	1	0	0	0
CD226	GTTGCACAACCAGTTGCA	176	140	0	0	0	0
CD24	ACACTGGTGTACGTCATG	302	122	0	0	0	0
CD24	CAACTGGTGTACGTACGT	145	156	0	0	0	0
CD24	ACACTGGTGTACGTACCA	377	250	0	1	0	0
CD24	GTACTGGTGTACGTACAC	65	16	0	0	0	0
CD24	TGTGGTTGTGACCAACTG	706	813	0	0	0	0
CD244	GTACTGGTGTGTGTTGAC	70	60	0	0	0	0
CD244	TGACTGGTGTGTGTTGTG	11	28	0	0	0	0
CD244	CAACTGGTGTGTCAGTGT	502	447	1	1	0	0
CD244	ACACTGGTGTGTCAGTCA	247	291	0	0	0	0
CD244	GTACTGGTGTGTCAGTAC	194	56	0	0	0	2
CD247	GTACACTGTGCACAACGT	358	414	1	4	0	57
CD247	CAACACTGTGCACAACCA	469	376	1	1	0	0
CD247	TGACACTGTGCACAACAC	16	27	0	0	0	0
CD247	ACACACTGTGCACAACTG	298	299	0	1	0	0
CD247	TGTGCAGTTGCACAGTTG	272	256	0	0	0	0
CD27	CACATGACTGACGTTGCA	420	658	0	1	1	0
CD27	GTCATGACTGACGTTGGT	182	604	0	0	0	0
CD27	TGCATGACTGACGTTGAC	71	223	0	0	0	0
CD27	ACCATGACTGACGTTGTG	284	196	0	0	0	0
CD27	CATGACACCACATGCACA	445	276	0	1	0	1
CD274	TGACACTGACCATGTGAC	69	122	0	0	0	0
CD274	ACACACTGACCATGTGTG	169	152	0	0	0	0
CD274	ACACACTGACACGTGTGT	289	503	0	0	0	0
CD274	TGACACTGACACGTGTCA	72	57	0	0	0	0
CD274	GTTGCAACCAACGTCATG	61	79	0	0	0	0
CD274	TGTGCAACCAACGTACGT	98	178	0	0	0	0
CD276	CATGGTCAACCATGTGGT	83	70	3	0	10	19

CD276	ACTGGTCAACCATGACTG	564	494	2	0	0	0
CD276	ACTGGTCAACCATGTGCA	316	306	0	0	0	2
CD276	GTTGGTCAACCATGTGAC	131	52	0	0	0	0
CD276	TGTGGTCAACCATGTGTG	67	65	0	0	0	0
CD28	TGACTGGTGTACGTACTG	172	305	1	0	0	0
CD28	ACACTGGTGTACGTTGGT	113	63	0	2	0	0
CD28	TGACTGGTGTACGTTGCA	21	37	0	0	0	0
CD28	CAACTGGTGTACGTTGAC	39	18	0	0	0	0
CD28	ACTGCAGTTGACCACATG	132	112	0	0	0	0
CD28	TGTGCACAACGTTGGTCA	408	277	0	0	0	0
CD2AP	CATGGTCAGTTGACTGAC	17	17	0	0	0	0
CD2AP	GTTGGTCAGTTGACTGTG	246	310	0	2	0	0
CD2AP	ACTGGTCAGTTGTGGTGT	790	601	2	6	0	1
CD2AP	TGTGGTCAGTTGTGGTCA	363	526	0	0	0	1
CD2AP	CATGGTCAGTTGTGGTAC	236	59	0	0	0	0
CD2BP2	ACACACTGTGTGACGTTG	456	482	1	0	0	0
CD2BP2	CAACACTGTGTGACCAGT	109	29	2	0	0	5
CD2BP2	ACACACTGTGTGACCACA	42	42	0	0	0	0
CD2BP2	GTACACTGTGTGACCAAC	10	14	0	0	0	0
CD2BP2	TGACACTGTGTGACCATG	303	502	0	1	0	5
CD2BP2	GTTGCACAACGTACGTCA	243	87	0	0	0	0
CD33	GTACTGGTGTCAGTCATG	317	375	0	134	21	0
CD33	ACACTGGTGTCAGTCAGT	189	159	0	0	0	0
CD33	TGACTGGTGTCAGTCACA	66	58	0	0	0	0
CD33	CAACTGGTGTCAGTCAAC	19	25	1	0	0	0
CD33	TGACTGGTGTCAGTACGT	200	138	0	0	0	0
CD34	TGACTGGTGTGTGTCAGT	56	87	0	1	0	0
CD34	GTACTGGTGTGTGTCACA	44	86	2	0	0	0
CD34	ACACTGGTGTGTGTCAAC	3	10	0	0	0	0
CD34	CAACTGGTGTGTGTCATG	117	112	183	1	0	2
CD34	GTACTGGTGTGTGTACGT	95	125	0	0	0	0
CD34	CATGCACAGTCATGACCA	121	265	0	0	0	0
CD36	GTACACTGACACACCAAC	36	35	0	0	0	0
CD36	TGACACTGACACACCATG	203	183	0	0	0	0
CD36	ACACACTGACACACACGT	252	275	0	0	0	0
CD36	CATGACGTCAACGTCATG	87	110	0	1	0	0
CD36	GTTGACGTCAACGTACGT	295	207	1	0	0	0
CD37	TGACTGGTGTGTACGTAC	32	48	2	0	0	0
CD37	ACACTGGTGTGTACGTTG	109	214	0	0	0	0
CD37	CAACTGGTGTGTACCAGT	26	43	0	0	0	0
CD37	ACACTGGTGTGTACCACA	89	43	0	0	0	0
CD37	GTACTGGTGTGTACCAAC	30	28	0	0	0	0
CD37	TGTGCACAGTCATGACAC	73	83	0	0	0	0
CD38	TGACACACGTTGCAGTCA	559	409	1	45	1	1
CD38	GTACACACGTTGCAGTTG	463	646	2	0	1	0
CD38	CAACACACGTTGCAGTAC	23	30	0	0	0	0
CD38	TGACACACGTTGCACAGT	425	445	0	0	0	2
CD38	GTACACACGTTGCACACA	12	2	0	0	0	0
CD3D	GTACACTGTGCAGTTGTG	25	60	0	0	0	0
CD3D	ACACACTGTGCACAGTGT	319	441	1	0	0	0
CD3D	TGACACTGTGCACAGTCA	282	317	0	4	0	0
CD3D	CAACACTGTGCACAGTAC	92	77	0	0	0	0
CD3D	GTACACTGTGCACAGTTG	460	484	2	0	0	1
CD3E	ACACACTGTGCAGTTGGT	553	894	0	0	0	1
CD3E	TGACACTGTGCAGTTGCA	93	89	0	0	0	0
CD3E	CAACACTGTGCAGTTGAC	43	96	0	0	0	0
CD3E	ACTGCAGTTGCACAGTCA	275	398	0	0	0	0
CD3E	GTTGCAGTTGCACAGTAC	18	6	0	0	0	0
CD3EAP	TGTGGTCATGCACACAAC	33	28	0	0	0	0
CD3EAP	ACTGGTCATGCACACATG	82	150	0	0	0	0
CD3EAP	CATGGTCATGCACAACGT	1142	779	0	0	0	0
CD3EAP	ACTGGTCATGCACAACCA	529	493	0	0	0	0
CD3EAP	GTTGGTCATGCACAACAC	15	25	0	0	0	0
CD3EAP	TGTGCACAACACCAACGT	115	144	0	0	0	0
CD3G	TGACACTGTGCACACAGT	592	346	0	0	0	0
CD3G	GTACACTGTGCACACACA	177	160	2	0	0	0
CD3G	ACACACTGTGCACACAAC	21	25	0	0	0	0
CD3G	CAACACTGTGCACACATG	187	227	0	1	0	0
CD3G	CATGCAGTTGCAGTTGGT	74	202	1	0	0	0
CD3G	GTTGCACACACATGGTCA	174	217	0	1	0	0
CD4	TGACTGGTGTCACAGTAC	53	55	0	0	0	0
CD4	ACACTGGTGTCACAGTTG	1026	1028	1	1	0	0
CD4	CAACTGGTGTCACACAGT	1001	573	2	4	0	1
CD4	ACACTGGTGTCACACACA	53	25	0	0	0	0
CD4	GTACTGGTGTCACACAAC	26	37	181	2	0	1

CD40	TGACCATGGTCAACCATG	208	109	0	0	0	0
CD40	ACACCATGGTCAACACGT	46	57	0	0	0	0
CD40	TGACCATGGTCAACACCA	100	94	0	0	0	0
CD40	CAACCATGGTCAACACAC	321	108	1	0	0	0
CD40	GTACCATGGTCAACACTG	659	424	0	0	0	0
CD40LG	GTACTGCAGTCACACATG	368	423	1	2	2	33
CD40LG	TGACTGCAGTCACACACA	70	66	0	0	0	0
CD40LG	CAACTGCAGTCACACAAC	22	23	0	0	0	0
CD40LG	TGACTGCAGTCACAACGT	48	335	0	2	0	0
CD40LG	GTACTGCAGTCACAACCA	71	47	1	0	0	0
CD44	TGACTGGTGTGTACTGGT	339	258	1	0	1	1
CD44	TGTGCAGTCACAACACTG	287	275	0	1	1	0
CD44	GTACTGGTGTGTACTGCA	83	126	0	0	0	0
CD44	ACACTGGTGTGTACTGAC	44	23	0	0	0	0
CD44	GTTGCAGTCACAACACAC	48	41	0	0	0	0
CD46	GTACACTGTGGTTGGTAC	55	40	0	0	0	0
CD46	TGACACTGTGGTTGGTTG	246	283	2	0	0	0
CD46	ACACACTGTGGTTGCAGT	112	143	0	0	0	0
CD46	TGACACTGTGGTTGCACA	71	77	0	0	0	0
CD46	CAACACTGTGGTTGCAAC	80	57	0	0	0	0
CD46	TGTGCATGGTGTGTTGGT	313	342	1	0	0	0
CD47	CAACGTGTACACTGGTAC	90	82	0	0	0	0
CD47	GTACGTGTACACTGGTTG	173	161	0	0	0	0
CD47	TGACGTGTACACTGCAGT	408	55	1	0	0	0
CD47	GTACGTGTACACTGCACA	92	156	2	0	0	0
CD47	ACTGCAGTCAGTACGTTG	1085	828	0	0	0	1
CD48	CATGCAACACGTCAACTG	670	1998	4	5	1	3
CD48	TGACTGGTGTCATGTGTG	623	631	4	2	0	1
CD48	TGACTGGTGTACGTGTGT	261	355	0	1	0	1
CD48	GTACTGGTGTACGTGTCA	179	281	1	0	0	0
CD48	ACACTGGTGTACGTGTAC	19	22	0	0	0	0
CD48	ACTGCAGTTGACGTACGT	89	50	0	0	0	0
CD5	CAACTGGTGTCATGCATG	339	474	1	0	1	1
CD5	GTACTGGTGTCATGACGT	495	176	1	0	0	0
CD5	CAACTGGTGTCATGACCA	529	616	3	0	0	0
CD5	TGACTGGTGTCATGACAC	32	24	0	0	0	0
CD5	ACTGCAGTTGACCAACCA	629	733	0	1	0	1
CD5	GTTGCAACTGCAACACGT	0	0	0	0	0	0
CD52	CAACTGGTGTGTACTGTG	367	378	0	1	0	0
CD52	TGACTGGTGTGTTGGTGT	310	469	1	0	0	0
CD52	GTACTGGTGTGTTGGTCA	194	137	0	1	0	0
CD52	TGTGCAGTTGACGTACCA	39	42	1	0	0	92
CD52	CATGACCACAGTCACAAC	20	23	0	0	0	0
CD53	CAACTGGTGTCACATGTG	261	286	0	0	0	0
CD53	TGACTGGTGTCAACGTGT	278	341	0	0	0	1
CD53	GTACTGGTGTCAACGTCA	311	343	0	1	0	1
CD53	ACACTGGTGTCAACGTAC	143	168	0	0	0	1
CD53	CAACTGGTGTCAACGTTG	879	782	1	1	0	0
CD55	GTACACTGTGGTCATGTG	794	1191	1	0	1	4
CD55	CAACACTGTGGTCATGAC	101	138	0	0	0	0
CD55	ACACACTGTGGTACGTGT	320	353	3	0	0	0
CD55	TGACACTGTGGTACGTCA	106	113	0	0	0	0
CD55	CAACACTGTGGTACGTAC	65	66	0	0	0	0
CD58	GTACTGGTGTGTACGTGT	620	359	25	27	21	54735
CD58	ACTGCACAGTCATGACTG	519	311	0	1	1	2
CD58	CAACTGGTGTGTCATGCA	542	466	0	1	0	2
CD58	TGACTGGTGTGTCATGAC	18	21	0	0	0	0
CD58	ACACTGGTGTGTCATGTG	319	428	0	0	0	0
CD58	CAACTGGTGTGTACGTCA	288	486	0	0	0	1
CD59	GTACACTGACTGACTGGT	143	152	0	0	0	0
CD59	CAACACTGACTGACTGCA	125	54	0	0	0	0
CD59	TGACACTGACTGACTGAC	288	52	1	0	0	0
CD59	ACACACTGACTGACTGTG	364	416	0	2	0	0
CD59	GTACACTGACTGTGGTGT	598	531	5	0	0	0
CD6	GTACTGGTGTCAGTACCA	199	74	0	0	0	0
CD6	ACACTGGTGTCAGTACAC	29	106	0	0	0	0
CD6	CAACTGGTGTCAGTACTG	118	112	2	0	0	0
CD6	GTACTGGTGTCAGTTGGT	264	377	1	0	0	0
CD6	CAACTGGTGTCAGTTGCA	234	265	0	0	0	0
CD63	TGACGTGTACACTGACAC	27	27	0	0	0	0
CD63	ACACGTGTACACTGACTG	270	173	0	0	0	0
CD63	CAACGTGTACACTGTGGT	130	77	0	0	0	0
CD63	ACACGTGTACACTGTGCA	74	130	0	0	0	0
CD63	GTACGTGTACACTGTGAC	33	83	0	0	0	0
CD68	CAACTGGTGTGTGTACCA	837	954	567051	596	89	59

CD68	TGACTGGTGTGTGTACAC	51	30	0	0	0	0
CD68	ACACTGGTGTGTGTACTG	136	189	1	0	0	0
CD68	CAACTGGTGTGTGTTGGT	136	108	3	1	0	81
CD68	ACACTGGTGTGTGTTGCA	84	86	0	0	0	0
CD69	CAACTGGTGTACCAACCA	419	298	1	0	1	0
CD69	TGACTGGTGTACCAACAC	9	12	0	0	0	0
CD69	ACACTGGTGTACCAACTG	229	298	0	0	0	0
CD69	CAACTGGTGTACCATGGT	56	74	0	0	0	0
CD69	ACACTGGTGTACCATGCA	77	80	0	0	0	0
CD69	CATGCACAGTCATGTGGT	486	587	1	0	0	0
CD7	ACACTGGTGTGTTGGTAC	152	72	0	0	0	0
CD7	CAACTGGTGTGTTGGTTG	294	449	0	0	0	0
CD7	GTACTGGTGTGTTGCAGT	419	260	0	0	0	0
CD7	CAACTGGTGTGTTGCACA	68	30	0	0	0	2
CD7	TGACTGGTGTGTTGCAAC	16	18	0	0	0	0
CD72	TGACACTGTGTGACTGGT	91	145	0	0	1	0
CD72	ACACACTGTGTGACACGT	58	24	0	1	0	0
CD72	TGACACTGTGTGACACCA	211	418	0	0	0	0
CD72	CAACACTGTGTGACACAC	282	85	0	0	0	0
CD72	GTACACTGTGTGACACTG	310	331	0	1	0	0
CD72	ACTGCACAGTCATGTGCA	68	94	0	0	0	0
CD74	TGACGTCAGTTGCATGCA	81	72	0	0	1	0
CD74	ACACGTCAGTTGCATGGT	913	397	3	0	0	0
CD74	CAACGTCAGTTGCATGAC	47	39	0	0	0	0
CD74	GTACGTCAGTTGCATGTG	116	243	0	0	0	0
CD74	ACACGTCAGTTGACGTGT	94	111	0	0	0	0
CD79A	GTACACTGTGTGTGTGTG	41	87	0	0	0	0
CD79A	ACACTGGTGTGTGTGTGT	91	153	1	0	0	0
CD79A	TGACTGGTGTGTGTGTCA	510	512	118	0	0	0
CD79A	CAACTGGTGTGTGTGTAC	43	65	0	0	0	0
CD79A	GTACTGGTGTGTGTGTTG	195	337	0	0	0	0
CD79B	GTACTGGTGTCATGGTTG	696	909	0	1	0	0
CD79B	TGACTGGTGTCATGCAGT	178	187	0	0	0	1
CD79B	GTACTGGTGTCATGCACA	37	55	0	0	0	0
CD79B	ACACTGGTGTCATGCAAC	15	16	0	0	0	0
CD79B	GTTGCAGTTGCATGTGTG	117	90	0	0	0	0
CD80	TGACACTGACGTTGCAGT	93	83	0	0	0	4
CD80	GTACACTGACGTTGCACA	48	53	0	0	0	0
CD80	ACACACTGACGTTGCAAC	14	47	0	0	0	0
CD80	CAACACTGACGTTGCATG	440	282	0	1	0	1
CD80	GTACACTGACGTTGACGT	124	97	0	0	0	0
CD81	TGACTGGTGTCAGTTGAC	22	36	0	0	0	0
CD81	ACACTGGTGTCAGTTGTG	276	333	0	1	0	0
CD81	GTACTGGTGTCACAGTGT	627	812	2	1	0	1
CD81	CAACTGGTGTCACAGTCA	74	73	0	0	0	1
CD81	GTTGCAGTCATGACGTTG	508	788	2	0	0	0
CD82	ACACCAACTGACCACACA	212	225	2	0	0	0
CD82	GTACCAACTGACCACAAC	40	30	1	0	0	0
CD82	TGACCAACTGACCACATG	506	599	0	1	0	0
CD82	TGTGGTTGTGACCATGCA	372	687	1	0	0	0
CD82	CATGGTTGTGACCATGAC	17	4	0	0	0	0
CD83	CAACACTGACACGTGTAC	327	77	0	0	0	0
CD83	GTACACTGACACGTGTTG	1824	1584	1	1	0	0
CD83	TGACACTGACACGTCAGT	51	52	0	0	0	0
CD83	GTACACTGACACGTCACA	43	38	0	0	0	0
CD83	ACACACTGACACGTCAAC	16	10	0	0	0	0
CD86	GTACGTGTACACCACAAC	9	15	0	0	0	0
CD86	TGACGTGTACACCACATG	256	89	0	2	0	0
CD86	ACACGTGTACACCAACGT	504	209	0	0	0	0
CD86	TGACGTGTACACCAACCA	515	501	0	1	0	0
CD86	ACTGCAGTACCACAACTG	1028	1160	1	2	0	3
CD86	GTTGCATGGTCATGACTG	553	467	1	0	0	0
CD8A	ACACTGGTGTGTTGTGGT	240	175	2	0	0	0
CD8A	TGACTGGTGTGTTGTGCA	110	118	0	0	0	1
CD8A	CAACTGGTGTGTTGTGAC	97	165	0	0	0	0
CD8A	TGTGACCATGCAACTGGT	576	751	1	1	0	6
CD8A	GTTGACCATGCAACTGCA	173	209	0	0	0	0
CD8B	TGACACTGTGTGTGACTG	114	173	0	0	0	0
CD8B	ACACACTGTGTGTGTGGT	91	113	0	0	0	0
CD8B	TGACACTGTGTGTGTGCA	189	150	0	0	0	0
CD8B	CAACACTGTGTGTGTGAC	15	18	0	0	0	3
CD8B	TGTGACCATGCACAACGT	119	71	0	0	0	0
CD9	TGACACTGTGTGCATGAC	43	38	0	0	0	0
CD9	ACACACTGTGTGCATGTG	855	1030	1	0	0	1
CD9	GTACACTGTGTGACGTGT	369	637	2	0	0	0

CD9	CAACACTGTGTGACGTCA	261	261	0	0	0	0
CD9	TGACACTGTGTGACGTAC	183	68	0	0	0	0
CD9	GTTGCAACCATGACCAAC	61	39	0	2	0	0
CD93	CAACCAGTACGTCACATG	797	919	0	1	0	1
CD93	GTACCAGTACGTCAACGT	421	526	1	1	0	0
CD93	CAACCAGTACGTCAACCA	170	159	0	0	0	0
CD93	TGACCAGTACGTCAACAC	88	52	3	0	0	3
CD93	ACACCAGTACGTCAACTG	264	247	0	1	0	1
CD96	GTACTGGTGTACGTTGTG	175	168	0	0	0	0
CD96	ACACTGGTGTACCAGTGT	108	142	0	0	0	1
CD96	TGACTGGTGTACCAGTCA	134	59	0	0	0	0
CD96	CAACTGGTGTACCAGTAC	54	84	0	0	0	0
CD96	GTACTGGTGTACCAGTTG	1385	1183	1	43	0	3
CD97	GTACGTGTTGTGGTCATG	216	153	0	0	0	0
CD97	TGACGTGTTGTGGTACGT	168	104	0	0	0	0
CD97	GTACGTGTTGTGGTACCA	197	165	0	0	0	0
CD97	ACACGTGTTGTGGTACAC	32	24	0	0	0	0
CD97	CAACGTGTTGTGGTACTG	831	1366	1	1	0	1
CD99	CAACACTGTGTGTGACGT	240	265	0	0	1	0
CD99	TGACACTGTGTGTGCAAC	57	63	0	0	0	0
CD99	ACACACTGTGTGTGCATG	273	264	0	0	0	3
CD99	ACACACTGTGTGTGACCA	86	119	0	0	0	0
CD99	GTACACTGTGTGTGACAC	16	11	0	0	0	0
CDA	ACACACACCAACCAGTGT	468	485	1	0	1	1
CDA	TGACACACCAACCAGTCA	124	194	0	0	0	0
CDA	CAACACACCAACCAGTAC	105	122	0	0	0	0
CDA	GTACACACCAACCAGTTG	290	295	0	0	0	0
CDA	TGACACACCAACCACAGT	123	303	1	1	0	0
CDAN1	ACACTGTGTGTGACTGGT	207	340	1	0	0	0
CDAN1	TGACTGTGTGTGACTGCA	127	81	1	0	0	0
CDAN1	CAACTGTGTGTGACTGAC	32	37	0	0	0	0
CDAN1	GTACTGTGTGTGACTGTG	819	460	1	0	0	0
CDAN1	ACACTGTGTGTGTGGTGT	426	481	0	0	0	0
CDC14A	TGCATGACACGTTGCAGT	238	277	0	0	0	0
CDC14A	GTCATGACACGTTGCACA	103	119	0	1	0	1
CDC14A	ACCATGACACGTTGCAAC	25	31	0	0	0	0
CDC14A	CACATGACACGTTGCATG	92	71	0	0	0	0
CDC14A	GTCATGACACGTTGACGT	897	445	0	1	0	0
CDC14B	TGTGGTTGGTACTGCAAC	10	13	0	2	1	0
CDC14B	TGCATGTGTGCATGGTTG	202	307	0	0	0	1
CDC14B	ACCATGTGTGCATGCAGT	162	325	2	0	0	0
CDC14B	CATGCAGTGTTGCACATG	164	155	1	0	0	0
CDC14B	GTTGCAGTGTTGCAACGT	259	215	0	1	0	0
CDC14B	ACTGCAACGTCATGACCA	114	154	0	0	0	0
CDC16	CATGACACCAACGTGTCA	55	17	0	0	1	0
CDC16	GTTGGTTGCACACAACTG	675	455	0	1	0	0
CDC16	TGTGGTTGCACACATGGT	174	345	1	0	0	0
CDC16	GTTGGTTGCACACATGCA	189	223	0	0	0	0
CDC16	ACTGGTTGCACACATGAC	5	37	0	0	0	0
CDC2	TGCATGCAACCACAACAC	18	27	0	0	0	0
CDC2	ACCATGCAACCACAACTG	901	825	0	1	0	1
CDC2	CACATGCAACCACATGGT	284	319	0	1	0	0
CDC2	ACCATGCAACCACATGCA	204	337	0	0	0	0
CDC2	TGTGGTTGGTGTCACATG	194	155	0	0	0	0
CDC2	TGTGCAACGTACGTTGGT	77	69	0	0	0	0
CDC20	TGCATGTGGTGTGTACTG	124	132	0	0	0	0
CDC20	ACCATGTGGTGTGTTGGT	36	28	0	0	0	0
CDC20	TGCATGTGGTGTGTTGCA	172	182	0	0	0	0
CDC20	CACATGTGGTGTGTTGAC	77	48	0	0	0	0
CDC20	GTCATGTGGTGTGTTGTG	70	136	0	0	0	0
CDC23	GTACGTGTACGTACTGAC	25	142	0	0	0	0
CDC23	TGACGTGTACGTACTGTG	82	98	0	0	0	0
CDC23	CAACGTGTACGTTGGTGT	297	383	0	0	0	0
CDC23	ACACGTGTACGTTGGTCA	314	261	0	0	0	0
CDC23	GTACGTGTACGTTGGTAC	3	7	0	0	0	0
CDC25A	TGTGGTTGTGACGTACCA	84	105	0	0	1	4
CDC25A	GTCATGACCACATGGTCA	181	240	0	1	0	0
CDC25A	ACCATGACCACATGGTAC	58	76	0	0	0	1
CDC25A	CACATGACCACATGGTTG	522	371	0	0	0	0
CDC25A	CATGGTTGTGACGTACAC	34	102	0	0	0	1
CDC25B	TGCATGACCAACACGTCA	353	136	0	0	0	0
CDC25B	CACATGACCAACACGTAC	116	134	0	1	0	0
CDC25B	GTCATGACCAACACGTTG	412	294	1	0	0	0
CDC25B	TGCATGACCAACACCAGT	378	322	2	0	0	0
CDC25B	GTCATGACCAACACCACA	165	193	1	0	0	0

CDC25C	ACCATGACCACATGCATG	300	132	0	0	1	0
CDC25C	GTTGCAGTCATGCATGTG	259	309	0	0	1	0
CDC25C	GTCATGACCACATGCAGT	160	128	1	0	0	0
CDC25C	CACATGACCACATGCACA	398	480	0	0	0	0
CDC25C	TGCATGACCACATGCAAC	82	11	0	0	0	0
CDC26	TGTGGTCACATGTGACTG	183	245	0	0	0	0
CDC26	ACTGGTCACATGTGTGGT	272	654	0	1	0	0
CDC26	TGTGGTCACATGTGTGCA	172	253	3	1	0	0
CDC26	CATGGTCACATGTGTGAC	5	13	0	0	0	0
CDC26	GTTGGTCACATGTGTGTG	313	323	1	1	0	0
CDC27	CATGGTCAACTGTGTGCA	184	591	0	1	1	0
CDC27	GTTGCAGTGTACTGTGTG	163	273	0	0	1	1
CDC27	TGTGGTCAACTGTGTGAC	36	67	0	0	0	0
CDC27	ACTGGTCAACTGTGTGTG	1295	839	1	0	0	0
CDC27	CATGGTCATGGTGTGTGT	716	232	0	1	0	0
CDC27	GTTGCAACGTACACGTTG	533	536	1	0	0	0
CDC2L1	TGACGTGTGTGTCACATG	106	131	0	0	0	0
CDC2L1	ACACGTGTGTGTCAACGT	251	265	0	1	0	0
CDC2L1	TGACGTGTGTGTCAACCA	693	152	1	0	0	0
CDC2L1	CAACGTGTGTGTCAACAC	4	9	0	0	0	0
CDC2L1	GTACGTGTGTGTCAACTG	225	228	0	0	0	0
CDC2L1	ACTGCAACGTACCAGTAC	40	55	0	1	0	0
CDC2L2	ACTGGTACTGACACGTCA	190	189	289348	300	24	19
CDC2L2	CAACGTGTCACATGTGGT	828	959	1	1	2	1
CDC2L2	TGACGTGTCACATGACAC	30	21	0	0	0	0
CDC2L2	ACACGTGTCACATGACTG	1446	890	1	0	0	126
CDC2L2	GTTGGTACTGACACGTAC	14	33	0	0	0	0
CDC2L5	GTCATGCAACACTGCACA	95	227	0	0	0	0
CDC2L5	ACCATGCAACACTGCAAC	34	50	0	0	0	0
CDC2L5	CACATGCAACACTGCATG	512	75	0	1	0	0
CDC2L5	GTCATGCAACACTGACGT	180	195	1	1	0	0
CDC2L5	ACTGGTACTGACGTTGGT	60	145	0	0	0	0
CDC2L6	TGCATGACACACGTGTCA	231	235	3	733	3	8137
CDC2L6	CACATGACACCATGTGCA	150	67	0	0	0	0
CDC2L6	TGCATGACACCATGTGAC	16	16	0	0	0	0
CDC2L6	ACCATGACACCATGTGTG	187	410	0	0	0	3
CDC2L6	ACCATGACACACGTGTGT	66	134	0	0	0	0
CDC34	TGACGTGTCATGTGTGAC	69	94	0	0	0	0
CDC34	ACACGTGTCATGTGTGTG	506	278	1	1	0	4
CDC34	GTACGTGTACGTGTGTGT	197	149	0	0	0	0
CDC34	CAACGTGTACGTGTGTCA	448	345	0	0	0	0
CDC34	TGACGTGTACGTGTGTAC	324	28	0	1	0	0
CDC34	CATGCAACGTACCAGTTG	716	687	30	0	0	1
CDC37	CATGGTGTTGGTGTACAC	49	39	0	0	0	0
CDC37	GTTGGTGTTGGTGTACTG	173	55	1	0	0	0
CDC37	TGTGGTGTTGGTGTTGGT	92	72	0	0	0	1
CDC37	GTTGGTGTTGGTGTTGCA	225	250	1	0	0	0
CDC37	ACTGGTGTTGGTGTTGAC	15	18	0	0	0	0
CDC40	TGCATGCATGACTGGTTG	336	228	0	0	1	0
CDC40	ACCATGCATGACTGCAGT	332	185	2	1	1	0
CDC40	ACCATGCATGACTGGTCA	646	313	0	0	0	9
CDC40	GTCATGCATGACTGGTAC	96	105	0	12	0	0
CDC40	TGCATGCATGACTGCACA	257	121	0	0	0	0
CDC42	GTACACCACAGTGTCATG	140	89	0	0	0	0
CDC42	TGACACCACAGTGTACGT	56	146	0	0	0	0
CDC42	GTACACCACAGTGTACCA	7	28	0	0	0	0
CDC42	ACACACCACAGTGTACAC	34	56	0	0	0	0
CDC42	CAACACCACAGTGTACTG	290	236	2	3	0	5
CDC45L	TGTGGTCAACTGCAACTG	416	1112	1	0	0	0
CDC45L	ACTGGTCAACTGCATGGT	244	869	0	0	0	0
CDC45L	TGTGGTCAACTGCATGCA	83	35	0	0	0	0
CDC45L	CATGGTCAACTGCATGAC	75	40	0	0	0	0
CDC45L	GTTGGTCAACTGCATGTG	265	523	0	0	0	1
CDC6	CATGGTACCAACGTGTAC	78	112	0	0	1	7
CDC6	CATGGTGTGTTGTGGTGT	647	536	1	1	0	6
CDC6	ACTGGTACCACATGTGTG	133	270	0	0	0	0
CDC6	ACTGGTACCAACGTGTGT	80	114	0	0	0	0
CDC6	TGTGGTACCAACGTGTCA	365	344	1	0	0	0
CDC7	TGCATGACACACGTTGTG	172	267	1	0	0	0
CDC7	CACATGACACACCAGTGT	93	63	0	1	0	0
CDC7	ACCATGACACACCAGTCA	785	1073	32	1	0	0
CDC7	GTCATGACACACCAGTAC	124	88	0	0	0	0
CDC7	TGCATGACACACCAGTTG	444	496	1	0	0	1
CDC7	TGTGCACATGCATGTGGT	157	158	0	0	0	0
CDCA8	GTACGTGTACTGGTTGAC	23	36	0	0	0	0

CDCA8	TGACGTGTACTGGTTGTG	112	158	0	0	0	0
CDCA8	CAACGTGTACTGCAGTGT	280	475	1	0	0	0
CDCA8	ACACGTGTACTGCAGTCA	185	181	0	0	0	0
CDCA8	CATGCAGTCAGTACCAGT	267	276	2	0	0	0
CDH1	GTACCATGGTTGACTGTG	675	267	0	0	0	0
CDH1	ACACCATGGTTGTGGTGT	55	29	0	0	0	0
CDH1	TGACCATGGTTGTGGTCA	390	104	0	0	0	0
CDH1	CAACCATGGTTGTGGTAC	143	143	0	0	0	0
CDH1	GTACCATGGTTGTGGTTG	1934	1183	4	0	0	11
CDH1	CATGCAACGTGTTGTGTG	90	60	0	0	0	0
CDH15	ACACACTGGTTGTGTGTG	754	371	0	1	1	2
CDH15	TGACACTGCAGTGTGTGT	1446	868	1	0	0	1
CDH15	GTACACTGCAGTGTGTCA	1267	676	1	1	0	0
CDH15	ACACACTGCAGTGTGTAC	13	6	0	0	0	0
CDH15	CATGCAACGTACCATGAC	242	89	1	0	0	0
CDH15	GTTGCAACGTACCATGTG	284	338	0	0	0	0
CDH2	ACACACTGGTACACACAC	57	46	0	0	0	0
CDH2	CAACACTGGTACACACTG	527	980	3	0	0	0
CDH2	GTACACTGGTACACTGGT	258	258	1	0	0	0
CDH2	CAACACTGGTACACTGCA	418	535	1	1	0	1
CDH2	TGACACTGGTACACTGAC	159	49	0	0	0	0
CDH2	ACTGCAACGTCAGTGTGT	299	315	1	0	0	9
CDH5	ACACACTGGTTGGTACGT	54	59	0	0	0	0
CDH5	TGACACTGGTTGGTACCA	212	125	0	0	0	0
CDH5	CAACACTGGTTGGTACAC	28	45	0	0	0	0
CDH5	GTACACTGGTTGGTACTG	141	147	0	0	0	0
CDH5	CATGCAGTTGCAGTCATG	96	69	0	0	0	0
CDH5	TGTGCAACGTCAGTGTCA	261	369	0	1	0	0
CDIPT	CAACCAACGTTGCACACA	64	48	22	3	1	0
CDIPT	GTACCAACGTTGCACAGT	498	688	1	0	0	0
CDIPT	TGACCAACGTTGCACAAC	17	19	0	0	0	0
CDIPT	ACACCAACGTTGCACATG	633	560	1	1	0	9
CDIPT	ACTGACACCACAACCACA	100	163	0	0	0	1
CDK10	TGCATGCAACACACACTG	263	238	0	0	0	0
CDK10	ACCATGCAACACACTGGT	176	232	0	0	0	0
CDK10	TGCATGCAACACACTGCA	389	166	1	0	0	0
CDK10	CACATGCAACACACTGAC	42	32	0	0	0	0
CDK10	GTCATGCAACACACTGTG	428	210	0	0	0	0
CDK2	GTCATGCAACCACATGAC	11	23	0	0	1	0
CDK2	TGCATGCAACCACATGTG	508	276	1	0	0	0
CDK2	CACATGCAACCAACGTGT	185	194	0	0	0	0
CDK2	ACCATGCAACCAACGTCA	359	261	1	0	0	0
CDK2	GTCATGCAACCAACGTAC	178	168	0	0	0	0
CDK2	TGTGCAACGTACTGGTTG	478	348	0	1	0	0
CDK3	CACATGCAACGTGTCAGT	176	144	0	0	0	0
CDK3	ACCATGCAACGTGTCACA	95	111	1	0	0	0
CDK3	GTCATGCAACGTGTCAAC	58	29	0	0	0	0
CDK3	TGCATGCAACGTGTCATG	66	52	0	0	0	0
CDK3	TGTGGTACTGACTGCATG	695	1132	1	2	0	3
CDK3	ACTGCACACACATGGTAC	114	139	0	0	0	1
CDK4	TGCATGCACAACCATGTG	753	495	2	0	1	0
CDK4	CACATGCACAACCATGGT	1191	352	0	1	0	0
CDK4	ACCATGCACAACCATGCA	864	892	1	1	0	1
CDK4	GTCATGCACAACCATGAC	34	28	0	0	0	0
CDK4	ACTGGTACTGACCAACTG	261	595	3	0	0	0
CDK4	ACTGCAACGTACTGCAGT	31	27	0	0	0	0
CDK5	GTACGTTGACCACAGTAC	74	139	0	0	0	0
CDK5	TGACGTTGACCACAGTTG	1061	472	1	0	0	1
CDK5	ACACGTTGACCACACAGT	136	230	0	0	0	0
CDK5	TGACGTTGACCACACACA	67	57	0	0	0	0
CDK5	GTTGGTACTGCATGGTTG	827	602	1	2	0	0
CDK5R1	GTACGTGTGTGTACGTCA	213	227	0	0	1	0
CDK5R1	ACACGTGTGTGTACGTAC	23	29	0	0	0	0
CDK5R1	CAACGTGTGTGTACGTTG	883	168	2	1	0	0
CDK5R1	GTACGTGTGTGTACCAGT	127	148	0	1	0	0
CDK5R1	CATGGTTGACACACTGAC	32	31	0	0	0	0
CDK5R2	CAACACGTCAGTGTGTCA	269	191	0	0	0	3
CDK5R2	TGACACGTCAGTGTGTAC	8	27	0	0	0	0
CDK5R2	ACACACGTCAGTGTGTTG	181	240	0	0	0	1
CDK5R2	CAACACGTCAGTGTCAGT	315	346	4	0	0	0
CDK5R2	ACACACGTCAGTGTCACA	81	66	0	0	0	2
CDK5RAP1	ACACACTGCATGTGGTGT	645	635	2	1	1	0
CDK5RAP1	ACACACTGCATGACTGGT	749	624	1	1	0	1
CDK5RAP1	TGACACTGCATGACTGCA	113	157	0	1	0	0
CDK5RAP1	CAACACTGCATGACTGAC	49	58	0	0	0	0

CDK5RAP1	GTACACTGCATGACTGTG	185	380	1	0	0	0
CDK5RAP1	CATGCATGGTCATGGTCA	163	153	0	0	0	0
CDK6	ACCATGCAACGTGTACGT	659	403	0	0	0	1
CDK6	TGCATGCAACGTGTACCA	227	289	0	0	0	0
CDK6	CACATGCAACGTGTACAC	2	3	0	0	0	0
CDK6	GTCATGCAACGTGTACTG	145	152	0	0	0	1
CDK6	CAACGTTGTGACGTACGT	77	110	0	0	0	0
CDK7	TGCATGCAACCAACGTTG	608	594	0	1	0	18
CDK7	ACCATGCAACCAACCAGT	111	105	0	0	0	0
CDK7	TGCATGCAACCAACCACA	199	108	0	1	0	0
CDK7	CACATGCAACCAACCAAC	5	3	0	0	0	0
CDK7	GTCATGCAACCAACCATG	384	161	0	0	0	0
CDK7	TGTGCAACGTACTGACGT	157	177	3	0	0	0
CDK8	TGCATGCAACGTGTTGGT	420	449	1	0	0	0
CDK8	GTCATGCAACGTGTTGCA	40	30	0	0	0	0
CDK8	ACCATGCAACGTGTTGAC	12	23	0	0	0	0
CDK8	CACATGCAACGTGTTGTG	54	155	0	0	0	0
CDK8	TGCATGCAACGTCAGTGT	286	271	0	1	0	0
CDK8	GTTGCACAGTCATGTGAC	23	26	0	0	0	0
CDK9	GTCATGCAACGTCAGTCA	446	590	0	0	0	0
CDK9	ACCATGCAACGTCAGTAC	39	41	0	0	0	0
CDK9	CACATGCAACGTCAGTTG	349	946	0	2	0	2
CDK9	GTCATGCAACGTCACAGT	111	98	0	0	0	0
CDK9	CACATGCAACGTCACACA	66	84	0	0	0	0
CDK9	CATGCAACGTACTGTGCA	321	172	0	0	0	0
CDKL1	CACATGTGTGTGGTCACA	221	195	11	1	0	0
CDKL1	TGCATGTGTGTGGTCAAC	26	17	0	0	0	0
CDKL1	ACCATGTGTGTGGTCATG	213	174	0	0	0	0
CDKL1	CACATGTGTGTGGTACGT	162	202	1	0	0	1
CDKL1	ACCATGTGTGTGGTACCA	32	38	0	0	0	0
CDKL2	ACCATGCAACTGGTGTGT	139	38	0	0	1	0
CDKL2	GTCATGCAACACTGTGAC	38	93	0	0	0	0
CDKL2	TGCATGCAACACTGTGTG	81	124	0	0	0	0
CDKL2	TGCATGCAACTGGTGTCA	239	344	0	0	0	0
CDKL2	CACATGCAACTGGTGTAC	91	69	0	0	0	1
CDKN1A	GTACCATGACGTGTGTTG	1202	1026	734369	723	50	64
CDKN1A	GTACCATGCATGTGTGTG	817	357	1	1	0	0
CDKN1A	ACACCATGACGTGTGTGT	338	263	0	0	0	7
CDKN1A	TGACCATGACGTGTGTCA	263	138	0	0	0	4
CDKN1A	CAACCATGACGTGTGTAC	29	31	0	0	0	0
CDKN1A	TGTGCAACGTTGCATGGT	176	189	0	2	0	0
CDKN1B	CAACCATGCAACCAGTCA	931	519	2	0	0	2
CDKN1B	TGACCATGCAACCAGTAC	78	61	0	0	0	0
CDKN1B	ACACCATGCAACCAGTTG	1502	1937	0	2	0	0
CDKN1B	CAACCATGCAACCACAGT	705	159	0	2	0	2
CDKN1B	ACTGCAGTCACACAGTTG	262	691	0	0	0	0
CDKN1B	GTTGCAACGTTGCATGCA	159	90	1	0	0	0
CDKN1C	CAACCATGGTTGTGTGGT	535	1124	2	1	1	0
CDKN1C	CAACCATGGTTGTGACCA	296	320	0	0	0	0
CDKN1C	TGACCATGGTTGTGACAC	138	122	16	0	0	0
CDKN1C	ACACCATGGTTGTGACTG	88	108	0	0	0	0
CDKN1C	ACACCATGGTTGTGTGCA	140	261	0	0	0	0
CDKN1C	CATGCACACACATGGTTG	254	317	1	0	0	0
CDKN2A	TGACCATGCAGTACCAGT	308	107	1	0	0	0
CDKN2A	GTACCATGCAGTACCACA	122	171	1	0	0	0
CDKN2A	ACACCATGCAGTACCAAC	55	80	1	0	0	0
CDKN2A	TGTGCAGTCAGTGTCAGT	375	546	0	0	0	0
CDKN2A	GTTGCAGTCAGTGTCACA	36	51	0	0	0	0
CDKN2B	TGTGCAGTGTTGCAGTCA	251	343	5	0	1	22
CDKN2B	CAACCAACTGACACACGT	360	183	0	0	0	0
CDKN2B	ACACCAACTGACACACCA	332	399	0	1	0	0
CDKN2B	ACTGCAGTGTTGCAGTGT	32	27	0	0	0	0
CDKN2B	CATGCAGTGTTGCAGTAC	207	160	0	0	0	0
CDKN2C	ACACCAACACTGGTCATG	296	360	1	0	1	1
CDKN2C	GTACCAACACTGGTCAGT	563	374	2	0	0	0
CDKN2C	CAACCAACACTGGTCACA	176	194	1	2	0	6
CDKN2C	TGACCAACACTGGTCAAC	22	32	0	0	0	0
CDKN2C	CAACCAACACTGGTACGT	77	80	0	0	0	0
CDKN2D	CAACACGTCAGTGTACAC	18	32	0	0	0	0
CDKN2D	GTACACGTCAGTGTACTG	126	256	0	0	0	0
CDKN2D	TGACACGTCAGTGTTGGT	59	42	0	0	0	0
CDKN2D	GTACACGTCAGTGTTGCA	47	39	0	0	0	0
CDKN2D	ACACACGTCAGTGTTGAC	27	36	0	0	0	0
CDKN3	ACCATGACCAACACACTG	185	211	1	0	0	0
CDKN3	CACATGACCAACACTGGT	1091	467	4	1	0	4

CDKN3	ACCATGACCAACACTGCA	308	301	0	0	0	1
CDKN3	GTCATGACCAACACTGAC	94	72	0	0	0	0
CDKN3	TGCATGACCAACACTGTG	271	206	0	0	0	0
CDKN3	CATGCAACGTTGCATGTG	163	440	0	2	0	0
CDO1	GTACACTGCATGCAGTGT	53	60	0	0	0	0
CDO1	CAACACTGCATGCAGTCA	48	62	0	0	0	0
CDO1	TGACACTGCATGCAGTAC	164	108	1	0	0	0
CDO1	ACACACTGCATGCAGTTG	149	315	0	0	0	0
CDO1	CAACACTGCATGCACAGT	362	644	0	1	0	2
CDR1	TGTGGTTGCACAACGTGT	507	188	0	0	1	0
CDR1	CATGGTTGCACACATGTG	292	464	2	0	0	0
CDR1	TGTGACGTGTACGTGTTG	422	682	0	3	0	0
CDR1	ACTGACGTGTACGTCAGT	32	43	0	0	0	0
CDR1	TGTGACGTGTACGTCACA	3	25	0	0	0	0
CDS1	ACACCAACGTCATGTGCA	276	252	0	0	0	0
CDS1	GTACCAACGTCATGTGAC	30	35	0	0	0	0
CDS1	TGACCAACGTCATGTGTG	451	521	896	3	0	0
CDS1	TGACCAACGTACGTGTGT	2142	1175	1	2	0	26
CDS1	GTACCAACGTACGTGTCA	649	425	2	0	0	0
CDS1	CATGCAACACACCACATG	1118	342	2	1	0	3
CDS2	GTTGCAACACACCAACGT	507	569	1	0	1	0
CDS2	GTACCAACGTACGTACAC	7	14	0	0	0	0
CDS2	TGACCAACGTACGTACTG	538	423	0	0	0	0
CDS2	ACACCAACGTACGTTGGT	70	63	0	0	0	0
CDS2	TGACCAACGTACGTTGCA	100	223	0	0	0	0
CDS2	CAACCAACGTACGTTGAC	24	34	0	0	0	0
CDT1	TGACTGTGGTGTGTACGT	112	158	0	0	0	0
CDT1	GTACTGTGGTGTGTACCA	88	209	0	0	0	0
CDT1	ACACTGTGGTGTGTACAC	106	70	0	0	0	0
CDT1	CATGCAGTTGTGACTGAC	22	27	0	0	0	0
CDT1	GTTGCAGTTGTGACTGTG	98	166	0	0	0	0
CDT1	ACTGCAACCACAACTGTG	287	272	1	0	0	0
CDX1	ACACGTCAGTTGCAGTAC	287	188	1	1	0	1
CDX1	CAACGTCAGTTGCAGTTG	609	632	0	1	0	1
CDX1	GTACGTCAGTTGCACAGT	675	702	1	1	0	0
CDX1	CAACGTCAGTTGCACACA	28	98	0	0	0	0
CDX1	TGACGTCAGTTGCACAAC	19	31	0	0	0	0
CDX2	CAACGTCATGGTACTGGT	89	329	0	0	0	0
CDX2	ACACGTCATGGTACTGCA	109	105	1	0	0	0
CDX2	GTACGTCATGGTACTGAC	111	77	0	0	0	0
CDX2	GTTGCAGTACACCACAAC	40	50	0	0	0	0
CDX2	TGTGCAGTACACCACATG	278	367	0	0	0	3
CDX2	ACTGCAACTGCAACCAAC	12	19	0	0	0	0
CDX4	TGACGTCATGGTACTGTG	479	594	1	0	0	0
CDX4	CAACGTCATGGTTGGTGT	641	887	1	0	0	0
CDX4	ACACGTCATGGTTGGTCA	128	189	0	0	0	0
CDX4	GTACGTCATGGTTGGTAC	41	94	0	1	0	0
CDX4	CATGCACACATGTGGTAC	67	59	0	0	0	0
CDX4	GTTGCACACATGTGGTTG	107	87	0	0	0	0
CEACAM1	ACACTGGTGTTGACACTG	240	312	22	0	0	0
CEACAM1	CAACTGGTGTTGACTGGT	294	445	3	1	0	0
CEACAM1	ACACTGGTGTTGACTGCA	290	310	0	0	0	0
CEACAM1	GTACTGGTGTTGACTGAC	75	25	0	0	0	0
CEACAM1	TGACTGGTGTTGACTGTG	413	303	0	0	0	1
CEBPA	ACACGTGTACGTGTGTTG	381	334	0	0	0	0
CEBPA	CAACGTGTACGTGTCAGT	38	12	0	0	0	0
CEBPA	ACACGTGTACGTGTCACA	67	93	1	0	0	0
CEBPA	GTACGTGTACGTGTCAAC	36	35	0	0	0	0
CEBPA	TGTGCAGTACGTGTGTAC	183	146	0	0	0	1
CEBPA	TGTGCATGGTCATGGTAC	94	65	0	0	0	0
CEBPB	ACACGTGTACCAGTCATG	296	169	0	0	0	0
CEBPB	CAACGTGTACCAGTACGT	249	648	0	0	0	0
CEBPB	ACACGTGTACCAGTACCA	913	592	0	0	0	1
CEBPB	GTACGTGTACCAGTACAC	13	16	0	0	0	0
CEBPB	TGACGTGTACCAGTACTG	147	182	0	0	0	0
CEBPB	ACTGCATGGTCATGGTTG	285	315	0	0	0	0
CEBPD	ACACGTCATGGTTGCAGT	270	499	1	0	1	1
CEBPD	TGACGTCATGGTTGGTTG	207	300	0	0	0	0
CEBPD	TGACGTCATGGTTGCACA	67	69	0	0	0	0
CEBPD	CAACGTCATGGTTGCAAC	27	28	0	0	0	0
CEBPD	GTTGCAGTACACCATGCA	208	218	1	0	0	1
CEL	TGACACCAGTCAACCACA	35	32	0	0	0	0
CEL	CAACACCAGTCAACCAAC	30	18	0	0	0	0
CEL	GTACACCAGTCAACCATG	134	97	0	0	0	0
CEL	TGACACCAGTCAACACGT	15	41	0	0	0	0

CEL	GTACACCAGTCAACACCA	57	236	0	0	0	0
CELSR1	CAACGTGTTGTGGTTGCA	175	180	0	0	1	2
CELSR1	GTACGTGTTGTGGTTGGT	836	504	1	0	0	0
CELSR1	TGACGTGTTGTGGTTGAC	40	36	0	0	0	0
CELSR1	ACACGTGTTGTGGTTGTG	139	162	0	0	0	0
CELSR1	CATGCAGTCAGTCAGTGT	1045	586	1	0	0	0
CELSR1	GTTGCACAACACTGTGTG	302	533	4	1	0	0
CELSR2	TGACGTGTTGTGCAGTAC	62	60	0	0	2	0
CELSR2	TGTGCACATGTGCAACGT	63	61	0	1	1	11
CELSR2	GTACGTGTTGTGCAGTGT	241	238	0	1	0	1
CELSR2	CAACGTGTTGTGCAGTCA	365	332	0	0	0	1
CELSR2	ACACGTGTTGTGCAGTTG	382	303	1	0	0	1
CELSR2	GTTGCACATGTGCAACCA	42	49	0	0	0	0
CELSR3	CATGCAGTACCAACTGCA	250	75	0	0	2	0
CELSR3	CAACGTGTTGTGCACAGT	276	123	0	0	0	12
CELSR3	ACACGTGTTGTGCACACA	129	266	1	0	0	1
CELSR3	GTACGTGTTGTGCACAAC	36	25	0	0	0	0
CELSR3	GTTGCAGTACCAACTGGT	773	442	1	1	0	0
CENTA1	TGTGGTGTCACAACACCA	409	318	0	0	0	0
CENTA1	GTTGGTTGCACAACGTCA	325	306	0	2	0	19
CENTA1	ACTGGTTGCACAACGTAC	36	181	0	0	0	0
CENTA1	CATGGTTGCACAACGTTG	1001	515	0	0	0	3
CENTA1	GTTGGTTGCACAACCAGT	246	260	0	1	0	0
CENTA1	GTTGCACAACACCACATG	553	171	0	2	0	140
CENTD1	CATGGTTGCACAACCACA	45	54	0	0	0	0
CENTD1	TGTGGTTGCACAACCAAC	4	15	0	0	0	0
CENTD1	ACTGGTTGCACAACCATG	87	89	0	0	0	0
CENTD1	CATGGTTGCACAACACGT	38	124	0	0	0	0
CENTD1	ACTGGTTGCACAACACCA	509	135	1	1	0	0
CENTD1	GTTGCAACCATGACTGCA	395	159	0	0	0	0
CENTD2	TGACACCACAGTACACTG	1033	504	4	0	8	2
CENTD2	ACACACCACAGTACTGGT	344	144	1	0	0	0
CENTD2	TGACACCACAGTACTGCA	219	282	0	0	0	0
CENTD2	CAACACCACAGTACTGAC	59	40	0	0	0	2
CENTD2	GTACACCACAGTACTGTG	617	604	2	2	0	0
CENTD3	GTACACCAGTTGGTCACA	115	121	0	0	1	0
CENTD3	GTACACCAGTTGGTGTTG	550	231	0	0	0	0
CENTD3	TGACACCAGTTGGTCAGT	30	34	0	0	0	0
CENTD3	ACACACCAGTTGGTCAAC	42	39	0	0	0	0
CENTD3	CAACACCAGTTGGTCATG	56	78	0	0	0	0
CEP290	CATGGTCATGCATGTGCA	218	280	2	5	0	7
CEP290	TGTGGTCATGCATGTGAC	152	95	0	0	0	0
CEP290	ACTGGTCATGCATGTGTG	327	313	0	1	0	0
CEP290	ACTGGTCATGACGTGTGT	314	286	0	0	0	0
CEP290	TGTGGTCATGACGTGTCA	134	148	0	0	0	0
CER1	GTTGGTACCAACGTGTTG	561	340	0	0	0	3
CER1	TGTGGTACCAACGTCAGT	234	266	1	0	0	0
CER1	GTTGGTACCAACGTCACA	251	135	0	69	0	0
CER1	ACTGGTACCAACGTCAAC	13	15	0	0	0	0
CER1	CATGGTACCAACGTCATG	209	258	0	0	0	0
CERK	ACACCAACACTGACTGTG	413	432	0	0	1	0
CERK	TGACCAACACTGACTGAC	17	11	0	0	0	0
CERK	GTACCAACACTGTGGTGT	519	690	1	0	0	0
CERK	CAACCAACACTGTGGTCA	425	461	0	1	0	6
CERK	TGACCAACACTGTGGTAC	254	197	1	0	0	0
CES1	ACTGACCATGGTGTACCA	166	185	1	0	0	0
CES1	GTTGACCATGGTGTACAC	164	38	0	0	0	0
CES1	TGTGACCATGGTGTACTG	252	370	0	1	0	1
CES1	ACTGACCATGGTGTTGGT	654	244	0	0	0	0
CES1	TGTGACCATGGTGTTGCA	137	99	1	0	0	0
CES2	GTACACCAGTCACACACA	66	82	0	1	0	0
CES2	ACACACCAGTCACACAAC	12	26	1	0	0	0
CES2	CAACACCAGTCACACATG	230	747	0	0	0	1
CES2	GTACACCAGTCACAACGT	233	341	0	0	0	0
CES2	GTTGCAGTCACATGTGAC	52	47	0	0	0	0
CES4	TGACACCAGTCAGTCAAC	42	68	0	0	0	0
CES4	TGACACCAGTCATGGTAC	43	14	0	0	0	0
CES4	ACACACCAGTCATGGTTG	197	130	0	0	0	0
CES4	CAACACCAGTCATGCAGT	210	232	0	0	0	0
CES4	ACACACCAGTCATGCACA	161	152	1	0	0	0
CES4	CATGCACAACTGTGGTCA	45	25	0	0	0	0
CES7	GTACACCAGTGTCACAGT	265	124	1	0	0	2
CES7	CAACACCAGTGTCACACA	382	407	0	0	0	0
CES7	TGACACCAGTGTCACAAC	31	40	0	0	0	0
CES7	ACACACCAGTGTCACATG	179	98	0	0	0	0

CES7	ACTGCAGTTGGTTGGTTG	275	544	0	0	0	0
CES7	GTTGCAACACGTACTGCA	71	69	0	0	0	0
CETN2	ACACACTGCAGTCATGCA	319	499	1	0	0	0
CETN2	GTACACTGCAGTCATGAC	481	335	0	0	0	1
CETN2	TGACACTGCAGTCATGTG	282	229	1	0	0	0
CETN2	CAACACTGCAGTACGTGT	298	462	0	1	0	1
CETN2	ACACACTGCAGTACGTCA	41	75	0	0	0	0
CETN2	CATGCACACAACTGTGCA	78	42	0	0	0	0
CETP	TGTGGTGTGTCAGTGTTG	1104	1458	1	3	2	1
CETP	ACTGGTGTGTCAGTCAGT	539	245	0	0	1	12
CETP	TGTGGTGTGTCAGTCACA	47	77	1	0	0	0
CETP	CATGGTGTGTCAGTCAAC	20	17	0	0	0	0
CETP	GTTGGTGTGTCAGTCATG	150	241	1	2	0	0
CETP	GTTGCACACACATGCAGT	1228	1050	1	8	0	3
CFB	TGCATGACTGCACAACAC	69	102	0	0	0	0
CFB	ACCATGACTGCACAACTG	140	140	0	1	0	0
CFB	CACATGACTGCACATGGT	563	513	0	0	0	2
CFB	ACCATGACTGCACATGCA	262	300	0	1	0	0
CFB	GTCATGACTGCACATGAC	33	12	0	0	0	0
CFD	ACACACTGTGCAGTACCA	173	169	1	0	0	0
CFD	GTACACTGTGCAGTACAC	94	58	0	0	0	0
CFD	TGACACTGTGCAGTACTG	397	648	0	2	0	0
CFD	GTTGCAGTTGCACACATG	78	63	0	0	0	0
CFD	TGTGACGTGTCACATGTG	14	10	0	0	0	0
CFH	CAACACTGTGGTGTCAGT	230	292	2	0	0	0
CFH	ACACACTGTGGTGTCACA	115	57	1	0	0	0
CFH	GTACACTGTGGTGTCAAC	39	22	1	0	0	0
CFH	TGACACTGTGGTGTCATG	48	33	0	0	0	0
CFH	ACACACTGTGGTGTACGT	78	121	0	0	0	0
CFHR1	CATGGTTGCAGTGTTGGT	367	418	12	9	4	6
CFHR1	GTTGGTTGCAGTGTACGT	554	332	0	1	0	1
CFHR1	CATGGTTGCAGTGTACCA	90	112	0	2	0	0
CFHR1	TGTGGTTGCAGTGTACAC	241	50	0	0	0	1
CFHR1	ACTGGTTGCAGTGTACTG	304	158	0	0	0	0
CFHR2	TGTGGTACACACACGTCA	477	365	1	14	2	2
CFHR2	GTTGGTACACACACGTTG	825	721	1	1	2	0
CFHR2	ACTGGTACACACACGTGT	317	705	0	0	0	0
CFHR2	CATGGTACACACACGTAC	53	13	0	0	0	0
CFHR2	TGTGGTACACACACCAGT	246	344	2	0	0	0
CFHR3	ACTGGTACACACGTGTTG	534	1187	1	1	0	0
CFHR3	CATGGTACACACGTCAGT	221	399	1	1	0	0
CFHR3	ACTGGTACACACGTCACA	198	287	0	0	0	16
CFHR3	GTTGGTTGCACAACACAC	55	48	1	0	0	0
CFHR3	TGTGGTTGCACAACACTG	323	237	0	0	0	0
CFHR4	CATGGTACACCAACGTCA	325	285	0	0	0	0
CFHR4	TGTGGTACACCAACGTAC	47	46	1	0	0	0
CFHR4	ACTGGTACACCAACGTTG	385	359	0	0	0	0
CFHR4	CATGGTACACCAACCAGT	280	295	1	1	0	5
CFHR4	TGTGGTACACACGTGTAC	24	134	0	0	0	0
CFHR5	ACTGGTACACTGGTACAC	3	14	0	0	0	0
CFHR5	CATGGTACACTGGTACTG	1419	1532	0	0	0	0
CFHR5	GTTGGTACACTGGTTGGT	315	211	0	0	0	0
CFHR5	CATGGTACACTGGTTGCA	246	237	0	0	0	0
CFHR5	TGTGGTACACTGGTTGAC	17	26	0	0	0	0
CFI	CAACACCATGTGCAGTTG	761	980	0	1	1	0
CFI	ACACACCATGTGCAGTAC	27	13	0	0	0	0
CFI	ACTGACACCAACACTGCA	312	198	0	1	0	0
CFI	GTTGACACCAACACTGAC	320	101	0	0	0	0
CFI	TGTGACACCAACACTGTG	1090	646	1	0	0	0
CFL1	GTACCAGTTGACCACACA	71	109	0	0	0	1
CFL1	ACACCAGTTGACCACAAC	10	30	0	0	0	0
CFL1	CAACCAGTTGACCACATG	537	586	2	0	0	1
CFL1	GTACCAGTTGACCAACGT	588	330	1	1	0	0
CFL1	CAACCAGTTGACCAACCA	372	355	0	0	0	0
CFL2	ACACCAGTTGACCAACTG	865	1086	1	0	1	1
CFL2	TGACCAGTTGACCAACAC	98	69	0	1	0	0
CFL2	CAACCAGTTGACCATGGT	704	857	0	1	0	1
CFL2	ACACCAGTTGACCATGCA	451	715	1	0	0	0
CFL2	GTACCAGTTGACCATGAC	9	22	0	0	0	0
CFLAR	GTACGTGTCATGCACAGT	364	265	0	0	0	0
CFLAR	CAACGTGTCATGCACACA	71	75	0	0	0	0
CFLAR	TGACGTGTCATGCACAAC	20	16	0	0	0	0
CFLAR	ACACGTGTCATGCACATG	356	626	0	6	0	7
CFLAR	CATGGTTGACCATGGTCA	138	143	0	0	0	0
CFLAR	GTTGCAACACGTACGTGT	104	61	0	0	0	0

CFP	GTTGGTGTGTACTGACTG	341	590	0	0	0	0
CFP	TGTGGTGTGTACTGTGGT	305	229	0	0	0	0
CFP	GTTGGTGTGTACTGTGCA	125	70	0	0	0	0
CFP	ACTGGTGTGTACTGTGAC	58	55	0	0	0	0
CFP	CATGGTGTGTACTGTGTG	592	964	1	1	0	1
CFP	TGTGCACACAGTGTTGCA	210	219	2	0	0	0
CFTR	GTACTGTGTGGTCAGTTG	1297	377	1	0	1	0
CFTR	TGACTGTGTGGTCAGTCA	533	356	1	1	0	3
CFTR	CAACTGTGTGGTCAGTAC	23	14	0	0	0	0
CFTR	TGACTGTGTGGTCACAGT	119	236	1	0	0	0
CFTR	GTACTGTGTGGTCACACA	19	24	0	0	0	0
CGN	ACCATGTGACTGCATGGT	106	82	0	0	0	0
CGN	TGCATGTGACTGCATGCA	101	107	1	0	0	0
CGN	CACATGTGACTGCATGAC	33	21	0	0	0	0
CGN	GTCATGTGACTGCATGTG	351	434	3	0	0	5
CGN	ACCATGTGACTGACGTGT	441	1087	0	0	0	1
CGN	GTTGCAACGTACGTACTG	439	160	0	1	0	1
CHAD	ACTGGTTGACTGGTACTG	395	770	2	0	1	0
CHAD	TGTGGTTGACTGGTACAC	4	35	0	0	0	0
CHAD	CATGGTTGACTGGTTGGT	666	526	1	2	0	1
CHAD	ACTGGTTGACTGGTTGCA	128	134	0	0	0	0
CHAD	GTTGGTTGACTGGTTGAC	19	32	0	0	0	2
CHAF1A	GTACTGTGGTACTGCAGT	25	48	0	0	0	0
CHAF1A	CAACTGTGGTACTGCACA	99	81	0	0	0	0
CHAF1A	TGACTGTGGTACTGCAAC	32	94	0	0	0	0
CHAF1A	ACACTGTGGTACTGCATG	96	320	0	0	0	0
CHAF1A	CAACTGTGGTACTGACGT	223	311	0	0	0	0
CHAT	ACACCACATGGTACGTCA	139	136	0	0	0	0
CHAT	GTACCACATGGTACGTAC	5	13	0	0	0	0
CHAT	TGACCACATGGTACGTTG	126	87	0	0	0	0
CHAT	ACACCACATGGTACCAGT	136	140	0	1	0	1
CHAT	TGACCACATGGTACCACA	294	223	0	0	0	0
CHD8	CAACGTACCATGTGCACA	135	82	1	0	0	0
CHD8	TGACGTACCATGTGCAAC	22	26	0	0	0	0
CHD8	ACACGTACCATGTGCATG	30	424	0	0	0	0
CHD8	CAACGTACCATGTGACGT	116	114	0	0	0	0
CHD8	ACACGTACCATGTGACCA	107	146	0	0	0	0
CHDH	TGACCAGTGTTGTGGTGT	444	240	0	0	0	0
CHDH	GTACCAGTGTTGTGGTCA	153	195	0	0	0	1
CHDH	ACACCAGTGTTGTGGTAC	197	185	0	0	0	79
CHDH	CAACCAGTGTTGTGGTTG	881	1031	2	0	0	0
CHDH	GTACCAGTGTTGTGCAGT	67	139	0	0	0	0
CHDH	TGTGCATGGTGTGTACCA	65	69	0	0	0	0
CHEK1	TGCATGCAACGTCACAAC	12	15	0	0	0	0
CHEK1	ACCATGCAACGTCACATG	271	307	1	1	0	0
CHEK1	CACATGCAACGTCAACGT	134	117	0	0	0	0
CHEK1	ACCATGCAACGTCAACCA	104	118	0	0	0	0
CHEK1	GTCATGCAACGTCAACAC	289	184	1	0	0	0
CHEK2	GTACCATGCAACCATGCA	102	71	0	0	0	13
CHEK2	ACACCATGCAACCATGAC	73	90	1	0	0	0
CHEK2	CAACCATGCAACCATGTG	801	704	2	0	0	0
CHEK2	TGACCATGCAACACGTGT	315	447	0	0	0	0
CHEK2	TGACTGTGACACACTGCA	256	240	2	1	0	0
CHGA	CAACACTGCAGTTGCAGT	58	122	0	0	0	0
CHGA	ACACACTGCAGTTGCACA	31	49	0	0	0	0
CHGA	GTACACTGCAGTTGCAAC	59	239	1	0	0	0
CHGA	TGACACTGCAGTTGCATG	180	180	0	0	0	0
CHGA	GTTGGTACTGCAACACAC	32	61	0	0	0	0
CHGB	CATGGTTGCACAACTGAC	41	28	0	0	1	0
CHGB	TGTGCACAGTCATGTGTG	54	47	0	0	1	0
CHGB	ACTGGTTGCACAACTGGT	203	222	1	0	0	0
CHGB	TGTGGTTGCACAACTGCA	50	42	0	0	0	0
CHGB	GTTGGTTGCACAACTGTG	548	405	1	0	0	0
CHGB	ACTGGTTGCACATGGTGT	1112	499	2	0	0	1
CHIA	ACTGGTGTACGTTGCAAC	37	44	0	0	0	0
CHIA	ACTGGTACGTACACACCA	103	178	0	0	0	0
CHIA	GTTGGTACGTACACACAC	61	76	0	0	0	0
CHIA	TGTGGTACGTACACACTG	72	70	0	0	0	0
CHIA	ACTGGTACGTACACTGGT	114	167	1	0	0	24
CHKA	GTCATGTGTGACACGTGT	554	676	0	0	0	1
CHKA	CACATGTGTGACACGTCA	40	31	0	0	0	0
CHKA	TGCATGTGTGACACGTAC	90	87	0	0	0	0
CHKA	ACCATGTGTGACACGTTG	239	73	1	0	0	0
CHKA	CACATGTGTGACACCAGT	317	316	0	0	0	0
CHKB	TGACACACTGCAGTACAC	35	39	0	0	0	0

CHKB	ACACACACTGCAGTACTG	304	984	0	0	0	0
CHKB	CAACACACTGCAGTTGGT	840	422	0	1	0	0
CHKB	ACACACACTGCAGTTGCA	227	300	0	0	0	0
CHKB	CAACCAACCAACCAGTTG	354	531	0	0	0	4
CHMP1B	CATGGTGTCACAGTACCA	1181	942	0	1	0	0
CHMP1B	ACTGGTTGGTGTGTCAGT	190	181	0	0	0	0
CHMP1B	TGTGGTTGGTGTGTCACA	64	54	0	0	0	0
CHMP1B	CATGGTTGGTGTGTCAAC	17	19	0	0	0	0
CHMP1B	GTTGGTTGGTGTGTCATG	144	575	0	0	0	0
CHN1	ACACACCACAGTACCATG	449	243	0	0	1	0
CHN1	CAACACCACAGTACGTTG	715	285	0	1	0	0
CHN1	GTACACCACAGTACCAGT	89	77	0	0	0	0
CHN1	CAACACCACAGTACCACA	203	41	0	0	0	0
CHN1	TGACACCACAGTACCAAC	107	53	0	0	0	0
CHP	GTACACTGGTACCACACA	37	40	0	0	0	0
CHP	ACACACTGGTACCACAAC	15	28	0	0	0	0
CHP	CAACACTGGTACCACATG	122	143	0	0	0	0
CHP	GTACACTGGTACCAACGT	214	277	0	0	0	0
CHP	ACTGGTTGTGCACATGTG	782	673	2	2	0	3
CHPF	CATGGTTGTGACACTGGT	577	238	0	1	0	0
CHPF	ACTGGTTGTGACACTGCA	58	55	0	0	0	0
CHPF	GTTGGTTGTGACACTGAC	13	26	0	0	0	0
CHPF	TGTGGTTGTGACACTGTG	871	776	0	0	0	0
CHPF	CATGGTTGTGACTGGTGT	26	51	0	0	0	0
CHPT1	TGACCAACGTACTGTGGT	290	292	0	2	0	0
CHPT1	GTACCAACGTACTGTGCA	116	98	1	0	0	0
CHPT1	ACACCAACGTACTGTGAC	20	32	0	1	0	0
CHPT1	CAACCAACGTACTGTGTG	284	333	0	1	0	0
CHPT1	GTACCAACGTTGGTGTGT	2249	991	3	0	0	8
CHRD	GTACTGCAGTCAGTCACA	103	182	0	0	1	0
CHRD	TGACGTTGTGTGGTGTAC	150	53	0	0	0	0
CHRD	ACACTGCAGTCAGTCAAC	29	41	0	0	0	0
CHRD	CAACTGCAGTCAGTCATG	162	199	1	1	0	0
CHRD	GTACTGCAGTCAGTACGT	189	146	0	0	0	0
CHRD	TGTGCATGGTACACTGCA	419	257	0	0	0	0
CHRM1	TGACGTGTTGTGCACATG	90	70	0	0	1	0
CHRM1	GTACGTGTTGTGCAACTG	548	518	0	1	1	0
CHRM1	ACACGTGTTGTGCAACGT	393	413	0	250	0	1
CHRM1	TGACGTGTTGTGCAACCA	184	168	0	1	0	0
CHRM1	CAACGTGTTGTGCAACAC	57	51	0	0	0	0
CHRM2	GTTGCAGTACCAACACCA	332	255	0	31	1	33
CHRM2	TGACGTGTTGTGCATGGT	439	635	1	1	0	0
CHRM2	GTACGTGTTGTGCATGCA	361	107	0	0	0	0
CHRM2	ACACGTGTTGTGCATGAC	132	45	0	1	0	0
CHRM2	TGTGCAGTACCAACACGT	133	41	0	0	0	0
CHRM4	TGACGTGTTGTGACGTGT	236	577	0	0	1	0
CHRM4	CAACGTGTTGTGCATGTG	1058	1381	2	1	0	0
CHRM4	GTACGTGTTGTGACGTCA	58	96	1	0	0	0
CHRM4	ACACGTGTTGTGACGTAC	7	6	0	0	0	0
CHRM4	CAACGTGTTGTGACGTTG	224	319	0	0	0	3
CHRM5	GTACGTGTTGTGACCAGT	136	115	0	0	0	0
CHRM5	CAACGTGTTGTGACCACA	159	85	0	0	0	0
CHRM5	TGACGTGTTGTGACCAAC	29	104	0	0	0	0
CHRM5	ACACGTGTTGTGACCATG	53	80	0	0	0	0
CHRM5	CAACGTGTTGTGACACGT	145	21	0	0	0	0
CHRNA1	TGACTGCAACCATGCAGT	160	67	0	0	0	0
CHRNA1	GTACTGCAACCATGCACA	155	168	7	0	0	0
CHRNA1	ACACTGCAACCATGCAAC	31	153	0	0	0	0
CHRNA1	CAACTGCAACCATGCATG	128	125	0	0	0	0
CHRNA1	GTACTGCAACCATGACGT	269	167	0	0	0	0
CHRNA10	CAACTGCAACCACAACAC	40	39	0	0	0	0
CHRNA10	GTACTGCAACCACAACTG	73	96	0	0	0	0
CHRNA10	TGACTGCAACCACATGGT	939	216	1	0	0	0
CHRNA10	GTACTGCAACCACATGCA	154	152	0	0	0	0
CHRNA10	ACACTGCAACCACATGAC	30	30	0	0	0	0
CHRNA10	TGTGCACATGACGTGTTG	529	330	1	0	0	0
CHRNA2	TGTGCACAGTACGTGTGT	468	512	3	1	1	0
CHRNA2	ACACTGCAACCATGTGCA	140	220	1	1	0	0
CHRNA2	GTACTGCAACCATGTGAC	66	125	1	0	0	0
CHRNA2	TGACTGCAACCATGTGTG	194	164	0	0	0	0
CHRNA2	TGACTGCAACACGTGTGT	877	851	0	0	0	0
CHRNA2	GTACTGCAACACGTGTCA	555	475	0	0	0	6
CHRNA3	ACACTGCAACACGTGTAC	27	36	0	0	1	0
CHRNA3	CAACTGCAACACGTGTTG	544	482	2	0	0	4
CHRNA3	GTACTGCAACACGTCAGT	523	479	0	1	0	1

CHRNA3	CAACTGCAACACGTCACA	101	112	0	0	0	1
CHRNA3	TGACTGCAACACGTCAAC	112	16	0	0	0	1
CHRNA3	TGTGCAACCACAGTTGCA	555	170	0	0	0	0
CHRNA4	TGACTGCAACACGTACTG	1077	831	1	1	1	7
CHRNA4	ACACTGCAACACGTCATG	99	76	1	0	0	0
CHRNA4	CAACTGCAACACGTACGT	207	222	1	0	0	0
CHRNA4	ACACTGCAACACGTACCA	300	635	2	0	0	1
CHRNA4	GTACTGCAACACGTACAC	20	25	0	0	0	0
CHRNA5	ACACTGCAACACGTTGGT	635	398	0	0	1	1
CHRNA5	GTACTGCAACACGTTGTG	1421	1212	0	1	1	0
CHRNA5	GTTGCAGTCAACTGGTGT	910	382	0	0	1	24
CHRNA5	TGACTGCAACACGTTGCA	166	165	0	0	0	4
CHRNA5	CAACTGCAACACGTTGAC	44	83	0	0	0	0
CHRNA6	TGACTGCAACGTGTCAGT	48	31	0	0	0	0
CHRNA6	GTACTGCAACGTGTCACA	18	22	0	0	0	0
CHRNA6	ACACTGCAACGTGTCAAC	11	17	0	0	0	0
CHRNA6	CAACTGCAACGTGTCATG	165	91	0	0	0	0
CHRNA6	GTACTGCAACGTGTACGT	109	112	0	0	0	0
CHRNA6	TGTGCAACACGTGTTGTG	86	137	0	0	0	1
CHRNA7	ACACTGCAACACCACAAC	114	47	0	0	0	0
CHRNA7	CAACTGCAACACCACATG	279	290	0	0	0	21
CHRNA7	GTACTGCAACACCAACGT	725	962	2	0	0	0
CHRNA7	CAACTGCAACACCAACCA	214	218	0	0	0	0
CHRNA7	TGTGGTGTCACACAACGT	80	48	1	0	0	0
CHRNA9	ACACTGACGTTGACGTTG	191	188	1	0	2	0
CHRNA9	TGACTGACGTTGACGTAC	29	34	0	0	0	0
CHRNA9	CAACTGACGTTGACCAGT	175	202	0	0	0	0
CHRNA9	ACACTGACGTTGACCACA	76	53	0	0	0	4
CHRNA9	GTACTGACGTTGACCAAC	6	20	0	0	0	0
CHRNB1	TGACTGCAACACACGTTG	862	476	3	4	1	0
CHRNB1	TGACTGCAACACCATGTG	917	226	0	0	0	2
CHRNB1	CAACTGCAACACACGTGT	1291	964	18	0	0	0
CHRNB1	ACACTGCAACACACGTCA	204	199	0	0	0	0
CHRNB1	GTACTGCAACACACGTAC	78	62	0	0	0	1
CHRNB2	TGACTGCAACACACACGT	84	76	0	0	0	1
CHRNB2	GTACTGCAACACACACCA	664	659	0	0	0	0
CHRNB2	ACACTGCAACACACACAC	20	40	0	0	0	0
CHRNB2	CAACTGCAACACACACTG	483	642	1	1	0	0
CHRNB2	GTTGCACAGTACGTGTCA	262	171	0	0	0	0
CHRNB2	ACTGCACAGTACGTGTAC	40	61	0	0	0	0
CHRNB3	GTACTGCAACACTGACTG	126	368	0	0	1	0
CHRNB3	ACACTGCAACACTGACGT	183	253	1	28	0	0
CHRNB3	TGACTGCAACACTGACCA	207	183	0	0	0	0
CHRNB3	CAACTGCAACACTGACAC	44	35	0	0	0	0
CHRNB3	TGACTGCAACACTGTGGT	299	697	0	2	0	0
CHRND	CAACTGCAACTGCAGTTG	132	435	0	0	0	0
CHRND	GTACTGCAACTGCACAGT	439	185	0	0	0	0
CHRND	CAACTGCAACTGCACACA	302	172	0	1	0	0
CHRND	TGACTGCAACTGCACAAC	17	13	0	0	0	0
CHRND	ACACTGCAACTGCACATG	261	295	0	1	0	1
CHRND	CATGCACACACATGCACA	10	11	0	0	0	0
CHRNE	TGACTGCAACTGACACAC	41	92	1	0	0	1
CHRNE	ACACTGCAACTGACACTG	158	150	0	0	0	0
CHRNE	CAACTGCAACTGACTGGT	153	319	1	0	0	52
CHRNE	ACACTGCAACTGACTGCA	187	239	0	0	0	2
CHRNE	GTACTGCAACTGACTGAC	90	71	0	0	0	0
CHRNG	CAACTGCAACTGTGACTG	842	907	1	0	3	3
CHRNG	GTACTGCAACTGTGACCA	134	184	0	1	0	0
CHRNG	ACACTGCAACTGTGACAC	123	69	0	0	0	0
CHRNG	GTACTGCAACTGTGTGGT	534	337	0	1	0	0
CHRNG	CAACTGCAACTGTGTGCA	84	110	0	0	0	0
CHST1	ACACCACACAACTGGTTG	1368	860	2	1	1	1
CHST1	ACACCACACAACTGCACA	171	138	0	0	1	0
CHST1	CAACCACACAACTGCAGT	191	264	0	0	0	0
CHST1	GTACCACACAACTGCAAC	125	112	0	0	0	0
CHST1	TGTGACACACCAACACAC	101	109	0	0	0	0
CHST11	ACACCAACGTACACACAC	49	32	0	0	0	0
CHST11	CAACCAACGTACACACTG	1990	1564	3	0	0	5
CHST11	GTACCAACGTACACTGGT	67	61	0	0	0	0
CHST11	CAACCAACGTACACTGCA	132	190	0	0	0	0
CHST11	TGACCAACGTACACTGAC	60	119	0	0	0	0
CHST12	CAACCAACGTTGGTGTCA	588	491	1	1	0	0
CHST12	TGACCAACGTTGGTGTAC	74	203	0	13	0	0
CHST12	ACACCAACGTTGGTGTTG	569	216	2	0	0	0
CHST12	CAACCAACGTTGGTCAGT	153	212	0	0	0	0

CHST12	ACACCAACGTTGGTCACA	88	112	1	0	0	1
CHST13	TGACCAACCAACCATGCA	145	79	0	0	0	0
CHST13	CAACCAACCAACCATGAC	41	239	0	0	0	0
CHST13	GTACCAACCAACCATGTG	471	800	2	0	0	0
CHST13	CATGCAGTACTGTGCACA	275	87	0	0	0	0
CHST13	TGTGCAGTACTGTGCAAC	27	17	0	0	0	0
CHST13	CATGCAACACTGTGGTGT	429	619	2	1	0	0
CHST14	TGACCAACCACATGGTGT	316	391	0	0	0	1
CHST14	GTACCAACCACATGGTCA	235	777	1	0	0	1
CHST14	ACACCAACCACATGGTAC	70	113	0	0	0	1
CHST14	CAACCAACCACATGGTTG	736	961	3	0	0	0
CHST14	ACTGCAGTACTGTGGTAC	102	278	2	1	0	0
CHST14	GTTGCATGCAGTGTGTAC	139	160	0	0	0	10
CHST2	TGACCACACATGGTACCA	63	129	1	0	0	1
CHST2	CAACCACACATGGTACAC	66	108	0	0	0	1
CHST2	GTACCACACATGGTACTG	400	370	1	0	0	0
CHST2	TGACCACACATGGTTGGT	289	366	0	0	0	0
CHST2	CATGCAGTCACATGACCA	398	250	0	0	0	0
CHST3	CATGCAGTCATGGTGTCA	286	324	2	1	1	2
CHST3	CAACCAACGTGTACACAC	33	24	0	0	0	0
CHST3	GTACCAACGTGTACACTG	275	351	1	0	0	0
CHST3	TGACCAACGTGTACTGGT	86	221	1	0	0	0
CHST3	GTACCAACGTGTACTGCA	182	130	0	0	0	0
CHST3	CATGCAACCACACAACCA	620	981	0	1	0	0
CHST4	GTACCACAACGTGTACTG	67	72	0	0	0	0
CHST4	TGACCACAACGTGTTGGT	130	142	0	0	0	0
CHST4	GTACCACAACGTGTTGCA	22	16	0	0	0	0
CHST4	ACACCACAACGTGTTGAC	100	123	0	0	0	0
CHST4	CAACCACAACGTGTTGTG	1220	733	0	0	0	0
CHST4	TGTGCACAACGTGTACGT	197	128	1	0	0	0
CHST6	TGACCAACCACAGTCAGT	131	177	0	0	0	2
CHST6	GTACCAACCACAGTCACA	39	88	0	0	0	0
CHST6	ACTGCAGTACTGACTGAC	230	21	0	0	0	0
CHST6	CATGCAGTACTGACTGTG	148	241	0	0	0	0
CHST6	CATGACACGTGTGTGTAC	409	85	0	0	0	0
CHST7	GTACCACAACACGTCATG	202	221	2	1	0	0
CHST7	TGACCACAACACGTACGT	235	378	0	0	0	11
CHST7	GTACCACAACACGTACCA	165	267	0	8	0	0
CHST7	ACACCACAACACGTACAC	87	60	0	0	0	1
CHST7	CAACCACAACACGTACTG	586	801	0	0	0	1
CHST7	CATGCAACTGGTCACAGT	88	74	0	0	0	1
CHSY1	GTACACGTCACAACTGGT	96	105	0	0	0	0
CHSY1	CAACACGTCACAACTGCA	630	547	1	0	0	8
CHSY1	TGACACGTCACAACTGAC	46	65	0	0	0	0
CHSY1	ACACACGTCACAACTGTG	509	852	0	1	0	2
CHSY1	GTTGCAGTTGGTCATGAC	27	52	0	0	0	0
CHUK	TGCATGCAACGTCAACTG	611	534	0	2	0	2
CHUK	ACCATGCAACGTCATGGT	160	316	16	23	0	0
CHUK	TGCATGCAACGTCATGCA	107	39	0	0	0	0
CHUK	CACATGCAACGTCATGAC	119	76	0	0	0	0
CHUK	GTCATGCAACGTCATGTG	54	55	0	0	0	0
CIDEB	TGTGGTCAACTGTGACGT	502	594	0	2	0	4
CIDEB	GTTGGTCAACTGTGACCA	217	161	0	1	0	0
CIDEB	ACTGGTCAACTGTGACAC	243	57	0	0	0	0
CIDEB	CATGGTCAACTGTGACTG	102	138	1	0	0	0
CIDEB	GTTGGTCAACTGTGTGGT	458	236	1	0	0	0
CIITA	CAACGTACTGCAACTGGT	463	162	0	1	1	0
CIITA	ACACGTACTGCAACTGCA	809	648	0	0	0	0
CIITA	GTACGTACTGCAACTGAC	17	6	0	0	0	0
CIITA	TGACGTACTGCAACTGTG	218	278	0	0	0	2
CIITA	CATGGTTGTGGTCACATG	147	172	0	1	0	0
CIP29	CATGGTCACAGTGTGTCA	242	408	0	0	0	0
CIP29	CATGGTACACCAACACAC	60	48	0	0	0	0
CIP29	GTTGGTACACCAACACTG	43	22	0	0	0	0
CIP29	TGTGGTACACCAACTGGT	186	127	0	1	0	0
CIP29	GTTGGTACACCAACTGCA	31	52	0	0	0	0
CIR	GTTGGTGTCATGTGGTTG	590	629	11	11	1	3
CIR	TGTGGTGTCATGTGCAGT	274	88	0	0	0	0
CIR	GTTGGTGTCATGTGCACA	104	81	0	0	0	0
CIR	ACTGGTGTCATGTGCAAC	7	16	0	0	0	8
CIR	CATGGTGTCATGTGCATG	23	266	0	0	0	0
CIRBP	GTACGTACACCAGTCATG	30	22	0	0	0	0
CIRBP	TGACGTACACCAGTACGT	95	108	0	0	0	0
CIRBP	GTACGTACACCAGTACCA	152	132	0	0	0	0
CIRBP	ACACGTACACCAGTACAC	82	64	0	0	0	0

CIRBP	CAACGTACACCAGTACTG	185	261	0	0	0	0
CIRBP	CATGCACAGTACGTGTTG	594	695	0	0	0	0
CISH	TGACACTGACGTGTGTTG	490	382	0	0	0	0
CISH	ACACACTGACGTGTCAGT	125	121	0	0	0	0
CISH	TGACACTGACGTGTCACA	47	165	0	1	0	38
CISH	CAACACTGACGTGTCAAC	51	32	0	0	0	0
CISH	CATGGTTGTGGTACTGCA	41	22	0	0	0	0
CISH	GTTGCAACACCACATGCA	467	341	0	0	0	11
CIT	ACACGTGTGTCACAACCA	233	208	0	0	0	0
CIT	GTACGTGTGTCACAACAC	118	104	0	0	0	0
CIT	TGACGTGTGTCACAACTG	151	130	1	1	0	0
CIT	ACACGTGTGTCACATGGT	327	606	1	0	0	0
CIT	ACTGCAGTGTTGCACAAC	18	22	0	1	0	0
CITED1	TGACGTACTGGTCAACCA	84	88	0	0	0	0
CITED1	CAACGTACTGGTCAACAC	46	44	0	0	0	0
CITED1	GTACGTACTGGTCAACTG	139	518	0	0	0	0
CITED1	TGACGTACTGGTCATGGT	579	156	1	0	0	0
CITED1	GTACGTACTGGTCATGCA	190	139	0	0	0	0
CITED1	GTTGCAACCAACCAGTGT	475	491	0	0	0	5
CITED2	GTACGTACCACACACACA	205	209	1	2	1	2
CITED2	ACACGTACCACACACAAC	41	51	0	0	0	1
CITED2	CAACGTACCACACACATG	170	210	0	0	0	0
CITED2	GTACGTACCACACAACGT	289	612	0	0	0	1
CITED2	CAACGTACCACACAACCA	326	320	0	0	0	0
CKB	ACCATGTGTGACACCACA	120	163	0	1	0	1
CKB	GTCATGTGTGACACCAAC	23	29	0	0	0	1
CKB	TGCATGTGTGACACCATG	336	318	0	1	0	0
CKB	ACCATGTGTGACACACGT	49	52	0	0	0	0
CKB	CACATGTGTGACTGACGT	270	181	0	0	0	0
CKM	GTTGGTACTGACGTTGTG	319	533	0	0	2	2
CKM	TGTGGTACTGACCAGTCA	728	872	0	4	1	0
CKM	ACTGGTACTGACCAGTGT	96	73	3	0	0	0
CKM	CATGGTACTGACCAGTAC	87	44	0	0	0	0
CKM	GTTGGTACTGACCAGTTG	682	504	0	1	0	7
CKM	CATGCAACACCAGTGTGT	755	993	1	2	0	0
CKMT1A	TGCATGTGTGACACACCA	67	55	0	0	0	0
CKMT1A	CACATGTGTGACACACAC	139	56	0	0	0	0
CKMT1A	GTCATGTGTGACACACTG	179	286	0	0	0	0
CKMT1A	TGCATGTGTGACACTGGT	99	64	0	0	0	0
CKMT1A	GTCATGTGTGACACTGCA	273	94	0	0	0	0
CKMT1B	ACCATGTGTGACTGACCA	139	139	1	0	0	2
CKMT1B	GTCATGTGTGACTGACAC	51	33	0	0	0	0
CKMT1B	TGCATGTGTGACTGACTG	109	205	0	0	0	0
CKMT1B	ACCATGTGTGACTGTGGT	108	122	0	0	0	0
CKMT1B	TGCATGTGTGACTGTGCA	292	345	3	0	0	0
CKMT2	CACATGTGTGACTGTGAC	8	16	0	0	0	0
CKMT2	GTCATGTGTGACTGTGTG	424	626	81	0	0	0
CKMT2	TGCATGTGTGTGGTGTGT	335	744	1	0	0	0
CKMT2	TGTGGTTGACACACACTG	550	156	0	0	0	0
CKMT2	ACTGGTTGACACACTGGT	385	1092	2	1	0	133
CKS1B	ACACCAACTGCAACCAGT	935	954	2	1	1	0
CKS1B	GTACGTTGTGACACCATG	199	226	0	0	0	0
CKS1B	TGACCAACTGCAACGTTG	275	353	0	0	0	0
CKS1B	TGACCAACTGCAACCACA	167	159	0	0	0	0
CKS1B	CAACCAACTGCAACCAAC	10	6	0	0	0	0
CKS1B	GTTGCACAGTACGTCAGT	38	59	0	0	0	4
CLC	GTACACCAACCATGCAGT	397	733	1	1	1	4
CLC	GTACACCAACCATGGTCA	92	61	0	0	0	0
CLC	ACACACCAACCATGGTAC	31	55	1	0	0	0
CLC	CAACACCAACCATGGTTG	58	84	1	0	0	3
CLC	CAACACCAACCATGCACA	216	171	1	1	0	0
CLC	TGTGCAACCATGGTGTCA	497	751	1	4	0	2
CLCA1	TGACACGTGTACCAACTG	1224	1112	1	1	1	3
CLCA1	GTACACGTGTACCAACAC	8	22	0	0	0	0
CLCA1	ACACACGTGTACCATGGT	478	654	0	1	0	4
CLCA1	TGACACGTGTACCATGCA	259	273	0	0	0	0
CLCA1	CAACACGTGTACCATGAC	167	74	0	0	0	0
CLCA2	GTACCATGTGTGACGTAC	53	84	0	0	0	0
CLCA2	TGACCATGTGTGACGTTG	98	407	0	0	0	0
CLCA2	ACACCATGTGTGACCAGT	76	90	0	0	0	0
CLCA2	TGACCATGTGTGACCACA	483	102	0	0	0	0
CLCA2	CAACCATGTGTGACCAAC	106	33	0	1	0	0
CLCA4	TGACCATGTGTGTGGTAC	51	56	0	0	0	1
CLCA4	ACACCATGTGTGTGGTTG	641	486	1	0	0	0
CLCA4	CAACCATGTGTGTGCAGT	308	345	3	0	0	10

CLCA4	ACACCATGTGTGTGCACA	56	59	0	0	0	0
CLCA4	GTACCATGTGTGTGCAAC	19	25	0	0	0	0
CLCA4	ACTGCACATGACACGTAC	87	88	0	0	0	0
CLCF1	TGTGGTTGCACATGGTCA	430	285	0	0	1	0
CLCF1	TGTGGTCATGGTCATGGT	369	371	4	0	0	0
CLCF1	GTTGGTCATGGTCATGCA	587	252	0	0	0	0
CLCF1	ACTGGTCATGGTCATGAC	37	34	0	0	0	0
CLCF1	CATGGTCATGGTCATGTG	413	273	0	0	0	38
CLCN2	ACTGCACAACCACATGGT	290	178	1	0	1	0
CLCN2	GTACACGTGTACCATGTG	468	431	0	0	0	0
CLCN2	ACACACGTGTACACGTGT	128	135	0	0	0	0
CLCN2	TGACACGTGTACACGTCA	95	47	0	0	0	0
CLCN2	GTTGCACAACCACAACAC	113	103	0	0	0	6
CLCN2	TGTGCACAACCACAACTG	826	438	1	0	0	2
CLCN7	ACACACGTGTTGGTCAGT	106	138	3	7	5	10670
CLCN7	TGTGCAGTCAACCAGTCA	321	531	1	0	1	1
CLCN7	TGACACGTGTTGGTCACA	80	142	1	0	0	0
CLCN7	CAACACGTGTTGGTCAAC	5	6	0	0	0	0
CLCN7	GTACACGTGTTGGTCATG	309	236	1	0	0	0
CLDN1	ACTGGTGTTGACCACACA	48	50	0	0	0	0
CLDN1	GTTGGTGTTGACCACAAC	5	8	0	0	0	0
CLDN1	TGTGGTGTTGACCACATG	46	46	0	0	0	0
CLDN1	ACTGGTGTTGACCAACGT	313	372	1	2	0	0
CLDN1	TGTGGTGTTGACCAACCA	342	346	0	0	0	0
CLDN1	TGTGCAACACCAGTGTTG	578	668	1	1	0	0
CLDN10	TGTGGTGTACTGTGACCA	368	487	0	0	1	1
CLDN10	GTTGGTGTACTGTGCAAC	9	23	0	0	0	0
CLDN10	TGTGGTGTACTGTGCATG	570	267	5	2356	0	1
CLDN10	ACTGGTGTACTGTGACGT	168	185	0	0	0	0
CLDN10	ACTGCATGTGGTTGGTAC	60	45	0	0	0	1
CLDN11	CATGGTGTTGGTCACACA	55	98	1	2	1	0
CLDN11	GTTGGTGTTGGTCACAGT	36	87	0	0	0	0
CLDN11	TGTGGTGTTGGTCACAAC	36	43	0	0	0	0
CLDN11	ACTGGTGTTGGTCACATG	1145	1006	2	1	0	4
CLDN11	ACTGCACAGTGTCATGTG	370	312	0	1	0	1
CLDN14	GTACTGTGACTGTGTGTG	688	780	2	4	2	1
CLDN14	CAACTGTGACTGTGTGAC	78	38	0	0	0	0
CLDN14	TGACTGTGTGGTGTGTGT	499	472	1	0	0	1
CLDN14	GTACTGTGTGGTGTGTCA	220	144	0	2	0	0
CLDN14	GTTGCACAGTGTGTACGT	164	170	0	0	0	0
CLDN14	ACTGCAACACCAGTGTCA	441	421	6	0	0	1
CLDN15	CATGCACAACTGGTGTTG	1247	325	9	0	1	5
CLDN15	ACTGGTGTTGCATGGTTG	106	101	0	0	0	0
CLDN15	TGTGCACAACTGGTGTGT	303	622	1	0	0	1
CLDN15	GTTGCACAACTGGTGTCA	41	75	0	0	0	0
CLDN15	ACTGCACAACTGGTGTAC	125	305	1	1	0	0
CLDN15	GTTGCACAACTGGTCAGT	254	477	0	0	0	0
CLDN16	CATGGTGTTGCATGGTCA	581	683	2	1	2	1
CLDN16	TGTGGTGTTGCAACTGAC	181	46	0	0	0	0
CLDN16	ACTGGTGTTGCAACTGTG	421	357	0	0	0	0
CLDN16	GTTGGTGTTGCATGGTGT	1644	1588	3	1	0	0
CLDN16	TGTGGTGTTGCATGGTAC	40	116	0	0	0	0
CLDN16	GTTGCAACACCAGTGTAC	61	93	1	0	0	0
CLDN17	CATGGTGTTGACGTTGCA	109	166	0	0	1	0
CLDN17	GTTGGTGTTGACGTTGGT	95	156	0	0	0	1
CLDN17	TGTGGTGTTGACGTTGAC	73	72	0	0	0	0
CLDN17	ACTGGTGTTGACGTTGTG	192	245	1	0	0	0
CLDN17	GTTGGTGTTGACCAGTGT	337	218	0	0	0	0
CLDN17	GTTGCACAACACCATGGT	176	307	1	0	0	0
CLDN18	CATGGTGTTGGTCAACGT	173	212	0	0	0	0
CLDN18	ACTGGTGTTGGTCAACCA	233	236	0	1	0	22
CLDN18	GTTGGTGTTGGTCAACAC	40	33	1	0	0	0
CLDN18	TGTGGTGTTGGTCAACTG	564	298	0	0	0	8
CLDN18	ACTGCACAGTGTACCACA	54	63	0	0	0	0
CLDN19	TGTGGTGTTGGTACACAC	7	32	0	0	0	2
CLDN19	ACTGGTGTTGGTACACTG	335	962	1	0	0	0
CLDN19	CATGGTGTTGGTACTGGT	517	306	1	0	0	0
CLDN19	ACTGGTGTTGGTACTGCA	113	119	0	0	0	0
CLDN19	GTTGGTGTTGGTACTGAC	75	88	0	0	0	0
CLDN19	TGTGCAACACACTGTGGT	572	469	1	0	0	0
CLDN2	ACTGGTGTACGTACACCA	147	178	0	0	0	0
CLDN2	GTTGGTGTACGTACACAC	27	19	0	0	0	0
CLDN2	TGTGGTGTACGTACACTG	168	413	0	0	0	0
CLDN2	ACTGGTGTACGTACTGGT	35	21	0	0	0	0
CLDN2	TGTGCACAGTGTCAACGT	265	212	0	0	0	0

CLDN20	ACTGGTGTTGCAGTTGGT	157	202	0	0	0	0
CLDN20	TGTGGTGTTGCAGTTGCA	204	300	0	0	0	0
CLDN20	CATGGTGTTGCAGTTGAC	15	20	0	0	0	0
CLDN20	GTTGGTGTTGCAGTTGTG	556	199	0	1	0	0
CLDN20	ACTGGTGTTGCACAGTGT	281	344	0	1	0	0
CLDN22	TGTGACCAACTGCAGTTG	1166	1518	0	1	1	0
CLDN22	ACTGCAGTGTACCACAGT	34	39	0	1	0	0
CLDN22	TGTGCAGTGTACCACACA	265	183	4	1	0	0
CLDN22	CATGCAGTGTACCACAAC	2	3	0	0	0	0
CLDN22	GTTGCAGTGTACCACATG	455	670	0	0	0	0
CLDN23	CATGGTTGCACATGGTAC	116	74	0	0	0	0
CLDN23	GTTGGTTGCACATGGTTG	1261	1231	2	1	0	2
CLDN23	TGTGGTTGCACATGCAGT	62	72	0	0	0	0
CLDN23	GTTGGTTGCACATGCACA	207	428	1	0	0	1
CLDN23	ACTGGTTGCACATGCAAC	22	36	0	0	0	0
CLDN5	ACTGGTGTACTGACCAGT	196	283	0	0	1	24
CLDN5	TGTGGTGTACTGACCACA	174	130	0	0	0	0
CLDN5	CATGGTGTACTGACCAAC	36	26	0	0	0	0
CLDN5	GTTGGTGTACTGACCATG	346	427	1	0	0	0
CLDN5	CATGGTTGTGACCACACA	240	306	0	1	0	0
CLDN7	CATGGTGTTGACCACAGT	227	218	0	1	2	9
CLDN7	TGTGGTGTCAGTGTACTG	150	243	0	0	0	0
CLDN7	TGTGGTGTTGACCAGTAC	595	201	2	1	0	0
CLDN7	ACTGGTGTTGACCAGTTG	280	663	0	0	0	0
CLDN7	TGTGCACAGTGTCATGAC	27	12	0	0	0	0
CLEC10A	ACACACTGGTCAGTACAC	51	59	0	0	0	0
CLEC10A	CAACACTGGTCAGTACTG	270	771	0	0	0	0
CLEC10A	GTACACTGGTCAGTTGGT	464	738	3	1	0	1
CLEC10A	CAACACTGGTCAGTTGCA	366	127	0	0	0	0
CLEC10A	CATGCAGTTGCAACTGTG	616	593	1	1	0	0
CLEC5A	ACTGCATGCAGTTGACTG	433	800	3	0	1	0
CLEC5A	TGACACTGGTACTGTGGT	19	40	0	0	0	0
CLEC5A	GTACACTGGTACTGTGCA	156	138	0	0	0	0
CLEC5A	ACACACTGGTACTGTGAC	23	36	0	0	0	0
CLEC5A	CAACACTGGTACTGTGTG	498	341	0	0	0	11
CLEC5A	ACTGCAGTTGCATGACCA	108	141	0	0	0	0
CLEC7A	TGCATGTGACCACATGGT	225	229	1	0	0	0
CLEC7A	GTCATGTGACCACATGCA	90	132	0	0	0	0
CLEC7A	ACCATGTGACCACATGAC	17	6	0	0	0	0
CLEC7A	GTTGACCAACTGCATGGT	497	145	0	0	0	0
CLEC7A	CATGACCAACTGCATGCA	189	168	0	1	0	1
CLIC1	CATGGTCATGACCAACTG	102	154	1	0	1	0
CLIC1	GTTGGTGTTGTGCACATG	128	176	1	1	0	0
CLIC1	GTTGGTCATGACCATGGT	309	495	0	0	0	2
CLIC1	CATGGTCATGACCATGCA	326	379	0	0	0	0
CLIC1	TGTGGTCATGACCATGAC	79	68	1	0	0	0
CLK1	ACCATGCAACTGCAGTCA	339	207	0	0	0	0
CLK1	GTCATGCAACTGCAGTAC	20	6	0	0	0	0
CLK1	TGCATGCAACTGCAGTTG	723	980	2	0	0	1
CLK1	ACCATGCAACTGCACAGT	22	64	0	1	0	0
CLK1	TGTGACCAGTTGACACGT	197	42	0	0	0	0
CLK2	ACCATGCAACTGGTTGCA	473	118	0	0	0	0
CLK2	GTCATGCAACTGGTTGAC	103	81	0	0	0	0
CLK2	TGCATGCAACTGGTTGTG	167	230	0	1	0	0
CLK2	CACATGCAACTGCAGTGT	628	277	0	0	0	0
CLK2	CAACGTTGACCATGGTTG	959	696	1	2	0	0
CLK3	GTCATGCAACTGGTACGT	518	455	0	0	0	0
CLK3	CACATGCAACTGGTACCA	26	37	0	0	0	0
CLK3	TGCATGCAACTGGTACAC	8	20	0	0	0	0
CLK3	ACCATGCAACTGGTACTG	205	200	1	1	0	0
CLK3	CACATGCAACTGGTTGGT	1237	764	2	2	0	0
CLK3	CATGCACAGTACGTCACA	246	68	0	0	0	0
CLK4	CACATGCATGTGGTCAAC	14	14	0	0	0	0
CLK4	GTCATGCATGTGGTCATG	1195	331	2	0	0	2
CLK4	TGCATGCATGTGGTACGT	44	81	1	0	0	0
CLK4	GTCATGCATGTGGTACCA	264	325	0	1	0	0
CLK4	GTTGGTTGACTGTGACAC	119	136	0	0	0	1
CLN3	TGACTGTGTGGTCATGTG	294	482	2	0	0	0
CLN3	CAACTGTGTGGTACGTGT	575	1013	2	1	0	0
CLN3	ACACTGTGTGGTACGTCA	55	92	0	0	0	0
CLN3	GTACTGTGTGGTACGTAC	12	13	0	0	0	0
CLN3	TGACTGTGTGGTACGTTG	1472	685	2	0	0	4
CLOCK	GTACGTACTGCAGTCAAC	12	7	0	0	0	0
CLOCK	TGACGTACTGCAGTCATG	73	87	0	0	0	1
CLOCK	ACACGTACTGCAGTACGT	140	208	0	0	0	1

CLOCK	TGACGTACTGCAGTACCA	205	191	0	0	0	0
CLOCK	CAACGTACTGCAGTACAC	53	46	1	0	0	0
CLOCK	TGTGCAACTGACACCATG	31	59	0	0	0	0
CLTA	GTACACTGCAGTTGTGCA	16	29	0	0	0	0
CLTA	ACACACTGCAGTTGTGAC	42	46	0	0	0	0
CLTA	CAACACTGCAGTTGTGTG	131	187	0	0	0	0
CLTA	ACACACTGCACAGTGTGT	502	225	0	0	0	1
CLTA	TGACACTGCACAGTGTCA	61	72	0	0	0	0
CLTB	TGACACTGCACAGTTGTG	414	469	1	1	1	0
CLTB	ACACACTGCACAGTACTG	231	209	0	0	0	0
CLTB	CAACACTGCACAGTTGGT	560	459	1	0	0	0
CLTB	ACACACTGCACAGTTGCA	168	158	0	0	0	0
CLTB	GTACACTGCACAGTTGAC	31	30	0	0	0	0
CLTB	ACTGCATGCACATGACAC	36	82	0	0	0	0
CLTC	ACTGGTGTACGTTGTGCA	293	253	2	0	1	0
CLTC	TGACGTGTTGGTGTCAAC	7	2	0	0	0	0
CLTC	ACACGTGTTGGTGTCATG	192	347	0	1	0	0
CLTC	CAACGTGTTGGTGTACGT	53	39	2	0	0	0
CLTC	ACACGTGTTGGTGTACCA	369	227	0	1	0	2
CLTCL1	ACACTGTGTGGTCACAAC	10	73	1	0	0	0
CLTCL1	CAACTGTGTGGTCACATG	110	159	1	0	0	0
CLTCL1	GTACTGTGTGGTCAACGT	653	788	0	2	0	0
CLTCL1	CAACTGTGTGGTCAACCA	319	269	0	0	0	59
CLTCL1	TGACTGTGTGGTCAACAC	53	71	1	0	0	0
CLU	GTACTGTGACACCAGTGT	218	234	0	0	1	1
CLU	CAACTGTGACACCAGTCA	100	52	1	0	0	0
CLU	TGACTGTGACACCAGTAC	31	49	0	0	0	0
CLU	ACACTGTGACACCAGTTG	684	502	0	1	0	0
CLU	CAACTGTGACACCACAGT	175	128	0	0	0	0
CLYBL	ACACTGTGCATGACTGCA	223	309	0	0	0	0
CLYBL	GTACTGTGCATGACTGAC	44	39	0	0	0	0
CLYBL	TGACTGTGCATGACTGTG	348	401	59	0	0	0
CLYBL	CAACTGTGCATGTGGTGT	690	775	5	0	0	16
CLYBL	ACACTGTGCATGTGGTCA	614	378	1	1	0	1
CMA1	ACTGCAACACCATGACTG	2069	1296	2	0	1	1
CMA1	CACATGACTGACCACAGT	193	200	0	0	0	0
CMA1	ACCATGACTGACCACACA	246	221	0	2	0	0
CMA1	GTCATGACTGACCACAAC	34	24	0	0	0	0
CMA1	TGCATGACTGACCACATG	389	343	0	0	0	0
CMA1	ACCATGACTGACCAACGT	73	76	0	0	0	0
CMAS	GTACACGTCACAACCATG	893	838	2	2	0	1
CMAS	TGACACGTCACAACACGT	101	46	0	0	0	0
CMAS	GTACACGTCACAACACCA	45	87	0	0	0	0
CMAS	ACACACGTCACAACACAC	110	94	0	0	0	1
CMAS	CAACACGTCACAACACTG	149	487	1	0	0	0
CMAS	ACTGCAACACACGTGTAC	36	8	0	0	0	0
CMBL	CAACACACGTCATGACTG	591	357	0	0	0	0
CMBL	GTACACACGTCATGTGGT	345	237	0	0	0	0
CMBL	CAACACACGTCATGTGCA	185	204	0	0	0	0
CMBL	TGACACACGTCATGTGAC	57	71	1	0	0	0
CMBL	ACACACACGTCATGTGTG	122	85	0	0	0	0
CMKLR1	ACACGTGTTGTGACACCA	100	49	0	0	0	0
CMKLR1	GTACGTGTTGTGACACAC	32	61	0	0	0	0
CMKLR1	TGACGTGTTGTGACACTG	320	318	0	1	0	0
CMKLR1	ACACGTGTTGTGACTGGT	59	76	0	0	0	0
CMKLR1	TGACGTGTTGTGACTGCA	270	209	0	0	0	0
CMKLR1	ACTGCACACAACTGTGTG	706	60	0	0	0	0
CNDP1	TGACACCATGACGTACTG	1004	500	1	2	1	2
CNDP1	ACACACCATGACGTTGGT	724	555	8	1	0	3
CNDP1	TGACACCATGACGTTGCA	45	29	0	0	0	0
CNDP1	CAACACCATGACGTTGAC	87	80	0	0	0	0
CNDP1	GTTGACCAGTGTCACAAC	21	22	0	0	0	0
CNGA3	ACACCATGTGTGGTTGGT	423	366	2	0	0	0
CNGA3	TGACCATGTGTGGTTGCA	128	156	0	0	0	0
CNGA3	CAACCATGTGTGGTTGAC	22	14	0	0	0	0
CNGA3	GTACCATGTGTGGTTGTG	661	481	0	1	0	0
CNGA3	CATGACACCATGCACAGT	73	81	0	1	0	0
CNGA4	CATGGTTGCACATGCATG	165	238	1	0	0	0
CNGA4	GTTGGTTGCACATGACGT	244	244	0	0	0	0
CNGA4	CATGGTTGCACATGACCA	243	381	0	0	0	0
CNGA4	TGTGGTTGCACATGACAC	23	29	0	0	0	0
CNGA4	TGTGACCAACTGTGCAAC	30	66	0	0	0	0
CNGB1	GTACCATGTGACTGGTCA	219	217	0	0	1	1
CNGB1	TGACCATGTGACTGGTGT	358	837	1	0	0	139
CNGB1	ACACCATGTGACTGGTAC	71	83	0	0	0	0

CNGB1	CAACCATGTGACTGGTTG	390	268	2	1	0	0
CNGB1	GTACCATGTGACTGCAGT	263	275	1	0	0	0
CNGB1	TGTGCACAGTACGTCAAC	11	20	0	0	0	0
CNP	CATGCAGTGTTGACGTGT	893	1268	3	1	0	0
CNP	ACTGCAGTGTTGACGTCA	138	93	0	0	0	0
CNP	GTTGCAGTGTTGACGTAC	34	39	0	0	0	0
CNP	TGTGCAGTGTTGACGTTG	326	648	1	1	0	0
CNP	ACTGCAGTGTTGACCAGT	494	533	1	2	0	0
CNPY3	GTTGGTCAACCAGTCATG	238	218	0	1	1	0
CNPY3	CATGGTCAACCAGTCAAC	76	47	0	0	0	0
CNPY3	TGTGGTCAACCAGTACGT	45	111	0	0	0	0
CNPY3	GTTGGTCAACCAGTACCA	367	560	0	0	0	0
CNPY3	ACTGGTCAACCAGTACAC	12	15	0	0	0	0
CNPY4	ACTGGTACCAACACACGT	89	96	8	0	0	0
CNPY4	TGTGGTACCAACACACCA	580	343	0	0	0	0
CNPY4	CATGGTACCAACACACAC	154	50	0	0	0	0
CNPY4	GTTGGTACCAACACACTG	466	509	1	0	0	1
CNPY4	TGTGGTACCAACACTGGT	308	254	0	0	0	0
CNPY4	CATGCAACACTGACGTAC	17	25	0	0	0	0
CNR1	CAACGTGTTGTGACTGAC	49	43	0	0	0	0
CNR1	GTACGTGTTGTGACTGTG	277	812	0	1	0	0
CNR1	ACACGTGTTGTGTGGTGT	141	260	1	0	0	0
CNR1	TGACGTGTTGTGTGGTCA	95	238	0	0	0	0
CNR1	CAACGTGTTGTGTGGTAC	38	28	0	0	0	0
CNR2	GTACGTGTTGTGTGGTTG	370	362	0	1	0	0
CNR2	TGACGTGTTGTGTGCAGT	154	284	0	1	0	0
CNR2	GTACGTGTTGTGTGCACA	58	62	0	0	0	0
CNR2	CATGCAGTACCATGGTCA	347	554	0	0	0	2
CNR2	ACTGCACAGTACGTCATG	78	71	1	0	0	0
CNR2	CATGCACAGTACGTACGT	624	406	1	1	0	1
CNTF	ACACTGCAGTCATGACCA	739	577	1	1	1	10
CNTF	GTACTGCAGTCATGACAC	43	72	0	0	0	0
CNTF	TGACTGCAGTCATGACTG	154	285	0	0	0	0
CNTF	ACACTGCAGTCATGTGGT	46	70	0	0	0	0
CNTF	TGACTGCAGTCATGTGCA	211	191	1	0	0	0
CNTF	TGTGCAACTGTGGTGTGT	485	924	1	0	0	0
CNTFR	CAACTGCAGTACTGGTGT	178	283	0	0	0	0
CNTFR	ACACTGCAGTACTGGTCA	591	597	0	0	0	3
CNTFR	GTACTGCAGTACTGGTAC	80	53	1	0	0	0
CNTFR	TGACTGCAGTACTGGTTG	736	1087	2	0	0	4
CNTFR	ACACTGCAGTACTGCAGT	39	120	0	0	0	0
CNTFR	ACTGCAACACACTGCACA	194	145	0	0	0	0
CNTN1	TGACTGTGGTGTCAACCA	535	360	1	2	1	13
CNTN1	ACACCATGGTACTGCAGT	352	186	1	0	0	1
CNTN1	GTACTGTGGTGTCACAAC	26	25	0	0	0	0
CNTN1	TGACTGTGGTGTCACATG	278	239	4	0	0	0
CNTN1	ACACTGTGGTGTCAACGT	52	71	0	0	0	0
CNTN1	TGTGCATGGTCAGTCAAC	40	47	0	0	0	0
COASY	GTACACGTCATGGTTGAC	39	29	0	0	0	0
COASY	TGACACGTCATGGTTGTG	837	288	0	0	0	0
COASY	CAACACGTCATGCAGTGT	514	204	1	0	0	0
COASY	ACACACGTCATGCAGTCA	126	598	0	0	0	1
COASY	GTACACGTCATGCAGTAC	99	121	0	0	0	0
COL10A1	TGACTGTGACACTGTGTG	512	726	0	0	1	1
COL10A1	CAACTGTGACACTGTGGT	352	547	2	1	0	1
COL10A1	ACACTGTGACACTGTGCA	106	148	0	0	0	0
COL10A1	GTACTGTGACACTGTGAC	12	10	0	0	0	0
COL10A1	ACACTGTGACTGGTGTGT	280	146	0	0	0	1
COL11A1	ACACTGTGTGACACACTG	471	704	0	1	0	6
COL11A1	CAACTGTGTGACACTGGT	149	160	0	1	0	0
COL11A1	ACACTGTGTGACACTGCA	221	239	0	0	0	0
COL11A1	GTACTGTGTGACACTGAC	4	3	0	0	0	0
COL11A1	TGACTGTGTGACACTGTG	324	346	0	0	0	5
COL11A2	ACACTGTGTGGTTGTGAC	38	35	0	0	0	0
COL11A2	CAACTGTGTGGTTGTGTG	53	48	0	0	0	0
COL11A2	ACACTGTGTGCAGTGTGT	152	530	0	0	0	0
COL11A2	TGACTGTGTGCAGTGTCA	369	575	0	0	0	0
COL11A2	CAACTGTGTGCAGTGTAC	122	86	0	0	0	0
COL11A2	CATGCAACGTTGTGACCA	179	144	0	0	0	0
COL12A1	GTTGGTGTCAGTGTCAGT	141	164	0	0	0	0
COL12A1	TGTGGTGTACGTACGTGT	258	314	0	0	0	0
COL12A1	GTTGGTGTACGTACGTCA	406	234	0	0	0	0
COL12A1	ACTGGTGTACGTACGTAC	11	19	0	0	0	0
COL12A1	CATGGTGTACGTACGTTG	258	529	0	1	0	0
COL13A1	GTTGGTGTACTGGTCAGT	180	152	0	0	2	0

COL13A1	TGTGGTGTACTGGTGTGT	220	200	0	0	0	0
COL13A1	GTTGGTGTACTGGTGTCA	126	112	0	1	0	0
COL13A1	ACTGGTGTACTGGTGTAC	52	52	0	0	0	0
COL13A1	CATGGTGTACTGGTGTTG	245	225	0	0	0	0
COL13A1	ACTGCAACACACGTCATG	62	70	0	0	0	0
COL15A1	CATGGTACGTGTACTGGT	99	123	1	0	0	0
COL15A1	ACTGGTACGTGTACTGCA	95	118	0	1	0	0
COL15A1	GTTGGTACGTGTACTGAC	7	6	22	0	0	0
COL15A1	TGTGGTACGTGTACTGTG	134	150	0	0	0	0
COL15A1	TGTGACACCAGTACACGT	150	81	0	0	0	0
COL16A1	ACTGGTGTACGTCAGTTG	631	734	1	0	0	37
COL16A1	CATGGTGTACGTCACAGT	137	191	1	0	0	0
COL16A1	ACTGGTGTACGTCACACA	40	29	0	0	0	0
COL16A1	GTTGGTGTACGTCACAAC	19	25	0	0	0	0
COL16A1	ACTGCAGTCACACATGAC	51	61	0	0	0	0
COL17A1	CATGGTCAGTTGGTGTGT	1700	1823	8	1	1	7
COL17A1	ACTGGTCAGTACTGTGTG	92	81	0	0	0	0
COL17A1	ACTGGTCAGTTGGTGTCA	386	231	1	0	0	0
COL17A1	GTTGGTCAGTTGGTGTAC	205	117	0	0	0	0
COL17A1	TGTGGTCAGTTGGTGTTG	1167	917	10	2	0	104
COL18A1	TGTGGTCAGTACGTTGGT	363	255	0	0	1	0
COL18A1	GTTGGTCAGTACGTACTG	123	183	0	0	0	0
COL18A1	GTTGGTCAGTACGTTGCA	259	94	0	0	0	1
COL18A1	ACTGGTCAGTACGTTGAC	107	60	0	0	0	0
COL18A1	GTTGACACGTCAGTACCA	83	68	0	0	0	0
COL19A1	ACTGGTGTTGCATGTGAC	8	30	0	0	0	0
COL19A1	CATGGTGTTGCATGTGTG	326	273	2	0	0	0
COL19A1	CATGGTGTTGACGTGTGT	158	146	0	0	0	0
COL19A1	ACTGGTGTTGACGTGTCA	127	429	0	0	0	0
COL19A1	GTTGGTGTTGACGTGTAC	35	90	0	0	0	0
COL1A1	TGACTGACGTGTTGGTTG	623	538	1	0	0	2
COL1A1	ACACTGACGTGTTGCAGT	139	97	0	0	0	0
COL1A1	TGACTGACGTGTTGCACA	226	121	0	0	0	0
COL1A1	CAACTGACGTGTTGCAAC	73	48	0	0	0	0
COL1A1	TGTGCAGTTGACTGTGAC	218	144	0	0	0	0
COL1A2	GTACTGTGACTGTGACAC	182	36	0	0	0	0
COL1A2	TGACTGTGACTGTGACTG	328	275	0	0	0	0
COL1A2	ACACTGTGACTGTGTGGT	200	289	0	0	0	0
COL1A2	TGACTGTGACTGTGTGCA	194	210	0	0	0	0
COL1A2	TGTGACGTCAACCAACGT	170	187	0	0	0	0
COL21A1	GTTGGTGTTGGTTGACCA	494	617	1	1	1	7
COL21A1	GTTGGTGTTGGTTGCATG	77	111	0	0	0	0
COL21A1	TGTGGTGTTGGTTGACGT	212	272	1	0	0	0
COL21A1	ACTGGTGTTGGTTGACAC	19	44	0	0	0	0
COL21A1	CATGGTGTTGGTTGACTG	446	1087	0	4	0	0
COL21A1	TGTGCAACCAGTCAACTG	649	386	2	0	0	0
COL22A1	GTTGGTTGTGTGTGCACA	114	243	0	0	1	48
COL22A1	ACTGGTTGTGTGTGCAAC	26	73	0	1	0	0
COL22A1	CATGGTTGTGTGTGCATG	488	172	0	0	0	1
COL22A1	GTTGGTTGTGTGTGACGT	39	79	0	0	0	16
COL22A1	CATGGTTGTGTGTGACCA	287	743	1	0	0	0
COL23A1	GTTGGTCACAGTGTGTGT	621	877	2	0	1	1
COL23A1	GTTGCAGTGTCAACCACA	254	229	0	0	1	0
COL23A1	CATGCAGTGTCAACGTAC	2	96	0	0	0	0
COL23A1	GTTGCAGTGTCAACGTTG	378	505	1	0	0	6
COL23A1	TGTGCAGTGTCAACCAGT	431	321	1	0	0	1
COL23A1	GTTGCATGGTACCATGCA	150	111	0	0	0	0
COL24A1	CATGGTGTTGCAACGTGT	143	166	0	0	0	0
COL24A1	ACTGGTGTTGCAACGTCA	28	42	0	0	0	0
COL24A1	GTTGGTGTTGCAACGTAC	54	72	1	0	0	0
COL24A1	TGTGGTGTTGCAACGTTG	474	632	0	0	0	0
COL24A1	ACTGGTGTTGCAACCAGT	86	96	0	0	0	0
COL25A1	TGTGGTGTGTTGTGACGT	72	74	1	0	0	0
COL25A1	GTTGGTGTACTGCAGTTG	147	445	0	0	0	0
COL25A1	TGTGGTGTACTGCACAGT	159	149	0	0	0	0
COL25A1	GTTGGTGTACTGCACACA	20	12	0	0	0	0
COL25A1	ACTGGTGTACTGCACAAC	40	20	0	0	0	0
COL27A1	CATGGTGTTGGTGTGTCA	246	134	0	0	0	0
COL27A1	TGTGGTGTTGGTGTGTAC	426	149	0	0	0	0
COL27A1	ACTGGTGTTGGTGTGTTG	726	236	2	1	0	0
COL27A1	CATGGTGTTGGTGTCAGT	229	139	0	0	0	0
COL27A1	TGTGCATGCACAGTTGGT	155	92	0	0	0	0
COL27A1	GTTGCATGCACAGTTGCA	107	125	0	0	0	0
COL2A1	ACTGGTGTGTGTCAGTCA	549	601	1	2	0	0
COL2A1	GTTGGTGTGTGTCAGTAC	153	35	0	0	0	0

COL2A1	TGTGGTGTGTGTCAGTTG	345	423	0	0	0	0
COL2A1	ACTGGTGTGTGTCACAGT	231	158	0	0	0	0
COL2A1	TGTGGTGTGTGTCACACA	43	37	0	0	0	0
COL2A1	TGTGCAACGTCAACGTAC	76	83	1	0	0	0
COL3A1	CACATGACACACTGCACA	205	142	1	1	0	0
COL3A1	TGCATGACACACTGCAAC	48	50	0	0	0	0
COL3A1	ACCATGACACACTGCATG	513	434	1	0	0	1
COL3A1	CACATGACACACTGACGT	174	167	0	0	0	0
COL3A1	ACCATGACACACTGACCA	154	275	1	0	0	0
COL4A1	GTTGGTGTACGTACCAGT	489	524	0	1	0	2
COL4A1	CATGGTGTACGTACCACA	354	95	0	0	0	0
COL4A1	TGTGGTGTACGTACCAAC	28	11	0	0	0	0
COL4A1	ACTGGTGTACGTACCATG	219	492	0	0	0	0
COL4A1	CATGGTGTACGTACACGT	37	53	0	0	0	0
COL4A2	TGTGGTGTACGTGTTGAC	29	43	0	0	0	0
COL4A2	ACTGGTGTACGTGTTGTG	31	95	1	1	0	0
COL4A2	GTTGGTGTACGTCAGTGT	498	205	0	0	0	0
COL4A2	CATGGTGTACGTCAGTCA	203	279	0	0	0	2
COL4A2	TGTGGTGTACGTCAGTAC	64	118	0	0	0	0
COL4A3	CAACTGTGTGTGCAGTCA	210	215	3	0	0	7
COL4A3	TGACTGTGTGTGCAGTAC	43	21	0	0	0	0
COL4A3	GTTGACCATGGTACTGGT	9	12	0	0	0	0
COL4A3	CATGACCATGGTACTGCA	135	152	0	0	0	0
COL4A3	TGTGACCATGGTACTGAC	59	69	0	0	0	0
COL4A3BP	TGACGTGTGTGTACGTGT	985	322	3	0	2	0
COL4A3BP	TGACGTGTGTGTCATGGT	190	221	0	0	0	0
COL4A3BP	GTACGTGTGTGTCATGCA	122	91	0	0	0	1
COL4A3BP	ACACGTGTGTGTCATGAC	24	30	0	0	0	0
COL4A3BP	CAACGTGTGTGTCATGTG	54	110	0	0	0	0
COL4A3BP	CATGCACAACGTGTCAAC	9	7	0	0	0	0
COL4A4	TGTGGTGTTGCATGACCA	103	269	0	2	0	0
COL4A4	CATGGTGTTGCATGACAC	12	22	0	0	0	0
COL4A4	GTTGGTGTTGCATGACTG	419	457	0	0	0	2
COL4A4	TGTGGTGTTGCATGTGGT	600	153	0	0	0	0
COL4A4	GTTGGTGTTGCATGTGCA	258	303	0	0	0	0
COL4A5	CAACTGTGACTGCACAAC	40	43	0	0	0	0
COL4A5	GTACTGTGACTGCACATG	117	130	0	0	0	0
COL4A5	TGACTGTGACTGCAACGT	161	234	0	0	0	0
COL4A5	GTACTGTGACTGCAACCA	405	574	1	1	0	0
COL4A5	ACACTGTGACTGCAACAC	18	33	0	0	0	0
COL4A5	ACTGCAACGTACGTACGT	15	11	0	0	0	0
COL4A6	TGTGGTGTGTTGGTTGGT	78	89	0	0	0	0
COL4A6	GTTGGTGTGTTGGTTGCA	82	62	0	1	0	0
COL4A6	ACTGGTGTGTTGGTTGAC	51	24	0	0	0	0
COL4A6	CATGGTGTGTTGGTTGTG	483	273	0	0	0	0
COL4A6	CATGCAGTCAGTTGGTTG	286	250	0	0	0	0
COL4A6	GTTGCAACGTACCACAGT	185	232	0	1	0	0
COL5A1	ACACTGTGACTGGTCAAC	47	52	0	0	0	0
COL5A1	CAACTGTGACTGGTCATG	146	127	0	0	0	0
COL5A1	GTACTGTGACTGGTACGT	185	175	2	0	0	0
COL5A1	CAACTGTGACTGGTACCA	179	216	0	0	0	0
COL5A1	TGACTGTGACTGGTACAC	7	12	0	0	0	0
COL5A2	TGTGGTGTGTACCACAGT	60	48	0	0	0	0
COL5A2	GTTGGTGTGTACCACACA	39	29	0	0	0	0
COL5A2	ACTGGTGTGTACCACAAC	5	9	0	0	0	0
COL5A2	GTTGGTGTCAGTGTGTCA	63	50	0	0	0	0
COL5A2	GTTGCACAGTGTGTTGAC	8	14	0	0	0	0
COL5A3	GTTGGTGTTGCACAACGT	583	788	3	0	1	0
COL5A3	ACTGGTGTTGCACACAAC	21	92	0	0	0	0
COL5A3	CATGGTGTTGCACACATG	124	128	1	0	0	6
COL5A3	CATGGTGTTGCACAACCA	154	190	1	0	0	1
COL5A3	TGTGGTGTTGCACAACAC	31	39	0	0	0	0
COL6A1	ACTGGTGTTGCAGTGTAC	28	31	1	0	0	0
COL6A1	CATGGTGTTGCAGTGTTG	509	395	1	1	0	0
COL6A1	GTTGGTGTTGCAGTCAGT	385	385	1	0	0	0
COL6A1	CATGGTGTTGCAGTCACA	262	240	0	1	0	1
COL6A1	TGTGGTGTTGCAGTCAAC	27	28	0	0	0	0
COL6A2	TGTGGTGTTGGTACTGTG	230	371	1	0	0	0
COL6A2	CATGGTGTTGGTTGGTGT	494	570	1	1	0	1
COL6A2	ACTGGTGTTGGTTGGTCA	434	230	0	0	0	0
COL6A2	GTTGGTGTTGGTTGGTAC	62	41	0	0	0	1
COL6A2	TGTGGTGTTGGTTGGTTG	712	845	2	0	0	2
COL6A3	TGCATGACTGACCAACCA	378	485	0	0	0	0
COL6A3	CACATGACTGACCAACAC	20	9	0	0	0	0
COL6A3	GTCATGACTGACCAACTG	1217	1002	1	0	0	1

COL6A3	TGCATGACTGACCATGGT	371	353	0	1	0	0
COL6A3	GTTGACGTACTGACCAGT	381	472	0	1	0	0
COL7A1	GTCATGACTGCATGACTG	686	927	0	3	1	20
COL7A1	ACCATGACTGCATGACGT	159	439	0	0	0	1
COL7A1	TGCATGACTGCATGACCA	102	138	0	0	0	0
COL7A1	CACATGACTGCATGACAC	78	53	0	0	0	0
COL7A1	CATGCAGTACGTCAACGT	324	531	1	0	0	0
COL7A1	CATGCAACGTTGCAGTCA	145	124	1	0	0	0
COL8A1	CATGGTGTACTGTGGTCA	196	260	1	0	0	0
COL8A1	TGTGGTGTACTGTGGTAC	2	9	0	0	0	0
COL8A1	ACTGGTGTACTGTGGTTG	27	117	1	0	0	0
COL8A1	CATGGTGTACTGTGCAGT	31	26	0	0	0	0
COL8A1	ACTGGTGTACTGTGCACA	79	48	0	0	0	0
COL8A2	CAACTGTGTGCAGTTGGT	107	116	0	0	0	0
COL8A2	ACACTGTGTGCAGTTGCA	30	31	0	0	0	0
COL8A2	GTACTGTGTGCAGTTGAC	49	59	0	0	0	0
COL8A2	TGACTGTGTGCAGTTGTG	246	220	0	0	0	0
COL8A2	CAACTGTGTGCACAGTGT	497	731	2	0	0	0
COL9A1	GTACTGTGTGACACGTTG	147	236	0	1	1	0
COL9A1	CAACTGTGTGACACGTAC	47	61	0	0	0	0
COL9A1	TGACTGTGTGACACCAGT	46	46	0	0	0	1
COL9A1	GTACTGTGTGACACCACA	59	42	0	0	0	4
COL9A1	ACACTGTGTGACACCAAC	120	129	0	0	0	0
COL9A2	TGTGGTGTGTGTACCATG	173	104	0	0	0	0
COL9A2	ACTGGTGTGTGTACACGT	348	96	0	0	0	0
COL9A2	TGTGGTGTGTGTACACCA	300	233	69	0	0	14
COL9A2	GTTGCACAGTGTGTCACA	60	167	0	1	0	0
COL9A2	GTTGCATGGTTGCAGTAC	86	131	0	0	0	0
COL9A2	TGTGCATGGTTGCAGTTG	387	407	0	0	0	0
COL9A3	CATGGTGTACTGACGTGT	398	544	1	0	1	6
COL9A3	ACTGGTGTACTGACGTCA	221	92	0	1	0	0
COL9A3	GTTGGTGTACTGACGTAC	35	33	0	0	0	1
COL9A3	TGTGGTGTACTGACGTTG	308	586	1	0	0	0
COL9A3	CATGCACAGTGTCAACTG	1022	461	2	0	0	68
COMP	TGACACTGCACAACGTAC	64	90	0	0	0	0
COMP	ACACACTGCACAACGTTG	140	287	0	0	0	0
COMP	CAACACTGCACAACCAGT	376	566	0	0	0	0
COMP	ACTGCAGTTGTGCATGAC	63	84	0	0	0	0
COMP	CATGCAGTTGTGCATGTG	300	214	0	0	0	0
COMT	CAACCAACCACAACGTCA	302	207	9	4	0	6
COMT	TGACCAACCACAACGTAC	34	36	0	0	0	0
COMT	ACACCAACCACAACGTTG	203	357	0	0	0	0
COMT	CAACCAACCACAACCAGT	1082	634	0	0	0	0
COMT	ACACCAACCACAACCACA	12	25	0	0	0	0
COMT	ACTGCACAACACGTCAAC	19	8	0	0	0	0
COPA	CAACTGACTGCAGTACTG	677	263	1	1	1	0
COPA	GTACTGACTGCAGTCATG	115	135	0	0	0	0
COPA	TGACTGACTGCAGTACGT	246	219	0	0	0	0
COPA	GTACTGACTGCAGTACCA	173	199	1	1	0	0
COPA	ACACTGACTGCAGTACAC	45	40	0	0	0	0
COPS3	ACTGCAACTGGTCAGTTG	1230	512	7	3	2	1
COPS3	CATGGTACACTGCATGTG	375	648	1	0	1	0
COPS3	GTTGGTACACTGACGTCA	115	262	0	1	1	0
COPS3	GTTGGTACACTGCATGCA	368	538	2	0	0	0
COPS3	ACTGGTACACTGCATGAC	137	135	0	0	0	0
COPS3	TGTGGTACACTGACGTGT	463	667	2	0	0	1
COPS5	ACACGTACTGACGTACTG	172	183	0	0	0	0
COPS5	CAACGTACTGACGTTGGT	328	850	0	1	0	0
COPS5	ACACGTACTGACGTTGCA	68	84	0	0	0	0
COPS5	GTACGTACTGACGTTGAC	47	42	0	0	0	0
COPS5	TGACGTACTGACGTTGTG	388	346	0	0	0	0
COQ2	TGACTGACTGTGGTCATG	331	363	0	0	0	1
COQ2	ACACTGACTGTGGTACGT	769	139	1	1	0	0
COQ2	TGACTGACTGTGGTACCA	92	106	0	0	0	0
COQ2	CAACTGACTGTGGTACAC	107	267	0	0	0	0
COQ2	GTACTGACTGTGGTACTG	139	137	0	0	0	0
COQ3	GTACCACATGGTTGTGCA	92	157	0	0	0	0
COQ3	ACACCACATGGTTGTGAC	20	34	0	0	0	0
COQ3	CAACCACATGGTTGTGTG	283	334	0	0	0	0
COQ3	ACACCACATGCAGTGTGT	516	545	0	0	0	1
COQ3	TGACCACATGCAGTGTCA	157	110	2	0	0	1
COQ5	TGTGGTTGGTACACCATG	219	239	0	0	0	0
COQ5	ACTGGTTGGTACACACGT	180	183	2	0	0	0
COQ5	TGTGGTTGGTACACACCA	277	174	1	0	0	0
COQ5	CATGGTTGGTACACACAC	74	138	0	1	0	0

COQ5	GTTGGTTGGTACACACTG	50	9	0	0	0	0
COQ6	TGACTGACACGTACCACA	199	228	0	0	0	0
COQ6	CAACTGACACGTACCAAC	8	25	0	0	0	0
COQ6	GTACTGACACGTACCATG	101	120	0	1	0	0
COQ6	CATGCATGCAACCACAAC	24	33	0	0	0	0
COQ6	GTTGCATGCAACCACATG	193	234	0	1	0	0
COQ7	ACTGGTTGGTCAACACTG	370	527	0	0	0	0
COQ7	CATGGTTGGTCAACTGGT	519	692	0	0	0	0
COQ7	ACTGGTTGGTCAACTGCA	798	954	1	1	0	0
COQ7	GTTGGTTGGTCAACTGAC	117	187	0	0	0	0
COQ7	ACTGGTTGTGGTTGGTTG	561	371	2	0	0	0
COQ7	GTTGCAACTGACACCAAC	56	53	0	0	0	0
COX10	ACACCACACACAACCATG	244	233	0	0	0	0
COX10	CAACCACACACAACACGT	33	7	0	0	0	0
COX10	ACACCACACACAACACCA	323	262	0	1	0	0
COX10	GTACCACACACAACACAC	17	30	0	0	0	0
COX10	TGTGCAGTCACACATGGT	115	131	0	1	0	0
COX10	GTTGCAACGTTGCACAAC	0	1	0	0	0	0
COX15	TGACACGTACTGACTGTG	588	823	0	0	0	0
COX15	CAACACGTACTGTGGTGT	907	1351	2	2	0	2
COX15	ACACACGTACTGTGGTCA	700	695	1	0	0	0
COX15	GTACACGTACTGTGGTAC	20	20	0	0	0	0
COX15	GTTGCAGTCAGTTGACAC	11	2	0	0	0	0
COX5A	TGACACGTACTGGTGTAC	22	63	0	0	0	0
COX5A	ACACACGTACTGGTGTTG	184	306	0	0	0	0
COX5A	CAACACGTACTGGTCAGT	20	42	0	0	0	0
COX5A	ACACACGTACTGGTCACA	213	210	0	0	0	0
COX5A	GTACACGTACTGGTCAAC	20	9	1	0	0	0
COX5A	TGTGCAACGTACTGTGAC	3	14	0	0	0	0
COX5B	ACTGCAACGTACTGTGTG	444	545	0	1	1	0
COX5B	GTACACGTTGCAACACGT	160	142	1	0	0	0
COX5B	CAACACGTTGCAACACCA	317	338	0	0	0	0
COX5B	TGACACGTTGCAACACAC	6	9	0	0	0	0
COX5B	ACACACGTTGCAACACTG	52	30	0	0	0	0
COX5B	TGTGCAGTTGGTCAACAC	52	80	0	0	0	0
COX6C	CAACACGTTGCAACTGGT	109	65	0	0	0	0
COX6C	ACACACGTTGCAACTGCA	82	79	0	0	0	0
COX6C	GTACACGTTGCAACTGAC	20	18	0	0	0	0
COX6C	TGACACGTTGCAACTGTG	208	236	0	0	0	0
COX6C	CAACACGTTGCATGGTGT	1261	413	1	1	0	1
COX6C	TGTGCAACGTTGACCAAC	21	28	0	0	0	0
CP	CAACACGTACTGTGTGCA	151	170	0	0	1	0
CP	ACTGCACACACATGCATG	202	190	0	0	1	75
CP	TGACACGTACTGTGTGAC	53	54	1	0	0	0
CP	ACACACGTACTGTGTGTG	54	42	0	0	0	0
CP	CAACACGTTGGTGTGTGT	535	477	2	0	0	1
CP	ACACACGTTGGTGTGTCA	154	120	0	0	0	0
CPA3	TGACACCAGTCAGTACTG	252	326	0	1	0	0
CPA3	ACACACCAGTCAGTTGGT	644	393	3	0	0	0
CPA3	TGACACCAGTCAGTTGCA	63	106	0	0	0	1
CPA3	CAACACCAGTCAGTTGAC	73	75	0	0	0	0
CPA3	GTACACCAGTCAGTTGTG	965	457	1	1	0	0
CPA3	ACTGCACAGTACGTACCA	90	236	0	0	0	0
CPB2	TGACACCAGTGTACTGTG	393	359	0	0	0	0
CPB2	CAACACCAGTGTTGGTGT	423	462	1	0	0	0
CPB2	ACACACCAGTGTTGGTCA	325	329	0	1	0	5
CPB2	GTACACCAGTGTTGGTAC	24	32	0	0	0	4
CPB2	TGACACCAGTGTTGGTTG	1193	1781	2	2	0	0
CPB2	ACTGCAACGTACACTGCA	356	141	2	0	0	1
CPE	CAACACACGTCACACACA	273	161	0	0	1	12
CPE	ACACACACGTCACAGTAC	62	50	0	0	0	0
CPE	CAACACACGTCACAGTTG	1605	1545	4	0	0	0
CPE	GTACACACGTCACACAGT	88	74	0	0	0	0
CPE	TGACACACGTCACACAAC	127	38	0	1	0	0
CPE	GTTGCACAGTACGTACAC	104	53	0	0	0	0
CPEB1	TGTGGTTGGTCACATGCA	336	394	0	0	0	0
CPEB1	CATGGTTGGTCACATGAC	11	17	0	0	0	0
CPEB1	GTTGGTTGGTCACATGTG	806	912	1	1	0	0
CPEB1	ACTGGTTGGTCAACGTGT	380	415	0	0	0	0
CPEB1	TGTGGTTGGTCAACGTCA	568	412	1	1	0	0
CPN1	TGACCAGTTGTGACTGTG	530	693	6	2	6	10
CPN1	ACACCAGTTGTGACTGCA	863	318	0	0	0	0
CPN1	TGACACCAGTACGTCACA	90	100	0	0	0	0
CPN1	CAACACCAGTACGTCAAC	3	3	0	0	0	0
CPN1	GTACACCAGTACGTCATG	63	53	0	0	0	0

CPN2	CATGCAGTGTACGTTGGT	1042	205	1	0	1	0
CPN2	GTTGCAGTGTACGTTGAC	69	53	2	0	1	0
CPN2	ACTGCAGTGTACGTTGCA	36	36	0	0	0	0
CPN2	TGTGCAGTGTACGTTGTG	111	60	0	0	0	0
CPN2	CATGCAGTGTACCAGTGT	1274	505	2	0	0	0
CPO	TGACACCAGTCATGTGGT	803	313	0	0	1	157
CPO	GTACACCAGTCATGACTG	302	22	0	0	0	0
CPO	GTACACCAGTCATGTGCA	171	372	2	1	0	1
CPO	ACACACCAGTCATGTGAC	21	27	0	0	0	0
CPO	CAACACCAGTCATGTGTG	117	103	0	0	0	0
CPO	TGTGCAACTGTGACGTTG	216	221	1	0	0	0
CPS1	GTACACGTCACAACGTAC	167	110	0	0	0	0
CPS1	TGACACGTCACAACGTTG	526	617	0	0	0	0
CPS1	ACACACGTCACAACCAGT	402	104	1	0	0	0
CPS1	TGACACGTCACAACCACA	285	142	0	0	0	0
CPS1	CAACACGTCACAACCAAC	57	56	0	0	0	0
CPS1	TGTGCAACACGTCACACA	118	301	0	0	0	0
CPSF1	CACATGCACAGTTGACGT	175	268	0	0	1	4
CPSF1	TGCATGCACAGTTGCAAC	0	1	0	0	0	0
CPSF1	ACCATGCACAGTTGCATG	49	20	0	0	0	0
CPSF1	ACCATGCACAGTTGACCA	216	237	0	1	0	0
CPSF1	GTCATGCACAGTTGACAC	55	45	0	0	0	0
CPSF2	GTACTGTGCAGTGTCACA	85	90	0	0	0	0
CPSF2	ACACTGTGCAGTGTCAAC	93	33	0	0	0	0
CPSF2	CAACTGTGCAGTGTCATG	303	250	0	0	0	0
CPSF2	GTACTGTGCAGTGTACGT	160	101	0	0	0	0
CPSF2	CAACTGTGCAGTGTACCA	133	238	0	0	0	0
CPSF3	ACACTGTGCAGTGTGTGT	124	157	0	0	0	0
CPSF3	TGACTGTGCAGTGTGTCA	420	722	0	1	0	0
CPSF3	CAACTGTGCAGTGTGTAC	396	372	0	0	0	0
CPSF3	GTACTGTGCAGTGTGTTG	1288	1165	0	0	0	1
CPSF3	TGACTGTGCAGTGTCAGT	198	215	0	0	0	0
CPSF3	CATGCAACCATGTGCACA	156	42	0	0	0	0
CPSF4	ACACTGTGGTTGTGACGT	70	152	0	0	0	0
CPSF4	TGACTGTGGTTGTGACCA	358	268	0	2	0	0
CPSF4	CAACTGTGGTTGTGACAC	41	29	2	0	0	0
CPSF4	CATGGTCACAACGTTGCA	221	376	1	0	0	0
CPSF4	TGTGCAGTCATGTGACGT	55	130	0	0	0	0
CPSF6	CACATGCACAGTTGGTTG	626	612	4	0	1	0
CPSF6	GTCATGCACAGTTGGTCA	345	303	0	0	0	1
CPSF6	ACCATGCACAGTTGGTAC	25	35	0	0	0	0
CPSF6	GTCATGCACAGTTGCAGT	386	240	0	0	0	1
CPSF6	CACATGCACAGTTGCACA	70	73	0	0	0	0
CPSF6	GTTGCACAACCACATGTG	526	222	1	0	0	1
CPT1A	ACACCAACCACATGCATG	192	145	0	0	0	0
CPT1A	CAACCAACCACATGACGT	745	426	1	0	0	0
CPT1A	ACACCAACCACATGACCA	142	139	0	0	0	1
CPT1A	GTACCAACCACATGACAC	89	90	0	0	0	0
CPT1A	TGACCAACCACATGACTG	821	1202	0	0	0	0
CPT1B	TGACGTTGTGACACCACA	26	39	0	0	0	1
CPT1B	CAACCAACCAACGTTGTG	211	218	0	0	0	0
CPT1B	TGACCAACCAACCAGTGT	288	284	0	0	0	0
CPT1B	GTACCAACCAACCAGTCA	171	284	0	0	0	0
CPT1B	ACACCAACCAACCAGTAC	65	89	0	0	0	0
CPT1C	ACACCACATGCAGTTGCA	274	646	3	2	2297	6
CPT1C	CAACCACATGCAGTTGGT	268	424	0	1	0	0
CPT1C	GTACCACATGCAGTTGAC	67	58	0	0	0	0
CPT1C	TGACCACATGCAGTTGTG	167	238	2	0	0	0
CPT1C	CAACCACATGCACAGTGT	1147	678	2	1	0	1
CPT1C	GTTGCAACACTGCAACAC	34	40	0	0	0	0
CPT2	GTCATGACACCACAGTCA	567	535	0	0	0	0
CPT2	ACCATGACACCACAGTAC	70	218	0	0	0	0
CPT2	CACATGACACCACAGTTG	782	983	1	1	0	0
CPT2	GTCATGACACCACACAGT	41	47	0	0	0	0
CPT2	CACATGACACCACACACA	44	35	0	0	0	0
CR1	GTACCAGTTGCACATGCA	170	99	0	0	0	0
CR1	ACACCAGTTGCACATGAC	39	86	550	1	0	0
CR1	CAACCAGTTGCACATGTG	779	619	2	1	0	7
CR1	TGACCAGTTGCAACGTGT	86	107	0	0	0	0
CR1	GTACCAGTTGCAACGTCA	314	299	1	1	0	2
CR2	CAACACTGACTGTGGTCA	56	42	0	0	0	0
CR2	TGACACTGACTGTGGTAC	84	219	0	0	0	0
CR2	ACACACTGACTGTGGTTG	1038	644	3	0	0	0
CR2	CAACACTGACTGTGCAGT	164	262	0	0	0	0
CR2	ACACACTGACTGTGCACA	97	81	1	0	0	0

CRADD	TGTGGTGTCAACTGACGT	301	365	0	0	0	0
CRADD	GTTGGTGTCAACTGACCA	246	277	0	0	0	0
CRADD	ACTGGTGTCAACTGACAC	77	8	0	0	0	0
CRADD	CATGGTGTCAACTGACTG	183	135	2	0	0	0
CRADD	GTTGCAGTCACACATGCA	538	434	0	1	0	0
CRAT	CAACCACATGACTGTGCA	190	170	0	0	0	0
CRAT	TGACCACATGACTGTGAC	48	104	1	0	0	0
CRAT	ACACCACATGACTGTGTG	232	191	0	0	0	1
CRAT	CAACCACATGTGGTGTGT	456	134	0	0	0	1
CRAT	TGTGCAGTACTGCAGTTG	227	459	3	1	0	0
CRAT	CATGCAACACCAACACGT	32	97	0	0	0	0
CREB1	TGACGTGTACGTGTCATG	93	138	0	0	0	0
CREB1	ACACGTGTACGTGTACGT	155	148	0	0	0	0
CREB1	TGACGTGTACGTGTACCA	92	84	1	0	0	0
CREB1	GTTGGTGTCAGTACACCA	85	82	0	0	0	7
CREB1	ACTGGTGTCAGTACACAC	111	195	0	0	0	2
CREB1	TGTGCAACACACTGACCA	265	235	0	1	0	1
CREB3	ACACGTTGCAGTACACCA	38	39	0	0	0	0
CREB3	GTACGTTGCAGTACACAC	28	54	0	0	0	0
CREB3	TGACGTTGCAGTACACTG	195	307	0	1	0	0
CREB3	ACACGTTGCAGTACTGGT	138	145	0	0	0	0
CREB3	TGACGTTGCAGTACTGCA	359	266	0	0	0	0
CREB3L1	ACACGTACCACATGTGAC	27	32	0	0	1	0
CREB3L1	CAACGTACCACATGACAC	83	99	0	0	0	0
CREB3L1	GTACGTACCACATGACTG	153	137	0	0	0	1
CREB3L1	TGACGTACCACATGTGGT	606	403	0	0	0	0
CREB3L1	GTACGTACCACATGTGCA	172	124	0	0	0	0
CREB3L2	ACACGTACCATGACACGT	53	56	0	0	0	0
CREB3L2	TGACGTACCATGACACCA	39	49	0	0	0	0
CREB3L2	CAACGTACCATGACACAC	305	51	0	1	0	1
CREB3L2	GTACGTACCATGACACTG	223	296	0	0	0	0
CREB3L2	CATGGTGTGTTGCACACA	7	5	0	0	0	0
CREB3L3	ACACGTACACGTGTTGCA	176	105	0	0	0	0
CREB3L3	GTACGTACACGTGTTGAC	28	31	0	0	0	0
CREB3L3	TGACGTACACGTGTTGTG	114	147	0	0	0	0
CREB3L3	CAACGTACACGTCAGTGT	478	441	0	0	0	0
CREB3L3	ACACGTACACGTCAGTCA	382	322	2	1	0	0
CREB3L3	CATGCAACGTGTTGGTCA	264	199	0	0	0	1
CREB5	ACTGCAGTGTTGGTGTAC	80	127	0	0	1	0
CREB5	CATGCAGTGTTGGTGTTG	1600	772	3	0	1	0
CREB5	GTACGTCAACTGACCATG	492	442	0	0	0	0
CREB5	TGACGTCAACTGACACGT	8	4	0	0	0	0
CREB5	GTACGTCAACTGACACCA	366	261	1	0	0	0
CREBBP	TGACGTGTGTACCATGAC	119	83	6113	9	0	1
CREBBP	ACACGTGTGTACCATGTG	75	73	0	0	0	0
CREBBP	GTACGTGTGTACACGTGT	225	244	1	0	0	0
CREBBP	CAACGTGTGTACACGTCA	94	207	0	0	0	0
CREBBP	ACTGGTTGACTGTGTGGT	387	968	3	0	0	0
CREM	GTACGTCATGGTTGTGGT	333	535	3	1	0	0
CREM	CAACGTCATGGTTGTGCA	347	120	9	1	0	0
CREM	TGACGTCATGGTTGTGAC	28	43	0	0	0	0
CREM	GTTGCAGTCAGTGTTGAC	14	15	0	0	0	0
CREM	TGTGCAGTCAGTGTTGTG	106	164	0	0	0	0
CRH	CAACGTGTTGTGTGCATG	368	603	3	0	3	3
CRH	ACACGTGTTGTGTGCAAC	29	38	0	0	0	0
CRH	GTACGTGTTGTGTGACGT	185	179	0	0	0	0
CRH	ACTGCACAGTACGTTGGT	157	420	0	0	0	0
CRH	TGTGCACAGTACGTTGCA	210	288	0	0	0	0
CRH	CATGCACAGTACGTTGAC	75	106	0	0	0	13
CRHBP	TGTGGTACGTTGGTCAGT	120	95	0	1	0	0
CRHBP	GTTGGTACGTTGGTCACA	21	11	0	0	0	0
CRHBP	ACTGGTACGTTGGTCAAC	110	40	0	0	0	0
CRHBP	CATGGTACGTTGGTCATG	149	217	0	0	0	0
CRHBP	GTTGGTACGTTGGTACGT	65	49	0	0	0	4
CRHR1	CAACGTGTTGTGTGACCA	85	196	0	0	0	0
CRHR1	TGACGTGTTGTGTGACAC	6	9	0	0	0	0
CRHR1	ACACGTGTTGTGTGACTG	194	152	0	0	0	0
CRHR1	CAACGTGTTGTGTGTGGT	188	244	0	1	0	0
CRHR1	ACACGTGTTGTGTGTGCA	231	215	0	0	0	1
CRHR2	GTACGTGTTGTGTGTGAC	29	29	0	0	0	0
CRHR2	TGACGTGTTGTGTGTGTG	218	697	0	0	0	0
CRHR2	ACACGTCAGTGTGTGTGT	149	215	0	0	0	0
CRHR2	TGACGTCAGTGTGTGTCA	434	764	0	0	0	0
CRHR2	CAACGTCAGTGTGTGTAC	66	372	0	0	0	13
CRHR2	TGTGCATGCACACAACTG	152	175	0	0	0	0

CRK	CAACGTTGACTGGTTGCA	93	118	0	1	0	0
CRK	TGACGTTGACTGGTTGAC	16	9	0	0	0	0
CRK	ACACGTTGACTGGTTGTG	255	302	1	0	0	0
CRK	GTACGTTGACTGCAGTGT	1211	589	2	1	0	1
CRK	ACACCATGACCAACGTAC	64	85	1	0	0	0
CRKL	ACACGTGTGTCAACACTG	74	45	1	0	0	0
CRKL	CAACGTGTGTCAACTGGT	431	448	0	0	0	0
CRKL	ACACGTGTGTCAACTGCA	58	85	0	0	0	0
CRKL	GTACGTGTGTCAACTGAC	74	77	1	0	0	0
CRKL	TGACGTGTGTCAACTGTG	219	204	0	0	0	0
CRLF2	GTTGACACGTTGACACGT	65	63	0	0	0	0
CRLF2	CATGACACGTTGACACCA	248	220	0	0	0	0
CRLF2	TGTGACACGTTGACACAC	150	107	0	2	0	0
CRLF2	ACTGACACGTTGACACTG	484	612	1	0	0	0
CRLF2	CATGACACGTTGACTGGT	85	33	1	0	0	0
CRLS1	ACTGGTCAACGTTGTGGT	234	324	0	0	0	0
CRLS1	TGTGGTCAACGTTGTGCA	433	422	2	1	0	1
CRLS1	CATGGTCAACGTTGTGAC	100	100	0	0	0	0
CRLS1	GTTGGTCAACGTTGTGTG	76	133	0	2	0	4
CRLS1	CATGGTCAACCAGTGTGT	648	790	2	1	0	0
CRP	GTACACTGACCATGTGGT	137	152	2	0	1	0
CRP	GTACACTGACCATGACCA	135	274	0	1	0	0
CRP	ACACACTGACCATGACAC	144	36	0	0	0	0
CRP	CAACACTGACCATGACTG	251	194	1	0	0	0
CRP	CAACACTGACCATGTGCA	249	153	0	0	0	1
CRTAM	ACTGGTCAACTGCACATG	574	598	1	2	3	0
CRTAM	TGTGGTCAACTGCACAAC	10	8	0	0	0	0
CRTAM	CATGGTCAACTGCAACGT	538	139	1	0	0	0
CRTAM	ACTGGTCAACTGCAACCA	283	394	0	0	0	9
CRTAM	GTTGGTCAACTGCAACAC	68	99	0	0	0	0
CRTC1	ACTGGTCAGTACACTGCA	85	139	1	0	0	0
CRTC1	GTTGGTCAGTACACTGAC	33	25	0	0	0	0
CRTC1	TGTGGTCAGTACACTGTG	294	274	0	0	0	0
CRTC1	CATGGTCAGTACTGGTGT	81	106	0	0	0	0
CRTC1	ACTGGTCAGTACTGGTCA	348	446	0	0	0	0
CRY1	GTACGTCAGTGTGTGTTG	209	335	0	1	0	0
CRY1	TGACGTCAGTGTGTCAGT	243	187	0	33	0	1
CRY1	GTACGTCAGTGTGTCACA	87	87	0	0	0	0
CRY1	ACACGTCAGTGTGTCAAC	10	7	0	0	0	0
CRY1	CAACGTCAGTGTGTCATG	357	305	0	0	0	36
CRY1	GTTGCAACCACACACACA	44	31	0	0	0	0
CRY2	GTACTGTGCATGACACGT	159	75	0	0	0	0
CRY2	CAACTGTGCATGACACCA	55	87	0	0	0	0
CRY2	TGACTGTGCATGACACAC	130	144	0	0	0	0
CRY2	ACACTGTGCATGACACTG	543	527	0	0	0	0
CRY2	CAACTGTGCATGACTGGT	186	310	1	0	0	0
CRYAB	GTTGGTGTCAGTTGGTGT	1909	1765	4	3	1	105
CRYAB	GTTGCACAGTACGTTGTG	289	367	0	0	1	0
CRYAB	ACCATGTGGTGTTGGTTG	185	183	0	0	0	0
CRYAB	CACATGTGGTGTTGCAGT	192	244	0	0	0	0
CRYAB	ACCATGTGGTGTTGCACA	106	105	0	0	0	0
CRYAB	CATGCAGTACGTTGACTG	421	383	0	2	0	0
CS	CAACACGTACGTGTACCA	47	45	0	0	0	0
CS	TGACACGTACGTGTACAC	10	5	0	0	0	0
CS	ACACACGTACGTGTACTG	151	165	0	0	0	0
CS	CAACACGTACGTGTTGGT	17	37	0	0	0	0
CS	ACACACGTACGTGTTGCA	29	22	1	0	0	0
CSAD	CAACTGTGACCAACACTG	207	359	0	0	0	0
CSAD	GTACTGTGACCAACTGGT	187	924	2	0	0	0
CSAD	CAACTGTGACCAACTGCA	281	153	0	0	0	0
CSAD	TGACTGTGACCAACTGAC	47	241	0	0	0	0
CSAD	ACACTGTGACCAACTGTG	189	307	0	0	0	1
CSDA	TGACGTCAACACACACCA	259	402	0	0	0	0
CSDA	CAACGTCAACACACACAC	166	140	0	1	0	0
CSDA	GTACGTCAACACACACTG	525	585	0	1	0	0
CSDA	TGACGTCAACACACTGGT	191	208	0	0	0	1
CSDA	GTACGTCAACACACTGCA	271	162	1	0	0	1
CSDA	ACTGCAACACGTGTACTG	93	68	0	0	0	0
CSE1L	TGACTGCATGCACAGTGT	464	279	0	0	1	0
CSE1L	GTACTGCATGCAGTTGCA	30	59	0	0	0	0
CSE1L	ACACTGCATGCAGTTGAC	2	3	0	0	0	0
CSE1L	CAACTGCATGCAGTTGTG	37	14	0	0	0	0
CSE1L	TGTGCATGGTCATGACCA	154	111	0	0	0	0
CSE1L	CATGCATGGTCATGACAC	131	10	0	1	0	0
CSF1	ACACTGGTACTGACCAGT	558	431	1	0	0	0

CSF1	TGACTGGTACTGACCACA	144	74	0	0	0	0
CSF1	CAACTGGTACTGACCAAC	43	37	1	0	0	0
CSF1	GTACTGGTACTGACCATG	31	49	0	0	0	0
CSF1	TGACTGGTACTGACACGT	161	238	1	0	0	6
CSF1	ACTGCAACTGTGACGTCA	237	275	1	0	0	0
CSF1R	CACATGACGTGTACTGTG	1054	436	1	0	1	0
CSF1R	ACCATGACGTGTACTGAC	57	33	0	0	0	0
CSF1R	TGCATGACGTGTTGGTGT	352	586	1	0	0	12
CSF1R	ACTGCAGTACGTACGTGT	165	138	0	0	0	0
CSF1R	TGTGCAGTACGTACGTCA	169	198	1	0	0	0
CSF1R	CATGCAACTGTGACGTGT	448	398	1	0	0	0
CSF2	GTACTGGTACACGTGTTG	515	342	0	0	1	0
CSF2	CAACTGGTACACGTGTAC	34	34	0	1	0	0
CSF2	TGACTGGTACACGTCAGT	442	396	1	0	0	0
CSF2	GTACTGGTACACGTCACA	30	56	0	0	0	0
CSF2	ACACTGGTACACGTCAAC	1	3	0	0	0	0
CSF2	ACTGCAACTGTGTGCACA	67	96	0	0	0	0
CSF2RA	CAACTGGTACGTGTGTGT	216	133	0	0	0	0
CSF2RA	ACACTGGTACGTGTGTCA	129	167	0	0	0	1
CSF2RA	GTACTGGTACGTGTGTAC	11	12	0	0	0	0
CSF2RA	TGACTGGTACGTGTGTTG	149	180	1	0	0	0
CSF2RA	TGTGCAGTTGTGACTGCA	36	43	0	0	0	1
CSF2RB	CAACTGCAGTACCAACGT	182	476	0	0	0	5
CSF2RB	ACACTGCAGTACCAACCA	96	163	1	0	0	5
CSF2RB	GTACTGCAGTACCAACAC	40	43	0	0	0	0
CSF2RB	TGACTGCAGTACCAACTG	40	43	0	0	0	0
CSF2RB	ACTGCAGTTGACACCAAC	11	20	0	0	0	0
CSF2RB	TGTGCACACAACTGGTTG	296	328	0	0	0	38
CSF3	TGACTGCAGTACACTGTG	291	337	1	0	1	0
CSF3	ACACTGCAGTACACACTG	238	272	0	0	0	0
CSF3	CAACTGCAGTACACTGGT	286	219	1	0	0	0
CSF3	ACACTGCAGTACACTGCA	74	18	0	0	0	0
CSF3	GTACTGCAGTACACTGAC	15	6	0	0	0	0
CSF3	ACTGCAACTGTGTGACGT	55	78	0	0	0	1
CSF3R	CAACTGGTACGTACGTTG	529	609	0	1	1	0
CSF3R	TGTGCAACTGTGTGCATG	282	304	1	0	1	0
CSF3R	CAACTGGTACGTCATGTG	600	786	0	0	0	1
CSF3R	TGACTGGTACGTACGTGT	615	88	0	0	0	0
CSF3R	GTACTGGTACGTACGTCA	240	238	1	0	0	0
CSF3R	ACACTGGTACGTACGTAC	58	70	0	0	0	0
CSGLCA-T	CAACCACACAACGTGTTG	955	937	0	3	0	1
CSGLCA-T	GTACCACACAACGTCAGT	154	175	0	0	0	0
CSGLCA-T	CAACCACACAACGTCACA	44	39	0	0	0	0
CSGLCA-T	TGACCACACAACGTCAAC	7	20	0	0	0	0
CSGLCA-T	ACACCACACAACGTCATG	425	346	0	0	0	1
CSK	CATGCACACATGGTGTGT	2521	2174	25	8	14	28
CSK	TGCATGCAACTGTGCAAC	10	14	0	0	0	0
CSK	ACCATGCAACTGTGCATG	194	197	18	0	0	0
CSK	CACATGCAACTGTGACGT	53	54	0	0	0	0
CSK	ACCATGCAACTGTGACCA	875	841	2	125	0	0
CSK	GTCATGCAACTGTGACAC	123	137	0	0	0	0
CSMD1	ACCATGTGTGCATGTGTG	183	129	2	0	1	0
CSMD1	GTCATGTGTGCATGTGGT	589	539	1	0	0	16
CSMD1	CACATGTGTGCATGTGCA	243	225	0	0	0	0
CSMD1	TGCATGTGTGCATGTGAC	34	23	0	0	0	0
CSMD1	ACCATGTGTGACGTGTGT	241	289	3	0	0	0
CSNK1A1	GTCATGTGTGACCATGGT	288	430	0	0	0	0
CSNK1A1	CACATGTGTGACCATGCA	203	211	1	0	0	0
CSNK1A1	TGCATGTGTGACCATGAC	71	119	1	0	0	0
CSNK1A1	ACCATGTGTGACCATGTG	377	750	0	0	0	0
CSNK1A1	ACCATGTGTGACTGCATG	177	144	0	0	0	0
CSNK1A1L	CAACGTGTCAACGTCACA	256	125	0	0	0	0
CSNK1A1L	TGACGTGTCAACGTCAAC	0	2	0	0	0	0
CSNK1A1L	ACACGTGTCAACGTCATG	319	153	0	1	0	0
CSNK1A1L	CAACGTGTCAACGTACGT	120	108	0	0	0	0
CSNK1A1L	ACACGTGTCAACGTACCA	298	157	0	1	0	0
CSNK1D	GTCATGCAACCAACTGGT	496	897	43358	44	3	1
CSNK1D	TGCATGCAACCAACACGT	22	30	1	0	0	0
CSNK1D	GTCATGCAACCAACACCA	494	483	0	0	0	0
CSNK1D	ACCATGCAACCAACACAC	50	115	0	0	0	0
CSNK1D	CACATGCAACCAACACTG	510	552	0	0	0	0
CSNK1D	CATGCAACCATGGTCATG	174	173	0	1	0	2
CSNK1E	CACATGCAACCAACTGCA	302	177	1	0	0	0
CSNK1E	TGCATGCAACCAACTGAC	7	15	0	0	0	0
CSNK1E	ACCATGCAACCAACTGTG	256	296	0	0	0	0

CSNK1E	GTCATGCAACCATGGTGT	252	265	1	0	0	0
CSNK1E	CACATGCAACCATGGTCA	595	508	1	2	0	0
CSNK1G1	GTTGGTACTGTGGTGTGT	1540	1329	8	1	1	9
CSNK1G1	GTTGGTACTGACTGTGCA	225	177	2	1	0	1
CSNK1G1	ACTGGTACTGACTGTGAC	43	38	0	0	0	0
CSNK1G1	CATGGTACTGACTGTGTG	116	206	0	0	0	0
CSNK1G1	CATGGTACTGTGGTGTCA	167	183	0	0	0	0
CSNK1G2	ACACCATGGTCACACAGT	234	427	355135	404	15	53
CSNK1G2	CAACCATGGTCACAGTGT	1295	828	1360	1	0	24
CSNK1G2	ACACCATGGTCACAGTCA	900	1025	8	1	0	0
CSNK1G2	GTACCATGGTCACAGTAC	117	50	0	0	0	0
CSNK1G2	TGACCATGGTCACAGTTG	686	600	1	0	0	0
CSNK1G2	ACTGCAACCATGTGTGGT	667	675	3	1	0	2
CSNK1G3	TGCATGCAACTGTGACTG	209	145	0	0	0	0
CSNK1G3	ACCATGCAACTGTGTGGT	90	54	0	0	0	0
CSNK1G3	TGCATGCAACTGTGTGCA	414	222	0	2	0	0
CSNK1G3	ACACGTTGTGACCAGTGT	120	148	0	0	0	0
CSNK1G3	ACTGGTTGGTGTCACACA	50	32	0	0	0	0
CSNK2A1	CACATGCAACCATGCAGT	620	631	0	0	1	1
CSNK2A1	TGCATGCAACCATGGTAC	42	58	0	0	0	0
CSNK2A1	ACCATGCAACCATGGTTG	228	425	0	0	0	0
CSNK2A1	ACCATGCAACCATGCACA	63	59	0	0	0	1
CSNK2A1	GTCATGCAACCATGCAAC	23	48	0	0	0	0
CSNK2A2	TGCATGCAACCATGCATG	30	41	0	0	0	0
CSNK2A2	ACCATGCAACCATGACGT	93	98	0	0	0	0
CSNK2A2	TGCATGCAACCATGACCA	575	429	1	0	0	0
CSNK2A2	CACATGCAACCATGACAC	17	27	0	0	0	0
CSNK2A2	GTCATGCAACCATGACTG	456	276	0	0	0	0
CSNK2B	ACCATGTGGTGTCAGTGT	814	743	0	7	0	6
CSNK2B	TGCATGTGGTGTCAGTCA	672	908	0	3	0	1
CSNK2B	CACATGTGGTGTCAGTAC	123	95	1	0	0	0
CSNK2B	TGACGTTGTGACACACGT	53	30	0	0	0	0
CSNK2B	ACTGGTACTGCATGACTG	1260	595	3	0	0	0
CSNK2B	CATGCAACTGTGCAGTAC	24	36	0	0	0	0
CSRP1	ACTGCACACATGGTGTCA	404	433	2	0	1	0
CSRP1	GTTGGTCAGTGTCACACA	119	149	0	0	0	0
CSRP1	ACTGGTCAGTGTCACAAC	19	15	0	0	0	0
CSRP1	CATGGTCAGTGTCACATG	228	264	0	2	0	0
CSRP1	GTTGGTCAGTGTCAACGT	66	84	0	0	0	0
CSRP1	CATGCAGTCAACTGGTCA	122	428	0	0	0	0
CSRP3	ACTGGTCAGTGTGTCATG	55	40	0	0	0	0
CSRP3	CATGGTCAGTGTGTACGT	306	356	0	0	0	0
CSRP3	ACTGGTCAGTGTGTACCA	192	37	0	0	0	2
CSRP3	GTTGGTCAGTGTGTACAC	41	12	0	0	0	0
CSRP3	GTTGGTACTGCAGTTGGT	116	67	0	0	0	0
CST3	ACACGTGTCAGTCATGTG	579	710	0	1	1	9
CST3	CAACGTGTCAGTCATGCA	433	746	0	1	0	0
CST3	TGACGTGTCAGTCATGAC	33	62	0	0	0	0
CST3	GTACGTGTCAGTACGTGT	107	145	0	0	0	0
CST3	CAACGTGTCAGTACGTCA	36	81	0	0	0	0
CST3	GTTGCAACCACAGTGTCA	138	111	0	0	0	1
CSTF1	ACTGGTTGCACATGACTG	301	778	1	0	0	0
CSTF1	CATGGTTGCACATGTGGT	474	791	1	4	0	20
CSTF1	ACTGGTTGCACATGTGCA	757	412	1	2	0	0
CSTF1	GTTGGTTGCACATGTGAC	28	53	0	0	0	0
CSTF1	TGTGGTTGCACATGTGTG	228	299	0	0	0	0
CSTF2	ACTGGTGTCACACAGTCA	399	318	0	0	0	0
CSTF2	GTTGGTACTGGTCAACCA	397	437	0	1	0	0
CSTF2	ACTGGTACTGGTCAACAC	47	47	0	0	0	0
CSTF2	CATGGTACTGGTCAACTG	521	672	0	0	0	6
CSTF2	GTTGGTACTGGTCATGGT	283	413	0	0	0	1
CSTF2T	TGTGGTCATGGTACGTGT	83	79	0	0	0	0
CSTF2T	GTTGGTCATGGTACGTCA	155	173	0	0	0	3
CSTF2T	ACTGGTCATGGTACGTAC	52	118	0	0	0	0
CSTF2T	CATGGTCATGGTACGTTG	232	271	0	1	0	0
CSTF2T	GTTGGTCATGGTACCAGT	119	81	0	0	0	0
CSTF3	TGTGACCACATGACACAC	45	35	0	0	0	0
CSTF3	ACTGACCACATGACACTG	405	227	0	2	0	0
CSTF3	CATGACCACATGACTGGT	378	305	0	0	0	0
CSTF3	ACTGACCACATGACTGCA	54	77	0	0	0	0
CSTF3	GTTGACCACATGACTGAC	135	94	1	0	0	0
CTBP1	ACACGTCATGGTTGTGTG	793	927	1	1	1	0
CTBP1	TGACGTCATGCAGTGTGT	251	375	0	0	0	0
CTBP1	GTACGTCATGCAGTGTCA	480	543	0	0	0	0
CTBP1	ACACGTCATGCAGTGTAC	55	78	0	0	0	0

CTBP1	CAACGTCATGCAGTGTTG	290	324	0	0	0	1
CTBP2	GTACGTCATGCAGTCAGT	71	181	0	0	0	0
CTBP2	CAACGTCATGCAGTCACA	75	92	0	0	0	1
CTBP2	TGACGTCATGCAGTCAAC	121	35	0	0	0	0
CTBP2	GTTGGTGTACGTACTGTG	180	184	0	0	0	0
CTBP2	ACTGGTGTACGTTGGTGT	246	327	1	0	0	0
CTBP2	CATGCACATGACTGACCA	229	208	1	0	0	5
CTBS	ACTGGTCATGGTGTTGTG	559	945	1	726	2	0
CTBS	TGTGGTCATGGTGTTGAC	117	142	0	0	0	0
CTBS	GTTGGTCATGGTCAGTGT	488	612	4	0	0	1
CTBS	CATGGTCATGGTCAGTCA	522	441	0	2	0	1
CTBS	TGTGGTCATGGTCAGTAC	102	109	0	0	0	0
CTBS	TGTGCACACATGGTGTTG	362	171	1	1	0	0
CTCF	CAACGTACGTCACAACTG	351	225	0	0	0	1
CTCF	GTACGTACGTCACATGGT	628	264	0	1	0	0
CTCF	CAACGTACGTCACATGCA	509	328	0	0	0	0
CTCF	TGACGTACGTCACATGAC	7	4	0	0	0	0
CTCF	ACACGTACGTCACATGTG	338	356	0	1	0	13
CTF1	TGACTGGTACTGACTGAC	112	121	0	0	0	1
CTF1	ACACTGGTACTGACTGTG	52	100	0	0	0	0
CTF1	GTACTGGTACTGTGGTGT	545	992	1	1	0	0
CTF1	CAACTGGTACTGTGGTCA	176	278	0	0	0	0
CTF1	TGTGCAGTTGACGTGTAC	28	27	0	0	0	0
CTGF	ACACTGCATGCATGTGTG	123	324	0	0	0	0
CTGF	ACACTGCATGACGTGTGT	192	221	0	0	0	0
CTGF	TGACTGCATGACGTGTCA	189	140	0	0	0	0
CTGF	CAACTGCATGACGTGTAC	133	124	0	0	0	0
CTGF	GTTGCAGTACGTCAACAC	32	117	0	0	0	0
CTH	ACACTGTGCATGGTTGAC	186	14	0	1	0	0
CTH	CAACTGTGCATGGTTGTG	701	931	2	0	0	1
CTH	TGACTGTGCATGCAGTGT	470	735	2	0	0	0
CTH	GTACTGTGCATGCAGTCA	187	238	1	0	0	0
CTH	TGTGCAGTTGTGTGCAGT	32	81	0	0	0	0
CTLA4	TGACACTGACGTCAACCA	103	88	0	0	0	0
CTLA4	CAACACTGACGTCAACAC	41	73	0	0	0	0
CTLA4	GTACACTGACGTCAACTG	827	863	0	0	0	0
CTLA4	TGACACTGACGTCATGGT	872	1319	2	1	0	2
CTLA4	GTTGCAGTTGACCACAGT	181	150	1	0	0	0
CTNNA1	ACACTGACGTCAGTACCA	109	107	1	0	0	0
CTNNA1	GTACTGACGTCAGTACAC	2	0	0	0	0	0
CTNNA1	TGACTGACGTCAGTACTG	37	94	0	0	0	0
CTNNA1	ACACTGACGTCAGTTGGT	253	485	83	1	0	0
CTNNA1	TGTGGTTGTGCACACACA	24	30	0	0	0	0
CTNNA2	CATGGTACGTGTGTCAGT	64	72	0	0	1	0
CTNNA2	ACTGGTACGTGTGTCACA	405	256	0	1	0	0
CTNNA2	GTTGGTACGTGTGTCAAC	39	23	0	0	0	0
CTNNA2	TGTGGTACGTGTGTCATG	296	337	0	0	0	0
CTNNA2	ACTGACGTACTGTGCAAC	11	15	0	0	0	0
CTNNA3	GTTGGTACACCACACATG	201	256	1	1	0	0
CTNNA3	TGTGGTACACCACAACGT	226	214	2	0	0	0
CTNNA3	GTTGGTACACCACAACCA	312	660	0	23	0	0
CTNNA3	ACTGGTACACCACAACAC	191	129	0	0	0	0
CTNNA3	CATGGTACACCACAACTG	175	177	0	0	0	3
CTNNA3	TGTGCACAACACACTGGT	149	204	0	0	0	0
CTNNAL1	TGTGGTGTTGACGTACGT	239	533	0	1	1	0
CTNNAL1	GTTGGTGTTGACGTCATG	52	129	0	0	0	0
CTNNAL1	GTTGGTGTTGACGTACCA	111	145	0	0	0	0
CTNNAL1	ACTGGTGTTGACGTACAC	141	21	0	0	0	0
CTNNAL1	CATGGTGTTGACGTACTG	173	107	0	0	0	0
CTNNAL1	ACTGCACAGTACCAGTGT	159	236	1	0	0	0
CTNNB1	GTCATGTGGTGTTGCAAC	21	26	0	0	0	0
CTNNB1	ACTGACCAACTGGTCAAC	45	16	0	0	0	0
CTNNB1	CATGACCAACTGGTCATG	91	113	0	0	0	0
CTNNB1	GTTGACCAACTGGTACGT	74	51	0	0	0	0
CTNNB1	CATGACCAACTGGTACCA	157	167	0	0	0	0
CTNNBIP1	ACACGTTGACTGGTCAGT	62	38	0	0	0	0
CTNNBIP1	TGACGTTGACTGGTCACA	27	77	0	0	0	0
CTNNBIP1	CAACGTTGACTGGTCAAC	47	17	0	0	0	0
CTNNBIP1	GTACGTTGACTGGTCATG	228	177	0	0	0	9
CTNNBIP1	ACTGCAGTACGTCAACCA	492	493	0	0	0	14
CTNND1	GTTGACCAACTGTGTGTG	847	948	0	1	1	2
CTNND1	GTTGGTCACAGTACCACA	86	80	0	0	0	0
CTNND1	ACTGGTCACAGTACCAAC	27	20	1	0	0	0
CTNND1	CATGGTCACAGTACCATG	88	111	1	0	0	0
CTNND1	CATGACCAACTGTGTGAC	159	150	0	0	0	2

CTPS	ACACACGTCAGTTGACAC	66	281	0	0	0	0
CTPS	CAACACGTCAGTTGACTG	222	289	4	0	0	0
CTPS	GTACACGTCAGTTGTGGT	223	223	0	0	0	0
CTPS	CAACACGTCAGTTGTGCA	44	70	0	0	0	0
CTPS	TGTGCACAGTACCAGTCA	258	607	1	0	0	0
CTPS	CATGCACAGTACCAGTAC	242	45	0	0	0	0
CTPS2	CAACACGTCACAGTCACA	110	134	0	0	0	0
CTPS2	TGACACGTCACAGTCAAC	8	13	0	0	0	0
CTPS2	ACACACGTCACAGTCATG	109	115	0	0	0	0
CTPS2	GTTGCATGGTCACATGAC	4	22	0	0	0	0
CTPS2	TGTGCATGGTCACATGTG	274	410	1	0	0	0
CTPS2	CATGCATGGTCAACGTGT	128	185	0	1	0	0
CTSA	GTACCAGTTGTGCATGTG	293	483	0	1	0	1
CTSA	ACACACACCAACGTTGGT	25	161	0	0	0	0
CTSA	TGACACACCAACGTTGCA	126	196	0	0	0	0
CTSA	CAACACACCAACGTTGAC	57	34	0	0	0	0
CTSA	GTACACACCAACGTTGTG	260	355	0	0	0	2
CTSB	TGCATGACTGACACCAAC	62	54	0	0	0	0
CTSB	ACCATGACTGACACCATG	164	240	0	0	0	0
CTSB	CACATGACTGACACACGT	17	97	0	0	0	0
CTSB	ACCATGACTGACACACCA	260	151	1	1	0	0
CTSB	GTCATGACTGACACACAC	41	62	0	0	0	0
CTSD	CATGCAACACTGTGACTG	302	345	0	0	2	1
CTSD	TGCATGACTGACACACTG	941	852	0	2	0	0
CTSD	ACCATGACTGACACTGGT	355	342	0	0	0	0
CTSD	TGCATGACTGACACTGCA	67	129	0	0	0	0
CTSD	CACATGACTGACACTGAC	73	128	0	0	0	0
CTSD	GTCATGACTGACACTGTG	130	134	0	0	0	0
CTSE	ACCATGACTGACTGGTGT	349	434	2	0	0	0
CTSE	TGCATGACTGACTGGTCA	309	536	0	0	0	0
CTSE	CACATGACTGACTGGTAC	169	192	0	2	0	0
CTSE	GTCATGACTGACTGGTTG	720	86	1	0	0	0
CTSE	GTTGGTTGTGGTGTCAGT	369	60	0	0	0	0
CTSE	GTTGCAACACTGTGTGGT	132	216	0	1	0	0
CTSG	CACATGACTGACTGCATG	760	802	511	1	6	0
CTSG	TGCATGACTGACTGCAGT	73	80	0	0	0	0
CTSG	GTCATGACTGACTGCACA	196	173	1	0	0	1
CTSG	ACCATGACTGACTGCAAC	15	8	0	0	0	0
CTSG	GTCATGACTGACTGACGT	232	268	0	0	0	0
CTSG	CATGCAACACTGTGTGCA	169	313	0	1	0	0
CTSK	TGTGCAACACTGTGTGAC	282	44	0	0	1	0
CTSK	GTCATGACTGACCATGCA	172	366	0	0	0	0
CTSK	ACCATGACTGACCATGAC	68	67	0	0	0	0
CTSK	CACATGACTGACCATGTG	351	530	3	0	0	0
CTSK	TGCATGACTGACACGTGT	1158	1236	12	0	0	3
CTSK	GTCATGACTGACACGTCA	133	200	0	0	0	1
CTSL1	CACATGACTGACTGACCA	41	114	0	0	0	0
CTSL1	TGCATGACTGACTGACAC	81	107	0	0	0	0
CTSL1	ACCATGACTGACTGACTG	610	579	0	0	0	1
CTSL1	CACATGACTGACTGTGGT	533	251	0	1	0	0
CTSL1	ACCATGACTGACTGTGCA	201	141	1	0	0	0
CTSL2	GTCATGACTGACACCAGT	226	593	0	0	1	0
CTSL2	ACCATGACTGACACGTAC	29	41	0	0	0	0
CTSL2	CACATGACTGACACGTTG	125	100	0	0	0	0
CTSL2	CACATGACTGACACCACA	256	219	0	0	0	0
CTSL2	ACTGCAACACTGTGTGTG	379	336	0	0	0	0
CTSL2	CATGCAACTGGTGTGTGT	2216	1720	26	1	0	1
CTSS	ACTGCAACTGGTGTGTCA	923	815	2	1	1	1
CTSS	ACCATGACTGTGGTACTG	464	366	0	1	0	0
CTSS	CACATGACTGTGGTTGGT	215	273	0	0	0	1
CTSS	ACCATGACTGTGGTTGCA	47	177	1	0	0	0
CTSS	GTCATGACTGTGGTTGAC	22	39	0	0	0	0
CTSS	TGCATGACTGTGGTTGTG	644	744	0	0	0	1
CTTN	GTACCATGACCACAGTGT	722	1027	1	0	0	0
CTTN	CAACCATGACCACAGTCA	813	331	0	0	0	0
CTTN	TGACCATGACCACAGTAC	70	37	0	0	0	1
CTTN	ACACCATGACCACAGTTG	854	661	0	1	0	2
CTTN	CATGCAGTCAGTTGCACA	127	85	0	0	0	1
CTTN	ACTGCAACCATGGTGTGT	281	281	0	0	0	1
CUGBP1	TGACGTACACCACACATG	513	512	0	1	1	321
CUGBP1	ACACGTACACCACACACA	265	98	0	0	0	0
CUGBP1	GTACGTACACCACACAAC	11	24	0	0	0	0
CUGBP1	ACACGTACACCACAACGT	475	691	1	0	0	0
CUGBP1	TGACGTACACCACAACCA	301	233	0	0	0	0
CUGBP2	ACACTGTGGTTGCAGTGT	606	392	0	0	0	0

CUGBP2	TGACTGTGGTTGCAGTCA	360	599	1	0	0	0
CUGBP2	CAACTGTGGTTGCAGTAC	38	94	0	0	0	0
CUGBP2	GTACTGTGGTTGCAGTTG	1016	707	0	0	0	27
CUGBP2	TGACTGTGGTTGCACAGT	230	501	2	1	0	0
CUL1	CACATGACTGGTGTTGTG	779	530	0	0	1	0
CUL1	TGCATGACTGGTGTTGGT	158	71	0	0	0	0
CUL1	GTCATGACTGGTGTTGCA	223	263	1	0	0	0
CUL1	ACCATGACTGGTGTTGAC	9	18	0	0	0	0
CUL1	ACTGCAGTACGTGTCACA	155	107	1	1	0	0
CUL1	ACTGCATGCACACATGGT	518	399	0	0	0	0
CUL2	ACACGTGTGTACTGACCA	449	298	1	0	1	0
CUL2	GTACGTGTGTACTGACAC	86	56	0	0	0	0
CUL2	TGACGTGTGTACTGACTG	345	409	0	1	0	0
CUL2	ACACGTGTGTACTGTGGT	140	123	1	0	0	0
CUL2	TGACGTGTGTACTGTGCA	179	146	0	0	0	0
CUL2	ACTGCAACCAGTACCAAC	15	18	0	0	0	0
CUL3	CAACTGTGGTGTGTACTG	879	230	1	0	0	0
CUL3	GTACTGTGGTGTGTTGGT	754	522	1	0	0	99
CUL3	CAACTGTGGTGTGTTGCA	932	398	2	1	0	0
CUL3	TGACTGTGGTGTGTTGAC	16	16	0	0	0	0
CUL3	ACACTGTGGTGTGTTGTG	123	68	0	0	0	0
CUZD1	ACTGGTACTGGTGTCAAC	46	52	0	0	0	0
CUZD1	CATGGTACTGGTGTCATG	164	133	0	1	0	0
CUZD1	GTTGGTACTGGTGTACGT	139	231	0	0	0	1
CUZD1	CATGGTACTGGTGTACCA	20	17	0	0	0	0
CUZD1	TGTGGTACTGGTGTACAC	41	48	0	0	0	0
CX3CL1	ACACTGGTCACAGTGTGT	275	191	0	0	0	0
CX3CL1	TGACTGGTCACAGTGTCA	238	327	1	0	0	0
CX3CL1	CAACTGGTCACAGTGTAC	135	44	0	0	0	0
CX3CL1	GTACTGGTCACAGTGTTG	355	361	128	0	0	4
CX3CL1	TGACTGGTCACAGTCAGT	110	67	0	0	0	0
CX3CR1	GTACGTCAGTGTGTACGT	200	229	0	0	0	0
CX3CR1	CAACGTCAGTGTGTACCA	438	350	1546	5	0	1
CX3CR1	TGACGTCAGTGTGTACAC	15	33	0	0	0	0
CX3CR1	ACACGTCAGTGTGTACTG	519	130	0	0	0	0
CX3CR1	CAACGTCAGTGTGTTGGT	912	336	1	0	0	0
CXCL10	TGACTGGTCACAACTGGT	1199	302	2	192	0	1
CXCL10	GTACTGGTCACAACTGCA	175	246	0	1	0	0
CXCL10	ACACTGGTCACAACTGAC	35	36	0	0	0	0
CXCL10	CAACTGGTCACAACTGTG	256	265	1	0	0	0
CXCL10	TGACTGGTCACATGGTGT	1081	1137	3	1	0	0
CXCL11	TGACTGGTCAGTGTGTGT	955	1024	1	1	0	4
CXCL11	GTACTGGTCAGTGTGTCA	60	106	1	0	0	0
CXCL11	ACACTGGTCAGTGTGTAC	74	30	0	0	0	0
CXCL11	CAACTGGTCAGTGTGTTG	1183	1494	3	1	0	1
CXCL11	GTACTGGTCAGTGTCAGT	110	132	0	0	0	0
CXCL11	ACTGCAACGTCACAGTCA	191	204	1	2	0	0
CXCL12	ACCATGACACTGGTCATG	47	60	0	0	0	0
CXCL12	CACATGACACTGGTACGT	128	122	0	0	0	0
CXCL12	ACCATGACACTGGTACCA	111	118	0	0	0	0
CXCL12	GTCATGACACTGGTACAC	51	46	0	0	0	0
CXCL12	TGCATGACACTGGTACTG	38	47	1	0	0	0
CXCL13	CAACTGGTCACAGTTGGT	259	193	0	0	0	0
CXCL13	ACACTGGTCACAGTTGCA	139	439	3	1	0	0
CXCL13	GTACTGGTCACAGTTGAC	9	11	1	0	0	0
CXCL13	TGACTGGTCACAGTTGTG	545	594	0	0	0	0
CXCL13	GTTGCACAACGTACTGTG	215	532	0	0	0	0
CXCL13	ACTGCACAACGTTGGTGT	419	366	0	1	0	0
CXCL2	GTACTGGTCAGTGTACAC	11	127	1	0	0	0
CXCL2	TGACTGGTCAGTGTTGCA	235	196	0	0	0	0
CXCL2	CAACTGGTCAGTGTTGAC	7	34	0	0	0	0
CXCL2	GTACTGGTCACAACACTG	389	379	0	1	0	0
CXCL2	CATGCAGTGTCATGACTG	109	141	0	0	0	1
CXCL3	TGACTGGTCAGTGTACTG	74	77	2	0	0	0
CXCL3	ACACTGGTCAGTGTTGGT	414	690	0	0	0	0
CXCL3	ACACTGGTCACAACACGT	31	67	0	0	0	0
CXCL3	TGACTGGTCACAACACCA	215	186	0	0	0	54
CXCL3	CAACTGGTCACAACACAC	286	154	0	0	0	0
CXCL6	TGACTGGTCAGTCAGTCA	295	326	1	587	1	0
CXCL6	GTACTGGTCAGTCAGTTG	1331	1006	1	4	1	0
CXCL6	GTACTGGTCAGTGTTGTG	719	514	0	1	0	1
CXCL6	ACACTGGTCAGTCAGTGT	495	427	0	0	0	0
CXCL6	CAACTGGTCAGTCAGTAC	256	114	0	0	0	0
CXCL9	TGACTGGTCACACATGAC	52	162	1	1	1	0
CXCL9	ACACTGGTCACACATGTG	132	182	0	0	0	1

CXCL9	GTACTGGTCACAACGTGT	494	409	2	0	0	0
CXCL9	CAACTGGTCACAACGTCA	477	411	2	0	0	0
CXCL9	TGACTGGTCACAACGTAC	142	10	0	0	0	0
CXCL9	GTTGCACAGTTGTGGTAC	47	44	0	0	0	0
CXCR3	ACACGTCAGTGTGTTGCA	34	42	0	0	0	0
CXCR3	GTACGTCAGTGTGTTGAC	58	33	0	0	0	0
CXCR3	TGACGTCAGTGTGTTGTG	479	927	1	0	0	1
CXCR3	CAACGTCAGTGTCAGTGT	126	100	0	0	0	17
CXCR3	ACACGTCAGTGTCAGTCA	168	191	0	0	0	0
CXCR3	CATGCACAGTACTGACAC	25	24	0	0	0	0
CXCR4	ACCATGTGGTTGCAGTTG	364	272	0	2	0	0
CXCR4	CACATGTGGTTGCACAGT	151	211	0	0	0	0
CXCR4	ACCATGTGGTTGCACACA	155	88	0	0	0	5
CXCR4	GTCATGTGGTTGCACAAC	7	11	0	0	0	0
CXCR4	TGCATGTGGTTGCACATG	15	24	0	0	0	0
CXCR6	GTACGTCAGTGTCAGTAC	17	34	0	0	0	1
CXCR6	TGACGTCAGTGTCAGTTG	182	205	1	1	0	7
CXCR6	ACACGTCAGTGTCACAGT	200	197	0	0	0	0
CXCR6	TGACGTCAGTGTCACACA	20	52	0	0	0	0
CXCR6	CAACGTCAGTGTCACAAC	3	0	0	0	0	0
CXCR6	CATGCACAACCACAACGT	297	62	0	1	0	0
CYB5R1	GTACACGTTGTGACTGGT	210	308	11	3	21	23
CYB5R1	CAACACGTTGTGACACTG	104	159	0	0	2	1
CYB5R1	GTACACGTTGTGACACCA	255	211	2	1	1	1
CYB5R1	TGACACGTTGTGACACGT	55	78	10188	12	0	0
CYB5R1	ACACACGTTGTGACACAC	36	36	0	0	0	0
CYB5R3	CAACCATGGTACACCATG	320	668	0	1	1	0
CYB5R3	ACACCATGGTACACCAAC	34	65	0	0	0	0
CYB5R3	GTACCATGGTACACACGT	64	68	0	0	0	0
CYB5R3	CAACCATGGTACACACCA	537	323	0	1	0	0
CYB5R3	TGACCATGGTACACACAC	28	53	0	0	0	0
CYBA	ACTGCAGTTGTGCAACGT	410	626	3	617	3	1
CYBA	CAACTGACACCATGGTTG	381	319	0	1	1	32
CYBA	GTACTGACACCATGCAGT	186	213	0	0	0	0
CYBA	CAACTGACACCATGCACA	478	163	0	0	0	0
CYBA	TGTGCAGTTGTGCACATG	65	100	0	0	0	0
CYBB	ACACTGACACCATGACCA	150	155	41	60	10	73
CYBB	ACACTGACACCATGCATG	292	448	2	0	1	0
CYBB	TGACTGACACCATGCAAC	74	89	0	0	0	2
CYBB	CAACTGACACCATGACGT	192	212	0	0	0	0
CYBB	TGTGCAGTTGTGCAACCA	281	241	0	0	0	0
CYBB	TGTGCACAACACACACCA	92	91	0	0	0	0
CYC1	GTACTGACACCATGTGTG	200	422	0	1	0	0
CYC1	GTACTGACACACGTGTGT	418	491	0	0	0	1
CYC1	CAACTGACACACGTGTCA	156	141	0	0	0	0
CYC1	TGACTGACACACGTGTAC	42	53	0	0	0	0
CYC1	ACACTGACACACGTGTTG	270	308	4	0	0	0
CYCS	TGACTGACACCAACCATG	901	548	2	0	2	0
CYCS	GTACTGCAACGTCATGGT	628	376	2	0	1	0
CYCS	ACACTGACACCAACACGT	34	37	0	0	0	0
CYCS	TGACTGACACCAACACCA	708	457	0	1	0	2
CYCS	CAACTGACACCAACACAC	105	254	0	0	0	0
CYGB	TGACTGCAGTTGACCAAC	22	19	0	0	0	0
CYGB	ACACTGCAGTTGACCATG	95	179	0	1	0	13
CYGB	CAACTGCAGTTGACACGT	98	391	1	0	0	1
CYGB	ACACTGCAGTTGACACCA	36	94	0	0	0	0
CYGB	GTTGCAGTTGACTGACCA	177	205	0	0	0	1
CYLD	ACACCATGGTTGACGTAC	88	50	0	0	1	0
CYLD	ACACCATGGTTGCATGAC	33	34	0	0	0	0
CYLD	CAACCATGGTTGCATGTG	47	127	0	0	0	0
CYLD	TGACCATGGTTGACGTGT	513	602	2	0	0	0
CYLD	GTACCATGGTTGACGTCA	36	104	2	1	0	1
CYLD	GTTGCAACGTGTACACCA	186	270	0	1	0	0
CYP11A1	TGACTGACCATGTGCATG	585	219	0	0	0	0
CYP11A1	ACACTGACCATGTGACGT	458	295	1	0	0	0
CYP11A1	TGACTGACCATGTGACCA	169	126	0	0	0	0
CYP11A1	CAACTGACCATGTGACAC	32	33	0	0	0	0
CYP11A1	GTACTGACCATGTGACTG	813	529	4	1	0	0
CYP11B1	CAACTGACACGTGTCACA	123	84	0	25	0	0
CYP11B1	TGACTGACACGTGTCAAC	51	106	0	0	0	0
CYP11B1	ACACTGACACGTGTCATG	121	44	0	0	0	0
CYP11B1	CAACTGACACGTGTACGT	75	46	0	0	0	0
CYP11B1	GTTGCAGTTGTGACGTCA	74	102	0	0	0	0
CYP11B2	ACACTGACACGTGTACCA	135	105	0	0	0	0
CYP11B2	GTACTGACACGTGTACAC	14	17	0	0	0	0

CYP11B2	TGACTGACACGTGTACTG	218	322	1	0	0	0
CYP11B2	ACACTGACACGTGTTGGT	227	119	1	0	0	129
CYP11B2	TGACTGACACGTGTTGCA	107	116	3	1	0	5
CYP17A1	CAACTGACACGTCAGTAC	32	55	1	0	1	0
CYP17A1	CAACTGACACGTGTTGAC	28	37	0	0	0	0
CYP17A1	GTACTGACACGTGTTGTG	600	503	0	2	0	0
CYP17A1	ACACTGACACGTCAGTGT	242	127	0	0	0	0
CYP17A1	TGACTGACACGTCAGTCA	117	123	0	0	0	0
CYP19A1	CAACTGACACGTCACATG	401	316	1	0	1	0
CYP19A1	GTACTGACACGTCAGTTG	368	299	0	0	0	0
CYP19A1	TGACTGACACGTCACAGT	264	371	0	0	0	0
CYP19A1	GTACTGACACGTCACACA	203	158	0	0	0	1
CYP19A1	ACACTGACACGTCACAAC	5	23	0	0	0	0
CYP19A1	TGTGCAACGTGTCAGTCA	108	203	1	0	0	0
CYP1A1	ACACTGACACACGTACGT	82	71	0	1	1	0
CYP1A1	CAACTGACACACGTCAGT	339	121	0	0	0	0
CYP1A1	ACACTGACACACGTCACA	15	18	0	0	0	0
CYP1A1	GTACTGACACACGTCAAC	72	55	0	0	0	0
CYP1A1	TGACTGACACACGTCATG	106	60	0	0	0	0
CYP1A1	TGTGCACATGTGCACACA	25	26	0	0	0	3
CYP1A2	ACACTGACACACGTTGAC	32	29	0	0	0	0
CYP1A2	CAACTGACACACGTTGTG	180	243	0	1	0	39
CYP1A2	TGACTGACACACCAGTGT	1197	654	3	1	0	10
CYP1A2	GTACTGACACACCAGTCA	219	150	0	0	0	0
CYP1A2	ACACTGACACACCAGTAC	259	155	0	0	0	4
CYP1B1	CAACTGCATGTGTGGTCA	990	1128	5	2	1	11
CYP1B1	TGACTGCATGTGACTGAC	26	42	0	0	0	0
CYP1B1	ACACTGCATGTGACTGTG	436	366	0	0	0	0
CYP1B1	GTACTGCATGTGTGGTGT	309	359	1	0	0	0
CYP1B1	TGACTGCATGTGTGGTAC	34	47	0	0	0	0
CYP1B1	CATGCACATGTGCACAAC	87	16	0	1	0	0
CYP21A2	GTACTGACACGTCAACGT	101	177	0	0	0	0
CYP21A2	CAACTGACACGTCAACCA	278	366	1	0	0	15
CYP21A2	TGACTGACACGTCAACAC	12	13	0	0	0	0
CYP21A2	ACACTGACACGTCAACTG	111	172	0	0	0	0
CYP21A2	CATGACCAGTTGTGCAGT	381	269	0	0	0	1
CYP24A1	CAACTGACACGTCATGGT	86	57	0	0	0	0
CYP24A1	ACACTGACACGTCATGCA	215	228	0	0	0	0
CYP24A1	GTACTGACACGTCATGAC	61	47	0	0	0	0
CYP24A1	TGACTGACACGTCATGTG	1356	373	1	1	0	2
CYP24A1	CATGCAGTCACAACTGAC	48	48	0	0	0	0
CYP26A1	ACACTGACACGTACCAGT	363	404	0	0	1	0
CYP26A1	CAACTGACACGTACGTGT	109	148	0	0	0	0
CYP26A1	ACACTGACACGTACGTCA	336	455	2	0	0	0
CYP26A1	GTACTGACACGTACGTAC	41	22	0	1	0	0
CYP26A1	TGACTGACACGTACGTTG	131	89	0	0	0	0
CYP26A1	GTTGCAACGTACACTGAC	288	56	0	0	0	0
CYP27A1	TGACTGCATGTGGTGTGT	463	322	0	0	1	0
CYP27A1	TGACTGCATGACTGTGCA	153	131	0	1	0	0
CYP27A1	CAACTGCATGACTGTGAC	64	67	0	0	0	0
CYP27A1	GTACTGCATGACTGTGTG	434	507	0	0	0	0
CYP27A1	GTACTGCATGTGGTGTCA	346	345	2	0	0	10
CYP27A1	CATGCAACGTGTCAGTAC	66	49	0	0	0	0
CYP27B1	TGACTGACACGTACACGT	324	110	0	0	0	0
CYP27B1	GTACTGACACGTACACCA	128	40	0	0	0	0
CYP27B1	ACACTGACACGTACACAC	44	49	0	1	0	0
CYP27B1	CAACTGACACGTACACTG	458	202	0	0	0	0
CYP27B1	CATGCAGTTGTGACGTTG	259	344	4	0	0	0
CYP2A13	TGACTGACCAACACTGGT	321	298	15	15	7	40373
CYP2A13	ACACTGACCAACACACGT	40	50	0	0	0	0
CYP2A13	TGACTGACCAACACACCA	298	251	0	1	0	0
CYP2A13	CAACTGACCAACACACAC	129	156	0	0	0	0
CYP2A13	GTACTGACCAACACACTG	748	448	0	2	0	0
CYP2A13	GTTGCAACCAGTTGGTAC	38	30	0	0	0	0
CYP2A6	ACACTGACACACCAACCA	871	596	2	0	1	1
CYP2A6	CAACTGACACACCAACGT	338	352	0	0	0	0
CYP2A6	GTACTGACACACCAACAC	173	142	0	0	0	0
CYP2A6	TGACTGACACACCAACTG	484	678	0	1	0	1
CYP2A6	ACACTGACACACCATGGT	296	494	0	1	0	1
CYP2A7	ACACTGACCAACCACAGT	246	216	1	0	0	153
CYP2A7	TGACTGACCAACCACACA	45	79	0	0	0	0
CYP2A7	CAACTGACCAACCACAAC	9	17	0	0	0	0
CYP2A7	GTACTGACCAACCACATG	375	303	0	0	0	0
CYP2A7	TGACTGACCAACCAACGT	135	215	0	0	0	0
CYP2B6	CATGCAGTTGTGGTTGCA	198	322	0	0	1	0

CYP2B6	GTACTGACCAACACTGCA	144	114	0	0	0	0
CYP2B6	ACACTGACCAACACTGAC	29	29	0	0	0	0
CYP2B6	CAACTGACCAACACTGTG	180	200	0	0	0	0
CYP2B6	TGTGCAGTTGTGGTTGAC	26	15	0	0	0	0
CYP2B6	ACTGCAACCATGGTCAAC	23	16	0	0	0	2
CYP2C18	GTACTGACCATGGTGTCA	456	543	1	1	1	4
CYP2C18	TGACTGACCAACTGTGCA	112	116	0	0	0	0
CYP2C18	CAACTGACCAACTGTGAC	79	106	0	0	0	0
CYP2C18	GTACTGACCAACTGTGTG	325	346	0	0	0	0
CYP2C18	TGACTGACCATGGTGTGT	186	226	1	0	0	0
CYP2C18	GTTGCAACGTGTGTGTCA	395	212	0	0	0	0
CYP2C19	TGACTGACCAACTGGTGT	327	176	1	0	1	0
CYP2C19	CAACTGACCAACTGGTTG	410	503	0	2	1	0
CYP2C19	GTACTGACCAACTGGTCA	222	286	0	0	0	1
CYP2C19	ACACTGACCAACTGGTAC	132	217	0	0	0	1
CYP2C19	GTACTGACCAACTGCAGT	116	101	0	0	0	0
CYP2C8	CAACTGACCAACTGCACA	106	104	0	0	0	61
CYP2C8	TGACTGACCAACTGCAAC	46	45	0	0	0	0
CYP2C8	ACACTGACCAACTGCATG	225	158	0	0	0	1
CYP2C8	CAACTGACCAACTGACGT	51	108	0	0	0	1
CYP2C8	GTTGGTTGTGCACAGTAC	173	107	1	0	0	0
CYP2C9	ACACTGACCAACTGACCA	229	349	0	0	1	0
CYP2C9	TGACTGACCAACTGACTG	459	415	0	0	1	0
CYP2C9	GTACTGACCAACTGACAC	103	98	0	0	0	0
CYP2C9	ACACTGACCAACTGTGGT	168	124	0	0	0	0
CYP2C9	ACTGCAGTTGTGGTTGTG	230	603	0	0	0	0
CYP2C9	ACTGCAACGTGTGTGTAC	133	174	3	0	0	0
CYP2D6	ACACTGACCATGGTGTAC	68	54	0	0	0	0
CYP2D6	CAACTGACCATGGTGTTG	502	682	0	0	0	0
CYP2D6	GTACTGACCATGGTCAGT	152	154	2	0	0	0
CYP2D6	CAACTGACCATGGTCACA	157	116	0	0	0	0
CYP2D6	TGACTGACCATGGTCAAC	56	35	0	0	0	0
CYP2E1	ACACTGACCATGGTCATG	130	138	0	0	0	0
CYP2E1	CAACTGACCATGGTACGT	208	299	0	0	0	6
CYP2E1	ACACTGACCATGGTACCA	238	334	0	0	0	1
CYP2E1	GTACTGACCATGGTACAC	24	22	0	0	0	0
CYP2E1	ACTGCAGTTGTGACGTAC	28	60	0	0	0	36
CYP2F1	TGTGCAACCAGTTGGTTG	343	503	0	0	1	0
CYP2F1	TGACTGACCATGGTACTG	443	328	0	0	0	0
CYP2F1	ACACTGACCATGGTTGGT	654	304	0	0	0	1
CYP2F1	TGACTGACCATGGTTGCA	80	213	0	1	0	1
CYP2F1	CAACTGACCATGGTTGAC	145	74	1	1	0	0
CYP2F1	GTACTGACCATGGTTGTG	73	126	0	0	0	0
CYP2J2	CAACTGACCATGCAACCA	798	782	1	0	1	8
CYP2J2	GTACTGACCATGCACACA	101	101	0	0	0	0
CYP2J2	ACACTGACCATGCACAAC	83	83	0	0	0	0
CYP2J2	CAACTGACCATGCACATG	133	119	1	0	0	0
CYP2J2	GTACTGACCATGCAACGT	358	288	1	1	0	1
CYP2J2	GTTGCAACCAGTGTTGCA	67	273	0	0	0	0
CYP2R1	ACACTGACACCACATGTG	366	249	0	0	1	0
CYP2R1	CAACTGACACCAACGTCA	1159	988	3	138	1	2
CYP2R1	TGACTGACACCACATGAC	78	91	0	0	0	0
CYP2R1	GTACTGACACCAACGTGT	40	111	1	0	0	0
CYP2R1	CATGGTACTGCAGTTGCA	191	79	1	0	0	0
CYP2S1	CATGGTACGTCAACTGCA	206	560	0	0	1	0
CYP2S1	TGTGGTACGTCAACTGAC	18	21	0	0	0	0
CYP2S1	ACTGGTACGTCAACTGTG	28	60	200	2	0	0
CYP2S1	GTTGGTACGTCATGGTGT	172	94	0	0	0	0
CYP2S1	GTTGACGTACCACACATG	216	166	0	0	0	0
CYP2U1	GTACTGACACCAGTTGAC	27	27	0	0	0	0
CYP2U1	TGACTGACACCAGTTGTG	268	205	0	0	0	0
CYP2U1	CAACTGACACCACAGTGT	501	281	0	0	0	0
CYP2U1	GTTGCAGTTGTGCATGCA	113	149	0	0	0	14
CYP2U1	GTTGACCAGTCACATGTG	1038	1523	2	0	0	5
CYP39A1	GTACTGACACCAGTCACA	114	128	0	0	0	0
CYP39A1	ACACTGACACCAGTCAAC	12	5	0	0	0	0
CYP39A1	CAACTGACACCAGTCATG	168	233	0	0	0	0
CYP39A1	GTACTGACACCAGTACGT	466	366	0	0	0	0
CYP39A1	CAACTGACACCAGTACCA	353	417	0	0	0	8
CYP39A1	CATGCATGCACAACGTAC	85	95	0	0	0	0
CYP3A4	TGACTGACCATGCAACAC	18	19	0	0	0	0
CYP3A4	ACACTGACCATGCAACTG	533	693	0	0	0	0
CYP3A4	CAACTGACCATGCATGGT	336	384	0	2	0	0
CYP3A4	ACACTGACCATGCATGCA	322	439	0	0	0	0
CYP3A4	GTACTGACCATGCATGAC	87	41	0	1	0	0

CYP3A4	ACTGCAACGTGTCAGTGT	519	436	1	1	0	0
CYP3A43	TGTGCAACGTACACTGTG	84	152	0	0	1	1
CYP3A43	GTACTGACCATGACACCA	118	211	0	0	0	0
CYP3A43	ACACTGACCATGACACAC	106	222	0	0	0	8
CYP3A43	CAACTGACCATGACACTG	1030	944	1	0	0	1
CYP3A43	GTACTGACCATGACTGGT	501	435	1	1	0	0
CYP3A43	CATGCAACGTACTGGTGT	1414	1249	5	1	0	1
CYP3A5	ACACTGACCATGACGTCA	380	254	0	29	721	0
CYP3A5	TGACTGACCATGCATGTG	1092	1124	2	0	0	0
CYP3A5	CAACTGACCATGACGTGT	552	429	1	1	0	0
CYP3A5	GTACTGACCATGACGTAC	43	53	0	0	0	0
CYP3A5	TGACTGACCATGACGTTG	223	310	1	0	0	0
CYP3A5	CATGCAACGTCACAACTG	1488	1019	3	0	0	0
CYP3A7	GTACTGACCAACACGTGT	1337	575	1	0	1	2
CYP3A7	TGACTGACCAACCATGAC	81	136	0	0	0	0
CYP3A7	ACACTGACCAACCATGTG	277	625	1	1	0	0
CYP3A7	CAACTGACCAACACGTCA	232	471	0	0	0	0
CYP3A7	TGACTGACCAACACGTAC	50	86	0	0	0	0
CYP3A7	TGTGCACATGTGACCATG	118	172	0	0	0	0
CYP46A1	GTACTGACCACAGTTGCA	51	68	0	0	0	0
CYP46A1	ACACTGACCACAGTTGAC	87	87	0	0	0	0
CYP46A1	CAACTGACCACAGTTGTG	188	323	0	0	0	0
CYP46A1	TGACTGACCACACAGTGT	255	297	0	0	0	0
CYP46A1	GTACTGACCACACAGTCA	54	74	0	0	0	0
CYP46A1	TGTGCACAACCAGTTGGT	131	137	0	1	0	0
CYP4A11	ACACCAGTACTGCATGAC	174	76	0	0	0	0
CYP4A11	CAACCAGTACTGCATGTG	324	398	0	0	0	0
CYP4A11	GTACCAGTACTGACGTCA	592	420	0	1	0	7
CYP4A11	ACACCAGTACTGACGTAC	87	30	0	0	0	0
CYP4A11	TGTGCAGTGTCAGTACCA	164	210	0	1	0	0
CYP4A11	GTTGCACATGTGACTGCA	219	198	0	0	0	0
CYP4A22	TGACCAGTACTGACGTGT	612	802	2	0	0	1
CYP4A22	TGTGACCAGTCAACGTCA	149	232	1	0	0	0
CYP4A22	CATGACCAGTCAACGTAC	73	82	0	0	0	0
CYP4A22	GTTGACCAGTCAACGTTG	126	394	3	1	0	0
CYP4A22	TGTGACCAGTCAACCAGT	494	279	2	0	0	12
CYP4B1	ACACTGACCATGACCAGT	122	202	1	0	0	0
CYP4B1	TGACTGACCATGACCACA	151	155	0	0	0	0
CYP4B1	CAACTGACCATGACCAAC	152	63	0	0	0	0
CYP4B1	GTACTGACCATGACCATG	124	146	0	1	0	1
CYP4B1	TGACTGACCATGACACGT	31	26	0	0	0	0
CYP4B1	ACTGCACATGTGACTGAC	44	40	0	0	0	0
CYP4F12	TGACTGACACGTGTGTGT	229	140	0	0	0	0
CYP4F12	GTACTGACACGTGTGTCA	597	666	1	0	0	18
CYP4F12	ACACTGACACGTGTGTAC	76	137	0	0	0	0
CYP4F12	CAACTGACACGTGTGTTG	252	320	2	0	0	2
CYP4F12	GTACTGACACGTGTCAGT	111	132	0	0	0	0
CYP4F2	TGACTGACACGTTGCATG	593	157	6	6	7	10126
CYP4F2	ACACTGACACGTTGACGT	508	399	0	1	1	0
CYP4F2	GTACTGACACGTTGCAAC	5	16	0	0	0	0
CYP4F2	TGACTGACACGTTGACCA	369	230	0	0	0	0
CYP4F2	CAACTGACACGTTGACAC	7	7	0	0	0	0
CYP4F3	CAACTGACACCAACTGTG	120	72	0	0	0	0
CYP4F3	TGACTGACACCATGGTGT	338	428	1	0	0	0
CYP4F3	GTACTGACACCATGGTCA	949	814	0	1	0	0
CYP4F3	ACACTGACACCATGGTAC	83	115	0	0	0	0
CYP4F3	GTTGACACCAACACGTTG	305	347	2	0	0	0
CYP51A1	GTACTGACACGTACTGGT	48	58	0	0	0	0
CYP51A1	CAACTGACACGTACTGCA	22	13	0	0	0	0
CYP51A1	TGACTGACACGTACTGAC	43	31	0	0	0	0
CYP51A1	ACACTGACACGTACTGTG	408	501	0	0	0	0
CYP51A1	GTACTGACACGTTGGTGT	290	618	0	0	0	1
CYP51A1	GTTGCAACGTGTCAGTTG	269	186	0	0	0	0
CYP7A1	GTACTGACCATGTGGTGT	536	587	0	0	1	0
CYP7A1	CAACTGACCATGACTGCA	62	90	0	0	0	0
CYP7A1	TGACTGACCATGACTGAC	121	117	1	0	0	1
CYP7A1	ACACTGACCATGACTGTG	239	201	0	0	0	1
CYP7A1	CAACTGACCATGTGGTCA	246	483	1	0	0	0
CYP7A1	TGTGCAACGTGTGTACTG	877	339	0	1	0	1
CYP7B1	GTACTGACACCAACACTG	678	2075	1	0	0	0
CYP7B1	TGACTGACACCAACTGGT	316	399	3	0	0	0
CYP7B1	GTACTGACACCAACTGCA	196	182	0	0	0	0
CYP7B1	ACACTGACACCAACTGAC	21	6	0	0	0	0
CYP7B1	TGTGCAGTTGTGCATGGT	205	418	0	0	0	0
CYP7B1	ACTGCAACGTGTGTTGGT	602	421	1	0	0	0

CYP8B1	ACACTGACCATGTGGTTG	979	1061	0	1	1	0
CYP8B1	TGACTGACCATGTGGTAC	44	84	0	0	0	0
CYP8B1	CAACTGACCATGTGCAGT	80	140	0	0	0	0
CYP8B1	ACACTGACCATGTGCACA	374	243	0	1	0	1
CYP8B1	GTACTGACCATGTGCAAC	9	12	0	0	0	0
CYP8B1	TGTGCAACGTGTCACAGT	96	100	0	0	0	0
CYR61	TGTGGTGTGTTGACACAC	30	43	0	0	0	0
CYR61	ACTGGTGTGTTGACACTG	240	220	0	1	0	0
CYR61	CATGGTCAGTGTCAACCA	147	160	0	0	0	1
CYR61	TGTGGTCAGTGTCAACAC	83	79	0	0	0	0
CYR61	ACTGGTCAGTGTCAACTG	486	550	0	0	0	0
CYSLTR1	GTACGTCAGTGTCACATG	125	101	0	0	0	0
CYSLTR1	TGACGTCAGTGTCAACGT	145	156	0	0	0	0
CYSLTR1	GTACGTCAGTGTCAACCA	568	516	1	0	0	0
CYSLTR1	ACACGTCAGTGTCAACAC	54	62	0	0	0	0
CYSLTR1	CAACGTCAGTGTCAACTG	278	243	1	2	0	1
CYSLTR1	GTTGCATGGTCATGTGCA	406	369	0	0	0	2
CYSLTR2	GTACGTCAGTGTCATGGT	567	393	0	1	0	0
CYSLTR2	CAACGTCAGTGTCATGCA	316	153	0	1	0	0
CYSLTR2	TGACGTCAGTGTCATGAC	70	248	0	0	0	0
CYSLTR2	ACACGTCAGTGTCATGTG	188	209	0	0	0	0
CYSLTR2	GTACGTCAGTGTACGTGT	275	374	0	0	0	0
CYSLTR2	ACTGCATGGTCATGTGAC	27	30	0	0	0	0
DAAM1	CATGGTCACAGTGTCAGT	316	244	1	0	0	0
DAAM1	ACTGGTCACAGTACTGCA	95	127	0	0	0	0
DAAM1	GTTGGTCACAGTACTGAC	24	22	0	0	0	0
DAAM1	TGTGGTCACAGTACTGTG	948	238	0	0	0	0
DAAM1	CATGGTCACAGTTGGTGT	599	396	2	0	0	0
DAAM1	GTTGCAACCATGACGTGT	625	621	0	1	0	0
DAB1	GTACTGACCACATGACCA	304	313	5	11	5	20211
DAB1	ACACTGACCACATGACAC	60	70	0	0	0	0
DAB1	CAACTGACCACATGACTG	539	468	0	0	0	1
DAB1	GTACTGACCACATGTGGT	754	872	2	1	0	0
DAB1	CAACTGACCACATGTGCA	483	604	0	0	0	19
DAB2	TGTGGTACACCAACCATG	309	287	1	0	1	0
DAB2	ACTGGTACACCAACCACA	95	67	2	0	0	0
DAB2	GTTGGTACACCAACCAAC	18	28	0	0	0	0
DAB2	ACTGGTACACCAACACGT	44	54	0	0	0	0
DAB2	TGTGGTACACCAACACCA	390	268	1	0	0	4
DACT1	GTTGGTTGCAGTGTGTTG	1146	1450	2	2	2	2
DACT1	CATGGTTGCAGTGTGTAC	76	155	0	0	0	0
DACT1	TGTGGTTGCAGTGTCAGT	101	158	0	0	0	0
DACT1	GTTGGTTGCAGTGTCACA	53	33	1	0	0	0
DACT1	ACTGGTTGCAGTGTCAAC	55	69	0	18	0	0
DAD1	GTACTGTGTGGTACACCA	169	159	0	0	0	0
DAD1	ACACTGTGTGGTACACAC	18	10	0	0	0	0
DAD1	CAACTGTGTGGTACACTG	170	165	0	0	0	0
DAD1	GTACTGTGTGGTACTGGT	267	289	1	0	0	0
DAD1	ACTGGTGTCAGTCAACTG	346	763	2	0	0	0
DAD1	TGTGCACAGTACCACAGT	99	108	0	0	0	0
DAG1	GTACACTGCACAACTGCA	117	136	7	12	3	14
DAG1	ACACACTGCACAACTGAC	120	142	0	0	0	0
DAG1	CAACACTGCACAACTGTG	53	77	0	0	0	0
DAG1	TGACACTGCACATGGTGT	204	240	1	0	0	0
DAG1	GTACACTGCACATGGTCA	213	238	0	0	0	7
DAO	TGACACGTTGCAACCAGT	7	13	0	0	0	0
DAO	GTACACGTTGCAACCACA	88	88	0	0	0	0
DAO	ACACACGTTGCAACCAAC	71	73	0	1	0	0
DAO	CAACACGTTGCAACCATG	542	159	0	1	0	0
DAO	ACTGACACACTGCAACTG	1068	549	2	2	0	0
DAP	GTTGGTGTCAACTGTGGT	948	330	0	2	0	6
DAP	CATGGTGTCAACTGTGCA	44	150	0	0	0	0
DAP	TGTGGTGTCAACTGTGAC	111	83	0	0	0	0
DAP	ACTGGTGTCAACTGTGTG	426	274	0	0	0	0
DAP	TGTGCACAGTGTCACACA	72	152	0	0	0	0
DAP	ACTGCACACATGGTCAGT	214	337	0	0	0	0
DAPK1	ACCATGCATGCAACACGT	17	8	0	0	0	0
DAPK1	TGCATGCATGCAACACCA	632	311	0	0	0	0
DAPK1	CACATGCATGCAACACAC	113	125	0	0	0	1
DAPK1	GTCATGCATGCAACACTG	1036	774	0	1	0	3
DAPK1	CAACGTTGTGACCACATG	603	317	0	1	0	6
DAPK2	TGCATGACGTCATGCAGT	121	77	0	0	1	2
DAPK2	TGCATGACGTCATGGTCA	955	606	1	2	0	0
DAPK2	CACATGACGTCATGGTAC	32	25	0	0	0	0
DAPK2	GTCATGACGTCATGGTTG	837	613	0	0	0	1

DAPK2	GTCATGACGTCATGCACA	168	210	1	0	0	1
DAPK3	CACATGCAACGTACACCA	234	420	0	0	0	0
DAPK3	TGCATGCAACGTACACAC	36	29	0	0	0	0
DAPK3	ACCATGCAACGTACACTG	341	391	1	0	0	8
DAPK3	CACATGCAACGTACTGGT	761	515	0	0	0	2
DAPK3	ACCATGCAACGTACTGCA	55	80	0	0	0	0
DAPK3	CATGCACACACATGACGT	281	160	0	1	0	0
DAPP1	TGCATGACCATGGTACGT	263	462	0	1	0	0
DAPP1	GTCATGACCATGGTACCA	38	54	0	0	0	0
DAPP1	ACCATGACCATGGTACAC	40	54	0	0	0	2
DAPP1	CACATGACCATGGTACTG	286	254	0	0	0	1
DAPP1	CATGGTCATGACCACAAC	12	23	0	0	0	0
DAPP1	CATGCACAACTGGTCACA	242	440	2	0	0	3
DARC	ACACGTCAGTCAACACCA	223	217	2	0	0	0
DARC	GTACGTCAGTCAACACAC	55	29	0	0	0	0
DARC	TGACGTCAGTCAACACTG	183	198	1	0	0	0
DARC	ACACGTCAGTCAACTGGT	416	257	0	0	0	0
DARC	TGACGTCAGTCAACTGCA	413	189	0	0	0	0
DARS	TGACACGTCAGTTGTGAC	87	84	0	1	0	0
DARS	ACACACGTCAGTTGTGTG	33	11	0	0	0	0
DARS	TGACACGTCACAGTGTGT	277	302	0	0	0	0
DARS	GTACTGTGCACAACCAGT	284	482	0	0	0	28
DARS	TGTGCAGTCAGTACACCA	407	427	1	1	0	4
DARS2	TGTGGTGTCACACACACA	201	120	0	1	0	0
DARS2	ACTGGTACCATGTGCACA	94	97	0	0	0	1
DARS2	GTTGGTACCATGTGCAAC	9	21	0	0	0	0
DARS2	TGTGGTACCATGTGCATG	60	62	0	0	0	0
DARS2	ACTGGTACCATGTGACGT	135	122	0	0	0	0
DAXX	GTCATGTGGTGTCAGTTG	1904	1205	3	0	2	0
DAXX	TGCATGTGGTGTCACAGT	101	120	1	0	0	0
DAXX	GTCATGTGGTGTCACACA	39	58	0	0	0	0
DAXX	ACCATGTGGTGTCACAAC	9	13	1	0	0	0
DAXX	CACATGTGGTGTCACATG	46	114	0	0	0	0
DAZAP2	CATGGTCAACTGCAGTTG	929	1259	0	1	2	0
DAZAP2	GTTGGTCAACTGCAGTCA	180	116	0	1	0	0
DAZAP2	ACTGGTCAACTGCAGTAC	65	24	0	0	0	0
DAZAP2	GTTGGTCAACTGCACAGT	79	107	0	1	0	0
DAZAP2	CATGGTCAACTGCACACA	105	129	0	2	0	0
DAZL	CAACTGTGTGTGGTCAAC	40	35	0	0	0	0
DAZL	GTACTGTGTGTGGTCATG	74	93	1	0	0	0
DAZL	TGACTGTGTGTGGTACGT	313	183	1	0	0	0
DAZL	GTACTGTGTGTGGTACCA	159	166	0	0	0	1
DAZL	ACACTGTGTGTGGTACAC	149	39	0	1	0	0
DAZL	CATGCAACTGACACGTCA	417	397	1	0	0	1
DBC1	CATGGTTGACTGCAGTGT	1728	1427	1	3	1	0
DBC1	TGTGGTTGACTGGTTGTG	335	410	1	0	0	0
DBC1	ACTGGTTGACTGCAGTCA	1570	795	1	1	0	0
DBC1	GTTGGTTGACTGCAGTAC	77	39	0	0	0	0
DBC1	TGTGGTTGACTGCAGTTG	217	321	1	0	0	0
DBH	ACACTGTGCAACACCAGT	212	279	2	66	18	0
DBH	TGACTGTGCAACACCACA	35	56	0	0	0	2
DBH	CAACTGTGCAACACCAAC	84	37	0	0	0	0
DBH	GTACTGTGCAACACCATG	110	117	1	0	0	0
DBH	TGACTGTGCAACACACGT	15	27	0	0	0	1
DBN1	ACACTGACCAACGTGTGT	421	585	1	0	2	0
DBN1	TGACTGACCACATGTGAC	5	11	0	0	0	0
DBN1	ACACTGACCACATGTGTG	739	235	1	1	0	8
DBN1	TGACTGACCAACGTGTCA	137	101	0	0	0	0
DBN1	CAACTGACCAACGTGTAC	125	334	1	0	0	0
DBN1	ACTGCAACCAGTGTACGT	584	382	1	0	0	1
DBP	ACACGTCATGCAGTCATG	119	88	0	0	0	0
DBP	CAACGTCATGCAGTACGT	275	582	1	0	0	0
DBP	ACACGTCATGCAGTACCA	155	172	0	0	0	0
DBP	ACTGCAGTGTGTCAACGT	266	256	0	0	0	1
DBP	TGTGCAGTGTGTCAACCA	38	64	0	0	0	0
DBP	TGTGCATGCAGTGTGTTG	260	158	0	0	0	0
DBT	ACACCAGTGTACTGGTGT	111	124	0	0	0	0
DBT	TGACCAGTGTACTGGTCA	117	146	0	0	0	0
DBT	CAACCAGTGTACTGGTAC	38	61	0	0	0	0
DBT	GTACCAGTGTACTGGTTG	204	279	0	0	0	0
DBT	TGACCAGTGTACTGCAGT	166	226	0	0	0	0
DCC	ACACCATGCACAGTCATG	253	122	0	0	1	0
DCC	CAACCATGCACAGTACGT	272	128	1	1	0	0
DCC	ACACCATGCACAGTACCA	382	379	0	0	0	0
DCC	GTACCATGCACAGTACAC	36	45	1	0	0	0

DCC	TGACCATGCACAGTACTG	84	83	0	0	0	1
DCI	CAACACCAACACCAGTTG	602	574	8	14	7	35
DCI	ACACACCAACACCAGTAC	48	91	0	0	0	0
DCI	GTACACCAACACCACAGT	86	103	0	0	0	0
DCI	CAACACCAACACCACACA	68	148	0	0	0	0
DCI	TGACACCAACACCACAAC	17	41	0	0	0	0
DCK	GTTGGTACTGTGCAGTCA	711	1414	1	1	1	7
DCK	TGACGTTGTGACACTGAC	24	40	0	0	0	0
DCK	CATGGTACTGTGGTTGTG	203	305	0	1	0	0
DCK	TGTGGTACTGTGCAGTGT	445	386	1	1	0	1
DCK	ACTGGTACTGTGCAGTAC	43	203	0	1	0	2
DCK	GTTGCACAGTACCACACA	148	98	0	0	0	2
DCLRE1A	CATGGTCACAACACTGAC	24	25	0	0	0	0
DCLRE1A	GTTGGTCACAACACTGTG	281	360	0	0	0	1
DCLRE1A	ACTGGTCACAACTGGTGT	192	224	0	0	0	0
DCLRE1A	TGTGGTCACAACTGGTCA	488	585	0	1	0	1
DCLRE1A	CATGGTCACAACTGGTAC	424	314	0	1	0	1
DCLRE1B	ACACTGACTGACTGTGAC	15	11	1	0	0	0
DCLRE1B	CAACTGACTGACTGTGTG	1135	779	1	0	0	3
DCLRE1B	GTACTGACTGTGGTGTGT	206	270	1	0	0	3
DCLRE1B	CAACTGACTGTGGTGTCA	129	212	0	0	0	0
DCLRE1B	TGACTGACTGTGGTGTAC	50	61	0	0	0	0
DCLRE1B	ACTGCAACTGCAACACTG	731	916	0	1	0	0
DCLRE1C	CAACTGTGTGACTGGTGT	388	754	0	0	0	0
DCLRE1C	ACACTGTGTGACTGGTCA	166	168	0	0	0	0
DCLRE1C	GTACTGTGTGACTGGTAC	19	35	0	0	0	0
DCLRE1C	TGACTGTGTGACTGGTTG	249	378	0	1	0	0
DCLRE1C	ACACTGTGTGACTGCAGT	88	114	0	0	0	1
DCN	TGACTGGTACTGACGTTG	325	453	1	0	1	0
DCN	CAACTGGTACTGACGTGT	323	352	1	1	0	0
DCN	ACACTGGTACTGACGTCA	479	492	0	0	0	12
DCN	GTACTGGTACTGACGTAC	9	20	0	0	0	0
DCN	TGTGCAGTGTCAGTGTAC	60	94	0	0	0	0
DCP1A	ACACGTTGCATGTGGTTG	956	551	0	0	2	1
DCP1A	CAACGTTGCATGTGGTCA	465	568	0	0	1	0
DCP1A	TGACGTTGCATGTGGTAC	59	84	0	0	0	0
DCP1A	CAACGTTGCATGTGCAGT	132	227	1	0	0	14
DCP1A	ACACGTTGCATGTGCACA	230	242	0	0	0	0
DCT	TGACACCAACACCAACTG	703	745	1	2	2	0
DCT	CAACACCAACACCAACGT	514	948	0	6	1	11
DCT	ACACACCAACACCACATG	305	273	2	1	0	12
DCT	ACACACCAACACCAACCA	636	524	0	1	0	1
DCT	GTACACCAACACCAACAC	59	38	0	0	0	0
DCT	ACTGCACAACCATGACAC	21	32	0	0	0	0
DCTD	GTTGACGTTGTGTGGTAC	232	72	0	1	1	0
DCTD	TGACACACACCAACCACA	80	55	0	0	0	0
DCTD	CAACACACACCAACCAAC	94	105	0	0	0	0
DCTD	GTACACACACCAACCATG	276	381	4	0	0	7
DCTD	TGACACACACCAACACGT	168	126	0	3	0	7
DCTN1	GTACTGACCAACGTGTTG	406	373	0	0	0	0
DCTN1	TGACTGACCAACGTCAGT	269	356	0	0	0	0
DCTN1	GTACTGACCAACGTCACA	37	44	1	0	0	0
DCTN1	ACACTGACCAACGTCAAC	18	23	0	0	0	0
DCTN1	CAACTGACCAACGTCATG	159	201	0	2	0	0
DCTN1	TGTGCACATGTGGTTGTG	102	196	0	1	0	0
DCXR	CAACCATGGTACCACACA	155	154	1	0	0	0
DCXR	TGACCATGGTACCACAAC	13	21	0	0	0	0
DCXR	ACACCATGGTACCACATG	158	134	0	0	0	0
DCXR	CAACCATGGTACCAACGT	179	105	0	0	0	0
DCXR	ACACCATGGTACCAACCA	443	219	1	1	0	0
DDB1	ACACTGTGACTGCATGTG	502	346	1	0	1	1
DDB1	ACTGCAGTACGTGTGTTG	385	386	0	0	1	0
DDB1	TGACTGTGACTGCATGAC	69	62	0	0	0	0
DDB1	GTACTGTGACTGACGTGT	468	642	0	0	0	1
DDB1	CAACTGTGACTGACGTCA	86	251	0	0	0	0
DDB2	TGTGGTGTGTACCATGTG	213	189	4	3	1	0
DDB2	CATGGTGTGTACCATGGT	76	106	0	0	0	0
DDB2	ACTGGTGTGTACCATGCA	95	100	1	0	0	2
DDB2	GTTGGTGTGTACCATGAC	29	28	0	0	0	0
DDB2	CATGGTGTGTACACGTGT	241	312	0	1	0	1
DDB2	ACTGCACACACATGACCA	326	564	0	1	0	0
DDC	ACCATGTGACACACACAC	12	42	0	0	0	0
DDC	CACATGTGACACACACTG	98	61	1	0	0	0
DDC	GTCATGTGACACACTGGT	370	462	0	276	0	0
DDC	CACATGTGACACACTGCA	370	332	0	0	0	0

DDC	TGCATGTGACACACTGAC	26	49	0	0	0	0
DDEF1	TGTGGTCACACACAACAC	272	64	0	0	1	0
DDEF1	ACTGGTCACACACAACTG	573	527	0	1	1	1
DDEF1	ACTGGTGTCAGTCACAAC	21	20	0	0	0	0
DDEF1	CATGGTCACACACAACCA	366	459	0	0	0	2
DDEF1	CATGGTCACACACATGGT	159	149	0	1	0	0
DDEF2	CAACCAGTTGACTGCAGT	118	201	0	0	0	0
DDEF2	ACACCAGTTGACTGCACA	191	308	0	0	0	0
DDEF2	GTACCAGTTGACTGCAAC	33	18	0	0	0	0
DDEF2	TGACCAGTTGACTGCATG	291	301	0	0	0	0
DDEF2	CATGGTGTCAGTCAGTAC	17	38	0	0	0	0
DDEF2	CATGCACAGTCAACACGT	118	118	0	1611	0	0
DDHD1	ACACACACCAGTACGTTG	1834	1065	7	9	4	11
DDHD1	ACACACACCAGTCATGTG	440	466	0	0	0	9
DDHD1	GTACACACCAGTACGTGT	240	241	0	0	0	0
DDHD1	CAACACACCAGTACGTCA	402	139	0	0	0	0
DDHD1	TGACACACCAGTACGTAC	101	231	0	0	0	0
DDIT3	ACACGTGTCATGACCAAC	16	35	0	0	0	0
DDIT3	CAACGTGTCATGACCATG	192	180	0	0	0	0
DDIT3	GTACGTGTCATGACACGT	15	28	0	0	0	0
DDIT3	CAACGTGTCATGACACCA	229	229	1	0	0	0
DDIT3	TGACGTGTCATGACACAC	51	26	0	0	0	0
DDIT4	CATGGTACTGCACAGTCA	698	980	0	1	1	5
DDIT4	GTACTGACGTGTGTTGCA	328	475	0	0	0	0
DDIT4	ACACTGACGTGTGTTGAC	59	53	0	0	0	0
DDIT4	CAACTGACGTGTGTTGTG	265	291	0	1	0	0
DDIT4	TGACTGACGTGTCAGTGT	314	375	0	1	0	0
DDO	TGACACGTTGGTACGTGT	265	71	0	0	0	0
DDO	GTACACGTTGGTACGTCA	99	173	1	0	0	0
DDO	ACACACGTTGGTACGTAC	29	26	0	0	0	0
DDO	CAACACGTTGGTACGTTG	482	587	6	0	0	0
DDO	GTACACGTTGGTACCAGT	345	634	0	40	0	0
DDOST	TGACCACATGCACATGAC	22	10	0	0	0	0
DDOST	ACACCACATGCACATGTG	80	136	0	0	0	0
DDOST	GTACCACATGCAACGTGT	639	745	0	0	0	5
DDOST	CAACCACATGCAACGTCA	364	156	1	0	0	3
DDOST	TGACCACATGCAACGTAC	264	82	0	0	0	0
DDR1	TGCATGCAACCATGTGGT	640	806	1	1	0	0
DDR1	GTCATGCAACCATGTGCA	41	168	0	1	0	0
DDR1	ACCATGCAACCATGTGAC	40	44	0	0	0	0
DDR1	CACATGCAACCATGTGTG	144	319	0	1	0	0
DDR1	GTTGGTCACAGTGTACTG	1066	773	4	1	0	0
DDX1	TGACACACGTGTACCAAC	34	49	0	0	0	0
DDX1	ACACACACGTGTACCATG	16	25	0	0	0	0
DDX1	CAACACACGTGTACACGT	37	58	0	0	0	0
DDX1	ACACACACGTGTACACCA	193	201	0	1	0	0
DDX1	GTACACACGTGTACACAC	1	7	0	0	0	0
DDX1	CATGCACACATGACCACA	288	236	0	0	0	0
DDX18	ACACACACGTCAGTGTTG	731	340	1	0	0	0
DDX18	CAACACACGTCAGTCAGT	15	27	0	0	0	0
DDX18	ACACACACGTCAGTCACA	41	60	7	0	0	0
DDX18	GTACACACGTCAGTCAAC	8	18	0	0	0	0
DDX18	TGACACACGTCAGTCATG	168	141	0	0	0	1
DDX19A	GTACACCATGTGGTTGCA	190	195	1	0	0	0
DDX19A	ACACACCATGTGGTTGAC	33	39	0	0	0	0
DDX19A	CAACACCATGTGGTTGTG	174	149	0	0	0	6
DDX19A	TGACACCATGTGCAGTGT	530	798	0	3	0	0
DDX19A	GTACACCATGTGCAGTCA	316	401	0	1	0	0
DDX20	ACACGTGTACTGGTACTG	1246	1187	2	0	1	0
DDX20	CAACGTGTACTGGTACCA	38	28	0	0	0	0
DDX20	TGACGTGTACTGGTACAC	28	31	1	0	0	0
DDX20	CAACGTGTACTGGTTGGT	139	230	0	0	0	1
DDX20	ACACGTGTACTGGTTGCA	107	100	0	0	0	0
DDX20	ACTGCAACGTGTTGGTTG	380	440	0	1	0	0
DDX23	CAACACACCAGTGTGTAC	46	68	0	0	0	0
DDX23	GTACACACCAGTGTGTTG	391	592	0	0	0	0
DDX23	TGACACACCAGTGTCAGT	164	188	1	1	0	1
DDX23	GTACACACCAGTGTCACA	27	15	0	0	0	0
DDX23	GTTGCAGTTGCAGTACGT	48	79	0	0	0	0
DDX4	CAACACACCACACACAGT	57	80	0	0	1	0
DDX4	TGACACACCACACAGTAC	20	35	0	0	0	0
DDX4	ACACACACCACACAGTTG	913	570	0	0	0	1
DDX4	ACACACACCACACACACA	214	128	0	1	0	1
DDX4	GTACACACCACACACAAC	7	10	0	0	0	0
DDX4	TGTGCACATGTGTGCAAC	18	18	1	0	0	0

DDX41	CACATGCATGACTGACTG	385	304	0	2	0	0
DDX41	GTCATGCATGACTGTGGT	74	107	0	0	0	0
DDX41	CACATGCATGACTGTGCA	108	133	0	0	0	0
DDX41	TGCATGCATGACTGTGAC	204	45	0	0	0	0
DDX41	ACCATGCATGACTGTGTG	492	553	1	0	0	0
DDX41	CATGCAACCATGCATGCA	91	122	0	0	0	0
DDX47	ACCATGTGACTGGTGTTG	153	713	0	0	0	0
DDX47	CACATGTGACTGGTCAGT	31	52	0	0	0	0
DDX47	ACCATGTGACTGGTCACA	78	53	0	0	0	0
DDX47	GTCATGTGACTGGTCAAC	148	35	0	0	0	0
DDX47	TGCATGTGACTGGTCATG	86	52	0	0	0	0
DDX50	ACACACACCAGTGTCAAC	36	62	0	0	0	0
DDX50	CAACACACCAGTGTCATG	138	191	1	1	0	1
DDX50	GTACACACCAGTGTACGT	503	93	0	0	0	0
DDX50	CAACACACCAGTGTACCA	170	174	1	0	0	0
DDX50	CATGGTGTCACACATGCA	264	76	1	0	0	0
DDX50	ACTGCACATGTGGTACTG	323	401	1	0	0	0
DDX51	CAACACCATGACGTACGT	424	785	2	1	2	0
DDX51	ACACACCATGACGTCATG	273	147	0	0	0	0
DDX51	ACACACCATGACGTACCA	181	73	0	0	0	0
DDX51	GTACACCATGACGTACAC	28	148	0	0	0	0
DDX51	GTTGCAGTCACATGGTTG	344	484	0	1	0	0
DDX52	GTACACACACACACCAGT	247	575	1	1	2	0
DDX52	CAACACACACACACCACA	141	66	0	0	0	1
DDX52	TGACACACACACACCAAC	16	12	0	0	0	0
DDX52	ACACACACACACACCATG	345	124	0	0	0	0
DDX52	CAACACACACACACACGT	36	48	0	1	0	0
DDX54	GTACACCATGCAACTGTG	50	63	4	0	1	4
DDX54	TGACACCATGCAACACTG	849	688	0	1	0	1
DDX54	ACACACCATGCAACTGGT	168	326	0	0	0	0
DDX54	TGACACCATGCAACTGCA	68	102	0	1	0	0
DDX54	CAACACCATGCAACTGAC	143	148	0	0	0	0
DDX55	TGACACACCACATGGTCA	616	488	0	2	3	0
DDX55	CAACACACCACAACTGAC	12	48	0	0	0	0
DDX55	GTACACACCACAACTGTG	406	355	0	0	0	0
DDX55	ACACACACCACATGGTGT	350	319	0	0	0	0
DDX55	ACTGCAGTTGCAGTACTG	118	165	0	0	0	0
DDX56	TGACACACACGTTGCACA	126	83	0	0	0	0
DDX56	CAACACACACGTTGCAAC	3	13	0	0	0	0
DDX56	GTACACACACGTTGCATG	112	96	0	0	0	0
DDX56	TGACACACACGTTGACGT	268	216	0	1	0	0
DDX56	GTTGGTGTCACACATGGT	497	243	2	0	0	0
DEDD	CAACGTCAACCACATGAC	562	167	0	0	0	0
DEDD	CATGGTACTGGTCAGTGT	966	720	0	1	0	2
DEDD	ACTGGTACTGGTCAGTCA	646	454	1	0	0	0
DEDD	GTTGGTACTGGTCAGTAC	138	280	0	0	0	0
DEDD	TGTGGTACTGGTCAGTTG	718	782	2	0	0	0
DEDD2	CATGCATGGTTGTGGTTG	579	918	0	855	3	0
DEDD2	GTTGGTTGGTTGTGTGCA	210	136	0	0	0	0
DEDD2	ACTGGTTGGTTGTGTGAC	257	20	0	0	0	0
DEDD2	CATGGTTGGTTGTGTGTG	440	479	2	0	0	0
DEDD2	ACTGGTTGCAGTGTGTGT	310	358	1	0	0	0
DEDD2	TGTGGTTGCAGTGTGTCA	66	62	17	1	0	1
DEFA1	CAACACTGACGTTGACCA	287	304	2	0	0	10
DEFA1	TGACACTGACGTTGACAC	80	69	0	0	0	0
DEFA1	ACACACTGACGTTGACTG	205	477	0	0	0	0
DEFA1	CAACACTGACGTTGTGGT	1315	772	1	0	0	3
DEFA1	GTACACTGACACACGTGT	187	215	0	1	0	0
DEFA1	TGTGCACATGACTGCAGT	303	284	0	0	0	14
DEFB1	CAACACTGACACACGTCA	595	431	1	0	0	5
DEFB1	TGACACTGACACACGTAC	26	53	0	0	0	0
DEFB1	ACACACTGACACACGTTG	708	771	2	0	0	0
DEFB1	CAACACTGACACACCAGT	183	266	1	0	0	0
DEFB1	ACACACTGACACACCACA	127	110	0	0	0	0
DEFB4	TGACACTGCATGTGTGTG	507	152	0	0	0	0
DEFB4	CAACACTGACGTGTGTGT	1438	787	2	1	0	1
DEFB4	ACACACTGACGTGTGTCA	119	151	0	0	0	0
DEFB4	GTACACTGACGTGTGTAC	9	19	0	0	0	0
DEFB4	ACTGCAGTTGCATGCATG	86	149	0	1	0	0
DEFB4	CATGCACATGTGGTGTAC	9	14	0	0	0	0
DEGS1	ACACTGACACACTGGTCA	397	423	1	0	0	0
DEGS1	GTACTGACACACTGGTAC	42	21	0	0	0	0
DEGS1	TGACTGACACACTGGTTG	352	356	0	0	0	0
DEGS1	ACACTGACACACTGCAGT	143	202	0	0	0	0
DEGS1	TGACTGACACACTGCACA	39	32	0	0	0	0

DEGS1	TGTGCAACACCACATGGT	737	531	3	1	0	0
DEGS2	CAACTGACACACACGTAC	72	93	0	0	0	0
DEGS2	GTACTGACACACACGTTG	127	131	0	0	0	0
DEGS2	TGACTGACACACACCAGT	116	111	0	1	0	0
DEGS2	GTACTGACACACACCACA	137	72	0	0	0	0
DEGS2	ACACTGACACACACCAAC	74	71	0	1	0	0
DERA	ACACACACTGCAGTGTGT	129	135	0	1	0	0
DERA	TGACACACTGCAGTGTCA	325	320	0	1	0	0
DERA	CAACACACTGCAGTGTAC	95	212	0	1	0	0
DERA	GTACACACTGCAGTGTTG	388	320	0	1	0	0
DERA	TGACACACTGCAGTCAGT	152	155	0	0	0	0
DES	ACACTGTGTGCAACCACA	22	23	1	0	0	0
DES	GTACTGTGTGCAACCAAC	11	3	0	0	0	0
DES	TGACTGTGTGCAACCATG	61	29	0	0	0	0
DES	TGTGGTGTCAGTGTGTGT	1145	888	3	0	0	1
DES	TGTGACGTACTGACTGCA	22	64	0	0	0	0
DFFA	TGTGGTTGGTACACTGGT	315	630	1	1	1	0
DFFA	CACATGACTGTGCAGTGT	224	229	0	0	0	0
DFFA	ACCATGACTGTGCAGTCA	134	93	1	1	0	6
DFFA	GTCATGACTGTGCAGTAC	75	122	0	0	0	0
DFFA	TGCATGACTGTGCAGTTG	540	537	1	0	0	0
DFFB	ACTGCAGTACGTTGTGTG	107	49	1	0	1	0
DFFB	ACCATGACTGTGCACAGT	187	373	30	0	0	0
DFFB	TGCATGACTGTGCACACA	29	42	0	0	0	0
DFFB	CACATGACTGTGCACAAC	38	45	0	0	0	0
DFFB	GTCATGACTGTGCACATG	263	91	0	0	0	0
DGAT1	ACACCAACCACAGTGTGT	1740	1106	8	1	3	3
DGAT1	GTACCAACCACAGTGTTG	668	1174	0	1	1	3
DGAT1	TGACCAACCACAGTGTCA	599	747	3	0	0	1
DGAT1	CAACCAACCACAGTGTAC	134	156	0	2	0	0
DGAT1	TGTGGTTGTGCAACGTAC	12	34	0	0	0	0
DGAT1	ACTGCACAACACTGCATG	866	766	0	0	0	0
DGAT2	CATGCAACTGTGGTGTTG	472	719	1	1	2	0
DGAT2	TGCATGTGACGTTGGTGT	961	508	0	1	1	0
DGAT2	GTCATGTGACGTACTGCA	76	90	0	0	0	0
DGAT2	ACCATGTGACGTACTGAC	64	95	0	0	0	0
DGAT2	CACATGTGACGTACTGTG	72	92	0	0	0	0
DGAT2	GTCATGTGACGTTGGTCA	47	100	0	0	0	0
DGKA	GTCATGTGTGTGGTACAC	92	24	0	0	0	0
DGKA	TGCATGTGTGTGGTACTG	11	47	0	0	0	0
DGKA	ACCATGTGTGTGGTTGGT	96	123	0	0	0	0
DGKA	TGCATGTGTGTGGTTGCA	117	95	0	0	0	0
DGKA	TGTGGTTGACACACTGCA	305	373	0	0	0	0
DGKB	TGCATGCAACTGCACACA	57	114	0	0	0	0
DGKB	CACATGCAACTGCACAAC	168	68	0	1	0	0
DGKB	GTCATGCAACTGCACATG	141	444	0	0	0	0
DGKB	TGCATGCAACTGCAACGT	228	248	0	1	0	0
DGKB	GTCATGCAACTGCAACCA	699	476	1	1	0	4
DGKB	GTTGCAACACACGTACAC	53	30	0	0	0	0
DGKD	CACATGCAACACCATGTG	864	658	1	1	1	0
DGKD	TGCATGCAACACCATGGT	309	228	0	0	0	0
DGKD	GTCATGCAACACCATGCA	151	247	0	0	0	1
DGKD	ACCATGCAACACCATGAC	21	19	0	0	0	0
DGKD	TGCATGCAACACACGTGT	237	331	0	0	0	1
DGKD	ACTGCAACTGACACACGT	121	152	0	0	0	0
DGKE	CACATGTGTGTGGTTGAC	24	25	0	0	0	6
DGKE	GTCATGTGTGTGGTTGTG	236	327	1	1	0	2
DGKE	ACCATGTGTGTGCAGTGT	119	393	0	0	0	0
DGKE	TGCATGTGTGTGCAGTCA	265	164	0	0	0	0
DGKE	CACATGTGTGTGCAGTAC	259	240	0	0	0	0
DGKE	CATGCACAGTACCACATG	203	388	0	0	0	0
DGKG	TGCATGCAACGTACCAGT	325	206	0	1	0	0
DGKG	GTCATGCAACGTACCACA	86	89	0	0	0	0
DGKG	ACCATGCAACGTACCAAC	42	29	0	0	0	0
DGKG	CACATGCAACGTACCATG	129	187	0	0	0	0
DGKG	GTCATGCAACGTACACGT	99	67	4	0	0	0
DGKH	CACATGCATGTGGTTGCA	64	113	0	0	0	2
DGKH	TGCATGCATGTGGTTGAC	51	49	0	0	0	0
DGKH	ACCATGCATGTGGTTGTG	346	322	0	0	0	5
DGKH	GTCATGCATGTGCAGTGT	161	453	0	0	0	0
DGKH	CACATGCATGTGCAGTCA	135	160	0	0	0	0
DGKH	CATGCATGCACACATGAC	11	7	0	0	0	0
DGKI	GTCATGTGTGTGCAGTTG	411	398	1	1	0	1
DGKI	TGCATGTGTGTGCACAGT	138	80	0	0	0	0
DGKI	GTCATGTGTGTGCACACA	32	45	0	1	0	0

DGKI	ACCATGTGTGTGCACAAC	9	28	0	0	0	0
DGKI	ACACGTTGTGACACTGTG	576	496	2	0	0	91
DGKI	CATGCATGCACATGACTG	259	317	0	0	0	4
DGKQ	CACATGTGTGTGCACATG	154	110	0	0	0	0
DGKQ	GTCATGTGTGTGCAACGT	81	54	0	0	0	0
DGKQ	CACATGTGTGTGCAACCA	420	372	0	0	0	1
DGKQ	TGCATGTGTGTGCAACAC	64	43	0	0	0	0
DGKQ	GTTGGTTGGTGTCACAAC	77	9	0	0	0	0
DGKZ	GTCATGCAACACCAACTG	1378	1211	469	33506	1204968	874
DGKZ	CACATGCAACACCAACAC	1	3	0	0	0	0
DGKZ	TGTGGTACTGTGGTCATG	124	164	0	0	0	0
DGKZ	ACTGGTACTGTGGTACGT	111	155	0	0	0	0
DGKZ	TGTGGTACTGTGGTACCA	33	37	0	0	0	0
DHCR24	GTACACGTTGTGGTCAGT	446	211	0	0	0	0
DHCR24	CAACACGTTGTGGTCACA	386	222	0	0	0	1
DHCR24	TGACACGTTGTGGTCAAC	23	29	0	0	0	0
DHCR24	ACACACGTTGTGGTCATG	146	171	1	0	0	0
DHCR24	CAACACGTTGTGGTACGT	383	252	0	0	0	0
DHCR7	GTACCATGACCAACGTCA	325	347	2	0	0	0
DHCR7	ACACACGTTGTGACCAGT	173	376	0	0	0	6
DHCR7	TGACACGTTGTGACCACA	45	55	0	0	0	0
DHCR7	CAACACGTTGTGACCAAC	10	18	0	0	0	0
DHCR7	GTACACGTTGTGACCATG	74	326	0	0	0	0
DHCR7	GTTGCACAGTACCAACGT	289	281	1	1	0	0
DHDDS	GTACACGTCAACTGGTTG	807	183	1	1	0	4
DHDDS	TGACACGTCAACTGCAGT	55	29	0	0	0	0
DHDDS	GTACACGTCAACTGCACA	83	110	0	0	0	0
DHDDS	ACACACGTCAACTGCAAC	33	45	1	0	0	0
DHDDS	GTACTGTGCACAACGTCA	279	138	0	0	0	0
DHFR	CAACCATGGTACTGGTGT	1293	1410	2	4	2	0
DHFR	TGACCATGGTACACTGTG	285	461	1	0	0	0
DHFR	ACACCATGGTACTGGTCA	538	573	1	1	0	0
DHFR	GTACCATGGTACTGGTAC	41	169	0	0	0	0
DHFR	TGACCATGGTACTGGTTG	446	542	3	2	0	0
DHH	GTACCAGTTGTGGTTGCA	147	197	0	0	0	0
DHH	GTACCACAGTGTACCAGT	213	200	0	1	0	0
DHH	CAACCACAGTGTACCACA	167	194	0	1	0	0
DHH	TGACCACAGTGTACCAAC	18	32	0	0	0	0
DHH	ACACCACAGTGTACCATG	135	101	0	0	0	0
DHH	ACTGCAACTGGTCATGAC	379	405	1	0	0	0
DHODH	TGACCAGTGTACACACTG	838	756	0	0	1	0
DHODH	ACACCAGTGTACACTGGT	107	63	0	0	0	0
DHODH	TGACCAGTGTACACTGCA	135	205	0	0	0	0
DHODH	CAACCAGTGTACACTGAC	123	139	0	0	0	3
DHODH	GTACCAGTGTACACTGTG	133	161	0	0	0	0
DHRS1	ACACCAGTGTACACCATG	215	260	1	0	0	0
DHRS1	CAACCAGTGTACACACGT	109	69	0	0	0	0
DHRS1	ACACCAGTGTACACACCA	599	414	0	1	0	0
DHRS1	GTACCAGTGTACACACAC	42	53	0	1	0	0
DHRS1	CAACCAGTGTACTGCATG	200	398	0	0	0	0
DHRS1	CATGCAACCACATGGTCA	748	528	2	0	0	0
DHRS2	TGACCAGTCATGGTTGCA	475	237	0	1	0	0
DHRS2	CAACCAGTCATGGTTGAC	32	42	0	0	0	0
DHRS2	GTACCAGTCATGGTTGTG	321	302	0	0	0	0
DHRS2	ACACCAGTCATGCAGTGT	564	457	1	1	0	0
DHRS2	TGACCAGTCATGCAGTCA	254	145	0	0	0	0
DHRS3	GTACCAACACGTCATGCA	202	144	0	0	0	0
DHRS3	ACACCAACACGTCATGAC	344	145	0	0	0	0
DHRS3	CAACCAACACGTCATGTG	301	386	1	0	0	0
DHRS3	TGACCAACACGTACGTGT	548	492	0	1	0	0
DHRS3	GTACCAACACGTACGTCA	295	60	0	0	0	5
DHRS4	TGACCAGTGTGTACCAGT	261	281	0	0	0	0
DHRS4	GTACCAGTGTGTACCACA	145	269	0	0	0	1
DHRS4	GTACCAGTCAACCAGTAC	22	28	0	0	0	0
DHRS4	TGACCAGTCAACCAGTTG	761	651	1	2	0	6
DHRS4	ACACCAGTCAACCACAGT	96	208	0	0	0	0
DHRS4	ACTGCATGCACATGCAGT	110	49	0	0	0	0
DHRS7	GTACCAGTGTTGTGACAC	29	44	0	0	0	0
DHRS7	TGACCAGTGTTGTGACTG	399	791	0	1	0	0
DHRS7	ACACCAGTGTTGTGTGGT	60	57	0	0	0	0
DHRS7	TGACCAGTGTTGTGTGCA	238	267	1	0	0	0
DHRS7	CAACCAGTGTTGTGTGAC	61	196	0	0	0	0
DHRS7	TGTGCACAACTGACTGAC	115	121	0	0	0	0
DHRSX	GTACCAGTGTGTTGACCA	150	267	2	0	0	0
DHRSX	ACACCAGTGTGTTGACAC	22	19	0	0	0	0

DHRSX	CAACCAGTGTGTTGACTG	701	825	1	1	0	1
DHRSX	GTACCAGTGTGTTGTGGT	474	401	0	0	0	0
DHRSX	CAACCAGTGTGTTGTGCA	303	202	0	0	0	0
DHRSX	CATGCAACACCACAACAC	168	196	0	0	0	0
DHX58	CAACACACCAACGTGTTG	709	412	0	0	0	0
DHX58	GTACACACCAACGTCAGT	273	241	0	0	0	0
DHX58	CAACACACCAACGTCACA	357	229	0	0	0	0
DHX58	TGACACACCAACGTCAAC	136	76	1	0	0	20
DHX58	ACACACACCAACGTCATG	453	253	85	0	0	1
DIABLO	GTTGACGTGTCACACACA	101	247	1	0	4	0
DIABLO	TGCATGTGCAGTTGTGAC	20	13	0	0	0	0
DIABLO	ACCATGTGCAGTTGTGTG	586	403	4	0	0	0
DIABLO	TGCATGTGCACAGTGTGT	341	410	0	0	0	2
DIABLO	ACTGACGTGTCACACAAC	144	57	0	0	0	0
DIAPH1	GTTGGTCAGTCAACTGCA	108	99	0	0	0	0
DIAPH1	ACTGGTCAGTCAACTGAC	16	165	0	0	0	1
DIAPH1	CATGGTCAGTCAACTGTG	580	906	1	2	0	0
DIAPH1	TGTGGTCAGTCATGGTGT	739	824	0	0	0	1
DIAPH1	GTTGGTCAGTCATGGTCA	738	856	1	0	0	0
DIAPH1	TGTGCATGCAGTTGACAC	69	57	9	0	0	0
DIAPH2	ACTGGTGTGTACGTGTAC	37	43	0	0	0	0
DIAPH2	CATGGTGTGTACGTGTTG	666	880	2	0	0	0
DIAPH2	GTTGGTGTGTACGTCAGT	267	82	0	0	0	0
DIAPH2	GTTGCAGTCAGTTGTGTG	296	215	0	0	0	0
DIAPH2	CATGCAGTCACAGTGTGT	1315	1188	3	1	0	9
DICER1	TGTGCAGTCAGTTGGTGT	648	708	2	1	3	2
DICER1	TGACACACCAACGTGTGT	616	913	1	0	0	0
DICER1	GTACACACCAACGTGTCA	239	283	3	0	0	3
DICER1	ACACACACCAACGTGTAC	39	42	0	0	0	0
DICER1	GTTGCAGTCAGTTGGTCA	247	221	1	13	0	0
DICER1	GTTGCATGGTACCAGTGT	150	119	0	0	0	0
DIDO1	ACACGTACCAGTGTTGAC	9	11	0	0	0	0
DIDO1	CAACGTACCAGTGTTGTG	748	108	0	0	0	0
DIDO1	TGACGTACCAGTCAGTGT	934	508	0	0	0	0
DIDO1	GTACGTACCAGTCAGTCA	83	100	0	0	0	0
DIDO1	CATGCAGTCAACTGACAC	1	3	0	0	0	0
DIO1	CATGGTACACGTACGTGT	175	854	0	0	1	0
DIO1	GTTGGTACACGTCATGAC	67	87	0	0	0	0
DIO1	TGTGGTACACGTCATGTG	417	352	0	1	0	9
DIO1	ACTGGTACACGTACGTCA	200	179	0	0	0	0
DIO1	TGTGGTTGCAACGTGTGT	691	655	2	2	0	0
DIO2	GTTGGTGTGTACTGCAAC	70	76	0	1	0	0
DIO2	TGTGGTGTGTACTGCATG	278	406	1	1	0	0
DIO2	ACTGGTGTGTACTGACGT	67	69	0	0	0	0
DIO2	TGTGGTGTGTACTGACCA	225	187	0	0	0	0
DIO2	CATGGTGTGTACTGACAC	52	48	0	0	0	0
DISC1	TGTGACGTTGTGTGACGT	290	839	17	7	10	15
DISC1	ACTGACGTTGTGTGCAGT	34	36	0	0	0	0
DISC1	TGTGACGTTGTGTGCACA	9	19	0	0	0	0
DISC1	CATGACGTTGTGTGCAAC	23	46	0	0	0	0
DISC1	GTTGACGTTGTGTGCATG	99	169	0	0	0	0
DIXDC1	CATGACACCAGTTGCAGT	404	501	2	7	1	1
DIXDC1	ACTGGTCACAACTGACTG	418	539	1	0	0	0
DIXDC1	CATGGTCACAACTGTGGT	123	118	0	0	0	0
DIXDC1	ACTGGTCACAACTGTGCA	325	273	0	0	0	1
DIXDC1	GTTGGTCACAACTGTGAC	3	4	0	0	0	0
DKC1	GTACCATGCAGTCATGTG	332	425	0	1	0	0
DKC1	ACACCATGCAGTACGTGT	946	1326	1	0	0	0
DKC1	TGACCATGCAGTACGTCA	70	77	0	0	0	0
DKC1	CAACCATGCAGTACGTAC	93	62	0	0	0	0
DKC1	GTACCATGCAGTACGTTG	214	231	0	0	0	0
DKC1	ACTGCAACGTCACACAGT	72	75	0	0	0	3
DKFZP434E1119	ACTGACCAACTGTGTGGT	588	667	9	0	3	1
DKFZP434E1119	CATGACCAACTGTGACGT	259	282	3	1062	1	0
DKFZP434E1119	ACTGACCAACTGTGACCA	95	271	0	1	0	0
DKFZP434E1119	GTTGACCAACTGTGACAC	159	59	0	0	0	0
DKFZP434E1119	TGTGACCAACTGTGACTG	188	318	0	0	0	0
DKK1	ACACCACAGTCACAACCA	161	134	0	0	0	0
DKK1	GTACCACAGTCACAACAC	118	155	0	0	0	0
DKK1	TGACCACAGTCACAACTG	199	332	1	0	0	0
DKK1	ACACCACAGTCACATGGT	1032	1237	1	1	0	6
DKK1	TGACCACAGTCACATGCA	166	145	1	0	0	0
DKK2	ACACCACAGTCAACGTGT	428	528	0	0	1	0
DKK2	CAACCACAGTCACATGAC	28	36	0	0	0	0
DKK2	GTACCACAGTCACATGTG	159	93	0	0	0	0

DKK2	TGACCACAGTCAACGTCA	110	200	0	0	0	0
DKK2	CAACCACAGTCAACGTAC	278	42	0	0	0	0
DKK3	GTACCACAGTCAACGTTG	507	260	0	0	0	1
DKK3	TGACCACAGTCAACCAGT	60	77	0	0	0	0
DKK3	GTACCACAGTCAACCACA	93	111	1	0	0	0
DKK3	ACACCACAGTCAACCAAC	12	23	0	0	0	0
DKK3	CAACCACAGTCAACCATG	352	541	0	0	0	1
DKK4	CATGGTGTCAACGTACAC	31	25	0	0	0	0
DKK4	GTTGGTGTCAACGTACTG	203	294	0	0	0	0
DKK4	TGTGGTGTCAACGTTGGT	334	403	0	0	0	0
DKK4	GTTGGTGTCAACGTTGCA	50	24	0	0	0	0
DKK4	ACTGGTGTCAACGTTGAC	18	26	0	0	0	0
DLAT	ACACCAACGTACTGGTTG	292	725	1	1	2	0
DLAT	ACACCAACGTACACTGTG	561	535	0	0	0	0
DLAT	GTACCAACGTACTGGTGT	98	136	0	0	0	0
DLAT	CAACCAACGTACTGGTCA	381	686	0	0	0	0
DLAT	TGACCAACGTACTGGTAC	12	51	0	0	0	0
DLAT	CATGCATGCAGTTGCATG	231	193	0	0	0	0
DLD	CAACCAGTGTACACGTTG	1232	521	0	2	0	0
DLD	GTACCAGTGTACACCAGT	83	77	0	0	0	0
DLD	CAACCAGTGTACACCACA	169	97	0	0	0	0
DLD	TGACCAGTGTACACCAAC	28	118	0	0	0	0
DLD	ACACCAGTGTACTGCAAC	18	19	0	0	0	0
DLG4	CACATGTGTGTGACGTGT	70	203	1	0	0	0
DLG4	ACCATGTGTGTGACGTCA	101	126	0	0	0	0
DLG4	GTCATGTGTGTGACGTAC	6	12	0	0	0	0
DLG4	TGCATGTGTGTGACGTTG	581	294	0	0	0	0
DLG4	ACCATGTGTGTGACCAGT	204	273	0	1	0	0
DLG7	ACACTGCATGTGCACAAC	136	63	0	0	2	1
DLG7	TGACTGCATGTGCACAGT	278	222	0	0	0	0
DLG7	GTACTGCATGTGCACACA	256	235	1	0	0	0
DLG7	CAACTGCATGTGCACATG	181	183	0	0	0	0
DLG7	GTACTGCATGTGCAACGT	705	652	2	0	0	0
DLG7	ACTGCAACACGTCATGTG	235	248	1	0	0	0
DLL1	GTACCACAGTCAGTACTG	424	478	0	1	0	190
DLL1	TGACCACAGTCAGTTGGT	121	151	0	0	0	0
DLL1	GTACCACAGTCAGTTGCA	195	181	0	0	0	1
DLL1	ACACCACAGTCAGTTGAC	66	50	0	0	0	0
DLL1	TGTGCAGTACTGCAACGT	107	343	0	0	0	1
DLL3	GTACCACAGTCATGGTAC	60	231	0	0	0	0
DLL3	TGACCACAGTCATGGTTG	668	641	0	1	0	0
DLL3	ACACCACAGTCATGCAGT	253	282	0	0	0	0
DLL3	TGACCACAGTCATGCACA	358	762	1	2	0	0
DLL3	CAACCACAGTCATGCAAC	10	5	0	0	0	0
DLL4	GTACCACAGTCATGCATG	83	64	0	0	0	0
DLL4	TGACCACAGTCATGACGT	63	83	0	0	0	0
DLL4	GTACCACAGTCATGACCA	154	172	1	0	0	0
DLL4	ACACCACAGTCATGACAC	90	122	0	1	0	0
DLL4	CAACCACAGTCATGACTG	447	494	2	1	0	0
DLL4	ACTGCATGCACAGTGTTG	86	119	0	0	0	0
DLST	ACACCACATGACGTCACA	105	102	0	0	0	3
DLST	GTACCACATGACGTCAAC	19	20	0	0	0	0
DLST	TGACCACATGACGTCATG	120	75	0	0	0	0
DLST	ACACCACATGACGTACGT	270	353	0	0	0	0
DLST	ACTGCAGTACTGCACAGT	302	341	0	0	0	0
DLST	GTTGCATGCACACATGTG	134	169	0	0	0	0
DLX1	TGTGCAGTACACACGTGT	627	702	7	1733	23	2
DLX1	GTACGTCATGCAGTACAC	79	69	1	0	0	0
DLX1	TGACGTCATGCAGTACTG	768	520	1	3	0	0
DLX1	ACACGTCATGCAGTTGGT	1021	831	2	0	0	3
DLX1	GTACTGACTGCATGTGAC	123	120	0	1	0	1
DLX3	TGACGTCATGCAGTTGCA	45	104	0	0	0	0
DLX3	CAACGTCATGCAGTTGAC	238	127	0	1	0	0
DLX3	GTACGTCATGCAGTTGTG	498	537	2	2	0	0
DLX3	ACACGTCATGCACAGTGT	208	382	0	0	0	0
DLX3	ACTGCATGTGGTGTCAAC	6	15	0	0	0	0
DLX6	TGACGTACACACACCACA	177	147	0	0	0	0
DLX6	CAACGTACACACACCAAC	5	17	0	0	0	0
DLX6	GTACGTACACACACCATG	114	333	1	0	0	0
DLX6	TGACGTACACACACACGT	12	7	0	0	0	0
DLX6	GTACGTACACACACACCA	459	234	0	1	0	0
DMC1	GTTGGTACCAACACGTGT	776	814	1	0	1	0
DMC1	TGTGGTACCAACCATGAC	57	62	0	0	0	0
DMC1	ACTGGTACCAACCATGTG	273	418	0	1	0	1
DMC1	CATGGTACCAACACGTCA	76	65	0	0	0	0

DMC1	TGTGGTACCAACACGTAC	15	87	0	0	0	0
DMC1	TGTGCAACTGGTCAGTAC	22	34	0	0	0	0
DMD	TGACACTGGTGTACGTAC	53	61	0	0	0	0
DMD	ACACACTGGTGTACGTTG	331	417	0	0	0	0
DMD	CAACACTGGTGTACCAGT	63	142	0	0	0	0
DMD	ACACACTGGTGTACCACA	128	237	0	0	0	0
DMD	GTACACTGGTGTACCAAC	6	9	0	0	0	0
DMD	GTTGCACAACGTCAACTG	711	385	1	0	0	0
DMGDH	CATGCAACTGACCATGCA	395	434	0	0	1	1
DMGDH	ACACCAGTCAACTGACCA	206	484	1	2	0	0
DMGDH	GTACCAGTCAACTGACAC	193	78	0	222	0	26
DMGDH	TGACCAGTCAACTGACTG	291	220	1	0	0	0
DMGDH	ACACCAGTCAACTGTGGT	653	187	1	0	0	0
DMGDH	TGACCAGTCAACTGTGCA	159	177	0	0	0	0
DNAJA1	TGTGGTGTACGTTGACAC	43	55	0	0	0	0
DNAJA1	ACTGGTACCAACTGCATG	87	119	0	0	0	0
DNAJA1	CATGGTACCAACTGACGT	62	64	0	0	0	0
DNAJA1	ACTGGTACCAACTGACCA	196	178	0	0	0	0
DNAJA1	GTTGGTACCAACTGACAC	61	62	0	0	0	0
DNAJA2	CAACGTCACAGTGTACGT	30	40	8	10	4	6
DNAJA2	ACACGTCACAGTGTACCA	133	267	0	0	0	0
DNAJA2	GTACGTCACAGTGTACAC	61	55	0	0	0	0
DNAJA2	TGACGTCACAGTGTACTG	297	448	0	1	0	0
DNAJA2	ACACGTCACAGTGTTGGT	79	160	0	0	0	18
DNAJA2	GTTGCACAACACTGACAC	122	55	0	0	0	0
DNAJA3	ACACGTCACAGTGTGTAC	50	62	0	0	0	2
DNAJA3	CAACGTCACAGTGTGTTG	264	339	0	0	0	0
DNAJA3	GTACGTCACAGTGTCAGT	1063	407	1	1	0	0
DNAJA3	CATGCAGTCATGCACACA	307	266	0	0	0	0
DNAJA3	TGTGCAGTCATGCACAAC	23	13	0	0	0	0
DNAJA4	GTACGTTGTGCAACGTCA	95	36	0	0	0	0
DNAJA4	ACACGTTGTGCAACGTAC	51	30	0	0	0	1
DNAJA4	CAACGTTGTGCAACGTTG	73	354	0	0	0	0
DNAJA4	GTACGTTGTGCAACCAGT	80	122	0	0	0	0
DNAJA4	CAACGTTGTGCAACCACA	111	172	0	0	0	0
DNAJB1	TGACGTCACAGTGTTGCA	65	554	0	0	0	0
DNAJB1	CAACGTCACAGTGTTGAC	67	57	0	0	0	0
DNAJB1	GTACGTCACAGTGTTGTG	582	641	0	0	0	0
DNAJB1	ACACGTCACAGTCAGTGT	891	789	1	0	0	4
DNAJB1	TGACGTCACAGTCAGTCA	564	413	1	1	0	11
DNAJB1	TGTGCACAACGTTGCAGT	115	186	1	0	0	0
DNAJB11	CAACGTCAACCAGTTGTG	610	343	0	0	1	1
DNAJB11	GTACGTTGTGCACATGCA	632	174	2	0	0	0
DNAJB11	ACACGTTGTGCACATGAC	116	88	0	0	0	0
DNAJB11	CAACGTTGTGCACATGTG	290	353	0	0	0	0
DNAJB11	TGACGTTGTGCAACGTGT	399	599	1130	0	0	0
DNAJB2	ACACGTCAACCAGTTGAC	51	52	0	0	0	0
DNAJB2	ACACGTTGTGCAGTTGTG	177	210	0	0	0	0
DNAJB2	GTACGTTGTGCACAGTGT	83	60	1	0	0	0
DNAJB2	CAACGTTGTGCACAGTCA	58	68	0	0	0	0
DNAJB2	TGACGTTGTGCACAGTAC	62	89	0	0	0	0
DNAJB4	ACACGTTGTGCACAGTTG	1413	1394	3	17	1006	10
DNAJB4	CAACGTTGTGCACACAGT	136	204	0	0	0	0
DNAJB4	ACACGTTGTGCACACACA	437	239	0	1	0	0
DNAJB4	GTACGTTGTGCACACAAC	55	11	0	0	0	0
DNAJB4	TGACGTTGTGCACACATG	10	29	0	0	0	0
DNAJB4	CATGCAACTGCAACCATG	23	68	0	0	0	0
DNAJB9	ACACGTTGTGCACAACGT	59	102	0	0	0	0
DNAJB9	TGACGTTGTGCACAACCA	30	33	0	0	0	0
DNAJB9	CAACGTTGTGCACAACAC	14	13	0	0	0	0
DNAJB9	GTACGTTGTGCACAACTG	343	391	1	0	0	2
DNAJB9	TGACGTTGTGCACATGGT	96	121	0	0	0	0
DNAJB9	ACTGCACATGGTTGCAGT	32	36	0	0	0	0
DNAJC3	GTTGGTACGTCAACACCA	201	170	1	0	1	0
DNAJC3	TGTGGTACGTCAACACGT	42	36	1	1	0	0
DNAJC3	ACTGGTACGTCAACACAC	31	58	0	0	0	0
DNAJC3	CATGGTACGTCAACACTG	364	360	0	0	0	0
DNAJC3	GTTGGTACGTCAACTGGT	293	169	0	0	0	0
DNAJC3	TGTGCAACTGCAGTGTAC	108	73	0	0	0	0
DNAJC4	CATGGTTGTGCACAACTG	920	559	0	1	1	3
DNAJC4	CAACGTCACAGTGTCACA	99	106	0	2	0	6
DNAJC4	TGACGTCACAGTGTCAAC	13	9	0	0	0	0
DNAJC4	ACACGTCACAGTGTCATG	36	26	0	0	0	0
DNAJC4	ACTGGTTGTGCACAACAC	14	13	0	0	0	0
DNAJC4	GTTGCACAACGTGTACCA	363	181	1	0	0	0

DNAJC5	TGTGGTACCACATGGTTG	904	634	2	0	1	2
DNAJC5	ACTGGTACCACATGCAGT	503	378	0	3	0	0
DNAJC5	TGTGGTACCACATGCACA	62	133	0	1	0	0
DNAJC5	CATGGTACCACATGCAAC	10	19	0	0	0	0
DNAJC5	GTTGGTACCACATGCATG	216	269	0	0	0	0
DNM1	GTACACACACCAGTGTCA	677	415	2	0	1	0
DNM1	ACACACACACCAGTGTAC	12	17	0	0	0	0
DNM1	CAACACACACCAGTGTTG	523	627	1	0	0	0
DNM1	GTACACACACCAGTCAGT	415	463	0	0	0	0
DNM1	CAACACACACCAGTCACA	4	14	0	0	0	0
DNM2	TGACGTGTGTTGACACGT	57	41	0	0	0	0
DNM2	GTACGTGTGTTGACACCA	161	344	1	1	0	0
DNM2	ACACGTGTGTTGACACAC	17	10	0	0	0	0
DNM2	CAACGTGTGTTGACACTG	485	409	4	1	0	0
DNM2	GTACGTGTGTTGACTGGT	430	199	2	0	0	0
DNMBP	TGTGGTCAACCATGGTCA	427	532	0	0	1	1
DNMBP	CATGGTCAACCATGGTAC	44	33	0	0	0	0
DNMBP	GTTGGTCAACCATGGTTG	718	710	0	0	0	0
DNMBP	TGTGGTCAACCATGCAGT	216	275	0	0	0	0
DNMBP	GTTGGTCAACCATGCACA	26	55	0	0	0	0
DNMT1	ACACGTTGACTGACTGGT	957	887	2	15	3	0
DNMT1	TGACGTTGACTGACACTG	238	540	0	1	1	0
DNMT1	ACACGTTGACTGACACCA	112	118	0	0	0	0
DNMT1	GTACGTTGACTGACACAC	95	86	0	0	0	0
DNMT1	TGACGTTGACTGACTGCA	151	147	0	0	0	0
DNMT1	CATGCACAGTACCAACCA	761	1068	1	2	0	0
DNMT3A	ACACCAACGTCAGTTGTG	439	169	0	0	0	0
DNMT3A	CATGCATGGTCACAACCA	276	430	2	1	0	0
DNMT3A	TGTGCATGGTCACAACAC	146	177	1	0	0	3
DNMT3A	ACTGCATGGTCACAACTG	220	142	1	0	0	0
DNMT3A	CATGCATGGTCACATGGT	524	479	0	0	0	1
DNMT3A	ACTGCATGGTCACATGCA	14	20	0	0	0	0
DNMT3B	CAACCAACGTGTTGGTTG	261	283	1	1	0	0
DNMT3B	GTACCAACGTGTTGCAGT	65	124	1	0	0	0
DNMT3B	CAACCAACGTGTTGCACA	26	67	1	0	0	0
DNMT3B	TGACCAACGTGTTGCAAC	58	21	0	0	0	0
DNMT3B	ACTGCAGTACTGCAACAC	0	5	0	0	0	0
DNMT3B	GTTGCATGGTCACAACGT	328	386	0	0	0	0
DNMT3L	ACACGTTGGTGTTGTGTG	203	165	0	0	0	0
DNMT3L	TGACGTTGGTCAGTGTGT	1303	433	0	0	0	38
DNMT3L	GTACGTTGGTCAGTGTCA	366	465	0	0	0	1
DNMT3L	TGTGCAGTACACTGACAC	81	210	0	0	0	0
DNMT3L	ACTGCATGGTCAACTGTG	131	212	0	0	0	0
DNMT3L	GTTGCATGGTCATGGTGT	526	173	1	0	0	0
DNTT	CAACCAACGTCATGGTAC	68	65	0	0	0	0
DNTT	GTACCAACGTCATGGTTG	401	440	1	0	0	7
DNTT	TGACCAACGTCATGCAGT	54	333	1	0	0	0
DNTT	GTACCAACGTCATGCACA	40	50	0	0	0	0
DNTT	ACACCAACGTCATGCAAC	5	8	0	0	0	0
DOCK1	TGACCAGTACGTCAGTCA	980	1278	18068	24	3	10
DOCK1	TGACCAGTACGTGTTGCA	141	82	0	0	0	0
DOCK1	CAACCAGTACGTGTTGAC	20	22	0	0	0	0
DOCK1	GTACCAGTACGTGTTGTG	64	93	0	0	0	0
DOCK1	ACACCAGTACGTCAGTGT	420	422	3	0	0	0
DOCK9	ACTGGTCAACTGGTCACA	260	323	296	24400	588646	412
DOCK9	ACTGGTCAACTGGTGTTG	631	626	694	5	77	0
DOCK9	GTTGGTCAACTGGTCAAC	22	41	0	0	1	0
DOCK9	GTTGCAACTGCAGTCAAC	36	33	0	0	1	0
DOCK9	CATGGTCAACTGGTCAGT	180	246	0	0	0	1
DOCK9	TGTGGTCAACTGGTCATG	204	219	0	0	0	0
DOK1	TGACCAACACTGCAGTCA	690	566	1	1	0	0
DOK1	CAACCAACACTGCAGTAC	125	119	0	0	0	0
DOK1	GTACCAACACTGCAGTTG	436	630	0	1	0	0
DOK1	TGACCAACACTGCACAGT	517	488	2	0	0	15
DOK1	GTACCAACACTGCACACA	80	106	0	0	0	0
DOK1	TGTGCACACAACTGCACA	24	46	0	0	0	0
DOK2	ACTGGTTGGTTGTGACGT	154	372	0	0	0	1
DOK2	TGTGGTTGGTTGTGACCA	138	165	0	0	0	0
DOK2	CATGGTTGGTTGTGACAC	133	86	0	0	0	1
DOK2	GTTGGTTGGTTGTGACTG	629	487	1	0	0	9
DOK2	TGTGGTTGGTTGTGTGGT	123	128	0	0	0	0
DOLPP1	TGACACACACGTCATGCA	738	409	0	0	1	1
DOLPP1	ACACACACACGTCATGGT	1077	996	29	0	0	0
DOLPP1	CAACACACACGTCATGAC	38	51	0	0	0	0
DOLPP1	GTACACACACGTCATGTG	345	308	0	1	0	94

DOLPP1	ACACACACACGTACGTGT	80	127	0	0	0	0
DOT1L	TGACGTTGGTTGACTGGT	365	596	1	0	0	0
DOT1L	GTACGTTGGTTGACTGCA	196	181	0	0	0	0
DOT1L	ACACGTTGGTTGACTGAC	176	164	1	0	0	0
DOT1L	GTTGGTTGTGCACATGGT	376	271	1	0	0	1
DOT1L	CATGGTTGTGCACATGCA	442	153	0	0	0	0
DOT1L	TGTGCAACACACACCACA	116	81	0	0	0	0
DPAGT1	GTACCAACGTTGCATGTG	443	439	2	0	0	1
DPAGT1	ACACCAACGTTGACGTGT	334	291	0	0	0	0
DPAGT1	TGACCAACGTTGACGTCA	398	292	0	0	0	0
DPAGT1	CAACCAACGTTGACGTAC	82	131	0	0	0	0
DPAGT1	GTACCAACGTTGACGTTG	149	144	0	0	0	0
DPEP1	CAACACGTTGTGTGCAGT	109	139	0	0	0	0
DPEP1	ACACACGTTGTGTGCACA	10	22	0	0	0	0
DPEP1	GTACACGTTGTGTGCAAC	119	25	0	0	0	0
DPEP1	TGACACGTTGTGTGCATG	63	53	0	0	0	0
DPEP1	ACTGCAGTTGGTCATGCA	386	431	0	0	0	0
DPF2	ACACGTACGTACCAACCA	274	223	0	2	1	0
DPF2	CAACGTACGTACCAACGT	928	264	0	1	0	0
DPF2	GTACGTACGTACCAACAC	48	31	0	0	0	0
DPF2	TGACGTACGTACCAACTG	77	92	0	1	0	0
DPF2	TGTGGTTGTGGTCAACAC	9	22	0	0	0	0
DPF2	CATGCAACACCAGTCAAC	22	20	0	0	0	0
DPM1	CAACCAACCACACAGTGT	2497	1533	13	4939	10	32
DPM1	ACACCAACCACACAGTCA	244	314	0	0	0	0
DPM1	GTACCAACCACACAGTAC	86	57	0	0	0	7
DPM1	TGACCAACCACACAGTTG	770	794	0	0	0	1
DPM1	ACACCAACCACACACAGT	262	192	1	0	0	0
DPM1	TGTGCACAGTACCAACAC	54	53	0	0	0	0
DPM2	CAACCAACCACACAACTG	1522	1616	57	1	1	29
DPM2	GTACCAACCACACACATG	695	864	0	1	0	0
DPM2	TGACCAACCACACAACGT	293	225	0	0	0	0
DPM2	GTACCAACCACACAACCA	429	148	0	0	0	2
DPM2	ACACCAACCACACAACAC	13	22	0	0	0	0
DPM2	TGTGCAACTGACGTACAC	106	122	0	1	0	0
DPP4	CAACCAGTTGTGCACACA	343	152	0	0	0	0
DPP4	GTACACACGTACCAGTAC	39	51	0	0	0	0
DPP4	TGACACACGTACCAGTTG	283	258	1	0	0	0
DPP4	ACACACACGTACCACAGT	99	65	0	1	0	0
DPP4	TGACACACGTACCACACA	123	76	0	0	0	0
DPPA2	CATGGTACTGCACAACAC	20	40	0	0	0	0
DPPA2	GTTGGTACTGCACAACTG	154	325	1	0	0	0
DPPA2	TGTGGTACTGCACATGGT	106	160	1	0	0	0
DPPA2	GTTGGTACTGCACATGCA	49	78	0	0	0	0
DPPA2	ACTGGTACTGCACATGAC	35	32	0	0	0	0
DPPA2	ACTGCATGCACAGTCACA	39	45	0	0	0	0
DPPA3	ACTGCAGTGTACACTGAC	25	11	0	0	0	0
DPPA3	CATGCAGTGTACACTGTG	528	149	1	1	0	0
DPPA3	TGTGCAGTGTACTGGTGT	945	468	1	1	0	77
DPPA3	GTTGCAGTGTACTGGTCA	776	1033	2	0	0	0
DPPA3	TGTGACGTACCAACCATG	14	36	0	0	0	0
DPPA4	CATGGTCATGACGTGTAC	63	65	0	0	0	0
DPPA4	GTTGGTCATGACGTGTTG	501	544	0	2	0	1
DPPA4	TGTGGTCATGACGTCAGT	76	93	0	0	0	0
DPPA4	GTTGGTCATGACGTCACA	40	38	0	0	0	0
DPPA4	GTTGGTTGCAACGTGTCA	114	140	0	0	0	0
DPPA5	TGTGGTTGACCATGTGGT	752	1215	0	0	0	1
DPPA5	GTTGGTTGACCATGTGCA	247	486	0	0	0	0
DPPA5	ACTGGTTGACCATGTGAC	48	24	1	0	0	0
DPPA5	GTTGACCACAGTTGGTCA	239	422	3	0	0	0
DPPA5	ACTGACCACAGTTGGTAC	70	104	0	0	0	1
DPYD	ACACCAGTGTACCATGAC	61	41	0	0	0	0
DPYD	CAACCAGTGTACCATGTG	119	85	0	0	0	0
DPYD	TGACCAGTGTACACGTGT	221	317	1	2	0	0
DPYD	GTACCAGTGTACACGTCA	119	110	0	0	0	0
DPYD	ACACCAGTGTACACGTAC	52	18	0	0	0	0
DPYS	TGACACCAGTGTGTTGGT	141	432	0	0	1	0
DPYS	CAACACCAGTGTGTACAC	121	93	0	0	0	0
DPYS	GTACACCAGTGTGTACTG	80	69	0	0	0	0
DPYS	GTACACCAGTGTGTTGCA	55	123	0	0	0	0
DPYS	ACACACCAGTGTGTTGAC	21	23	0	0	0	0
DPYS	ACTGCACAGTACCAACTG	795	927	1	5	0	1
DPYSL2	CAACCAGTTGTGACGTAC	83	128	0	0	0	0
DPYSL2	CAACACCAGTGTCAACGT	190	80	0	0	0	0
DPYSL2	ACACACCAGTGTCAACCA	18	13	0	0	0	0

DPYSL2	GTACACCAGTGTCAACAC	61	53	0	1	0	0
DPYSL2	TGACACCAGTGTCAACTG	214	197	0	0	0	0
DPYSL5	CAACACCAGTGTTGACTG	381	464	1	0	1	0
DPYSL5	GTACCAGTTGTGACCACA	114	140	1	0	0	0
DPYSL5	GTACACCAGTGTTGACCA	90	113	0	0	0	0
DPYSL5	ACACACCAGTGTTGACAC	21	21	0	0	0	0
DPYSL5	GTACACCAGTGTTGTGGT	433	596	0	0	0	0
DPYSL5	TGTGCAACCACAACTGAC	72	83	0	0	0	0
DRAM	CATGGTTGACCATGTGTG	455	557	2	1	1	64
DRAM	ACTGGTTGACACGTGTCA	477	545	1	0	1	0
DRAM	TGTGGTTGACACGTGTTG	271	352	12	0	1	0
DRAM	CATGGTTGACACGTGTGT	362	388	0	2	0	0
DRAM	GTTGGTTGACACGTGTAC	84	103	0	0	0	1
DRD1	CAACGTCAGTGTACGTCA	10	5	0	0	0	0
DRD1	TGACGTCAGTGTACGTAC	58	49	1	0	0	0
DRD1	ACACGTCAGTGTACGTTG	102	77	0	0	0	0
DRD1	CAACGTCAGTGTACCAGT	98	80	0	0	0	0
DRD1	ACACGTCAGTCACAGTTG	40	84	0	0	0	0
DRD2	ACACGTCAGTGTACCACA	27	64	0	0	0	2
DRD2	GTACGTCAGTGTACCAAC	3	6	0	0	0	0
DRD2	TGACGTCAGTGTACCATG	111	89	0	0	0	0
DRD2	ACACGTCAGTGTACACGT	7	12	0	0	0	0
DRD2	CAACGTCAGTCACACAGT	265	202	2	2	0	0
DRD2	TGTGCAACGTTGACGTGT	650	461	0	0	0	0
DRD3	TGACGTCAGTGTACACCA	382	438	0	0	0	0
DRD3	CAACGTCAGTGTACACAC	17	12	0	0	0	0
DRD3	GTACGTCAGTGTACACTG	50	28	0	0	0	0
DRD3	TGACGTCAGTGTACTGGT	131	183	1	0	0	175
DRD3	ACACGTCAGTCACACACA	183	132	0	0	0	0
DRD4	GTTGCATGCACATGTGGT	469	501	229356	235	10	26
DRD4	CATGCATGCACATGTGCA	105	171	0	0	2	0
DRD4	TGACGTACGTGTTGTGGT	251	307	1	0	1	0
DRD4	TGACGTACGTGTTGACCA	205	184	0	0	0	0
DRD4	CAACGTACGTGTTGACAC	29	23	0	0	0	0
DRD4	GTACGTACGTGTTGACTG	362	320	0	0	0	0
DRD5	GTACGTCAGTGTACTGCA	61	74	0	0	0	0
DRD5	ACACGTCAGTGTACTGAC	40	136	0	0	0	0
DRD5	CAACGTCAGTGTACTGTG	544	356	0	0	0	0
DRD5	GTACGTCAGTCACACAAC	3	9	0	0	0	0
DRD5	TGACGTCAGTCACACATG	243	597	1	0	0	0
DSE	GTTGGTCATGCACAGTCA	669	748	2	0	6	0
DSE	ACTGGTCATGCACAGTAC	57	89	0	0	0	0
DSE	CATGGTCATGCACAGTTG	386	388	0	0	0	1
DSE	GTTGGTCATGCACACAGT	675	474	1	0	0	0
DSE	CATGGTCATGCACACACA	78	104	0	0	0	0
DSP	ACTGGTGTACTGCATGCA	146	353	1	0	1	1
DSP	TGTGGTGTACTGCATGTG	1064	820	0	0	1	2
DSP	ACTGGTGTACTGCAACTG	200	435	1	1	0	6
DSP	CATGGTGTACTGCATGGT	340	217	0	1	0	0
DSP	GTTGGTGTACTGCATGAC	161	89	1	0	0	0
DTNBP1	GTACTGTGTGACCAACAC	36	68	0	0	0	0
DTNBP1	TGACTGTGTGACCAACTG	215	157	0	0	0	0
DTNBP1	ACACTGTGTGACCATGGT	60	82	1	0	0	96
DTNBP1	TGACTGTGTGACCATGCA	153	231	0	0	0	0
DTNBP1	GTTGCATGACCAGTCACA	26	65	0	0	0	0
DTX1	CACATGTGACGTGTCATG	189	124	0	0	0	0
DTX1	GTCATGTGACGTGTACGT	137	148	0	0	0	1
DTX1	CACATGTGACGTGTACCA	152	269	0	0	0	0
DTX1	TGCATGTGACGTGTACAC	63	46	0	0	0	0
DTX1	ACCATGTGACGTGTACTG	144	118	0	0	0	0
DTX2	GTCATGTGCACAGTACAC	57	69	0	0	0	0
DTX2	TGCATGTGCACAGTACTG	1006	1653	1	1	0	1
DTX2	ACCATGTGCACAGTTGGT	63	115	0	0	0	0
DTX2	TGCATGTGCACAGTTGCA	188	241	0	0	0	0
DTX2	TGTGCAGTGTCAACACAC	48	94	0	0	0	0
DTX2	TGTGCAACTGCAGTACCA	222	271	0	0	0	0
DTX3	CAACTGTGGTGTGTGTGT	915	618	0	2	0	0
DTX3	ACACTGTGGTGTGTGTCA	250	728	1	7	0	8
DTX3	GTACTGTGGTGTGTGTAC	4	3	0	0	0	0
DTX3	TGACTGTGGTGTGTGTTG	944	711	2	0	0	0
DTX3	ACACTGTGGTGTGTCAGT	47	71	0	0	0	0
DTX3	GTTGCATGGTTGACACTG	206	178	0	0	0	0
DTX3L	CAACTGACTGTGTGTGCA	409	561	0	9	1	0
DTX3L	CAACTGACTGTGTGACTG	291	417	0	0	0	0
DTX3L	GTACTGACTGTGTGTGGT	140	169	0	0	0	0

DTX3L	TGACTGACTGTGTGTGAC	3	16	0	0	0	0
DTX3L	ACACTGACTGTGTGTGTG	329	644	0	1	0	0
DTX3L	ACTGCATGGTTGTGGTAC	63	115	0	0	0	0
DTX4	GTTGGTTGTGCATGTGTG	273	369	0	1	1	0
DTX4	TGACCATGACCACACATG	447	495	0	2	0	0
DTX4	CATGGTTGTGCATGTGAC	109	171	0	0	0	0
DTX4	GTTGGTTGTGACGTGTGT	243	336	0	0	0	0
DTX4	CATGGTTGTGACGTGTCA	449	428	0	1	0	7
DTYMK	ACACGTGTGTCATGCAGT	642	598	9	0	0	0
DTYMK	TGACGTGTGTCATGCACA	71	112	0	0	0	0
DTYMK	CAACGTGTGTCATGCAAC	44	31	0	1	0	0
DTYMK	GTACGTGTGTCATGCATG	90	118	0	0	0	0
DTYMK	GTTGGTACTGTGACCACA	51	33	1	0	0	0
DUOX1	TGACACGTACTGGTTGGT	262	818	1	0	0	0
DUOX1	GTACACGTACTGGTTGCA	52	88	0	1	0	0
DUOX1	ACACACGTACTGGTTGAC	18	23	0	0	0	0
DUOX1	CAACACGTACTGGTTGTG	607	533	0	0	0	0
DUOX1	TGACACGTACTGCAGTGT	2055	1045	3	1	0	0
DUOX1	TGTGCATGGTCAACTGAC	37	34	0	0	0	0
DUSP1	ACCATGACCAACACCAAC	48	24	0	0	0	0
DUSP1	CACATGACCAACACCATG	503	727	0	0	0	0
DUSP1	GTCATGACCAACACACGT	45	46	0	0	0	0
DUSP1	CACATGACCAACACACCA	362	332	0	0	0	0
DUSP1	TGCATGACCAACACACAC	37	41	0	1	0	0
DUSP1	GTTGCACAACACACTGCA	162	134	0	0	0	0
DUSP10	ACCATGACGTACACCAGT	165	138	0	0	0	0
DUSP10	TGCATGACGTACACCACA	34	92	0	0	0	0
DUSP10	CACATGACGTACACCAAC	43	48	0	0	0	0
DUSP10	GTCATGACGTACACCATG	405	240	0	0	0	0
DUSP10	TGCATGACGTACACACGT	39	23	0	0	0	0
DUSP10	TGTGCAACACCAGTACGT	102	131	0	0	0	0
DUSP14	TGCATGACGTACCATGTG	785	1369	1	1	1	0
DUSP14	TGCATGACGTACACGTTG	177	158	0	1	1	0
DUSP14	CACATGACGTACACGTGT	960	404	0	1	0	1
DUSP14	ACCATGACGTACACGTCA	374	116	0	0	0	1
DUSP14	GTCATGACGTACACGTAC	13	25	0	0	0	0
DUSP4	GTACGTGTCACAGTCATG	151	132	1	0	0	0
DUSP4	TGACGTGTCACAGTACGT	70	233	1	0	0	0
DUSP4	GTACGTGTCACAGTACCA	289	240	0	0	0	0
DUSP4	GTTGGTTGTGTGCAGTGT	813	228	1	0	0	0
DUSP4	CATGGTTGTGTGCAGTCA	337	384	0	0	0	1
DUSP4	GTTGCAACACCAGTACCA	1013	392	2	1	0	1
DUSP6	CACATGACCACATGACGT	545	347	0	0	0	0
DUSP6	ACCATGACCACATGACCA	141	99	0	0	0	0
DUSP6	GTCATGACCACATGACAC	1	4	0	0	0	0
DUSP6	TGACACTGGTTGACACAC	34	37	0	0	0	0
DUSP6	TGTGGTTGTGCAGTGTCA	177	210	0	0	0	1
DUSP8	GTACTGTGACACTGACGT	188	101	0	0	0	0
DUSP8	CAACTGTGACACTGACCA	353	388	2	1	0	0
DUSP8	TGACTGTGACACTGACAC	75	75	0	0	0	5
DUSP8	ACACTGTGACACTGACTG	554	570	1	1	0	8
DUSP8	TGTGGTTGTGACGTGTAC	18	33	0	0	0	0
DUSP9	ACCATGACCACAACTGAC	33	70	2	0	0	0
DUSP9	CACATGACCACAACTGTG	607	583	0	0	0	0
DUSP9	TGCATGACCACATGGTGT	501	363	0	0	0	0
DUSP9	GTTGGTTGTGCATGGTCA	196	318	1	0	0	4
DUSP9	ACTGGTTGTGCATGGTAC	80	104	0	3	0	0
DUSP9	GTTGCACAGTACCATGAC	52	50	0	0	0	0
DUT	ACACACACGTTGCACAAC	4	4	0	0	0	0
DUT	CAACACACGTTGCACATG	413	459	0	50	0	1
DUT	GTACACACGTTGCAACGT	229	309	0	2	0	1
DUT	CAACACACGTTGCAACCA	110	94	0	0	0	0
DUT	TGACACACGTTGCAACAC	0	3	0	0	0	0
DVL1	ACACCACAGTGTTGCAAC	9	13	0	0	0	0
DVL1	CAACCACAGTGTTGCATG	133	175	0	0	0	0
DVL1	GTTGACACACTGCATGAC	41	36	0	0	0	0
DVL1	TGTGACACACTGCATGTG	1055	756	1	0	0	0
DVL1	CATGACACACTGACGTGT	338	496	0	1	0	0
DVL2	ACACCACAGTGTTGACTG	42	71	0	2	2	1
DVL2	GTACCACAGTGTTGACGT	63	64	0	0	0	0
DVL2	CAACCACAGTGTTGACCA	470	334	0	0	0	1
DVL2	TGACCACAGTGTTGACAC	452	396	0	0	0	0
DVL2	CAACCACAGTGTTGTGGT	501	823	0	0	0	5
DVL3	TGACCACAGTGTTGTGTG	195	272	0	0	4	0
DVL3	ACACCACAGTGTTGTGCA	91	201	0	0	0	0

DVL3	GTACCACAGTGTTGTGAC	20	14	0	0	0	0
DVL3	GTACCACAGTCAGTGTGT	629	613	0	0	0	0
DVL3	CAACCACAGTCAGTGTCA	1008	909	3	1	0	0
DYRK1A	GTCATGCAACGTACTGAC	28	34	0	0	0	0
DYRK1A	TGCATGCAACGTACTGTG	211	133	0	0	0	0
DYRK1A	CACATGCAACGTTGGTGT	771	1107	1	1	0	5
DYRK1A	ACCATGCAACGTTGGTCA	318	547	0	0	0	0
DYRK1A	GTCATGCAACGTTGGTAC	153	160	0	0	0	0
DYRK1B	GTCATGACCAGTCAGTTG	756	1101	5	2	2	7
DYRK1B	GTCATGACCAGTGTTGTG	225	144	0	0	0	0
DYRK1B	ACCATGACCAGTCAGTGT	918	1022	1	0	0	1
DYRK1B	TGCATGACCAGTCAGTCA	60	92	0	0	0	0
DYRK1B	CACATGACCAGTCAGTAC	241	187	0	0	0	2
DYRK1B	GTTGCACAACCAACGTTG	350	270	0	0	0	0
DYSF	GTCATGCATGCACAACCA	422	457	3	0	0	0
DYSF	ACCATGCATGCACAACAC	9	17	0	0	0	0
DYSF	CACATGCATGCACAACTG	144	107	0	0	0	0
DYSF	GTCATGCATGCACATGGT	502	479	0	0	0	0
DYSF	CACATGCATGCACATGCA	185	374	1	0	0	0
DYSF	ACTGCAACCAGTTGTGTG	315	319	0	0	0	0
E2F1	TGCATGCACACAACACTG	927	622	0	0	0	1
E2F1	ACCATGCACACAACTGGT	126	206	0	0	0	0
E2F1	TGCATGCACACAACTGCA	481	236	1	0	0	0
E2F1	CACATGCACACAACTGAC	187	168	2	0	0	0
E2F1	GTCATGCACACAACTGTG	114	151	0	0	0	0
E2F1	ACTGCACATGACTGCAAC	56	47	0	0	0	0
E2F2	TGACGTCATGCACAGTCA	339	356	1	1	0	0
E2F2	CAACGTCATGCACAGTAC	38	31	0	0	0	0
E2F2	GTACGTCATGCACAGTTG	519	449	3	0	0	0
E2F2	TGACGTCATGCACACAGT	107	139	0	0	0	0
E2F2	GTTGCAGTACACCAACTG	316	248	1	0	0	2
E2F3	GTACGTCATGCACACACA	145	198	0	1	0	0
E2F3	ACACGTCATGCACACAAC	46	25	1	1	0	1
E2F3	CAACGTCATGCACACATG	483	450	0	4	0	0
E2F3	GTACGTCATGCACAACGT	271	335	3	1588	0	0
E2F3	CAACGTCATGCACAACCA	183	240	0	1	0	0
E2F3	CATGCACATGACTGCATG	108	94	0	1	0	0
E2F4	ACACGTCATGCACAACTG	951	1436	1	0	1	0
E2F4	ACACGTCATGCACATGCA	232	258	1	2	1	1
E2F4	TGACGTCATGCACAACAC	79	160	1	0	0	0
E2F4	CAACGTCATGCACATGGT	351	341	0	0	0	0
E2F4	ACTGGTGTGTTGCATGGT	567	897	0	0	0	0
E2F5	TGACGTCATGCACATGTG	463	145	0	0	1	0
E2F5	GTACGTCATGCACATGAC	10	11	0	0	0	0
E2F5	CAACGTCATGCAACGTGT	153	221	0	0	0	0
E2F5	ACACGTCATGCAACGTCA	78	70	0	0	0	8
E2F5	GTACGTCATGCAACGTAC	4	9	0	0	0	0
EARS2	TGTGGTACTGGTTGGTGT	388	876	0	1	1	1
EARS2	GTTGGTACTGGTTGGTCA	379	438	2	0	0	0
EARS2	ACTGGTACTGGTTGGTAC	69	60	0	0	0	0
EARS2	CATGGTACTGGTTGGTTG	481	584	2	1	0	15
EARS2	GTTGGTTGGTACACCAAC	2	2	0	0	0	0
EBF1	TGACGTCATGCAACGTTG	156	548	0	1	0	0
EBF1	ACACGTCATGCAACCAGT	342	314	2	0	0	0
EBF1	TGACGTCATGCAACCACA	66	86	0	0	0	2
EBF1	CAACGTCATGCAACCAAC	25	29	0	0	0	0
EBF1	GTACGTCATGCAACCATG	243	224	1	0	0	0
EBI2	TGACGTCAGTGTTGGTGT	1990	1562	6	1	1	4
EBI2	GTACGTCAGTGTTGGTCA	362	340	1	0	0	1
EBI2	ACACGTCAGTGTTGGTAC	6	5	0	0	0	0
EBI2	CAACGTCAGTGTTGGTTG	334	1358	1	0	0	0
EBI2	GTACGTCAGTGTTGCAGT	221	43	0	0	0	0
EBI3	TGACTGCAACGTACTGGT	475	808	2	1	0	0
EBI3	GTACTGCAACGTACTGCA	193	312	0	0	0	0
EBI3	ACACTGCAACGTACTGAC	49	34	0	0	0	0
EBI3	CAACTGCAACGTACTGTG	321	361	1	0	0	0
EBI3	CATGCAACGTGTTGACAC	32	28	0	0	0	0
EBI3	GTTGCAACGTGTTGACTG	280	328	0	1	0	73
EBP	TGACACCAACTGCACATG	202	143	1	0	0	0
EBP	ACACACCAACTGCAACGT	733	849	0	0	0	1
EBP	TGACACCAACTGCAACCA	285	199	1	0	0	0
EBP	CAACACCAACTGCAACAC	8	14	0	0	0	0
EBP	ACTGCACAACCAGTTGAC	90	39	0	0	0	0
EBP	CATGCACAACCAGTTGTG	175	192	1	0	0	0
ECE2	GTACCAGTTGTGCACAGT	84	93	1	0	0	0

ECE2	CAACACCAGTCACAACCA	329	367	0	1	0	0
ECE2	TGACACCAGTCACAACAC	17	26	0	0	0	0
ECE2	ACACACCAGTCACAACTG	361	411	0	1	0	1
ECE2	CAACACCAGTCACATGGT	1049	1617	3	1	0	1
ECGF1	ACTGCACAACTGCACAAC	311	338	856	969938	164196	291
ECGF1	ACACTGGTACCATGCAGT	227	182	0	0	1	0
ECGF1	ACACTGGTACCATGGTCA	422	424	1	1	0	0
ECGF1	GTACTGGTACCATGGTAC	85	62	0	0	0	0
ECGF1	TGACTGGTACCATGGTTG	1308	1143	2	1	0	0
ECGF1	TGACTGGTACCATGCACA	213	226	0	0	0	0
ECHS1	CAACACACTGGTACGTGT	1016	849	2	0	1	0
ECHS1	ACACACACTGGTCATGCA	95	208	0	0	0	0
ECHS1	GTACACACTGGTCATGAC	253	188	0	0	0	0
ECHS1	TGACACACTGGTCATGTG	270	190	1	0	0	0
ECHS1	ACACACACTGGTACGTCA	187	173	0	0	0	0
ECHS1	GTTGCACATGACTGACGT	319	200	2	2	0	0
ECSIT	CATGGTGTACCACACACA	29	69	0	0	0	0
ECSIT	CATGGTGTACCATGACTG	115	117	0	2	0	0
ECSIT	GTTGGTGTACCATGTGGT	985	918	0	1	0	1
ECSIT	CATGGTGTACCATGTGCA	41	37	0	0	0	0
ECSIT	TGTGGTGTACCATGTGAC	45	60	0	0	0	0
ECSIT	CATGCAACCAACACGTTG	358	220	0	0	0	0
EDA	CAACTGGTTGACCAGTCA	576	519	13	1	0	0
EDA	CATGACGTACACACTGGT	359	412	1	0	0	0
EDA	ACTGACGTACACACTGCA	302	213	0	1	0	0
EDA	GTTGACGTACACACTGAC	44	46	0	0	0	0
EDA	TGTGACGTACACACTGTG	153	109	0	1	0	0
EDG1	CAACGTCAGTGTTGCACA	318	240	0	0	0	0
EDG1	TGACGTCAGTGTTGCAAC	35	29	0	0	0	0
EDG1	ACACGTCAGTGTTGCATG	100	253	0	0	0	0
EDG1	ACACGTCAGTCACAACGT	400	424	0	0	0	0
EDG1	TGACGTCAGTCACAACCA	279	292	0	7	0	0
EDG4	CAACGTCAGTGTTGACGT	235	299	1	0	0	0
EDG4	ACACGTCAGTGTTGACCA	103	215	0	0	0	0
EDG4	GTACGTCAGTGTTGACAC	11	18	0	0	0	0
EDG4	TGACGTCAGTGTTGACTG	373	534	1	0	0	1
EDG4	ACACGTCAGTGTTGTGGT	315	257	0	0	0	0
EDG7	TGACGTCAGTGTTGTGCA	38	43	1	0	1	0
EDG7	CAACGTCAGTGTTGTGAC	43	62	0	0	0	0
EDG7	GTACGTCAGTGTTGTGTG	621	645	0	0	0	0
EDG7	CAACGTCAGTCACAACAC	4	7	0	0	0	0
EDG7	GTACGTCAGTCACAACTG	307	230	1	2	0	1
EDG7	CATGCACAACACCATGCA	189	308	0	0	0	0
EDN1	ACCATGTGGTGTTGACGT	326	359	2	6	6	17
EDN1	TGCATGTGGTGTTGCATG	45	78	0	0	0	0
EDN1	TGCATGTGGTGTTGACCA	148	320	0	0	0	0
EDN1	CACATGTGGTGTTGACAC	26	34	0	0	0	0
EDN1	GTCATGTGGTGTTGACTG	659	391	86	0	0	0
EDN1	TGTGCAACACGTGTCAGT	150	125	0	0	0	0
EDN3	ACTGGTGTTGTGACACGT	97	78	1	0	0	0
EDN3	TGTGGTGTTGTGACACCA	234	224	0	0	0	0
EDN3	CATGGTGTTGTGACACAC	189	95	0	0	0	0
EDN3	GTTGGTGTTGTGACACTG	156	149	0	0	0	0
EDN3	TGTGACGTGTTGTGTGCA	172	173	0	0	0	0
EDNRA	ACACGTCAGTCAGTGTCA	208	257	0	0	1	1
EDNRA	CAACGTCAGTCAGTGTGT	113	101	0	0	0	0
EDNRA	GTACGTCAGTCAGTGTAC	31	44	0	0	0	0
EDNRA	TGACGTCAGTCAGTGTTG	287	450	0	0	0	0
EDNRA	ACACGTCAGTCAGTCAGT	64	114	0	0	0	0
EDNRA	TGTGCACAGTACCATGTG	296	316	127	0	0	0
EDNRB	TGACGTCAGTCAGTCACA	176	130	0	0	1	19
EDNRB	CAACGTCAGTCAGTCAAC	89	60	1	0	0	0
EDNRB	GTACGTCAGTCAGTCATG	494	132	0	1	0	2
EDNRB	TGACGTCAGTCAGTACGT	54	72	0	0	0	0
EDNRB	TGACGTCAGTCACATGGT	340	320	0	2	0	0
EDNRB	GTTGCACACACATGACAC	101	45	0	0	0	0
EEA1	ACACCAGTACACACCAAC	6	16	0	0	0	0
EEA1	CAACCAGTACACACCATG	127	109	0	2	0	0
EEA1	GTACCAGTACACACACGT	9	4	0	0	0	0
EEA1	CAACCAGTACACACACCA	578	460	0	0	0	0
EEA1	TGACCAGTACACACACAC	40	66	0	0	0	0
EED	GTACGTTGACGTCAGTTG	741	576	0	2	0	2
EED	TGACGTTGACGTCACAGT	538	157	0	0	0	0
EED	GTACGTTGACGTCACACA	228	130	0	0	0	0
EED	ACACGTTGACGTCACAAC	128	16	0	0	0	0

EED	CAACGTTGACGTCACATG	433	133	0	0	0	0
EED	CATGCAACTGGTTGACCA	124	174	1	0	0	0
EEF1A2	TGTGGTCAACGTTGGTGT	1417	930	0	1	1	1
EEF1A2	GTTGGTCAACGTTGGTCA	252	314	0	0	1	2
EEF1A2	ACTGGTCAACGTACTGAC	22	33	0	0	0	0
EEF1A2	CATGGTCAACGTACTGTG	397	725	0	1	0	0
EEF1A2	ACTGGTCAACGTTGGTAC	166	163	0	0	0	0
EEF1A2	CATGCAACTGACACCAGT	237	135	1	0	0	4
EEF1B2	GTACACTGGTTGCATGTG	154	194	0	0	0	0
EEF1B2	ACTGGTCATGGTCAACGT	350	395	1	0	0	1
EEF1B2	TGTGGTCATGGTCAACCA	133	217	0	0	0	0
EEF1B2	CATGGTCATGGTCAACAC	70	82	0	0	0	0
EEF1B2	GTTGGTCATGGTCAACTG	397	1166	1	0	0	4
EEF1D	GTACACCAGTACCATGCA	408	802	0	512	116	1
EEF1D	ACTGCAACTGACACCACA	466	628	2	1	2	0
EEF1D	CAACACCAGTACCAACAC	32	18	0	0	0	0
EEF1D	GTACACCAGTACCAACTG	155	220	0	0	0	0
EEF1D	TGACACCAGTACCATGGT	314	199	1	0	0	6
EEF1D	TGTGCAGTTGCACATGAC	20	22	0	0	0	0
EEF1G	ACTGCAGTGTGTTGACTG	2734	2562	1992	829756	569276	884992
EEF1G	GTTGCAGTGTGTTGACGT	157	144	0	0	0	0
EEF1G	CATGCAGTGTGTTGACCA	385	278	0	0	0	0
EEF1G	TGTGCAGTGTGTTGACAC	19	51	0	0	0	0
EEF1G	CATGACCAACCACAACCA	222	186	0	0	0	0
EEF2	GTACACCACAGTTGGTTG	247	903	0	0	0	0
EEF2	TGACACCACAGTTGCAGT	300	341	1	0	0	1
EEF2	GTACACCACAGTTGCACA	53	60	0	0	0	0
EEF2	ACACACCACAGTTGCAAC	33	34	0	0	0	0
EEF2	CAACACCACAGTTGCATG	307	372	0	1	0	12
EEF2K	CAACGTGTGTGTACCACA	91	64	0	0	0	1
EEF2K	TGACGTGTGTGTACCAAC	147	87	0	0	0	0
EEF2K	ACACGTGTGTGTACCATG	66	141	0	0	0	0
EEF2K	CAACGTGTGTGTACACGT	32	29	0	0	0	0
EEF2K	ACACGTGTGTGTACACCA	257	175	0	0	0	0
EFNA1	CAACGTGTACGTGTACAC	14	24	0	0	0	0
EFNA1	GTACGTGTACGTGTACTG	644	178	0	0	0	0
EFNA1	TGACGTGTACGTGTTGGT	100	97	0	0	0	1
EFNA1	GTACGTGTACGTGTTGCA	208	281	0	1	0	0
EFNA1	ACTGCATGCAACTGACCA	299	217	0	0	0	0
EFNA2	ACACTGGTACGTGTCAGT	173	128	0	0	0	0
EFNA2	TGACTGGTACGTGTCACA	130	110	0	0	0	1
EFNA2	CAACTGGTACGTGTCAAC	37	42	0	0	0	0
EFNA2	GTACTGGTACGTGTCATG	368	171	0	1	0	0
EFNA2	ACTGCAGTTGACGTGTTG	447	478	0	1	0	0
EFNA2	TGTGCATGGTGTTGGTTG	753	699	2	1	0	0
EFNA3	ACACTGGTTGCAGTACCA	367	423	0	1	1	1
EFNA3	CAACTGGTTGCAGTACGT	425	364	0	0	0	2
EFNA3	GTACTGGTTGCAGTACAC	12	18	0	0	0	0
EFNA3	TGACTGGTTGCAGTACTG	305	326	0	0	0	0
EFNA3	ACACTGGTTGCAGTTGGT	104	58	0	0	0	0
EFNA4	CAACTGCAGTACGTACAC	134	272	0	0	0	0
EFNA4	GTACTGCAGTACGTACTG	194	114	0	1	0	0
EFNA4	TGACTGCAGTACGTTGGT	243	229	0	0	0	0
EFNA4	GTACTGCAGTACGTTGCA	104	154	0	0	0	0
EFNA4	ACACTGCAGTACGTTGAC	79	51	0	0	0	0
EFNA5	TGACTGGTCATGACTGCA	255	240	1	0	0	0
EFNA5	CAACTGGTCATGACTGAC	90	13	0	1	0	0
EFNA5	GTACTGGTCATGACTGTG	286	209	0	1	0	0
EFNA5	ACACTGGTCATGTGGTGT	896	429	0	0	0	0
EFNA5	TGACTGGTCATGTGGTCA	1082	1023	1	1	0	1
EFNA5	CATGCACAGTACACGTGT	409	551	0	0	0	0
EFNB1	CATGCATGGTTGCAACTG	100	422	0	0	1	0
EFNB1	TGACTGGTTGGTACGTCA	195	290	1	0	0	0
EFNB1	CAACTGGTTGGTACGTAC	331	234	0	0	0	0
EFNB1	GTACTGGTTGGTACGTTG	86	72	0	0	0	0
EFNB1	TGACTGGTTGGTACCAGT	136	158	0	0	0	0
EFNB1	TGTGGTTGCATGGTCATG	85	26	0	0	0	0
EFNB2	TGACTGGTACCATGTGAC	114	122	0	0	0	0
EFNB2	ACACTGGTACCATGTGTG	259	461	3	1	0	1
EFNB2	ACACTGGTACACGTGTGT	311	548	0	0	0	0
EFNB2	TGACTGGTACACGTGTCA	53	45	0	0	0	0
EFNB2	CATGCAGTGTTGGTTGAC	48	123	0	0	0	0
EFTUD2	CAACTGTGGTTGCACATG	63	71	0	0	0	0
EFTUD2	GTACTGTGGTTGCAACGT	520	228	1	0	0	0
EFTUD2	CAACTGTGGTTGCAACCA	278	442	0	0	0	4

EFTUD2	TGACTGTGGTTGCAACAC	62	82	0	0	0	0
EFTUD2	ACACTGTGGTTGCAACTG	225	177	0	0	0	0
EGF	TGACACTGGTTGTGGTTG	1006	1311	0	1	1	17
EGF	ACACACTGGTTGTGCAGT	231	223	0	0	0	0
EGF	TGACACTGGTTGTGCACA	104	89	0	0	0	0
EGF	CAACACTGGTTGTGCAAC	78	54	0	0	0	0
EGF	GTACACTGGTTGTGCATG	85	81	0	0	0	0
EGF	TGTGCACAACCATGGTTG	1177	1136	1	1	0	1
EGFR	TGTGGTCACAGTCAGTGT	208	240	1	0	1	16
EGFR	CAACCATGGTTGACGTTG	352	402	2	0	0	0
EGFR	GTACCATGGTTGACCAGT	26	42	0	0	0	1
EGFR	TGTGGTCACAGTGTACCA	109	171	0	0	0	0
EGFR	CATGGTCACAGTGTTGTG	149	326	1	0	0	0
EGLN1	TGCATGCATGACCAGTGT	469	438	2	0	0	1
EGLN1	GTCATGCATGACCAGTCA	386	433	0	1	0	3
EGLN1	ACCATGCATGACCAGTAC	37	44	0	0	0	0
EGLN1	CACATGCATGACCAGTTG	495	655	0	0	0	1
EGLN1	GTACGTCAACCACAGTCA	761	747	1	1	0	0
EGLN1	GTTGCACATGTGTGCAGT	145	164	1	0	0	0
EGLN3	GTCATGCATGACCACAGT	79	31	0	0	0	0
EGLN3	CACATGCATGACCACACA	154	233	1	0	0	0
EGLN3	TGCATGCATGACCACAAC	21	20	0	0	0	0
EGLN3	ACCATGCATGACCACATG	159	213	3	0	0	0
EGLN3	CACATGCATGACCAACGT	132	156	0	0	0	0
EGR1	TGACGTCATGCAACACGT	384	170	0	1	1	0
EGR1	GTACGTCATGCAACACCA	330	233	0	1	0	0
EGR1	ACACGTCATGCAACACAC	183	48	0	0	0	0
EGR1	CAACGTCATGCAACACTG	808	720	1	0	0	3
EGR1	GTACGTCATGCAACTGGT	181	167	0	0	0	2
EGR1	GTTGCATGGTTGCATGGT	337	663	1	0	0	0
EGR2	CAACGTCATGCATGGTCA	312	786	1	2	1	1
EGR2	CAACGTCATGCAACTGCA	66	96	1	0	0	0
EGR2	TGACGTCATGCAACTGAC	229	131	0	0	0	0
EGR2	ACACGTCATGCAACTGTG	681	870	0	1	0	27
EGR2	GTACGTCATGCATGGTGT	569	328	1	0	0	0
EGR2	CATGCACATGGTTGACTG	855	666	0	0	0	0
EGR3	TGACGTCATGCATGGTAC	109	127	0	0	0	0
EGR3	ACACGTCATGCATGGTTG	618	536	1	4	0	0
EGR3	CAACGTCATGCATGCAGT	33	54	0	0	0	0
EGR3	ACACGTCATGCATGCACA	110	83	0	0	0	0
EGR3	GTACGTCATGCATGCAAC	62	65	0	0	0	0
EGR4	TGACGTCATGCATGCATG	169	303	0	0	0	31
EGR4	ACACGTCATGCATGACGT	238	236	1	0	0	0
EGR4	TGACGTCATGCATGACCA	125	140	0	0	0	0
EGR4	CAACGTCATGCATGACAC	31	17	0	0	0	0
EGR4	CAACTGTGACACACCACA	182	141	0	0	0	0
EGR4	ACTGCACAGTACACGTCA	191	417	0	1	0	0
EHD2	GTACACTGGTCAGTACCA	319	645	0	0	1	0
EHD2	CAACACTGGTCAGTCAAC	37	38	0	0	0	0
EHD2	GTACACTGGTCAGTCATG	459	149	0	1	0	0
EHD2	TGACACTGGTCAGTACGT	286	322	0	0	0	0
EHD2	TGTGCAGTCATGTGTGAC	109	94	1	0	0	0
EHD2	TGTGCAACACGTCAACGT	430	1086	1	2	0	0
EHHADH	CAACCAACACGTTGGTAC	86	56	0	0	0	0
EHHADH	GTACCAACACGTTGGTTG	171	125	0	0	0	0
EHHADH	TGACCAACACGTTGCAGT	69	61	0	0	0	0
EHHADH	GTACCAACACGTTGCACA	193	249	1	0	0	0
EHHADH	ACTGACCACAGTACACGT	82	56	0	0	0	0
EHMT1	GTACCAACGTTGTGACCA	199	318	39	30	21	86106
EHMT1	ACACCAACGTTGTGACAC	51	39	7	0	3	14
EHMT1	TGACCAACGTTGTGACGT	42	40	0	0	0	0
EHMT1	ACTGGTTGTGGTCAGTGT	468	633	3	0	0	5
EHMT1	TGTGGTTGTGGTCAGTCA	625	372	4	1	0	3
EHMT2	CATGGTGTACCAACCATG	32	19	0	0	0	0
EHMT2	GTTGGTGTACCAACACGT	0	2	0	0	0	0
EHMT2	CATGGTGTACCAACACCA	51	78	0	0	0	0
EHMT2	TGTGGTGTACCAACACAC	22	50	0	0	0	0
EHMT2	ACTGGTGTACCAACACTG	830	710	0	0	0	2
EHMT2	CATGCAACCAGTGTGTCA	1227	1123	4	2	0	22
EI24	TGTGGTCACAACCAACCA	172	145	0	0	0	1
EI24	ACTGGTTGCAACGTGTAC	165	354	0	0	0	0
EI24	CATGGTTGCAACGTGTTG	698	534	1	0	0	19
EI24	GTTGGTTGCAACGTCAGT	152	275	0	0	0	0
EI24	CATGGTTGCAACGTCACA	149	110	1	0	0	1
EIF1	CAACTGCATGGTTGTGGT	81	43	0	0	0	0

EIF1	ACACTGCATGGTTGTGCA	216	298	0	0	0	0
EIF1	GTACTGCATGGTTGTGAC	69	96	0	0	0	0
EIF1	TGACTGCATGGTTGTGTG	147	273	0	0	0	0
EIF1	ACTGGTTGTGACGTTGAC	33	66	0	0	0	0
EIF1AX	ACTGGTACTGCAGTTGTG	199	448	1	1	1	0
EIF1AX	CAACTGACGTCAGTGTTG	217	249	0	0	0	16
EIF1AX	GTACTGACGTCAGTCAGT	3	0	0	0	0	0
EIF1AX	TGTGCAGTCACACAACCA	21	29	0	0	0	0
EIF1AX	CATGCAGTCACACAACAC	67	52	1	0	0	0
EIF1AY	ACTGGTCACAACACACCA	492	474	0	0	0	0
EIF1AY	GTTGGTCACAACACACAC	30	14	0	0	0	0
EIF1AY	TGTGGTCACAACACACTG	374	423	0	0	0	0
EIF1AY	ACTGGTCACAACACTGGT	439	366	1	0	0	0
EIF1AY	TGTGGTCACAACACTGCA	73	57	0	0	0	0
EIF2AK1	CATGGTACTGACCATGGT	211	391	1	0	1	0
EIF2AK1	ACTGGTACTGACCATGCA	314	502	0	0	0	0
EIF2AK1	GTTGGTACTGACCATGAC	25	51	0	0	0	0
EIF2AK1	TGTGGTACTGACCATGTG	244	182	4	0	0	0
EIF2AK1	CATGGTACTGACACGTGT	132	108	0	0	0	0
EIF2AK1	CATGCACATGACGTGTGT	297	197	0	0	0	0
EIF2AK2	GTCATGCATGTGCACAAC	18	26	0	0	0	0
EIF2AK2	TGCATGCATGTGCACATG	14	52	0	0	0	11
EIF2AK2	ACCATGCATGTGCAACGT	60	71	0	0	0	0
EIF2AK2	TGCATGCATGTGCAACCA	128	150	1	0	0	0
EIF2AK2	CACATGCATGTGCAACAC	47	130	0	0	0	0
EIF2AK2	TGTGCACACAGTCATGTG	346	659	1	0	0	0
EIF2AK3	GTCATGCATGTGACACAC	28	29	1	0	4	0
EIF2AK3	TGCATGCATGTGACACTG	164	189	0	0	0	2
EIF2AK3	ACCATGCATGTGACTGGT	180	208	0	0	0	0
EIF2AK3	TGCATGCATGTGACTGCA	81	141	3	0	0	0
EIF2AK3	CACATGCATGTGACTGAC	43	42	0	0	0	0
EIF2AK4	TGACGTTGTGACTGACCA	150	115	1	0	0	0
EIF2AK4	ACACTGTGACACCACACA	35	65	0	0	0	0
EIF2AK4	GTACTGTGACACCACAAC	39	27	4	1	0	0
EIF2AK4	TGACTGTGACACCACATG	102	178	0	0	0	0
EIF2AK4	TGTGGTTGTGCACAACGT	28	41	0	0	0	0
EIF2B1	CATGGTGTGTACGTCACA	61	42	0	0	0	0
EIF2B1	TGTGGTGTGTACGTCAAC	15	4	0	0	0	0
EIF2B1	ACTGGTGTGTACGTCATG	79	131	1	0	0	0
EIF2B1	CATGGTGTGTACGTACGT	81	95	0	0	0	0
EIF2B1	ACTGGTGTGTACGTACCA	93	134	0	0	0	0
EIF2B2	CATGGTGTGTACGTTGAC	14	10	2	1	0	5
EIF2B2	GTTGGTGTGTACGTTGTG	494	425	0	0	0	0
EIF2B2	ACTGGTGTGTACCAGTGT	747	888	1	1	0	1
EIF2B2	TGTGGTGTGTACCAGTCA	438	438	0	0	0	0
EIF2B2	ACTGGTACTGTGTGCAGT	430	336	0	1	0	13
EIF2B4	ACACTGTGTGACTGACAC	48	157	0	0	0	1
EIF2B4	CAACTGTGTGACTGACTG	121	90	0	0	0	0
EIF2B4	GTACTGTGTGACTGTGGT	291	247	0	0	0	0
EIF2B4	CATGCACAGTGTGTACCA	341	411	0	0	0	0
EIF2B4	TGTGCACAGTGTGTACAC	13	8	0	0	0	0
EIF2B5	CATGGTGTGTACCACATG	116	97	1	0	0	0
EIF2B5	GTTGGTGTGTACCAACGT	116	124	0	0	0	0
EIF2B5	CATGGTGTGTACCAACCA	119	131	0	1	0	0
EIF2B5	TGTGGTGTGTACCAACAC	23	39	0	0	0	0
EIF2B5	ACTGGTGTGTACCAACTG	354	629	0	0	0	0
EIF2S1	TGTGGTACCAACCAGTTG	1016	672	0	0	1	1
EIF2S1	CATGGTTGACACTGACCA	269	291	1	0	1	0
EIF2S1	ACTGGTACCAACCAGTCA	307	68	0	0	0	0
EIF2S1	GTTGGTACCAACCAGTAC	6	11	0	0	0	0
EIF2S1	ACTGGTACCAACCACAGT	111	129	0	0	0	0
EIF2S2	ACACTGTGGTTGACACAC	2	24	0	0	0	0
EIF2S2	CAACTGTGGTTGACACTG	95	223	0	2	0	0
EIF2S2	GTACTGTGGTTGACTGGT	45	21	0	0	0	0
EIF2S2	CAACTGTGGTTGACTGCA	63	38	1	0	0	0
EIF2S2	TGACTGTGGTTGACTGAC	17	7	0	0	0	0
EIF2S3	TGTGGTCACATGTGCAAC	254	81	0	1	0	1
EIF2S3	ACTGGTCACATGTGCATG	196	216	1	0	0	0
EIF2S3	CATGGTCACATGTGACGT	137	127	0	0	0	0
EIF2S3	ACTGGTCACATGTGACCA	35	33	0	0	0	0
EIF2S3	GTTGGTCACATGTGACAC	24	46	0	0	0	0
EIF3A	CATGTGACTGGTACGTGT	80	99	0	16	7	0
EIF3A	TGTGTGACACACCAGTAC	15	8	0	0	0	0
EIF3A	ACTGTGACACACCAGTTG	176	228	0	0	0	0
EIF3A	CATGTGACACACCACAGT	51	32	0	0	0	0

EIF3A	TGTGTGACACACCACATG	146	53	0	0	0	0
EIF3A	ACTGTGACACACCAACGT	148	183	0	0	0	0
EIF3A	TGTGTGACACACCAACCA	182	119	0	0	0	0
EIF3A	TGTGTGACACACCATGGT	498	896	0	1	0	1
EIF3A	GTTGTGACACACCATGCA	404	263	0	0	0	1
EIF3A	ACTGTGACACACCATGAC	147	143	1	1	0	0
EIF3A	GTTGTGACACACACGTCA	54	57	0	0	0	0
EIF3A	ACTGTGACACACACGTAC	0	2	0	0	0	0
EIF3A	CATGTGACACACACGTTG	54	56	0	0	0	0
EIF3A	GTTGTGACTGGTCATGAC	1	3	0	0	0	0
EIF3A	TGTGTGACTGGTCATGTG	381	75	2	1	0	0
EIF3A	TGTGTGACTGGTTGGTAC	25	36	0	0	0	0
EIF3A	ACTGTGACTGGTTGGTTG	106	232	0	0	0	0
EIF3A	CATGTGACTGGTTGCAGT	159	482	1	0	0	0
EIF3A	TGTGTGACTGTGTGTGTG	158	185	0	0	0	0
EIF3A	ACTGTGTGGTGTGTGTGT	424	579	0	0	0	0
EIF3A	TGTGTGTGGTGTGTGTCA	281	368	0	1	0	0
EIF3H	CAACTGTGGTACGTACCA	573	410	1	0	1	0
EIF3H	GTACTGTGGTACGTCACA	44	59	0	0	0	0
EIF3H	ACACTGTGGTACGTCAAC	20	13	0	0	0	0
EIF3H	CAACTGTGGTACGTCATG	194	211	0	0	0	0
EIF3H	GTACTGTGGTACGTACGT	251	705	0	0	0	0
EIF4A1	CAACACACACTGGTACCA	237	383	1	0	0	0
EIF4A1	TGACACACACTGGTACAC	30	19	0	0	0	0
EIF4A1	ACACACACACTGGTACTG	554	463	1	0	0	0
EIF4A1	CAACACACACTGGTTGGT	334	696	0	0	0	0
EIF4A1	ACACACACACTGGTTGCA	215	197	0	0	0	0
EIF4A2	TGACACACACCAACGTTG	1404	557	1	0	2	0
EIF4A2	CAACACACACCAACGTGT	445	279	0	0	1	0
EIF4A2	ACACACACACCAACGTCA	227	246	0	0	0	0
EIF4A2	GTACACACACCAACGTAC	60	69	0	0	0	3
EIF4A2	ACACACACACCAACCAGT	108	151	0	0	0	0
EIF4B	ACACTGACGTGTTGTGTG	80	104	0	0	0	0
EIF4B	TGACTGACGTCAGTGTGT	310	306	0	0	0	0
EIF4B	GTACTGACGTCAGTGTCA	392	338	0	0	0	1
EIF4B	ACACTGACGTCAGTGTAC	13	12	0	0	0	0
EIF4B	GTTGGTTGTGCACAACCA	95	209	0	0	0	0
EIF4E	GTACTGACGTGTTGCATG	273	271	0	0	0	0
EIF4E	TGACTGACGTGTTGACGT	535	316	0	2	0	6
EIF4E	GTACTGACGTGTTGACCA	502	357	0	0	0	0
EIF4E	ACACTGACGTGTTGACAC	9	28	0	0	0	0
EIF4E	CAACTGACGTGTTGACTG	253	239	0	0	0	0
EIF4E2	GTTGGTACCAGTTGCACA	71	119	0	0	0	0
EIF4E2	ACTGGTACCAGTTGCAAC	11	10	0	0	0	0
EIF4E2	CATGGTACCAGTTGCATG	210	269	1	0	0	0
EIF4E2	GTTGGTACCAGTTGACGT	419	1022	1	1	0	0
EIF4E2	GTTGGTACTGTGACTGAC	36	49	0	0	0	0
EIF4EBP1	ACACCATGACGTTGCATG	41	49	0	0	1	0
EIF4EBP1	GTACCATGACGTTGCAGT	178	647	2	0	0	1
EIF4EBP1	CAACCATGACGTTGCACA	15	20	0	0	0	0
EIF4EBP1	TGACCATGACGTTGCAAC	62	25	0	0	0	0
EIF4EBP1	CAACCATGACGTTGACGT	58	92	0	0	0	0
EIF4EBP2	TGTGGTGTTGTGCAACGT	394	441	1	2	0	0
EIF4EBP2	GTTGGTGTTGTGCAACCA	160	219	0	1	0	0
EIF4EBP2	ACTGGTGTTGTGCAACAC	60	41	0	0	0	0
EIF4EBP2	CATGGTGTTGTGCAACTG	444	578	1	0	0	0
EIF4EBP2	GTTGCAGTCAACCATGAC	56	80	0	0	0	0
EIF4EBP2	ACTGCATGCAGTGTCAGT	19	23	0	0	0	0
EIF4G1	TGACTGCATGCAGTACCA	90	52	1	0	0	0
EIF4G1	CAACTGCATGCAGTACAC	58	102	0	0	0	0
EIF4G1	GTACTGCATGCAGTACTG	673	639	0	0	0	0
EIF4G1	TGACTGCATGCAGTTGGT	130	230	0	1	0	0
EIF4G1	ACTGCAGTGTCAACACTG	482	509	1	0	0	0
EIF4G2	GTTGGTACGTGTTGACCA	307	373	0	0	0	0
EIF4G2	ACTGGTACGTGTTGACAC	35	17	0	0	0	0
EIF4G2	CATGGTACGTGTTGACTG	221	147	0	0	0	1
EIF4G2	GTTGGTACGTGTTGTGGT	520	985	1	0	0	0
EIF4G2	CATGGTACGTGTTGTGCA	174	87	1	0	0	0
EIF4G3	GTTGGTCATGACACCAAC	25	18	0	0	0	0
EIF4G3	TGTGGTCATGACACCATG	55	20	0	0	0	4
EIF4G3	ACTGGTCATGACACACGT	286	293	27	441	0	0
EIF4G3	TGTGGTCATGACACACCA	222	210	1	0	0	2
EIF4G3	ACTGGTTGTGGTCAACTG	29	41	1	0	0	0
EIF5	CATGGTGTCACAACACAC	194	138	1	0	0	0
EIF5	GTTGGTGTCACAACACTG	255	312	0	0	0	0

EIF5	CATGGTCATGACACACAC	55	62	0	0	0	0
EIF5	GTTGGTCATGACACACTG	414	346	1	1	0	3
EIF5	TGTGGTCATGACACTGGT	186	154	0	0	0	0
EIF5A2	ACTGGTACGTCAACCAGT	136	106	1	0	0	0
EIF5A2	TGTGGTACGTCAACCACA	171	67	0	0	0	2
EIF5A2	CATGGTACGTCAACCAAC	12	17	0	0	0	0
EIF5A2	GTTGGTACGTCAACCATG	357	206	1	1	0	0
EIF5A2	GTTGCAGTCACAGTCATG	136	394	0	0	0	0
ELA1	ACCATGACTGTGGTGTGT	511	810	2	1	1	2
ELA1	GTCATGACTGACTGTGAC	31	22	0	0	0	0
ELA1	TGCATGACTGACTGTGTG	164	192	0	0	0	0
ELA1	TGCATGACTGTGGTGTCA	360	353	0	0	0	1
ELA1	GTTGCAGTACGTTGTGGT	345	321	0	0	0	0
ELA2	GTCATGACTGTGGTGTTG	718	1582	6	5	4	6750
ELA2	GTCATGACTGTGGTCACA	171	335	0	0	1	0
ELA2	CACATGACTGTGGTGTAC	353	229	0	0	0	1
ELA2	TGCATGACTGTGGTCAGT	325	370	0	0	0	2
ELA2	CATGCAGTACGTTGTGCA	471	544	2	0	0	4
ELA2A	TGACACCAGTCACATGTG	834	281	0	1	1	0
ELA2A	ACACACCAGTCACATGCA	371	418	3	0	0	0
ELA2A	GTACACCAGTCACATGAC	163	180	4	1	0	0
ELA2A	CAACACCAGTCAACGTGT	428	456	0	0	0	0
ELA2A	TGTGCAGTTGGTTGGTAC	118	82	0	1	0	0
ELA2B	ACTGACGTTGACACCAGT	283	126	0	2	1	0
ELA2B	ACACACACCAACTGGTTG	335	266	3	1	0	0
ELA2B	CAACACACCAACTGCAGT	452	820	0	2	0	21
ELA2B	ACACACACCAACTGCACA	224	194	0	0	0	0
ELA2B	GTACACACCAACTGCAAC	18	42	0	0	0	0
ELA3A	ACACACCAGTCAGTCATG	524	400	2	0	0	0
ELA3A	CAACACCAGTCAGTACGT	845	690	1	0	0	0
ELA3A	ACACACCAGTCAGTACCA	59	100	0	0	0	3
ELA3A	GTACACCAGTCAGTACAC	86	127	0	0	0	0
ELA3A	GTTGCAGTTGCACAACCA	780	368	0	0	0	1
ELA3B	CAACTGTGGTACGTTGGT	392	382	1	1	2	5
ELA3B	TGACTGTGGTACGTACAC	6	4	0	0	0	0
ELA3B	ACACTGTGGTACGTACTG	308	368	1	0	0	0
ELA3B	ACACTGTGGTACGTTGCA	380	116	0	0	0	0
ELA3B	GTACTGTGGTACGTTGAC	25	17	0	0	0	0
ELAVL1	GTACGTACACCAGTTGGT	405	461	0	0	0	2
ELAVL1	CAACGTACACCAGTTGCA	29	99	0	0	0	0
ELAVL1	TGACGTACACCAGTTGAC	23	8	0	0	0	0
ELAVL1	ACACGTACACCAGTTGTG	730	739	1	0	0	0
ELAVL1	GTACGTACACCACAGTGT	612	674	2	1	0	0
ELK1	TGACGTGTACCAGTTGCA	153	379	0	3	1	1
ELK1	ACACGTGTACCAGTTGGT	849	638	1	0	0	0
ELK1	CAACGTGTACCAGTTGAC	81	81	1	0	0	0
ELK1	GTACGTGTACCAGTTGTG	190	246	0	0	0	0
ELK1	ACACGTGTACCACAGTGT	440	543	2	0	0	0
ELK1	ACTGCACATGACGTTGTG	638	396	0	1	0	0
ELK3	ACACGTCATGCATGTGAC	65	56	3	0	2	0
ELK3	GTACGTCATGCATGACTG	436	335	3	0	0	2
ELK3	TGACGTCATGCATGTGGT	310	472	0	0	0	0
ELK3	GTACGTCATGCATGTGCA	260	226	0	0	0	0
ELK3	CAACGTCATGCATGTGTG	295	296	0	3	0	0
ELK4	CAACGTCATGACGTGTGT	810	1334	0	2	1	0
ELK4	ACACGTCATGACGTGTCA	58	53	0	0	0	0
ELK4	GTACGTCATGACGTGTAC	6	9	0	0	0	0
ELK4	TGACGTCATGACGTGTTG	12	44	0	0	0	0
ELK4	ACACGTCATGACGTCAGT	103	131	0	0	0	0
ELOVL2	GTCATGTGCATGACACTG	185	211	0	206	6	2
ELOVL2	TGCATGTGCATGACACCA	47	97	1	0	0	0
ELOVL2	CACATGTGCATGACACAC	36	41	0	0	0	0
ELOVL2	TGCATGTGCATGACTGGT	93	166	0	0	0	0
ELOVL2	GTCATGTGCATGACTGCA	95	131	0	0	0	1
ELOVL5	ACTGGTACCAACGTTGCA	159	348	0	0	1	0
ELOVL5	CATGGTACCAACGTTGGT	193	302	0	0	0	0
ELOVL5	GTTGGTACCAACGTTGAC	47	20	0	0	0	0
ELOVL5	TGTGGTACCAACGTTGTG	288	320	0	1	0	0
ELOVL5	CATGGTACCAACCAGTGT	466	529	0	0	0	0
ELOVL6	ACTGGTTGCAACGTACCA	141	302	0	1	1	2
ELOVL6	GTTGGTACTGCACAGTGT	534	658	2	0	0	0
ELOVL6	TGTGGTTGCAACGTCAAC	2	0	0	0	0	0
ELOVL6	ACTGGTTGCAACGTCATG	223	364	1	1	0	1
ELOVL6	CATGGTTGCAACGTACGT	265	495	0	0	0	0
ELOVL6	CATGCACATGTGGTTGGT	377	339	1	1	0	0

ELSPBP1	GTTGGTTGCAACGTACAC	9	30	0	0	0	0
ELSPBP1	TGTGGTTGCAACGTACTG	620	606	1	0	0	2
ELSPBP1	TGTGACACGTTGACCAGT	165	479	6	0	0	0
ELSPBP1	GTTGACACGTTGACCACA	146	74	0	0	0	0
ELSPBP1	ACTGACACGTTGACCAAC	140	24	0	0	0	0
EME1	ACACACCATGCACAGTTG	628	689	0	0	0	8
EME1	CAACACCATGCACACAGT	284	632	1	0	0	1
EME1	ACACACCATGCACACACA	212	226	2	0	0	0
EME1	GTACACCATGCACACAAC	24	14	0	0	0	0
EME1	CATGCAACACTGCATGAC	10	15	0	1	0	0
EME1	GTTGCAACACTGCATGTG	376	324	1	1	0	0
EME2	TGACACCATGCACACATG	128	128	2	0	0	0
EME2	ACTGCAGTTGCAACACGT	279	205	0	0	0	0
EME2	CATGACGTTGACCAGTAC	24	6	0	0	0	0
EME2	GTTGACGTTGACCAGTTG	62	64	0	0	0	0
EME2	TGTGACGTTGACCACAGT	79	84	0	0	0	0
EMP1	TGTGGTGTTGTGACTGGT	78	123	0	0	0	0
EMP1	GTTGGTGTTGTGACTGCA	768	414	1	0	0	0
EMP1	ACTGGTGTTGTGACTGAC	77	91	0	0	0	0
EMP1	CATGGTGTTGTGACTGTG	187	351	0	1	0	0
EMP1	TGTGGTGTTGTGTGGTGT	402	387	0	0	0	0
EMP1	GTTGCACAGTACACGTAC	76	75	0	0	0	0
EMR1	GTACGTCAGTCAGTTGGT	335	262	0	0	1	1
EMR1	GTACGTCAGTCAGTACCA	214	289	0	0	0	0
EMR1	ACACGTCAGTCAGTACAC	21	23	0	0	0	0
EMR1	CAACGTCAGTCAGTACTG	239	295	1	0	0	0
EMR1	CAACGTCAGTCAGTTGCA	82	119	0	0	0	0
EMR2	ACACGTCAGTCAGTTGTG	831	947	1	0	1	0
EMR2	TGACGTCAGTCAGTTGAC	45	45	0	0	0	0
EMR2	GTACGTCAGTCACAGTGT	815	776	0	0	0	0
EMR2	CAACGTCAGTCACAGTCA	184	236	0	1	0	0
EMR2	TGACGTCAGTCACAGTAC	37	30	0	0	0	0
EMR2	GTTGCAACTGGTACCAGT	82	115	0	0	0	0
EMR3	GTACGTCAGTCAACGTCA	321	415	3	0	1	0
EMR3	GTACGTCAGTCACATGCA	324	836	6	1	0	0
EMR3	ACACGTCAGTCACATGAC	105	131	0	0	0	1
EMR3	CAACGTCAGTCACATGTG	968	1189	1	0	0	0
EMR3	TGACGTCAGTCAACGTGT	642	178	0	0	0	0
EMX2	TGACGTCATGACGTCACA	10	8	0	0	0	0
EMX2	CAACGTCATGACGTCAAC	24	23	0	0	0	0
EMX2	GTACGTCATGACGTCATG	81	105	0	0	0	0
EMX2	TGACGTCATGACGTACGT	133	381	0	1	0	0
EMX2	GTACGTCATGACGTACCA	51	61	0	6	0	0
EMX2	TGTGCATGCAGTGTCACA	46	48	0	0	0	0
EN1	CAACGTCATGACGTTGCA	56	82	0	0	1	0
EN1	ACACGTCATGACGTACAC	33	21	0	0	0	0
EN1	CAACGTCATGACGTACTG	233	297	0	0	0	1
EN1	GTACGTCATGACGTTGGT	454	700	0	0	0	1
EN1	TGACGTCATGACGTTGAC	92	135	0	0	0	0
ENDOG	GTACCATGGTTGACACAC	25	30	0	0	0	0
ENDOG	TGACCATGGTTGACACTG	323	449	0	0	0	0
ENDOG	GTTGCAGTTGGTGTCAGT	224	160	1	0	0	0
ENDOG	TGTGCACAGTGTTGCAAC	11	17	0	0	0	0
ENDOG	TGTGACGTACACCATGCA	229	259	0	0	0	1
ENG	GTACTGTGTGCAACGTGT	261	273	2	1	0	0
ENG	CAACTGTGTGCAACGTCA	97	169	0	0	0	0
ENG	TGACTGTGTGCAACGTAC	151	172	1	0	0	0
ENG	ACACTGTGTGCAACGTTG	66	63	0	0	0	0
ENG	CAACTGTGTGCAACCAGT	41	55	0	0	0	0
ENG	TGTGCACACACATGACTG	587	788	2	1	0	0
ENO1	TGACCAGTACACACGTCA	136	110	0	0	0	1
ENO1	CAACCAGTACACACGTAC	9	17	0	0	0	0
ENO1	GTACCAGTACACACGTTG	149	217	0	0	0	0
ENO1	TGACCAGTACACACCAGT	415	373	4	0	0	0
ENO1	GTACCAGTACACACCACA	164	161	1	0	0	0
ENO1	ACTGCAACGTACACGTGT	105	180	0	0	0	0
ENO2	CATGGTACGTACACGTTG	886	1126	732	40592	1492328	740
ENO2	ACTGGTACGTACACGTAC	122	86	0	0	1	0
ENO2	ACTGGTGTACCACAACCA	45	63	0	0	0	0
ENO2	TGTGGTACGTACACGTGT	233	131	0	1	0	0
ENO2	GTTGGTACGTACACGTCA	91	105	0	1	0	0
ENO2	TGTGCAACGTACACGTCA	132	157	0	0	0	0
ENO3	ACTGGTCAGTCATGCATG	304	200	1	1	0	0
ENO3	CATGGTCAGTCATGACGT	283	179	0	0	0	0
ENO3	ACTGGTCAGTCATGACCA	125	143	0	0	0	0

ENO3	GTTGGTCAGTCATGACAC	44	80	1	2	0	3
ENO3	TGTGGTCAGTCATGACTG	651	929	0	2	0	0
ENO3	TGTGCAACGTACACCAGT	211	191	1	0	0	35
ENPEP	CAACACACACGTACACCA	157	113	0	1	0	0
ENPEP	TGACACACACGTACACAC	60	43	0	0	0	0
ENPEP	ACACACACACGTACACTG	244	339	2	0	0	0
ENPEP	CAACACACACGTACTGGT	310	241	0	0	0	0
ENPEP	TGTGACACACGTTGCAGT	244	77	0	0	0	0
ENPP1	CACATGACCAACTGGTGT	157	162	0	0	0	0
ENPP1	ACCATGACCAACTGGTCA	48	66	0	0	0	0
ENPP1	GTCATGACCAACTGGTAC	68	166	0	0	0	0
ENPP1	TGCATGACCAACTGGTTG	132	130	0	1	0	0
ENPP1	ACCATGACCAACTGCAGT	160	219	0	0	0	1
ENPP1	ACTGCACATGTGCACAGT	208	350	2	0	0	0
ENPP2	ACACACCAACCACATGTG	855	872	47	7	1	1
ENPP2	TGACACCAACCACATGAC	115	94	0	0	0	0
ENPP2	GTACACCAACCAACGTGT	746	319	0	2	0	0
ENPP2	CAACACCAACCAACGTCA	516	437	0	1	0	0
ENPP2	TGACACCAACCAACGTAC	79	84	0	0	0	14
ENPP3	GTACACCAACCAACCAAC	149	178	0	0	1	0
ENPP3	ACACACCAACCAACGTTG	835	1007	2	0	0	0
ENPP3	CAACACCAACCAACCAGT	380	508	0	0	0	13
ENPP3	ACACACCAACCAACCACA	123	99	0	0	0	1
ENPP3	TGACACCAACCAACCATG	212	245	0	0	0	0
ENPP6	TGACGTGTCACAACTGCA	527	343	0	318	1	1
ENPP6	CAACGTGTCACAACTGAC	104	100	1	0	0	0
ENPP6	GTACGTGTCACAACTGTG	127	66	0	0	0	0
ENPP6	ACACGTGTCACATGGTGT	362	335	1	0	0	1
ENPP6	CATGCAGTACCAACGTGT	1015	1295	1	1	0	0
ENPP7	ACCATGACCATGCACACA	17	28	0	0	0	0
ENPP7	GTCATGACCATGCACAAC	57	47	0	0	0	0
ENPP7	TGCATGACCATGCACATG	236	161	1	0	0	0
ENPP7	ACCATGACCATGCAACGT	199	456	0	0	0	0
ENPP7	ACTGACGTGTACTGACTG	53	72	0	0	0	0
ENTPD1	GTACACCATGTGACCACA	171	195	0	0	1	0
ENTPD1	GTACACCATGTGACGTTG	91	89	0	0	0	0
ENTPD1	TGACACCATGTGACCAGT	239	165	0	0	0	1
ENTPD1	ACACACCATGTGACCAAC	36	21	0	0	0	0
ENTPD1	TGTGGTTGACCATGGTAC	104	101	0	0	0	0
ENTPD3	TGACACACGTGTCACATG	353	370	1	2	1	0
ENTPD3	CAACACACGTGTCACAGT	55	90	0	1	0	4
ENTPD3	ACACACACGTGTCACACA	98	271	0	0	0	0
ENTPD3	GTACACACGTGTCACAAC	27	50	0	0	0	0
ENTPD3	ACACTGTGACACACGTAC	58	81	0	0	0	0
ENTPD3	CATGCACACACAACTGTG	118	133	0	0	0	0
ENTPD4	GTACACACCAACTGACTG	330	196	0	2	0	6
ENTPD4	TGACACACCAACTGTGGT	746	842	1	0	0	2
ENTPD4	GTACACACCAACTGTGCA	218	202	0	0	0	1
ENTPD4	ACACACACCAACTGTGAC	50	71	0	0	0	0
ENTPD4	CATGGTTGTGCAACGTCA	112	108	1	0	0	0
ENTPD5	GTACACACGTGTACGTCA	126	114	1	1	0	0
ENTPD5	ACACACACGTGTACGTAC	141	48	0	0	0	0
ENTPD5	CAACACACGTGTACGTTG	455	480	1	1	0	12
ENTPD5	GTACACACGTGTACCAGT	45	39	0	0	0	0
ENTPD5	CAACACACGTGTACCACA	103	96	0	0	0	0
ENTPD5	TGTGCACACACATGGTGT	321	342	0	0	0	0
ENTPD6	ACACACCATGTGTGTGTG	163	135	1	0	0	0
ENTPD6	CAACACACGTGTGTGTGT	99	131	0	0	0	0
ENTPD6	ACACACACGTGTGTGTCA	131	116	0	0	0	0
ENTPD6	GTACACACGTGTGTGTAC	12	31	0	0	0	0
ENTPD6	GTTGCAGTCATGGTCAAC	70	38	0	0	0	0
ENTPD6	ACTGCACACACAACTGAC	32	57	0	0	0	0
ENTPD7	GTACACACACACACACAC	52	35	0	0	0	0
ENTPD7	TGACACACACACACACTG	391	474	0	1	0	0
ENTPD7	ACACACACACACACTGGT	80	57	0	0	0	0
ENTPD7	TGACACACACACACTGCA	469	338	0	0	0	0
ENTPD7	ACTGCAGTTGCAACGTTG	256	222	0	0	0	0
ENTPD8	ACACACCATGACACACAC	44	69	0	0	0	0
ENTPD8	CAACACCATGACACACTG	465	980	2	1	0	2
ENTPD8	GTACACCATGACACTGGT	161	160	0	0	0	0
ENTPD8	TGTGCAGTTGGTTGTGGT	512	807	0	0	0	0
ENTPD8	GTTGCAGTTGGTTGTGCA	148	172	0	0	0	1
EOMES	TGACGTCAACACCAGTTG	542	513	0	1	1	0
EOMES	ACACGTCAACACCAGTCA	142	140	0	0	0	0
EOMES	GTACGTCAACACCAGTAC	112	30	0	0	0	0

EOMES	ACACGTCAACACCACAGT	442	428	0	0	0	1
EOMES	TGTGCAGTACACGTTGAC	92	70	0	0	0	0
EOMES	TGTGCAACACTGGTACCA	177	83	0	0	0	0
EP300	CAACCATGCAACGTGTGT	506	403	1	0	2	0
EP300	GTACCATGCACATGTGCA	141	125	0	0	0	0
EP300	ACACCATGCACATGTGAC	24	26	0	0	0	0
EP300	CAACCATGCACATGTGTG	343	355	1	0	0	0
EP300	CATGGTTGTGGTGTACGT	77	37	0	0	0	0
EPAS1	CACATGTGGTGTCATGGT	526	895	1	2	1	2
EPAS1	GTCATGTGGTGTCAACGT	143	232	0	0	0	0
EPAS1	CACATGTGGTGTCAACCA	210	308	0	0	0	0
EPAS1	TGCATGTGGTGTCAACAC	9	13	0	0	0	0
EPAS1	ACCATGTGGTGTCAACTG	339	695	1	1	0	0
EPB49	ACTGGTACACTGGTGTCA	179	311	1	0	1	2
EPB49	GTTGGTACACTGGTGTAC	193	82	0	0	0	0
EPB49	TGTGGTACACTGGTGTTG	660	715	0	1	0	0
EPB49	ACTGGTACACTGGTCAGT	169	219	2	0	0	0
EPB49	TGTGGTACACTGGTCACA	214	97	0	0	0	0
EPB49	TGTGCACAGTACACGTTG	156	164	0	0	0	1
EPHA2	TGACGTGTGTCATGACGT	47	46	0	0	0	0
EPHA2	GTACGTGTGTCATGACCA	357	526	3	0	0	1
EPHA2	ACACGTGTGTCATGACAC	50	21	0	0	0	0
EPHA2	CAACGTGTGTCATGACTG	404	650	1	0	0	60
EPHA2	GTACGTGTGTCATGTGGT	489	464	0	0	0	0
EPHA4	GTTGGTTGACACCATGCA	97	145	1	0	1	0
EPHA4	CATGGTTGACACCAACAC	63	121	0	0	0	0
EPHA4	GTTGGTTGACACCAACTG	240	231	0	0	0	0
EPHA4	TGTGGTTGACACCATGGT	430	405	2	0	0	0
EPHA4	ACTGGTTGACACCATGAC	98	163	0	0	0	0
EPHB1	GTCATGCAACTGTGTGTG	301	242	6	2	3	0
EPHB1	CACATGCAACTGTGTGAC	81	31	0	0	0	0
EPHB1	TGCATGCATGGTGTGTGT	372	245	0	0	0	1
EPHB1	GTCATGCATGGTGTGTCA	264	264	1	0	0	2
EPHB1	ACCATGCATGGTGTGTAC	116	10	0	0	0	0
EPHB2	CAACGTGTGTCATGTGCA	262	284	2	0	0	0
EPHB2	TGACGTGTGTCATGTGAC	18	21	0	0	0	0
EPHB2	ACACGTGTGTCATGTGTG	66	34	0	0	0	0
EPHB2	ACACGTGTGTACGTGTGT	379	393	0	0	0	1
EPHB2	TGACGTGTGTACGTGTCA	161	133	1	0	0	0
EPHB3	CAACGTGTGTACGTGTAC	45	50	0	0	0	0
EPHB3	GTACGTGTGTACGTGTTG	1011	760	3	0	0	3
EPHB3	TGACGTGTGTACGTCAGT	41	55	0	0	0	0
EPHB3	GTACGTGTGTACGTCACA	194	124	0	1	0	0
EPHB3	GTTGCAGTGTGTACACAC	23	27	0	0	0	0
EPHB4	GTTGCAGTACGTCATGTG	510	677	0	0	1	2
EPHB4	ACCATGACGTCATGACTG	223	364	1	0	0	0
EPHB4	CACATGACGTCATGTGGT	74	64	0	0	0	0
EPHB4	ACCATGACGTCATGTGCA	27	17	0	0	0	0
EPHB4	GTCATGACGTCATGTGAC	91	44	0	0	0	0
EPHX1	ACTGCACACACATGTGGT	765	1311	27	11679	13118	20
EPHX1	ACACACACGTGTTGGTGT	707	558	2	0	0	2
EPHX1	TGACACACGTGTTGGTCA	122	129	0	0	0	0
EPHX1	CAACACACGTGTTGGTAC	58	146	0	0	0	0
EPHX1	GTACACACGTGTTGGTTG	281	233	0	0	0	0
EPHX1	TGACACACGTGTTGCAGT	133	167	0	0	0	0
EPHX2	ACACACACGTCAGTACGT	205	303	0	0	0	0
EPHX2	TGACACACGTCAGTACCA	218	293	40	0	0	0
EPHX2	CAACACACGTCAGTACAC	48	63	0	0	0	0
EPHX2	GTACACACGTCAGTACTG	310	268	0	0	0	4
EPHX2	TGACACACGTCAGTTGGT	162	188	0	0	0	0
EPN1	GTACTGTGCACATGGTTG	721	1267	3	1	1	0
EPN1	ACACTGTGCACATGGTGT	517	785	0	1	0	1
EPN1	TGACTGTGCACATGGTCA	313	157	1	0	0	0
EPN1	CAACTGTGCACATGGTAC	63	67	0	0	0	0
EPN1	CATGCAGTTGTGTGCATG	16	6	0	0	0	0
EPO	TGACTGGTTGGTCAACTG	306	408	2	0	0	0
EPO	ACACTGGTTGGTCATGGT	433	633	0	0	0	3
EPO	TGACTGGTTGGTCATGCA	152	225	0	1	0	0
EPO	CAACTGGTTGGTCATGAC	50	72	0	0	0	0
EPO	GTTGCAGTTGACGTGTGT	1064	810	1	0	0	11
EPOR	ACACTGGTACGTTGGTGT	418	546	0	0	0	6
EPOR	TGACTGGTACGTTGGTCA	415	525	1	0	0	0
EPOR	CAACTGGTACGTTGGTAC	44	49	0	1	0	0
EPOR	GTACTGGTACGTTGGTTG	400	488	0	0	0	1
EPOR	TGTGCAGTTGCATGTGCA	234	345	1	0	0	0

EPOR	TGTGCACACACATGTGCA	26	27	0	0	0	0
EPPK1	CATGGTGTTGCAGTACGT	537	286	0	0	1	0
EPPK1	ACTGGTGTTGCAGTCATG	87	141	0	1	0	0
EPPK1	ACTGGTGTTGCAGTACCA	280	286	1	0	0	0
EPPK1	GTTGGTGTTGCAGTACAC	25	50	0	0	0	0
EPPK1	TGTGGTGTTGCAGTACTG	400	242	0	1	0	0
EPPK1	GTTGCAACGTGTGTTGTG	280	228	0	1	0	0
EPRS	CAACACGTCATGACCAGT	199	218	1	1	5	9
EPRS	CAACACGTCATGACGTCA	209	125	0	0	0	0
EPRS	TGACACGTCATGACGTAC	7	14	0	0	0	0
EPRS	ACACACGTCATGACGTTG	277	533	0	0	0	0
EPRS	CATGCAGTCAGTTGTGAC	31	64	0	0	0	0
EPS15	GTACGTGTTGGTGTGTCA	127	134	1	0	0	0
EPS15	ACACGTGTTGGTGTGTAC	37	9	0	0	0	0
EPS15	CAACGTGTTGGTGTGTTG	650	244	2	0	0	0
EPS15	GTACGTGTTGGTGTCAGT	136	76	0	0	0	0
EPS15	CAACGTGTTGGTGTCACA	151	121	0	0	0	0
EPS8	CAACTGCATGGTGTACTG	235	294	1	0	0	0
EPS8	GTACTGCATGGTGTTGGT	303	198	0	0	0	0
EPS8	CAACTGCATGGTGTTGCA	154	26	0	0	0	0
EPS8	TGACTGCATGGTGTTGAC	86	91	1	0	0	0
EPS8	ACACTGCATGGTGTTGTG	160	472	0	0	0	0
EPX	ACACACGTTGGTTGACTG	421	853	0	0	3	1
EPX	CAACACGTTGGTTGTGGT	228	138	1	1	0	1
EPX	ACACACGTTGGTTGTGCA	156	124	0	0	0	0
EPX	GTACACGTTGGTTGTGAC	37	43	0	0	0	0
EPX	TGACACGTTGGTTGTGTG	178	299	0	1	0	0
ERAF	TGTGGTCAGTGTTGGTAC	37	152	0	0	0	0
ERAF	ACTGGTCAGTGTTGGTTG	133	218	0	0	0	0
ERAF	CATGGTCAGTGTTGCAGT	283	429	0	0	0	0
ERAF	CATGACGTTGCAACCACA	69	40	0	0	0	0
ERAF	TGTGACGTTGCAACCAAC	56	57	0	0	0	0
ERAP1	ACACTGCAACGTACCACA	85	92	0	0	1	0
ERAP1	GTACCAGTTGTGCAGTCA	315	459	0	2	0	1
ERAP1	GTACTGCAACGTACCAAC	16	31	0	0	0	0
ERAP1	TGACTGCAACGTACCATG	46	182	0	0	0	0
ERAP1	ACTGACCATGACTGACAC	36	24	0	0	0	0
ERBB2	ACACGTTGTGACGTTGGT	305	208	1	0	0	0
ERBB2	TGACCATGCACATGACCA	48	76	0	0	0	0
ERBB2	CAACCATGCACATGACAC	79	91	0	0	0	0
ERBB2	GTACCATGCACATGACTG	192	136	0	1	0	1
ERBB2	TGACCATGCACATGTGGT	272	305	1	0	0	1
ERBB3	CACATGCAACACGTGTGT	31	37	0	0	0	0
ERBB3	ACCATGCAACACGTGTCA	92	281	0	0	0	0
ERBB3	GTCATGCAACACGTGTAC	29	30	0	0	0	0
ERBB3	TGCATGCAACACGTGTTG	135	153	0	0	0	0
ERBB3	ACCATGCAACACGTCAGT	65	96	0	1	0	0
ERBB4	TGCATGCATGTGTGGTCA	275	425	0	0	1	0
ERBB4	GTCATGCATGTGACTGTG	880	499	4	11	0	0
ERBB4	ACCATGCATGTGTGGTGT	305	570	1	0	0	1
ERBB4	CACATGCATGTGTGGTAC	41	35	0	0	0	0
ERBB4	GTCATGCATGTGTGGTTG	171	234	0	0	0	0
ERBB4	TGTGCACACATGTGCAGT	139	164	1	0	0	0
ERCC1	TGACACCATGCAACGTGT	465	384	0	0	1	0
ERCC1	GTACACCATGCACATGCA	897	449	0	1	0	0
ERCC1	ACACACCATGCACATGAC	52	67	0	0	0	0
ERCC1	CAACACCATGCACATGTG	1062	475	1	3	0	0
ERCC1	GTACACCATGCAACGTCA	145	245	1	0	0	0
ERCC2	ACACGTTGACTGTGACTG	164	186	0	0	0	0
ERCC2	CAACGTTGACTGTGTGGT	365	293	0	0	0	2
ERCC2	ACACGTTGACTGTGTGCA	110	160	0	0	0	1
ERCC2	GTACGTTGACTGTGTGAC	188	45	0	0	0	0
ERCC2	ACTGCAGTGTACTGTGGT	192	185	0	0	0	0
ERCC3	GTACGTTGTGGTCACATG	213	227	0	0	0	0
ERCC3	TGACGTTGTGGTCAACGT	64	97	0	0	0	1
ERCC3	GTACGTTGTGGTCAACCA	416	253	0	0	0	2
ERCC3	ACACGTTGTGGTCAACAC	53	52	0	0	0	1
ERCC3	CAACGTTGTGGTCAACTG	172	805	0	0	0	0
ERCC3	CATGCACACACATGTGAC	45	32	0	0	0	0
ERCC4	TGACTGTGACACGTACGT	441	565	0	0	2	1
ERCC4	GTACTGTGACACGTCATG	106	259	0	0	1	0
ERCC4	ACACTGTGACACGTACAC	69	78	4	0	1	0
ERCC4	GTACTGTGACACGTACCA	210	217	0	0	0	0
ERCC4	ACTGCAGTTGTGTGCAAC	17	60	1	0	0	0
ERCC4	GTTGCACACATGTGCACA	120	137	0	0	0	1

ERCC5	CAACACACGTACCACAAC	19	35	0	0	0	0
ERCC5	GTACACACGTACCACATG	276	127	0	0	0	0
ERCC5	TGACACACGTACCAACGT	180	637	0	1	0	0
ERCC5	GTACACACGTACCAACCA	176	147	205	63	0	1
ERCC5	ACACACACGTACCAACAC	30	24	0	0	0	0
EREG	TGACCATGCATGACCATG	68	240	0	0	0	0
EREG	ACACCATGCATGACACGT	191	222	0	0	0	0
EREG	TGACCATGCATGACACCA	168	205	0	0	0	0
EREG	CAACCATGCATGACACAC	267	128	1	0	0	0
EREG	ACTGCACAGTGTTGCATG	71	57	0	2	0	3
ERG	ACACGTCATGACGTTGTG	1047	810	1	0	0	0
ERG	GTACGTCATGACCAGTGT	478	533	1	1	0	0
ERG	CAACGTCATGACCAGTCA	368	352	0	1	0	0
ERG	TGACGTCATGACCAGTAC	81	106	0	0	0	0
ERG	TGTGACGTGTTGACTGGT	435	440	0	0	0	0
ERN1	CATGACGTCATGCAGTAC	50	37	0	1	0	0
ERN1	GTTGACGTCATGCAGTTG	767	926	0	0	0	0
ERN1	TGTGACGTCATGCACAGT	282	657	0	1	0	0
ERN1	GTTGACGTCATGCACACA	150	124	0	0	0	0
ERN1	ACTGACGTCATGCACAAC	35	37	0	0	0	0
ERN2	CAACGTTGACCAGTACCA	150	134	0	0	0	0
ERN2	TGACGTTGACCAGTACAC	48	44	0	0	0	0
ERN2	ACACGTTGACCAGTACTG	774	530	0	0	0	0
ERN2	CAACGTTGACCAGTTGGT	596	845	2	1	0	1
ERN2	TGTGGTCATGACCACACA	2	16	0	0	0	0
ESCO1	CACATGTGACCATGACCA	317	336	1	4	4	9
ESCO1	CACATGTGACCATGCATG	250	372	0	0	0	1
ESCO1	GTCATGTGACCATGACGT	81	83	0	0	0	0
ESCO1	TGCATGTGACCATGACAC	64	61	0	0	0	0
ESCO1	TGTGCAGTACCAGTTGCA	169	178	0	0	0	0
ESCO2	GTTGGTCAACGTGTTGAC	26	42	0	0	0	1
ESCO2	TGTGGTCAACGTGTTGTG	608	592	1	3	0	6
ESCO2	CATGGTCAACGTCAGTGT	461	501	0	0	0	0
ESCO2	ACTGGTCAACGTCAGTCA	758	835	1	1	0	0
ESCO2	GTTGGTCAACGTCAGTAC	19	15	0	0	0	3
ESD	TGACACCACATGGTTGCA	604	613	3	4	2	8
ESD	GTACACCACATGGTACAC	19	26	0	0	0	0
ESD	TGACACCACATGGTACTG	307	151	1	0	0	0
ESD	ACACACCACATGGTTGGT	96	418	0	0	0	0
ESD	GTTGCAGTTGGTACCATG	117	123	0	0	0	0
ESR1	ACCATGACACACTGTGGT	1688	1229	1	1	3	7
ESR1	GTCATGACACACTGACAC	28	14	0	0	0	0
ESR1	TGCATGACACACTGACTG	1751	1228	0	0	0	0
ESR1	TGCATGACACACTGTGCA	807	464	2	1	0	1
ESR1	ACTGCAGTACGTACCAAC	9	29	0	0	0	1
ESR2	GTCATGACACTGCACACA	179	308	0	0	0	0
ESR2	ACCATGACACTGCACAAC	34	32	0	0	0	1
ESR2	CACATGACACTGCACATG	211	124	0	0	0	0
ESR2	CATGACCATGACCATGAC	15	61	0	0	0	0
ESR2	GTTGACCATGACCATGTG	562	546	0	1	0	0
ESRRA	TGACGTTGTGGTTGGTGT	634	659	0	0	1	0
ESRRA	CAACGTTGTGGTTGGTTG	357	476	1	0	1	13
ESRRA	GTACGTTGTGGTTGGTCA	256	238	1	0	0	0
ESRRA	ACACGTTGTGGTTGGTAC	15	11	0	0	0	0
ESRRA	GTACGTTGTGGTTGCAGT	20	59	0	0	0	2
ESRRA	TGTGCAACCAGTACCAGT	129	183	0	0	0	0
ETFA	CAACTGACACGTTGTGTG	576	570	1782	1	1	1
ETFA	GTACTGACACGTTGACTG	332	162	0	0	0	2
ETFA	TGACTGACACGTTGTGGT	378	288	2	1	0	0
ETFA	GTACTGACACGTTGTGCA	382	274	0	0	0	0
ETFA	ACACTGACACGTTGTGAC	21	19	0	0	0	0
ETS1	GTCATGTGACACTGTGCA	40	40	2	0	0	0
ETS1	ACCATGTGACACTGTGAC	32	49	0	0	0	0
ETS1	CACATGTGACACTGTGTG	91	235	1	0	0	0
ETS1	GTCATGTGACTGGTGTGT	353	1098	278	0	0	0
ETS1	CACATGTGACTGGTGTCA	482	360	0	0	0	0
ETS2	TGACGTCATGACCACATG	774	272	1	2	1	0
ETS2	ACACGTCATGACCAGTTG	307	362	0	0	0	1
ETS2	CAACGTCATGACCACAGT	26	32	1	0	0	0
ETS2	ACACGTCATGACCACACA	81	142	0	0	0	0
ETS2	GTACGTCATGACCACAAC	64	50	0	13	0	0
ETV3	ACACGTCATGACCAACGT	808	251	1	0	1	1
ETV3	GTACGTCATGACCAACTG	1190	645	0	44	1	0
ETV3	TGACGTCATGACCAACCA	286	301	0	0	0	0
ETV3	CAACGTCATGACCAACAC	63	126	0	0	0	0

ETV3	TGACGTCATGACCATGGT	125	173	0	0	0	1
ETV3	CATGCAACCAACACCACA	123	188	0	0	0	0
ETV5	GTACGTCATGACCATGCA	118	242	0	0	0	2
ETV5	ACACGTCATGACCATGAC	93	99	0	0	0	0
ETV5	CAACGTCATGACCATGTG	883	357	1	2	0	0
ETV5	TGACGTCATGACACGTGT	492	601	0	0	0	0
ETV5	GTACGTCATGACACGTCA	424	449	1	0	0	0
ETV5	CATGCACACATGGTCAAC	35	40	0	2	0	4
ETV6	ACCATGTGGTCAGTGTGT	1009	1027	0	1	1	1
ETV6	TGCATGTGGTGTTGTGGT	303	171	0	0	0	0
ETV6	GTCATGTGGTGTTGTGCA	52	45	1	0	0	0
ETV6	ACCATGTGGTGTTGTGAC	29	58	0	0	0	1
ETV6	CACATGTGGTGTTGTGTG	482	191	1	0	0	0
ETV7	TGACGTACACGTCAGTTG	178	184	1	0	0	9
ETV7	ACACGTACACGTCACAGT	121	145	0	0	0	0
ETV7	TGACGTACACGTCACACA	89	79	0	0	0	0
ETV7	CAACGTACACGTCACAAC	22	15	0	0	0	0
ETV7	GTACGTACACGTCACATG	190	110	0	1	0	0
ETV7	CATGCATGCAGTGTCAAC	24	24	1	0	0	1
EXO1	TGACCATGCAGTTGCACA	140	173	0	0	0	0
EXO1	CAACCATGCAGTTGCAAC	25	30	0	0	0	0
EXO1	GTACCATGCAGTTGCATG	49	61	0	0	0	0
EXO1	TGACCATGCAGTTGACGT	484	395	0	0	0	0
EXO1	GTACCATGCAGTTGACCA	69	20	0	0	0	0
EXOC7	TGTGGTCACATGACCATG	121	215	0	0	0	0
EXOC7	ACTGGTCACATGACACGT	56	59	0	0	0	0
EXOC7	TGTGGTCACATGACACCA	170	215	0	0	0	0
EXOC7	CATGGTCACATGACACAC	41	39	0	0	0	0
EXOC7	GTTGGTCACATGACACTG	271	131	0	0	0	7
EXT1	GTACCATGCAACACTGTG	222	354	1	1	0	0
EXT1	ACACCATGCAACTGGTGT	304	358	1	0	0	0
EXT1	TGACCATGCAACTGGTCA	419	599	4	0	0	0
EXT1	CAACCATGCAACTGGTAC	28	11	0	0	0	0
EXT1	GTACCATGCAACTGGTTG	515	334	0	0	0	0
EXT2	GTACCATGCACAACGTAC	35	59	0	0	0	7
EXT2	TGACCATGCACAACGTTG	1001	379	0	0	0	1
EXT2	ACACCATGCACAACCAGT	1227	1742	7	0	0	1
EXT2	TGACCATGCACAACCACA	250	148	1	0	0	0
EXT2	CAACCATGCACAACCAAC	78	92	0	0	0	0
EXTL1	ACTGGTTGTGACTGGTCA	602	483	2	0	1	0
EXTL1	GTTGGTTGTGACTGGTAC	152	92	0	0	0	0
EXTL1	TGTGGTTGTGACTGGTTG	367	607	1	0	0	1
EXTL1	ACTGGTTGTGACTGCAGT	124	92	1	0	0	0
EXTL1	TGTGGTTGTGACTGCACA	95	119	0	0	0	0
EXTL2	CATGGTCATGTGGTACGT	378	344	0	0	1	5
EXTL2	CATGGTCATGTGGTCACA	90	108	0	0	0	0
EXTL2	TGTGGTCATGTGGTCAAC	6	6	0	0	0	0
EXTL2	ACTGGTCATGTGGTCATG	267	320	0	0	0	1
EXTL2	ACTGACCACATGGTTGAC	78	45	0	0	0	0
EXTL3	ACTGGTGTCAGTCATGCA	144	326	0	0	0	0
EXTL3	CATGGTCATGACGTCATG	193	189	0	0	0	0
EXTL3	GTTGGTCATGACGTACGT	173	118	0	0	0	0
EXTL3	CATGGTCATGACGTACCA	395	70	0	0	0	0
EXTL3	TGTGGTCATGACGTACAC	306	97	0	0	0	0
EZH1	TGCATGACCACATGACTG	144	313	0	1	0	0
EZH1	ACCATGACCACATGTGGT	436	424	1	2	0	0
EZH1	TGCATGACCACATGTGCA	274	224	0	0	0	0
EZH1	CACATGACCACATGTGAC	10	17	0	0	0	0
EZH1	TGTGCAGTACGTACACAC	16	14	0	0	0	0
EZH1	TGTGCAACCACACAGTCA	188	243	2	1	0	0
EZH2	ACACCATGCATGCATGTG	341	280	1	0	1	0
EZH2	TGACCATGGTACTGCACA	143	117	0	0	0	0
EZH2	CAACCATGGTACTGCAAC	18	7	0	0	0	0
EZH2	GTACCATGCATGACGTGT	540	780	0	0	0	0
EZH2	CAACCATGCATGACGTCA	69	72	0	0	0	0
EZH2	ACTGCAACTGGTACACCA	513	372	1	0	0	0
F10	CAACGTGTCAGTTGACGT	262	472	0	0	1	0
F10	TGACGTGTCAGTTGCAAC	7	17	0	0	0	0
F10	ACACGTGTCAGTTGCATG	364	197	0	0	0	0
F10	ACACGTGTCAGTTGACCA	112	78	0	0	0	0
F10	GTTGCAGTACCAGTTGTG	353	393	1	1	0	0
F11	TGACGTGTCAGTTGACTG	450	982	0	7	1	1
F11	GTACGTGTCAGTTGACAC	6	4	0	0	0	0
F11	ACACGTGTCAGTTGTGGT	390	274	1	0	0	6
F11	TGACGTGTCAGTTGTGCA	72	88	0	0	0	0

F11	CAACGTGTCAGTTGTGAC	31	25	0	0	0	0
F11R	CAACTGCATGGTACCACA	59	63	0	0	0	0
F11R	TGACTGCATGGTACCAAC	122	15	0	0	0	0
F11R	ACACTGCATGGTACCATG	633	239	0	1	0	0
F11R	CAACTGCATGGTACACGT	56	73	0	0	0	0
F11R	CATGCAGTTGTGGTACTG	738	685	1	0	0	0
F11R	GTTGCAACACGTTGCAGT	429	602	1	0	0	0
F12	GTACGTGTCAGTTGTGTG	272	278	1	0	0	0
F12	CAACGTGTCACAGTGTGT	542	642	1	1	0	0
F12	ACACGTGTCACAGTGTCA	729	425	7	0	0	8
F12	GTACGTGTCACAGTGTAC	14	14	0	0	0	0
F12	GTTGACACACACACCAAC	79	98	0	0	0	0
F13A1	ACACACTGCAGTGTCATG	66	117	4	0	13	10
F13A1	TGACACTGCAGTGTACTG	32	219	0	0	1	0
F13A1	CAACACTGCAGTGTACGT	216	195	0	0	0	0
F13A1	ACACACTGCAGTGTACCA	178	202	0	0	0	0
F13A1	GTACACTGCAGTGTACAC	2	6	0	0	0	0
F13B	GTACTGTGTGTGCATGCA	242	206	0	0	0	0
F13B	ACACTGTGTGTGCATGAC	27	10	0	1	0	0
F13B	CAACTGTGTGTGCATGTG	624	634	2	1	0	0
F13B	TGACTGTGTGTGACGTGT	385	333	1	0	0	0
F13B	GTACTGTGTGTGACGTCA	156	155	0	0	0	0
F2R	ACCATGACTGTGGTCAAC	27	8	0	0	0	0
F2R	CACATGACTGTGGTCATG	79	111	0	1	0	1
F2R	GTCATGACTGTGGTACGT	171	165	0	0	0	0
F2R	CACATGACTGTGGTACCA	75	116	1	0	0	3
F2R	TGCATGACTGTGGTACAC	56	203	0	0	0	0
F2R	ACTGCACAACCATGCAGT	141	163	0	0	0	2
F2RL1	ACACGTGTCAGTACGTTG	55	310	0	0	0	0
F2RL1	CAACGTGTCAGTACCAGT	420	542	3	0	0	0
F2RL1	ACACGTGTCAGTACCACA	243	406	0	0	0	0
F2RL1	GTACGTGTCAGTACCAAC	23	29	1	1	0	0
F2RL1	ACTGCAGTCATGACGTGT	463	389	2	0	0	0
F2RL2	TGACGTGTCAGTACCATG	101	72	0	0	0	1
F2RL2	ACACGTGTCAGTACACGT	36	32	0	0	0	0
F2RL2	TGACGTGTCAGTACACCA	215	297	0	0	0	18
F2RL2	CAACGTGTCAGTACACAC	30	74	0	0	0	0
F2RL2	GTACGTGTCAGTACACTG	213	306	1	0	0	0
F2RL3	ACACGTCAGTCAACGTAC	49	49	0	0	0	0
F2RL3	CAACGTCAGTCAACGTTG	390	516	0	0	0	0
F2RL3	ACTGCAGTACCATGCACA	165	277	0	1	0	0
F2RL3	GTTGCAGTACCATGCAAC	47	25	0	1	0	0
F2RL3	TGTGACCATGCAGTGTCA	307	277	1	2	0	0
F3	ACACTGACTGACCATGCA	299	302	1	1	1	1
F3	CATGCACATGCACAACCA	515	616	0	0	1	2
F3	TGACTGACTGACCAACAC	45	84	0	0	0	0
F3	ACACTGACTGACCAACTG	572	619	0	1	0	0
F3	CAACTGACTGACCATGGT	83	99	1	0	0	0
F3	GTACTGACTGACCATGAC	105	75	0	0	0	0
F5	ACACTGTGTGTGTGACTG	223	277	0	1	1	0
F5	CAACTGTGTGTGTGTGGT	296	544	3	0	0	15
F5	ACACTGTGTGTGTGTGCA	22	18	0	0	0	0
F5	GTACTGTGTGTGTGTGAC	66	79	0	0	0	0
F5	TGACTGTGTGTGTGTGTG	61	16	0	0	0	0
F7	GTACGTGTCAGTACTGCA	272	579	1	0	1	0
F7	TGACGTGTCAGTACTGGT	50	94	0	0	0	0
F7	ACACGTGTCAGTACTGAC	47	64	0	0	0	0
F7	CAACGTGTCAGTACTGTG	267	123	0	1	0	0
F7	TGACGTGTCAGTTGGTGT	156	192	2	0	0	0
F8	CATGACCAGTACCATGCA	167	344	0	0	0	0
F8	TGTGACCAGTACCATGAC	15	14	0	0	0	0
F8	ACTGACCAGTACCATGTG	661	663	1277	3	0	0
F8	GTTGACCAGTACACGTGT	238	290	1	0	0	0
F8	CATGACCAGTACACGTCA	92	115	0	0	0	0
F8A1	TGTGGTCATGCACAACTG	483	878	0	1	1	31
F8A1	ACTGGTCATGCACATGGT	758	669	1	0	0	0
F8A1	TGTGGTCATGCACATGCA	336	446	1	0	0	2
F8A1	CATGGTCATGCACATGAC	69	23	0	0	0	0
F8A1	GTTGGTCATGCACATGTG	888	789	2	1	0	0
F9	GTACGTGTCAGTTGGTCA	194	342	0	0	0	3
F9	ACACGTGTCAGTTGGTAC	60	71	0	0	0	0
F9	CAACGTGTCAGTTGGTTG	777	817	1	1	0	1
F9	GTACGTGTCAGTTGCAGT	325	362	0	1	0	0
F9	CAACGTGTCAGTTGCACA	75	128	0	0	0	0
F9	TGTGCACATGCACAACAC	78	5	0	1	0	0

FAAH	ACACACACGTCAACCAAC	61	87	0	0	0	0
FAAH	CAACACACGTCAACCATG	127	154	0	0	0	0
FAAH	GTACACACGTCAACACGT	84	73	0	0	0	0
FAAH	CAACACACGTCAACACCA	287	118	0	0	0	4
FAAH	TGTGCAGTTGCAGTCAGT	311	427	2	1	0	0
FAAH	GTTGCACACACATGTGTG	257	276	0	0	0	3
FABP1	GTACTGCACAGTACGTTG	1080	896	2	0	0	0
FABP1	TGACTGCACAGTACCAGT	205	215	0	0	0	0
FABP1	GTACTGCACAGTACCACA	186	32	0	0	0	0
FABP1	ACACTGCACAGTACCAAC	48	107	0	0	0	0
FABP1	GTTGCAGTTGACACTGAC	323	72	0	826	0	0
FABP1	GTTGCACACAACGTGTGT	248	189	2	0	0	0
FABP2	ACACTGCACAGTACACTG	229	246	0	1	0	0
FABP2	CAACTGCACAGTACTGGT	439	836	0	0	0	0
FABP2	ACACTGCACAGTACTGCA	43	84	0	0	0	0
FABP2	GTACTGCACAGTACTGAC	55	120	0	0	0	0
FABP2	TGACTGCACAGTACTGTG	176	220	1	0	0	0
FABP2	CATGCACATGCATGGTCA	54	69	0	0	0	0
FABP3	TGACTGCACAGTTGCACA	129	106	0	0	1	0
FABP3	ACACTGCACAGTTGCAGT	53	18	0	0	0	0
FABP3	CAACTGCACAGTTGCAAC	0	3	0	0	0	0
FABP3	CATGGTGTCACAACCAGT	459	407	0	0	0	0
FABP3	ACTGGTTGTGTGGTTGTG	313	88	0	0	0	0
FABP4	GTACTGCACAGTCAACAC	41	34	0	0	0	0
FABP4	TGACTGCACAGTCAACTG	512	275	1	0	0	0
FABP4	ACACTGCACAGTCATGGT	448	503	1	1	0	6
FABP4	TGACTGCACAGTCATGCA	68	95	0	0	0	0
FABP4	ACTGCAGTTGACACTGCA	371	473	1	1	0	0
FABP4	CATGCACACAACGTGTCA	216	330	2	0	0	6
FABP5	TGACTGCACACAGTGTGT	436	501	0	0	0	0
FABP5	GTACTGCACACAGTGTCA	370	301	1	0	0	0
FABP5	ACACTGCACACAGTGTAC	39	49	0	0	0	0
FABP5	CAACTGCACACAGTGTTG	280	324	0	0	0	0
FABP5	GTACTGCACACAGTCAGT	596	378	3	0	0	0
FABP5	TGTGCACACAACGTGTAC	48	42	0	0	0	0
FABP6	GTACTGCACACAGTTGTG	262	192	1	0	1	0
FABP6	CAACTGCACACACAGTAC	366	250	0	1	1	0
FABP6	CAACTGCACACAGTTGAC	35	41	0	0	0	0
FABP6	ACACTGCACACACAGTGT	256	176	1	1	0	17
FABP6	TGACTGCACACACAGTCA	292	213	0	0	0	9
FABP7	GTACTGCACACACAGTTG	949	1001	1	1	0	1
FABP7	TGACTGCACACACACAGT	322	260	0	1	0	2
FABP7	GTACTGCACACACACACA	356	237	0	1	0	0
FABP7	ACACTGCACACACACAAC	39	43	0	0	0	0
FABP7	TGTGCAGTTGACACTGTG	37	54	0	0	0	0
FADD	CATGCAACACACTGACAC	75	72	0	31	1	0
FADD	GTACCATGACCAGTTGGT	513	511	1	0	0	1
FADD	CAACCATGACCAGTTGCA	343	416	1	1	0	1
FADD	TGACCATGACCAGTTGAC	46	51	0	0	0	0
FADD	ACACCATGACCAGTTGTG	491	267	0	0	0	0
FADD	GTTGCAACACACTGACTG	313	536	0	1	0	0
FADS1	CAACTGACACACACCATG	598	218	1	0	0	2
FADS1	GTACTGACACACACACGT	36	24	0	0	0	0
FADS1	CAACTGACACACACACCA	109	184	0	0	0	0
FADS1	TGACTGACACACACACAC	24	110	0	0	0	0
FADS1	ACACTGACACACACACTG	490	591	0	0	0	0
FADS1	ACTGCAACGTGTACCAGT	86	96	0	0	0	0
FADS2	GTACTGACACTGGTACCA	99	112	0	1	0	0
FADS2	ACACTGACACTGGTACAC	41	55	0	0	0	0
FADS2	CAACTGACACTGGTACTG	447	413	1	0	0	14
FADS2	GTACTGACACTGGTTGGT	143	208	0	0	0	0
FADS2	CAACTGACACTGGTTGCA	129	198	0	0	0	0
FADS2	TGTGCAACGTGTACCACA	20	16	0	0	0	0
FAF1	CACATGTGCAGTGTCAGT	143	225	0	0	0	0
FAF1	ACCATGTGCAGTGTCACA	52	71	0	0	0	0
FAF1	GTCATGTGCAGTGTCAAC	4	7	0	0	0	0
FAF1	TGCATGTGCAGTGTCATG	163	181	0	0	0	0
FAF1	ACCATGTGCAGTGTACGT	303	262	0	1	0	0
FAF1	GTTGCAACCAGTACACGT	89	76	0	0	0	0
FAH	ACTGCACACAACGTGTTG	324	208	0	0	1	0
FAH	TGACACACGTCATGGTTG	218	272	5	1	0	0
FAH	ACACACACGTCATGCAGT	133	183	1	0	0	0
FAH	TGACACACGTCATGCACA	101	80	0	0	0	0
FAH	CAACACACGTCATGCAAC	101	157	0	0	0	1
FAH	GTTGCAGTCACATGACGT	490	352	2	1	0	0

FAM132A	ACTGCAGTGTACTGGTAC	45	38	0	0	0	0
FAM132A	CATGCAGTGTACTGGTTG	343	303	1	0	0	0
FAM132A	GTTGCAGTGTACTGCAGT	53	70	0	0	0	0
FAM132A	CATGCAGTGTACTGCACA	113	79	1	1	0	0
FAM132A	GTTGACACCATGCATGCA	191	285	0	0	0	1
FAM50A	ACTGGTCAACTGACGTGT	390	391	0	0	0	1
FAM50A	TGTGGTCAACTGACGTCA	169	185	0	0	0	0
FAM50A	CATGGTCAACTGACGTAC	84	59	0	0	0	0
FAM50A	GTTGGTCAACTGACGTTG	203	666	0	1	0	0
FAM50A	TGTGGTCAACTGACCAGT	69	46	0	0	0	0
FAM50A	TGTGCACACATGGTCACA	158	144	0	0	0	0
FAM59A	TGTGGTCACAACACGTGT	602	800	0	0	0	1
FAM59A	GTTGGTCACAACACGTCA	136	101	0	0	0	0
FAM59A	ACTGGTCACAACACGTAC	97	116	0	0	0	0
FAM59A	CATGGTCACAACACGTTG	626	452	1	0	0	0
FAM59A	ACTGCACATGACACCATG	274	236	0	0	0	0
FAM59A	CATGCACATGACACACGT	108	93	0	0	0	0
FANCA	ACTGGTCAGTTGGTTGTG	637	863	0	198	10	0
FANCA	GTTGGTCAGTTGGTTGGT	278	213	0	1	0	0
FANCA	CATGGTCAGTTGGTTGCA	162	199	0	0	0	1
FANCA	TGTGGTCAGTTGGTTGAC	49	186	0	0	0	0
FANCA	GTTGGTCAGTTGCAGTGT	435	405	0	0	0	3
FANCB	ACTGGTTGCAACGTTGGT	231	328	0	26	0	0
FANCB	TGTGGTTGCAACGTTGCA	296	139	1	0	0	0
FANCB	CATGGTTGCAACGTTGAC	143	215	1	0	0	3
FANCB	GTTGGTTGCAACGTTGTG	65	273	0	0	0	1
FANCB	ACTGGTTGCAACCAGTGT	43	95	0	0	0	0
FANCC	CAACTGTGTGACACACCA	295	401	1	38	229	0
FANCC	CAACTGTGTGACACCATG	107	98	0	0	0	0
FANCC	GTACTGTGTGACACACGT	110	104	0	0	0	0
FANCC	TGACTGTGTGACACACAC	53	84	0	0	0	0
FANCC	TGTGCAGTTGTGTGACAC	44	38	0	0	0	0
FANCD2	CAACTGTGACTGCAACTG	1407	1479	3	1	0	0
FANCD2	GTACTGTGACTGCATGGT	22	40	0	0	0	0
FANCD2	CAACTGTGACTGCATGCA	432	233	1	0	0	0
FANCD2	ACTGCACAGTGTGTTGCA	216	347	0	1	0	0
FANCD2	ACTGACCAGTACTGCATG	244	335	1	0	0	0
FANCE	CATGGTCAGTACCACACA	223	240	0	1	1	0
FANCE	GTTGGTCAGTACCACAGT	84	111	1	0	0	0
FANCE	TGTGGTCAGTACCACAAC	11	16	0	0	0	0
FANCE	ACTGGTCAGTACCACATG	110	119	0	0	0	0
FANCE	CATGGTCAGTACCAACGT	348	417	0	0	0	0
FANCF	TGACTGTGTGGTTGACCA	127	95	0	0	0	1
FANCF	CAACTGTGTGGTTGACAC	93	34	0	0	0	0
FANCF	GTACTGTGTGGTTGACTG	310	235	127	0	0	0
FANCF	TGACTGTGTGGTTGTGGT	201	342	2	0	0	0
FANCF	GTACTGTGTGGTTGTGCA	62	64	0	0	0	0
FANCG	ACACTGTGACTGACGTTG	1187	483	919	1	1	0
FANCG	TGACTGTGACTGACGTAC	204	40	0	0	0	0
FANCG	CAACTGTGACTGACCAGT	142	109	0	0	0	0
FANCG	ACACTGTGACTGACCACA	177	129	0	0	0	0
FANCG	ACTGCACAGTGTGTCAAC	30	37	0	0	0	0
FANCG	GTTGCACACATGACGTCA	246	43	0	0	0	0
FANCI	GTTGCAGTGTGTGTTGGT	232	312	1	0	3	0
FANCI	GTTGCAGTGTGTGTACCA	112	208	0	0	0	0
FANCI	ACTGCAGTGTGTGTACAC	46	77	0	0	0	0
FANCI	CATGCAGTGTGTGTACTG	798	792	1	1	0	1
FANCI	CATGCAGTGTGTGTTGCA	144	142	0	1	0	0
FANCL	CAACTGTGTGACCACACA	50	84	0	0	0	0
FANCL	TGACTGTGTGACCACAAC	98	21	0	0	0	0
FANCL	ACACTGTGTGACCACATG	278	270	0	0	0	0
FANCL	CAACTGTGTGACCAACGT	129	85	0	0	0	0
FANCL	ACACTGTGTGACCAACCA	295	223	0	0	0	0
FANCM	TGACACACCAACTGGTAC	131	149	0	0	1	0
FANCM	TGACACACCAACACTGAC	136	157	0	0	0	0
FANCM	ACACACACCAACACTGTG	808	490	0	1	0	0
FANCM	GTACACACCAACTGGTGT	377	319	0	0	0	0
FANCM	CAACACACCAACTGGTCA	1116	729	0	1	0	1
FARS2	ACACACGTACGTACCACA	88	81	0	0	1	0
FARS2	CAACACGTACGTACCAGT	218	311	1	0	0	0
FARS2	GTACACGTACGTACCAAC	19	28	0	0	0	0
FARS2	TGACACGTACGTACCATG	225	196	0	0	0	0
FARS2	ACACACGTACGTACACGT	18	10	0	0	0	0
FARSA	CAACACGTACACACGTGT	637	1022	0	1	1	2
FARSA	ACACACGTACACACGTCA	116	152	0	0	0	0

FARSA	GTACACGTACACACGTAC	60	123	0	0	0	0
FARSA	TGACACGTACACACGTTG	187	185	0	0	0	0
FARSA	ACACACGTACACACCAGT	44	65	0	0	0	0
FARSB	ACACACGTACACCACAAC	51	48	0	0	0	0
FARSB	CAACACGTACACCACATG	212	343	0	0	0	0
FARSB	GTACACGTACACCAACGT	355	307	0	0	0	0
FARSB	CAACACGTACACCAACCA	460	448	1	0	0	0
FARSB	TGACACGTACACCAACAC	38	44	0	0	0	0
FAS	GTTGCAGTGTCATGTGGT	588	489	1	0	1	25
FAS	TGACCATGGTCACATGAC	58	75	0	0	0	0
FAS	ACACCATGGTCACATGTG	116	126	0	0	0	0
FAS	GTACCATGGTCAACGTGT	364	391	2	0	0	4
FAS	CAACCATGGTCAACGTCA	236	340	0	0	0	0
FAS	TGTGCAACTGGTACTGCA	106	61	0	0	0	0
FASLG	CAACTGCAGTGTCATGGT	473	1130	0	0	1	0
FASLG	TGACTGCAGTGTCAACAC	68	36	0	0	0	0
FASLG	ACACTGCAGTGTCAACTG	137	107	0	0	0	0
FASLG	ACACTGCAGTGTCATGCA	185	217	0	0	0	0
FASLG	TGTGCAGTTGACACACAC	7	2	0	0	0	0
FASLG	ACTGCACACACAACCACA	70	230	0	0	0	0
FASN	GTCATGACACCATGCATG	533	551	0	0	1	4
FASN	TGCATGACACCATGGTTG	405	498	1	0	0	0
FASN	ACCATGACACCATGCAGT	103	267	0	1	0	0
FASN	TGCATGACACCATGCACA	62	65	0	0	0	0
FASN	CACATGACACCATGCAAC	52	54	0	1	0	1
FAT	GTACCAACTGACCAGTGT	367	336	2	2	0	0
FAT	CAACCAACTGACCAGTCA	189	213	0	0	0	0
FAT	TGACCAACTGACCAGTAC	101	113	0	0	0	0
FAT	ACACCAACTGACCAGTTG	1081	655	1	0	0	0
FAT	CAACCAACTGACCACAGT	246	395	0	0	0	0
FAU	CAACGTGTCAACTGGTCA	520	373	0	0	0	1
FAU	TGACGTGTCAACTGGTAC	74	79	0	0	0	0
FAU	ACACGTGTCAACTGGTTG	1083	1499	2	1	0	2
FAU	TGTGCAGTACCACACAGT	52	94	0	0	0	0
FAU	TGTGACCACACAACACTG	1040	524	2	0	0	0
FBL	ACTGGTTGGTTGTGGTTG	133	173	0	1	0	0
FBL	CATGGTTGGTTGTGCAGT	89	108	0	0	0	0
FBL	ACTGGTTGGTTGTGCACA	104	159	0	0	0	0
FBL	GTTGGTTGGTTGTGCAAC	30	31	0	0	0	0
FBL	TGTGGTTGGTTGTGCATG	32	34	0	0	0	0
FBL	GTTGCACATGACACCAGT	20	9	0	0	0	0
FBLN1	ACACACTGCAGTGTTGGT	141	179	0	0	0	0
FBLN1	TGACACTGCAGTGTTGCA	208	302	0	0	0	0
FBLN1	CAACACTGCAGTGTTGAC	13	17	0	0	0	0
FBLN1	GTACACTGCAGTGTTGTG	156	175	0	0	0	0
FBLN1	GTTGCAGTCAACCAACGT	299	621	0	0	0	0
FBLN2	GTACACTGCAGTACGTAC	35	31	0	0	0	0
FBLN2	TGACACTGCAGTACGTTG	101	712	0	0	0	0
FBLN2	ACACACTGCAGTACCAGT	66	77	0	0	0	0
FBLN2	TGACACTGCAGTACCACA	10	34	0	0	0	0
FBLN2	CAACACTGCAGTACCAAC	48	81	1	0	0	0
FBP1	TGACACCATGACCACAGT	526	56	0	0	0	0
FBP1	GTACACCATGACCACACA	294	127	0	0	0	0
FBP1	ACACACCATGACCACAAC	44	123	0	0	0	0
FBP1	CAACACCATGACCACATG	492	448	1	0	0	0
FBP1	GTTGCAGTCAACACTGGT	1353	766	1	1	0	0
FBP1	ACTGCAACGTACACCAAC	49	28	0	0	0	0
FBP2	ACACACACGTACGTTGCA	35	59	0	0	0	0
FBP2	GTACACACGTACGTTGAC	40	14	0	0	0	0
FBP2	TGACACACGTACGTTGTG	74	164	1	0	0	0
FBP2	CAACACACGTACCAGTGT	396	351	0	0	0	12
FBP2	ACACACACGTACCAGTCA	178	267	0	0	0	0
FBP2	TGTGCAACACTGACCAGT	116	140	0	0	0	0
FBXW11	GTCATGTGCAGTCAGTCA	178	300	0	0	0	0
FBXW11	ACCATGTGCAGTCAGTAC	185	144	0	0	0	1
FBXW11	CACATGTGCAGTCAGTTG	351	437	0	0	0	3
FBXW11	GTCATGTGCAGTCACAGT	155	143	0	0	0	0
FBXW11	CACATGTGCAGTCACACA	161	138	2	0	0	1
FBXW2	ACTGCACAACACTGACCA	534	136	0	0	1	0
FBXW2	CAACGTGTGTTGGTGTTG	132	124	0	0	0	0
FBXW2	GTACGTGTGTTGGTCAGT	234	273	0	0	0	0
FBXW2	CAACGTGTGTTGGTCACA	238	53	0	0	0	0
FBXW2	TGACGTGTGTTGGTCAAC	38	26	1	0	0	0
FBXW2	ACACGTGTGTTGGTCATG	277	196	1	0	0	7
FBXW7	CAACGTGTGTTGGTACGT	497	772	1	0	0	0

FBXW7	ACACGTGTGTTGGTACCA	298	423	1	0	0	0
FBXW7	GTACGTGTGTTGGTACAC	27	33	0	64	0	0
FBXW7	TGACGTGTGTTGGTACTG	1281	913	0	0	0	1
FBXW7	CATGGTTGTGCATGGTTG	392	403	1	0	0	3
FCER1A	ACACCAGTTGCACAACGT	1332	559	29	14	44	65
FCER1A	CAACCAGTTGCACACAGT	74	117	1	1	0	0
FCER1A	ACACCAGTTGCACACACA	98	88	1	0	0	0
FCER1A	GTACCAGTTGCACACAAC	32	39	0	0	0	0
FCER1A	TGACCAGTTGCACACATG	385	256	1	0	0	0
FCER1A	TGTGCATGGTTGCATGAC	86	36	0	0	0	0
FCER1G	ACACACTGTGTGCAACAC	116	219	0	0	0	0
FCER1G	CAACACTGTGTGCAACTG	762	908	1	1	0	5
FCER1G	GTACACTGTGTGCATGGT	105	128	0	0	0	0
FCER1G	CAACACTGTGTGCATGCA	243	217	0	0	0	0
FCER1G	TGACTGACTGCATGGTCA	281	407	1	0	0	1
FCER1G	GTTGCACACATGGTCATG	14	22	0	0	0	0
FCER2	TGACCAGTTGCACAACCA	190	172	0	0	0	0
FCER2	CAACCAGTTGCACAACAC	113	75	0	906	0	0
FCER2	GTACCAGTTGCACAACTG	735	497	2	0	0	0
FCER2	TGACCAGTTGCACATGGT	968	539	3	0	0	2
FCER2	GTTGCAGTACTGGTTGAC	73	62	0	0	0	26
FCGR1A	TGACCAGTTGGTTGACTG	627	279	0	2	0	0
FCGR1A	ACACCAGTTGGTTGTGGT	35	25	0	0	0	0
FCGR1A	TGACCAGTTGGTTGTGCA	484	386	1	0	0	0
FCGR1A	CAACCAGTTGGTTGTGAC	89	70	0	0	0	0
FCGR1A	GTACCAGTTGGTTGTGTG	536	720	0	0	0	0
FCGR1A	TGTGCAACTGCAACCAGT	152	117	0	1	0	1
FCGR2A	CAACCAGTTGCAGTGTGT	940	739	0	0	1	0
FCGR2A	ACACCAGTTGCAGTGTCA	163	228	0	0	1	0
FCGR2A	GTACCAGTTGCAGTGTAC	197	188	1	0	0	0
FCGR2A	TGACCAGTTGCAGTGTTG	401	775	2	0	0	18
FCGR2A	CAACCAGTTGCAGTCAAC	8	13	0	0	0	0
FCGR2B	ACACCAGTTGCAGTCAGT	156	354	0	0	1	0
FCGR2B	TGACCAGTTGCAGTCACA	97	85	0	0	1	0
FCGR2B	GTACCAGTTGCAGTCATG	637	706	0	0	0	0
FCGR2B	TGACCAGTTGCAGTACGT	284	245	0	0	0	0
FCGR2B	GTACCAGTTGCAGTACCA	66	91	0	0	0	1
FCGR3A	TGACCAGTTGCAGTTGAC	40	20	0	0	0	0
FCGR3A	TGTGGTACGTACCATGGT	562	510	2	1	0	8
FCGR3A	ACTGGTACGTACCATGAC	19	29	1	0	0	0
FCGR3A	CATGGTACGTACCATGTG	85	110	0	0	0	0
FCGR3A	ACTGACGTACGTTGTGAC	5	6	0	0	0	1
FCGR3B	ACACCAGTTGCAGTACAC	13	20	0	0	0	3
FCGR3B	CAACCAGTTGCAGTACTG	105	531	0	1	0	0
FCGR3B	GTACCAGTTGCAGTTGGT	133	243	0	0	0	0
FCGR3B	CAACCAGTTGCAGTTGCA	107	233	0	0	0	5
FCGR3B	GTTGGTACGTACCATGCA	71	64	0	0	0	0
FCGRT	CAACCATGTGGTGTGTGT	937	720	212	1	2	15
FCGRT	ACACCATGTGGTGTGTCA	238	330	1	0	0	0
FCGRT	GTACCATGTGGTGTGTAC	5	0	0	0	0	0
FCGRT	TGACCATGTGGTGTGTTG	153	174	0	1	0	0
FCGRT	ACACCATGTGGTGTCAGT	72	94	0	0	0	0
FCN1	ACACACTGCACACAACAC	64	64	0	0	0	0
FCN1	CAACACTGCACACAACTG	991	1560	1	0	0	4
FCN1	GTACACTGCACACATGGT	1298	665	0	0	0	1
FCN1	CAACACTGCACACATGCA	299	57	0	0	0	0
FCN1	GTTGCAGTTGTGACCAGT	274	265	0	0	0	1
FCN1	GTTGCACATGGTGTGTGT	1462	1099	1	0	0	0
FDFT1	TGACCAACCAACCAACTG	851	1162	3	1	2	4
FDFT1	CAACCAACCAACCAACGT	68	39	0	0	0	0
FDFT1	ACACCAACCAACCAACCA	110	103	0	0	0	0
FDFT1	GTACCAACCAACCAACAC	157	211	0	0	0	0
FDFT1	ACACCAACCAACCATGGT	151	402	1	1	0	0
FDPS	CAACCAACCAACGTGTCA	295	343	0	0	1	0
FDPS	TGACCAACCAACGTGTAC	22	29	0	0	0	0
FDPS	ACACCAACCAACGTGTTG	130	247	1	0	0	9
FDPS	CAACCAACCAACGTCAGT	383	837	1	0	0	0
FDPS	ACACCAACCAACGTCACA	70	146	0	0	0	0
FDXR	TGACACGTTGACACGTAC	24	55	0	0	0	0
FDXR	ACACACGTTGACACGTTG	102	271	0	0	0	0
FDXR	CAACACGTTGACACCAGT	467	207	0	0	0	1
FDXR	ACACACGTTGACACCACA	59	63	0	0	0	0
FDXR	ACTGCAGTTGGTACGTCA	116	134	0	0	0	0
FDXR	CATGCACATGTGACACAC	108	144	0	0	0	0
FECH	ACTGGTGTGTGTCATGTG	299	350	1	0	1	0

FECH	TGTGGTGTGTGTCATGAC	19	31	0	0	0	0
FECH	GTTGGTGTGTGTACGTGT	93	102	1	0	0	0
FECH	CATGGTGTGTGTACGTCA	172	250	0	0	0	0
FECH	TGTGGTGTGTGTACGTAC	100	85	0	0	0	0
FEN1	GTACACCATGCAGTACCA	282	412	0	0	0	0
FEN1	ACACACCATGCAGTACAC	247	179	3	1	0	4
FEN1	CAACACCATGCAGTACTG	670	1212	1	1	0	1
FEN1	GTACACCATGCAGTTGGT	367	269	0	0	0	0
FEN1	CAACACCATGCAGTTGCA	115	132	0	1	0	0
FEN1	ACTGCAACCAGTTGGTCA	918	908	1	1	0	0
FES	TGCATGCAACACGTCACA	196	103	0	0	0	0
FES	CACATGCAACACGTCAAC	50	112	0	0	0	0
FES	GTCATGCAACACGTCATG	176	281	0	0	0	0
FES	TGCATGCAACACGTACGT	245	349	0	0	0	0
FES	ACTGGTACTGTGACTGCA	207	68	1	2	0	4
FES	ACTGCACATGTGACCACA	222	255	0	0	0	2
FEZ1	CAACTGACGTCACAGTAC	103	152	0	0	0	0
FEZ1	GTACTGACGTCACAGTTG	725	1285	1	1	0	7
FEZ1	CATGCAACGTTGGTGTGT	1285	1247	2	1	0	2
FEZ1	ACTGCAACGTTGGTGTCA	413	279	0	0	0	0
FEZ1	GTTGCAACGTTGGTGTAC	25	24	0	0	0	0
FEZ1	TGTGCAACGTTGGTGTTG	219	339	2	0	0	0
FGA	CAACTGTGTGTGGTACTG	234	307	0	0	0	0
FGA	GTACTGTGTGTGGTTGGT	309	220	0	0	0	0
FGA	TGTGACCACAGTTGACTG	522	599	0	1	0	1
FGA	ACTGACCACAGTTGTGGT	180	120	0	0	0	0
FGA	TGTGACCACAGTTGTGCA	97	222	0	0	0	0
FGB	GTTGACCACAGTTGTGTG	46	81	29	28	3	28
FGB	TGTGGTGTGTACGTGTGT	170	294	0	0	1	0
FGB	TGTGGTGTGTCATGTGTG	142	154	0	0	0	0
FGB	GTTGGTGTGTACGTGTCA	189	157	0	0	0	1
FGB	CATGACCACACAGTGTGT	232	347	2	0	0	0
FGF1	ACACTGACTGTGGTGTTG	241	198	0	0	1	0
FGF1	GTTGCACAGTGTTGCAGT	94	145	0	0	1	1
FGF1	CAACTGACTGTGGTCAGT	158	475	0	0	0	1
FGF1	ACTGCAGTTGTGTGGTGT	366	966	1	1	0	0
FGF1	TGTGCAGTTGTGTGGTCA	166	150	0	0	0	30
FGF1	GTTGCAACGTCACACATG	186	235	1	0	0	0
FGF10	CAACTGGTACTGGTGTTG	590	488	2	1	1	0
FGF10	TGTGCACACATGGTACGT	254	152	0	0	1	0
FGF10	TGACTGGTACTGGTGTGT	607	607	0	0	0	1
FGF10	GTACTGGTACTGGTGTCA	223	284	0	0	0	0
FGF10	ACACTGGTACTGGTGTAC	191	279	0	0	0	0
FGF10	GTACTGGTACTGGTCAGT	214	426	0	1	0	1
FGF11	ACACTGGTACCAGTTGAC	32	35	0	0	0	0
FGF11	CAACTGGTACCAGTTGTG	484	124	0	0	0	1
FGF11	TGACTGGTACCACAGTGT	1211	1431	2	3	0	1
FGF11	GTACTGGTACCACAGTCA	610	413	2	1	0	16
FGF11	GTACTGACTGCAACACAC	200	90	0	0	0	0
FGF12	ACACTGGTACACACCACA	19	28	0	0	0	0
FGF12	GTACTGGTACACACCAAC	16	27	0	0	0	0
FGF12	TGACTGGTACACACCATG	561	381	2	0	0	0
FGF12	ACACTGGTACACACACGT	17	30	1	0	0	110
FGF12	TGACTGGTACACACACCA	115	192	2	0	0	0
FGF12	ACTGCAACACCACATGAC	216	99	0	0	0	0
FGF13	CAACTGCAGTCAACACAC	134	100	0	0	0	0
FGF13	GTACTGCAGTCAACACTG	112	237	0	0	0	0
FGF13	TGACTGCAGTCAACTGGT	388	245	0	0	0	1
FGF13	GTACTGCAGTCAACTGCA	55	49	0	0	0	0
FGF13	ACACTGCAGTCAACTGAC	56	67	0	0	0	0
FGF13	CATGCAACGTACGTTGTG	166	285	0	0	0	0
FGF18	TGACCACAGTTGTGCATG	458	519	0	0	0	0
FGF18	ACACCACAGTTGTGACGT	62	91	0	0	0	0
FGF18	TGACCACAGTTGTGACCA	195	332	0	0	0	0
FGF18	CAACCACAGTTGTGACAC	50	172	0	0	0	0
FGF18	GTACCACAGTTGTGACTG	411	536	0	0	0	0
FGF18	TGTGCACAGTACACCACA	103	95	0	1	0	0
FGF2	GTCATGACACTGCAACGT	176	339	0	0	0	0
FGF2	CACATGACACTGCAACCA	624	645	0	1	0	0
FGF2	TGCATGACACTGCAACAC	22	45	0	0	0	0
FGF2	ACCATGACACTGCAACTG	681	628	2	1	0	0
FGF2	CACATGACACTGCATGGT	196	228	0	0	0	0
FGF20	ACACTGGTACCATGACAC	53	36	0	0	0	2
FGF20	CAACTGGTACCATGACTG	1077	883	0	1	0	10
FGF20	GTACTGGTACCATGTGGT	791	518	1	1	0	1

FGF20	CAACTGGTACCATGTGCA	560	650	0	1	0	0
FGF20	TGTGCAGTTGACACGTCA	216	384	0	0	0	0
FGF20	TGTGCACATGGTTGCACA	73	85	0	0	0	0
FGF3	GTACCAACTGACACACAC	73	86	0	0	0	0
FGF3	TGACCAACTGACACACTG	227	595	0	0	0	1
FGF3	ACACCAACTGACACTGGT	364	876	0	1	0	1
FGF3	TGACCAACTGACACTGCA	123	129	0	0	0	0
FGF3	TGTGCAGTACTGTGACTG	354	518	0	0	0	10
FGF3	CATGCAACGTCATGCACA	114	120	0	0	0	0
FGF4	ACACCAACTGACACGTAC	102	102	1	0	0	0
FGF4	CAACCAACTGACACGTTG	680	1275	0	202	0	0
FGF4	ACTGCAGTACTGTGCATG	142	138	0	0	0	0
FGF4	CATGCAGTACTGTGACGT	178	422	0	1	0	1
FGF4	CATGCACAGTACACCAAC	51	87	0	0	0	0
FGF4	GTTGCACAGTACACCATG	181	132	1	0	0	0
FGF5	TGTGACCACAACCAGTCA	398	569	0	0	1	2
FGF5	ACACTGGTTGTGGTCAAC	24	18	0	0	0	0
FGF5	CAACTGGTTGTGGTCATG	686	464	1	0	0	4
FGF5	GTACTGGTTGTGGTACGT	186	121	0	1	0	0
FGF5	CAACTGGTTGTGGTACCA	524	520	0	0	0	4
FGF6	TGACCAACTGACACGTGT	725	990	3	0	1	0
FGF6	GTACCAACTGACCATGCA	792	946	346	0	0	62
FGF6	ACACCAACTGACCATGAC	218	106	0	0	0	0
FGF6	CAACCAACTGACCATGTG	497	452	0	0	0	0
FGF6	GTACCAACTGACACGTCA	122	94	0	0	0	0
FGF6	CATGCACATGCACACATG	382	488	2	0	0	1
FGF7	ACACTGGTACACCAGTCA	527	503	1	0	0	0
FGF7	GTACTGGTACACCAGTAC	60	24	0	0	0	0
FGF7	TGACTGGTACACCAGTTG	821	764	2	0	0	0
FGF7	ACACTGGTACACCACAGT	366	194	0	2	0	0
FGF7	TGACTGGTACACCACACA	217	101	0	0	0	8
FGF7	GTTGCAACGTCACATGGT	579	499	0	0	0	0
FGF8	CAACTGGTACGTACTGAC	32	20	0	0	0	0
FGF8	GTACTGGTACGTACTGTG	105	124	0	0	0	0
FGF8	CATGCAACGTCACATGCA	160	221	1	0	0	1
FGF8	TGTGCAACGTCACATGAC	35	19	0	0	0	0
FGF8	ACTGCAACGTCACATGTG	168	181	0	0	0	0
FGF8	GTTGCAACGTCAACGTGT	115	107	0	0	0	0
FGF9	TGACTGGTTGTGCATGAC	91	130	0	0	0	0
FGF9	ACACTGGTTGTGCATGTG	181	279	0	0	0	1
FGF9	GTACTGGTTGTGACGTGT	271	172	0	0	0	0
FGF9	CAACTGGTTGTGACGTCA	123	174	0	0	0	0
FGF9	TGACTGGTTGTGACGTAC	62	111	0	0	0	0
FGF9	TGTGCACAGTACACACGT	60	63	1	0	0	0
FGFR1	ACCATGCACATGGTACGT	82	134	0	0	0	1
FGFR1	TGCATGCACATGGTACCA	402	320	0	0	0	0
FGFR1	CACATGCACATGGTACAC	21	25	0	0	0	0
FGFR1	GTCATGCACATGGTACTG	273	340	0	0	0	0
FGFR1	TGTGGTCACAGTGTTGGT	312	519	0	1	0	2
FGFR2	CACATGCACAACACGTGT	745	1043	1	1	0	0
FGFR2	ACCATGCACAACACGTCA	48	59	0	0	0	0
FGFR2	GTCATGCACAACACGTAC	79	43	0	0	0	0
FGFR2	TGCATGCACAACACGTTG	239	373	1	0	0	0
FGFR2	ACCATGCACAACACCAGT	323	461	0	0	0	9
FGFR3	CATGGTTGGTGTTGCATG	156	193	0	0	1	0
FGFR3	TGCATGCACAACACCACA	81	63	0	0	0	0
FGFR3	CACATGCACAACACCAAC	28	49	0	0	0	0
FGFR3	GTCATGCACAACACCATG	350	435	2	0	0	0
FGFR3	GTTGGTTGGTGTTGACGT	61	96	0	0	0	0
FGFR3	GTTGCACATGACTGTGAC	72	79	0	0	0	1
FGFR4	GTCATGCAACACGTACCA	42	46	0	0	0	0
FGFR4	ACCATGCAACACGTACAC	17	24	0	0	0	0
FGFR4	CACATGCAACACGTACTG	470	453	0	0	0	0
FGFR4	TGTGGTTGACACTGACAC	16	30	0	0	0	0
FGFR4	ACTGGTTGACACTGACTG	1067	912	0	1	0	2
FGG	TGTGGTGTGTCAGTACGT	96	99	0	0	0	1
FGG	GTTGGTGTGTCAGTACCA	192	157	0	1	0	0
FGG	ACTGGTGTGTCAGTACAC	1	4	0	0	0	0
FGG	CATGGTGTGTCAGTACTG	183	182	2	0	0	1
FGG	GTTGGTGTGTCAGTTGGT	289	390	0	0	0	0
FGL2	TGTGGTCAGTGTTGACCA	29	41	1	1	0	1
FGL2	CATGGTCAGTGTTGACAC	12	7	0	0	0	0
FGL2	GTTGGTCAGTGTTGACTG	131	122	0	0	0	1
FGL2	TGTGGTCAGTGTTGTGGT	1307	982	0	0	0	0
FGL2	GTTGGTCAGTGTTGTGCA	244	247	1	0	0	2

FGL2	ACTGCACAACCACACATG	32	56	0	2	0	0
FGR	GTCATGACGTGTTGGTCA	455	618	2	149	6	2
FGR	CATGGTACTGTGCAGTTG	666	958	5	2	2	203
FGR	ACCATGACGTGTTGGTAC	63	31	0	0	0	8
FGR	CACATGACGTGTTGGTTG	1182	572	0	0	0	0
FGR	GTCATGACGTGTTGCAGT	102	84	0	0	0	0
FH	GTTGCAGTGTGTACGTCA	37	51	0	0	1	0
FH	GTACACACTGGTACGTAC	31	32	0	0	0	0
FH	TGACACACTGGTACGTTG	203	272	1	0	0	1
FH	ACACACACTGGTACCAGT	177	169	0	0	0	0
FH	TGACACACTGGTACCACA	57	55	0	0	0	0
FH	CATGCAACCAGTTGTGCA	243	305	0	1	0	0
FHIT	GTTGCACAGTACACACCA	127	277	0	0	2	1
FHIT	CAACACACCACACATGTG	62	61	0	0	0	0
FHIT	TGACACACCACAACGTGT	1419	1402	1	1	0	0
FHIT	GTACACACCACAACGTCA	320	460	2	0	0	3
FHIT	ACACACACCACAACGTAC	55	61	0	0	0	0
FHIT	CAACACACCACAACGTTG	175	218	0	1	0	0
FHL2	GTCATGTGACTGTGGTAC	291	113	6063	3	2	0
FHL2	TGCATGTGACTGACTGTG	180	182	1	0	0	0
FHL2	CACATGTGACTGTGGTGT	410	378	1	1	0	35
FHL2	ACCATGTGACTGTGGTCA	149	103	0	0	0	0
FHL2	GTTGCAGTGTTGACTGGT	941	506	1	1	0	4
FHL5	GTTGCATGGTACTGACGT	388	59	1	0	1	0
FHL5	CATGGTGTACCAGTGTCA	151	195	0	0	0	0
FHL5	TGTGGTGTACCAGTGTAC	21	11	0	0	0	0
FHL5	TGTGGTCATGACTGTGCA	230	230	0	0	0	0
FHL5	CATGGTCATGACTGTGAC	61	74	0	0	0	0
FHL5	GTTGGTCATGACTGTGTG	87	116	0	0	0	0
FIGF	CATGCAACCACATGTGTG	1136	878	4	1	2	0
FIGF	CAACTGGTTGACACCACA	17	98	0	0	0	0
FIGF	TGACTGGTTGACACCAAC	81	182	0	0	0	0
FIGF	ACACTGGTTGACACCATG	274	146	0	0	0	0
FIGF	CAACTGGTTGACACACGT	75	93	0	0	0	0
FIGF	ACACTGGTTGACACACCA	313	208	0	0	0	3
FKBP1A	TGCATGTGTGCAACCAGT	630	466	2	1	1	0
FKBP1A	TGCATGTGTGCAACGTCA	119	110	0	0	0	0
FKBP1A	CACATGTGTGCAACGTAC	46	55	0	0	0	0
FKBP1A	GTCATGTGTGCAACGTTG	553	701	1	0	0	3
FKBP1A	GTCATGTGTGCAACCACA	86	93	1	0	0	0
FKBP1A	CATGCAACGTTGGTCAAC	46	30	0	0	0	0
FKBP5	GTCATGCACACAACGTCA	507	459	0	0	0	0
FKBP5	ACCATGCACACAACGTAC	25	13	0	0	0	0
FKBP5	CACATGCACACAACGTTG	697	752	0	2	0	0
FKBP5	GTCATGCACACAACCAGT	528	325	1	0	0	0
FKBP5	CACATGCACACAACCACA	174	181	0	0	0	16
FKBP5	GTTGCAACGTTGGTCATG	95	114	0	0	0	0
FLAD1	ACACACGTCATGGTGTGT	71	44	0	0	0	0
FLAD1	TGACACGTCATGGTGTCA	33	38	0	0	0	0
FLAD1	CAACACGTCATGGTGTAC	56	150	0	0	0	0
FLAD1	GTACACGTCATGGTGTTG	396	500	0	0	0	0
FLAD1	TGACACGTCATGGTCAGT	112	72	0	0	0	1
FLCN	CACATGTGTGCAACTGGT	257	226	0	0	1	0
FLCN	TGCATGTGTGCAACACAC	9	12	0	0	0	0
FLCN	ACCATGTGTGCAACACTG	253	178	0	2	0	0
FLCN	ACCATGTGTGCAACTGCA	189	154	1	0	0	0
FLCN	TGTGGTACCAGTACACTG	334	130	0	0	0	0
FLI1	CACATGTGACCACATGTG	228	269	0	0	0	0
FLI1	TGCATGTGACCAACGTGT	363	348	0	0	0	0
FLI1	GTCATGTGACCAACGTCA	449	338	0	0	0	0
FLI1	ACCATGTGACCAACGTAC	189	93	0	1	0	9
FLI1	CACATGTGACCAACGTTG	160	115	0	0	0	0
FLI1	TGTGCACAACACTGGTGT	483	665	0	1	0	0
FLJ21865	ACTGGTCAACCAGTGTCA	920	726	0	0	2	1
FLJ21865	TGTGGTCAACCAGTGTTG	1109	1151	3	0	1	2
FLJ21865	GTTGGTCAACCAGTGTAC	201	100	0	1	0	0
FLJ21865	ACTGGTCAACCAGTCAGT	54	104	0	0	0	1
FLJ21865	TGTGGTCAACCAGTCACA	240	269	0	0	0	0
FLJ21865	ACTGCACATGACACACCA	305	288	0	0	0	1
FLNA	CAACTGACGTGTTGGTGT	327	333	1	3	4	5
FLNA	TGACTGACGTGTACTGTG	332	292	2	0	0	0
FLNA	ACACTGACGTGTTGGTCA	58	50	0	0	0	0
FLNA	GTACTGACGTGTTGGTAC	41	55	0	0	0	0
FLNA	CATGACACCAGTACTGCA	85	242	0	0	0	0
FLNC	GTACTGACGTGTACCACA	89	62	0	0	0	1

FLNC	ACACTGACGTGTACCAAC	9	13	0	0	0	0
FLNC	CAACTGACGTGTACCATG	132	74	0	0	0	1
FLNC	GTACTGACGTGTACACGT	57	44	0	0	0	0
FLNC	CAACTGACGTGTACACCA	123	88	0	0	0	0
FLOT1	ACACCAGTACACCATGGT	522	719	2	1	0	0
FLOT1	TGACCAGTACACCATGCA	192	326	0	1	0	0
FLOT1	CAACCAGTACACCATGAC	76	41	0	0	0	0
FLOT1	GTACCAGTACACCATGTG	66	60	0	0	0	0
FLOT1	ACACCAGTACACACGTGT	572	828	0	0	0	105
FLOT1	CATGCACAACACGTCATG	92	91	0	0	0	0
FLOT2	CATGGTCATGACACTGTG	985	312	1	0	1	1
FLOT2	GTTGGTCATGACACTGCA	71	91	0	0	0	0
FLOT2	ACTGGTCATGACACTGAC	56	87	0	0	0	0
FLOT2	TGTGGTCATGACTGGTGT	2056	2233	13	0	0	4
FLOT2	ACTGCAGTCAACACTGTG	105	113	0	0	0	0
FLT1	GTCATGCAACACGTTGGT	467	609	0	0	0	0
FLT1	CACATGCAACACGTTGCA	190	298	0	0	0	0
FLT1	TGCATGCAACACGTTGAC	28	30	0	0	0	0
FLT1	ACCATGCAACACGTTGTG	156	179	1	0	0	0
FLT1	GTCATGCAACACCAGTGT	453	545	1	0	0	1
FLT1	GTTGCATGCACACACAGT	141	56	0	1	0	0
FLT3	CACATGCAACTGCATGCA	102	88	0	0	0	1
FLT3	TGCATGCAACTGCATGAC	21	33	0	0	0	0
FLT3	ACCATGCAACTGCATGTG	190	192	1	0	0	0
FLT3	CAACCATGCAGTGTCAGT	188	173	0	0	0	9
FLT3	ACACCATGCAGTGTCACA	299	131	0	17	0	0
FLT3LG	GTACACACTGCACAACCA	345	299	1	2630	624	2
FLT3LG	ACACACACTGCACAACAC	79	34	0	0	0	0
FLT3LG	CAACACACTGCACAACTG	176	215	0	0	0	0
FLT3LG	GTACACACTGCACATGGT	246	409	1	0	0	0
FLT3LG	GTTGCAGTTGCAACACTG	195	582	1	1	0	0
FLT4	ACCATGCAACACCACACA	397	347	1	1335	18	1
FLT4	CACATGCAACACCAGTCA	351	287	2	554	0	0
FLT4	TGCATGCAACACCAGTAC	36	35	0	0	0	0
FLT4	ACCATGCAACACCAGTTG	74	76	0	0	0	0
FLT4	CACATGCAACACCACAGT	1084	902	1	0	0	2
FMN2	TGTGGTCACAGTTGGTTG	337	302	62982	66	7	8
FMN2	ACTGGTCACAGTTGGTCA	290	115	1	1	0	0
FMN2	GTTGGTCACAGTTGGTAC	138	83	1	1	0	0
FMN2	ACTGGTCACAGTTGCAGT	259	278	1	1	0	0
FMN2	TGTGGTCACAGTTGCACA	218	190	0	0	0	0
FMO1	TGACTGACACCAGTACAC	111	25	0	0	0	0
FMO1	ACACTGACACCAGTACTG	210	246	0	0	0	0
FMO1	CAACTGACACCAGTTGGT	398	561	0	1	0	3
FMO1	ACACTGACACCAGTTGCA	55	253	0	0	0	0
FMO1	GTTGCAGTTGTGCAACTG	621	915	0	0	0	1
FMO1	ACTGCACAGTACACACAC	30	43	0	0	0	0
FMO2	ACACTGACACCACAGTCA	408	370	0	1	0	0
FMO2	GTACTGACACCACAGTAC	20	47	0	0	0	0
FMO2	TGACTGACACCACAGTTG	363	244	1	0	0	37
FMO2	ACACTGACACCACACAGT	72	146	0	4	0	0
FMO2	TGACTGACACCACACACA	129	127	0	1	0	0
FMO3	TGACTGACACCACAACGT	383	249	1	0	2	0
FMO3	CAACTGACACCACACAAC	7	3	0	0	0	0
FMO3	GTACTGACACCACACATG	80	132	3	0	0	0
FMO3	GTACTGACACCACAACCA	447	389	0	7	0	1
FMO3	ACACTGACACCACAACAC	60	38	0	0	0	0
FMO4	TGACTGACACCAACGTAC	22	25	0	0	0	0
FMO4	ACACTGACACCAACGTTG	128	90	0	0	0	0
FMO4	CAACTGACACCAACCAGT	82	137	0	0	0	0
FMO4	ACACTGACACCAACCACA	89	154	0	0	0	1
FMO4	GTACTGACACCAACCAAC	160	166	0	0	0	0
FMO4	CATGCACAGTACACACTG	349	274	1	0	0	22
FMOD	ACTGGTACCAACACGTTG	380	727	2	0	0	0
FMOD	CATGGTACCAACACCAGT	282	299	0	0	0	0
FMOD	ACTGGTACCAACACCACA	475	344	0	1	0	0
FMOD	GTTGGTACCAACACCAAC	6	9	0	0	0	0
FMOD	TGTGGTACCAACACCATG	104	74	0	1	0	0
FN1	GTACTGACACTGCATGCA	237	263	0	0	0	2
FN1	ACACTGACACTGCATGAC	99	140	1	0	0	0
FN1	CAACTGACACTGCATGTG	316	1062	1	1	0	0
FN1	TGACTGACACTGACGTGT	746	1068	1	1	0	0
FN1	GTACTGACACTGACGTCA	328	326	854	0	0	0
FN3K	CAACCAACACTGACGTGT	620	286	1	0	0	10
FN3K	ACACCAACACTGACGTCA	172	122	0	0	0	0

FN3K	GTACCAACACTGACGTAC	39	38	1	0	0	0
FN3K	TGACCAACACTGACGTTG	462	419	0	0	0	0
FN3K	ACACCAACACTGACCAGT	502	318	0	0	0	1
FN3K	GTTGCATGCAGTGTCATG	84	129	0	0	0	1
FNTA	ACACCACACACATGTGCA	149	163	0	0	0	0
FNTA	GTACCACACACATGTGAC	58	185	0	0	0	0
FNTA	TGACCACACACATGTGTG	353	195	0	0	0	0
FNTA	CATGGTACTGTGTGGTGT	176	168	1	0	0	1
FNTA	CATGACCAGTTGGTCACA	72	80	0	0	0	0
FNTB	CAACCACACAACCAACCA	257	451	0	0	1	1
FNTB	ACACCACACAACCACAAC	15	30	0	0	0	0
FNTB	CAACCACACAACCACATG	193	340	0	0	0	0
FNTB	GTACCACACAACCAACGT	213	133	5	0	0	0
FNTB	TGACCACACAACCAACAC	77	54	0	0	0	0
FOLH1	TGACGTGTCACAGTGTTG	607	524	1	0	0	0
FOLH1	ACACGTGTCACAGTCAGT	98	134	0	0	0	0
FOLH1	TGACGTGTCACAGTCACA	93	79	0	0	0	1
FOLH1	CAACGTGTCACAGTCAAC	22	13	0	0	0	0
FOLH1	ACTGGTCAGTGTTGTGAC	52	51	0	0	0	0
FOLH1	GTTGCACACATGGTACCA	276	392	1	2	0	0
FOLR1	TGACTGCACATGTGTGCA	371	378	0	524	1	0
FOLR1	GTACTGCACATGTGACAC	52	98	0	0	0	0
FOLR1	TGACTGCACATGTGACTG	228	405	1	2	0	0
FOLR1	ACACTGCACATGTGTGGT	388	539	0	0	0	0
FOLR1	CAACTGCACATGTGTGAC	29	38	0	0	0	0
FOLR1	ACTGCACATGGTACACTG	595	438	0	0	0	0
FOS	GTACGTACCAACCAACTG	229	606	0	0	0	0
FOS	TGACGTACCAACCATGGT	99	150	0	0	0	1
FOS	GTACGTACCAACCATGCA	111	120	0	0	0	0
FOS	ACACGTACCAACCATGAC	136	120	1	1	0	0
FOS	CATGCAGTGTTGTGGTCA	148	210	2	1	0	0
FOS	GTTGCACAACACGTACGT	151	161	0	0	0	0
FOSB	TGACGTACCAACACACTG	427	472	2	360	823	6
FOSB	ACACGTACCAACACTGGT	317	309	0	2	0	0
FOSB	TGACGTACCAACACTGCA	305	444	0	0	0	0
FOSB	CAACGTACCAACACTGAC	63	59	1	0	0	0
FOSB	GTACGTACCAACACTGTG	364	422	0	0	0	5
FOSB	TGTGCACAACCACATGCA	158	258	0	0	0	0
FOSL1	GTACGTTGGTGTGTTGCA	91	436	0	0	0	1
FOSL1	ACACGTTGGTGTGTTGAC	13	9	0	0	0	0
FOSL1	CAACGTTGGTGTGTTGTG	136	167	0	0	0	0
FOSL1	TGACGTTGGTGTCAGTGT	409	257	0	0	0	0
FOSL1	CATGCAGTACACTGACCA	234	188	0	0	0	31
FOSL2	TGACGTACCAACTGACAC	93	106	0	0	0	0
FOSL2	ACACGTACCAACTGACTG	764	503	4	0	0	0
FOSL2	CAACGTACCAACTGTGGT	160	160	1	0	0	0
FOSL2	ACACGTACCAACTGTGCA	109	150	0	0	0	0
FOSL2	ACTGACGTGTCATGTGAC	18	38	0	0	0	0
FOXA1	GTTGCAACTGCATGTGGT	730	1427	4	2	1	0
FOXA1	TGACGTACGTACTGTGAC	35	33	0	0	0	0
FOXA1	ACACGTACGTACTGTGTG	50	38	0	0	0	0
FOXA1	CAACGTACGTTGGTGTGT	550	1105	0	0	0	0
FOXA1	ACACGTACGTTGGTGTCA	252	193	2	0	0	0
FOXA1	GTACGTACGTTGGTGTAC	33	50	0	0	0	0
FOXA2	CAACGTACGTTGGTACTG	213	155	0	0	0	0
FOXA2	GTACGTACGTTGGTTGGT	272	288	0	1	0	0
FOXA2	CAACGTACGTTGGTTGCA	65	109	0	0	0	0
FOXA2	TGACGTACGTTGGTTGAC	12	28	0	0	0	0
FOXA2	ACTGCAGTACACACCATG	404	497	2	1	0	1
FOXA2	CATGCAACTGCATGTGCA	83	76	2	0	0	0
FOXA3	CAACGTACGTTGCACAGT	121	98	0	0	0	1
FOXA3	ACACGTACGTTGCACACA	100	64	0	0	0	0
FOXA3	GTACGTACGTTGCACAAC	31	14	0	0	0	0
FOXA3	TGACGTACGTTGCACATG	123	302	0	0	0	0
FOXA3	ACACGTACGTTGCAACGT	147	184	0	0	0	0
FOXA3	TGTGCAACTGCATGTGAC	30	63	0	0	0	0
FOXC2	TGACGTCATGACACCAAC	17	17	2	0	0	0
FOXC2	ACACGTACCAGTACACTG	202	300	0	0	0	0
FOXC2	CAACGTACCAGTACTGGT	117	197	0	0	0	0
FOXC2	ACACGTACCAGTACTGCA	120	141	0	0	0	5
FOXC2	GTACGTACCAGTACTGAC	29	28	0	0	0	0
FOXC2	ACTGCACACATGTGCAAC	19	9	0	0	0	0
FOXD3	GTACGTACCAGTTGACCA	165	169	0	0	0	0
FOXD3	ACACGTACCAGTTGACAC	39	79	0	0	0	0
FOXD3	CAACGTACCAGTTGACTG	351	308	0	0	0	0

FOXD3	GTACGTACCAGTTGTGGT	1352	664	1	1	0	0
FOXD3	ACACGTACACACACTGTG	239	239	0	1	0	0
FOXD3	TGTGCACAACACCATGAC	85	124	0	0	0	0
FOXH1	GTACGTCAACTGGTGTCA	466	315	1	0	0	1
FOXH1	ACACGTCAACTGGTGTAC	64	52	0	0	0	0
FOXH1	CAACGTCAACTGGTGTTG	683	496	2	2	0	9
FOXH1	GTACGTCAACTGGTCAGT	589	183	0	0	0	0
FOXH1	CATGCAGTACACCAACAC	115	125	0	2	0	0
FOXH1	ACTGCACAGTACACCAGT	123	98	0	0	0	0
FOXK1	GTACGTTGCAACCAACCA	113	223	0	0	0	1
FOXK1	ACACGTTGCAACCAACAC	64	53	5	1	0	1
FOXK1	CATGGTCACAACCATGTG	44	65	0	0	0	0
FOXK1	CATGGTACTGTGTGCAAC	42	14	0	0	0	0
FOXK1	GTTGGTACTGTGTGCATG	32	13	1	0	0	0
FOXL1	ACACGTCATGACACGTAC	183	108	0	0	0	0
FOXL1	CAACGTCATGACACGTTG	320	118	1	0	0	0
FOXL1	GTACGTCATGACACCAGT	265	260	0	0	0	0
FOXL1	CAACGTCATGACACCACA	96	363	0	1	0	0
FOXL1	CATGACACGTGTACGTCA	17	20	0	0	0	0
FOXM1	ACACGTACCAGTTGTGTG	286	434	0	0	2	0
FOXM1	GTACGTACCACAGTGTCA	450	347	1	0	1	0
FOXM1	CAACGTACCAGTTGTGCA	235	182	1	2	0	0
FOXM1	TGACGTACCAGTTGTGAC	14	23	0	0	0	0
FOXM1	TGACGTACCACAGTGTGT	588	420	0	0	0	0
FOXN1	GTACGTCAACACCACATG	177	85	0	0	0	0
FOXN1	TGACGTCAACACCAACGT	1165	787	3	10	0	2
FOXN1	GTACGTCAACACCAACCA	536	231	1	0	0	1
FOXN1	ACACGTCAACACCAACAC	250	66	0	0	0	0
FOXN1	CAACGTCAACACCAACTG	301	284	1	1	0	0
FOXN1	GTTGCAACGTTGTGACGT	49	82	0	0	0	1
FOXO1	GTACGTTGCAACACGTGT	359	404	0	0	0	0
FOXO1	TGACCATGGTACTGTGAC	50	63	0	0	0	0
FOXO1	ACACCATGGTACTGTGTG	220	182	0	0	0	4
FOXO1	CAACCATGGTTGGTGTGT	91	95	0	0	0	1
FOXO1	ACACCATGGTTGGTGTCA	315	263	1	0	0	0
FOXO3	GTACCATGCATGTGGTCA	224	453	3	6	2	8
FOXO3	GTACCATGCATGTGCAGT	188	456	0	0	1	1
FOXO3	TGACCATGCATGTGGTGT	489	607	0	0	0	1
FOXO3	ACACCATGCATGTGGTAC	99	129	0	0	0	5
FOXO3	CAACCATGCATGTGGTTG	99	126	0	0	0	1
FOXO4	TGACCATGCAGTCAGTGT	380	362	0	0	0	0
FOXO4	GTACCATGCAGTCAGTCA	254	292	0	0	0	1
FOXO4	ACACCATGCAGTCAGTAC	169	363	0	0	0	0
FOXO4	CAACCATGCAGTCAGTTG	156	238	1	1	0	0
FOXO4	GTACCATGCAGTCACAGT	65	58	0	0	0	0
FOXP1	CAACGTACCACACATGGT	419	267	0	0	1	0
FOXP1	GTACGTACCACACATGAC	179	232	6	3699	1	0
FOXP1	TGACGTACCACACAACAC	20	14	0	0	0	0
FOXP1	ACACGTACCACACAACTG	466	754	0	0	0	0
FOXP1	ACACGTACCACACATGCA	435	755	1	0	0	2
FOXP3	ACACGTACTGCAGTGTTG	133	178	0	0	1	0
FOXP3	CAACGTACTGCAGTGTCA	234	283	0	0	0	0
FOXP3	TGACGTACTGCAGTGTAC	92	96	0	0	0	0
FOXP3	CAACGTACTGCAGTCAGT	397	241	0	0	0	0
FOXP3	ACACGTACTGCAGTCACA	43	67	0	0	0	0
FOXP3	ACTGCATGCACAGTACGT	140	153	0	0	0	0
FPGS	CAACACGTACACTGCAGT	580	233	0	0	1	3
FPGS	ACACACGTACACTGCACA	148	186	0	0	0	3
FPGS	GTACACGTACACTGCAAC	61	76	0	0	0	0
FPGS	TGACACGTACACTGCATG	784	631	1	116	0	0
FPGS	ACTGCAGTGTCAGTGTTG	171	133	0	0	0	0
FPGT	TGACCAACGTCAACCAAC	41	40	0	0	1	0
FPGT	CAACCAACGTCAACGTTG	366	479	0	0	0	0
FPGT	GTACCAACGTCAACCAGT	116	51	0	0	0	0
FPGT	CAACCAACGTCAACCACA	114	84	0	0	0	0
FPGT	ACACCAACGTCAACCATG	194	171	2	0	0	2
FPR1	ACACGTCAGTCAACCATG	208	138	0	0	1	0
FPR1	GTACGTCAGTCAACCAGT	259	392	0	2	0	0
FPR1	CAACGTCAGTCAACCACA	12	11	0	0	0	0
FPR1	TGACGTCAGTCAACCAAC	11	28	0	0	0	0
FPR1	CAACGTCAGTCAACACGT	27	29	0	0	0	0
FRAP1	TGACCATGGTCACACACA	90	70	0	0	0	0
FRAP1	CAACCATGGTCACACAAC	19	15	0	0	0	0
FRAP1	GTACCATGGTCACACATG	85	105	0	0	0	0
FRAP1	TGACCATGGTCACAACGT	135	490	0	0	0	0

FRAP1	GTACCATGGTCACAACCA	104	89	0	0	0	0
FRAP1	CATGCAACCAACGTGTGT	443	923	1	0	0	0
FRAT1	GTACTGACGTGTACGTTG	392	644	0	1	1	0
FRAT1	TGACTGACGTGTACGTCA	95	76	0	0	0	1
FRAT1	CAACTGACGTGTACGTAC	65	61	0	0	0	0
FRAT1	TGACTGACGTGTACCAGT	204	122	0	0	0	0
FRAT1	TGTGCAGTTGTGGTCACA	91	65	0	0	0	0
FRAT1	ACTGCATGGTTGCATGTG	64	24	0	0	0	0
FRAT2	CATGCACAGTGTCACAAC	96	50	0	0	1	0
FRAT2	ACTGGTGTCAGTTGCACA	145	100	0	0	0	0
FRAT2	CATGGTGTCAACCAACGT	569	357	1	0	0	0
FRAT2	ACTGGTGTCAACCAACCA	199	298	0	0	0	0
FRAT2	GTTGGTGTCAACCAACAC	0	0	0	0	0	0
FRAT2	GTTGCATGGTTGACGTGT	411	343	2	0	0	0
FRS2	GTACTGCATGGTTGGTTG	1087	399	1	2	4	5
FRS2	TGACTGCATGGTTGCAGT	185	144	0	0	0	0
FRS2	GTACTGCATGGTTGCACA	158	167	0	0	0	0
FRS2	ACACTGCATGGTTGCAAC	21	24	0	0	0	0
FRS2	CATGGTGTCAGTTGGTCA	199	335	0	0	0	0
FRZB	ACACGTACGTCAGTGTGT	569	516	1	0	1	1
FRZB	GTACGTACGTGTTGTGCA	290	217	0	0	0	0
FRZB	ACACGTACGTGTTGTGAC	32	55	0	0	0	0
FRZB	CAACGTACGTGTTGTGTG	130	88	0	0	0	0
FRZB	TGACGTACGTCAGTGTCA	398	538	1	0	0	0
FSCN1	CATGGTCACAGTTGACTG	883	466	1022	2	2	0
FSCN1	GTTGGTCACAGTTGTGGT	836	1621	2	0	0	38
FSCN1	CATGGTCACAGTTGTGCA	57	84	0	0	0	0
FSCN1	TGTGGTCACAGTTGTGAC	57	41	0	0	0	0
FSCN1	ACTGGTCACAGTTGTGTG	867	348	1	0	0	1
FSCN2	CATGGTCAGTTGCACAGT	381	250	0	0	3	9
FSCN2	ACTGGTCAGTTGCACACA	109	152	0	2	1	0
FSCN2	CATGGTCAGTTGCAGTCA	119	167	0	0	0	1
FSCN2	TGTGGTCAGTTGCAGTAC	16	23	0	0	0	0
FSCN2	ACTGGTCAGTTGCAGTTG	409	455	0	0	0	0
FSCN3	TGTGGTGTACGTACTGCA	196	214	3	0	0	1
FSCN3	GTTGGTACACACGTCAAC	131	40	1	0	0	0
FSCN3	TGTGGTACACACGTCATG	115	143	0	0	0	0
FSCN3	ACTGGTACACACGTACGT	126	150	0	0	0	1
FSCN3	TGTGGTACACACGTACCA	302	299	0	0	0	0
FSCN3	GTTGCACATGCAGTACAC	36	34	0	0	0	0
FSHB	TGTGGTGTGTGTGTTGTG	279	321	2	0	0	0
FSHB	CATGGTGTGTGTCAGTGT	1225	1304	3	0	0	23
FSHB	ACTGCACAGTGTGTGTGT	210	223	0	1	0	0
FSHB	TGTGCACAGTGTGTGTCA	388	636	2	0	0	6
FSHB	ACTGACCAGTACTGACCA	129	111	0	0	0	0
FSHR	GTCATGACTGTGACGTGT	891	855	0	2	1	1
FSHR	GTCATGACTGTGCATGGT	561	513	0	1	0	0
FSHR	CACATGACTGTGCATGCA	39	77	0	0	0	0
FSHR	TGCATGACTGTGCATGAC	22	28	0	0	0	0
FSHR	ACCATGACTGTGCATGTG	200	285	0	0	0	0
FSHR	TGTGCATGCAGTTGCAGT	102	69	0	0	0	0
FST	TGACTGGTACGTTGCAGT	103	94	0	0	0	0
FST	GTACTGGTACGTTGCACA	15	159	0	0	0	0
FST	ACACTGGTACGTTGCAAC	12	23	0	0	0	0
FST	CAACTGGTACGTTGCATG	31	67	0	0	0	0
FST	CATGCAGTTGACCAACGT	283	718	1	0	0	1
FST	ACTGCACAACACTGGTAC	138	162	0	0	0	2
FSTL1	ACACACTGGTACACTGTG	185	222	0	0	1	0
FSTL1	ACACGTCAACCACACATG	73	114	0	0	0	0
FSTL1	GTACACTGGTACTGGTGT	455	909	1	0	0	0
FSTL1	CAACACTGGTACTGGTCA	83	105	1	0	0	0
FSTL1	TGACACTGGTACTGGTAC	29	53	0	0	0	0
FTCD	ACACCACAACCATGCACA	779	504	1	1	1	20
FTCD	TGACCACAACCATGGTAC	142	130	0	0	0	0
FTCD	ACACCACAACCATGGTTG	240	256	0	0	0	0
FTCD	CAACCACAACCATGCAGT	216	196	0	0	0	0
FTCD	GTACCACAACCATGCAAC	30	44	0	0	0	0
FTH1	ACACCAGTACTGACCATG	213	244	1	0	0	0
FTH1	CAACCAGTACTGACACGT	98	96	1	0	0	0
FTH1	ACACCAGTACTGACACCA	319	381	0	0	0	0
FTH1	GTACCAGTACTGACACAC	28	25	0	0	0	0
FTH1	ACTGACGTTGCACAACGT	13	15	0	0	0	0
FTMT	CATGGTTGGTCATGTGCA	212	407	7	0	1	3
FTMT	TGTGGTTGGTCATGTGAC	31	14	0	0	0	0
FTMT	ACTGGTTGGTCATGTGTG	548	241	0	2	0	5

FTMT	ACTGGTTGGTACGTGTGT	823	496	2	0	0	1
FTMT	TGTGGTTGGTACGTGTCA	829	648	1	0	0	22
FTMT	GTTGCATGGTACGTGTAC	22	29	0	0	0	0
FTO	TGTGGTTGACACCACATG	83	87	0	29	0	0
FTO	TGTGCAGTGTACCATGAC	118	150	0	2	0	0
FTO	ACTGCAGTGTACCATGTG	553	491	0	0	0	0
FTO	GTTGCAGTGTACACGTGT	351	247	0	0	0	0
FTO	CATGCAGTGTACACGTCA	186	332	1	0	0	1
FUCA1	GTTGGTCAGTACACGTTG	300	267	0	0	0	6
FUCA1	TGTGGTCAGTACACCAGT	177	317	0	1	0	13
FUCA1	GTTGGTCAGTACACCACA	143	107	0	1	0	0
FUCA1	ACTGGTCAGTACACCAAC	34	15	0	0	0	0
FUCA1	CATGGTCAGTACACCATG	124	115	0	0	0	0
FUCA2	TGTGGTTGCAACCAGTCA	130	136	0	0	0	0
FUCA2	CATGGTTGCAACCAGTAC	61	84	0	0	0	0
FUCA2	GTTGGTTGCAACCAGTTG	423	880	0	0	0	11
FUCA2	TGTGGTTGCAACCACAGT	45	49	0	0	0	0
FUCA2	GTTGGTTGCAACCACACA	48	67	0	0	0	0
FUK	ACACCAACTGGTTGACAC	69	73	0	0	0	0
FUK	CAACCAACTGGTTGACTG	644	309	0	1	0	0
FUK	GTACCAACTGGTTGTGGT	158	165	0	0	0	0
FUK	CAACCAACTGGTTGTGCA	218	192	0	0	0	1
FUK	TGACCAACTGGTTGTGAC	66	252	0	0	0	0
FURIN	GTACCAGTTGTGACACGT	235	249	2	1	1	57
FURIN	ACACTGTGCACAGTTGTG	308	146	0	1	0	0
FURIN	GTACTGTGCACACAGTGT	387	310	2	0	0	0
FURIN	CAACTGTGCACACAGTCA	787	699	3	0	0	0
FURIN	TGACTGTGCACACAGTAC	48	95	0	0	0	0
FURIN	TGTGCAACCAACACACTG	786	324	0	1	0	0
FUT1	ACACCAACCAGTGTACCA	238	213	0	0	0	2
FUT1	GTACCAACCAGTGTACAC	55	83	0	1	0	0
FUT1	TGACCAACCAGTGTACTG	1135	1543	1	0	0	13
FUT1	ACACCAACCAGTGTTGGT	340	334	1	0	0	0
FUT1	TGACCAACCAGTGTTGCA	348	129	0	0	0	0
FUT2	ACACCAACCAGTCATGCA	182	279	0	0	1	0
FUT2	GTACCAACCAGTCATGAC	38	24	0	0	0	0
FUT2	TGACCAACCAGTCATGTG	278	380	0	0	0	0
FUT2	GTTGACACACTGGTGTCA	350	270	0	0	0	1
FUT2	ACTGACACACTGGTGTAC	61	80	0	0	0	0
FUT3	GTACCAACGTACGTTGTG	560	912	0	0	0	0
FUT3	ACACCAACGTACCAGTGT	334	310	0	0	0	0
FUT3	TGACCAACGTACCAGTCA	185	127	0	1	0	0
FUT3	ACTGCAGTACTGCAGTCA	163	152	0	0	0	0
FUT3	CATGACACACTGGTACGT	385	420	0	0	0	1
FUT4	ACTGGTTGCAACCACAAC	3	3	0	0	0	0
FUT4	CATGGTTGCAACCACATG	93	89	0	0	0	1
FUT4	GTTGGTTGCAACCAACGT	8	6	0	0	0	0
FUT4	CATGGTTGCAACCAACCA	113	112	0	0	0	0
FUT4	TGTGGTTGCAACCAACAC	35	24	0	0	0	0
FUT5	CAACCAACGTACCAGTAC	101	101	0	0	0	0
FUT5	ACACCAACGTACCATGCA	816	830	1	0	0	0
FUT5	CAACCAACGTACTGCAGT	373	350	0	0	0	0
FUT5	ACACCAACGTACTGCACA	178	267	0	1	0	0
FUT5	GTACCAACGTACTGCAAC	19	22	0	0	0	0
FUT6	TGACCAACGTACCATGTG	536	303	0	0	1	11
FUT6	ACACCAACGTACCAACTG	288	333	0	0	0	0
FUT6	CAACCAACGTACCATGGT	285	234	1	0	0	0
FUT6	GTACCAACGTACCATGAC	92	56	0	0	0	0
FUT6	TGACCAACGTACTGCATG	331	469	0	0	0	0
FUT7	GTACCAACCACAACACTG	947	894	3	0	1	1
FUT7	TGACCAACCACAACTGGT	317	379	0	1	0	97
FUT7	GTACCAACCACAACTGCA	234	222	1	0	0	0
FUT7	ACACCAACCACAACTGAC	120	51	0	1	0	0
FUT7	CAACCAACCACAACTGTG	478	433	1	0	0	0
FUT8	GTACCAACGTTGGTCAAC	21	5	0	0	0	1
FUT8	TGACCAACGTTGGTCATG	158	256	0	0	0	0
FUT8	ACACCAACGTTGGTACGT	147	75	1	0	0	0
FUT8	TGACCAACGTTGGTACCA	123	135	0	0	0	0
FUT8	CAACCAACGTTGGTACAC	39	33	0	0	0	0
FUT8	GTTGCATGGTACTGCACA	39	31	0	0	0	0
FUT9	ACACCAACGTTGACACTG	1863	933	4	0	1	9
FUT9	CATGGTTGTGGTGTGTTG	307	412	0	1	1	0
FUT9	GTACCAACGTTGACACGT	31	63	0	0	0	0
FUT9	CAACCAACGTTGACACCA	128	126	0	0	0	0
FUT9	TGACCAACGTTGACACAC	28	16	1	0	0	0

FVT1	GTACTGACACTGACTGTG	10	38	0	0	0	0
FVT1	ACACTGACACTGTGGTGT	413	505	1	0	0	0
FVT1	TGACTGACACTGTGGTCA	305	151	0	0	0	1
FVT1	CAACTGACACTGTGGTAC	105	123	0	1	0	0
FVT1	GTACTGACACTGTGGTTG	270	165	0	0	0	0
FVT1	GTTGCACAGTACACTGGT	8	122	0	0	0	0
FXYD2	GTACCATGACACCAACGT	497	747	0	0	0	2
FXYD2	CAACCATGACACCAACCA	238	330	0	0	0	0
FXYD2	TGACCATGACACCAACAC	121	114	0	0	0	0
FXYD2	ACACCATGACACCAACTG	487	556	0	0	0	0
FXYD2	CAACCATGACACCATGGT	371	331	1	0	0	0
FXYD2	GTTGCATGGTGTCAGTCA	225	199	0	0	0	0
FYN	TGCATGACACCACAACTG	1470	1414	2	2	4	1
FYN	ACCATGACACCACAACCA	234	275	0	0	0	7
FYN	GTCATGACACCACAACAC	18	21	0	0	0	0
FYN	ACCATGACACCACATGGT	214	318	1	0	0	0
FYN	TGACGTTGTGACGTACTG	218	344	0	0	0	0
FYN	ACTGCAACTGGTACCATG	116	227	0	0	0	0
FZD1	CAACGTCAGTCAACTGAC	34	29	0	0	0	0
FZD1	GTACGTCAGTCAACTGTG	745	160	1	0	0	0
FZD1	ACACGTCAGTCATGGTGT	660	566	1	0	0	4
FZD1	TGACGTCAGTCATGGTCA	228	299	0	0	0	0
FZD1	CAACGTCAGTCATGGTAC	156	208	0	0	0	0
FZD1	CATGCACAGTACACTGCA	150	139	1	0	0	0
FZD10	ACACGTCAGTCATGCAAC	191	56	14	9727	735	1
FZD10	GTACGTCAGTCATGGTTG	370	412	0	1	0	0
FZD10	TGACGTCAGTCATGCAGT	200	133	0	0	0	0
FZD10	GTACGTCAGTCATGCACA	47	34	0	0	0	0
FZD10	CAACGTCAGTCATGCATG	220	384	0	7	0	1
FZD10	CATGCAACACACACGTGT	1516	1936	6	1147	0	0
FZD2	GTACGTCAGTCATGACGT	188	151	0	0	0	0
FZD2	CAACGTCAGTCATGACCA	188	217	0	1	0	3
FZD2	TGACGTCAGTCATGACAC	45	47	0	0	0	0
FZD2	ACACGTCAGTCATGACTG	140	223	0	1	0	1
FZD2	TGTGCACAACCAACCAGT	337	269	0	0	0	9
FZD2	GTTGCACAACCAACCACA	68	81	0	0	0	0
FZD3	ACTGCAACACACACGTCA	276	196	0	0	1	0
FZD3	CAACGTCAGTCATGTGGT	378	321	1	1	0	0
FZD3	ACACGTCAGTCATGTGCA	161	177	0	0	0	0
FZD3	GTACGTCAGTCATGTGAC	48	28	0	0	0	0
FZD3	TGACGTCAGTCATGTGTG	290	642	0	0	0	0
FZD3	TGACGTCAGTACGTGTGT	67	55	0	0	0	0
FZD4	GTACGTCAGTACGTGTCA	121	335	1	0	0	0
FZD4	ACACGTCAGTACGTGTAC	15	10	0	0	0	0
FZD4	CAACGTCAGTACGTGTTG	464	375	1	0	0	0
FZD4	GTACGTCAGTACGTCAGT	16	47	0	0	0	0
FZD4	CAACGTCAGTACGTCACA	68	107	0	0	0	0
FZD4	TGTGCAACACTGCACAAC	5	10	0	0	0	0
FZD5	TGACGTCAGTACGTCAAC	5	0	0	0	0	0
FZD5	ACACGTCAGTACGTCATG	291	193	0	0	0	0
FZD5	CAACGTCAGTACGTACGT	110	100	0	0	0	0
FZD5	ACACGTCAGTACGTACCA	299	355	0	0	0	0
FZD5	ACTGCAGTACCATGGTTG	530	366	1	0	0	0
FZD6	GTACGTCAGTACGTACAC	27	22	0	0	0	0
FZD6	TGACGTCAGTACGTACTG	148	183	0	0	0	0
FZD6	ACACGTCAGTACGTTGGT	194	208	0	0	0	0
FZD6	TGACGTCAGTACGTTGCA	93	102	1	0	0	0
FZD6	CAACGTCAGTACGTTGAC	16	13	0	0	0	0
FZD7	GTACGTCAGTACCAGTTG	1409	840	3	0	1	0
FZD7	GTACGTCAGTACGTTGTG	318	213	1	0	0	0
FZD7	ACACGTCAGTACCAGTGT	326	337	2	0	0	1
FZD7	TGACGTCAGTACCAGTCA	34	36	0	0	0	0
FZD7	CAACGTCAGTACCAGTAC	89	73	0	0	0	12
FZD7	TGTGCACAGTACACTGAC	36	21	0	0	0	0
FZD8	ACACGTCAGTACCACAAC	89	31	0	0	1	0
FZD8	TGACGTCAGTACCACAGT	202	507	0	0	0	0
FZD8	GTACGTCAGTACCACACA	9	10	0	0	0	0
FZD8	CAACGTCAGTACCACATG	94	134	1	0	0	0
FZD8	GTACGTCAGTACCAACGT	319	455	0	0	0	0
FZD8	TGTGCAACGTGTCAACAC	102	68	1	0	0	0
FZD9	ACACGTCAGTACCAACTG	689	640	0	2	1	0
FZD9	CAACGTCAGTACCAACCA	190	150	1	0	0	0
FZD9	TGACGTCAGTACCAACAC	47	39	0	0	0	0
FZD9	CAACGTCAGTACCATGGT	346	398	0	0	0	0
FZD9	ACACGTCAGTACCATGCA	419	547	0	1	0	0

FZR1	CACATGTGGTTGTGCATG	99	164	3	0	0	0
FZR1	GTCATGTGGTTGTGACGT	321	520	1	0	0	0
FZR1	TGTGGTTGTGGTACTGAC	209	91	0	0	0	1
FZR1	ACTGGTTGTGGTACTGTG	50	74	0	0	0	0
FZR1	GTTGGTTGTGGTTGGTGT	835	960	0	2	0	0
G0S2	ACTGGTTGCAACCAACTG	85	232	0	0	0	0
G0S2	CATGGTTGCAACCATGGT	264	308	0	0	0	0
G0S2	ACTGGTTGCAACCATGCA	56	105	0	1	0	0
G0S2	GTTGGTTGCAACCATGAC	62	54	0	0	0	0
G0S2	TGTGGTTGCAACCATGTG	918	414	3	0	0	57
G0S2	GTTGCAACCAACGTACCA	277	141	0	2	0	0
G3BP2	CAACACCAGTTGTGGTTG	315	314	0	0	0	0
G3BP2	GTACACCAGTTGTGCAGT	148	159	0	0	0	0
G3BP2	CAACACCAGTTGTGCACA	122	130	1	0	0	0
G3BP2	TGACACCAGTTGTGCAAC	73	31	0	0	0	0
G3BP2	ACACACCAGTTGTGCATG	54	64	0	0	0	0
G6PC	TGTGCAGTTGTGACGTGT	611	435	3	0	1	0
G6PC	TGACACACACCAGTTGCA	75	53	0	0	0	0
G6PC	CAACACACACCAGTTGAC	27	16	1	0	0	0
G6PC	GTACACACACCAGTTGTG	81	124	0	1	0	0
G6PC	ACACACACACCACAGTGT	735	531	0	0	0	0
G6PC2	GTACACCATGACTGCAAC	30	30	0	0	0	0
G6PC2	TGACACCATGACTGCATG	161	175	0	0	0	0
G6PC2	ACACACCATGACTGACGT	295	549	1	0	0	0
G6PC2	TGACACCATGACTGACCA	156	136	0	0	0	0
G6PC2	CAACACCATGACTGACAC	85	73	0	0	0	0
G6PD	CAACCAGTGTACGTACTG	76	114	0	0	0	0
G6PD	GTACCAGTGTACGTTGGT	544	498	0	0	0	0
G6PD	CAACCAGTGTACGTTGCA	329	218	1	0	0	2
G6PD	TGACCAGTGTACGTTGAC	66	172	0	0	0	0
G6PD	ACACCAGTGTACGTTGTG	184	177	0	0	0	0
GAA	CAACACCATGGTGTACCA	373	385	1	0	0	0
GAA	TGACACCATGGTGTACAC	7	21	0	0	0	0
GAA	ACACACCATGGTGTACTG	3	40	0	0	0	0
GAA	CAACACCATGGTGTTGGT	181	219	0	0	0	0
GAA	GTTGCAGTCATGTGCATG	179	218	1	0	0	0
GAB1	ACACTGTGGTACTGACCA	122	164	0	0	0	0
GAB1	GTACTGTGGTACTGACAC	108	20	0	1	0	0
GAB1	TGACTGTGGTACTGACTG	215	224	2	1	0	1
GAB1	ACACTGTGGTACTGTGGT	189	165	0	0	0	1
GAB1	TGACTGTGGTACTGTGCA	52	47	1	0	0	0
GAB2	ACTGGTACGTACCAACGT	134	167	21	18519	5	8
GAB2	ACTGCAACGTTGTGGTGT	961	357	1	0	1	2
GAB2	TGTGGTACGTACCAACCA	21	28	0	0	0	0
GAB2	CATGGTACGTACCAACAC	48	67	0	0	0	0
GAB2	GTTGGTACGTACCAACTG	162	107	0	0	0	0
GAB2	GTTGCAACGTTGACTGTG	158	141	0	0	0	0
GABARAP	TGACTGCAACCATGGTCA	307	268	0	1	1	1
GABARAP	CAACTGCAACCATGGTAC	119	233	0	0	0	0
GABARAP	GTACTGCAACCATGGTTG	481	892	1	0	0	0
GABARAP	TGTGCAGTCAGTACCATG	1100	331	0	1	0	1
GABARAP	ACTGCAGTCAGTACACGT	126	106	0	0	0	0
GABARAP	ACTGCACAACCAACCAAC	243	106	0	0	0	8
GABARAPL1	GTACTGCAACCACACAAC	116	123	1	0	0	0
GABARAPL1	TGACTGCAACCACACATG	385	327	0	1	0	0
GABARAPL1	ACACTGCAACCACAACGT	241	338	1	0	0	1
GABARAPL1	TGACTGCAACCACAACCA	395	171	0	0	0	0
GABARAPL1	ACTGGTCACAGTGTCACA	47	54	0	0	0	0
GABARAPL2	TGACACCACAACCAACGT	526	540	0	0	0	1
GABARAPL2	GTACACCACAACCAACCA	323	215	1	0	0	1
GABARAPL2	ACACACCACAACCAACAC	41	40	0	0	0	0
GABARAPL2	CAACACCACAACCAACTG	484	505	1	1	0	0
GABARAPL2	GTACACCACAACCATGGT	395	755	0	0	0	0
GABARAPL2	GTTGCAACTGTGACGTAC	56	47	0	0	0	0
GABBR1	GTACGTCAGTACACGTAC	30	26	0	0	0	0
GABBR1	TGACGTCAGTACACGTTG	194	116	0	0	0	0
GABBR1	ACACGTCAGTACACCAGT	340	255	1	0	0	1
GABBR1	TGACGTCAGTACACCACA	82	113	0	0	0	0
GABBR1	CAACGTCAGTACACCAAC	40	42	0	0	0	0
GABBR1	ACTGCACATGACCACACA	76	61	0	0	0	5
GABPA	TGACGTACACTGCACAAC	24	24	0	0	0	0
GABPA	ACACGTACACTGCACATG	174	158	0	0	0	1
GABPA	CAACGTACACTGCAACGT	120	129	0	1	0	0
GABPA	ACACGTACACTGCAACCA	310	451	0	2	0	0
GABPA	GTACGTACACTGCAACAC	28	18	0	0	0	0

GABRA1	CAACTGCAACACCAGTAC	25	29	0	0	0	0
GABRA1	GTACTGCAACACCAGTTG	611	639	1	0	0	30
GABRA1	TGACTGCAACACCACAGT	211	335	0	0	0	0
GABRA1	GTACTGCAACACCACACA	99	187	0	0	0	0
GABRA1	CATGCATGACTGTGTGGT	340	277	1	1	0	0
GABRA2	TGACTGCAACACCAACAC	31	23	0	0	0	0
GABRA2	ACACTGCAACACCAACTG	405	739	4	1	0	1
GABRA2	CAACTGCAACACCATGGT	480	745	3	0	0	0
GABRA2	ACACTGCAACACCATGCA	70	94	1	0	0	0
GABRA2	GTACTGCAACACCATGAC	99	115	1	1	0	0
GABRA2	CATGCACACAACGTCAGT	954	441	0	0	0	1
GABRA3	ACACTGCAACACACCAGT	84	208	0	0	0	0
GABRA3	TGACTGCAACACACCACA	54	59	0	0	0	0
GABRA3	CAACTGCAACACACCAAC	83	47	0	0	0	0
GABRA3	GTACTGCAACACACCATG	133	153	0	0	0	0
GABRA3	ACTGCAGTTGACTGACAC	164	143	0	0	0	0
GABRA4	GTACTGCAACACACTGGT	952	469	0	0	0	0
GABRA4	CAACTGCAACACACTGCA	115	119	0	0	0	0
GABRA4	TGACTGCAACACACTGAC	27	32	0	0	0	2
GABRA4	ACACTGCAACACACTGTG	131	108	0	0	0	0
GABRA4	GTACTGCAACACTGGTGT	307	220	0	0	0	0
GABRA5	GTTGCACAACCATGACCA	425	361	0	1	1	0
GABRA5	GTACTGCAACACTGTGCA	664	584	0	0	0	1
GABRA5	ACACTGCAACACTGTGAC	79	20	0	1	0	0
GABRA5	CAACTGCAACACTGTGTG	148	232	0	0	0	0
GABRA5	GTACTGCAACTGGTGTGT	103	195	0	0	0	0
GABRA5	CAACTGCAACTGGTGTCA	609	711	1	0	0	0
GABRA6	GTACTGCAACTGGTACTG	218	210	6	3711	2	0
GABRA6	TGACTGCAACTGGTTGGT	358	502	1	0	0	3
GABRA6	GTACTGCAACTGGTTGCA	127	100	0	0	0	0
GABRA6	ACACTGCAACTGGTTGAC	16	39	0	0	0	0
GABRA6	ACTGCACACAACGTCACA	53	121	0	0	0	0
GABRA6	GTTGCACACAACGTCAAC	0	1	0	0	0	0
GABRB1	ACACTGACCAGTACTGGT	177	205	0	0	0	2
GABRB1	TGACTGACCAGTACTGCA	322	63	1	0	0	0
GABRB1	CAACTGACCAGTACTGAC	78	80	0	0	0	0
GABRB1	GTACTGACCAGTACTGTG	181	183	0	1	0	0
GABRB1	GTTGCAGTTGTGCAGTGT	621	882	2	0	0	1
GABRB1	GTTGCACATGACTGGTTG	450	1012	2	1028	0	2
GABRB2	CAACTGCAACTGTGGTGT	645	363	0	1	1	1
GABRB2	TGACTGCAACTGACTGTG	26	28	0	0	0	0
GABRB2	ACACTGCAACTGTGGTCA	398	421	0	0	0	0
GABRB2	GTACTGCAACTGTGGTAC	147	31	0	0	0	0
GABRB2	TGACTGCAACTGTGGTTG	768	677	3	1	0	0
GABRB3	TGACTGCAACTGTGTGAC	80	244	0	1	0	5
GABRB3	ACACTGCAACTGTGTGTG	210	240	0	0	0	0
GABRB3	CAACTGCATGGTGTGTGT	984	948	2	3	0	8
GABRB3	ACACTGCATGGTGTGTCA	139	174	0	0	0	0
GABRB3	GTACTGCATGGTGTGTAC	44	37	2	1044	0	0
GABRB3	ACTGCATGGTACACTGGT	633	178	0	0	0	0
GABRD	CATGCAGTTGACTGACTG	667	533	1	0	2	0
GABRD	CAACTGCATGGTCACAGT	521	652	0	0	1	0
GABRD	ACACTGCATGGTCACACA	37	58	0	0	0	1
GABRD	GTACTGCATGGTCACAAC	62	43	0	0	0	0
GABRD	TGACTGCATGGTCACATG	183	181	1	0	0	0
GABRE	CAACTGCATGGTCATGTG	269	347	0	0	1	0
GABRE	GTACTGCATGGTCATGCA	384	531	0	1	0	1
GABRE	ACACTGCATGGTCATGAC	54	101	0	0	0	0
GABRE	TGACTGCATGGTACGTGT	661	682	0	0	0	0
GABRE	GTACTGCATGGTACGTCA	78	73	0	0	0	0
GABRG1	ACACTGACGTCAACTGTG	493	456	1	1	0	0
GABRG1	GTACTGACGTCATGGTGT	1663	1323	903	2	0	8
GABRG1	CAACTGACGTCATGGTCA	303	427	0	0	0	0
GABRG1	TGACTGACGTCATGGTAC	33	46	0	0	0	2
GABRG1	ACACTGACGTCATGGTTG	88	167	0	1	0	32
GABRG2	ACACACCACAACCAGTCA	392	589	0	0	0	0
GABRG2	GTACACCACAACCAGTAC	102	74	0	0	0	0
GABRG2	TGACACCACAACCAGTTG	548	568	2	1	0	0
GABRG2	TGTGCATGTGCAGTGTTG	684	466	0	0	0	0
GABRG2	ACTGCATGTGCAGTCAGT	178	249	0	0	0	0
GABRG3	GTACTGACGTACCACAAC	49	44	0	0	0	0
GABRG3	TGACTGACGTACCACATG	110	132	1	1	0	0
GABRG3	ACACTGACGTACCAACGT	157	202	0	1	0	0
GABRG3	TGACTGACGTACCAACCA	265	245	1	0	0	0
GABRG3	CAACTGACGTACCAACAC	35	39	0	0	0	0

GAD1	CAACTGTGACCAGTGTTG	342	944	2	0	0	0
GAD1	GTTGACGTTGACACTGGT	95	52	1	0	0	0
GAD1	CATGACGTTGACACTGCA	127	104	0	0	0	0
GAD1	TGTGACGTTGACACTGAC	58	74	0	0	0	0
GAD1	ACTGACGTTGACACTGTG	174	138	0	0	0	0
GAD2	CAACTGTGACCAGTCACA	222	179	0	0	1	1
GAD2	GTACTGTGACCAGTCAGT	407	162	0	0	0	2
GAD2	TGACTGTGACCAGTCAAC	37	53	0	0	0	0
GAD2	ACACTGTGACCAGTCATG	278	612	0	0	0	1
GAD2	CAACTGTGACCAGTACGT	439	731	0	2	0	2
GAD2	ACTGCACAGTACACTGTG	306	415	2	0	0	0
GADD45A	GTACACTGGTTGACCACA	95	111	0	0	1	0
GADD45A	ACACTGCATGTGTGACGT	114	229	0	0	0	6
GADD45A	TGACTGCATGTGTGACCA	252	114	0	0	0	11
GADD45A	CAACTGCATGTGTGACAC	67	38	0	0	0	0
GADD45A	TGTGGTTGTGGTCACAGT	334	394	0	1	0	0
GADD45A	CATGCACATGGTTGCAAC	59	39	1	0	0	0
GADD45B	TGTGGTGTCAACACTGTG	194	198	0	0	0	0
GADD45B	CATGGTGTCAACTGGTGT	637	548	3	1	0	0
GADD45B	ACTGGTGTCAACTGGTCA	267	399	0	1	0	0
GADD45B	GTTGGTGTCAACTGGTAC	64	54	0	0	0	0
GADD45B	GTTGACCAACACCACAAC	12	8	0	0	0	0
GADD45G	ACTGGTGTCAACACCAAC	4	5	0	0	0	0
GADD45G	CATGGTGTCAACACCATG	372	66	3	2	0	2
GADD45G	GTTGGTGTCAACACACGT	40	26	0	0	0	0
GADD45G	CATGGTGTCAACACACCA	55	50	0	0	0	0
GADD45G	GTTGCACATGGTTGCATG	41	38	0	0	0	0
GADD45G	TGTGCACATGGTTGACGT	372	282	0	0	0	0
GAL	CAACTGTGTGCAACTGTG	656	917	3	2	1	1
GAL	TGACTGTGTGCAACTGGT	1215	358	0	1	0	4
GAL	GTACTGTGTGCAACTGCA	278	357	1	7	0	0
GAL	ACACTGTGTGCAACTGAC	16	40	0	0	0	0
GAL	TGACTGTGTGCATGGTGT	121	158	1	0	0	2
GAL3ST1	TGACCAACGTGTCAACGT	224	331	33	0	1	0
GAL3ST1	TGACCAACGTGTCACACA	282	266	0	0	0	0
GAL3ST1	CAACCAACGTGTCACAAC	31	28	0	0	0	0
GAL3ST1	GTACCAACGTGTCACATG	397	231	0	2	0	0
GAL3ST1	GTTGCAGTACTGCACATG	375	86	0	1	0	0
GAL3ST1	GTTGCACACATGGTGTAC	101	36	1	0	0	0
GALC	GTACACCAACTGTGACGT	93	87	0	0	0	0
GALC	CAACACCAACTGTGACCA	30	36	0	0	0	0
GALC	TGACACCAACTGTGACAC	125	87	0	0	0	0
GALC	ACACACCAACTGTGACTG	230	265	0	0	0	0
GALC	TGTGACCAACCAGTTGCA	36	45	0	0	0	0
GALE	CAACACCAACTGACCACA	172	408	2	0	1	14
GALE	TGACACCAACTGACCAAC	58	62	1	0	0	0
GALE	ACACACCAACTGACCATG	211	153	0	0	0	0
GALE	CAACACCAACTGACACGT	70	109	0	0	0	0
GALE	ACACACCAACTGACACCA	435	224	1	0	0	0
GALK1	GTTGGTTGGTGTACCAGT	15	58	0	0	0	0
GALK1	CATGGTTGGTGTACCACA	36	40	0	0	0	0
GALK1	TGTGGTTGGTGTACCAAC	29	33	0	0	0	0
GALK1	ACTGGTTGGTGTACCATG	104	162	0	1	0	0
GALK1	TGTGCACAGTCAGTACGT	98	163	0	0	0	0
GALK1	GTTGCACAGTACTGGTGT	1227	1587	24	27	0	4
GALK2	GTACGTTGTGACCAACGT	635	201	2	0	0	0
GALK2	ACTGGTACTGACTGACGT	764	301	4	1	0	0
GALK2	TGTGGTACTGACTGACCA	263	401	0	1	0	0
GALK2	CATGGTACTGACTGACAC	102	71	1	0	0	0
GALK2	GTTGGTACTGACTGACTG	326	141	0	0	0	0
GALNAC4S-6ST	CATGGTACGTTGACGTCA	58	84	0	0	1	0
GALNAC4S-6ST	ACTGGTACGTTGCATGTG	290	257	0	1	0	0
GALNAC4S-6ST	GTTGGTACGTTGACGTGT	199	203	0	0	0	0
GALNAC4S-6ST	TGTGGTACGTTGACGTAC	25	39	0	0	0	0
GALNAC4S-6ST	ACTGGTACGTTGACGTTG	327	292	1	1	0	0
GALNS	ACACACACACGTTGGTCA	264	280	0	1	0	5
GALNS	GTACACACACGTTGGTAC	111	34	0	0	0	1
GALNS	TGACACACACGTTGGTTG	329	823	3	2	0	6
GALNS	ACACACACACGTTGCAGT	193	103	0	0	0	2
GALNS	CATGCAGTGTCAACACCA	31	16	0	0	0	0
GALNT1	ACACCAACGTGTGTACTG	582	562	0	0	1	1
GALNT1	CAACCAACGTGTGTTGGT	116	208	0	0	0	0
GALNT1	ACACCAACGTGTGTTGCA	223	149	0	0	0	0
GALNT1	GTACCAACGTGTGTTGAC	415	17	0	0	0	1
GALNT1	TGACCAACGTGTGTTGTG	530	535	0	1	0	0

GALNT1	GTTGCACATGTGGTGTTG	189	172	3	1	0	0
GALNT10	GTACCACATGTGTGGTTG	572	141	0	1	1	0
GALNT10	CAACCACATGTGTGGTAC	40	33	0	0	0	0
GALNT10	TGACCACATGTGTGCAGT	91	17	0	0	0	2
GALNT10	GTACCACATGTGTGCACA	181	124	0	0	0	0
GALNT10	ACACCACATGTGTGCAAC	43	34	0	0	0	0
GALNT11	TGACCACACAACACGTTG	364	418	1	0	1	0
GALNT11	ACACCACACAACACCAGT	687	520	2	0	1	1
GALNT11	CAACCACACAACACGTGT	363	350	0	1	0	0
GALNT11	ACACCACACAACACGTCA	197	368	1	0	0	1
GALNT11	GTACCACACAACACGTAC	12	19	0	0	0	0
GALNT12	ACACCACATGGTGTTGGT	161	130	2	0	0	0
GALNT12	TGACCACATGGTGTTGCA	529	347	0	2	0	0
GALNT12	CAACCACATGGTGTTGAC	41	89	0	0	0	0
GALNT12	GTACCACATGGTGTTGTG	762	218	1	0	0	0
GALNT12	ACACCACATGGTCAGTGT	299	212	0	0	0	0
GALNT13	ACACCACATGCAACACGT	126	311	0	1	0	1
GALNT13	TGACCACATGCAACACCA	318	222	0	1	0	0
GALNT13	CAACCACATGCAACACAC	42	74	0	0	0	0
GALNT13	GTACCACATGCAACACTG	367	1007	2	2	0	11
GALNT13	ACTGCAGTACTGCATGTG	284	412	0	0	0	0
GALNT14	GTACCACAACTGACCAAC	42	53	0	0	0	0
GALNT14	TGACCACAACTGACCATG	94	64	0	0	0	0
GALNT14	ACACCACAACTGACACGT	37	32	0	0	0	0
GALNT14	TGACCACAACTGACACCA	740	654	29	1	0	0
GALNT14	CATGCACAGTGTCAGTGT	330	315	0	1	0	0
GALNT17	TGTGGTGTCAGTCATGTG	354	353	0	0	1	4
GALNT17	GTTGGTGTCAGTCATGAC	40	25	0	0	0	0
GALNT17	CATGCAGTGTACGTACCA	30	58	0	0	0	0
GALNT17	TGTGCAGTGTACGTACAC	2	9	0	0	0	0
GALNT17	ACTGCAGTGTACGTACTG	329	766	0	2	0	0
GALNT2	GTACCACATGACGTGTGT	347	308	0	0	0	0
GALNT2	CAACCACATGACGTGTCA	344	225	1	0	0	0
GALNT2	TGACCACATGACGTGTAC	65	108	0	0	0	0
GALNT2	ACACCACATGACGTGTTG	1047	479	2	0	0	0
GALNT2	CAACCACATGACGTCAGT	269	353	0	0	0	1
GALNT2	TGTGCACATGGTTGTGAC	68	135	0	0	0	0
GALNT3	ACACCACATGACCATGGT	87	86	0	0	0	1
GALNT3	TGACCACATGACCATGCA	167	156	0	0	0	0
GALNT3	CAACCACATGACCATGAC	21	44	0	0	0	0
GALNT3	GTACCACATGACCATGTG	69	34	0	0	0	0
GALNT3	ACACCACATGACACGTGT	283	300	0	1	0	1
GALNT3	ACTGCACATGGTTGTGTG	171	238	0	1	0	1
GALNT4	ACACCACATGCAACGTTG	435	657	4	0	0	0
GALNT4	CAACCACATGCAACCAGT	416	351	0	1	0	0
GALNT4	ACACCACATGCAACCACA	48	91	0	0	0	0
GALNT4	GTACCACATGCAACCAAC	50	42	0	0	0	0
GALNT4	TGACCACATGCAACCATG	209	412	0	0	0	0
GALNT5	CAACCAACCATGGTCAAC	8	20	0	0	0	0
GALNT5	GTACCAACCATGGTCATG	431	339	0	2	0	0
GALNT5	TGACCAACCATGGTACGT	300	92	0	1	0	0
GALNT5	GTACCAACCATGGTACCA	116	119	0	0	0	0
GALNT5	ACACCAACCATGGTACAC	26	10	0	0	0	0
GALNT6	GTACCACATGACTGTGGT	282	527	1	1	1	1
GALNT6	TGACCACATGACTGACGT	364	760	0	0	0	1
GALNT6	GTACCACATGACTGACCA	559	373	0	0	0	0
GALNT6	ACACCACATGACTGACAC	48	51	0	0	0	0
GALNT6	CAACCACATGACTGACTG	127	105	0	0	0	0
GALNT7	CAACCACATGTGACGTTG	834	415	0	0	0	1
GALNT7	GTACCACATGTGACCAGT	143	256	0	0	0	0
GALNT7	CAACCACATGTGACCACA	135	127	0	0	0	0
GALNT7	TGACCACATGTGACCAAC	18	49	0	0	0	0
GALNT7	ACACCACATGTGACCATG	586	426	0	0	0	0
GALNT7	TGTGCACATGGTGTACCA	143	255	0	1	0	0
GALNT8	GTACCACATGTGCAGTGT	1411	901	1	2	0	0
GALNT8	CAACCACATGTGCAGTCA	244	313	0	0	0	0
GALNT8	TGACCACATGTGCAGTAC	36	99	0	0	0	1
GALNT8	ACACCACATGTGCAGTTG	2356	1898	2	2	0	3
GALNT8	CAACCACATGTGCACAGT	184	146	0	0	0	0
GALNT8	CATGCACATGGTGTACAC	41	55	0	0	0	0
GALNT9	CAACCAACGTGTCAACTG	111	111	0	0	1	0
GALNT9	GTACCAACGTGTCAACCA	272	278	0	0	0	0
GALNT9	ACACCAACGTGTCAACAC	233	22	0	0	0	0
GALNT9	GTACCAACGTGTCATGGT	403	743	0	0	0	0
GALNT9	GTTGCATGACTGTGTGAC	59	42	0	0	0	0

GALNTL1	GTACCACAACCAGTGTCA	215	359	3	0	0	0
GALNTL1	ACACCACAACCAGTGTAC	89	204	0	0	0	0
GALNTL1	CAACCACAACCAGTGTTG	1390	1055	2	20	0	0
GALNTL1	GTACCACAACCAGTCAGT	236	222	0	1	0	7
GALNTL1	CAACCACAACCAGTCACA	89	133	0	0	0	0
GALNTL2	ACACCACATGACGTTGAC	56	50	1	0	0	0
GALNTL2	CAACCACATGACGTTGTG	1004	443	0	0	0	0
GALNTL2	TGACCACATGACCAGTGT	219	261	0	0	0	0
GALNTL2	GTACCACATGACCAGTCA	68	85	0	0	0	1
GALNTL2	ACACCACATGACCAGTAC	192	113	0	0	0	0
GALNTL4	CAACCAACCAGTGTTGAC	33	50	0	0	0	0
GALNTL4	GTACCAACCAGTGTTGTG	277	601	0	0	0	0
GALNTL4	ACACCAACCAGTCAGTGT	644	984	0	1	0	0
GALNTL4	TGACCAACCAGTCAGTCA	235	258	1	0	0	2
GALNTL4	CAACCAACCAGTCAGTAC	23	23	0	0	0	0
GALT	CAACCACATGTGCAACAC	48	31	0	0	0	0
GALT	GTACCACATGTGCAACTG	649	766	0	1	0	0
GALT	TGACCACATGTGCATGGT	145	179	1	0	0	1
GALT	GTACCACATGTGCATGCA	67	80	0	0	0	0
GALT	ACACCACATGTGCATGAC	244	145	0	0	0	1
GALT	ACTGCAACACACACACAC	51	43	0	1	0	0
GAMT	ACACTGTGTGGTCAACTG	290	514	0	0	0	0
GAMT	CAACTGTGTGGTCATGGT	651	1015	2	0	0	2
GAMT	ACACTGTGTGGTCATGCA	280	287	0	0	0	2
GAMT	GTACTGTGTGGTCATGAC	38	105	0	0	0	0
GAMT	CATGCAACCAACACTGAC	64	58	0	0	0	0
GAMT	GTTGCAACCAACACTGTG	237	290	0	0	0	2
GANAB	TGACACCATGGTCAACGT	156	149	0	0	0	0
GANAB	GTACACCATGGTCAACCA	133	143	0	1	0	0
GANAB	ACACACCATGGTCAACAC	59	54	0	0	0	0
GANAB	CAACACCATGGTCAACTG	679	1261	1	1	0	4
GANAB	CATGCAGTCACACAGTCA	652	687	2	2	0	98
GANC	CAACACCATGGTCAGTGT	1497	1796	2	2	1	0
GANC	ACACACCATGGTGTTGCA	41	50	0	0	0	0
GANC	GTACACCATGGTGTTGAC	17	11	0	0	0	0
GANC	TGACACCATGGTGTTGTG	214	506	1	0	0	0
GANC	ACACACCATGGTCAGTCA	431	503	0	0	0	0
GAP43	GTACACCAGTACTGCACA	28	49	0	0	0	0
GAP43	ACACACCAGTACTGCAAC	21	47	0	0	0	0
GAP43	CAACACCAGTACTGCATG	253	244	0	0	0	0
GAP43	GTACACCAGTACTGACGT	212	429	0	0	0	0
GAP43	CAACACCAGTACTGACCA	1050	859	2	0	0	14
GAP43	GTTGCATGGTCATGCAAC	3	17	0	0	0	0
GAPDH	CATGTGACACCAACGTGT	696	463	7	4	14	2
GAPDH	ACACCAGTGTACGTGTCA	267	228	1	0	0	0
GAPDH	GTACCAGTGTACGTGTAC	72	51	0	0	0	0
GAPDH	TGACCAGTGTACGTGTTG	858	481	1	1	0	0
GAPDH	ACACCAGTGTACGTCAGT	201	374	0	0	0	0
GAPDH	TGACCAGTGTACGTCACA	37	37	0	0	0	0
GAPDH	ACTGTGACACCAGTCATG	242	190	0	0	0	0
GAPDH	CATGTGACACCAGTACGT	721	275	0	0	0	0
GAPDH	ACTGTGACACCAGTACCA	245	309	1	0	0	0
GAPDH	ACTGTGACACCAGTTGGT	356	406	0	0	0	0
GAPDH	TGTGTGACACCAGTTGCA	127	97	0	0	0	0
GAPDH	CATGTGACACCAGTTGAC	126	114	1	1	0	2
GAPDH	TGTGTGACACCACAGTCA	388	292	1	1	0	0
GAPDH	CATGTGACACCACAGTAC	121	101	0	0	0	0
GAPDH	GTTGTGACACCACAGTTG	578	492	0	0	0	0
GAPDH	ACTGTGACACCACACAAC	23	47	0	0	0	0
GAPDH	CATGTGACACCACACATG	137	69	0	0	0	0
GAPDH	GTTGTGACACCACAACGT	293	343	0	0	0	1
GAPDH	ACTGTGACACCACAACTG	495	348	0	2	0	1
GAPDH	CATGTGACACCACATGGT	65	22	0	0	0	0
GAPDH	ACTGTGACACCACATGCA	54	59	0	0	0	0
GAPDH	ACTGTGACACCAACGTCA	234	283	0	0	0	0
GAPDH	GTTGTGACACCAACGTAC	54	52	0	1	0	0
GAPDH	TGTGTGACACCAACCACA	109	101	0	0	0	0
GAPDH	CATGTGACACCAACCAAC	68	85	0	0	0	0
GAPDH	GTTGTGACACCAACCATG	83	125	0	0	0	0
GAPDHS	GTACCAGTCACACATGTG	805	1173	9	6187	443	0
GAPDHS	TGACCAGTCACACATGCA	115	181	0	0	0	0
GAPDHS	CAACCAGTCACACATGAC	65	173	0	0	0	0
GAPDHS	ACACCAGTCACAACGTGT	573	852	2	2	0	1
GAPDHS	CATGCAGTACTGGTACCA	327	509	0	1	0	0
GARS	CAACACGTACCACAGTCA	423	285	1	0	1	1

GARS	TGACACGTACCACAGTAC	8	26	0	0	0	0
GARS	ACACACGTACCACAGTTG	433	658	1	0	0	0
GARS	CAACACGTACCACACAGT	226	169	0	0	0	0
GARS	ACACACGTACCACACACA	266	311	2	0	0	0
GART	CAACCACAACGTTGGTGT	411	324	8	0	1	1289
GART	TGACCACAACGTACTGTG	76	45	0	0	0	0
GART	ACACCACAACGTTGGTCA	246	245	2	0	0	9
GART	GTACCACAACGTTGGTAC	19	39	0	0	0	0
GART	ACTGCAGTCAGTTGGTAC	82	66	0	0	0	0
GART	GTTGCATGGTCAGTACAC	41	28	0	0	0	0
GAS1	CATGGTCAGTGTACCAAC	13	45	0	0	0	0
GAS1	GTTGGTCAGTGTACCATG	29	90	0	0	0	0
GAS1	TGTGGTCAGTGTACACGT	32	22	0	0	0	0
GAS1	GTTGGTCAGTGTACACCA	152	201	0	0	0	4
GAS1	ACTGGTCAGTGTACACAC	36	32	0	0	0	0
GAS1	CATGCACAGTACTGGTCA	445	276	0	0	0	0
GAS2	ACTGGTGTGTTGACTGCA	535	99	1	0	0	0
GAS2	CATGGTGTTGTGACCAGT	589	437	1	0	0	0
GAS2	ACTGGTGTTGTGACCACA	48	181	1	1	0	0
GAS2	GTTGGTGTTGTGACCAAC	6	5	0	0	0	0
GAS2	TGTGGTGTTGTGACCATG	172	86	0	0	0	0
GAS2	ACTGCATGGTACCACACA	190	183	0	0	0	0
GAS6	ACACACTGCACATGCATG	232	263	1	0	0	3
GAS6	CAACACTGCACATGACGT	113	61	0	0	0	0
GAS6	ACACACTGCACATGACCA	622	768	0	1	0	8
GAS6	GTACACTGCACATGACAC	45	28	0	0	0	0
GAS6	TGACACTGCACATGACTG	526	486	2	2	0	0
GAS6	TGTGCACACAACGTCATG	57	59	0	0	0	0
GAST	CATGGTTGTGTGTGTGGT	341	456	2	0	0	0
GAST	ACTGGTTGTGTGTGTGCA	190	190	0	0	0	0
GAST	GTTGGTTGTGTGTGTGAC	41	54	0	0	0	0
GAST	TGTGGTTGTGTGTGTGTG	603	672	2	1	0	1
GAST	CATGCAGTGTGTGTGTGT	230	182	1	0	0	0
GATA1	CAACGTACTGACCAGTGT	905	627	1	0	1	0
GATA1	ACACGTACTGACCAGTCA	723	574	0	0	0	2
GATA1	GTACGTACTGACCAGTAC	19	15	0	0	0	0
GATA1	TGACGTACTGACCAGTTG	360	365	2	0	0	0
GATA1	TGTGCAGTACACTGCAGT	98	61	0	0	0	1
GATA1	ACTGCATGCACATGTGTG	86	102	0	0	0	1
GATA2	ACACGTACTGACACGTTG	968	800	1	0	0	3
GATA2	CAACGTACTGACACCAGT	210	442	0	0	0	0
GATA2	ACACGTACTGACACCACA	76	161	0	0	0	1
GATA2	GTACGTACTGACACCAAC	33	36	0	0	0	0
GATA2	GTTGGTGTGTTGCAACAC	274	292	0	0	0	0
GATA2	TGTGCATGCACAGTCATG	306	267	0	0	0	0
GATA3	GTTGCAGTGTTGTGGTGT	2580	1924	3	3	2	5
GATA3	GTACGTACTGACACACTG	299	449	0	0	0	1
GATA3	TGACGTACTGACACTGGT	511	349	3	0	0	0
GATA3	GTACGTACTGACACTGCA	127	129	0	0	0	0
GATA3	TGTGGTGTGTTGCAACTG	330	346	0	0	0	0
GATA4	ACACGTTGCACAGTGTTG	455	849	1	1	0	7
GATA4	CAACGTTGCACAGTCAGT	932	679	2	0	0	0
GATA4	ACACGTTGCACAGTCACA	63	85	1	0	0	0
GATA4	GTACGTTGCACAGTCAAC	13	14	0	0	0	0
GATA4	TGACGTTGCACAGTCATG	195	207	0	0	0	0
GATA6	ACCATGTGACCATGACTG	71	69	1	0	0	0
GATA6	CACATGTGACCATGTGGT	116	101	0	0	0	0
GATA6	ACCATGTGACCATGTGCA	336	300	0	0	0	0
GATA6	GTCATGTGACCATGTGAC	4	23	0	0	0	0
GATA6	GTTGCAGTACCAGTACAC	104	94	0	0	0	0
GATM	CAACCACAACTGGTGTAC	261	257	0	0	0	0
GATM	GTACCACAACTGGTGTTG	201	226	0	1	0	0
GATM	TGACCACAACTGGTCAGT	233	167	2	0	0	0
GATM	GTACCACAACTGGTCACA	73	66	0	1	0	0
GATM	ACACCACAACTGGTCAAC	33	28	0	0	0	0
GBA	CAACCAGTACACCAGTTG	1577	2605	4	1	1	0
GBA	GTACCAGTACACCACAGT	280	251	2	0	0	0
GBA	CAACCAGTACACCACACA	129	154	0	0	0	0
GBA	TGACCAGTACACCACAAC	37	28	0	0	0	0
GBA	CATGCAGTACTGGTTGGT	432	515	0	1	0	0
GBA3	ACACACCATGGTCATGTG	324	309	3	0	1	185
GBA3	GTACACCATGGTCATGGT	96	108	0	0	0	0
GBA3	CAACACCATGGTCATGCA	180	178	1	1	0	0
GBA3	TGACACCATGGTCATGAC	223	94	0	0	0	0
GBA3	GTACACCATGGTACGTGT	825	822	1	0	0	3

GBA3	TGTGCAACGTTGCAACCA	230	112	0	0	0	0
GBE1	ACACTGTGTGCACAGTCA	1090	1084	2	0	0	0
GBE1	GTACTGTGTGCACAGTAC	7	17	0	0	0	0
GBE1	TGACTGTGTGCACAGTTG	103	95	0	0	0	0
GBE1	ACACTGTGTGCACACAGT	51	66	1	0	0	0
GBE1	TGACTGTGTGCACACACA	166	156	0	1	0	0
GBF1	ACTGGTACTGGTCATGTG	564	732	0	1	1	0
GBF1	CATGGTGTACGTTGGTAC	63	67	0	1	0	0
GBF1	CATGGTACTGGTCATGCA	703	514	0	2	0	1
GBF1	TGTGGTACTGGTCATGAC	122	50	0	0	0	0
GBF1	GTTGGTACTGGTACGTGT	391	428	18	0	0	0
GBF1	ACTGCACACATGGTACAC	35	39	0	0	0	0
GBGT1	CAACCAACCACATGTGAC	131	237	3	8	3	9
GBGT1	ACACCAACCACATGTGGT	444	555	2	0	0	15
GBGT1	TGACCAACCACATGTGCA	125	177	0	0	0	0
GBGT1	GTACCAACCACATGTGTG	531	558	2	0	0	0
GBGT1	GTACCAACCAACGTGTGT	785	839	0	1	0	0
GBL	CAACCATGCAGTACACCA	397	516	4	1	2	11
GBL	GTACCATGCAGTACACGT	212	135	1	0	1	0
GBL	ACACCAACACACACGTTG	306	297	0	0	0	0
GBL	CAACCATGCAGTACCATG	46	56	0	0	0	0
GBL	TGACCATGCAGTACACAC	99	127	0	0	0	0
GBP1	TGTGGTGTACACACACCA	323	355	0	0	0	0
GBP1	CATGGTGTACACACACAC	47	52	0	0	0	0
GBP1	GTTGGTGTACACACACTG	82	53	0	0	0	0
GBP1	TGTGGTGTACACACTGGT	357	785	1	1	0	0
GBP1	GTTGGTGTACACACTGCA	535	369	1	1	0	0
GBP1	TGTGCACAGTACTGGTAC	30	29	0	0	0	0
GBP2	CATGGTGTACACTGGTTG	660	1074	2	0	0	3
GBP2	GTTGGTGTACACTGCAGT	332	702	1	1	0	0
GBP2	CATGGTGTACACTGCACA	109	96	0	1	0	1
GBP2	TGTGGTGTACACTGCAAC	18	17	0	0	0	0
GBP2	ACTGGTGTACACTGCATG	88	123	0	0	0	0
GBX2	ACACGTTGCACAGTACGT	358	299	0	0	0	0
GBX2	TGACGTTGCACAGTACCA	591	616	3	1	0	6
GBX2	CAACGTTGCACAGTACAC	32	27	0	0	0	0
GBX2	GTACGTTGCACAGTACTG	694	547	0	1	0	0
GBX2	CATGCAGTACACTGGTAC	71	80	0	0	0	0
GC	TGTGGTCAGTACCAACTG	1697	1901	307	9	1	141
GC	ACTGGTCAGTACCAACCA	220	134	0	0	0	1
GC	GTTGGTCAGTACCAACAC	21	16	0	0	0	0
GC	ACTGGTCAGTACCATGGT	471	511	0	0	0	0
GC	TGTGGTCAGTACCATGCA	70	338	0	1	0	0
GCA	GTACACTGGTTGCAGTCA	678	397	2	0	1	4
GCA	CAACACTGGTTGCAGTTG	636	788	1	1	1	3
GCA	CAACACTGGTTGGTTGTG	137	152	0	0	0	0
GCA	TGACACTGGTTGCAGTGT	73	57	0	0	0	0
GCA	ACACACTGGTTGCAGTAC	72	75	0	0	0	1
GCA	TGTGCAACACCAACGTGT	271	222	0	0	0	0
GCAT	GTACCACACACATGACGT	224	665	0	0	0	2
GCAT	CAACCACACACATGACCA	226	269	0	0	0	8
GCAT	TGACCACACACATGACAC	65	69	0	0	0	0
GCAT	ACACCACACACATGACTG	1077	630	2	0	0	0
GCAT	CAACCACACACATGTGGT	144	199	0	0	0	0
GCAT	GTTGCAACACACACGTAC	8	9	0	0	0	0
GCDH	GTACCAGTGTCAACACCA	508	566	0	1	1	0
GCDH	ACACCAGTGTCAACACAC	69	53	0	0	0	0
GCDH	CAACCAGTGTCAACACTG	1163	938	2	0	0	0
GCDH	GTACCAGTGTCAACTGGT	58	158	1	0	0	0
GCDH	CAACCAGTGTCAACTGCA	1175	470	1	0	0	2
GCG	ACTGCAACCAACACCATG	538	482	0	0	1	0
GCG	CATGGTGTGTTGGTCAGT	270	342	1	0	0	0
GCG	ACTGGTGTGTTGGTCACA	69	31	28	0	0	0
GCG	GTTGGTGTGTTGGTCAAC	33	71	0	0	0	0
GCG	TGTGGTGTGTTGGTCATG	102	101	0	1	0	0
GCG	GTTGGTGTGTTGTGCATG	202	318	1	0	0	8
GCGR	GTACGTCAGTACACCATG	57	51	0	0	0	0
GCGR	TGACGTCAGTACACACGT	19	27	0	0	0	0
GCGR	GTACGTCAGTACACACCA	140	136	0	4	0	0
GCGR	ACACGTCAGTACACACAC	287	65	0	0	0	0
GCGR	CAACGTTGTGACTGTGTG	446	694	1	0	0	0
GCH1	GTACACACGTACGTGTTG	406	406	5	0	0	0
GCH1	TGACACACGTACGTCAGT	126	84	0	1	0	0
GCH1	GTACACACGTACGTCACA	27	33	0	0	0	0
GCH1	ACACACACGTACGTCAAC	18	18	0	0	0	0

GCH1	CAACACACGTACGTCATG	238	231	0	0	0	0
GCK	GTACGTCAACCACATGTG	820	457	0	1	0	2
GCK	TGTGGTACTGCATGCAGT	207	166	8	1	0	0
GCK	GTTGGTACTGCATGCACA	249	320	1	0	0	0
GCK	ACTGGTACTGCATGCAAC	13	29	1	0	0	0
GCK	CATGGTACTGCATGCATG	164	241	0	1	0	0
GCK	TGTGCAACGTACGTACCA	157	65	0	0	0	0
GCKR	CAACACACTGCACATGCA	401	235	0	0	0	0
GCKR	TGACACACTGCACATGAC	121	290	0	0	0	0
GCKR	ACACACACTGCACATGTG	142	404	1	0	0	0
GCKR	GTACACACTGCAACGTGT	296	349	1	0	0	0
GCKR	CAACACACTGCAACGTCA	394	297	1	0	0	0
GCKR	ACTGCATGGTTGGTACTG	567	698	0	0	0	16
GCLC	CAACACCACAACACGTCA	639	239	21	0	0	39
GCLC	TGACACCACAACACGTAC	21	28	0	0	0	0
GCLC	ACACACCACAACACGTTG	1040	1162	2	1	0	0
GCLC	CAACACCACAACACCAGT	537	614	1	0	0	0
GCLC	ACACACCACAACACCACA	56	77	0	0	0	0
GCLM	GTACACCACAACACCAAC	47	127	0	0	0	0
GCLM	TGACACCACAACACCATG	256	198	0	1	0	0
GCLM	ACACACCACAACACACGT	74	54	0	0	0	0
GCLM	TGACACCACAACACACCA	829	316	0	0	0	0
GCLM	ACTGGTCACAGTGTACGT	84	171	0	0	0	0
GCM1	TGACGTCAACACACCATG	58	61	20	55	12	41
GCM1	ACACGTCAACACACCACA	199	267	0	231	8	0
GCM1	CAACGTCAACACACCAGT	362	249	0	0	0	0
GCM1	GTACGTCAACACACCAAC	8	3	0	0	0	0
GCM1	ACACGTCAACACACACGT	78	25	0	0	0	0
GCNT1	GTACCACAACTGTGCAGT	141	244	3	1	3	5
GCNT1	CAACCACAACTGTGCACA	56	25	0	0	0	0
GCNT1	TGACCACAACTGTGCAAC	44	29	0	0	0	0
GCNT1	ACACCACAACTGTGCATG	350	213	0	0	0	1
GCNT1	CAACCACAACTGTGACGT	217	277	0	0	0	0
GCNT2	GTTGCATGGTACACACAC	97	55	0	1	1	0
GCNT2	GTCATGTGCATGACCAAC	21	64	0	0	0	0
GCNT2	TGCATGTGCATGACCATG	28	24	0	0	0	0
GCNT2	ACCATGTGCATGACACGT	28	28	0	0	0	0
GCNT2	TGTGGTTGGTACCACACA	44	70	0	1	0	0
GCNT2	TGTGCATGGTACACACTG	214	284	0	0	0	0
GCS1	TGACACCATGGTCAGTTG	1168	536	0	0	1	0
GCS1	GTACACCATGGTCAGTAC	73	57	0	0	0	0
GCS1	ACACACCATGGTCACAGT	160	101	1	0	0	0
GCS1	ACTGACACTGGTGTACGT	273	192	0	0	0	0
GCS1	TGTGACACTGGTGTACCA	247	274	0	1	0	1
GDF1	CAACTGGTACCAGTGTCA	962	734	6	10	1	17
GDF1	TGACTGGTACCAGTGTAC	96	125	0	0	0	0
GDF1	ACACTGGTACCAGTGTTG	278	232	0	0	0	0
GDF1	TGTGGTACCACATGACGT	348	166	0	0	0	0
GDF1	GTTGGTACCACATGACCA	118	271	0	0	0	0
GDF10	ACACTGGTACCACACATG	200	339	54	0	0	0
GDF10	CAACTGGTACCACAACGT	661	1199	1	2	0	0
GDF10	ACACTGGTACCACAACCA	318	372	1	0	0	0
GDF10	GTACTGGTACCACAACAC	52	61	0	0	0	0
GDF10	ACTGCAGTTGACCAGTAC	48	41	0	0	0	0
GDF10	CATGCACATGACCAACAC	26	21	0	0	0	0
GDF11	CAACTGGTTGTGTGCACA	487	363	0	0	1	0
GDF11	GTACTGGTTGTGTGCAGT	132	131	0	0	0	0
GDF11	TGACTGGTTGTGTGCAAC	26	32	0	0	0	0
GDF11	ACACTGGTTGTGTGCATG	207	191	0	0	0	0
GDF11	TGTGCAGTTGACTGCACA	17	29	1	0	0	0
GDF11	GTTGCACATGACCAACTG	183	229	0	0	0	0
GDF15	GTACTGGTACCAACACGT	47	27	0	0	0	0
GDF15	CAACTGGTACCAACACCA	378	380	0	0	0	0
GDF15	TGACTGGTACCAACACAC	105	72	0	0	0	0
GDF15	ACACTGGTACCAACACTG	408	314	0	1	0	0
GDF15	CAACTGGTACCAACTGGT	203	235	0	0	0	0
GDF15	ACTGCACACATGCAACGT	137	107	0	0	0	0
GDF2	ACTGCACAACTGACGTCA	346	377	0	10	1	0
GDF2	ACACTGGTACCACAGTAC	36	45	0	0	0	0
GDF2	CAACTGGTACCACAGTTG	42	54	0	0	0	0
GDF2	GTACTGGTACCACACAGT	422	191	1	0	0	1
GDF2	CAACTGGTACCACACACA	175	197	0	0	0	0
GDF2	TGACTGGTACCACACAAC	26	47	0	0	0	0
GDF3	CAACTGGTTGCAACCAAC	44	34	1	0	0	0
GDF3	GTACTGGTTGCAACCATG	294	279	0	0	0	0

GDF3	TGACTGGTTGCAACACGT	131	172	0	0	0	0
GDF3	GTACTGGTTGCAACACCA	127	133	0	0	0	0
GDF3	ACACTGGTTGCAACACAC	31	34	0	0	0	0
GDF3	ACTGCACATGCACAGTGT	197	226	0	0	0	0
GDF5	ACACTGGTACCAGTACGT	549	190	0	1	1	0
GDF5	CAACTGGTACCAGTCAGT	219	147	0	1	0	1
GDF5	ACACTGGTACCAGTCACA	63	88	0	1	0	1
GDF5	GTACTGGTACCAGTCAAC	66	76	0	0	0	0
GDF5	TGACTGGTACCAGTCATG	82	187	0	0	0	0
GDF5	TGTGCACAACGTCATGGT	67	201	0	0	0	0
GDF6	CATGGTCATGACACCAGT	709	402	1	1	1	0
GDF6	TGTGGTGTGTTGTGCACA	112	147	0	0	0	3
GDF6	TGTGGTCATGACACGTAC	106	188	1	0	0	1
GDF6	ACTGGTCATGACACGTTG	1285	556	8	2	0	0
GDF6	ACTGGTCATGACACCACA	39	41	0	0	0	0
GDF9	CAACTGGTACGTTGTGGT	667	567	0	0	0	0
GDF9	ACACTGGTACGTTGTGCA	50	80	0	0	0	0
GDF9	GTACTGGTACGTTGTGAC	85	164	0	0	0	0
GDF9	TGACTGGTACGTTGTGTG	66	89	0	0	0	0
GDF9	GTACTGGTACCAGTGTGT	261	215	1	0	0	0
GDI1	GTACACCAGTTGGTACGT	240	155	0	0	0	0
GDI1	CAACACCAGTTGGTACCA	234	166	0	0	0	0
GDI1	TGACACCAGTTGGTACAC	48	85	0	0	0	0
GDI1	ACACACCAGTTGGTACTG	70	172	71	0	0	0
GDI1	CAACACCAGTTGGTTGGT	292	501	0	6	0	0
GDI1	ACTGCACAGTACTGGTTG	427	774	11	0	0	1
GDI2	CAACACCAGTTGCAGTGT	1209	1115	1	0	1	0
GDI2	ACACACCAGTTGGTTGCA	137	172	0	0	0	0
GDI2	GTACACCAGTTGGTTGAC	19	41	0	0	0	0
GDI2	TGACACCAGTTGGTTGTG	187	217	0	0	0	0
GDI2	ACACACCAGTTGCAGTCA	728	746	1	1	0	3
GDI2	ACTGCACAACACGTTGCA	127	103	0	0	0	0
GDNF	TGACTGGTTGACACGTGT	444	433	0	0	0	2
GDNF	GTACTGGTTGACACGTCA	137	153	0	0	0	0
GDNF	ACACTGGTTGACACGTAC	5	12	0	0	0	0
GDNF	CAACTGGTTGACACGTTG	63	66	0	0	0	0
GDNF	GTACTGGTTGACACCAGT	66	66	0	0	0	0
GEN1	CAACACACACCAACACTG	311	254	1	0	1	0
GEN1	GTACACACACCAACACCA	186	192	0	0	0	0
GEN1	ACACACACACCAACACAC	44	51	0	0	0	0
GEN1	GTACACACACCAACTGGT	413	229	0	0	0	0
GEN1	CAACACACACCAACTGCA	233	334	0	0	0	0
GFAP	GTTGGTGTGTGTTGACAC	3	4	0	0	0	0
GFAP	TGTGGTGTGTGTTGACTG	491	830	4	1354	0	1
GFAP	ACTGGTGTGTGTTGTGGT	590	742	1	2	0	0
GFAP	TGTGGTGTGTGTTGTGCA	94	202	0	0	0	0
GFAP	CATGGTGTGTGTTGTGAC	227	101	0	0	0	0
GFAP	ACTGCAACTGCAGTTGAC	0	12	0	0	0	0
GFI1	GTACGTTGCACACAGTCA	773	762	2	1	1	0
GFI1	TGACGTTGCACACAGTGT	306	364	0	0	0	0
GFI1	ACACGTTGCACACAGTAC	64	75	0	0	0	0
GFI1	CAACGTTGCACACAGTTG	892	878	8	0	0	0
GFI1	GTACGTTGCACACACAGT	137	132	0	0	0	0
GFPT1	CAACTGTGCACAGTCAAC	9	10	0	0	0	0
GFPT1	GTACTGTGCACAGTCATG	180	292	1	0	0	2
GFPT1	TGACTGTGCACAGTACGT	167	175	0	0	0	0
GFPT1	GTACTGTGCACAGTACCA	31	39	0	0	0	1
GFPT1	ACACTGTGCACAGTACAC	41	41	0	0	0	0
GFPT1	CATGCACAGTACTGCAGT	528	252	2	0	0	1
GFPT2	TGACCAGTTGTGCACAAC	20	14	0	0	0	0
GFPT2	CAACTGTGCACAGTACTG	602	325	0	0	0	0
GFPT2	GTACTGTGCACAGTTGGT	325	488	0	1	0	8
GFPT2	CAACTGTGCACAGTTGCA	45	35	0	0	0	0
GFPT2	TGACTGTGCACAGTTGAC	133	122	1	0	0	0
GFPT2	TGTGCACACATGACCAAC	48	29	0	1	0	0
GFRA1	GTACTGCAACGTTGGTCA	204	331	1	0	2	0
GFRA1	TGACTGCAACGTTGGTGT	402	648	0	1	0	1
GFRA1	ACACTGCAACGTTGGTAC	21	56	0	1	0	0
GFRA1	CAACTGCAACGTTGGTTG	134	112	0	0	0	0
GFRA1	GTACTGCAACGTTGCAGT	197	157	0	0	0	0
GGA1	GTACTGACTGGTTGGTCA	72	47	0	0	0	0
GGA1	ACACTGACTGGTTGGTAC	61	112	1	0	0	0
GGA1	CAACTGACTGGTTGGTTG	394	474	1	0	0	0
GGA1	GTACTGACTGGTTGCAGT	68	115	1	0	0	0
GGA1	CATGCAGTTGTGCAACAC	31	87	0	0	0	0

GGA3	GTACTGACTGGTCATGGT	670	602	1	2	1	10
GGA3	CAACTGACTGGTCATGCA	467	506	3	0	1	7
GGA3	GTACTGACTGGTCAACCA	300	122	0	1	0	0
GGA3	ACACTGACTGGTCAACAC	53	69	0	0	0	0
GGA3	CAACTGACTGGTCAACTG	440	312	1	0	0	0
GGA3	GTTGCAACCAACTGCACA	70	62	0	1	0	0
GGCX	CAACTGTGACCAACCAAC	17	31	0	0	0	0
GGCX	GTACTGTGACCAACCATG	431	539	1	0	0	2
GGCX	TGACTGTGACCAACACGT	62	31	0	0	0	0
GGCX	GTACTGTGACCAACACCA	135	164	0	0	0	0
GGCX	ACACTGTGACCAACACAC	23	22	0	0	0	0
GGH	ACTGCACAGTACTGCACA	209	170	0	0	1	0
GGH	GTACACACCAACACACCA	100	101	0	0	0	0
GGH	ACACACACCAACACACAC	78	71	1	0	0	275
GGH	CAACACACCAACACACTG	675	778	0	1	0	0
GGH	GTACACACCAACACTGGT	756	625	2	0	0	0
GGH	CAACACACCAACACTGCA	112	107	0	0	0	0
GGPS1	GTACACGTACCACAGTGT	596	647	0	0	1	0
GGPS1	GTACACGTACCAGTTGGT	158	312	2	0	0	0
GGPS1	CAACACGTACCAGTTGCA	305	322	1	0	0	1
GGPS1	TGACACGTACCAGTTGAC	11	9	0	0	0	0
GGPS1	ACACACGTACCAGTTGTG	341	337	0	0	0	0
GGT1	TGACCACACACACATGGT	472	401	1	0	1	0
GGT1	TGACCACACACACAACCA	147	155	0	0	0	0
GGT1	CAACCACACACACAACAC	39	19	0	0	0	1
GGT1	GTACCACACACACAACTG	277	552	0	0	0	0
GGT1	ACACCAACGTCACACACA	56	50	0	0	0	0
GGTL3	TGACGTTGACCAACTGGT	173	170	2	1	4	5
GGTL3	TGACGTTGACCAACACCA	403	767	0	1	0	0
GGTL3	CAACGTTGACCAACACAC	12	7	0	0	0	0
GGTL3	GTACGTTGACCAACACTG	412	527	0	0	0	0
GGTL3	GTACGTTGACCAACTGCA	130	142	0	0	0	0
GGTL3	ACTGCATGGTACACACCA	401	427	0	0	0	2
GH1	GTACTGGTACTGGTTGTG	379	161	1	0	0	0
GH1	ACACTGGTACTGCAGTGT	330	910	1	0	0	1
GH1	TGACTGGTACTGCAGTCA	53	71	0	0	0	0
GH1	CATGGTTGCAGTGTCATG	34	43	0	0	0	0
GH1	CATGCAACCATGGTTGGT	313	187	0	2	0	0
GH1	ACTGCAACCATGGTTGCA	136	53	0	1	0	0
GHR	GTACTGGTTGACACTGTG	1032	946	1	1	1	6
GHR	CAACTGGTTGACACTGAC	38	84	93	0	0	0
GHR	ACACTGGTTGACTGGTGT	489	273	1	0	0	1
GHR	TGACTGGTTGACTGGTCA	646	438	1	0	0	0
GHR	CAACTGGTTGACTGGTAC	125	115	0	0	0	2
GHRH	ACTGCATGGTTGGTGTGT	725	714	1	0	3	2
GHRH	ACACTGTGTGCAACACGT	80	67	0	0	0	0
GHRH	TGACTGTGTGCAACACCA	215	100	0	0	0	0
GHRH	CAACTGTGTGCAACACAC	6	2	0	0	0	0
GHRH	GTACTGTGTGCAACACTG	264	291	1	0	0	0
GHRH	CATGCACAGTGTGTTGGT	269	570	0	0	0	0
GHRHR	ACACGTCAGTACACTGTG	388	364	0	0	1	1
GHRHR	CAACGTCAGTACACACTG	263	247	0	0	0	0
GHRHR	GTACGTCAGTACACTGGT	112	118	0	0	0	0
GHRHR	CAACGTCAGTACACTGCA	101	179	1	0	0	0
GHRHR	TGACGTCAGTACACTGAC	65	30	0	0	0	0
GHRL	ACACTGTGCAACCAACTG	1468	1775	3	1	0	0
GHRL	CAACTGTGCAACCATGGT	69	81	1	0	0	0
GHRL	ACACTGTGCAACCATGCA	488	369	60	0	0	0
GHRL	GTACTGTGCAACCATGAC	46	52	0	0	0	0
GHRL	TGACTGTGCAACCATGTG	102	154	0	0	0	0
GHSR	GTACGTCAGTACTGGTGT	71	79	0	0	0	1
GHSR	CAACGTCAGTACTGGTCA	326	171	0	0	0	0
GHSR	TGACGTCAGTACTGGTAC	18	18	0	0	0	0
GHSR	ACACGTCAGTACTGGTTG	560	644	0	0	0	2
GHSR	CAACGTCAGTACTGCAGT	406	438	0	0	0	0
GIP	CATGGTACACCATGGTTG	703	745	0	0	2	0
GIP	ACTGGTACACCATGCATG	363	441	1	1	1	0
GIP	GTTGGTACACCATGCAGT	45	118	0	1	0	0
GIP	CATGGTACACCATGCACA	145	274	3	1	0	0
GIP	TGTGGTACACCATGCAAC	104	124	1	1	0	0
GIPR	ACACGTCAGTACTGCACA	33	46	0	0	0	0
GIPR	GTACGTCAGTACTGCAAC	44	18	0	0	0	0
GIPR	TGACGTCAGTACTGCATG	170	160	0	0	0	0
GIPR	ACACGTCAGTACTGACGT	28	71	0	0	0	0
GIPR	TGACGTCAGTACTGACCA	132	105	0	0	0	1

GIT1	GTACGTCAGTACTGACTG	481	410	1	0	1	2
GIT1	CAACGTCAGTACTGACAC	4	4	0	0	0	0
GIT1	TGACGTCAGTACTGTGGT	695	961	2	0	0	1
GIT1	GTACGTCAGTACTGTGCA	184	246	0	0	0	0
GIT1	TGTGCAGTACCATGACCA	205	175	0	0	0	0
GJA1	ACACTGCACACACAACTG	1130	1405	0	0	1	1
GJA1	CAACTGCACACACACATG	114	143	0	1	0	0
GJA1	GTACTGCACACACAACGT	360	381	0	2	0	0
GJA1	CAACTGCACACACAACCA	275	224	0	0	0	0
GJA1	TGACTGCACACACAACAC	62	72	0	0	0	48
GJA3	CAACTGCACACACATGGT	635	443	0	1	0	0
GJA3	ACACTGCACACACATGCA	301	255	0	0	0	0
GJA3	GTACTGCACACACATGAC	36	42	0	0	0	0
GJA3	GTTGCAGTTGACTGCATG	175	279	0	1	0	0
GJA3	TGTGACACCAGTGTTGCA	155	112	0	1	0	0
GJA4	TGACTGCACACACATGTG	1146	862	0	1	0	1
GJA4	CAACTGCACACAACGTGT	298	348	1	0	0	0
GJA4	ACACTGCACACAACGTCA	192	396	0	0	0	0
GJA4	GTACTGCACACAACGTAC	104	137	0	0	0	0
GJA4	CATGACCAGTCAGTACAC	213	136	0	0	0	10
GJB1	ACACTGCACAACGTGTCA	1043	907	1	0	0	1
GJB1	GTACTGCACAACGTGTAC	46	249	0	0	0	0
GJB1	TGACTGCACAACGTGTTG	916	551	2	0	0	46
GJB1	ACACTGCACAACGTCAGT	322	408	2	0	0	0
GJB1	ACACTGACTGCATGCAAC	46	36	0	0	0	0
GJD2	GTACTGACTGCATGCACA	184	200	0	0	0	0
GJD2	TGACTGTGGTACGTTGTG	241	312	0	0	0	0
GJD2	CAACTGTGGTACCAGTGT	236	362	2	0	0	4
GJD2	ACACTGTGGTACCAGTCA	503	470	0	0	0	1
GJD2	TGTGCACATGCACAGTCA	163	66	0	0	0	0
GJD2	CATGCACATGCACAGTAC	105	173	0	0	0	0
GK2	ACACCAACACTGCACAAC	28	44	0	0	0	0
GK2	CAACCAACACTGCACATG	28	96	0	0	0	0
GK2	GTACCAACACTGCAACGT	307	113	0	0	0	1
GK2	CAACCAACACTGCAACCA	481	323	1	0	0	0
GK2	TGACCAACACTGCAACAC	12	8	0	0	0	0
GLA	ACTGGTGTGTGTGTGTGT	465	389	0	0	0	0
GLA	TGTGGTGTGTGTGTGTCA	316	471	0	13	0	0
GLA	CATGGTGTGTGTGTGTAC	38	30	0	0	0	0
GLA	GTTGGTGTGTGTGTGTTG	220	303	2	0	0	0
GLA	TGTGGTGTGTGTGTCAGT	327	256	0	0	0	0
GLB1	TGACTGTGTGACGTCATG	277	395	2	0	0	0
GLB1	ACACTGTGTGACGTACGT	162	145	1	0	0	0
GLB1	TGACTGTGTGACGTACCA	13	47	0	0	0	0
GLB1	CAACTGTGTGACGTACAC	7	10	0	0	0	0
GLB1	GTACTGTGTGACGTACTG	354	349	1	0	0	0
GLB1	ACTGCACATGCACAACTG	1085	1085	2	1	0	0
GLCE	TGACACCAACTGTGCAGT	304	198	2	1	0	4
GLCE	GTACACCAACTGTGCACA	136	66	0	0	0	11
GLCE	ACACACCAACTGTGCAAC	33	104	0	0	0	0
GLCE	CAACACCAACTGTGCATG	174	257	0	0	0	0
GLCE	CATGCAGTTGGTTGACAC	135	69	0	0	0	0
GLG1	ACTGGTCACAACCAACGT	1157	754	0	1	1	8
GLG1	CATGGTCATGTGACGTGT	562	692	0	1	1	0
GLG1	TGTGGTCACAACCACATG	60	68	0	0	0	0
GLG1	ACTGGTCATGTGACGTCA	206	214	0	0	0	2
GLG1	GTTGGTCATGTGACGTAC	30	52	0	0	0	0
GLI1	GTACGTTGCACACATGTG	430	481	1	0	0	0
GLI1	ACACGTTGCACAACGTGT	154	153	0	0	0	0
GLI1	TGACGTTGCACAACGTCA	188	63	0	0	0	0
GLI1	CAACGTTGCACAACGTAC	48	48	0	0	0	0
GLI1	GTACGTTGCACAACGTTG	143	280	0	1	0	0
GLI1	CATGCACACATGTGCATG	75	35	0	0	0	0
GLI2	TGACCACAGTGTTGGTCA	38	60	0	0	0	0
GLI2	CAACCACAGTGTTGGTAC	212	23	0	0	0	0
GLI2	GTACCACAGTGTTGGTTG	541	515	2	1	0	10
GLI2	TGACCACAGTGTTGCAGT	153	192	0	0	0	0
GLI2	GTACCACAGTGTTGCACA	28	25	0	0	0	0
GLI3	CAACGTTGCACAACACCA	289	309	0	0	0	0
GLI3	TGACGTTGCACAACACAC	111	78	0	1	0	0
GLI3	ACACGTTGCACAACACTG	653	396	0	0	0	0
GLI3	CAACGTTGCACAACTGGT	272	181	0	0	0	0
GLI3	TGTGCAGTACACTGGTCA	112	153	0	0	0	0
GLI3	CATGCACATGTGTGACGT	154	178	0	0	0	0
GLI4	ACTGGTGTCATGACACCA	30	35	0	0	0	0

GLI4	ACTGGTGTACGTGTCAGT	108	117	0	0	0	0
GLI4	TGTGGTGTACGTGTCACA	58	65	7	0	0	0
GLI4	CATGGTGTACGTGTCAAC	17	28	0	1	0	0
GLI4	GTTGGTGTACGTGTCATG	385	325	1	0	0	0
GLO1	GTTGGTCAGTCACAACCA	273	329	1	1	0	0
GLO1	ACTGGTCAGTCACAACAC	63	53	0	0	0	0
GLO1	CATGGTCAGTCACAACTG	175	310	1	0	0	0
GLO1	GTTGGTCAGTCACATGGT	988	471	0	0	0	0
GLO1	CATGGTCAGTCACATGCA	550	345	0	0	0	0
GLP1R	TGCATGTGCACAACGTTG	703	788	0	1	0	1
GLP1R	ACCATGTGCACAACCAGT	1422	1089	2	0	0	1
GLP1R	TGCATGTGCACAACCACA	104	86	0	0	0	0
GLP1R	CACATGTGCACAACCAAC	82	131	0	1	0	0
GLP1R	GTCATGTGCACAACCATG	132	135	0	1	0	0
GLP1R	CATGCAACCACACAGTAC	59	67	0	0	0	0
GLP2R	CAACGTCACAGTCAGTAC	164	83	0	0	0	0
GLP2R	GTACGTCACAGTCAGTTG	987	784	4134	3	0	2
GLP2R	TGACGTCACAGTCACAGT	94	150	0	0	0	0
GLP2R	GTACGTCACAGTCACACA	154	232	0	1	0	0
GLP2R	CATGCACACATGGTACTG	143	188	1	0	0	0
GLP2R	GTTGCACACATGGTTGGT	24	74	0	0	0	0
GLRX	GTACCAACCACACATGGT	594	727	109	0	0	94
GLRX	CAACCAACCACACATGCA	240	311	1	0	0	107
GLRX	TGACCAACCACACATGAC	27	37	0	0	0	0
GLRX	ACACCAACCACACATGTG	625	687	1	0	0	0
GLRX	GTACCAACCACAACGTGT	560	589	2	1	0	0
GLRX	GTTGCACAGTACTGCAAC	15	4	0	0	0	0
GLRX2	CAACTGACACGTTGGTCA	844	693	0	0	0	0
GLRX2	TGACTGACACGTTGGTAC	134	112	0	0	0	0
GLRX2	ACACTGACACGTTGGTTG	1120	820	2	0	0	6
GLRX2	CAACTGACACGTTGCAGT	37	10	0	1	0	0
GLRX2	ACACTGACACGTTGCACA	236	101	0	0	0	0
GLS	TGACACACCACAGTGTTG	277	327	0	1	1	0
GLS	CAACACACCACAGTGTGT	552	536	2	0	0	0
GLS	ACACACACCACAGTGTCA	514	363	0	0	0	0
GLS	GTACACACCACAGTGTAC	107	181	0	0	0	0
GLS	ACACACACCACAGTCAGT	469	168	0	0	0	0
GLS	GTTGCAACACGTCACATG	657	389	3	0	0	2
GLS2	TGACACACCAACCATGTG	169	182	0	0	0	0
GLS2	CAACACACCAACACGTGT	269	417	0	0	0	8
GLS2	ACACACACCAACACGTCA	388	391	0	0	0	0
GLS2	GTACACACCAACACGTAC	65	55	1	0	0	0
GLS2	TGACACACCAACACGTTG	470	408	0	0	0	0
GLS2	ACTGCAACCAACTGGTGT	772	577	2	0	0	3
GLUD1	ACACCAGTGTCACACAAC	15	19	0	0	0	0
GLUD1	GTACCAGTGTCACAACGT	99	169	0	0	0	0
GLUD1	CAACCAGTGTCAACGTGT	701	989	3	0	0	1
GLUD1	ACACCAGTGTCAACGTCA	199	65	0	0	0	0
GLUD1	GTACCAGTGTCAACGTAC	131	99	0	0	0	1
GLUD1	GTTGCACACATGTGACGT	166	96	0	1	0	0
GLUD2	CAACCAGTGTCACACATG	325	228	0	1	0	0
GLUD2	CAACCAGTGTCACAACCA	325	487	4	2	0	7
GLUD2	TGACCAGTGTCACAACAC	25	31	0	0	0	0
GLUD2	TGACCAGTGTCAACGTTG	700	726	0	0	0	13
GLUD2	CAACCAGTGTCATGTGTG	200	258	3	0	0	0
GLUL	TGACACGTCATGCAACGT	662	426	0	1	0	0
GLUL	GTACACGTCATGCAACCA	181	211	23	0	0	1
GLUL	ACACACGTCATGCAACAC	62	75	0	0	0	0
GLUL	CAACACGTCATGCAACTG	527	774	3	1	0	0
GLUL	CAACTGTGCACACATGTG	372	368	4	0	0	5
GMDS	CAACACACTGGTACCAAC	15	13	0	0	0	0
GMDS	GTACACACTGGTACCATG	133	106	0	0	0	0
GMDS	TGACACACTGGTACACGT	200	152	0	0	0	0
GMDS	GTACACACTGGTACACCA	585	418	0	0	0	0
GMDS	ACACACACTGGTACACAC	61	92	0	0	0	0
GMNN	CATGGTCAGTGTCATGGT	130	183	0	0	0	0
GMNN	ACTGGTCAGTGTCATGCA	535	645	0	0	0	2
GMNN	GTTGGTCAGTGTCATGAC	78	117	3	0	0	0
GMNN	TGTGGTCAGTGTCATGTG	257	501	0	0	0	0
GMNN	CATGGTCAGTGTACGTGT	342	359	1	0	0	0
GMPPA	CATGGTACGTTGACCAGT	17	38	0	0	0	0
GMPPA	ACTGGTACGTTGACCACA	33	34	0	0	0	0
GMPPA	GTTGGTACGTTGACCAAC	7	13	0	0	0	0
GMPPA	TGTGGTACGTTGACCATG	55	73	0	0	0	0
GMPPA	ACTGGTACGTTGACACGT	238	168	0	0	0	0

GMPPB	TGTGGTTGTGGTACCACA	10	23	0	0	0	0
GMPPB	CATGGTTGTGGTACCAAC	35	132	0	0	0	0
GMPPB	GTTGGTTGTGGTACCATG	247	427	0	0	0	2
GMPPB	TGTGGTTGTGGTACACGT	89	73	0	0	0	0
GMPPB	GTTGGTTGTGGTACACCA	231	212	0	0	0	0
GMPPB	TGTGCACATGACCATGGT	1558	856	0	1	0	4
GNA11	ACACCAACCATGACGTAC	219	143	0	0	0	0
GNA11	CAACCAACCATGACGTTG	453	458	0	0	0	1
GNA11	GTACCAACCATGACCAGT	157	227	2	0	0	0
GNA11	CAACCAACCATGACCACA	224	237	0	0	0	2
GNA11	TGACCAACCATGACCAAC	50	45	0	0	0	2
GNA11	TGTGCACAGTACTGCATG	112	157	0	0	0	0
GNA12	TGACCAACACCAACGTCA	857	460	2	2	0	0
GNA12	CAACCAACACCAACGTAC	89	77	0	0	0	0
GNA12	GTACCAACACCAACGTTG	848	355	2643	2	0	5
GNA12	GTTGGTGTCAGTTGCAAC	4	7	0	0	0	0
GNA12	CATGCAGTACTGACACAC	63	65	0	0	0	0
GNA13	CAACCAACACACGTACCA	174	27	0	0	0	0
GNA13	TGACCAACACACGTACAC	15	34	0	1	0	0
GNA13	ACACCAACACACGTACTG	211	280	1	0	0	0
GNA13	CAACCAACACACGTTGGT	152	151	0	0	0	0
GNA13	ACACCAACACACGTTGCA	462	335	0	0	0	0
GNA13	ACTGCATGGTTGACTGAC	27	35	0	0	0	0
GNA15	ACACCAACCATGACCATG	305	288	0	0	0	0
GNA15	CAACCAACCATGACACGT	37	31	0	0	0	0
GNA15	ACACCAACCATGACACCA	672	556	0	0	0	0
GNA15	GTACCAACCATGACACAC	56	101	0	0	0	0
GNA15	TGACCAACCATGACACTG	487	282	0	0	0	0
GNA15	ACTGCACAGTACTGACGT	181	139	5	1	0	5
GNAI1	ACACCAACCATGACTGGT	449	414	0	18	0	1
GNAI1	TGACCAACCATGACTGCA	104	39	1	0	0	1
GNAI1	CAACCAACCATGACTGAC	110	136	0	0	0	0
GNAI1	GTACCAACCATGACTGTG	374	330	0	0	0	0
GNAI1	ACACCAACCATGTGGTGT	300	299	2	0	0	6
GNAI2	GTACCAGTTGGTCAGTAC	109	70	0	0	0	0
GNAI2	TGACCAGTTGGTCAGTTG	229	236	0	0	0	0
GNAI2	ACACCAGTTGGTCACAGT	167	478	1	0	0	0
GNAI2	TGACCAGTTGGTCACACA	62	75	0	0	0	0
GNAI2	CAACCAGTTGGTCACAAC	15	36	0	0	0	0
GNAI3	GTACCAACCATGTGCACA	249	215	0	0	1	0
GNAI3	TGACCAACCATGTGGTCA	18	31	0	0	0	0
GNAI3	CAACCAACCATGTGGTAC	75	100	0	0	0	0
GNAI3	GTACCAACCATGTGGTTG	1057	1109	1	1	0	1
GNAI3	TGACCAACCATGTGCAGT	156	149	0	0	0	0
GNAI3	GTTGCACAACCACAGTCA	316	587	0	1	0	0
GNAL	ACACCAACCATGTGCAAC	30	12	0	0	0	0
GNAL	CAACCAACCATGTGCATG	413	260	0	0	0	0
GNAL	GTACCAACCATGTGACGT	296	292	0	0	0	0
GNAL	GTTGGTGTACCAACGTTG	260	184	0	0	0	0
GNAL	ACTGCAGTACTGACCACA	49	79	0	1	0	0
GNAO1	ACTGCATGTGTGACGTGT	668	490	0	2	1	0
GNAO1	CAACCAACCATGTGACCA	169	173	0	0	0	0
GNAO1	TGACCAACCATGTGACAC	31	26	1	0	0	0
GNAO1	TGTGCATGTGTGACGTCA	136	209	0	1	0	0
GNAO1	CATGCATGTGTGACGTAC	96	105	0	0	0	0
GNAQ	TGACCAACACCAACCAGT	142	164	1	0	0	0
GNAQ	GTACCAACACCAACCACA	69	42	0	0	0	0
GNAQ	ACACCAACACCAACCAAC	29	36	0	0	0	1
GNAQ	CAACCAACACCAACCATG	292	197	0	0	0	0
GNAQ	GTACCAACACCAACACGT	63	37	0	0	0	0
GNAS	TGACTGTGTGACTGCACA	176	64	0	0	0	0
GNAS	CAACTGTGTGACTGCAAC	12	36	0	0	0	0
GNAS	GTACTGTGTGACTGCATG	171	165	0	1	0	0
GNAS	TGACTGTGTGACTGACGT	300	146	0	0	0	0
GNAS	GTACTGTGTGACTGACCA	206	220	0	0	0	0
GNAT1	CAACCAACACCAACACCA	206	348	2	1	0	0
GNAT1	TGACCAACACCAACACAC	72	59	0	0	0	0
GNAT1	ACACCAACACCAACACTG	1343	1151	1	2	0	0
GNAT1	CAACCAACACCAACTGGT	401	395	1	0	0	0
GNAT1	GTTGCAACACACGTCAGT	159	117	2	1	0	1
GNAT1	CATGCAACACACGTCACA	104	117	0	0	0	0
GNAT3	ACTGCAGTGTGTCAGTTG	434	416	0	0	0	0
GNAT3	CATGCAGTGTGTCACAGT	212	247	0	0	0	2
GNAT3	ACTGCAGTGTGTCACACA	28	52	0	0	0	0
GNAT3	GTTGCAGTGTGTCACAAC	71	8	0	1	0	0

GNAT3	GTTGACCAACTGACGTGT	1008	2021	0	7	0	1
GNAZ	ACACCAACACCAACTGCA	655	335	0	0	0	68
GNAZ	GTACCAACACCAACTGAC	37	42	0	0	0	0
GNAZ	TGACCAACACCAACTGTG	319	354	0	0	0	0
GNAZ	CAACCAACACCATGGTGT	381	224	2	0	0	1
GNAZ	ACACCAACACCATGGTCA	192	208	0	0	0	0
GNB1	ACACCAACACCATGCAGT	254	267	0	0	1	0
GNB1	CAACCACACACACACAGT	141	124	0	0	0	1
GNB1	GTACCAACACCATGGTAC	62	78	0	0	0	0
GNB1	TGACCAACACCATGGTTG	265	268	0	1	0	1
GNB1	TGACCAACACCATGCACA	112	86	0	1	0	0
GNB1	ACTGCAACCAACTGACTG	91	192	0	0	0	0
GNB1L	ACACCAACACACCAGTCA	57	85	1	0	0	0
GNB1L	GTACCAACACACCAGTAC	32	23	0	0	0	0
GNB1L	TGACCAACACACCAGTTG	471	608	0	1	0	0
GNB1L	ACACCAACACACCACAGT	368	358	3	1	0	0
GNB1L	GTTGCAGTTGCACATGGT	1472	919	0	20	0	1
GNB2L1	GTACGTGTGTACCACATG	262	134	0	0	0	6
GNB2L1	TGACGTGTGTACCAACGT	203	508	1	0	0	0
GNB2L1	GTACGTGTGTACCAACCA	106	106	1	0	0	0
GNB2L1	ACTGCAGTGTTGCATGCA	59	308	0	0	0	0
GNB2L1	GTTGCAGTGTTGCATGAC	32	32	0	0	0	0
GNB3	GTACCAACACCATGACCA	343	237	2	0	1	0
GNB3	CAACCAACACCATGCAAC	6	10	0	0	0	0
GNB3	GTACCAACACCATGCATG	356	407	2	1	0	0
GNB3	TGACCAACACCATGACGT	902	538	2	2	0	0
GNB3	ACACCAACACCATGACAC	228	121	1	1	0	0
GNB3	CATGCAACCATGACGTCA	390	241	65	0	0	0
GNE	TGCATGTGTGTGTGCATG	228	183	0	0	0	0
GNE	ACCATGTGTGTGTGACGT	429	325	4	1	0	0
GNE	TGCATGTGTGTGTGACCA	94	89	0	0	0	0
GNE	CACATGTGTGTGTGACAC	88	36	0	0	0	0
GNE	GTCATGTGTGTGTGACTG	125	193	0	0	0	1
GNG13	CAACCAACACACCAGTGT	1299	1832	2	0	1	10
GNG13	GTACCAACACACGTTGAC	25	45	0	0	0	0
GNG13	TGACCAACACACGTTGTG	266	229	1	0	0	0
GNG13	CATGCAGTTGCACAACTG	1004	642	1	1	0	8
GNG13	TGTGCACAACTGTGGTAC	135	144	0	0	0	0
GNG13	ACTGCACAACTGTGGTTG	1105	1492	0	1	0	0
GNG3	ACACCAACACCATGTGTG	308	369	0	0	0	1
GNG3	ACACCAACACACGTGTGT	443	512	0	0	0	0
GNG3	TGACCAACACACGTGTCA	474	218	0	0	0	3
GNG3	CAACCAACACACGTGTAC	68	278	2	0	0	0
GNG3	GTACCAACACACGTGTTG	243	321	0	0	0	0
GNG3	ACTGCACAACACCATGTG	402	389	1	0	0	0
GNGT1	TGACCAACACACGTCAGT	787	820	0	0	1	3
GNGT1	GTACCAACACACGTCACA	111	98	1	0	0	0
GNGT1	ACACCAACACACGTCAAC	214	59	0	0	0	0
GNGT1	CAACCAACACACGTCATG	97	80	1	0	0	0
GNGT1	GTACCAACACACGTACGT	237	61	0	0	0	0
GNMT	ACCATGCACAACCAGTGT	789	506	1	0	7	15
GNMT	ACCATGCACAACGTTGGT	637	698	0	1	0	0
GNMT	TGCATGCACAACGTTGCA	162	474	2	1	0	0
GNMT	CACATGCACAACGTTGAC	100	127	0	0	0	1
GNMT	GTCATGCACAACGTTGTG	289	353	0	0	0	1
GNPDA1	GTACACCAACTGGTTGGT	144	162	0	1	0	1
GNPDA1	CAACACCAACTGGTTGCA	237	249	1	0	0	0
GNPDA1	TGACACCAACTGGTTGAC	80	31	0	0	0	0
GNPDA1	ACTGCAGTCATGCATGGT	204	430	1	0	0	0
GNPDA1	TGTGCAGTCATGCATGCA	27	62	0	0	0	0
GNPDA2	GTACACACGTTGACACCA	555	292	1	6	1	2
GNPDA2	ACACACACGTTGACACAC	20	10	0	0	0	0
GNPDA2	CAACACACGTTGACACTG	124	127	0	1	0	0
GNPDA2	GTACACACGTTGACTGGT	209	308	1	1	0	0
GNPDA2	CAACACACGTTGACTGCA	344	203	0	0	0	0
GNPDA2	GTTGCAACCACATGGTGT	760	926	0	1	0	9
GNPNAT1	TGACCACACAACCAGTCA	604	519	1	0	1	84
GNPNAT1	CAACCACACAACCAGTAC	36	25	0	0	0	0
GNPNAT1	GTACCACACAACCAGTTG	54	48	0	0	0	0
GNPNAT1	TGACCACACAACCACAGT	287	331	1	0	0	0
GNPNAT1	GTACCACACAACCACACA	27	40	0	0	0	0
GNRH1	CATGGTCAGTTGCATGTG	362	280	0	0	1	5
GNRH1	ACTGGTCAGTTGCATGAC	164	75	0	0	0	0
GNRH1	TGTGGTCAGTTGACGTGT	459	531	1	0	0	15
GNRH1	GTTGGTCAGTTGACGTCA	389	472	0	10	0	0

GNRH1	ACTGGTCAGTTGACGTAC	39	37	0	0	0	0
GNRHR	GTACGTCACAGTCAACGT	279	256	0	0	1	0
GNRHR	ACACGTCACAGTCACAAC	33	43	41	0	0	0
GNRHR	CAACGTCACAGTCACATG	213	240	1	0	0	0
GNRHR	CAACGTCACAGTCAACCA	50	81	1	0	0	0
GNRHR	TGACGTCACAGTCAACAC	146	82	0	0	0	0
GNS	GTACACACACGTTGTGGT	322	276	0	0	0	9
GNS	CAACACACACGTTGTGCA	130	74	0	0	0	0
GNS	TGACACACACGTTGTGAC	42	21	0	0	0	0
GNS	ACACACACACGTTGTGTG	51	51	0	0	0	0
GNS	TGACACACACCAGTGTGT	185	68	0	0	0	0
GOLGA2	TGTGGTTGCAACACGTTG	369	326	0	0	1	0
GOLGA2	CATGGTTGCAACACGTGT	477	373	1	1	0	0
GOLGA2	ACTGGTTGCAACACGTCA	100	177	0	1	0	42
GOLGA2	GTTGGTTGCAACACGTAC	114	61	0	10	0	0
GOLGA2	ACTGCAGTCAGTGTACTG	249	256	0	0	0	0
GOLGA5	TGTGCATGGTACTGCAGT	147	171	0	0	1	0
GOLGA5	GTTGGTCAGTACACACGT	70	84	1	0	0	0
GOLGA5	CATGGTCAGTACACACCA	148	78	0	0	0	0
GOLGA5	TGTGGTCAGTACACACAC	98	125	0	0	0	0
GOLGA5	ACTGGTCAGTACACACTG	433	375	4	0	0	0
GOLGA5	CATGGTCAGTACACTGGT	368	324	0	0	0	2
GOPC	CAACACCAGTACCAGTCA	722	602	2	0	0	2
GOPC	TGACACCAGTACCAGTAC	155	254	1	0	0	0
GOPC	ACACACCAGTACCAGTTG	833	489	0	0	0	0
GOPC	CAACACCAGTACCACAGT	498	539	0	0	0	49
GOPC	CATGACCAGTCAGTTGTG	126	214	0	1	0	0
GORASP1	ACTGGTACACCAGTTGCA	57	50	0	0	0	0
GORASP1	GTTGGTACACCAGTTGAC	23	36	0	0	0	0
GORASP1	TGTGGTACACCAGTTGTG	421	486	0	1	0	24
GORASP1	CATGGTACACCACAGTGT	667	1190	1	1	0	0
GORASP1	ACTGGTACACCACAGTCA	111	81	0	0	0	0
GORASP1	GTTGCATGGTACACGTCA	104	63	0	0	0	0
GOSR1	GTACTGCACATGTGTGTG	172	197	0	0	0	0
GOSR1	ACACTGCAACGTGTGTGT	82	82	0	0	0	0
GOSR1	TGACTGCAACGTGTGTCA	1487	937	4	0	0	3
GOSR1	CAACTGCAACGTGTGTAC	37	91	0	0	0	0
GOSR1	GTACTGCAACGTGTGTTG	291	451	2	0	0	6
GOSR2	CAACTGCAACGTCACAAC	85	75	0	0	0	0
GOSR2	GTACTGCAACGTCACATG	73	144	0	0	0	0
GOSR2	TGACTGCAACGTCAACGT	491	476	0	0	0	0
GOSR2	GTACTGCAACGTCAACCA	304	465	0	0	0	0
GOSR2	ACACTGCAACGTCAACAC	18	21	0	0	0	0
GOT1	ACACCACACATGACACTG	169	158	0	0	0	0
GOT1	CAACCACACATGACTGGT	105	130	0	0	0	0
GOT1	ACACCACACATGACTGCA	241	239	0	0	0	0
GOT1	ACTGCAGTCACACACACA	134	158	0	0	0	0
GOT1	GTTGCAGTCACACACAAC	151	68	0	0	0	0
GOT1	TGTGCACAGTACTGACCA	150	166	0	1	0	7
GOT2	GTACCACAACGTGTCAAC	52	36	0	0	0	0
GOT2	TGACCACAACGTGTCATG	115	109	0	0	0	0
GOT2	ACACCACAACGTGTACGT	36	67	0	0	0	0
GOT2	TGACCACAACGTGTACCA	541	74	0	0	0	0
GOT2	CAACCACAACGTGTACAC	5	3	0	0	0	0
GP1BA	ACTGGTCAGTCAACACGT	81	20	0	0	0	0
GP1BA	TGTGGTCAGTCAACACCA	154	165	0	1	0	1
GP1BA	CATGGTCAGTCAACACAC	80	42	0	0	0	0
GP1BA	GTTGGTCAGTCAACACTG	523	275	0	0	0	0
GP1BA	TGTGGTCAGTCAACTGGT	577	385	0	0	0	0
GP5	TGTGGTACCAGTGTCACA	41	41	0	0	0	0
GP5	CATGGTACCAGTGTCAAC	53	26	0	0	0	0
GP5	GTTGGTACCAGTGTCATG	351	329	0	0	0	0
GP5	TGTGGTACCAGTGTACGT	138	164	1	0	0	0
GP5	GTTGGTACCAGTGTACCA	113	110	0	0	0	0
GP5	CATGCACACATGGTTGCA	107	94	0	0	0	0
GP6	ACACTGACCAGTCACACA	208	196	0	0	0	0
GP6	GTACTGACCAGTCACAAC	32	28	0	0	0	0
GP6	TGACTGACCAGTCACATG	312	280	2	0	0	1
GP6	ACACTGACCAGTCAACGT	335	269	1	1	0	0
GP6	GTTGACACACACACGTGT	705	184	2	0	0	0
GP9	GTTGGTCAGTTGCATGCA	345	246	0	1	1	1
GP9	CATGGTCAGTTGCAACAC	81	40	1	0	0	0
GP9	GTTGGTCAGTTGCAACTG	328	366	1	0	0	1
GP9	TGTGGTCAGTTGCATGGT	296	322	1	0	0	0
GP9	ACTGCACAGTGTACACGT	132	119	0	0	0	0

GPAA1	GTTGGTTGTGTGCAACTG	335	424	2	2	1	3
GPAA1	TGTGGTTGTGTGCAACCA	219	132	0	0	0	0
GPAA1	CATGGTTGTGTGCAACAC	97	289	0	0	0	0
GPAA1	TGTGGTTGTGTGCATGGT	930	348	1	2	0	0
GPAA1	GTTGACCAACCAGTTGTG	522	396	1	0	0	0
GPAM	CAACCAACGTTGTGGTGT	567	646	2	1	1	0
GPAM	TGACCAACGTTGACTGTG	296	179	0	1	0	0
GPAM	ACACCAACGTTGTGGTCA	312	391	3	0	0	0
GPAM	GTACCAACGTTGTGGTAC	22	18	0	0	0	0
GPAM	GTTGCAGTACTGCATGGT	51	116	0	0	0	0
GPC5	ACTGGTTGCAACACCAGT	83	82	0	0	0	0
GPC5	TGTGGTTGCAACACCACA	26	24	0	0	0	0
GPC5	CATGGTTGCAACACCAAC	17	32	0	0	0	0
GPC5	GTTGGTTGCAACACCATG	134	220	0	2	0	3
GPC5	ACTGCACAGTGTCAACAC	86	78	0	0	0	0
GPD2	ACACCAGTGTCAGTCATG	68	67	0	1	0	0
GPD2	CAACCAGTGTCAGTACGT	66	43	0	0	0	0
GPD2	ACACCAGTGTCAGTACCA	330	452	1	0	0	0
GPD2	GTACCAGTGTCAGTACAC	18	19	0	0	0	0
GPD2	TGACCAGTGTCAGTACTG	234	278	1	0	0	0
GPHN	TGTGGTGTCAGTTGTGGT	209	288	0	0	1	0
GPHN	CATGGTCAGTTGACGTTG	88	117	0	0	1	0
GPHN	GTTGGTCAGTTGACCAGT	395	428	1	1	1	0
GPHN	GTTGGTGTCAGTTGACTG	146	167	0	0	0	2
GPHN	GTTGGTGTCAGTTGTGCA	153	177	1	0	0	1
GPI	ACACACCAACACCATGGT	124	121	0	0	0	1
GPI	TGACACCAACACCATGCA	119	198	0	0	0	2
GPI	CAACACCAACACCATGAC	102	40	0	0	0	0
GPI	GTACACCAACACCATGTG	714	698	0	0	0	0
GPI	ACACACCAACACACGTGT	123	205	1	1	0	0
GPI	GTTGCAACGTTGTGCACA	13	20	0	0	0	9
GPLD1	ACACACACGTACACACGT	22	7	0	0	0	0
GPLD1	TGACACACGTACACACCA	205	223	1	0	0	0
GPLD1	CAACACACGTACACACAC	176	85	0	0	0	1
GPLD1	GTACACACGTACACACTG	181	204	0	0	0	0
GPLD1	TGACACACGTACACTGGT	144	105	1	0	0	0
GPLD1	TGTGCATGGTACGTACGT	56	56	0	0	0	0
GPR109B	GTACGTCACAACCAGTCA	476	676	0	0	0	0
GPR109B	ACACGTCACAACCAGTAC	147	149	1	0	0	0
GPR109B	CAACGTCACAACCAGTTG	940	1370	2	1	0	0
GPR109B	GTACGTCACAACCACAGT	176	208	5	0	0	2
GPR109B	CAACGTCACAACCACACA	63	43	0	0	0	0
GPR109B	CATGCACAACGTACGTTG	31	63	0	0	0	0
GPR132	TGACGTCAGTACCATGTG	105	426	1	0	1	0
GPR132	GTACGTCAGTACCATGAC	245	82	0	0	0	0
GPR132	CAACGTCAGTACACGTGT	1111	1082	2	2	0	2
GPR132	ACACGTCAGTACACGTCA	271	170	1	0	0	1
GPR132	ACTGCAGTACCATGACGT	278	238	0	4	0	0
GPR132	GTTGCATGGTACTGGTTG	1346	701	1	1	0	0
GPR161	CAACGTACGTGTGTCACA	174	45	0	0	1	5
GPR161	CAACGTACGTGTGTGTTG	177	490	0	2	0	0
GPR161	GTACGTACGTGTGTCAGT	149	138	0	0	0	4
GPR161	TGACGTACGTGTGTCAAC	29	26	0	0	0	0
GPR161	ACACGTACGTGTGTCATG	79	71	0	0	0	0
GPR171	TGACGTCACAACGTTGGT	58	52	0	0	0	0
GPR171	GTACGTCACAACGTTGCA	58	99	0	0	0	0
GPR171	ACACGTCACAACGTTGAC	13	19	0	0	0	0
GPR171	CAACGTCACAACGTTGTG	151	167	0	0	0	0
GPR171	TGACGTCACAACCAGTGT	196	212	0	0	0	0
GPR171	ACTGCATGGTTGTGCATG	15	79	1	0	0	0
GPR18	ACACGTCACAGTCATGCA	97	89	281	0	1	0
GPR18	ACACGTCACAGTCAACTG	270	339	0	0	0	0
GPR18	CAACGTCACAGTCATGGT	256	185	1	1	0	0
GPR18	GTACGTCACAGTCATGAC	102	50	0	0	0	0
GPR18	TGACGTCACAGTCATGTG	352	819	2	0	0	0
GPR34	GTACGTCACAGTACTGGT	835	647	3	0	1	0
GPR34	GTACGTCACAGTACACCA	191	226	0	0	0	0
GPR34	ACACGTCACAGTACACAC	79	81	0	0	0	0
GPR34	CAACGTCACAGTACACTG	835	676	2	1	0	1
GPR34	CATGACACCATGACTGAC	20	41	0	0	0	0
GPR35	CAACGTCACAGTACTGCA	127	74	0	0	0	6
GPR35	TGACGTCACAGTACTGAC	11	25	0	0	0	0
GPR35	ACACGTCACAGTACTGTG	221	280	0	2	0	0
GPR35	GTACGTCACAGTTGGTGT	429	976	0	0	0	1
GPR35	CAACGTCACAGTTGGTCA	374	478	0	0	0	0

GPR37	TGACGTCACAGTTGGTAC	20	22	0	0	0	0
GPR37	ACACGTCACAGTTGGTTG	359	499	2	0	0	1
GPR37	CAACGTCACAGTTGCAGT	255	130	0	0	0	0
GPR37	ACACGTCACAGTTGCACA	194	50	0	0	0	0
GPR37	GTACGTCACAGTTGCAAC	48	44	1	0	0	1
GPR37	ACTGCATGGTTGTGACCA	105	91	1	0	0	0
GPR39	TGCATGTGCACAACACGT	38	30	0	0	0	0
GPR39	GTCATGTGCACAACACCA	78	93	0	0	0	0
GPR39	ACCATGTGCACAACACAC	41	27	0	0	0	0
GPR39	CACATGTGCACAACACTG	739	433	0	1	0	0
GPR39	ACTGCAGTACCAGTCATG	164	147	1	0	0	0
GPR45	GTCATGTGACTGACCACA	96	207	0	1	0	0
GPR45	ACCATGTGACTGACCAAC	12	12	0	0	0	0
GPR45	CACATGTGACTGACCATG	182	415	1	0	0	0
GPR45	GTCATGTGACTGACACGT	42	25	0	0	0	0
GPR45	CACATGTGACTGACACCA	230	151	0	0	0	0
GPR45	ACTGCATGGTTGCAACAC	7	7	0	0	0	0
GPR50	TGACGTCACAGTTGCATG	354	312	2	0	0	0
GPR50	ACACGTCACAGTTGACGT	105	127	0	0	0	0
GPR50	TGACGTCACAGTTGACCA	261	251	0	0	0	0
GPR50	CAACGTCACAGTTGACAC	29	22	0	0	0	0
GPR50	GTACGTCACAGTTGACTG	439	424	27	0	0	1
GPR64	TGACGTCACAGTTGTGGT	227	233	0	0	1	0
GPR64	GTACGTCACAGTTGTGCA	253	139	0	0	0	0
GPR64	ACACGTCACAGTTGTGAC	55	55	0	0	0	0
GPR64	CAACGTCACAGTTGTGTG	186	220	0	0	0	0
GPR64	CATGACACCATGGTACTG	180	185	1	0	0	4
GPR65	ACACGTCACACAGTGTGT	628	460	2	0	0	0
GPR65	TGACGTCACACAGTGTCA	577	598	3	1	0	1
GPR65	CAACGTCACACAGTGTAC	121	61	0	0	0	0
GPR65	GTACGTCACACAGTGTTG	685	536	2	2	0	26
GPR65	TGACGTCACACAGTCAGT	163	244	1	1	0	0
GPR68	GTACGTCACACAGTCACA	77	80	0	0	0	0
GPR68	ACACGTCACACAGTCAAC	124	133	0	1	0	1
GPR68	CAACGTCACACAGTCATG	139	237	0	0	0	0
GPR68	GTACGTCACACAGTACGT	148	132	0	0	0	1
GPR68	CAACGTCACACAGTACCA	89	134	0	0	0	0
GPR75	TGACGTCACACAGTTGTG	405	389	0	1	0	0
GPR75	CAACGTCACACACAGTGT	403	349	0	0	0	0
GPR75	ACACGTCACACACAGTCA	470	433	1	0	0	0
GPR75	GTACGTCACACACAGTAC	48	145	0	0	0	0
GPR75	TGACGTCACACACAGTTG	188	187	1	0	0	0
GPR75	CATGCACAACCACATGAC	42	47	0	0	0	0
GPR77	ACACGTCACACACACAGT	51	107	0	0	0	0
GPR77	TGACGTCACACACACACA	78	132	1	0	0	0
GPR77	CAACGTCACACACACAAC	26	75	1	4	0	4
GPR77	GTACGTCACACACACATG	488	163	0	0	0	0
GPR77	TGTGCAGTACACGTCACA	79	109	0	1	0	0
GPR77	CATGCACATGGTACACCA	97	61	2	0	0	0
GPR81	GTACGTCACACACAACCA	615	937	0	0	1	1
GPR81	TGACGTCACACACAACGT	223	297	1	0	0	12
GPR81	ACACGTCACACACAACAC	50	37	0	0	0	0
GPR81	CAACGTCACACACAACTG	275	136	0	0	0	0
GPR81	GTACGTCACACACATGGT	258	459	0	0	0	0
GPR81	TGTGCAACCATGTGACTG	611	677	1	0	0	1
GPR83	ACACGTCACACAGTACTG	173	178	0	0	1	0
GPR83	ACACGTCACACAGTTGCA	333	272	1	0	1	0
GPR83	CAACGTCACACAGTTGGT	1096	639	0	0	0	0
GPR83	GTACGTCACACAGTTGAC	104	83	0	0	0	0
GPR83	TGTGCATGCAACACTGGT	161	121	0	1	0	1
GPRC5A	TGCATGTGACTGGTTGGT	400	562	0	0	1	1
GPRC5A	ACCATGTGACTGGTACGT	311	425	0	1	0	0
GPRC5A	TGCATGTGACTGGTACCA	119	55	0	0	0	0
GPRC5A	CACATGTGACTGGTACAC	75	70	0	0	0	0
GPRC5A	GTCATGTGACTGGTACTG	675	261	1	1	0	0
GPRC5B	CAACGTCACACACATGCA	445	597	2	1	12	8
GPRC5B	TGACGTCACACACATGAC	41	47	0	0	0	0
GPRC5B	ACACGTCACACACATGTG	220	361	0	0	0	0
GPRC5B	GTACGTCACACAACGTGT	840	668	0	1	0	0
GPRC5B	CAACGTCACACAACGTCA	253	213	0	0	0	0
GPRC5B	GTTGCACAACTGTGGTGT	526	484	1	2	0	2
GPRC5C	TGACGTCACACAACGTAC	38	33	0	0	0	0
GPRC5C	ACACGTCACACAACGTTG	630	1077	1	0	0	7
GPRC5C	CAACGTCACACAACCAGT	671	912	0	0	0	3
GPRC5C	ACACGTCACACAACCACA	310	338	0	1	0	4

GPRC5C	GTACGTCACACAACCAAC	5	4	0	0	0	0
GPRC5D	CACATGTGACTGGTTGTG	290	385	1	2	2	0
GPRC5D	GTCATGTGACTGGTTGCA	269	242	1	0	0	0
GPRC5D	ACCATGTGACTGGTTGAC	39	35	0	1	0	0
GPRC5D	TGCATGTGACTGCAGTGT	376	531	1	0	0	0
GPRC5D	GTCATGTGACTGCAGTCA	251	405	0	0	0	0
GPRC5D	TGTGCACATGGTACCAGT	146	133	0	0	0	0
GPSM1	CAACCAACACCATGACTG	141	198	0	0	0	0
GPSM1	GTACCAACACCATGTGGT	395	103	2	1	0	1
GPSM1	CAACCAACACCATGTGCA	309	298	0	0	0	4
GPSM1	TGACCAACACCATGTGAC	23	34	0	0	0	0
GPSM1	ACTGACACGTACTGACGT	162	150	2	0	0	2
GPSN2	TGTGGTTGCAACACACGT	55	57	0	0	0	0
GPSN2	GTTGGTTGCAACACACCA	358	217	0	0	0	0
GPSN2	ACTGGTTGCAACACACAC	40	39	0	0	0	0
GPSN2	CATGGTTGCAACACACTG	258	263	1	0	0	0
GPSN2	GTTGGTTGCAACACTGGT	194	285	0	0	0	0
GPT	TGACCACAACCAACGTTG	558	457	10	10	7	9
GPT	ACACCACAACCAACCAGT	96	370	0	0	0	0
GPT	TGACCACAACCAACCACA	59	60	0	0	0	0
GPT	CAACCACAACCAACCAAC	21	46	0	0	0	0
GPT	GTACCACAACCAACCATG	461	671	0	1	0	0
GPT	CATGCACACATGTGACCA	398	274	0	0	0	0
GPT2	GTACCACAACACGTGTAC	33	16	0	0	0	0
GPT2	TGACCACAACACGTGTTG	272	286	1	0	0	11
GPT2	ACACCACAACACGTCAGT	408	300	0	0	0	4
GPT2	TGACCACAACACGTCACA	118	178	0	0	0	1
GPT2	CAACCACAACACGTCAAC	31	39	0	0	0	0
GPT2	GTTGCAACCAGTACCACA	78	83	0	0	0	0
GPX1	ACACACGTTGCAGTTGAC	29	13	0	0	0	0
GPX1	CAACACGTTGCAGTTGTG	320	149	0	0	0	0
GPX1	TGACACGTTGCACAGTGT	451	680	1	1	0	0
GPX1	ACTGACGTGTGTACTGCA	65	157	0	0	0	0
GPX1	GTTGACGTGTGTACTGAC	28	34	0	0	0	0
GPX2	CAACACGTTGCACAGTTG	580	528	1	0	2	2
GPX2	GTACACGTTGCACAGTCA	232	180	1	0	0	0
GPX2	ACACACGTTGCACAGTAC	109	86	0	0	0	0
GPX2	GTACACGTTGCACACAGT	80	228	0	0	0	0
GPX2	CAACACGTTGCACACACA	73	79	0	0	0	0
GPX3	GTACGTCAGTTGACCACA	55	79	0	0	2	0
GPX3	ACACGTCAGTTGACCAAC	59	18	0	0	0	0
GPX3	CAACGTCAGTTGACCATG	45	60	0	0	0	0
GPX3	GTACGTCAGTTGACACGT	61	81	0	0	0	0
GPX3	CAACGTCAGTTGACACCA	106	130	0	0	0	0
GPX4	GTACACGTTGCACAACAC	100	94	0	0	0	1
GPX4	TGACACGTTGCACAACTG	802	871	0	0	0	0
GPX4	ACACACGTTGCACATGGT	1441	758	0	0	0	1
GPX4	TGACACGTTGCACATGCA	127	174	0	0	0	0
GPX4	CAACACGTTGCACATGAC	32	28	0	0	0	0
GPX5	ACTGCACACAACGTACGT	249	303	0	0	1	0
GPX5	CAACACGTTGGTCACAGT	172	137	0	0	0	0
GPX5	ACACACGTTGGTCACACA	255	135	0	0	0	0
GPX5	GTACACGTTGGTCACAAC	5	12	0	0	0	0
GPX5	TGACACGTTGGTCACATG	401	248	1	0	0	0
GPX5	ACACACGTTGGTCAACGT	131	162	0	0	0	0
GPX6	GTACACGTTGGTGTGTAC	13	43	0	0	0	0
GPX6	TGACACGTTGGTGTGTTG	29	35	0	0	0	0
GPX6	ACACACGTTGGTGTCAGT	51	144	0	0	0	0
GPX6	TGACACGTTGGTGTCACA	66	159	0	0	0	0
GPX6	CAACACGTTGGTGTCAAC	24	12	0	0	0	0
GPX7	GTACACGTTGGTACTGTG	179	150	0	0	0	0
GPX7	ACACACGTTGGTTGGTGT	304	398	0	0	0	0
GPX7	TGACACGTTGGTTGGTCA	237	190	0	0	0	1
GPX7	CAACACGTTGGTTGGTAC	64	41	0	0	0	0
GPX7	TGTGACACACACGTGTCA	38	82	4	0	0	0
GRAP	GTTGGTGTCAACACCACA	146	56	0	0	0	0
GRAP	GTTGACACGTTGACTGAC	78	70	0	0	0	0
GRAP2	ACTGGTGTCAACACACTG	627	257	1	0	1	0
GRAP2	ACTGGTGTCAACACTGCA	511	254	0	0	1	0
GRAP2	TGTGGTGTCAACACACAC	0	6	0	0	0	0
GRAP2	CATGGTGTCAACACTGGT	64	139	0	0	0	0
GRAP2	GTTGGTGTCAACACTGAC	32	115	0	0	0	0
GRAP2	CATGCAACCACAGTGTTG	635	644	3	46	0	9
GRASP	TGACTGCAACTGTGCACA	101	119	1	1	1	0
GRASP	ACACTGCAACTGTGCAGT	263	305	1	1	0	0

GRASP	CAACTGCAACTGTGCAAC	52	40	1	0	0	3
GRASP	GTACTGCAACTGTGCATG	363	240	0	0	0	1
GRASP	TGACTGCAACTGTGACGT	154	140	0	0	0	0
GRB10	ACACTGACCAGTTGGTGT	405	421	0	0	0	0
GRB10	TGACTGACCAGTTGGTCA	340	101	1	0	0	0
GRB10	CAACTGACCAGTTGGTAC	60	65	0	0	0	0
GRB10	GTACTGACCAGTTGGTTG	799	358	1	0	0	0
GRB10	TGACTGACCAGTTGCAGT	85	105	0	0	0	1
GRB14	GTACTGCATGGTCAACTG	1024	1637	1	3	2	0
GRB14	ACACTGCATGGTCAACGT	950	955	1	0	0	2
GRB14	TGACTGCATGGTCAACCA	194	138	0	0	0	0
GRB14	CAACTGCATGGTCAACAC	190	127	0	0	0	0
GRB14	TGACTGCATGGTCATGGT	122	160	0	0	0	0
GRB2	ACACCAGTACACTGACAC	31	37	0	0	0	0
GRB2	CAACCAGTACACTGACTG	586	527	1	1	0	0
GRB2	GTACCAGTACACTGTGGT	45	33	0	0	0	0
GRB2	CAACCAGTACACTGTGCA	46	58	2	0	0	4
GRB2	TGACCAGTACACTGTGAC	12	3	0	0	0	0
GRB7	GTACTGCAACTGACCACA	1	6	0	0	0	0
GRB7	ACACTGCAACTGACCAAC	23	38	0	0	0	0
GRB7	CAACTGCAACTGACCATG	99	71	1	0	0	0
GRB7	GTACTGCAACTGACACGT	86	111	0	0	0	0
GRB7	CAACTGCAACTGACACCA	771	1091	1	1	0	0
GREB1	TGTGACACCACACAACGT	345	701	0	0	1	1
GREB1	GTCATGCACAACGTGTCA	453	389	1	0	0	0
GREB1	CATGGTCATGCAACACCA	95	142	0	0	0	0
GREB1	TGTGCAGTGTTGCAACAC	124	49	0	0	0	13
GREB1	GTTGACACCACACACATG	400	418	0	0	0	0
GRHPR	TGACACGTTGTGGTGTGT	651	421	0	0	1	17
GRHPR	CAACCATGACCACATGTG	367	293	1	0	0	0
GRHPR	TGACCATGACCAACGTGT	1130	484	1	1	0	1
GRHPR	GTACACGTTGACTGTGTG	189	218	0	0	0	0
GRHPR	GTACACGTTGTGGTGTCA	56	32	1	0	0	0
GRHPR	GTTGCACAACACACGTGT	220	252	1	0	0	0
GRIA1	ACACTGCATGGTACGTAC	98	232	1	0	0	2
GRIA1	CAACTGCATGGTACGTTG	604	290	0	1	0	0
GRIA1	GTACTGCATGGTACCAGT	145	125	0	0	0	0
GRIA1	GTTGCAGTTGACTGTGGT	930	555	2	0	0	5
GRIA1	CATGCACAACACGTACCA	220	245	0	0	0	0
GRIA1	TGTGCACAACACGTACAC	33	25	0	0	0	0
GRIA2	CAACTGCATGGTACTGAC	56	44	0	0	0	0
GRIA2	GTACTGCATGGTACTGTG	173	120	0	0	0	0
GRIA2	ACACTGCATGGTTGGTGT	1661	1094	1	0	0	3
GRIA2	TGACTGCATGGTTGGTCA	202	52	0	0	0	0
GRIA2	CAACTGCATGGTTGGTAC	227	36	0	0	0	0
GRIA3	GTACTGCATGGTTGACGT	391	538	1	2	2	1
GRIA3	CAACTGCATGGTTGCATG	71	181	2	0	0	0
GRIA3	CATGGTGTCACACACAAC	21	17	0	0	0	0
GRIA3	GTTGGTGTCACACACATG	163	152	1	0	0	0
GRIA3	CATGCAGTTGTGGTGTGT	58	123	0	0	0	0
GRIA4	TGACTGACGTACGTGTTG	360	757	0	0	0	2
GRIA4	ACACTGACGTACGTCAGT	26	40	0	0	0	0
GRIA4	GTTGGTGTCACACAACCA	143	156	0	0	0	0
GRIA4	ACTGACACCACAGTGTGT	39	117	0	0	0	0
GRIA4	TGTGACACCACAGTGTCA	472	267	0	1	0	7
GRID2	GTACTGACGTACCAACTG	172	220	1058	2	1	0
GRID2	TGACTGACGTACCATGGT	461	435	0	0	1	1
GRID2	GTACTGACGTACCATGCA	29	68	0	0	0	0
GRID2	ACACTGACGTACCATGAC	45	26	0	0	0	0
GRID2	CAACTGACGTACCATGTG	221	247	0	2	0	0
GRID2	TGTGCACACAACGTACCA	88	70	0	0	0	0
GRIK3	TGACTGACGTTGGTACAC	9	28	0	0	0	0
GRIK3	ACACTGACGTTGGTACTG	201	157	0	0	0	0
GRIK3	CAACTGACGTTGGTTGGT	367	267	1	0	0	0
GRIK3	ACACTGACGTTGGTTGCA	292	281	0	1	0	0
GRIK3	GTACTGACGTTGGTTGAC	74	81	0	0	0	0
GRIK3	GTTGCATGGTCAACGTAC	104	69	0	1	0	0
GRIK4	TGACTGACGTTGCAGTTG	867	1187	0	38	1	0
GRIK4	TGACTGACGTTGGTTGTG	806	1337	388	0	0	0
GRIK4	CAACTGACGTTGCAGTGT	366	340	0	0	0	0
GRIK4	ACACTGACGTTGCAGTCA	267	255	0	0	0	0
GRIK4	GTACTGACGTTGCAGTAC	71	47	0	0	0	3
GRIK4	TGTGCATGGTCATGTGGT	584	550	1	1	0	8
GRIN1	GTACTGACGTTGACGTGT	386	412	1	4	1	0
GRIN1	CAACTGACGTTGCATGCA	467	543	0	1	0	2

GRIN1	TGACTGACGTTGCATGAC	39	74	0	0	0	0
GRIN1	ACACTGACGTTGCATGTG	57	94	0	0	0	0
GRIN1	CAACTGACGTTGACGTCA	292	441	0	0	0	0
GRIN2A	TGACTGACGTTGTGGTGT	306	398	0	1	0	0
GRIN2A	GTACTGACGTTGTGGTCA	66	221	0	0	0	0
GRIN2A	ACACTGACGTTGTGGTAC	39	61	0	0	0	0
GRIN2A	CAACTGACGTTGTGGTTG	220	362	2	1	0	0
GRIN2A	GTACTGACGTTGTGCAGT	67	44	0	0	0	0
GRIN2A	ACTGCACAACCAACACTG	774	604	0	1	0	1
GRIN2B	CATGCACAACCAACTGGT	544	393	0	1	1	0
GRIN2B	GTACTGACCAGTGTGTAC	181	207	0	0	0	0
GRIN2B	TGACTGACCAGTGTGTTG	254	266	1	0	0	0
GRIN2B	ACACTGACCAGTGTCAGT	188	254	0	0	0	0
GRIN2B	TGACTGACCAGTGTCACA	62	70	0	0	0	0
GRIN2B	CAACTGACCAGTGTCAAC	92	113	0	0	0	0
GRIN2C	GTACTGACCAGTGTTGGT	938	639	0	2	0	1
GRIN2C	CAACTGACCAGTGTTGCA	166	189	0	0	0	0
GRIN2C	TGACTGACCAGTGTTGAC	28	103	0	0	0	1
GRIN2C	ACACTGACCAGTGTTGTG	620	332	0	1	0	0
GRIN2C	GTACTGACCAGTCAGTGT	787	386	1	1	0	0
GRIN2D	CATGCAGTGTCAACTGGT	206	211	2	1869	1	2
GRIN2D	CAACTGACCAGTCAGTCA	234	304	1	0	0	1
GRIN2D	TGACTGACCAGTCAGTAC	51	44	1	0	0	0
GRIN2D	ACACTGACCAGTCAGTTG	185	281	0	0	0	0
GRIN2D	CAACTGACCAGTCACAGT	242	154	0	0	0	0
GRIN3A	CAACTGACGTACTGTGGT	264	324	1	0	1	0
GRIN3A	ACACTGACGTACTGTGCA	110	115	0	0	0	0
GRIN3A	GTACTGACGTACTGTGAC	104	43	0	0	0	0
GRIN3A	TGACTGACGTACTGTGTG	23	74	1	0	0	0
GRIN3A	ACACTGACGTTGGTGTGT	369	484	0	0	0	0
GRIN3A	TGTGCAACCAACCACATG	259	277	0	0	0	0
GRIN3B	CATGGTTGACTGTGGTTG	590	520	10	3	20	30
GRIN3B	TGTGGTTGACTGTGCAAC	31	40	0	0	1	0
GRIN3B	GTTGGTTGACTGTGCAGT	267	220	0	0	0	0
GRIN3B	CATGGTTGACTGTGCACA	116	73	0	0	0	0
GRIN3B	ACTGGTTGACTGTGCATG	150	153	0	1	0	0
GRIP1	ACTGCAGTGTCACAGTAC	111	150	13	14195	11	1
GRIP1	CATGCAGTGTCACAGTTG	256	383	0	29	0	0
GRIP1	GTTGCAGTGTCACACAGT	323	153	0	1	0	0
GRIP1	CATGCAGTGTCACACACA	9	52	0	1	0	0
GRIP1	TGTGCAGTGTCACACAAC	28	23	0	0	0	0
GRK1	GTCATGACGTGTCAGTAC	68	19	0	0	0	0
GRK1	TGCATGACGTGTCAGTTG	1074	533	1	0	0	0
GRK1	ACCATGACGTGTCACAGT	146	213	1	0	0	0
GRK1	TGCATGACGTGTCACACA	21	40	0	0	0	0
GRK1	ACTGGTTGGTGTTGGTGT	1064	516	3	0	0	1
GRK4	TGCATGCATGCAACTGGT	332	277	1	0	0	1
GRK4	GTCATGCATGCAACTGCA	78	28	1	0	0	0
GRK4	ACCATGCATGCAACTGAC	76	53	0	0	0	0
GRK4	CACATGCATGCAACTGTG	360	502	0	0	0	0
GRK4	TGCATGCATGCATGGTGT	141	198	0	0	0	0
GRK5	TGCATGCATGGTCACAGT	92	152	0	0	0	5
GRK5	GTCATGCATGGTCACACA	69	66	0	0	0	0
GRK5	ACCATGCATGGTCACAAC	43	19	0	0	0	0
GRK5	CACATGCATGGTCACATG	341	243	0	0	0	0
GRK5	ACTGGTTGTGACGTACGT	74	44	0	0	0	0
GRM1	TGACGTCACACAACCATG	131	244	0	0	2	1
GRM1	ACACGTCACACAACACGT	72	73	0	0	0	0
GRM1	TGACGTCACACAACACCA	153	355	0	0	0	0
GRM1	CAACGTCACACAACACAC	51	60	0	0	0	0
GRM1	GTACGTCACACAACACTG	604	521	0	0	0	0
GRM1	ACTGCACAACCAACTGCA	383	359	0	0	0	0
GRM2	TGACGTCACACAACTGGT	268	222	0	0	0	19
GRM2	GTACGTCACACAACTGCA	711	260	0	0	0	0
GRM2	ACACGTCACACAACTGAC	32	41	0	0	0	0
GRM2	CAACGTCACACAACTGTG	152	188	0	0	0	0
GRM2	GTTGCAGTACCATGACTG	367	352	0	0	0	0
GRM3	TGACGTCACACATGGTGT	415	388	0	0	0	81
GRM3	GTACGTCACACATGGTCA	266	317	0	0	0	0
GRM3	ACACGTCACACATGGTAC	218	103	0	0	0	0
GRM3	CAACGTCACACATGGTTG	131	134	0	0	0	0
GRM3	ACACGTTGTGACTGTGAC	14	24	0	0	0	0
GRM4	TGTGCAGTACCATGTGGT	1081	986	4	2	1	2
GRM4	GTACGTCACACATGCAGT	163	133	6	0	0	0
GRM4	CAACGTCACACATGCACA	18	36	0	0	0	0

GRM4	TGACGTCACACATGCAAC	38	125	0	0	0	0
GRM4	ACACGTCACACATGCATG	1055	716	1	1	0	4
GRM4	GTTGCACAGTACTGACTG	435	275	0	0	0	0
GRM5	ACACGTCACACATGACCA	131	117	0	0	1	0
GRM5	CAACGTCACACATGACGT	1	0	0	0	0	0
GRM5	GTACGTCACACATGACAC	62	25	0	0	0	0
GRM5	TGACGTCACACATGACTG	1637	943	1	8	0	1
GRM5	ACACGTCACACATGTGGT	91	87	0	0	0	0
GRM5	TGTGCACAGTACTGTGGT	298	148	0	0	0	0
GRM6	TGACGTCACACATGTGCA	109	115	20	0	0	0
GRM6	CAACGTCACACATGTGAC	50	46	1	0	0	0
GRM6	GTACGTCACACATGTGTG	251	341	0	0	0	1
GRM6	GTACGTCACAACGTGTGT	880	713	0	1	0	0
GRM6	CAACGTCACAACGTGTCA	329	559	0	0	0	0
GRM7	TGACGTCACAACGTGTAC	86	85	0	0	0	0
GRM7	ACACGTCACAACGTGTTG	150	118	0	0	0	0
GRM7	CAACGTCACAACGTCAGT	117	148	1	0	0	0
GRM7	ACACGTCACAACGTCACA	148	140	0	0	0	0
GRM7	GTACGTCACAACGTCAAC	8	92	0	0	0	0
GRM8	TGACGTCACAACGTCATG	44	101	0	0	0	0
GRM8	ACACGTCACAACGTACGT	105	104	0	0	0	1
GRM8	TGACGTCACAACGTACCA	191	368	0	0	0	0
GRM8	CAACGTCACAACGTACAC	59	50	0	0	0	0
GRM8	GTACGTCACAACGTACTG	108	63	0	0	0	0
GRM8	GTTGCACAGTACTGTGCA	82	80	0	0	0	0
GRN	GTTGGTGTACACGTTGAC	25	23	0	0	0	0
GRN	TGTGGTGTACACGTTGTG	131	397	0	0	0	0
GRN	CATGGTGTACACCAGTGT	583	345	0	1	0	0
GRN	ACTGGTGTACACCAGTCA	339	375	0	2	0	1
GRN	GTTGGTGTACACCAGTAC	28	20	0	0	0	1
GRWD1	ACTGGTCACATGGTACTG	587	555	280	274	301	703231
GRWD1	CATGGTCACATGGTACCA	146	286	1	0	0	0
GRWD1	TGTGGTCACATGGTACAC	69	49	0	0	0	10
GRWD1	CATGGTCACATGGTTGGT	38	63	2	0	0	0
GRWD1	CATGGTTGCAACACTGCA	224	269	0	0	0	1
GRWD1	TGTGCATGCAGTGTACGT	79	107	0	0	0	0
GSK3A	CAACCATGGTCACAACTG	312	427	0	0	1	0
GSK3A	GTACGTTGTGACGTACAC	10	8	0	0	0	0
GSK3A	ACACCATGGTCACAACAC	13	31	2	0	0	0
GSK3A	GTACCATGGTCACATGGT	44	52	0	0	0	0
GSK3A	CAACCATGGTCACATGCA	75	98	1	0	0	0
GSK3B	CACATGCATGGTGTGTTG	251	208	1	1	1	0
GSK3B	GTCATGCATGGTGTCAGT	111	139	0	0	0	1
GSK3B	CACATGCATGGTGTCACA	97	73	0	0	0	0
GSK3B	ACACGTCAACCATGGTCA	284	399	0	0	0	1
GSK3B	GTACGTCAACCATGGTAC	67	89	0	0	0	0
GSK3B	CATGCAACACGTCACAAC	61	78	0	0	0	0
GSN	ACACCAGTTGACACCAGT	135	104	0	0	0	0
GSN	TGACCAGTTGACACCACA	383	116	0	0	0	0
GSN	CAACCAGTTGACACCAAC	11	28	1	0	0	0
GSN	GTACCAGTTGACACCATG	161	191	1	0	0	0
GSN	TGACCAGTTGACACACGT	284	223	0	0	0	0
GSR	CAACACGTTGACCATGCA	82	90	7	0	0	0
GSR	TGACACGTTGACCATGAC	11	15	0	3	0	0
GSR	ACACACGTTGACCATGTG	405	286	0	1	0	0
GSR	GTACACGTTGACACGTGT	698	545	2	0	0	15
GSR	CAACACGTTGACACGTCA	534	320	1	0	0	2
GSS	CAACACGTACCACATGTG	1405	2223	25	12	22	21
GSS	GTACACGTACCACAACTG	339	366	0	0	0	0
GSS	TGACACGTACCACATGGT	521	423	0	0	0	0
GSS	GTACACGTACCACATGCA	87	160	1	0	0	0
GSS	ACACACGTACCACATGAC	20	42	0	0	0	1
GSS	TGTGCATGGTTGGTGTCA	399	500	0	0	0	0
GSTA1	ACTGGTTGGTACCATGTG	1320	565	2	0	1	4
GSTA1	GTTGGTACACGTCAACGT	124	135	0	0	0	0
GSTA1	CATGGTTGGTACCAACTG	100	45	0	0	0	1
GSTA1	GTTGGTTGGTACCATGGT	92	80	0	0	0	0
GSTA1	TGTGGTTGGTACCATGAC	20	16	0	0	0	0
GSTA2	CATGGTACCATGTGGTCA	670	394	2	0	0	0
GSTA2	TGTGGTACACGTCACAGT	262	264	0	1	0	1
GSTA2	GTTGGTACACGTCACACA	114	106	1	0	0	0
GSTA2	ACTGGTACACGTCACAAC	9	8	0	0	0	0
GSTA2	CATGGTACACGTCACATG	193	205	0	0	0	0
GSTA2	CATGCAACACACACTGCA	302	313	1	0	0	0
GSTA3	CATGGTTGGTACCATGCA	556	261	1	1	1	9

GSTA3	ACTGGTCACACATGTGAC	37	79	1	0	0	1
GSTA3	CATGGTCACACATGTGTG	631	995	3	0	0	0
GSTA3	CATGGTCACAACGTGTGT	461	452	1	0	0	0
GSTA3	ACTGGTCACAACGTGTCA	230	250	0	0	0	0
GSTA3	CATGCAACTGCAGTTGTG	130	133	0	0	0	0
GSTA4	ACTGGTACGTTGTGCATG	198	205	0	2	1	3
GSTA4	GTTGGTACGTTGTGCAGT	149	144	0	0	0	0
GSTA4	CATGGTACGTTGTGCACA	36	53	0	0	0	0
GSTA4	TGTGGTACGTTGTGCAAC	27	41	0	0	0	0
GSTA4	CATGGTACGTTGTGACGT	269	372	0	0	0	0
GSTA4	TGTGCAACACTGACTGTG	244	334	0	0	0	0
GSTA5	GTTGGTACCATGTGGTGT	442	500	1	7	2	18
GSTA5	TGTGGTACCATGTGGTAC	58	47	0	0	0	0
GSTA5	ACTGGTACCATGTGGTTG	259	326	1	1	0	6
GSTA5	CATGGTACCATGTGCAGT	328	457	0	2	0	0
GSTA5	CATGCAACTGCAGTACAC	29	26	0	0	0	0
GSTA5	GTTGCAACTGCAGTACTG	87	118	0	0	0	3
GSTK1	TGTGGTACTGTGTGTGAC	75	38	1	0	1	1
GSTK1	GTTGGTACTGTGTGTGGT	1176	1911	2	0	0	0
GSTK1	CATGGTACTGTGTGTGCA	156	235	4	1	0	0
GSTK1	ACTGGTACTGTGTGTGTG	11	20	0	0	0	0
GSTK1	CATGGTTGGTGTGTGTGT	627	1021	0	0	0	0
GSTK1	CATGCACAACTGACCAAC	46	129	0	1	0	0
GSTM1	ACTGGTGTGTGTTGGTAC	126	167	0	0	1	0
GSTM1	CATGGTGTGTGTACTGTG	571	351	1	0	0	0
GSTM1	TGTGGTGTGTGTTGGTGT	652	780	2	1	0	0
GSTM1	GTTGGTGTGTGTTGGTCA	237	225	0	0	0	0
GSTM1	CATGGTACTGGTACGTCA	151	259	110	0	0	0
GSTM2	ACTGGTACTGGTACGTTG	1043	1192	0	0	2	0
GSTM2	TGTGGTACTGGTACGTAC	47	55	0	0	0	0
GSTM2	CATGGTACTGGTACCAGT	98	156	0	0	0	0
GSTM2	ACTGGTTGGTCACACATG	474	285	0	0	0	0
GSTM2	GTTGGTTGGTACACGTGT	803	639	1	0	0	4
GSTM3	TGTGGTACGTCAGTGTGT	442	529	0	0	1	0
GSTM3	ACTGGTGTCACACAACAC	16	38	0	0	0	0
GSTM3	TGTGGTACGTGTTGTGAC	20	50	0	0	0	0
GSTM3	ACTGGTACGTGTTGTGTG	38	54	0	0	0	0
GSTM3	GTTGGTACGTCAGTGTCA	342	317	1	0	0	0
GSTM4	GTTGCAGTCACACAGTGT	1268	1405	1	1	1	0
GSTM4	GTTGGTTGGTCACACAGT	107	92	0	0	0	0
GSTM4	CATGGTTGGTCACACACA	54	57	0	0	0	0
GSTM4	TGTGGTTGGTCACACAAC	32	20	0	0	0	0
GSTM4	ACTGCAGTCACAGTTGTG	389	403	2	0	0	2
GSTM5	GTTGGTACGTGTACCACA	212	106	1	0	1	4
GSTM5	CATGGTACGTGTACGTAC	46	49	1	0	0	0
GSTM5	GTTGGTACGTGTACGTTG	274	313	2	0	0	0
GSTM5	TGTGGTACGTGTACCAGT	21	16	0	0	0	0
GSTM5	ACTGGTACGTGTACCAAC	14	10	0	0	0	0
GSTM5	GTTGCAACACTGACTGAC	194	56	0	0	0	0
GSTO1	ACTGGTACCACAGTGTTG	388	166	4	0	5	6
GSTO1	TGTGGTACCACAGTGTAC	21	115	0	0	0	0
GSTO1	CATGGTACCACAGTCAGT	75	76	0	1	0	0
GSTO1	ACTGGTACCACAGTCACA	121	56	0	0	0	0
GSTO1	CATGGTTGGTCAACCATG	42	44	0	0	0	0
GSTO1	TGTGCACACATGGTTGAC	22	24	0	0	0	0
GSTO2	TGTGGTTGGTGTGTACGT	95	83	1	0	0	1
GSTO2	GTTGGTTGGTGTGTACCA	146	418	1	1	0	0
GSTO2	ACTGGTTGGTGTGTACAC	24	36	0	0	0	0
GSTO2	CATGGTTGGTGTGTACTG	123	210	0	0	0	0
GSTO2	GTTGGTTGGTGTGTTGGT	284	235	0	0	0	0
GSTP1	CATGGTGTGTCACACAGT	257	165	1	0	1	0
GSTP1	TGTGGTGTGTCACAGTAC	59	37	0	0	0	0
GSTP1	ACTGGTGTGTCACAGTTG	613	851	4	1	0	1
GSTP1	ACTGGTGTGTCACACACA	167	236	0	0	0	1
GSTP1	GTTGGTGTGTCACACAAC	7	15	0	0	0	0
GSTP1	ACTGCACAACACGTACTG	1087	1033	2	0	0	4
GSTT1	TGTGGTGTGTCAACCAAC	51	44	0	0	0	0
GSTT1	ACTGGTGTGTCAACCATG	201	117	1	1	0	0
GSTT1	CATGGTGTGTCAACACGT	58	28	0	0	0	0
GSTT1	ACTGGTGTGTCAACACCA	82	60	0	0	0	1
GSTT1	GTTGGTGTGTCAACACAC	2	10	0	0	0	0
GSTT1	ACTGCACAGTACTGTGAC	28	14	0	0	0	0
GSTZ1	TGACCAACCAGTACCACA	210	278	0	49	0	1
GSTZ1	CAACCAACCAGTACCAAC	51	30	0	0	0	0
GSTZ1	GTACCAACCAGTACCATG	146	301	0	0	0	0

GSTZ1	TGACCAACCAGTACACGT	69	84	0	0	0	0
GSTZ1	GTACCAACCAGTACACCA	30	42	0	0	0	7
GSTZ1	TGTGCAACACACACTGAC	135	100	0	0	0	0
GTF2A1	GTACGTACTGGTCAGTGT	725	1276	1	0	0	6
GTF2A1	CAACGTTGCACATGCAAC	28	38	9	0	0	0
GTF2A1	GTACGTTGCACATGCATG	507	453	0	0	0	0
GTF2A1	TGACGTTGCACATGACGT	646	186	0	0	0	2
GTF2A1	GTACGTTGCACATGACCA	73	83	1	0	0	0
GTF2A1L	TGACGTACACCATGCAGT	183	400	0	1	1	1
GTF2A1L	CAACGTACACCATGGTAC	29	34	0	0	0	0
GTF2A1L	GTACGTACACCATGGTTG	109	105	0	0	0	0
GTF2A1L	GTACGTACACCATGCACA	161	157	1	0	0	0
GTF2A1L	ACACGTACACCATGCAAC	15	34	0	0	0	0
GTF2A1L	TGTGCAACTGTGCAGTCA	585	390	0	0	0	0
GTF2A2	GTACGTTGCACATGTGGT	900	406	3	0	0	1
GTF2A2	CAACGTTGCACATGTGCA	896	111	0	0	0	0
GTF2A2	TGACGTTGCACATGTGAC	48	23	0	0	0	0
GTF2A2	ACACGTTGCACATGTGTG	388	270	0	1	0	0
GTF2A2	ACACGTTGCAACGTTGCA	44	63	0	0	0	0
GTF2A2	ACTGCACACATGGTTGTG	383	442	0	0	0	0
GTF2B	CAACGTTGCAACCAGTGT	893	1271	1	1	0	10
GTF2B	ACACGTTGCAACCAGTCA	368	276	0	1	0	0
GTF2B	GTACGTTGCAACCAGTAC	23	33	0	0	0	0
GTF2B	TGACGTTGCAACCAGTTG	157	239	0	0	0	17
GTF2B	GTACTGTGACACACACAC	65	57	0	0	0	0
GTF2E1	GTTGCACAACGTGTCATG	127	143	0	0	1	19
GTF2E1	CAACGTTGCAACACGTCA	116	331	0	0	0	0
GTF2E1	TGACGTTGCAACACGTAC	26	43	0	0	0	0
GTF2E1	ACACGTTGCAACACGTTG	924	686	1	0	0	5
GTF2E1	CAACGTTGCAACACCAGT	320	301	2	0	0	0
GTF2E1	TGTGCAGTGTTGCACAGT	340	303	0	0	0	0
GTF2E2	CAACGTTGCAACTGGTTG	385	383	0	1	1	0
GTF2E2	GTACGTTGCAACTGGTCA	354	649	1	0	0	0
GTF2E2	ACACGTTGCAACTGGTAC	59	132	0	0	0	0
GTF2E2	GTACGTTGCAACTGCAGT	116	81	0	1	0	0
GTF2E2	CAACGTTGCAACTGCACA	12	18	0	0	0	0
GTF2F1	CAACGTTGCATGGTTGAC	3	12	0	0	0	0
GTF2F1	GTACGTTGCATGGTTGTG	165	108	0	0	0	0
GTF2F1	ACACGTTGCATGCAGTGT	672	487	0	0	0	0
GTF2F1	TGACGTTGCATGCAGTCA	333	347	0	0	0	0
GTF2F1	CAACGTTGCATGCAGTAC	52	134	0	0	0	0
GTF2F1	GTTGCACAGTTGGTGTGT	38	23	0	0	0	0
GTF2F2	TGACGTTGCATGCAACAC	17	32	0	0	0	0
GTF2F2	ACACGTTGCATGCAACTG	413	377	0	0	0	0
GTF2F2	CAACGTTGCATGCATGGT	434	508	0	1	0	2
GTF2F2	ACACGTTGCATGCATGCA	142	200	0	0	0	2
GTF2F2	GTACGTTGCATGCATGAC	83	143	0	0	0	0
GTF2F2	GTTGCACACATGCAGTGT	398	272	1	0	0	0
GTF2H1	GTCATGTGTGTGGTGTCA	278	267	1	0	0	0
GTF2H1	ACCATGTGTGTGGTGTAC	15	21	0	0	0	0
GTF2H1	CACATGTGTGTGGTGTTG	205	343	0	0	0	0
GTF2H1	GTCATGTGTGTGGTCAGT	17	22	0	0	0	0
GTF2H1	ACTGGTGTGTTGACGTGT	442	240	0	1	0	0
GTF2H1	ACTGCAACCACACAACTG	435	508	1	2	0	0
GTF2H2	ACACGTTGCATGACCAGT	101	139	0	0	0	0
GTF2H2	TGACGTTGCATGACCACA	360	100	0	0	0	9
GTF2H2	CAACGTTGCATGACCAAC	131	104	0	1	0	0
GTF2H2	GTACGTTGCATGACCATG	186	139	1	0	0	0
GTF2H2	TGACGTTGCATGACACGT	68	54	0	0	0	0
GTF2H3	GTACGTTGCATGTGCAAC	30	9	0	0	0	0
GTF2H3	TGACGTTGCATGTGCATG	214	244	0	0	0	0
GTF2H3	ACACGTTGCATGTGACGT	96	93	0	0	0	0
GTF2H3	TGACGTTGCATGTGACCA	122	242	2	0	0	0
GTF2H3	CAACGTTGCATGTGACAC	25	40	0	0	0	0
GTF2H3	ACTGCATGGTCAGTCATG	74	196	0	0	0	0
GTF2H4	CAACGTACCATGCAACTG	369	508	2	28	2	67
GTF2H4	GTACGTACCATGCAACCA	410	466	2	1	0	0
GTF2H4	ACACGTACCATGCAACAC	66	69	0	0	0	1
GTF2H4	GTACGTACCATGCATGGT	272	432	0	1	0	1
GTF2H4	CAACGTACCATGCATGCA	249	191	0	0	0	2
GTF2H5	GTACTGACGTCAACGTAC	181	22	0	1	0	1
GTF2H5	TGACTGACGTCAACGTTG	499	577	1	1	0	0
GTF2H5	ACACTGACGTCAACCAGT	607	142	0	0	0	1
GTF2H5	TGACTGACGTCAACCACA	121	166	0	0	0	0
GTF2H5	CAACTGACGTCAACCAAC	42	43	0	0	0	0

GTF2I	ACACGTACTGACTGGTAC	195	129	6	1	2	0
GTF2I	CAACGTACTGACTGGTTG	1411	1497	69	0	1	0
GTF2I	GTACGTACTGACTGCAGT	118	147	1	0	0	0
GTF2I	TGACGTACTGTGCATGTG	358	309	0	0	0	0
GTF2I	CAACGTACTGTGACGTGT	811	944	3	1	0	0
GTF2IRD1	TGACGTACACCAGTGTTG	539	532	0	0	1	4
GTF2IRD1	TGACGTCAACTGACGTTG	60	24	0	0	0	0
GTF2IRD1	ACACGTCAACTGACCAGT	131	131	0	0	0	0
GTF2IRD1	TGACGTCAACTGACCACA	446	211	0	0	0	0
GTF2IRD1	CAACGTCAACTGACCAAC	188	261	147	3	0	0
GTF2IRD1	TGTGCACAACTGACCACA	26	19	0	0	0	4
GTF3C3	GTACGTCAACTGCAGTTG	335	298	3	2	0	0
GTF3C3	TGACGTCAACTGCACAGT	90	86	0	0	0	0
GTF3C3	GTACGTCAACTGCACACA	144	97	0	1	0	1
GTF3C3	ACACGTCAACTGCACAAC	65	3	0	0	0	0
GTF3C3	CAACGTCAACTGCACATG	174	58	0	0	0	0
GUCA1A	TGACACTGCAACGTGTAC	60	184	0	0	1	0
GUCA1A	CAACACTGCAACGTGTCA	434	502	0	1	0	6
GUCA1A	ACACACTGCAACGTGTTG	98	104	1	0	0	0
GUCA1A	CAACACTGCAACGTCAGT	439	334	0	0	0	17
GUCA1A	ACACACTGCAACGTCACA	29	34	0	0	0	0
GUCA1B	GTTGACCAGTCAGTGTGT	1134	1441	1	1	1	0
GUCA1B	TGACACTGCAACCAGTGT	706	281	1	1	0	0
GUCA1B	GTACACTGCAACCAGTCA	856	776	1	1	0	0
GUCA1B	ACACACTGCAACCAGTAC	78	58	0	0	0	0
GUCA1B	CATGACCAGTCAGTGTCA	104	86	1	0	0	0
GUCA1C	ACACACTGCAACCAACCA	558	460	0	0	1	1
GUCA1C	ACACACTGCAACCACATG	106	256	0	0	0	0
GUCA1C	CAACACTGCAACCAACGT	594	352	6283	9	0	1
GUCA1C	GTACACTGCAACCAACAC	30	49	0	0	0	0
GUCA1C	TGACACTGCAACCAACTG	403	303	0	0	0	0
GUCY1A2	GTACTGTGCATGTGCATG	200	118	0	0	0	0
GUCY1A2	TGACTGTGCATGTGACGT	291	220	0	0	0	0
GUCY1A2	GTACTGTGCATGTGACCA	303	201	0	1	0	3
GUCY1A2	ACACTGTGCATGTGACAC	54	57	0	0	0	1
GUCY1A2	CAACTGTGCATGTGACTG	180	198	0	1	0	41
GUCY1A2	CATGCACAGTTGGTGTCA	292	330	1	0	0	0
GUCY1A3	GTACTGACGTACACTGTG	204	176	1	0	1	0
GUCY1A3	TGACTGACGTACACTGCA	125	229	0	0	0	0
GUCY1A3	CAACTGACGTACACTGAC	35	45	0	0	0	0
GUCY1A3	ACACTGACGTACTGGTGT	390	638	4	0	0	0
GUCY1A3	TGACTGACGTACTGGTCA	261	668	1	0	0	0
GUCY1B3	CAACTGACGTACTGCATG	126	125	1	1	0	0
GUCY1B3	GTACTGACGTACTGACGT	184	176	0	1	0	0
GUCY1B3	CAACTGACGTACTGACCA	123	29	2	0	0	0
GUCY1B3	TGACTGACGTACTGACAC	71	74	0	0	0	0
GUCY1B3	ACACTGACGTACTGACTG	360	185	0	0	0	0
GUCY1B3	TGTGCACAGTTGGTGTAC	84	223	0	0	0	0
GUSB	ACACCAGTACACGTTGAC	43	44	0	0	0	0
GUSB	CAACCAGTACACGTTGTG	676	252	0	1	0	0
GUSB	TGACCAGTACACCAGTGT	568	754	1	3	0	4
GUSB	GTACCAGTACACCAGTCA	325	349	2	0	0	1
GUSB	ACACCAGTACACCAGTAC	121	70	0	0	0	0
GYG1	GTTGGTGTACACACGTGT	309	368	0	0	0	0
GYG1	CATGGTGTACACACGTCA	243	219	0	0	0	0
GYG1	TGTGGTGTACACACGTAC	160	20	0	0	0	0
GYG1	ACTGGTGTACACACGTTG	83	136	0	0	0	0
GYG1	CATGGTGTACACACCAGT	212	165	0	1	0	0
GYG1	ACTGCAACCAACGTTGTG	333	621	1	0	0	0
GYPA	TGTGGTGTACTGACACGT	59	49	0	0	0	0
GYPA	GTTGGTGTACTGACACCA	200	212	0	0	0	3
GYPA	ACTGGTGTACTGACACAC	64	81	0	0	0	1
GYPA	CATGGTGTACTGACACTG	147	144	1	0	0	0
GYPA	GTTGACCACATGTGACCA	227	329	0	0	0	1
GYS1	TGACACGTACGTGTGTCA	286	317	4	1	1	0
GYS1	CATGGTTGTGCAGTCATG	244	133	0	0	1	0
GYS1	CAACACGTACGTGTGTAC	22	14	0	0	0	0
GYS1	GTACACGTACGTGTGTTG	101	57	0	0	0	0
GYS1	TGACACGTACGTGTCAGT	35	35	0	0	0	0
GYS2	GTACACGTACGTGTTGAC	52	43	0	0	0	0
GYS2	TGACACGTACGTGTTGTG	299	615	0	0	0	0
GYS2	CAACACGTACGTCAGTGT	616	582	9	0	0	3
GYS2	ACACACGTACGTCAGTCA	105	171	1	0	0	3
GYS2	GTACACGTACGTCAGTAC	51	129	0	0	0	0
GZMA	ACACGTGTGTACGTACTG	312	537	0	0	0	1

GZMA	CAACGTGTGTACGTTGGT	123	131	1	0	0	0
GZMA	ACACGTGTGTACGTTGCA	289	160	0	0	0	0
GZMA	GTACGTGTGTACGTTGAC	53	50	0	0	0	10
GZMA	TGACGTGTGTACGTTGTG	190	385	0	0	0	0
GZMA	ACTGCACAACACACACGT	49	51	0	0	0	0
GZMB	ACACGTGTGTACGTCAAC	20	32	0	0	0	0
GZMB	CAACGTGTGTACGTCATG	220	294	0	1	0	0
GZMB	GTACGTGTGTACGTACGT	81	115	0	0	0	0
GZMB	CAACGTGTGTACGTACCA	154	121	0	0	0	0
GZMB	TGACGTGTGTACGTACAC	27	15	0	0	0	0
H19	TGTGACGTTGACGTCAAC	11	29	0	0	0	0
H19	ACTGACGTTGACGTCATG	60	20	0	0	0	0
H19	CATGACGTTGACGTACGT	184	126	0	0	0	2
H19	ACTGACGTTGACGTACCA	176	205	0	1	0	1
H19	GTTGACGTTGACGTACAC	20	21	0	0	0	0
H2AFX	TGACTGTGGTGTGTCACA	201	60	0	0	0	0
H2AFX	CAACTGTGGTGTGTCAAC	30	15	0	0	0	0
H2AFX	GTACTGTGGTGTGTCATG	421	365	0	0	0	1
H2AFX	CATGGTGTCAGTACTGCA	95	87	0	0	0	0
H2AFX	TGTGGTGTCAGTACTGAC	38	58	0	0	0	0
H2AFY	ACTGGTGTCAGTACTGTG	189	221	2	1	0	0
H2AFY	GTTGGTGTCACAACTGCA	41	22	1	0	0	0
H2AFY	ACTGGTGTCACAACTGAC	43	51	0	0	0	0
H2AFY	CATGGTGTCACAACTGTG	451	884	4	0	0	0
H2AFY	TGTGGTGTCACATGGTGT	536	672	0	1	0	0
H2AFY	TGTGCAACCAACTGACAC	40	50	0	0	0	0
H2AFY2	GTTGGTGTCAGTACTGGT	395	458	0	0	0	0
H2AFY2	TGTGGTGTCACATGACTG	261	275	0	0	0	0
H2AFY2	ACTGGTGTCACATGTGGT	106	122	0	1	0	0
H2AFY2	TGTGGTGTCACATGTGCA	235	252	1	0	0	0
H2AFY2	CATGGTGTCACATGTGAC	89	149	0	0	0	0
H6PD	TGACGTTGTGTGGTTGGT	84	181	2	0	0	0
H6PD	GTACGTTGTGTGGTTGCA	232	216	0	0	0	1
H6PD	ACACGTTGTGTGGTTGAC	25	34	0	0	0	0
H6PD	CAACGTTGTGTGGTTGTG	218	255	0	0	0	0
H6PD	GTACCAGTGTGTGTACTG	192	277	0	0	0	0
HADH	CAACCAGTGTGTACACCA	276	247	0	1	0	0
HADH	TGACCAGTGTGTACACAC	17	20	0	0	0	0
HADH	ACACCAGTGTGTACACTG	61	35	0	0	0	0
HADH	CAACCAGTGTGTACTGGT	115	75	0	0	0	0
HADH	ACACCAGTGTGTACTGCA	93	106	0	0	0	1
HADHA	GTACCAACACCAGTCAAC	87	99	1	0	1	0
HADHA	CAACCAACACCAGTCAGT	249	206	0	0	0	0
HADHA	ACACCAACACCAGTCACA	86	76	4	0	0	1
HADHA	TGACCAACACCAGTCATG	284	218	0	376	0	0
HADHA	ACACCAACACCAGTACGT	111	94	0	1	0	0
HADHB	TGACCAGTGTGTTGCACA	135	163	0	0	1	0
HADHB	ACACCAGTGTGTTGCAGT	126	162	0	0	0	0
HADHB	CAACCAGTGTGTTGCAAC	4	5	0	0	0	0
HADHB	GTACCAGTGTGTTGCATG	157	188	0	0	0	0
HADHB	TGACCAGTGTGTTGACGT	88	119	0	0	0	0
HAGH	TGACACACGTACTGCAAC	7	15	0	0	0	0
HAGH	ACACACACGTACTGCATG	275	295	1	0	0	0
HAGH	CAACACACGTACTGACGT	383	472	0	0	0	0
HAGH	ACACACACGTACTGACCA	110	129	0	0	0	0
HAGH	GTACACACGTACTGACAC	178	39	0	0	0	0
HAL	TGACTGTGCATGCAACTG	71	77	0	0	0	0
HAL	ACACTGTGCATGCATGGT	375	505	4	0	0	0
HAL	TGACTGTGCATGCATGCA	325	342	0	0	0	5
HAL	CAACTGTGCATGCATGAC	31	41	0	0	0	0
HAL	GTTGCAGTTGTGTGCACA	46	57	0	0	0	0
HAL	GTTGCACACATGACCAGT	69	43	0	0	0	0
HAND1	GTACGTCAACTGCATGAC	90	119	0	0	1	0
HAND1	CAACGTCAACTGACGTGT	736	528	1	2	1	0
HAND1	CAACGTCAACTGCATGGT	907	713	2	0	0	0
HAND1	ACACGTCAACTGCATGCA	539	315	0	1	0	2
HAND1	TGACGTCAACTGCATGTG	950	474	0	0	0	0
HAND1	CATGCACACATGCAGTCA	587	550	1	0	0	12
HAO1	CAACACGTTGGTCATGTG	882	320	6	1	3	3
HAO1	GTACACGTTGGTCATGCA	387	143	1	0	0	0
HAO1	ACACACGTTGGTCATGAC	146	111	0	0	0	0
HAO1	CATGCAGTTGGTACGTGT	291	555	1	0	0	0
HAO1	GTTGCACATGACGTCATG	32	64	0	0	0	0
HAO1	TGTGCACATGACGTACGT	143	138	0	0	0	1
HAO2	TGTGCAGTTGGTCATGTG	956	223	1	0	1	0

HAO2	TGACACGTTGGTCAACCA	161	160	1	0	0	9
HAO2	CAACACGTTGGTCAACAC	98	106	0	0	0	0
HAO2	GTACACGTTGGTCAACTG	327	410	9	0	0	0
HAO2	TGACACGTTGGTCATGGT	338	273	40	0	0	0
HAP1	TGTGGTTGCAACACTGAC	16	19	0	0	0	0
HAP1	ACTGGTTGCAACACTGTG	645	296	0	0	0	0
HAP1	GTTGGTTGCAACTGGTGT	516	297	0	1	0	6
HAP1	CATGGTTGCAACTGGTCA	329	441	0	1	0	1
HAP1	GTTGACACCATGCAGTGT	679	335	0	1	0	0
HARS	GTACACGTACGTCATGGT	265	228	0	0	0	1
HARS	CAACACGTACGTCATGCA	147	139	0	0	0	0
HARS	TGACACGTACGTCATGAC	34	35	0	0	0	0
HARS	ACACACGTACGTCATGTG	472	332	0	0	0	0
HARS	GTACACGTACGTACGTGT	101	119	0	0	0	0
HARS	TGTGCAACGTTGTGCAGT	137	125	0	0	0	0
HARS2	CAACACGTACGTCAACTG	667	239	0	0	1	0
HARS2	GTACACGTACGTCACATG	94	112	0	0	0	0
HARS2	TGACACGTACGTCAACGT	240	126	0	0	0	0
HARS2	GTACACGTACGTCAACCA	111	166	0	0	0	1
HARS2	ACACACGTACGTCAACAC	26	41	0	0	0	0
HARS2	GTTGCAACGTTGTGGTTG	152	198	0	0	0	0
HAT1	GTACCACACATGCAGTCA	451	168	0	0	0	0
HAT1	ACACCACACATGCAGTAC	23	25	0	0	0	0
HAT1	CAACCACACATGCAGTTG	86	112	0	0	0	0
HAT1	GTACCACACATGCACAGT	77	80	0	0	0	0
HAT1	CAACCACACATGCACACA	153	95	0	2	0	0
HBA2	CAACCAGTACGTACCAAC	45	41	0	0	0	0
HBA2	GTACCAGTACGTACCATG	171	257	0	0	0	0
HBA2	TGACCAGTACGTACACGT	175	56	0	0	0	0
HBA2	GTACCAGTACGTACACCA	103	277	0	0	0	0
HBA2	ACACCAGTACGTACACAC	21	24	0	0	0	0
HBA2	GTTGCAACGTGTCATGAC	48	33	0	0	0	0
HBB	GTACCAGTACGTACGTAC	57	54	0	0	1	0
HBB	TGACCAGTACGTACGTTG	77	104	0	0	1	0
HBB	TGTGCATGGTCAGTGTGT	1187	896	1	0	1	0
HBB	ACACCAGTACGTACGTCA	124	139	0	0	0	0
HBB	ACACCAGTACGTACCAGT	382	244	0	0	0	2
HBB	TGACCAGTACGTACCACA	42	51	0	0	0	0
HBEGF	ACACTGCATGTGCAGTGT	291	458	1	0	0	0
HBEGF	TGACTGCATGTGCAGTCA	44	56	0	0	0	0
HBEGF	CAACTGCATGTGCAGTAC	52	57	0	0	0	0
HBEGF	GTACTGCATGTGCAGTTG	376	100	0	0	0	0
HBEGF	CATGCACAGTACCATGGT	565	525	191	0	0	1
HBEGF	ACTGCACAGTACCATGCA	222	213	0	0	0	2
HBQ1	ACACTGCACAACGTTGTG	201	257	1	0	0	0
HBQ1	GTACTGCACAACCAGTGT	1215	1021	0	0	0	0
HBQ1	CAACTGCACAACCAGTCA	495	445	0	0	0	0
HBQ1	CATGCAACGTGTTGCAGT	74	49	0	0	0	9
HBQ1	ACTGCAACGTGTTGCACA	16	94	0	0	0	0
HBXIP	GTTGGTACCAGTACGTCA	238	393	0	0	0	1
HBXIP	ACTGGTACCAGTACGTAC	109	118	0	0	0	0
HBXIP	CATGGTACCAGTACGTTG	206	769	2	0	0	3
HBXIP	GTTGGTACCAGTACCAGT	239	166	0	0	0	0
HBXIP	CATGGTACCAGTACCACA	58	51	0	0	0	0
HCCS	ACACACGTCATGACTGAC	147	90	0	0	0	1
HCCS	CAACACGTCATGACTGTG	289	279	0	0	0	0
HCCS	TGACACGTCATGTGGTGT	320	220	1	0	0	0
HCCS	GTACACGTCATGTGGTCA	145	164	0	0	0	0
HCCS	ACACACGTCATGTGGTAC	47	71	0	0	0	0
HCK	ACTGCACAGTGTTGTGGT	549	354	1	0	1	0
HCK	CATGCACAGTCAGTGTGT	452	435	0	0	1	0
HCK	TGTGCACAGTGTTGTGCA	192	170	0	0	0	0
HCK	CATGCACAGTGTTGTGAC	49	58	0	0	0	0
HCK	GTTGCACAGTGTTGTGTG	181	188	0	0	0	0
HCK	GTTGCATGGTTGGTACGT	257	290	0	1	0	2
HCLS1	GTACGTACGTCACAACCA	334	425	1	0	1	0
HCLS1	CAACGTACGTCACACAAC	98	33	0	0	0	0
HCLS1	GTACGTACGTCACACATG	58	112	0	0	0	0
HCLS1	TGACGTACGTCACAACGT	160	139	0	0	0	0
HCLS1	ACACGTACGTCACAACAC	41	12	0	0	0	0
HCST	ACTGCAGTGTGTGTGTCA	301	580	0	1	0	0
HCST	GTTGCAGTGTGTGTGTAC	232	198	0	0	0	1
HCST	TGTGCAGTGTGTGTGTTG	78	61	0	0	0	0
HCST	ACTGCAGTGTGTGTCAGT	107	103	0	0	0	0
HCST	CATGACGTTGGTACGTCA	82	73	0	1	0	1

HDAC1	GTCATGTGCACATGACTG	140	164	0	1	0	0
HDAC1	TGCATGTGCACATGTGGT	278	283	0	1	0	0
HDAC1	GTCATGTGCACATGTGCA	81	91	0	0	0	0
HDAC1	ACCATGTGCACATGTGAC	23	43	0	0	0	0
HDAC1	CACATGTGCACATGTGTG	245	219	0	0	0	0
HDAC1	TGTGCACATGTGGTCAGT	450	279	0	0	0	0
HDAC10	ACCATGTGCAACACGTAC	89	82	0	0	0	0
HDAC10	CACATGTGCAACACGTTG	729	781	0	2	0	0
HDAC10	GTCATGTGCAACACCAGT	477	257	0	0	0	0
HDAC10	CACATGTGCAACACCACA	113	48	0	0	0	0
HDAC10	TGCATGTGCAACACCAAC	28	15	0	0	0	0
HDAC11	CAACGTACACACACGTGT	372	475	6	1	0	0
HDAC11	ACACGTACACACACGTCA	152	185	0	1	0	3
HDAC11	GTACGTACACACACGTAC	36	52	3	0	0	0
HDAC11	TGACGTACACACACGTTG	732	992	118	0	0	33
HDAC11	ACACGTACACACACCAGT	133	359	0	0	0	0
HDAC11	ACTGCACATGTGCATGTG	166	336	1	0	0	6
HDAC2	TGCATGTGCAACGTGTTG	271	230	0	0	1	0
HDAC2	GTACCATGGTACTGCATG	186	269	0	0	1	6
HDAC2	CACATGTGCAACGTGTGT	193	186	0	1	0	0
HDAC2	ACCATGTGCAACGTGTCA	520	321	0	12	0	0
HDAC2	GTCATGTGCAACGTGTAC	263	76	0	0	0	0
HDAC3	ACCATGTGCAACGTCAGT	233	212	0	0	0	0
HDAC3	TGCATGTGCAACGTCACA	150	101	0	0	0	0
HDAC3	CACATGTGCAACGTCAAC	31	32	0	0	0	0
HDAC3	GTCATGTGCAACGTCATG	130	203	0	1	0	0
HDAC3	TGCATGTGCAACGTACGT	261	181	0	0	0	0
HDAC3	GTTGCACATGTGGTCACA	103	54	0	0	0	0
HDAC4	GTCATGTGCAACGTACCA	439	389	0	0	0	0
HDAC4	ACCATGTGCAACGTACAC	16	18	0	0	0	0
HDAC4	CACATGTGCAACGTACTG	127	176	1	0	0	1
HDAC4	GTCATGTGCAACGTTGGT	221	309	0	0	0	0
HDAC4	CACATGTGCAACGTTGCA	192	161	0	0	0	0
HDAC5	TGCATGTGCAACGTTGAC	17	11	0	0	0	0
HDAC5	ACCATGTGCAACGTTGTG	349	531	0	0	0	0
HDAC5	GTCATGTGCAACCAGTGT	1592	1017	1	0	0	5
HDAC5	CACATGTGCAACCAGTCA	626	593	1	0	0	0
HDAC5	TGCATGTGCAACCAGTAC	62	33	0	0	0	0
HDAC6	ACCATGTGCAACCACACA	338	206	0	2	1	0
HDAC6	ACCATGTGCAACCAGTTG	1948	796	3	1	0	0
HDAC6	CACATGTGCAACCACAGT	148	100	0	0	0	0
HDAC6	GTCATGTGCAACCACAAC	23	32	0	0	0	0
HDAC6	TGCATGTGCAACCACATG	317	124	1	0	0	1
HDAC6	ACTGCACATGTGGTCAAC	73	56	0	0	0	0
HDAC8	ACCATGTGCAACCAACGT	374	561	15	11309	788	4
HDAC8	TGCATGTGCAACCAACCA	463	479	0	0	0	0
HDAC8	CACATGTGCAACCAACAC	49	82	0	0	0	0
HDAC8	GTCATGTGCAACCAACTG	253	528	1	0	0	0
HDAC8	TGCATGTGCAACCATGGT	663	464	0	2	0	0
HDAC8	GTTGCACATGGTACCACA	70	112	0	0	0	0
HDAC9	GTCATGTGCAACACGTCA	531	582	1	3	2	7388
HDAC9	GTCATGTGCAACCATGCA	570	299	2	0	1	0
HDAC9	GTTGCACAACTGCAGTTG	1329	1092	38	2	1	4
HDAC9	ACCATGTGCAACCATGAC	14	11	0	0	0	0
HDAC9	CACATGTGCAACCATGTG	234	199	1	1	0	0
HDAC9	TGCATGTGCAACACGTGT	540	378	0	0	0	1
HDC	TGACTGTGACGTTGACGT	405	244	0	0	0	0
HDC	GTACTGTGACGTTGACCA	36	55	0	0	0	0
HDC	ACACTGTGACGTTGACAC	13	18	0	0	0	0
HDC	ACTGGTGTCACAGTCAAC	3	11	0	0	0	0
HDC	ACTGCAGTGTGTACACCA	262	330	0	1	0	0
HDGF	CATGGTGTGTTGTGCAAC	20	38	0	0	0	0
HDGF	CATGGTCAGTGTACACTG	16	69	0	0	0	0
HDGF	GTTGGTCAGTGTACTGGT	71	55	0	0	0	0
HDGF	CATGGTCAGTGTACTGCA	178	106	0	0	0	1
HDGF	TGTGGTCAGTGTACTGAC	39	52	0	0	0	0
HDGF	TGTGCACACATGCAGTAC	343	302	2	0	0	0
HEL308	ACACACACCATGTGACAC	50	58	0	0	0	1
HEL308	CAACACACCATGTGACTG	291	289	1	0	0	1
HEL308	GTACACACCATGTGTGGT	862	519	0	1	0	0
HEL308	CAACACACCATGTGTGCA	587	474	297	103	0	1
HEL308	TGACACACCATGTGTGAC	10	20	0	0	0	0
HELB	CATGGTACGTACACCACA	184	183	5	1	13	1
HELB	GTTGGTACGTACACCAGT	26	79	1	0	0	0
HELB	TGTGGTACGTACACCAAC	12	8	0	0	0	0

HELB	ACTGGTACGTACACCATG	201	259	1	1	0	0
HELB	CATGGTACGTACACACGT	176	267	0	0	0	0
HELB	CATGCATGCACAACACCA	353	505	0	0	0	0
HELLS	ACCATGCACAACGTGTAC	22	35	0	0	0	0
HELLS	CACATGCACAACGTGTTG	393	501	0	0	0	0
HELLS	GTCATGCACAACGTCAGT	554	453	1	0	0	2
HELLS	CACATGCACAACGTCACA	124	104	0	1	0	0
HELLS	TGCATGCACAACGTCAAC	18	15	0	0	0	0
HELLS	ACTGCATGGTGTTGCAGT	35	78	0	0	0	0
HEMK1	CAACCACACAACGTACGT	147	219	1	0	0	0
HEMK1	ACACCACACAACGTACCA	87	94	0	0	0	0
HEMK1	GTACCACACAACGTACAC	41	26	0	0	0	0
HEMK1	TGACCACACAACGTACTG	1138	1489	3	2	0	4
HEMK1	ACACCACACAACGTTGGT	193	185	0	0	0	0
HERPUD1	CACATGTGCAGTACACCA	335	337	0	1	0	1
HERPUD1	TGCATGTGCAGTACACAC	51	112	0	0	0	0
HERPUD1	ACCATGTGCAGTACACTG	218	174	0	0	0	0
HERPUD1	CATGCAGTACGTGTACAC	105	93	1	0	0	0
HERPUD1	GTTGCAGTACGTGTACTG	306	282	1	0	0	0
HES1	CAACGTACTGCACAACGT	646	645	1	0	1	1
HES1	GTACGTACTGCACACAGT	110	297	0	0	0	30
HES1	CAACGTACTGCACACACA	151	131	0	0	0	0
HES1	TGACGTACTGCACACAAC	12	10	0	0	0	0
HES1	ACACGTACTGCACACATG	184	224	0	0	0	12
HES1	ACTGCACATGGTGTTGAC	7	8	0	0	0	0
HES2	CAACGTACCATGGTTGGT	129	77	0	0	0	0
HES2	ACACGTACCATGGTTGCA	320	411	0	0	0	0
HES2	GTACGTACCATGGTTGAC	348	87	1	0	0	1
HES2	TGACGTACCATGGTTGTG	178	212	0	0	0	0
HES2	CAACGTACCATGCAGTGT	859	830	1	0	0	1
HES3	TGACGTTGCAACGTACAC	8	29	0	0	0	0
HES3	ACACGTTGCAACGTACTG	176	131	0	0	0	0
HES3	CAACGTTGCAACGTTGGT	322	213	0	0	0	0
HES3	GTACGTTGCAACGTTGAC	33	44	0	0	0	0
HES3	TGACGTTGCAACGTTGTG	81	86	0	0	0	0
HES6	ACACGTACACACACACAC	33	20	0	0	0	0
HES6	CAACGTACACACACACTG	181	267	0	1	0	0
HES6	GTACGTACACACACTGGT	242	122	0	0	0	0
HES6	CAACGTACACACACTGCA	126	131	0	0	0	0
HES6	TGACGTACACACACTGAC	23	27	0	0	0	0
HES7	TGTGGTGTACCAACCAGT	385	379	0	0	0	1
HES7	ACTGGTTGTGCAACGTTG	49	102	1	0	0	0
HES7	CATGGTTGTGCAACCAGT	220	159	0	0	0	0
HES7	ACTGGTTGTGCAACCACA	98	104	0	0	0	0
HES7	GTTGGTTGTGCAACCAAC	13	3	0	0	0	0
HES7	GTTGCAACGTGTTGGTGT	521	566	1	0	0	0
HESX1	TGACGTCAACACTGTGCA	146	415	0	0	0	1
HESX1	CAACGTCAACACTGTGAC	24	64	0	0	0	0
HESX1	GTACGTCAACACTGTGTG	59	106	2	0	0	0
HESX1	TGACGTCAACTGGTGTGT	352	348	1	0	0	0
HESX1	TGACTGACTGACGTGTGT	281	291	0	0	0	1
HESX1	ACTGCACAGTTGGTGTTG	217	130	0	0	0	0
HEXA	TGACCAGTACACGTACCA	292	280	2	0	0	1
HEXA	CAACCAGTACACGTACAC	185	92	0	0	0	0
HEXA	GTACCAGTACACGTACTG	175	181	0	0	0	0
HEXA	TGACCAGTACACGTTGGT	166	271	0	0	0	0
HEXA	GTACCAGTACACGTTGCA	10	16	0	0	0	0
HEXB	CAACCAGTACACGTCAGT	196	221	0	0	0	0
HEXB	ACACCAGTACACGTCACA	227	101	0	1	0	0
HEXB	GTACCAGTACACGTCAAC	36	34	0	0	0	0
HEXB	TGACCAGTACACGTCATG	309	368	0	0	0	0
HEXB	ACACCAGTACACGTACGT	667	436	0	0	0	0
HEXB	CATGCACATGTGGTCATG	211	188	0	1	0	0
HEY1	GTACGTTGGTTGTGGTCA	1195	735	0	0	1	2
HEY1	CAACGTTGGTTGACTGTG	64	32	0	0	0	0
HEY1	TGACGTTGGTTGTGGTGT	378	477	1	0	0	0
HEY1	ACACGTTGGTTGTGGTAC	35	27	0	0	0	0
HEY1	CAACGTTGGTTGTGGTTG	1553	1198	2	0	0	4
HFE	TGACTGCACAACCAGTAC	316	306	1	1	0	0
HFE	ACACTGCACAACCAGTTG	697	860	1	2	0	0
HFE	CAACTGCACAACCACAGT	156	305	0	0	0	0
HFE	ACACTGCACAACCACACA	110	123	0	0	0	0
HFE	GTACTGCACAACCACAAC	3	10	0	0	0	0
HGD	TGCATGCACAGTTGACTG	852	704	0	0	0	0
HGD	ACCATGCACAGTTGTGGT	293	314	1	0	0	0

HGD	TGCATGCACAGTTGTGCA	962	341	0	2	0	0
HGD	CACATGCACAGTTGTGAC	22	27	0	0	0	0
HGD	GTCATGCACAGTTGTGTG	158	147	0	0	0	0
HGD	CATGCACAGTTGGTCAGT	60	59	0	0	0	0
HGF	CACATGACACTGGTGTTG	945	1362	8	4	10	26
HGF	CACATGACACTGGTCACA	170	69	2	2	2	5
HGF	ACCATGACACTGGTGTAC	54	66	3	0	0	0
HGF	GTCATGACACTGGTCAGT	228	77	0	0	0	3
HGF	TGCATGACACTGGTCAAC	4	5	0	0	0	0
HGFAC	TGACACACCAGTGTTGTG	679	970	1	2	1	1
HGFAC	CAACACACCAGTCAGTGT	488	450	1	0	0	0
HGFAC	ACACACACCAGTCAGTCA	401	364	0	0	0	0
HGFAC	GTACACACCAGTCAGTAC	50	35	0	1	0	0
HGFAC	GTTGCAGTTGCAGTCACA	101	105	0	0	0	0
HGFAC	ACTGCATGCACAACACTG	368	357	0	0	0	0
HGS	CAACCAACTGCACAGTAC	167	59	0	0	0	0
HGS	GTACCAACTGCACAGTTG	489	497	0	0	0	0
HGS	TGACCAACTGCACACAGT	156	190	2	0	0	0
HGS	GTACCAACTGCACACACA	67	226	0	0	0	0
HGS	ACACCAACTGCACACAAC	30	48	1	0	0	0
HGS	ACTGCAACTGCATGGTCA	148	100	0	0	0	2
HGSNAT	TGTGCAGTGTCACAGTGT	409	232	5	0	1	1384
HGSNAT	ACTGCAGTGTCAGTTGAC	25	28	0	0	0	0
HGSNAT	CATGCAGTGTCAGTTGTG	181	198	1	0	0	0
HGSNAT	GTTGCAGTGTCACAGTCA	552	199	0	0	0	0
HGSNAT	TGTGACACTGGTCAGTGT	136	309	0	0	0	0
HHEX	CAACGTACGTCAACACAC	26	19	0	0	0	0
HHEX	GTACGTACGTCAACACTG	200	199	4	2	0	4
HHEX	TGACGTACGTCAACTGGT	733	188	0	0	0	0
HHEX	GTACGTACGTCAACTGCA	16	38	0	0	0	0
HHEX	ACTGCAGTCATGCAGTAC	23	23	0	0	0	0
HHIP	GTTGGTCAACCAACACAC	30	17	0	0	1	0
HHIP	ACTGGTGTCAGTTGACGT	60	31	0	0	0	0
HHIP	CATGGTCAACCAACACGT	49	110	0	0	0	1
HHIP	ACTGGTCAACCAACACCA	151	284	0	0	0	0
HHIP	TGTGGTCAACCAACACTG	881	792	1	0	0	0
HHIP	TGTGCAACCAACCAACCA	378	262	1	0	0	0
HIBCH	CAACTGTGACACGTACTG	137	168	0	0	0	1
HIBCH	GTACTGTGACACGTTGGT	231	298	0	0	0	0
HIBCH	CAACTGTGACACGTTGCA	391	151	0	0	0	0
HIBCH	TGACTGTGACACGTTGAC	48	37	0	0	0	0
HIBCH	ACACTGTGACACGTTGTG	394	393	0	0	0	0
HIC1	CAACGTACGTCATGGTTG	577	559	1	0	0	0
HIC1	GTACGTACGTCATGCAGT	84	162	0	0	0	2
HIC1	CAACGTACGTCATGCACA	71	68	1	0	0	0
HIC1	TGACGTACGTCATGCAAC	12	11	0	0	0	0
HIC1	ACACGTACGTCATGCATG	87	85	0	0	0	0
HIF1A	ACCATGTGGTGTCATGCA	187	130	0	0	0	0
HIF1A	GTCATGTGGTGTCATGAC	17	15	0	0	0	0
HIF1A	TGCATGTGGTGTCATGTG	211	392	3	0	0	0
HIF1A	CACATGTGGTGTACGTGT	538	399	0	0	0	0
HIF1A	TGACACTGGTTGCATGCA	237	353	0	0	0	1
HIF1A	CATGCATGGTTGCAGTGT	406	463	0	0	0	0
HIG2	ACTGGTTGCAACTGGTTG	555	559	0	2	1	0
HIG2	CATGGTTGCAACTGCAGT	154	205	1	0	1	3
HIG2	TGTGGTTGCAACTGGTAC	57	43	1	0	0	0
HIG2	ACTGGTTGCAACTGCACA	128	173	0	0	0	0
HIG2	GTTGGTTGCAACTGCAAC	32	21	0	0	0	0
HIP1	CAACCACAGTACGTTGGT	182	190	1	0	0	0
HIP1	ACACCACAGTACGTTGCA	117	154	1	0	0	0
HIP1	GTACCACAGTACGTTGAC	85	42	0	0	0	0
HIP1	TGACCACAGTACGTTGTG	676	717	2	2	0	1
HIP1	TGTGCAGTCAACACGTTG	880	795	0	0	0	0
HIP1R	CATGGTGTTGACGTCAAC	236	112	3	0	1	3
HIP1R	TGTGGTGTTGACGTGTTG	608	551	0	1	0	2
HIP1R	ACTGGTGTTGACGTCAGT	135	107	0	0	0	0
HIP1R	TGTGGTGTTGACGTCACA	75	139	0	0	0	0
HIP1R	TGTGCACAGTGTACCATG	80	380	0	0	0	0
HIP2	TGACGTGTACCACAGTCA	509	614	17	2	6	26
HIP2	TGTGCAACACCACAACCA	153	73	1	0	1	0
HIP2	CAACGTGTACCACAGTAC	19	38	0	0	0	0
HIP2	GTACGTGTACCACAGTTG	195	213	0	1	0	0
HIP2	TGACGTGTACCACACAGT	75	86	0	0	0	0
HIP2	GTACGTGTACCACACACA	116	83	0	0	0	0
HIPK2	ACCATGACACACACCACA	262	175	1	0	1	0

HIPK2	TGCATGACACACACGTAC	20	18	0	0	0	0
HIPK2	ACCATGACACACACGTTG	326	600	0	0	0	0
HIPK2	CACATGACACACACCAGT	397	381	0	0	0	6
HIPK2	ACTGCAGTACGTTGGTCA	549	419	0	0	0	0
HIST1H2AA	CATGGTGTCACATGGTTG	462	456	1	6	2	1
HIST1H2AA	GTTGGTGTCACATGGTCA	214	505	0	0	0	0
HIST1H2AA	ACTGGTGTCACATGGTAC	55	57	0	0	0	1
HIST1H2AA	GTTGGTGTCACATGCAGT	338	273	0	0	0	1
HIST1H2AA	CATGGTGTCACATGCACA	43	67	0	0	0	0
HIST2H3C	TGTGGTGTCACATGCAAC	51	39	0	0	0	0
HIST2H3C	ACTGGTGTCACATGCATG	370	445	30	0	0	7
HIST2H3C	CATGGTGTCACATGACGT	79	127	0	0	0	0
HIST2H3C	ACTGGTGTCACATGACCA	106	98	0	0	0	1
HIST2H3C	GTTGGTGTCACATGACAC	142	39	0	0	0	0
HIST2H3C	CATGCATGCAGTTGACCA	176	169	1	0	0	3
HIST3H3	TGACTGTGGTACTGGTGT	434	348	1	0	0	0
HIST3H3	GTACTGTGGTACTGGTCA	135	207	0	0	0	0
HIST3H3	ACACTGTGGTACTGGTAC	21	18	0	0	0	0
HIST3H3	CAACTGTGGTACTGGTTG	1233	924	1	1	0	0
HIST3H3	CATGGTGTCAGTGTTGAC	5	16	0	0	0	0
HIST3H3	GTTGCAACCAGTTGTGGT	88	180	0	1	0	0
HK1	ACACCAACACTGCATGCA	376	197	0	0	1	0
HK1	ACACCAACACTGCAACTG	732	305	0	0	0	0
HK1	CAACCAACACTGCATGGT	179	238	0	0	0	0
HK1	GTACCAACACTGCATGAC	250	36	1	0	0	0
HK1	TGACCAACACTGCATGTG	447	428	1	0	0	0
HK1	CATGCAACGTACGTACAC	32	36	0	0	0	0
HK2	TGACCAACACTGACCACA	125	129	0	0	0	0
HK2	CAACCAACACTGACCAAC	7	11	0	0	0	7
HK2	GTACCAACACTGACCATG	224	92	0	0	0	0
HK2	TGACCAACACTGACACGT	44	79	0	1	0	0
HK2	ACTGGTACTGTGACACTG	84	102	1	0	0	0
HK3	GTACCAACACTGACTGGT	1434	817	0	1	1	1
HK3	GTACCAACACTGACACCA	561	404	2	1	0	1
HK3	ACACCAACACTGACACAC	21	38	0	0	0	0
HK3	CAACCAACACTGACACTG	1290	1070	2	3	0	29
HK3	CAACCAACACTGACTGCA	202	335	2	0	0	3
HK3	ACTGCACAGTTGGTCACA	132	208	0	0	0	0
HLA-A	ACACACTGTGCACATGCA	376	395	2	1	2	2
HLA-A	GTACACTGTGCACATGAC	64	61	0	0	0	0
HLA-A	TGACACTGTGCACATGTG	480	491	0	0	0	0
HLA-A	CAACACTGTGCAACGTGT	194	159	0	0	0	0
HLA-A	ACACACTGTGCAACGTCA	281	287	0	0	0	0
HLA-B	GTACACTGTGACACACAC	137	179	3	2652	2059	2
HLA-B	ACACACTGTGACCAACGT	137	140	0	0	0	0
HLA-B	ACACACTGTGACACCATG	279	409	1	0	0	0
HLA-B	CAACACTGTGACACACGT	153	57	0	0	0	1
HLA-B	ACACACTGTGACACACCA	632	428	0	0	0	1
HLA-C	CAACACTGTGACCACAGT	209	236	0	0	0	0
HLA-C	ACACACTGTGACCACACA	66	73	1	0	0	0
HLA-C	GTACACTGTGACCACAAC	5	19	0	0	0	0
HLA-C	TGACACTGTGACCACATG	357	775	1	0	0	0
HLA-C	CATGACGTACCATGACGT	122	248	0	0	0	4
HLA-DMA	ACACACTGTGACACGTAC	160	81	0	1	1	0
HLA-DMA	TGACACTGTGACACGTGT	460	1013	0	0	0	2
HLA-DMA	GTACACTGTGACACGTCA	71	74	0	7	0	0
HLA-DMA	TGTGCAGTTGACCACAAC	22	51	0	0	0	0
HLA-DMA	GTTGCAACCAGTCACAGT	237	203	0	0	0	0
HLA-DMA	CATGCAACCAGTCACACA	123	71	0	0	0	0
HLA-DMB	GTACACTGTGACGTTGGT	426	371	6	8	13	26991
HLA-DMB	CAACACTGTGACGTACTG	383	455	0	0	0	0
HLA-DMB	CAACACTGTGACGTTGCA	247	52	0	0	0	0
HLA-DMB	TGACACTGTGACGTTGAC	48	88	0	0	0	0
HLA-DMB	ACTGCAGTCAACACACAC	32	46	0	0	0	0
HLA-DMB	GTTGCAACCAGTCAGTCA	70	142	3	0	0	0
HLA-DOA	GTACACTGTGCATGCAAC	10	7	0	0	0	3
HLA-DOA	TGACACTGTGCATGCATG	149	230	0	0	0	4
HLA-DOA	ACACACTGTGCATGACGT	108	143	0	1	0	1
HLA-DOA	TGACACTGTGCATGACCA	201	444	0	0	0	1
HLA-DOA	CAACACTGTGCATGACAC	41	38	0	0	0	0
HLA-DOB	GTACACTGTGACCAACTG	797	495	0	0	0	1
HLA-DOB	TGACACTGTGACCATGGT	178	99	0	0	0	0
HLA-DOB	GTACACTGTGACCATGCA	632	590	0	1	0	3
HLA-DOB	ACACACTGTGACCATGAC	72	108	0	0	0	0
HLA-DOB	CAACACTGTGACCATGTG	351	512	0	0	0	0

HLA-DPA1	CAACACTGTGACACGTTG	1548	593	0	1	0	1
HLA-DPA1	GTACACTGTGACACCAGT	355	234	0	1	0	0
HLA-DPA1	CAACACTGTGACACCACA	173	180	0	0	0	0
HLA-DPA1	TGACACTGTGACACCAAC	62	54	0	0	0	0
HLA-DPA1	ACTGCAGTCAACCAGTGT	463	431	3	2	0	5
HLA-DPA1	ACTGCAACTGACCAGTCA	400	352	0	1	0	1
HLA-DPB1	GTACACTGTGACACTGTG	388	354	1	0	1	0
HLA-DPB1	TGACACTGTGACACACTG	437	608	0	1	0	1
HLA-DPB1	ACACACTGTGACACTGGT	521	290	1	0	0	0
HLA-DPB1	TGACACTGTGACACTGCA	168	150	0	0	0	0
HLA-DPB1	CAACACTGTGACACTGAC	93	106	0	0	0	8
HLA-DPB1	GTTGCAACTGACCAGTAC	111	63	0	0	0	0
HLA-DQA1	CATGGTTGGTTGTGGTCA	468	500	1	1	0	15
HLA-DQA1	TGTGGTTGGTTGTGGTAC	74	106	0	0	0	0
HLA-DQA1	CATGACACGTACACTGCA	321	123	1	1	0	59
HLA-DQA1	TGTGACACGTACACTGAC	94	25	0	0	0	0
HLA-DQA1	ACTGACACGTACACTGTG	718	357	0	1	0	0
HLA-DQA2	GTTGGTTGGTTGACTGGT	457	466	1	1	1	0
HLA-DQA2	ACTGGTTGGTTGACTGTG	1049	1212	0	1	1	1
HLA-DQA2	CATGGTTGGTTGACTGCA	33	32	0	0	0	0
HLA-DQA2	TGTGGTTGGTTGACTGAC	10	22	0	0	0	0
HLA-DQA2	GTTGGTTGGTTGTGGTGT	523	873	0	2	0	0
HLA-DQB1	GTACACTGTGCAACGTAC	59	59	0	0	0	0
HLA-DQB1	TGACACTGTGCAACGTTG	73	160	0	0	0	0
HLA-DQB1	ACACACTGTGCAACCAGT	453	636	0	0	0	6
HLA-DQB1	TGACACTGTGCAACCACA	156	105	0	0	0	0
HLA-DQB1	CAACACTGTGCAACCAAC	27	63	0	0	0	1
HLA-DQB1	TGTGCAACTGACGTTGTG	673	364	2	1	0	0
HLA-DQB2	GTACACTGTGCAACACCA	183	337	2	0	1	0
HLA-DQB2	GTACACTGTGCAACCATG	112	142	0	0	0	1
HLA-DQB2	TGACACTGTGCAACACGT	68	203	1	0	0	0
HLA-DQB2	ACACACTGTGCAACACAC	55	24	0	0	0	0
HLA-DQB2	CAACACTGTGCAACACTG	324	599	2	2	0	0
HLA-DRA	GTACACTGTGACCAGTGT	1038	604	1	1	1	17
HLA-DRA	ACACACTGTGACGTTGTG	270	578	0	2	0	0
HLA-DRA	CAACACTGTGACCAGTCA	431	216	0	0	0	0
HLA-DRA	TGACACTGTGACCAGTAC	88	112	0	0	0	0
HLA-DRA	ACACACTGTGACCAGTTG	1014	1173	1	0	0	0
HLA-DRB1	CAACACTGTGACGTGTGT	654	877	0	0	2	0
HLA-DRB1	CAACACTGTGCACATGGT	895	158	1	1	0	0
HLA-DRB1	ACACACTGTGACGTACAC	24	40	0	0	0	0
HLA-DRB1	TGACACTGTGACCAACCA	105	231	0	0	0	0
HLA-DRB1	CAACACTGTGACCAACAC	23	12	0	0	0	0
HLA-DRB1	GTTGCACAGTTGGTCAAC	5	25	0	0	0	0
HLA-E	CAACACTGTGACGTCAAC	16	57	0	0	0	0
HLA-E	GTACACTGTGACGTCATG	248	750	1	0	0	0
HLA-E	TGACACTGTGACGTACGT	255	151	0	0	0	0
HLA-E	GTACACTGTGACGTACCA	39	25	0	0	0	0
HLA-E	GTTGACCAGTCACAACAC	59	123	0	0	0	0
HLA-F	CAACACTGTGCATGGTCA	1680	1592	2	2	0	8
HLA-F	TGACACTGTGCATGGTAC	55	129	0	0	0	0
HLA-F	ACACACTGTGCATGGTTG	736	574	1	0	0	1
HLA-F	CAACACTGTGCATGCAGT	270	190	0	0	0	0
HLA-F	ACACACTGTGCATGCACA	95	101	0	0	0	1
HLA-G	CATGCAACTGACCAGTGT	592	806	3	3	1	100
HLA-G	GTACACTGTGCATGACTG	237	126	0	0	0	1
HLA-G	TGACACTGTGCATGTGGT	962	518	1	1	0	3
HLA-G	GTACACTGTGCATGTGCA	300	111	0	0	0	0
HLA-G	ACACACTGTGCATGTGAC	49	39	0	0	0	0
HLA-G	CAACACTGTGCATGTGTG	376	500	2	0	0	0
HLCS	GTACACGTACCAGTCATG	102	51	0	0	0	0
HLCS	TGACACGTACCAGTACGT	327	411	1	0	0	0
HLCS	GTACACGTACCAGTACCA	116	134	0	0	0	0
HLCS	ACACACGTACCAGTACAC	151	35	1	0	0	4
HLCS	CAACACGTACCAGTACTG	654	222	0	0	0	0
HLX	ACACGTACGTACACTGCA	199	226	8	9	1	3
HLX	CAACGTACGTACACTGGT	274	290	1	0	0	10
HLX	GTACGTACGTACACTGAC	6	11	0	0	0	0
HLX	TGACGTACGTACACTGTG	92	82	0	0	0	0
HLX	CAACGTACGTACTGGTGT	813	436	480	1	0	0
HLX	CATGCAACCACAACTGCA	373	278	0	0	0	0
HMBS	GTACACGTACCAACTGTG	378	239	0	0	0	0
HMBS	ACACACGTACCATGGTGT	318	606	0	0	0	0
HMBS	TGACACGTACCATGGTCA	114	90	0	0	0	0
HMBS	CAACACGTACCATGGTAC	59	56	1	0	0	0

HMBS	GTACACGTACCATGGTTG	825	1619	3	3	0	6
HMGA1	GTTGCAACACACTGGTGT	1242	1253	6	2	1	0
HMGA1	ACACGTACTGGTTGTGCA	212	229	0	0	0	0
HMGA1	GTACGTACTGGTTGTGAC	25	22	0	0	0	0
HMGA1	TGACGTACTGGTTGTGTG	248	138	0	0	0	0
HMGA1	GTACGTACTGCAGTGTGT	737	469	1	0	0	1
HMGA1	CATGCAACACACTGGTCA	145	160	0	0	0	0
HMGB1	ACACGTACTGGTCAGTTG	2321	2133	2	2	1	1
HMGB1	CAACGTACTGGTCAGTCA	146	189	0	0	0	3
HMGB1	TGACGTACTGGTCAGTAC	87	56	0	0	0	0
HMGB1	ACTGGTGTCAGTGTTGGT	353	310	0	1	0	1
HMGB1	TGTGGTGTCAGTGTTGCA	162	182	0	0	0	0
HMGB2	ACACGTACTGCACAACCA	165	140	1	0	1	0
HMGB2	TGACGTACTGCACAACTG	1003	937	1	1	1	0
HMGB2	GTACGTACTGCACAACAC	55	69	0	0	0	0
HMGB2	ACACGTACTGCACATGGT	585	624	1	2	0	0
HMGB2	TGACGTACTGCACATGCA	390	338	1	2	0	0
HMGB2	GTTGCAACGTGTACGTAC	27	12	0	0	0	0
HMGB3	GTACGTACACGTCAGTAC	344	71	1	0	0	0
HMGB3	ACACGTACTGGTCATGAC	87	112	0	0	0	0
HMGB3	CAACGTACTGGTCATGTG	480	717	0	19	0	7
HMGB3	TGACGTACTGGTACGTGT	388	248	2	0	0	0
HMGB3	TGACTGACTGACGTTGCA	86	69	0	1	0	0
HMGCL	CAACTGTGCATGCAACGT	722	73	2	0	1	0
HMGCL	TGACTGTGCATGCACAAC	6	5	0	0	0	0
HMGCL	ACACTGTGCATGCACATG	185	190	0	0	0	0
HMGCL	ACACTGTGCATGCAACCA	389	338	1	0	0	0
HMGCL	GTACTGTGCATGCAACAC	63	53	0	1	0	0
HMGCR	TGACACGTTGACACACCA	326	751	0	1	1	10
HMGCR	GTACACGTTGACACCAAC	16	17	0	0	0	0
HMGCR	TGACACGTTGACACCATG	185	212	0	0	0	0
HMGCR	ACACACGTTGACACACGT	37	71	0	0	0	0
HMGCR	CAACACGTTGACACACAC	33	114	0	0	0	0
HMGCR	CATGCACACAACGTACAC	24	29	0	0	0	0
HMGCS1	GTACACGTACCATGACGT	171	155	0	0	0	0
HMGCS1	CAACACGTACCATGACCA	771	797	1	0	0	0
HMGCS1	TGACACGTACCATGACAC	16	24	0	1	0	0
HMGCS1	ACACACGTACCATGACTG	949	345	0	88	0	1
HMGCS1	CATGGTTGTGGTCAGTAC	45	44	0	0	0	0
HMGCS2	GTACACGTACACGTCAGT	189	249	3	5	3	11
HMGCS2	CAACACGTACACGTGTTG	107	70	0	0	0	0
HMGCS2	CAACACGTACACGTCACA	69	40	0	0	0	0
HMGCS2	TGACACGTACACGTCAAC	0	5	0	0	0	0
HMGCS2	TGTGACGTACGTGTCAAC	3	1	0	0	0	0
HMGN1	TGACGTACTGCAACACAC	42	24	0	0	1	0
HMGN1	ACACGTACTGCAACACTG	403	491	2	0	1	5
HMGN1	GTACGTACTGCAACACGT	157	136	0	0	0	0
HMGN1	CAACGTACTGCAACACCA	276	287	0	0	0	0
HMGN1	CATGACGTCACATGACTG	169	134	1	0	0	0
HMOX1	GTACACGTGTTGTGACGT	69	79	0	0	0	0
HMOX1	CAACACGTGTTGTGACCA	246	256	0	1	0	0
HMOX1	TGACACGTGTTGTGACAC	2	12	0	0	0	0
HMOX1	ACACACGTGTTGTGACTG	359	302	27	0	0	0
HMOX1	CAACACGTGTTGTGTGGT	249	271	0	0	0	0
HMOX1	ACTGCACAGTTGGTACGT	61	115	0	0	0	0
HMOX2	ACACACGTGTTGTGTGCA	25	55	4	0	0	12
HMOX2	GTACACGTGTTGTGTGAC	95	18	0	0	0	0
HMOX2	TGACACGTGTTGTGTGTG	118	150	0	0	0	0
HMOX2	GTACACGTCAGTGTGTGT	1442	1429	0	1	0	1
HMOX2	GTTGCAGTCAACGTCAGT	208	196	0	1	0	0
HMOX2	CATGCACATGGTGTGTCA	271	263	0	0	0	0
HNF4A	CAACGTACTGACGTACCA	110	142	0	0	0	0
HNF4A	TGACGTACTGACGTACAC	31	23	0	0	0	0
HNF4A	TGTGACCACACAACTGCA	170	161	2	0	0	4
HNF4A	CATGACCACACAACTGAC	50	77	0	0	0	0
HNF4A	GTTGACCACACAACTGTG	35	27	0	0	0	0
HNF4G	GTACGTACTGACCAACCA	126	301	0	0	1	0
HNF4G	ACACGTACTGACCAACAC	255	112	0	0	0	0
HNF4G	CAACGTACTGACCAACTG	176	277	0	0	0	0
HNF4G	GTACGTACTGACCATGGT	256	270	0	0	0	0
HNF4G	CAACGTACTGACCATGCA	49	47	0	0	0	0
HNMT	ACACCAACCATGCAGTTG	1122	1496	23	19	1	32
HNMT	GTACCAACCATGCAGTGT	1281	1080	5	1	0	52
HNMT	CAACCAACCATGCAGTCA	238	144	1	0	0	0
HNMT	TGACCAACCATGCAGTAC	76	57	0	0	0	0

HNMT	CAACCAACCATGCACAGT	329	230	4	0	0	2
HNRNPC	ACACGTGTGTTGACCAGT	3	14	0	0	0	0
HNRNPC	TGACGTGTGTTGACCACA	25	11	0	0	0	0
HNRNPC	CAACGTGTGTTGACCAAC	14	17	0	0	0	0
HNRNPC	GTACGTGTGTTGACCATG	19	64	0	0	0	0
HNRNPC	ACTGCAGTGTCAACTGCA	7	9	0	0	0	0
HNRPK	ACACTGACGTGTCACATG	95	163	0	1	1	0
HNRPK	TGACTGACGTGTCAACTG	729	711	0	0	1	1
HNRPK	CAACTGACGTGTCAACGT	350	356	0	0	0	0
HNRPK	ACACTGACGTGTCAACCA	117	55	0	0	0	0
HNRPK	GTACTGACGTGTCAACAC	47	29	0	0	0	0
HNRPK	ACTGCAACGTGTACACAC	8	20	0	0	0	0
HOPX	TGACGTACCATGCATGAC	22	22	0	0	0	0
HOPX	ACACGTACCATGCATGTG	307	845	2	0	0	5
HOPX	GTACGTACCATGACGTGT	235	201	0	0	0	0
HOPX	CAACGTACCATGACGTCA	110	103	0	0	0	0
HOPX	TGACGTACCATGACGTAC	32	44	0	0	0	0
HOXA1	ACACGTACGTTGTGTGCA	258	360	8	0	2	0
HOXA1	ACACGTACGTTGTGACTG	77	91	0	0	0	0
HOXA1	CAACGTACGTTGTGTGGT	140	71	0	0	0	0
HOXA1	GTACGTACGTTGTGTGAC	23	18	0	0	0	0
HOXA1	TGTGGTTGTGCACAGTTG	947	405	0	0	0	0
HOXA10	CAACGTCAACCAACACCA	714	560	2	0	1	0
HOXA10	TGACGTACCAGTCATGCA	130	71	0	0	0	0
HOXA10	CAACGTACCAGTCATGAC	116	66	0	0	0	0
HOXA10	GTACGTACCAGTCATGTG	434	528	0	0	0	11
HOXA10	ACACGTACCAGTACGTGT	145	132	0	0	0	0
HOXA11	GTACGTACCAGTACACGT	53	57	0	3	0	0
HOXA11	CAACGTACCAGTACACCA	131	97	0	0	0	0
HOXA11	TGACGTACCAGTACACAC	12	51	0	0	0	0
HOXA11	GTACTGACTGACGTGTCA	167	244	2	0	0	0
HOXA11	ACACTGACTGACGTGTAC	3	2	0	0	0	0
HOXA13	TGACGTACCAGTACTGTG	206	345	0	0	0	0
HOXA13	CAACGTACCAGTTGGTGT	369	465	1	0	0	0
HOXA13	ACACGTACCAGTTGGTCA	106	117	0	0	0	2
HOXA13	GTACGTACCAGTTGGTAC	58	95	0	0	0	0
HOXA13	TGACGTACCAGTTGGTTG	676	768	3	0	0	0
HOXA4	GTACGTACCATGTGCAGT	637	794	68	48	59	51231
HOXA4	CAACGTACACCATGTGGT	221	246	0	0	0	0
HOXA4	ACACGTACACCATGTGCA	150	150	1	0	0	1
HOXA4	GTACGTACACCATGTGAC	58	77	0	0	0	0
HOXA4	GTTGCAGTACACACACAC	40	67	0	0	0	0
HOXA7	TGACGTCAACCAACCAGT	344	199	0	0	0	0
HOXA7	GTACGTCAACCAACCACA	83	94	0	0	0	0
HOXA7	ACACGTCAACCAACCAAC	33	49	0	0	0	0
HOXA7	TGACGTACCAGTGTTGGT	257	217	0	1	0	0
HOXA7	GTACGTACCAGTGTTGCA	166	125	0	0	0	0
HOXA9	CAACGTCAACCAACCATG	24	48	0	0	0	0
HOXA9	GTACGTCAACCAACACGT	76	32	0	0	0	0
HOXA9	TGACGTACCAGTCACAAC	9	9	0	0	0	0
HOXA9	ACACGTACCAGTCACATG	450	350	1	0	0	0
HOXA9	CAACGTACCAGTCAACGT	281	225	0	0	0	0
HOXB4	ACACGTACCACAACCAGT	1724	1608	1	1	2	3
HOXB4	TGACGTACCACAACGTTG	299	326	0	0	0	1
HOXB4	TGACGTACCACAACCACA	266	154	1	0	0	0
HOXB4	CAACGTACCACAACCAAC	29	21	0	0	0	0
HOXB4	GTACGTACCACAACCATG	101	59	0	0	0	0
HOXB6	ACACGTACCAACGTCAGT	116	129	0	0	0	0
HOXB6	TGACGTACCAACGTCACA	48	46	0	0	0	0
HOXB6	CAACGTACCAACGTCAAC	10	17	0	0	0	0
HOXB6	GTACGTACCAACGTCATG	69	55	0	0	0	0
HOXB6	CATGGTGTCAGTACCAAC	10	12	0	0	0	0
HOXB6	TGTGCAACTGACCAACGT	497	249	0	2	0	0
HOXC13	GTACGTACTGACTGACAC	15	5	0	0	0	0
HOXC13	TGACGTACTGACTGACTG	690	390	2	1	0	0
HOXC13	ACACGTACTGACTGTGGT	740	444	0	1	0	1
HOXC13	TGACGTACTGACTGTGCA	231	173	0	0	0	0
HOXC13	CAACGTACTGACTGTGAC	24	32	0	0	0	0
HOXC13	TGTGCAACTGACGTGTCA	225	199	0	1	0	0
HOXD1	TGACGTTGGTGTTGACGT	391	134	0	1	1	2
HOXD1	TGACGTTGGTGTTGCACA	37	22	0	0	0	0
HOXD1	CAACGTTGGTGTTGCAAC	7	12	0	0	0	0
HOXD1	GTACGTTGGTGTTGCATG	523	472	1	0	0	0
HOXD1	GTACGTTGGTGTTGACCA	192	237	0	0	0	1
HOXD1	GTTGCACATGTGCATGGT	330	224	0	0	0	0

HOXD10	TGACGTTGGTCAGTACTG	603	454	1	0	0	0
HOXD10	ACACGTTGGTCAGTTGGT	140	145	2	0	0	0
HOXD10	TGACGTTGGTCAGTTGCA	513	183	0	0	0	0
HOXD10	CAACGTTGGTCAGTTGAC	19	30	0	0	0	0
HOXD10	GTACGTTGGTCAGTTGTG	696	285	0	0	0	0
HOXD11	TGACGTTGGTCAACCACA	157	168	649	0	0	1
HOXD11	CAACGTTGGTCAACCAAC	8	19	0	0	0	0
HOXD11	GTACGTTGGTCAACCATG	142	126	0	0	0	0
HOXD11	TGACGTTGGTCAACACGT	12	50	0	0	0	0
HOXD11	CAACTGACTGCATGTGGT	241	239	0	1	0	24
HOXD11	CATGCAACTGGTACACGT	261	113	0	0	0	1
HOXD13	GTACGTTGGTCATGTGCA	245	196	0	0	0	0
HOXD13	ACACGTTGGTCATGTGAC	34	62	0	0	0	0
HOXD13	CAACGTTGGTCATGTGTG	169	150	0	0	0	0
HOXD13	CAACGTTGGTACGTGTGT	674	177	0	1	0	0
HOXD13	TGTGCAGTGTTGACTGAC	35	33	0	0	0	0
HP1BP3	ACTGGTGTCAGTCAGTGT	564	543	1	0	1	0
HP1BP3	TGTGGTTGCAACTGCATG	291	96	0	0	0	0
HP1BP3	ACTGGTTGCAACTGACGT	89	116	0	0	0	1
HP1BP3	TGTGGTTGCAACTGACCA	358	561	1	1	0	7
HP1BP3	CATGGTTGCAACTGACAC	46	116	0	0	0	1
HPCAL1	CAACACTGCAACCAGTTG	522	936	1	0	1	5
HPCAL1	GTACACTGCAACCACAGT	239	325	0	0	0	7
HPCAL1	CAACACTGCAACCACACA	122	214	5	0	0	0
HPCAL1	TGACACTGCAACCACAAC	65	49	0	0	0	0
HPCAL1	CATGCAGTTGCATGCACA	174	142	0	0	0	0
HPCAL1	TGTGCAACCACAGTACTG	192	491	1	0	0	0
HPD	TGACTGACACTGTGTGTG	160	260	0	1	2	1
HPD	CAACTGACTGGTGTGTAC	385	285	0	0	1	28
HPD	ACACTGACTGGTGTGTGT	34	50	0	0	0	0
HPD	TGACTGACTGGTGTGTCA	340	238	1	0	0	0
HPD	GTACTGACTGGTGTGTTG	119	140	0	0	0	0
HPGD	CACATGCACACAGTGTGT	834	257	3	1	0	0
HPGD	ACCATGCACACAGTGTCA	190	244	0	1	0	0
HPGD	GTCATGCACACAGTGTAC	54	34	0	0	0	0
HPGD	TGCATGCACACAGTGTTG	495	544	0	0	0	0
HPGD	ACCATGCACACAGTCAGT	213	218	0	0	0	3
HPN	CAACGTTGTGTGGTCAGT	290	402	2	0	0	0
HPN	GTACGTTGTGTGGTCAAC	43	28	0	0	0	0
HPN	GTACTGTGGTGTCAGTGT	1446	967	1	0	0	0
HPN	CAACTGTGGTGTCAGTCA	243	264	0	0	0	0
HPN	TGACTGTGGTGTCAGTAC	44	59	0	0	0	0
HPR	ACACTGTGCAGTTGGTAC	87	17	0	0	0	0
HPR	CAACTGTGCAGTTGGTTG	912	1145	2	3	0	21
HPR	GTACTGTGCAGTTGCAGT	252	300	1	0	0	0
HPR	CAACTGTGCAGTTGCACA	72	45	0	0	0	0
HPR	TGACTGTGCAGTTGCAAC	16	24	0	0	0	0
HPRT1	CAACCACAACACCAGTCA	249	355	2	1	0	0
HPRT1	TGACCACAACACCAGTAC	77	34	0	0	0	0
HPRT1	ACACCACAACACCAGTTG	902	1268	1	0	0	0
HPRT1	CAACCACAACACCACAGT	310	456	0	0	0	0
HPRT1	ACACCACAACACCACACA	285	146	0	0	0	0
HPRT1	TGTGCACAGTTGGTACCA	134	120	0	0	0	0
HPSE	ACTGGTCATGGTCAGTTG	723	594	1	0	0	17
HPSE	CATGGTCATGGTCACAGT	62	105	0	0	0	0
HPSE	ACTGGTCATGGTCACACA	212	104	0	0	0	0
HPSE	GTTGGTCATGGTCACAAC	37	26	0	0	0	0
HPSE	TGTGGTCATGGTCACATG	503	417	1	0	0	0
HPSE	GTTGCAACCACACATGAC	89	85	0	1	0	0
HPSE2	GTTGGTTGCAACTGACTG	1520	865	1	1	1	0
HPSE2	TGTGGTTGCAACTGTGGT	152	148	0	0	0	0
HPSE2	GTTGGTTGCAACTGTGCA	162	112	0	1	0	0
HPSE2	ACTGGTTGCAACTGTGAC	9	16	1	0	0	0
HPSE2	ACTGACCATGCACACAGT	176	282	1	0	0	0
HPX	ACTGCACATGACGTACAC	67	25	0	4	1	1
HPX	GTACTGCACATGGTACGT	433	248	0	0	0	0
HPX	CAACTGCACATGGTACCA	149	212	0	2	0	0
HPX	TGACTGCACATGGTACAC	122	13	0	0	0	0
HPX	ACACTGCACATGGTACTG	411	682	1	0	0	0
HPX	CAACTGCACATGGTTGGT	108	364	0	0	0	0
HR	TGACGTTGGTTGGTCAGT	183	330	0	0	0	0
HR	GTACGTTGGTTGGTCACA	88	222	0	0	0	0
HR	ACACGTTGGTTGGTCAAC	30	36	0	0	0	0
HR	CAACGTTGGTTGGTCATG	115	156	2	0	0	0
HR	GTACGTTGGTTGGTACGT	112	133	0	0	0	0

HRAS	GTACCACAGTGTGTGTAC	26	16	0	0	1	0
HRAS	TGACCAGTTGTGTGTGAC	149	164	0	0	0	0
HRAS	ACACCAGTTGTGTGTGTG	79	89	0	0	0	0
HRAS	CAACCACAGTGTGTGTGT	200	260	0	1	0	0
HRAS	ACACCACAGTGTGTGTCA	413	520	0	698	0	1
HRB	ACACTGCACATGGTTGCA	128	111	0	0	0	0
HRB	GTACTGCACATGGTTGAC	53	42	0	0	0	0
HRB	TGACTGCACATGGTTGTG	287	202	1	0	0	1
HRB	CAACTGCACATGCAGTGT	662	512	1	0	0	0
HRB	ACACTGCACATGCAGTCA	142	93	0	0	0	2
HRG	TGACTGTGGTCAGTGTAC	28	40	0	0	0	0
HRG	ACACTGTGGTCAGTGTTG	94	98	0	0	0	0
HRG	CAACTGTGGTCAGTCAGT	344	310	1	0	0	0
HRG	ACACTGTGGTCAGTCACA	90	25	0	0	0	0
HRG	GTACTGTGGTCAGTCAAC	85	77	5684	5	0	0
HRG	ACTGCACATGTGTGCATG	94	269	1	1	0	0
HRH1	TGACGTCACAACCACAAC	34	44	0	0	0	0
HRH1	ACACGTCACAACCACATG	380	524	1	0	0	5
HRH1	CAACGTCACAACCAACGT	741	1135	2	2	0	0
HRH1	ACACGTCACAACCAACCA	266	378	0	0	0	0
HRH1	GTTGCAGTGTTGGTCAGT	136	235	0	0	0	4
HRH1	ACTGCACATGTGTGGTAC	41	46	1	0	0	0
HRH2	CATGCACATGTGTGGTTG	431	739	0	1	1	1
HRH2	GTACGTCACAACCAACAC	38	121	0	0	0	0
HRH2	TGACGTCACAACCAACTG	898	524	1	1	0	0
HRH2	ACACGTCACAACCATGGT	441	689	2	0	0	1
HRH2	TGACGTCACAACCATGCA	591	631	0	1	0	1
HRH2	GTTGCAGTACACGTACCA	503	83	0	0	0	0
HRH3	CAACGTCACAACCATGAC	101	226	1	0	0	0
HRH3	GTACGTCACAACCATGTG	809	643	0	0	0	1
HRH3	ACACGTCACAACACGTGT	400	622	0	0	0	1
HRH3	TGACGTCACAACACGTCA	187	148	0	0	0	0
HRH3	CAACGTCACAACACGTAC	83	78	0	0	0	0
HRH3	CATGCACAACCAACCATG	238	185	1	0	0	0
HRH4	GTACGTCACAACACGTTG	823	295	0	0	0	0
HRH4	TGACGTCACAACACCAGT	106	110	0	0	0	0
HRH4	GTACGTCACAACACCACA	82	100	0	0	0	0
HRH4	ACACGTCACAACACCAAC	15	30	0	0	0	0
HRH4	CAACGTCACAACACCATG	299	69	0	0	0	0
HRH4	TGTGCAACGTGTTGGTAC	54	159	0	0	0	0
HRK	GTACCACAGTTGCACACA	118	87	1417	0	0	0
HRK	ACACCACAGTTGCACAAC	53	10	0	0	0	2
HRK	CAACCACAGTTGCACATG	214	214	0	1	0	1
HRK	GTACCACAGTTGCAACGT	94	111	0	1	0	1
HRK	TGTGCAGTACTGCACACA	12	14	0	0	0	0
HRK	CATGCACAGTTGGTACAC	11	18	0	0	0	0
HS2ST1	ACTGCACATGACTGGTGT	1902	1606	3	1	1	1
HS2ST1	TGACCACAACCAGTCAAC	24	32	0	0	0	0
HS2ST1	ACACCACAACCAGTCATG	235	123	0	0	0	0
HS2ST1	CAACCACAACCAGTACGT	361	408	2	1	0	0
HS2ST1	ACACCACAACCAGTACCA	98	84	0	0	0	0
HS2ST1	GTACCACAACCAGTACAC	65	115	0	0	0	0
HS3ST1	GTCATGTGACCAACCAGT	173	127	0	0	0	0
HS3ST1	CACATGTGACCAACCACA	180	211	0	0	0	8
HS3ST1	TGCATGTGACCAACCAAC	45	35	0	0	0	0
HS3ST1	ACCATGTGACCAACCATG	335	146	0	0	0	1
HS3ST1	ACTGCAGTCATGACCAAC	192	31	0	0	0	0
HS3ST2	CAACCAACGTACGTGTTG	232	338	0	0	1	0
HS3ST2	ACACCAACGTACGTGTAC	35	31	1	0	0	0
HS3ST2	GTACCAACGTACGTCAGT	154	492	0	2	0	0
HS3ST2	CAACCAACGTACGTCACA	43	50	0	0	0	0
HS3ST2	TGACCAACGTACGTCAAC	57	143	0	0	0	0
HS3ST2	GTTGCACAACGTACCAGT	78	112	1	0	0	0
HS3ST3A1	ACACCAACGTCATGGTGT	735	862	2	1	1	0
HS3ST3A1	GTACCAACGTCAACTGTG	172	231	0	0	0	0
HS3ST3A1	TGACCAACGTCATGGTCA	543	667	0	0	0	1
HS3ST3A1	ACACCAACGTCATGACTG	290	421	1	0	0	0
HS3ST3A1	CAACCAACGTCATGTGGT	300	344	2	0	0	0
HS3ST3A1	CATGCACAACGTACCACA	195	194	0	0	0	1
HS3ST3B1	TGACCAACGTCAACTGCA	263	268	44	0	0	0
HS3ST3B1	CAACCAACGTCAACTGAC	13	19	0	0	0	0
HS3ST3B1	GTTGCAGTACTGCAACCA	140	100	0	0	0	0
HS3ST3B1	TGTGCACAACGTACCAAC	15	11	0	0	0	0
HS3ST3B1	ACTGCACAACGTACCATG	150	190	1	0	0	0
HS3ST3B1	CATGCACAACGTACACGT	45	56	0	0	0	0

HS3ST5	CAACCAACCAACTGTGCA	161	129	0	0	0	0
HS3ST5	TGACCAACCAACTGTGAC	61	98	0	0	0	0
HS3ST5	ACACCAACCAACTGTGTG	62	87	1	0	0	0
HS3ST5	CAACCAACCATGGTGTGT	607	404	47	1	0	0
HS3ST5	ACACCAACCATGGTGTCA	137	121	0	0	0	0
HS6ST1	TGACCACACACAACACTG	202	206	1	1	0	0
HS6ST1	ACACCACACACAACTGGT	619	619	1	0	0	7
HS6ST1	TGACCACACACAACTGCA	140	329	0	0	0	0
HS6ST1	TGACCAACGTTGCATGCA	329	303	1	0	0	0
HS6ST1	CAACCAACGTTGCATGAC	75	58	0	0	0	0
HS6ST1	ACTGCACACATGCAGTTG	36	64	0	0	0	0
HS6ST2	GTACCAACCAACGTCAAC	33	26	0	0	0	0
HS6ST2	TGACCAACCAACGTCATG	268	399	0	0	0	0
HS6ST2	ACACCAACCAACGTACGT	175	112	0	0	0	0
HS6ST2	TGACCAACCAACGTACCA	152	153	0	0	0	0
HS6ST2	CAACCAACCAACGTACAC	21	30	0	0	0	0
HS6ST3	CAACCAACCAACACCATG	350	83	2	1	0	1
HS6ST3	GTACCAACCAACACACGT	34	37	0	0	0	0
HS6ST3	CAACCAACCAACACACCA	460	428	0	1	0	2
HS6ST3	TGACCAACCAACACACAC	16	25	0	0	0	0
HS6ST3	ACACCAACCAACACACTG	1081	1960	2	0	0	2
HSD11B1	ACACCAGTGTGTACGTGT	403	405	0	0	0	0
HSD11B1	TGACCAGTGTGTACGTCA	170	159	0	0	0	0
HSD11B1	CAACCAGTGTGTACGTAC	100	219	0	0	0	0
HSD11B1	GTACCAGTGTGTACGTTG	239	147	1	0	0	0
HSD11B1	ACTGCAGTACTGGTACTG	418	477	0	0	0	1
HSD11B1	TGTGCATGCACAACACAC	16	22	0	0	0	0
HSD11B2	ACACCAGTGTGTCACATG	115	87	0	0	0	0
HSD11B2	CAACCAGTGTGTCAACGT	405	338	0	1	0	10
HSD11B2	ACACCAGTGTGTCAACCA	111	133	0	0	0	0
HSD11B2	GTACCAGTGTGTCAACAC	59	47	0	0	0	0
HSD11B2	TGACCAGTGTGTCAACTG	289	184	0	2	0	0
HSD17B1	TGACCAGTGTGTCAGTGT	402	606	2	0	1	8
HSD17B1	GTTGCACAGTTGGTACTG	44	33	0	0	1	0
HSD17B1	CAACCAGTGTGTGTTGTG	677	415	2	0	0	0
HSD17B1	GTACCAGTGTGTCAGTCA	40	98	0	0	0	0
HSD17B1	ACACCAGTGTGTCAGTAC	76	97	0	0	0	0
HSD17B1	GTTGCAGTACTGGTCACA	297	234	0	0	0	0
HSD17B10	TGACCAGTGTCAGTGTGT	1067	911	62	2	2	0
HSD17B10	CAACCAGTGTCAGTGTTG	382	629	1	0	1	0
HSD17B10	TGACCAGTGTCATGTGGT	778	1004	3	0	1	0
HSD17B10	GTACCAGTGTCAGTGTCA	423	613	1	1	0	0
HSD17B10	ACACCAGTGTCAGTGTAC	75	111	0	0	0	0
HSD17B12	ACACCAGTCAGTGTTGTG	315	509	3	2	15	3
HSD17B12	GTACCAGTCAGTGTTGGT	308	677	0	0	1	0
HSD17B12	CAACCAGTCAGTGTACTG	305	334	0	0	0	0
HSD17B12	CAACCAGTCAGTGTTGCA	184	116	0	0	0	0
HSD17B12	TGACCAGTCAGTGTTGAC	29	136	0	0	0	0
HSD17B2	GTACGTTGTGTGTGACCA	304	353	262	212	221	715623
HSD17B2	TGACGTTGTGTGTGACGT	140	161	0	0	0	0
HSD17B2	ACACGTTGTGTGTGACAC	18	20	0	0	0	0
HSD17B2	CAACGTTGTGTGTGACTG	250	321	0	1	0	0
HSD17B2	GTACGTTGTGTGTGTGGT	544	825	0	1	0	59
HSD17B2	TGTGCACAGTTGGTTGGT	74	45	0	0	0	0
HSD17B3	TGACCAGTGTGTGTCATG	237	619	113	0	0	0
HSD17B3	ACACCAGTGTGTGTACGT	142	167	0	0	0	0
HSD17B3	TGACCAGTGTGTGTACCA	84	108	2	0	0	0
HSD17B3	CAACCAGTGTGTGTACAC	12	23	0	0	0	0
HSD17B3	ACACCAGTGTGTGTTGAC	35	7	0	0	0	0
HSD17B3	GTTGCACACAACGTACTG	61	27	0	0	0	0
HSD17B4	GTACGTTGTGTGACTGAC	22	9	0	0	0	7
HSD17B4	TGACGTTGTGTGACTGTG	830	203	0	0	0	1
HSD17B4	CAACGTTGTGTGTGGTGT	310	434	1	0	0	3
HSD17B4	ACACGTTGTGTGTGGTCA	44	47	0	0	0	0
HSD17B4	GTACGTTGTGTGTGGTAC	92	54	0	0	0	0
HSD17B4	GTTGCACAGTTGGTTGCA	369	517	0	1	0	2
HSD17B6	GTACACCAACTGACTGTG	567	460	1	1	0	0
HSD17B6	ACACACCAACTGTGGTGT	108	221	1	0	0	0
HSD17B6	TGACACCAACTGTGGTCA	332	161	0	0	0	0
HSD17B6	CAACACCAACTGTGGTAC	88	79	0	0	0	0
HSD17B6	GTACACCAACTGTGGTTG	977	720	1	0	0	0
HSD17B6	TGTGCAACCAGTTGACGT	164	81	0	0	0	0
HSD17B7	TGACCAGTCAGTGTCACA	105	149	2	2	2	9
HSD17B7	TGACCAGTCAGTGTGTTG	1139	1028	3	2	1	4
HSD17B7	GTACCAGTCAGTGTGTAC	95	88	0	0	0	0

HSD17B7	ACACCAGTCAGTGTCAGT	175	124	0	0	0	0
HSD17B7	CAACCAGTCAGTGTCAAC	86	119	0	0	0	0
HSD17B8	ACACCAGTCATGTGGTTG	1222	773	2	2	0	26
HSD17B8	CAACCAGTCATGTGCAGT	289	241	1	1	0	0
HSD17B8	ACACCAGTCATGTGCACA	190	186	0	0	0	0
HSD17B8	GTACCAGTCATGTGCAAC	30	25	0	0	0	0
HSD17B8	TGACCAGTCATGTGCATG	132	233	1	0	0	0
HSD17B8	CATGCAACCAACCAACAC	46	28	0	0	0	0
HSD3B1	TGACCAACACCAGTTGGT	39	37	0	0	0	0
HSD3B1	GTACCAACACCAGTTGCA	285	520	0	1	0	0
HSD3B1	TGACCAACACCACAGTGT	293	390	1	1	0	0
HSD3B1	GTTGACACCAGTGTCAGT	185	536	3041	1	0	1
HSD3B1	CATGACACCAGTGTCACA	38	52	0	0	0	0
HSD3B2	ACACCAACACCAGTTGAC	23	46	1	0	0	0
HSD3B2	CAACCAACACCAGTTGTG	60	45	0	0	0	0
HSD3B2	TGACCAACACCACACAAC	28	47	0	0	0	0
HSD3B2	ACACCAACACCACACATG	4	6	0	0	0	0
HSD3B2	CAACCAACACCACAACGT	455	310	0	0	0	0
HSD3B7	CAACCAGTCATGACCAAC	149	73	0	0	0	0
HSD3B7	GTACCAGTCATGACCATG	601	252	1	1	0	0
HSD3B7	TGACCAGTCATGACACGT	162	81	0	0	0	0
HSD3B7	GTACCAGTCATGACACCA	166	116	0	0	0	6
HSD3B7	ACTGCAGTGTTGGTTGGT	479	189	0	0	0	1
HSF1	TGACGTGTACCACATGTG	109	94	0	0	0	0
HSF1	CAACGTGTACCAACGTGT	573	433	2	1	0	0
HSF1	ACACGTGTACCAACGTCA	177	298	0	0	0	0
HSF1	GTACGTGTACCAACGTAC	32	46	0	0	0	0
HSF1	TGACGTGTACCAACGTTG	870	523	0	2	0	0
HSF2	GTACGTACCAACACCAGT	79	122	0	0	0	0
HSF2	CAACGTACCAACACCACA	180	236	0	0	0	0
HSF2	TGACGTTGGTTGACACCA	478	401	0	0	0	0
HSF2	CAACGTTGGTTGACACAC	19	16	1	0	0	0
HSF2	GTACGTTGGTTGACACTG	468	471	0	0	0	0
HSF2	ACTGCACAACACCAGTCA	141	182	0	0	0	1
HSP90AA1	TGCATGCATGACGTCATG	556	532	0	1	1	0
HSP90AA1	ACCATGCATGACGTCACA	25	33	0	0	0	0
HSP90AA1	GTCATGCATGACGTCAAC	51	21	0	0	0	0
HSP90AA1	ACCATGCATGACGTACGT	22	14	0	0	0	0
HSP90AA1	CAACGTCAGTTGGTGTCA	65	52	0	0	0	0
HSP90AB1	TGACGTCAGTTGTGGTTG	1385	1214	3	0	1	208
HSP90AB1	GTACGTCAGTTGTGGTAC	121	112	1	0	0	0
HSP90AB1	ACACGTCAGTTGTGCAGT	164	155	0	0	0	0
HSP90AB1	TGACGTCAGTTGTGCACA	276	215	0	0	0	0
HSP90AB1	CAACGTCAGTTGTGCAAC	24	21	0	0	0	0
HSP90AB1	TGTGCAACCAACACGTGT	256	290	0	0	0	0
HSP90B1	CAACCAGTACTGACGTTG	1186	351	1	0	0	1
HSP90B1	GTACCAGTACTGACCAGT	201	197	0	0	0	0
HSP90B1	CAACCAGTACTGACCACA	104	99	0	0	0	43
HSP90B1	TGACCAGTACTGACCAAC	19	49	0	0	0	0
HSP90B1	TGTGCAGTGTTGGTACTG	845	417	0	0	0	0
HSP90B1	ACTGCACACACAGTACTG	69	118	0	0	0	0
HSPA12A	GTTGACCAACTGTGGTCA	286	290	3	3	2	8
HSPA12A	TGTGACCAACTGTGGTGT	736	874	1	0	0	1
HSPA12A	ACTGACCAACTGTGGTAC	168	200	0	0	0	0
HSPA12A	CATGACCAACTGTGGTTG	158	146	0	0	0	0
HSPA12A	GTTGACCAACTGTGCAGT	378	267	0	0	0	0
HSPA1A	GTTGCAACTGTGCAGTTG	539	475	0	0	1	2
HSPA1A	ACACGTCAGTTGGTGTTG	389	251	2	0	0	0
HSPA1A	CAACGTCAGTTGGTCAGT	87	109	0	0	0	0
HSPA1A	ACACGTCAGTTGTGACAC	38	50	1	0	0	0
HSPA1A	CAACGTCAGTTGTGACTG	168	350	0	0	0	0
HSPA1A	GTACGTCAGTTGTGTGGT	604	303	0	0	0	1
HSPA1B	CAACGTCAGTTGTGTGCA	229	109	4	2	0	7
HSPA1B	TGACGTCAGTTGTGTGAC	34	42	0	1	0	1
HSPA1B	ACACGTCAGTTGTGTGTG	265	325	0	0	0	3
HSPA1B	TGACGTCACAGTGTGTGT	832	442	1	2	0	0
HSPA1B	GTACGTCACAGTGTGTCA	459	755	0	0	0	0
HSPA1B	TGTGCAACTGTGCACAGT	86	114	1	0	0	4
HSPA1L	TGACCAGTACTGACACTG	1245	815	1	6	0	6
HSPA1L	ACACCAGTACTGACTGGT	162	106	0	0	0	0
HSPA1L	TGACCAGTACTGACTGCA	345	269	0	0	0	0
HSPA1L	CAACCAGTACTGACTGAC	185	92	0	0	0	0
HSPA1L	GTACCAGTACTGACTGTG	351	490	2	0	0	0
HSPA2	TGACGTCAGTTGGTGTAC	87	110	0	0	0	0
HSPA2	TGACCAGTACTGTGACAC	17	17	0	1	0	0

HSPA2	ACACCAGTACTGTGACTG	363	363	0	0	0	0
HSPA2	CAACCAGTACTGTGTGGT	1230	934	2	0	0	0
HSPA2	ACACCAGTACTGTGTGCA	131	86	0	0	0	0
HSPA2	ACTGCACATGTGTGTGGT	499	493	0	0	0	0
HSPA5	TGCATGCATGACGTGTAC	28	32	0	0	0	0
HSPA5	ACCATGCATGACGTGTTG	233	268	0	0	0	9
HSPA5	CACATGCATGACGTCAGT	583	574	2	0	0	2
HSPA5	CAACGTCAGTACTGTGTG	1561	806	2	1	0	1
HSPA5	GTACGTCAGTTGGTGTGT	348	746	0	0	0	0
HSPA5	CATGCAACACGTCAGTGT	88	81	0	0	0	0
HSPA8	GTTGCAACTGCAGTGTGT	662	406	0	0	1	0
HSPA8	CAACGTACACCACAGTCA	251	331	1	1	0	0
HSPA8	TGACGTACACCACAGTAC	39	167	0	0	0	0
HSPA8	ACACGTACACCACAGTTG	1962	2466	3	1	0	3
HSPA8	CAACGTACACCACACAGT	439	624	1	0	0	0
HSPA8	ACACGTACACCAACGTAC	71	128	0	0	0	0
HSPA9	GTACCAGTACTGTGCACA	92	167	0	0	0	0
HSPA9	ACACCAGTACTGTGCAAC	32	88	0	0	0	0
HSPA9	CAACCAGTACTGTGCATG	52	76	0	0	0	0
HSPA9	GTACCAGTACTGTGACGT	99	139	0	0	0	0
HSPA9	CAACCAGTACTGTGACCA	220	252	0	0	0	0
HSPB1	GTACGTCAGTTGTGCATG	906	851	0	2	0	0
HSPB1	TGACGTCAGTTGTGACGT	199	373	1	0	0	0
HSPB1	GTACGTCAGTTGTGACCA	597	448	0	0	0	0
HSPB1	TGTGCAGTACCAACTGAC	49	78	0	0	0	0
HSPB1	ACTGCAGTACCAACTGTG	318	267	0	0	0	0
HSPB1	GTTGCACAACGTGTGTAC	256	32	2	1	0	1
HSPB2	ACCATGTGGTGTACGTCA	87	120	0	0	0	0
HSPB2	GTCATGTGGTGTACGTAC	35	35	0	0	0	0
HSPB2	TGCATGTGGTGTACGTTG	67	121	0	0	0	0
HSPB2	ACCATGTGGTGTACCAGT	326	331	1	1	0	0
HSPB2	GTTGCAGTACGTTGACCA	45	38	0	0	0	0
HSPB2	ACTGCATGCACACAGTAC	261	191	1	0	0	0
HSPD1	ACACCAGTACTGTGGTGT	306	335	0	0	0	0
HSPD1	TGACCAGTACTGTGGTCA	149	160	0	0	0	1
HSPD1	CAACCAGTACTGTGGTAC	111	95	0	0	0	4
HSPD1	GTACCAGTACTGTGGTTG	675	495	1	0	0	0
HSPD1	TGACCAGTACTGTGCAGT	369	207	0	1	0	0
HSPE1	GTACTGACGTGTTGTGGT	112	157	0	0	0	0
HSPE1	CAACTGACGTGTTGTGCA	399	295	2	0	0	0
HSPE1	TGACTGACGTGTTGTGAC	6	9	0	0	0	0
HSPE1	ACTGGTGTCAGTTGTGAC	59	44	0	0	0	0
HSPE1	ACTGCAGTCACACAACGT	486	169	1	0	0	0
HSPE1	ACTGCACAGTTGGTTGAC	27	57	0	0	0	0
HSPG2	TGACTGTGGTGTTGCAGT	331	396	3017	2	0	0
HSPG2	GTACTGTGGTGTTGCACA	116	134	1	0	0	1
HSPG2	ACACTGTGGTGTTGCAAC	0	4	0	0	0	0
HSPG2	CAACTGTGGTGTTGCATG	43	64	0	0	0	0
HSPG2	TGTGACACACCACAGTGT	318	334	0	0	0	0
HTATIP	GTACGTTGCAGTCAACTG	930	664	1	0	2	0
HTATIP	CAACGTTGCAGTCAACAC	127	72	0	0	0	0
HTATIP	TGACGTTGCAGTCATGGT	229	327	0	1	0	0
HTATIP	GTACGTTGCAGTCATGCA	177	182	0	0	0	0
HTATIP	ACACGTTGCAGTCATGAC	57	102	1	0	0	0
HTR1A	CAACGTCACAACACTGGT	641	420	0	1	1	0
HTR1A	GTACGTCACAACACACGT	69	66	0	0	0	0
HTR1A	CAACGTCACAACACACCA	428	564	0	0	0	0
HTR1A	TGACGTCACAACACACAC	28	23	0	0	0	0
HTR1A	ACACGTCACAACACACTG	658	962	0	0	0	0
HTR1B	ACACGTCACAACACTGCA	130	179	0	0	0	0
HTR1B	GTACGTCACAACACTGAC	90	100	0	0	0	0
HTR1B	TGACGTCACAACACTGTG	443	577	0	0	0	0
HTR1B	GTTGCAGTACCATGTGCA	27	45	0	0	0	0
HTR1B	ACTGCAGTACCATGTGAC	60	91	0	1	0	0
HTR1B	CATGCACAGTTGGTTGTG	548	464	1	1	0	0
HTR1D	GTACTGACGTACACCAGT	283	241	0	1	0	0
HTR1D	CAACTGACGTACACCACA	271	94	1	0	0	0
HTR1D	TGACTGACGTACACCAAC	14	11	0	0	0	0
HTR1D	ACACTGACGTACACCATG	342	309	1	0	0	0
HTR1D	CAACTGACGTACACACGT	105	109	0	0	0	0
HTR1E	TGTGCACAGTTGCAGTGT	1052	463	0	1	1	1
HTR1E	ACACTGACGTACACACCA	206	206	0	0	0	0
HTR1E	GTACTGACGTACACACAC	34	62	0	0	0	0
HTR1E	TGACTGACGTACACACTG	843	662	2	1	0	0
HTR1E	ACACTGACGTACACTGGT	252	303	0	0	0	0

HTR1E	GTTGGTTGACACCACAAC	22	15	0	0	0	0
HTR1F	CAACGTCACAACTGGTGT	1427	855	0	0	0	0
HTR1F	ACACGTCACAACTGGTCA	855	1120	1	1	0	0
HTR1F	GTACGTCACAACTGGTAC	24	17	0	0	0	0
HTR1F	TGACGTCACAACTGGTTG	420	321	0	0	0	0
HTR1F	ACACGTCACAACTGCAGT	413	507	1	1	0	0
HTR1F	TGTGCACATGCAACCACA	288	406	0	0	0	1
HTR2A	CAACGTCACAACTGCAAC	57	56	105	0	1	0
HTR2A	TGACGTCACAACTGCACA	61	42	0	0	0	0
HTR2A	GTACGTCACAACTGCATG	128	257	1	0	0	0
HTR2A	TGACGTCACAACTGACGT	51	177	0	0	0	0
HTR2A	GTACGTCACAACTGACCA	674	342	0	1	0	0
HTR2B	CAACGTACGTCAGTGTAC	367	46	0	0	0	0
HTR2B	GTACGTACGTCAGTGTTG	572	428	1	0	0	0
HTR2B	TGACGTACGTCAGTCAGT	147	106	0	0	0	0
HTR2B	GTACGTACGTCAGTCACA	12	17	0	0	0	3
HTR2B	ACACGTACGTCAGTCAAC	27	18	0	0	0	0
HTR2C	ACACGTCACAACTGACAC	43	39	0	0	0	0
HTR2C	CAACGTCACAACTGACTG	272	619	3	0	0	2
HTR2C	GTACGTCACAACTGTGGT	518	247	0	0	0	0
HTR2C	CAACGTCACAACTGTGCA	163	202	0	1	0	0
HTR2C	TGACGTCACAACTGTGAC	74	62	0	0	0	0
HTR2C	GTTGCACAGTTGCAGTCA	61	49	0	0	0	0
HTR3A	CAACGTCACATGGTGTGT	1333	562	0	1	1	5
HTR3A	TGACGTCACATGGTGTTG	309	327	1	0	1	0
HTR3A	ACACGTCACAACTGTGTG	167	149	0	0	0	0
HTR3A	ACACGTCACATGGTGTCA	362	346	0	1	0	0
HTR3A	GTACGTCACATGGTGTAC	96	74	0	0	0	0
HTR3B	ACACGTCACATGGTCAGT	256	60	1	0	0	0
HTR3B	TGACGTCACATGGTCACA	18	9	0	0	0	0
HTR3B	CAACGTCACATGGTCAAC	23	18	0	0	0	0
HTR3B	GTACGTCACATGGTCATG	419	321	1	0	0	0
HTR3B	TGACGTCACATGGTACGT	285	185	0	0	0	0
HTR3C	ACACTGACGTCATGTGAC	32	35	0	0	0	0
HTR3C	CAACTGACGTCATGTGTG	386	417	0	0	0	0
HTR3C	CAACTGACGTACGTGTGT	295	386	0	1	0	0
HTR3C	ACACTGACGTACGTGTCA	85	68	0	0	0	0
HTR3C	GTACTGACGTACGTGTAC	16	36	0	0	0	0
HTR3D	GTACTGCAACCAACCAGT	357	530	0	0	0	0
HTR3D	CAACTGCAACCAACCACA	189	179	0	0	0	0
HTR3D	TGACTGCAACCAACCAAC	58	37	0	0	0	0
HTR3D	ACACTGCAACCAACCATG	711	618	1	0	0	0
HTR3D	CAACTGCAACCAACACGT	21	20	0	0	0	0
HTR3E	GTTGACGTCACACAACAC	163	163	1	0	1	0
HTR3E	CAACTGCAACGTCATGCA	307	648	0	0	0	0
HTR3E	TGACTGCAACGTCATGAC	52	100	0	0	0	0
HTR3E	ACACTGCAACGTCATGTG	102	66	1	0	0	0
HTR3E	TGTGACGTCACACAACTG	92	103	0	0	0	0
HTR4	GTACGTCACATGGTACCA	411	190	0	0	0	0
HTR4	ACACGTCACATGGTACAC	11	16	0	0	0	0
HTR4	CAACGTCACATGGTACTG	657	367	1	0	0	0
HTR4	GTACGTCACATGGTTGGT	596	597	1	0	0	1
HTR4	ACTGACCATGACCAACCA	155	225	2	0	0	59
HTR6	CATGCAGTTGTGCAGTCA	458	845	1	0	1	118
HTR6	TGACTGACGTTGACTGGT	359	493	0	0	0	0
HTR6	GTACTGACGTTGACTGCA	75	58	0	0	0	0
HTR6	ACACTGACGTTGACTGAC	18	23	0	0	0	0
HTR6	CAACTGACGTTGACTGTG	86	97	1	0	0	0
HTR6	ACTGCACAGTTGCAGTAC	22	22	0	0	0	0
HTR7	GTACGTCACATGCAGTGT	344	659	0	0	1	4
HTR7	CAACGTCACATGGTTGCA	212	199	0	0	0	0
HTR7	TGACGTCACATGGTTGAC	50	82	0	0	0	0
HTR7	ACACGTCACATGGTTGTG	150	245	0	1	0	0
HTR7	CAACGTCACATGCAGTCA	94	149	0	0	0	2
HTR7	TGTGCATGGTTGGTCAGT	198	203	0	0	0	0
HTRA2	CAACTGTGCACAGTGTGT	2571	2692	16	4	5030	67
HTRA2	TGACTGTGCAGTTGTGCA	288	352	0	1	2	0
HTRA2	ACACTGTGCAGTTGTGGT	208	215	1	1	0	0
HTRA2	CAACTGTGCAGTTGTGAC	70	183	1	19	0	0
HTRA2	GTACTGTGCAGTTGTGTG	666	721	0	0	0	0
HUS1	TGACGTACTGGTGTGTTG	352	229	7	2	1	9
HUS1	ACACGTACTGGTGTGTCA	459	292	0	2	0	0
HUS1	GTACGTACTGGTGTGTAC	43	37	0	0	0	0
HUS1	ACACGTACTGGTGTCAGT	623	444	0	0	0	1
HUS1	TGACGTACTGGTGTCACA	106	125	0	0	0	0

HUS1	ACTGCATGGTTGCAGTCA	539	429	0	0	0	1
HYAL1	ACACTGACGTTGCACAGT	36	101	0	0	0	0
HYAL1	TGACTGACGTTGCACACA	139	44	0	0	0	0
HYAL1	CAACTGACGTTGCACAAC	30	45	0	0	0	29
HYAL1	GTACTGACGTTGCACATG	437	277	1	0	0	0
HYAL1	CATGCAACTGACGTGTAC	197	100	0	0	0	0
HYAL1	GTTGCAACTGACGTGTTG	697	576	1	1	0	0
HYAL2	TGACTGACGTTGGTGTCA	169	75	0	0	0	0
HYAL2	CAACTGACGTTGGTGTAC	147	187	0	1	0	0
HYAL2	GTACTGACGTTGGTGTTG	448	311	0	0	0	0
HYAL2	TGACTGACGTTGGTCAGT	65	57	0	0	0	0
HYAL2	GTACTGACGTTGGTCACA	21	27	0	0	0	0
HYAL3	CAACTGACGTCATGCAGT	198	316	0	0	1	75
HYAL3	ACACTGACGTCATGCACA	55	92	0	0	0	47
HYAL3	GTACTGACGTCATGCAAC	131	38	1	0	0	0
HYAL3	TGACTGACGTCATGCATG	112	122	0	0	0	0
HYAL3	ACACTGACGTCATGACGT	198	228	0	0	0	0
HYAL3	ACTGCAACGTCAACGTTG	790	370	1	0	0	0
HYAL4	TGTGGTTGACTGTGGTGT	436	676	1	422	1	0
HYAL4	GTTGGTTGACTGACTGCA	289	130	0	0	0	0
HYAL4	ACTGGTTGACTGACTGAC	41	31	0	0	0	0
HYAL4	CATGGTTGACTGACTGTG	470	547	1	0	0	0
HYAL4	GTTGGTTGACTGTGGTCA	439	401	1	49	0	1
HYAL4	CATGCACAACACACGTCA	273	388	0	2	0	0
HYI	GTTGGTGTACGTTGACGT	22	31	0	0	0	0
HYI	GTTGGTACTGGTACACTG	514	917	0	0	0	0
HYI	TGTGGTACTGGTACTGGT	70	322	0	1	0	0
HYI	GTTGGTACTGGTACTGCA	716	302	1	243	0	0
HYI	ACTGGTACTGGTACTGAC	81	54	1	0	0	0
HYI	GTTGCAACTGCAACTGAC	40	49	0	0	0	0
HYOU1	ACACCAGTACCACATGTG	554	417	0	0	1	0
HYOU1	TGACCAGTACCACATGAC	84	74	0	0	0	0
HYOU1	GTACCAGTACCAACGTGT	223	327	0	0	0	0
HYOU1	CAACCAGTACCAACGTCA	951	1184	0	2	0	1
HYOU1	TGACCAGTACCAACGTAC	73	64	0	0	0	0
HYOU1	TGTGCAACGTGTGTCAAC	38	26	0	0	0	0
IAPP	ACTGGTCAGTTGGTCAGT	32	86	0	0	0	0
IAPP	TGTGGTCAGTTGGTCACA	189	230	0	0	0	1
IAPP	CATGGTCAGTTGGTCAAC	147	85	0	0	0	0
IAPP	GTTGGTCAGTTGGTCATG	309	210	1	0	0	0
IAPP	TGTGCACAGTGTACACCA	321	84	0	0	0	0
IAPP	TGTGCACACAACGTTGGT	403	474	1	1	0	1
IARS	TGACACGTACCATGTGTG	704	255	0	0	1	0
IARS	TGACACGTACACGTGTGT	138	115	0	0	0	0
IARS	GTACACGTACACGTGTCA	251	420	1	0	0	0
IARS	ACACACGTACACGTGTAC	93	84	0	0	0	0
IARS	ACTGCACAACTGTGTGAC	57	30	0	0	0	0
IARS	CATGCACAACTGTGTGTG	124	74	4	0	0	0
IARS2	ACACACGTACGTACTGAC	15	32	0	0	0	0
IARS2	CAACACGTACGTACTGTG	133	146	0	0	0	0
IARS2	TGACACGTACGTTGGTGT	265	258	0	0	0	0
IARS2	GTACACGTACGTTGGTCA	474	48	0	0	0	0
IARS2	ACACACGTACGTTGGTAC	31	41	1	0	0	2
IBSP	CATGGTTGCAACTGTGTG	758	597	1	1	1	1
IBSP	GTTGGTTGCATGGTGTGT	1455	507	0	0	1	2
IBSP	ACTGCACATGTGCAGTCA	206	198	0	1	1	0
IBSP	ACTGGTCACATGGTTGCA	305	126	4	1	0	10
IBSP	CATGGTTGCATGGTGTCA	387	327	0	0	0	1
IBSP	TGTGGTTGCATGGTGTAC	99	248	0	0	0	0
ICAM1	CAACCAGTTGCAACCACA	126	66	1	0	1	0
ICAM1	ACACCAGTTGCAACGTAC	155	29	0	0	0	17
ICAM1	CAACCAGTTGCAACGTTG	436	467	0	0	0	0
ICAM1	GTACCAGTTGCAACCAGT	114	261	0	0	0	0
ICAM1	TGACCAGTTGCAACCAAC	68	91	0	1	0	0
ICAM1	ACTGCACACAACACTGCA	144	185	0	0	0	0
ICAM2	CAACTGGTGTTGACCATG	131	59	2	0	0	0
ICAM2	GTACTGGTGTTGACACGT	145	114	0	0	0	0
ICAM2	CAACTGGTGTTGACACCA	152	182	0	0	0	19
ICAM2	TGACTGGTGTTGACACAC	94	73	0	0	0	0
ICAM2	TGTGACACTGGTACACAC	123	70	0	0	0	0
ICAM5	TGACTGGTGTTGACCAGT	109	58	0	0	1	0
ICAM5	CAACTGGTGTTGACGTAC	33	67	0	0	0	0
ICAM5	GTACTGGTGTTGACGTTG	501	391	3	0	0	1
ICAM5	GTACTGGTGTTGACCACA	45	84	0	0	0	0
ICAM5	ACACTGGTGTTGACCAAC	27	31	0	0	0	0

ICAM5	TGTGCACATGACTGGTCA	367	208	1	1	0	0
ICOS	GTACACTGACCACAGTCA	1013	668	594	4	0	1
ICOS	ACACACTGACCACAGTAC	110	143	0	0	0	0
ICOS	CAACACTGACCACAGTTG	481	509	1	1	0	0
ICOS	GTACACTGACCACACAGT	218	200	0	0	0	0
ICOS	CAACACTGACCACACACA	128	95	0	0	0	0
ICOS	GTTGCATGCACATGACCA	178	224	0	2	0	0
ICOSLG	ACACACTGACACCAACAC	36	53	0	0	1	0
ICOSLG	GTACACTGACACCATGGT	528	695	1	4	1	12
ICOSLG	GTACACTGACACCAACCA	667	376	1	0	0	1
ICOSLG	CAACACTGACACCAACTG	314	234	0	0	0	8
ICOSLG	CATGACGTTGACTGGTCA	420	274	0	0	0	1
ID1	CAACGTACTGCACATGAC	79	29	0	0	0	0
ID1	GTACGTACTGCACATGTG	1168	1444	4	0	0	1
ID1	ACACGTACTGCAACGTGT	271	945	0	1	0	0
ID1	TGACGTACTGCAACGTCA	138	173	0	0	0	0
ID1	CAACGTACTGCAACGTAC	54	76	0	0	0	0
ID1	ACTGCATGGTTGACACGT	110	70	1	0	0	0
ID2	GTACGTTGCATGTGACTG	72	49	0	0	0	0
ID2	TGACGTTGCATGTGTGGT	606	181	1	0	0	0
ID2	GTACGTTGCATGTGTGCA	653	481	0	0	0	0
ID2	ACACGTTGCATGTGTGAC	66	179	0	0	0	0
ID2	CAACGTTGCATGTGTGTG	171	169	0	0	0	0
ID3	GTACGTACACCAACCAGT	148	148	0	0	1	0
ID3	CAACGTACACCACAACAC	132	52	0	0	0	0
ID3	GTACGTACACCACAACTG	689	844	4	0	0	0
ID3	TGACGTACACCACATGGT	280	360	0	0	0	0
ID3	CAACGTACACCAACGTTG	185	293	0	0	0	0
ID3	ACTGCATGCACAGTTGAC	38	46	0	0	0	2
ID4	ACACGTACACCAACTGGT	547	661	15	3	35	22
ID4	TGACGTACACCAACACTG	25	29	0	0	1	0
ID4	ACACGTACACCAACACCA	130	215	0	0	0	0
ID4	GTACGTACACCAACACAC	60	56	0	0	0	0
ID4	TGACGTACACCAACTGCA	208	184	1	0	0	0
ID4	CATGCAACCACAGTTGAC	89	158	1	0	0	0
IDE	ACCATGCACACAACCATG	207	241	0	0	0	0
IDE	CACATGCACACAACACGT	24	21	0	0	0	0
IDE	ACCATGCACACAACACCA	141	307	0	0	0	0
IDE	GTCATGCACACAACACAC	178	106	0	0	0	0
IDE	TGTGCAGTGTCACAACTG	344	257	0	0	0	0
IDE	ACTGCACACATGACCATG	127	170	0	0	0	0
IDH1	CAACGTTGTGTGACACCA	456	389	0	0	0	5
IDH1	TGACGTTGTGTGACACAC	12	21	0	0	0	0
IDH1	ACACGTTGTGTGACACTG	807	199	1	0	0	1
IDH1	CAACGTTGTGTGACTGGT	596	316	1	0	0	0
IDH1	ACACGTTGTGTGACTGCA	111	126	0	0	0	0
IDH1	CATGCATGGTGTTGACTG	146	107	0	0	0	0
IDH2	TGACGTTGTGTGACCAGT	21	17	0	0	0	0
IDH2	GTACGTTGTGTGACCACA	73	74	0	0	0	20
IDH2	ACACGTTGTGTGACCAAC	3	15	0	0	0	0
IDH2	CAACGTTGTGTGACCATG	231	170	1	0	0	1
IDH2	GTACGTTGTGTGACACGT	227	286	0	0	0	0
IDH2	GTTGCATGGTGTTGTGGT	370	391	2	2	0	4
IDH3A	TGACGTTGTGTGCATGCA	82	185	0	0	1	0
IDH3A	TGACGTTGTGTGCAACTG	128	212	1	0	0	0
IDH3A	ACACGTTGTGTGCATGGT	73	75	0	0	0	0
IDH3A	CAACGTTGTGTGCATGAC	42	57	0	0	0	0
IDH3A	GTACCAGTGTGTGTTGCA	58	86	0	0	0	0
IDH3A	CATGCATGGTGTTGTGCA	55	64	0	0	0	0
IDH3B	TGACGTTGTGTGCAGTGT	470	484	0	0	0	0
IDH3B	GTACGTTGTGTGCAGTCA	459	309	0	1	0	1
IDH3B	ACACGTTGTGTGCAGTAC	64	56	0	0	0	0
IDH3B	CAACGTTGTGTGCAGTTG	320	426	0	0	0	31
IDH3B	GTACGTTGTGTGCACAGT	46	73	0	0	0	0
IDH3B	ACTGCATGGTGTTGACAC	5	5	0	0	0	0
IDH3G	GTACGTTGTGTGGTACTG	574	287	5	0	1	0
IDH3G	TGACGTTGTGTGGTCATG	103	147	0	1	0	0
IDH3G	ACACGTTGTGTGGTACGT	781	205	2	0	0	0
IDH3G	TGACGTTGTGTGGTACCA	91	376	0	0	0	0
IDH3G	CAACGTTGTGTGGTACAC	35	40	0	0	0	0
IDH3G	TGTGCATGGTGTTGTGAC	88	90	0	0	0	1
IDI1	ACACACCAACACACACTG	202	248	0	3	1	0
IDI1	CAACACCAACACACCATG	176	176	0	0	0	16
IDI1	GTACACCAACACACACGT	83	103	0	0	0	1
IDI1	CAACACCAACACACACCA	159	161	1	0	0	0

IDI1	TGACACCAACACACACAC	48	61	0	0	0	0
IDI2	TGACACCAACTGACGTGT	539	614	0	0	0	0
IDI2	GTACACCAACTGACGTCA	73	42	0	0	0	0
IDI2	ACACACCAACTGACGTAC	51	32	0	0	0	0
IDI2	CAACACCAACTGACGTTG	194	222	0	0	0	0
IDI2	GTACACCAACTGACCAGT	504	641	1	1	0	0
IDI2	ACTGCAACACGTACCACA	32	64	0	0	0	0
IDS	ACACACACCATGTGGTCA	541	325	15	2	18	17
IDS	ACACACACCATGTGCAGT	364	392	4	3330	1	4
IDS	CAACACACCATGTGGTGT	306	656	0	2	0	2
IDS	GTACACACCATGTGGTAC	16	10	0	0	0	0
IDS	TGACACACCATGTGGTTG	577	637	0	1	0	7
IDUA	ACACCAGTACCAACCACA	146	157	0	0	1	0
IDUA	CAACCAGTACCAACCAGT	339	448	1	1	0	0
IDUA	GTACCAGTACCAACCAAC	45	17	0	0	0	0
IDUA	TGACCAGTACCAACCATG	218	179	0	0	0	0
IDUA	ACACCAGTACCAACACGT	11	14	0	0	0	0
IFI16	ACACGTACTGCATGGTCA	142	193	0	0	1	0
IFI16	ACACGTACTGCATGCAGT	406	471	0	0	1	0
IFI16	CAACGTACTGCATGGTGT	454	410	3	0	0	1
IFI16	GTACGTACTGCATGGTAC	42	32	0	0	0	0
IFI16	TGACGTACTGCATGGTTG	762	1464	2	0	0	0
IFI16	ACTGCACAACGTGTACAC	27	34	0	0	0	0
IFI27	ACTGGTGTACCATGTGTG	628	651	1	1	0	0
IFI27	ACTGGTGTACACGTGTGT	820	607	0	0	0	0
IFI27	TGTGGTGTACACGTGTCA	197	174	0	193	0	0
IFI27	CATGGTGTACACGTGTAC	144	136	1	0	0	1
IFI27	GTTGGTGTACACGTGTTG	781	303	2	0	0	5
IFI30	ACACCAGTACACCACATG	117	134	0	0	0	0
IFI30	CAACCAGTACACCAACGT	140	174	0	0	0	2
IFI30	ACACCAGTACACCAACCA	426	814	0	1	0	34
IFI30	GTACCAGTACACCAACAC	39	18	1	0	0	0
IFI30	TGACCAGTACACCAACTG	315	322	0	0	0	0
IFI30	GTTGCATGGTACACCAGT	172	327	0	0	0	0
IFI35	ACTGGTACCAGTGTACAC	136	40	0	0	1	0
IFI35	CATGGTACCAGTGTACTG	382	216	0	0	0	1
IFI35	GTTGGTACCAGTGTTGGT	450	479	1	0	0	0
IFI35	CATGGTACCAGTGTTGCA	1072	648	1	0	0	2
IFI35	TGTGGTACCAGTGTTGAC	66	86	0	0	0	0
IFI44	GTTGGTGTTGCAACACCA	24	17	0	0	0	0
IFI44	ACTGGTGTTGCAACACAC	15	29	0	0	0	0
IFI44	CATGGTGTTGCAACACTG	397	421	1	19	0	0
IFI44	GTTGGTGTTGCAACTGGT	120	129	0	1	0	0
IFI44	CATGGTGTTGCAACTGCA	220	383	1	0	0	0
IFIH1	ACACACACGTTGGTCATG	562	570	12	16	2	7
IFIH1	CAACACACGTTGGTGTTG	231	326	0	1	0	0
IFIH1	GTACACACGTTGGTCAGT	319	336	0	0	0	0
IFIH1	CAACACACGTTGGTCACA	78	92	0	0	0	0
IFIH1	TGACACACGTTGGTCAAC	50	12	0	0	0	0
IFIH1	TGTGCATGGTGTACGTCA	80	63	0	0	0	0
IFIT1	ACTGGTTGCATGGTGTTG	1216	999	0	1	1	1
IFIT1	CATGGTACACTGGTCAAC	115	77	0	0	0	1
IFIT1	GTTGGTACACTGGTCATG	21	78	1	0	0	0
IFIT1	TGTGGTACACTGGTACGT	44	64	0	0	0	0
IFIT1	GTTGGTACACTGGTACCA	251	42	0	0	0	0
IFIT2	CATGGTCAACACACACTG	569	851	0	0	0	1
IFIT2	GTTGGTCAACACACTGGT	91	95	0	0	0	0
IFIT2	CATGGTCAACACACTGCA	321	213	0	0	0	0
IFIT2	TGTGGTCAACACACTGAC	46	23	0	0	0	0
IFIT2	ACTGACACTGGTACTGCA	166	176	0	0	0	0
IFIT3	TGTGGTGTACACGTCAGT	377	369	0	0	0	0
IFIT3	GTTGGTGTACACGTCACA	166	179	0	0	0	0
IFIT3	ACTGGTGTACACGTCAAC	6	13	0	0	0	0
IFIT3	CATGGTGTACACGTCATG	83	81	0	0	0	0
IFIT3	GTTGGTGTACACGTACGT	126	108	0	0	0	0
IFITM1	GTACACTGTGTGTGGTCA	79	63	0	0	0	0
IFITM1	ACACACTGTGTGTGGTAC	66	287	1	0	0	0
IFITM1	CAACACTGTGTGTGGTTG	576	544	0	1	0	0
IFITM1	GTACACTGTGTGTGCAGT	190	395	1	0	0	0
IFITM1	CAACACTGTGTGTGCACA	312	141	0	0	0	0
IFITM3	ACACTGACTGCAACACCA	522	618	0	2	5	2
IFITM3	CATGGTCAGTGTGTGTTG	713	1137	2	0	0	1
IFITM3	GTTGGTCAGTGTGTCAGT	238	249	0	0	0	0
IFITM3	CATGGTCAGTGTGTCACA	360	269	0	0	0	1
IFITM3	TGTGGTCAGTGTGTCAAC	32	40	0	0	0	0

IFNA1	ACCATGTGTGGTCATGAC	47	61	0	0	1	0
IFNA1	CACATGTGTGGTCATGTG	236	516	0	1	0	0
IFNA1	TGCATGTGTGGTACGTGT	346	275	0	0	0	0
IFNA1	GTCATGTGTGGTACGTCA	178	164	0	0	0	1
IFNA1	TGCATGTGTGGTACTGCA	134	123	0	0	0	0
IFNA1	GTTGCACATGTGGTACGT	117	157	0	0	0	0
IFNA10	CACATGTGACTGTGACTG	961	839	1	2	0	0
IFNA10	CACATGTGACTGTGTGCA	208	215	0	0	0	0
IFNA10	TGCATGTGACTGTGTGAC	38	120	0	0	0	0
IFNA10	ACCATGTGACTGTGTGTG	359	362	0	0	0	0
IFNA10	CACATGTGTGGTGTGTGT	43	64	0	0	0	0
IFNA13	CACATGTGTGGTACTGAC	19	16	0	0	0	2
IFNA13	GTCATGTGTGGTACTGTG	313	300	0	1	0	1
IFNA13	ACCATGTGTGGTTGGTGT	524	494	0	1	0	0
IFNA13	TGCATGTGTGGTTGGTCA	156	173	0	0	0	3
IFNA13	CATGCACATGTGGTACCA	284	353	2	1	0	2
IFNA14	ACCATGTGACTGTGACAC	28	34	8	0	0	0
IFNA14	ACCATGTGTGGTGTGTCA	246	224	0	0	0	0
IFNA14	GTCATGTGTGGTGTGTAC	129	149	0	0	0	0
IFNA14	TGCATGTGTGGTGTGTTG	292	239	0	0	0	0
IFNA14	ACCATGTGTGGTGTCAGT	64	70	0	0	0	0
IFNA16	GTCATGTGTGGTGTTGGT	475	685	0	0	0	0
IFNA16	CACATGTGTGGTGTTGCA	229	48	1	0	0	1
IFNA16	TGCATGTGTGGTGTTGAC	57	25	0	0	0	0
IFNA16	ACCATGTGTGGTGTTGTG	86	81	0	0	0	0
IFNA16	CATGACACACCAACCATG	202	176	2	1	0	0
IFNA17	GTCATGTGTGGTGTACCA	186	220	1	0	0	0
IFNA17	CACATGTGTGGTCAGTCA	141	223	0	0	0	0
IFNA17	GTTGCACATGCAACTGGT	79	114	0	0	0	0
IFNA17	CATGCACATGCAACTGCA	700	465	0	0	0	3
IFNA17	TGTGCACATGCAACTGAC	7	5	0	0	0	0
IFNA17	ACTGCACATGCAACTGTG	994	679	3	1	0	1
IFNA2	ACCATGTGTGGTACGTAC	7	15	0	0	0	0
IFNA2	CACATGTGTGGTACGTTG	104	265	0	0	0	0
IFNA2	GTCATGTGTGGTACCAGT	175	77	0	0	0	0
IFNA2	TGCATGTGTGGTACCAAC	48	123	0	0	0	0
IFNA2	ACCATGTGTGGTACCATG	104	152	0	0	0	0
IFNA2	ACTGCACATGCATGTGAC	20	10	0	0	0	0
IFNA21	TGCATGTGTGGTGTACGT	427	323	0	0	1	0
IFNA21	TGCATGTGACTGTGACGT	640	700	0	1	0	0
IFNA21	GTCATGTGACTGTGACCA	191	165	1	0	0	0
IFNA21	GTCATGTGACTGTGTGGT	1207	359	0	1	0	0
IFNA21	CACATGTGTGGTGTACTG	429	310	1	0	0	0
IFNA21	TGTGCACAGTTGCACAAC	6	15	0	0	0	0
IFNA4	ACCATGTGTGGTGTACAC	29	21	0	0	0	0
IFNA4	GTCATGTGTGGTCAGTGT	1257	977	3	0	0	0
IFNA4	ACCATGTGTGGTCAGTTG	224	223	0	0	0	0
IFNA4	CACATGTGTGGTCACAGT	352	221	1	0	0	3
IFNA4	CACATGTGTGGTACCACA	26	19	0	0	0	0
IFNA5	ACTGCACAGTTGCACATG	791	986	10	1	2	5
IFNA5	TGCATGTGTGGTCAGTAC	62	48	1	0	0	0
IFNA5	ACCATGTGTGGTCACACA	45	139	0	0	0	0
IFNA5	GTCATGTGTGGTCACAAC	29	50	0	0	0	0
IFNA5	TGCATGTGTGGTCACATG	334	376	1	0	0	0
IFNA5	ACCATGTGTGGTCAACGT	295	305	0	0	0	0
IFNA6	GTCATGTGTGGTCAACTG	847	1043	0	1	1	25
IFNA6	TGCATGTGTGGTCAACCA	105	269	0	0	0	0
IFNA6	CACATGTGTGGTCAACAC	69	46	0	0	0	0
IFNA6	TGCATGTGTGGTCATGGT	241	191	0	0	0	0
IFNA6	GTCATGTGTGGTCATGCA	512	176	0	1	0	2
IFNA6	GTTGCACATGACGTGTAC	21	36	0	0	0	0
IFNA7	TGCATGTGTGGTGTCACA	60	150	0	0	0	0
IFNA7	CACATGTGTGGTGTCAAC	10	14	0	0	0	0
IFNA7	GTCATGTGTGGTGTCATG	500	253	2	0	0	0
IFNA7	TGTGACCACACACACATG	147	348	0	0	0	0
IFNA7	ACTGACCACACACAACGT	1203	920	2	0	0	1
IFNA8	CACATGTGTGGTACACGT	41	27	0	0	0	0
IFNA8	ACCATGTGTGGTACACCA	118	109	1	0	0	0
IFNA8	GTCATGTGTGGTACACAC	2	3	0	0	0	0
IFNA8	TGCATGTGTGGTACACTG	121	156	0	0	0	0
IFNA8	ACCATGTGTGGTACTGGT	100	147	0	0	0	0
IFNAR1	TGACTGCAGTGTACGTTG	581	515	1	0	0	1
IFNAR1	ACACTGCAGTGTACCAGT	51	36	0	0	0	0
IFNAR1	TGACTGCAGTGTACCACA	95	66	0	0	0	0
IFNAR1	CAACTGCAGTGTACCAAC	33	20	0	0	0	0

IFNAR1	GTACTGCAGTGTACCATG	94	48	0	0	0	0
IFNAR2	GTTGCAGTGTACGTACGT	301	326	24	13710	1515	4
IFNAR2	ACACTGGTTGACGTCAGT	23	23	0	0	0	0
IFNAR2	TGACTGGTTGACGTCACA	98	121	0	0	0	1
IFNAR2	CAACTGGTTGACGTCAAC	74	45	0	0	0	0
IFNAR2	GTACTGGTTGACGTCATG	129	142	1	0	0	2
IFNB1	TGCATGTGACTGTGGTTG	209	112	0	0	1	0
IFNB1	ACCATGTGACTGTGCAGT	184	148	0	0	0	1
IFNB1	TGCATGTGACTGTGCACA	93	80	0	0	0	0
IFNB1	CACATGTGACTGTGCAAC	16	24	0	0	0	0
IFNB1	GTCATGTGACTGTGCATG	388	60	1	0	0	0
IFNE1	CACATGTGTGGTTGGTAC	61	76	0	0	0	0
IFNE1	GTCATGTGTGGTTGGTTG	560	260	0	0	0	0
IFNE1	TGCATGTGTGGTTGCAGT	352	187	0	0	0	0
IFNE1	GTCATGTGTGGTTGCACA	112	209	0	0	0	3
IFNE1	ACCATGTGTGGTTGCAAC	11	16	0	0	0	0
IFNG	ACACTGGTACACTGGTAC	37	47	0	0	0	0
IFNG	CAACTGGTACACTGGTTG	926	902	0	0	0	3
IFNG	GTACTGGTACACTGCAGT	103	125	0	0	0	0
IFNG	CAACTGGTACACTGCACA	381	311	0	0	0	0
IFNG	CATGACGTTGCAACGTTG	953	1004	0	0	0	0
IFNGR1	TGACTGGTTGACGTACGT	171	148	0	0	0	0
IFNGR1	GTACTGGTTGACGTACCA	34	40	0	0	0	0
IFNGR1	ACACTGGTTGACGTACAC	44	73	0	0	0	0
IFNGR1	CAACTGGTTGACGTACTG	117	176	0	0	0	0
IFNGR1	ACTGCAGTCACAACGTAC	15	27	0	0	0	0
IFNGR1	GTTGCACACAACACTGAC	84	77	0	0	0	0
IFNGR2	CAACTGCAGTCAGTTGGT	225	300	1	0	1	1
IFNGR2	CAACTGCAGTCAGTACCA	569	636	1	0	0	0
IFNGR2	TGACTGCAGTCAGTACAC	14	31	0	0	0	0
IFNGR2	ACACTGCAGTCAGTACTG	908	658	0	0	0	4
IFNGR2	ACTGCAGTTGACTGCAGT	19	36	0	0	0	0
IFNK	GTACTGGTTGGTACTGAC	32	32	0	0	0	9
IFNK	TGACTGGTTGGTACTGTG	150	232	1	1	0	0
IFNK	CAACTGGTTGGTTGGTGT	100	114	0	0	0	0
IFNK	ACACTGGTTGGTTGGTCA	291	282	0	0	0	12
IFNK	GTACTGGTTGGTTGGTAC	111	249	0	0	0	0
IFRD1	ACTGGTACGTCATGCACA	344	426	63	272	115	1
IFRD1	ACTGGTACGTCATGGTTG	467	862	2	0	1	0
IFRD1	CATGGTACGTCATGGTCA	604	310	0	0	0	1
IFRD1	TGTGGTACGTCATGGTAC	89	96	0	0	0	0
IFRD1	CATGGTACGTCATGCAGT	508	114	0	0	0	0
IFT172	ACACTGTGACACCATGAC	36	39	0	0	0	0
IFT172	TGTGGTCAACGTCAGTTG	1060	341	1	1	0	1
IFT172	ACTGGTCAACGTCACAGT	82	288	0	2	0	0
IFT172	TGTGGTCAACGTCACACA	100	173	0	0	0	0
IFT172	CATGGTCAACGTCACAAC	34	8	0	0	0	0
IFT57	CAACTGACGTACGTTGCA	11	48	0	0	1	0
IFT57	GTACTGACGTACGTACCA	78	91	0	0	0	0
IFT57	ACACTGACGTACGTACAC	60	53	0	0	0	0
IFT57	CAACTGACGTACGTACTG	190	216	1	0	0	0
IFT57	GTACTGACGTACGTTGGT	36	31	0	0	0	0
IFT57	TGTGCAACCACAACGTTG	799	921	1	0	0	1
IGF1	TGACCATGACGTTGTGCA	102	111	0	0	0	0
IGF1	CAACCATGACGTTGTGAC	29	63	0	0	0	0
IGF1	GTACCATGACGTTGTGTG	72	83	1	0	0	0
IGF1	CAACCATGACCAGTGTGT	150	202	0	0	0	1
IGF1	ACACCATGACCAGTGTCA	92	99	0	0	0	0
IGF1	ACTGCAACCACAGTTGGT	119	220	0	0	0	0
IGF1R	TGCATGCACATGGTTGGT	512	229	0	0	0	5
IGF1R	GTCATGCACATGGTTGCA	71	73	0	0	0	0
IGF1R	ACCATGCACATGGTTGAC	42	38	0	0	0	0
IGF1R	CACATGCACATGGTTGTG	113	114	0	0	0	0
IGF1R	TGCATGCACATGCAGTGT	364	422	0	1	0	0
IGF1R	CATGCACATGCATGTGTG	46	38	0	0	0	0
IGF2	CAACTGACGTGTCAGTTG	654	308	0	0	0	1
IGF2	GTACTGACGTGTCACAGT	65	98	0	0	0	0
IGF2	CAACTGACGTGTCACACA	197	120	0	0	0	0
IGF2	TGACTGACGTGTCACAAC	25	15	0	0	0	0
IGF2	TGACTGACTGACGTCAAC	7	14	0	0	0	0
IGF2	GTTGCACAACACGTCACA	107	46	0	0	0	1
IGF2R	GTACTGCACATGCATGGT	86	63	1	0	0	0
IGF2R	CAACTGCACATGCATGCA	280	238	0	1	0	3
IGF2R	TGACTGCACATGCATGAC	40	38	0	0	0	0
IGF2R	ACACTGCACATGCATGTG	228	201	0	0	0	0

IGF2R	GTACTGCACATGACGTGT	391	328	1	0	0	3
IGFBP1	GTACTGTGGTCACACAGT	124	49	1	0	0	0
IGFBP1	CAACTGTGGTCACACACA	135	193	0	0	0	0
IGFBP1	TGACTGTGGTCACACAAC	12	71	0	0	0	0
IGFBP1	ACACTGTGGTCACACATG	175	227	0	1	0	0
IGFBP1	CATGGTACTGGTACACAC	21	13	0	0	0	0
IGFBP2	ACACGTGTGTTGCACAAC	15	18	0	0	0	0
IGFBP2	CAACGTGTGTTGCACATG	117	52	1	0	0	0
IGFBP2	GTACGTGTGTTGCAACGT	389	159	0	1	0	0
IGFBP2	CAACGTGTGTTGCAACCA	122	186	0	0	0	1
IGFBP2	GTTGCAGTACGTGTTGCA	53	45	1	0	0	0
IGFBP3	CAACTGACTGACTGACAC	187	95	0	0	0	0
IGFBP3	GTACTGACTGACTGACTG	243	257	1	0	0	0
IGFBP3	TGACTGACTGACTGTGGT	262	598	1	0	0	2
IGFBP3	GTACTGACTGACTGTGCA	355	390	0	1	0	1
IGFBP3	TGTGCAGTCAGTTGCAAC	13	16	0	0	0	0
IGFBP4	TGACTGACTGACTGGTAC	190	34	0	0	0	0
IGFBP4	ACACTGACTGACTGGTTG	618	473	1	0	0	0
IGFBP4	CAACTGACTGACTGCAGT	220	237	1	1	0	1
IGFBP4	TGTGCAGTTGTGACACTG	234	201	1	0	0	1
IGFBP4	ACTGCAGTTGTGACTGGT	144	245	0	0	0	0
IGFBP5	TGACTGCATGTGGTTGCA	181	198	0	0	1	0
IGFBP5	TGACTGCATGTGGTACTG	609	902	0	0	0	0
IGFBP5	ACACTGCATGTGGTTGGT	165	203	1	0	0	0
IGFBP5	CAACTGCATGTGGTTGAC	113	32	0	0	0	0
IGFBP5	GTACTGCATGTGGTTGTG	109	226	0	0	0	0
IGFBP6	CATGCAGTCAACACACTG	947	1030	2	3	1	1
IGFBP6	TGACTGACGTGTGTACCA	78	63	0	0	0	0
IGFBP6	CAACTGACGTGTGTACAC	48	39	0	0	0	0
IGFBP6	GTACTGACGTGTGTACTG	128	131	0	0	0	0
IGFBP6	TGACTGACGTGTGTTGGT	54	52	0	0	0	0
IGFBP7	ACACTGTGCACATGACTG	314	330	0	1	0	26
IGFBP7	CAACTGTGCACATGTGGT	171	195	1	0	0	0
IGFBP7	ACACTGTGCACATGTGCA	400	492	0	0	0	0
IGFBP7	GTACTGTGCACATGTGAC	19	3	0	0	0	0
IGFBP7	CAACTGTGACACCATGTG	224	368	0	0	0	0
IGFBP7	CATGCACAGTTGCAACGT	1724	1259	1	1	0	1
IHH	CAACCACAGTGTACACGT	36	27	0	0	0	0
IHH	ACACCACAGTGTACACCA	820	363	1	0	0	3
IHH	GTACCACAGTGTACACAC	67	61	0	0	0	0
IHH	TGACCACAGTGTACACTG	418	465	1	1	0	1
IHH	ACACCACAGTGTACTGGT	345	393	0	0	0	0
IKBKAP	CAACCAACTGCAGTGTTG	163	286	0	0	0	0
IKBKAP	GTACCAACTGCAGTCAGT	99	150	0	0	0	0
IKBKAP	CAACCAACTGCAGTCACA	111	107	1	0	0	0
IKBKAP	TGACCAACTGCAGTCAAC	49	28	0	1	0	0
IKBKAP	ACACCAACTGCAGTCATG	69	54	0	0	0	0
IKBKB	CAACGTACTGGTGTACTG	390	311	1	0	0	0
IKBKB	GTACGTACTGGTGTTGGT	609	417	1	0	0	0
IKBKB	CAACGTACTGGTGTTGCA	58	32	0	0	0	1
IKBKB	TGACGTACTGGTGTTGAC	75	57	0	0	0	0
IKBKB	ACACGTACTGGTGTTGTG	443	526	0	0	0	1
IKBKE	CATGGTTGTGCAGTTGGT	356	390	0	0	0	0
IKBKE	ACTGGTTGTGCAGTTGCA	281	95	0	0	0	0
IKBKE	GTTGGTTGTGCAGTTGAC	33	40	0	0	0	0
IKBKE	TGTGGTTGTGCAGTTGTG	511	373	2	0	0	3
IKBKE	CATGGTTGTGCACAGTGT	376	271	9	0	0	1
IKBKG	CAACGTTGTGGTACGTCA	165	129	1	0	0	0
IKBKG	TGACGTTGTGGTACGTAC	69	90	0	0	0	0
IKBKG	ACACGTTGTGGTACGTTG	491	295	0	2	0	0
IKBKG	CAACGTTGTGGTACCAGT	253	199	0	0	0	0
IKBKG	ACACGTTGTGGTACCACA	88	97	0	0	0	0
IKZF1	CATGGTGTCATGTGACCA	205	369	0	1	1	0
IKZF1	GTTGGTGTCATGTGACGT	314	241	0	0	0	1
IKZF1	TGTGGTGTCATGTGACAC	16	8	0	0	0	0
IKZF1	ACTGGTGTCATGTGACTG	493	431	0	0	0	0
IKZF1	CATGGTGTCATGTGTGGT	614	513	1	1	0	0
IKZF2	ACACGTTGACTGTGCAAC	28	21	0	0	0	0
IKZF2	CAACGTTGACTGTGCATG	282	234	161	0	0	0
IKZF2	GTACGTTGACTGTGACGT	233	144	0	0	0	1
IKZF2	CAACGTTGACTGTGACCA	215	210	1	0	0	0
IKZF2	TGACGTTGACTGTGACAC	20	12	0	0	0	0
IKZF3	ACACGTACACACGTTGGT	130	215	0	0	0	0
IKZF3	TGACGTACACACGTTGCA	277	360	0	1	0	0
IKZF3	CAACGTACACACGTTGAC	6	10	0	0	0	0

IKZF3	TGTGCAGTACACACTGCA	150	142	0	0	0	1
IKZF3	TGTGCATGACTGCACATG	232	96	0	0	0	0
IL10	TGACTGGTACACCAACGT	633	709	0	0	1	6
IL10	CAACTGGTACACCACAAC	29	23	0	1	0	0
IL10	GTACTGGTACACCACATG	75	71	0	0	0	0
IL10	GTACTGGTACACCAACCA	255	470	2	1	0	1
IL10	ACACTGGTACACCAACAC	152	121	0	0	0	0
IL10	CATGCAACTGCACATGAC	25	39	0	0	0	0
IL10RA	CAACTGGTTGCACAGTAC	143	218	1	0	0	0
IL10RA	GTACTGGTTGCACAGTTG	354	462	3	0	0	0
IL10RA	TGACTGGTTGCACACAGT	143	97	0	0	0	0
IL10RA	GTACTGGTTGCACACACA	114	132	0	0	0	0
IL10RA	ACACTGGTTGCACACAAC	7	12	0	0	0	0
IL10RA	ACTGCAACTGCACATGGT	150	258	0	0	0	0
IL10RB	GTACTGGTACGTGTTGGT	136	187	2	0	0	0
IL10RB	CAACTGGTACGTGTTGCA	45	61	0	0	0	0
IL10RB	TGACTGGTACGTGTTGAC	36	92	1	0	0	0
IL10RB	ACACTGGTACGTGTTGTG	426	326	1	0	0	0
IL10RB	ACACTGACTGCATGGTGT	771	476	1	0	0	0
IL10RB	TGTGCAACTGCACATGCA	9	19	0	0	0	0
IL11	GTTGCAACTGCACATGTG	1226	1831	0	2	1	0
IL11	TGACTGCAGTGTGTACTG	214	221	0	0	0	0
IL11	ACACTGCAGTGTGTTGGT	144	123	0	0	0	0
IL11	ACTGCAGTTGACACACTG	136	148	0	0	0	0
IL11	ACTGCAACTGCAACGTGT	141	216	0	0	0	1
IL11	TGTGCAACTGCAACGTCA	139	116	0	0	0	0
IL11RA	ACACTGGTTGTGACCACA	5	4	0	0	0	0
IL11RA	GTACTGGTTGTGACCAAC	18	24	0	0	0	0
IL11RA	TGACTGGTTGTGACCATG	133	156	0	0	0	0
IL11RA	ACACTGGTTGTGACACGT	168	63	0	0	0	0
IL11RA	TGACTGGTTGTGACACCA	401	519	0	1	0	0
IL11RA	GTTGCAACACACTGCAAC	11	18	0	0	0	0
IL12A	GTACTGGTTGCATGTGCA	127	106	0	0	1	0
IL12A	CAACTGGTTGCATGACAC	135	52	0	0	0	0
IL12A	GTACTGGTTGCATGACTG	412	464	2	0	0	0
IL12A	TGACTGGTTGCATGTGGT	707	547	1	0	0	4
IL12A	ACACTGGTTGCATGTGAC	36	52	0	0	0	0
IL12A	CATGCAACTGCAACGTAC	36	52	0	0	0	0
IL12B	CAACTGGTTGCATGGTCA	149	115	5	0	1	8
IL12B	GTACTGGTTGCATGGTGT	1643	1640	2	2	0	1
IL12B	TGACTGGTTGCATGGTAC	174	215	0	0	0	0
IL12B	ACACTGGTTGCATGGTTG	209	298	0	0	0	0
IL12B	CAACTGGTTGCATGCAGT	162	223	0	0	0	0
IL12B	GTTGCAACTGCATGGTAC	76	109	0	1	0	0
IL12RB1	ACTGGTTGACACCACACA	291	106	0	0	1	0
IL12RB1	CAACTGGTACACGTCATG	454	474	0	0	0	1
IL12RB1	GTACTGGTACACGTACGT	198	198	1	0	0	0
IL12RB1	CATGCAGTTGTGACACGT	211	148	0	0	0	0
IL12RB1	TGTGCAACTGCATGGTTG	106	410	0	0	0	0
IL12RB2	TGACTGGTCATGGTGTTG	639	539	0	1	1	0
IL12RB2	GTACTGGTCATGGTGTAC	93	107	0	0	0	0
IL12RB2	ACACTGGTCATGGTCAGT	206	664	0	0	0	1
IL12RB2	TGACTGGTCATGGTCACA	71	81	0	0	0	0
IL12RB2	CAACTGGTCATGGTCAAC	33	56	0	0	0	0
IL12RB2	TGTGCAACTGCATGCACA	11	20	0	0	0	0
IL13	TGACTGGTACTGTGGTAC	37	158	0	0	0	0
IL13	ACACTGGTACTGTGGTTG	583	488	3	0	0	1
IL13	CAACTGGTACTGTGCAGT	116	69	0	0	0	0
IL13	ACACTGGTACTGTGCACA	77	84	0	0	0	0
IL13	GTACTGGTACTGTGCAAC	29	17	0	0	0	0
IL13	CATGCAACTGTGCACATG	317	254	0	0	0	0
IL13RA1	ACACTGGTCATGCATGAC	143	225	1	0	0	0
IL13RA1	CAACTGGTCATGCATGTG	469	424	1	0	0	0
IL13RA1	TGACTGGTCATGACGTGT	876	402	0	1	0	6
IL13RA1	GTACTGGTCATGACGTCA	448	368	0	1	0	0
IL13RA1	ACACTGGTCATGACGTAC	101	104	1	0	0	1
IL13RA1	GTTGCAACTGTGCACACA	155	165	0	0	0	3
IL13RA2	CAACTGGTACTGGTACGT	253	582	1	0	1	0
IL13RA2	CAACTGGTACTGGTCACA	34	35	0	0	0	0
IL13RA2	TGACTGGTACTGGTCAAC	23	24	0	0	0	0
IL13RA2	ACACTGGTACTGGTCATG	100	79	0	0	0	0
IL13RA2	ACACTGGTACTGGTACCA	95	60	0	0	0	0
IL13RA2	ACTGCAACTGTGCACAAC	33	41	0	0	0	1
IL15	ACTGCAACTGTGCAACTG	2200	1322	29	64	7	69
IL15	ACACTGGTTGTGACGTTG	350	100	0	0	0	1

IL15	CAACTGGTTGTGACCAGT	77	45	0	0	0	7
IL15	GTTGCAGTTGACACACGT	324	272	0	0	0	0
IL15	TGTGCAACTGTGCAACAC	240	35	0	0	0	0
IL15	CATGCAACTGTGCATGGT	344	207	0	1	0	0
IL15RA	GTACTGGTACGTACCAGT	89	87	0	0	0	0
IL15RA	CAACTGGTACGTACCACA	55	23	0	0	0	0
IL15RA	GTTGCAGTTGACGTACTG	280	573	0	0	0	0
IL15RA	TGTGCAGTTGACGTTGGT	187	433	0	0	0	0
IL15RA	GTTGCAACTGTGCAACGT	53	55	0	0	0	0
IL15RA	CATGCAACTGTGCAACCA	707	631	2	1	0	1
IL16	CAACTGCAGTCAACGTCA	721	550	1	2	1	2
IL16	TGACTGCAGTCACATGAC	58	39	0	0	0	0
IL16	ACACTGCAGTCACATGTG	516	521	2	0	0	0
IL16	GTACTGCAGTCAACGTGT	556	473	0	0	0	0
IL16	TGACTGCAGTCAACGTAC	30	107	0	0	0	0
IL17A	GTACACCACATGCAGTTG	741	905	0	1	1	0
IL17A	TGACACCACATGCACAGT	82	103	0	0	0	0
IL17A	GTACACCACATGCACACA	49	93	0	0	0	0
IL17A	ACACACCACATGCACAAC	78	87	0	0	0	0
IL17A	CAACACCACATGCACATG	32	55	0	0	0	0
IL17A	GTTGCAACTGTGTGTGCA	71	135	0	0	0	0
IL17C	GTACTGGTTGGTTGCATG	923	403	24	16409	5	5
IL17C	TGACTGGTTGGTTGGTTG	578	1186	0	0	0	0
IL17C	ACACTGGTTGGTTGCAGT	531	155	2	1	0	0
IL17C	TGACTGGTTGGTTGCACA	115	101	0	0	0	1
IL17C	CAACTGGTTGGTTGCAAC	20	33	0	0	0	0
IL17C	CATGCAACTGTGTGACAC	68	35	0	0	0	0
IL17D	GTACTGGTACACGTTGAC	2	8	0	0	0	0
IL17D	TGACTGGTACACGTTGTG	319	526	0	0	0	0
IL17D	CAACTGGTACACCAGTGT	236	236	0	0	0	0
IL17D	GTTGCAGTTGACACGTTG	65	68	0	0	0	0
IL17D	CATGCAACCACAACCAAC	66	56	0	0	0	0
IL17D	GTTGCAACCACAACCATG	60	35	0	0	0	0
IL17F	CAACTGGTTGGTGTGTCA	221	515	1	0	0	2
IL17F	TGACTGGTTGGTGTGTAC	5	59	0	0	0	0
IL17F	ACACTGGTTGGTGTGTTG	275	222	1	2	0	0
IL17F	CAACTGGTTGGTGTCAGT	35	159	0	0	0	0
IL17F	ACACTGGTTGGTGTCACA	137	152	0	0	0	0
IL17RA	CAACTGCAGTCAACTGTG	208	148	1	0	0	0
IL17RA	TGACTGCAGTCATGGTGT	231	283	0	1	0	2
IL17RA	GTACTGCAGTCATGGTCA	419	344	2	0	0	1
IL17RA	ACACTGCAGTCATGGTAC	58	79	0	0	0	0
IL17RA	CAACTGCAGTCATGGTTG	507	726	1	0	0	0
IL17RB	ACACTGGTTGACCAACGT	1313	1528	189	727	6687	8
IL17RB	TGACTGGTTGACCACATG	112	88	0	0	0	0
IL17RB	TGACTGGTTGACCAACCA	601	357	3	0	0	11
IL17RB	CAACTGGTTGACCAACAC	76	107	0	0	0	0
IL17RB	GTACTGGTTGACCAACTG	153	202	0	0	0	0
IL17RB	TGTGCAACTGTGTGACCA	454	158	0	0	0	0
IL17RC	TGACTGGTTGTGGTGTCA	523	346	0	0	0	0
IL17RC	CAACTGGTTGTGGTGTAC	95	158	28	0	0	0
IL17RC	GTACTGGTTGTGGTGTTG	323	568	1	0	0	0
IL17RC	TGACTGGTTGTGGTCAGT	413	162	0	0	0	0
IL17RC	GTACTGGTTGTGGTCACA	374	249	0	0	0	0
IL17RC	CATGCAACTGCACAACGT	451	506	1	1	0	0
IL17RD	GTACTGGTTGGTCATGTG	188	162	0	0	0	0
IL17RD	ACACTGGTTGGTACGTGT	215	301	1	2	0	6
IL17RD	TGTGCAGTTGACCATGCA	224	221	0	0	0	0
IL17RD	CATGCAGTTGACCATGAC	365	202	0	0	0	0
IL17RD	GTTGCAGTTGACCATGTG	489	985	0	0	0	0
IL17RD	ACTGCAACTGCAGTACGT	71	96	0	0	0	0
IL17RE	ACTGCAACTGCACAACCA	679	670	306	273911	21765	72
IL17RE	ACACTGGTCATGCACACA	46	65	0	0	0	0
IL17RE	GTACTGGTCATGCACAAC	18	8	0	0	0	0
IL17RE	TGACTGGTCATGCACATG	349	620	1	0	0	0
IL17RE	ACACTGGTCATGCAACGT	609	122	0	1	0	0
IL17RE	GTTGCAACTGCACAACAC	113	121	0	0	0	1
IL18	TGACTGGTTGCAACGTTG	620	609	4	7	1	0
IL18	ACACTGGTTGCAACGTCA	186	249	0	1	0	0
IL18	GTACTGGTTGCAACGTAC	94	255	0	1	0	0
IL18	ACACTGGTTGCAACCAGT	313	316	0	0	0	0
IL18	TGACTGGTTGCAACCACA	254	190	0	0	0	1
IL18R1	CACATGTGACTGACGTAC	111	142	0	0	1	0
IL18R1	TGCATGTGACTGACGTCA	221	137	1	0	0	0
IL18R1	GTCATGTGACTGACGTTG	396	461	1	0	0	0

IL18R1	TGCATGTGACTGACCAGT	150	168	0	0	0	0
IL18R1	ACTGCAACTGTGTGTGAC	38	138	0	0	0	0
IL18R1	CATGCAACTGTGTGTGTG	337	319	1	0	0	0
IL19	TGACTGGTTGCAGTTGCA	156	245	1	0	0	8
IL19	CAACTGGTTGCAGTTGAC	45	36	0	0	0	0
IL19	GTACTGGTTGCAGTTGTG	140	117	0	0	0	0
IL19	ACACTGGTTGCACAGTGT	553	739	1	0	0	0
IL19	TGACTGGTTGCACAGTCA	89	87	0	0	0	0
IL19	ACTGCATGGTTGGTCAAC	26	102	0	0	0	0
IL1A	CAACTGCAGTACCAGTTG	180	160	0	0	0	0
IL1A	GTACTGCAGTACCACAGT	428	265	0	0	0	0
IL1A	CAACTGCAGTACCACACA	134	161	0	1	0	0
IL1A	TGACTGCAGTACCACAAC	30	30	0	0	0	0
IL1A	ACACTGCAGTACCACATG	350	221	0	1	0	0
IL1A	CATGCATGGTGTACTGGT	60	45	0	1	0	0
IL1B	TGACTGGTACCAACCAGT	64	65	0	0	0	0
IL1B	GTACTGGTACCAACCACA	67	68	0	0	0	0
IL1B	ACACTGGTACCAACCAAC	57	27	0	0	0	1
IL1B	CAACTGGTACCAACCATG	466	501	1	0	0	0
IL1B	GTTGCATGGTGTACACGT	104	53	0	0	0	1
IL1B	CATGCATGGTGTACACCA	204	245	0	0	0	0
IL1R1	TGACTGCAGTACTGCACA	83	49	0	0	1	0
IL1R1	CAACTGCAGTACTGCAAC	19	25	0	0	0	0
IL1R1	GTACTGCAGTACTGCATG	42	46	0	1	0	0
IL1R1	TGACTGCAGTACTGACGT	139	150	0	0	0	0
IL1R1	TGTGGTGTGTACACCACA	0	6	0	0	0	0
IL1R1	ACTGCATGGTGTACTGCA	29	31	1	0	0	0
IL1R2	GTACTGGTACTGGTACAC	21	27	0	1	0	0
IL1R2	TGACTGGTACTGGTACTG	429	300	0	0	0	0
IL1R2	ACACTGGTACTGGTTGGT	424	324	0	0	0	0
IL1R2	TGACTGGTACTGGTTGCA	203	296	0	0	0	0
IL1R2	CAACTGGTACTGGTTGAC	29	9	0	0	0	0
IL1R2	GTTGCATGGTGTACTGAC	22	35	0	0	0	0
IL1RAP	GTACTGGTACTGCAGTTG	1292	1627	19833	23	1	29
IL1RAP	CAACTGGTACTGCAGTAC	43	65	0	0	0	0
IL1RAP	TGACTGGTACTGCACAGT	390	453	1	0	0	0
IL1RAP	GTACTGGTACTGCACACA	137	96	2	0	0	0
IL1RAP	ACACTGGTACTGCACAAC	25	29	0	0	0	0
IL1RAP	GTTGCAACTGCAACGTTG	311	611	0	0	0	0
IL1RL1	ACTGCATGGTGTACACTG	209	233	0	371	14	0
IL1RL1	ACACTGGTACACTGTGGT	432	588	1	1	1	10
IL1RL1	TGACTGGTACACTGACTG	407	634	1	1	0	2
IL1RL1	TGACTGGTACACTGTGCA	593	574	0	0	0	2
IL1RL1	CAACTGGTACACTGTGAC	161	46	0	0	0	0
IL1RL1	GTACTGGTACACTGTGTG	130	106	0	0	0	0
IL1RN	TGACTGCAGTGTACACGT	65	53	0	0	1	0
IL1RN	GTACTGCAGTGTACACCA	108	230	0	0	0	0
IL1RN	ACACTGCAGTGTACACAC	42	58	0	0	0	0
IL1RN	CAACTGCAGTGTACACTG	118	132	0	0	0	0
IL1RN	GTACTGCAGTGTACTGGT	119	263	0	0	0	0
IL1RN	TGTGCATGGTGTACACAC	17	29	0	0	0	0
IL2	TGACTGCAGTCAGTTGTG	128	98	0	0	1	0
IL2	ACACTGCAGTCAGTTGCA	156	122	1	0	0	1
IL2	GTACTGCAGTCAGTTGAC	85	28	0	0	0	0
IL2	CAACTGCAGTCACAGTGT	375	364	0	0	0	0
IL2	CATGCAGTTGACACACCA	60	100	0	0	0	0
IL21	ACACTGCAGTACACCAAC	25	40	0	0	0	0
IL21	CAACTGCAGTACACCATG	97	88	1	0	0	0
IL21	GTACTGCAGTACACACGT	118	200	0	0	0	1
IL21	CAACTGCAGTACACACCA	176	159	1	2	0	0
IL21	TGACTGCAGTACACACAC	110	241	0	0	0	0
IL21	ACTGCACATGACGTCAGT	59	77	0	0	0	0
IL21R	GTACTGGTCATGGTTGGT	638	740	0	1	0	0
IL21R	CAACTGGTCATGGTTGCA	211	122	1	1	0	0
IL21R	TGACTGGTCATGGTTGAC	32	41	0	0	0	0
IL21R	ACACTGGTCATGGTTGTG	179	511	2	0	0	0
IL21R	GTACTGGTCATGCAGTGT	1146	460	0	0	0	0
IL21R	TGTGCATGGTTGCACACA	53	49	0	0	0	0
IL22	TGACTGGTTGTGTGACTG	410	723	0	0	1	1
IL22	CAACTGGTTGTGTGACGT	159	236	0	1	0	2
IL22	ACACTGGTTGTGTGACCA	48	52	0	0	0	0
IL22	GTACTGGTTGTGTGACAC	47	42	0	1	0	0
IL22	ACACTGACTGCAACTGGT	335	165	0	1	0	0
IL22RA1	CAACTGGTTGTGACACAC	216	141	0	0	0	0
IL22RA1	GTACTGGTTGTGACACTG	340	448	0	0	0	1

IL22RA1	TGACTGGTTGTGACTGGT	105	129	4	0	0	0
IL22RA1	GTACTGGTTGTGACTGCA	141	78	0	0	0	0
IL22RA1	ACACTGGTTGTGACTGAC	22	23	0	0	0	0
IL22RA1	CATGCATGGTTGACGTCA	125	108	0	0	0	0
IL22RA2	CAACTGGTACACCAACTG	2107	1720	0	3	1	1
IL22RA2	GTACTGGTACACCATGGT	502	669	0	0	0	0
IL22RA2	CAACTGGTACACCATGCA	349	511	2	2	0	0
IL22RA2	TGACTGGTACACCATGAC	48	49	0	0	0	0
IL22RA2	ACACTGGTACACCATGTG	449	344	0	0	0	0
IL22RA2	TGTGCATGGTTGACGTAC	20	19	0	0	0	0
IL23A	CAACTGGTTGACTGCATG	621	248	0	0	1	0
IL23A	GTACTGGTTGACTGGTTG	1025	560	0	1	0	6
IL23A	TGACTGGTTGACTGCAGT	471	221	0	0	0	0
IL23A	GTACTGGTTGACTGCACA	63	299	0	2	0	1
IL23A	ACACTGGTTGACTGCAAC	79	130	0	0	0	1
IL23A	GTTGCATGGTGTTGCATG	439	122	0	0	0	1
IL23R	TGTGCAACTGCACAACTG	530	701	0	1	1	0
IL23R	CAACTGCAACCACATGTG	225	247	0	0	0	0
IL23R	TGACTGCAACCAACGTGT	535	504	0	0	0	0
IL23R	GTACTGCAACCAACGTCA	388	534	1	0	0	3
IL23R	ACACTGCAACCAACGTAC	38	28	0	0	0	0
IL23R	CAACTGCAACCAACGTTG	253	171	0	0	0	0
IL24	CAACTGGTACACGTACCA	45	48	0	0	0	0
IL24	TGACTGGTACACGTACAC	24	41	0	0	0	0
IL24	ACACTGGTACACGTACTG	287	341	0	0	0	0
IL24	CAACTGGTACACGTTGGT	112	71	1	0	0	0
IL24	ACACTGGTACACGTTGCA	157	156	0	0	0	0
IL24	ACTGCATGGTTGACGTTG	140	382	1	0	0	0
IL25	TGTGCAACTGTGTGTGGT	602	603	20	14	18	42
IL25	CAACTGGTTGACCATGTG	590	678	0	1	1	0
IL25	TGACTGGTTGACCATGGT	345	324	0	0	0	1
IL25	GTACTGGTTGACCATGCA	226	557	0	1	0	4
IL25	ACACTGGTTGACCATGAC	34	32	0	0	0	0
IL25	GTTGCAACTGTGTGACTG	428	365	0	0	0	1
IL27	CAACTGGTACACACACAC	121	68	0	0	0	0
IL27	GTACTGGTACACACACTG	349	362	1	0	0	0
IL27	TGACTGGTACACACTGGT	181	173	0	2	0	0
IL27	GTACTGGTACACACTGCA	249	150	0	0	0	1
IL27	ACACTGGTACACACTGAC	24	42	0	0	0	6
IL27RA	CAACTGGTCATGTGCATG	828	352	1	1	0	2
IL27RA	GTACTGGTCATGTGACGT	1149	495	1	0	0	0
IL27RA	CAACTGGTCATGTGACCA	145	206	1	0	0	1
IL27RA	TGACTGGTCATGTGACAC	33	43	0	0	0	0
IL27RA	ACACTGGTCATGTGACTG	167	194	0	1	0	0
IL27RA	CATGCAACCAGTGTCAGT	24	9	0	0	0	0
IL28B	ACACTGGTTGGTGTACGT	106	98	0	0	1	0
IL28B	GTACTGGTTGGTGTCAAC	6	15	0	0	0	0
IL28B	TGACTGGTTGGTGTCATG	322	599	0	4	0	0
IL28B	TGACTGGTTGGTGTACCA	50	56	0	0	0	0
IL28B	CAACTGCAGTGTTGGTCA	387	221	0	0	0	1
IL28B	CATGCATGGTGTTGCAAC	15	8	0	0	0	0
IL28RA	GTACTGCAGTGTCAGTTG	1118	2621	5	218	1	5
IL28RA	TGACTGCAGTGTCAGTCA	208	242	1	0	0	0
IL28RA	CAACTGCAGTGTCAGTAC	73	37	0	0	0	0
IL28RA	TGACTGCAGTGTCACAGT	105	199	0	0	0	0
IL28RA	GTACTGCAGTGTCACACA	285	125	0	0	0	0
IL28RA	TGTGCATGGTCAGTACTG	147	359	0	0	0	0
IL2RA	GTACTGCAGTCATGCAGT	40	45	0	0	0	0
IL2RA	CAACTGCAGTCATGCACA	231	128	0	0	0	0
IL2RA	TGACTGCAGTCATGCAAC	24	24	0	0	0	0
IL2RA	ACACTGCAGTCATGCATG	167	276	1	0	0	0
IL2RA	CAACTGCAGTCATGACGT	141	148	0	0	0	0
IL2RA	GTTGCACACAACGTTGCA	154	65	1	0	0	0
IL2RB	CAACTGGTTGCAACACTG	130	228	1	1	0	0
IL2RB	GTACTGGTTGCAACTGGT	693	493	0	0	0	0
IL2RB	CAACTGGTTGCAACTGCA	313	239	2	0	0	0
IL2RB	TGACTGGTTGCAACTGAC	18	13	0	0	0	0
IL2RB	ACACTGGTTGCAACTGTG	754	833	0	1	0	0
IL2RB	GTTGCAACTGGTGTACCA	183	176	1	0	0	0
IL2RG	GTACTGGTACACACGTGT	513	551	1	81	1	7
IL2RG	ACACTGGTACACACGTTG	582	678	49	0	1	0
IL2RG	CAACTGGTACACACGTCA	156	197	0	0	0	0
IL2RG	TGACTGGTACACACGTAC	63	47	0	0	0	0
IL2RG	CAACTGGTACACACCAGT	81	83	0	0	0	0
IL2RG	TGTGCACACAACACTGTG	154	155	0	0	0	0

IL3	GTACTGGTACTGCAACGT	606	730	15	0	2	0
IL3	CAACTGGTACTGCACATG	337	435	0	0	0	0
IL3	CAACTGGTACTGCAACCA	426	363	0	0	0	1
IL3	TGACTGGTACTGCAACAC	86	151	1	0	0	0
IL3	ACTGCATGGTCAGTTGGT	262	324	0	0	0	1
IL3	TGTGCATGGTCAGTTGCA	288	135	0	0	0	0
IL3RA	ACACTGGTACCAACTGCA	156	170	0	0	0	0
IL3RA	GTACTGGTACCAACTGAC	37	45	0	0	0	0
IL3RA	TGACTGGTACCAACTGTG	384	524	2	1	0	1
IL3RA	CAACTGGTACCATGGTGT	541	825	1	1	0	0
IL3RA	CATGCAGTTGACGTACAC	26	36	0	0	0	0
IL3RA	ACTGCATGGTCACAGTGT	130	200	4	0	0	0
IL4	ACACTGGTCATGCAGTTG	321	242	1	9	13	0
IL4	GTTGCATGGTGTGTGTGT	576	594	0	0	1	3
IL4	CAACTGGTCATGCAGTCA	303	232	0	0	0	0
IL4	TGACTGGTCATGCAGTAC	119	104	0	0	0	0
IL4	CAACTGGTCATGCACAGT	143	397	0	0	0	0
IL4	CATGCATGGTGTGTGTCA	245	249	0	36	0	0
IL4R	CAACTGGTTGGTTGTGCA	241	171	0	0	1	0
IL4R	GTACTGGTTGGTTGTGGT	217	359	1	0	0	1
IL4R	TGTGACGTTGCAACTGCA	63	47	0	0	0	1
IL4R	CATGACGTTGCAACTGAC	14	35	0	0	0	5
IL4R	GTTGACGTTGCAACTGTG	287	417	0	0	0	0
IL5	ACTGCATGGTCACACAAC	15	12	0	0	1	3
IL5	CAACTGGTTGCACACATG	171	106	0	0	0	0
IL5	GTACTGGTTGCACAACGT	280	368	0	0	0	11
IL5	CAACTGGTTGCACAACCA	279	443	0	0	0	0
IL5	TGACTGGTTGCACAACAC	43	43	0	0	0	0
IL5	ACACTGGTTGCACAACTG	151	223	0	0	0	0
IL5RA	CAACTGCAGTCACAACTG	433	842	50	180	1	1
IL5RA	ACACTGCAGTCACAACAC	28	13	0	0	0	3
IL5RA	GTACTGCAGTCACATGGT	426	294	0	0	0	0
IL5RA	CAACTGCAGTCACATGCA	296	224	0	0	0	0
IL5RA	CATGCAGTTGACACCATG	539	252	1	1	0	0
IL5RA	GTTGCATGGTCACACACA	88	79	0	0	0	2
IL6	TGTGCACATGCACATGTG	262	454	1	1	1	7
IL6	CAACTGCAGTACGTTGTG	517	514	0	2	0	2
IL6	TGACTGCAGTACCAGTGT	547	666	2	2	0	1
IL6	GTACTGCAGTACCAGTCA	118	134	1	0	0	0
IL6	ACACTGCAGTACCAGTAC	73	120	0	0	0	0
IL6	GTTGCACATGCACATGAC	35	28	0	0	0	0
IL6R	CAACTGGTTGCATGTGTG	90	320	0	0	0	0
IL6R	CAACTGGTTGACGTGTGT	534	417	2	0	0	0
IL6R	ACACTGGTTGACGTGTCA	365	384	0	0	0	1
IL6R	GTACTGGTTGACGTGTAC	59	157	1	0	0	0
IL6R	TGACTGGTTGACGTGTTG	362	819	1	0	0	0
IL6R	CATGCATGGTTGACCAGT	149	310	0	0	0	0
IL6ST	CAACTGGTACGTCAACAC	146	181	1	0	0	0
IL6ST	GTACTGGTACGTCAACTG	334	308	1	0	0	0
IL6ST	TGACTGGTACGTCATGGT	239	249	0	0	0	0
IL6ST	GTACTGGTACGTCATGCA	176	292	1	0	0	9
IL6ST	ACACTGGTACGTCATGAC	22	32	0	0	0	0
IL6ST	TGTGCATGGTCAACGTTG	374	192	0	0	0	1
IL7	CAACTGCAGTCATGTGAC	122	93	0	0	0	0
IL7	GTACTGCAGTCATGTGTG	307	476	1	0	0	0
IL7	GTACTGCAGTACGTGTGT	413	424	0	1	0	1
IL7	CAACTGCAGTACGTGTCA	172	211	0	0	0	1
IL7	CATGCAGTTGACGTGTCA	270	269	1	0	0	0
IL7	TGTGCATGGTCAACCACA	105	111	0	0	0	0
IL7R	GTACTGGTCATGTGGTTG	1414	1671	1	0	1	12
IL7R	CAACTGGTCATGTGGTAC	133	197	0	0	0	0
IL7R	TGACTGGTCATGTGCAGT	178	215	2	0	0	0
IL7R	GTACTGGTCATGTGCACA	129	201	0	0	0	0
IL7R	ACACTGGTCATGTGCAAC	24	15	0	0	0	0
IL7R	ACTGCATGGTCAACCAGT	157	330	0	0	0	0
IL8	ACACTGGTCACAACGTTG	1021	642	1	1	0	101
IL8	CAACTGGTCACAACCAGT	83	127	0	0	0	0
IL8	ACACTGGTCACAACCACA	300	271	0	0	0	0
IL8	GTACTGGTCACAACCAAC	18	128	0	0	0	0
IL8	TGACTGGTCACAACCATG	114	73	0	0	0	0
IL8	CATGCATGGTCAACCAAC	47	51	0	2	0	0
IL8RA	TGACGTCACATGCAGTAC	31	45	0	0	0	0
IL8RA	ACACGTCACATGCAGTTG	1097	1638	2	2	0	4
IL8RA	CAACGTCACATGCACAGT	258	435	1	2	0	0
IL8RA	ACACGTCACATGCACACA	264	165	1	0	0	0

IL8RA	GTACGTCACATGCACAAC	14	24	0	0	0	0
IL8RA	TGTGCATGGTACGTGTTG	287	252	0	1	0	0
IL8RB	ACTGCATGGTACGTCAGT	102	319	0	283	1	0
IL8RB	TGACGTCACATGCACATG	248	308	0	0	0	0
IL8RB	ACACGTCACATGCAACGT	109	68	0	0	0	0
IL8RB	TGACGTCACATGCAACCA	146	181	1	0	0	0
IL8RB	CAACGTCACATGCAACAC	401	208	0	0	0	0
IL8RB	GTACGTCACATGCAACTG	914	936	0	0	0	0
IL9	TGACTGCAGTGTGTTGCA	150	84	0	1	0	0
IL9	CAACTGCAGTGTGTTGAC	112	70	0	0	0	0
IL9	GTACTGCAGTGTGTTGTG	47	52	0	1	0	0
IL9	ACACTGCAGTGTCAGTGT	462	121	1	1	0	0
IL9	CATGCACATGCACATGGT	334	375	0	0	0	0
IL9	ACTGCACATGCACATGCA	192	107	0	0	0	0
IL9R	GTACTGGTACGTTGACGT	116	48	0	1	0	0
IL9R	CAACTGGTACGTTGACCA	367	332	0	0	0	0
IL9R	TGACTGGTACGTTGACAC	40	26	0	0	0	0
IL9R	ACACTGGTACGTTGACTG	484	602	1	0	0	0
IL9R	TGTGCATGGTACGTCACA	30	38	0	0	0	0
IL9R	CATGCATGGTACGTCAAC	14	21	0	0	0	0
ILK	TGCATGCATGCACATGAC	25	29	0	0	0	0
ILK	ACCATGCATGCACATGTG	95	60	1	0	0	0
ILK	GTCATGCATGCAACGTGT	53	347	1	0	0	1
ILK	CACATGCATGCAACGTCA	384	670	0	0	0	0
ILK	TGCATGCATGCAACGTAC	38	25	0	0	0	0
ILVBL	ACCATGACCATGCAGTTG	419	571	2	0	1	25
ILVBL	CACATGACCATGCAGTCA	230	224	0	1	0	0
ILVBL	TGCATGACCATGCAGTAC	18	19	0	0	0	0
ILVBL	CACATGACCATGCACAGT	341	204	0	0	0	0
ILVBL	GTTGCAGTGTCAGTGTGT	183	164	0	0	0	0
ILVBL	GTTGCATGGTACGTCATG	272	229	0	0	0	0
IMMT	ACTGGTCAACGTCAACAC	15	5	0	0	1	0
IMMT	GTTGGTCACAACCACAAC	11	8	0	0	0	0
IMMT	GTTGGTCAACGTCACATG	113	162	0	0	0	0
IMMT	TGTGGTCAACGTCAACGT	302	350	0	0	0	0
IMMT	GTTGGTCAACGTCAACCA	147	78	0	1	0	0
IMPA1	TGACACACACCACACAGT	336	389	2	3	4	5762
IMPA1	GTACACACACCACAGTTG	1464	1394	2	0	1	8
IMPA1	TGACACACACCACAGTCA	531	304	2	1	0	0
IMPA1	CAACACACACCACAGTAC	30	39	0	0	0	0
IMPA1	GTACACACACCACACACA	55	109	0	0	0	0
IMPA1	TGTGCACATGGTGTTGGT	298	536	0	0	0	0
IMPA2	CAACACCATGTGACCATG	185	225	0	0	1	0
IMPA2	GTACACCATGTGACACGT	170	161	0	0	0	0
IMPA2	CAACACCATGTGACACCA	268	322	0	0	0	0
IMPA2	TGACACCATGTGACACAC	18	16	0	0	0	2
IMPA2	ACACACCATGTGACACTG	425	531	0	0	0	6
IMPA2	ACTGCACAACTGGTCATG	246	177	1	2	0	0
IMPDH1	ACACCAGTCATGCAACTG	2311	3129	1	3	1	59
IMPDH1	CAACCAGTCATGCACATG	248	192	0	0	0	0
IMPDH1	GTACCAGTCATGCAACGT	536	374	0	1	0	0
IMPDH1	CAACCAGTCATGCAACCA	949	879	0	3	0	0
IMPDH1	TGACCAGTCATGCAACAC	43	149	0	0	0	0
IMPDH2	GTACCAGTCATGCAGTTG	665	402	2	0	3	0
IMPDH2	CAACCAGTCATGCAGTAC	321	286	1	0	0	0
IMPDH2	TGACCAGTCATGCACAGT	363	224	1	0	0	21
IMPDH2	GTACCAGTCATGCACACA	19	28	0	0	0	0
IMPDH2	ACACCAGTCATGCACAAC	77	62	0	0	0	0
INDO	TGACACTGACGTACCAAC	19	26	0	0	0	0
INDO	ACACACTGACGTACCATG	128	92	0	0	0	0
INDO	CAACACTGACGTACACGT	56	49	0	0	0	0
INDO	ACACACTGACGTACACCA	346	415	4	0	0	0
INDO	GTACACTGACGTACACAC	14	15	0	0	0	0
INHA	CAACTGACCACAACTGGT	234	284	0	0	0	0
INHA	ACACTGACCACAACTGCA	345	544	0	0	0	0
INHA	GTACTGACCACAACTGAC	62	83	0	0	0	1
INHA	CAACTGACTGCATGGTAC	70	97	0	0	0	0
INHA	GTACTGACTGCATGGTTG	131	564	1	0	0	0
INHA	CATGCACATGGTACTGGT	153	172	0	0	0	0
INHBA	GTACTGCAGTACTGACCA	98	54	0	0	0	0
INHBA	ACACTGCAGTACTGACAC	38	37	0	0	0	0
INHBA	CAACTGCAGTACTGACTG	266	276	1	0	0	0
INHBA	GTACTGCAGTACTGTGGT	340	277	0	0	0	0
INHBA	CAACTGCAGTACTGTGCA	262	471	0	0	0	0
INHBB	GTACTGGTACCATGACCA	503	621	3	0	1	1

INHBB	CAACTGGTACCATGCAAC	3	15	0	0	0	0
INHBB	GTACTGGTACCATGCATG	85	150	0	1	0	1
INHBB	TGACTGGTACCATGACGT	188	186	0	0	0	1
INHBB	GTTGCAGTTGACGTTGCA	306	310	4	0	0	0
INHBB	ACTGCACATGGTACTGCA	11	25	1	0	0	0
INHBC	TGACTGCAGTGTGTCAAC	25	45	0	0	0	0
INHBC	ACACTGCAGTGTGTCATG	309	314	0	0	0	0
INHBC	CAACTGCAGTGTGTACGT	894	918	3	0	0	0
INHBC	ACACTGCAGTGTGTACCA	223	115	0	0	0	0
INHBC	GTACTGCAGTGTGTACAC	31	119	0	1	0	0
INHBC	TGTGCAACGTACGTGTAC	47	29	1	0	0	0
INPP1	TGACACACACGTGTACCA	140	82	0	0	0	0
INPP1	CAACACACACGTGTACAC	130	71	1	0	0	0
INPP1	GTACACACACGTGTACTG	203	424	1	0	0	0
INPP1	TGACACACACGTGTTGGT	94	129	0	0	0	0
INPP1	GTACACACACGTGTTGCA	80	115	0	0	0	0
INPP1	CATGCACACAACTGCAAC	45	64	0	0	0	0
INPP4A	TGCATGCAACGTTGGTTG	148	164	0	0	0	0
INPP4A	ACCATGCAACGTTGCAGT	16	20	0	0	0	0
INPP4A	TGCATGCAACGTTGCACA	6	8	0	0	0	0
INPP4A	CACATGCAACGTTGCAAC	7	22	5	0	0	0
INPP4A	GTCATGCAACGTTGCATG	72	88	0	0	0	0
INPP4A	ACTGCACAGTTGCAACCA	59	56	0	0	0	0
INPP4B	GTACACACTGCATGTGTG	255	505	0	1	0	0
INPP4B	GTACACACTGACGTGTGT	207	420	0	0	0	1
INPP4B	CAACACACTGACGTGTCA	556	869	0	1	0	2
INPP4B	TGACACACTGACGTGTAC	272	61	0	0	0	0
INPP4B	ACACACACTGACGTGTTG	197	224	0	0	0	0
INPP4B	GTTGCACAGTTGCAACAC	21	33	0	0	0	0
INPP5A	CAACACACGTGTTGCATG	139	185	1	1	1	0
INPP5A	GTACACACGTGTTGCACA	24	25	0	0	0	0
INPP5A	ACACACACGTGTTGCAAC	37	28	0	0	0	0
INPP5A	GTACACACGTGTTGACGT	79	62	0	0	0	0
INPP5A	CAACACACGTGTTGACCA	38	122	0	0	0	0
INPP5B	TGACACACACGTACCAGT	264	96	0	0	0	0
INPP5B	GTACACACACGTACCACA	47	37	0	0	0	0
INPP5B	ACACACACACGTACCAAC	23	25	0	0	0	5
INPP5B	CAACACACACGTACCATG	249	371	1	0	0	0
INPP5B	GTACACACACGTACACGT	67	122	0	0	0	0
INPP5D	GTACCATGCAACGTGTAC	66	57	0	0	0	0
INPP5D	TGACCATGCAACGTGTTG	106	97	0	0	0	0
INPP5D	ACACCATGCAACGTCAGT	47	39	0	0	0	0
INPP5D	TGACCATGCAACGTCACA	91	79	0	0	0	0
INPP5D	CATGCACAGTGTTGACGT	230	181	0	0	0	0
INPP5E	ACACTGTGACACTGGTGT	1463	624	2	0	1	0
INPP5E	GTACTGTGACACACTGTG	223	340	0	0	0	0
INPP5E	TGACTGTGACACTGGTCA	193	321	0	0	0	0
INPP5E	CAACTGTGACACTGGTAC	325	258	0	0	0	0
INPP5E	GTACTGTGACACTGGTTG	259	274	0	0	0	0
INPPL1	TGACACACTGACGTACCA	267	303	0	0	2	0
INPPL1	CAACACACTGACGTACAC	40	46	0	0	0	0
INPPL1	GTACACACTGACGTACTG	654	625	1	0	0	1
INPPL1	TGACACACTGACGTTGGT	662	182	0	0	0	0
INPPL1	GTACACACTGACGTTGCA	325	305	1	0	0	0
INPPL1	GTTGCACACAACTGCATG	163	209	1	1	0	5
INS	GTTGGTGTGTACTGGTGT	1055	367	0	0	1	0
INS	GTTGGTGTGTACACTGGT	132	354	0	0	0	6
INS	CATGGTGTGTACACTGCA	163	100	0	0	0	0
INS	TGTGGTGTGTACACTGAC	25	38	0	0	0	0
INS	ACTGGTGTGTACACTGTG	234	234	1	0	0	0
INSIG2	CATGGTTGTGACACCATG	12	13	0	0	0	0
INSIG2	GTTGGTTGTGACACACGT	111	143	0	0	0	0
INSIG2	CATGGTTGTGACACACCA	197	208	0	0	0	2
INSIG2	TGTGGTTGTGACACACAC	53	53	0	0	0	0
INSIG2	ACTGGTTGTGACACACTG	149	180	0	1	0	0
INSR	TGCATGCACAACACACGT	23	12	0	0	0	0
INSR	GTCATGCACAACACACCA	380	493	0	2	0	2
INSR	ACCATGCACAACACACAC	67	40	0	0	0	0
INSR	CACATGCACAACACACTG	1090	1011	2	0	0	4
INSR	ACACCATGCAGTGTGTTG	379	400	1	0	0	0
IPMK	GTACACACTGCACACATG	455	946	1	1	2	0
IPMK	ACACACACTGCACACAGT	242	162	0	0	0	0
IPMK	TGACACACTGCACACACA	80	94	1	0	0	1
IPMK	CAACACACTGCACACAAC	39	39	0	0	0	0
IPMK	TGACACACTGCACAACGT	795	978	0	0	0	0

IQGAP2	CATGCAGTTGGTTGGTCA	352	532	1	1	1	2
IQGAP2	GTACACCAGTTGACGTGT	87	96	0	0	0	0
IQGAP2	CAACACCAGTTGACGTCA	65	96	0	0	0	0
IQGAP2	TGACACCAGTTGACGTAC	46	39	0	0	0	0
IQGAP2	ACACACCAGTTGACGTTG	460	481	0	0	0	0
IRAK1	CACATGCAACGTTGACTG	444	492	34634	30	3	5
IRAK1	GTCATGCAACGTTGTGGT	387	375	0	0	1	1
IRAK1	TGCATGCAACGTTGACGT	693	178	1	0	0	0
IRAK1	GTCATGCAACGTTGACCA	204	138	0	0	0	0
IRAK1	ACCATGCAACGTTGACAC	76	113	0	0	0	0
IRAK1BP1	CAACACACTGCAGTCATG	213	384	0	1	1	0
IRAK1BP1	GTACACACTGCAGTCACA	136	90	0	0	0	0
IRAK1BP1	ACACACACTGCAGTCAAC	24	28	0	0	0	0
IRAK1BP1	GTACACACTGCAGTACGT	46	71	0	0	0	0
IRAK1BP1	CAACACACTGCAGTACCA	194	448	0	0	0	0
IRAK2	CACATGCAACGTTGTGCA	257	336	1	0	0	0
IRAK2	TGCATGCAACGTTGTGAC	18	33	0	0	0	0
IRAK2	ACCATGCAACGTTGTGTG	897	381	1	0	0	0
IRAK2	TGCATGCAACCAGTGTGT	392	709	0	0	0	0
IRAK2	CATGGTACTGCAACTGAC	153	71	0	0	0	0
IRAK3	TGCATGCATGGTACTGAC	68	89	0	0	0	0
IRAK3	ACCATGCATGGTACTGTG	319	131	0	0	0	0
IRAK3	GTCATGCATGGTTGGTGT	470	266	3	0	0	0
IRAK3	CACATGCATGGTTGGTCA	172	186	0	0	0	0
IRAK3	TGCATGCATGGTTGGTAC	14	28	0	0	0	0
IRAK3	GTTGCACAACCAACACGT	38	33	0	0	0	0
IRAK4	GTCATGACGTTGTGGTAC	236	114	2	2	1	0
IRAK4	TGCATGACGTTGACTGTG	328	216	0	0	0	0
IRAK4	CACATGACGTTGTGGTGT	449	1009	0	1	0	0
IRAK4	ACCATGACGTTGTGGTCA	476	400	1	0	0	1
IRAK4	TGTGCAGTACGTACCAGT	59	60	0	0	0	0
IRAK4	ACTGCACAACTGACACAC	39	40	27	0	0	0
IREB2	ACACACTGCATGCAACGT	342	277	1	0	1	23
IREB2	ACACACTGCATGCACACA	255	270	0	0	0	0
IREB2	GTACACTGCATGCACAAC	28	18	0	0	0	0
IREB2	TGACACTGCATGCACATG	207	207	0	1	0	3
IREB2	TGACACTGCATGCAACCA	314	461	0	1	0	0
IRF1	TGACGTACGTCATGTGCA	27	68	0	0	0	0
IRF1	CAACGTACGTCATGTGAC	98	148	1	1	0	0
IRF1	GTACGTACGTCATGTGTG	215	124	0	0	0	0
IRF1	GTACGTACGTACGTGTGT	209	330	0	0	0	0
IRF1	ACTGGTTGTGGTGTACCA	60	68	0	0	0	0
IRF1	TGTGCACAGTTGCAACTG	326	272	2	0	0	0
IRF2	GTACGTACGTACGTACTG	86	134	0	0	1	0
IRF2	CAACGTACGTACGTACAC	34	23	0	0	0	0
IRF2	TGACGTACGTACGTTGGT	210	285	0	0	0	0
IRF2	GTACGTACGTACGTTGCA	209	54	0	0	0	0
IRF2	ACACGTACGTACGTTGAC	16	75	0	0	0	0
IRF2BP1	ACACCACACAGTCATGTG	1308	799	1	0	1	0
IRF2BP1	CAACCACACAGTACGTCA	234	211	0	0	1	0
IRF2BP1	TGACCACACAGTCATGAC	92	99	0	0	0	0
IRF2BP1	GTACCACACAGTACGTGT	1282	1398	2	2	0	22
IRF2BP1	TGACCACACAGTACGTAC	127	92	0	0	0	0
IRF2BP1	TGTGCAACTGCAGTCATG	74	121	0	0	0	0
IRF2BP2	GTACCACACAGTTGACAC	74	113	1	0	0	0
IRF2BP2	TGACCACACAGTTGACTG	128	74	0	0	0	0
IRF2BP2	ACACCACACAGTTGTGGT	481	857	2	0	0	7
IRF2BP2	TGACCACACAGTTGTGCA	265	346	1	1	0	0
IRF2BP2	CAACCACACAGTTGTGAC	116	188	0	1	0	0
IRF3	TGCATGTGTGCAGTACCA	103	127	0	0	0	33
IRF3	CACATGTGTGCAGTACAC	118	38	0	0	0	0
IRF3	GTCATGTGTGCAGTACTG	118	309	0	0	0	0
IRF3	TGCATGTGTGCAGTTGGT	492	445	0	1	0	0
IRF3	GTCATGTGTGCAGTTGCA	165	171	0	0	0	0
IRF4	GTACGTACGTACCATGTG	140	165	16	0	0	1
IRF4	ACACGTACGTACACGTGT	135	260	1	0	0	0
IRF4	TGACGTACGTACACGTCA	350	339	1	0	0	2
IRF4	CAACGTACGTACACGTAC	18	16	0	0	0	0
IRF4	GTACGTACGTACACGTTG	76	97	0	0	0	0
IRF4	TGTGCACAGTTGTGACGT	102	86	0	0	0	2
IRF5	GTACGTACGTACACACGT	3	2	0	0	0	0
IRF5	CAACGTACGTACACACCA	184	176	1	0	0	0
IRF5	TGACGTACGTACACACAC	34	20	0	0	0	0
IRF5	ACACGTACGTACACACTG	193	263	0	0	0	1
IRF5	ACTGCAGTGTTGACTGTG	498	332	1	0	0	0

IRF6	CAACGTACGTACTGCAAC	37	50	0	0	0	0
IRF6	GTACGTACGTACTGCATG	46	47	0	0	0	0
IRF6	TGACGTACGTACTGACGT	52	34	1	0	0	0
IRF6	GTACGTACGTACTGACCA	25	31	0	0	0	0
IRF6	ACACGTACGTACTGACAC	18	11	0	0	0	0
IRF6	CATGCAACTGACGTTGGT	69	58	0	0	0	0
IRF7	CAACGTACGTACTGACTG	247	188	1	0	0	0
IRF7	GTACGTACGTACTGTGGT	404	336	0	0	0	0
IRF7	CAACGTACGTACTGTGCA	316	177	0	0	0	0
IRF7	TGTGGTTGTGTGTGACAC	50	41	0	0	0	0
IRF7	ACTGGTTGTGTGTGACTG	303	239	0	0	0	1
IRF8	GTACGTTGCATGACACCA	192	153	0	1	0	0
IRF8	ACACGTTGCATGACACAC	58	58	0	0	0	0
IRF8	CAACGTTGCATGACACTG	413	159	0	0	0	0
IRF8	GTACGTTGCATGACTGGT	467	628	0	0	0	0
IRF8	CAACGTTGCATGACTGCA	145	144	0	0	0	0
IRF9	GTACGTTGTGGTCAGTAC	52	38	2	1575	0	0
IRF9	TGACGTTGTGGTCAGTTG	672	478	2	0	0	0
IRF9	ACACGTTGTGGTCACAGT	126	136	0	0	0	0
IRF9	TGACGTTGTGGTCACACA	132	145	0	0	0	0
IRF9	CAACGTTGTGGTCACAAC	9	14	0	0	0	0
IRS1	CAACCATGCAACGTACTG	799	323	1	10	1	6
IRS1	GTACCATGCAACGTACCA	54	110	0	0	0	0
IRS1	ACACCATGCAACGTACAC	19	68	0	0	0	0
IRS1	GTACCATGCAACGTTGGT	881	468	0	0	0	0
IRS1	CAACCATGCAACGTTGCA	364	170	1	0	0	5
IRS1	CATGCACAACGTGTACTG	99	108	0	0	0	0
IRS2	TGACTGCAACACTGCATG	554	137	0	1	1	3
IRS2	GTTGCATGTGGTTGTGTG	857	1132	1	1	1	0
IRS2	ACACTGCAACACTGCACA	70	131	0	0	0	2
IRS2	GTACTGCAACACTGCAAC	40	38	0	0	0	0
IRS2	CATGCATGTGGTTGTGAC	53	62	0	0	0	0
IRS4	ACACTGACCAGTCATGAC	219	117	0	0	0	0
IRS4	CAACTGACCAGTCATGTG	854	619	4	1	0	0
IRS4	TGACTGACCAGTACGTGT	349	346	1	1	0	0
IRS4	GTACTGACCAGTACGTCA	220	169	0	0	0	0
IRS4	CATGCAGTTGTGCACAGT	41	65	0	0	0	5
IRS4	ACTGCACAGTTGCATGGT	899	1130	1	1	0	3
IRX3	GTACGTACACGTACGTGT	160	113	0	0	0	0
IRX3	CAACGTACACGTACGTCA	239	224	0	0	0	0
IRX3	TGACGTACACGTACGTAC	17	16	0	0	0	0
IRX3	ACACGTACACGTACGTTG	408	843	1	1	0	0
IRX3	CAACGTACACGTACCAGT	153	262	0	1	0	0
ISG15	CAACGTGTACCAGTGTTG	285	399	1	0	1	0
ISG15	ACACGTGTACCAGTGTAC	35	47	0	0	0	0
ISG15	GTACGTGTACCAGTCAGT	104	122	0	0	0	0
ISG15	CAACGTGTACCAGTCACA	95	121	0	0	0	0
ISG15	TGACGTGTACCAGTCAAC	22	98	0	0	0	0
ISG15	CATGCACACATGACACGT	25	31	1	0	0	0
ISG20	CAACACCATGCAACGTTG	493	477	0	0	1	0
ISG20	ACACACCATGCAACGTAC	37	36	0	0	0	0
ISG20	GTACACCATGCAACCAGT	526	319	2	1	0	3
ISG20	CAACACCATGCAACCACA	83	69	1	0	0	0
ISG20	ACTGCAGTTGCACATGTG	1164	309	3	1	0	1
ISL1	TGACGTACGTTGGTGTTG	533	405	0	1	0	0
ISL1	ACACGTACGTTGGTCAGT	97	111	0	0	0	0
ISL1	TGACGTACGTTGGTCACA	3	1	0	0	0	0
ISL1	CAACGTACGTTGGTCAAC	19	3	0	0	0	0
ISL1	GTACGTACGTTGGTCATG	119	108	0	0	0	0
ISL1	TGTGCACAGTTGCATGCA	161	364	0	0	0	0
ISLR	TGTGGTACACGTGTACTG	185	224	1	0	0	0
ISLR	ACTGGTACACGTGTTGGT	228	127	0	1	0	0
ISLR	TGTGGTACACGTGTTGCA	22	48	0	0	0	0
ISLR	CATGGTACACGTGTTGAC	46	39	0	0	0	0
ISLR	GTTGGTACACGTGTTGTG	667	777	2	1	0	0
ISYNA1	ACACACGTACACACTGTG	496	467	0	0	1	2
ISYNA1	GTACACGTACACTGGTGT	770	571	387	7	0	3
ISYNA1	CAACACGTACACTGGTCA	503	516	0	0	0	1
ISYNA1	TGACACGTACACTGGTAC	32	20	0	0	0	0
ISYNA1	ACACACGTACACTGGTTG	257	452	0	1	0	0
ISYNA1	GTTGCAACACACGTGTCA	799	604	1	0	0	0
ITCH	TGCATGACGTTGTGACGT	71	151	0	0	0	0
ITCH	GTCATGACGTTGTGACCA	88	111	0	0	0	0
ITCH	ACCATGACGTTGTGACAC	24	34	0	0	0	0
ITCH	CACATGACGTTGTGACTG	480	627	0	1	0	1

ITCH	CAACGTTGTGACTGACAC	23	23	0	0	0	0
ITCH	ACTGCATGGTGTGTGTTG	299	334	0	0	0	0
ITGA1	GTACTGGTGTACTGACTG	344	139	0	0	0	0
ITGA1	TGACTGGTGTACTGTGGT	409	673	0	2	0	0
ITGA1	GTACTGGTGTACTGTGCA	155	244	0	0	0	1
ITGA1	ACACTGGTGTACTGTGAC	6	22	0	0	0	0
ITGA1	CAACTGGTGTACTGTGTG	312	383	0	0	0	0
ITGA1	GTTGCATGCAGTTGCACA	47	41	0	0	0	0
ITGA2	CAACTGGTGTACTGCAGT	156	176	2	1	1	0
ITGA2	GTACTGGTGTACTGGTGT	751	414	0	1	0	0
ITGA2	CAACTGGTGTACTGGTCA	7	36	0	0	0	0
ITGA2	TGACTGGTGTACTGGTAC	50	45	0	0	0	0
ITGA2	ACACTGGTGTACTGGTTG	102	465	0	1	0	0
ITGA2B	GTACTGGTGTTGGTGTGT	829	300	1	2	0	1
ITGA2B	CAACTGGTGTTGGTGTCA	508	169	0	1	0	0
ITGA2B	TGACTGGTGTTGGTGTAC	126	92	0	0	0	0
ITGA2B	ACACTGGTGTTGGTGTTG	534	532	466	1	0	0
ITGA2B	CAACTGGTGTTGGTCAGT	63	74	0	0	0	0
ITGA2B	GTTGCACAACCAACTGAC	69	219	1	0	0	0
ITGA3	GTACTGGTGTACACCATG	237	244	1	0	3	0
ITGA3	CAACTGGTGTACACCAAC	17	17	0	0	0	0
ITGA3	TGACTGGTGTACACACGT	21	45	0	0	0	0
ITGA3	GTACTGGTGTACACACCA	185	113	0	0	0	0
ITGA3	ACACTGGTGTACACACAC	39	39	0	0	0	0
ITGA3	CATGCACAGTTGCATGAC	52	41	0	0	0	0
ITGA4	CAACCAGTTGCATGCATG	460	395	1	0	0	0
ITGA4	GTACCAGTTGCATGACGT	166	181	0	0	0	0
ITGA4	CAACCAGTTGCATGACCA	406	189	1	0	0	2
ITGA4	TGACCAGTTGCATGACAC	30	32	0	0	0	0
ITGA4	ACACCAGTTGCATGACTG	650	773	0	0	0	0
ITGA5	GTACCAGTTGCATGGTTG	1458	2046	2	0	1	0
ITGA5	TGACCAGTTGCATGCAGT	364	456	0	1	0	3
ITGA5	GTACCAGTTGCATGCACA	121	77	0	0	0	0
ITGA5	ACACCAGTTGCATGCAAC	7	16	0	0	0	0
ITGA5	TGTGGTTGTGGTGTTGCA	115	146	1	0	0	0
ITGA6	CAACTGGTGTTGGTACAC	42	77	0	0	0	0
ITGA6	GTACTGGTGTTGGTACTG	147	85	0	0	0	0
ITGA6	TGACTGGTGTTGGTTGGT	819	807	0	0	0	1
ITGA6	GTACTGGTGTTGGTTGCA	74	88	0	0	0	0
ITGA6	ACACTGGTGTTGGTTGAC	18	4	0	0	0	0
ITGA6	ACTGCACACAACGTTGAC	23	10	0	0	0	0
ITGA9	ACACTGGTGTACTGCACA	73	90	0	0	0	1
ITGA9	GTACTGGTGTACTGCAAC	45	25	0	0	0	0
ITGA9	TGACTGGTGTACTGCATG	143	207	1	1	0	0
ITGA9	ACACTGGTGTACTGACGT	45	80	1	0	0	0
ITGA9	GTTGACGTACCATGGTCA	141	440	1	1	0	1
ITGAE	CAACTGGTGTTGGTTGTG	275	321	2	0	0	0
ITGAE	TGACTGGTGTTGCAGTGT	389	500	0	0	0	0
ITGAE	GTACTGGTGTTGCAGTCA	639	319	0	0	0	0
ITGAE	ACACTGGTGTTGCAGTAC	121	155	0	0	0	0
ITGAE	TGTGACACACTGTGGTAC	122	150	0	0	0	0
ITGAL	GTACCAGTTGCACAGTGT	883	1133	2	0	1	2
ITGAL	ACACCAGTTGCACAGTTG	866	614	3	0	1	0
ITGAL	ACACCAGTTGCAGTTGTG	55	16	0	0	0	0
ITGAL	CAACCAGTTGCACAGTCA	484	532	0	0	0	0
ITGAL	TGACCAGTTGCACAGTAC	85	98	0	0	0	0
ITGAL	GTTGCACAGTTGCATGTG	213	334	2	0	0	1
ITGAM	ACACCAGTACGTGTACCA	429	303	1	0	0	1
ITGAM	GTACCAGTACGTGTACAC	7	9	0	0	0	0
ITGAM	TGACCAGTACGTGTACTG	209	349	1	0	0	1
ITGAM	ACACCAGTACGTGTTGGT	340	491	1	0	0	0
ITGAM	TGTGACCAACACACTGCA	180	304	0	0	0	0
ITGAV	GTCATGACACACACTGCA	184	168	1	0	1	0
ITGAV	GTCATGACACACACACTG	870	208	1	0	0	0
ITGAV	TGCATGACACACACTGGT	6	70	0	0	0	0
ITGAV	ACTGCAGTACGTACACTG	47	48	0	0	0	0
ITGAV	CATGCAGTACGTACTGGT	57	102	0	0	0	0
ITGAX	GTACCAGTACGTGTCAGT	210	243	0	0	0	0
ITGAX	CAACCAGTACGTGTCACA	35	27	0	0	0	0
ITGAX	TGACCAGTACGTGTCAAC	8	14	0	0	0	0
ITGAX	ACACCAGTACGTGTCATG	125	138	0	0	0	0
ITGAX	CAACCAGTACGTGTACGT	230	225	0	0	0	5
ITGB1	CAACCAGTTGCAACTGAC	45	46	0	0	0	0
ITGB1	GTACCAGTTGCAACTGTG	80	89	0	0	0	0
ITGB1	ACACCAGTTGCATGGTGT	617	464	0	0	0	1

ITGB1	TGACCAGTTGCATGGTCA	343	120	0	0	0	0
ITGB1	CAACCAGTTGCATGGTAC	100	70	1	0	0	0
ITGB1	CATGCAACCAGTTGACTG	164	84	0	0	0	0
ITGB1BP3	CAACGTTGTGACACCAAC	29	69	0	0	2	0
ITGB1BP3	ACTGGTTGGTACGTCAAC	30	15	0	0	0	0
ITGB1BP3	CATGGTTGGTACGTCATG	558	401	0	0	0	0
ITGB1BP3	GTTGGTTGGTACGTACGT	437	311	1	0	0	3
ITGB1BP3	ACTGACACTGGTACGTGT	312	473	0	0	0	1
ITGB2	GTACCAGTTGCAACACAC	90	114	1	0	0	0
ITGB2	TGACCAGTTGCAACACTG	440	504	1	2	0	0
ITGB2	ACACCAGTTGCAACTGGT	154	126	0	1	0	0
ITGB2	TGACCAGTTGCAACTGCA	107	134	0	1	0	0
ITGB2	CATGGTTGTGCAACTGTG	150	193	0	0	0	0
ITGB2	CATGCACACAACTGGTGT	264	274	251	1	0	0
ITGB3	GTCATGACACACACCAAC	40	37	0	0	0	0
ITGB3	TGCATGACACACACCATG	67	44	0	0	0	2
ITGB3	ACCATGACACACACACGT	5	15	0	0	0	0
ITGB3	TGCATGACACACACACCA	107	128	0	0	0	0
ITGB3	CACATGACACACACACAC	100	108	0	0	0	85
ITGB4	ACACTGGTGTTGGTCACA	229	214	0	0	0	0
ITGB4	GTACTGGTGTTGGTCAAC	38	62	0	0	0	0
ITGB4	TGACTGGTGTTGGTCATG	261	576	1	0	0	0
ITGB4	ACACTGGTGTTGGTACGT	97	85	0	0	0	0
ITGB4	TGACTGGTGTTGGTACCA	145	223	0	0	0	0
ITGB4BP	GTACTGGTGTACCATGAC	50	76	0	0	0	0
ITGB4BP	TGACTGGTGTACCATGTG	298	495	32	0	0	0
ITGB4BP	CAACTGGTGTACACGTGT	294	252	0	3	0	0
ITGB4BP	TGACTGACTGCAACTGCA	200	204	0	0	0	0
ITGB4BP	CATGCATGCAGTACACGT	85	244	0	0	0	0
ITGB4BP	ACTGCATGCAGTACACCA	64	79	1	0	0	0
ITGB5	ACTGCAGTCACAACTGGT	164	205	0	1	3	2
ITGB5	TGACTGGTGTACTGACCA	244	380	0	221	2	225
ITGB5	CAACTGGTGTACTGACAC	17	10	0	0	0	0
ITGB5	GTTGACCAACTGGTTGAC	49	64	0	0	0	0
ITGB5	TGTGACCAACTGGTTGTG	220	349	0	0	0	0
ITGB6	ACACTGGTGTACACGTCA	43	82	0	0	0	0
ITGB6	GTACTGGTGTACACGTAC	94	94	0	0	0	0
ITGB6	TGACTGGTGTACACGTTG	718	844	12	1	0	0
ITGB6	ACACTGGTGTACACCAGT	240	38	0	0	0	0
ITGB6	TGACTGGTGTACACCACA	51	111	0	0	0	0
ITGB6	ACTGCACATGCAGTACCA	59	78	0	0	0	0
ITGB7	CAACTGGTGTACACACTG	706	655	1	0	0	0
ITGB7	GTACTGGTGTACACTGGT	152	74	0	0	0	0
ITGB7	CAACTGGTGTACACTGCA	294	170	0	0	0	0
ITGB7	TGACTGGTGTACACTGAC	123	142	0	0	0	0
ITGB7	ACACTGGTGTACACTGTG	416	685	0	0	0	0
ITGB7	ACTGCACAGTTGACGTGT	171	299	0	0	0	0
ITK	ACTGGTACTGTGGTCACA	148	154	1	0	1	0
ITK	TGTGGTACTGTGGTGTAC	46	50	0	0	0	0
ITK	ACTGGTACTGTGGTGTTG	69	94	0	0	0	0
ITK	CATGGTACTGTGGTCAGT	205	158	0	0	0	0
ITK	CATGCACAGTCAGTCAAC	18	15	0	0	0	0
ITK	CATGCAACACCAACCACA	259	271	1	0	0	0
ITM2B	GTACTGACCAACGTACGT	352	437	0	0	0	0
ITM2B	CAACTGACCAACGTACCA	877	912	0	2	0	3
ITM2B	TGACTGACCAACGTACAC	31	78	0	0	0	0
ITM2B	ACACTGACCAACGTACTG	298	302	0	1	0	0
ITM2B	CAACTGACCAACGTTGGT	571	485	1	0	0	3
ITM2B	GTTGCATGCAGTGTACCA	171	182	0	0	0	0
ITPA	ACTGCATGCAGTTGCAAC	31	76	0	0	1	0
ITPA	TGACACACGTGTTGACAC	52	17	1	0	0	0
ITPA	ACACACACGTGTTGACTG	106	127	0	0	0	0
ITPA	CAACACACGTGTTGTGGT	294	244	0	0	0	2
ITPA	ACACACACGTGTTGTGCA	169	206	0	0	0	0
ITPA	GTACACACGTGTTGTGAC	55	24	0	0	0	0
ITPK1	TGACCAACACTGTGCATG	152	185	0	0	1	0
ITPK1	GTACCAACACTGTGCAAC	37	75	0	0	0	0
ITPK1	ACACCAACACTGTGACGT	240	225	1	0	0	0
ITPK1	TGACCAACACTGTGACCA	155	86	0	0	0	0
ITPK1	CAACCAACACTGTGACAC	30	73	0	0	0	0
ITPKA	TGACGTTGTGACTGCATG	255	194	1	0	0	3
ITPKA	ACACCAACACACTGCATG	187	239	1	0	0	0
ITPKA	CAACCAACACACTGACGT	100	96	0	0	0	0
ITPKA	ACACCAACACACTGACCA	177	222	0	0	0	0
ITPKA	GTACCAACACACTGACAC	94	90	5	0	0	1

ITPKA	TGTGCACAGTTGACGTCA	181	144	0	0	0	0
ITPKB	ACACCAACTGGTGTGTTG	234	192	0	0	0	0
ITPKB	CAACCAACTGGTGTCAGT	241	118	0	0	0	0
ITPKB	ACACCAACTGGTGTCACA	38	59	0	0	0	0
ITPKB	GTACCAACTGGTGTCAAC	33	27	0	0	0	0
ITPKB	CATGGTACTGTGACTGGT	393	162	0	0	0	0
ITPR1	ACACGTCAACCAACGTGT	50	120	1	0	0	14
ITPR1	CAACTGCAACACTGGTCA	312	391	0	0	0	1
ITPR1	TGACTGCAACACTGGTAC	9	26	0	0	0	0
ITPR1	ACACTGCAACACTGGTTG	692	523	2	0	0	0
ITPR1	CAACTGCAACACTGCAGT	56	83	0	1	0	0
ITPR2	ACACTGCAACTGGTGTTG	460	583	102	1	1	26
ITPR2	TGACTGCAACTGGTGTAC	45	46	0	0	0	0
ITPR2	CAACTGCAACTGGTCAGT	171	182	0	0	0	0
ITPR2	ACACTGCAACTGGTCACA	71	95	0	0	0	0
ITPR2	ACTGACCATGTGGTGTCA	372	118	0	0	0	0
ITPR3	TGACGTCAACCAACGTCA	228	149	0	0	0	3
ITPR3	CAACTGCAACTGGTTGTG	446	261	0	0	0	0
ITPR3	TGACTGCAACTGCAGTGT	497	796	0	1	0	0
ITPR3	GTACTGCAACTGCAGTCA	176	208	1	0	0	8
ITPR3	ACACTGCAACTGCAGTAC	49	59	0	0	0	0
ITSN1	ACCATGACGTTGCAACCA	403	347	0	0	0	1
ITSN1	GTCATGACGTTGCAACAC	82	60	0	0	0	0
ITSN1	TGCATGACGTTGCAACTG	64	199	0	0	0	0
ITSN1	ACCATGACGTTGCATGGT	73	83	0	0	0	0
ITSN1	CATGGTTGTGCAGTGTAC	39	16	0	0	0	0
IVD	ACACCAGTCATGGTACCA	310	1047	0	0	1	1
IVD	CAACCAGTCATGGTACGT	237	228	0	0	0	0
IVD	GTACCAGTCATGGTACAC	14	11	0	0	0	0
IVD	TGACCAGTCATGGTACTG	535	1028	0	0	0	1
IVD	ACACCAGTCATGGTTGGT	382	490	1	1	0	0
IVD	TGTGCACAACCATGCACA	68	168	0	0	0	0
JAG1	ACACGTTGTGTGGTGTTG	188	890	1	0	0	0
JAG1	ACACGTTGTGTGGTCACA	59	62	0	0	0	0
JAG1	CAACCACAGTACGTACCA	142	280	2	237	0	0
JAG1	TGACCACAGTACGTACAC	73	105	0	0	0	0
JAG1	ACACCACAGTACGTACTG	343	322	0	0	0	0
JAG1	CATGCACACAACGTTGTG	784	910	2	25	0	1
JAG2	GTACCACAGTCATGTGGT	115	187	0	0	0	0
JAG2	CAACCACAGTCATGTGCA	201	187	0	0	0	0
JAG2	TGACCACAGTCATGTGAC	24	56	0	0	0	0
JAG2	ACACCACAGTCATGTGTG	166	182	0	0	0	1
JAG2	GTTGCAACACCAACTGTG	332	370	0	0	0	2
JAG2	ACTGCAACACCATGGTGT	440	415	0	0	0	8
JAK1	TGCATGACACCACATGCA	64	102	0	0	0	0
JAK1	CACATGACACCACATGAC	134	126	0	0	0	0
JAK1	GTCATGACACCACATGTG	452	393	3	0	0	0
JAK1	ACCATGACACCAACGTGT	154	167	0	0	0	0
JAK1	GTTGGTCACAGTGTTGCA	405	640	0	0	0	1
JAK2	TGCATGACACACCAACGT	492	268	0	1	0	2
JAK2	GTCATGACACACCAACCA	418	445	1	0	0	0
JAK2	ACCATGACACACCAACAC	31	25	0	0	0	0
JAK2	CACATGACACACCAACTG	668	876	1	0	0	0
JAK2	GTCATGACACACCATGGT	585	306	0	0	0	7
JAK2	GTTGCACATGTGCACATG	96	67	0	0	0	0
JAK3	GTCATGCACAACTGGTGT	485	664	0	2	1	0
JAK3	GTCATGCACAACACTGGT	549	427	1	1	0	0
JAK3	CACATGCACAACACTGCA	319	241	0	0	0	0
JAK3	TGCATGCACAACACTGAC	216	167	0	0	0	0
JAK3	ACCATGCACAACACTGTG	213	259	0	1	0	0
JAM2	CATGCAACACCATGGTAC	58	56	0	0	1	1
JAM2	ACACTGGTGTTGTGCAGT	101	103	0	0	0	0
JAM2	TGACTGGTGTTGTGCACA	61	43	0	0	0	0
JAM2	CAACTGGTGTTGTGCAAC	45	34	0	0	0	0
JAM2	GTACTGGTGTTGTGCATG	159	131	0	0	0	0
JAM2	GTTGCAACACCATGGTTG	253	293	1	0	0	1
JAZF1	GTACTGTGACCATGGTGT	588	710	5	0	0	1
JAZF1	CAACTGTGACCATGGTCA	790	386	1	0	0	0
JAZF1	TGACTGTGACCATGGTAC	53	31	0	0	0	0
JAZF1	ACACTGTGACCATGGTTG	461	419	0	0	0	13
JAZF1	CAACTGTGACCATGCAGT	197	106	1	0	0	0
JPH3	GTACTGTGTGCAGTGTTG	808	414	0	0	1	1
JPH3	TGACTGTGTGCAGTCAGT	84	163	0	0	0	0
JPH3	GTACTGTGTGCAGTCACA	108	76	0	0	0	0
JPH3	ACACTGTGTGCAGTCAAC	13	15	0	0	0	0

JPH3	ACTGCACAGTGTGTACTG	321	227	0	1	0	0
JPH3	TGTGCAACACCATGGTCA	265	297	0	0	0	1
JUN	GTACCATGGTTGGTCATG	104	61	0	0	0	0
JUN	TGACCATGGTTGGTACGT	175	312	1	1	0	18
JUN	GTACCATGGTTGGTACCA	449	542	2	1	0	0
JUN	ACACCATGGTTGGTACAC	27	31	0	0	0	0
JUN	GTACCATGACCAACCAGT	281	574	0	0	0	1
JUNB	ACACGTACGTTGCATGAC	102	76	0	0	0	6
JUNB	CAACGTACGTTGCATGTG	287	663	1	0	0	0
JUNB	TGACGTACGTTGACGTGT	276	476	0	0	0	1
JUNB	CATGGTTGTGGTACGTGT	414	411	0	0	0	0
JUNB	CATGGTTGTGGTTGGTCA	591	673	0	2	0	0
JUND	GTACGTACGTTGACTGTG	176	144	0	0	1	0
JUND	ACACGTACGTTGTGGTGT	509	532	0	1	1	2
JUND	CAACGTACGTTGACTGAC	12	23	0	0	0	0
JUND	CATGCAGTACACACCACA	103	130	0	0	0	0
JUND	ACTGACCATGTGACCATG	98	102	0	0	0	0
JUP	ACTGGTGTGTGTTGCATG	370	305	0	1	1	5
JUP	TGTGGTGTGTGTTGCAAC	19	26	0	2	0	0
JUP	CATGGTGTGTGTTGACGT	208	219	0	1	0	0
JUP	ACTGGTGTGTGTTGACCA	100	62	0	0	0	0
JUP	TGTGCAGTCAACGTTGCA	112	110	0	0	0	1
JUP	CATGCACATGACACCACA	79	60	1	0	0	0
KALRN	GTACACCACAACGTACGT	461	228	1	2	1	1
KALRN	CAACACCACAACGTCATG	225	209	0	0	0	0
KALRN	CAACACCACAACGTACCA	464	410	0	0	0	0
KALRN	TGACACCACAACGTACAC	35	24	0	0	0	0
KALRN	ACACACCACAACGTACTG	322	321	0	1	0	1
KARS	GTACACGTACCAACACAC	31	42	0	0	0	2
KARS	TGACACGTACCAACACTG	41	64	0	0	0	0
KARS	ACACACGTACCAACTGGT	250	323	0	0	0	0
KARS	TGACACGTACCAACTGCA	64	61	0	0	0	0
KARS	CAACACGTACCAACTGAC	57	51	0	0	0	0
KARS	GTTGCACAACGTGTTGGT	85	48	1	0	0	0
KATNAL1	ACTGGTGTTGGTCATGGT	122	119	3	0	0	0
KATNAL1	TGTGGTGTTGGTCATGCA	159	236	0	1	0	0
KATNAL1	CATGGTGTTGGTCATGAC	238	129	0	0	0	0
KATNAL1	GTTGGTGTTGGTCATGTG	193	175	0	0	0	0
KATNAL1	ACTGGTGTTGGTACGTGT	59	113	0	0	0	0
KATNB1	ACTGGTGTACTGCAGTGT	862	2291	0	1	2	0
KATNB1	CATGGTGTCAGTGTGTTG	748	1046	3	0	0	1
KATNB1	TGTGGTGTACTGCAGTCA	480	512	0	1	0	5
KATNB1	CATGGTGTACTGCAGTAC	67	55	0	0	0	0
KATNB1	GTTGCACAGTGTACGTGT	1092	1821	3	1	0	0
KCNA4	CAACCATGTGACTGACGT	565	326	0	0	1	5
KCNA4	ACACCATGTGACTGCATG	523	367	0	0	0	0
KCNA4	ACACCATGTGACTGACCA	316	345	0	0	0	0
KCNA4	GTACCATGTGACTGACAC	24	17	0	0	0	0
KCNA4	TGACCATGTGACTGACTG	636	518	0	1	0	0
KCNA4	ACTGCAACTGACTGACCA	95	83	0	0	0	0
KCNA5	GTACCATGTGTGGTGTCA	265	126	0	0	1	0
KCNA5	ACACCATGTGTGGTGTAC	12	20	0	0	0	0
KCNA5	CAACCATGTGTGGTGTTG	251	422	0	0	0	0
KCNA5	GTACCATGTGTGGTCAGT	100	112	0	1	0	0
KCNA5	ACTGCAGTTGGTGTTGGT	441	283	1	0	0	0
KCNA5	GTTGCAACTGACTGACAC	39	57	0	0	0	0
KCNA7	TGACCATGTGTGCACAGT	681	533	1	1	1	4
KCNA7	GTACCATGTGTGCACACA	65	28	0	0	0	0
KCNA7	ACACCATGTGTGCACAAC	57	18	0	0	0	0
KCNA7	CAACCATGTGTGCACATG	21	42	0	0	0	0
KCNA7	GTACCATGTGTGCAACGT	871	400	0	0	0	0
KCNA7	TGTGCAACTGACTGACTG	898	410	0	0	0	0
KCNB1	TGACACGTGTACCAGTGT	638	421	0	9	0	0
KCNB1	GTACACGTGTACCAGTCA	497	227	2	0	0	0
KCNB1	ACACACGTGTACCAGTAC	87	178	0	0	0	0
KCNB1	CAACACGTGTACCAGTTG	606	464	1	1	0	0
KCNB1	GTACACGTGTACCACAGT	168	191	0	0	0	0
KCNB1	TGTGCAACTGTGTGGTAC	55	63	1	0	0	1
KCNC4	ACACACGTGTACACACTG	330	241	30468	22	1	1
KCNC4	CAACACGTGTACACCATG	186	361	0	0	0	0
KCNC4	GTACACGTGTACACACGT	88	46	0	0	0	4
KCNC4	CAACACGTGTACACACCA	42	46	0	0	0	2
KCNC4	TGACACGTGTACACACAC	25	32	0	0	0	0
KCNE1	TGACACGTGTTGGTGTTG	1123	738	1	1	1	0
KCNE1	ACACACGTGTACTGTGTG	110	44	0	0	0	0

KCNE1	CAACACGTGTTGGTGTGT	41	77	0	0	0	0
KCNE1	ACACACGTGTTGGTGTCA	104	141	2	0	0	0
KCNE1	GTACACGTGTTGGTGTAC	40	147	0	0	0	0
KCNE2	GTACACGTGTACTGTGGT	741	1004	2	2	1	0
KCNE2	CAACACGTGTACTGACTG	290	281	0	0	0	0
KCNE2	CAACACGTGTACTGTGCA	502	236	0	0	0	1
KCNE2	TGACACGTGTACTGTGAC	31	24	0	0	0	0
KCNE2	CAACTGACTGCATGCATG	220	486	1	0	0	0
KCNE2	ACTGCAACTGTGTGGTTG	487	641	1	0	0	120
KCNG2	TGTGCAGTTGGTCAGTCA	760	862	0	3	1	0
KCNG2	GTACCATGTGTGACTGGT	303	551	1	0	0	0
KCNG2	CAACCATGTGTGACTGCA	257	137	1	0	0	0
KCNG2	ACTGACACGTTGCAACCA	354	350	0	1	0	0
KCNG2	GTTGACACGTTGCAACAC	9	18	0	0	0	0
KCNH2	GTACGTTGACACACACGT	47	29	0	0	0	0
KCNH2	CAACGTTGACACACACCA	164	185	0	0	0	0
KCNH2	TGACGTTGACACACACAC	13	23	0	0	0	0
KCNH2	ACACGTTGACACACACTG	258	375	0	0	0	0
KCNH2	CAACGTTGACACACTGGT	754	622	0	0	0	6
KCNH2	GTTGCAACTGTGGTACAC	34	70	0	0	0	0
KCNH7	GTACGTTGACGTTGTGCA	488	218	2	0	2	2
KCNH7	TGACGTTGACGTTGTGGT	131	44	0	1	0	0
KCNH7	ACACGTTGACGTTGTGAC	66	24	0	0	0	0
KCNH7	GTTGCATGGTGTACCACA	73	50	0	0	0	0
KCNH7	ACTGCATGGTGTACCAAC	38	30	0	0	0	0
KCNH7	CATGCATGGTGTACCATG	141	68	2	0	0	0
KCNIP3	GTACGTACACACGTTGTG	91	122	0	0	0	0
KCNIP3	ACACGTACACACCAGTGT	321	277	0	0	0	0
KCNIP3	TGACGTACACACCAGTCA	107	140	0	0	0	0
KCNIP3	CAACGTACACACCAGTAC	48	35	0	0	0	0
KCNIP3	GTACGTACACACCAGTTG	400	318	1	0	0	1
KCNJ11	ACACACGTGTGTTGCACA	25	37	0	0	0	0
KCNJ11	GTACACGTGTGTTGCAAC	22	12	0	0	0	0
KCNJ11	TGACACGTGTGTTGCATG	130	356	0	0	0	0
KCNJ11	ACACACGTGTGTTGACGT	349	82	0	0	0	0
KCNJ11	TGACACGTGTGTTGACCA	277	273	4	0	0	0
KCNJ12	ACACACGTGTGTTGTGAC	28	83	0	0	0	0
KCNJ12	CAACACGTGTGTTGTGTG	504	235	0	0	0	1
KCNJ12	ACACACGTGTCAGTGTGT	736	617	2	0	0	0
KCNJ12	TGACACGTGTCAGTGTCA	255	97	0	0	0	0
KCNJ12	CAACACGTGTCAGTGTAC	36	51	34	0	0	0
KCNJ2	GTACCATGTGTGACCATG	278	398	0	0	0	1
KCNJ2	TGACCATGTGTGACACGT	81	120	0	0	0	0
KCNJ2	GTACCATGTGTGACACCA	620	523	2	2	0	6
KCNJ2	ACACCATGTGTGACACAC	28	33	0	0	0	0
KCNJ2	CAACCATGTGTGACACTG	190	195	0	0	0	0
KCNJ2	GTTGCAACACACACCATG	102	83	0	0	0	0
KCNJ3	GTACCATGTGTGTGACTG	822	381	1	1	1	0
KCNJ3	TGACCATGTGTGTGCATG	638	631	0	0	0	0
KCNJ3	ACACCATGTGTGTGACGT	272	84	1	0	0	0
KCNJ3	TGACCATGTGTGTGACCA	368	436	1	0	0	1
KCNJ3	CAACCATGTGTGTGACAC	32	68	0	0	0	0
KCNJ3	GTTGCAACTGACCAACCA	391	176	0	0	0	0
KCNJ4	GTACACGTGTGTGTGTCA	300	333	0	737	0	0
KCNJ4	ACACACGTGTGTGTGTAC	55	72	0	0	0	0
KCNJ4	CAACACGTGTGTGTGTTG	391	275	1	1	0	0
KCNJ4	GTACACGTGTGTGTCAGT	83	96	0	0	0	0
KCNJ4	CAACACGTGTGTGTCACA	56	97	0	0	0	0
KCNJ4	GTTGCAACTGGTGTGTAC	17	17	0	0	0	0
KCNJ5	CATGCAACTGACCAACTG	427	389	2	0	1	1
KCNJ5	TGCATGTGACTGGTGTAC	24	21	0	0	0	16
KCNJ5	GTTGCAGTACCAGTCAGT	36	56	0	0	0	0
KCNJ5	CATGCAGTACCAGTCACA	59	62	0	0	0	0
KCNJ5	TGTGCAGTACCAGTCAAC	37	48	0	0	0	0
KCNJ5	ACTGCAACTGACCAACAC	12	23	0	0	0	0
KCNJ9	ACTGCAACTGACCATGTG	349	348	0	0	1	0
KCNJ9	TGACACGTGTGTACACGT	150	93	0	1	0	0
KCNJ9	GTACACGTGTGTACACCA	116	66	0	0	0	0
KCNJ9	ACACACGTGTGTACACAC	44	31	0	0	0	0
KCNJ9	GTTGCAGTTGGTGTTGTG	360	399	1	0	0	3
KCNJ9	GTTGCAACTGACACGTGT	175	137	0	0	0	0
KCNK1	TGACACGTGTACGTGTAC	275	43	0	0	0	0
KCNK1	ACACACGTGTACGTGTTG	644	437	1	1	0	1
KCNK1	CAACACGTGTACGTCAGT	260	232	1	0	0	1
KCNK1	ACACACGTGTACGTCACA	108	78	0	0	0	1

KCNK1	GTACACGTGTACGTCAAC	19	18	0	0	0	0
KCNK1	TGTGCAACGTCATGCAAC	25	17	0	0	0	0
KCNK3	TGACCATGTGACGTCAGT	246	149	1	0	1517	1
KCNK3	CAACCATGTGACGTGTAC	59	67	0	0	0	0
KCNK3	GTACCATGTGACGTGTTG	220	248	0	0	0	0
KCNK3	GTACCATGTGACGTCACA	64	126	0	0	0	0
KCNK3	CAACTGACTGCATGACCA	333	454	0	1	0	81
KCNK3	CATGCACAGTTGACGTAC	60	44	0	0	0	0
KCNMA1	ACCATGCACACAGTTGTG	276	373	1	0	1	0
KCNMA1	CACATGCACACAGTTGCA	149	101	1	0	0	0
KCNMA1	TGCATGCACACAGTTGAC	75	52	0	0	0	0
KCNMA1	GTCATGCACACACAGTGT	672	619	0	0	0	0
KCNMA1	CACATGCACACACAGTCA	142	176	1	0	0	0
KCNMA1	ACTGCAACTGTGGTTGGT	27	93	0	0	0	0
KCNMB1	ACACCATGTGACGTACTG	1044	448	1	1	0	0
KCNMB1	CAACCATGTGACGTTGGT	332	312	1	0	0	0
KCNMB1	ACACCATGTGACGTTGCA	33	51	0	0	0	0
KCNMB1	GTACCATGTGACGTTGAC	20	16	0	0	0	0
KCNMB1	TGACCATGTGACGTTGTG	178	103	0	0	0	0
KCNMB1	TGTGCAACTGACACTGGT	140	231	1	0	0	0
KCNMB2	TGACACGTGTTGCAGTAC	30	23	1	0	0	0
KCNMB2	ACACACGTGTTGCAGTTG	184	202	0	0	0	0
KCNMB2	CAACACGTGTTGCACAGT	606	380	1	0	0	6
KCNMB2	ACACACGTGTTGCACACA	9	8	0	0	0	0
KCNMB2	GTACACGTGTTGCACAAC	37	64	0	0	0	3
KCNMB2	TGTGCATGGTTGACCATG	82	112	0	0	0	0
KCNMB3	ACACACGTGTGTGTACCA	103	54	0	0	1	0
KCNMB3	TGACACGTGTGTGTCAAC	18	9	0	0	0	0
KCNMB3	ACACACGTGTGTGTCATG	47	34	0	1	0	0
KCNMB3	CAACACGTGTGTGTACGT	158	140	0	0	0	0
KCNMB3	GTACACGTGTGTGTACAC	2	1	0	0	0	0
KCNMB3	ACTGCAACTGACTGTGGT	345	308	0	2	0	0
KCNMB4	TGACACGTGTGTCATGTG	144	188	0	0	0	0
KCNMB4	CAACACGTGTGTACGTGT	242	327	0	0	0	0
KCNMB4	ACACACGTGTGTACGTCA	113	131	0	0	0	0
KCNMB4	GTACACGTGTGTACGTAC	47	55	0	0	0	0
KCNMB4	ACTGCAGTCACAACCATG	287	301	0	0	0	0
KCNMB4	TGTGCAACTGTGGTACTG	74	141	0	0	0	0
KCNN3	TGACCATGTGACCAGTTG	668	721	0	0	1	0
KCNN3	ACACCATGTGACCACAGT	141	173	0	0	0	0
KCNN3	TGACCATGTGACCACACA	41	80	0	0	0	0
KCNN3	CAACCATGTGACCACAAC	26	30	0	0	0	0
KCNN3	GTACCATGTGACCACATG	83	91	0	0	0	0
KCNN4	ACACCATGTGACACGTTG	269	547	0	0	1	1
KCNN4	TGACCATGTGACACGTAC	88	92	0	0	0	0
KCNN4	CAACCATGTGACACCAGT	481	595	1	0	0	0
KCNN4	ACACCATGTGACACCACA	531	337	0	0	0	0
KCNN4	GTACCATGTGACACCAAC	37	55	0	0	0	0
KCNN4	GTTGCAACTGACACTGCA	306	204	0	0	0	0
KCNQ1	TGACCATGTGACACCATG	338	297	1	0	0	0
KCNQ1	ACACCATGTGACACACGT	139	116	0	0	0	0
KCNQ1	TGACCATGTGACACACCA	12	33	0	0	0	0
KCNQ1	CAACCATGTGACACACAC	21	26	0	0	0	0
KCNQ1	TGTGCAGTGTACGTCAGT	171	166	2	1	0	41
KCNQ1	ACTGCATGCACATGGTCA	177	268	1	0	0	0
KCNQ2	GTACCATGTGACACACTG	248	274	0	0	1	0
KCNQ2	TGACCATGTGACACTGGT	232	291	0	0	0	0
KCNQ2	GTACCATGTGACACTGCA	335	164	1	0	0	0
KCNQ2	ACACCATGTGACACTGAC	81	117	0	0	0	0
KCNQ2	CAACCATGTGACACTGTG	287	654	0	1	0	0
KCNQ2	ACTGCAACTGTGGTACCA	271	231	6910	5	0	5
KCNQ5	GTACACGTGTCATGGTCA	160	173	0	0	1	0
KCNQ5	CAACACGTGTCAACTGTG	3	55	0	0	0	0
KCNQ5	TGACACGTGTCATGGTGT	471	1356	1	0	0	19
KCNQ5	ACACACGTGTCATGGTAC	39	58	0	0	0	0
KCNQ5	CAACACGTGTCATGGTTG	488	404	1	1	0	3
KCNQ5	GTTGCATGGTCAGTGTCA	296	278	0	0	0	0
KDELR1	CAACACTGGTTGCATGAC	156	128	1	0	0	0
KDELR1	ACACTGACGTTGGTCAAC	6	13	0	0	0	0
KDELR1	CAACTGACGTTGGTCATG	98	84	0	0	0	0
KDELR1	GTACTGACGTTGGTACGT	329	190	0	0	0	0
KDELR1	CAACTGACGTTGGTACCA	147	95	0	0	0	0
KDELR1	ACTGCATGCACAACGTGT	384	724	0	0	0	0
KDELR2	TGACTGACGTTGCAACGT	473	311	0	0	0	0
KDELR2	GTACTGACGTTGCAACCA	34	81	0	0	0	0

KDELR2	ACACTGACGTTGCAACAC	21	127	0	0	0	0
KDELR2	CAACTGACGTTGCAACTG	598	293	0	0	0	0
KDELR2	GTACTGACGTTGCATGGT	666	774	0	0	0	14
KDELR3	TGACTGACGTTGACCATG	517	539	1	1	0	0
KDELR3	ACACTGACGTTGACACGT	4	2	0	0	0	0
KDELR3	TGACTGACGTTGACACCA	290	281	1	0	0	0
KDELR3	CAACTGACGTTGACACAC	51	48	0	0	0	0
KDELR3	GTACTGACGTTGACACTG	66	40	0	0	0	0
KDELR3	TGTGCACATGGTGTGTAC	104	61	0	0	0	0
KDR	CACATGACGTCAACGTTG	117	137	0	0	0	0
KDR	GTCATGACGTCAACCAGT	137	324	0	0	0	0
KDR	CACATGACGTCAACCACA	49	44	0	0	0	0
KDR	TGCATGACGTCAACCAAC	9	5	0	0	0	0
KDR	ACCATGACGTCAACCATG	154	210	1	0	0	0
KDR	GTTGCACATGACGTTGGT	352	288	0	0	0	0
KEAP1	ACTGGTACGTTGCAACAC	29	32	0	0	0	0
KEAP1	CATGGTACGTTGCAACTG	451	472	0	1	0	0
KEAP1	GTTGGTACGTTGCATGGT	152	291	1	0	0	0
KEAP1	CATGGTACGTTGCATGCA	763	123	0	0	0	2
KEAP1	TGTGGTACGTTGCATGAC	80	85	0	0	0	0
KERA	ACACTGTGTGTGACCATG	58	70	1	0	0	0
KERA	CAACTGTGTGTGACACGT	190	72	0	0	0	0
KERA	ACACTGTGTGTGACACCA	102	85	0	0	0	0
KERA	GTACTGTGTGTGACACAC	40	41	0	0	0	0
KERA	TGACTGTGTGTGACACTG	272	299	0	0	0	0
KHK	TGACCAACTGGTGTCATG	332	170	1	0	0	2
KHK	ACACCAACTGGTGTACGT	448	255	0	0	0	0
KHK	GTTGGTTGGTCAACACGT	20	69	0	0	0	0
KHK	CATGGTTGGTCAACACCA	202	251	0	0	0	0
KHK	TGTGGTTGGTCAACACAC	6	5	0	0	0	0
KHK	GTTGCACAACCAGTACTG	140	148	1	0	0	0
KIAA1303	GTACCATGCAGTACTGAC	89	64	0	0	1	0
KIAA1303	ACACCATGCAGTACACTG	241	265	0	0	0	0
KIAA1303	CAACCATGCAGTACTGGT	445	420	0	0	0	1
KIAA1303	ACACCATGCAGTACTGCA	453	205	0	0	0	0
KIAA1303	TGACCATGCAGTACTGTG	53	104	0	0	0	0
KIAA1394	CATGCAGTGTCATGCAAC	8	82	0	0	0	0
KIAA1394	GTTGCAGTGTCATGCATG	155	186	0	0	0	0
KIAA1394	TGTGCAGTGTCATGACGT	216	186	0	0	0	0
KIAA1394	GTTGCAGTGTCATGACCA	72	38	1	0	0	0
KIAA1394	ACTGCAGTGTCATGACAC	26	15	0	0	0	0
KIF11	TGTGTGACACACACACTG	339	372	0	0	0	0
KIF11	ACTGTGACACACACTGGT	243	305	0	0	0	0
KIF11	TGTGTGACACACACTGCA	195	167	0	0	0	0
KIF11	ACTGTGACACACTGGTGT	536	358	1	0	0	0
KIF11	TGTGTGACACACTGGTCA	189	216	0	0	0	0
KIF11	CATGTGACACACTGGTAC	24	35	0	0	0	0
KIF11	GTTGTGACACACTGCACA	27	17	0	0	0	0
KIF11	ACTGTGACACACTGCAAC	3	6	0	0	0	0
KIF11	CATGTGACACACTGCATG	10	37	0	0	0	0
KIF11	TGTGTGACACACTGACAC	131	31	0	0	0	0
KIF11	ACTGTGACACACTGACTG	397	458	1	0	0	0
KIF11	CATGTGACACACTGTGGT	685	932	0	0	0	1
KIF11	CATGTGACTGGTGTCACA	46	113	0	0	0	0
KIF11	TGTGTGACTGGTGTCAAC	23	61	0	0	0	0
KIF11	ACTGTGACTGGTGTCATG	501	324	0	0	0	1
KIF11	GTTGTGACTGGTGTACAC	12	9	0	0	0	0
KIF11	TGTGTGACTGGTGTACTG	156	125	0	0	0	1
KIF11	ACTGTGACTGGTGTTGGT	72	62	0	0	0	0
KIF11	GTTGTGACTGGTGTTGTG	150	683	0	1	0	0
KIF11	ACTGTGACTGGTCAGTGT	236	546	0	0	0	0
KIF11	TGTGTGACTGGTCAGTCA	116	143	0	0	0	0
KIF3A	ACTGGTGTTGGTACCAAC	3	4	0	0	0	0
KIF3A	CATGGTGTTGGTACCATG	23	52	0	0	0	0
KIF3A	GTTGGTGTTGGTACACGT	387	349	0	0	0	5
KIF3A	CATGGTGTTGGTACACCA	68	43	0	0	0	0
KIF3A	CATGGTACACTGGTGTGT	613	684	1	0	0	1
KIF5B	ACTGGTGTCAGTGTCATG	253	484	0	0	1	410
KIF5B	TGTGGTGTACCAGTACCA	130	320	1	0	0	1
KIF5B	CATGGTGTACCAGTACAC	172	194	0	1	0	0
KIF5B	GTTGGTGTACCAGTACTG	82	75	0	0	0	0
KIF5B	TGTGGTGTACCAGTTGGT	1063	507	1	3	0	10
KIF5B	CATGCACACATGCACAGT	37	63	0	0	0	0
KIT	ACCATGCACAACTGGTTG	1282	805	0	1	1	1
KIT	CACATGCACAACTGGTCA	699	901	2	0	0	1

KIT	TGCATGCACAACTGGTAC	117	170	0	1	0	0
KIT	CACATGCACAACTGCAGT	162	62	0	0	0	0
KIT	ACCATGCACAACTGCACA	181	250	0	0	0	0
KIT	TGTGCACACAACCAGTGT	433	552	1	0	0	1
KITLG	TGACTGCAGTACACGTCA	123	175	1	0	0	0
KITLG	CAACTGCAGTACACGTAC	21	58	0	0	0	0
KITLG	GTACTGCAGTACACGTTG	198	171	0	1	0	0
KITLG	TGACTGCAGTACACCAGT	28	20	0	0	0	0
KITLG	GTACTGCAGTACACCACA	108	115	0	0	0	0
KL	ACTGGTTGCATGGTACGT	126	160	0	0	0	0
KL	TGTGGTTGCATGGTACCA	70	162	0	0	0	0
KL	CATGGTTGCATGGTACAC	12	11	0	0	0	0
KL	GTTGGTTGCATGGTACTG	95	120	0	0	0	1
KL	TGTGGTTGCATGGTTGGT	159	380	0	0	0	0
KLC1	GTTGGTTGCATGGTTGCA	182	120	2	0	0	0
KLC1	ACTGGTTGCATGGTTGAC	19	12	0	0	0	0
KLC1	CATGGTTGCATGGTTGTG	1041	578	4	1	0	4
KLC1	TGTGGTTGCATGCAGTGT	717	1316	0	0	0	0
KLC1	GTTGGTTGCATGCAGTCA	55	35	0	0	0	0
KLF10	CAACGTTGACGTGTCACA	188	145	0	1	0	1
KLF10	TGACGTTGACGTGTCAAC	36	44	0	0	0	0
KLF10	ACACGTTGACGTGTCATG	55	113	0	0	0	0
KLF10	CAACGTTGACGTGTACGT	227	182	0	0	0	0
KLF10	ACACGTTGACGTGTACCA	91	104	0	0	0	0
KLF10	ACTGCACAACGTCAACGT	159	245	1	0	0	0
KLF11	GTACGTCAACACCATGGT	196	183	0	0	0	0
KLF11	CAACGTCAACACCATGCA	208	227	0	0	0	2
KLF11	TGACGTCAACACCATGAC	52	93	0	0	0	0
KLF11	CATGCAGTGTACACACAC	58	123	1	0	0	0
KLF11	GTTGCAGTGTACACACTG	264	259	0	0	0	1
KLF2	ACACGTTGCAACACCACA	200	134	0	1	0	1
KLF2	GTACGTTGCAACACCAAC	126	139	0	0	0	0
KLF2	TGACGTTGCAACACCATG	120	135	0	0	0	0
KLF2	ACACGTTGCAACACACGT	192	83	0	0	0	0
KLF2	CATGCAGTACTGGTGTAC	102	116	0	0	0	0
KLK1	CAACACCAGTACGTGTGT	135	131	0	0	0	0
KLK1	ACACACCAGTACGTGTCA	243	285	0	0	0	0
KLK1	GTACACCAGTACGTGTAC	59	35	0	0	0	0
KLK1	TGACACCAGTACGTGTTG	217	328	0	0	0	0
KLK1	ACACACCAGTACGTCAGT	88	122	2	0	0	0
KLK1	TGTGCAACACACGTACTG	298	218	2	0	0	0
KLK10	GTACACACCACAACCAGT	136	145	0	0	0	0
KLK10	CAACACACCACAACCACA	140	270	0	0	0	0
KLK10	TGACACACCACAACCAAC	41	35	0	0	0	0
KLK10	ACACACACCACAACCATG	193	187	1	0	0	0
KLK10	CAACACACCACAACACGT	73	59	0	0	0	0
KLK2	GTCATGTGACCAGTTGGT	461	810	0	0	1	1
KLK2	TGCATGTGACCAGTACGT	195	318	0	0	0	0
KLK2	GTCATGTGACCAGTACCA	328	585	0	0	0	0
KLK2	ACCATGTGACCAGTACAC	54	52	0	0	0	0
KLK2	CACATGTGACCAGTACTG	256	254	1	0	0	0
KLK6	GTACACACACCATGCAAC	224	59	0	0	0	0
KLK6	TGACACACACCATGCATG	451	452	8	1	0	5
KLK6	ACACACACACCATGACGT	95	108	0	0	0	0
KLK6	TGACACACACCATGACCA	485	585	1	1	0	0
KLK6	GTTGCAGTTGCAACGTGT	506	459	0	1	0	1
KLK7	GTACACCAGTGTACCACA	86	92	0	0	0	0
KLK7	ACACACCAGTGTACCAAC	24	53	0	0	0	0
KLK7	CAACACCAGTGTACCATG	213	280	2	2	0	0
KLK7	GTACACCAGTGTACACGT	52	49	0	0	0	0
KLK7	CAACACCAGTGTACACCA	40	90	1	0	0	0
KLKB1	ACACACACGTCAACACTG	219	268	0	0	1	0
KLKB1	TGACACACGTCAACACAC	23	25	0	0	0	0
KLKB1	CAACACACGTCAACTGGT	132	148	0	0	0	0
KLKB1	ACACACACGTCAACTGCA	194	233	0	0	0	6
KLKB1	GTACACACGTCAACTGAC	6	46	0	0	0	0
KLRA1	TGACACTGTGTGCACACA	176	162	0	0	1	2
KLRA1	TGACACTGTGTGCAACGT	1196	419	0	0	1	1
KLRA1	CAACACTGTGTGCACAAC	36	42	0	0	0	0
KLRA1	GTACACTGTGTGCACATG	157	250	0	0	0	9
KLRA1	GTACACTGTGTGCAACCA	191	172	0	0	0	0
KLRC1	ACACACTGTGACTGGTGT	632	796	1	0	0	0
KLRC1	TGACACTGTGACTGGTCA	398	408	3	0	0	0
KLRC1	CAACACTGTGACTGGTAC	48	69	0	0	0	0
KLRC1	GTACACTGTGACTGGTTG	149	193	0	0	0	0

KLRC1	ACACACTGTGTGGTGTGT	66	56	0	0	0	0
KLRC2	GTACACTGTGACTGTGAC	57	44	0	0	0	0
KLRC2	TGACACTGTGTGGTGTCA	202	260	1	0	0	0
KLRC2	CAACACTGTGTGGTGTAC	90	124	1	0	0	2
KLRC2	GTACACTGTGTGGTGTTG	253	278	1	0	0	0
KLRC2	TGACACTGTGTGGTCAGT	344	315	0	0	0	0
KLRC3	TGACACTGTGACTGTGTG	1099	883	2	1	0	0
KLRC3	GTTGACCACATGACGTTG	750	844	1	4	0	0
KLRC3	TGTGACCACATGACCAGT	481	665	1	0	0	0
KLRC3	GTTGACCACATGACCACA	32	47	0	0	0	0
KLRC3	ACTGACCACATGACCAAC	27	28	0	0	0	0
KLRC4	CAACACTGTGACTGACCA	349	256	0	1	0	1
KLRC4	TGACACTGTGACTGACAC	84	92	0	1	0	0
KLRC4	ACACACTGTGACTGACTG	1016	1215	0	4	0	2
KLRC4	CAACACTGTGACTGTGGT	964	195	16	0	0	0
KLRC4	ACTGACACGTGTCACAGT	338	186	0	0	0	2
KLRD1	GTACACTGTGTGGTCACA	106	92	0	0	0	0
KLRD1	ACACACTGTGTGGTCAAC	11	15	0	0	0	0
KLRD1	CAACACTGTGTGGTCATG	95	205	0	0	0	0
KLRD1	CATGCACAACTGCACATG	116	131	0	1	0	0
KLRD1	GTTGCACAACTGCAACGT	44	31	0	0	0	1
KLRD1	CATGCACAACTGCAACCA	146	166	0	0	0	0
KLRK1	GTACACTGTGTGGTACGT	191	133	1	0	1	0
KLRK1	ACACACTGTGACTGTGCA	233	262	0	0	0	0
KLRK1	CAACACTGTGTGGTACCA	108	109	0	0	0	0
KLRK1	TGACACTGTGTGGTACAC	33	44	0	0	0	0
KLRK1	ACACACTGTGTGGTACTG	227	202	0	0	0	3
KLRK1	TGTGCACAACACGTTGTG	252	264	1	0	0	0
KMO	ACACTGACACCAGTGTGT	269	252	0	0	0	3
KMO	TGACTGACACCAGTGTCA	351	329	1	0	0	1
KMO	CAACTGACACCAGTGTAC	79	78	0	0	0	0
KMO	GTACTGACACCAGTGTTG	807	1321	0	1	0	0
KMO	TGACTGACACCAGTCAGT	380	551	0	1	0	0
KNG1	GTACTGTGGTCAGTGTGT	965	1154	2	0	1	1
KNG1	ACACTGTGGTGTTGTGCA	23	37	0	0	0	0
KNG1	GTACTGTGGTGTTGTGAC	72	180	0	0	0	0
KNG1	TGACTGTGGTGTTGTGTG	162	214	0	1	0	19
KNG1	CAACTGTGGTCAGTGTCA	248	59	1	0	0	0
KPNA2	GTACTGACTGCACACAAC	31	44	0	0	0	0
KPNA2	TGACTGACTGCACACATG	261	290	2	0	0	2
KPNA2	ACACTGACTGCACAACGT	270	356	0	0	0	0
KPNA2	TGACTGACTGCACAACCA	86	119	0	0	0	0
KPNA2	ACTGCAGTCAGTTGACCA	314	418	0	1	0	1
KPNA3	GTACTGACTGCACAACTG	503	811	1	1	1	1
KPNA3	CAACTGACTGCACAACAC	30	12	0	0	0	0
KPNA3	TGACTGACTGCACATGGT	517	456	4	2	0	1
KPNA3	GTACTGACTGCACATGCA	102	135	0	0	0	0
KPNA3	ACACTGACTGCACATGAC	510	126	2	1031	0	0
KPNA6	ACACTGACTGGTACGTTG	613	564	0	2	0	0
KPNA6	CAACTGACTGGTACCAGT	152	104	0	0	0	0
KPNA6	ACACTGACTGGTACCACA	108	188	0	0	0	0
KPNA6	GTACTGACTGGTACCAAC	36	53	0	0	0	0
KPNA6	ACTGCAGTCAGTCATGTG	346	471	0	0	0	13
KPNA6	TGTGCAACTGACCAGTTG	407	538	1	0	0	0
KPNB1	GTTGGTCACACAACTGGT	98	46	0	0	0	0
KPNB1	CATGGTCACACAACTGCA	24	42	0	0	0	0
KPNB1	TGTGGTCACACAACTGAC	46	22	0	0	0	0
KPNB1	ACTGGTCACACAACTGTG	29	73	0	0	0	0
KPNB1	CATGCAGTCAACGTGTTG	118	129	0	0	0	0
KPNB1	ACTGCAACTGACCACAGT	174	134	0	0	0	0
KRAS	GTACCAGTTGTGTGACCA	66	39	0	0	0	1
KRAS	ACACCAGTTGTGTGACAC	4	8	0	0	0	0
KRAS	CAACCAGTTGTGTGACTG	281	636	1	1	0	0
KRAS	GTACCAGTTGTGTGTGGT	676	422	0	1	0	0
KRAS	CAACCAGTTGTGTGTGCA	278	122	1	0	0	0
KREMEN1	GTACTGTGGTACCAGTAC	35	26	0	0	0	0
KREMEN1	TGACTGTGGTACCAGTTG	500	1058	0	0	0	1
KREMEN1	ACACTGTGGTACCACAGT	160	71	0	0	0	0
KREMEN1	TGACTGTGGTACCACACA	64	108	0	0	0	0
KREMEN1	CAACTGTGGTACCACAAC	45	26	0	0	0	0
KREMEN1	CATGCAACTGGTTGCATG	25	36	0	0	0	0
KRT1	GTACTGCAACGTTGACAC	43	62	0	0	0	0
KRT1	TGACTGCAACGTTGACTG	83	133	1	0	0	0
KRT1	ACACTGCAACGTTGTGGT	69	129	0	1	0	0
KRT1	TGACTGCAACGTTGTGCA	201	160	0	0	0	2

KRT1	CATGGTGTTGACCAGTCA	127	145	0	0	0	0
KRT17	GTACTGTGACTGACCAAC	6	18	0	0	0	0
KRT17	TGACTGTGACTGACCATG	137	150	0	0	0	0
KRT17	ACACTGTGACTGACACGT	138	269	0	0	0	0
KRT17	TGACTGTGACTGACACCA	454	283	0	1	0	5
KRT17	CAACTGTGACTGACACAC	23	18	0	0	0	0
KRT17	GTTGCACACAACCAGTCA	408	46	3	0	0	0
KRT18	CAACTGCATGTGTGTGTG	376	719	1	0	0	0
KRT18	GTACTGACGTGTGTGTGT	317	661	0	2	0	0
KRT18	CAACTGACGTGTGTGTCA	184	248	1	1	0	4
KRT18	TGACTGACGTGTGTGTAC	65	98	0	0	0	0
KRT18	ACACTGACGTGTGTGTTG	208	193	0	0	0	0
KRT19	ACACTGCATGTGTGCACA	102	169	0	0	1	0
KRT19	ACACTGCATGTGTGGTTG	186	272	0	0	0	5
KRT19	CAACTGCATGTGTGCAGT	106	82	0	0	0	1
KRT19	GTACTGCATGTGTGCAAC	1	6	0	0	0	0
KRT19	TGACTGCATGTGTGCATG	393	235	1	1	0	1
KRT7	ACACTGACGTGTGTACGT	67	61	0	0	0	0
KRT7	TGACTGTGACTGTGGTGT	1051	882	3	0	0	23
KRT7	CATGGTGTGTACACACTG	450	545	0	1	0	0
KRT7	GTTGGTGTACTGGTACAC	21	14	0	0	0	0
KRT7	TGTGGTGTACTGGTACTG	162	770	1	0	0	0
KRT8	CAACTGACGTGTGTCAGT	122	101	0	0	0	0
KRT8	ACACTGACGTGTGTCACA	97	50	0	0	0	0
KRT8	GTACTGACGTGTGTCAAC	20	26	0	0	0	0
KRT8	TGACTGACGTGTGTCATG	131	137	0	0	0	0
KRT8	GTTGGTGTTGCAGTGTCA	137	141	0	0	0	0
KTN1	CAACTGACGTTGTGACGT	173	325	0	0	1	0
KTN1	ACACTGACGTTGTGACCA	385	205	0	0	0	0
KTN1	GTACTGACGTTGTGACAC	21	19	0	0	0	0
KTN1	TGACTGACGTTGTGACTG	154	216	0	1	0	0
KTN1	ACACTGACGTTGTGTGGT	40	80	1	0	0	0
L1CAM	GTTGCACATGTGACACTG	294	519	7	4	4	8
L1CAM	TGACTGACCACAACTGTG	1123	1074	201	1	0	6
L1CAM	CAACTGACCACATGGTGT	681	857	0	0	0	2
L1CAM	ACACTGACCACATGGTCA	44	38	1	0	0	0
L1CAM	GTACTGACCACATGGTAC	250	225	0	1	0	0
L1CAM	TGACTGACCACATGGTTG	630	779	1	0	0	0
LALBA	GTACACTGCAACACGTTG	305	483	1	0	0	0
LALBA	TGACACTGCAACACCAGT	136	119	2	0	0	0
LALBA	GTACACTGCAACACCACA	83	170	0	0	0	0
LALBA	ACACACTGCAACACCAAC	32	49	0	0	0	0
LALBA	CAACACTGCAACACCATG	279	256	0	0	0	0
LAMA1	GTTGGTCAGTTGTGACGT	146	87	1	0	0	0
LAMA1	CATGGTCAGTTGTGACCA	167	45	0	0	0	0
LAMA1	TGTGGTCAGTTGTGACAC	59	99	0	0	0	0
LAMA1	CATGACACGTTGCACACA	46	37	0	0	0	0
LAMA1	TGTGACACGTTGCACAAC	3	6	0	0	0	0
LAMB1	ACACTGTGTGTGGTGTCA	135	193	0	0	0	0
LAMB1	GTACTGTGTGTGGTGTAC	55	79	0	0	0	0
LAMB1	TGACTGTGTGTGGTGTTG	280	305	0	0	0	0
LAMB1	ACACTGTGTGTGGTCAGT	107	147	0	0	0	0
LAMB1	TGACTGTGTGTGGTCACA	257	76	0	0	0	0
LAMB1	GTTGCACAGTTGACGTTG	1068	540	1	1	0	1
LAMC1	GTTGGTCAGTTGTGGTTG	873	1043	11	10	7	16
LAMC1	GTTGCACATGGTTGACCA	443	205	0	0	1	0
LAMC1	TGTGGTCAGTTGTGCAGT	362	323	2	0	0	0
LAMC1	GTTGGTCAGTTGTGCACA	258	153	0	0	0	0
LAMC1	ACTGGTCAGTTGTGCAAC	36	49	2	0	0	0
LAMC1	CATGGTCAGTTGTGCATG	183	135	1	0	0	0
LAMP1	CAACCAGTACCATGCACA	372	150	1	2	0	3
LAMP1	TGACCAGTACCATGCAAC	31	43	0	0	0	0
LAMP1	ACACCAGTACCATGCATG	755	507	0	1	0	1
LAMP1	CAACCAGTACCATGACGT	463	228	0	0	0	1
LAMP1	ACACCAGTACCATGACCA	438	318	5	0	0	17
LAMP1	TGTGCACAACTGCAACAC	44	51	0	0	0	0
LAMP2	TGACCAGTACCATGGTGT	716	528	0	1	1	0
LAMP2	GTACCAGTACCATGGTCA	84	174	0	0	0	0
LAMP2	ACACCAGTACCATGGTAC	130	75	0	0	0	0
LAMP2	CAACCAGTACCATGGTTG	71	62	0	0	0	0
LAMP2	GTACCAGTACCATGCAGT	445	319	1	0	0	0
LAMP2	TGTGCACAGTTGACCAGT	227	139	1	0	0	0
LAP3	ACACACACGTTGCAACTG	725	759	2	0	1	433
LAP3	CAACACACGTTGCATGGT	428	459	0	1	0	0
LAP3	ACACACACGTTGCATGCA	330	586	1	0	0	1

LAP3	GTACACACGTTGCATGAC	69	92	0	0	0	0
LAP3	TGACACACGTTGCATGTG	504	353	1	0	0	0
LARS	TGACACGTCAGTACTGTG	120	132	0	0	1	0
LARS	CAACACGTCAGTACTGGT	358	142	1	1	0	0
LARS	ACACACGTCAGTACTGCA	67	73	0	0	0	0
LARS	GTACACGTCAGTACTGAC	36	30	0	0	0	0
LARS	CAACACGTCAGTTGGTGT	221	303	0	0	0	0
LARS2	TGACACGTACACCAGTCA	683	821	1	0	0	0
LARS2	CAACACGTACACCAGTAC	72	51	0	1	0	0
LARS2	GTACACGTACACCAGTTG	560	248	0	0	0	0
LARS2	TGACACGTACACCACAGT	78	109	0	1	0	0
LARS2	GTACACGTACACCACACA	30	73	0	0	0	0
LAT	GTACCAGTTGACACACCA	936	1019	0	0	0	9
LAT	ACACCAGTTGACACACAC	92	62	0	0	0	0
LAT	CAACCAGTTGACACACTG	1277	1147	1	0	0	1
LAT	GTACCAGTTGACACTGGT	120	72	0	0	0	0
LAT	CAACCAGTTGACACTGCA	218	165	0	0	0	0
LAT2	CATGGTCAACCAACGTTG	1140	277	3	0	0	0
LAT2	GTTGGTCAACCAACCAGT	212	342	8	3	0	2
LAT2	CATGGTCAACCAACCACA	393	193	0	1	0	0
LAT2	TGTGGTCAACCAACCAAC	40	54	0	0	0	0
LAT2	ACTGGTCAACCAACCATG	47	49	0	0	0	0
LAT2	GTTGCAACGTCAGTTGAC	85	27	0	0	0	0
LBP	GTACCAGTTGACGTTGTG	368	619	1	1	1	0
LBP	ACTGCATGCAGTTGGTGT	550	459	3	0	1	0
LBP	TGACCAGTTGACGTACTG	1330	732	0	2	0	0
LBP	ACACCAGTTGACGTTGGT	298	331	1	0	0	2
LBP	TGACCAGTTGACGTTGCA	434	131	0	1	0	0
LBP	CAACCAGTTGACGTTGAC	36	130	0	0	0	0
LCAT	ACTGCACACAACTGGTCA	1592	1561	1	1	1	5
LCAT	CAACCACACAACTGTGTG	137	438	0	0	0	0
LCAT	GTACCACACATGGTGTGT	219	671	0	0	0	6
LCAT	CAACCACACATGGTGTCA	541	293	0	1	0	0
LCAT	TGACCACACATGGTGTAC	101	114	0	0	0	0
LCAT	ACACCACACATGGTGTTG	62	122	0	0	0	0
LCK	CACATGACGTGTTGCACA	20	26	0	0	0	0
LCK	TGCATGACGTGTTGCAAC	8	13	0	0	0	0
LCK	ACCATGACGTGTTGCATG	216	319	0	0	0	0
LCK	CACATGACGTGTTGACGT	767	286	0	0	0	0
LCK	ACCATGACGTGTTGACCA	17	22	0	0	0	0
LCMT1	GTACCACAACACCAACTG	350	350	1	1	0	1
LCMT1	TGACCACAACACCATGGT	859	656	0	0	0	0
LCMT1	GTACCACAACACCATGCA	179	166	0	0	0	0
LCMT1	ACACCACAACACCATGAC	18	1	0	0	0	0
LCMT1	CAACCACAACACCATGTG	85	95	0	0	0	0
LCMT2	GTACCAACACACCAACCA	182	187	0	0	0	7
LCMT2	ACACCAACACACCAACAC	29	89	0	0	0	0
LCMT2	CAACCAACACACCAACTG	525	977	16	3	0	0
LCMT2	GTACCAACACACCATGGT	816	968	2	1	0	1
LCMT2	CAACCAACACACCATGCA	227	276	0	0	0	0
LCMT2	GTTGCATGCACAACACGT	60	75	0	0	0	0
LCN2	TGACTGCACATGACCATG	102	143	0	0	1	0
LCN2	GTACTGCACATGACCAAC	83	70	0	0	0	0
LCN2	ACACTGCACATGACACGT	35	47	0	0	0	0
LCN2	TGACTGCACATGACACCA	443	391	0	1	0	0
LCN2	CAACTGCACATGACACAC	146	114	1	0	0	0
LCP2	GTACCAGTTGACTGACTG	1211	979	2	2	7	0
LCP2	CAACCAGTTGACTGACAC	127	91	0	0	1	1
LCP2	ACACCAGTTGACTGACGT	117	103	0	0	0	0
LCP2	TGACCAGTTGACTGACCA	363	712	0	0	0	0
LCP2	TGACCAGTTGACTGTGGT	162	321	0	0	0	1
LCT	GTACACCATGGTGTCACA	98	109	0	0	0	0
LCT	ACACACCATGGTGTCAAC	5	2	0	0	0	0
LCT	CAACACCATGGTGTCATG	121	175	0	0	0	0
LCT	GTACACCATGGTGTACGT	119	42	0	0	0	0
LCT	ACTGGTTGTGGTACGTCA	141	190	1	0	0	0
LCT	GTTGCATGCACATGGTAC	34	58	0	0	0	0
LDB1	TGTGGTTGTGCATGTGCA	508	466	1	0	1	0
LDB1	CAACGTTGACTGGTGTGT	1005	537	4	0	0	0
LDB1	ACACGTTGACTGGTGTCA	56	38	0	0	0	0
LDB1	GTACGTTGACTGGTGTAC	65	58	0	0	0	0
LDB1	TGACGTTGACTGGTGTTG	171	229	0	0	0	0
LDHA	CAACCAGTCATGGTGTTG	291	270	0	1	0	3
LDHA	GTACCAGTCATGGTCAGT	216	200	0	0	0	0
LDHA	CAACCAGTCATGGTCACA	223	336	1	0	0	0

LDHA	TGACCAGTCATGGTCAAC	16	6	0	0	0	0
LDHA	ACACCAGTCATGGTCATG	185	280	0	0	0	0
LDHA	CATGCACAACGTGTTGCA	156	159	1	0	0	0
LDHAL6A	ACACCAACACGTCACACA	49	67	0	0	0	0
LDHAL6A	GTACCAACACGTCACAAC	31	28	0	0	0	0
LDHAL6A	TGACCAACACGTCACATG	104	90	1	1	0	0
LDHAL6A	CATGCAGTACTGTGGTTG	537	294	0	0	0	0
LDHAL6A	GTTGCAGTACTGTGCAGT	561	643	1	1	0	0
LDHAL6B	ACACCAGTGTACTGTGCA	134	132	0	0	0	0
LDHAL6B	GTACCAGTGTACTGTGAC	29	14	0	0	0	0
LDHAL6B	TGACCAGTGTACTGTGTG	458	593	1	0	0	0
LDHAL6B	ACACCAGTGTTGGTGTGT	358	557	0	1	0	0
LDHAL6B	TGACCAGTGTTGGTGTCA	106	164	1	0	0	0
LDHAL6B	TGTGCAACGTCACAACGT	56	58	0	0	0	0
LDHB	TGACCAGTCAACCACACA	160	127	0	0	0	0
LDHB	CAACCAGTCAACCACAAC	15	26	0	0	0	0
LDHB	GTACCAGTCAACCACATG	255	392	1	0	0	1
LDHB	TGACCAGTCAACCAACGT	227	248	0	0	0	0
LDHB	GTACCAGTCAACCAACCA	132	179	0	0	0	1
LDHC	CAACCAGTCAACTGTGAC	79	79	0	0	0	0
LDHC	GTACCAGTCAACTGTGTG	144	202	71	0	0	0
LDHC	TGACCAGTCATGGTGTGT	1106	725	1	2	0	1
LDHC	GTACCAGTCATGGTGTCA	167	165	0	0	0	0
LDHC	ACACCAGTCATGGTGTAC	15	12	1	0	0	0
LDHC	GTTGCACATGGTACTGAC	32	5	0	0	0	0
LDLR	ACACACTGCAACACTGCA	217	244	1	0	1	1
LDLR	GTACACTGCAACACTGAC	26	19	0	0	0	0
LDLR	TGACACTGCAACACTGTG	71	134	0	0	0	0
LDLR	CAACACTGCAACTGGTGT	365	328	1	0	0	1
LDLR	GTTGCAGTGTTGGTTGTG	762	339	1	0	0	1
LDLR	ACTGCACATGGTGTGTTG	154	236	0	0	0	1
LEF1	GTACGTTGGTTGACGTGT	230	296	0	1	1	0
LEF1	CAACGTTGGTTGACCAGT	215	208	1	1	1	0
LEF1	CAACGTTGGTTGACGTCA	58	47	1	0	0	0
LEF1	TGACGTTGGTTGACGTAC	13	38	0	0	0	0
LEF1	ACACGTTGGTTGACGTTG	269	307	0	0	0	0
LEF1	CATGCAACCACAACGTGT	681	424	0	0	0	1
LEFTY1	TGACTGGTACGTGTACGT	403	376	0	1	0	0
LEFTY1	CAACTGGTTGTGCACAAC	140	37	0	0	0	0
LEFTY1	CATGCAGTTGACACGTAC	335	200	1	0	0	0
LEFTY1	CATGCAACTGTGACACTG	598	573	3	0	0	0
LEFTY1	GTTGCAACTGTGACTGGT	169	178	0	0	0	0
LEFTY1	CATGCAACTGTGACTGCA	87	137	0	0	0	0
LEFTY2	CAACTGGTACGTGTACTG	612	729	0	0	1	0
LEFTY2	GTACTGGTACGTGTACCA	92	91	0	0	0	0
LEFTY2	ACACTGGTACGTGTACAC	17	14	0	0	0	0
LEFTY2	ACACTGGTTGTGCACAGT	301	201	0	0	0	0
LEFTY2	TGACTGGTTGTGCACACA	349	149	0	0	0	2
LEFTY2	ACTGCAACTGTGACCAGT	69	320	0	0	0	0
LEP	GTACTGGTACCAGTACTG	566	312	0	0	1	15
LEP	GTACTGGTACCAGTTGCA	265	488	0	0	1	0
LEP	TGACTGGTACCAGTACCA	215	380	1	0	0	0
LEP	CAACTGGTACCAGTACAC	95	77	0	0	0	0
LEP	TGACTGGTACCAGTTGGT	372	361	0	0	0	0
LEP	ACTGCACACAACCAGTAC	244	251	21	1	0	0
LEPR	GTACTGGTTGACGTTGGT	445	565	1	0	0	0
LEPR	CAACTGGTTGACGTTGCA	43	57	0	0	0	0
LEPR	TGACTGGTTGACGTTGAC	38	43	0	1	0	0
LEPR	ACACTGGTTGACGTTGTG	17	40	0	0	0	0
LEPR	GTACTGGTTGACCAGTGT	237	444	0	0	0	1
LFNG	CATGGTGTCACACAACTG	940	786	6	0	0	352
LFNG	ACTGGTACTGGTTGCATG	90	90	0	0	0	0
LFNG	GTTGACCATGACGTGTGT	251	620	0	1	0	0
LFNG	CATGACCATGACGTGTCA	206	218	0	1	0	0
LFNG	TGTGACCATGACGTGTAC	114	103	0	0	0	0
LGALS1	CAACACTGTGTGGTTGGT	187	132	0	0	0	0
LGALS1	ACACACTGTGTGGTTGCA	168	492	0	0	0	0
LGALS1	GTACACTGTGTGGTTGAC	144	72	0	0	0	0
LGALS1	TGACACTGTGTGGTTGTG	507	463	0	1	0	20
LGALS1	ACTGCAGTTGACGTTGAC	173	46	0	1	0	0
LGMN	GTACCAGTACCAACTGCA	339	192	0	0	0	1
LGMN	ACACCAGTACCAACTGAC	38	56	0	0	0	0
LGMN	CAACCAGTACCAACTGTG	790	371	0	0	0	0
LGMN	ACTGCAGTCAACGTACCA	141	134	1	0	0	0
LGMN	GTTGCAGTCAACGTACAC	9	21	0	0	0	0

LHB	TGACTGTGTGTGGTTGAC	92	101	0	1	1	0
LHB	CAACTGTGACTGCAGTGT	1060	505	1	1	0	0
LHB	CAACTGTGTGTGGTTGCA	155	95	0	0	0	1
LHB	ACACTGTGTGTGGTTGTG	295	261	0	1	0	0
LHB	GTACTGTGTGTGCAGTGT	575	736	1	0	0	1
LHB	TGTGCAACGTCAACCATG	390	241	0	0	0	0
LHCGR	CAACGTCACATGACACGT	55	65	0	0	0	0
LHCGR	ACACGTCACATGACACCA	554	479	0	0	0	1
LHCGR	GTACGTCACATGACACAC	83	147	1	0	0	7
LHCGR	CATGCAGTACCATGACAC	62	88	0	0	0	0
LHCGR	CATGACCATGTGTGACCA	326	245	0	0	0	7
LHPP	CATGCAGTTGCAACCAGT	333	432	0	0	1	0
LHPP	CAACACCATGACGTGTTG	387	427	0	0	0	10
LHPP	GTACACCATGACGTCAGT	348	295	0	1	0	4
LHPP	CAACACCATGACGTCACA	52	66	0	0	0	0
LHPP	TGACACCATGACGTCAAC	10	44	0	0	0	0
LHX2	GTTGCATGGTTGGTGTTG	657	597	1	1	2	0
LHX2	GTACGTCAACTGCAACGT	137	249	0	0	0	9
LHX2	CAACGTCAACTGCAACCA	225	244	0	0	0	0
LHX2	TGACGTCAACTGCAACAC	52	29	0	0	0	0
LHX2	ACACGTCAACTGCAACTG	625	764	1	0	0	0
LHX2	ACACTGACTGCATGTGCA	196	154	1	0	0	0
LHX8	CAACGTACACCATGCATG	377	345	0	1	0	0
LHX8	GTACGTACACCATGACGT	147	112	1	0	0	0
LHX8	CAACGTACACCATGACCA	141	154	0	0	0	0
LHX8	TGACGTACACCATGACAC	6	17	0	0	0	0
LHX8	ACACGTACACCATGACTG	313	343	0	0	0	1
LIAS	CAACTGTGCAACACGTGT	136	127	0	0	0	0
LIAS	ACACTGTGCAACACGTCA	35	107	0	0	0	0
LIAS	GTACTGTGCAACACGTAC	35	19	0	0	0	0
LIAS	TGACTGTGCAACACGTTG	383	305	0	0	0	0
LIAS	GTTGGTTGTGGTGTACAC	22	7	0	0	0	0
LIF	GTACTGGTACTGTGACTG	1144	1024	0	0	1	5
LIF	TGACTGGTACTGTGCATG	250	419	31	0	0	0
LIF	ACACTGGTACTGTGACGT	14	56	0	0	0	0
LIF	TGACTGGTACTGTGACCA	521	320	2	1	0	0
LIF	CAACTGGTACTGTGACAC	748	133	0	0	0	0
LIF	TGTGCACAACCAACTGTG	272	70	0	0	0	0
LIFR	ACACTGGTTGCATGACGT	125	160	0	0	1	0
LIFR	ACACTGGTTGCATGCACA	11	37	0	0	0	0
LIFR	GTACTGGTTGCATGCAAC	63	122	0	0	0	0
LIFR	TGACTGGTTGCATGCATG	128	159	1	0	0	0
LIFR	TGACTGGTTGCATGACCA	102	104	0	1	0	0
LIG1	GTACACCACAACACACTG	470	337	3	316	1340	0
LIG1	CAACACCACAACACACAC	57	57	0	0	0	0
LIG1	TGACACCACAACACTGGT	177	196	0	0	0	0
LIG1	GTACACCACAACACTGCA	553	172	0	1	0	0
LIG1	ACACACCACAACACTGAC	71	80	1	0	0	0
LIG1	CATGCACACAACCAGTTG	834	788	1	0	0	43
LIG3	CAACACCACAACTGGTTG	3399	2588	16	3	14	10
LIG3	CAACACCACAACACTGTG	139	183	0	0	0	1
LIG3	TGACACCACAACTGGTGT	329	310	2	0	0	0
LIG3	GTACACCACAACTGGTCA	650	174	1	0	0	59
LIG3	ACACACCACAACTGGTAC	7	12	0	0	0	0
LIG4	TGACCATGCAACTGCAGT	333	382	1	0	0	0
LIG4	GTACCATGCAACTGCACA	141	154	0	2	0	0
LIG4	ACACCATGCAACTGCAAC	18	10	0	0	0	0
LIG4	CAACCATGCAACTGCATG	270	161	1	0	0	0
LIG4	GTACCATGCAACTGACGT	439	475	0	0	0	0
LILRB3	CAACTGCAACGTCAACTG	1130	845	1	0	2	1
LILRB3	ACACACTGACGTTGGTGT	294	618	0	0	0	170
LILRB3	TGACACTGACGTTGGTCA	112	168	0	0	0	0
LILRB3	CAACACTGACGTTGGTAC	99	64	0	0	0	0
LILRB3	GTACACTGACGTTGGTTG	593	732	1	0	0	0
LIMK1	TGCATGCATGGTGTCAAC	14	24	0	0	0	0
LIMK1	ACCATGCATGGTGTCATG	108	211	0	0	0	0
LIMK1	CATGGTACTGACGTGTTG	321	364	0	2	0	0
LIMK1	GTTGGTACTGACGTCAGT	262	199	1	1	0	0
LIMK1	CATGGTACTGACGTCACA	88	111	0	0	0	1
LIMK1	CATGCACATGGTGTCAGT	219	278	0	0	0	0
LIN7A	ACTGGTGTCAGTGTACCA	155	286	1	1	3	5
LIN7A	GTTGGTGTCAGTGTACAC	152	47	0	0	0	3
LIN7A	ACTGGTGTTGGTTGCAGT	211	28	0	0	0	0
LIN7A	TGTGGTGTTGGTTGCACA	65	75	1	0	0	0
LIN7A	CATGGTGTTGGTTGCAAC	35	54	0	0	0	0

LIPA	GTACCAGTACCAACACTG	1558	1866	1	0	1	0
LIPA	TGACCAGTACCAACACCA	606	304	2	0	0	9
LIPA	CAACCAGTACCAACACAC	22	20	0	0	0	0
LIPA	TGACCAGTACCAACTGGT	365	419	0	1	0	0
LIPA	CATGACGTACGTACACTG	158	165	0	0	0	0
LIPC	ACACACACGTACGTGTGT	49	20	0	0	0	0
LIPC	TGACACACGTACGTGTCA	252	187	0	0	0	1
LIPC	CAACACACGTACGTGTAC	50	136	0	0	0	0
LIPC	GTTGCACACAACCACAGT	32	67	0	0	0	0
LIPC	CATGCACACAACCACACA	284	500	0	0	0	3
LIPC	TGTGCACACAACCACAAC	16	23	0	0	0	0
LIPE	GTACACACACACTGGTTG	482	452	1	0	1	2
LIPE	CAACACACACACTGCATG	276	297	0	1	1	0
LIPE	TGACACACACACTGCAGT	544	633	1	1	0	0
LIPE	GTACACACACACTGCACA	60	124	0	0	0	0
LIPE	ACACACACACACTGCAAC	3	8	0	0	0	0
LIPE	TGTGCAACACGTGTGTCA	354	566	0	0	0	1
LIPF	TGACACACCACAGTCACA	141	76	1	0	0	0
LIPF	CAACACACCACAGTCAAC	8	4	0	0	0	0
LIPF	GTACACACCACAGTCATG	259	144	0	0	0	0
LIPF	TGACACACCACAGTACGT	423	394	0	0	0	0
LIPF	ACTGGTACTGCAACCATG	576	500	0	0	0	0
LIPF	ACTGCACACATGCACACA	85	199	0	0	0	0
LIPT1	CAACCACATGTGGTACTG	395	752	0	0	0	1
LIPT1	GTACCACATGTGGTTGGT	318	346	4	1	0	1
LIPT1	CAACCACATGTGGTTGCA	100	110	0	0	0	0
LIPT1	TGACCACATGTGGTTGAC	37	205	0	0	0	0
LIPT1	ACACCACATGTGGTTGTG	115	81	1	0	0	0
LIPT1	GTTGCAACACCATGCACA	71	76	0	0	0	0
LITAF	ACACGTCAACTGACGTCA	103	54	0	0	0	0
LITAF	GTACGTCAACTGACGTAC	22	32	0	0	0	0
LITAF	CATGCAGTCATGCAACGT	249	1105	3	3	0	0
LITAF	ACTGCAGTCATGCAACCA	125	90	0	1	0	0
LITAF	GTTGCAGTCATGCAACAC	1	0	0	0	0	0
LMAN1	CAACACTGTGTGCAGTGT	189	185	1	0	0	0
LMAN1	ACACACTGTGTGCAGTCA	191	410	1	0	0	0
LMAN1	GTACACTGTGTGCAGTAC	97	86	1	0	0	0
LMAN1	TGACACTGTGTGCAGTTG	146	292	0	0	0	1
LMAN1	ACACACTGTGTGCACAGT	37	33	0	0	0	0
LMAN1	CATGCACATGCAACCAAC	29	33	0	0	0	0
LMNA	TGACTGCATGCAACGTCA	173	210	0	0	0	0
LMNA	CAACTGCATGCAACGTAC	64	75	0	0	0	0
LMNA	GTACTGCATGCAACGTTG	348	520	0	1	0	1
LMNA	TGACTGCATGCAACCAGT	174	173	0	0	0	0
LMNA	TGTGCAGTGTTGTGACCA	104	139	0	1	0	0
LMNB1	GTACCAGTACCATGACAC	75	85	0	0	0	0
LMNB1	TGACCAGTACCATGACTG	663	503	7973	10	0	1
LMNB1	ACACCAGTACCATGTGGT	488	555	0	0	0	3
LMNB1	TGACCAGTACCATGTGCA	355	220	1	1	0	0
LMNB1	CAACCAGTACCATGTGAC	77	76	0	0	0	0
LMNB1	TGTGCAACTGTGACTGAC	14	25	0	0	0	0
LMNB2	ACACTGACTGACACTGTG	377	509	0	1	1	0
LMNB2	CAACTGACTGACACTGCA	586	189	0	2	0	0
LMNB2	TGACTGACTGACACTGAC	34	63	0	0	0	0
LMNB2	GTACTGACTGACTGGTGT	430	653	20	0	0	0
LMNB2	CAACTGACTGACTGGTCA	839	864	0	0	0	0
LMNB2	ACTGCAACTGTGACTGTG	649	635	1	1	0	0
LMO1	ACACGTACCAGTGTGTTG	452	929	1	0	0	0
LMO1	CAACGTACCAGTGTCAGT	254	355	0	0	0	1
LMO1	ACACGTACCAGTGTCACA	117	117	0	0	0	0
LMO1	GTACGTACCAGTGTCAAC	30	16	0	0	0	0
LMO1	TGACACTGGTTGACGTCA	157	245	0	0	0	0
LMO1	GTTGCACAGTTGACCACA	90	66	0	0	0	0
LMO2	TGACGTACACGTTGCAAC	7	6	0	0	0	0
LMO2	ACACGTACACGTTGCATG	76	89	0	0	0	2
LMO2	CAACGTACACGTTGACGT	169	205	0	0	0	1
LMO2	ACACGTACACGTTGACCA	226	113	0	35	0	0
LMO2	GTACGTACACGTTGACAC	43	34	0	0	0	0
LMO2	GTTGCACAACACGTTGAC	81	22	0	0	0	0
LMX1B	TGACGTACACCATGTGTG	233	535	1	0	0	0
LMX1B	TGACGTACACACGTGTGT	891	446	115	1	0	0
LMX1B	GTACGTACACACGTGTCA	186	390	0	0	0	0
LMX1B	ACACGTACACACGTGTAC	108	15	0	0	0	0
LMX1B	TGACTGACTGCATGTGTG	506	971	2	0	0	0
LMX1B	ACTGCACAGTTGACCAAC	13	25	0	0	0	0

LNPEP	CAACCAGTTGTGGTTGTG	396	321	0	0	0	0
LNPEP	TGACCAGTTGTGCAGTGT	85	219	2	0	0	0
LNPEP	CAACTGTGGTCATGTGCA	145	229	0	0	0	0
LNPEP	TGACTGTGGTCATGTGAC	165	106	0	0	0	0
LNPEP	ACACTGTGGTCATGTGTG	430	645	2	1	0	7
LOC283398	ACACACCACATGGTGTAC	30	18	0	0	0	0
LOC283398	CAACACCACATGGTGTTG	136	172	0	1	0	0
LOC283398	GTACACCACATGGTCAGT	109	111	1	0	0	0
LOC283398	ACTGACACGTTGGTGTTG	174	247	0	0	0	1
LOC283398	CATGACACGTTGGTCAGT	150	195	0	1	0	0
LOC283412	GTACTGACTGTGTGGTAC	8	42	0	0	0	0
LOC283412	TGTGACACGTACACACGT	90	101	0	0	0	0
LOC283412	GTTGACACGTACACACCA	97	63	0	0	0	0
LOC283412	ACTGACACGTACACACAC	50	62	0	0	0	0
LOC283412	CATGACACGTACACACTG	187	242	1	0	0	0
LOC284064	ACTGGTTGACACGTTGTG	429	473	0	20	1	0
LOC284064	TGACTGACTGTGTGGTTG	675	504	1	0	0	1
LOC284064	ACACTGACTGTGTGCAGT	312	321	0	0	0	0
LOC284064	TGACTGACTGTGTGCACA	226	223	1	0	0	0
LOC284064	TGTGGTTGACACGTTGAC	18	18	0	0	0	0
LOC284064	GTTGCATGGTGTGTCAAC	105	19	0	0	0	0
LOC284288	ACTGGTTGACACCAGTTG	504	480	2	0	1	1
LOC284288	CATGGTTGACACCAGTCA	804	342	1	1	0	0
LOC284288	TGTGGTTGACACCAGTAC	36	39	0	0	0	0
LOC284288	CATGGTTGACACCACAGT	57	112	0	1	0	0
LOC284288	ACTGACCATGGTCATGCA	156	200	0	0	0	1
LOC285053	ACTGGTGTTGACTGCAAC	28	30	0	0	0	0
LOC285053	CATGGTGTTGACTGCATG	732	650	0	0	0	0
LOC285053	GTTGGTGTTGACTGACGT	143	178	0	0	0	0
LOC285053	CATGGTGTTGACTGACCA	229	124	0	0	0	0
LOC285053	GTTGGTTGACACGTTGGT	333	399	0	0	0	0
LOC347292	ACTGACACGTACTGCACA	26	262	0	0	1	0
LOC347292	CATGGTGTTGTGGTGTAC	57	314	0	0	0	0
LOC347292	GTTGGTGTTGTGGTGTTG	174	140	0	0	0	0
LOC347292	CATGCACAGTGTACCAGT	287	329	0	0	0	0
LOC347292	GTTGACACGTACTGCAAC	9	3	0	0	0	0
LOC388720	ACTGGTTGTGCATGCATG	949	656	2	0	1	2
LOC388720	TGACGTGTCAACTGACCA	178	100	0	0	0	1
LOC388720	GTACGTGTCAACTGACTG	136	27	0	0	0	0
LOC388720	CATGGTTGTGCATGACGT	134	95	0	0	0	0
LOC388720	ACTGGTTGTGCATGACCA	241	391	0	0	0	6
LOC390243	ACTGCAGTGTCACACATG	115	115	0	2	0	0
LOC390243	CATGCAGTGTCACAACGT	151	185	0	0	0	0
LOC390243	ACTGCAGTGTCACAACCA	121	162	0	0	0	0
LOC390243	CATGACACCATGACGTTG	221	173	0	0	0	0
LOC390243	GTTGACACCATGACCAGT	206	206	1	0	0	0
LOC390876	ACTGACACGTTGCATGGT	300	287	1	1	0	0
LOC390876	TGTGACACGTTGCATGCA	394	247	0	0	0	1
LOC390876	CATGACACGTTGCATGAC	88	37	0	0	0	0
LOC390876	GTTGACACGTTGCATGTG	540	426	0	1	0	1
LOC390876	ACTGACACGTTGACGTGT	177	482	1	0	0	0
LOC391656	ACACTGTGGTTGACTGTG	48	374	0	0	0	0
LOC391656	GTACTGTGGTTGTGGTGT	837	1705	2	151	0	21
LOC391656	CATGCAGTCACAACGTTG	929	976	1	0	0	0
LOC391656	GTTGCAGTCACAACCAGT	75	72	0	0	0	0
LOC391656	GTTGACACGTACACGTAC	7	7	0	0	0	0
LOC400652	CATGACCATGGTCACATG	38	40	0	0	0	0
LOC400652	GTTGACCATGGTCAACGT	2	20	0	0	0	0
LOC400652	CATGACCATGGTCAACCA	504	296	1	0	0	0
LOC400652	TGTGACCATGGTCAACAC	86	82	0	1	0	0
LOC400652	ACTGACCATGGTCAACTG	205	234	0	0	0	0
LOC402057	GTACTGTGGTTGTGCAAC	8	7	0	0	0	0
LOC402057	TGACTGTGGTTGTGCATG	15	8	0	0	0	0
LOC402057	GTTGCAGTGTACGTCACA	43	30	0	6	0	0
LOC402057	ACTGCAGTGTACGTCAAC	37	26	0	0	0	0
LOC402057	CATGCAGTGTACGTCATG	56	57	0	0	0	0
LOC439992	TGTGCAGTCAACCAACAC	10	5	0	0	0	0
LOC439992	ACTGCAGTCAACCAACTG	531	356	0	1	0	0
LOC439992	CATGCAGTCAACCATGGT	1285	1679	4005	3	0	2
LOC439992	ACTGCAGTCAACCATGCA	325	269	0	1	0	0
LOC439992	CATGACCAACTGACACAC	50	40	0	0	0	0
LOC441996	TGTGACCATGGTACGTTG	299	367	0	1	1	3
LOC441996	TGTGACCATGGTCATGTG	130	162	0	0	0	0
LOC441996	CATGACCATGGTACGTGT	161	267	0	0	0	0
LOC441996	ACTGACCATGGTACGTCA	183	195	0	0	0	0

LOC441996	GTTGACCATGGTACGTAC	16	12	0	0	0	0
LOC643224	ACTGACCAACTGACGTTG	588	683	0	1	0	0
LOC643224	CATGACCAACTGACCAGT	473	382	1	0	0	0
LOC643224	ACTGACCAACTGACCACA	208	162	0	0	0	0
LOC643224	GTTGACCAACTGACCAAC	94	96	1	0	0	1
LOC643224	TGTGACCAACTGACCATG	329	144	0	0	0	0
LOC654264	CATGCAGTGTCATGTGCA	227	242	1	0	1	0
LOC654264	CATGGTGTACTGTGACAC	61	216	1	0	0	0
LOC654264	GTTGGTGTACTGTGACTG	449	550	0	0	0	1
LOC654264	CATGACCATGGTACCAAC	31	79	0	0	0	0
LOC654264	GTTGACCATGGTACCATG	803	649	1	2	0	2
LOC728919	CATGACACGTTGGTTGTG	612	993	12	6056	4	38
LOC728919	TGTGACACGTTGGTTGGT	51	57	0	0	0	0
LOC728919	GTTGACACGTTGGTTGCA	31	64	0	0	0	0
LOC728919	ACTGACACGTTGGTTGAC	46	45	0	0	0	0
LOC728919	TGTGACACGTTGCAGTGT	895	865	0	1	0	0
LPA	TGACACACACACCAGTAC	172	134	1	0	1	0
LPA	ACACACACACACCAGTTG	1258	1160	0	0	1	0
LPA	ACACACACACACCACACA	148	194	0	0	1	0
LPA	CAACACACACACCACAGT	285	328	1	1	0	195
LPA	GTACACACACACCACAAC	11	5	0	0	0	0
LPHN1	TGACGTCACATGCATGGT	1	2	0	0	0	0
LPHN1	GTACGTCACATGCATGCA	200	225	0	0	0	0
LPHN1	ACACGTCACATGCATGAC	126	90	0	0	0	0
LPHN1	CAACGTCACATGCATGTG	411	743	2	0	0	0
LPHN1	TGTGACGTTGCATGCAGT	44	125	0	0	0	0
LPHN2	TGACGTCACATGACACTG	233	263	0	0	0	0
LPHN2	ACACGTCACATGACTGGT	330	275	0	0	0	0
LPHN2	TGACGTCACATGACTGCA	44	64	0	0	0	7
LPHN2	CAACGTCACATGACTGAC	49	589	0	0	0	0
LPHN2	GTACGTCACATGACTGTG	188	251	62	0	0	0
LPHN3	ACTGCAGTCATGCACATG	192	295	0	0	2	13
LPHN3	TGACGTCACATGACGTGT	249	318	2	0	0	6
LPHN3	GTACGTCACATGACGTCA	127	163	0	0	0	2
LPHN3	ACACGTCACATGACGTAC	106	108	0	0	0	2
LPHN3	TGTGGTACTGCAACTGCA	63	64	0	0	0	0
LPIN1	TGTGGTGTCAGTTGACCA	270	352	0	0	0	0
LPIN1	TGTGGTACACTGCAACCA	151	55	0	0	0	0
LPIN1	CATGGTACACTGCAACAC	25	52	0	0	0	0
LPIN1	GTTGGTACACTGCAACTG	779	174	1	0	0	0
LPIN1	TGTGGTACACTGCATGGT	685	568	3	0	0	1
LPIN1	TGTGCAACACACGTTGCA	1	16	0	0	0	0
LPIN2	GTTGGTTGGTTGACCATG	210	254	0	0	0	1
LPIN2	TGTGGTTGGTTGACACGT	232	277	9	0	0	0
LPIN2	GTTGGTTGGTTGACACCA	111	129	0	0	0	0
LPIN2	ACTGGTTGGTTGACACAC	167	215	0	0	0	0
LPIN2	CATGGTTGGTTGACACTG	190	247	0	0	0	0
LPIN2	CATGCAACACACGTTGAC	39	46	0	0	0	0
LPIN3	TGTGGTTGGTACTGACTG	459	712	8	3551	4746	4
LPIN3	ACTGGTTGGTACTGACCA	511	290	200	150	201	401
LPIN3	ACTGGTTGGTACTGCATG	109	302	0	0	3	0
LPIN3	CATGGTTGGTACTGACGT	491	1090	1	0	1	0
LPIN3	GTTGGTTGGTACTGACAC	43	35	0	0	0	0
LPL	CAACACACACACACTGAC	15	38	0	0	0	0
LPL	GTACACACACACACTGTG	568	554	0	0	0	0
LPL	ACACACACACACTGGTGT	688	736	0	0	0	0
LPL	TGACACACACACTGGTCA	75	65	0	0	0	0
LPL	CAACACACACACTGGTAC	66	219	0	0	0	0
LPO	TGACACGTACTGCACAAC	43	21	0	0	0	0
LPO	ACACACGTACTGCACATG	233	529	0	0	0	0
LPO	CAACACGTACTGCAACGT	325	517	2	0	0	0
LPO	ACACACGTACTGCAACCA	596	670	0	1	0	0
LPO	GTACACGTACTGCAACAC	42	32	0	0	0	0
LRDD	ACTGGTACTGCACAGTTG	558	673	0	1	0	0
LRDD	CATGGTACTGCACACAGT	385	253	1	0	0	0
LRDD	ACTGGTACTGCACACACA	29	115	0	1	0	0
LRDD	ACTGGTTGCATGCAGTAC	40	72	0	0	0	0
LRDD	CATGGTTGCATGCAGTTG	1267	1196	2	2	0	44
LRIG1	ACACTGTGGTGTCATGAC	92	25	0	0	0	0
LRIG1	CAACTGTGGTGTCATGTG	67	70	1	0	0	0
LRIG1	TGACTGTGGTGTACGTGT	179	249	0	0	0	0
LRIG1	TGTGCAGTCATGGTGTAC	167	288	0	1	0	2
LRIG1	ACTGCAGTCATGGTGTTG	826	944	2	0	0	0
LRMP	CACATGTGTGCAGTCAGT	76	95	0	0	0	0
LRMP	ACCATGTGTGCAGTCACA	26	31	0	0	0	0

LRMP	GTCATGTGTGCAGTCAAC	59	60	0	0	0	0
LRMP	TGCATGTGTGCAGTCATG	85	38	1	0	0	0
LRMP	ACCATGTGTGCAGTACGT	421	243	1	0	0	0
LRP1	TGACACTGGTGTACCATG	182	269	0	1	0	0
LRP1	ACACACTGGTGTACACGT	101	150	0	0	0	0
LRP1	TGACACTGGTGTACACCA	33	30	0	0	0	0
LRP1	CAACACTGGTGTACACAC	72	66	0	0	0	0
LRP1	GTACACTGGTGTACACTG	100	102	0	1	0	0
LRP2	CAACACTGGTGTACTGTG	383	439	52	0	2	0
LRP2	TGACACTGGTGTACTGGT	324	389	1	0	1	0
LRP2	GTACACTGGTGTACTGCA	33	235	0	0	0	0
LRP2	ACACACTGGTGTACTGAC	25	24	0	0	0	0
LRP2	TGACACTGGTGTTGGTGT	541	997	1	0	0	0
LRP5	GTACCACAGTCAACACGT	97	55	0	0	0	0
LRP5	CAACCACAGTCAACACCA	95	126	0	0	0	0
LRP5	TGACCACAGTCAACACAC	21	16	0	0	0	0
LRP5	ACACCACAGTCAACACTG	260	208	0	0	0	0
LRP5	CAACCACAGTCAACTGGT	218	332	0	0	0	0
LRP5	CATGCACAGTTGACCATG	98	119	0	0	0	0
LRP6	ACACCACAGTCAACTGCA	279	436	0	0	0	1
LRP6	GTACCACAGTCAACTGAC	67	111	0	0	0	0
LRP6	TGACCACAGTCAACTGTG	215	213	1	0	0	0
LRP6	CAACCACAGTCATGGTGT	61	82	0	0	0	0
LRP6	ACACCACAGTCATGGTCA	353	297	1	0	0	0
LRP8	TGACACTGGTTGTGACGT	338	325	1	0	0	0
LRP8	GTACACTGGTTGTGACCA	384	602	0	0	0	0
LRP8	ACACACTGGTTGTGACAC	77	117	0	0	0	0
LRP8	ACTGCAGTCAACCACAAC	66	54	1	0	0	2
LRP8	CATGCAGTCAACCACATG	173	193	0	0	0	0
LRPAP1	GTACACTGGTGTTGCAGT	60	82	0	0	1	0
LRPAP1	GTACACTGGTGTTGGTCA	179	357	0	0	0	0
LRPAP1	ACACACTGGTGTTGGTAC	177	49	0	0	0	0
LRPAP1	CAACACTGGTGTTGGTTG	412	386	0	1	0	0
LRPAP1	CAACACTGGTGTTGCACA	116	81	0	0	0	0
LRPAP1	GTTGCACAGTTGACACGT	59	46	0	0	0	0
LRPPRC	TGACCATGACGTTGGTGT	544	894	0	2	1	0
LRPPRC	CAACCATGACGTACTGTG	144	182	0	0	0	0
LRPPRC	GTACCATGACGTTGGTCA	150	148	0	0	0	4
LRPPRC	ACACCATGACGTTGGTAC	13	14	0	0	0	0
LRPPRC	CAACCATGACGTTGGTTG	605	615	3	0	0	0
LRPPRC	CATGCATGCAGTACTGAC	193	198	0	0	0	2
LRRC15	CATGGTCATGGTTGTGGT	302	736	2	0	1	0
LRRC15	GTTGGTCATGGTTGACGT	74	118	0	0	0	0
LRRC15	CATGGTCATGGTTGACCA	302	219	0	0	0	0
LRRC15	TGTGGTCATGGTTGACAC	28	17	0	0	0	0
LRRC15	ACTGGTCATGGTTGACTG	271	445	0	0	0	1
LRRC41	GTTGGTTGGTTGACGTAC	30	48	0	0	0	0
LRRC41	TGTGGTTGGTTGACGTTG	279	310	0	0	0	0
LRRC41	ACTGGTTGGTTGACCAGT	227	246	0	0	0	0
LRRC41	TGTGGTTGGTTGACCACA	6	25	0	0	0	0
LRRC41	CATGGTTGGTTGACCAAC	25	14	0	0	0	0
LRRK1	GTACGTGTCAACGTACAC	0	1	0	0	0	0
LRRK1	TGACGTGTCAACGTACTG	200	229	1	1	0	0
LRRK1	ACACGTGTCAACGTTGGT	312	344	0	1	0	0
LRRK1	TGACGTGTCAACGTTGCA	80	66	0	0	0	0
LRRK1	CAACGTGTCAACGTTGAC	1	3	0	0	0	7
LRRK2	CAACGTTGACCACAGTGT	2110	2407	124	1	1	0
LRRK2	ACACGTTGACCAGTTGCA	93	90	0	0	0	3
LRRK2	GTACGTTGACCAGTTGAC	25	26	0	0	0	0
LRRK2	TGACGTTGACCAGTTGTG	325	701	3	216	0	2
LRRK2	ACACGTTGACCACAGTCA	223	206	1	0	0	2
LRRTM3	ACTGGTCAACACGTTGGT	154	243	0	0	0	0
LRRTM3	TGTGGTCAACACGTTGCA	70	81	0	0	0	0
LRRTM3	CATGGTCAACACGTTGAC	24	14	0	0	0	0
LRRTM3	GTTGGTCAACACGTTGTG	115	765	0	1	0	0
LRRTM3	ACTGGTCAACACCAGTGT	382	453	0	0	0	0
LRWD1	CATGGTTGGTACACTGTG	501	244	3	1	1	0
LRWD1	TGTGGTTGGTACTGGTGT	229	303	0	0	0	0
LRWD1	GTTGGTTGGTACTGGTCA	130	150	0	0	0	1
LRWD1	ACTGGTTGGTACTGGTAC	74	198	0	0	0	0
LRWD1	CATGGTTGGTACTGGTTG	362	306	0	0	0	0
LRWD1	ACTGCAACACTGTGGTCA	239	315	2	0	0	23
LSM8	TGACTGTGGTTGACCACA	27	14	0	0	0	0
LSM8	CAACTGTGGTTGACCAAC	36	33	0	0	0	0
LSM8	GTACTGTGGTTGACCATG	72	176	0	0	0	0

LSM8	TGACTGTGGTTGACACGT	83	105	0	0	0	0
LSM8	GTACTGTGGTTGACACCA	155	243	1	0	0	0
LSS	CATGCAGTTGGTCAACCA	516	436	0	0	1	0
LSS	CAACACGTCACATGCAGT	153	219	0	0	0	0
LSS	ACACACGTCACATGCACA	164	200	0	1	0	0
LSS	GTACACGTCACATGCAAC	37	37	0	0	0	0
LSS	TGTGACGTCACAACTGTG	118	110	0	0	0	1
LTA	CAACTGGTCATGACGTTG	331	496	0	0	0	0
LTA	GTACTGGTCATGACCAGT	88	72	0	0	0	0
LTA	CAACTGGTCATGACCACA	83	72	0	0	0	0
LTA	TGACTGGTCATGACCAAC	42	97	0	0	0	0
LTA	ACACTGGTCATGACCATG	131	169	0	0	0	0
LTA	GTTGCACAACACCAGTAC	35	42	0	0	0	0
LTA4H	ACACACACGTTGGTTGGT	238	153	1	0	0	0
LTA4H	TGACACACGTTGGTTGCA	30	43	0	0	0	1
LTA4H	CAACACACGTTGGTTGAC	29	73	0	0	0	0
LTA4H	GTACACACGTTGGTTGTG	427	744	1	1	0	0
LTA4H	ACACACACGTTGCAGTGT	275	357	3	0	0	0
LTA4H	CATGCACAGTTGACACCA	429	453	2	1	0	3
LTB	CATGCAGTTGACGTTGTG	450	462	1	1	1	0
LTB	TGACTGCAGTACGTGTAC	72	63	0	0	0	0
LTB	ACACTGCAGTACGTGTTG	356	698	1	22	0	0
LTB	CAACTGCAGTACGTCAGT	232	230	0	0	0	0
LTB	TGTGCACAGTTGACACAC	79	57	0	0	0	0
LTB	ACTGCACAGTTGACACTG	286	728	1	0	0	0
LTB4R	CAACGTACGTGTTGCAGT	355	164	1	0	0	0
LTB4R	ACACGTACGTGTTGCACA	201	111	1	0	0	1
LTB4R	GTACGTACGTGTTGCAAC	28	11	0	0	0	0
LTB4R	TGACGTACGTGTTGCATG	43	18	0	0	0	0
LTB4R	ACACGTACGTGTTGACGT	143	255	0	0	0	0
LTB4R	CATGCATGCACAGTCAGT	426	213	0	0	0	0
LTB4R2	ACACGTCACATGTGGTGT	610	387	1	0	0	2
LTB4R2	TGACGTCACATGTGGTCA	156	194	0	0	0	0
LTB4R2	CATGCAGTACACGTCAAC	38	66	0	0	0	0
LTB4R2	GTTGCAGTACACGTCATG	437	242	0	2	0	0
LTB4R2	ACTGACACACCAACTGCA	82	78	0	0	0	0
LTBP1	CAACTGACTGCAACCACA	245	263	1	0	1	0
LTBP1	TGACACTGGTGTTGTGCA	121	454	0	1	0	0
LTBP1	CAACACTGGTGTTGTGAC	27	23	0	0	0	0
LTBP1	GTACACTGGTGTTGTGTG	403	311	0	2	0	0
LTBP1	CAACACTGGTCAGTGTGT	314	657	0	0	0	0
LTBP4	CAACACTGCAGTACACTG	435	582	5	2	0	2
LTBP4	GTACACTGCAGTACTGGT	251	287	0	0	0	0
LTBP4	CAACACTGCAGTACTGCA	234	221	0	0	0	0
LTBP4	TGACACTGCAGTACTGAC	56	28	0	0	0	0
LTBP4	TGTGCAGTTGCATGCAAC	17	60	0	0	0	1
LTBR	GTACTGGTACCACATGTG	580	521	0	1	0	0
LTBR	ACACTGGTACCAACGTGT	847	956	2	0	0	0
LTBR	TGACTGGTACCAACGTCA	446	448	0	0	0	0
LTBR	CAACTGGTACCAACGTAC	74	216	0	0	0	0
LTBR	GTACTGGTACCAACGTTG	159	492	1	0	0	0
LTBR	CATGCACAGTTGACTGGT	352	314	0	0	0	1
LTC4S	GTTGCAGTTGGTCAACGT	541	595	1	0	1	3
LTC4S	CAACACGTCAACACCACA	134	114	1	0	0	0
LTC4S	TGACACGTCAACACCAAC	63	73	0	0	0	0
LTC4S	CATGCAGTTGGTCACATG	144	89	0	0	0	1
LTC4S	CATGCAACACACCAACCA	367	327	0	0	0	0
LTC4S	TGTGCAACACACCAACAC	29	67	3	0	0	1
LTF	ACACACCAGTCAACACAC	30	57	0	0	0	0
LTF	CAACACCAGTCAACACTG	632	756	0	0	0	0
LTF	GTACACCAGTCAACTGGT	1139	395	1	1	0	4
LTF	CAACACCAGTCAACTGCA	371	166	1	0	0	0
LTF	TGACACCAGTCAACTGAC	46	18	0	0	0	0
Luc	GTTGTGACTGCACACATG	310	267	42	3	3	1
Luc	GTTGTGACTGCAGTGTTG	1439	419	1	0	1	0
Luc	TGTGTGACTGGTTGCATG	1131	613	0	0	0	1
Luc	ACTGTGACTGGTTGACGT	295	333	0	0	0	0
Luc	TGTGTGACTGGTTGACCA	318	267	1	1	0	1
Luc	TGTGTGACTGGTTGTGGT	337	598	0	0	0	0
Luc	GTTGTGACTGGTTGTGCA	105	195	0	0	0	0
Luc	ACTGTGACTGGTTGTGAC	17	24	0	1	0	0
Luc	TGTGTGACTGCAGTGTCA	207	195	0	0	0	0
Luc	CATGTGACTGCAGTGTAC	110	73	0	0	0	2
Luc	ACTGTGACTGCAGTCAAC	33	29	0	0	0	43
Luc	CATGTGACTGCAGTCATG	574	805	0	1	0	0

Luc	GTTGTGACTGCAGTACGT	1061	1352	1	0	0	1
Luc	ACTGTGACTGCAGTACTG	204	394	0	0	0	0
Luc	CATGTGACTGCAGTTGGT	183	182	0	0	0	0
Luc	ACTGTGACTGCAGTTGCA	94	64	0	0	0	0
Luc	CATGTGACTGCACAGTGT	300	211	0	0	0	0
Luc	ACTGTGACTGCACAGTCA	101	108	0	0	0	1
Luc	GTTGTGACTGCACAGTAC	31	145	0	0	0	0
Luc	TGTGTGACTGCACACACA	105	186	0	0	0	0
Luc	CATGTGACTGCACACAAC	19	33	0	0	0	0
LUM	ACTGGTACACTGGTTGTG	206	146	0	2	0	1
LUM	GTTGGTACACTGCAGTGT	311	426	1	0	0	1
LUM	CATGGTACACTGCAGTCA	464	379	0	0	0	0
LUM	TGTGGTACACTGCAGTAC	75	65	0	0	0	0
LUM	ACTGGTACACTGCAGTTG	184	142	0	0	0	0
LXN	TGACTGTGGTGTACCAAC	17	14	1	0	0	0
LXN	ACACTGTGGTGTACCATG	26	61	0	0	0	0
LXN	CAACTGTGGTGTACACGT	90	116	0	0	0	0
LXN	ACACTGTGGTGTACACCA	233	173	0	0	0	0
LXN	GTACTGTGGTGTACACAC	40	34	0	0	0	0
LXN	TGTGCAACACGTGTACAC	33	48	0	0	0	0
LY6E	GTTGGTACCACACATGTG	185	196	0	0	0	0
LY6E	ACTGGTACCACAACGTGT	547	394	4	56	0	0
LY6E	TGTGGTACCACAACGTCA	260	183	1	16	0	0
LY6E	CATGGTACCACAACGTAC	26	33	0	0	0	2
LY6E	GTTGGTACCACAACGTTG	98	120	0	0	0	0
LY6E	ACTGCACAGTTGACTGCA	151	198	0	0	0	0
LY75	TGACACTGTGACTGCAGT	410	196	0	0	0	0
LY75	GTACACTGTGACTGCACA	46	99	0	0	0	0
LY75	ACACACTGTGACTGCAAC	79	103	0	1	0	0
LY75	CAACACTGTGACTGCATG	212	175	0	0	0	0
LY75	GTACACTGTGACTGACGT	160	188	0	1	0	1
LY75	GTTGCACAGTTGACTGAC	34	50	0	0	0	0
LY86	ACTGGTCATGACGTACTG	255	220	1	1	0	0
LY86	CATGGTCATGACGTTGGT	291	162	1	0	0	0
LY86	ACTGGTCATGACGTTGCA	90	211	1	1	0	0
LY86	GTTGGTCATGACGTTGAC	37	55	0	0	0	0
LY86	TGTGGTCATGACGTTGTG	174	658	0	1	0	0
LY9	GTTGGTACCATGTGACTG	418	511	0	1	0	1
LY9	GTTGGTTGCATGCACAGT	969	755	1	0	0	0
LY9	CATGGTTGCATGCACACA	171	182	5	1	0	2
LY9	TGTGGTTGCATGCACAAC	60	17	0	0	0	0
LY9	CATGGTTGTGGTGTCACA	115	297	0	0	0	0
LY96	TGACACTGACACACACCA	594	755	1	2	0	0
LY96	CAACACTGACACACACAC	44	30	0	0	0	0
LY96	GTACACTGACACACACTG	702	1288	1	0	0	19
LY96	TGACACTGACACACTGGT	57	100	0	1	0	0
LY96	GTACACTGACACACTGCA	587	405	1	0	0	0
LYCAT	ACACCAACGTACGTACCA	552	493	1	269	183	1
LYCAT	CAACCAACGTACGTACGT	547	466	0	0	1	0
LYCAT	ACACCAACGTACGTCATG	241	267	0	1	0	0
LYCAT	TGTGACGTCATGTGACCA	163	124	0	0	0	0
LYCAT	CATGACGTCATGTGACAC	31	42	0	0	0	0
LYN	TGTGGTTGACACACGTGT	68	108	0	0	0	0
LYN	GTTGGTTGACACACGTCA	137	132	1	0	0	0
LYN	ACTGGTTGACACACGTAC	82	58	0	0	0	0
LYN	CATGGTTGACACACGTTG	399	432	1	1	0	0
LYN	CATGGTTGACTGTGTGAC	74	53	0	0	0	0
LYN	TGTGCACAGTTGACTGTG	262	141	0	0	0	0
MAD1L1	ACACGTGTGTTGGTTGGT	303	130	2	0	0	0
MAD1L1	TGACGTGTGTTGGTTGCA	89	142	0	0	0	0
MAD1L1	CAACGTGTGTTGGTTGAC	46	62	0	0	0	0
MAD1L1	GTACGTGTGTTGGTTGTG	91	79	0	0	0	0
MAD1L1	ACACGTGTGTTGCAGTGT	508	555	0	0	0	0
MAD2L2	TGACGTGTGTTGCAGTCA	228	454	0	2	0	0
MAD2L2	CAACGTGTGTTGCAGTAC	77	87	0	0	0	0
MAD2L2	GTACGTGTGTTGCAGTTG	870	1023	0	2	0	0
MAD2L2	TGACGTGTGTTGCACAGT	116	92	0	0	0	0
MAD2L2	GTACGTGTGTTGCACACA	90	108	0	0	0	0
MAD2L2	CATGCACAACCATGGTGT	884	981	2	0	0	0
MADCAM1	CAACTGGTGTTGTGGTGT	259	258	0	0	0	0
MADCAM1	ACACTGGTGTTGTGGTCA	9	17	0	0	0	0
MADCAM1	GTACTGGTGTTGTGGTAC	34	302	0	0	0	0
MADCAM1	TGACTGGTGTTGTGGTTG	832	769	4	1	0	1
MADCAM1	CATGACACGTGTCAGTGT	254	389	0	2	0	0
MADD	ACACCAACTGCAGTACCA	253	273	1	0	1	1

MADD	CAACCAACTGCAGTACGT	142	200	1	0	0	0
MADD	GTACCAACTGCAGTACAC	184	125	0	0	0	0
MADD	TGACCAACTGCAGTACTG	155	253	0	0	0	1
MADD	ACACCAACTGCAGTTGGT	54	40	0	0	0	0
MADD	TGTGCAACCAGTCATGCA	483	827	1	0	0	0
MAF	ACCATGCACACATGGTGT	1660	1233	0	1	0	16
MAF	TGCATGCACACATGGTCA	217	232	0	0	0	176
MAF	CACATGCACACATGGTAC	113	138	0	0	0	0
MAF	CATGGTACTGCAACCACA	26	43	0	0	0	0
MAF	GTTGCAGTCAGTGTGTTG	271	531	0	0	0	0
MAFA	CAACGTACCATGCACAAC	44	54	0	0	0	0
MAFA	GTACGTACCATGCACATG	350	350	2	0	0	0
MAFA	TGACGTACCATGCAACGT	126	87	0	0	0	0
MAFA	TGTGGTGTGTTGACGTCA	77	112	0	0	0	0
MAFA	CATGGTGTGTTGACGTAC	48	42	0	0	0	0
MAFB	CAACGTACACACCATGGT	194	169	0	0	0	0
MAFB	ACACGTACACACCATGCA	33	48	0	0	0	0
MAFB	GTACGTACACACCATGAC	37	73	0	0	0	0
MAFB	TGACGTACACACCATGTG	195	306	1	1	0	0
MAFB	ACACTGTGACACACACCA	668	552	0	1	0	0
MAFB	TGTGCATGCAGTTGGTCA	205	318	1	1	0	1
MAFF	CAACGTACCATGACTGTG	316	651	0	0	1	0
MAFF	TGACGTACCATGTGGTGT	616	489	0	0	1	0
MAFF	TGACGTACCATGACTGGT	113	137	0	0	0	0
MAFF	GTACGTACCATGACTGCA	165	128	0	0	0	2
MAFF	ACACGTACCATGACTGAC	83	88	13	0	0	0
MAFF	TGTGCAACTGGTGTGTTG	739	406	1	0	0	0
MAFG	ACACGTACCAGTCAACCA	103	95	1	0	0	0
MAFG	GTACGTACCAGTCAACAC	44	57	0	0	0	0
MAFG	TGACGTACCAGTCAACTG	155	123	0	0	0	0
MAFG	ACACGTACCAGTCATGGT	340	531	0	0	0	0
MAFG	TGTGGTGTGTTGCACAAC	20	28	0	0	0	0
MAFK	GTACGTCAACACGTCACA	73	76	0	0	0	0
MAFK	ACACGTCAACACGTCAAC	47	32	0	0	0	0
MAFK	CAACGTCAACACGTCATG	126	252	0	0	0	0
MAFK	GTACGTCAACACGTACGT	156	112	0	0	0	0
MAFK	CAACGTCAACACGTACCA	518	419	0	0	0	1
MAG	TGTGGTTGTGTGACCAAC	172	57	0	0	0	0
MAG	ACTGGTTGTGTGACCATG	45	281	0	1	0	0
MAG	CATGGTTGTGTGACACGT	27	46	0	0	0	0
MAG	ACTGGTTGTGTGACACCA	206	208	0	0	0	0
MAG	GTTGGTTGTGTGACACAC	11	35	0	0	0	0
MAG	GTTGCAACGTTGACACAC	73	76	0	0	0	0
MAGEH1	CATGGTGTACCATGCAAC	14	13	0	0	0	0
MAGEH1	GTTGGTGTACCATGCATG	184	185	0	0	0	0
MAGEH1	TGTGGTGTACCATGACGT	225	175	0	1	0	0
MAGEH1	GTTGGTGTACCATGACCA	490	490	0	1	0	0
MAGEH1	ACTGGTGTACCATGACAC	40	51	1	0	0	0
MAGEH1	TGTGCAACGTTGTGGTCA	191	153	2	1	0	0
MAGI1	ACACTGTGACACCAACGT	148	270	0	0	0	0
MAGI1	TGACTGTGACACCAACCA	247	316	0	0	0	0
MAGI1	CAACTGTGACACCAACAC	14	30	0	0	0	0
MAGI1	TGTGGTTGTGTGCAGTAC	103	100	0	0	0	0
MAGI1	ACTGGTTGTGTGCAGTTG	843	178	0	0	0	1
MAGI2	ACTGGTTGGTCATGCAGT	357	750	1	1	1	1
MAGI2	TGTGGTTGGTCATGCACA	149	147	0	0	0	1
MAGI2	CATGGTTGGTCATGCAAC	16	17	0	0	0	0
MAGI2	GTTGGTTGGTCATGCATG	412	341	0	0	0	10
MAGI2	GTTGGTTGTGACGTTGCA	252	115	0	0	0	0
MAGI3	ACACCAACTGGTTGTGTG	305	194	0	0	0	0
MAGI3	TGACCAACTGCAGTGTGT	351	214	1	0	0	0
MAGI3	GTACCAACTGCAGTGTCA	134	76	0	0	0	0
MAGI3	ACACCAACTGCAGTGTAC	157	107	0	0	0	0
MAGI3	ACTGCAGTTGGTGTGTAC	56	83	0	0	0	0
MAGI3	GTTGCATGGTACCAACTG	1018	967	0	3	0	4
MAL	TGTGGTGTTGTGTGACTG	438	702	1	0	0	0
MAL	ACTGGTGTTGTGTGTGGT	75	85	0	0	0	0
MAL	TGTGGTGTTGTGTGTGCA	341	104	1	0	0	3
MAL	CATGGTGTTGTGTGTGAC	110	88	1	0	0	0
MAL	TGTGCACAGTGTACTGGT	481	263	0	1	0	0
MAL	GTTGCACATGACACTGTG	313	366	0	1	0	0
MALT1	CAACTGTGGTCATGCAAC	28	110	0	0	0	0
MALT1	GTACTGTGGTCATGCATG	254	199	0	0	0	1
MALT1	TGACTGTGGTCATGACGT	148	154	0	0	0	0
MALT1	GTACTGTGGTCATGACCA	98	80	1	0	0	0

MALT1	TGTGCAGTGTTGGTCAAC	15	22	0	0	0	0
MALT1	ACTGCATGGTGTACGTGT	276	282	0	0	0	0
MAML1	GTCATGACACTGACTGGT	318	414	0	0	1	2
MAML1	ACCATGACACTGACACAC	182	128	0	0	0	0
MAML1	CACATGACACTGACACTG	153	279	0	0	0	0
MAML1	CACATGACACTGACTGCA	310	451	1	0	0	0
MAML1	TGTGACCAACTGCACACA	217	612	1	1	0	3
MAML3	GTACTGACCACAACACGT	121	124	0	1	0	0
MAML3	CAACTGACCACAACACCA	317	217	1	1	0	0
MAML3	TGACTGACCACAACACAC	155	71	0	0	0	0
MAML3	ACACTGACCACAACACTG	423	345	0	0	0	30
MAML3	GTTGGTTGTGCAACGTGT	316	171	0	0	0	0
MAN1A1	ACACACCATGGTACGTTG	665	387	0	1	1	1
MAN1A1	CAACACCATGGTACGTCA	133	116	0	0	0	0
MAN1A1	TGACACCATGGTACGTAC	19	37	0	0	0	0
MAN1A1	CAACACCATGGTACCAGT	197	97	1	0	0	0
MAN1A1	ACACACCATGGTACCACA	309	95	1	0	0	0
MAN1A1	ACTGCAACTGCATGTGTG	431	662	14345	19	0	0
MAN1B1	TGTGGTACGTCACACAGT	154	430	0	1	3	2
MAN1B1	GTTGGTACGTCACACACA	136	136	0	0	0	0
MAN1B1	ACTGGTACGTCACACAAC	18	10	0	0	0	0
MAN1B1	CATGGTACGTCACACATG	550	221	2	0	0	3
MAN1B1	CATGGTTGACTGTGACGT	882	290	0	0	0	0
MAN2B1	TGACACCATGGTACACCA	288	491	0	0	0	0
MAN2B1	CAACACCATGGTACACAC	25	113	0	0	0	0
MAN2B1	GTACACCATGGTACACTG	529	667	0	1	0	0
MAN2B1	TGACACCATGGTACTGGT	252	388	0	0	0	0
MAN2B1	GTACACCATGGTACTGCA	449	269	0	0	0	4
MAN2B2	CAACACCATGGTTGGTTG	244	193	0	0	1	0
MAN2B2	TGTGCAGTTGGTTGACCA	230	293	1	0	1	2
MAN2B2	GTACACCATGGTTGCAGT	275	406	1	0	0	0
MAN2B2	CAACACCATGGTTGCACA	87	45	0	0	0	0
MAN2B2	TGACACCATGGTTGCAAC	33	35	0	0	0	0
MAN2C1	GTACACCATGGTACCAAC	6	4	0	0	0	0
MAN2C1	TGACACCATGGTACCATG	562	512	0	0	0	0
MAN2C1	ACACACCATGGTACACGT	86	112	0	0	0	0
MAN2C1	GTTGCAGTTGGTTGCAAC	40	25	0	0	0	0
MAN2C1	CATGACGTCAGTCACAGT	68	89	0	0	0	0
MANBA	GTACACCATGGTTGGTCA	162	171	1	0	1	2
MANBA	ACACACCATGGTACTGAC	9	16	0	0	0	0
MANBA	CAACACCATGGTACTGTG	83	131	1	0	0	0
MANBA	TGACACCATGGTTGGTGT	318	512	0	0	0	0
MANBA	ACACACCATGGTTGGTAC	27	26	1	0	0	0
MAOA	GTACACGTACTGACCACA	36	37	0	0	0	0
MAOA	ACACACGTACTGACCAAC	25	20	0	0	0	0
MAOA	CAACACGTACTGACCATG	233	302	0	0	0	0
MAOA	GTACACGTACTGACACGT	127	70	1	0	0	0
MAOA	CAACACGTACTGACACCA	239	255	0	0	0	0
MAOB	TGACACGTACTGACACAC	25	55	1	0	0	0
MAOB	ACACACGTACTGACACTG	747	814	0	0	0	5
MAOB	CAACACGTACTGACTGGT	878	274	0	1	0	0
MAOB	ACACACGTACTGACTGCA	208	192	0	0	0	0
MAOB	GTACACGTACTGACTGAC	79	83	0	1	0	0
MAP1A	ACTGGTGTACTGGTCATG	214	228	5	2471	1	2
MAP1A	CATGGTGTACTGGTCACA	60	93	1	0	0	0
MAP1A	TGTGGTGTACTGGTCAAC	26	88	0	0	0	0
MAP1A	CATGGTGTACTGGTACGT	225	280	1	0	0	0
MAP1A	ACTGGTGTACTGGTACCA	99	64	0	0	0	0
MAP1B	ACTGGTGTTGGTGTACGT	728	177	1	0	1	0
MAP1B	ACTGGTGTTGGTGTCACA	367	329	0	0	0	0
MAP1B	GTTGGTGTTGGTGTCAAC	42	27	0	0	0	0
MAP1B	TGTGGTGTTGGTGTCATG	196	447	1	0	0	0
MAP1B	TGTGGTGTTGGTGTACCA	147	139	1	955	0	0
MAP1LC3A	GTTGGTTGTGACACCACA	52	140	1	0	2	3
MAP1LC3A	TGTGGTTGTGACACCAGT	215	307	0	0	1	1
MAP1LC3A	GTTGGTCACAGTGTCAAC	15	24	0	0	0	0
MAP1LC3A	TGTGGTCACAGTGTCATG	169	160	0	0	0	0
MAP1LC3A	ACTGGTTGTGACACCAAC	103	24	0	0	0	0
MAP1LC3A	ACTGCATGCAGTACTGGT	338	308	1	0	0	0
MAP1LC3B	TGTGGTACACGTGTCAAC	7	17	0	0	0	0
MAP1LC3B	ACTGGTACACGTGTCATG	459	467	89	0	0	2
MAP1LC3B	CATGGTACACGTGTACGT	127	237	0	0	0	0
MAP1LC3B	ACTGGTACACGTGTACCA	170	160	0	1	0	0
MAP1LC3B	GTTGGTACACGTGTACAC	20	34	0	0	0	0
MAP2	TGTGGTGTACGTGTACGT	144	157	0	0	0	0

MAP2	GTTGGTGTACGTGTACCA	423	175	1	0	0	1
MAP2	ACTGGTGTACGTGTACAC	9	41	1	0	0	0
MAP2	CATGGTGTACGTGTACTG	418	392	0	0	0	1
MAP2	GTTGGTGTACGTGTTGGT	627	348	0	0	0	0
MAP2K1	GTCATGACCACACAACCA	400	430	1	0	1	0
MAP2K1	GTCATGACCACACACATG	282	353	1	0	0	0
MAP2K1	TGCATGACCACACAACGT	158	205	0	0	0	0
MAP2K1	ACCATGACCACACAACAC	30	29	0	0	0	0
MAP2K1	CACATGACCACACAACTG	813	704	1	0	0	0
MAP2K1	CATGCAACGTCAGTGTAC	101	102	0	0	0	0
MAP2K2	CAACGTGTCAACGTGTTG	1546	234	15	16	5	31
MAP2K2	GTACGTGTCAACGTGTCA	165	69	0	0	0	0
MAP2K2	ACACGTGTCAACGTGTAC	22	16	0	0	0	0
MAP2K2	GTACGTGTCAACGTCAGT	161	274	1	0	0	1
MAP2K2	ACACACTGGTTGACACTG	658	307	2	735	0	2
MAP2K2	GTTGCAACACCAACGTCA	432	515	3	0	0	0
MAP2K3	ACTGGTTGACACACCATG	236	216	0	0	1	0
MAP2K3	CATGGTTGACACACCACA	370	132	0	0	0	0
MAP2K3	TGTGGTTGACACACCAAC	25	4	0	0	0	0
MAP2K3	CATGGTTGACACACACGT	63	87	0	0	0	0
MAP2K3	ACTGGTTGACACACACCA	470	373	1	0	0	4
MAP2K4	TGCATGCATGTGACGTGT	328	277	1	0	0	0
MAP2K4	GTCATGCATGTGACGTCA	220	214	0	0	0	0
MAP2K4	ACCATGCATGTGACGTAC	35	58	0	0	0	0
MAP2K4	CACATGCATGTGACGTTG	425	500	0	2	0	0
MAP2K4	GTCATGCATGTGACCAGT	624	242	0	0	0	0
MAP2K4	ACTGCAACTGACGTGTGT	417	463	0	0	0	1
MAP2K5	GTCATGACGTGTGTGTTG	676	1334	2	0	1	6
MAP2K5	TGCATGACGTGTGTCAGT	101	142	1	0	0	0
MAP2K5	GTCATGACGTGTGTCACA	12	19	0	0	0	0
MAP2K5	ACCATGACGTGTGTCAAC	56	59	0	0	0	0
MAP2K5	CACATGACGTGTGTCATG	334	84	1	0	0	16
MAP2K5	ACTGCAACACCATGCAAC	34	103	0	3	0	1
MAP2K6	ACTGCACAGTCAGTGTCA	288	283	0	0	0	1
MAP2K6	GTTGCACAGTCAGTGTAC	221	396	1	0	0	2
MAP2K6	TGTGCACAGTCAGTGTTG	478	565	1	0	0	0
MAP2K6	ACTGCACAGTCAGTCAGT	136	121	0	1	0	0
MAP2K6	TGTGCACAGTCAGTCACA	75	74	0	0	0	0
MAP2K7	TGCATGCATGACACGTCA	249	161	1	0	0	0
MAP2K7	CACATGCATGACACGTAC	114	199	0	0	0	0
MAP2K7	GTTGCAGTACGTGTGTGT	177	236	1	0	0	0
MAP2K7	ACTGCAGTACGTCATGGT	238	256	1	0	0	3
MAP2K7	TGTGCAGTACGTCATGCA	243	281	1	0	0	1
MAP3K1	TGCATGTGTGTGTGGTAC	216	183	0	0	0	0
MAP3K1	ACCATGTGTGTGTGGTTG	470	225	0	1	0	0
MAP3K1	CACATGTGTGTGTGCAGT	100	181	0	0	0	0
MAP3K1	ACCATGTGTGTGTGCACA	61	64	0	0	0	0
MAP3K1	GTCATGTGTGTGTGCAAC	11	27	0	0	0	0
MAP3K10	CATGCAACACCATGCATG	142	138	0	0	1	0
MAP3K10	TGCATGACGTTGGTACCA	50	44	0	0	0	0
MAP3K10	CACATGACGTTGGTACAC	19	17	0	0	0	0
MAP3K10	GTCATGACGTTGGTACTG	134	89	0	0	0	0
MAP3K10	TGTGGTTGGTGTCATGGT	852	406	0	2	0	5
MAP3K10	GTTGGTTGGTGTCATGCA	324	374	1	1	0	3
MAP3K11	ACACGTTGACCAACCACA	143	206	2	2	6	5
MAP3K11	CAACGTTGACCAACCAGT	289	186	0	0	0	0
MAP3K11	GTACGTTGACCAACCAAC	9	5	0	0	0	0
MAP3K11	TGACGTTGACCAACCATG	49	122	1	0	0	0
MAP3K11	ACACGTTGACCAACACGT	93	81	0	0	0	0
MAP3K12	CACATGCATGCATGCACA	41	41	0	0	0	0
MAP3K12	TGCATGCATGCATGCAAC	5	7	0	0	0	0
MAP3K12	ACCATGCATGCATGCATG	36	26	0	0	0	0
MAP3K12	CACATGCATGCATGACGT	84	28	0	0	0	0
MAP3K12	ACCATGCATGCATGACCA	295	258	0	1	0	10
MAP3K12	GTTGCAACACCATGACGT	107	137	0	0	0	0
MAP3K13	GTTGCATGCACAGTACTG	98	195	1	0	1	21
MAP3K13	CAACGTGTCAACCAACCA	299	280	0	0	0	27
MAP3K13	TGACGTGTCAACCAACAC	70	44	0	0	0	0
MAP3K13	ACACGTGTCAACCAACTG	410	428	2	0	0	1
MAP3K13	CAACGTGTCAACCATGGT	704	991	3	1	0	7
MAP3K13	ACACGTGTCAACCATGCA	194	311	0	0	0	0
MAP3K14	TGTGGTTGTGGTGTCAAC	43	39	0	0	1	0
MAP3K14	GTCATGCAACTGGTCACA	103	93	0	0	0	0
MAP3K14	ACCATGCAACTGGTCAAC	13	10	0	0	0	0
MAP3K14	CACATGCAACTGGTCATG	76	81	0	0	0	0

MAP3K14	ACTGGTTGTGGTGTCATG	95	140	0	0	0	1
MAP3K2	ACCATGACGTTGACACTG	583	253	1	0	0	0
MAP3K2	CACATGACGTTGACTGGT	376	457	1	0	0	0
MAP3K2	ACCATGACGTTGACTGCA	326	304	1	0	0	0
MAP3K2	GTCATGACGTTGACTGAC	20	45	0	0	0	0
MAP3K2	GTACGTTGTGACACTGGT	143	158	0	0	0	0
MAP3K3	CACATGACCACAGTACCA	765	752	0	0	1	3
MAP3K3	ACCATGACCACAGTCAAC	44	30	1	0	0	0
MAP3K3	CACATGACCACAGTCATG	141	135	0	0	0	0
MAP3K3	GTCATGACCACAGTACGT	152	156	0	0	0	1
MAP3K3	TGTGGTTGACTGGTGTCA	144	118	1	0	0	0
MAP3K4	TGCATGCATGGTCATGTG	289	241	1	1	1650	2
MAP3K4	ACCATGCATGGTCATGCA	67	56	0	0	0	0
MAP3K4	GTCATGCATGGTCATGAC	14	21	0	0	0	0
MAP3K4	CACATGCATGGTACGTGT	144	155	0	0	0	0
MAP3K4	TGACTGTGACACCATGGT	226	167	0	0	0	0
MAP3K5	GTCATGCATGCATGGTCA	367	221	0	1	0	0
MAP3K5	ACCATGCATGCATGGTAC	18	17	0	0	0	0
MAP3K5	CACATGCATGCATGGTTG	347	373	2	0	0	5
MAP3K5	GTCATGCATGCATGCAGT	64	71	0	0	0	0
MAP3K5	CATGGTTGACTGGTGTAC	64	38	0	1	0	0
MAP3K5	GTTGCAACACCACACAAC	10	5	0	0	0	0
MAP3K7	TGTGCAACACCATGACAC	211	57	5	3	6	8
MAP3K7	TGCATGACGTGTACACCA	134	361	0	0	0	1
MAP3K7	CACATGACGTGTACACAC	54	61	0	1	0	0
MAP3K7	GTCATGACGTGTACACTG	362	570	0	1	0	1
MAP3K7	TGCATGACGTGTACTGGT	63	29	0	0	0	0
MAP3K7	GTCATGACGTGTACTGCA	272	184	0	0	0	0
MAP3K7IP1	CACATGACGTACCACATG	37	48	0	0	0	27
MAP3K7IP1	GTCATGACGTACCAACGT	153	332	0	0	0	0
MAP3K7IP1	CACATGACGTACCAACCA	276	352	0	1	0	0
MAP3K7IP1	TGCATGACGTACTGGTAC	354	87	0	0	0	0
MAP3K7IP1	ACCATGACGTACTGGTTG	273	364	1	0	0	3
MAP3K7IP2	CATGCAACACCATGACCA	296	345	0	0	1	0
MAP3K7IP2	GTCATGTGCAGTTGACCA	258	165	0	0	0	0
MAP3K7IP2	ACCATGTGCAGTTGACAC	132	48	0	0	0	0
MAP3K7IP2	CACATGTGCAGTTGACTG	201	165	0	0	0	0
MAP3K7IP2	GTCATGTGCAGTTGTGGT	349	259	0	0	0	0
MAP3K7IP2	CACATGTGCAGTTGTGCA	351	203	0	0	0	0
MAP3K7IP3	GTACGTGTACACACCAGT	77	127	0	0	0	0
MAP3K7IP3	CAACGTGTACACACCACA	147	109	0	0	0	0
MAP3K7IP3	TGACGTGTACACACCAAC	6	1	0	0	0	0
MAP3K7IP3	ACACGTGTACACACCATG	692	432	0	0	0	0
MAP3K7IP3	CAACGTGTACACACACGT	16	9	0	0	0	0
MAP3K8	TGTGGTACTGACGTCAAC	36	50	0	0	0	0
MAP3K8	ACTGGTACTGACGTCATG	128	218	0	0	0	0
MAP3K8	CATGGTACTGACGTACGT	89	61	2	0	0	0
MAP3K8	ACTGGTACTGACGTACCA	37	36	0	0	0	0
MAP3K8	GTTGGTACTGACGTACAC	23	32	0	0	0	0
MAP3K8	CATGCAACACACCAGTAC	93	126	0	0	0	0
MAP3K9	CAACGTTGACCACACAAC	41	51	0	0	0	0
MAP3K9	GTACGTTGACCACACATG	619	552	1	1	0	0
MAP3K9	TGACGTTGACCACAACGT	67	122	1	0	0	0
MAP3K9	GTACGTTGACCACAACCA	488	332	2	0	0	0
MAP3K9	ACACGTTGACCACAACAC	13	12	0	0	0	0
MAP4K1	TGCATGACGTCAGTACGT	225	239	2	0	2	0
MAP4K1	GTCATGACGTCAGTCATG	105	124	0	0	0	0
MAP4K1	GTCATGACGTCAGTACCA	309	180	0	0	0	0
MAP4K1	ACCATGACGTCAGTACAC	43	234	0	0	0	0
MAP4K1	CACATGACGTCAGTACTG	129	477	0	0	0	0
MAP4K2	ACCATGACCAGTACTGTG	106	75	0	0	0	0
MAP4K2	GTCATGACCAGTTGGTGT	438	715	2	0	0	0
MAP4K2	CACATGACCAGTTGGTCA	265	466	0	0	0	0
MAP4K2	TGCATGACCAGTTGGTAC	67	103	0	0	0	0
MAP4K2	ACCATGACCAGTTGGTTG	1433	1390	1	1	0	0
MAP4K2	GTTGCAACACACCAGTTG	1163	1436	1	1	0	2
MAP4K5	ACCATGACCAGTACCAGT	123	126	1	0	1	16
MAP4K5	ACCATGACCAGTACGTCA	102	130	0	0	0	0
MAP4K5	GTCATGACCAGTACGTAC	57	66	0	0	0	0
MAP4K5	TGCATGACCAGTACGTTG	462	288	1	0	0	0
MAP4K5	TGCATGACCAGTACCACA	220	138	0	0	0	0
MAPK1	TGTGGTACTGCATGGTCA	1448	1114	6	5	5	9965
MAPK1	ACACGTTGTGACGTACCA	224	318	0	1	0	2
MAPK1	GTTGGTACTGCAACTGTG	689	328	0	2	0	0
MAPK1	ACTGGTACTGCATGGTGT	195	207	0	0	0	0

MAPK1	GTTGCACAGTCAGTCATG	213	257	1	102	0	0
MAPK10	TGCATGCATGCATGTGCA	162	130	0	0	0	0
MAPK10	CACATGCATGCATGTGAC	10	13	0	0	0	0
MAPK10	GTCATGCATGCATGTGTG	413	1323	1	1	0	0
MAPK10	GTCATGCATGACGTGTGT	434	414	0	0	0	0
MAPK10	CACATGCATGACGTGTCA	545	614	0	0	0	4
MAPK10	ACTGCAACCATGCAGTCA	306	276	0	0	0	0
MAPK11	CATGGTACTGTGGTACAC	108	34	1	0	0	0
MAPK11	GTTGGTACTGTGGTACTG	172	164	0	0	0	0
MAPK11	TGTGGTACTGTGGTTGGT	14	34	0	0	0	0
MAPK11	GTTGGTACTGTGGTTGCA	552	380	0	0	0	1
MAPK11	ACTGGTACTGTGGTTGAC	24	34	0	0	0	0
MAPK12	TGCATGTGTGTGACCACA	133	120	0	0	0	0
MAPK12	CACATGTGTGTGACCAAC	35	43	0	0	0	0
MAPK12	GTCATGTGTGTGACCATG	112	104	0	0	0	0
MAPK12	TGCATGTGTGTGACACGT	120	61	0	1	0	0
MAPK12	GTCATGTGTGTGACACCA	132	152	919	2	0	1
MAPK13	CACATGCATGGTGTACGT	162	141	0	2	0	0
MAPK13	ACCATGCATGGTGTACCA	42	116	0	0	0	0
MAPK13	GTCATGCATGGTGTACAC	58	90	0	0	0	1
MAPK13	TGCATGCATGGTGTACTG	462	247	1	0	0	0
MAPK13	ACTGGTACTGACACCAGT	12	191	4	0	0	0
MAPK13	ACTGCAACCATGCACAGT	55	65	0	0	0	0
MAPK14	ACCATGCAACGTACGTGT	224	250	0	0	0	0
MAPK14	TGCATGCAACGTACGTCA	377	400	2	0	0	0
MAPK14	CACATGCAACGTACGTAC	13	27	0	0	0	0
MAPK14	GTCATGCAACGTACGTTG	470	1027	0	0	0	2
MAPK14	ACACGTTGTGACGTCATG	175	238	0	0	0	13
MAPK14	GTTGCAACCATGCAGTAC	71	79	0	0	0	1
MAPK3	GTCATGTGTGTGACTGGT	317	311	0	0	2	0
MAPK3	ACCATGTGTGTGACACAC	9	14	0	0	0	2
MAPK3	CACATGTGTGTGACACTG	183	200	0	0	0	0
MAPK3	GTTGGTTGACTGGTGTTG	230	243	0	0	0	1
MAPK3	TGTGGTTGACTGGTCAGT	229	230	0	2	0	0
MAPK7	ACCATGCATGTGGTACAC	25	32	0	0	0	0
MAPK7	CACATGCATGTGGTACTG	196	263	1	0	0	0
MAPK7	GTCATGCATGTGGTTGGT	561	528	2	0	0	0
MAPK7	CATGGTTGGTGTACACGT	115	191	0	0	0	0
MAPK7	ACTGGTTGGTGTACACCA	92	163	0	1	0	0
MAPK7	CATGCAACCATGCAGTGT	119	110	0	0	0	0
MAPK8	ACCATGCATGACCAACCA	123	205	0	1	1	0
MAPK8	GTCATGCATGACCAACAC	55	55	0	0	0	0
MAPK8	TGCATGCATGACCAACTG	117	139	0	0	0	0
MAPK8	CAACACTGGTTGACTGGT	210	214	0	0	0	0
MAPK8	TGTGGTACTGACTGTGGT	253	360	0	0	0	4
MAPK8	TGTGCAACCATGGTTGTG	218	297	0	0	0	0
MAPK8IP1	GTCATGCATGACGTACTG	101	210	0	0	1	0
MAPK8IP1	TGCATGCATGACGTTGGT	318	581	0	0	1	0
MAPK8IP1	TGCATGCATGACGTACCA	107	137	0	0	0	0
MAPK8IP1	CACATGCATGACGTACAC	21	21	0	0	0	0
MAPK8IP1	CATGCAGTGTTGCAACCA	574	410	1	0	0	0
MAPK8IP1	TGTGCAACCATGCAGTTG	631	670	1	0	0	0
MAPK8IP2	GTACCAACTGACGTGTAC	70	134	0	0	0	0
MAPK8IP2	TGACCAACTGACGTGTTG	584	572	0	0	0	2
MAPK8IP2	ACACCAACTGACGTCAGT	537	440	0	0	0	2
MAPK8IP2	TGACCAACTGACGTCACA	57	113	0	1	0	0
MAPK8IP2	CATGCAGTACTGTGTGAC	74	125	0	0	0	0
MAPK8IP3	CAACCAACTGCATGTGTG	80	79	0	0	0	0
MAPK8IP3	CAACCAACTGACGTGTGT	380	448	0	0	0	0
MAPK8IP3	ACACCAACTGACGTGTCA	50	85	1	0	0	0
MAPK8IP3	GTTGCAGTTGGTGTGTCA	448	354	0	0	0	12
MAPK8IP3	ACTGACCATGACACCAAC	31	23	0	0	0	0
MAPK9	TGCATGCATGCAGTTGTG	235	312	1	0	0	1
MAPK9	CACATGCATGCACAGTGT	1374	1115	1	1	0	1
MAPK9	ACCATGCATGCACAGTCA	655	589	0	0	0	0
MAPK9	GTCATGCATGCACAGTAC	51	94	0	0	0	0
MAPK9	TGCATGCATGCACAGTTG	611	291	1	1	0	0
MAPK9	ACTGCAACCATGACCACA	29	44	0	0	0	0
MAPKAPK2	CACATGACCAGTTGTGTG	1140	564	0	1	1	0
MAPKAPK2	ACCATGACCAGTTGTGAC	67	105	0	0	0	0
MAPKAPK2	ACCATGACCACAGTGTGT	748	564	1	0	0	0
MAPKAPK2	TGCATGACCACAGTGTCA	438	605	0	1	0	1
MAPKAPK2	CACATGACCACAGTGTAC	166	146	0	1	0	0
MAPKAPK2	TGTGCATGCACATGGTTG	535	480	0	1	0	0
MAPKAPK3	CACATGTGTGTGACTGCA	129	248	0	0	0	0

MAPKAPK3	TGCATGTGTGTGACTGAC	129	68	0	0	0	1
MAPKAPK3	ACCATGTGTGTGACTGTG	589	494	1	1	0	0
MAPKAPK3	GTCATGTGTGTGTGGTGT	2046	1748	2	2	0	1
MAPKAPK3	CACATGTGTGTGTGGTCA	36	49	0	1	0	0
MAPKAPK5	TGCATGCAACACACCAAC	93	11	0	0	0	0
MAPKAPK5	ACCATGCAACACACCATG	52	160	0	0	0	0
MAPKAPK5	CACATGCAACACACACGT	16	3	0	0	0	0
MAPKAPK5	ACCATGCAACACACACCA	247	503	0	0	0	0
MAPKAPK5	GTCATGCAACACACACAC	18	16	0	0	0	0
MAPKAPK5	TGTGCAACCATGACCATG	49	82	0	0	0	0
MAPRE2	GTTGGTGTACTGACTGGT	132	277	0	0	0	0
MAPRE2	CATGGTGTACTGACTGCA	596	226	2	0	0	0
MAPRE2	TGTGGTGTACTGACTGAC	63	45	0	0	0	0
MAPRE2	ACTGGTGTACTGACTGTG	423	344	1	0	0	0
MAPRE2	GTTGGTGTACTGTGGTGT	500	242	0	0	0	0
MAPRE2	GTTGCACATGCACAACGT	262	181	0	2	0	0
MAPT	CATGGTGTGTACCAGTAC	48	73	0	0	0	0
MAPT	GTTGGTGTGTACCAGTTG	1440	1512	1	0	0	13
MAPT	CATGGTGTCAGTGTCACA	192	224	0	0	0	0
MAPT	TGTGACCAACGTTGACGT	191	244	0	0	0	0
MAPT	GTTGACCAACGTTGACCA	243	238	0	0	0	0
MARCKS	CAACCAGTACGTGTGTTG	320	421	0	0	1	0
MARCKS	CAACCAGTCATGTGTGTG	563	555	0	1	0	0
MARCKS	TGACCAGTACGTGTGTGT	230	295	1	0	0	0
MARCKS	GTACCAGTACGTGTGTCA	276	278	0	0	0	0
MARCKS	ACACCAGTACGTGTGTAC	63	272	0	0	0	0
MARCO	CAACCAGTTGCATGTGGT	214	174	0	0	1	0
MARCO	GTACCAGTTGCATGTGAC	49	44	1	0	1	0
MARCO	TGACCAGTTGACGTGTGT	429	509	1	0	1	2
MARCO	ACACCAGTTGCATGTGCA	138	146	0	0	0	1
MARCO	TGACCAGTTGCATGTGTG	364	408	1	0	0	1
MARS	ACACACGTCATGACCACA	178	283	0	0	0	0
MARS	GTACACGTCATGACCAAC	9	9	0	0	0	0
MARS	TGACACGTCATGACCATG	93	104	0	0	0	0
MARS	ACACACGTCATGACACGT	181	227	0	0	0	0
MARS	TGTGCAGTCAGTTGTGCA	65	56	0	0	0	2
MARS	ACTGCAACGTGTCATGCA	29	62	1	0	0	1
MARS2	CAACACGTACGTTGGTTG	479	1125	1	0	0	0
MARS2	GTACACGTACGTTGCAGT	79	70	0	0	0	0
MARS2	CAACACGTACGTTGCACA	78	38	0	0	0	0
MARS2	TGACACGTACGTTGCAAC	111	10	0	0	0	0
MARS2	ACACACGTACGTTGCATG	57	58	1	0	0	0
MAS1	CAACGTCACATGTGGTAC	105	127	0	0	0	0
MAS1	GTACGTCACATGTGGTTG	1244	1864	1	0	0	1
MAS1	TGACGTCACATGTGCAGT	129	192	0	0	0	0
MAS1	GTACGTCACATGTGCACA	61	54	0	0	0	0
MAS1	ACACGTCACATGTGCAAC	28	48	0	0	0	1
MAS1	ACTGCACAACCATGGTCA	555	446	503	29	0	1
MASP1	CAACACGTTGTGTGGTCA	902	545	3	1	1	0
MASP1	ACACACGTTGTGACTGTG	250	480	0	0	0	0
MASP1	GTACACGTTGTGTGGTGT	187	320	4	0	0	0
MASP1	TGACACGTTGTGTGGTAC	35	22	0	0	0	0
MASP1	ACACACGTTGTGTGGTTG	657	308	0	1	0	1
MASP2	GTACACTGGTCACAACTG	670	899	0	0	1	5
MASP2	TGACACTGGTCACATGGT	162	110	2	0	0	0
MASP2	GTACACTGGTCACATGCA	83	33	0	0	0	0
MASP2	ACACACTGGTCACATGAC	25	28	0	0	0	0
MASP2	CAACACTGGTCACATGTG	511	558	1	1	0	0
MASP2	TGTGCAACCATGACTGGT	126	169	0	0	0	0
MAT1A	CAACCAACCATGGTACTG	230	346	1	1	0	0
MAT1A	GTACCAACCATGGTTGGT	152	217	0	0	0	0
MAT1A	CAACCAACCATGGTTGCA	23	72	0	0	0	0
MAT1A	TGACCAACCATGGTTGAC	95	74	0	0	0	0
MAT1A	ACACCAACCATGGTTGTG	354	408	1	0	0	0
MAT2A	ACACCAACCATGCATGAC	77	92	0	0	0	0
MAT2A	CAACCAACCATGCATGTG	491	678	0	0	0	0
MAT2A	TGACCAACCATGACGTGT	362	587	0	0	0	0
MAT2A	GTACCAACCATGACGTCA	339	395	0	1	0	1
MAT2A	CATGGTGTACGTTGCATG	158	174	1	0	0	0
MAT2B	GTACCACACACACATGCA	723	327	0	1	0	0
MAT2B	ACACCACACACACATGAC	98	155	0	0	0	0
MAT2B	CAACCACACACACATGTG	738	771	1	0	0	1
MAT2B	TGACCACACACAACGTGT	913	2270	2	1	0	52
MAT2B	ACTGGTACTGTGTGGTCA	700	450	0	1	0	0
MAX	GTACCATGCACAACTGGT	262	347	0	0	1	0

MAX	CAACCATGCACAACTGCA	14	48	0	0	0	0
MAX	TGACCATGCACAACTGAC	115	154	0	0	0	11
MAX	ACACCATGCACAACTGTG	1116	943	3	0	0	0
MAX	GTACCATGCACATGGTGT	353	255	0	1	0	0
MAX	CATGCAACACCAACTGAC	8	26	0	0	0	0
MB	TGACTGCAGTACTGTGAC	51	73	0	0	0	0
MB	ACACTGCAGTACTGTGTG	151	155	0	0	0	0
MB	CAACTGCAGTTGGTGTGT	1244	765	0	1	0	5
MB	ACACTGCAGTTGGTGTCA	116	104	0	0	0	0
MB	ACTGACGTGTACGTTGTG	159	173	0	0	0	0
MBD1	ACACGTACCAGTTGCAGT	90	84	2	0	0	0
MBD1	TGACGTACCAGTTGCACA	83	82	0	0	0	0
MBD1	CAACGTACCAGTTGCAAC	37	48	0	0	0	0
MBD1	GTACGTACCAGTTGCATG	338	503	1	2	0	0
MBD1	TGACGTACCAGTTGACGT	365	309	0	1	0	0
MBD1	TGTGCACAACTGTGTGGT	648	615	0	1	0	5
MBD4	CAACGTCAACTGGTCACA	38	45	0	0	0	1
MBD4	TGACGTCAACTGGTCAAC	33	41	0	0	0	0
MBD4	ACACGTCAACTGGTCATG	481	207	0	0	0	0
MBD4	CAACGTCAACTGGTACGT	269	363	1	0	0	0
MBD4	ACACGTCAACTGGTACCA	57	78	0	0	0	0
MBD4	CATGCACAGTTGTGGTGT	320	368	1	0	0	0
MBL2	GTACACTGTGGTGTGTGT	454	407	2	1	1	98
MBL2	CAACACTGACTGTGTGTG	1314	746	0	3	0	0
MBL2	CAACACTGTGGTGTGTCA	373	575	2	1	0	0
MBL2	TGACACTGTGGTGTGTAC	2	11	0	0	0	0
MBL2	ACACACTGTGGTGTGTTG	169	159	32	0	0	0
MBL2	ACTGCACACAACCACATG	581	641	0	0	0	0
MBP	CATGGTGTGTTGTGTGCA	140	168	0	0	0	0
MBP	TGTGGTGTACACTGTGCA	171	202	0	0	0	1
MBP	CATGGTGTACACTGTGAC	76	85	0	0	0	1
MBP	GTTGGTGTACACTGTGTG	265	380	1	0	0	0
MBP	CATGCACAGTGTCATGCA	185	155	1	0	0	0
MBTPS1	CAACGTCAACACTGACGT	184	163	0	0	0	0
MBTPS1	ACACGTCAACACTGACCA	136	167	0	0	0	0
MBTPS1	GTACGTCAACACTGACAC	34	33	0	0	0	0
MBTPS1	TGACGTCAACACTGACTG	124	140	0	0	0	1
MBTPS1	ACACGTCAACACTGTGGT	743	701	1	0	0	10
MBTPS2	GTACGTACACGTGTGTTG	495	786	0	1	0	0
MBTPS2	TGACGTACACGTGTCAGT	77	129	0	0	0	0
MBTPS2	GTACGTACACGTGTCACA	125	90	0	0	0	0
MBTPS2	ACACGTACACGTGTCAAC	3	8	0	0	0	0
MBTPS2	CAACGTACACGTGTCATG	211	26	0	0	0	0
MBTPS2	TGTGCACAACTGTGCATG	78	106	1	0	0	0
MC2R	CAACGTCACATGTGCATG	689	700	1	0	0	0
MC2R	GTACGTCACATGTGACGT	64	66	0	0	0	0
MC2R	CAACGTCACATGTGACCA	182	93	0	0	0	0
MC2R	TGACGTCACATGTGACAC	61	97	0	1	0	0
MC2R	ACACGTCACATGTGACTG	872	464	1	0	0	0
MC3R	CAACGTCAACGTGTGTGT	270	486	0	0	3	1
MC3R	CAACGTCACATGTGTGGT	514	359	0	1	0	0
MC3R	ACACGTCACATGTGTGCA	287	135	0	0	0	1
MC3R	GTACGTCACATGTGTGAC	15	12	0	0	0	0
MC3R	TGACGTCACATGTGTGTG	468	456	0	0	0	25
MC3R	GTTGCAACGTTGACCAGT	162	186	0	0	0	0
MC4R	TGACGTCAACGTGTCACA	70	109	0	299	2	1
MC4R	ACACGTCAACGTGTGTCA	64	60	0	0	0	0
MC4R	GTACGTCAACGTGTGTAC	23	6	0	0	0	0
MC4R	TGACGTCAACGTGTGTTG	718	251	1	2	0	0
MC4R	ACACGTCAACGTGTCAGT	41	39	0	0	0	0
MC5R	GTACGTCAACGTGTCATG	158	238	1	0	1	0
MC5R	CAACGTCAACGTGTCAAC	35	33	0	0	0	0
MC5R	TGACGTCAACGTGTACGT	617	216	0	0	0	0
MC5R	GTACGTCAACGTGTACCA	359	227	0	0	0	0
MC5R	ACTGCACAACGTACACCA	81	95	0	0	0	0
MC5R	GTTGCACAACGTACACAC	11	7	0	0	0	0
MCAT	TGTGGTTGGTCAACTGTG	1014	592	1	1	0	0
MCAT	CATGGTTGGTCATGGTGT	444	1474	0	0	0	0
MCAT	ACTGGTTGGTCATGGTCA	148	156	0	0	0	0
MCAT	GTTGGTTGGTCATGGTAC	27	47	0	0	0	0
MCAT	TGTGGTTGGTCATGGTTG	228	258	0	0	0	0
MCC	TGACCAACTGACTGGTCA	490	403	0	0	1	0
MCC	CAACCAACTGACACTGAC	208	73	0	0	0	0
MCC	GTACCAACTGACACTGTG	277	531	0	0	0	1
MCC	ACACCAACTGACTGGTGT	738	843	96	2	0	0

MCC	ACTGACACACACTGACCA	134	147	0	0	0	0
MCF2	CAACCATGACCAACGTTG	454	474	2	3	13	8
MCF2	CAACCAACTGACTGGTAC	24	20	0	0	0	0
MCF2	GTACCAACTGACTGGTTG	1254	1708	2	1	0	0
MCF2	TGACCAACTGACTGCAGT	223	157	1	0	0	0
MCF2	GTACCAACTGACTGCACA	189	196	0	0	0	1
MCFD2	GTTGGTCAGTACGTCAAC	5	13	0	0	0	0
MCFD2	TGTGGTCAGTACGTCATG	102	132	0	0	0	0
MCFD2	ACTGGTCAGTACGTACGT	175	526	0	0	0	0
MCFD2	TGTGGTCAGTACGTACCA	124	328	0	0	0	0
MCFD2	CATGGTCAGTACGTACAC	28	38	0	0	0	0
MCHR1	TGACGTCACAGTACCACA	123	137	0	0	0	0
MCHR1	CAACGTCACAGTACCAAC	62	15	0	0	0	0
MCHR1	GTACGTCACAGTACCATG	154	142	0	0	0	0
MCHR1	TGACGTCACAGTACACGT	24	32	0	0	0	0
MCHR1	GTTGACCAACACTGGTTG	429	345	2	0	0	0
MCL1	CACATGTGACACACCAAC	8	5	0	0	0	0
MCL1	GTCATGTGACACACCATG	181	162	1	0	0	0
MCL1	TGCATGTGACACACACGT	9	18	0	0	0	0
MCL1	GTCATGTGACACACACCA	383	617	0	0	0	0
MCL1	GTTGGTACTGTGCATGTG	266	337	0	0	0	0
MCM10	TGTGGTTGTGTGGTCACA	70	161	0	0	0	0
MCM10	CATGGTTGTGTGGTCAAC	11	24	0	0	0	0
MCM10	GTTGGTTGTGTGGTCATG	433	461	0	0	0	0
MCM10	TGTGGTTGTGTGGTACGT	83	68	0	0	0	0
MCM10	GTTGGTTGTGTGGTACCA	53	57	0	0	0	0
MCM2	ACACGTTGGTGTGTACGT	13	16	0	0	0	0
MCM2	TGACGTTGGTGTGTACCA	303	81	0	0	0	1
MCM2	CAACGTTGGTGTGTACAC	26	28	0	0	0	0
MCM2	GTACGTTGGTGTGTACTG	153	115	0	1	0	0
MCM2	TGACGTTGGTGTGTTGGT	325	296	0	0	0	0
MCM3	TGACGTTGGTGTCACAAC	7	6	0	0	0	0
MCM3	ACACGTTGGTGTCACATG	160	128	1	0	0	0
MCM3	CAACGTTGGTGTCAACGT	180	206	0	0	0	0
MCM3	ACACGTTGGTGTCAACCA	28	45	0	0	0	0
MCM3	GTACGTTGGTGTCAACAC	10	13	0	0	0	0
MCM4	GTACTGTGGTACACACTG	72	51	0	0	0	0
MCM4	TGACTGTGGTACACTGGT	170	376	0	0	0	1
MCM4	GTACTGTGGTACACTGCA	342	154	0	0	0	2
MCM4	ACACTGTGGTACACTGAC	70	91	0	0	0	0
MCM4	CAACTGTGGTACACTGTG	401	545	0	1	0	1
MCM5	GTCATGCACACATGCACA	109	110	0	0	1	0
MCM5	GTCATGCACACATGGTTG	475	264	0	0	0	0
MCM5	TGCATGCACACATGCAGT	105	110	0	0	0	0
MCM5	ACCATGCACACATGCAAC	43	23	0	0	0	0
MCM5	CATGCAGTACGTCATGAC	21	31	0	0	0	0
MCM6	ACACGTTGGTGTTGACAC	7	16	0	0	0	0
MCM6	CAACGTTGGTGTTGACTG	64	137	0	0	0	0
MCM6	GTACGTTGGTGTTGTGGT	310	380	0	0	0	0
MCM6	CAACGTTGGTGTTGTGCA	120	150	0	0	0	0
MCM6	TGACGTTGGTGTTGTGAC	13	8	0	0	0	1
MCM7	ACACGTTGGTCACAGTGT	119	111	0	0	0	0
MCM7	TGACGTTGGTCACAGTCA	95	115	0	0	0	0
MCM7	CAACGTTGGTCACAGTAC	116	117	1	0	0	0
MCM7	GTACGTTGGTCACAGTTG	782	532	0	0	0	1
MCM7	ACTGCATGCATGGTACCA	186	211	1	1	0	0
MCPH1	TGTGGTGTGTCACAACCA	622	400	0	27	1	16
MCPH1	TGTGGTGTGTCACACATG	499	395	0	1	0	0
MCPH1	ACTGGTGTGTCACAACGT	263	324	1	0	0	0
MCPH1	CATGGTGTGTCACAACAC	20	30	0	0	0	0
MCPH1	GTTGGTGTGTCACAACTG	413	398	1	1	0	2
MCRS1	TGTGGTCATGCAACTGTG	190	244	0	0	0	0
MCRS1	CATGGTCATGCATGGTGT	505	831	0	0	0	10
MCRS1	ACTGGTCATGCATGGTCA	367	337	1	1	0	0
MCRS1	GTTGGTCATGCATGGTAC	160	282	2	0	0	0
MCRS1	ACTGGTTGCATGCACATG	226	237	0	0	0	0
MDC1	TGACGTACACTGACGTCA	104	104	0	0	0	0
MDC1	CAACGTACACTGACGTAC	193	110	0	0	0	2
MDC1	GTACGTACACTGACGTTG	90	105	3	0	0	4
MDC1	TGACGTACACTGACCAGT	413	402	0	0	0	0
MDC1	GTACGTACACTGACCACA	187	211	0	0	0	0
MDC1	TGTGCACAACTGCAGTCA	161	93	1	0	0	0
MDH1	GTACCAGTCAACACACTG	332	309	0	0	1	4
MDH1	TGACCAGTCAACGTGTCA	169	347	2	0	0	0
MDH1	CAACCAGTCAACGTGTAC	59	61	0	0	0	0

MDH1	GTACCAGTCAACGTGTTG	637	436	1	1	0	0
MDH1	TGACCAGTCAACGTCAGT	415	221	0	0	0	0
MDH1	GTTGCAACACCATGTGAC	18	23	0	0	0	0
MDH2	GTACCAGTCACATGACCA	319	310	0	0	1	0
MDH2	GTACCAGTCACATGTGGT	605	870	1	2	1	0
MDH2	TGACCAGTCACATGACGT	425	128	0	0	0	0
MDH2	ACACCAGTCACATGACAC	27	45	0	0	0	0
MDH2	CAACCAGTCACATGACTG	185	414	0	0	0	0
MDH2	TGTGCAACACCATGTGTG	419	242	0	0	0	0
MDM2	GTCATGACACTGTGTGCA	155	111	0	1	0	0
MDM2	ACCATGACACTGTGTGAC	40	36	0	0	0	0
MDM2	CACATGACACTGTGTGTG	200	196	0	0	0	0
MDM2	GTCATGACTGGTGTGTGT	275	625	2	0	0	0
MDM2	CACATGACTGGTGTGTCA	151	230	1	0	0	0
MDM4	TGCATGTGGTCAGTGTCA	834	565	0	0	1	0
MDM4	TGACCATGGTACTGACGT	353	165	0	1	1	0
MDM4	CACATGTGGTCAGTGTAC	66	79	0	0	0	0
MDM4	GTCATGTGGTCAGTGTTG	556	412	0	0	0	0
MDM4	TGCATGTGGTCAGTCAGT	428	86	0	0	0	0
ME1	GTACTGACACACTGTGGT	382	459	0	0	1	0
ME1	CAACTGACACACTGACTG	580	500	2	0	0	1
ME1	CAACTGACACACTGTGCA	177	285	2	0	0	0
ME1	TGACTGACACACTGTGAC	112	72	0	0	0	0
ME1	ACACTGACACACTGTGTG	466	783	1	1	0	0
ME2	ACACTGACACTGGTGTCA	536	730	0	3	2	0
ME2	CAACTGACACTGGTGTGT	158	642	0	2	0	0
ME2	GTACTGACACTGGTGTAC	77	58	0	0	0	0
ME2	TGACTGACACTGGTGTTG	365	214	0	0	0	0
ME2	TGTGGTGTACCACATGCA	364	99	0	1	0	0
ME3	ACACTGACACTGACGTAC	21	40	1	0	0	0
ME3	CAACTGACACTGACGTTG	1250	1516	1	2	0	0
ME3	GTACTGACACTGACCAGT	189	232	0	0	0	0
ME3	CAACTGACACTGACCACA	182	151	0	0	0	0
ME3	TGACTGACACTGACCAAC	44	61	0	1	0	0
MECP2	CAACGTTGACGTACGTGT	234	171	0	0	0	0
MECP2	ACACGTTGACGTACGTCA	42	73	0	0	0	0
MECP2	GTACGTTGACGTACGTAC	21	14	0	0	0	0
MECP2	TGACGTTGACGTACGTTG	991	418	1	1	0	0
MECP2	ACACGTTGACGTACCAGT	168	158	0	0	0	0
MECP2	GTTGCACAACTGTGTGCA	231	134	0	0	0	0
MECR	TGACTGACGTACACGTGT	168	199	0	0	0	0
MECR	GTACTGACGTACACGTCA	226	113	0	0	0	0
MECR	ACACTGACGTACACGTAC	179	155	0	1	0	0
MECR	CAACTGACGTACACGTTG	244	423	0	0	0	3
MECR	ACTGGTTGTGACCATGGT	145	109	0	0	0	0
MEF2A	GTTGCACAACGTCAGTGT	256	229	0	0	1	5
MEF2A	ACCATGTGACGTCAACAC	60	45	8	0	0	9
MEF2A	CACATGTGACGTCAACTG	600	914	4	3	0	6
MEF2A	GTCATGTGACGTCATGGT	665	421	1	1	0	1
MEF2A	CACATGTGACGTCATGCA	100	86	0	1	0	0
MEF2A	TGCATGTGACGTCATGAC	79	83	0	0	0	0
MEF2B	TGACGTACCACAACACGT	43	45	0	0	0	0
MEF2B	GTACGTACCACAACACCA	513	163	0	1	0	1
MEF2B	ACACGTACCACAACACAC	176	103	1	0	0	0
MEF2B	CAACGTACCACAACACTG	44	54	0	0	0	0
MEF2B	TGTGGTTGTGGTTGGTAC	8	9	0	0	0	0
MEF2B	TGTGCACAACGTACACTG	461	566	1	0	0	29
MEF2C	TGACGTACCACATGCATG	371	337	0	1	1	0
MEF2C	TGACGTACCACATGACCA	140	115	0	0	1	0
MEF2C	CAACGTCAACCACAACGT	316	375	3	1	0	163
MEF2C	GTACGTACCACATGCAAC	12	14	0	0	0	0
MEF2C	ACACGTACCACATGACGT	408	152	0	0	0	0
MEF2C	GTTGCAACCACACAGTTG	1183	679	1	1	0	0
MEF2D	TGACGTACCAACGTACGT	729	128	0	1	0	88
MEF2D	GTACGTACCAACGTACCA	267	72	0	0	0	0
MEF2D	ACACGTACCAACGTACAC	21	18	0	0	0	0
MEF2D	CAACGTACCAACGTACTG	38	22	0	1	0	0
MEF2D	GTACGTACCAACGTTGGT	188	588	0	0	0	0
MEFV	TGACCACACAGTGTTGTG	395	539	0	0	0	2
MEFV	CAACCACACAGTCAGTGT	586	565	1	0	0	3
MEFV	ACACCACACAGTCAGTCA	297	182	1	0	0	0
MEFV	GTACCACACAGTCAGTAC	249	50	0	0	0	0
MEFV	TGACCACACAGTCAGTTG	381	558	0	0	0	0
MEFV	CATGCACACAACCAACGT	221	773	0	0	0	0
MEIS1	TGACGTCAACCAACACAC	170	72	0	0	0	0

MEIS1	ACACGTCAACCAACACTG	512	491	1	0	0	0
MEIS1	CAACGTCAACCAACTGGT	37	16	0	0	0	0
MEIS1	ACACGTCAACCAACTGCA	119	92	0	0	0	1
MEIS1	ACACGTACCAACCACACA	192	146	0	0	0	13
MELK	GTCATGACGTCAGTTGGT	171	294	0	0	0	0
MELK	CACATGACGTCAGTTGCA	95	77	0	0	0	1
MELK	TGCATGACGTCAGTTGAC	9	5	0	0	0	0
MELK	ACCATGACGTCAGTTGTG	80	318	0	0	0	0
MELK	GTCATGACGTCACAGTGT	758	724	1	2	0	0
MEN1	TGACCATGACGTGTTGTG	212	204	0	0	1	5
MEN1	CAACCATGACGTCAGTGT	677	892	0	6	1	12
MEN1	ACACCATGACGTGTTGCA	32	44	0	0	0	0
MEN1	GTACCATGACGTGTTGAC	2	8	0	0	0	0
MEN1	ACACCATGACGTCAGTCA	51	56	0	0	0	7
MEN1	ACTGCAACCAGTCATGGT	321	305	1	1	0	0
MEOX2	GTACGTACACTGCAGTCA	491	382	0	0	0	0
MEOX2	ACACGTACACTGCAGTAC	60	40	0	0	0	5
MEOX2	CAACGTACACTGCAGTTG	225	294	5	0	0	0
MEOX2	GTACGTACACTGCACAGT	450	424	1	1	0	0
MEOX2	CAACGTACACTGCACACA	109	70	0	0	0	0
MEPE	CATGGTACACACCAGTTG	1003	446	0	0	2	0
MEPE	ACTGGTACACACCAGTAC	69	57	0	0	0	0
MEPE	GTTGGTACACACCACAGT	177	192	0	1	0	7
MEPE	CATGGTACACACCACACA	298	175	1	2	0	0
MEPE	TGTGGTACACACCACAAC	2	4	0	0	0	0
MERTK	CACATGCATGGTACCAAC	9	4	0	0	0	0
MERTK	GTCATGCATGGTACCATG	101	130	0	0	0	40
MERTK	TGCATGCATGGTACACGT	14	22	0	0	0	0
MERTK	GTCATGCATGGTACACCA	107	84	0	0	0	12
MERTK	TGTGGTACTGACACCACA	28	29	0	0	0	0
MEST	GTACCAGTTGTGACGTTG	956	488	0	0	1	0
MEST	TGACCAGTTGTGACCAGT	39	55	1	0	0	0
MEST	ACACTGTGCACACAACGT	43	19	1	0	0	0
MEST	TGACTGTGCACACAACCA	158	228	0	0	0	0
MEST	CAACTGTGCACACAACAC	60	62	0	0	0	0
MEST	ACTGCATGGTACGTTGTG	519	469	0	0	0	2
MET	GTCATGCACAACTGCAAC	25	20	0	0	0	0
MET	TGCATGCACAACTGCATG	146	293	0	0	0	1
MET	ACCATGCACAACTGACGT	190	253	0	0	0	0
MET	TGCATGCACAACTGACCA	446	670	0	0	0	0
MET	CAACCATGCATGGTTGGT	87	84	0	0	0	0
METTL2B	CAACCACACAACCATGGT	846	1335	14	11	3	5
METTL2B	ACACCACACAACCATGCA	284	229	1	0	0	0
METTL2B	GTACCACACAACCATGAC	26	28	0	0	0	0
METTL2B	TGACCACACAACCATGTG	146	65	1	0	0	0
METTL2B	CATGGTACGTTGTGGTTG	110	211	0	0	0	0
METTL6	CATGGTACGTACTGCATG	850	457	0	0	1	4
METTL6	ACTGGTACGTACTGCAAC	27	33	0	0	0	0
METTL6	GTTGGTACGTACTGACGT	423	520	0	0	0	0
METTL6	CATGGTACGTACTGACCA	338	336	0	0	0	0
METTL6	TGTGGTACGTACTGACAC	26	31	0	0	0	0
MFGE8	GTACCAGTACCATGTGTG	262	257	0	0	0	0
MFGE8	GTACCAGTACACGTGTGT	625	594	0	0	0	1
MFGE8	CAACCAGTACACGTGTCA	400	434	6	0	0	0
MFGE8	TGACCAGTACACGTGTAC	92	47	0	0	0	0
MFGE8	ACACCAGTACACGTGTTG	360	502	0	1	0	5
MFGE8	ACTGCACAACGTACTGGT	92	54	0	0	0	0
MFNG	CATGGTGTCAACGTCAGT	445	133	0	0	0	0
MFNG	ACTGGTGTCAACGTCACA	156	179	1	0	0	0
MFNG	GTTGGTGTCAACGTCAAC	22	48	0	0	0	0
MFNG	GTTGCACAGTGTCAGTAC	14	25	0	0	0	0
MFNG	TGTGCAACACCACACATG	211	228	0	0	0	0
MFNG	ACTGCAACACCACAACGT	305	119	0	0	0	0
MGAM	TGACACCATGGTCACACA	119	251	0	0	0	0
MGAM	CAACACCATGGTCACAAC	14	29	0	0	0	0
MGAM	GTACACCATGGTCACATG	388	165	0	0	0	0
MGAM	CATGACACGTACTGGTCA	82	197	0	0	0	0
MGAM	TGTGACACGTACTGGTAC	99	9	0	1	0	0
MGAT1	CAACGTACACTGCATGAC	46	15	0	0	0	0
MGAT1	TGACCACAACTGCATGAC	56	55	0	0	0	0
MGAT1	ACACCACAACTGCATGTG	1239	709	0	0	0	0
MGAT1	GTACCACAACTGACGTGT	517	639	1	0	0	1
MGAT1	CAACCACAACTGACGTCA	539	283	0	0	0	1
MGAT1	CATGCACAACCATGCAAC	151	66	1	0	0	0
MGAT2	ACCATGTGACGTACACGT	42	24	0	0	0	0

MGAT2	TGCATGTGACGTACACCA	102	120	0	0	0	0
MGAT2	CACATGTGACGTACACAC	23	12	0	0	0	0
MGAT2	GTCATGTGACGTACACTG	301	157	0	0	0	0
MGAT2	TGCATGTGACGTACTGGT	169	242	0	0	0	0
MGAT3	GTACCAACGTCACACAAC	16	42	0	0	0	0
MGAT3	TGACCAACGTCACACATG	303	319	1	0	0	0
MGAT3	ACACCAACGTCACAACGT	67	103	0	0	0	0
MGAT3	TGACCAACGTCACAACCA	431	435	0	1	0	0
MGAT3	TGTGACACACTGACGTTG	324	327	0	0	0	0
MGAT4A	CAACCAACGTCAACACGT	65	84	0	0	0	0
MGAT4A	ACACCAACGTCAACACCA	122	139	0	1	0	0
MGAT4A	GTACCAACGTCAACACAC	25	61	0	0	0	0
MGAT4A	TGACCAACGTCAACACTG	107	109	0	0	0	0
MGAT4A	ACACCAACGTCAACTGGT	178	167	1	1	0	0
MGAT4B	GTACCAACGTCATGACGT	185	155	8	3	20	10
MGAT4B	GTACGTACACTGCATGTG	337	607	1	0	0	7
MGAT4B	CAACCAACGTCATGCATG	115	90	0	0	0	0
MGAT4B	CAACCAACGTCATGACCA	367	317	1	0	0	0
MGAT4B	TGACCAACGTCATGACAC	100	40	0	0	0	0
MGAT5	CAACCAACGTTGTGACTG	685	564	0	1	0	0
MGAT5	GTACCAACGTTGTGTGGT	355	520	0	0	0	0
MGAT5	CAACCAACGTTGTGTGCA	63	116	0	0	0	0
MGAT5	TGACCAACGTTGTGTGAC	91	159	0	0	0	0
MGAT5	ACACCAACGTTGTGTGTG	142	169	0	0	0	0
MGC5139	GTTGGTACTGTGTGGTAC	13	6	0	0	0	0
MGC5139	TGTGGTACTGTGTGGTTG	147	233	0	1	0	0
MGC5139	ACTGCAGTGTACCAGTCA	700	501	1	0	0	0
MGC5139	GTTGCAGTGTACCAGTAC	7	14	0	0	0	0
MGC5139	TGTGCAGTGTACCAGTTG	269	445	1	1	0	0
MGMT	CAACGTTGTGCATGGTAC	38	35	1	0	0	0
MGMT	GTACGTTGTGCATGGTTG	543	582	2	0	0	1
MGMT	TGACGTTGTGCATGCAGT	92	188	0	0	0	0
MGMT	GTACGTTGTGCATGCACA	87	124	0	0	0	0
MGMT	ACACGTTGTGCATGCAAC	31	42	0	0	0	0
MGP	TGACACTGGTCACAACCA	187	189	0	0	1	2
MGP	GTACACTGGTCACACAAC	23	22	0	0	0	0
MGP	TGACACTGGTCACACATG	142	523	0	0	0	0
MGP	ACACACTGGTCACAACGT	274	220	0	0	0	1
MGP	CAACACTGGTCACAACAC	74	77	0	1	0	36
MGST1	CATGGTTGGTCAACGTAC	40	27	0	0	0	0
MGST1	GTTGGTTGGTCAACGTTG	160	548	0	0	0	0
MGST1	TGTGGTTGGTCAACCAGT	68	94	1	1	0	0
MGST1	GTTGGTTGGTCAACCACA	130	67	2	0	0	0
MGST1	ACTGGTTGGTCAACCAAC	13	12	0	0	0	2
MGST1	ACTGCAACACACCAACTG	133	222	1	0	0	0
MIB1	CACATGTGCACAGTCACA	135	148	0	0	0	0
MIB1	TGCATGTGCACAGTCAAC	50	30	0	0	0	0
MIB1	ACCATGTGCACAGTCATG	217	185	1	0	0	1
MIB1	CACATGTGCACAGTACGT	130	91	0	0	0	0
MIB1	ACCATGTGCACAGTACCA	271	349	1	0	0	73
MIB2	TGACCACACAGTTGCAAC	25	17	0	0	0	0
MIB2	ACACCACACAGTTGCATG	50	50	0	0	0	0
MIB2	CAACCACACAGTTGACGT	260	318	0	0	0	1
MIB2	ACACCACACAGTTGACCA	189	127	0	1	0	0
MIB2	CATGACACGTACCAGTAC	40	143	0	1	0	0
MICA	GTACTGCAACTGGTCAAC	18	28	0	0	0	0
MICA	TGACTGCAACTGGTCATG	93	145	0	0	0	1
MICA	ACACTGCAACTGGTACGT	98	101	0	0	0	0
MICA	TGACTGCAACTGGTACCA	455	464	1	0	0	2
MICA	CAACTGCAACTGGTACAC	6	22	0	0	0	0
MICA	ACTGCACACAACCAACCA	538	447	3	0	0	1
MIF	GTACTGACTGCAACTGTG	374	364	0	0	1	0
MIF	ACACACTGACCAGTCACA	73	75	0	0	0	0
MIF	GTACACTGACCAGTCAAC	29	24	0	0	0	0
MIF	TGACACTGACCAGTCATG	118	63	0	0	0	0
MIF	ACACACTGACCAGTACGT	49	49	0	0	0	0
MIF	ACTGCACAGTTGTGGTCA	618	440	0	0	0	6
MINPP1	GTTGCAACCACAACTGGT	616	831	271986	286	13	31
MINPP1	ACACACACTGACGTCACA	91	78	0	0	1	0
MINPP1	CAACACACTGACGTCAGT	575	896	1	117	0	0
MINPP1	GTACACACTGACGTCAAC	11	12	0	0	0	6
MINPP1	TGACACACTGACGTCATG	236	329	0	0	0	0
MINPP1	ACACACACTGACGTACGT	134	111	0	0	0	0
MIOX	TGACACGTTGACGTACAC	41	56	0	0	0	0
MIOX	ACACACGTTGACGTACTG	549	481	0	0	0	0

MIOX	CAACACGTTGACGTTGGT	246	215	0	0	0	0
MIOX	ACACACGTTGACGTTGCA	181	622	1	1	0	5
MIOX	GTACACGTTGACGTTGAC	19	3	0	0	0	0
MITF	ACACGTACTGCATGACAC	158	248	0	0	0	0
MITF	CAACGTACTGCATGACTG	316	304	1	0	0	0
MITF	GTACGTACTGCATGTGGT	213	212	1	0	0	0
MITF	CAACGTACTGCATGTGCA	663	222	0	0	0	2
MITF	TGACGTACTGCATGTGAC	112	124	0	0	0	0
MKI67	GTTGGTGTTGTGTGGTCA	622	306	1	0	0	0
MKI67	ACTGGTGTTGTGTGGTAC	16	28	0	0	0	0
MKI67	CATGGTGTTGTGTGGTTG	386	863	0	3	0	0
MKI67	GTTGGTGTTGTGTGCAGT	90	37	0	0	0	0
MKI67	CATGGTGTTGTGTGCACA	30	29	0	0	0	0
MKKS	TGACTGACTGTGCACAAC	10	5	0	0	0	0
MKKS	ACACTGACTGTGCACATG	556	314	2	1	0	8
MKKS	CAACTGACTGTGCAACGT	499	376	0	0	0	0
MKKS	ACACTGACTGTGCAACCA	180	165	0	0	0	0
MKKS	ACTGCAGTTGTGACACCA	180	191	144	0	0	0
MKKS	CATGCAACTGACTGACGT	595	589	1	1	0	0
MKNK1	TGACGTGTGTGTACACTG	281	198	0	0	1	0
MKNK1	CATGGTACTGACTGCAGT	751	709	1	1	1	1
MKNK1	GTACGTGTGTGTACACAC	45	49	0	0	0	0
MKNK1	ACACGTGTGTGTACTGGT	535	187	0	0	0	0
MKNK1	ACTGGTACTGACTGCACA	241	304	0	2	0	1
MKNK2	ACCATGTGTGTGCAACTG	804	651	1	2	0	5
MKNK2	CACATGTGTGTGCATGGT	190	244	0	0	0	0
MKNK2	ACCATGTGTGTGCATGCA	183	192	0	0	0	0
MKNK2	GTCATGTGTGTGCATGAC	0	1	0	0	0	0
MKNK2	TGCATGTGTGTGCATGTG	99	118	0	0	0	0
MLC1	GTACTGCACAACCATGCA	410	319	0	1	1	0
MLC1	TGACTGCACAACCATGGT	1012	546	2	129	0	5
MLC1	ACACTGCACAACCATGAC	298	542	1	0	0	4
MLC1	CAACTGCACAACCATGTG	435	348	0	0	0	0
MLC1	TGACTGCACAACACGTGT	327	481	0	0	0	7
MLH1	TGACCATGCATGCAGTTG	2157	1353	2	3	1	0
MLH1	GTACCATGCATGCAGTAC	21	41	0	0	0	0
MLH1	ACACCATGCATGCACAGT	634	521	0	0	0	0
MLH1	TGACCATGCATGCACACA	180	191	0	0	0	1
MLH1	CAACCATGCATGCACAAC	16	78	0	0	0	0
MLH1	ACTGCATGGTCAACGTCA	236	507	0	0	0	0
MLH3	GTACCATGGTCATGCAGT	419	791	1	0	1	28
MLH3	GTACCATGGTCATGGTCA	40	29	0	0	0	0
MLH3	ACACCATGGTCATGGTAC	4	75	0	0	0	0
MLH3	CAACCATGGTCATGGTTG	342	207	0	0	0	0
MLH3	TGTGCAGTTGGTGTGTGT	466	439	1	1	0	0
MLL	ACCATGTGTGCAACCAAC	14	15	0	0	0	0
MLL	CACATGTGTGCAACCATG	171	145	0	0	0	0
MLL	GTCATGTGTGCAACACGT	58	106	0	0	0	0
MLL	CACATGTGTGCAACACCA	234	179	0	0	0	0
MLL	ACACCACACACACAGTTG	367	288	0	0	0	0
MLXIPL	ACACGTTGGTTGGTGTGT	295	282	15	9	1	14
MLXIPL	TGACGTTGGTTGGTGTCA	392	182	0	0	0	10
MLXIPL	CAACGTTGGTTGGTGTAC	82	81	0	0	0	0
MLXIPL	GTACGTTGGTTGGTGTTG	209	240	0	0	0	0
MLXIPL	TGTGACCAACGTACACAC	36	30	0	0	0	0
MLYCD	TGACTGTGACCAGTTGCA	322	314	1	0	0	0
MLYCD	CAACTGTGACCAGTTGAC	49	38	0	0	0	0
MLYCD	GTACTGTGACCAGTTGTG	462	258	0	0	0	0
MLYCD	ACACTGTGACCACAGTGT	254	225	0	0	0	2
MLYCD	TGACTGTGACCACAGTCA	151	129	1	0	0	1
MMAB	ACTGGTGTGTACGTTGGT	433	194	0	0	1	60
MMAB	GTTGGTGTGTACGTACAC	18	26	0	0	0	0
MMAB	TGTGGTGTGTACGTACTG	117	331	0	0	0	0
MMAB	TGTGGTGTGTACGTTGCA	7	16	0	0	0	0
MMAB	TGTGCACAGTGTGTTGTG	143	297	0	0	0	0
MME	ACACACCAGTGTCATGGT	214	348	0	0	0	3
MME	TGACACCAGTGTCATGCA	116	139	0	0	0	0
MME	CAACACCAGTGTCATGAC	241	69	0	0	0	0
MME	GTACACCAGTGTCATGTG	2231	885	1	1	0	18
MME	ACACACCAGTGTACGTGT	723	62	0	0	0	0
MME	GTTGCACAACCATGTGGT	114	142	0	0	0	1
MMP10	TGACACCATGCAACCAAC	5	14	0	0	0	0
MMP10	ACACACCATGCAACCATG	209	207	0	0	0	0
MMP10	CAACACCATGCAACACGT	80	115	0	0	0	0
MMP10	ACACACCATGCAACACCA	152	169	0	0	0	0

MMP10	GTACACCATGCAACACAC	37	71	0	0	0	0
MMP10	ACTGCACAGTTGTGCAGT	10	12	0	0	0	0
MMP13	ACACACACACGTCAGTAC	28	22	0	0	0	0
MMP13	CAACACACACGTCAGTTG	416	479	0	1	0	1
MMP13	GTACACACACGTCACAGT	352	428	0	0	0	0
MMP13	CAACACACACGTCACACA	121	124	0	0	0	0
MMP13	TGACACACACGTCACAAC	1	5	0	0	0	0
MMP13	ACTGCACATGACTGACTG	182	179	0	0	0	0
MMP14	CAACACACGTTGGTACGT	316	460	1	0	0	0
MMP14	ACACACACGTTGGTACCA	284	148	1	0	0	0
MMP14	GTACACACGTTGGTACAC	9	3	1	0	0	0
MMP14	TGACACACGTTGGTACTG	410	336	0	0	0	0
MMP14	CATGCACATGACTGTGGT	633	676	2	1	0	2
MMP14	ACTGCACATGACTGTGCA	59	152	1	0	0	0
MMP2	GTACACACCATGACTGAC	380	98	0	0	1	0
MMP2	ACACACACCATGACTGCA	37	34	1	0	0	0
MMP2	TGACACACCATGACTGTG	153	164	0	0	0	0
MMP2	GTTGCAGTCAACTGACTG	147	175	0	0	0	1
MMP2	TGTGCAGTCAACTGTGGT	991	1023	2	1	0	0
MMP3	ACCATGACACTGCATGCA	130	264	0	0	0	0
MMP3	GTCATGACACTGCATGAC	37	32	0	0	0	2
MMP3	TGCATGACACTGCATGTG	223	201	0	0	0	0
MMP3	CACATGACACTGACGTGT	1054	553	18	0	0	5
MMP3	ACTGCAGTACGTACTGCA	102	73	0	0	0	0
MMP7	CAACACACACCACATGGT	567	666	1	0	1	0
MMP7	GTACACACACCACATGAC	246	181	0	0	1	2
MMP7	ACACACACACCACAACTG	195	362	0	1	0	0
MMP7	ACACACACACCACATGCA	679	280	0	0	0	0
MMP7	TGACACACACCACATGTG	396	628	1	1	0	0
MMP8	GTACACACACACCAACTG	201	209	0	0	0	0
MMP8	TGACACACACACCATGGT	376	518	0	0	0	12
MMP8	GTACACACACACCATGCA	238	225	0	0	0	0
MMP8	ACACACACACACCATGAC	52	85	1	0	0	0
MMP8	CAACACACACACCATGTG	815	278	0	1	0	0
MMP8	TGTGCACAGTTGTGCACA	5	2	0	0	0	0
MMP9	GTACACACCATGGTACTG	238	373	1	0	0	0
MMP9	TGACACACCATGGTTGGT	705	347	0	0	0	0
MMP9	GTACACACCATGGTTGCA	92	148	1	1	0	0
MMP9	ACACACACCATGGTTGAC	42	67	0	0	0	0
MMP9	ACTGACCACATGCAGTAC	26	41	0	0	0	0
MMRN1	GTACACTGGTTGCACAGT	389	214	7	4	12	21232
MMRN1	CAACACTGGTTGCACACA	98	90	0	0	0	0
MMRN1	TGACACTGGTTGCACAAC	25	30	0	0	0	0
MMRN1	ACACACTGGTTGCACATG	168	149	0	0	0	0
MMRN1	CAACACTGGTTGCAACGT	119	97	0	0	0	0
MMS19L	ACTGGTGTCATGTGTGCA	169	582	0	0	1	1
MMS19L	GTTGGTGTCATGTGTGAC	148	58	0	0	0	0
MMS19L	TGTGGTGTCATGTGTGTG	227	175	0	0	0	0
MMS19L	CATGGTGTACGTGTGTGT	142	239	0	0	0	0
MMS19L	ACTGCATGCAGTGTACAC	40	58	0	0	0	0
MMS19L	CATGCATGCAGTGTACTG	292	443	1	1	0	2
MNAT1	ACACGTTGGTACCACACA	25	38	0	0	0	0
MNAT1	GTACGTTGGTACCACAAC	23	26	0	0	0	0
MNAT1	TGACGTTGGTACCACATG	111	80	0	0	0	0
MNAT1	ACACGTTGGTACCAACGT	528	340	1	0	0	0
MNAT1	TGACGTTGGTACCAACCA	13	20	0	0	0	0
MOBP	ACACTGACCAACGTTGCA	296	257	1	0	1	6
MOBP	GTACTGACCAACGTTGAC	223	165	0	0	0	0
MOBP	TGACTGACCAACGTTGTG	57	74	0	0	0	0
MOBP	CAACTGACCAACCAGTGT	570	659	0	1	0	0
MOBP	GTTGACACGTGTGTACGT	346	268	0	1	0	0
MOG	CATGGTACGTGTCATGAC	22	19	0	0	0	0
MOG	GTTGGTACGTGTCATGTG	249	129	0	0	0	0
MOG	ACTGGTACGTGTACGTGT	384	631	0	1	0	1
MOG	TGTGGTACGTGTACGTCA	83	115	0	0	0	0
MOG	TGTGACGTTGCAACACTG	729	263	0	0	0	0
MPG	CAACACACCACATGTGGT	1348	1023	0	0	1	0
MPG	ACACACACCACATGACTG	525	735	1	0	0	0
MPG	ACACACACCACATGTGCA	224	253	0	0	0	0
MPG	GTACACACCACATGTGAC	132	70	0	0	0	0
MPG	TGACACACCACATGTGTG	562	452	0	0	0	0
MPI	CAACACCAACACTGGTGT	764	321	3	0	1	0
MPI	CAACACCAACACACTGGT	366	337	4	0	0	0
MPI	ACACACCAACACACTGCA	329	301	7	1	0	0
MPI	GTACACCAACACACTGAC	62	89	1	0	0	0

MPI	TGACACCAACACACTGTG	703	542	0	1	0	5
MPI	CATGCACAGTTGTGCAAC	5	3	0	0	0	0
MPL	ACACTGCAGTACGTCACA	170	174	0	0	0	0
MPL	GTACTGCAGTACGTCAAC	3	16	0	0	0	0
MPL	TGACTGCAGTACGTCATG	191	22	0	0	0	0
MPL	ACACTGCAGTACGTACGT	30	33	0	0	0	0
MPL	TGACTGCAGTACGTACCA	80	121	0	0	0	0
MPL	TGTGCACACATGTGACAC	29	140	0	0	0	0
MPO	ACACACGTACTGACGTGT	659	192	2	2	0	0
MPO	TGACACGTACTGACGTCA	41	60	0	0	0	0
MPO	CAACACGTACTGACGTAC	23	34	0	0	0	0
MPO	GTACACGTACTGACGTTG	666	514	1	1	0	0
MPO	TGACACGTACTGACCAGT	506	214	2	0	0	0
MPO	GTTGCACACAACCAACAC	8	19	0	0	0	0
MPPED2	GTACACACGTACGTACGT	174	82	0	0	0	0
MPPED2	CAACACACGTACGTACCA	211	305	0	0	0	0
MPPED2	TGACACACGTACGTACAC	35	17	0	0	0	0
MPPED2	ACACACACGTACGTACTG	1198	670	3	0	0	0
MPPED2	CAACACACGTACGTTGGT	338	641	0	1	0	0
MPPED2	CATGCACAGTCATGGTAC	90	42	0	0	0	0
MPST	GTACCAACCAGTTGCAAC	23	23	0	0	0	0
MPST	TGACCAACCAGTTGCATG	113	140	0	0	0	1
MPST	ACACCAACCAGTTGACGT	317	329	0	0	0	1
MPST	TGACCAACCAGTTGACCA	359	445	0	0	0	1
MPST	CAACCAACCAGTTGACAC	41	44	0	0	0	0
MR1	ACACACTGTGACGTGTCA	644	455	0	1	0	0
MR1	GTACACTGTGACGTGTAC	13	3	0	0	0	0
MR1	TGACACTGTGACGTGTTG	851	1260	1	0	0	1
MR1	ACACACTGTGACGTCAGT	122	309	0	0	0	0
MR1	TGACACTGTGACGTCACA	100	162	0	0	0	0
MR1	TGTGCACAGTTGGTCATG	158	127	0	0	0	1
MRE11A	CAACCATGCACATGGTCA	552	383	0	1	0	0
MRE11A	TGACCATGCACATGGTAC	7	37	0	0	0	0
MRE11A	ACACCATGCACATGGTTG	300	316	0	1	0	0
MRE11A	CAACCATGCACATGCAGT	662	204	1	0	0	4
MRE11A	ACACCATGCACATGCACA	13	29	0	0	0	0
MRPL19	GTACTGCATGCAACCACA	255	324	0	0	0	0
MRPL19	ACACTGCATGCAACCAAC	34	31	0	0	0	0
MRPL19	CAACTGCATGCAACCATG	197	142	0	0	0	0
MRPL19	TGTGCAGTTGTGGTACGT	539	495	1	0	0	0
MRPL19	GTTGCAGTTGTGGTACCA	73	136	0	0	0	0
MS4A1	GTACTGCATGGTGTACCA	163	537	0	1	2	0
MS4A1	GTACTGCATGGTGTCATG	74	113	0	0	0	0
MS4A1	TGACTGCATGGTGTACGT	296	459	0	1	0	0
MS4A1	ACACTGCATGGTGTACAC	85	63	0	0	0	0
MS4A1	GTTGCAGTTGTGGTTGGT	331	368	2	0	0	0
MSH2	TGACCATGGTTGTGCAGT	502	607	1	0	0	0
MSH2	GTACCATGGTTGTGCACA	167	111	0	0	0	0
MSH2	ACACCATGGTTGTGCAAC	10	17	0	0	0	0
MSH2	CAACCATGGTTGTGCATG	200	277	0	0	0	1
MSH2	GTACCATGGTTGTGACGT	869	481	0	1	0	2
MSH2	TGTGCACACAACCAACTG	311	336	2	1	0	0
MSH3	CATGGTGTGTACTGGTCA	260	222	1	0	0	5
MSH3	TGTGGTGTGTACTGGTAC	29	39	0	0	0	0
MSH3	ACTGGTGTGTACTGGTTG	668	905	1	0	0	0
MSH3	CATGGTGTGTACTGCAGT	280	327	0	0	0	0
MSH3	ACTGGTGTGTACTGCACA	19	20	0	0	0	0
MSH4	CATGGTACCAACTGCACA	311	342	0	15	1	3138
MSH4	ACTGGTACCAACTGGTAC	188	138	1	0	0	0
MSH4	CATGGTACCAACTGGTTG	618	522	0	0	0	0
MSH4	GTTGGTACCAACTGCAGT	114	36	0	0	0	1
MSH4	TGTGGTACCAACTGCAAC	55	18	0	0	0	0
MSH5	CAACCATGCAACACGTTG	778	1069	0	0	1	0
MSH5	ACACCATGCAACACGTAC	48	33	0	0	0	0
MSH5	GTACCATGCAACACCAGT	429	574	0	0	0	3
MSH5	CAACCATGCAACACCACA	52	34	0	0	0	0
MSH5	ACTGGTTGTGACGTCACA	57	125	0	0	0	0
MSH6	ACACTGACTGACGTCATG	46	56	0	0	0	0
MSH6	TGACTGTGACCATGACCA	332	324	0	0	0	0
MSH6	CAACTGTGACCATGACAC	181	178	1	2	0	1
MSH6	GTACTGTGACCATGACTG	99	126	0	0	0	0
MSH6	TGACTGTGACCATGTGGT	676	507	0	0	0	0
MSH6	CATGCACAGTACTGTGTG	224	211	0	0	0	0
MSI1	ACACTGACCAACCAGTCA	1159	1303	3	2	1	0
MSI1	ACTGCAGTTGTGCAGTTG	563	402	1	0	1	8

MSI1	GTACTGACCAACCAGTAC	53	66	0	1	0	0
MSI1	TGACTGACCAACCAGTTG	1020	1120	6	1	0	1
MSI1	TGTGCAGTCAGTCAGTTG	888	1645	3	3	0	1
MSI1	TGTGCAACTGACACGTAC	71	54	0	0	0	1
MSLN	ACTGGTTGTGTGGTACAC	14	26	0	0	0	0
MSLN	CATGGTTGTGTGGTACTG	126	109	0	0	0	0
MSLN	GTTGGTTGTGTGGTTGGT	347	142	0	0	0	0
MSLN	CATGGTTGTGTGGTTGCA	126	100	1	0	0	0
MSLN	TGTGGTTGTGTGGTTGAC	24	20	0	0	0	0
MSR1	GTACCAGTTGACGTGTCA	645	686	1	1	0	15
MSR1	ACACCAGTTGACGTGTAC	27	19	0	0	0	0
MSR1	CAACCAGTTGACGTGTTG	597	511	0	0	0	0
MSR1	GTACCAGTTGACGTCAGT	154	114	0	0	0	0
MSR1	CAACCAGTTGACGTCACA	45	27	0	0	0	0
MST1	ACACACTGGTACGTACCA	377	299	0	0	1	0
MST1	GTTGCATGCAGTGTTGGT	514	182	0	1	1	0
MST1	CAACACTGGTACGTCACA	87	72	0	0	0	0
MST1	TGACACTGGTACGTCAAC	23	22	0	0	0	0
MST1	ACACACTGGTACGTCATG	248	492	1	0	0	2
MST1	CAACACTGGTACGTACGT	375	325	2	0	0	0
MST1R	TGCATGACACCAACGTCA	113	110	0	0	0	2
MST1R	CACATGACACCAACGTAC	75	54	0	0	0	0
MST1R	GTCATGACACCAACGTTG	515	374	2	1	0	0
MST1R	TGCATGACACCAACCAGT	110	139	0	0	0	0
MST1R	ACTGGTCACAGTGTTGAC	45	56	1	0	0	0
MSTN	ACACCAGTTGTGGTCACA	152	149	0	0	0	0
MSTN	GTACCAGTTGTGGTCAAC	0	5	0	0	0	0
MSTN	ACACTGCAGTCAACGTTG	272	286	0	0	0	0
MSTN	CAACTGCAGTCAACCAGT	297	406	0	1	0	9
MSTN	ACACTGCAGTCAACCACA	136	77	0	0	0	0
MSX1	CAACGTTGCAGTTGTGGT	363	403	1	492	9	1
MSX1	ACACGTTGCAGTTGACTG	546	1004	1	0	1	8
MSX1	TGACGTTGCAGTTGACAC	68	32	0	0	0	0
MSX1	ACACGTTGCAGTTGTGCA	17	29	0	0	0	0
MSX1	GTACGTTGCAGTTGTGAC	43	90	0	0	0	0
MT2A	ACTGGTACGTCAGTGTAC	58	167	0	0	0	0
MT2A	CATGGTACGTCAGTGTTG	395	656	1	1	0	84
MT2A	GTTGGTACGTCAGTCAGT	171	347	0	0	0	0
MT2A	CATGGTACGTCAGTCACA	181	114	0	0	0	1
MT2A	GTTGGTACACACGTTGCA	222	109	0	0	0	0
MT2A	CATGCATGCAGTGTTGCA	67	79	1	0	0	1427
MT3	GTACTGACTGTGCAGTCA	432	438	2	0	0	0
MT3	ACACTGACTGTGCAGTAC	94	76	0	0	0	0
MT3	CAACTGACTGTGCAGTTG	453	396	1	0	0	0
MT3	GTACTGACTGTGCACAGT	187	60	0	0	0	0
MT3	CAACTGACTGTGCACACA	174	166	0	0	0	0
MTA1	GTACGTCAACTGGTACAC	55	62	0	0	0	2
MTA1	TGACGTCAACTGGTACTG	93	79	0	0	0	0
MTA1	ACACGTCAACTGGTTGGT	281	265	0	0	0	0
MTA1	TGACGTCAACTGGTTGCA	58	66	0	0	0	0
MTA1	CATGGTTGTGGTCAACCA	230	265	0	0	0	0
MTA2	CAACGTCAACTGGTTGAC	18	23	0	0	0	0
MTA2	GTACGTCAACTGGTTGTG	416	1230	2	1	0	0
MTA2	ACACGTCAACTGCAGTGT	496	145	1	0	0	0
MTA2	TGACGTCAACTGCAGTCA	208	137	1	1	0	0
MTA2	CAACGTCAACTGCAGTAC	130	103	0	0	0	4
MTA2	ACTGCAACGTGTACGTCA	95	55	0	0	0	0
MTA3	TGTGGTTGACTGTGACTG	542	742	0	0	1	0
MTA3	GTACGTACCATGTGGTCA	144	213	0	0	0	0
MTA3	ACACGTACCATGTGGTAC	16	31	0	0	0	0
MTA3	CAACGTACCATGTGGTTG	659	682	0	0	0	0
MTA3	ACACTGTGACACACCATG	233	343	0	0	0	0
MTAP	CACATGTGTGGTTGCATG	235	95	0	0	1	2
MTAP	GTCATGTGTGGTTGACGT	174	303	0	0	0	0
MTAP	CACATGTGTGGTTGACCA	51	230	0	0	0	0
MTAP	TGCATGTGTGGTTGACAC	33	9	0	0	0	0
MTAP	ACCATGTGTGGTTGACTG	67	134	0	0	0	0
MTFMT	TGACCACAACACACGTGT	320	223	1	1	0	9
MTFMT	GTACCACAACACACGTCA	217	105	0	1	0	0
MTFMT	ACACCACAACACACGTAC	45	136	0	0	0	0
MTFMT	CAACCACAACACACGTTG	345	291	0	0	0	0
MTFMT	GTACCACAACACACCAGT	122	129	0	0	0	0
MTHFD1	ACACCAACACGTCAACGT	80	140	0	0	0	0
MTHFD1	TGACCAACACGTCAACCA	120	135	0	0	0	4
MTHFD1	CAACCAACACGTCAACAC	24	55	0	0	0	0

MTHFD1	GTACCAACACGTCAACTG	183	231	1	0	0	1
MTHFD1	TGACCAACACGTCATGGT	466	443	0	0	0	1
MTHFD1	ACTGCAACGTGTGTCATG	486	282	1	1	0	0
MTHFD1L	TGACACGTCACACAGTCA	606	490	0	0	1	0
MTHFD1L	ACACCAACACGTTGACTG	509	619	0	0	0	0
MTHFD1L	ACACACGTCACACAGTGT	121	111	0	0	0	0
MTHFD1L	CAACACGTCACACAGTAC	215	178	0	0	0	0
MTHFD1L	GTACACGTCACACAGTTG	41	64	0	0	0	0
MTHFD2	TGACCAACACGTACACTG	132	133	0	0	0	0
MTHFD2	ACACCAACACGTACTGGT	272	275	1	0	0	0
MTHFD2	TGACCAACACGTACTGCA	81	85	1	0	0	0
MTHFD2	CAACCAACACGTACTGAC	14	12	0	0	0	0
MTHFD2	CATGCAGTCATGTGCAAC	11	12	0	0	0	0
MTHFR	GTACACGTTGACACTGCA	271	112	0	0	1	8
MTHFR	GTACACGTTGACACACTG	269	229	0	1	0	0
MTHFR	TGACACGTTGACACTGGT	693	388	0	0	0	7
MTHFR	ACACACGTTGACACTGAC	18	15	0	0	0	0
MTHFR	CAACACGTTGACACTGTG	425	304	1	0	0	0
MTHFS	CAACACGTCAGTTGCAAC	32	12	0	0	0	0
MTHFS	GTACACGTCAGTTGCATG	149	181	0	0	0	0
MTHFS	TGACACGTCAGTTGACGT	58	99	0	0	0	0
MTHFS	GTACACGTCAGTTGACCA	250	229	1	0	0	0
MTHFS	TGTGCAGTCACACAGTAC	38	59	1	0	0	0
MTHFS	GTTGCACAACGTTGCACA	101	141	0	0	0	0
MTIF2	TGTGGTACTGGTACACCA	382	283	0	0	1	0
MTIF2	ACTGGTACTGGTACCACA	98	98	0	0	0	0
MTIF2	GTTGGTACTGGTACCAAC	7	4	0	0	0	0
MTIF2	TGTGGTACTGGTACCATG	93	131	0	1	0	0
MTIF2	ACTGGTACTGGTACACGT	194	179	0	0	0	0
MTMR1	ACCATGACACGTTGACTG	288	364	1	0	1	0
MTMR1	CACATGACACGTTGACCA	198	130	0	0	0	0
MTMR1	TGCATGACACGTTGACAC	33	71	0	0	0	0
MTMR1	CACATGACACGTTGTGGT	46	65	0	0	0	0
MTMR1	ACCATGACACGTTGTGCA	121	156	0	1	0	1
MTMR2	GTCATGACACGTTGTGAC	45	48	0	0	1	0
MTMR2	TGCATGACACGTTGTGTG	124	275	0	0	0	0
MTMR2	GTCATGACACCAGTGTGT	1422	1338	2	3	0	2
MTMR2	CACATGACACCAGTGTCA	624	394	1	0	0	2
MTMR2	TGCATGACACCAGTGTAC	65	66	0	0	0	0
MTMR6	TGACGTGTCACAACACTG	531	566	2	3	4	17
MTMR6	ACACGTGTCACAACACCA	57	22	0	0	0	0
MTMR6	GTACGTGTCACAACACAC	12	22	0	0	0	0
MTMR6	ACACGTGTCACAACTGGT	1075	674	2	2	0	0
MTMR6	TGTGCAGTACCACAACAC	47	39	0	0	0	0
MTNR1A	ACACGTCAACGTGTACAC	9	11	0	0	0	0
MTNR1A	CAACGTCAACGTGTACTG	251	314	1	0	0	2
MTNR1A	GTACGTCAACGTGTTGGT	433	411	0	0	0	0
MTNR1A	ACTGCAGTACACGTCAGT	301	210	0	0	0	0
MTNR1A	ACTGACGTTGTGCACATG	582	533	2	0	0	3
MTNR1B	CAACGTCAACGTGTTGCA	125	144	0	0	0	0
MTNR1B	TGACGTCAACGTGTTGAC	53	31	0	0	0	0
MTNR1B	ACACGTCAACGTGTTGTG	174	133	1	0	0	0
MTNR1B	GTACGTCAACGTCAGTGT	707	674	1	0	0	0
MTNR1B	CAACGTCAACGTCAGTCA	177	186	2	0	0	0
MTR	GTACCACATGCACAACCA	795	466	0	1	1	1
MTR	ACACCACATGCACAACAC	85	3	0	0	0	0
MTR	CAACCACATGCACAACTG	1442	902	1	3	0	1
MTR	GTACCACATGCACATGGT	803	260	0	2	0	0
MTR	CAACCACATGCACATGCA	326	132	0	0	0	0
MTR	ACTGCACACAACCATGGT	223	452	0	0	0	4
MTRR	ACACCACATGCATGGTAC	186	171	0	0	1	0
MTRR	CAACCACATGCATGGTTG	686	627	1	0	1	3
MTRR	GTACCACATGCATGGTCA	397	438	1	0	0	3
MTRR	GTACCACATGCATGCAGT	392	350	0	1	0	0
MTRR	CAACCACATGCATGCACA	492	420	0	0	0	0
MTRR	GTTGCACAGTTGTGCATG	204	119	0	0	0	0
MTTP	GTACTGTGTGCATGGTCA	82	55	0	0	0	0
MTTP	ACACTGTGTGCATGGTAC	39	71	0	0	0	0
MTTP	CAACTGTGTGCATGGTTG	73	97	0	0	0	0
MTTP	GTACTGTGTGCATGCAGT	172	80	0	0	0	0
MTTP	CAACTGTGTGCATGCACA	65	101	0	0	0	0
MUC1	CATGACACGTACGTTGAC	134	74	2	0	2	1
MUC1	GTTGGTCACAGTCATGTG	489	663	0	0	1	0
MUC1	ACTGGTCACAGTACGTGT	22	23	0	0	0	0
MUC1	GTTGACACGTACGTTGTG	179	153	1	0	0	1

MUC1	ACTGACACGTACCAGTGT	99	118	0	0	0	0
MUC5AC	GTTGACACGTACTGACTG	189	332	0	0	0	0
MUC5AC	TGTGACACGTACTGTGGT	197	142	0	1	0	0
MUC5AC	GTTGACACGTACTGTGCA	94	85	0	0	0	0
MUC5AC	ACTGACACGTACTGTGAC	23	28	0	0	0	0
MUC5AC	CATGACACGTACTGTGTG	255	294	156	0	0	0
MUS81	ACACACCATGCAGTCAGT	72	58	0	0	0	0
MUS81	TGACACCATGCAGTCACA	113	55	0	0	0	5
MUS81	CAACACCATGCAGTCAAC	25	128	0	0	0	0
MUS81	GTACACCATGCAGTCATG	395	439	3	1	0	3
MUS81	TGACACCATGCAGTACGT	543	864	2	2	0	0
MUSK	ACCATGACCAGTCAACTG	430	505	0	16	1	3
MUSK	CACATGACCAGTCAACCA	321	203	1	0	0	3
MUSK	TGCATGACCAGTCAACAC	41	27	0	0	0	0
MUSK	CACATGACCAGTCATGGT	638	1179	198	2	0	1
MUSK	CATGCAGTACGTACGTAC	35	62	0	0	0	0
MUSK	CATGCACAACGTCAGTCA	157	228	0	0	0	0
MUT	TGACACCAACCATGCAAC	16	13	0	0	0	0
MUT	ACACACCAACCATGCATG	218	179	2	0	0	0
MUT	CAACACCAACCATGACGT	668	633	1	1	0	0
MUT	ACACACCAACCATGACCA	385	267	1	0	0	0
MUT	TGTGGTACTGCACAGTAC	24	27	0	0	0	0
MUT	TGTGCACACAACCATGCA	370	559	1	1	0	0
MUTYH	CAACACTGCATGCAACAC	33	29	0	0	0	0
MUTYH	GTACACTGCATGCAACTG	806	1114	1	0	0	6
MUTYH	TGACACTGCATGCATGGT	866	1214	1	0	0	0
MUTYH	ACTGCAGTTGCACAACAC	122	102	0	0	0	0
MUTYH	TGTGACACGTTGTGTGAC	111	50	2	0	0	1
MVD	ACACTGTGACCAGTACCA	545	331	0	0	0	0
MVD	GTACTGTGACCAGTACAC	32	31	0	0	0	0
MVD	TGACTGTGACCAGTACTG	189	554	2	0	0	0
MVD	ACACTGTGACCAGTTGGT	64	78	0	0	0	0
MVD	CATGGTTGTGGTTGCAGT	40	0	0	0	0	0
MVD	GTTGCACAGTTGTGACCA	584	362	1	0	0	0
MVK	ACTGGTTGACTGGTGTGT	338	387	1	0	2	456
MVK	ACTGGTTGACACTGTGCA	145	214	0	0	0	0
MVK	GTTGGTTGACACTGTGAC	41	60	0	0	0	0
MVK	TGTGGTTGACACTGTGTG	449	603	1	0	0	0
MVK	GTTGCACAGTCAGTACCA	474	330	1	3	0	2
MVK	CATGCACACAACCATGAC	438	187	1	75	0	0
MVP	ACTGGTTGCATGCATGGT	970	611	3	3	1	2
MVP	CATGGTTGCATGCAACGT	174	248	0	0	0	0
MVP	ACTGGTTGCATGCAACCA	28	64	0	0	0	0
MVP	GTTGGTTGCATGCAACAC	17	21	0	0	0	0
MVP	TGTGGTTGCATGCAACTG	331	256	0	0	0	2
MVP	ACTGCAACCACAGTCATG	55	332	0	0	0	1
MX1	ACACACTGACCAACGTGT	344	387	0	0	0	5
MX1	TGACACTGACCAACGTCA	238	253	0	0	0	0
MX1	CAACACTGACCAACGTAC	32	31	0	0	0	0
MX1	GTACACTGACCAACGTTG	122	128	0	0	0	0
MX1	TGACACTGACCAACCAGT	220	255	0	29	0	0
MX1	CATGCAACCAGTGTACAC	60	71	0	1	0	0
MXI1	CAACGTTGCACACACACA	33	68	0	0	0	0
MXI1	TGACGTTGCACACACAAC	16	28	0	0	0	0
MXI1	ACACGTTGCACACACATG	259	179	1	0	0	0
MXI1	CAACGTTGCACACAACGT	664	355	0	1	0	0
MXI1	ACACGTTGCACACAACCA	555	296	45	20	0	0
MYB	GTACCATGCATGCACATG	166	111	1	0	0	0
MYB	TGACCATGCATGCAACGT	136	110	0	0	0	0
MYB	GTACCATGCATGCAACCA	222	113	0	0	0	6
MYB	ACACCATGCATGCAACAC	19	49	0	0	0	0
MYB	ACTGCAGTCACATGCAAC	14	8	0	0	0	0
MYBPC1	CATGGTACGTGTACCATG	56	78	0	0	0	0
MYBPC1	GTTGGTACGTGTACACGT	13	22	0	0	0	0
MYBPC1	CATGGTACGTGTACACCA	260	264	0	0	0	1
MYBPC1	TGTGGTACGTGTACACAC	26	18	0	0	0	0
MYBPC1	ACTGGTACGTGTACACTG	156	252	0	0	0	0
MYBPC2	ACTGGTTGCATGACGTGT	433	379	0	0	1	0
MYBPC2	TGTGGTTGCATGCATGCA	249	195	0	0	0	0
MYBPC2	CATGGTTGCATGCATGAC	19	18	0	0	0	0
MYBPC2	GTTGGTTGCATGCATGTG	157	569	370	0	0	0
MYBPC2	TGTGGTTGCATGACGTCA	82	86	0	0	0	0
MYBPC3	TGACTGTGACTGTGCAAC	53	69	0	0	0	0
MYBPC3	ACACTGTGACTGTGCATG	68	92	0	0	0	1
MYBPC3	CAACTGTGACTGTGACGT	102	19	0	0	0	0

MYBPC3	ACACTGTGACTGTGACCA	361	201	0	1	0	0
MYBPC3	ACTGCAGTTGTGTGACTG	385	650	0	0	0	1
MYBPC3	ACTGCACATGTGCAACAC	48	81	0	0	0	0
MYC	CAACCATGGTTGACCACA	250	83	0	1	0	1
MYC	TGACCATGGTTGACCAAC	48	52	1	0	0	0
MYC	ACACCATGGTTGACCATG	138	113	0	0	0	0
MYC	CAACCATGGTTGACACGT	65	71	0	0	0	0
MYC	ACACCATGGTTGACACCA	223	174	0	1	0	0
MYCBP	GTACGTTGACACGTTGCA	71	305	0	0	0	0
MYCBP	ACACGTTGACACGTTGAC	26	28	0	0	0	0
MYCBP	CAACGTTGACACGTTGTG	222	202	0	0	0	0
MYCBP	TGACGTTGACACCAGTGT	654	436	2	0	0	0
MYCBP	GTACGTTGACACCAGTCA	115	92	1	0	0	0
MYCN	CAACGTTGCAACCAACTG	370	544	162	0	1	16
MYCN	GTACGTTGCAACCATGGT	261	887	1	0	0	0
MYCN	CAACGTTGCAACCATGCA	250	271	0	0	0	0
MYCN	TGACGTTGCAACCATGAC	7	3	0	0	0	0
MYCN	ACACGTTGCAACCATGTG	310	412	0	0	0	0
MYD88	ACACGTGTTGGTACCAGT	180	259	0	0	1	0
MYD88	TGACGTGTTGGTACGTTG	353	336	1	0	0	0
MYD88	TGACGTGTTGGTACCACA	167	356	0	0	0	0
MYD88	CAACGTGTTGGTACCAAC	31	41	0	0	0	0
MYD88	TGTGCAGTACCATGGTAC	145	121	0	1	0	0
MYEF2	ACACGTTGACACTGGTCA	242	232	1	0	0	0
MYEF2	GTACGTTGACACTGGTAC	245	106	0	0	0	0
MYEF2	TGACGTTGACACTGGTTG	382	404	0	0	0	0
MYEF2	ACACGTTGACACTGCAGT	595	518	2	0	0	0
MYEF2	TGACGTTGACACTGCACA	47	66	1	0	0	0
MYF5	TGACGTTGCAACACTGGT	214	297	0	0	1	0
MYF5	TGACGTTGCAACACACCA	170	170	0	0	0	1
MYF5	CAACGTTGCAACACACAC	24	9	0	0	0	0
MYF5	GTACGTTGCAACACACTG	263	272	0	0	0	0
MYF5	GTACGTTGCAACACTGCA	85	68	0	0	0	0
MYF5	TGTGCACAACGTCAGTAC	3	3	1	0	0	0
MYF6	GTACGTTGCAACTGTGTG	655	600	1	0	0	0
MYF6	TGACGTTGCATGGTGTGT	1363	1053	0	0	0	25
MYF6	GTACGTTGCATGGTGTCA	555	60	0	0	0	0
MYF6	ACACGTTGCATGGTGTAC	91	102	0	0	0	0
MYF6	CAACGTTGCATGGTGTTG	471	558	0	0	0	0
MYF6	ACTGCACAGTTGTGACAC	25	31	0	0	0	0
MYH2	TGTGGTGTGTGTGTACAC	193	100	0	0	0	0
MYH2	ACTGGTGTGTGTGTACTG	342	493	3	1	0	0
MYH2	CATGGTGTGTGTGTTGGT	795	356	1	0	0	0
MYH2	ACTGGTGTGTGTGTTGCA	54	67	1	0	0	1
MYH2	GTTGGTGTGTGTGTTGAC	15	4	0	0	0	0
MYH3	GTTGGTTGGTTGCATGAC	84	48	0	0	1	0
MYH3	TGTGGTTGGTTGCATGTG	1539	544	1	0	1	0
MYH3	ACTGGTTGGTTGCATGCA	143	140	0	1	0	0
MYH3	CATGGTTGGTTGACGTGT	1429	462	1	1	0	0
MYH3	ACTGGTTGGTTGACGTCA	186	237	1	0	0	0
MYH6	CAACCAGTTGTGTGGTGT	313	372	0	0	0	0
MYH6	ACACCAGTTGTGTGGTCA	322	175	0	1	0	0
MYH6	GTACCAGTTGTGTGGTAC	7	35	0	0	0	0
MYH6	TGACCAGTTGTGTGGTTG	743	1128	1	2	0	0
MYH6	TGACTGTGTGTGTGGTCA	32	43	0	0	0	0
MYH6	ACTGCATGGTGTCAGTAC	61	55	0	0	0	0
MYH8	CATGGTACGTCACAGTAC	51	59	6	2	1	1
MYH8	ACTGGTACGTCACAGTGT	482	356	2	0	0	8
MYH8	TGTGGTACGTCACAGTCA	33	55	0	0	0	0
MYH8	GTTGGTACGTCACAGTTG	587	723	0	1	0	0
MYH8	GTTGACACTGCAGTACAC	26	29	0	0	0	0
MYL1	ACACACTGGTACCAGTGT	197	259	1	0	1	0
MYL1	CAACACTGGTACGTTGAC	70	76	1	0	0	0
MYL1	GTACACTGGTACGTTGTG	435	576	0	0	0	9
MYL1	GTTGCAACGTTGACGTCA	53	130	0	0	0	0
MYL1	ACTGCAACGTTGACGTAC	49	90	0	0	0	0
MYL1	CATGCAACGTTGACGTTG	135	119	0	0	0	0
MYL2	GTACACTGGTACACGTAC	42	46	0	0	0	0
MYL2	TGACACTGGTACACGTTG	138	135	1	0	0	0
MYL2	ACACACTGGTACACCAGT	188	786	2	0	0	1
MYL2	TGACACTGGTACACCACA	519	293	1	0	0	1
MYL2	CAACACTGGTACACCAAC	78	70	3	2527	0	1
MYL2	GTTGCACACAACCATGTG	1034	1122	1	0	0	1
MYL3	TGACACTGGTACTGCATG	526	706	49	113	10	85
MYL3	ACACACTGGTACTGACGT	195	148	0	1	0	0

MYL3	TGACACTGGTACTGACCA	447	430	1	1	0	0
MYL3	CAACACTGGTACTGACAC	231	71	0	0	0	0
MYL3	GTACACTGGTACTGACTG	1101	503	0	1	0	11
MYL3	ACTGCACACAACACGTGT	398	357	0	1	0	4
MYL4	GTACACTGGTTGGTGTGT	441	339	1	0	0	0
MYL4	CAACACTGGTTGGTGTCA	85	293	0	1	0	1
MYL4	TGACACTGGTTGGTGTAC	21	35	0	0	0	0
MYL4	ACACACTGGTTGGTGTTG	1076	443	1	1	0	0
MYL4	CATGCAGTTGCATGGTTG	182	198	0	0	0	1
MYL9	ACTGGTGTCACAACGTTG	1352	924	2	0	2	0
MYL9	TGACACTGGTCAGTTGAC	57	54	0	0	0	0
MYL9	ACACACTGGTCAGTTGTG	253	200	0	0	0	0
MYL9	GTACACTGGTCACAGTGT	593	529	1	0	0	0
MYL9	CAACACTGGTCACAGTCA	469	607	2	0	0	0
MYL9	TGTGCATGCAGTACTGCA	237	712	0	0	0	0
MYLK	ACCATGCATGCAGTTGCA	95	271	1	0	0	0
MYLK	GTCATGCATGCAGTTGAC	44	17	0	0	0	0
MYLK	TGACGTTGTGACCACAGT	376	293	1	0	0	0
MYLK	ACACGTTGTGACCACAAC	167	42	0	0	0	0
MYLK	GTTGGTACTGACCACACA	46	65	0	0	0	1
MYLK2	TGCATGCATGCAGTACAC	12	11	0	0	0	0
MYLK2	ACCATGCATGCAGTACTG	295	179	1	0	0	0
MYLK2	CACATGCATGCAGTTGGT	181	218	0	1	0	0
MYLK2	GTACGTTGTGACCAGTTG	518	1005	1	2	0	0
MYLK2	TGTGGTTGACACCAACCA	133	158	0	0	0	0
MYLK2	ACTGCAACGTTGGTCAGT	61	34	0	0	0	0
MYO1C	TGTGGTCACAGTCACAAC	7	5	0	0	0	0
MYO1C	ACTGGTCACAGTCACATG	105	145	0	0	0	0
MYO1C	CATGGTCACAGTCAACGT	812	526	0	0	0	0
MYO1C	ACTGGTCACAGTCAACCA	92	73	0	0	0	0
MYO1C	GTTGGTCACAGTCAACAC	13	55	0	0	0	0
MYOD1	GTACGTACTGTGGTCAGT	212	183	1	0	0	1
MYOD1	CAACGTACTGTGGTCACA	114	122	33	0	0	6
MYOD1	TGACGTACTGTGGTCAAC	3	31	0	0	0	0
MYOD1	ACACGTACTGTGGTCATG	723	290	1	0	0	0
MYOD1	CAACGTACTGTGGTACGT	248	249	0	0	0	0
MYOG	TGACGTACTGTGACGTTG	765	481	0	3	1	0
MYOG	ACACGTACTGTGACCAGT	362	447	1	1	0	0
MYOG	TGACGTACTGTGACCACA	124	70	0	0	0	15
MYOG	CAACGTACTGTGACCAAC	43	57	0	0	0	0
MYOG	GTACGTACTGTGACCATG	72	60	0	0	0	0
MYST3	TGACGTCAACACGTACAC	27	8	0	0	0	0
MYST3	ACACGTCAACACGTACTG	56	69	0	0	0	0
MYST3	CAACGTCAACACGTTGGT	616	295	1	1	0	0
MYST3	ACACGTCAACACGTTGCA	10	12	0	0	0	0
MYST3	CATGCAGTGTTGTGCAGT	127	96	1	0	0	0
MYST4	ACACGTACTGTGCAGTGT	341	373	1	0	1	0
MYST4	TGACGTACTGTGGTTGCA	139	131	0	0	0	1
MYST4	CAACGTACTGTGGTTGAC	21	48	0	0	0	0
MYST4	GTACGTACTGTGGTTGTG	261	436	1	0	0	0
MYST4	TGACGTACTGTGCAGTCA	198	179	0	0	0	0
MYT1	CAACGTACTGTGTGGTCA	487	352	1	0	1	0
MYT1	TGACGTACTGTGTGGTAC	32	44	0	0	0	1
MYT1	ACACGTACTGTGTGGTTG	1158	528	0	1	0	0
MYT1	CAACGTACTGTGTGCAGT	13	33	0	0	0	0
MYT1	ACACGTACTGTGTGCACA	182	412	1	0	0	1
NACA	GTACGTTGCATGCAACGT	162	120	0	0	0	0
NACA	CAACGTTGCATGCAACCA	221	119	0	0	0	0
NACA	TGACGTTGCATGACTGAC	5	7	1	0	0	0
NACA	ACACGTTGCATGACTGTG	112	90	0	0	0	0
NACA	GTACGTTGCATGTGGTGT	472	465	0	1	0	0
NADK	TGACCAACACTGTGTGGT	707	320	1	0	1	9
NADK	GTACCAACACTGTGACTG	328	513	0	0	0	0
NADK	GTACCAACACTGTGTGCA	386	372	26	0	0	4
NADK	ACACCAACACTGTGTGAC	55	58	0	0	0	0
NADK	TGTGGTACTGTGACACAC	36	32	0	0	0	0
NADSYN1	GTACACGTCAACGTACCA	287	163	1	0	0	0
NADSYN1	ACACACGTCAACGTACAC	86	27	0	0	0	0
NADSYN1	CAACACGTCAACGTACTG	877	740	3	2	0	1
NADSYN1	GTACACGTCAACGTTGGT	95	71	0	0	0	0
NADSYN1	ACTGGTTGTGGTTGACGT	64	98	1	0	0	0
NAE1	ACACGTGTCATGCATGGT	417	104	0	0	0	0
NAE1	TGACGTGTCATGCATGCA	199	212	0	1	0	0
NAE1	CAACGTGTCATGCATGAC	17	48	0	0	0	2
NAE1	GTACGTGTCATGCATGTG	546	398	4	109	0	0

NAE1	ACACGTGTCATGACGTGT	228	312	0	0	0	0
NAGA	ACACTGTGTGGTGTGTAC	129	106	18	8623	372	1
NAGA	CAACTGTGTGGTGTGTTG	1694	803	5	5	1	7
NAGA	GTACTGTGTGGTGTCAGT	136	227	1	0	0	0
NAGA	CAACTGTGTGGTGTCACA	60	66	0	0	0	0
NAGA	TGACTGTGTGGTGTCAAC	138	18	0	0	0	0
NAGA	TGTGCACACAACACGTCA	54	64	0	0	0	0
NAGLU	TGACTGACCAGTTGTGTG	72	117	0	0	0	0
NAGLU	GTACTGACCACAGTGTGT	1056	1145	1	0	0	4
NAGLU	CAACTGACCACAGTGTCA	146	205	1	0	0	0
NAGLU	TGACTGACCACAGTGTAC	314	319	1	1	0	0
NAGLU	ACACTGACCACAGTGTTG	338	388	0	0	0	0
NAGS	TGACACGTACCATGCAGT	145	165	1	1	0	0
NAGS	GTACACGTACCATGCACA	185	231	2	0	0	0
NAGS	ACACACGTACCATGCAAC	25	11	0	0	0	0
NAGS	CAACACGTACCATGCATG	176	156	0	0	0	1
NAGS	TGTGCAGTTGGTACGTTG	136	62	0	0	0	1
NAIP	CAACTGACCACAGTCAGT	332	234	0	0	0	1
NAIP	ACACTGACCACAGTCACA	18	18	0	0	0	0
NAIP	GTACTGACCACAGTCAAC	138	33	0	0	0	0
NAIP	TGACTGACCACAGTCATG	145	146	0	1	0	0
NAIP	ACACTGACCACAGTACGT	98	73	0	0	0	0
NANOG	ACCATGTGCAACTGACTG	46	52	0	0	0	0
NANOG	CACATGTGCAACTGTGGT	614	576	1	0	0	0
NANOG	ACCATGTGCAACTGTGCA	163	107	0	0	0	0
NANOG	GTCATGTGCAACTGTGAC	54	50	0	0	0	0
NANOG	TGCATGTGCAACTGTGTG	92	156	0	0	0	0
NANOG	GTTGCACATGTGACGTGT	152	252	1	0	0	4
NANOS1	ACTGGTCAGTGTCAGTGT	726	564	6	0	1	1
NANOS1	GTTGGTCAGTGTCAGTTG	1204	1037	2	1	1	12
NANOS1	TGTGGTCAGTGTCAGTCA	330	295	0	0	0	0
NANOS1	CATGGTCAGTGTCAGTAC	83	100	0	0	0	0
NANOS1	TGTGGTCAGTGTCACAGT	691	228	2	1	0	0
NAPG	ACACCAGTACCAGTCAAC	51	40	0	0	0	0
NAPG	CAACCAGTACCAGTCATG	315	192	2	0	0	0
NAPG	GTACCAGTACCAGTACGT	97	56	0	0	0	0
NAPG	CAACCAGTACCAGTACCA	487	235	0	0	0	1
NAPG	TGACCAGTACCAGTACAC	18	25	0	0	0	0
NAPG	CATGCACAGTTGTGACTG	1309	624	3	0	0	3
NARS	TGACACGTACCAACGTGT	311	327	0	0	0	0
NARS	GTACACGTACCAACGTCA	76	137	0	0	0	0
NARS	ACACACGTACCAACGTAC	14	10	0	0	0	0
NARS	CAACACGTACCAACGTTG	316	301	1	0	0	0
NARS	GTACACGTACCAACCAGT	89	77	0	0	0	0
NAT1	ACACCACAACCACAGTGT	391	377	0	0	0	0
NAT1	TGACCACAACCACAGTCA	566	672	1	0	0	0
NAT1	CAACCACAACCACAGTAC	46	61	0	0	0	0
NAT1	GTACCACAACCACAGTTG	332	349	1	0	0	0
NAT1	TGACCACAACCACACAGT	595	359	1	0	0	1
NAT2	ACACCACAACGTTGCAGT	308	372	1	0	1	0
NAT2	TGACCACAACGTTGGTTG	306	366	1	0	0	1
NAT2	TGACCACAACGTTGCACA	215	107	0	0	0	9
NAT2	CAACCACAACGTTGCAAC	3	2	0	0	0	0
NAT2	CATGCAGTACTGCAGTGT	506	552	1	1	0	0
NAT5	CAACCACATGTGCATGTG	395	200	1	0	0	0
NAT5	TGACCACATGTGACGTGT	104	104	1	0	0	50
NAT5	GTACCACATGTGACGTCA	261	297	0	0	0	0
NAT5	ACACCACATGTGACGTAC	20	14	0	0	0	0
NAT5	GTTGGTGTACCACAACAC	23	17	0	0	0	0
NAT6	GTACCACATGACTGCATG	460	403	1	1	1	1
NAT6	ACACCACATGACTGCAGT	92	95	1	0	0	1
NAT6	TGACCACATGACTGCACA	35	57	0	0	0	0
NAT6	CAACCACATGACTGCAAC	97	47	0	0	0	0
NAT6	CATGCAGTGTTGGTCACA	53	30	0	0	0	0
NBL1	TGACCAACTGACCATGGT	475	209	23	21	15	10
NBL1	ACACCAACTGACCAACGT	144	440	0	0	0	0
NBL1	TGACCAACTGACCAACCA	264	282	1	3	0	2
NBL1	CAACCAACTGACCAACAC	78	109	0	0	0	0
NBL1	GTACCAACTGACCAACTG	1115	1372	0	0	0	3
NBN	GTACCATGACGTGTACGT	184	173	0	0	0	0
NBN	CAACCATGACGTGTACCA	121	107	0	0	0	0
NBN	TGACCATGACGTGTACAC	23	25	0	0	0	0
NBN	ACACCATGACGTGTACTG	55	119	0	0	0	0
NBN	CAACCATGACGTGTTGGT	364	383	0	0	0	0
NCAM1	GTACTGTGGTGTACGTCA	29	16	0	0	0	0

NCAM1	ACACTGTGGTGTACGTAC	30	58	0	0	0	0
NCAM1	CAACTGTGGTGTACGTTG	626	190	1	0	0	1
NCAM1	GTACTGTGGTGTACCAGT	293	148	1	1	0	0
NCAM1	CAACTGTGGTGTACCACA	89	46	0	0	0	0
NCAM1	CATGCACATGCAACGTGT	553	924	2	0	0	1
NCF1	TGTGCAGTGTCAACGTCA	51	53	6	0	2	12
NCF1	TGACCATGGTACGTTGGT	85	79	0	0	0	0
NCF1	GTACCATGGTACGTTGCA	72	66	0	0	0	1
NCF1	ACACCATGGTACGTTGAC	13	26	0	0	0	0
NCF1	CAACCATGGTACGTTGTG	169	245	1	0	0	0
NCF2	TGACCATGGTACCAGTGT	365	414	1	0	0	0
NCF2	GTACCATGGTACCAGTCA	420	331	0	0	0	0
NCF2	ACACCATGGTACCAGTAC	23	15	0	0	0	0
NCF2	CAACCATGGTACCAGTTG	256	334	1	0	0	0
NCF2	GTACCATGGTACCACAGT	237	191	2	0	0	1
NCF4	GTACTGACACCATGACAC	47	58	2	0	1	1
NCF4	TGACTGACACCATGACTG	887	480	2	0	0	1
NCF4	ACACTGACACCATGTGGT	387	379	1	0	0	82
NCF4	TGACTGACACCATGTGCA	202	221	2	0	0	0
NCF4	CAACTGACACCATGTGAC	44	10	0	0	0	0
NCK1	GTACGTTGTGACGTTGTG	51	214	0	0	0	0
NCK1	TGTGGTCACAGTCAACTG	313	439	1	2	0	6
NCK1	ACTGGTCACAGTCATGGT	169	212	1	1	0	14
NCK1	TGTGGTCACAGTCATGCA	142	242	0	0	0	0
NCK1	CATGGTCACAGTCATGAC	60	51	0	0	0	0
NCK2	GTTGGTCACAGTCAGTCA	61	70	0	0	0	0
NCK2	ACTGGTCACAGTCAGTAC	28	27	0	0	0	0
NCK2	CATGGTCACAGTCAGTTG	1827	2042	3	2	0	2
NCK2	GTTGGTCACAGTCACAGT	186	238	0	0	0	0
NCK2	CATGGTCACAGTCACACA	44	56	0	0	0	0
NCKAP1	GTTGGTACCAACCAACCA	147	34	0	0	0	0
NCKAP1	ACTGGTACCAACCAACAC	48	52	0	1	0	0
NCKAP1	CATGGTACCAACCAACTG	126	135	0	0	0	0
NCKAP1	GTTGGTACCAACCATGGT	329	588	2	0	0	0
NCKAP1	CATGGTACCAACCATGCA	324	368	0	0	0	0
NCL	GTTGGTTGACGTCAGTCA	145	142	0	0	1	0
NCL	TGACTGCATGTGACCACA	78	63	0	0	0	0
NCL	CAACTGCATGTGACCAAC	27	34	0	0	0	0
NCL	GTACTGCATGTGACCATG	1	18	0	0	0	0
NCL	TGACTGCATGTGACACGT	220	152	1	0	0	0
NCOA1	GTCATGTGGTTGCAACTG	390	329	3	0	0	11
NCOA1	TGCATGTGGTTGCATGGT	1476	1261	0	1	0	2
NCOA1	GTCATGTGGTTGCATGCA	80	79	0	0	0	0
NCOA1	ACCATGTGGTTGCATGAC	70	84	0	0	0	0
NCOA1	CACATGTGGTTGCATGTG	972	501	1	0	0	0
NCOA2	GTACGTTGGTGTACACGT	114	47	0	0	0	0
NCOA2	CAACGTTGGTGTACACCA	166	368	0	58	0	1
NCOA2	TGACGTTGGTGTACACAC	30	7	0	0	0	0
NCOA2	ACACGTTGGTGTACACTG	233	142	0	0	0	0
NCOA2	CAACGTTGGTGTACTGGT	417	183	0	0	0	0
NCOA3	TGACGTTGGTCAGTCAAC	14	16	0	0	0	0
NCOA3	ACACGTTGGTCAGTCATG	120	117	0	0	0	0
NCOA3	CAACGTTGGTCAGTACGT	95	102	1	0	0	0
NCOA3	ACACGTTGGTCAGTACCA	306	241	1	0	0	0
NCOA3	GTACGTTGGTCAGTACAC	2	14	0	0	0	0
NCOA3	TGTGCATGCACACACAAC	19	28	0	0	0	0
NCOA4	GTACGTTGGTCACACACA	109	130	51	42	66	80
NCOA4	TGACGTTGGTCACACAGT	147	95	0	0	0	2
NCOA4	ACACGTTGGTCACACAAC	19	17	0	0	0	0
NCOA4	CAACGTTGGTCACACATG	302	646	0	2	0	1
NCOA4	GTTGGTGTCAGTACGTAC	169	50	0	0	0	0
NCOR1	CAACTGCAACGTTGCACA	204	203	0	0	0	1
NCOR1	TGACTGCAACGTTGCAAC	26	27	0	0	0	0
NCOR1	ACACTGCAACGTTGCATG	348	279	1	0	0	0
NCOR1	CAACTGCAACGTTGACGT	220	107	0	0	0	0
NCOR1	ACACTGCAACGTTGACCA	109	109	0	0	0	3
NCOR1	ACTGCAACACTGGTGTTG	900	447	3	1	0	4
NCOR2	GTACTGCAACCAGTGTAC	48	98	1	0	0	0
NCOR2	TGACTGCAACCAGTGTTG	391	464	0	0	0	0
NCOR2	ACACTGCAACCAGTCAGT	51	36	0	0	0	0
NCOR2	TGACTGCAACCAGTCACA	52	88	0	0	0	0
NCOR2	CAACTGCAACCAGTCAAC	9	24	0	0	0	0
NCR1	CAACTGACCAGTGTACTG	1092	476	0	0	2	0
NCR1	ACACTGACCAGTGTACAC	64	65	0	0	1	0
NCR1	GTACTGACCAGTGTCATG	149	429	1	0	0	0

NCR1	TGACTGACCAGTGTACGT	149	154	0	0	0	0
NCR1	GTACTGACCAGTGTACCA	203	433	0	0	0	0
NCSTN	CAACTGACGTCAACACTG	1624	961	1	0	1	1
NCSTN	ACACTGACGTCAACACAC	62	41	0	0	0	0
NCSTN	GTACTGACGTCAACTGGT	203	278	1	0	0	0
NCSTN	CAACTGACGTCAACTGCA	183	163	0	0	0	0
NCSTN	TGACTGACGTCAACTGAC	28	13	0	0	0	0
NDE1	GTTGGTTGTGTGACGTCA	434	383	4	1	0	4
NDE1	ACTGGTTGTGTGACGTAC	21	10	0	0	0	0
NDE1	CATGGTTGTGTGACGTTG	186	219	0	1	0	0
NDE1	GTTGGTTGTGTGACCAGT	102	132	0	0	0	0
NDE1	CATGGTTGTGTGACCACA	84	127	0	0	0	0
NDE1	GTTGCACATGGTCAACAC	23	30	0	0	0	0
NDEL1	GTTGGTCACAACTGGTTG	249	275	0	0	0	1
NDEL1	TGTGGTCACAACTGCAGT	265	233	2	0	0	5
NDEL1	GTTGGTCACAACTGCACA	77	76	0	0	0	0
NDEL1	ACTGGTCACAACTGCAAC	106	53	0	0	0	0
NDEL1	CATGGTCACAACTGCATG	192	294	2	1	0	1
NDNL2	TGTGGTGTACCATGGTTG	853	1383	2	2	2	0
NDNL2	ACTGGTGTACCATGGTCA	616	443	0	2	0	24
NDNL2	GTTGGTGTACCATGGTAC	72	38	0	0	0	0
NDNL2	ACTGGTGTACCATGCAGT	180	306	0	0	0	0
NDNL2	TGTGGTGTACCATGCACA	49	74	0	0	0	0
NDNL2	ACTGCATGGTACACGTAC	31	43	1	0	0	0
NDRG2	CATGGTTGCATGACGTAC	12	17	0	0	0	0
NDRG2	GTTGGTTGCATGACGTTG	205	153	0	1	0	1
NDRG2	TGTGGTTGCATGACCAGT	134	120	0	0	0	0
NDRG2	GTTGGTTGCATGACCACA	12	29	0	0	0	0
NDRG2	ACTGGTTGCATGACCAAC	24	27	0	0	0	1
NDST1	TGACGTCAGTTGACGTCA	26	25	0	0	0	0
NDST1	CAACGTCAGTTGACGTAC	104	29	0	0	0	0
NDST1	GTACGTCAGTTGACGTTG	95	159	0	0	0	0
NDST1	TGACGTCAGTTGACCAGT	188	80	1	0	0	0
NDST1	TGTGGTGTCAGTTGCATG	242	290	0	0	0	0
NDST2	TGACCACACAACACCACA	31	76	0	1	0	0
NDST2	CAACCACACAACACCAAC	40	27	0	0	0	0
NDST2	GTACCACACAACACCATG	332	184	0	0	0	0
NDST2	TGACCACACAACACACGT	26	39	0	0	0	0
NDST2	CATGGTGTCAGTTGCAGT	315	321	0	0	0	2
NDST2	GTTGCATGGTTGTGACAC	110	84	0	0	0	0
NDST3	CAACCAACGTTGCAACGT	816	711	31	8171	1296	8
NDST3	ACACCAACGTTGCAACCA	287	544	3	0	0	3
NDST3	GTACCAACGTTGCAACAC	45	32	0	0	0	0
NDST3	TGACCAACGTTGCAACTG	51	74	0	0	0	0
NDST3	ACACCAACGTTGCATGGT	506	364	0	0	0	1
NDST3	GTTGCACACATGCACAAC	12	20	0	0	0	0
NDST4	TGTGCACATGACACGTGT	632	1127	2	1	1	0
NDST4	GTACCAACCAGTCAGTTG	66	62	0	0	0	1
NDST4	TGACCAACCAGTCACAGT	576	697	16	1	0	9
NDST4	GTACCAACCAGTCACACA	22	27	0	0	0	0
NDST4	ACACCAACCAGTCACAAC	76	47	0	0	0	1
NDST4	CAACCAACCAGTCACATG	122	98	0	0	0	0
NDUFA1	TGACCAGTCACAACGTCA	186	194	0	1	0	1
NDUFA1	CAACCAGTCACAACGTAC	162	94	0	0	0	0
NDUFA1	GTACCAGTCACAACGTTG	321	229	1	1	0	0
NDUFA1	TGACCAGTCACAACCAGT	44	66	1	7	0	0
NDUFA1	GTACCAGTCACAACCACA	291	56	0	0	0	0
NDUFA1	CATGCAACGTGTGTGTTG	1311	565	0	1	0	1
NDUFA10	CAACCAGTCAGTCACAGT	150	430	0	0	1	1
NDUFA10	GTACCAGTCAGTCAGTGT	445	673	2	1	0	5
NDUFA10	CAACCAGTCAGTCAGTCA	137	177	0	0	0	3
NDUFA10	TGACCAGTCAGTCAGTAC	243	49	0	0	0	0
NDUFA10	ACACCAGTCAGTCAGTTG	1398	1082	0	1	0	2
NDUFA11	ACACCAGTGTACCACACA	29	32	1	0	0	0
NDUFA11	GTACCAGTGTACCACAAC	35	34	1	0	0	0
NDUFA11	TGACCAGTGTACCACATG	171	200	0	0	0	0
NDUFA11	ACACCAGTGTACCAACGT	241	258	0	0	0	0
NDUFA11	TGACCAGTGTACCAACCA	311	175	1	1	0	0
NDUFA12	ACACTGACCAACACGTTG	1295	881	1	1	1	2
NDUFA12	CAACTGACCAACACCAGT	645	388	0	0	0	0
NDUFA12	ACACTGACCAACACCACA	73	85	0	0	0	0
NDUFA12	GTACTGACCAACACCAAC	25	22	0	0	0	0
NDUFA12	TGACTGACCAACACCATG	746	361	1	2	0	6
NDUFA13	TGACTGACTGGTGTCAGT	178	170	0	0	0	0
NDUFA13	GTACTGACTGGTGTCACA	169	132	0	0	0	0

NDUFA13	ACACTGACTGGTGTCAAC	12	11	0	0	0	0
NDUFA13	CAACTGACTGGTGTCATG	354	222	1	0	0	2
NDUFA13	GTACTGACTGGTGTACGT	142	212	1	0	0	0
NDUFA4	GTACCAGTCACAGTACTG	892	870	0	1	0	0
NDUFA4	TGACCAGTCACAGTTGGT	456	470	0	1	0	2
NDUFA4	GTACCAGTCACAGTTGCA	194	205	0	0	0	12
NDUFA4	ACACCAGTCACAGTTGAC	18	27	0	0	0	0
NDUFA4	CAACCAGTCACAGTTGTG	1298	1227	1	0	0	0
NDUFA5	TGACCAGTCAGTTGTGTG	529	732	0	2	1	0
NDUFA5	CAACCAGTCAGTTGTGGT	376	267	0	0	0	0
NDUFA5	ACACCAGTCAGTTGTGCA	437	242	0	0	0	0
NDUFA5	GTACCAGTCAGTTGTGAC	131	107	0	0	0	0
NDUFA5	GTACCAGTCACAGTGTGT	531	684	1	0	0	1
NDUFA5	GTTGCAACGTGTGTCAGT	293	380	0	0	0	2
NDUFA8	CAACCAGTCAGTACGTTG	1393	1620	2	1	1	1
NDUFA8	GTACCAGTCAGTACCAGT	6	6	0	0	0	0
NDUFA8	CAACCAGTCAGTACCACA	114	78	0	0	0	1
NDUFA8	TGACCAGTCAGTACCAAC	24	29	0	0	0	0
NDUFA8	ACACCAGTCAGTACCATG	388	157	2	0	0	0
NDUFB11	GTTGGTGTCAACCAGTCA	805	489	4	2	1	0
NDUFB11	CATGGTGTCAACGTTGTG	341	661	0	1	0	0
NDUFB11	TGTGGTGTCAACCAGTGT	475	475	1	1	0	0
NDUFB11	ACTGGTGTCAACCAGTAC	13	20	0	0	0	0
NDUFB11	ACTGACACACACGTACTG	38	49	0	0	0	0
NDUFB2	TGACTGACACTGGTTGAC	30	38	0	13	0	0
NDUFB2	ACACTGACACTGGTTGTG	334	297	0	0	0	3
NDUFB2	GTACTGACACTGCAGTGT	777	721	0	0	0	0
NDUFB2	CAACTGACACTGCAGTCA	515	357	0	0	0	0
NDUFB2	TGACTGACACTGCAGTAC	177	114	1	0	0	0
NDUFB4	CAACCAGTGTTGACGTCA	194	120	0	0	0	0
NDUFB4	TGACCAGTGTTGACGTAC	129	51	0	0	0	0
NDUFB4	ACACCAGTGTTGACGTTG	70	93	0	0	0	2
NDUFB4	CAACCAGTGTTGACCAGT	232	310	1	0	0	0
NDUFB4	ACACCAGTGTTGACCACA	208	82	1	0	0	0
NDUFB5	TGACCAGTGTTGCAGTTG	649	783	0	1	1	0
NDUFB5	ACACCAGTGTTGCACAGT	88	139	0	0	0	0
NDUFB5	TGACCAGTGTTGCACACA	120	114	1	0	0	0
NDUFB5	CAACCAGTGTTGCACAAC	54	45	0	0	0	0
NDUFB5	GTACCAGTGTTGCACATG	275	148	1	0	0	0
NDUFB6	CAACCAGTGTTGGTACCA	195	216	0	0	0	0
NDUFB6	TGACCAGTGTTGGTACAC	101	63	0	0	0	0
NDUFB6	ACACCAGTGTTGGTACTG	813	982	1	1	0	0
NDUFB6	CAACCAGTGTTGGTTGGT	293	243	1	0	0	0
NDUFB6	ACACCAGTGTTGGTTGCA	181	272	0	0	0	0
NDUFB7	ACACCAGTGTTGGTCAAC	34	91	0	0	0	0
NDUFB7	CAACCAGTGTTGGTCATG	94	286	0	1	0	0
NDUFB7	GTACCAGTGTTGGTACGT	90	110	0	0	0	0
NDUFB7	CATGCAGTACTGGTCATG	321	528	0	1	0	2
NDUFB7	GTTGCAGTACTGGTACGT	213	247	0	0	0	0
NDUFB8	CAACCAGTGTTGGTGTAC	94	44	0	0	0	0
NDUFB8	GTACCAGTGTTGGTGTTG	1153	462	38	0	0	0
NDUFB8	TGACCAGTGTTGGTCAGT	74	107	0	0	0	0
NDUFB8	GTACCAGTGTTGGTCACA	203	418	0	0	0	0
NDUFB8	GTTGACGTTGCAGTTGGT	143	189	0	1	0	0
NDUFS1	TGACTGACACACGTACCA	72	77	0	0	0	0
NDUFS1	CAACTGACACACGTACAC	36	41	0	0	0	1
NDUFS1	GTACTGACACACGTACTG	193	439	0	0	0	130
NDUFS1	TGACTGACACACGTTGGT	160	275	0	0	0	0
NDUFS1	GTACTGACACACGTTGCA	56	32	0	0	0	0
NDUFS2	CAACCAACACGTTGTGGT	66	90	0	0	0	0
NDUFS2	ACACCAACACGTTGTGCA	87	131	0	0	0	0
NDUFS2	GTACCAACACGTTGTGAC	28	43	0	1	0	0
NDUFS2	TGTGCAGTCACATGTGTG	261	403	0	0	0	0
NDUFS2	TGTGCAGTCAACGTGTGT	407	395	0	1	0	66
NDUFS4	ACACCAACACCAGTGTTG	820	639	160628	166	12	12
NDUFS4	TGACCAACACGTTGTGTG	415	646	2	0	1	4
NDUFS4	GTACCAACACCAGTGTGT	948	1610	1	2	0	3
NDUFS4	CAACCAACACCAGTGTCA	1454	1252	1	1	0	0
NDUFS4	TGACCAACACCAGTGTAC	168	33	0	0	0	0
NDUFS4	GTTGCACAGTTGTGTGGT	533	372	1	1	0	1
NDUFS7	CAACGTTGTGTGTGTGCA	64	69	0	0	0	0
NDUFS7	TGACGTTGTGTGTGTGAC	30	22	0	0	0	0
NDUFS7	ACACGTTGTGTGTGTGTG	114	105	0	0	0	0
NDUFS7	GTACCAGTGTGTGTGTGT	1080	257	1	0	0	0
NDUFS7	CAACCAGTGTGTGTGTCA	110	102	0	0	0	0

NDUFS8	TGTGCAGTCATGTGGTTG	1091	863	2	1	1	12
NDUFS8	TGACCAACACCAGTACCA	97	186	30	0	0	1
NDUFS8	CAACCAACACCAGTACAC	193	87	0	0	0	0
NDUFS8	GTACCAACACCAGTACTG	617	561	1	1	0	0
NDUFS8	ACTGCAGTCATGTGCAGT	156	183	0	0	0	11
NDUFS8	CATGCACAGTTGTGTGCA	169	275	1	0	0	0
NDUFV1	CAACCAGTGTACCAACAC	161	318	6	0	0	0
NDUFV1	GTACCAGTGTACCAACTG	783	1035	3	0	0	3
NDUFV1	TGACCAGTGTACCATGGT	343	460	1	1	0	0
NDUFV1	GTACCAGTGTACCATGCA	438	335	0	1	0	0
NDUFV1	GTACCAGTGTACTGCACA	155	174	0	0	0	522
NDUFV1	ACTGCAACCAACCATGAC	151	231	61	0	0	0
NDUFV2	CAACCAGTGTACCAGTCA	752	1189	1	1	2	1
NDUFV2	GTACCAGTGTACCAGTGT	335	283	2	0	0	3
NDUFV2	TGACCAGTGTACCAGTAC	31	78	0	0	0	0
NDUFV2	ACACCAGTGTACCAGTTG	339	272	1	1	0	0
NDUFV2	CAACCAGTGTACCACAGT	188	404	0	0	0	0
NDUFV2	ACTGCACATGCAACGTCA	613	657	0	1	0	11
NEDD4L	ACCATGCATGCAGTGTGT	536	549	0	0	1	0
NEDD4L	TGCATGCATGGTTGTGGT	345	561	0	0	0	0
NEDD4L	GTCATGCATGGTTGTGCA	238	78	0	1	0	0
NEDD4L	ACCATGCATGGTTGTGAC	8	7	0	0	0	0
NEDD4L	CACATGCATGGTTGTGTG	79	21	0	0	0	0
NEDD8	ACACGTGTGTACACCACA	8	2	0	0	0	0
NEDD8	GTACGTGTGTACACCAAC	9	20	0	0	0	0
NEDD8	TGACGTGTGTACACCATG	69	77	0	0	0	0
NEDD8	ACACGTGTGTACACACGT	11	6	0	0	0	0
NEDD8	TGACGTGTGTACACACCA	158	147	0	0	0	0
NEDD8	ACTGCACAACGTTGCAAC	13	13	0	0	0	0
NEFH	TGACTGACCACAGTACCA	232	243	1	0	0	0
NEFH	CAACTGACCACAGTACAC	48	76	0	0	0	0
NEFH	GTACTGACCACAGTACTG	705	540	0	1	0	0
NEFH	TGACTGACCACAGTTGGT	446	258	1	0	0	0
NEFH	CATGGTGTGTTGTGACTG	352	1348	1	0	0	1
NEFL	ACTGGTGTGTGTACGTTG	524	456	1	1	0	13
NEFL	CATGGTGTGTGTACCAGT	62	129	0	0	0	0
NEFL	ACTGGTGTGTGTACCACA	40	86	0	0	0	0
NEFL	GTTGGTGTGTGTACCAAC	16	60	0	0	0	0
NEFL	GTTGACCATGTGTGCACA	139	102	1	0	0	0
NEFM	GTTGGTGTACTGCAACGT	68	82	0	0	1	0
NEFM	TGTGGTGTACTGCAACAC	341	127	1	0	1	6
NEFM	GTTGGTGTGTTGTGACCA	182	247	169	1	0	5
NEFM	CATGGTGTACTGCACATG	768	275	0	2	0	1
NEFM	CATGGTGTACTGCAACCA	304	182	0	0	0	0
NEFM	ACTGCACACATGTGACTG	166	133	0	0	0	0
NEIL1	CAACACCATGCAGTGTGT	2042	1032	2	0	1	1
NEIL1	TGACACCATGCAGTGTTG	1400	993	2	1	1	2
NEIL1	ACACACCATGCAGTGTCA	1165	992	1	2	0	4
NEIL1	GTACACCATGCAGTGTAC	57	45	0	0	0	0
NEIL1	TGTGGTTGTGACGTTGGT	439	315	0	0	0	2
NEIL1	TGTGCACATGTGCATGAC	15	9	1	0	0	0
NEIL2	CAACTGACCAACCATGCA	760	924	2	0	1	0
NEIL2	GTACTGACCAACCAACCA	887	781	2	1	0	1
NEIL2	ACACTGACCAACCAACAC	38	21	0	0	0	0
NEIL2	CAACTGACCAACCAACTG	639	641	3	0	0	4
NEIL2	GTACTGACCAACCATGGT	547	238	0	0	0	0
NEIL2	ACTGCAACACGTACTGAC	24	34	0	0	0	0
NEIL3	CAACGTGTACTGCACAAC	43	68	0	0	0	0
NEIL3	GTACGTGTACTGCACATG	153	179	1	0	0	31
NEIL3	TGACGTGTACTGCAACGT	271	255	0	0	0	0
NEIL3	GTACGTGTACTGCAACCA	91	104	0	0	0	0
NEIL3	ACACGTGTACTGCAACAC	37	64	0	0	0	0
NEIL3	CATGCACATGGTCAACGT	193	226	0	0	0	0
NEK1	ACACGTTGACCAACTGAC	8	5	2	0	0	0
NEK1	CAACGTTGACCAACTGTG	334	257	2	0	0	82
NEK1	TGACGTTGACCATGGTGT	495	1702	2	1	0	0
NEK1	GTACGTTGACCATGGTCA	334	317	0	2	0	1
NEK1	ACACGTTGACCATGGTAC	59	85	0	0	0	0
NEK2	ACCATGCATGCACACAGT	60	77	0	0	0	0
NEK2	TGCATGCATGCACACACA	119	85	0	0	0	0
NEK2	CACATGCATGCACACAAC	26	38	0	0	0	0
NEK2	GTCATGCATGCACACATG	213	308	0	1	0	0
NEK2	TGCATGCATGCACAACGT	242	137	0	0	0	0
NES	CAACGTACGTACCAGTTG	691	951	0	0	0	0
NES	GTACGTACGTACCACAGT	99	108	0	0	0	0

NES	CAACGTACGTACCACACA	145	154	0	0	0	0
NES	TGACGTACGTACCACAAC	19	7	0	0	0	0
NES	ACACGTACGTACCACATG	156	185	1	0	0	0
NEU1	CAACTGTGTGCAGTCATG	312	256	0	0	0	0
NEU1	GTACTGTGTGCAGTACGT	38	37	0	0	0	0
NEU1	CAACTGTGTGCAGTACCA	389	223	1	0	0	0
NEU1	TGACTGTGTGCAGTACAC	57	93	0	0	0	0
NEU1	ACACTGTGTGCAGTACTG	410	658	1	1	0	0
NEU2	GTTGGTTGTGACTGTGGT	247	825	0	0	1	0
NEU2	CATGGTTGTGACTGTGCA	280	145	0	0	0	0
NEU2	TGTGGTTGTGACTGTGAC	42	59	0	0	0	0
NEU2	ACTGGTTGTGACTGTGTG	166	399	1	0	0	0
NEU2	CATGCACACATGTGTGGT	331	858	1	0	0	1
NEU2	ACTGCACACATGTGTGCA	120	60	2	0	0	0
NEU3	ACCATGTGACGTCATGTG	402	500	0	1	0	0
NEU3	GTCATGTGACGTACGTGT	163	197	0	1	0	0
NEU3	CACATGTGACGTACGTCA	37	53	2	0	0	0
NEU3	TGCATGTGACGTACGTAC	5	22	0	0	0	0
NEU3	GTTGGTTGTGCAACTGCA	107	316	1	0	0	0
NEU3	TGTGCAACACCATGCAGT	32	61	0	0	0	0
NEU4	TGTGGTTGTGGTTGACCA	99	217	0	0	0	0
NEU4	CATGGTTGTGGTTGACAC	79	29	0	0	0	0
NEU4	GTTGGTTGTGGTTGACTG	783	1344	1	1	0	25
NEU4	TGTGGTTGTGGTTGTGGT	543	385	1	1	0	8
NEU4	GTTGGTTGTGGTTGTGCA	368	371	1	0	0	2
NEU4	CATGCAACGTTGTGGTAC	61	88	1	1	0	1
NEURL	ACACCACACACAGTCAGT	327	562	1	0	0	2
NEURL	TGACCACACACAGTCACA	93	74	0	0	0	1
NEURL	CAACCACACACAGTCAAC	34	47	0	0	0	0
NEURL	GTACCACACACAGTCATG	443	208	0	0	0	0
NEURL	TGACCACACACAGTACGT	274	477	0	0	0	0
NEURL	TGTGCAACCATGTGTGCA	178	233	0	0	0	1
NEUROD1	TGACGTTGGTACGTCACA	11	4	0	0	0	0
NEUROD1	CAACGTTGGTACGTCAAC	15	6	0	0	0	0
NEUROD1	GTACGTTGGTACGTCATG	127	203	0	0	0	0
NEUROD1	TGACGTTGGTACGTACGT	46	81	0	0	0	0
NEUROD1	TGACTGTGACACACCAAC	52	40	0	0	0	0
NEUROG1	CAACGTACACCAACACGT	86	77	4	1	2	1
NEUROG1	TGACGTACACCAACCAAC	46	23	0	0	1	0
NEUROG1	CAACGTACACCAACCACA	73	53	0	0	0	0
NEUROG1	ACACGTACACCAACCATG	139	43	0	0	0	0
NEUROG1	TGTGCATGTGGTCACACA	66	77	0	0	0	0
NEUROG3	GTACGTTGGTACTGACGT	98	90	0	0	0	0
NEUROG3	CAACGTTGGTACTGACCA	252	307	1	0	0	0
NEUROG3	TGACGTTGGTACTGACAC	41	18	0	0	0	0
NEUROG3	CATGCAGTACACTGTGGT	1071	526	2	0	0	2
NEUROG3	GTTGACCACAGTACCAAC	262	65	0	0	0	0
NF1	GTACCATGCAGTGTACTG	749	953	1	0	0	7
NF1	TGACCATGCAGTGTTGGT	270	292	1	0	0	0
NF1	GTACCATGCAGTGTTGCA	225	110	0	0	0	0
NF1	ACACCATGCAGTGTTGAC	44	22	0	0	0	3
NF1	CAACCATGCAGTGTTGTG	280	821	0	0	0	1
NFAM1	GTTGGTCAACACTGCAAC	101	80	0	0	2	0
NFAM1	CATGGTCAACACTGCAGT	78	149	0	0	0	0
NFAM1	ACTGGTCAACACTGCACA	208	79	0	0	0	0
NFAM1	TGTGGTCAACACTGCATG	491	392	0	2	0	0
NFAM1	ACTGGTCAACACTGACGT	455	617	1	0	0	30
NFAT5	ACACGTTGGTACACACCA	92	63	0	0	0	0
NFAT5	GTACGTTGGTACACACAC	16	16	0	0	0	1
NFAT5	TGACGTTGGTACACACTG	303	315	2	0	0	0
NFAT5	ACACGTTGGTACACTGGT	189	160	0	2	0	0
NFAT5	TGACGTTGGTACACTGCA	496	397	0	0	0	4
NFAT5	TGTGCATGGTGTCATGCA	148	109	0	0	0	0
NFATC1	GTACGTACACCAACTGTG	333	508	0	0	1	0
NFATC1	CAACGTACACCAACTGAC	47	34	0	0	0	1
NFATC1	ACACGTACACCATGGTGT	1752	1224	1	1	0	2
NFATC1	TGACGTACACCATGGTCA	250	473	0	0	0	0
NFATC1	ACTGCAGTACACACTGGT	263	308	0	1	0	0
NFATC1	CATGCATGGTGTGTACAC	56	24	0	0	0	0
NFATC2	ACACGTACCATGGTGTGT	565	571	1	0	1	0
NFATC2	GTACGTACCAACTGTGAC	63	65	0	0	0	0
NFATC2	TGACGTACCAACTGTGTG	525	511	0	0	0	1
NFATC2	TGACGTACCATGGTGTCA	228	161	0	1	0	0
NFATC2	CAACGTACCATGGTGTAC	57	146	0	0	0	0
NFATC3	CAACGTACGTCAGTTGGT	58	51	0	0	0	1

NFATC3	ACACGTACGTCAGTTGCA	60	61	0	0	0	0
NFATC3	GTACGTACGTCAGTTGAC	45	58	1	4	0	0
NFATC3	TGACGTACGTCAGTTGTG	519	546	0	0	0	0
NFATC3	CAACGTACGTCACAGTGT	559	765	1664	1	0	0
NFATC3	GTTGCATGGTGTCATGTG	105	214	0	2	0	0
NFATC4	ACACGTACGTCAACGTTG	659	521	3	0	1	0
NFATC4	GTACGTACGTCAACGTGT	242	276	1	0	0	0
NFATC4	CAACGTACGTCAACGTCA	279	98	1	0	0	0
NFATC4	TGACGTACGTCAACGTAC	19	23	0	0	0	0
NFATC4	CAACGTACGTCAACCAGT	634	424	0	1	0	2
NFE2	ACACGTACGTCAACCACA	154	160	0	0	0	0
NFE2	GTACGTACGTCAACCAAC	12	11	0	0	0	0
NFE2	TGACGTACGTCAACCATG	55	371	0	0	0	3
NFE2	ACACGTACGTCAACACGT	193	314	0	0	0	0
NFE2	TGACGTACGTCAACACCA	157	443	0	84	0	0
NFE2	CATGCACAACGTTGCATG	59	95	0	0	0	0
NFE2L2	GTACGTGTACCATGTGCA	130	49	0	0	1	0
NFE2L2	ACACGTGTACCATGTGAC	32	41	0	0	0	0
NFE2L2	CAACGTGTACCATGTGTG	384	206	1	1	0	0
NFE2L2	CAACGTGTACACGTGTGT	351	315	1	0	0	1
NFE2L2	ACACGTGTACACGTGTCA	335	172	0	0	0	0
NFE2L2	CATGCAACCAACCATGTG	523	439	6	0	0	0
NFKB1	GTACGTGTGTACTGCAGT	335	254	1	2	0	1
NFKB1	CAACGTGTGTACTGCACA	299	363	0	1	0	0
NFKB1	TGACGTGTGTACTGCAAC	7	23	0	0	0	0
NFKB1	ACACGTGTGTACTGCATG	52	92	1	0	0	0
NFKB1	CAACGTGTGTACTGACGT	134	388	0	0	0	1
NFKB2	CAACGTGTGTACACTGTG	327	365	0	1	0	1
NFKB2	TGACGTGTGTACTGGTGT	899	633	1	0	0	3
NFKB2	GTACGTGTGTACTGGTCA	683	338	2	1	0	0
NFKB2	ACACGTGTGTACTGGTAC	22	34	0	0	0	0
NFKB2	CAACGTGTGTACTGGTTG	276	703	0	0	0	0
NFKBIA	GTCATGTGCACAGTGTCA	50	197	0	0	0	2
NFKBIA	ACCATGTGCACAGTGTAC	124	102	0	0	0	4
NFKBIA	CACATGTGCACAGTGTTG	984	907	0	2	0	2
NFKBIA	GTCATGTGCACAGTCAGT	61	71	0	0	0	0
NFKBIA	GTTGGTGTCAGTACCATG	92	143	0	0	0	0
NFKBIA	GTTGCACATGCAGTTGTG	152	265	0	0	0	0
NFKBIB	GTACGTACTGGTTGGTTG	1012	296	2	1	0	2
NFKBIB	TGACGTACTGGTTGCAGT	27	54	0	0	0	0
NFKBIB	GTACGTACTGGTTGCACA	110	88	0	0	0	0
NFKBIB	ACACGTACTGGTTGCAAC	9	8	0	0	0	0
NFKBIB	CAACGTACTGGTTGCATG	269	278	0	1	0	1
NFKBIE	GTACACTGACCAGTTGCA	343	387	0	0	0	0
NFKBIE	ACACACTGACCAGTTGAC	71	163	1	0	0	0
NFKBIE	CAACACTGACCAGTTGTG	267	282	1	0	0	0
NFKBIE	TGACACTGACCACAGTGT	860	437	3213	7	0	0
NFKBIE	TGTGACCACACACATGGT	798	316	0	0	0	0
NFKBIL1	CAACACTGACCAGTGTCA	614	427	0	0	0	9
NFKBIL1	TGACACTGACCAGTGTAC	22	40	0	0	0	0
NFKBIL1	ACACACTGACCAGTGTTG	1296	1071	1	1	0	6
NFKBIL1	CAACACTGACCAGTCAGT	226	140	0	0	0	0
NFKBIL1	CATGCAGTTGACCAGTTG	432	775	0	0	0	0
NFKBIL1	ACTGCAACTGTGCATGCA	61	84	0	0	0	146
NFKBIL2	CAACGTACACGTCATGCA	122	126	0	0	0	0
NFKBIL2	TGACGTACACGTCATGAC	33	42	0	0	0	0
NFKBIL2	ACACGTACACGTCATGTG	314	428	0	0	0	0
NFKBIL2	GTACTGTGGTACCACATG	143	162	0	0	0	0
NFKBIL2	GTTGGTTGGTACTGCAGT	187	104	0	0	0	0
NFS1	TGTGGTTGGTGTTGACAC	10	7	0	0	0	0
NFS1	ACTGGTTGGTGTTGACTG	320	673	1	0	0	9
NFS1	CATGGTTGGTGTTGTGGT	776	509	2238	6	0	1
NFS1	ACTGGTTGGTGTTGTGCA	65	90	1	0	0	8
NFS1	GTTGGTTGGTGTTGTGAC	275	113	0	0	0	0
NFS1	ACTGCATGCACACACATG	150	212	0	0	0	0
NFYA	ACACGTACGTTGGTTGTG	170	172	0	0	0	44
NFYA	GTACGTACGTTGCAGTGT	734	541	0	0	0	0
NFYA	CAACGTACGTTGCAGTCA	417	445	0	1	0	0
NFYA	TGACGTACGTTGCAGTAC	156	229	0	0	0	0
NFYA	ACACGTACGTTGCAGTTG	661	654	0	1	0	0
NFYA	GTTGCATGGTTGCACATG	85	109	0	0	0	0
NFYB	GTACGTACGTTGACCAGT	163	140	0	0	1	0
NFYB	GTACGTACGTTGACGTCA	33	264	0	0	0	0
NFYB	ACACGTACGTTGACGTAC	123	99	0	0	0	6
NFYB	CAACGTACGTTGACGTTG	94	82	0	0	0	0

NFYB	CAACGTACGTTGACCACA	16	16	0	0	0	0
NFYC	TGACGTACGTTGTGGTCA	248	269	1	0	0	0
NFYC	CAACGTACGTTGTGGTAC	92	55	1	0	0	0
NFYC	GTACGTACGTTGTGGTTG	191	267	1	0	0	0
NFYC	TGACGTACGTTGTGCAGT	124	138	0	0	0	0
NFYC	GTACGTACGTTGTGCACA	85	69	0	0	0	0
NFYC	CATGCACATGACCAGTCA	62	81	0	0	0	0
NGB	ACACTGCACAGTGTGTTG	149	235	0	0	0	0
NGB	CAACTGCACAGTGTCAGT	140	147	0	0	0	0
NGB	ACACTGCACAGTGTCACA	67	146	0	0	0	0
NGB	GTACTGCACAGTGTCAAC	23	12	0	0	0	0
NGB	TGACTGCACAGTGTCATG	133	229	1	0	0	0
NGFR	CAACTGGTCAACTGTGCA	69	74	0	0	0	0
NGFR	TGACTGGTCAACTGTGAC	70	57	0	0	0	1
NGFR	ACACTGGTCAACTGTGTG	69	106	0	0	0	0
NGFR	CAACTGGTCATGGTGTGT	233	412	0	1	0	0
NGFR	ACACTGGTCATGGTGTCA	104	98	0	0	0	0
NGFR	TGTGCACAGTTGTGTGAC	161	85	0	0	0	0
NKD1	CATGGTTGCATGACCATG	25	26	0	0	0	0
NKD1	GTTGGTTGCATGACACGT	97	113	0	0	0	0
NKD1	CATGGTTGCATGACACCA	740	552	1	0	0	0
NKD1	TGTGGTTGCATGACACAC	30	47	0	0	0	2
NKD1	ACTGGTTGCATGACACTG	336	433	0	2	0	0
NKD1	GTTGCATGCACAGTCAAC	32	54	0	0	0	0
NKD2	TGTGGTTGCATGACTGTG	504	482	2	1	1	0
NKD2	ACTGCATGCAGTGTTGTG	293	311	1	1	1	13
NKD2	CATGGTTGCATGACTGGT	189	188	0	0	0	0
NKD2	ACTGGTTGCATGACTGCA	97	168	0	0	0	0
NKD2	GTTGGTTGCATGACTGAC	32	25	0	0	0	0
NKD2	CATGGTTGCATGTGGTGT	315	452	0	0	0	0
NKIRAS1	TGACACCAGTTGACACCA	257	304	2	0	0	0
NKIRAS1	CAACACCAGTTGACACAC	32	30	0	0	0	0
NKIRAS1	GTACACCAGTTGACACTG	908	1058	0	1	0	0
NKIRAS1	TGACACCAGTTGACTGGT	365	875	2	0	0	35
NKIRAS1	GTACACCAGTTGACTGCA	135	151	0	0	0	0
NKIRAS2	ACACACCAGTTGACTGAC	94	127	0	0	0	0
NKIRAS2	CAACACCAGTTGACTGTG	391	455	1	2	0	0
NKIRAS2	TGACACCAGTTGTGGTGT	451	399	0	0	0	10
NKIRAS2	GTACACCAGTTGTGGTCA	163	313	0	0	0	0
NKIRAS2	ACACACCAGTTGTGGTAC	6	32	0	0	0	0
NKRF	ACACGTACCATGACGTTG	125	290	0	0	0	0
NKRF	CAACGTACCATGACCAGT	314	237	0	0	0	0
NKRF	ACACGTACCATGACCACA	124	63	0	0	0	0
NKRF	GTACGTACCATGACCAAC	12	7	0	0	0	0
NKRF	TGACGTACCATGACCATG	87	84	0	0	0	0
NKX2-2	TGACGTACACGTTGACTG	170	153	0	2	0	0
NKX2-2	ACACGTACACGTTGTGGT	42	268	0	0	0	0
NKX2-2	TGACGTACACGTTGTGCA	290	199	3	0	0	0
NKX2-2	CAACGTACACGTTGTGAC	29	46	0	0	0	0
NKX2-2	GTACTGTGCACACAACTG	378	444	0	0	0	0
NKX2-2	CATGCATGCACACAACGT	421	495	4	0	0	5
NKX6-1	CAACGTACACACGTACGT	221	80	0	0	1	0
NKX6-1	ACACGTACACACGTACCA	527	136	41	0	0	0
NKX6-1	GTACGTACACACGTACAC	6	8	0	0	0	0
NKX6-1	TGACGTACACACGTACTG	688	580	1	0	0	0
NKX6-1	GTTGCACAACGTTGACGT	525	170	0	0	0	0
NKX6-1	CATGCACAACGTTGACCA	553	374	0	0	0	0
NLK	ACCATGACGTTGTGCAGT	281	229	1	0	1	2
NLK	TGCATGACGTTGTGGTTG	769	704	0	0	0	0
NLK	TGCATGACGTTGTGCACA	60	49	0	0	0	0
NLK	CACATGACGTTGTGCAAC	0	5	0	0	0	0
NLK	GTCATGACGTTGTGCATG	85	130	1	0	0	0
NLN	TGACACCAGTGTCAGTGT	158	184	0	0	1	0
NLN	CAACACCAGTGTGTTGTG	17	13	0	0	0	0
NLN	GTACACCAGTGTCAGTCA	351	335	0	0	0	2
NLN	ACACACCAGTGTCAGTAC	68	63	0	0	0	0
NLN	CAACACCAGTGTCAGTTG	373	294	0	0	0	0
NLRP2	CATGGTCAACGTTGGTTG	309	771	0	1	0	0
NLRP2	GTTGGTCAACGTTGCAGT	74	140	0	0	0	0
NLRP2	CATGGTCAACGTTGCACA	160	110	1	0	0	1
NLRP2	TGTGGTCAACGTTGCAAC	189	17	0	0	0	0
NLRP2	ACTGGTCAACGTTGCATG	333	196	0	1	0	0
NLRP2	TGTGCACATGGTCATGCA	312	303	1	0	0	0
NME1	CAACGTTGTGACCATGGT	256	153	1	0	0	0
NME1	TGTGGTTGGTGTTGGTCA	117	155	0	0	0	0

NME1	CATGGTTGGTGTTGGTAC	79	84	0	0	0	0
NME1	GTTGGTTGGTGTTGGTTG	200	293	2	1	0	0
NME1	TGTGGTTGGTGTTGCAGT	259	23	0	0	0	0
NME2	TGACGTTGTGACACGTTG	126	390	1	0	0	0
NME2	ACACACTGGTTGACGTGT	831	442	3	2	0	9
NME2	GTTGGTACTGCATGACGT	233	381	1	0	0	0
NME2	CATGGTACTGCATGACCA	171	183	0	0	0	0
NME2	TGTGGTACTGCATGACAC	10	47	0	1	0	0
NMI	ACACGTTGTGGTGTTGCA	65	62	0	0	0	0
NMI	GTACGTTGTGGTGTTGAC	25	37	0	2	0	0
NMI	TGACGTTGTGGTGTTGTG	152	207	0	0	0	0
NMI	CAACGTTGTGGTCAGTGT	543	584	0	1	0	0
NMI	ACACGTTGTGGTCAGTCA	58	74	0	0	0	0
NMI	TGTGCACACATGCACATG	26	261	0	0	0	0
NMNAT1	ACACCACATGACACCAAC	48	51	0	0	0	0
NMNAT1	CAACCACATGACACCATG	291	240	0	0	0	0
NMNAT1	GTACCACATGACACACGT	166	151	1	0	0	0
NMNAT1	CAACCACATGACACACCA	61	247	0	0	0	0
NMNAT1	TGACCACATGACACACAC	46	44	0	0	0	0
NMNAT2	CAACCACATGACCAGTTG	842	834	0	0	0	0
NMNAT2	GTACCACATGACCACAGT	170	161	0	1	0	0
NMNAT2	CAACCACATGACCACACA	141	147	0	0	0	1
NMNAT2	TGACCACATGACCACAAC	47	58	1	0	0	0
NMNAT2	ACACCACATGACCACATG	100	92	0	0	0	0
NMNAT3	TGACCACATGCAACTGGT	426	298	1	0	1	0
NMNAT3	GTACCACATGCAACTGCA	113	54	0	0	0	0
NMNAT3	ACACCACATGCAACTGAC	59	81	1	0	0	0
NMNAT3	CAACCACATGCAACTGTG	156	91	0	0	0	0
NMNAT3	TGACCACATGCATGGTGT	440	471	0	0	0	0
NMNAT3	TGTGCATGGTTGACTGGT	116	144	0	0	0	0
NMU	CATGGTACCACAACTGGT	530	548	1	0	1	0
NMU	CATGGTACCACAACACCA	265	225	1	0	0	0
NMU	TGTGGTACCACAACACAC	15	15	0	0	0	0
NMU	ACTGGTACCACAACACTG	538	371	0	1	0	2
NMU	ACTGGTACCACAACTGCA	775	578	537	0	0	0
NMU	ACTGCACAACCACAGTAC	103	298	0	0	0	0
NMUR2	TGACGTCAACGTCAGTAC	71	27	0	0	0	1
NMUR2	ACACGTCAACGTCAGTTG	364	492	0	0	0	0
NMUR2	CAACGTCAACGTCACAGT	85	145	0	0	0	0
NMUR2	ACACGTCAACGTCACACA	13	18	0	0	0	0
NMUR2	TGTGCAGTACACGTACGT	212	169	0	0	0	0
NNMT	CAACCACAACTGACTGTG	150	164	0	2	0	0
NNMT	TGACCACAACTGTGGTGT	216	260	1	0	0	0
NNMT	GTACCACAACTGTGGTCA	332	357	1	1	0	1
NNMT	ACACCACAACTGTGGTAC	17	12	0	0	0	0
NNMT	CAACCACAACTGTGGTTG	88	35	0	0	0	0
NNT	TGACCAGTCACATGGTTG	662	2330	4	2	1	11
NNT	ACACCAGTCACATGCAGT	201	266	0	0	0	0
NNT	TGACCAGTCACATGCACA	96	110	1	0	0	0
NNT	CAACCAGTCACATGCAAC	21	10	0	0	0	0
NNT	GTACCAGTCACATGCATG	1090	801	2	0	0	1
NOD1	TGTGGTACACGTTGTGGT	383	562	0	1	0	0
NOD1	GTTGGTACACGTTGTGCA	152	180	0	0	0	8
NOD1	ACTGGTACACGTTGTGAC	33	38	0	0	0	0
NOD1	CATGGTACACGTTGTGTG	131	613	0	0	0	10
NOD1	ACTGGTACACCAGTGTGT	387	398	1	0	0	0
NOD1	TGTGCACAACGTACTGCA	139	226	0	0	0	1
NOD2	TGTGCACATGACCAACCA	830	338	0	0	1	0
NOD2	TGACACTGACACTGTGCA	82	123	0	0	0	4
NOD2	CAACACTGACACTGTGAC	53	30	732	3	0	0
NOD2	GTACACTGACACTGTGTG	434	324	1	0	0	0
NOD2	TGACACTGACTGGTGTGT	432	679	1	1	0	3
NOD2	GTACACTGACTGGTGTCA	123	69	1	0	0	0
NODAL	TGACTGGTTGGTTGTGAC	66	46	0	0	0	0
NODAL	ACACTGGTTGGTTGTGTG	21	37	0	0	0	0
NODAL	TGACTGGTTGCAGTGTGT	224	252	0	0	0	1
NODAL	GTACTGGTTGCAGTGTCA	198	187	0	0	0	0
NODAL	ACACTGGTTGCAGTGTAC	47	63	0	0	0	0
NOG	CAACTGGTACTGACACTG	347	979	4195	1	1	1
NOG	GTACTGGTACTGACACCA	205	256	0	0	0	0
NOG	ACACTGGTACTGACACAC	63	85	0	0	0	0
NOG	GTACTGGTACTGACTGGT	163	144	0	0	0	0
NOG	CAACTGGTACTGACTGCA	111	93	0	0	0	0
NOLA2	ACACTGACTGTGGTCACA	167	215	0	0	0	0
NOLA2	GTACTGACTGTGGTCAAC	36	72	10	0	0	0

NOLA2	GTTGCAGTCAGTCATGGT	262	208	0	0	0	1
NOLA2	CATGCAGTCAGTCATGCA	243	153	1	0	0	1
NOLA2	TGTGCAGTCAGTCATGAC	28	12	0	0	0	0
NOS1	CAACACGTCACACAACCA	540	408	0	0	1	0
NOS1	ACACACGTCACACAACTG	593	597	0	36	1	1
NOS1	TGACACGTCACACAACAC	98	76	0	0	0	0
NOS1	CAACACGTCACACATGGT	950	654	3	1	0	1
NOS1	ACTGCAGTTGGTCACAAC	22	66	0	0	0	0
NOS1	ACTGCACATGCAACCAGT	92	102	1	0	0	0
NOS1AP	TGACACGTCATGCAGTTG	1018	790	1	3	1	2
NOS1AP	TGTGCATGGTTGACACCA	234	533	0	0	1	0
NOS1AP	ACACACGTCATGCACAGT	227	311	2	2	0	2099
NOS1AP	TGACACGTCATGCACACA	354	207	0	48	0	0
NOS1AP	CAACACGTCATGCACAAC	38	33	0	0	0	0
NOS1AP	GTACACGTCATGCACATG	213	251	2	0	0	0
NOS2A	CACATGACTGTGTGGTTG	598	681	0	0	0	6
NOS2A	GTCATGACTGTGTGCAGT	134	205	0	0	0	0
NOS2A	CACATGACTGTGTGCACA	206	395	0	1	0	21
NOS2A	TGCATGACTGTGTGCAAC	5	8	0	0	0	0
NOS2A	ACCATGACTGTGTGCATG	350	238	0	0	0	1
NOS2A	TGTGCAACTGGTTGACAC	32	47	0	0	0	0
NOS3	GTACACGTCACATGGTGT	310	294	51	35	33	80
NOS3	CAACACGTCACATGGTCA	545	558	4	0	0	24
NOS3	TGACACGTCACATGGTAC	29	50	0	0	0	0
NOS3	ACACACGTCACATGGTTG	627	430	1	0	0	0
NOS3	ACTGACGTCAACCAGTCA	408	420	0	1	0	0
NOTCH1	TGCATGACACTGACTGAC	48	49	0	0	0	0
NOTCH1	ACCATGACACTGACTGTG	171	637	0	0	0	0
NOTCH1	GTCATGACACTGTGGTGT	964	447	1	0	0	0
NOTCH1	CACATGACACTGTGGTCA	269	290	0	0	0	1
NOTCH1	TGCATGACACTGTGGTAC	238	88	0	0	0	0
NOTCH2	CACATGTGCATGGTACCA	182	497	0	3	5	0
NOTCH2	GTCATGTGCATGGTCACA	102	41	0	0	0	0
NOTCH2	ACCATGTGCATGGTCAAC	20	36	1	0	0	73
NOTCH2	CACATGTGCATGGTCATG	228	827	3	2	0	5
NOTCH2	GTCATGTGCATGGTACGT	152	188	1	0	0	0
NOTCH2	ACTGCAACTGGTTGACTG	245	372	0	1	0	1
NOTCH3	CAACGTTGGTTGTGTGAC	260	111	1	2	0	0
NOTCH3	GTACGTTGGTTGTGTGTG	767	589	1	3	0	16
NOTCH3	CAACGTTGCAGTGTGTGT	1473	450	0	2	0	1
NOTCH3	ACACGTTGCAGTGTGTCA	382	624	1	18	0	1
NOTCH3	GTACGTTGCAGTGTGTAC	139	327	1	1	0	0
NOTCH3	CATGCACACAACACGTAC	36	47	0	0	0	0
NOTCH4	GTACGTTGCAGTGTCATG	53	56	0	0	0	0
NOTCH4	TGACGTTGCAGTGTACGT	274	346	0	0	0	0
NOTCH4	GTACGTTGCAGTGTACCA	115	91	0	0	0	0
NOTCH4	ACACGTTGCAGTGTACAC	45	33	0	0	0	0
NOTCH4	TGTGACGTACTGTGACAC	21	34	0	0	0	274
NOV	TGTGGTGTCAACCAACTG	877	643	1	0	0	0
NOV	ACTGGTGTCAACCATGGT	774	1040	1	0	0	1
NOV	TGTGGTGTCAACCATGCA	462	523	2	0	0	2
NOV	CATGGTGTCAACCATGAC	5	3	0	0	0	0
NOV	GTTGGTGTCAACCATGTG	170	119	0	0	0	0
NOV	TGTGCAACCACAACACGT	20	27	0	0	0	0
NOX1	GTACACGTTGGTGTCATG	28	36	0	0	0	0
NOX1	TGACACGTTGGTGTACGT	3	16	0	0	0	0
NOX1	GTACACGTTGGTGTACCA	388	154	0	0	0	0
NOX1	ACACACGTTGGTGTACAC	15	17	0	0	0	0
NOX1	CAACACGTTGGTGTACTG	167	167	1	0	0	0
NOX1	ACTGCAACACCAACGTAC	39	52	0	0	0	0
NOX4	ACACACGTTGGTGTTGTG	233	254	4	0	1	14
NOX4	GTACACGTTGGTGTTGGT	145	147	0	0	0	0
NOX4	CAACACGTTGGTGTTGCA	61	125	1	0	0	0
NOX4	TGACACGTTGGTGTTGAC	37	54	0	0	0	1
NOX4	GTACACGTTGGTCAGTGT	441	465	1	0	0	0
NOX4	GTTGCAACCAACACCAGT	155	141	0	0	0	0
NOXO1	CAACACGTTGGTCAGTCA	470	357	0	1	0	4
NOXO1	TGACACGTTGGTCAGTAC	85	217	1	0	0	0
NOXO1	ACACACGTTGGTCAGTTG	617	334	0	1	0	0
NOXO1	GTTGCAGTTGGTACGTAC	26	38	0	0	0	0
NOXO1	ACTGCATGTGTGACCAAC	50	49	0	0	0	0
NP	TGACTGTGTGACGTTGGT	492	357	0	0	0	0
NP	GTACTGTGTGACGTTGCA	11	28	0	0	0	0
NP	ACACTGTGTGACGTTGAC	19	20	0	0	0	0
NP	CAACTGTGTGACGTTGTG	388	234	0	0	0	0

NP	CATGCAGTTGTGTGTGGT	157	125	0	0	0	1
NP	GTTGCACACAACACGTTG	313	296	0	1	0	0
NPAS2	TGACGTTGTGGTCATGAC	149	45	0	0	1	0
NPAS2	GTACGTTGTGGTCATGGT	115	166	0	0	0	0
NPAS2	CAACGTTGTGGTCATGCA	225	200	0	0	0	0
NPAS2	ACACGTTGTGGTCATGTG	652	313	0	0	0	0
NPAS2	GTACGTTGTGGTACGTGT	182	289	1	0	0	0
NPC1	GTCATGTGACACGTCAGT	236	277	0	0	0	119
NPC1	CACATGTGACACGTCACA	116	43	0	0	0	0
NPC1	TGCATGTGACACGTCAAC	19	6	0	0	0	0
NPC1	ACCATGTGACACGTCATG	83	143	1	0	0	0
NPC1	CACATGTGACACGTACGT	433	376	2	0	0	2
NPEPPS	ACTGCAACGTCATGCATG	179	131	0	1	0	0
NPEPPS	ACACCAGTTGTGGTTGAC	75	71	0	0	0	0
NPEPPS	TGACACACACTGTGTGCA	87	105	0	0	0	0
NPEPPS	ACACTGTGGTCATGACAC	111	66	0	0	0	0
NPEPPS	CAACTGTGGTCATGACTG	238	309	1	0	0	0
NPEPPS	GTACTGTGGTCATGTGGT	158	284	0	0	0	0
NPM1	TGACTGCATGTGACGTTG	342	905	2	1	1	0
NPM1	TGTGCAGTCAGTTGACTG	304	154	1	0	1	0
NPM1	GTACTGCATGTGACGTAC	48	75	1	0	0	0
NPM1	ACACTGCATGTGACCAGT	619	577	1	0	0	0
NPM1	ACTGCAGTCAGTTGTGGT	658	751	0	2	0	0
NPM2	GTTGGTACGTTGTGGTCA	273	171	1	0	2	1
NPM2	ACTGGTACGTTGACTGAC	31	21	0	0	0	0
NPM2	CATGGTACGTTGACTGTG	334	306	1	1	0	0
NPM2	TGTGGTACGTTGTGGTGT	929	867	0	1	0	2
NPM2	ACTGGTACGTTGTGGTAC	161	56	0	0	0	0
NPPA	GTTGGTACCACACAACAC	70	44	0	0	0	0
NPPA	TGTGGTACCACACAACTG	66	127	0	0	0	0
NPPA	ACTGGTACCACACATGGT	526	695	0	0	0	0
NPPA	TGTGGTACCACACATGCA	95	111	0	0	0	0
NPPA	CATGGTACCACACATGAC	64	70	0	0	0	0
NPPB	CATGGTTGTGACTGACTG	1064	746	3	1	1	0
NPPB	GTTGGTTGTGACTGCATG	357	418	0	2	0	0
NPPB	TGTGGTTGTGACTGACGT	227	210	0	1	0	0
NPPB	GTTGGTTGTGACTGACCA	91	329	0	0	0	2
NPPB	ACTGGTTGTGACTGACAC	70	40	0	0	0	0
NPR1	ACACGTGTACGTGTTGAC	6	30	0	0	0	0
NPR1	CAACGTGTACGTGTTGTG	162	157	0	0	0	0
NPR1	TGACGTGTACGTCAGTGT	120	163	4	0	0	0
NPR1	GTACGTGTACGTCAGTCA	131	420	0	0	0	0
NPR1	ACACGTGTACGTCAGTAC	18	24	0	0	0	2
NPR2	CACATGCACATGCAGTTG	578	590	1	0	2	2
NPR2	GTCATGCACATGCAGTCA	830	614	0	243	0	13
NPR2	ACCATGCACATGCAGTAC	42	30	0	0	0	0
NPR2	GTCATGCACATGCACAGT	13	2	0	0	0	0
NPR2	TGCATGCATGTGGTCACA	56	81	0	0	0	0
NPR3	GTACGTCAACGTCACAAC	98	23	0	0	0	0
NPR3	TGACGTCAACGTCACATG	199	212	0	0	0	0
NPR3	ACACGTCAACGTCAACGT	582	438	2	1	0	0
NPR3	TGACGTCAACGTCAACCA	38	42	0	0	0	2
NPR3	CAACGTCAACGTCAACAC	62	76	0	0	0	0
NPTX1	TGACTGCACAGTGTTGGT	214	248	0	1	0	0
NPTX1	GTACTGCACAGTGTTGCA	437	112	1	0	0	0
NPTX1	ACACTGCACAGTGTTGAC	23	140	0	0	0	0
NPTX1	CAACTGCACAGTGTTGTG	717	535	0	0	0	1
NPTX1	GTTGACCATGGTCACACA	55	61	0	0	0	0
NPY	GTACGTCAACGTCAACTG	550	426	0	0	1	0
NPY	TGTGCATGGTTGTGTGCA	215	388	0	0	1	0
NPY	TGACGTCAACGTCATGGT	573	335	1	1	0	0
NPY	GTACGTCAACGTCATGCA	143	235	3	1	0	54
NPY	CATGCAGTACCATGTGTG	554	593	1	1	0	0
NPY	CATGCATGGTTGTGTGAC	8	26	0	0	0	0
NPY1R	ACACGTCAACGTCATGAC	55	212	1	0	0	0
NPY1R	CAACGTCAACGTCATGTG	306	370	1	0	0	0
NPY1R	TGACGTCAACGTACGTGT	861	955	21	0	0	0
NPY1R	GTACGTCAACGTACGTCA	440	384	1	0	0	0
NPY1R	ACACGTCAACGTACGTAC	14	7	0	0	0	0
NPY2R	CAACGTCAACGTACCACA	147	186	3	2	4	7
NPY2R	CAACGTCAACGTACGTTG	119	96	0	0	0	0
NPY2R	GTACGTCAACGTACCAGT	136	120	1	0	0	0
NPY2R	TGACGTCAACGTACCAAC	35	9	0	0	0	0
NPY2R	CATGCAGTACACGTGTGT	347	273	1	0	0	0
NPY2R	ACTGCATGGTGTGTTGAC	15	14	0	0	0	0

NQO1	ACCATGACTGTGTGACCA	386	346	0	0	2	2
NQO1	CACATGACTGTGTGACGT	173	72	0	0	1	0
NQO1	ACCATGACTGTGTGTGGT	510	419	1	0	1	3
NQO1	GTCATGACTGTGTGACAC	22	14	0	0	0	0
NQO1	TGCATGACTGTGTGACTG	376	314	0	0	0	0
NQO2	GTACCAACACCACAGTCA	765	173	1	0	1	0
NQO2	ACACCAACACCACAGTAC	125	27	0	0	0	0
NQO2	CAACCAACACCACAGTTG	2506	1497	3	1	0	1
NQO2	GTACCAACACCACACAGT	157	255	0	1	0	54
NQO2	CAACCAACACCACACACA	145	123	1	0	0	0
NR0B1	GTACGTTGTGGTACCAAC	21	66	0	0	0	0
NR0B1	TGACGTTGTGGTACCATG	150	105	0	0	0	4
NR0B1	ACACGTTGTGGTACACGT	351	140	0	0	0	1
NR0B1	TGACGTTGTGGTACACCA	316	376	0	0	0	1
NR0B1	CAACGTTGTGGTACACAC	1	11	0	0	0	0
NR0B2	TGACCACACAGTGTGTCA	324	362	1	0	0	0
NR0B2	CAACCACACAGTGTGTAC	60	19	0	0	0	0
NR0B2	GTACCACACAGTGTGTTG	495	350	4	2	0	0
NR0B2	TGACCACACAGTGTCAGT	67	65	0	0	0	0
NR0B2	GTACCACACAGTGTCACA	148	141	0	0	0	0
NR0B2	ACTGCACATGTGGTTGCA	228	242	0	7	0	0
NR1D1	GTACGTTGTGGTACACTG	53	108	0	0	0	0
NR1D1	TGACGTTGTGGTACTGGT	18	41	0	0	0	0
NR1D1	GTACGTTGTGGTACTGCA	49	38	0	0	0	0
NR1D1	ACACGTTGTGGTACTGAC	11	53	0	0	0	0
NR1D1	CAACGTTGTGGTACTGTG	313	196	0	0	0	0
NR1D2	ACACGTTGTGGTTGACCA	136	127	0	0	1	0
NR1D2	CAACGTTGTGGTTGCACA	74	76	1	0	0	0
NR1D2	TGACGTTGTGGTTGCAAC	11	28	0	0	0	0
NR1D2	ACACGTTGTGGTTGCATG	180	183	0	0	0	0
NR1D2	CAACGTTGTGGTTGACGT	158	119	0	0	0	0
NR1H2	GTACGTTGTGGTTGTGTG	652	567	0	1	1	0
NR1H2	CAACGTTGTGCAGTGTGT	1090	1067	1	1	1	0
NR1H2	ACACGTTGTGCAGTGTCA	498	477	14	2	1	0
NR1H2	CAACGTTGTGGTTGTGAC	42	73	0	1	0	0
NR1H2	ACTGCAGTGTACTGCATG	74	108	0	0	0	0
NR1H3	GTACGTTGTGCAGTCATG	125	71	0	0	0	24
NR1H3	TGACGTTGTGCAGTACGT	45	112	0	0	0	0
NR1H3	GTACGTTGTGCAGTACCA	529	419	0	1	0	0
NR1H3	ACACGTTGTGCAGTACAC	51	69	0	0	0	0
NR1H3	ACTGCAGTACTGGTGTGT	493	257	0	0	0	8
NR1H3	TGTGCACAACGTGTTGAC	95	85	0	0	0	0
NR1H4	GTACGTTGACCATGCAGT	241	326	0	0	0	0
NR1H4	CAACGTTGACCATGCACA	148	228	0	0	0	0
NR1H4	TGACGTTGACCATGCAAC	7	16	0	0	0	0
NR1H4	ACACGTTGACCATGCATG	145	262	0	0	0	0
NR1H4	CAACGTTGACCATGACGT	535	262	1	0	0	0
NR1H4	CATGCACACATGACTGAC	45	41	0	0	0	0
NR1I2	CAACGTTGACCATGTGAC	27	27	0	0	0	0
NR1I2	GTACGTTGACCATGTGTG	479	382	0	0	0	0
NR1I2	GTACGTTGACACGTGTGT	811	850	3	0	0	0
NR1I2	CAACGTTGACACGTGTCA	135	138	0	0	0	1
NR1I2	TGACGTTGACACGTGTAC	199	45	0	0	0	0
NR1I2	CATGCAACGTCAGTTGGT	447	247	1	1	0	0
NR1I3	ACACGTTGACACGTACGT	38	97	1	0	0	0
NR1I3	TGACGTTGACACGTACCA	67	85	0	0	0	32
NR1I3	CAACGTTGACACGTACAC	105	373	0	0	0	0
NR1I3	GTACGTTGACACGTACTG	267	159	0	0	0	0
NR1I3	TGACGTTGACACGTTGGT	234	290	1	1	0	0
NR2C2	TGACGTTGACACCACAAC	21	31	0	0	1	0
NR2C2	ACACGTTGACACCAGTAC	42	21	0	0	0	0
NR2C2	CAACGTTGACACCAGTTG	595	797	0	2	0	1
NR2C2	GTACGTTGACACCACAGT	403	487	0	0	0	0
NR2C2	CAACGTTGACACCACACA	34	30	0	0	0	1
NR2E1	ACACGTTGACACCAACCA	520	425	1	0	0	1
NR2E1	GTACGTTGACACCAACAC	225	31	0	1	0	0
NR2E1	TGACGTTGACACCAACTG	322	352	0	1	0	0
NR2E1	ACACGTTGACACCATGGT	626	527	0	1	0	0
NR2E1	CAACCAGTCAACTGGTTG	315	97	0	0	0	0
NR2E1	ACTGCAACACGTGTTGCA	8	24	0	0	0	0
NR2F1	TGACGTTGACACACTGTG	262	432	2	0	1	0
NR2F1	CAACGTTGACACTGGTGT	671	931	0	0	1	15
NR2F1	ACACGTTGACACACTGCA	337	187	2	0	0	0
NR2F1	GTACGTTGACACACTGAC	11	31	0	0	0	0
NR2F1	TGTGCAGTACACTGTGTG	663	830	1	0	0	0

NR2F2	TGCATGTGACGTCACACA	49	50	0	0	0	0
NR2F2	CACATGTGACGTCACAAC	10	20	0	0	0	0
NR2F2	GTCATGTGACGTCACATG	56	63	0	0	0	0
NR2F2	TGCATGTGACGTCAACGT	691	390	0	1	0	0
NR2F2	GTCATGTGACGTCAACCA	233	168	23	159	0	0
NR2F2	CATGCATGGTTGTGCACA	208	95	0	0	0	0
NR2F6	ACACCACACAGTGTTGCA	497	139	0	0	1	0
NR2F6	TGACCACACAGTGTACAC	70	46	0	0	0	0
NR2F6	ACACCACACAGTGTACTG	192	305	0	0	0	0
NR2F6	CAACCACACAGTGTTGGT	159	281	0	0	0	0
NR2F6	GTACCACACAGTGTTGAC	91	125	0	0	0	0
NR3C1	CAACGTACTGACTGCACA	185	177	2	0	1	0
NR3C1	ACACGTACTGACTGACCA	267	162	0	0	1	0
NR3C1	TGACGTACTGACTGCAAC	9	14	0	0	0	0
NR3C1	ACACGTACTGACTGCATG	48	64	0	0	0	1
NR3C1	CAACGTACTGACTGACGT	192	326	0	0	0	259
NR3C2	GTACGTACTGTGCAGTTG	265	329	10	9	1	11
NR3C2	CAACGTACTGTGCAGTAC	40	38	0	0	0	0
NR3C2	TGACGTACTGTGCACAGT	168	205	0	0	0	0
NR3C2	GTACGTACTGTGCACACA	24	25	0	0	0	0
NR3C2	ACACGTACTGTGCACAAC	72	54	0	0	0	0
NR3C2	TGTGCACAGTTGTGGTTG	307	334	0	0	0	0
NR4A1	TGACGTACTGTGACTGAC	134	58	0	0	1	0
NR4A1	CAACGTACTGTGACTGCA	364	825	1	0	0	4
NR4A1	ACACGTACTGTGACTGTG	246	190	0	0	0	0
NR4A1	GTACGTACTGTGTGGTGT	973	1018	2	1	0	0
NR4A1	TGTGCATGGTGTACTGTG	230	288	3	0	0	0
NR4A1	CATGCATGGTGTTGGTGT	41	146	0	0	0	0
NR4A2	GTACGTTGGTGTGTGTGT	456	683	2	0	5	0
NR4A2	CAACGTTGGTGTGTGTCA	428	411	1	0	0	0
NR4A2	TGACGTTGGTGTGTGTAC	107	51	2	0	0	0
NR4A2	GTACCAGTCAACTGGTCA	505	333	2	1	0	2
NR4A2	ACACCAGTCAACTGGTAC	17	45	0	0	0	0
NR4A2	ACTGCATGGTGTTGGTCA	788	304	1	2	0	0
NR4A3	GTACGTTGGTGTCAGTCA	259	293	0	0	0	0
NR4A3	ACACGTTGGTGTCAGTAC	200	38	1	0	0	0
NR4A3	CAACGTTGGTGTCAGTTG	1064	2024	4	4	0	1
NR4A3	GTACGTTGGTGTCACAGT	340	583	0	0	0	0
NR4A3	CAACGTTGGTGTCACACA	114	263	0	0	0	0
NR4A3	GTTGCATGGTGTTGGTAC	42	222	1	0	0	0
NR5A1	ACACGTTGGTGTACGTGT	98	410	0	0	0	0
NR5A1	TGACGTTGGTGTACGTCA	95	78	0	0	0	0
NR5A1	CAACGTTGGTGTACGTAC	307	158	1	0	0	0
NR5A1	CATGCAGTACACTGCATG	33	44	0	0	0	0
NR5A1	GTTGCAGTACACTGACGT	442	67	0	0	0	0
NR5A1	CATGCAACTGCAACTGGT	147	236	0	0	0	12
NR5A2	CAACGTTGGTGTTGGTGT	183	179	1	1	1	2
NR5A2	ACACGTTGGTGTACTGCA	69	35	0	0	0	0
NR5A2	GTACGTTGGTGTACTGAC	26	18	0	0	0	0
NR5A2	TGACGTTGGTGTACTGTG	43	207	0	0	0	0
NR5A2	ACTGCAGTACACTGCAAC	4	4	0	0	0	0
NR6A1	CAACGTTGGTCACATGGT	245	331	0	1	2	0
NR6A1	ACACGTTGGTCACATGCA	199	168	1	0	0	8
NR6A1	GTACGTTGGTCACATGAC	78	171	0	0	0	0
NR6A1	TGACGTTGGTCACATGTG	455	318	0	0	0	0
NR6A1	GTTGCAGTACACTGGTTG	416	445	0	3	0	0
NRAS	ACACCAGTTGTGTGCAGT	43	25	0	0	0	0
NRAS	TGACCAGTTGTGTGCACA	70	96	0	1	0	0
NRAS	CAACCAGTTGTGTGCAAC	30	34	0	1	0	0
NRAS	GTACCAGTTGTGTGCATG	73	53	1	0	0	0
NRAS	TGACCAGTTGTGTGACGT	318	204	0	0	0	0
NRAS	GTTGCACAACCATGGTAC	35	49	0	0	0	0
NRCAM	TGTGGTCACACAGTTGCA	230	142	0	0	2	0
NRCAM	ACTGGTCACACAGTTGGT	151	196	0	0	0	0
NRCAM	CATGGTCACACAGTTGAC	46	17	0	0	0	0
NRCAM	GTTGGTCACACAGTTGTG	740	874	1	0	0	22
NRCAM	ACTGACCAACGTTGGTCA	173	252	1	0	0	0
NRF1	ACACGTACACGTACCACA	98	55	0	0	0	1
NRF1	GTACGTACACGTACCAAC	12	5	0	0	0	0
NRF1	TGACGTACACGTACCATG	187	215	3	0	0	0
NRF1	ACACGTACACGTACACGT	50	51	0	0	0	0
NRF1	TGTGACCATGACGTCATG	254	162	0	0	0	0
NRG1	GTACTGGTACGTACACAC	8	18	0	0	0	0
NRG1	TGACTGGTACGTACACTG	652	362	0	0	0	0
NRG1	ACACTGGTACGTACTGGT	385	51	0	0	0	5

NRG1	TGACTGGTACGTACTGCA	465	137	0	0	0	0
NRG1	GTTGCAGTTGACCAGTCA	90	176	1	2	0	0
NRG2	ACTGCACAACTGCAACTG	408	406	1	85	21	0
NRG2	ACACTGGTTGACTGTGCA	83	83	0	0	0	0
NRG2	GTACTGGTTGACTGTGAC	267	84	0	1	0	1
NRG2	TGACTGGTTGACTGTGTG	476	955	2	0	0	0
NRG2	ACACTGGTTGTGGTGTGT	610	315	0	1	0	0
NRG2	CATGCACAACTGCATGGT	129	185	0	1	0	0
NRG3	GTACTGACTGCAACCAGT	200	186	1	0	0	0
NRG3	GTTGGTACACTGTGCACA	80	89	0	0	0	0
NRG3	ACTGGTACACTGTGCAAC	24	23	0	0	0	0
NRG3	CATGACCAGTACTGTGAC	152	76	0	0	0	0
NRG3	GTTGACCAGTACTGTGTG	183	158	0	1	0	0
NRIP1	CAACGTTGGTCAACACTG	445	490	0	0	1	1
NRIP1	GTACGTTGGTCAACACCA	19	4	0	0	0	0
NRIP1	ACACGTTGGTCAACACAC	94	57	0	0	0	0
NRIP1	GTACGTTGGTCAACTGGT	65	83	0	0	0	0
NRIP1	CAACGTTGGTCAACTGCA	327	262	0	0	0	0
NRP1	ACACTGACCAGTGTGTCA	213	334	0	0	1	0
NRP1	TGACTGACGTTGTGTGCA	256	156	0	0	0	0
NRP1	CAACTGACGTTGTGTGAC	165	42	0	0	0	0
NRP1	GTACTGACGTTGTGTGTG	173	113	0	0	0	3
NRP1	CAACTGACCAGTGTGTGT	823	754	2	0	0	0
NSD1	ACACTGCAACTGCAACCA	120	172	1	4	1	4521
NSD1	CAACTGCAACTGCAACGT	291	190	1	1	0	1
NSD1	GTACTGCAACTGCAACAC	44	47	0	0	0	0
NSD1	TGACTGCAACTGCAACTG	1903	1100	3	1	0	2
NSD1	ACTGGTTGTGACGTGTTG	332	303	1	0	0	0
NSD1	TGTGCATGGTGTGTGTAC	84	96	0	1	0	1
NSDHL	TGACCAGTCAGTTGGTCA	230	302	1	0	1	0
NSDHL	TGACCAGTCAGTACTGCA	185	134	0	0	0	0
NSDHL	CAACCAGTCAGTACTGAC	162	92	0	0	0	0
NSDHL	GTACCAGTCAGTACTGTG	155	140	1	0	0	0
NSDHL	ACACCAGTCAGTTGGTGT	869	731	1	1	0	0
NSDHL	GTTGCACATGGTGTACTG	360	488	0	0	0	0
NSF	TGTGACCATGGTCAGTCA	88	172	0	0	1	0
NSF	ACACCAGTACCAACGTTG	614	737	1	3	0	1
NSF	GTTGACCATGGTGTTGTG	1101	970	5	1	0	1
NSF	ACTGACCATGGTCAGTGT	315	352	0	0	0	1
NSF	CATGACCATGGTCAGTAC	43	36	0	0	0	1
NSMAF	GTTGGTCATGGTACACAC	72	33	0	0	0	0
NSMAF	TGTGGTCATGGTACACTG	181	775	0	0	0	0
NSMAF	ACTGGTCATGGTACTGGT	97	129	0	0	0	0
NSMAF	TGTGGTCATGGTACTGCA	233	129	0	0	0	0
NSMAF	CATGGTCATGGTACTGAC	63	66	0	0	0	0
NSMAF	GTTGCATGCAGTCAGTGT	453	556	0	1	0	0
NSMCE2	TGTGGTTGACACGTCACA	140	167	0	0	0	0
NSMCE2	CATGGTTGACACGTCAAC	14	24	0	0	0	0
NSMCE2	GTTGGTTGACACGTCATG	98	90	0	0	0	0
NSMCE2	TGTGGTTGACACGTACGT	70	56	0	0	0	0
NSMCE2	GTTGGTTGACACGTACCA	555	524	17	3	0	18
NSMCE2	CATGCATGGTGTGTTGTG	314	313	2	0	0	1
NSUN2	ACTGGTTGCATGTGGTCA	318	382	0	1	0	3
NSUN2	GTTGGTTGCATGTGGTAC	42	40	0	0	0	0
NSUN2	TGTGGTTGCATGTGGTTG	412	493	0	0	0	0
NSUN2	ACTGGTTGCATGTGCAGT	385	320	0	0	0	0
NSUN2	TGTGGTTGCATGTGCACA	36	50	0	0	0	0
NT5C	CAACGTTGGTACCAACAC	11	10	0	0	0	0
NT5C	GTACGTTGGTACCAACTG	216	225	1	1	0	1
NT5C	TGACGTTGGTACCATGGT	232	330	2	0	0	0
NT5C	GTACGTTGGTACCATGCA	80	109	0	0	0	0
NT5C	ACACGTTGGTACCATGAC	22	24	0	0	0	0
NT5C	TGTGCACAACTGGTCAAC	12	100	0	0	0	0
NT5C1A	CAACACACACGTGTCAGT	44	33	1	0	0	0
NT5C1A	ACACACACACGTGTCACA	30	26	0	0	0	0
NT5C1A	GTACACACACGTGTCAAC	19	13	0	0	0	0
NT5C1A	TGACACACACGTGTCATG	75	168	1	1	0	0
NT5C1A	ACACACACACGTGTACGT	109	124	0	0	0	0
NT5C1A	TGTGCAACGTGTACTGAC	19	78	0	1	0	0
NT5C1B	TGACACACCAGTGTACAC	18	20	0	0	0	0
NT5C1B	ACACACACCAGTGTACTG	12	10	0	0	0	0
NT5C1B	CAACACACCAGTGTTGGT	126	209	0	1	0	0
NT5C1B	ACACACACCAGTGTTGCA	116	180	0	0	0	0
NT5C1B	GTACACACCAGTGTTGAC	34	31	0	0	0	0
NT5C2	ACTGCAACCAACACGTAC	101	85	3	1	12	20

NT5C2	ACACACACGTTGTGGTTG	224	159	0	0	0	0
NT5C2	CAACACACGTTGTGCAGT	986	560	0	1	0	38
NT5C2	ACACACACGTTGTGCACA	72	171	0	0	0	0
NT5C2	GTACACACGTTGTGCAAC	50	65	0	0	0	0
NT5C2	TGACACACGTTGTGCATG	136	129	0	1	0	0
NT5C3	CAACGTTGGTACACTGAC	15	20	0	0	0	0
NT5C3	GTACGTTGGTACACTGTG	50	53	0	0	0	0
NT5C3	ACACGTTGGTACTGGTGT	360	349	1	1	0	0
NT5C3	TGACGTTGGTACTGGTCA	32	34	0	0	0	0
NT5C3	CAACGTTGGTACTGGTAC	72	69	1	0	0	0
NT5E	GTACACCACATGACACCA	476	268	0	0	1	10
NT5E	CAACACCACATGACCAAC	13	8	0	0	0	0
NT5E	GTACACCACATGACCATG	127	129	0	0	0	1
NT5E	TGACACCACATGACACGT	118	104	0	0	0	0
NT5E	ACACACCACATGACACAC	45	54	0	0	0	0
NT5E	ACTGCACAGTTGTGTGTG	349	718	1	1	0	0
NT5M	CAACACACCATGCAGTTG	792	577	2	1	0	0
NT5M	GTACACACCATGCACAGT	351	264	0	0	0	0
NT5M	CAACACACCATGCACACA	230	188	0	0	0	0
NT5M	TGACACACCATGCACAAC	42	44	0	0	0	1
NT5M	ACACACACCATGCACATG	331	239	0	0	0	0
NTAN1	TGACACCAACTGTGTGTG	538	419	12	1	3	13
NTAN1	CAACACCAACTGTGTGGT	181	258	0	0	0	0
NTAN1	ACACACCAACTGTGTGCA	163	314	0	0	0	0
NTAN1	GTACACCAACTGTGTGAC	89	71	0	0	0	0
NTAN1	ACTGGTCACAACCAGTTG	355	379	0	0	0	0
NTF3	CAACTGGTTGACTGACCA	215	373	0	0	1	2
NTF3	GTACTGGTTGACTGACGT	268	213	3	0	0	0
NTF3	TGACTGGTTGACTGACAC	63	28	0	0	0	0
NTF3	ACACTGGTTGACTGACTG	356	358	0	0	0	1
NTF3	CAACTGGTTGACTGTGGT	457	430	0	0	0	0
NTHL1	ACACGTGTACTGCATGTG	375	318	1	1	1	1
NTHL1	GTACGTGTACTGACGTGT	260	173	0	0	1	8
NTHL1	GTTGCAGTACCACACACA	295	233	0	0	1	0
NTHL1	TGACGTGTACTGCATGAC	24	25	0	0	0	0
NTHL1	CAACGTGTACTGACGTCA	266	271	0	1	0	0
NTRK1	TGCATGACGTTGGTTGGT	73	125	0	0	0	0
NTRK1	GTCATGACGTTGGTTGCA	474	222	0	0	0	0
NTRK1	ACCATGACGTTGGTTGAC	3	6	0	0	0	0
NTRK1	CACATGACGTTGGTTGTG	268	172	0	0	0	11
NTRK1	TGCATGACGTTGCAGTGT	648	608	0	1	0	0
NTRK2	ACCATGACCAGTTGACGT	317	254	0	1	0	0
NTRK2	ACTGGTACTGACACACAC	42	44	0	0	0	3
NTRK2	CATGGTACTGACACACTG	115	485	2	0	0	0
NTRK2	GTTGGTACTGACACTGGT	88	110	0	0	0	0
NTRK2	TGTGCAGTGTTGCATGTG	781	1000	1	1317	0	0
NTSR1	TGACGTCAACGTACACTG	816	432	0	13	11	118
NTSR1	GTACGTCAACGTACACAC	46	40	0	0	1	0
NTSR1	ACACGTCAACGTACCATG	36	46	0	0	0	0
NTSR1	CAACGTCAACGTACACGT	16	33	0	0	0	0
NTSR1	ACACGTCAACGTACACCA	134	131	0	0	0	1
NUDC	TGTGGTCATGTGACACGT	387	411	1	1	1	0
NUDC	CATGGTCATGTGACCAAC	22	43	0	0	0	0
NUDC	GTTGGTCATGTGACCATG	19	13	0	0	0	0
NUDC	GTTGGTCATGTGACACCA	213	197	0	0	0	0
NUDC	ACTGGTCATGTGACACAC	31	37	0	0	0	0
NUDC	CATGCATGCAGTCAGTCA	707	374	1	1	0	0
NUDT1	TGACACCATGACTGGTAC	27	34	0	0	0	0
NUDT1	ACACACCATGACTGGTTG	1647	1289	14	1	0	2
NUDT1	CAACACCATGACTGCAGT	190	207	0	0	0	1
NUDT1	ACACACCATGACTGCACA	81	55	0	0	0	0
NUDT1	ACTGCAGTCAACTGTGAC	57	51	0	0	0	0
NUDT12	CAACACACACCATGACAC	14	26	0	0	0	0
NUDT12	GTACACACACCATGACTG	127	255	0	0	0	0
NUDT12	TGACACACACCATGTGGT	228	164	0	0	0	2
NUDT12	GTACACACACCATGTGCA	415	185	0	1	0	0
NUDT12	ACACACACACCATGTGAC	29	29	0	0	0	0
NUDT4	TGACACACGTTGGTGTGT	266	270	1	0	0	0
NUDT4	GTACACACGTTGGTGTCA	217	188	1	0	0	0
NUDT4	ACACACACGTTGGTGTAC	38	20	0	0	0	0
NUDT4	TGACACACACACGTGTTG	440	451	0	0	0	0
NUDT4	ACACACACACACGTCAGT	302	338	0	0	0	1
NUMA1	ACTGCAGTCAGTCACAGT	63	64	2	3	4	3
NUMA1	TGACTGTGGTACCAACGT	237	370	0	0	0	0
NUMA1	GTACTGTGGTACCAACCA	143	197	1	0	0	0

NUMA1	ACACTGTGGTACCAACAC	21	37	0	0	0	0
NUMA1	CAACTGTGGTACCAACTG	508	647	0	0	0	0
NUMB	ACACGTGTCATGGTTGAC	75	77	0	0	0	2
NUMB	CAACGTGTCATGGTTGTG	202	305	1	0	0	0
NUMB	TGACGTGTCATGCAGTGT	1227	860	3	0	0	1
NUMB	GTACGTGTCATGCAGTCA	401	272	0	0	0	0
NUMB	ACACGTGTCATGCAGTAC	79	110	0	0	0	0
NUMBL	ACACTGACCACATGCAGT	336	363	1	0	0	0
NUMBL	TGACTGACCACATGCACA	51	53	0	0	0	0
NUMBL	CAACTGACCACATGCAAC	190	31	0	0	0	0
NUMBL	GTACTGACCACATGCATG	78	97	0	1	0	9
NUMBL	TGACTGACCACATGACGT	243	294	0	0	0	0
NUP153	GTACTGCACATGCACATG	108	220	0	0	0	0
NUP153	TGACTGCACATGCAACGT	35	66	0	1	0	3
NUP153	GTACTGCACATGCAACCA	1039	572	1	0	0	5
NUP153	ACACTGCACATGCAACAC	33	55	0	0	0	0
NUP153	CAACTGCACATGCAACTG	1603	843	1	2	0	3
NUP153	TGTGCAACTGCACAGTGT	209	244	2	0	0	14
NUP214	ACCATGTGACACTGGTTG	132	39	0	0	2	0
NUP214	ACCATGTGACACACTGTG	151	315	0	0	0	1
NUP214	GTCATGTGACACTGGTGT	356	567	0	1	0	0
NUP214	CACATGTGACACTGGTCA	191	171	0	1	0	0
NUP214	TGCATGTGACACTGGTAC	64	55	0	1	0	0
NUP214	CATGCAACTGCATGCAAC	24	14	0	0	0	0
NXT1	GTTGGTCATGTGCAGTTG	2166	1749	12	5	16	23845
NXT1	TGTGGTCACAACCAGTAC	115	126	0	0	0	0
NXT1	TGTGGTCATGTGCAGTCA	40	32	0	0	0	0
NXT1	CATGGTCATGTGCAGTAC	41	58	0	0	0	0
NXT1	TGTGGTCATGTGCACAGT	498	365	0	0	0	0
NXT1	ACTGCAACCAACCAGTTG	555	735	0	0	0	0
OAS1	TGCATGTGCATGTGTGCA	51	59	0	0	0	0
OAS1	CACATGTGCATGTGTGAC	36	108	0	0	0	0
OAS1	GTCATGTGCATGTGTGTG	179	207	0	0	0	0
OAS1	ACCATGTGACGTGTGTGT	197	266	0	0	0	0
OAS1	TGCATGTGACGTGTGTCA	131	113	0	0	0	1
OAS2	CACATGTGACGTGTGTAC	42	65	0	0	0	0
OAS2	GTCATGTGACGTGTGTTG	709	647	0	0	0	4
OAS2	TGCATGTGACGTGTCAGT	28	50	0	0	0	0
OAS2	GTCATGTGACGTGTCACA	53	54	0	0	0	0
OAS2	ACCATGTGACGTGTCAAC	5	7	0	0	0	0
OAT	TGACCACAACGTCATGCA	702	562	1	0	2	8
OAT	ACACCACAACGTCATGGT	755	371	0	1	1	0
OAT	ACACCACAACGTCAACCA	1074	776	1	1	0	2
OAT	GTACCACAACGTCAACAC	30	27	0	0	0	0
OAT	TGACCACAACGTCAACTG	258	265	0	1	0	0
OAT	TGTGCACACAACACCAGT	244	204	0	0	0	0
OAZ1	CAACTGTGACCACACATG	187	381	1	0	0	0
OAZ1	GTACTGTGACCACAACGT	1138	338	1	1	0	0
OAZ1	CAACTGTGACCACAACCA	676	791	0	1	0	2
OAZ1	TGACTGTGACCACAACAC	24	75	3	1	0	0
OAZ1	ACACTGTGACCACAACTG	177	476	0	0	0	0
OBFC1	ACTGGTCAACCATGCAAC	88	61	0	0	1	0
OBFC1	CATGGTCAACCATGCATG	105	124	0	0	0	0
OBFC1	GTTGGTCAACCATGACGT	20	88	0	0	0	0
OBFC1	CATGGTCAACCATGACCA	146	119	0	0	0	0
OBFC1	TGTGGTCAACCATGACAC	141	48	0	0	0	4
OBFC2B	CATGGTCAACCAGTACTG	341	339	0	0	0	3
OBFC2B	TGTGGTCATGGTGTCACA	179	77	0	0	0	0
OBFC2B	CATGGTCATGGTGTCAAC	25	17	0	0	0	0
OBFC2B	GTTGGTCATGGTGTCATG	208	229	1	0	0	0
OBFC2B	TGTGGTCATGGTGTACGT	185	331	0	1	0	0
OCIAD1	ACTGCACATGGTCATGGT	1085	1199	1	2	1	0
OCIAD1	CATGGTTGCATGTGCAAC	1	2	0	0	0	0
OCIAD1	GTTGGTTGCATGTGCATG	148	262	0	0	0	0
OCIAD1	TGTGGTTGCATGTGACGT	355	633	0	2	0	0
OCIAD1	GTTGGTTGCATGTGACCA	54	90	0	0	0	0
OCIAD1	GTTGCAGTGTTGTGACTG	105	190	0	0	0	0
OCRL	GTACACCACAACGTGTTG	459	254	0	0	0	0
OCRL	TGACACCACAACGTCAGT	51	276	0	1	0	0
OCRL	GTACACCACAACGTCACA	45	60	0	0	0	0
OCRL	ACACACCACAACGTCAAC	25	31	0	0	0	0
OCRL	ACTGCAGTTGGTACCAGT	106	258	0	0	0	0
OCRL	GTTGCAACACGTACACTG	356	225	0	0	0	0
ODC1	TGACTGTGACGTACACAC	45	38	0	0	0	0
ODC1	ACACTGTGACGTACACTG	163	340	0	0	0	1

ODC1	CAACTGTGACGTACTGGT	15	8	0	0	0	0
ODC1	ACACTGTGACGTACTGCA	46	77	0	0	0	0
ODC1	TGTGCACACAGTGTGTGT	63	110	0	0	0	0
ODC1	GTTGCACACAGTGTGTCA	380	156	0	0	0	0
OGDH	ACACCAGTGTCATGGTTG	169	287	0	0	0	0
OGDH	CAACCAGTGTCATGCAGT	294	361	1	0	0	16
OGDH	ACACCAGTGTCATGCACA	42	35	0	0	0	0
OGDH	GTACCAGTGTCATGCAAC	48	80	0	0	0	0
OGDH	CAACCAGTGTACGTGTGT	355	310	0	1	0	0
OGDHL	ACACCAACACCACAACCA	108	176	0	0	0	0
OGDHL	GTACCAACACCACAACAC	23	29	0	0	0	0
OGDHL	TGACCAACACCACAACTG	953	1257	1	1	0	0
OGDHL	ACACCAACACCACATGGT	718	1195	0	0	0	5
OGDHL	TGACCAACACCACATGCA	241	264	0	1	0	0
OGDHL	ACTGCACATGGTCACATG	239	223	0	1	0	0
OGFR	ACACTGACCAGTACACCA	515	658	1	0	1	0
OGFR	ACACTGACCAGTACCATG	420	211	0	0	0	0
OGFR	CAACTGACCAGTACACGT	35	112	0	0	0	0
OGFR	GTACTGACCAGTACACAC	7	13	0	0	0	0
OGFR	TGACTGACCAGTACACTG	685	444	1	0	0	0
OGG1	GTCATGTGCATGCAACCA	274	323	0	1	1	0
OGG1	GTCATGTGCATGCACATG	126	162	0	0	0	0
OGG1	TGCATGTGCATGCAACGT	53	43	0	0	0	0
OGG1	ACCATGTGCATGCAACAC	199	32	0	0	0	0
OGG1	CACATGTGCATGCAACTG	142	159	0	0	0	0
OGG1	TGTGCAACACGTACTGGT	278	281	0	0	0	0
OLA1	TGTGGTCACATGGTTGTG	200	653	0	1	0	1
OLA1	CATGGTCACATGCAGTGT	534	239	1	1	0	0
OLA1	ACTGGTCACATGCAGTCA	206	214	1	0	0	0
OLA1	GTTGGTCACATGCAGTAC	474	170	0	2	0	0
OLA1	TGTGGTCACATGCAGTTG	947	1097	1	2	0	0
OLAH	ACACACCAACCAGTACTG	895	743	1	0	0	21
OLAH	CAACACCAACCAGTTGGT	436	378	0	0	0	1
OLAH	ACACACCAACCAGTTGCA	246	90	1	0	0	4
OLAH	GTACACCAACCAGTTGAC	32	15	0	0	0	0
OLAH	TGACACCAACCAGTTGTG	960	572	0	0	0	0
OLAH	ACTGCACATGGTCAACCA	343	448	0	0	0	0
OLIG1	GTTGCAGTACTGGTGTTG	408	636	1	1	1	1
OLIG1	ACACGTTGCAACACTGAC	55	180	0	0	0	0
OLIG1	CAACGTTGCAACACTGTG	164	467	1	0	0	0
OLIG1	TGACGTTGCAACTGGTGT	110	142	0	0	0	0
OLIG1	TGTGCATGTGTGACCAGT	140	156	1	0	0	10
OLIG2	TGACGTACACTGGTACCA	191	145	0	0	0	0
OLIG2	CAACGTACACTGGTACAC	17	27	0	0	0	0
OLIG2	GTACGTACACTGGTACTG	448	706	0	0	0	0
OLIG2	TGACGTACACTGGTTGGT	792	227	1	0	0	0
OLIG2	TGTGCAGTACACACACTG	715	733	0	7	0	0
OLR1	GTACTGCAACGTACGTGT	207	162	0	0	0	0
OLR1	CAACTGCAACGTACGTCA	212	190	0	0	0	0
OLR1	TGACTGCAACGTACGTAC	30	27	0	0	0	0
OLR1	ACACTGCAACGTACGTTG	433	726	0	1	0	0
OLR1	CAACTGCAACGTACCAGT	282	148	0	0	0	4
ONECUT1	ACACGTACGTTGACACCA	93	278	1	0	1	0
ONECUT1	TGACGTACGTTGACTGCA	171	227	1	0	1	0
ONECUT1	ACTGCAACTGCAGTGTTG	100	91	0	0	1	0
ONECUT1	GTACGTACGTTGACACAC	16	14	0	0	0	0
ONECUT1	TGACGTACGTTGACACTG	635	638	1	0	0	0
ONECUT1	ACACGTACGTTGACTGGT	167	184	0	0	0	0
OPRD1	ACACGTCAACGTACTGGT	64	102	0	0	0	0
OPRD1	TGACGTCAACGTACTGCA	153	171	1	0	0	0
OPRD1	CAACGTCAACGTACTGAC	50	171	0	0	0	0
OPRD1	GTACGTCAACGTACTGTG	173	179	0	0	0	0
OPRD1	ACACGTCAACGTTGGTGT	568	1026	0	1	0	0
OPRK1	TGACGTCAACGTTGGTCA	321	412	0	0	0	3
OPRK1	CAACGTCAACGTTGGTAC	32	23	0	0	0	0
OPRK1	GTACGTCAACGTTGGTTG	574	596	1	0	0	0
OPRK1	TGACGTCAACGTTGCAGT	307	234	0	0	0	0
OPRK1	CATGGTTGACACGTACTG	267	202	0	0	0	0
OPRL1	GTACGTCAACGTTGCACA	76	84	0	0	0	0
OPRL1	ACACGTCAACGTTGCAAC	79	52	0	0	0	0
OPRL1	CAACGTCAACGTTGCATG	64	23	0	0	0	0
OPRL1	GTACGTCAACGTTGACGT	138	137	0	0	0	0
OPRL1	CAACGTCAACGTTGACCA	277	258	3	0	0	1
OPRM1	GTTGCAGTACACGTGTAC	202	45	0	0	1	0
OPRM1	TGACGTCAACGTTGACAC	11	10	0	0	0	0

OPRM1	ACACGTCAACGTTGACTG	514	286	0	0	0	7
OPRM1	ACTGCAGTACACGTGTCA	325	79	0	0	0	0
OPRM1	TGTGCAGTACACGTGTTG	510	517	2	1	0	2
OPRS1	ACTGCAACACACTGACGT	138	169	0	0	1	0
OPRS1	CAACTGCAACCATGACCA	441	533	1	0	0	3
OPRS1	TGACTGCAACCATGACAC	82	49	0	0	0	0
OPRS1	ACACTGCAACCATGACTG	222	230	0	0	0	0
OPRS1	CAACTGCAACCATGTGGT	857	972	1	1	0	1
OPRS1	TGTGCAACACACTGCATG	551	186	2	0	0	0
ORC1L	TGTGGTCATGACCAGTTG	677	618	3	0	1	0
ORC1L	CATGGTCATGACCAGTGT	289	616	0	1	0	2
ORC1L	ACTGGTCATGACCAGTCA	288	144	1	0	0	0
ORC1L	GTTGGTCATGACCAGTAC	138	106	0	0	0	7
ORC1L	ACTGGTCATGACCACAGT	119	413	0	0	0	0
ORC1L	ACTGCATGCAGTTGTGCA	556	213	0	0	0	4
ORC2L	CAACGTTGCAGTCATGTG	271	711	0	0	0	1
ORC2L	TGACGTTGCAGTACGTGT	61	61	0	0	0	0
ORC2L	GTACGTTGCAGTACGTCA	108	149	0	1	0	0
ORC2L	ACACGTTGCAGTACGTAC	51	45	0	0	0	0
ORC2L	CAACGTTGCAGTACGTTG	63	47	0	0	0	0
ORC2L	ACTGCATGCACAACCAAC	344	151	0	0	0	5
ORC3L	GTTGGTACACGTACACCA	347	254	1	0	1	1
ORC3L	TGTGGTACACGTACCACA	25	47	0	0	0	0
ORC3L	CATGGTACACGTACCAAC	32	20	1	0	0	0
ORC3L	GTTGGTACACGTACCATG	235	226	1606	3	0	0
ORC3L	TGTGGTACACGTACACGT	43	100	2	0	0	0
ORC3L	GTTGCATGCACATGCATG	95	81	0	0	0	0
ORC4L	CATGGTACACACTGACTG	1026	860	5	2	0	569
ORC4L	GTTGGTACACACTGTGGT	721	665	3	1	0	0
ORC4L	CATGGTACACACTGTGCA	299	321	3	1	0	0
ORC4L	TGTGGTACACACTGTGAC	16	11	0	0	0	4
ORC4L	ACTGGTACACACTGTGTG	152	240	1	0	0	0
ORC4L	GTTGCATGCACAACCACA	69	86	0	0	0	0
ORC5L	TGTGGTACTGCAGTTGAC	40	62	0	0	0	0
ORC5L	GTTGGTACTGCACACAAC	15	17	0	0	0	0
ORC5L	TGTGGTACTGCACACATG	416	227	0	0	0	0
ORC5L	ACTGGTACTGCACAACGT	412	516	1	1	0	0
ORC5L	TGTGGTACTGCACAACCA	75	97	0	0	0	0
ORC5L	CATGCATGCACAACCATG	508	189	0	0	0	0
ORM1	ACCATGTGACCACAGTTG	924	1058	0	2	2	10
ORM1	TGCATGTGACCACAGTAC	60	70	1	0	0	0
ORM1	CACATGTGACCACACAGT	507	411	0	0	0	0
ORM1	ACCATGTGACCACACACA	86	318	0	0	0	1
ORM1	GTCATGTGACCACACAAC	20	35	0	0	0	0
ORM1	TGTGCACATGGTACTGTG	691	183	0	0	0	1
OSBP	ACTGGTACGTTGTGACCA	492	254	1	0	0	1
OSBP	GTTGGTACGTTGTGACAC	23	27	0	0	0	0
OSBP	TGTGGTACGTTGTGACTG	1034	1293	1	0	0	19
OSBP	ACTGGTACGTTGTGTGGT	656	1108	1	0	0	1
OSBP	TGTGGTACGTTGTGTGCA	70	146	0	1	0	0
OSBPL1A	ACTGGTACACGTCATGCA	86	193	0	0	2	0
OSBPL1A	CATGGTACACGTCAACCA	584	277	1	0	0	2
OSBPL1A	TGTGGTACACGTCAACAC	40	30	0	0	0	0
OSBPL1A	ACTGGTACACGTCAACTG	293	193	0	0	0	0
OSBPL1A	CATGGTACACGTCATGGT	326	141	0	1	0	0
OSBPL1A	ACTGCAACCAGTACACTG	80	122	0	0	0	0
OSBPL3	CATGCAACACCATGTGGT	724	495	1	0	1	0
OSBPL3	CATGGTACCATGACACTG	318	73	0	0	0	0
OSBPL3	GTTGGTACCATGACTGGT	86	224	0	0	0	0
OSBPL3	CATGGTACCATGACTGCA	165	132	0	1	0	0
OSBPL3	TGTGGTACCATGACTGAC	18	7	0	0	0	0
OSBPL3	ACTGGTACCATGACTGTG	685	596	0	1	0	5
OSBPL5	ACTGGTTGCATGTGACAC	66	57	0	0	0	0
OSBPL5	CATGGTTGCATGTGACTG	789	526	0	0	0	0
OSBPL5	GTTGGTTGCATGTGTGGT	225	250	1	0	0	0
OSBPL5	CATGGTTGCATGTGTGCA	95	122	0	0	0	0
OSBPL5	TGTGCAACACACCACAGT	284	327	1	0	0	4
OSBPL5	GTTGCAACACACCACACA	152	189	0	1	0	0
OSCAR	ACACTGCAACCAGTGTCA	145	225	0	0	1	0
OSCAR	CAACTGCAACGTTGTGAC	101	29	0	0	0	0
OSCAR	GTACTGCAACGTTGTGTG	10	23	0	0	0	0
OSCAR	CAACTGCAACCAGTGTGT	316	444	0	1	0	0
OSCAR	ACTGACACTGGTCAGTAC	36	20	0	0	0	0
OTC	CAACCACAACACTGCATG	552	459	0	0	1	1
OTC	TGACCACAACACTGCAGT	278	312	0	0	0	0

OTC	GTACCACAACACTGCACA	76	106	0	1	0	0
OTC	ACACCACAACACTGCAAC	57	48	0	0	0	0
OTC	GTACCACAACACTGACGT	154	233	0	0	0	0
OTUB1	GTCATGTGCAGTGTGTGT	999	678	1	0	2	0
OTUB1	CACATGTGCAGTGTGTCA	1128	568	2	0	1	4
OTUB1	ACCATGTGGTTGTGTGCA	332	183	0	0	0	0
OTUB1	GTCATGTGGTTGTGTGAC	28	14	0	0	0	0
OTUB1	TGCATGTGGTTGTGTGTG	148	178	0	0	0	0
OTUB1	TGTGCACATGGTCAACTG	198	229	0	1	0	0
OTX2	GTACGTACCACAGTTGTG	913	780	5	3	1	0
OTX2	ACACGTACCACAGTTGGT	473	266	0	0	0	0
OTX2	TGACGTACCACAGTTGCA	188	106	0	0	0	0
OTX2	CAACGTACCACAGTTGAC	15	24	1	0	0	0
OTX2	ACTGGTACTGCAACTGGT	711	842	2	0	0	11
OTX2	TGTGCAACTGTGACACGT	211	117	0	0	0	0
OXA1L	ACACACGTACTGCATGGT	793	608	1	0	1	1
OXA1L	TGACACGTACTGCATGCA	245	285	0	1	1	2
OXA1L	TGACACGTACTGCAACTG	615	756	0	1	0	0
OXA1L	CAACACGTACTGCATGAC	201	188	0	0	0	3
OXA1L	GTACACGTACTGCATGTG	342	177	0	0	0	0
OXA1L	ACTGCACACATGACACCA	265	358	0	0	0	0
OXCT1	GTACCAACGTTGACTGAC	53	45	12	13	12	16
OXCT1	CAACCAACGTTGACTGGT	155	185	0	0	0	0
OXCT1	ACACCAACGTTGACTGCA	153	229	0	1	0	3
OXCT1	GTTGCAGTACTGCAGTAC	222	23	0	0	0	0
OXCT1	ACTGACACGTCATGACTG	278	292	1	0	0	0
OXCT2	TGACCAACCAGTGTGTGT	1069	1112	0	7	2	0
OXCT2	CAACCAACCAGTGTGTTG	816	726	25	0	1	1
OXCT2	GTACCAACCAGTGTGTCA	563	615	1	0	0	1
OXCT2	ACACCAACCAGTGTGTAC	24	38	0	0	0	0
OXCT2	GTTGCACATGACCACAAC	120	74	0	0	0	0
OXCT2	TGTGCACATGACCACATG	208	160	0	0	0	0
OXSM	ACACACACGTACACCACA	100	219	0	0	1	0
OXSM	CAACACACGTACACCAGT	56	67	0	0	0	0
OXSM	GTACACACGTACACCAAC	13	25	0	0	0	0
OXSM	TGACACACGTACACCATG	101	102	0	1	0	1
OXSM	GTTGCACATGGTCATGTG	526	620	1	0	0	0
OXSM	ACTGCACATGGTACGTGT	553	176	1	0	0	14
OXTR	CAACGTCAACGTTGTGGT	0	1	0	0	0	0
OXTR	ACACGTCAACGTTGTGCA	130	168	0	0	0	0
OXTR	GTACGTCAACGTTGTGAC	23	37	0	0	0	0
OXTR	TGACGTCAACGTTGTGTG	442	385	0	0	0	1
OXTR	GTACGTTGTGACTGTGCA	447	338	1	0	0	1
OXTR	GTTGCATGCACACAACAC	28	18	0	0	0	0
P11	GTACACCATGACCAACGT	174	321	2	0	0	0
P11	CAACACCATGACCAACCA	248	408	0	1	0	1
P11	TGACACCATGACCAACAC	8	40	0	0	0	0
P11	ACACACCATGACCAACTG	432	403	0	0	0	0
P11	CAACACCATGACCATGGT	613	656	0	1	0	0
P11	ACTGCAACACACTGGTTG	237	293	0	0	0	1
P2RX7	CAACACGTGTTGCAGTCA	150	144	0	1	1	3
P2RX7	CAACACGTGTTGGTTGCA	78	84	0	0	0	0
P2RX7	TGACACGTGTTGGTTGAC	15	25	0	0	0	0
P2RX7	ACACACGTGTTGGTTGTG	435	402	0	1	0	1
P2RX7	GTACACGTGTTGCAGTGT	658	569	1	0	0	0
P2RY1	GTACGTCAACCAGTGTGT	555	536	1	0	1	0
P2RY1	CAACGTCAACCAGTGTCA	851	634	32	2	0	0
P2RY1	TGACGTCAACCAGTGTAC	66	70	0	0	0	0
P2RY1	ACACGTCAACCAGTGTTG	712	585	1	0	0	0
P2RY1	CAACGTCAACCAGTCAGT	362	410	1	0	0	0
P2RY1	GTTGCATGGTCAACTGGT	791	338	0	0	0	0
P2RY12	ACACGTCAACCAGTCACA	272	368	0	1	0	0
P2RY12	GTACGTCAACCAGTCAAC	30	29	0	0	0	0
P2RY12	TGACGTCAACCAGTCATG	157	229	0	0	0	0
P2RY12	ACACGTCAACCAGTACGT	197	401	3	0	0	1
P2RY12	TGACGTCAACCAGTACCA	74	72	0	0	0	0
P2RY12	CATGCATGGTCATGTGTG	120	111	0	0	0	0
P2RY2	GTACGTCAACCAGTACTG	896	845	6	0	1	1
P2RY2	CAACGTCAACCAGTACAC	40	43	0	0	0	0
P2RY2	TGACGTCAACCAGTTGGT	1367	382	1	2	0	0
P2RY2	ACTGCAGTACACGTACAC	142	122	0	0	0	0
P2RY2	CATGCAGTACACGTACTG	229	116	1	0	0	0
P2RY2	CATGCATGGTCAACTGCA	73	208	0	1	0	0
P2RY5	TGACGTCATGACTGACAC	81	94	0	0	0	0
P2RY5	ACACGTCATGACTGACTG	156	136	1	0	0	0

P2RY5	CAACGTCATGACTGTGGT	274	250	1	0	0	0
P2RY5	ACACGTCATGACTGTGCA	399	311	1	0	0	1
P2RY5	GTACGTCATGACTGTGAC	46	41	0	0	0	0
P2RY6	CATGCATGGTACGTGTGT	880	841	1	0	1	1
P2RY6	TGACGTCATGACTGTGTG	215	131	0	0	0	0
P2RY6	ACACGTCATGTGGTGTGT	1267	1129	4	0	0	1
P2RY6	TGACGTCATGTGGTGTCA	175	190	0	0	0	0
P2RY6	CAACGTCATGTGGTGTAC	99	76	0	0	0	0
P2RY6	GTACGTCATGTGGTGTTG	272	331	0	0	0	0
P4HB	ACACACCAACACTGGTCA	111	65	0	0	0	0
P4HB	GTACACCAACACTGGTAC	59	88	4	0	0	6
P4HB	TGACACCAACACTGGTTG	1553	984	2	1	0	0
P4HB	ACACACCAACACTGCAGT	598	419	2	0	0	1
P4HB	TGACACCAACACTGCACA	53	53	0	0	0	0
PA2G4	GTACCAGTTGTGCAACAC	44	55	0	0	0	0
PA2G4	TGACCAGTTGTGCAACTG	846	654	0	2	0	2
PA2G4	TGACACACGTGTGTACGT	41	25	0	0	0	0
PA2G4	GTACACACGTGTGTACCA	236	172	0	0	0	0
PA2G4	ACACACACGTGTGTACAC	18	12	0	0	0	0
PABPC1	CAACACTGGTTGACCATG	440	905	0	0	0	0
PABPC1	GTACACTGGTTGACACGT	72	194	1	0	0	0
PABPC1	CAACACTGGTTGACACCA	304	305	0	0	0	0
PABPC1	GTACTGTGGTTGCACACA	312	284	1	0	0	159
PABPC1	ACACTGTGGTTGCACAAC	21	78	0	0	0	0
PACAP	TGTGGTTGCATGTGTGAC	89	153	0	0	0	0
PACAP	ACTGGTTGCATGTGTGTG	1158	1448	2	3	0	2
PACAP	GTTGGTTGACGTGTGTGT	122	137	0	0	0	0
PACAP	CATGGTTGACGTGTGTCA	773	424	2	1	0	5
PACAP	TGTGCAACTGCAACTGTG	415	327	0	1	0	0
PACAP	CATGCAACTGCATGGTGT	430	403	1	0	0	0
PACRG	CATGGTCAACGTACACAC	100	112	0	0	1	1
PACRG	TGTGGTCAACGTACACCA	140	226	0	0	0	0
PACRG	GTTGGTCAACGTACACTG	256	788	0	0	0	0
PACRG	TGTGGTCAACGTACTGGT	66	86	0	0	0	0
PACRG	GTTGGTCAACGTACTGCA	93	64	0	0	0	0
PACRG	TGTGCAACACTGCAACTG	69	100	0	0	0	0
PADI2	GTACACACCATGCAGTCA	906	863	0	0	1	0
PADI2	CAACACACCATGGTTGTG	591	212	0	0	0	1
PADI2	TGACACACCATGCAGTGT	529	651	1	0	0	1
PADI2	ACACACACCATGCAGTAC	42	56	0	0	0	0
PADI2	ACTGACACCATGACACCA	334	320	1	0	0	0
PAF1	ACCATGTGACACGTACCA	359	95	0	0	0	1
PAF1	GTCATGTGACACGTACAC	23	11	0	0	0	0
PAF1	TGCATGTGACACGTACTG	177	199	0	0	0	0
PAF1	ACCATGTGACACGTTGGT	349	320	0	0	0	0
PAF1	TGTGGTACTGTGACTGTG	73	71	0	0	0	1
PAFAH1B1	GTACACACGTTGTGTGCA	84	167	0	0	0	0
PAFAH1B1	ACACACACGTTGTGTGAC	14	14	0	0	0	0
PAFAH1B1	CAACACACGTTGTGTGTG	30	59	0	0	0	0
PAFAH1B1	ACACACACCAGTGTGTGT	161	143	0	0	0	0
PAFAH1B1	TGACACACCAGTGTGTCA	22	27	0	0	0	0
PAFAH1B1	TGTGCACAACCATGACGT	190	156	0	0	0	0
PAFAH1B2	TGACACCACATGTGTGGT	163	537	0	0	0	0
PAFAH1B2	GTACACCACATGTGTGCA	126	139	0	0	0	0
PAFAH1B2	ACACACCACATGTGTGAC	34	51	0	0	0	0
PAFAH1B2	CAACACCACATGTGTGTG	256	285	0	0	0	0
PAFAH1B2	GTTGGTTGTGGTCAGTTG	711	707	0	0	0	1
PAFAH1B3	CAACACCAACGTGTCACA	306	115	0	0	1	0
PAFAH1B3	TGACACCAACGTGTCAAC	18	12	0	0	0	0
PAFAH1B3	ACACACCAACGTGTCATG	171	205	0	0	0	0
PAFAH1B3	CAACACCAACGTGTACGT	699	851	2	1	0	0
PAFAH1B3	TGTGCAGTCATGTGCACA	100	59	0	0	0	0
PAFAH1B3	TGTGCAACCAACCATGGT	338	335	0	2	0	2
PAFAH2	CAACACCAACGTGTTGAC	50	39	0	0	0	0
PAFAH2	GTACACCAACGTGTTGTG	38	48	0	0	0	1
PAFAH2	ACACACCAACGTCAGTGT	152	202	0	0	0	0
PAFAH2	TGACACCAACGTCAGTCA	97	97	1	0	0	0
PAFAH2	ACTGCAGTCACATGTGCA	95	215	0	0	0	0
PAG1	TGTGGTCACACATGACCA	181	357	0	0	0	0
PAG1	CATGGTCACACATGACAC	22	19	0	0	0	0
PAG1	GTTGGTCACACATGACTG	548	738	0	1	0	0
PAG1	TGTGGTCACACATGTGGT	59	89	0	0	0	0
PAG1	GTTGGTCACACATGTGCA	44	53	0	0	0	0
PAH	GTACACTGCATGCATGCA	479	389	0	0	0	0
PAH	ACACACTGCATGCATGAC	59	78	0	0	0	0

PAH	CAACACTGCATGCATGTG	752	1523	1	2	0	0
PAH	TGACACTGCATGACGTGT	321	274	0	0	0	0
PAH	GTACACTGCATGACGTCA	352	440	0	0	0	0
PAH	GTTGCACACAACACCACA	104	126	2	0	0	0
PAK1	CAACGTTGTGACACTGCA	459	741	1	2	1	0
PAK1	CACATGACCAGTACCAAC	203	48	0	0	0	0
PAK1	GTCATGACCAGTACCATG	504	276	1	0	0	0
PAK1	TGCATGACCAGTACACGT	15	31	0	0	0	0
PAK1	GTCATGACCAGTACACCA	434	244	0	1	0	0
PAK2	TGCATGACCAGTGTGTGT	687	444	2	0	1	2
PAK2	GTCATGACGTTGTGTGGT	704	923	0	2	0	0
PAK2	CACATGACGTTGTGTGCA	124	63	0	0	0	0
PAK2	TGCATGACGTTGTGTGAC	47	37	0	0	0	0
PAK2	ACCATGACGTTGTGTGTG	107	120	1	0	0	23
PAK3	ACCATGACACACACTGAC	42	35	0	0	0	0
PAK3	CACATGACACACACTGTG	608	885	3	1	0	2
PAK3	TGCATGACACACTGGTGT	863	757	3	0	0	2
PAK3	GTCATGACACACTGGTCA	125	185	0	0	0	0
PAK3	ACCATGACACACTGGTAC	159	159	0	1	0	0
PAK6	CACATGACGTCATGCATG	652	512	1	0	1	2
PAK6	ACCATGACGTCATGCAAC	55	58	0	0	0	0
PAK6	GTCATGACGTCATGACGT	13	3	0	0	0	0
PAK6	CACATGACGTCATGACCA	232	239	0	0	0	0
PAK6	TGCATGACGTCATGACAC	31	125	0	0	0	0
PAK6	ACTGCAACACCACACACA	109	124	0	0	0	0
PALB2	TGTGGTTGACTGACTGGT	199	193	0	0	1	1
PALB2	ACTGGTTGACTGACACGT	36	39	0	0	0	0
PALB2	TGTGGTTGACTGACACCA	376	327	0	1	0	0
PALB2	CATGGTTGACTGACACAC	127	132	1	0	0	0
PALB2	GTTGGTTGACTGACACTG	541	768	0	1	0	2
PAN2	CACATGTGCAGTACTGGT	518	465	0	0	0	0
PAN2	ACCATGTGCAGTACTGCA	91	124	0	0	0	1
PAN2	GTCATGTGCAGTACTGAC	62	24	0	0	0	0
PAN2	TGCATGTGCAGTACTGTG	161	182	1	0	0	0
PAN2	CATGCAGTCACAGTACTG	659	724	0	0	0	0
PANK1	CAACCAACACACTGCACA	328	347	0	2	1	0
PANK1	CAACCAACACACTGGTTG	657	298	0	0	0	1
PANK1	GTACCAACACACTGCAGT	473	203	2	0	0	0
PANK1	TGACCAACACACTGCAAC	103	26	0	0	0	0
PANK1	CATGGTACTGTGACGTAC	42	51	0	0	0	0
PANK1	GTTGCAACTGGTACACAC	72	96	0	0	0	0
PANK2	TGACCAACTGGTGTTGGT	381	185	0	1	1	0
PANK2	TGACCAACTGGTGTACCA	153	171	0	0	0	9
PANK2	CAACCAACTGGTGTACAC	47	39	0	0	0	0
PANK2	GTACCAACTGGTGTACTG	347	622	0	0	0	2
PANK2	ACTGGTTGACTGTGGTAC	115	49	0	0	0	0
PANK2	CATGCAACTGCACACACA	40	87	0	0	0	0
PANK3	CAACGTTGTGACACGTGT	99	45	0	0	0	0
PANK3	CAACCAACACTGTGTGTG	361	921	1	1	0	0
PANK3	GTACCAACTGGTGTGTGT	142	261	1	0	0	1
PANK3	CAACCAACTGGTGTGTCA	352	357	1	0	0	0
PANK3	TGACCAACTGGTGTGTAC	274	145	0	0	0	0
PANK3	TGTGCAACTGCACACAAC	1	1	0	0	0	0
PANK4	ACACCAACACTGTGGTTG	538	704	0	0	1	1
PANK4	CAACGTTGTGACTGGTCA	325	180	0	0	0	0
PANK4	ACACGTTGTGACTGGTTG	91	97	0	0	0	0
PANK4	CAACCAACACTGTGCAGT	344	286	1	0	0	0
PANK4	ACACCAACACTGTGCACA	215	170	0	0	0	0
PANK4	TGTGCACATGGTCACAAC	24	19	0	0	0	0
PAPOLA	ACACACACTGTGGTCAGT	407	171	0	0	0	2
PAPOLA	TGACACACTGTGGTCACA	72	88	0	0	0	0
PAPOLA	CAACACACTGTGGTCAAC	39	13	0	0	0	0
PAPOLA	GTACACACTGTGGTCATG	128	73	0	0	0	0
PAPOLA	TGACACACTGTGGTACGT	36	89	0	0	0	0
PAPPA	GTACACCAGTGTGTGTGT	358	310	1	0	0	0
PAPPA	CAACACCAGTGTGTGTCA	429	247	1	1	0	1
PAPPA	TGACACCAGTGTGTGTAC	51	86	0	0	0	0
PAPPA	ACACACCAGTGTGTGTTG	223	212	0	0	0	1
PAPPA	CAACACCAGTGTGTCAGT	100	129	0	1	0	0
PAPSS1	ACTGCAGTACGTGTTGAC	27	23	0	0	0	0
PAPSS1	CATGCAGTACGTGTTGTG	862	1316	1	1	0	0
PAPSS1	TGTGCAGTACGTCAGTGT	714	538	0	0	0	0
PAPSS1	GTTGCAGTACGTCAGTCA	386	200	0	0	0	0
PAPSS1	ACTGCACAGTCAGTTGTG	753	1029	1	2	0	0
PAPSS2	GTACACGTCACATGACTG	409	459	0	0	1	0

PAPSS2	TGACACGTCACATGCATG	312	220	0	0	0	0
PAPSS2	ACACACGTCACATGACGT	140	69	0	0	0	0
PAPSS2	TGACACGTCACATGACCA	230	154	0	0	0	0
PAPSS2	CAACACGTCACATGACAC	23	65	0	0	0	0
PARC	ACCATGTGCAGTTGCAGT	78	60	0	0	0	0
PARC	TGCATGTGCAGTTGCACA	46	49	0	0	0	0
PARC	CACATGTGCAGTTGCAAC	25	33	0	0	0	0
PARC	GTCATGTGCAGTTGCATG	254	290	0	0	0	0
PARC	TGCATGTGCAGTTGACGT	65	117	0	0	0	0
PARD3	ACTGCAACACGTACGTTG	985	779	1	0	1	0
PARD3	ACTGGTCAGTGTACGTCA	80	101	0	0	0	0
PARD3	GTTGGTCAGTGTACGTAC	108	26	0	0	0	0
PARD3	TGTGGTCAGTGTACGTTG	1120	975	1	1	0	0
PARD3	ACTGGTCAGTGTACCAGT	109	312	0	0	0	0
PARD3	TGTGGTCAGTGTACCACA	34	28	0	0	0	0
PARG	GTACACACGTACACGTGT	131	89	0	0	0	0
PARG	CAACACACGTACACGTCA	400	148	0	0	0	0
PARG	TGACACACGTACACGTAC	23	38	0	0	0	0
PARG	ACACACACGTACACGTTG	194	325	0	0	0	0
PARG	GTACACACCAACCACACA	105	91	0	0	0	1
PARK2	ACTGCACAACTGCATGCA	201	238	0	122	24	0
PARK2	CACATGCACACATGTGGT	264	357	0	0	0	2
PARK2	ACCATGCACACATGTGCA	97	100	0	1	0	19
PARK2	GTCATGCACACATGTGAC	28	26	0	0	0	0
PARK2	TGCATGCACACATGTGTG	278	266	0	0	0	0
PARK2	TGCATGCACAACGTGTGT	100	126	0	0	0	0
PARK7	GTCATGTGCATGCATGGT	733	1006	1	0	0	1
PARK7	CACATGTGCATGCATGCA	6	13	0	0	0	0
PARK7	TGCATGTGCATGCATGAC	4	21	0	0	0	0
PARK7	ACCATGTGCATGCATGTG	323	147	0	0	0	0
PARK7	GTCATGTGCATGACGTGT	368	431	0	0	0	0
PARP1	GTACGTGTACTGACACTG	631	746	6	1	0	1
PARP1	TGACGTGTACTGACTGGT	552	228	1	2	0	6
PARP1	GTACGTGTACTGACTGCA	92	58	0	0	0	0
PARP1	ACACGTGTACTGACTGAC	27	12	0	0	0	0
PARP1	CAACGTGTACTGACTGTG	177	231	0	0	0	0
PARP1	CATGCACATGACCACAGT	196	253	0	0	0	0
PARP2	TGACGTGTACTGTGGTGT	268	215	1	0	0	0
PARP2	GTACGTGTACTGTGGTCA	456	159	3	0	0	1
PARP2	ACACGTGTACTGTGGTAC	55	40	0	0	0	0
PARP2	CAACGTGTACTGTGGTTG	1261	1256	2	0	0	1
PARP2	TGTGGTTGTGCAACCATG	111	97	0	0	0	0
PARP4	ACACACACTGACCATGGT	374	341	0	0	0	0
PARP4	TGACACACTGACCATGCA	429	386	1	0	0	0
PARP4	CAACACACTGACCATGAC	260	90	0	0	0	0
PARP4	GTACACACTGACCATGTG	1136	602	3	0	0	1
PARP4	ACACACACTGACACGTGT	419	291	1	0	0	0
PAWR	GTACGTTGCAGTCAGTGT	508	654	2	1	0	0
PAWR	CAACGTTGCAGTCAGTCA	298	137	0	0	0	0
PAWR	TGACGTTGCAGTCAGTAC	31	37	0	0	0	0
PAWR	ACACGTTGCAGTCAGTTG	698	545	2	1	0	0
PAWR	CAACGTTGCAGTCACAGT	44	84	0	0	0	0
PAX1	GTACGTACCACAACTGGT	351	288	0	1	0	0
PAX1	CAACGTACCACAACTGCA	300	141	0	0	0	0
PAX1	TGACGTACCACAACTGAC	8	18	0	0	0	0
PAX1	ACACGTACCACAACTGTG	304	182	0	0	0	0
PAX1	GTACGTACCACATGGTGT	1358	2232	0	3	0	11
PAX2	CAACGTACCACATGTGTG	549	337	0	0	3	0
PAX2	CAACGTACCAACGTGTGT	180	185	0	0	0	0
PAX2	ACACGTACCAACGTGTCA	106	86	0	0	0	0
PAX2	GTACGTACCAACGTGTAC	193	238	0	1	0	0
PAX2	TGACGTACCAACGTGTTG	35	69	0	0	0	0
PAX3	CAACGTACCAACGTTGCA	28	40	0	0	0	1
PAX3	TGACGTACCAACGTTGAC	36	66	0	1	0	0
PAX3	ACACGTACCAACGTTGTG	229	344	0	2	0	0
PAX3	GTACGTACCAACCAGTGT	486	475	1	1	0	0
PAX3	TGTGCATGCAGTACCAAC	91	89	0	0	0	0
PAX3	ACTGCATGCAGTACCATG	59	74	0	1	0	0
PAX4	GTACGTACCAACCACAAC	28	24	0	0	0	0
PAX4	TGACGTACCAACCACATG	116	103	0	1	0	0
PAX4	ACACGTACCAACCAACGT	458	606	0	0	0	0
PAX4	TGACGTACCAACCAACCA	330	187	0	0	0	0
PAX4	CAACGTACCAACCAACAC	56	78	0	0	0	0
PAX4	ACTGCACAACGTTGACTG	89	77	0	0	0	0
PAX5	GTTGCACATGCAGTGTCA	440	365	1	1	1	12

PAX5	TGACGTACCAACACCAAC	7	1	0	0	0	0
PAX5	ACACGTACCAACACCATG	334	355	0	0	0	0
PAX5	CAACGTACCAACACACGT	17	27	0	0	0	0
PAX5	ACACGTACCAACACACCA	560	713	1	1	0	10
PAX5	GTACGTACCAACACACAC	248	85	1	0	0	0
PAX6	GTACGTACCAACTGCACA	132	165	0	1	0	0
PAX6	ACACGTACCAACTGCAAC	121	61	0	1	0	12
PAX6	CAACGTACCAACTGCATG	220	164	915	3	0	0
PAX6	GTACGTACCAACTGACGT	126	131	0	0	0	0
PAX6	CAACGTACCAACTGACCA	94	73	0	0	0	1
PAX8	ACACGTTGACGTACACAC	30	54	0	0	0	0
PAX8	CAACGTTGACGTACACTG	286	264	0	0	0	0
PAX8	GTACGTTGACGTACTGGT	275	86	0	0	0	0
PAX8	TGTGACCAACGTTGGTTG	1300	644	0	1	0	1
PAX8	ACTGACCAACGTTGCAGT	381	233	1	0	0	0
PAXIP1	CATGGTCAACACGTCACA	107	216	0	0	1	0
PAXIP1	GTTGGTCAACACGTCAGT	115	121	1	0	0	0
PAXIP1	TGTGGTCAACACGTCAAC	5	6	0	0	0	0
PAXIP1	ACTGGTCAACACGTCATG	31	64	0	0	0	4
PAXIP1	CATGGTCAACACGTACGT	521	629	1	1	0	2
PBEF1	ACTGGTGTACACACTGAC	76	71	0	0	0	1
PBEF1	CATGGTGTACACACTGTG	275	211	0	0	0	0
PBEF1	TGTGGTGTACACTGGTGT	375	461	1	0	0	0
PBEF1	GTTGGTGTACACTGGTCA	424	406	0	0	0	0
PBEF1	ACTGGTGTACACTGGTAC	54	38	0	0	0	0
PC	CAACTGTGACGTTGACTG	442	468	1	0	1	14
PC	GTACTGTGACGTTGTGGT	827	133	1	0	0	65
PC	CAACTGTGACGTTGTGCA	259	539	1	0	0	0
PC	TGACTGTGACGTTGTGAC	37	50	0	0	0	0
PC	ACACTGTGACGTTGTGTG	179	242	0	0	0	0
PCAF	GTACGTACTGGTACGTCA	481	212	2652	3	0	2
PCAF	ACACGTACTGGTACGTAC	31	29	0	0	0	0
PCAF	CAACGTACTGGTACGTTG	406	291	0	1	0	5
PCAF	GTACGTACTGGTACCAGT	163	134	0	0	0	0
PCAF	CAACGTACTGGTACCACA	65	36	0	0	0	0
PCGF2	GTACGTTGTGGTGTGTTG	763	669	0	2	0	0
PCGF2	TGACGTTGTGGTGTCAGT	28	81	2	0	0	0
PCGF2	GTACGTTGTGGTGTCACA	119	98	0	0	0	0
PCGF2	ACACGTTGTGGTGTCAAC	16	11	0	0	0	0
PCGF2	CAACGTTGTGGTGTCATG	190	184	0	0	0	0
PCK1	GTACGTTGTGACACACCA	185	200	0	0	0	0
PCK1	GTACACACTGCAACACTG	1144	1243	3	1	0	0
PCK1	TGACACACTGCAACTGGT	184	234	0	0	0	3
PCK1	GTACACACTGCAACTGCA	57	87	0	0	0	0
PCK1	ACACACACTGCAACTGAC	60	69	0	0	0	0
PCK1	TGTGCATGCACATGTGAC	17	14	0	0	0	0
PCK2	GTACACACTGCATGGTCA	222	498	1	0	1	7
PCK2	TGTGGTGTGTTGTGGTTG	318	424	0	0	1	0
PCK2	CAACACACTGCAACTGTG	440	213	0	0	0	0
PCK2	TGACACACTGCATGGTGT	333	333	1	1	0	0
PCK2	TGTGACCAGTACACCATG	38	37	0	0	0	0
PCNA	GTCATGTGGTCAGTCACA	112	123	0	1	0	0
PCNA	ACCATGTGGTCAGTCAAC	86	60	0	0	0	0
PCNA	CACATGTGGTCAGTCATG	66	41	0	0	0	0
PCNA	GTCATGTGGTCAGTACGT	156	189	0	0	0	0
PCNA	TGTGCAGTACGTTGTGAC	40	84	0	0	0	0
PCNP	CATGGTCATGGTGTACTG	371	778	0	0	1	0
PCNP	GTTGGTCATGGTGTACCA	109	143	2	1	0	0
PCNP	ACTGGTCATGGTGTACAC	121	159	0	0	0	0
PCNP	GTTGGTCATGGTGTTGGT	202	293	0	0	0	0
PCNP	CATGGTCATGGTGTTGCA	84	91	0	0	0	0
PCNP	ACTGCACATGCAGTTGGT	178	147	0	0	0	0
PCNT	GTTGGTTGGTTGCAACGT	283	238	0	0	1	1
PCNT	CATGGTTGGTTGCAACCA	56	72	0	0	0	0
PCNT	TGTGGTTGGTTGCAACAC	29	26	0	0	0	0
PCNT	ACTGGTTGGTTGCAACTG	231	310	0	52	0	12
PCNT	CATGGTTGGTTGCATGGT	198	149	0	0	0	0
PCSK1	GTACACACCATGCATGTG	631	563	1	0	0	0
PCSK1	ACACACACCATGACGTGT	121	186	0	0	0	0
PCSK1	TGACACACCATGACGTCA	488	486	0	1	0	0
PCSK1	CAACACACCATGACGTAC	51	90	0	0	0	7
PCSK1	GTACACACCATGACGTTG	456	606	1	0	0	17
PCSK1	TGTGCAACCAACTGGTCA	229	222	1	0	0	0
PCSK2	CAACCAGTTGTGACACCA	85	100	1	0	0	0
PCSK2	ACACCAGTTGTGACACTG	341	346	0	0	0	0

PCSK2	GTACACACACGTTGACCA	74	60	0	0	0	0
PCSK2	ACACACACACGTTGACAC	36	100	0	0	0	0
PCSK2	CAACACACACGTTGACTG	444	427	0	0	0	0
PCSK2	CATGCAACCAACTGGTAC	81	99	0	0	0	0
PCYT1A	TGACCAACGTCAGTCACA	346	218	1	0	1	1
PCYT1A	CAACCAACGTCAGTCAAC	54	162	0	0	0	0
PCYT1A	GTACCAACGTCAGTCATG	158	150	0	1	0	0
PCYT1A	TGACCAACGTCAGTACGT	113	85	0	0	0	0
PCYT1A	GTACCAACGTCAGTACCA	245	433	0	1	0	0
PCYT1A	ACTGCATGCAGTCAGTTG	381	195	0	1	0	4
PCYT1B	ACACCAACGTGTACTGAC	9	11	0	0	0	0
PCYT1B	CAACCAACGTGTACTGTG	470	182	0	0	0	0
PCYT1B	TGACCAACGTGTTGGTGT	145	159	0	0	0	17
PCYT1B	GTACCAACGTGTTGGTCA	153	152	1	0	0	0
PCYT1B	ACACCAACGTGTTGGTAC	82	183	0	0	0	0
PDCD1	ACACTGTGTGTGACGTAC	47	53	0	0	0	0
PDCD1	CAACTGTGTGTGACGTTG	493	630	0	2	0	10
PDCD1	GTACTGTGTGTGACCAGT	431	456	0	0	0	1
PDCD1	CAACTGTGTGTGACCACA	213	187	5	0	0	0
PDCD1	TGACTGTGTGTGACCAAC	77	30	1	0	0	0
PDCD1	GTTGCACACATGACACAC	70	75	0	0	0	0
PDCD2	GTTGGTGTCATGACGTCA	145	161	0	0	0	0
PDCD2	ACTGGTGTCATGACGTAC	22	15	0	0	0	0
PDCD2	CATGGTGTCATGACGTTG	335	341	0	0	0	0
PDCD2	GTTGGTGTCATGACCAGT	209	66	1	0	0	0
PDCD2	TGTGCACAGTGTCAGTTG	638	835	0	1	0	2
PDCD2	ACTGCAACACCATGTGCA	58	112	0	0	0	0
PDCD4	ACACTGCAGTCAGTGTGT	103	84	0	0	0	0
PDCD4	TGACTGCAGTCAGTGTCA	146	110	0	0	0	0
PDCD4	CAACTGCAGTCAGTGTAC	79	75	0	0	0	0
PDCD4	GTACTGCAGTCAGTGTTG	477	934	1	0	0	0
PDCD4	TGACTGCAGTCAGTCAGT	366	454	0	1	0	0
PDE10A	CACATGTGACACCAGTAC	107	65	0	0	0	9
PDE10A	GTCATGTGACACCAGTTG	142	175	0	0	0	0
PDE10A	TGCATGTGACACCACAGT	262	252	0	0	0	0
PDE10A	GTCATGTGACACCACACA	147	290	0	0	0	12
PDE10A	ACCATGTGACACCACAAC	46	54	0	0	0	0
PDE10A	CATGCACAACCACAGTTG	1211	1136	3	1	0	0
PDE11A	ACACACCAACCAGTCAAC	24	31	0	0	0	0
PDE11A	CAACACCAACCAGTCATG	83	160	0	0	0	1
PDE11A	GTACACCAACCAGTACGT	968	366	0	0	0	1
PDE11A	CAACACCAACCAGTACCA	779	716	0	0	0	0
PDE11A	TGACACCAACCAGTACAC	51	67	0	0	0	0
PDE1A	ACACACCACATGACGTCA	222	469	2	106	11	0
PDE1A	ACACACCACATGACCAGT	419	486	0	1	1	0
PDE1A	GTACACCACATGACGTAC	113	134	1	0	0	0
PDE1A	TGACACCACATGACGTTG	436	260	1	0	0	0
PDE1A	TGACACCACATGACCACA	349	359	0	0	0	0
PDE1B	TGACACCAACCACACACA	69	150	0	1	2	0
PDE1B	GTACACCAACCACAACCA	280	389	13	1	1	1
PDE1B	CAACACCAACCACACAAC	1	6	0	0	0	0
PDE1B	GTACACCAACCACACATG	261	204	1	0	0	3
PDE1B	TGACACCAACCACAACGT	159	140	0	0	0	0
PDE1B	GTTGCAACTGCAACCACA	193	184	0	0	0	0
PDE1C	CAACACCACATGACACTG	773	1420	0	1	1	0
PDE1C	GTACACCACATGACTGGT	88	137	0	0	0	0
PDE1C	TGTGGTGTACCAACGTCA	578	298	70	0	0	0
PDE1C	CATGCAGTTGGTACTGCA	61	114	0	0	0	0
PDE1C	TGTGCAGTTGGTACTGAC	36	22	0	0	0	0
PDE1C	TGTGCACACATGACACTG	235	255	1	0	0	0
PDE2A	TGACACCACATGTGGTAC	112	19	0	0	0	0
PDE2A	ACACACCACATGTGGTTG	1019	654	3	0	0	1
PDE2A	CAACACCACATGTGCAGT	527	411	0	0	0	1
PDE2A	ACACACCACATGTGCACA	43	67	0	0	0	0
PDE2A	GTACACCACATGTGCAAC	22	20	0	1	0	0
PDE2A	CATGCACACAGTGTGTTG	447	468	0	0	0	0
PDE3A	TGACACCACATGTGCATG	76	100	0	0	0	0
PDE3A	ACACACCACATGTGACGT	216	202	0	0	0	0
PDE3A	TGACACCACATGTGACCA	278	282	0	0	0	3
PDE3A	CAACACCACATGTGACAC	45	18	0	0	0	0
PDE3A	GTACACCACATGTGACTG	162	203	1	1	0	0
PDE3B	TGACACCAACGTGTGTGT	203	123	0	0	0	0
PDE3B	GTACACCAACGTGTGTCA	871	346	1	0	0	0
PDE3B	ACACACCAACGTGTGTAC	45	120	0	0	0	0
PDE3B	CAACACCAACGTGTGTTG	340	340	0	3	0	3

PDE3B	GTACACCAACGTGTCAGT	136	85	1	0	0	0
PDE4A	ACACACCAACGTGTACCA	99	148	0	0	0	0
PDE4A	GTACACCAACGTGTACAC	3	6	0	0	0	0
PDE4A	TGACACCAACGTGTACTG	253	351	0	0	0	0
PDE4A	ACACACCAACGTGTTGGT	48	76	0	0	0	0
PDE4A	TGACACCAACGTGTTGCA	371	263	0	0	0	0
PDE4A	CATGCACAACGTTGTGGT	729	389	0	0	0	0
PDE4B	TGTGCAGTTGGTACACGT	202	288	0	1	1	0
PDE4B	CAACACCAACGTCAGTAC	158	182	0	0	0	0
PDE4B	GTACACCAACGTCAGTTG	709	407	1	0	0	0
PDE4B	TGACACCAACGTCACAGT	238	304	0	0	0	0
PDE4B	ACTGGTGTACCAACGTGT	340	113	0	0	0	0
PDE4C	ACACACCAACGTCAACTG	486	823	1	0	1	0
PDE4C	CAACACCAACGTCAACCA	374	395	1	0	0	0
PDE4C	TGACACCAACGTCAACAC	17	9	0	0	0	0
PDE4C	CAACACCAACGTCATGGT	145	624	0	1	0	0
PDE4C	CATGCAGTTGGTACCAAC	109	85	0	0	0	0
PDE4C	GTTGCACACAGTGTCAGT	30	44	0	1	0	0
PDE4D	ACACACCAACGTCATGCA	153	221	0	0	0	0
PDE4D	GTACACCAACGTCATGAC	68	63	0	0	0	0
PDE4D	TGACACCAACGTCATGTG	345	542	1	0	0	0
PDE4D	CAACACCAACGTACGTGT	659	361	0	1	0	0
PDE4D	ACACACCAACGTACGTCA	191	159	0	0	0	0
PDE5A	CAACACCACATGCATGGT	699	1242	10	7531	1	1
PDE5A	ACACACCACATGCATGCA	97	26	0	0	0	0
PDE5A	GTACACCACATGCATGAC	93	200	0	0	0	0
PDE5A	TGACACCACATGCATGTG	278	306	0	0	0	0
PDE5A	CAACACCACATGACGTGT	571	951	1	0	0	0
PDE6A	CAACGTCAGTTGGTACAC	11	10	0	0	0	0
PDE6A	GTACGTCAGTTGGTACTG	1216	860	0	1	0	0
PDE6A	TGACGTCAGTTGGTTGGT	239	164	1	0	0	0
PDE6A	ACTGCAGTACCAACCAGT	862	429	3	0	0	2
PDE6A	TGTGCAGTACCAACCACA	175	171	0	0	0	0
PDE6A	ACTGCACACAACTGCAGT	210	242	1	1	0	0
PDE6B	GTACACCAACCACATGGT	598	1001	0	4	1	0
PDE6B	ACACACCAACCACAACAC	42	64	0	0	0	0
PDE6B	CAACACCAACCACAACTG	135	241	0	0	0	0
PDE6B	CAACACCAACCACATGCA	127	139	0	0	0	0
PDE6B	CATGCACACAGTGTCACA	184	197	0	0	0	1
PDE6B	TGTGCACACAGTGTCAAC	13	20	0	0	0	0
PDE6C	GTACACCAACGTACGTAC	36	155	0	0	0	0
PDE6C	TGACACCAACGTACGTTG	487	803	1	2	0	0
PDE6C	ACACACCAACGTACCAGT	93	83	0	1	0	0
PDE6C	TGACACCAACGTACCACA	87	52	0	0	0	0
PDE6C	CAACACCAACGTACCAAC	26	66	0	0	0	0
PDE6C	TGTGCAACACGTCAGTTG	579	594	1	0	0	1
PDE6D	CATGGTGTACCACATGAC	101	115	0	0	1	0
PDE6D	GTACACCAACGTACCATG	127	129	0	1	0	0
PDE6D	TGACACCAACGTACACGT	36	30	0	0	0	0
PDE6D	GTACACCAACGTACACCA	106	104	0	0	0	0
PDE6D	ACTGCAGTCAGTACTGAC	25	21	0	0	0	0
PDE6G	GTACACCAACGTACTGGT	271	211	1	0	1	0
PDE6G	ACACACCAACGTACACAC	24	37	0	0	0	0
PDE6G	CAACACCAACGTACACTG	131	189	0	0	0	1
PDE6G	CAACACCAACGTACTGCA	27	37	0	0	0	0
PDE6G	TGACACCAACGTACTGAC	54	50	1	0	0	0
PDE6H	CAACACCAACGTTGGTCA	365	207	0	1	1	0
PDE6H	ACACACCAACGTACTGTG	66	43	0	0	0	0
PDE6H	GTACACCAACGTTGGTGT	275	330	0	0	0	0
PDE6H	TGACACCAACGTTGGTAC	21	12	1	0	0	0
PDE6H	ACACACCAACGTTGGTTG	440	380	0	0	0	0
PDE6H	ACTGCACAACGTTGTGCA	52	106	0	1	0	0
PDE7A	GTTGCAACTGCAGTTGCA	220	287	0	0	1	0
PDE7A	CAACACCAACGTTGCAGT	46	58	0	0	0	0
PDE7A	ACACACCAACGTTGCACA	111	121	2	0	0	1
PDE7A	GTACACCAACGTTGCAAC	7	9	0	0	0	0
PDE7A	TGACACCAACGTTGCATG	201	125	0	0	0	0
PDE7A	CATGCAGTTGGTTGCAGT	20	35	0	0	0	0
PDE7B	TGACACCAACCAGTGTCA	142	76	0	1	0	0
PDE7B	CAACACCAACCAGTGTAC	68	67	0	0	0	0
PDE7B	GTACACCAACCAGTGTTG	401	564	0	0	0	0
PDE7B	TGACACCAACCAGTCAGT	111	129	0	1	0	0
PDE7B	GTACACCAACCAGTCACA	114	118	0	0	0	1
PDE8A	ACACACCAACGTTGACGT	68	266	0	0	0	0
PDE8A	TGACACCAACGTTGACCA	35	72	0	1	0	12

PDE8A	CAACACCAACGTTGACAC	18	15	0	0	0	0
PDE8A	GTACACCAACGTTGACTG	417	512	0	0	0	7
PDE8A	GTTGCAGTCATGGTGTGT	170	195	0	0	0	1
PDE8B	GTTGGTGTACCACATGTG	692	1503	1	1	1	6
PDE8B	GTACACCACATGCAACGT	535	370	0	1	0	1
PDE8B	CAACACCACATGCAACCA	413	357	0	0	0	0
PDE8B	TGACACCACATGCAACAC	42	34	0	0	0	0
PDE8B	ACACACCACATGCAACTG	443	757	1	0	0	7
PDGFA	ACACGTTGTGCAACCATG	206	181	68	51	43	113
PDGFA	TGACGTTGTGCAACCAAC	45	24	0	0	0	0
PDGFA	CAACGTTGTGCAACACGT	138	128	0	0	0	0
PDGFA	CATGGTACCACACAGTTG	508	886	1	1	0	0
PDGFA	GTTGGTACCACACACAGT	212	214	0	1	0	0
PDGFB	GTACCATGGTACTGACCA	55	135	0	0	0	0
PDGFB	ACACCATGGTACTGACAC	18	22	0	0	0	0
PDGFB	CAACCATGGTACTGACTG	329	433	0	1	0	6
PDGFB	GTACCATGGTACTGTGGT	270	349	0	0	0	1
PDGFB	CAACCATGGTACTGTGCA	88	115	0	0	0	0
PDGFB	ACTGCACACAGTGTCATG	262	281	0	0	0	3
PDGFRA	CACATGCATGTGTGACCA	502	414	1	2	0	1
PDGFRA	TGCATGCATGTGTGACAC	35	144	0	0	0	0
PDGFRA	ACCATGCATGTGTGACTG	361	379	1	0	0	4
PDGFRA	CACATGCATGTGTGTGGT	304	294	0	0	0	0
PDGFRA	ACCATGCATGTGTGTGCA	258	237	0	0	0	0
PDGFRB	CACATGACGTTGCAGTTG	954	750	4	0	1	38
PDGFRB	GTCATGACGTTGCAGTCA	167	799	1	0	0	2
PDGFRB	ACCATGACGTTGCAGTAC	111	71	0	0	0	0
PDGFRB	GTCATGACGTTGCACAGT	66	73	0	0	0	0
PDGFRB	CACATGACGTTGCACACA	92	71	0	0	0	0
PDHA1	CATGCACACAGTGTACGT	351	547	1	0	1	0
PDHA1	ACACCAGTGTCAACCAGT	292	547	4	0	0	0
PDHA1	TGACCAGTGTCAACCACA	17	9	0	0	0	0
PDHA1	CAACCAGTGTCAACCAAC	56	46	0	0	0	0
PDHA1	GTACCAGTGTCAACCATG	355	415	1	0	0	46
PDHA1	TGACCAGTGTCAACACGT	7	20	0	0	0	0
PDHA2	TGACCAGTGTCACATGTG	1257	1506	3	504	3	0
PDHA2	ACACCAGTGTCACAACTG	288	657	1	0	0	0
PDHA2	CAACCAGTGTCACATGGT	361	613	0	1	0	3
PDHA2	ACACCAGTGTCACATGCA	181	336	0	0	0	1
PDHA2	GTACCAGTGTCACATGAC	13	36	0	0	0	0
PDHB	GTACCAGTGTCACAGTTG	293	400	2	0	1	0
PDHB	TGACCAGTGTCACAGTCA	686	667	1	0	0	0
PDHB	CAACCAGTGTCACAGTAC	51	76	0	10	0	0
PDHB	TGACCAGTGTCACACAGT	155	174	0	0	0	0
PDHB	GTACCAGTGTCACACACA	171	174	0	1	0	0
PDHB	ACTGCACACAGTGTACCA	55	89	0	0	0	0
PDHX	TGACGTTGTGTGTGGTTG	371	429	2	1	0	1
PDHX	ACACGTTGTGTGTGCAGT	62	42	0	0	0	0
PDHX	TGACGTTGTGTGTGCACA	153	136	2	0	0	1
PDHX	CAACGTTGTGTGTGCAAC	22	20	1	0	0	0
PDHX	GTACGTTGTGTGTGCATG	186	117	0	0	0	332
PDHX	GTTGCACACAGTGTACAC	24	166	0	0	0	1
PDIA3	ACACGTCAGTACTGTGAC	170	72	0	0	0	0
PDIA3	CATGGTCATGTGTGTGTG	730	445	3	1978	0	0
PDIA3	GTTGGTACGTGTGTGTGT	464	491	1	0	0	1
PDIA3	CATGGTACGTGTGTGTCA	862	513	3	2	0	0
PDIA3	TGTGGTACGTGTGTGTAC	7	4	0	0	0	0
PDK1	CAACGTGTGTCAGTGTCA	703	787	0	0	4	3
PDK1	TGACGTGTGTGTTGTGTG	711	249	1	0	1	0
PDK1	GTACGTGTGTGTTGTGAC	15	13	0	0	0	0
PDK1	GTACGTGTGTCAGTGTGT	830	889	966	2	0	0
PDK1	TGTGGTACTGACCACAGT	194	130	0	0	0	0
PDK2	TGCATGACCACAGTACAC	55	154	0	0	0	0
PDK2	ACCATGACCACAGTACTG	519	720	0	0	0	0
PDK2	CACATGACCACAGTTGGT	610	526	0	0	0	0
PDK2	ACCATGACCACAGTTGCA	504	501	1	1	0	0
PDK2	GTCATGACCACAGTTGAC	24	25	0	0	0	12
PDK2	CATGCATGCAGTCACAGT	122	138	0	0	0	0
PDLIM4	GTACTGTGGTACCATGGT	1211	595	0	0	0	0
PDLIM4	CAACTGTGGTACCATGCA	124	184	2	0	0	0
PDLIM4	TGACTGTGGTACCATGAC	14	20	0	0	0	0
PDLIM4	ACACTGTGGTACCATGTG	445	447	1	0	0	1
PDLIM4	GTACTGTGGTACACGTGT	197	214	1	0	0	0
PDLIM4	ACTGCAACCACACAGTGT	521	191	2	1	0	0
PDLIM5	TGACCAGTTGACACTGAC	45	48	0	0	0	0

PDLIM5	ACTGGTGTGTTGTGGTCA	522	521	0	0	0	0
PDLIM5	GTTGGTGTGTTGTGGTAC	71	55	0	0	0	0
PDLIM5	TGTGCAGTGTTGGTTGCA	64	92	0	1	0	0
PDLIM5	TGTGACGTTGACTGTGGT	1027	301	0	0	0	0
PDPK1	GTCATGACGTGTGTACGT	283	154	0	0	0	0
PDPK1	CACATGACGTGTGTACCA	121	224	0	0	0	0
PDPK1	TGCATGACGTGTGTACAC	11	16	0	0	0	0
PDPK1	ACCATGACGTGTGTACTG	249	506	0	0	0	8
PDPK1	CACATGACGTGTGTTGGT	69	87	0	0	0	0
PDXK	GTTGGTTGACTGGTCACA	45	69	0	1	3	3438
PDXK	ACTGGTTGACTGGTCAAC	18	18	0	0	0	0
PDXK	CATGGTTGACTGGTCATG	45	49	0	0	0	0
PDXK	GTTGGTTGACTGGTACGT	423	266	0	0	0	3
PDXK	CATGGTTGACTGGTACCA	388	585	1	0	0	0
PDXP	ACACACCATGACCATGCA	907	638	0	2	0	0
PDXP	GTACACCATGACCATGAC	238	216	1	1	0	0
PDXP	TGACACCATGACCATGTG	1041	933	1	1	0	0
PDXP	GTTGGTTGTGACGTCAAC	7	19	0	0	0	0
PDXP	TGTGGTTGTGACGTCATG	113	264	0	0	0	0
PDZK1	CAACTGCACAGTTGGTGT	336	438	0	4	0	0
PDZK1	ACACTGCACAGTTGGTCA	211	216	0	0	0	0
PDZK1	GTACTGCACAGTTGGTAC	21	21	0	0	0	0
PDZK1	TGACTGCACAGTTGGTTG	487	168	0	0	0	0
PDZK1	ACTGGTGTCACAACCACA	39	45	0	0	0	0
PEBP1	ACACTGTGACACACTGGT	593	710	1	0	1	0
PEBP1	TGACTGCAGTGTTGCATG	80	71	0	0	0	0
PEBP1	ACACTGCAGTGTTGACGT	268	210	1	0	0	0
PEBP1	TGACTGCAGTGTTGACCA	185	175	0	1	0	0
PEBP1	CAACTGCAGTGTTGACAC	262	82	0	0	0	0
PECAM1	GTACTGGTGTTGCATGTG	729	713	4	0	2	1
PECAM1	TGACTGGTGTTGACGTCA	218	242	0	0	2	0
PECAM1	TGACTGGTGTTGCATGCA	60	92	1	0	0	0
PECAM1	CAACTGGTGTTGCATGAC	59	69	0	0	0	2
PECAM1	ACACTGGTGTTGACGTGT	332	254	93	0	0	0
PECR	ACACACGTTGTGCACAAC	42	53	0	0	0	0
PECR	CAACACGTTGTGCACATG	542	618	0	0	0	3
PECR	GTACACGTTGTGCAACGT	295	464	1	0	0	0
PECR	CAACACGTTGTGCAACCA	587	788	0	0	0	0
PECR	TGACACGTTGTGCAACAC	35	24	0	0	0	0
PEG3	TGACGTTGCAGTTGTGTG	204	197	0	0	0	0
PEG3	GTACGTTGCACAGTGTGT	208	366	0	0	0	0
PEG3	CAACGTTGCACAGTGTCA	656	506	0	0	0	0
PEG3	TGACGTTGCACAGTGTAC	85	50	0	0	0	0
PEG3	ACTGCATGCAACACTGAC	20	15	0	0	0	0
PELI1	TGTGCACATGCATGCATG	264	150	0	1	1	0
PELI1	TGTGGTACCACACAGTGT	143	162	0	0	0	0
PELI1	GTTGGTACCACACAGTCA	241	266	0	0	0	0
PELI1	ACTGGTACCACACAGTAC	120	131	0	0	0	0
PELI1	ACTGCACATGCATGACGT	336	332	0	0	0	0
PELI1	TGTGCACATGCATGACCA	221	203	0	0	0	0
PELI2	ACTGGTACACTGACGTAC	58	61	0	0	1	0
PELI2	CATGGTACACTGACGTTG	531	308	0	1	1	0
PELI2	GTTGGTACACTGACCAGT	205	213	0	0	0	0
PELI2	CATGGTACACTGACCACA	41	72	0	1	0	0
PELI2	TGTGGTACACTGACCAAC	43	46	0	0	0	0
PELI3	GTTGGTCATGCATGACCA	117	138	0	0	1	0
PELI3	CATGGTCATGCATGACTG	1063	1282	2	0	1	1
PELI3	TGTGGTCATGCATGACGT	114	131	0	0	0	2
PELI3	ACTGGTCATGCATGACAC	57	68	0	0	0	1
PELI3	GTTGGTCATGCATGTGGT	1324	614	0	1	0	0
PELI3	CATGCAACACGTACTGTG	26	46	0	0	0	0
PELP1	ACTGGTCATGACGTCAAC	41	76	0	0	0	0
PELP1	ACTGGTACGTTGGTGTGT	227	254	0	0	0	0
PELP1	TGTGGTACGTTGGTGTCA	235	194	0	0	0	0
PELP1	CATGGTACGTTGGTGTAC	48	39	0	0	0	0
PELP1	GTTGGTACGTTGGTGTTG	468	378	0	0	0	0
PELP1	TGTGCAACTGCAGTTGGT	203	243	1	0	0	0
PEMT	ACACCACATGACACACTG	438	897	0	0	0	0
PEMT	CAACCACATGACACTGGT	994	489	1	1	0	0
PEMT	ACACCACATGACACTGCA	342	305	27	0	0	0
PEMT	GTACCACATGACACTGAC	62	43	0	0	0	0
PEMT	TGACCACATGACACTGTG	466	309	3	0	0	0
PEMT	GTTGCAACACTGGTACTG	664	141	0	0	0	1
PENK	ACTGGTCAGTCACACAGT	52	49	0	0	0	0
PENK	TGTGGTCAGTCACACACA	106	77	0	0	0	2

PENK	CATGGTCAGTCACACAAC	54	53	0	1	0	0
PENK	GTTGGTCAGTCACACATG	147	165	0	2	0	0
PENK	TGTGGTCAGTCACAACGT	88	53	0	0	0	0
PER1	CAACTGTGGTACACGTCA	264	216	1	0	0	0
PER1	TGACTGTGGTACACGTAC	57	50	0	0	0	0
PER1	ACACTGTGGTACACGTTG	388	444	0	0	0	0
PER1	CAACTGTGGTACACCAGT	36	33	0	0	0	0
PER1	ACACTGTGGTACACCACA	99	97	0	0	0	2
PER1	TGTGCACACAGTGTACTG	130	183	0	0	0	0
PER2	TGACGTACTGGTACCAAC	22	66	0	0	0	0
PER2	ACACGTACTGGTACCATG	145	119	0	0	0	0
PER2	CAACGTACTGGTACACGT	229	86	0	0	0	0
PER2	ACACGTACTGGTACACCA	919	893	0	2	0	5
PER2	GTACGTACTGGTACACAC	105	42	0	0	0	0
PER3	CAACGTACTGGTCACAGT	144	99	0	0	0	0
PER3	ACACGTACTGGTCACACA	74	157	0	0	0	0
PER3	GTACGTACTGGTCACAAC	3	5	0	0	0	0
PER3	TGACGTACTGGTCACATG	266	177	1	0	0	0
PER3	ACACGTACTGGTCAACGT	97	181	0	0	0	0
PER3	TGTGCACATGGTCAGTGT	460	497	0	0	0	0
PERP	CATGGTCACAACGTCAAC	21	34	1	0	0	0
PERP	GTTGGTCACAACGTCATG	51	80	1	0	0	0
PERP	TGTGGTCACAACGTACGT	111	172	1	0	0	0
PERP	GTTGGTCACAACGTACCA	220	135	0	0	0	0
PERP	ACTGGTCACAACGTACAC	45	43	0	0	0	0
PERP	GTTGCATGGTTGTGCAGT	86	105	0	0	0	0
PES1	TGTGGTGTTGTGCACACA	340	166	0	2	1	0
PES1	TGTGGTGTTGTGCAGTTG	675	1311	2	1	0	0
PES1	ACTGGTGTTGTGCACAGT	289	489	1	1	0	0
PES1	CATGGTGTTGTGCACAAC	98	27	0	0	0	0
PES1	ACTGCACAGTGTACGTTG	865	413	0	0	0	0
PES1	TGTGCAACACACACGTTG	95	193	1	0	0	0
PF4	GTACTGGTCAGTCATGAC	18	56	0	0	0	0
PF4	TGACTGGTCAGTCATGTG	528	469	1	1	0	0
PF4	CAACTGGTCAGTACGTGT	387	356	0	0	0	0
PF4	ACACTGGTCAGTACGTCA	102	179	0	0	0	2
PF4	CATGCAGTTGTGACCACA	67	67	0	0	0	0
PF4	ACTGCACACAGTGTTGGT	99	154	0	0	0	0
PFDN2	ACTGGTACTGGTCACAGT	250	106	0	0	0	0
PFDN2	TGTGGTACTGGTCACACA	82	60	0	0	0	1
PFDN2	CATGGTACTGGTCACAAC	3	7	0	0	0	0
PFDN2	GTTGGTACTGGTCACATG	118	134	0	1	0	1
PFDN2	TGTGGTACTGGTCAACGT	28	52	0	0	0	0
PFKFB1	ACACGTTGTGACTGACGT	173	139	0	0	0	0
PFKFB1	TGACCAACACTGGTGTGT	335	378	1	0	0	0
PFKFB1	GTACCAACACTGGTGTCA	179	174	0	1	0	0
PFKFB1	ACACCAACACTGGTGTAC	49	26	0	0	0	0
PFKFB1	CAACCAACACTGGTGTTG	359	642	0	2	0	0
PFKFB3	GTACGTGTACGTCACAGT	162	80	0	0	1	0
PFKFB3	CAACGTGTACGTCAGTTG	313	271	0	0	0	0
PFKFB3	CAACGTGTACGTCACACA	159	121	0	0	0	0
PFKFB3	TGACGTGTACGTCACAAC	9	1	0	0	0	0
PFKFB3	ACACGTGTACGTCACATG	163	376	0	0	0	0
PFKFB4	GTACCAACTGGTGTTGCA	361	214	2	1	0	0
PFKFB4	ACACCAACTGGTGTTGAC	45	77	0	0	0	0
PFKFB4	CAACCAACTGGTGTTGTG	218	615	0	1	0	0
PFKFB4	TGACCAACTGGTCAGTGT	183	548	0	1	0	0
PFKFB4	GTACCAACTGGTCAGTCA	48	70	0	0	0	0
PFKFB4	ACTGCAACCACACACAAC	8	19	0	0	0	0
PFKL	CATGGTACTGCATGTGGT	513	542	2	0	0	0
PFKL	ACTGGTACTGCATGTGCA	195	154	0	0	0	0
PFKL	GTTGGTACTGCATGTGAC	50	44	0	0	0	0
PFKL	TGTGGTACTGCATGTGTG	198	271	0	0	0	0
PFKL	TGTGGTACTGACGTGTGT	217	153	0	0	0	0
PFKM	ACACCAACTGGTCAGTAC	639	203	0	1	0	0
PFKM	CAACCAACTGGTCAGTTG	518	319	0	1	0	0
PFKM	GTACCAACTGGTCACAGT	417	798	1	0	0	0
PFKM	CAACCAACTGGTCACACA	12	30	0	0	0	0
PFKM	TGACCAACTGGTCACAAC	3	0	0	0	0	0
PFKP	ACACCAACTGGTCACATG	319	377	0	0	0	0
PFKP	CAACCAACTGGTCAACGT	350	367	0	1	0	0
PFKP	ACACCAACTGGTCAACCA	702	799	0	4	0	1
PFKP	GTACCAACTGGTCAACAC	148	46	0	1	0	0
PFKP	CATGGTACTGTGACCATG	85	75	1	0	0	30
PFN1	CAACGTCAACCACACACA	202	373	1	0	3	0

PFN1	GTACGTCAACCACACAGT	129	66	0	0	0	0
PFN1	GTACTGACGTCAACCATG	64	66	0	0	0	0
PFN1	TGACTGACGTCAACACGT	8	10	0	0	0	0
PFN1	GTACTGACGTCAACACCA	176	250	0	0	0	1
PFN1	GTTGCAACGTACACACGT	140	75	1	0	0	8
PFN2	ACACTGACGTCACATGCA	255	126	0	1	0	0
PFN2	GTACTGACGTCACATGAC	47	59	0	0	0	0
PFN2	TGACTGACGTCACATGTG	749	613	2	1	0	1
PFN2	CAACTGACGTCAACGTGT	471	508	2	0	0	0
PFN2	ACACTGACGTCAACGTCA	162	506	0	2	0	0
PGA3	ACTGCAGTGTACGTGTGT	434	1508	1	1	1	0
PGA3	TGTGCAGTGTACGTGTCA	718	482	1	1	1	4
PGA3	CATGCAGTGTACGTGTAC	56	79	0	0	0	0
PGA3	ACTGCAGTCAGTGTCAAC	26	79	0	0	0	0
PGA3	CATGCAGTCAGTGTCATG	240	390	1	0	0	2
PGA4	CATGCAGTCAGTGTACCA	435	494	5	1	0	3330
PGA4	TGTGCAGTCAGTGTACAC	38	58	0	0	0	0
PGA4	CATGACACCAACACACCA	150	155	0	1	0	0
PGA4	TGTGACACCAACACACAC	83	92	0	0	0	0
PGA4	GTTGCAGTCAGTGTACGT	295	313	0	0	0	0
PGA5	CAACTGTGGTCACAACGT	426	340	2	1	0	0
PGA5	ACACTGTGGTCACAACCA	242	219	0	0	0	0
PGA5	GTACTGTGGTCACAACAC	56	159	0	0	0	0
PGA5	TGACTGTGGTCACAACTG	531	655	1	0	0	0
PGA5	ACTGCAGTGTCATGTGTG	87	64	0	0	0	0
PGA5	TGTGCAACTGGTTGCAGT	249	767	0	1	0	5
PGAM1	TGACCAGTACTGTGTGTG	539	550	4	3	1	0
PGAM1	GTACCAGTACTGTGTGAC	57	33	0	0	0	0
PGAM1	ACACCAGTTGGTGTGTGT	623	866	17622	21	0	1
PGAM1	TGACCAGTTGGTGTGTCA	92	101	0	0	0	0
PGAM1	CAACCAGTTGGTGTGTAC	123	131	0	0	0	0
PGAM1	ACTGCATGCAGTCACACA	15	33	0	0	0	0
PGAP1	GTACACCAGTACGTACCA	305	128	40	54	15	66
PGAP1	TGACACCAGTACGTACGT	75	124	0	0	0	0
PGAP1	ACACACCAGTACGTACAC	33	28	0	0	0	0
PGAP1	CAACACCAGTACGTACTG	422	407	1	0	0	1
PGAP1	GTACACCAGTACGTTGGT	160	208	0	0	0	0
PGD	ACACCAGTGTCAGTTGGT	780	763	0	0	0	1
PGD	TGACCAGTGTCAGTTGCA	434	231	1	1	0	0
PGD	CAACCAGTGTCAGTTGAC	23	37	0	0	0	0
PGD	GTACCAGTGTCAGTTGTG	700	950	3	0	0	0
PGD	ACACCAGTGTCACAGTGT	1020	1117	0	2	0	6
PGDS	ACACACGTCAACGTGTCA	88	122	0	1	1	0
PGDS	TGACACGTCAACGTGTTG	240	302	3	2	1	10
PGDS	GTACACGTCAACGTGTAC	12	6	0	0	0	0
PGDS	ACACACGTCAACGTCAGT	293	219	3	0	0	0
PGDS	GTTGGTCACATGGTTGAC	39	90	1	1	0	0
PGF	CAACTGGTCATGTGTGGT	777	846	1	0	0	0
PGF	ACACTGGTCATGTGTGCA	284	338	1	0	0	0
PGF	GTACTGGTCATGTGTGAC	24	31	0	0	0	0
PGF	TGACTGGTCATGTGTGTG	75	161	0	1	0	0
PGF	ACTGCAGTCACATGGTGT	1047	1061	2	1	0	1
PGK1	TGCATGTGTGTGTGTGGT	35	46	0	0	0	0
PGK1	GTCATGTGTGTGTGTGCA	318	451	0	0	0	8
PGK1	ACCATGTGTGTGTGTGAC	86	91	0	0	0	0
PGK1	CACATGTGTGTGTGTGTG	343	410	0	0	0	0
PGK1	CAACGTGTGTGTGTGTGT	336	445	1	0	0	0
PGK1	CATGCAACACACACCAAC	48	71	0	0	0	1
PGK2	GTACCAACTGGTCATGTG	778	161	0	0	1	0
PGK2	TGACCAACTGGTCAACTG	136	146	0	0	0	0
PGK2	ACACCAACTGGTCATGGT	307	642	0	0	0	29
PGK2	TGACCAACTGGTCATGCA	46	54	0	0	0	0
PGK2	CAACCAACTGGTCATGAC	57	64	0	0	0	22
PGK2	GTTGCATGCAGTCACAAC	8	3	0	0	0	0
PGLS	ACACACACCAGTACACGT	33	63	0	0	0	0
PGLS	TGACACACCAGTACACCA	130	282	1	0	0	0
PGLS	CAACACACCAGTACACAC	267	152	1	0	0	0
PGLS	GTACACACCAGTACACTG	289	223	0	0	0	5
PGLS	TGACACACCAGTACTGGT	258	260	1	0	0	0
PGLYRP2	CAACACACGTGTGTACTG	141	175	0	0	0	0
PGLYRP2	GTACACACGTGTGTTGGT	654	768	0	1	0	1
PGLYRP2	CAACACACGTGTGTTGCA	182	138	0	0	0	0
PGLYRP2	TGACACACGTGTGTTGAC	21	38	0	0	0	0
PGLYRP2	GTTGCAGTTGGTTGACTG	631	800	0	1	0	2
PGLYRP2	CATGCAACACGTACCAGT	382	351	1	0	0	0

PGM1	TGACACCAACCATGACTG	676	602	2	706	18	0
PGM1	GTACACCAACCATGACAC	135	91	0	0	0	0
PGM1	ACACACCAACCATGTGGT	1054	748	1	1	0	1
PGM1	TGACACCAACCATGTGCA	106	113	0	1	0	0
PGM1	CAACACCAACCATGTGAC	75	64	0	0	0	0
PGM1	TGTGCAACACGTCATGAC	50	34	0	1	0	0
PGM3	GTACACCAACACGTGTGT	574	737	1	2	1	1
PGM3	GTTGCACAACTGGTTGTG	1088	662	0	1	1	5
PGM3	GTACACCAACCATGTGTG	103	53	0	0	0	0
PGM3	CAACACCAACACGTGTCA	117	172	0	0	0	0
PGM3	TGACACCAACACGTGTAC	94	43	0	0	0	0
PGM3	ACACACCAACACGTGTTG	323	374	2	0	0	0
PGR	TGCATGACACTGCAGTCA	1023	551	0	0	0	6
PGR	CACATGACACTGCAGTAC	326	24	0	0	0	0
PGR	GTCATGACACTGCAGTTG	1299	1383	2	1	0	3
PGR	TGCATGACACTGCACAGT	64	61	0	0	0	0
PGR	CATGACACGTCACAGTCA	100	122	0	0	0	0
PHB2	ACACTGCAACCAACACCA	607	471	0	0	0	0
PHB2	GTACTGCAACCAACACAC	9	14	0	0	0	0
PHB2	TGACTGCAACCAACACTG	101	150	0	0	0	0
PHB2	ACACTGCAACCAACTGGT	137	114	0	0	0	0
PHB2	TGACTGCAACCAACTGCA	227	337	0	0	0	0
PHB2	ACTGCATGCAGTCATGAC	15	18	0	0	0	0
PHC1	GTTGGTACCACAGTACTG	239	168	0	0	0	0
PHC1	TGTGGTACCACAGTTGGT	112	162	0	0	0	0
PHC1	GTTGGTACCACAGTTGCA	471	68	0	0	0	0
PHC1	ACTGGTACCACAGTTGAC	480	128	0	0	0	0
PHC1	CATGGTACCACAGTTGTG	78	112	0	0	0	0
PHCA	GTCATGTGACGTTGTGTG	221	71	1	0	0	0
PHCA	CACATGTGACCAGTGTGT	22	9	0	0	0	0
PHCA	ACCATGTGACCAGTGTCA	773	473	0	1	0	0
PHCA	GTCATGTGACCAGTGTAC	113	150	0	0	0	0
PHCA	CATGGTCACAACCAGTCA	201	363	2	0	0	0
PHF1	GTACGTACTGACGTCACA	40	91	3	0	1	1
PHF1	TGACGTACTGACGTCAGT	117	121	0	1	0	1
PHF1	ACACGTACTGACGTCAAC	35	27	0	0	0	3
PHF1	CAACGTACTGACGTCATG	250	86	0	0	0	0
PHF1	GTACGTACTGACGTACGT	65	39	0	0	0	0
PHF1	TGTGCACATGTGTGGTGT	716	942	3	0	0	1
PHF11	TGACGTTGGTTGCAGTTG	533	603	0	0	0	0
PHF11	ACACGTTGGTTGCACAGT	321	306	1	1	0	4
PHF11	TGACGTTGGTTGCACACA	53	27	0	0	0	0
PHF11	CAACGTTGGTTGCACAAC	65	25	0	0	0	0
PHF11	GTTGACCATGACACTGAC	66	113	0	0	0	0
PHF21A	GTACCAACACACACGTGT	730	555	3	0	1	0
PHF21A	TGACCAACACACCATGAC	95	107	0	0	0	0
PHF21A	ACACCAACACACCATGTG	380	326	0	1	0	4
PHF21A	CAACCAACACACACGTCA	285	383	0	0	0	0
PHF21A	TGACCAACACACACGTAC	28	18	0	0	0	3
PHF21A	ACTGCAACCACAACGTCA	455	337	0	0	0	1
PHKA1	GTACGTGTGTGTGTCATG	346	102	0	3	1	0
PHKA1	TGACGTGTGTGTGTCACA	44	83	0	0	0	0
PHKA1	CAACGTGTGTGTGTCAAC	20	12	0	0	0	0
PHKA1	TGACGTGTGTGTGTACGT	130	144	0	0	0	0
PHKA1	GTACGTGTGTGTGTACCA	130	94	0	0	0	0
PHKA2	ACACGTGTGTGTGTACAC	5	0	0	0	0	0
PHKA2	CAACGTGTGTGTGTACTG	786	333	2	0	0	0
PHKA2	GTACGTGTGTGTGTTGGT	177	241	0	0	0	5
PHKA2	CAACGTGTGTGTGTTGCA	108	143	0	0	0	0
PHKA2	GTTGGTACTGTGACGTTG	241	181	0	0	0	0
PHKA2	CATGCACACAGTGTTGAC	96	51	0	0	0	0
PHKB	TGACGTGTGTGTGTTGAC	41	14	0	0	0	0
PHKB	ACACGTGTGTGTGTTGTG	121	116	0	0	0	0
PHKB	GTACGTGTGTGTCAGTGT	334	254	1	0	0	0
PHKB	CAACGTGTGTGTCAGTCA	410	225	3	0	0	1
PHKB	TGACGTGTGTGTCAGTAC	24	23	0	0	0	0
PHKG1	GTTGGTTGACACACCAGT	345	449	1	1	2	0
PHKG1	ACACGTGTGTGTCAGTTG	737	542	0	0	0	0
PHKG1	CAACGTGTGTGTCACAGT	79	33	0	0	0	0
PHKG1	ACACGTGTGTGTCACACA	55	86	0	2	0	1
PHKG1	GTACGTGTGTGTCACAAC	30	47	0	0	0	0
PHPT1	CAACACACGTCATGGTGT	807	1238	0	1	1	0
PHPT1	TGACACACGTCAACTGTG	268	659	0	0	0	53
PHPT1	ACACACACGTCATGGTCA	345	346	0	0	0	0
PHPT1	GTACACACGTCATGGTAC	34	169	0	1	0	0

PHPT1	ACTGACACACACGTTGCA	210	215	0	0	0	0
PI3	TGACTGTGGTCAGTTGGT	690	846	6	36	45	0
PI3	GTACTGTGGTCAGTACTG	1501	1025	4	1	0	0
PI3	GTACTGTGGTCAGTTGCA	111	127	3	0	0	0
PI3	ACACTGTGGTCAGTTGAC	142	44	0	0	0	0
PI3	CAACTGTGGTCAGTTGTG	94	433	0	0	0	0
PI3	GTTGCATGCAGTTGGTTG	235	438	1	0	0	0
PI4KB	TGTGCACACAGTCAGTCA	569	489	7	1	10	8
PI4KB	CACATGTGACTGCAGTTG	332	394	0	0	1	3
PI4KB	GTCATGTGACTGCACAGT	143	156	2	14	1	9
PI4KB	ACCATGTGACTGCAGTAC	161	198	0	10	0	0
PI4KB	CACATGTGACTGCACACA	83	56	0	0	0	2
PI4KB	TGCATGTGACTGCACAAC	41	34	0	0	0	0
PIAS1	CACATGTGGTTGACTGAC	72	98	1	0	0	0
PIAS1	GTCATGTGGTTGACTGTG	742	257	0	1	0	0
PIAS1	ACCATGTGGTTGTGGTGT	379	349	6	2487	0	2
PIAS1	TGCATGTGGTTGTGGTCA	83	42	1	0	0	0
PIAS1	CATGCAGTGTGTACACGT	96	73	0	1	0	0
PIAS1	CATGCACAACTGTGCAGT	117	200	1	0	0	1
PIAS3	GTACGTTGCAACTGACAC	75	129	1	0	0	0
PIAS3	TGACGTTGCAACTGACTG	391	647	0	0	0	0
PIAS3	ACACGTTGCAACTGTGGT	106	115	1	0	0	0
PIAS3	TGACGTTGCAACTGTGCA	443	175	0	7	0	4
PIAS3	CAACGTTGCAACTGTGAC	59	82	0	1	0	0
PIAS3	TGTGCATGCAGTCACATG	137	118	1	0	0	0
PIAS4	GTCATGTGGTTGACCAGT	60	81	0	0	0	0
PIAS4	CACATGTGGTTGACCACA	139	200	1	0	0	20
PIAS4	TGCATGTGGTTGACCAAC	24	27	0	0	0	0
PIAS4	ACCATGTGGTTGACCATG	155	192	1	0	0	0
PIAS4	CACATGTGGTTGACACGT	75	50	0	0	0	0
PIAS4	TGTGCAACTGACCATGAC	40	39	0	0	0	0
PICALM	GTTGGTGTACGTTGTGAC	7	17	0	0	0	0
PICALM	CATGGTCAGTTGACCACA	104	71	1	0	0	0
PICALM	TGTGGTCAGTTGACCAAC	72	155	0	0	0	0
PICALM	ACTGGTCAGTTGACCATG	89	155	0	0	0	7
PICALM	CATGGTCAGTTGACACGT	87	86	0	0	0	0
PIF1	GTTGGTTGTGTGACTGTG	298	280	0	0	1	0
PIF1	TGTGGTTGTGTGACACTG	161	221	0	0	0	0
PIF1	ACTGGTTGTGTGACTGGT	332	263	1	0	0	6
PIF1	TGTGGTTGTGTGACTGCA	47	76	0	0	0	0
PIF1	CATGGTTGTGTGACTGAC	27	30	0	0	0	0
PIGA	CAACTGTGCACAACACGT	39	28	0	0	0	0
PIGA	CAACTGTGTGACCATGAC	144	183	0	1	0	0
PIGA	GTACTGTGTGACCATGTG	49	89	1	0	0	0
PIGA	ACACTGTGTGACACGTGT	88	101	0	0	0	0
PIGA	TGACTGTGTGACACGTCA	75	53	0	0	0	0
PIGB	GTTGGTACACACACCACA	32	22	0	0	0	0
PIGB	ACTGGTACACACACCAAC	84	58	1	0	0	0
PIGB	CATGGTACACACACCATG	50	36	0	0	0	0
PIGB	GTTGGTACACACACACGT	157	107	0	0	0	1
PIGB	CATGGTACACACACACCA	685	331	0	0	0	0
PIGC	ACTGCAACTGCACACATG	156	225	0	95	8	0
PIGC	GTTGGTTGGTCAGTACTG	379	223	1	1	0	1
PIGC	TGTGGTTGGTCAGTTGGT	869	752	1	0	0	2
PIGC	GTTGGTTGGTCAGTTGCA	183	169	0	0	0	0
PIGC	ACTGGTTGGTCAGTTGAC	18	36	0	0	0	0
PIGC	CATGGTTGGTCAGTTGTG	281	345	0	0	0	0
PIGF	ACACTGTGCACAACACCA	165	154	1	0	2	0
PIGF	GTTGGTACCAGTCATGCA	422	347	2	0	0	0
PIGF	ACTGGTACCAGTCATGAC	34	36	0	0	0	0
PIGF	CATGGTACCAGTCATGTG	752	1298	2	2	0	0
PIGF	TGTGGTACCAGTACGTGT	366	431	4	0	0	0
PIGG	CATGGTCACATGTGGTTG	424	217	1	1	1	0
PIGG	GTTGGTCACATGTGGTCA	307	249	0	0	0	0
PIGG	ACTGGTCACATGTGGTAC	121	175	0	0	0	0
PIGG	GTTGGTCACATGTGCAGT	150	107	0	0	0	0
PIGG	CATGGTCACATGTGCACA	79	92	0	0	0	7
PIGG	TGTGCATGGTTGTGCAAC	26	55	0	0	0	0
PIGH	ACTGGTACTGGTTGTGGT	742	1024	2	0	0	0
PIGH	TGTGGTACTGGTTGTGCA	165	351	1	0	0	0
PIGH	CATGGTACTGGTTGTGAC	72	93	0	0	0	0
PIGH	GTTGGTACTGGTTGTGTG	278	269	0	1	0	0
PIGH	CATGGTACTGCAGTGTGT	1015	769	2	0	0	13
PIGK	CAACACCATGACACTGCA	191	273	0	1	0	3
PIGK	TGACACCATGACACTGAC	29	34	0	0	0	0

PIGK	ACACACCATGACACTGTG	507	163	0	0	0	0
PIGK	GTACACCATGACTGGTGT	76	52	0	0	0	0
PIGK	CAACACCATGACTGGTCA	228	324	0	1	0	0
PIGL	TGACACACCATGACCAGT	490	404	2	0	1	0
PIGL	GTACACACCATGACCACA	71	68	0	0	0	0
PIGL	ACACACACCATGACCAAC	83	104	0	0	0	0
PIGL	CAACACACCATGACCATG	372	341	1	0	0	1
PIGL	GTACACACCATGACACGT	115	69	0	0	0	0
PIGM	GTTGGTACACACCATGTG	887	518	1	2	1	4
PIGM	ACTGGTACACACCATGGT	457	147	1	0	0	0
PIGM	TGTGGTACACACCATGCA	18	39	0	0	0	0
PIGM	CATGGTACACACCATGAC	8	15	0	0	0	0
PIGM	TGTGGTTGACGTGTGTAC	384	200	2	0	0	0
PIGN	TGTGGTCATGCAACCAGT	205	198	0	0	0	0
PIGN	GTTGGTCATGCAACCACA	66	44	0	0	0	0
PIGN	ACTGGTCATGCAACCAAC	171	57	0	0	0	0
PIGN	CATGGTCATGCAACCATG	44	36	0	1	0	1
PIGN	GTTGGTCATGCAACACGT	232	161	0	0	0	0
PIGO	CATGCAACTGGTCATGTG	498	1065	5	1	1	40
PIGO	CAACACACGTCACATGAC	170	186	0	148	0	46
PIGO	GTACACACGTCACATGTG	521	578	1	1	0	0
PIGO	ACACACACGTCAACGTGT	274	551	1	0	0	0
PIGO	TGACACACGTCAACGTCA	107	73	1	0	0	0
PIGO	CATGCAGTCACACACAGT	90	124	0	0	0	0
PIGP	ACTGGTACACACCACATG	152	394	0	0	0	0
PIGP	CATGGTACACACCAACGT	960	603	1	0	0	1
PIGP	ACTGGTACACACCAACCA	511	467	1	0	0	0
PIGP	GTTGGTACACACCAACAC	0	72	0	0	0	0
PIGP	TGTGGTACACACCAACTG	1193	799	1	0	0	1
PIGQ	GTTGGTTGTGACCATGTG	338	392	1	0	0	0
PIGQ	ACTGGTTGTGACACGTGT	395	256	0	1	0	0
PIGQ	TGTGGTTGTGACACGTCA	72	97	0	0	0	0
PIGQ	CATGGTTGTGACACGTAC	36	60	0	0	0	0
PIGQ	GTTGGTTGTGACACGTTG	84	149	0	0	0	0
PIGQ	TGTGCAACACTGGTGTAC	57	229	0	0	0	22
PIGS	ACTGGTCAACCATGGTGT	1124	487	4	0	1	0
PIGS	ACTGGTCAACCAACTGGT	555	151	1	0	0	0
PIGS	TGTGGTCAACCAACTGCA	98	98	0	0	0	0
PIGS	CATGGTCAACCAACTGAC	35	22	0	0	0	0
PIGS	GTTGGTCAACCAACTGTG	191	193	3	0	0	0
PIGS	TGTGCAACTGGTACGTGT	468	259	3	0	0	0
PIGT	ACTGGTCAACTGGTTGAC	22	32	1	3	41	0
PIGT	GTTGGTCAACTGGTTGCA	172	298	1	0	0	0
PIGT	CATGGTCAACTGGTTGTG	179	167	1	0	0	0
PIGT	TGTGGTCAACTGCAGTGT	544	666	0	0	0	0
PIGT	ACTGGTTGACGTGTGTTG	1260	1641	4	2	0	6
PIGU	TGTGGTCAACCAACGTGT	850	805	2	1	13	14
PIGU	CATGGTCAACCACATGTG	211	464	0	0	0	0
PIGU	GTTGGTCAACCAACGTCA	272	225	0	0	0	0
PIGU	ACTGGTCAACCAACGTAC	107	106	0	0	0	0
PIGU	CATGACGTACCAACCAGT	174	111	0	0	0	1
PIGV	TGTGGTCACATGACGTAC	86	83	0	0	0	0
PIGV	ACTGGTCACATGACGTTG	531	971	0	1	0	0
PIGV	CATGGTCACATGACCAGT	236	262	0	0	0	1
PIGV	ACTGGTCACATGACCACA	48	137	1	0	0	0
PIGV	GTTGGTCACATGACCAAC	8	12	0	0	0	1
PIGV	CATGCAACACGTACGTCA	195	234	1	1	0	0
PIGW	GTTGGTTGACCATGGTGT	488	376	0	0	0	0
PIGW	GTTGGTTGTGTGCATGCA	185	234	0	0	0	0
PIGW	ACTGGTTGTGTGCATGAC	64	69	2	0	0	0
PIGW	CATGGTTGTGTGCATGTG	445	868	0	0	0	1
PIGW	TGTGGTTGTGTGACGTGT	403	446	1	0	0	0
PIGX	GTTGCAACTGGTTGGTTG	739	630	10	1	2	4
PIGX	GTTGGTCATGCAGTGTGT	1123	1504	1	0	1	1
PIGX	ACTGGTCATGGTTGTGCA	64	41	0	0	0	0
PIGX	GTTGGTCATGGTTGTGAC	37	50	0	0	0	0
PIGX	TGTGGTCATGGTTGTGTG	380	375	0	1	0	1
PIGX	CATGGTCATGCAGTGTCA	280	415	0	0	0	0
PIK3AP1	ACACCACAGTGTCAACGT	205	94	0	0	0	0
PIK3AP1	TGACCACAGTGTCAACCA	85	56	0	0	0	0
PIK3AP1	CAACCACAGTGTCAACAC	31	134	0	0	0	0
PIK3AP1	GTACCACAGTGTCAACTG	380	230	1	0	0	0
PIK3AP1	TGTGGTTGGTACGTTGTG	120	93	2	0	0	0
PIK3C2A	ACCATGACCAGTACACAC	53	70	0	0	0	0
PIK3C2A	CACATGACCAGTACACTG	549	151	1	0	0	8

PIK3C2A	GTCATGACCAGTACTGGT	0	48	1	0	0	0
PIK3C2A	CACATGACCAGTACTGCA	844	703	1	0	0	1
PIK3C2A	TGCATGACCAGTACTGAC	40	25	1	0	0	0
PIK3C2A	GTTGCACACAGTGTTGTG	183	197	0	0	0	0
PIK3C2B	GTCATGACCAGTGTGTCA	243	333	0	0	0	0
PIK3C2B	ACCATGACCAGTGTGTAC	9	10	0	0	0	0
PIK3C2B	CACATGACCAGTGTGTTG	409	388	0	1	0	0
PIK3C2B	GTCATGACCAGTGTCAGT	69	81	0	0	0	0
PIK3C2B	CATGGTTGACACCATGTG	9	23	0	0	0	0
PIK3C2B	CATGCAACGTCATGACGT	136	130	1	0	0	0
PIK3C2G	ACCATGACCACACATGTG	742	760	0	0	2	0
PIK3C2G	GTCATGACCACACATGGT	208	423	0	0	0	0
PIK3C2G	CACATGACCACACATGCA	34	97	0	0	0	0
PIK3C2G	TGCATGACCACACATGAC	43	51	0	0	0	1
PIK3C2G	GTCATGACCACAACGTGT	91	86	0	0	0	0
PIK3C3	ACACCAACTGGTACGTGT	854	1400	2	0	0	6
PIK3C3	TGACCAACTGGTACGTCA	164	224	0	0	0	0
PIK3C3	CAACCAACTGGTACGTAC	251	204	1	0	0	0
PIK3C3	GTACCAACTGGTACGTTG	378	305	0	0	0	0
PIK3C3	TGACCAACTGGTACCAGT	53	60	0	0	0	0
PIK3CA	ACACCATGGTTGGTTGTG	138	231	0	0	0	0
PIK3CA	GTACCATGGTTGCAGTGT	662	303	1	0	0	0
PIK3CA	CAACCATGGTTGCAGTCA	180	352	0	0	0	1
PIK3CA	TGACCATGGTTGCAGTAC	44	46	0	0	0	0
PIK3CA	CAACTGTGACACACTGAC	60	23	0	0	0	0
PIK3CB	TGACCATGCAACACCAAC	87	82	0	0	0	0
PIK3CB	ACACCATGCAACACCATG	613	563	1	0	0	19
PIK3CB	CAACCATGCAACACACGT	43	36	0	0	0	0
PIK3CB	ACACCATGCAACACACCA	158	187	0	0	0	0
PIK3CB	GTTGGTTGGTGTACTGTG	216	181	3	0	0	0
PIK3CB	GTTGCACAACGTTGTGAC	10	20	0	0	0	0
PIK3CD	TGACCACAGTGTGTACGT	259	191	0	0	0	0
PIK3CD	GTACCACAGTGTGTACCA	510	394	0	0	0	4
PIK3CD	ACACCACAGTGTGTACAC	11	17	0	0	0	0
PIK3CD	CAACCACAGTGTGTACTG	315	498	0	0	0	0
PIK3CD	GTACCACAGTGTGTTGGT	190	283	1	0	0	0
PIK3CD	TGTGCAACCACACAACAC	126	118	0	0	0	0
PIK3CG	CAACCACAGTGTGTTGCA	106	309	0	0	0	0
PIK3CG	TGACCACAGTGTGTTGAC	39	37	0	0	0	0
PIK3CG	ACACCACAGTGTGTTGTG	87	68	0	0	0	0
PIK3CG	GTACCACAGTGTCAGTGT	271	171	0	0	0	0
PIK3CG	CAACCACAGTGTCAGTCA	263	458	0	0	0	0
PIK3CG	CATGCAACCATGACCAGT	68	74	0	0	0	0
PIK3R1	TGACCACAGTGTCAGTAC	99	165	0	0	0	0
PIK3R1	ACACCACAGTGTCAGTTG	84	82	0	0	0	0
PIK3R1	CAACCACAGTGTCACAGT	240	211	0	0	0	0
PIK3R1	GTACCATGCAACGTCATG	284	193	0	0	0	0
PIK3R1	TGACCATGCAACGTACGT	320	384	4	0	0	14
PIK3R1	ACTGCATGCACAACTGCA	369	422	2	6	0	5
PIK3R2	GTACCATGGTTGTGTGAC	274	92	0	1	0	0
PIK3R2	TGACCATGGTTGTGTGTG	170	261	0	0	0	3
PIK3R2	GTACCATGCAGTGTGTGT	145	229	0	0	0	1
PIK3R2	CAACCATGCAGTGTGTCA	161	182	0	0	0	0
PIK3R2	TGACCATGCAGTGTGTAC	121	153	0	0	0	0
PIK3R3	ACACGTTGTGACACACAC	17	56	0	0	1	1
PIK3R3	CAACGTTGTGACACACTG	927	759	2	0	0	0
PIK3R3	ACACCACAGTGTCACACA	67	61	0	0	0	0
PIK3R3	GTACCACAGTGTCACAAC	1	3	0	0	0	0
PIK3R3	TGACCACAGTGTCACATG	80	90	1	0	0	0
PIK3R4	ACCATGCATGGTTGGTTG	241	527	0	0	0	7
PIK3R4	CACATGCATGGTTGCAGT	253	336	0	0	0	2
PIK3R4	ACCATGCATGGTTGCACA	72	105	0	0	0	0
PIK3R4	GTCATGCATGGTTGCAAC	10	17	0	0	0	0
PIK3R4	GTTGGTTGGTGTTGCACA	97	182	1	1	0	0
PIK3R4	GTTGCAACTGGTTGCACA	66	108	0	0	0	0
PIK3R5	TGACCACAGTGTGTGTTG	762	777	15	0	0	1
PIK3R5	ACACCACAGTGTGTCAGT	85	92	0	0	0	0
PIK3R5	TGACCACAGTGTGTCACA	96	158	0	0	0	0
PIK3R5	CAACCACAGTGTGTCAAC	134	31	0	0	0	0
PIK3R5	GTACCACAGTGTGTCATG	512	324	0	17	0	7
PIK4CA	CAACGTTGACGTGTTGAC	14	17	0	0	0	0
PIK4CA	GTACGTTGACGTGTTGTG	114	260	0	0	0	0
PIK4CA	ACACGTTGACGTCAGTGT	148	182	0	0	0	0
PIK4CA	TGACGTTGACGTCAGTCA	39	72	0	0	0	0
PIK4CA	CAACGTTGACGTCAGTAC	138	178	0	0	0	0

PIK4CA	ACTGCACACAGTCAGTGT	843	909	6	0	0	0
PIN1	GTCATGCATGACGTTGCA	327	205	1	0	1	0
PIN1	ACCATGCATGACGTTGAC	86	104	0	0	1	0
PIN1	CACATGCATGACGTTGTG	259	215	0	0	0	0
PIN1	CAACACCAACACTGCAAC	18	15	0	0	0	0
PIN1	GTTGCAGTACGTGTCAAC	26	19	0	0	0	0
PINK1	TGACGTGTCAACCACAGT	63	95	0	1	0	0
PINK1	GTACGTGTCAACCACACA	155	52	0	1	0	0
PINK1	ACACGTGTCAACCACAAC	25	28	0	0	0	0
PINK1	CAACGTGTCAACCACATG	144	262	1	0	0	0
PINK1	GTACGTGTCAACCAACGT	251	431	1	0	0	0
PINX1	CAACTGACTGACGTACGT	357	388	0	1	0	0
PINX1	GTTGGTCAACCAGTTGGT	318	559	1	0	0	17
PINX1	CATGGTCAACCAGTTGCA	123	120	1	0	0	0
PINX1	TGTGGTCAACCAGTTGAC	17	34	0	0	0	0
PINX1	ACTGGTCAACCAGTTGTG	274	405	0	1	0	0
PIP3-E	ACTGGTCAACGTGTCAAC	76	29	0	0	2	0
PIP3-E	TGTGGTCAACGTGTCAGT	365	241	0	0	0	0
PIP3-E	GTTGGTCAACGTGTCACA	43	74	0	0	0	0
PIP3-E	CATGGTCAACGTGTCATG	81	102	0	1	0	0
PIP3-E	GTTGGTCAACGTGTACGT	23	43	0	0	0	0
PIP5K2B	TGTGCAACCAACTGCAGT	212	234	0	1	1	1
PIP5K2B	ACCATGTGTGACGTCAAC	16	34	1	0	0	0
PIP5K2B	CACATGTGTGACGTCATG	76	97	0	1	0	0
PIP5K2B	TGCATGTGTGACTGGTGT	420	364	0	0	0	0
PIP5K2B	GTCATGTGTGACTGGTCA	270	249	0	0	0	8
PIP5K2B	ACACGTTGTGACACCAGT	138	149	0	0	0	0
PIR	CAACGTACTGACACGTCA	159	171	0	0	1	2
PIR	TGACGTACTGACCATGAC	52	61	0	0	0	0
PIR	ACACGTACTGACCATGTG	415	724	2	0	0	0
PIR	GTACGTACTGACACGTGT	401	297	1	0	0	3
PIR	TGACGTACTGACACGTAC	209	117	0	1	0	0
PITPNB	TGTGGTACTGGTGTCAGT	111	379	0	0	1	0
PITPNB	TGTGGTACTGGTGTGTCA	481	484	1	1	0	2
PITPNB	CATGGTACTGGTGTGTAC	33	27	0	0	0	0
PITPNB	GTTGGTACTGGTGTGTTG	383	537	0	0	0	0
PITPNB	GTTGGTACTGGTGTCACA	54	78	0	1	0	0
PITX2	GTACGTTGCACACAACAC	36	52	0	0	0	0
PITX2	TGACGTTGCACACAACTG	563	572	1	1	0	0
PITX2	ACACGTTGCACACATGGT	259	261	0	0	0	0
PITX2	TGACGTTGCACACATGCA	324	281	2	0	0	1
PITX2	CAACGTTGCACACATGAC	29	30	0	0	0	0
PITX2	CATGCAACTGCAGTGTCA	411	455	1	0	0	0
PIWIL1	TGACGTGTACACTGTGTG	154	272	0	0	0	0
PIWIL1	ACACGTGTACTGGTGTGT	889	917	1	0	0	0
PIWIL1	TGACGTGTACTGGTGTCA	59	41	0	0	0	2
PIWIL1	CAACGTGTACTGGTGTAC	47	46	0	0	0	0
PIWIL1	GTACGTGTACTGGTGTTG	261	274	0	0	0	0
PIWIL2	TGACGTGTACTGGTCAGT	234	244	0	0	0	0
PIWIL2	GTACGTGTACTGGTCACA	101	100	0	0	0	0
PIWIL2	ACACGTGTACTGGTCAAC	90	32	0	0	0	0
PIWIL2	CAACGTGTACTGGTCATG	133	53	0	0	0	0
PIWIL2	GTACGTGTACTGGTACGT	150	131	0	0	0	5
PIWIL2	CATGCAACTGCAACACCA	673	800	3	1	0	0
PIWIL3	TGTGGTACTGCAGTGTTG	1004	355	1	0	1	0
PIWIL3	ACTGGTACTGCAGTGTCA	63	99	0	0	0	2
PIWIL3	GTTGGTACTGCAGTGTAC	64	59	0	0	0	0
PIWIL3	ACTGGTACTGCAGTCAGT	502	206	1	1	0	2
PIWIL3	TGTGGTACTGCAGTCACA	140	117	0	0	0	0
PKLR	CAACGTGTGTCATGGTGT	805	637	2	0	0	0
PKLR	ACACGTGTGTCATGGTCA	177	264	0	0	0	0
PKLR	GTACGTGTGTCATGGTAC	12	20	0	0	0	0
PKLR	TGACGTGTGTCATGGTTG	620	295	0	0	0	0
PKLR	TGTGGTACTGTGACCAGT	440	575	1	1	0	0
PKM2	CATGGTACTGTGACACCA	197	210	1	6	1	140
PKM2	CAACCAACACTGGTTGAC	20	39	0	0	0	0
PKM2	GTACCAACACTGGTTGTG	276	306	1	0	0	0
PKM2	ACACCAACACTGCAGTGT	207	207	0	0	0	0
PKM2	GTTGGTACTGTGACACGT	78	110	0	0	0	0
PKMYT1	TGACGTGTGTGTACTGCA	254	265	0	0	0	0
PKMYT1	CAACGTGTGTGTACTGAC	157	72	0	1	0	0
PKMYT1	GTACGTGTGTGTACTGTG	325	201	1	1	0	0
PKMYT1	GTTGGTACTGACGTGTCA	473	427	0	0	0	1
PKMYT1	ACTGGTACTGACGTGTAC	47	50	0	0	0	0
PKN1	ACCATGACGTGTGTTGCA	73	87	0	0	0	0

PKN1	GTCATGACGTGTGTTGAC	27	21	0	0	0	0
PKN1	TGCATGACGTGTGTTGTG	314	1122	0	0	0	0
PKN1	CACATGACGTGTCAGTGT	536	391	1	0	0	38
PKN1	ACCATGACGTGTCAGTCA	118	126	0	0	0	0
PKN2	TGACGTGTGTGTTGCAGT	70	144	2	0	0	0
PKN2	GTACGTGTGTGTTGCACA	128	81	0	0	0	0
PKN2	ACACGTGTGTGTTGCAAC	23	24	0	0	0	0
PKN2	CAACGTGTGTGTTGCATG	267	284	0	0	0	0
PKN2	GTACGTGTGTGTTGACGT	200	124	1	0	0	0
PKP4	TGTGGTCACACAACACGT	12	32	0	0	0	2
PKP4	GTTGGTCACACAACACCA	364	546	0	0	0	0
PKP4	ACTGGTCACACAACACAC	159	154	0	0	0	0
PKP4	CATGGTCACACAACACTG	256	464	1	1	0	0
PKP4	CATGGTCACAACGTACTG	374	540	0	0	0	0
PLA1A	GTACTGTGCATGGTGTGT	152	282	0	0	0	0
PLA1A	CAACTGTGCATGGTGTCA	312	238	0	0	0	6
PLA1A	TGACTGTGCATGGTGTAC	182	81	0	0	0	0
PLA1A	ACACTGTGCATGGTGTTG	473	1126	0	0	0	0
PLA1A	CAACTGTGCATGGTCAGT	331	373	0	0	0	0
PLA1A	GTTGCACAACTGTGCAAC	0	2	0	0	0	0
PLA2G10	CAACACACGTTGACGTGT	1043	371	1	2	0	0
PLA2G10	ACACACACGTTGACGTCA	244	342	0	0	0	0
PLA2G10	GTACACACGTTGACGTAC	21	15	0	0	0	0
PLA2G10	TGACACACGTTGACGTTG	360	664	1	1	0	1
PLA2G10	CATGCACACAGTCAGTAC	221	125	1	0	0	0
PLA2G12A	CAACACACCAACGTACGT	893	717	1	1	1	0
PLA2G12A	ACACACACCAACGTACCA	166	214	0	0	1	0
PLA2G12A	CATGCAGTCAACGTCACA	88	97	0	0	1	0
PLA2G12A	GTACACACCAACGTACAC	30	26	0	0	0	0
PLA2G12A	TGACACACCAACGTACTG	537	381	1	1	0	0
PLA2G12B	GTACTGTGCATGGTCAAC	66	59	0	0	1	0
PLA2G12B	ACACTGTGCATGGTCACA	149	319	1	0	0	0
PLA2G12B	TGACTGTGCATGGTCATG	320	145	0	0	0	0
PLA2G12B	ACACTGTGCATGGTACGT	290	263	0	0	0	0
PLA2G12B	CATGCAGTTGTGTGACCA	127	111	0	0	0	0
PLA2G1B	TGACACACCATGACACAC	165	146	0	0	2	0
PLA2G1B	CAACACACCATGACTGGT	198	138	0	0	1	0
PLA2G1B	CAACACACCATGACACCA	309	369	1	0	0	0
PLA2G1B	ACACACACCATGACACTG	267	291	0	0	0	0
PLA2G1B	CATGCAGTTGCAGTGTAC	221	258	0	1	0	0
PLA2G2A	TGACACACACACCACATG	181	366	0	0	0	0
PLA2G2A	ACACACACACACCAACGT	756	396	0	0	0	0
PLA2G2A	TGACACACACACCAACCA	651	914	2	1	0	0
PLA2G2A	CAACACACACACCAACAC	196	57	0	0	0	0
PLA2G2A	CATGCAGTTGGTTGTGTG	365	505	1	0	0	0
PLA2G2A	GTTGCAACCAACCAACTG	1627	873	0	0	0	0
PLA2G2D	CAACACACGTCAACGTAC	72	85	0	0	0	0
PLA2G2D	GTACACACGTCAACGTTG	716	707	1	0	0	0
PLA2G2D	TGACACACGTCAACCAGT	850	307	2	0	0	0
PLA2G2D	GTACACACGTCAACCACA	3	14	0	0	0	0
PLA2G2D	CATGCAACCATGTGGTTG	148	222	0	0	0	0
PLA2G2D	GTTGCAACCATGTGCAGT	377	188	0	0	0	0
PLA2G2E	GTACACACCATGTGACCA	570	321	1968	4	2	0
PLA2G2E	TGACACACCATGTGCACA	66	104	0	0	0	0
PLA2G2E	CAACACACCATGTGCAAC	76	54	0	1	0	0
PLA2G2E	GTACACACCATGTGCATG	413	269	0	0	0	0
PLA2G2E	TGACACACCATGTGACGT	291	405	1	0	0	1
PLA2G2E	ACTGCACAACTGGTACCA	219	204	0	2	0	0
PLA2G2F	GTACACACCATGGTCAAC	63	61	0	1	1	1
PLA2G2F	TGACACACCATGGTGTAC	39	38	0	0	0	0
PLA2G2F	ACACACACCATGGTGTTG	440	550	1	0	0	0
PLA2G2F	CAACACACCATGGTCAGT	167	287	0	0	0	0
PLA2G2F	ACACACACCATGGTCACA	153	150	0	0	0	0
PLA2G3	TGACACACACCAGTCAAC	60	127	0	1	0	0
PLA2G3	ACACACACACCAGTCATG	435	131	0	1	0	0
PLA2G3	CAACACACACCAGTACGT	381	490	0	1	0	1
PLA2G3	ACACACACACCAGTACCA	578	535	2	0	0	20
PLA2G3	GTACACACACCAGTACAC	22	108	0	0	0	0
PLA2G4A	CAACACCATGTGCATGAC	44	48	0	0	0	0
PLA2G4A	GTACACCATGTGCATGTG	200	289	0	0	0	1
PLA2G4A	ACACACCATGTGACGTGT	303	450	1	0	0	0
PLA2G4A	TGACACCATGTGACGTCA	173	170	0	0	0	0
PLA2G4A	CAACACCATGTGACGTAC	33	17	0	0	0	0
PLA2G4A	ACTGCAACTGGTTGCAAC	15	5	0	0	0	0
PLA2G4B	ACACGTGTCAACACGTCA	312	310	216	0	1	2

PLA2G4B	GTACGTGTCAACCATGAC	67	125	0	0	0	0
PLA2G4B	TGACGTGTCAACCATGTG	726	536	0	0	0	2
PLA2G4B	CAACGTGTCAACACGTGT	1325	551	1	1	0	1
PLA2G4B	GTACGTGTCAACACGTAC	16	98	0	0	0	0
PLA2G4C	CAACACTGGTTGTGACTG	727	769	0	0	2	0
PLA2G4C	GTACACTGGTTGTGTGGT	923	153	2	0	0	0
PLA2G4C	CAACACTGGTTGTGTGCA	186	167	1	0	0	0
PLA2G4C	TGACACTGGTTGTGTGAC	8	24	0	0	0	0
PLA2G4C	TGTGCACACAGTCACAGT	72	226	0	0	0	0
PLA2G4C	GTTGCACACAGTCACACA	177	322	0	0	0	0
PLA2G4D	TGACTGTGCATGGTACCA	204	170	1	0	0	0
PLA2G4D	CAACTGTGCATGGTACAC	38	38	0	0	0	0
PLA2G4D	GTACTGTGCATGGTACTG	136	138	0	0	0	0
PLA2G4D	TGACTGTGCATGGTTGGT	153	264	0	0	0	0
PLA2G4D	GTACTGTGCATGGTTGCA	100	141	1	0	0	0
PLA2G4D	TGTGCATGGTACACCAAC	27	110	0	0	0	0
PLA2G5	ACACACACCAACACCAGT	615	305	0	0	0	5
PLA2G5	TGACACACCAACACCACA	143	178	0	0	0	0
PLA2G5	CAACACACCAACACCAAC	49	80	0	0	0	3
PLA2G5	GTACACACCAACACCATG	505	119	0	2	0	0
PLA2G5	TGACACACCAACACACGT	177	212	0	0	0	0
PLA2G6	CAACACACCAGTACCAGT	483	366	0	0	0	0
PLA2G6	ACACACACCAGTACCACA	114	86	0	0	0	0
PLA2G6	GTACACACCAGTACCAAC	15	31	0	0	0	0
PLA2G6	TGACACACCAGTACCATG	464	518	0	1	0	1
PLA2G6	CATGGTACTGCAACACGT	487	230	0	0	0	0
PLA2G7	GTACACCAACGTCACACA	65	51	0	0	0	0
PLA2G7	ACACACCAACGTCACAAC	21	19	0	0	0	0
PLA2G7	CAACACCAACGTCACATG	167	184	0	0	0	0
PLA2G7	GTACACCAACGTCAACGT	280	437	1	1	0	0
PLA2G7	ACTGCAGTTGGTACTGTG	1022	625	1	0	0	0
PLA2G7	ACTGCATGCAGTCAACGT	105	144	0	1	0	0
PLAGL1	ACACGTTGACGTCATGCA	434	802	33	44	21	43
PLAGL1	CAACGTTGACGTCATGGT	495	551	2	0	0	1
PLAGL1	GTACGTTGACGTCATGAC	65	72	0	0	0	0
PLAGL1	TGACGTTGACGTCATGTG	298	200	0	1	0	0
PLAGL1	GTTGGTTGTGGTGTTGTG	28	34	0	0	0	0
PLAT	ACACACACGTTGACCAGT	80	103	0	0	0	0
PLAT	TGACACACGTTGACCACA	183	65	0	0	0	0
PLAT	CAACACACGTTGACCAAC	21	33	0	0	0	0
PLAT	GTACACACGTTGACCATG	171	98	0	0	0	0
PLAT	TGACACACGTTGACACGT	32	36	0	0	0	0
PLAT	ACTGCAACGTCAGTTGCA	110	28	0	0	0	0
PLAU	TGACACACCAGTTGCAAC	14	38	0	0	0	0
PLAU	ACACACACCAGTTGCATG	176	172	0	1	0	1
PLAU	CAACACACCAGTTGACGT	805	714	0	0	0	0
PLAU	ACACACACCAGTTGACCA	475	482	0	0	0	10
PLAU	GTACACACCAGTTGACAC	287	151	1	0	0	0
PLAUR	TGACACACTGCAACGTAC	73	117	0	0	0	0
PLAUR	ACACACACTGCAACGTTG	201	254	0	0	0	0
PLAUR	CAACACACTGCAACCAGT	87	82	0	0	0	0
PLAUR	ACACACACTGCAACCACA	113	213	0	0	0	0
PLAUR	GTACACACTGCAACCAAC	55	68	0	1	0	0
PLCB1	ACACGTGTCACATGTGCA	615	344	1	0	0	0
PLCB1	GTACGTGTCACATGTGAC	42	56	0	0	0	0
PLCB1	TGACGTGTCACATGTGTG	276	161	1	0	0	0
PLCB1	TGACGTGTCAACGTGTGT	96	265	1	0	0	0
PLCB1	ACACTGTGCACAACCATG	158	112	0	0	0	0
PLCB2	CACATGACCAGTGTTGAC	28	70	0	1	1	0
PLCB2	GTCATGACCAGTGTACAC	26	51	0	0	0	0
PLCB2	TGCATGACCAGTGTACTG	197	269	0	0	0	1
PLCB2	ACCATGACCAGTGTTGGT	401	275	0	0	0	0
PLCB2	TGCATGACCAGTGTTGCA	75	97	0	0	0	0
PLCB3	CACATGCACATGCACACA	185	65	0	0	0	0
PLCB3	TGCATGCACATGCACAAC	4	14	0	0	0	0
PLCB3	ACCATGCACATGCACATG	17	21	0	0	0	0
PLCB3	CACATGCACATGCAACGT	315	393	0	0	0	0
PLCB3	ACCATGCACATGCAACCA	98	192	1	0	0	0
PLCB3	TGTGCACATGACGTTGAC	213	47	0	0	0	0
PLCD1	CACATGACCACAACGTCA	383	167	0	0	0	0
PLCD1	TGCATGACCACAACGTAC	78	79	0	0	0	0
PLCD1	ACCATGACCACAACGTTG	322	276	0	0	0	0
PLCD1	CACATGACCACAACCAGT	103	415	0	0	0	0
PLCD1	CATGCAGTACGTTGGTGT	566	398	0	1	0	0
PLCE1	GTACACACCAGTACTGCA	83	110	1	0	1	0

PLCE1	ACACACACCAGTACTGAC	57	44	0	0	0	0
PLCE1	CAACACACCAGTACTGTG	221	180	0	0	0	0
PLCE1	TGACACACCAGTTGGTGT	921	1000	1	1	0	1
PLCE1	GTACACACCAGTTGGTCA	170	94	0	2	0	1
PLCG1	TGACGTGTCACATGGTCA	448	555	0	0	0	1
PLCG1	CAACGTGTCACATGGTAC	124	46	0	0	0	1
PLCG1	GTACGTGTCACATGGTTG	224	502	0	1	0	0
PLCG1	TGACGTGTCACATGCAGT	208	167	0	0	0	0
PLCG1	GTTGGTTGTGGTCAACGT	296	250	1	0	0	0
PLCG2	TGCATGACCACAGTTGTG	220	246	0	1	0	0
PLCG2	CACATGACCACACAGTGT	588	611	1	0	0	0
PLCG2	ACCATGACCACACAGTCA	370	337	2	0	0	0
PLCG2	GTCATGACCACACAGTAC	85	352	0	0	0	0
PLCG2	TGTGCAGTACGTGTCATG	224	188	0	0	0	0
PLD1	GTCATGCATGCATGACAC	0	0	0	0	0	0
PLD1	TGCATGCATGCATGACTG	147	179	0	0	0	2
PLD1	ACCATGCATGCATGTGGT	39	17	0	0	0	0
PLD1	TGACTGTGCACAACCAAC	10	7	0	0	0	0
PLD1	TGTGCAGTACGTCAACTG	452	319	0	0	0	1
PLD2	CAACACACCACAGTTGCA	630	433	17	22	2	23
PLD2	ACACACACCACAGTTGTG	663	711	2	1	1	0
PLD2	TGACACACCACAGTTGAC	18	18	0	0	0	0
PLD2	GTACACACCACACAGTGT	948	493	26	1	0	1
PLD2	CAACACACCACACAGTCA	845	292	2	2	0	83
PLD2	TGTGCAACCAACGTGTTG	445	399	1	0	0	0
PLD3	CAACACACACACTGTGGT	344	253	0	0	0	0
PLD3	ACACACACACACTGTGCA	143	145	0	0	0	0
PLD3	GTACACACACACTGTGAC	98	146	1	0	0	0
PLD3	TGACACACACACTGTGTG	349	577	0	0	0	0
PLD3	ACACACACACTGGTGTGT	57	63	0	0	0	3
PLEC1	ACACTGTGACTGGTACTG	423	262	0	2	0	0
PLEC1	CAACTGTGACTGGTTGGT	562	364	0	0	0	0
PLEC1	ACACTGTGACTGGTTGCA	115	159	0	0	0	0
PLEC1	GTACTGTGACTGGTTGAC	315	202	0	0	0	0
PLEC1	TGACTGTGACTGGTTGTG	747	469	1	0	0	0
PLEKHA8	ACTGGTCATGACCATGTG	426	519	0	0	1	11
PLEKHA8	GTTGGTACGTCAGTTGTG	734	332	1	0	1	0
PLEKHA8	GTTGGTGTCACAACCAAC	6	8	0	0	0	0
PLEKHA8	GTTGGTCATGACACGTGT	2047	2074	1	1	0	24
PLEKHA8	CATGGTCATGACACGTCA	22	38	0	0	0	0
PLG	GTACTGACTGACCACACA	61	113	0	0	0	0
PLG	ACACTGACTGACCACAAC	2	9	0	0	0	0
PLG	CAACTGACTGACCACATG	190	219	0	0	0	0
PLG	GTACTGACTGACCAACGT	565	469	1	1	0	1
PLG	CAACTGACTGACCAACCA	308	277	0	0	0	0
PLG	ACTGCACACAGTCACAAC	12	26	0	0	0	0
PLIN	CATGGTTGACGTGTCAGT	241	475	1	0	0	3
PLIN	ACTGGTTGACGTGTCACA	72	61	0	0	0	0
PLIN	GTTGGTTGACGTGTCAAC	1	14	0	0	0	2
PLIN	TGTGGTTGACGTGTCATG	397	371	2	0	0	1
PLIN	ACTGACGTACACTGGTCA	108	160	0	0	0	0
PLK1	ACTGTGACACGTGTGTTG	929	841	9	5	8	32
PLK1	TGTGTGACACGTCAGTGT	582	1111	0	1	1	2
PLK1	GTTGTGACACGTCAGTCA	391	276	0	0	1	1
PLK1	GTTGTGACACTGCAACCA	144	100	0	0	1	0
PLK1	ACACGTGTGTGTTGGTGT	561	357	0	0	0	0
PLK1	TGACGTGTGTGTTGGTCA	82	75	0	0	0	0
PLK1	CAACGTGTGTGTTGGTAC	14	8	0	0	0	0
PLK1	GTACGTGTGTGTTGGTTG	515	769	1	1	0	0
PLK1	CATGGTCACAGTGTACAC	27	42	0	0	0	0
PLK1	CATGTGACACGTGTGTCA	395	430	0	14	0	0
PLK1	TGTGTGACACGTGTGTAC	90	165	0	0	0	0
PLK1	GTTGTGACACGTGTCAAC	8	6	0	0	0	0
PLK1	TGTGTGACACGTGTCATG	112	153	0	0	0	0
PLK1	ACTGTGACACGTGTACGT	100	144	0	0	0	0
PLK1	GTTGTGACACGTGTACTG	103	159	0	0	0	0
PLK1	TGTGTGACACGTGTTGGT	75	120	0	0	0	0
PLK1	GTTGTGACACGTGTTGCA	53	112	0	0	0	0
PLK1	ACTGTGACACGTCAGTAC	93	80	0	0	0	0
PLK1	TGTGTGACACTGCAACGT	98	119	0	0	0	0
PLK1	ACTGTGACACTGCAACAC	39	44	0	0	0	0
PLK1	CATGTGACACTGCATGCA	218	249	0	0	0	0
PLK1	TGTGTGACACTGCATGAC	38	100	0	1	0	0
PLK1	ACTGTGACACTGCATGTG	696	938	1	0	0	8
PLK1	TGTGTGACACTGACGTAC	42	52	0	0	0	0

PLK1	ACTGTGACACTGACGTTG	209	269	1	0	0	0
PLK1	CATGTGACACTGACCAGT	222	143	2	13	0	0
PLLP	ACTGGTTGACGTGTACGT	113	147	1	1	0	0
PLLP	TGTGGTTGACGTGTACCA	500	453	0	0	0	1
PLLP	CATGGTTGACGTGTACAC	51	76	0	0	0	0
PLLP	GTTGGTTGACGTGTACTG	398	139	0	0	0	0
PLLP	TGTGCACAACTGACACGT	170	97	0	0	0	0
PLLP	GTTGCACAACTGACACCA	101	77	0	0	0	0
PLOD1	ACACTGCATGTGCATGCA	89	105	0	0	0	1
PLOD1	GTACTGCATGTGCATGAC	42	54	0	0	0	0
PLOD1	TGACTGCATGTGCATGTG	399	410	0	0	0	0
PLOD1	CAACTGCATGTGACGTGT	402	383	0	0	0	0
PLOD1	ACACTGCATGTGACGTCA	261	309	0	0	0	0
PLOD1	ACTGCATGCACACAACCA	141	128	0	0	0	0
PLP1	GTTGGTGTGTTGGTGTGT	132	129	0	0	0	1
PLP1	CATGGTGTGTTGGTGTCA	488	959	8	1	0	8
PLP1	TGTGGTGTGTTGGTGTAC	78	77	1	0	0	0
PLP1	ACTGGTGTGTTGGTGTTG	7	0	0	0	0	0
PLP1	ACTGGTGTGTTGTGTGTG	82	100	0	0	0	0
PLSCR1	TGACACTGCAACGTCATG	204	251	3	1	1	0
PLSCR1	GTACACTGCAACGTCAAC	19	15	0	0	0	0
PLSCR1	ACACACTGCAACGTACGT	815	308	0	0	0	1
PLSCR1	TGACACTGCAACGTACCA	258	230	0	0	0	0
PLSCR1	CAACACTGCAACGTACAC	81	193	0	0	0	0
PLSCR1	TGTGCACATGCAACACGT	97	115	0	0	0	9
PLSCR4	TGACACTGGTCAGTGTTG	700	1135	1	3	1	0
PLSCR4	ACACACTGGTCAGTGTCA	552	701	1	0	0	2
PLSCR4	GTACACTGGTCAGTGTAC	23	12	1	0	0	0
PLSCR4	ACACACTGGTCAGTCAGT	304	381	0	0	0	1
PLSCR4	TGACACTGGTCAGTCACA	114	152	0	0	0	0
PLSCR4	TGTGCACATGCAGTACTG	52	69	0	0	0	0
PLTP	CATGGTGTCACAGTCATG	93	106	0	0	0	0
PLTP	CATGGTCATGTGCATGGT	413	345	0	2	0	0
PLTP	ACTGGTCATGTGCATGCA	485	415	1	2	0	0
PLTP	GTTGGTCATGTGCATGAC	72	47	0	1	0	0
PLTP	TGTGGTCATGTGCATGTG	491	464	0	0	0	0
PMAIP1	ACTGGTACACGTACACAC	72	43	0	0	0	0
PMAIP1	CATGGTACACGTACACTG	396	472	1	1	0	1
PMAIP1	GTTGGTACACGTACTGGT	99	63	0	0	0	0
PMAIP1	CATGGTACACGTACTGCA	289	179	0	0	0	0
PMAIP1	TGTGGTACACGTACTGAC	16	115	1	0	0	0
PMAIP1	ACTGCACATGCAACACAC	51	57	0	0	0	0
PMCH	GTTGGTACCAACTGGTCA	405	347	0	114	2	0
PMCH	CATGGTACCAACACTGTG	199	231	1	0	1	0
PMCH	GTTGGTACCAACACTGCA	328	584	0	1	0	2
PMCH	ACTGGTACCAACACTGAC	218	49	0	1	0	0
PMCH	TGTGGTACCAACTGGTGT	513	590	0	1	0	0
PML	CACATGTGGTCAGTTGGT	556	765	0	1	1	0
PML	CACATGTGGTCAGTACCA	383	440	1	0	0	0
PML	TGCATGTGGTCAGTACAC	58	50	0	0	0	0
PML	ACCATGTGGTCAGTACTG	148	185	0	0	0	0
PML	ACCATGTGGTCAGTTGCA	175	154	0	0	0	0
PMM1	CAACACCAACACGTCAGT	416	247	1	0	0	0
PMM1	ACACACCAACACGTCACA	34	9	0	0	0	0
PMM1	GTACACCAACACGTCAAC	17	45	0	0	0	0
PMM1	TGACACCAACACGTCATG	311	152	0	1	0	0
PMM1	ACACACCAACACGTACGT	124	123	0	0	0	0
PMM1	CATGCACACAGTCACATG	415	244	1	0	0	0
PMP22	TGACTGTGACTGGTGTCA	341	261	1	0	1	0
PMP22	CAACTGTGACTGGTGTAC	245	220	0	9	0	3
PMP22	GTACTGTGACTGGTGTTG	373	582	0	0	0	0
PMP22	TGACTGTGACTGGTCAGT	161	181	0	0	0	0
PMP22	GTACTGTGACTGGTCACA	65	143	0	0	0	0
PMP22	GTTGCAACTGCACACAGT	553	231	1	0	0	0
PMS1	GTTGGTTGTGCAACACTG	184	546	0	0	1	0
PMS1	ACTGGTTGTGCAACACGT	80	84	0	0	0	0
PMS1	TGTGGTTGTGCAACACCA	95	106	0	1	0	0
PMS1	CATGGTTGTGCAACACAC	82	85	0	0	0	1
PMS1	TGTGGTTGTGCAACTGGT	292	577	623	1	0	0
PMS2	GTACTGTGACCATGTGCA	50	68	0	0	0	5
PMS2	ACACTGTGACCATGTGAC	23	21	0	1	0	0
PMS2	CAACTGTGACCATGTGTG	370	441	1	1	0	0
PMS2	CAACTGTGACACGTGTGT	680	867	0	0	0	0
PMS2	ACACTGTGACACGTGTCA	421	398	0	0	0	0
PMS2L3	TGTGGTGTCATGCAACCA	813	424	1	1	1	1

PMS2L3	TGTGGTGTCATGCACATG	622	254	0	0	0	0
PMS2L3	ACTGGTGTCATGCAACGT	965	895	120	2	0	2
PMS2L3	CATGGTGTCATGCAACAC	76	55	0	0	0	0
PMS2L3	GTTGGTGTCATGCAACTG	710	662	2	1	0	3
PMVK	TGACGTGTGTGTGTGTTG	527	712	0	0	1	0
PMVK	ACACGTGTGTGTGTGTCA	73	62	0	0	0	0
PMVK	GTACGTGTGTGTGTGTAC	196	52	0	0	0	0
PMVK	ACACGTGTGTGTGTCAGT	345	71	0	0	0	0
PMVK	CATGGTTGGTGTTGACCA	160	164	1	0	0	0
PNKP	CAACACCATGTGTGCAAC	157	68	0	0	0	0
PNKP	GTACACCATGTGTGCATG	87	28	0	0	0	0
PNKP	TGACACCATGTGTGACGT	198	237	0	0	0	0
PNKP	GTACACCATGTGTGACCA	348	472	1	0	0	1
PNKP	CATGGTTGTGGTACACTG	365	296	0	1	0	0
PNKP	TGTGCATGCAGTCAACCA	351	332	1	0	0	0
PNLIP	TGACACCATGACACCACA	146	90	8	0	0	0
PNLIP	CAACACCATGACACCAAC	51	49	0	1	0	0
PNLIP	GTACACCATGACACCATG	99	107	0	0	0	0
PNLIP	TGACACCATGACACACGT	71	145	0	1	0	1
PNLIP	GTACACCATGACACACCA	253	345	0	0	0	0
PNMT	GTACCAACGTGTGTGTTG	2211	1018	4	0	1	0
PNMT	TGACCAACGTGTGTCAGT	487	729	2199	4	1	2
PNMT	TGACCAACGTGTGTGTCA	288	153	1	0	0	3
PNMT	CAACCAACGTGTGTGTAC	194	74	0	0	0	0
PNMT	GTACCAACGTGTGTCACA	86	86	0	0	0	0
PNMT	CATGCATGCAGTCAACAC	209	56	0	0	0	0
PNPLA3	TGACACCATGACACGTTG	701	659	0	5	2	0
PNPLA3	CAACACCATGACACGTGT	1368	1162	3	3	0	1
PNPLA3	ACACACCATGACACGTCA	243	347	0	1	0	0
PNPLA3	GTACACCATGACACGTAC	169	53	0	0	0	0
PNPLA3	ACACACCATGACACCAGT	159	183	0	1	0	2
PNPLA3	GTTGCAACGTTGGTACCA	100	142	0	0	0	0
PNPO	TGACACGTACTGTGGTTG	82	171	1	0	0	0
PNPO	ACACACGTACTGTGCAGT	136	310	0	1	0	0
PNPO	TGACACGTACTGTGCACA	70	174	0	0	0	0
PNPO	CAACACGTACTGTGCAAC	43	36	0	0	0	0
PNPO	GTACACGTACTGTGCATG	72	117	0	1	0	0
PODXL	TGTGGTGTTGCACACAGT	191	206	1	0	5	2
PODXL	TGTGGTGTTGCACAGTCA	336	266	1	0	0	0
PODXL	CATGGTGTTGCACAGTAC	30	46	0	0	0	0
PODXL	GTTGGTGTTGCACAGTTG	421	745	1	0	0	0
PODXL	GTTGGTGTTGCACACACA	175	228	1	0	0	0
POFUT1	TGACCACAACCAGTACTG	151	216	0	1	0	0
POFUT1	ACACCACAACCAGTTGGT	284	377	0	0	0	0
POFUT1	TGACCACAACCAGTTGCA	309	224	0	0	0	30
POFUT1	CAACCACAACCAGTTGAC	53	45	0	0	0	0
POFUT1	GTACCACAACCAGTTGTG	234	284	0	0	0	0
POFUT1	TGTGCAACTGTGACCACA	48	45	0	0	0	0
POLA	GTACACACTGACACGTTG	1572	610	0	2	2	0
POLA	TGACACACTGACACGTCA	238	285	0	0	0	5
POLA	CAACACACTGACACGTAC	16	27	1	0	0	0
POLA	CATGCAGTCATGTGACTG	344	428	0	2	0	0
POLA	GTTGCAGTCATGTGTGGT	200	513	0	1	0	0
POLA	GTTGCACATGGTGTTGCA	73	108	0	0	0	0
POLA2	ACACACACTGTGACGTAC	70	81	0	0	0	0
POLA2	CAACACACTGTGACGTTG	649	1276	2	0	0	1
POLA2	GTACACACTGTGACCAGT	600	362	0	0	0	0
POLA2	CAACACACTGTGACCACA	95	110	0	0	0	0
POLA2	TGACACACTGTGACCAAC	54	89	0	0	0	1
POLA2	TGTGCAACCAACGTTGAC	212	74	0	0	0	0
POLB	TGACGTGTACTGACGTAC	28	23	0	0	0	0
POLB	ACACGTGTACTGACGTTG	273	243	1	0	0	0
POLB	CAACGTGTACTGACCAGT	75	95	0	0	0	0
POLB	ACACGTGTACTGACCACA	148	226	0	0	0	0
POLB	GTTGCAGTCAGTACGTGT	945	489	2	0	0	0
POLB	GTTGCACACAGTCAACGT	712	274	1	0	0	0
POLD1	GTACGTGTACTGACCAAC	15	11	0	0	0	0
POLD1	TGACGTGTACTGACCATG	257	221	0	0	0	0
POLD1	ACACGTGTACTGACACGT	39	38	0	0	0	0
POLD1	TGACGTGTACTGACACCA	126	107	0	0	0	0
POLD1	CAACGTGTACTGACACAC	29	28	0	0	0	0
POLD1	CATGCACACAGTCAACCA	326	315	0	0	0	2
POLD3	ACACACACTGACACTGCA	555	255	1	0	0	0
POLD3	GTACACACTGACACTGAC	102	60	0	0	0	0
POLD3	TGACACACTGACACTGTG	332	406	0	1	0	1

POLD3	CAACACACTGACTGGTGT	310	268	0	0	0	1
POLD3	GTTGGTTGTGGTACGTAC	25	33	0	0	0	0
POLD4	TGACACTGGTGTCACACA	108	72	0	0	0	0
POLD4	CAACACTGGTGTCACAAC	25	22	0	0	0	0
POLD4	GTACACTGGTGTCACATG	150	189	0	0	0	0
POLD4	TGACACTGGTGTCAACGT	702	183	2	0	0	1
POLD4	CATGACGTCACAGTCAGT	884	243	0	0	0	1
POLE	CAACACACTGACACCATG	166	120	0	1	1	0
POLE	TGACACACTGACACCAGT	443	403	0	0	0	0
POLE	GTACACACTGACACCACA	79	195	0	1	0	0
POLE	ACACACACTGACACCAAC	4	10	0	0	0	0
POLE	GTACACACTGACACACGT	128	150	0	0	0	0
POLE2	TGACACACTGACACACAC	46	44	0	0	1	0
POLE2	CAACACACTGACACTGGT	72	233	0	0	1	0
POLE2	CAACACACTGACACACCA	262	374	0	1	0	1
POLE2	ACACACACTGACACACTG	162	150	0	0	0	0
POLE2	ACTGGTTGTGGTACACAC	28	53	0	0	0	0
POLE2	CATGCATGCACAGTTGTG	754	777	2	0	0	1
POLE3	TGTGGTGTCATGCATGGT	164	200	0	0	0	0
POLE3	GTTGGTGTCATGCATGCA	106	124	0	0	0	0
POLE3	ACTGGTGTCATGCATGAC	10	19	0	0	0	2
POLE3	CATGGTGTCATGCATGTG	442	306	57	2	0	0
POLE3	TGTGGTGTCATGACGTGT	275	320	0	1	0	0
POLE3	GTTGCATGCAGTCAACTG	666	547	0	1	0	0
POLE4	CAACACTGGTGTCAGTGT	279	180	0	0	0	0
POLE4	ACACACTGGTGTCAGTCA	986	735	1	0	0	0
POLE4	GTACACTGGTGTCAGTAC	118	197	0	0	0	0
POLE4	TGACACTGGTGTCAGTTG	117	196	1	0	0	1
POLE4	ACACACTGGTGTCACAGT	217	58	0	1	0	0
POLG	ACACACACTGACTGCAGT	185	180	1	0	1	0
POLG	ACACACACTGACTGGTCA	172	137	0	0	0	0
POLG	GTACACACTGACTGGTAC	77	62	0	1	0	0
POLG	TGACACACTGACTGGTTG	410	447	0	0	0	1
POLG	TGACACACTGACTGCACA	283	183	0	1	0	1
POLG	TGTGCACACAGTCAACAC	144	64	0	0	0	0
POLG2	TGACACACTGTGCAACCA	296	404	1085	3	1	0
POLG2	ACACACACTGTGCAACGT	93	145	0	0	0	0
POLG2	CAACACACTGTGCAACAC	100	80	0	0	0	0
POLG2	GTACACACTGTGCAACTG	279	339	1	0	0	0
POLG2	TGACACACTGTGCATGGT	668	282	1	0	0	0
POLH	CAACACACTGACTGACTG	245	180	1	0	1	0
POLH	GTACACACTGACTGTGGT	433	569	1	0	1	1
POLH	CAACACACTGACTGTGCA	302	234	1	1	0	0
POLH	TGACACACTGACTGTGAC	20	33	0	0	0	0
POLH	ACACACACTGACTGTGTG	137	129	0	0	0	0
POLH	GTTGCACAACCACACAGT	111	124	2	1	0	1
POLI	ACACACACTGTGCAGTTG	647	719	0	1	1	0
POLI	CAACACACTGTGCACAGT	209	158	0	0	0	0
POLI	ACACACACTGTGCACACA	258	324	0	0	0	0
POLI	GTACACACTGTGCACAAC	25	25	0	0	0	1
POLI	TGACACACTGTGCACATG	112	108	0	0	0	8
POLK	GTTGGTGTACACCATGGT	315	312	0	0	1	0
POLK	CATGGTGTACACCAACTG	337	182	1	0	0	1
POLK	CATGGTGTACACCATGCA	72	103	0	0	0	1
POLK	TGTGGTGTACACCATGAC	36	48	1	0	0	0
POLK	ACTGGTGTACACCATGTG	1083	613	2	0	0	2
POLK	TGTGCACAACTGACGTTG	354	975	1	0	0	0
POLL	GTACACACTGTGTGCACA	238	24	0	2	1	2
POLL	ACACACACTGTGTGCAAC	30	13	0	0	0	0
POLL	CAACACACTGTGTGCATG	226	379	0	0	0	7
POLL	GTACACACTGTGTGACGT	706	407	1	0	0	96
POLL	CAACACACTGTGTGACCA	201	208	0	0	0	0
POLM	ACACACACTGTGTGTGCA	262	117	92	127	761	0
POLM	TGACACACTGTGTGACAC	30	37	0	0	0	0
POLM	ACACACACTGTGTGACTG	57	57	0	0	0	1
POLM	CAACACACTGTGTGTGGT	224	210	0	0	0	0
POLM	TGTGGTTGTGCACATGAC	35	49	0	0	0	0
POLM	CATGCACAACACACACAC	19	29	0	0	0	0
POLQ	CAACACACTGACTGCAAC	25	20	0	0	0	0
POLQ	GTACACACTGACTGCATG	116	202	0	1	0	0
POLQ	TGACACACTGACTGACGT	280	222	0	0	0	0
POLQ	GTACACACTGACTGACCA	89	105	0	0	0	0
POLQ	ACACACACTGACTGACAC	50	138	0	1	0	0
POLR1A	ACACACACTGTGACCATG	331	257	0	0	0	0
POLR1A	CAACACACTGTGACACGT	24	36	0	0	0	0

POLR1A	ACACACACTGTGACACCA	289	355	3	0	0	0
POLR1A	GTACACACTGTGACACAC	25	12	0	0	0	0
POLR1A	TGACACACTGTGACACTG	250	397	0	0	0	0
POLR1B	ACACACACTGACCAACCA	189	223	1	2	3	3
POLR1B	ACACACACTGACCACATG	384	606	1	1	0	0
POLR1B	CAACACACTGACCAACGT	484	388	0	0	0	0
POLR1B	GTACACACTGACCAACAC	46	38	0	0	0	0
POLR1B	TGACACACTGACCAACTG	96	235	0	0	0	0
POLR1C	ACACACACTGACCAGTAC	186	112	0	0	0	0
POLR1C	CAACACACTGACCAGTTG	1682	1403	102	0	0	0
POLR1C	GTACACACTGACCACAGT	391	94	0	1	0	0
POLR1C	CAACACACTGACCACACA	70	88	0	0	0	0
POLR1C	TGACACACTGACCACAAC	30	33	0	0	0	0
POLR1D	GTACACTGGTGTGTGTTG	765	1196	0	0	1	0
POLR1D	TGACACTGGTGTGTCAGT	99	82	0	1	0	0
POLR1D	GTACACTGGTGTGTCACA	59	76	0	0	0	1
POLR1D	ACACACTGGTGTGTCAAC	21	47	0	0	0	0
POLR1D	CAACACTGGTGTGTCATG	173	112	0	0	0	1
POLR1E	CATGCACATGACCATGTG	585	720	1	0	1	0
POLR1E	GTACACTGGTGTCAACCA	356	470	0	0	0	1
POLR1E	ACACACTGGTGTCAACAC	43	30	0	0	0	0
POLR1E	CAACACTGGTGTCAACTG	529	599	0	0	0	0
POLR1E	GTACACTGGTGTCATGGT	365	364	1	0	0	0
POLR1E	ACTGCACATGACCATGAC	229	175	0	0	0	0
POLR2A	CAACGTTGACGTACTGCA	68	118	0	0	0	0
POLR2A	TGACGTTGACGTACTGAC	2	6	0	0	0	0
POLR2A	ACACGTTGACGTACTGTG	179	243	0	0	0	0
POLR2A	GTACGTTGACGTTGGTGT	798	1829	2	0	0	7
POLR2A	CAACGTTGACGTTGGTCA	73	66	0	0	0	1
POLR2A	GTTGCAACGTCAGTGTTG	101	124	0	0	0	0
POLR2B	CATGTGACTGCAACCAGT	222	619	1	0	1	0
POLR2B	TGACGTTGACGTTGGTAC	30	5	0	0	0	0
POLR2B	ACACGTTGACGTTGGTTG	72	94	0	0	0	0
POLR2B	CAACGTTGACGTTGCAGT	66	86	1	0	0	0
POLR2B	ACACGTTGACGTTGCACA	112	141	0	0	0	0
POLR2B	GTACGTTGACGTTGCAAC	9	18	0	0	0	0
POLR2B	ACTGCACACAGTCAACTG	603	816	0	0	0	0
POLR2B	CATGTGACACGTCACACA	39	36	0	0	0	0
POLR2B	TGTGTGACACGTCACAAC	17	4	0	0	0	2
POLR2B	ACTGTGACACGTCACATG	101	41	0	0	0	0
POLR2B	GTTGTGACACGTCAACAC	35	27	0	0	0	0
POLR2B	TGTGTGACACGTCAACTG	59	23	0	0	0	1
POLR2B	ACTGTGACACGTCATGGT	50	311	0	0	0	0
POLR2B	GTTGTGACACGTCATGTG	157	720	0	2	0	0
POLR2B	ACTGTGACACGTACGTGT	72	61	0	0	0	0
POLR2B	TGTGTGACACGTACGTCA	38	59	0	1	0	0
POLR2B	TGTGTGACACGTACCAGT	82	101	0	1	0	0
POLR2B	GTTGTGACACGTACCACA	114	224	0	0	0	0
POLR2B	ACTGTGACACGTACCAAC	14	24	0	0	0	0
POLR2B	CATGTGACACGTACACCA	98	87	0	0	0	0
POLR2B	TGTGTGACACGTACACAC	119	43	0	1	0	0
POLR2B	ACTGTGACACGTACACTG	214	129	0	0	0	1
POLR2B	ACTGTGACTGCACATGTG	536	507	0	0	0	0
POLR2B	GTTGTGACTGCAACGTGT	1329	684	0	1	0	0
POLR2B	CATGTGACTGCAACGTCA	467	412	0	0	0	0
POLR2B	ACTGTGACTGCAACCACA	338	83	0	0	0	0
POLR2B	GTTGTGACTGCAACCAAC	36	23	0	0	0	0
POLR2C	TGACGTTGACGTTGCATG	544	339	0	0	0	0
POLR2C	ACACGTTGACGTTGACGT	405	53	0	0	0	0
POLR2C	TGACGTTGACGTTGACCA	183	268	3	1	0	0
POLR2C	CAACGTTGACGTTGACAC	12	22	0	0	0	0
POLR2C	GTACGTTGACGTTGACTG	290	420	1	0	0	0
POLR2D	CAACGTTGACGTTGTGTG	570	158	0	1	0	0
POLR2D	ACACGTTGACCAGTGTGT	95	83	0	0	0	0
POLR2D	TGACGTTGACCAGTGTCA	54	62	0	0	0	0
POLR2D	CAACGTTGACCAGTGTAC	85	309	0	0	0	0
POLR2D	CATGCAGTCAACTGTGTG	611	532	1	0	0	0
POLR2D	TGTGCAACGTCAGTCAGT	33	58	0	0	0	0
POLR2F	TGACGTTGACTGGTACGT	69	50	0	0	0	0
POLR2F	GTACGTTGACTGGTACCA	105	136	0	0	0	0
POLR2F	ACACGTTGACTGGTACAC	57	56	0	0	0	0
POLR2F	CAACGTTGACTGGTACTG	216	215	1	0	0	0
POLR2F	GTACGTTGACTGGTTGGT	247	272	0	0	0	0
POLR2F	TGTGCAACGTCAGTACAC	7	25	0	0	0	0
POLR2G	CAACGTTGACTGCAGTCA	111	162	0	0	0	0

POLR2G	TGACGTTGACTGCAGTAC	6	23	0	0	0	0
POLR2G	ACACGTTGACTGCAGTTG	296	613	0	0	0	0
POLR2G	CAACGTTGACTGCACAGT	100	252	0	0	0	0
POLR2G	ACACGTTGACTGCACACA	83	120	0	0	0	0
POLR2G	CATGCACACAGTCATGGT	487	614	0	3	0	1
POLR2H	GTACACACTGTGCATGCA	50	87	0	0	0	0
POLR2H	ACACACACTGTGCATGAC	85	74	0	1	0	0
POLR2H	CAACACACTGTGCATGTG	419	689	2	2	0	1
POLR2H	TGACACACTGTGACGTGT	1081	547	1	0	0	2
POLR2H	GTACACACTGTGACGTCA	231	229	0	1	0	0
POLR2H	GTTGCAACGTCAGTCACA	166	175	0	0	0	3
POLR2I	GTACGTACACGTACTGCA	156	350	0	1	0	0
POLR2I	ACACGTACACGTACTGAC	21	8	0	0	0	0
POLR2I	CAACGTACACGTACTGTG	217	228	0	0	0	0
POLR2I	TGACGTACACGTTGGTGT	130	116	0	0	0	0
POLR2I	ACTGACGTCACAACTGCA	334	181	0	0	0	0
POLR2J	GTACGTTGACTGCACAAC	10	15	1	0	1	1
POLR2J	TGACGTTGACTGCACATG	244	340	0	0	0	0
POLR2J	ACACGTTGACTGCAACGT	41	83	0	0	0	0
POLR2J	TGACGTTGACTGCAACCA	72	81	0	0	0	0
POLR2J	GTACACACTGTGGTACCA	117	151	0	0	0	0
POLR2K	ACACGTTGACTGACGTAC	101	64	19	10	24	28
POLR2K	CAACGTTGACTGACGTTG	249	244	0	0	1	0
POLR2K	CAACGTTGACTGCATGTG	114	233	0	0	0	0
POLR2K	TGACGTTGACTGACGTGT	52	8	0	0	0	0
POLR2K	GTACGTTGACTGACGTCA	134	106	0	0	0	0
POLR2L	CAACGTTGACTGACTGAC	22	41	0	0	0	0
POLR2L	GTACGTTGACTGACTGTG	335	740	0	0	0	0
POLR2L	ACTGCAGTGTACTGACCA	10	14	0	0	0	0
POLR2L	GTTGCAGTGTACTGACAC	58	67	0	0	0	0
POLR2L	TGTGCAGTGTACTGACTG	269	416	1	1	0	0
POLR3A	ACACACACTGTGGTTGTG	182	215	0	1	0	0
POLR3A	GTACACACTGTGCAGTGT	1181	766	1	0	0	0
POLR3A	CAACACACTGTGCAGTCA	308	432	1	0	0	0
POLR3A	TGACACACTGTGCAGTAC	34	38	0	0	0	0
POLR3A	GTTGGTTGTGACCAACAC	3	9	0	0	0	0
POLR3B	ACACACTGGTGTGTACTG	305	270	1	0	0	1
POLR3B	CAACACTGGTGTGTTGGT	1034	196	1	0	0	1
POLR3B	ACACACTGGTGTGTTGCA	20	41	1	0	0	0
POLR3B	GTACACTGGTGTGTTGAC	64	67	0	0	0	35
POLR3B	TGACACTGGTGTGTTGTG	194	207	0	0	0	0
POLR3G	CAACGTTGACGTGTGTTG	697	857	0	0	1	16
POLR3G	TGACGTTGACGTGTGTGT	271	325	0	0	0	0
POLR3G	GTACGTTGACGTGTGTCA	430	359	0	1	0	0
POLR3G	ACACGTTGACGTGTGTAC	60	77	0	0	0	0
POLR3G	GTACGTTGACGTGTCAGT	94	79	0	0	0	0
POLR3GL	TGTGGTACCAGTCAACCA	308	357	1	0	0	1
POLR3GL	CATGGTACCAGTCAACAC	128	125	1	0	0	0
POLR3GL	GTTGGTACCAGTCAACTG	1200	769	1	1	0	220
POLR3GL	TGTGGTACCAGTCATGGT	657	767	0	0	0	0
POLR3GL	GTTGGTTGTGACCACAGT	615	496	0	0	0	13
POLR3GL	GTTGCAACGTGTACCATG	110	152	1	0	0	0
POLR3H	CAACACACTGTGGTGTGT	164	156	0	0	0	0
POLR3H	ACACACACTGTGGTGTCA	172	157	1	1	0	0
POLR3H	GTACACACTGTGGTGTAC	17	18	0	0	0	0
POLR3H	TGACACACTGTGGTGTTG	1370	1258	3	0	0	12
POLR3H	GTTGCAGTCAGTACTGCA	57	80	0	0	0	0
POLR3K	GTACGTACACACCAACGT	107	138	0	0	0	0
POLR3K	CAACGTACACACCAACCA	270	250	0	1	0	0
POLR3K	TGACGTACACACCAACAC	43	38	0	0	0	0
POLR3K	ACACGTACACACCAACTG	971	833	3	3	0	0
POLR3K	CATGCAGTACACACTGAC	32	41	0	0	0	0
POLR3K	ACTGCAACGTCAGTCAAC	33	47	0	0	0	0
POLRMT	ACACACACTGTGACTGGT	139	212	1	1	0	0
POLRMT	TGACACACTGTGACTGCA	32	20	0	0	0	0
POLRMT	CAACACACTGTGACTGAC	84	75	0	0	0	0
POLRMT	GTACACACTGTGACTGTG	716	514	0	0	0	0
POLRMT	CATGACACGTCACAACAC	55	61	0	0	0	0
POLS	ACACACACTGTGGTACAC	10	9	0	0	0	0
POLS	CAACACACTGTGGTACTG	271	182	1	0	0	0
POLS	GTACACACTGTGGTTGGT	25	34	0	0	0	0
POLS	CAACACACTGTGGTTGCA	89	49	0	0	0	0
POLS	TGACACACTGTGGTTGAC	32	40	0	0	0	0
POMC	CATGGTGTGTCAGTTGCA	47	94	0	0	0	0
POMC	TGTGGTGTGTCAGTTGAC	57	32	0	0	0	0

POMC	ACTGGTGTGTCAGTTGTG	300	227	1	1	0	1
POMC	GTTGGTGTGTCACAGTGT	625	694	0	0	0	1
POMC	CATGGTGTGTCACAGTCA	393	421	0	0	0	1
PON1	GTTGCATGCACAGTGTGT	410	1092	0	0	1	0
PON1	ACACACCATGTGTGGTCA	131	138	201	0	0	1
PON1	GTACACCATGTGTGGTAC	31	42	0	0	0	0
PON1	TGACACCATGTGTGGTTG	728	550	0	0	0	0
PON1	ACACACCATGTGTGCAGT	114	59	0	0	0	0
PON1	TGACACCATGTGTGCACA	57	34	0	0	0	0
PON2	ACACACACACGTCACATG	78	62	0	0	0	0
PON2	CAACACACACGTCAACGT	408	329	0	0	0	0
PON2	ACACACACACGTCAACCA	284	300	0	2	0	0
PON2	GTACACACACGTCAACAC	16	67	0	0	0	0
PON2	TGACACACACGTCAACTG	167	130	0	0	0	0
PON3	TGACACACGTACTGACTG	60	59	0	0	0	0
PON3	ACACACACGTACTGTGGT	176	151	0	0	0	0
PON3	TGACACACGTACTGTGCA	110	120	18	0	0	0
PON3	CAACACACGTACTGTGAC	103	127	0	0	0	0
PON3	GTACACACGTACTGTGTG	217	256	0	0	0	0
PON3	ACTGCATGGTACCATGAC	60	41	0	0	0	0
POR	ACACACGTTGTGGTACCA	102	132	0	15	483	0
POR	ACACACGTTGTGGTTGGT	191	277	1	0	1	7
POR	GTACACGTTGTGGTACAC	60	57	0	0	0	0
POR	TGACACGTTGTGGTACTG	308	250	0	1	0	2
POR	TGACACGTTGTGGTTGCA	259	381	1	0	0	0
PORCN	GTTGGTGTCAGTCAACGT	829	742	1	0	0	0
PORCN	ACTGGTACGTTGGTTGCA	74	114	3	0	0	0
PORCN	GTTGGTACGTTGGTTGAC	87	28	0	0	0	0
PORCN	TGTGGTACGTTGGTTGTG	435	407	0	0	0	0
PORCN	CATGGTACGTTGCAGTGT	486	515	0	0	0	0
POSTN	TGTGGTCACACAGTGTGT	383	498	2	0	0	27
POSTN	GTTGGTCACACAGTGTCA	165	220	0	1	0	11
POSTN	ACTGGTCACACAGTGTAC	115	75	0	0	0	3
POSTN	CATGGTCACACAGTGTTG	305	361	1	0	0	0
POSTN	GTTGGTCACACAGTCAGT	110	71	0	0	0	5
POSTN	TGTGCACAACGTTGACAC	104	103	0	0	0	4
POU2AF1	GTACGTTGACACCATGTG	206	133	0	0	0	0
POU2AF1	ACACGTTGACACACGTGT	236	239	0	0	0	0
POU2AF1	TGACGTTGACACACGTCA	116	142	0	0	0	2
POU2AF1	CAACGTTGACACACGTAC	110	94	0	0	0	0
POU2AF1	TGTGCAGTACTGGTGTCA	286	392	0	1	0	0
POU2AF1	TGTGCACAACGTTGTGTG	376	456	1	0	0	4
POU2F2	GTACGTTGCATGGTCAGT	398	365	0	28	0	0
POU2F2	CAACGTTGCATGGTCACA	195	53	0	0	0	1
POU2F2	TGACGTTGCATGGTCAAC	68	47	0	1	0	0
POU2F2	ACACGTTGCATGGTCATG	247	47	0	0	0	0
POU2F2	CAACGTTGCATGGTACGT	448	341	1	0	0	0
POU2F2	ACTGCACACAGTCATGCA	255	340	1	1	0	0
POU5F1	ACCATGTGCAACTGCAAC	2	6	0	0	0	0
POU5F1	CACATGTGCAACTGCATG	206	141	0	0	0	0
POU5F1	GTCATGTGCAACTGACGT	418	688	0	1	0	1
POU5F1	CACATGTGCAACTGACCA	126	110	1	2	0	0
POU5F1	TGCATGTGCAACTGACAC	67	47	10	0	0	0
PPAP2A	ACCATGACCAACTGTGTG	22	113	0	0	0	0
PPAP2A	CACATGACCATGGTGTGT	190	191	0	0	0	0
PPAP2A	ACCATGACCATGGTGTCA	528	373	2	2	0	3
PPAP2A	GTCATGACCATGGTGTAC	112	87	0	0	0	0
PPAP2A	GTTGCAGTGTTGCACACA	150	243	0	0	0	0
PPAP2A	GTTGCATGGTACGTACCA	214	190	0	0	0	0
PPAP2B	GTACACACGTGTCAACTG	273	315	0	1	2	0
PPAP2B	ACACACACGTGTCAACGT	140	192	0	1	0	0
PPAP2B	TGACACACGTGTCAACCA	173	94	0	1	0	0
PPAP2B	CAACACACGTGTCAACAC	18	12	0	0	0	0
PPAP2B	ACTGCAGTTGGTTGCACA	110	200	1	1	0	0
PPAP2B	ACTGCATGGTACGTACAC	44	27	0	0	0	0
PPAP2C	ACCATGACCATGGTCAGT	153	101	0	0	1	0
PPAP2C	GTCATGACCATGGTCATG	319	289	0	0	1	0
PPAP2C	TGCATGACCATGGTGTTG	1075	394	0	1	0	1
PPAP2C	TGCATGACCATGGTCACA	69	71	0	0	0	0
PPAP2C	CACATGACCATGGTCAAC	12	34	0	0	0	0
PPAP2C	CATGCATGGTACGTACTG	283	816	0	0	0	2
PPARA	CACATGACGTCACACAGT	241	255	2	0	1	0
PPARA	ACCATGACGTCACACACA	272	254	0	0	0	1
PPARA	GTCATGACGTCACACAAC	24	32	0	0	0	0
PPARA	TGCATGACGTCACACATG	135	116	0	0	0	3

PPARA	ACCATGACGTCACAACGT	99	74	0	0	0	0
PPARBP	TGACGTTGGTCAACTGAC	96	166	0	0	0	0
PPARBP	ACACGTTGGTCAACTGTG	647	493	0	0	0	0
PPARBP	GTACGTTGGTCATGGTGT	992	1282	0	0	0	1
PPARBP	CAACGTTGGTCATGGTCA	860	458	0	1	0	1
PPARBP	TGACGTTGGTCATGGTAC	102	66	0	0	0	0
PPARBP	CATGCATGGTCACACATG	191	172	1	0	0	0
PPARD	CACATGACGTCACAGTCA	482	458	0	0	0	0
PPARD	TGCATGACGTCACAGTAC	100	121	0	0	0	0
PPARD	ACCATGACGTCACAGTTG	1291	833	2	2	0	0
PPARD	GTTGCAGTACGTACGTTG	400	641	1	1	0	0
PPARD	GTTGCATGGTACGTTGGT	154	139	0	0	0	1
PPARD	CATGCATGGTACGTTGCA	258	302	1	0	0	0
PPARG	TGCATGACGTCACAACCA	427	395	3	351	2159	1
PPARG	CACATGACGTCACAACAC	43	90	0	0	0	0
PPARG	GTCATGACGTCACAACTG	188	167	0	0	0	0
PPARG	TGCATGACGTCACATGGT	154	337	1	1	0	1
PPARG	GTCATGACGTCACATGCA	274	503	1	0	0	1
PPARGC1A	CACATGCATGACACTGGT	141	232	0	0	0	0
PPARGC1A	ACCATGCATGACACTGCA	35	87	0	0	0	0
PPARGC1A	GTCATGCATGACACTGAC	61	42	0	0	0	0
PPARGC1A	TGCATGCATGACACTGTG	670	332	4	7	0	6
PPARGC1A	CACATGCATGACTGGTGT	329	654	0	0	0	1
PPAT	ACACTGTGCACAGTGTCA	311	261	0	0	0	0
PPAT	GTACTGTGCACAGTGTAC	11	9	0	0	0	0
PPAT	TGACTGTGCACAGTGTTG	315	680	2	0	0	1
PPAT	ACACTGTGCACAGTCAGT	99	51	0	0	0	0
PPAT	TGACTGTGCACAGTCACA	61	73	0	1	0	0
PPAT	TGTGCATGGTACCATGGT	251	254	1	0	0	0
PPBP	GTACTGGTCACATGACAC	106	40	1	0	0	0
PPBP	TGACTGGTCACATGACTG	286	268	6	0	0	0
PPBP	ACACTGGTCACATGTGGT	199	264	0	0	0	0
PPBP	TGACTGGTCACATGTGCA	258	440	0	0	0	0
PPBP	CAACTGGTCACATGTGAC	124	109	0	0	0	1
PPCDC	ACTGGTACCAACTGTGGT	1030	405	0	2	1	0
PPCDC	TGTGGTACCAACTGACTG	653	545	2	0	0	1
PPCDC	TGTGGTACCAACTGTGCA	224	161	0	0	0	0
PPCDC	CATGGTACCAACTGTGAC	34	56	0	0	0	0
PPCDC	ACTGGTTGACACGTACAC	97	144	0	0	0	0
PPCS	TGTGGTCATGCAGTTGGT	835	415	3	0	1	0
PPCS	GTTGGTCATGCAGTTGCA	178	274	0	1	0	0
PPCS	ACTGGTCATGCAGTTGAC	6	19	0	0	0	0
PPCS	CATGGTCATGCAGTTGTG	186	181	0	0	0	0
PPCS	TGTGGTCATGCACAGTGT	1114	1050	66	1	0	1
PPFIA4	GTACACACTGCATGCAGT	119	120	1	0	0	2
PPFIA4	CAACACACTGCATGCACA	69	141	0	0	0	11
PPFIA4	GTTGCAGTTGCATGGTCA	1019	1191	0	1	0	4
PPFIA4	CATGCAGTTGACTGTGCA	431	532	1	0	0	1
PPFIA4	TGTGACGTACTGTGGTCA	67	41	0	0	0	0
PPIC	GTACACCAACACTGCATG	326	278	0	0	0	0
PPIC	TGACACCAACACTGACGT	173	157	0	0	0	0
PPIC	GTACACCAACACTGACCA	256	107	0	1	0	1
PPIC	ACACACCAACACTGACAC	52	44	0	0	0	2
PPIC	CAACACCAACACTGACTG	456	388	1	0	0	0
PPIE	ACACACCAACACACCAAC	52	44	0	0	0	0
PPIE	ACACACCAACTGCAGTTG	1026	520	0	0	0	4
PPIE	CAACACCAACTGCACAGT	196	111	0	1	0	0
PPIE	ACACACCAACTGCACACA	311	386	0	0	0	0
PPIE	GTACACCAACTGCACAAC	26	17	0	0	0	0
PPM1D	GTCATGACACGTACTGTG	304	168	0	0	0	0
PPM1D	ACCATGACACGTTGGTGT	844	767	0	1	0	3
PPM1D	TGCATGACACGTTGGTCA	163	241	0	0	0	1
PPM1D	CACATGACACGTTGGTAC	54	55	0	0	0	2
PPM1D	GTCATGACACGTTGGTTG	458	612	0	0	0	0
PPM1D	CATGCATGGTACCAACAC	56	92	47	0	0	0
PPME1	ACTGCACAACTGTGCACA	324	184	0	5	1	3
PPME1	GTCATGACACCAGTTGCA	194	296	6	0	0	5
PPME1	ACCATGACACCAGTTGAC	30	54	0	0	0	0
PPME1	CACATGACACCAGTTGTG	608	1000	0	0	0	0
PPME1	TGCATGACACCACAGTGT	342	231	1	0	0	0
PPME1	TGACCAGTTGTGACACAC	50	60	1	0	0	0
PPOX	GTACACGTACTGCAGTCA	447	435	1	0	0	0
PPOX	ACACACGTACTGCAGTAC	15	40	0	0	0	0
PPOX	CAACACGTACTGCAGTTG	488	633	0	1	0	0
PPOX	GTACACGTACTGCACAGT	400	682	1	1	0	0

PPOX	CAACACGTACTGCACACA	62	87	0	0	0	1
PPP1CA	GTCATGACCACATGTGTG	504	402	1	0	0	0
PPP1CA	GTCATGACCAACGTGTGT	1185	606	0	0	0	0
PPP1CA	CACATGACCAACGTGTCA	122	82	0	1	0	0
PPP1CA	TGCATGACCAACGTGTAC	245	80	0	0	0	0
PPP1CA	ACCATGACCAACGTGTTG	397	189	0	0	0	1
PPP1CB	CACATGACCAACGTCAGT	350	654	0	1	0	0
PPP1CB	ACCATGACCAACGTCACA	111	110	0	0	0	1
PPP1CB	GTCATGACCAACGTCAAC	15	30	0	0	0	0
PPP1CB	TGCATGACCAACGTCATG	163	229	0	0	0	0
PPP1CB	ACCATGACCAACGTACGT	55	31	0	0	0	0
PPP1CC	TGCATGACCAACGTACCA	217	278	0	0	0	0
PPP1CC	CACATGACCAACGTACAC	23	38	0	0	0	0
PPP1CC	GTCATGACCAACGTACTG	215	215	3	0	0	0
PPP1CC	TGCATGACCAACGTTGGT	248	93	0	0	0	0
PPP1CC	GTCATGACCAACGTTGCA	284	131	0	0	0	0
PPP1CC	GTTGCACACAGTCATGAC	19	32	0	0	0	0
PPP1R10	CACATGACCAACGTTGTG	346	335	1	0	1	0
PPP1R10	ACCATGACCAACGTTGAC	67	65	0	0	0	1
PPP1R10	TGCATGACCAACCAGTGT	323	193	0	0	0	0
PPP1R10	GTCATGACCAACCAGTCA	93	58	0	0	0	0
PPP1R10	ACCATGACCAACCAGTAC	56	54	0	0	0	0
PPP1R10	ACTGCAACTGACACTGAC	46	210	0	0	0	0
PPP1R12B	ACACGTGTCACAGTACAC	39	97	0	0	0	0
PPP1R12B	CAACGTGTCACAGTACTG	951	904	0	2	0	0
PPP1R12B	TGTGGTTGTGGTCATGTG	252	207	0	1	0	0
PPP1R12B	CATGCAACGTGTACACTG	940	582	1	0	0	1
PPP1R12B	GTTGCAACGTGTACTGGT	150	120	0	0	0	0
PPP1R12B	CATGCAACGTGTACTGCA	256	234	0	0	0	1
PPP1R13B	CACATGACGTACTGCAGT	229	407	0	0	0	0
PPP1R13B	ACCATGACGTACTGCACA	62	86	0	0	0	1
PPP1R13B	GTCATGACGTACTGCAAC	23	17	0	0	0	0
PPP1R13B	TGCATGACGTACTGCATG	44	45	0	0	0	9
PPP1R13B	TGTGACACCATGCACATG	229	431	1	0	0	1
PPP1R13L	GTACGTTGTGGTTGACAC	73	56	0	0	0	0
PPP1R13L	TGACGTTGTGGTTGACTG	160	282	1	0	0	0
PPP1R13L	ACACGTTGTGGTTGTGGT	568	350	0	0	0	2
PPP1R13L	TGACGTTGTGGTTGTGCA	185	260	0	0	0	4
PPP1R13L	GTTGCAGTCATGGTTGCA	401	373	3	0	0	2
PPP1R1B	GTACACACTGACCAGTCA	463	731	0	1	1	0
PPP1R1B	ACACACACTGACGTTGAC	123	92	1	0	0	0
PPP1R1B	CAACACACTGACGTTGTG	558	540	1	1	0	0
PPP1R1B	TGACACACTGACCAGTGT	1163	1057	0	1	0	37
PPP1R1B	TGTGCATGGTTGTGACTG	187	599	1	0	0	2
PPP1R1B	ACTGCATGGTTGTGTGGT	226	219	1	0	0	0
PPP1R3A	GTACGTGTCACAGTTGGT	147	298	2	0	0	0
PPP1R3A	CAACGTGTCACAGTTGCA	112	127	0	1	0	0
PPP1R3A	TGACGTGTCACAGTTGAC	26	39	0	0	0	0
PPP1R3A	ACACGTGTCACAGTTGTG	329	292	1	0	0	0
PPP1R3A	GTACGTGTCACACAGTGT	798	874	0	2	0	0
PPP2CA	CACATGACCAACCAGTTG	537	633	0	1	2	0
PPP2CA	GTCATGACCAACCACAGT	128	170	0	0	0	0
PPP2CA	CACATGACCAACCACACA	260	206	0	0	0	0
PPP2CA	TGCATGACCAACCACAAC	39	24	0	0	0	0
PPP2CA	ACCATGACCAACCACATG	755	707	0	0	0	0
PPP2R1A	GTCATGACCAACTGACCA	38	38	0	0	0	0
PPP2R1A	ACCATGACCAACTGACAC	81	80	0	0	0	0
PPP2R1A	CATGGTTGTGGTCATGGT	209	148	0	0	0	0
PPP2R1A	ACTGGTTGTGGTCATGCA	167	130	1	0	0	0
PPP2R1A	GTTGGTTGTGGTCATGAC	27	24	0	0	0	0
PPP2R1A	CATGCATGCACAACTGGT	577	717	1	0	0	4
PPP2R2A	CACATGACCAACCAACGT	312	358	1	1	0	2
PPP2R2A	ACCATGACCAACCAACCA	43	189	0	0	0	0
PPP2R2A	GTCATGACCAACCAACAC	124	192	1	0	0	0
PPP2R2A	TGCATGACCAACCAACTG	1161	828	0	3	0	49
PPP2R2A	ACCATGACCAACCATGGT	413	361	0	1	0	0
PPP2R2A	CATGCATGCACACACACA	585	293	1	1	0	0
PPP2R2C	CAACGTGTCACACAGTCA	958	647	0	1	0	1
PPP2R2C	TGACGTGTCACACAGTAC	120	134	0	1	0	4
PPP2R2C	ACACGTGTCACACAGTTG	966	543	0	1	0	0
PPP2R2C	CAACGTGTCACACACAGT	215	239	0	0	0	0
PPP2R2C	ACACGTGTCACACACACA	60	75	0	0	0	0
PPP2R5B	TGACCATGCAACCACATG	220	299	166	4	27	18
PPP2R5B	ACACCATGCAACCACACA	76	107	0	0	0	0
PPP2R5B	GTACCATGCAACCACAAC	24	86	0	0	0	11

PPP2R5B	ACACCATGCAACCAACGT	99	105	1	1	0	0
PPP2R5B	TGACCATGCAACCAACCA	601	157	1	1	0	0
PPP2R5B	GTTGCATGGTTGGTCACA	43	40	0	1	0	0
PPP2R5C	ACCATGACCAACACGTGT	1065	908	3	0	1	0
PPP2R5C	TGCATGACCAACCATGCA	381	552	0	0	0	0
PPP2R5C	CACATGACCAACCATGAC	19	19	0	0	0	0
PPP2R5C	GTCATGACCAACCATGTG	537	604	0	0	0	1
PPP2R5C	TGACTGTGACACACGTGT	562	500	1	0	0	0
PPP2R5C	TGTGCATGGTTGCAACGT	426	622	3	2	0	83
PPP2R5D	GTCATGACCAACTGCATG	117	126	5	2923	1	1
PPP2R5D	TGCATGACCAACTGCACA	107	162	1	1	0	0
PPP2R5D	CACATGACCAACTGCAAC	47	35	0	0	0	0
PPP2R5D	TGCATGACCAACTGACGT	33	38	0	0	0	0
PPP2R5D	GTACTGTGACACACGTCA	120	110	0	0	0	0
PPP2R5D	GTTGCATGGTTGCAACCA	343	157	0	0	0	0
PPP3CA	ACCATGACCATGCATGAC	76	94	0	0	0	0
PPP3CA	CACATGACCATGCATGTG	156	182	0	2	0	0
PPP3CA	TGCATGACCATGACGTGT	1080	1043	0	0	0	2
PPP3CA	GTCATGACCATGACGTCA	141	209	0	0	0	0
PPP3CA	ACCATGACCATGACGTAC	172	97	1	0	0	0
PPP3CB	TGCATGACCATGTGACAC	74	200	0	0	1	0
PPP3CB	ACCATGACCATGTGACTG	182	150	0	0	0	0
PPP3CB	CACATGACCATGTGTGGT	242	230	0	0	0	0
PPP3CB	ACCATGACCATGTGTGCA	166	183	0	0	0	2
PPP3CB	GTCATGACCATGTGTGAC	19	49	0	0	0	0
PPP3CB	GTTGCACATGCATGCAAC	9	1	0	0	0	0
PPP3CC	GTACGTGTCACACACAAC	31	37	0	0	0	0
PPP3CC	TGACGTGTCACACACATG	60	79	0	0	0	0
PPP3CC	ACACGTGTCACACAACGT	271	208	0	0	0	8
PPP3CC	TGACGTGTCACACAACCA	147	145	0	0	0	0
PPP3CC	CAACGTGTCACACAACAC	17	79	0	0	0	7
PPP3R1	CACATGACCATGACCACA	63	73	0	0	1	0
PPP3R1	CACATGACCATGACGTTG	1360	1213	1	0	0	0
PPP3R1	GTCATGACCATGACCAGT	304	339	0	0	0	1
PPP3R1	TGCATGACCATGACCAAC	12	4	0	0	0	0
PPP3R1	ACCATGACCATGACCATG	62	108	0	0	0	0
PPP3R2	GTACGTGTCACACAACTG	224	223	0	0	0	0
PPP3R2	TGACGTGTCACACATGGT	444	173	1	0	0	3
PPP3R2	GTACGTGTCACACATGCA	135	134	0	0	0	2
PPP3R2	ACACGTGTCACACATGAC	50	34	0	0	0	0
PPP3R2	CAACGTGTCACACATGTG	301	397	1	1	0	1
PPP5C	GTCATGACCATGTGCACA	68	67	0	0	0	1
PPP5C	ACCATGACCATGTGCAAC	84	23	0	0	0	0
PPP5C	CACATGACCATGTGCATG	94	109	0	0	0	0
PPP5C	GTCATGACCATGTGACGT	139	143	1	0	0	0
PPP5C	CACATGACCATGTGACCA	102	122	0	1	0	4
PPP5C	TGTGCAACCAACCAGTAC	194	241	2	1	0	1
PPP6C	CACATGACCATGACACGT	29	18	0	0	0	0
PPP6C	ACCATGACCATGACACCA	198	436	0	0	0	0
PPP6C	GTCATGACCATGACACAC	37	35	0	0	0	0
PPP6C	TGCATGACCATGACACTG	288	291	0	0	0	0
PPP6C	CAACTGTGACACACGTTG	159	175	1	0	0	0
PPP6C	CATGCAACCAACGTTGCA	49	76	0	0	0	11
PPT1	CAACCAGTACCACATGCA	789	492	1	0	3	0
PPT1	CAACCAGTACCACAACTG	520	343	1	1	1	0
PPT1	ACACCAGTACCACAACAC	167	58	0	0	0	0
PPT1	GTACCAGTACCACATGGT	542	863	0	1	0	0
PPT1	TGTGCAGTCAGTACGTAC	58	148	0	1	0	0
PPT2	TGACCAGTACCACACACA	59	55	2	1	0	0
PPT2	CAACCAGTACCACACAAC	75	43	0	0	0	0
PPT2	GTACCAGTACCACACATG	253	179	0	0	0	0
PPT2	TGACCAGTACCACAACGT	650	582	3	0	0	0
PPT2	GTACCAGTACCACAACCA	438	428	0	0	0	14
PPT2	GTTGCAACCAACTGGTTG	973	869	1	1	0	3
PPYR1	TGACGTCATGTGGTCAGT	315	410	0	1	0	0
PPYR1	GTACGTCATGTGGTCACA	57	52	0	0	0	2
PPYR1	ACACGTCATGTGGTCAAC	20	30	0	0	0	0
PPYR1	CAACGTCATGTGGTCATG	224	179	1	0	0	0
PPYR1	ACTGACGTTGACGTGTAC	41	22	0	0	0	0
PRB1	GTACGTTGACTGTGCACA	144	176	0	0	1	1
PRB1	TGACTGTGCACATGTGTG	254	363	0	0	0	0
PRB1	TGACTGTGCAACGTGTGT	439	731	0	0	0	1
PRB1	GTACTGTGCAACGTGTCA	740	433	2	1	0	0
PRB1	ACACTGTGCAACGTGTAC	45	134	0	0	0	0
PRC1	GTTGGTTGGTTGCAGTTG	394	579	1	0	0	0

PRC1	TGTGGTTGGTTGCACAGT	162	279	2	0	0	0
PRC1	GTTGGTTGGTTGCACACA	189	126	1	0	0	0
PRC1	ACTGGTTGGTTGCACAAC	23	17	0	0	0	0
PRC1	CATGGTTGGTTGCACATG	478	381	1	0	0	0
PRDM1	ACACGTCATGGTCAGTAC	184	157	1	1	0	0
PRDM1	CAACGTCATGGTCAGTTG	1380	1804	8	1	0	12
PRDM1	GTACGTCATGGTCACAGT	64	32	0	0	0	0
PRDM1	CAACGTCATGGTCACACA	62	87	1	0	0	0
PRDM1	TGACGTCATGGTCACAAC	11	45	0	0	0	0
PRDM2	ACACGTCAACCATGTGTG	304	397	0	0	1	1
PRDM2	CAACGTCAACCATGACTG	256	329	0	0	0	0
PRDM2	GTACGTCAACCATGTGGT	188	444	0	1	0	0
PRDM2	CAACGTCAACCATGTGCA	147	91	0	2	0	0
PRDM2	TGACGTCAACCATGTGAC	154	50	0	0	0	1
PRDX1	CAACCAGTTGGTCAGTGT	469	247	4	4	2	0
PRDX1	ACACCAGTTGGTGTTGCA	234	450	0	0	0	0
PRDX1	GTACCAGTTGGTGTTGAC	51	114	0	0	0	0
PRDX1	TGACCAGTTGGTGTTGTG	326	309	0	0	0	0
PRDX1	ACACCAGTTGGTCAGTCA	603	510	0	0	0	4
PRDX1	CATGCATGCACATGGTGT	1165	1082	0	1	0	1
PRDX2	CATGCATGCAACACGTCA	864	821	2	0	1	0
PRDX2	CAACTGACACTGTGACCA	360	50	1	0	0	0
PRDX2	GTTGCAGTCAACCACACA	55	60	0	0	0	0
PRDX2	GTTGCATGCAACACGTGT	184	157	0	0	0	0
PRDX2	TGTGCATGCAACACGTAC	41	52	0	0	0	0
PRDX3	ACACTGACACTGACCATG	118	157	0	0	0	1
PRDX3	CAACTGACACTGACACGT	60	85	0	0	0	1
PRDX3	ACACTGACACTGACACCA	90	150	2	0	0	0
PRDX3	GTACTGACACTGACACAC	37	50	0	0	0	0
PRDX3	ACTGCAGTCACAGTGTCA	473	539	2	1	0	1
PRDX3	TGTGCATGCACATGACGT	236	227	0	0	0	0
PRDX4	TGACTGACACTGCATGGT	839	567	10	6997	1	31
PRDX4	ACACTGACACTGCAACGT	667	695	0	0	0	7
PRDX4	TGACTGACACTGCAACCA	183	165	2	1	0	10
PRDX4	CAACTGACACTGCAACAC	16	27	0	0	0	0
PRDX4	GTACTGACACTGCAACTG	108	89	1	0	0	0
PRDX4	CATGCACAACGTACTGAC	164	58	0	0	0	0
PRDX5	GTACTGTGGTACACCAAC	16	12	0	0	0	0
PRDX5	TGACTGTGGTACACCATG	385	250	1	0	0	0
PRDX5	ACACTGTGGTACACACGT	51	43	0	0	0	0
PRDX5	TGACTGTGGTACACACCA	382	132	0	0	0	0
PRDX5	GTTGCATGCACAACTGAC	38	38	0	0	0	0
PRDX5	TGTGCATGCACAACTGTG	445	216	1	0	0	0
PRDX6	GTACACACACACTGACGT	392	489	0	2	1	0
PRDX6	TGTGCATGGTCACACAGT	184	295	0	0	1	0
PRDX6	CAACACACACACTGACCA	286	329	0	0	0	0
PRDX6	TGACACACACACTGACAC	61	56	0	0	0	0
PRDX6	ACACACACACACTGACTG	126	107	0	0	0	0
PRDX6	GTTGCATGGTCACAGTTG	1329	754	2	0	0	2
PREP	CAACACCATGTGGTCAGT	338	330	1	0	0	0
PREP	ACACACCATGTGGTCACA	195	150	1	0	0	0
PREP	GTACACCATGTGGTCAAC	35	19	0	0	0	0
PREP	TGACACCATGTGGTCATG	136	207	0	0	0	0
PREP	GTTGCAGTCAACTGTGCA	244	377	1	0	0	1
PRF1	GTACGTGTACTGTGCAGT	243	116	0	0	0	0
PRF1	CAACGTGTACTGTGCACA	101	231	0	0	0	0
PRF1	TGACGTGTACTGTGCAAC	13	24	0	0	0	0
PRF1	ACACGTGTACTGTGCATG	119	143	0	0	0	0
PRF1	CAACGTGTACTGTGACGT	44	83	0	0	0	0
PRIM1	TGTGGTTGGTCACAGTGT	782	956	1	1	0	4
PRIM1	GTTGGTTGGTCACAGTCA	359	591	1	0	0	2
PRIM1	ACTGGTTGGTCACAGTAC	42	101	0	0	0	4
PRIM1	CATGGTTGGTCACAGTTG	1185	722	0	0	0	0
PRIM1	GTTGCAGTGTTGTGTGCA	470	191	0	0	0	0
PRIM2	TGTGACGTGTGTGTCATG	71	88	0	0	1	0
PRIM2	TGCATGCACACAGTCACA	148	106	0	0	0	0
PRIM2	CACATGCACACAGTCAAC	49	99	0	0	0	0
PRIM2	GTCATGCACACAGTCATG	115	82	0	0	0	0
PRIM2	GTTGACGTGTGTGTCAAC	37	13	0	0	0	0
PRKAA1	ACCATGCATGGTACGTCA	75	168	0	0	0	0
PRKAA1	GTCATGCATGGTACGTAC	29	15	0	0	0	0
PRKAA1	TGCATGCATGGTACGTTG	103	181	0	0	0	1
PRKAA1	ACCATGCATGGTACCAGT	106	185	0	0	0	0
PRKAA1	TGCATGCATGGTACCACA	11	0	0	0	0	0
PRKAA2	ACCATGACCAGTCATGCA	160	103	0	0	0	0

PRKAA2	GTCATGACCAGTCATGAC	16	17	0	0	0	3
PRKAA2	TGCATGACCAGTCATGTG	367	81	0	0	0	0
PRKAA2	CACATGACCAGTACGTGT	206	274	41	0	0	0
PRKAA2	GTACGTTGTGACCACACA	142	231	0	0	0	0
PRKAB1	ACCATGTGCACATGGTTG	561	415	1	980	5	1
PRKAB1	CACATGTGCACATGGTCA	500	316	1	0	1	0
PRKAB1	ACCATGTGCACATGCACA	26	27	0	0	1	1
PRKAB1	TGCATGTGCACATGGTAC	33	39	0	0	0	0
PRKAB1	CACATGTGCACATGCAGT	39	28	0	0	0	0
PRKAB2	TGCATGACACCATGACGT	349	433	0	0	0	1
PRKAB2	GTCATGACACCATGACCA	152	171	0	1	0	0
PRKAB2	ACCATGACACCATGACAC	12	35	0	0	0	0
PRKAB2	CACATGACACCATGACTG	1531	1289	2	0	0	2
PRKAB2	GTCATGACACCATGTGGT	157	230	0	0	0	0
PRKACA	TGCATGCATGTGCAGTAC	7	16	0	0	0	0
PRKACA	ACCATGCATGTGCAGTTG	218	211	16372	17	0	1
PRKACA	CACATGCATGTGCACAGT	101	52	0	0	0	0
PRKACA	ACCATGCATGTGCACACA	17	18	0	0	0	0
PRKACA	GTACGTCAACCAACGTTG	353	391	0	0	0	0
PRKACB	TGCATGACGTTGCACAAC	14	1	0	0	0	0
PRKACB	ACCATGACGTTGCACATG	263	307	0	0	0	0
PRKACB	CACATGACGTTGCAACGT	338	453	0	0	0	0
PRKACB	CATGGTACTGACACCAAC	10	12	0	0	0	0
PRKACB	GTTGGTACTGACACCATG	546	188	1	5	0	12
PRKACG	ACACGTTGTGCAACACCA	87	97	0	0	0	0
PRKACG	GTACGTTGTGCAACACAC	149	24	0	0	0	0
PRKACG	TGACGTTGTGCAACACTG	650	821	1	2	0	0
PRKACG	ACACGTTGTGCAACTGGT	425	1001	3	8	0	183
PRKACG	GTTGGTACTGACTGCAAC	23	128	0	0	0	0
PRKACG	TGTGCAACGTCATGTGCA	213	428	1	2	0	0
PRKAG1	GTCATGACACCAACCACA	71	87	0	0	0	0
PRKAG1	ACCATGACACCAACCAAC	102	47	0	0	0	0
PRKAG1	CACATGACACCAACCATG	597	508	0	0	0	1
PRKAG1	GTCATGACACCAACACGT	2	8	0	0	0	0
PRKAG1	CACATGACACCAACACCA	334	121	0	0	0	0
PRKAG2	CACATGACACACGTGTAC	25	132	0	1	0	0
PRKAG2	GTCATGACACACGTGTTG	2421	2480	6	1	0	4
PRKAG2	TGCATGACACACGTCAGT	23	92	0	0	0	1
PRKAG2	GTCATGACACACGTCACA	115	284	0	0	0	0
PRKAG2	TGTGACCATGTGACGTGT	540	683	1	0	0	0
PRKAR1A	GTACGTTGTGACTGGTGT	1651	1185	2	0	1	14
PRKAR1A	TGACGTTGTGACTGGTAC	242	149	0	0	0	0
PRKAR1A	CAACCATGCAACCAACAC	221	77	0	0	0	0
PRKAR1A	GTACCATGCAACCAACTG	94	65	0	0	0	0
PRKAR1A	TGACCATGCAACCATGGT	985	896	0	0	0	0
PRKAR1B	TGACGTTGTGACCATGTG	583	541	0	0	1	0
PRKAR1B	GTACACACTGCAGTTGAC	66	44	0	0	0	0
PRKAR1B	TGACACACTGCAGTTGTG	865	447	2	1	0	0
PRKAR1B	CAACACACTGCACAGTGT	277	249	0	0	0	1
PRKAR1B	ACACACACTGCACAGTCA	652	596	1	2	0	0
PRKAR2A	GTACGTTGTGACACGTAC	26	31	0	0	0	1
PRKAR2A	GTACCAACTGGTACCACA	29	52	0	0	0	0
PRKAR2A	ACACCAACTGGTACCAAC	61	67	0	0	0	0
PRKAR2A	CAACCAACTGGTACCATG	114	147	1	0	0	0
PRKAR2A	GTACCAACTGGTACACGT	12	19	0	0	0	0
PRKAR2B	CAACCAACTGGTACACCA	111	83	0	0	0	0
PRKAR2B	TGACCAACTGGTACACAC	52	78	0	0	0	0
PRKAR2B	ACACCAACTGGTACACTG	530	542	0	0	0	0
PRKAR2B	CAACCAACTGGTACTGGT	183	196	0	0	0	0
PRKAR2B	ACTGGTACTGTGACCAAC	3	1	0	0	0	0
PRKCA	CACATGACGTCAACACGT	26	19	0	0	0	0
PRKCA	ACCATGACGTCAACACCA	100	206	0	0	0	0
PRKCA	GTCATGACGTCAACACAC	21	9	0	1	0	0
PRKCA	TGCATGACGTCAACACTG	467	332	0	0	0	0
PRKCA	GTTGGTTGGTGTACGTCA	225	213	0	0	0	19
PRKCB1	ACCATGACACCAACTGCA	233	339	116	0	1	0
PRKCB1	TGCATGACACCAACACAC	51	30	0	0	0	0
PRKCB1	ACCATGACACCAACACTG	84	112	0	0	0	0
PRKCB1	CACATGACACCAACTGGT	372	371	0	1	0	0
PRKCB1	CAACGTCAACCAACGTAC	50	41	0	0	0	0
PRKCD	ACTGGTTGGTGTCAACGT	183	130	0	2	1	0
PRKCD	TGTGGTTGGTGTCAACCA	13	31	0	0	0	3
PRKCD	CATGGTTGGTGTCAACAC	21	62	1	0	0	0
PRKCD	GTTGGTTGGTGTCAACTG	222	638	0	0	0	0
PRKCD	CATGCAGTGTTGTGTGTG	139	209	0	0	0	0

PRKCE	ACCATGCATGGTCAACTG	163	243	0	1	1	0
PRKCE	GTCATGCATGGTCAACGT	100	84	0	0	0	0
PRKCE	CACATGCATGGTCAACCA	441	490	0	0	0	0
PRKCE	TGCATGCATGGTCAACAC	142	250	1	0	0	0
PRKCE	CACATGCATGGTCATGGT	356	365	0	0	0	2
PRKCG	TGCATGACCACACAGTTG	2013	1511	3	0	2	9
PRKCG	ACCATGACCACACACAGT	33	58	0	0	0	2
PRKCG	TGCATGACCACACACACA	109	66	0	0	0	0
PRKCG	CACATGACCACACACAAC	22	27	0	0	0	0
PRKCG	CATGGTACTGACGTTGAC	87	64	0	0	0	1
PRKCH	GTACGTGTGTCACAGTCA	359	467	0	0	0	22
PRKCH	ACACGTGTGTCACAGTAC	30	45	0	0	0	0
PRKCH	CAACGTGTGTCACAGTTG	1524	1289	2	2	0	10
PRKCH	GTACGTGTGTCACACAGT	161	178	1	0	0	0
PRKCH	CAACGTGTGTCACACACA	304	76	0	0	0	0
PRKCI	GTCATGTGTGACCAACCA	396	261	0	56	2	0
PRKCI	TGCATGTGTGACCAACGT	234	306	0	0	0	0
PRKCI	ACCATGTGTGACCAACAC	2	15	0	0	0	0
PRKCI	CACATGTGTGACCAACTG	853	1154	2	1	0	40
PRKCI	TGCATGTGTGACTGCAAC	8	35	0	0	0	7
PRKCQ	CACATGACGTACGTGTTG	246	264	1	0	0	2
PRKCQ	GTCATGACGTACGTCAGT	259	183	2	1	0	0
PRKCQ	CACATGACGTACGTCACA	12	24	0	0	0	0
PRKCQ	TGCATGACGTACGTCAAC	8	24	0	0	0	0
PRKCQ	ACCATGACGTACGTCATG	15	25	0	0	0	0
PRKCZ	CACATGCATGACTGCAAC	23	25	0	0	0	0
PRKCZ	GTCATGCATGACTGCATG	467	305	0	0	0	0
PRKCZ	TGCATGCATGACTGACGT	291	226	1	0	0	0
PRKCZ	GTCATGCATGACTGACCA	273	271	0	1	0	2
PRKCZ	ACCATGCATGACTGACAC	27	40	0	0	0	0
PRKD1	CACATGACCAGTGTACGT	224	690	0	0	1	17
PRKD1	CACATGACCAGTGTCACA	189	381	0	0	0	0
PRKD1	TGCATGACCAGTGTCAAC	7	2	0	0	0	0
PRKD1	ACCATGACCAGTGTCATG	81	70	0	0	0	0
PRKD1	ACCATGACCAGTGTACCA	472	460	0	0	0	0
PRKD3	TGCATGCATGTGTGCAGT	434	424	0	0	1	0
PRKD3	GTCATGCATGTGTGACGT	361	418	0	0	1	2
PRKD3	GTCATGCATGTGTGCACA	73	154	0	0	0	0
PRKD3	ACCATGCATGTGTGCAAC	14	15	0	0	0	0
PRKD3	CACATGCATGTGTGCATG	42	91	0	0	0	0
PRKDC	CAACGTGTGTGTTGTGGT	791	810	150	92842	53	39
PRKDC	CAACGTGTGTGTTGACCA	26	24	0	0	0	0
PRKDC	TGACGTGTGTGTTGACAC	28	26	0	0	0	0
PRKDC	ACACGTGTGTGTTGACTG	99	102	0	0	0	0
PRKDC	ACACGTGTGTGTTGTGCA	116	139	0	0	0	0
PRKDC	CATGCATGGTTGACACAC	48	97	0	0	0	0
PRKG2	GTTGCACAACCAGTGTGT	1737	1552	9	2	1	3
PRKG2	ACCATGACGTGTACGTTG	1002	321	2	2	0	0
PRKG2	CACATGACGTGTACCAGT	68	30	0	0	0	0
PRKG2	ACCATGACGTGTACCACA	53	86	0	1	0	0
PRKG2	GTCATGACGTGTACCAAC	35	30	0	0	0	0
PRKG2	TGCATGACGTGTACCATG	149	100	1	0	0	0
PRKRA	ACACGTTGTGACACGTCA	101	127	0	0	0	0
PRKRA	TGACACACTGCAACCATG	118	122	0	0	0	3
PRKRA	ACACACACTGCAACACGT	158	113	0	0	0	0
PRKRA	TGACACACTGCAACACCA	151	162	1	0	0	0
PRKRA	CAACACACTGCAACACAC	47	60	0	0	0	0
PRKRA	TGTGCATGCACACAGTGT	530	468	0	0	0	0
PRKRIR	GTCATGTGACGTTGACAC	49	35	1	0	0	0
PRKRIR	TGCATGTGACGTTGACTG	107	128	0	0	0	0
PRKRIR	ACCATGTGACGTTGTGGT	673	442	1	1	0	4
PRKRIR	TGCATGTGACGTTGTGCA	36	39	0	0	0	0
PRKRIR	CACATGTGACGTTGTGAC	52	29	0	0	0	0
PRKRIR	GTTGCAACCACACAACGT	450	115	0	0	0	1
PRKX	TGCATGACGTCATGTGTG	305	280	0	0	0	13
PRKX	TGCATGACGTACGTGTGT	652	860	0	9	0	4
PRKX	GTCATGACGTACGTGTCA	120	114	0	0	0	0
PRKX	ACCATGACGTACGTGTAC	33	67	0	0	0	0
PRKX	CAACGTGTGTCACATGAC	140	267	14	0	0	0
PRKX	ACTGCACACATGACTGGT	500	241	1	1	0	0
PRKY	TGACGTGTGTCACATGCA	431	534	3	70	2	1
PRKY	GTACGTGTGTCACATGTG	582	816	0	0	0	0
PRKY	ACACGTGTGTCAACGTGT	227	188	0	0	0	0
PRKY	ACTGGTACTGTGACGTGT	328	250	0	0	0	0
PRKY	TGTGGTACTGTGACGTCA	179	184	0	0	0	0

PRL	CAACTGGTTGGTACACCA	259	308	0	1	0	0
PRL	TGACTGGTTGGTACACAC	20	30	0	0	0	0
PRL	ACACTGGTTGGTACACTG	250	260	1	0	0	0
PRL	CAACTGGTTGGTACTGGT	168	76	0	0	0	0
PRL	ACACTGGTTGGTACTGCA	171	150	0	0	0	0
PRLR	ACACTGCAGTCACAGTCA	1244	1257	14	1	0	12
PRLR	GTACTGCAGTCACAGTAC	79	88	0	0	0	0
PRLR	TGACTGCAGTCACAGTTG	762	690	1	0	0	0
PRLR	ACACTGCAGTCACACAGT	256	227	2	1	0	4
PRLR	CATGCAGTTGACACTGGT	1100	691	0	0	0	14
PRMT1	ACACGTACACTGACGTGT	99	110	0	0	1	0
PRMT1	ACACCAACGTACTGACGT	163	440	0	0	0	0
PRMT1	TGACCAACGTACTGACCA	103	168	1	0	0	1
PRMT1	CAACCAACGTACTGACAC	38	30	0	0	0	0
PRMT1	GTACCAACGTACTGACTG	489	198	0	0	0	0
PRMT2	CAACCAACGTACACGTGT	332	498	11	5	1	4
PRMT2	ACACCAACGTACACGTCA	265	422	0	0	1	0
PRMT2	GTACCAACGTACACGTAC	35	37	0	0	0	0
PRMT2	TGACCAACGTACACGTTG	284	250	2	0	0	0
PRMT2	ACACCAACGTACACCAGT	106	145	0	0	0	0
PRMT3	CAACCACACACAACTGAC	76	81	0	0	0	0
PRMT3	GTACCACACACAACTGTG	120	97	0	0	0	0
PRMT3	ACACCACACACATGGTGT	286	248	0	0	0	0
PRMT3	TGACCACACACATGGTCA	295	266	0	0	0	1
PRMT3	CAACCACACACATGGTAC	12	19	0	0	0	0
PRMT5	TGTGGTGTCAACGTCATG	73	105	0	0	0	0
PRMT5	ACTGGTGTCAACGTACGT	155	133	0	0	0	0
PRMT5	TGTGGTGTCAACGTACCA	561	709	0	0	0	0
PRMT5	TGTGGTACTGCAACCAAC	26	10	0	0	0	0
PRMT5	TGTGCAGTCACAACCAAC	83	53	0	0	0	0
PRMT6	ACTGGTGTCAGTTGGTTG	946	1282	6	0	3	5
PRMT6	TGACCACACAACTGCATG	71	109	0	0	0	0
PRMT6	ACACCACACAACTGACGT	681	529	1	0	0	0
PRMT6	TGACCACACAACTGACCA	397	154	0	0	0	0
PRMT6	GTTGCAGTCAGTCAGTAC	237	281	1	0	0	0
PRMT6	CATGCACATGGTCACACA	14	42	0	0	0	0
PRMT7	ACACCACAACTGGTACTG	117	137	0	0	1	0
PRMT7	CAACCACAACTGGTTGGT	93	164	0	0	0	0
PRMT7	ACACCACAACTGGTTGCA	115	85	0	0	0	6
PRMT7	GTACCACAACTGGTTGAC	102	81	0	0	0	0
PRMT7	TGACCACAACTGGTTGTG	194	453	0	0	0	1
PRMT7	ACTGCACATGGTTGGTCA	842	1004	1	1	0	2
PRMT8	TGACCACACATGCATGCA	149	267	1	0	1	11
PRMT8	GTACCACACATGCAACAC	55	60	1	0	0	0
PRMT8	TGACCACACATGCAACTG	338	503	0	1	0	0
PRMT8	ACACCACACATGCATGGT	693	1036	1	2	0	0
PRMT8	TGTGGTGTCAGTTGGTAC	18	113	0	0	0	0
PRNP	ACACTGTGTGTGCACACA	157	126	0	0	2	0
PRNP	ACACTGTGTGTGCAGTTG	698	757	0	0	0	0
PRNP	CAACTGTGTGTGCACAGT	141	98	0	0	0	0
PRNP	GTACTGTGTGTGCACAAC	23	21	0	0	0	0
PRNP	TGACTGTGTGTGCACATG	287	247	0	0	0	0
PROC	GTACACCATGTGGTGTGT	706	543	2	0	0	1
PROC	CAACACCATGTGGTGTCA	162	147	0	0	0	0
PROC	TGACACCATGTGGTGTAC	33	208	0	0	0	0
PROC	ACACACCATGTGGTGTTG	136	163	1	0	0	0
PROC	CATGCACACAGTACGTGT	489	311	0	0	0	0
PROC	ACTGCACACAGTACGTCA	36	41	0	0	0	0
PROCR	TGACTGCAACTGACGTCA	112	177	0	0	0	0
PROCR	CAACTGCAACTGACGTAC	24	32	0	1	0	0
PROCR	GTACTGCAACTGACGTTG	163	325	1	0	0	0
PROCR	TGACTGCAACTGACCAGT	42	97	0	0	0	0
PROCR	ACTGCATGACCACAACAC	104	136	0	0	0	0
PRODH	ACACCAGTCATGACGTCA	198	63	0	0	0	0
PRODH	GTACCAGTCATGACGTAC	31	16	0	0	0	0
PRODH	TGACCAGTCATGACGTTG	215	149	0	0	0	0
PRODH	ACACCAGTCATGACCAGT	64	48	0	0	0	0
PRODH	TGACCAGTCATGACCACA	42	75	0	0	0	0
PRODH	ACTGCAACCACATGGTTG	784	642	0	0	0	1
PROM1	GTACTGCATGACTGCAGT	454	146	1	0	0	0
PROM1	CAACTGCATGACTGCACA	200	148	0	0	0	1
PROM1	TGACTGCATGACTGCAAC	45	31	0	0	0	0
PROM1	ACACTGCATGACTGCATG	272	156	1	0	0	0
PROM1	GTTGCACAACGTCATGCA	215	175	0	1	0	0
PROM1	ACTGCACAACGTCATGAC	125	47	0	0	0	0

PROM2	TGTGGTTGACGTGTTGGT	222	169	0	0	0	1
PROM2	GTTGGTTGACGTGTTGCA	93	125	0	0	0	0
PROM2	ACTGGTTGACGTGTTGAC	5	12	0	0	0	0
PROM2	CATGGTTGACGTGTTGTG	478	475	2	0	0	0
PROM2	TGTGGTTGACGTCAGTGT	51	309	0	0	0	0
PROM2	CATGCAACACGTACACAC	16	25	0	0	0	0
PROS1	CAACACTGCAACCATGAC	16	20	0	0	0	4
PROS1	GTACACTGCAACCATGTG	489	681	1	1	0	0
PROS1	ACACACTGCAACACGTGT	321	301	0	0	0	0
PROS1	TGACACTGCAACACGTCA	147	360	1	0	0	0
PROS1	CAACACTGCAACACGTAC	69	153	0	0	0	0
PROS1	GTTGCACACAGTACGTAC	15	13	0	0	0	1
PROX1	ACACGTACCATGCAGTCA	294	217	1	0	0	0
PROX1	GTACGTACCATGCAGTAC	20	19	0	0	0	0
PROX1	TGACGTACCATGCAGTTG	196	478	1	1	0	0
PROX1	ACACGTACCATGCACAGT	171	155	0	0	0	2
PROX1	TGACGTACCATGCACACA	86	107	0	0	0	0
PROZ	TGACACTGCACACACACA	288	134	0	0	0	0
PROZ	CAACACTGCACACACAAC	34	19	1	0	0	0
PROZ	GTACACTGCACACACATG	124	99	0	0	0	0
PROZ	TGACACTGCACACAACGT	1125	1043	3	31	0	0
PROZ	GTACACTGCACACAACCA	370	541	0	1	0	0
PROZ	TGTGCACACAGTACGTTG	447	436	3	0	0	7
PRPF19	TGACGTGTGTTGCAACAC	23	30	0	0	0	0
PRPF19	ACACGTGTGTTGCAACTG	227	350	1	0	0	0
PRPF19	CAACGTGTGTTGCATGGT	315	693	0	0	0	0
PRPF19	ACACGTGTGTTGCATGCA	98	27	3	0	0	0
PRPF19	GTACGTGTGTTGCATGAC	27	112	0	0	0	1
PRPF40A	GTTGCAGTGTGTCAACTG	213	253	0	0	0	0
PRPF40A	TGTGCAGTGTGTCATGGT	138	171	0	0	0	0
PRPF40A	GTTGCAGTGTGTCATGCA	305	247	0	0	0	0
PRPF40A	ACTGCAGTGTGTCATGAC	12	19	0	0	0	0
PRPF40A	CATGCAGTGTGTCATGTG	107	68	2	0	0	0
PRPF40B	CAACTGTGCAGTACTGTG	299	330	2	0	1	0
PRPF40B	TGACTGTGCAGTTGGTGT	671	630	1	1	1	13
PRPF40B	GTACTGTGCAGTACTGCA	86	117	0	0	0	0
PRPF40B	ACACTGTGCAGTACTGAC	200	116	0	0	0	0
PRPF40B	GTACTGTGCAGTTGGTCA	261	193	0	0	0	0
PRPS1	GTTGCAACCACAACACCA	524	722	0	0	1	2
PRPS1	ACTGCAGTCATGACACTG	295	310	2	1	0	0
PRPS1	CATGCAGTCATGACTGGT	305	285	0	0	0	1
PRPS1	ACTGCAGTCATGACTGCA	38	31	0	0	0	0
PRPS1	GTTGCAGTCATGACTGAC	49	41	0	0	0	0
PRPS1	CATGCACAGTCAGTACTG	404	348	0	1	0	0
PRPS1L1	ACACACGTACACCAACTG	170	206	0	0	0	0
PRPS1L1	CAACACGTACACCATGGT	56	103	0	0	0	0
PRPS1L1	ACACACGTACACCATGCA	428	472	0	0	0	7
PRPS1L1	GTACACGTACACCATGAC	60	68	0	0	0	0
PRPS1L1	TGACACGTACACCATGTG	622	526	0	0	0	1
PRPS1L1	CATGCATGGTTGACTGTG	706	403	1	0	0	0
PRPS2	CAACGTTGTGACTGCAGT	508	75	0	1	0	2
PRPS2	TGTGCAGTGTACTGCAAC	11	11	0	0	0	0
PRPS2	GTTGCACAGTCAGTTGGT	283	287	1	1	0	0
PRPS2	CATGCACAGTCAGTTGCA	382	362	1	1	0	1
PRPS2	TGTGCACAGTCAGTTGAC	20	31	0	0	0	0
PRTN3	ACACACGTTGTGTGACGT	325	169	1	0	1	7
PRTN3	TGACACGTTGTGTGACCA	163	17	0	0	0	0
PRTN3	CAACACGTTGTGTGACAC	210	123	0	0	0	0
PRTN3	GTACACGTTGTGTGACTG	29	29	0	0	0	0
PRTN3	CATGACCACACAACGTTG	1396	361	0	0	0	0
PSAP	GTTGGTGTGTCAACGTCA	387	519	0	0	0	0
PSAP	ACTGGTGTGTCAACGTAC	90	53	0	0	0	0
PSAP	CATGGTGTGTCAACGTTG	621	740	3	0	0	23
PSAP	GTTGGTGTGTCAACCAGT	98	75	0	0	0	0
PSAP	CATGGTGTGTCAACCACA	98	86	0	0	0	1
PSAT1	GTACCACATGGTCAGTTG	594	518	1	0	1	0
PSAT1	ACACCACACAACCAACTG	1095	1325	2	0	0	12
PSAT1	TGACCACATGGTCAGTCA	260	244	0	0	0	0
PSAT1	CAACCACATGGTCAGTAC	214	168	1	1	0	0
PSAT1	TGACCACATGGTCACAGT	96	115	0	0	0	0
PSCA	CATGGTTGTGTGGTGTGT	148	146	0	1	0	0
PSCA	ACTGGTTGTGTGGTGTCA	207	231	1	0	0	0
PSCA	GTTGGTTGTGTGGTGTAC	6	6	0	0	0	0
PSCA	TGTGGTTGTGTGGTGTTG	963	684	1	1	0	0
PSCA	ACTGGTTGTGTGGTCAGT	122	178	1	0	0	9

PSCD1	TGACTGCATGACACTGGT	511	529	0	1	0	0
PSCD1	GTACTGCATGACACTGCA	149	193	0	0	0	0
PSCD1	ACACTGCATGACACTGAC	56	58	0	0	0	0
PSCD1	CAACTGCATGACACTGTG	253	225	0	0	0	2
PSCD1	TGACTGCATGACTGGTGT	179	208	0	0	0	0
PSCD1	ACTGCACATGGTCAGTAC	67	71	0	0	0	0
PSCD2	CAACTGCATGACACCAGT	485	214	0	0	0	0
PSCD2	ACACTGCATGACACCACA	81	65	0	0	0	0
PSCD2	GTACTGCATGACACCAAC	52	44	0	0	0	0
PSCD2	TGACTGCATGACACCATG	269	114	0	0	0	0
PSCD2	TGTGCAGTGTGTCACATG	91	72	0	15	0	1
PSCD4	TGTGGTTGTGTGCACATG	210	217	1	0	1	0
PSCD4	CATGGTTGTGTGCACAGT	105	194	0	0	0	0
PSCD4	ACTGGTTGTGTGCACACA	103	51	1	0	0	0
PSCD4	GTTGGTTGTGTGCACAAC	22	160	0	0	0	0
PSCD4	ACTGGTTGTGTGCAACGT	163	225	0	1	0	0
PSCD4	CATGCACATGGTCAGTTG	317	315	0	2	0	15
PSEN1	TGACCAGTTGTGGTGTAC	203	112	0	0	0	0
PSEN1	ACACCAGTTGTGGTGTTG	99	209	0	0	0	0
PSEN1	CAACTGCATGGTTGACCA	167	166	0	0	0	0
PSEN1	TGACTGCATGGTTGACAC	17	21	0	0	0	0
PSEN1	ACACTGCATGGTTGACTG	454	360	3	0	0	1
PSEN1	TGTGCACAACACTGACTG	110	104	0	0	0	0
PSEN2	CAACCAGTTGTGGTCAGT	224	429	0	0	0	0
PSEN2	ACACTGTGGTCACATGGT	43	66	0	0	0	0
PSEN2	TGACTGTGGTCACATGCA	366	358	0	0	0	0
PSEN2	CAACTGTGGTCACATGAC	33	30	0	0	0	0
PSEN2	GTACTGTGGTCACATGTG	301	354	0	0	0	12
PSEN2	ACTGCACAACACACTGAC	42	62	2	0	0	0
PSENEN	GTTGGTACGTACGTACCA	66	79	0	0	0	0
PSENEN	ACTGGTACGTACGTACAC	34	30	0	0	0	0
PSENEN	CATGGTACGTACGTACTG	232	340	0	0	0	1
PSENEN	GTTGGTACGTACGTTGGT	135	186	1	0	0	0
PSENEN	CATGGTACGTACGTTGCA	159	114	0	1	0	0
PSENEN	TGTGCATGGTGTTGACGT	504	320	0	0	0	1
PSMA1	GTCATGTGGTCAGTTGAC	1	2	0	0	0	0
PSMA1	TGCATGTGGTCAGTTGTG	39	319	0	0	0	0
PSMA1	CACATGTGGTCACAGTGT	103	54	0	0	0	0
PSMA1	ACCATGTGGTCACAGTCA	489	399	0	0	0	0
PSMA1	GTCATGTGGTCACAGTAC	15	18	0	0	0	0
PSMA1	TGTGCAACACTGTGCACA	2	0	0	0	0	0
PSMA2	TGCATGTGGTCACAGTTG	172	163	0	0	0	0
PSMA2	ACCATGTGGTCACACAGT	59	38	0	0	0	0
PSMA2	TGCATGTGGTCACACACA	86	87	1	0	0	0
PSMA2	CACATGTGGTCACACAAC	4	3	0	0	0	0
PSMA2	GTTGCAGTGTTGCAGTTG	220	280	1	0	0	0
PSMA3	GTCATGTGGTCACACATG	27	29	0	1	0	0
PSMA3	TGCATGTGGTCACAACGT	106	7	0	0	0	0
PSMA3	GTCATGTGGTCACAACCA	74	84	0	1	0	0
PSMA3	CATGGTGTGTTGACCATG	47	57	0	0	0	0
PSMA3	CATGCAGTCAACACTGCA	11	7	0	0	0	0
PSMA3	CATGCAACACTGTGCAAC	5	11	0	0	0	0
PSMA4	CACATGTGGTCACAACTG	696	782	20	0	4	1
PSMA4	ACCATGTGGTCACAACAC	5	7	0	0	0	0
PSMA4	GTCATGTGGTCACATGGT	214	647	0	1	0	0
PSMA4	CACATGTGGTCACATGCA	17	29	0	0	0	0
PSMA4	TGCATGTGGTCACATGAC	24	21	0	0	0	0
PSMA4	GTTGCAACACTGTGCATG	25	10	0	0	0	2
PSMA5	ACCATGTGGTCACATGTG	67	58	0	0	0	0
PSMA5	GTCATGTGGTCAACGTGT	651	654	0	1	0	0
PSMA5	CACATGTGGTCAACGTCA	58	49	37	0	0	0
PSMA5	TGCATGTGGTCAACGTAC	23	31	0	0	0	0
PSMA5	ACCATGTGGTCAACGTTG	120	175	0	1	0	0
PSMA5	TGTGCAACACTGTGACGT	112	97	0	0	0	0
PSMA6	CAACGTTGTGCATGCATG	61	72	0	0	0	0
PSMA6	GTACGTTGTGCATGACGT	5	16	0	0	0	1
PSMA6	CAACGTTGTGCATGACCA	47	45	0	0	0	0
PSMA6	TGACGTTGTGCATGACAC	22	43	1	0	0	0
PSMA6	TGACCAGTTGTGCATGCA	80	64	0	0	0	0
PSMA6	GTTGCAACACTGTGACCA	2	1	0	0	0	0
PSMA7	CACATGTGGTCAACCAGT	353	292	1	0	0	0
PSMA7	ACCATGTGGTCAACCACA	34	51	0	0	0	0
PSMA7	GTCATGTGGTCAACCAAC	22	13	0	0	0	0
PSMA7	TGCATGTGGTCAACCATG	282	336	0	0	0	0
PSMA7	CAACCAGTTGTGCATGAC	24	34	0	0	0	0

PSMA7	ACTGCAACACTGTGACAC	16	6	0	0	0	0
PSMB1	TGCATGTGGTCAACTGGT	465	212	9	4	8	1
PSMB1	ACCATGTGGTCAACACGT	8	11	0	0	0	0
PSMB1	TGCATGTGGTCAACACCA	54	71	0	0	0	0
PSMB1	CACATGTGGTCAACACAC	58	39	0	0	0	0
PSMB1	GTCATGTGGTCAACACTG	209	101	0	0	0	0
PSMB1	CATGCAACACTGGTCAGT	95	125	0	0	0	0
PSMB10	ACACCAGTTGTGCAACCA	637	576	2	0	1	3
PSMB10	GTCATGTGGTACGTTGCA	220	268	0	0	0	1
PSMB10	ACCATGTGGTACGTTGAC	162	69	1	1	0	0
PSMB10	CACATGTGGTACGTTGTG	242	434	0	0	0	0
PSMB10	TGTGCAGTGTTGACACGT	46	54	0	0	0	0
PSMB10	GTTGCAACACTGTGGTAC	104	88	0	0	0	0
PSMB2	GTCATGTGGTCAACTGCA	345	218	7	1	6	11
PSMB2	ACCATGTGGTCAACTGAC	2	38	0	0	0	0
PSMB2	CACATGTGGTCAACTGTG	68	139	0	0	0	0
PSMB2	TGCATGTGGTCATGGTGT	82	72	1	0	0	0
PSMB2	GTCATGTGGTCATGGTCA	11	54	0	0	0	0
PSMB2	ACTGCAACACTGGTCACA	22	19	0	0	0	0
PSMB3	ACCATGTGGTCATGGTAC	48	36	0	0	0	0
PSMB3	CACATGTGGTCATGGTTG	268	668	2	0	0	0
PSMB3	GTCATGTGGTCATGCAGT	110	160	0	0	0	9
PSMB3	CACATGTGGTCATGCACA	41	60	0	0	0	0
PSMB3	ACTGCAGTCAGTCAGTCA	71	102	0	1	0	1
PSMB3	GTTGCAACACTGGTCAAC	5	9	0	0	0	0
PSMB4	TGCATGTGGTCATGCAAC	95	29	0	0	0	0
PSMB4	ACCATGTGGTCATGCATG	102	143	0	0	0	0
PSMB4	CACATGTGGTCATGACGT	39	36	0	0	0	0
PSMB4	ACCATGTGGTCATGACCA	97	115	1	0	0	0
PSMB4	ACACCAGTTGTGCATGGT	207	187	0	15	0	0
PSMB4	TGTGCAACACTGGTCATG	60	58	0	0	0	0
PSMB5	GTCATGTGGTCATGACAC	0	0	0	0	0	0
PSMB5	TGCATGTGGTCATGACTG	531	316	0	0	0	0
PSMB5	ACCATGTGGTCATGTGGT	226	435	0	0	0	0
PSMB5	TGCATGTGGTCATGTGCA	94	152	0	0	0	0
PSMB5	CACATGTGGTCATGTGAC	59	56	0	0	0	0
PSMB5	ACTGCAACACTGGTACGT	246	334	1	0	0	0
PSMB6	TGTGCAACACTGTGGTTG	243	329	2	0	2	0
PSMB6	GTCATGTGGTCATGTGTG	678	560	0	0	0	2
PSMB6	GTCATGTGGTACGTGTGT	501	1028	63	1	0	2
PSMB6	CACATGTGGTACGTGTCA	394	385	1	0	0	2
PSMB6	TGCATGTGGTACGTGTAC	9	31	0	0	0	0
PSMB6	ACCATGTGGTACGTGTTG	70	53	0	0	0	0
PSMB7	CACATGTGGTACGTCAGT	400	135	1	1	0	0
PSMB7	ACCATGTGGTACGTCACA	31	27	1	0	0	0
PSMB7	GTCATGTGGTACGTCAAC	1	2	0	0	0	0
PSMB7	TGCATGTGGTACGTCATG	111	85	0	1	0	0
PSMB7	ACACCAGTTGTGCACATG	29	10	0	0	0	0
PSMB7	ACTGCAACACTGTGCAGT	14	12	0	0	0	0
PSMB8	ACACGTGTCATGACACTG	708	623	1	0	0	0
PSMB8	CAACGTGTCATGACTGGT	168	101	0	0	0	0
PSMB8	ACACGTGTCATGACTGCA	311	580	0	0	0	0
PSMB8	GTACGTGTCATGACTGAC	62	31	0	0	0	0
PSMB8	TGTGACCACAACACTGAC	36	138	0	0	0	0
PSMB9	ACCATGTGGTACGTACGT	313	100	0	1	0	0
PSMB9	TGCATGTGGTACGTACCA	180	319	1	0	0	0
PSMB9	CACATGTGGTACGTACAC	60	56	0	0	0	6
PSMB9	GTCATGTGGTACGTACTG	376	596	0	1	0	0
PSMB9	TGCATGTGGTACGTTGGT	65	60	1	0	0	0
PSMC1	TGACACACGTGTACACTG	183	249	0	0	0	0
PSMC1	ACACACACGTGTACTGGT	213	158	19	0	0	0
PSMC1	TGACACACGTGTACTGCA	45	62	0	0	0	0
PSMC1	CAACACACGTGTACTGAC	25	79	0	0	0	0
PSMC1	GTACACACGTGTACTGTG	308	252	183	49	0	0
PSMC1	GTTGCAACTGCACAGTCA	13	27	0	0	0	0
PSMC2	ACTGCAACTGCACAGTAC	143	30	0	1	1	0
PSMC2	CAACGTGTCAACTGCAGT	188	132	2	0	0	0
PSMC2	ACACGTGTCAACTGCACA	389	385	1	0	0	1
PSMC2	GTACGTGTCAACTGCAAC	39	42	0	0	0	0
PSMC2	TGACGTGTCAACTGCATG	205	714	1	1	0	0
PSMC2	ACACGTGTCAACTGACGT	59	140	0	0	0	0
PSMC3	ACACGTTGGTTGTGACCA	408	353	0	0	0	0
PSMC3	GTACGTTGGTTGTGACAC	31	16	0	0	0	0
PSMC3	TGACGTTGGTTGTGACTG	599	353	0	1	0	0
PSMC3	ACACGTTGGTTGTGTGGT	76	281	0	0	0	0

PSMC3	TGACGTTGGTTGTGTGCA	389	237	0	0	0	1
PSMC6	TGACACACGTCACATGCA	392	310	0	0	1	0
PSMC6	ACACACACGTCACAACCA	597	350	2	0	0	0
PSMC6	GTACACACGTCACAACAC	27	34	0	0	0	0
PSMC6	TGACACACGTCACAACTG	870	1112	2	1	0	0
PSMC6	ACACACACGTCACATGGT	496	134	1	1	0	3
PSMC6	CATGCAACTGCACAGTTG	5	17	0	0	0	0
PSMD1	CAACTGGTCAACACGTAC	87	50	1	1792	331	0
PSMD1	ACACTGGTCAACACGTGT	37	18	0	0	0	0
PSMD1	TGACTGGTCAACACGTCA	122	175	1	0	0	0
PSMD1	GTACTGGTCAACACGTTG	9	21	0	0	0	0
PSMD1	TGACTGGTCAACACCAGT	11	21	0	0	0	0
PSMD1	TGTGCAACTGACACACCA	348	329	0	0	0	0
PSMD11	ACCATGTGGTACCACATG	175	121	0	0	1	1
PSMD11	CACATGTGGTACCAGTTG	30	28	0	0	0	0
PSMD11	GTCATGTGGTACCACAGT	183	141	0	1	0	0
PSMD11	CACATGTGGTACCACACA	20	15	0	0	0	0
PSMD11	TGCATGTGGTACCACAAC	5	5	0	0	0	0
PSMD11	ACTGCATGGTCAACACAC	4	19	0	0	0	0
PSMD12	GTACTGGTCACATGTGTG	256	213	1	0	0	0
PSMD12	GTACTGGTCAACGTGTGT	693	1035	0	1	0	0
PSMD12	CAACTGGTCAACGTGTCA	411	392	1	0	0	0
PSMD12	TGACTGGTCAACGTGTAC	20	31	0	0	0	0
PSMD12	ACACTGGTCAACGTGTTG	56	61	0	0	0	0
PSMD13	GTACTGGTCAACACCACA	163	414	0	0	0	3
PSMD13	ACACTGGTCAACACCAAC	14	25	0	0	0	0
PSMD13	CAACTGGTCAACACCATG	35	18	0	0	0	0
PSMD13	GTACTGGTCAACACACGT	46	65	0	0	0	0
PSMD13	CAACTGGTCAACACACCA	262	303	0	0	0	0
PSMD13	CATGCATGGTCAACACTG	287	296	0	1	0	0
PSMD14	CAACGTGTGTACCAGTGT	437	552	1	0	0	0
PSMD14	ACACGTGTGTACCAGTCA	110	168	0	1	0	0
PSMD14	GTACGTGTGTACCAGTAC	88	52	0	0	0	0
PSMD14	CAACCAGTTGTGACTGGT	241	228	0	0	0	0
PSMD14	GTACCAGTTGTGACTGAC	24	22	0	0	0	0
PSMD2	GTACTGGTCAACCAGTCA	490	347	0	1	0	1
PSMD2	ACACTGGTCAACCAGTAC	20	26	0	0	0	0
PSMD2	CAACTGGTCAACCAGTTG	166	183	1	0	0	0
PSMD2	GTACTGGTCAACCACAGT	82	290	0	1	0	0
PSMD2	CAACTGGTCAACCACACA	68	127	0	0	0	0
PSMD2	CATGCAACTGACACACAC	58	60	0	0	0	0
PSMD3	CAACTGGTCAACACTGGT	491	285	0	0	1	0
PSMD3	TGACTGGTCAACACACAC	220	41	0	0	0	0
PSMD3	ACACTGGTCAACACACTG	149	184	0	0	0	0
PSMD3	ACACTGGTCAACACTGCA	165	626	0	0	0	2
PSMD3	GTACTGGTCAACACTGAC	4	9	0	0	0	0
PSMD3	GTTGCAACTGACACACTG	34	13	0	0	0	0
PSMD4	TGCATGTGGTACCAGTGT	96	161	0	0	0	0
PSMD4	GTCATGTGGTACCAGTCA	441	431	0	1	0	16
PSMD4	ACCATGTGGTACCAGTAC	10	30	0	0	0	0
PSMD4	ACTGCAGTGTTGCAACTG	123	118	0	0	0	0
PSMD4	CATGCAGTGTTGCATGGT	153	213	0	0	0	0
PSMD6	ACTGCACAACTGCAGTGT	42	56	0	19	3	0
PSMD6	ACTGGTCATGCATGCAGT	843	736	1	153	1	2
PSMD6	GTTGGTCATGCATGCATG	576	243	0	0	1	1
PSMD6	TGTGGTCATGCATGGTTG	1230	337	3	0	0	0
PSMD6	TGTGGTCATGCATGCACA	140	191	0	0	0	3
PSMD6	CATGGTCATGCATGCAAC	32	42	0	0	0	0
PSMD7	CAACTGGTCAACGTCAGT	231	141	0	0	1	0
PSMD7	ACACTGGTCAACGTCACA	275	85	0	0	0	0
PSMD7	GTACTGGTCAACGTCAAC	26	35	0	0	0	0
PSMD7	TGACTGGTCAACGTCATG	102	68	0	0	0	0
PSMD7	CATGCAGTCACATGCATG	60	103	0	0	0	0
PSMD8	TGACTGGTCAACCATGCA	322	313	0	1	1	0
PSMD8	TGACTGGTCAACCAACTG	766	913	1	1	0	0
PSMD8	ACACTGGTCAACCATGGT	135	117	3	0	0	0
PSMD8	CAACTGGTCAACCATGAC	165	236	0	0	0	2
PSMD8	GTACTGGTCAACCATGTG	117	72	0	1	0	0
PSME1	TGACTGGTCAACGTTGGT	735	895	3	1	0	6
PSME1	GTACTGGTCAACGTTGCA	115	141	0	0	0	0
PSME1	ACACTGGTCAACGTTGAC	13	2	0	0	0	0
PSME1	CAACTGGTCAACGTTGTG	360	411	1	1	0	0
PSME1	TGACTGGTCAACCAGTGT	375	525	1	1	0	24
PSME1	GTTGCATGGTACTGTGAC	65	45	0	0	0	0
PSME2	TGACTGGTCAACCACAAC	100	117	1	0	0	0

PSME2	ACACTGGTCAACCACATG	337	101	1	0	0	0
PSME2	CAACTGGTCAACCAACGT	680	337	0	0	0	0
PSME2	ACACTGGTCAACCAACCA	196	265	0	0	0	0
PSME2	GTACTGGTCAACCAACAC	16	23	0	0	0	3
PSME2	ACTGCATGGTACTGCAAC	16	15	0	1	0	0
PSME3	ACACTGGTCAACGTACGT	52	64	0	0	0	3
PSME3	TGACTGGTCAACGTACCA	364	691	0	1	0	1
PSME3	CAACTGGTCAACGTACAC	12	11	0	0	0	0
PSME3	GTACTGGTCAACGTACTG	139	189	0	0	0	0
PSME3	CAACTGACTGCAACACGT	127	340	0	0	0	0
PSME3	CATGCATGGTACTGCATG	63	99	0	0	0	0
PSMF1	GTACCACACACAGTACCA	226	203	0	0	0	0
PSMF1	ACACCACACACAGTACAC	8	29	0	0	0	0
PSMF1	CAACCACACACAGTACTG	807	226	2	0	0	1
PSMF1	CAACCACACACACAGTCA	144	104	1	0	0	0
PSMF1	TGACCACACACACAGTAC	15	20	0	0	0	0
PSMF1	TGTGCATGGTACTGTGTG	260	494	1	0	0	1
PTAFR	GTACGTCATGTGGTACGT	306	317	1	1	0	0
PTAFR	CAACGTCATGTGGTACCA	283	272	1	0	0	0
PTAFR	TGACGTCATGTGGTACAC	14	9	0	0	0	0
PTAFR	ACACGTCATGTGGTACTG	260	254	1	0	0	0
PTAFR	CAACGTCATGTGGTTGGT	1145	679	0	1	0	0
PTCH1	ACACCATGCATGGTTGCA	139	135	0	1	1	12
PTCH1	CAACCATGCATGCAGTGT	1367	756	2	40	1	15
PTCH1	GTACCATGCATGGTTGAC	121	151	0	0	0	0
PTCH1	TGACCATGCATGGTTGTG	148	267	1	0	0	0
PTCH1	ACACCATGCATGCAGTCA	150	136	0	0	0	0
PTCH2	TGACCACAGTGTACTGCA	141	355	1	0	0	0
PTCH2	CAACCACAGTGTACTGAC	16	14	0	0	0	0
PTCH2	GTACCACAGTGTACTGTG	174	146	0	1	0	0
PTCH2	ACACCACAGTGTTGGTGT	174	204	1	0	0	0
PTCH2	TGTGGTGTGTTGACTGTG	285	433	0	0	0	1
PTCRA	TGACTGACGTACGTTGAC	26	47	0	1	0	0
PTCRA	ACACTGACGTACGTTGTG	163	106	0	0	0	0
PTCRA	GTACTGACGTACCAGTGT	299	279	2	0	0	1
PTCRA	ACTGCAGTTGTGCACACA	167	213	0	1	0	2
PTCRA	GTTGCAGTTGTGCACAAC	34	19	0	0	0	0
PTEN	TGCATGACCATGCAACCA	191	415	0	1	0	1
PTEN	CACATGACCATGCAACAC	41	55	0	1	0	0
PTEN	GTCATGACCATGCAACTG	791	916	1	2	0	1
PTEN	TGCATGACCATGCATGGT	190	402	0	0	0	7
PTEN	GTCATGACCATGCATGCA	93	80	1	0	0	0
PTGDR	ACACGTCATGTGGTTGCA	191	78	0	0	0	0
PTGDR	GTACGTCATGTGGTTGAC	44	88	0	0	0	0
PTGDR	TGACGTCATGTGGTTGTG	126	168	0	0	0	0
PTGDR	CAACGTCATGTGCAGTGT	610	655	0	0	0	0
PTGDR	ACACGTCATGTGCAGTCA	45	59	0	0	0	0
PTGDS	TGACACGTCAACGTCACA	88	97	0	0	1	0
PTGDS	CAACACGTCAACGTCAAC	87	47	0	0	0	0
PTGDS	GTACACGTCAACGTCATG	249	441	0	0	0	0
PTGDS	TGACACGTCAACGTACGT	52	110	0	0	0	0
PTGDS	TGTGCATGTGGTACGTAC	49	36	0	0	0	0
PTGER1	GTACGTCATGTGCAGTAC	93	58	0	0	0	0
PTGER1	TGACGTCATGTGCAGTTG	571	230	0	0	0	0
PTGER1	ACACGTCATGTGCACAGT	68	151	0	0	0	0
PTGER1	TGACGTCATGTGCACACA	51	316	0	0	0	0
PTGER1	CAACGTCATGTGCACAAC	12	73	0	0	0	0
PTGER2	GTACGTCATGTGCACATG	440	252	1	1	0	0
PTGER2	TGACGTCATGTGCAACGT	153	90	0	0	0	0
PTGER2	GTACGTCATGTGCAACCA	111	161	1	0	0	0
PTGER2	ACACGTCATGTGCAACAC	25	58	0	0	0	0
PTGER2	CAACGTCATGTGCAACTG	170	122	1	0	0	0
PTGER2	CATGCATGCACAGTGTCA	112	108	0	0	0	0
PTGER3	GTACGTCATGTGCATGGT	63	50	0	0	0	0
PTGER3	CAACGTCATGTGCATGCA	166	239	1	0	0	1
PTGER3	TGACGTCATGTGCATGAC	30	45	0	0	0	34
PTGER3	ACACGTCATGTGCATGTG	150	152	0	0	0	0
PTGER3	GTACGTCATGTGACGTGT	469	586	0	0	0	1
PTGER4	CACATGCACACAGTACTG	662	588	1	2	1	63
PTGER4	TGCATGCACACAGTACGT	153	135	1	0	0	0
PTGER4	GTCATGCACACAGTACCA	405	374	1	1	0	1
PTGER4	ACCATGCACACAGTACAC	47	92	0	0	0	0
PTGER4	GTCATGCACACAGTTGGT	420	457	0	0	0	0
PTGES	ACACACGTACACTGACGT	64	74	11	0	0	0
PTGES	TGACACGTACACTGACCA	400	378	1	0	0	1

PTGES	CAACACGTACACTGACAC	20	58	1	0	0	0
PTGES	GTACACGTACACTGACTG	257	282	1	0	0	0
PTGES	TGACACGTACACTGTGGT	169	165	0	1	0	0
PTGES2	TGACACGTACGTCAGTTG	1026	1260	0	1	0	4
PTGES2	ACACACGTACGTCACAGT	41	58	0	0	0	0
PTGES2	TGACACGTACGTCACACA	112	65	0	0	0	0
PTGES2	CAACACGTACGTCACAAC	8	9	0	0	0	0
PTGES2	CATGCAGTCAGTCACAAC	87	44	0	0	0	0
PTGFR	ACACGTCATGTGACGTTG	230	214	0	0	1	0
PTGFR	CAACGTCATGTGACGTCA	61	50	0	0	0	6
PTGFR	TGACGTCATGTGACGTAC	4	6	0	0	0	0
PTGFR	CAACGTCATGTGACCAGT	261	321	0	0	0	0
PTGFR	ACACGTCATGTGACCACA	143	97	0	0	0	0
PTGIR	TGACGTCATGTGACTGGT	489	896	1	0	1	0
PTGIR	CAACGTCATGTGACACAC	61	54	0	0	0	0
PTGIR	GTACGTCATGTGACACTG	1031	551	0	1	0	0
PTGIR	GTACGTCATGTGACTGCA	125	143	0	0	0	0
PTGIR	ACACGTCATGTGACTGAC	31	34	0	0	0	0
PTGIS	TGACACGTGTTGTGCAGT	51	36	0	0	0	0
PTGIS	GTACACGTGTTGTGCACA	56	68	0	0	0	0
PTGIS	ACACACGTGTTGTGCAAC	25	25	0	0	0	0
PTGIS	CAACACGTGTTGTGCATG	74	109	0	1	0	0
PTGIS	TGTGACGTTGTGACCAGT	316	116	0	0	0	0
PTGS1	TGACACGTCAACCAGTAC	41	37	0	0	0	0
PTGS1	ACACACGTCAACCAGTTG	940	1275	0	0	0	0
PTGS1	CAACACGTCAACCACAGT	194	160	1	0	0	0
PTGS1	ACACACGTCAACCACACA	541	195	0	0	0	8
PTGS1	GTACACGTCAACCACAAC	13	19	0	0	0	0
PTGS1	ACTGCAACGTTGACTGGT	521	421	1	1	0	0
PTGS2	TGACACGTCAACCATGGT	191	279	0	0	0	0
PTGS2	GTACACGTCAACCATGCA	81	105	0	0	0	0
PTGS2	ACACACGTCAACCATGAC	90	127	0	0	0	4
PTGS2	CAACACGTCAACCATGTG	307	327	3	0	0	58
PTGS2	TGACACGTCAACACGTGT	625	599	3	1	0	0
PTGS2	ACTGCACACAGTACCAGT	195	168	0	0	0	0
PTH	CAACTGTGCAACGTGTTG	415	531	2	0	0	0
PTH	GTACTGTGCAACGTCAGT	130	174	0	0	0	0
PTH	CAACTGTGCAACGTCACA	302	23	0	0	0	0
PTH	TGACTGTGCAACGTCAAC	22	69	0	0	0	0
PTH	ACACTGTGCAACGTCATG	235	186	1	0	0	0
PTH2R	CAACGTCATGTGACTGTG	252	290	0	0	0	0
PTH2R	TGACGTCATGTGTGGTGT	1639	1118	2	1	0	6
PTH2R	GTACGTCATGTGTGGTCA	298	311	1	0	0	0
PTH2R	ACACGTCATGTGTGGTAC	38	52	0	0	0	0
PTH2R	CAACGTCATGTGTGGTTG	308	366	1	0	0	0
PTHR1	GTCATGACACTGGTGTCA	539	551	0	3	3	3
PTHR1	CACATGACACACTGTGAC	72	95	0	0	0	0
PTHR1	GTCATGACACACTGTGTG	223	221	0	0	0	0
PTHR1	TGCATGACACTGGTGTGT	320	286	0	0	0	0
PTHR1	CATGCAGTACGTACCATG	103	198	0	0	0	3
PTK2	GTTGCATGGTGTACGTTG	554	680	0	1	1	1
PTK2	TGCATGACGTCAGTGTTG	933	236	1	0	0	0
PTK2	ACCATGACGTCAGTCAGT	105	89	0	0	0	0
PTK2	TGCATGACGTCAGTCACA	82	104	0	64	0	0
PTK2	CACATGACGTCAGTCAAC	18	29	0	0	0	0
PTK2	TGACGTTGTGACGTTGCA	68	176	1	0	0	0
PTK2B	ACCATGCAACTGCAACAC	43	258	2	0	2	1
PTK2B	CACATGCAACTGCAACTG	342	347	0	0	0	1
PTK2B	GTCATGCAACTGCATGGT	203	131	0	0	0	0
PTK2B	CATGGTACTGACACTGCA	298	322	0	0	0	0
PTK2B	TGTGGTACTGACACTGAC	190	31	0	0	0	0
PTK2B	CATGCATGGTGTACGTAC	16	24	0	1	0	0
PTK6	GTACGTTGACCACATGGT	562	685	3	3	4	1
PTK6	CAACGTTGACCACAACTG	699	569	6	0	1	0
PTK6	ACACGTTGACCACATGTG	1979	966	1	0	1	1
PTK6	CAACGTTGACCACATGCA	118	197	0	1	0	0
PTK6	TGACGTTGACCACATGAC	37	36	0	0	0	0
PTK6	TGTGCATGGTGTACCAGT	148	227	0	0	0	0
PTPN1	ACCATGACCATGACTGGT	68	66	0	0	0	0
PTPN1	TGCATGACCATGACTGCA	131	194	0	0	0	0
PTPN1	CACATGACCATGACTGAC	26	47	0	0	0	0
PTPN1	GTCATGACCATGACTGTG	138	251	0	1	0	0
PTPN1	GTTGCAGTACGTACTGAC	25	25	0	0	0	0
PTPN1	TGTGCAACGTTGACTGCA	110	282	9	1	0	12
PTPN11	ACCATGTGCATGTGTGGT	179	483	0	0	1	1

PTPN11	CACATGTGCATGTGACGT	150	216	1	0	0	0
PTPN11	ACCATGTGCATGTGACCA	129	151	0	1	0	0
PTPN11	GTCATGTGCATGTGACAC	21	12	0	0	0	0
PTPN11	TGCATGTGCATGTGACTG	222	215	0	0	0	0
PTPN12	GTCATGACACGTGTACCA	328	347	1	2	0	0
PTPN12	ACCATGACACGTGTACAC	12	11	0	0	0	0
PTPN12	CACATGACACGTGTACTG	85	185	0	0	0	0
PTPN12	GTCATGACACGTGTTGGT	449	772	0	1	0	0
PTPN12	CACATGACACGTGTTGCA	202	235	0	0	0	0
PTPN12	TGTGCAACCAGTGTGTAC	34	60	0	0	0	0
PTPN2	TGCATGACCATGTGGTCA	640	364	1	0	1	1
PTPN2	ACCATGACCATGTGGTGT	513	630	1	3	0	1
PTPN2	CACATGACCATGTGGTAC	48	69	0	0	0	0
PTPN2	GTCATGACCATGTGGTTG	766	334	2	0	0	1
PTPN2	TGCATGACCATGTGCAGT	198	349	1	0	0	1
PTPN2	CATGCAACGTTGACTGAC	55	22	0	0	0	8
PTPN22	ACCATGACACCAGTACGT	239	265	0	0	0	0
PTPN22	TGCATGACACCAGTACCA	253	328	0	1	0	0
PTPN22	CACATGACACCAGTACAC	136	199	0	0	0	0
PTPN22	GTCATGACACCAGTACTG	889	736	0	1	0	0
PTPN22	TGCATGACACCAGTTGGT	243	130	0	0	0	0
PTPN22	CATGCAACGTCATGTGAC	46	26	0	0	0	0
PTPN6	TGACGTGTCACAACGTGT	898	820	2	1	0	2
PTPN6	GTACGTGTCACAACGTCA	293	393	1	1	0	0
PTPN6	ACACGTGTCACAACGTAC	40	40	1	0	0	0
PTPN6	CAACGTGTCACAACGTTG	194	179	0	0	0	0
PTPN6	CATGCAGTGTGTCAACAC	65	88	0	0	0	0
PTPN7	CACATGACACGTGTGTGT	337	259	0	0	1	1
PTPN7	TGCATGACCATGTGTGTG	504	424	0	0	0	0
PTPN7	ACCATGACACGTGTGTCA	311	261	2	2	0	0
PTPN7	GTCATGACACGTGTGTAC	30	16	0	0	0	0
PTPN7	TGCATGACACGTGTGTTG	365	128	0	0	0	0
PTPN7	ACTGCAACCAGTGTGTTG	46	19	0	0	0	0
PTPN9	ACCATGACACGTGTCAGT	100	82	1	0	0	0
PTPN9	TGCATGACACGTGTCACA	13	20	0	0	0	0
PTPN9	CACATGACACGTGTCAAC	124	45	0	0	0	0
PTPN9	GTCATGACACGTGTCATG	5	22	0	0	0	0
PTPN9	TGCATGACACGTGTACGT	303	143	0	0	0	0
PTPRA	TGCATGACACGTGTTGAC	32	49	2	2	0	4
PTPRA	ACCATGACACGTGTTGTG	108	101	1	0	0	0
PTPRA	GTCATGACACGTCAGTGT	354	463	2	0	0	27
PTPRA	CACATGACACGTCAGTCA	433	315	0	0	0	0
PTPRA	TGCATGACACGTCAGTAC	81	116	0	0	0	0
PTPRA	TGTGCAACCAGTGTACCA	233	234	0	0	0	0
PTPRB	ACCATGACACGTCAGTTG	784	1031	2	2	0	16
PTPRB	CACATGACACGTCACAGT	129	154	0	0	0	0
PTPRB	ACCATGACACGTCACACA	305	224	0	0	0	0
PTPRB	GTCATGACACGTCACAAC	33	57	0	0	0	2
PTPRB	TGCATGACACGTCACATG	321	142	0	0	0	0
PTPRB	ACTGCAACCAGTGTTGAC	87	48	0	0	0	0
PTPRC	ACCATGACACGTCAACGT	52	57	1	0	0	0
PTPRC	TGCATGACACGTCAACCA	352	466	0	1	0	0
PTPRC	CACATGACACGTCAACAC	49	100	0	0	0	0
PTPRC	GTCATGACACGTCAACTG	393	206	0	0	0	0
PTPRC	TGCATGACACGTCATGGT	73	88	0	0	0	0
PTPRF	TGCATGACACGTACGTGT	694	315	0	0	1	1
PTPRF	GTCATGACACGTCATGCA	136	386	0	0	0	128
PTPRF	ACCATGACACGTCATGAC	43	31	0	0	0	0
PTPRF	CACATGACACGTCATGTG	26	23	0	0	0	0
PTPRF	TGTGCAGTCAACACTGAC	147	111	1	0	0	0
PTPRF	CATGCAACCAGTCATGAC	47	31	0	0	0	0
PTPRH	GTCATGACACGTACGTCA	27	33	0	0	0	0
PTPRH	ACCATGACACGTACGTAC	24	29	0	0	0	0
PTPRH	CACATGACACGTACGTTG	88	77	0	0	0	0
PTPRH	GTTGACCAGTTGACGTAC	119	65	0	0	0	0
PTPRH	TGTGACCAGTTGACGTTG	705	747	2	0	0	0
PTPRJ	GTTGCAACCAGTCATGTG	510	721	2	0	1	0
PTPRJ	GTCATGACACGTACCAGT	341	408	2	0	0	149
PTPRJ	CACATGACACGTACCACA	66	53	1	0	0	0
PTPRJ	TGCATGACACGTACCAAC	11	16	0	0	0	0
PTPRJ	ACCATGACACGTACCATG	123	182	0	0	0	0
PTPRJ	ACTGCAACCAGTACGTGT	237	232	0	0	0	0
PTPRN	CACATGACACGTACACGT	44	42	0	0	0	0
PTPRN	ACCATGACACGTACACCA	248	304	0	0	0	1
PTPRN	GTCATGACACGTACACAC	39	49	0	0	0	0

PTPRN	TGCATGACACGTACACTG	177	145	0	0	0	0
PTPRN	ACCATGACACGTACTGGT	131	168	0	0	0	0
PTPRN	TGTGCAACCAGTACGTCA	351	269	0	0	0	0
PTPRO	TGCATGACACGTACTGCA	261	399	1	0	0	0
PTPRO	CACATGACACGTACTGAC	31	24	0	0	0	0
PTPRO	TGTGCACATGTGTGTGCA	356	131	1	1	0	0
PTPRO	CATGCACATGTGTGTGAC	27	34	0	0	0	0
PTPRO	GTTGCACATGTGTGTGTG	206	246	0	0	0	0
PTPRO	TGTGCAACGTGTGTGTGT	187	83	0	0	0	0
PTPRU	TGCATGACGTACCACAGT	156	155	0	0	0	0
PTPRU	GTCATGACGTACCACACA	106	87	0	0	0	0
PTPRU	ACCATGACGTACCACAAC	7	6	0	0	0	0
PTPRU	GTCATGACGTACTGGTGT	563	1088	0	0	0	1
PTPRU	CACATGACGTACTGGTCA	183	193	0	0	0	10
PTTG1	GTACGTACCAGTCACAGT	342	699	0	0	1	0
PTTG1	TGTGCAGTACACCATGGT	148	133	0	0	1	0
PTTG1	ACACGTACCAGTCAGTAC	80	48	0	0	0	1
PTTG1	CAACGTACCAGTCAGTTG	1181	1078	1	1	0	1
PTTG1	CAACGTACCAGTCACACA	103	80	1	0	0	0
PTTG1	GTTGCACATGCATGTGCA	137	153	0	0	0	1
PTX3	TGTGCATGCAGTCATGGT	985	979	1	1	1	6
PTX3	TGTGGTCATGTGGTGTGT	754	910	2	1	0	7
PTX3	GTTGGTCATGTGGTGTCA	218	224	0	1	0	0
PTX3	ACTGGTCATGTGGTGTAC	41	409	0	0	0	0
PTX3	CATGGTCATGTGGTGTTG	1350	1078	4	2	0	0
PTX3	GTTGGTCATGTGGTCAGT	382	370	0	17	0	0
PUM1	ACTGGTCATGTGTGGTTG	1220	732	2	0	1	1
PUM1	CATGGTGTCACACAGTGT	231	547	1	0	0	0
PUM1	CATGGTCATGTGTGCAGT	51	64	0	0	0	0
PUM1	ACTGGTCATGTGTGCACA	325	125	1	0	0	0
PUM1	GTTGGTCATGTGTGCAAC	32	103	0	0	0	0
PUM2	GTACTGCATGCAACACGT	76	70	0	0	0	0
PUM2	CAACTGCATGCAACACCA	186	315	0	0	0	0
PUM2	TGACTGCATGCAACACAC	58	75	0	0	0	0
PUM2	ACACTGCATGCAACACTG	447	603	1	1	0	0
PUM2	CAACTGCATGCAACTGGT	234	374	0	0	0	0
PUM2	CATGCACATGTGTGCACA	253	60	0	0	0	1
PURA	ACACGTACGTCACAGTCA	356	349	0	1	0	0
PURA	GTACGTACGTCACAGTAC	68	39	0	0	0	0
PURA	TGACGTACGTCACAGTTG	597	1075	1	0	0	1
PURA	ACACGTACGTCACACAGT	78	95	11	1	0	0
PURA	TGACGTACGTCACACACA	128	103	0	1	0	0
PXN	TGTGGTCACACACATGTG	1420	580	1	1	0	0
PXN	CATGGTCACACAACGTGT	1074	1332	2	1	0	0
PXN	ACTGGTCACACAACGTCA	94	100	0	0	0	0
PXN	GTTGGTCACACAACGTAC	65	31	0	0	0	6
PXN	TGTGGTCACACAACGTTG	379	981	1	0	0	0
PXN	TGTGCACACAGTACCACA	66	145	0	0	0	0
PYCARD	GTACTGCAGTGTTGACTG	126	162	2	0	1	20
PYCARD	TGACTGCAGTGTTGTGGT	619	703	0	0	0	0
PYCARD	GTACTGCAGTGTTGTGCA	141	149	1	0	0	0
PYCARD	ACACTGCAGTGTTGTGAC	56	33	0	0	0	0
PYCARD	CAACTGCAGTGTTGTGTG	499	498	1	221	0	0
PYCARD	ACTGCAACACACCACAAC	15	16	0	0	0	0
PYCR1	ACACCATGGTACACACTG	296	753	0	0	0	0
PYCR1	CAACCATGGTACACTGGT	126	159	0	0	0	0
PYCR1	ACACCATGGTACACTGCA	210	191	0	0	0	1
PYCR1	GTACCATGGTACACTGAC	32	27	0	0	0	0
PYCR1	TGTGCAACTGACCACACA	112	116	0	0	0	0
PYCR1	CATGCAACTGACCACAAC	32	20	0	0	0	0
PYGB	GTTGGTACCAACGTACGT	268	95	1	0	0	0
PYGB	CATGGTACCAACGTACCA	130	169	0	0	0	0
PYGB	TGTGGTACCAACGTACAC	21	24	0	0	0	0
PYGB	ACTGGTACCAACGTACTG	72	78	0	0	0	1
PYGB	CATGCAGTCAACTGCAGT	116	278	1	0	0	0
PYGL	ACTGGTCAGTTGACACCA	124	127	0	1	1	0
PYGL	GTTGGTCAGTTGACACAC	102	62	0	0	0	0
PYGL	TGTGGTCAGTTGACACTG	809	827	2	0	0	6
PYGL	ACTGGTCAGTTGACTGGT	163	244	0	0	0	1
PYGL	TGTGGTCAGTTGACTGCA	45	79	0	0	0	0
PYGM	GTTGGTCAGTACTGTGGT	52	142	0	0	0	6
PYGM	CATGGTCAGTACTGTGCA	105	94	0	0	0	0
PYGM	TGTGGTCAGTACTGTGAC	47	50	1	0	0	0
PYGM	GTTGCACAGTGTACTGCA	81	43	0	0	0	0
PYGM	ACTGCACAACGTCAGTTG	197	168	0	0	0	0

PYGM	CATGCACAACGTCACAGT	89	449	0	0	0	0
PYGO1	CATGGTCAACGTACCAGT	128	62	0	0	0	0
PYGO1	ACTGGTCAACGTACCACA	56	149	0	0	0	0
PYGO1	GTTGGTCAACGTACCAAC	24	13	0	0	0	0
PYGO1	TGTGGTCAACGTACCATG	310	442	0	9	0	12
PYGO1	ACTGGTCAACGTACACGT	89	68	0	0	0	0
PYGO1	TGTGCATGGTTGGTTGTG	933	297	0	0	0	0
QARS	GTTGGTTGTGGTACTGGT	829	676	1	0	1	1
QARS	TGACACGTCATGGTACAC	69	76	0	0	0	0
QARS	ACACACGTCATGGTACTG	84	380	0	0	0	0
QARS	CAACACGTCATGGTTGGT	1429	387	1	2	0	0
QARS	ACACACGTCATGGTTGCA	231	197	0	0	0	5
QARS	TGTGCACACATGACTGCA	324	60	1	0	0	0
QPCT	ACACCAGTTGTGACGTGT	137	318	0	0	1	0
QPCT	GTACCACACAACGTGTCA	843	826	0	2	1	0
QPCT	TGACCAGTTGTGACGTCA	318	260	0	0	0	0
QPCT	TGACCACACAACGTGTGT	1201	1305	5	1	0	1
QPCT	ACACCACACAACGTGTAC	152	299	0	0	0	1
QPRT	TGACCACAACGTCACAAC	69	183	4	0	2	2
QPRT	CAACCACAACGTCAACGT	367	397	1	1	1	0
QPRT	GTACCACAACGTCACAGT	382	298	0	0	0	1
QPRT	CAACCACAACGTCACACA	152	230	1	0	0	0
QPRT	ACACCACAACGTCACATG	128	186	1	0	0	0
RAB10	CAACCAGTACCACAGTGT	582	379	0	0	1	0
RAB10	ACACCAGTACCACAGTCA	460	459	1	0	0	0
RAB10	GTACCAGTACCACAGTAC	41	57	0	0	0	0
RAB10	TGACCAGTACCACAGTTG	995	1511	1	2	0	1
RAB10	ACACCAGTACCACACAGT	545	347	1	0	0	0
RAB11A	CAACTGACTGTGTGCAAC	17	16	0	0	0	0
RAB11A	GTACTGACTGTGTGCATG	134	119	0	0	0	0
RAB11A	TGACTGACTGTGTGACGT	734	436	0	0	0	2
RAB11A	GTACTGACTGTGTGACCA	278	464	1	0	0	1
RAB11A	ACACTGACTGTGTGACAC	31	39	0	0	0	0
RAB1A	TGTGGTGTCACAGTACAC	34	28	1	0	0	0
RAB1A	CATGGTACGTTGGTACCA	164	175	0	0	0	0
RAB1A	TGTGGTACGTTGGTACAC	29	34	0	0	0	0
RAB1A	ACTGGTACGTTGGTACTG	356	543	0	0	0	2
RAB1A	CATGGTACGTTGGTTGGT	134	181	1	0	0	17
RAB1B	TGACACCAGTACACGTGT	727	731	1	0	1	0
RAB1B	ACACACCAGTACCATGAC	42	67	0	0	0	0
RAB1B	CAACACCAGTACCATGTG	728	387	0	0	0	0
RAB1B	GTACACCAGTACACGTCA	229	134	0	0	0	0
RAB1B	TGTGCAGTCAACACCACA	319	190	1	0	0	0
RAB23	CAACACCACACATGTGCA	713	519	1	2	1	0
RAB23	GTACACCACACATGACCA	180	168	0	0	0	1
RAB23	ACACACCACACATGACAC	23	64	0	0	0	0
RAB23	CAACACCACACATGACTG	381	233	0	0	0	0
RAB23	GTACACCACACATGTGGT	280	247	0	0	0	0
RAB24	ACACACCACAGTTGGTGT	342	459	0	0	0	0
RAB24	TGACACCACAGTTGGTCA	799	736	1	37	0	8
RAB24	CAACACCACAGTTGGTAC	147	56	0	0	0	0
RAB24	GTTGCAGTGTCATGGTAC	60	64	0	0	0	0
RAB24	TGTGCAGTGTCATGGTTG	1344	1563	1	1	0	2
RAB27A	GTCATGTGACCACAACTG	487	741	3	198	1070	4
RAB27A	TGCATGTGACCACACATG	78	67	0	0	0	0
RAB27A	ACCATGTGACCACAACGT	212	179	0	1	0	0
RAB27A	TGCATGTGACCACAACCA	287	170	1	0	0	0
RAB27A	CACATGTGACCACAACAC	17	27	0	0	0	0
RAB35	ACACACCACAGTCATGAC	54	61	0	1	0	0
RAB35	CAACACCACAGTCATGTG	2108	579	1	0	0	0
RAB35	TGACACCACAGTACGTGT	113	95	0	0	0	0
RAB35	GTACACCACAGTACGTCA	101	341	0	0	0	0
RAB35	ACACACCACAGTACGTAC	282	185	0	0	0	0
RAB3A	TGACACCACACAGTACCA	736	660	13703	9	4	2
RAB3A	ACTGCAGTTGGTCAACTG	1264	585	1	2	1	0
RAB3A	GTACACCACACAGTCAAC	21	47	0	0	0	0
RAB3A	TGACACCACACAGTCATG	222	248	5	0	0	0
RAB3A	ACACACCACACAGTACGT	389	323	1	0	0	0
RAB3B	TGCATGCACACACAGTAC	53	89	0	0	0	1
RAB3B	ACCATGCACACACAGTTG	511	599	1	0	0	0
RAB3B	CACATGCACACACACAGT	30	82	0	1	0	0
RAB3B	ACCATGCACACACACACA	94	136	1	0	0	0
RAB3B	GTCATGCACACACACAAC	2	4	0	0	0	0
RAB3B	CATGCAACCACAACACTG	252	670	1	1	0	0
RAB4A	GTACGTGTTGGTGTACAC	103	63	0	0	0	0

RAB4A	TGACGTGTTGGTGTACTG	159	174	0	0	0	0
RAB4A	ACACGTGTTGGTGTTGGT	152	209	0	0	0	0
RAB4A	TGACGTGTTGGTGTTGCA	98	156	0	0	0	0
RAB4A	ACTGGTGTGTTGTGCAGT	188	337	0	0	0	1
RAB4A	CATGCAACCACACACATG	169	283	0	0	0	0
RAB5A	TGACGTGTACTGTGTGCA	37	28	0	0	0	0
RAB5A	CAACGTGTACTGTGTGAC	257	48	0	0	0	0
RAB5A	GTACGTGTACTGTGTGTG	106	154	0	1	0	0
RAB5A	TGACGTGTTGGTGTGTGT	1623	359	5	1	0	0
RAB5A	ACTGCAGTCATGTGTGTG	346	351	1	0	0	0
RAB5A	GTTGCATGCAGTCATGCA	63	133	0	0	0	0
RAB5C	CAACTGACTGTGACACCA	746	476	0	0	0	0
RAB5C	TGACTGACTGTGACACAC	43	94	0	0	0	0
RAB5C	ACACTGACTGTGACACTG	157	148	1	0	0	0
RAB5C	CAACTGACTGTGACTGGT	255	325	0	0	0	0
RAB5C	ACACTGACTGTGACTGCA	148	231	0	0	0	0
RAB6A	GTACACCACACAGTACTG	1140	992	3	0	1	0
RAB6A	GTACACCACACAGTTGCA	231	443	5	1	1	463
RAB6A	CAACACCACACAGTACAC	143	28	0	0	0	0
RAB6A	TGACACCACACAGTTGGT	223	239	0	0	0	0
RAB6A	ACACACCACACAGTTGAC	207	127	0	0	0	0
RAB7A	CAACGTGTTGGTGTTGAC	47	138	1	0	0	0
RAB7A	GTACGTGTTGGTGTTGTG	339	415	0	0	0	0
RAB7A	ACACGTGTTGGTCAGTGT	905	573	1	0	0	0
RAB7A	TGACGTGTTGGTCAGTCA	558	525	7	1	0	6
RAB7A	CAACGTGTTGGTCAGTAC	111	122	0	0	0	0
RAB9A	CAACACCACACAACACCA	956	688	0	0	1	0
RAB9A	ACACACCACACAACCAAC	31	22	0	0	0	0
RAB9A	CAACACCACACAACCATG	748	401	0	0	0	0
RAB9A	GTACACCACACAACACGT	185	196	0	0	0	0
RAB9A	TGACACCACACAACACAC	166	68	0	0	0	0
RABGAP1L	GTTGCAGTCACAACTGTG	233	368	4	0	0	1
RABGAP1L	GTTGACCAACGTACCACA	34	38	0	0	0	0
RABGAP1L	ACTGACCAACGTACCAAC	11	15	0	0	0	0
RABGAP1L	CATGACCAACGTACCATG	454	499	1	0	0	0
RABGAP1L	GTTGACCAACGTACACGT	34	70	0	0	0	0
RAC1	ACCATGTGCAACTGGTGT	1394	1112	2	1	1	0
RAC1	ACCATGTGCAACACTGGT	405	682	1	1	0	0
RAC1	TGCATGTGCAACACTGCA	88	112	0	0	0	0
RAC1	CACATGTGCAACACTGAC	85	35	0	0	0	0
RAC1	GTCATGTGCAACACTGTG	100	196	1	0	0	0
RAC2	TGTGCAGTCACAGTGTTG	110	119	0	0	0	0
RAC2	ACTGCAGTCACAGTCAGT	89	148	1	0	0	0
RAC2	TGTGCAGTCACAGTCACA	257	228	0	0	0	0
RAC2	CATGCAGTCACAGTCAAC	39	63	0	0	0	0
RAC2	GTTGCAGTTGGTACACCA	147	174	0	0	0	0
RAC2	ACTGCATGGTGTCATGGT	1192	742	2	1	0	1
RAC3	ACACACCAGTACACGTAC	52	78	0	0	0	0
RAC3	CAACACCAGTACACGTTG	1333	521	0	1	0	3
RAC3	GTACACCAGTACACCAGT	103	167	0	0	0	0
RAC3	CAACACCAGTACACCACA	204	159	0	0	0	0
RAC3	CATGCAGTCAACGTACGT	503	319	3	1	0	1
RAD1	GTACGTTGACCAGTACGT	129	187	0	0	0	0
RAD1	CAACTGACGTCAGTCACA	284	218	0	0	0	1
RAD1	TGACTGACGTCAGTCAAC	4	3	0	0	0	0
RAD1	ACACTGACGTCAGTCATG	204	120	0	0	0	0
RAD1	CAACTGACGTCAGTACGT	173	265	1	0	0	6
RAD17	ACACGTACACTGACCAAC	18	24	0	0	0	0
RAD17	CAACGTACACTGACCATG	440	65	3	1	0	0
RAD17	GTACGTACACTGACACGT	39	97	0	0	0	0
RAD17	CAACGTACACTGACACCA	266	115	0	2	0	0
RAD17	TGACGTACACTGACACAC	49	44	0	0	0	0
RAD17	CATGCACACAGTACCAAC	36	29	0	0	0	0
RAD18	CACATGACTGGTACTGGT	1098	624	2	0	2	3
RAD18	TGCATGACTGGTACACAC	36	40	0	0	0	444
RAD18	ACCATGACTGGTACACTG	351	513	1	0	0	0
RAD18	ACCATGACTGGTACTGCA	51	55	0	0	0	0
RAD18	ACTGCAGTACCAGTGTAC	102	65	0	0	0	3
RAD18	GTTGCAACGTGTTGCAAC	1	0	0	0	0	0
RAD21	CATGGTACGTGTGTTGTG	152	805	0	0	1	1
RAD21	GTTGGTACGTGTGTTGCA	45	126	0	0	0	1
RAD21	ACTGGTACGTGTGTTGAC	47	19	0	0	0	0
RAD21	TGTGGTACGTGTCAGTGT	308	346	0	0	0	0
RAD21	TGTGCAGTCAACGTACTG	339	451	0	1	0	0
RAD21	CATGCACAACCAGTGTCA	459	440	0	1	0	0

RAD23A	GTCATGTGTGCAACTGAC	21	9	0	0	0	0
RAD23A	TGCATGTGTGCAACTGTG	209	231	0	0	0	0
RAD23A	CACATGTGTGCATGGTGT	875	601	1	0	0	1
RAD23A	ACCATGTGTGCATGGTCA	56	65	0	0	0	0
RAD23A	GTCATGTGTGCATGGTAC	35	65	0	0	0	0
RAD23A	CATGCAACCACATGACAC	46	58	0	0	0	0
RAD23B	CACATGTGGTACCAACGT	166	232	1	0	0	1
RAD23B	ACCATGTGGTACCAACCA	221	87	0	0	0	0
RAD23B	GTCATGTGGTACCAACAC	52	113	0	0	0	0
RAD23B	TGCATGTGGTACCAACTG	744	504	0	2	0	1
RAD23B	ACCATGTGGTACCATGGT	117	65	0	0	0	0
RAD50	CAACCATGCATGTGCACA	8	30	0	0	0	0
RAD50	TGACCATGCATGTGCAAC	24	14	0	0	0	0
RAD50	ACACCATGCATGTGCATG	88	187	0	1	0	0
RAD50	CAACCATGCATGTGACGT	82	102	0	0	0	0
RAD50	ACACCATGCATGTGACCA	210	136	0	0	0	0
RAD50	TGTGCAACCACATGCATG	125	133	1	0	0	0
RAD51	ACACGTACACTGTGACAC	29	39	0	0	0	0
RAD51	CAACGTACACTGTGACTG	73	76	0	0	0	0
RAD51	GTACGTACACTGTGTGGT	300	430	0	0	0	0
RAD51	CAACGTACACTGTGTGCA	38	41	0	0	0	0
RAD51	GTTGCAGTACACTGCACA	317	185	129	1	0	0
RAD51	ACTGCAACCACATGACGT	120	128	0	1	0	0
RAD51C	GTTGGTCATGACTGCAGT	241	596	0	0	2	1
RAD51C	ACACTGACTGACGTACCA	147	175	29	0	0	0
RAD51C	GTTGGTCATGACTGGTCA	105	138	0	0	0	0
RAD51C	ACTGGTCATGACTGGTAC	14	15	0	0	0	0
RAD51C	CATGGTCATGACTGGTTG	296	259	0	0	0	0
RAD51C	TGTGCAACGTTGGTTGAC	24	46	0	0	0	0
RAD51L1	TGTGGTACCACAACCAGT	434	130	1	0	1	0
RAD51L1	GTTGGTACCACAACCACA	178	163	1	0	1	0
RAD51L1	ACTGGTACCACAACCAAC	17	65	0	0	0	0
RAD51L1	CATGGTACCACAACCATG	64	109	0	0	0	0
RAD51L1	GTTGGTACCACAACACGT	32	23	0	0	0	0
RAD51L3	CATGGTACACCATGACGT	156	235	0	0	0	0
RAD51L3	ACTGGTACACCATGACCA	563	713	0	3	0	1
RAD51L3	GTTGGTACACCATGACAC	126	59	2	0	0	1
RAD51L3	TGTGGTACACCATGACTG	394	449	1	0	0	0
RAD51L3	ACTGGTACACCATGTGGT	536	353	1	2	0	1
RAD51L3	GTTGCAACCACATGCAAC	41	28	0	0	0	0
RAD52	TGACGTACACTGTGCACA	60	45	0	0	1	0
RAD52	CAACGTACACTGTGCAAC	31	11	0	0	0	0
RAD52	GTACGTACACTGTGCATG	324	359	1	1	0	0
RAD52	TGACGTACACTGTGACGT	209	379	0	1	0	0
RAD52	GTACGTACACTGTGACCA	78	228	0	0	0	0
RAD52	GTTGCAACCAACCACAAC	39	55	0	0	0	0
RAD54B	ACACACACCAACCACAAC	12	12	1	0	0	0
RAD54B	CAACACACCAACCACATG	116	118	14	1	0	7
RAD54B	GTACACACCAACCAACGT	164	241	1	1	0	0
RAD54B	CAACACACCAACCAACCA	121	126	0	0	0	0
RAD54B	TGACACACCAACCAACAC	225	52	0	0	0	0
RAD54B	ACTGCAACCAACCACACA	385	360	0	1	0	0
RAD54L	TGCATGCACACACATGGT	140	147	0	0	0	0
RAD54L	GTCATGCACACACATGCA	208	194	0	0	0	0
RAD54L	ACCATGCACACACATGAC	94	43	0	0	0	0
RAD54L	CACATGCACACACATGTG	173	209	0	0	0	0
RAD54L	TGCATGCACACAACGTGT	437	719	0	0	0	1
RAD54L	TGTGCAACCACATGACCA	121	100	1	0	0	0
RAD9A	CAACGTACTGGTGTGTGT	323	212	0	0	1	0
RAD9A	TGACGTCAACCATGGTTG	147	219	0	1	0	0
RAD9A	TGACGTACACTGTGTGAC	119	102	1	221	0	2
RAD9A	ACACGTACACTGTGTGTG	267	276	0	0	0	0
RAD9A	CATGCAACCACATGCAGT	237	358	0	0	0	0
RAD9A	ACTGCAACCACATGCACA	356	106	0	0	0	0
RAF1	ACCATGCATGACCATGGT	156	223	0	0	0	47
RAF1	TGCATGCATGACCATGCA	352	222	2	0	0	7
RAF1	CACATGCATGACCATGAC	86	66	0	0	0	10
RAF1	GTCATGCATGACCATGTG	361	406	1	1	0	4
RAF1	ACCATGCATGACACGTGT	15	27	0	0	0	0
RAG1	GTCATGACTGGTCACAGT	111	203	0	0	0	0
RAG1	CACATGACTGGTCACACA	117	81	0	0	0	0
RAG1	TGCATGACTGGTCACAAC	19	23	0	0	0	1
RAG1	ACCATGACTGGTCACATG	418	837	1	1	0	4
RAG1	CATGCAGTACGTTGCAAC	17	118	0	0	0	0
RAG1	ACTGCACACAACACCAAC	24	23	0	0	0	0

RAG2	GTACACACCACATGGTTG	2074	2081	6	2	1	195
RAG2	GTACACACCACATGCACA	79	115	0	0	1	0
RAG2	CAACACACCACATGGTAC	75	59	0	0	0	0
RAG2	TGACACACCACATGCAGT	189	174	0	0	0	0
RAG2	ACACACACCACATGCAAC	45	33	0	0	0	0
RALA	ACCATGTGCAACACACCA	499	486	0	0	1	1
RALA	ACCATGTGCAACACCATG	19	105	0	0	0	0
RALA	CACATGTGCAACACACGT	21	7	0	0	0	0
RALA	GTCATGTGCAACACACAC	49	131	0	0	0	0
RALA	TGCATGTGCAACACACTG	417	267	0	2	0	0
RALB	GTACTGACTGTGACTGAC	88	107	0	0	0	15
RALB	TGACTGACTGTGACTGTG	270	173	0	0	0	0
RALB	CAACTGACTGTGTGGTGT	617	375	2	100	0	0
RALB	ACACTGACTGTGTGGTCA	102	91	0	1	0	0
RALB	ACTGCAGTCAACACGTCA	388	129	0	0	0	0
RALBP1	GTACACCACAGTTGACGT	61	511	0	0	1	1
RALBP1	CAACACCACAGTTGACCA	310	278	1	0	0	2
RALBP1	TGACACCACAGTTGACAC	75	67	0	0	0	0
RALBP1	ACACACCACAGTTGACTG	330	400	2	0	0	2
RALBP1	CAACACCACAGTTGTGGT	160	218	1	0	0	0
RALGDS	ACACACCACACACATGGT	936	958	0	0	1	0
RALGDS	ACACACCACACACAACCA	267	259	0	0	0	0
RALGDS	GTACACCACACACAACAC	59	65	0	0	0	0
RALGDS	TGACACCACACACAACTG	354	391	0	0	0	0
RALGDS	ACTGCAGTGTTGGTCATG	170	272	0	1	0	0
RAMP1	GTACGTCATGTGTGACAC	240	24	0	1	0	1
RAMP1	TGACGTCATGTGTGACTG	304	527	0	0	0	0
RAMP1	ACACGTCATGTGTGTGGT	192	122	0	0	0	0
RAMP1	TGACGTCATGTGTGTGCA	180	175	0	0	0	0
RAMP1	ACTGCAGTCAGTGTTGCA	28	24	1	0	0	0
RAN	CAACACCACACAGTGTCA	490	261	3	2	1	7
RAN	ACACACCACAGTTGTGCA	33	17	0	0	0	0
RAN	GTACACCACAGTTGTGAC	16	7	0	0	0	0
RAN	TGACACCACAGTTGTGTG	20	68	0	0	0	0
RAN	GTACACCACACAGTGTGT	22	231	0	0	0	0
RAN	CATGCACAACCATGTGCA	4	5	0	0	0	0
RANBP1	GTACACCACAACACGTGT	979	1127	1	0	1	0
RANBP1	CAACACCACAACCATGCA	541	517	1	0	0	0
RANBP1	TGACACCACAACCATGAC	58	27	0	0	0	0
RANBP1	ACACACCACAACCATGTG	816	601	2	0	0	2
RANBP1	CATGGTTGGTTGCAGTAC	76	52	1	0	0	0
RANBP1	GTTGCACAACACGTGTTG	449	483	1	0	0	0
RANBP2	CACATGACTGGTCATGAC	41	33	0	0	1	0
RANBP2	TGCATGACTGGTCATGCA	75	109	0	0	0	0
RANBP2	GTCATGACTGGTCATGTG	152	229	2	0	0	0
RANBP2	ACCATGACTGGTACGTGT	44	60	0	0	0	0
RANBP2	TGCATGACTGGTACGTCA	46	102	0	0	0	0
RANBP9	ACACGTGTACCACAACTG	448	332	0	0	0	0
RANBP9	CAACGTGTACCACATGGT	626	528	0	0	0	0
RANBP9	ACACGTGTACCACATGCA	306	392	0	0	0	0
RANBP9	GTACGTGTACCACATGAC	31	22	0	0	0	0
RANBP9	TGTGGTGTCACAGTTGTG	186	174	0	0	0	0
RANGAP1	TGACACCAGTACTGACAC	20	48	0	0	0	0
RANGAP1	ACACACCAGTACTGACTG	457	418	0	0	0	0
RANGAP1	CAACACCAGTACTGTGGT	627	601	2	1	0	1
RANGAP1	ACACACCAGTACTGTGCA	609	307	1	0	0	1
RANGAP1	TGTGGTGTCAGTCAGTCA	128	303	0	0	0	0
RAP1B	ACACCAGTACCAGTACTG	333	363	4	0	0	4
RAP1B	CAACCAGTACCAGTTGGT	408	478	0	0	0	1
RAP1B	ACACCAGTACCAGTTGCA	254	235	0	0	0	0
RAP1B	GTACCAGTACCAGTTGAC	38	49	0	0	0	0
RAP1B	TGACCAGTACCAGTTGTG	540	669	3	0	0	0
RAP1GAP	ACACTGTGGTGTCAGTTG	1017	470	1	0	0	0
RAP1GAP	CAACTGTGGTGTCACAGT	405	265	1	0	0	2
RAP1GAP	ACACTGTGGTGTCACACA	127	69	0	0	0	0
RAP1GAP	GTTGCACACAGTACCATG	85	152	0	0	0	0
RAP1GAP	TGTGCACACAGTACACGT	97	242	0	0	0	0
RAP1GAP	GTTGCACACAGTACACCA	269	116	1	0	0	3
RAP2B	CAACACCACAGTCAGTCA	608	622	0	0	0	0
RAP2B	TGACACCACAGTCAGTAC	163	276	0	1	0	0
RAP2B	ACACACCACAGTCAGTTG	569	628	0	0	0	24
RAP2B	ACTGCAGTTGGTACACAC	41	48	0	0	0	0
RAP2B	GTTGCATGACTGACACAC	142	63	0	0	0	0
RAPGEF1	TGACACCACACAACTGTG	657	219	0	1	1	0
RAPGEF1	CAACACCACACATGGTGT	89	139	0	0	0	0

RAPGEF1	ACACACCACACATGGTCA	68	54	0	0	0	0
RAPGEF1	GTACACCACACATGGTAC	31	133	0	0	0	0
RAPGEF1	TGACACCACACATGGTTG	1397	632	2	1	0	9
RAPSN	GTACGTGTACCAGTGTCA	503	726	0	2	1	1
RAPSN	CAACGTGTACGTTGTGCA	221	54	0	0	0	0
RAPSN	TGACGTGTACGTTGTGAC	58	36	0	0	0	0
RAPSN	ACACGTGTACGTTGTGTG	697	487	1	0	0	0
RAPSN	TGACGTGTACCAGTGTGT	1673	1815	7	1	0	63
RARA	GTACGTTGCAGTTGGTTG	854	422	0	0	1	0
RARA	CAACGTTGCAGTTGGTAC	280	243	0	0	0	88
RARA	TGACGTTGCAGTTGCAGT	90	38	1	0	0	0
RARA	TGTGCAGTGTACTGTGCA	191	256	0	0	0	1
RARA	CATGCAGTGTACTGTGAC	49	38	0	0	0	0
RARB	ACACGTTGACGTGTTGGT	158	126	20	30	16	71
RARB	GTACGTTGACGTGTACAC	68	52	0	0	0	0
RARB	TGACGTTGACGTGTACTG	98	80	0	0	0	0
RARB	TGACGTTGACGTGTTGCA	47	127	0	0	0	1
RARB	TGACCAGTCAACTGGTGT	231	232	1	0	0	0
RARB	GTTGCAACGTCACAGTAC	37	16	0	0	0	0
RARG	ACACGTTGACGTCAACTG	1005	969	1	1	1	0
RARG	GTACGTTGACGTCAACGT	408	120	0	0	0	0
RARG	CAACGTTGACGTCAACCA	163	228	1	1	0	0
RARG	TGACGTTGACGTCAACAC	15	23	0	0	0	0
RARG	CATGGTTGTGACTGCAAC	24	27	0	0	0	0
RARS	ACACACGTCACACATGCA	157	90	0	0	0	0
RARS	GTACACGTCACACATGAC	32	30	0	0	0	0
RARS	TGACACGTCACACATGTG	148	536	0	0	0	0
RARS	CAACACGTCACAACGTGT	377	343	0	0	0	0
RARS	ACACACGTCACAACGTCA	60	109	1	0	0	0
RASA1	TGCATGTGTGACGTGTCA	321	427	0	0	0	0
RASA1	CACATGTGTGACGTGTAC	38	46	0	0	0	0
RASA1	GTCATGTGTGACGTGTTG	523	324	1	2	0	1
RASA1	TGCATGTGTGACGTCAGT	151	194	1	0	0	0
RASA1	GTCATGTGTGACGTCACA	185	70	0	0	0	0
RASA1	CATGCACATGACTGGTAC	74	46	0	0	0	0
RASAL1	CACATGTGACGTGTTGGT	80	67	0	0	0	0
RASAL1	ACCATGTGACGTGTTGCA	102	44	0	0	0	0
RASAL1	GTCATGTGACGTGTTGAC	154	83	0	0	0	0
RASAL1	TGCATGTGACGTGTTGTG	162	196	0	1	0	0
RASAL1	ACTGCAGTACCAGTACCA	150	112	0	0	0	0
RASAL1	TGTGCACACATGCAACCA	531	339	0	0	0	0
RASGRF1	TGACACCACACACATGCA	392	345	0	0	0	3
RASGRF1	CAACACCACACACATGAC	344	82	0	0	0	0
RASGRF1	GTACACCACACACATGTG	576	282	2	0	0	0
RASGRF1	ACACACCACACAACGTGT	166	160	0	0	0	0
RASGRF1	TGACACCACACAACGTCA	759	554	2	0	0	0
RASGRF1	GTTGCAACTGACCACATG	52	77	0	0	0	0
RASGRP1	ACACACCACAACCACAGT	175	416	0	0	0	0
RASGRP1	TGACACCACAACCACACA	36	50	0	0	0	0
RASGRP1	CAACACCACAACCACAAC	171	22	0	0	0	0
RASGRP1	GTACACCACAACCACATG	385	334	0	0	0	0
RASGRP1	GTTGCAGTTGGTTGGTGT	244	349	1	0	0	0
RASGRP1	TGTGCACAACACGTCAGT	179	271	0	1	0	0
RASGRP3	CAACGTTGTGACGTCACA	56	65	1	0	0	0
RASGRP3	ACACACCACACAACACTG	501	491	1	0	0	1
RASGRP3	CAACACCACACAACTGGT	484	536	1	1	0	2
RASGRP3	ACACACCACACAACTGCA	505	725	1	2	0	20
RASGRP3	GTACACCACACAACTGAC	263	78	0	0	0	0
RASGRP3	GTTGCAACTGACGTTGAC	22	51	0	0	0	0
RB1	GTACCATGACGTCATGGT	817	851	1	0	1	7
RB1	TGACCATGACGTCAACGT	490	707	0	0	0	15
RB1	GTACCATGACGTCAACCA	212	217	3	0	0	0
RB1	ACACCATGACGTCAACAC	72	126	0	0	0	0
RB1	CAACCATGACGTCAACTG	592	491	2	1	0	2
RBBP4	CATGGTGTACACGTTGGT	353	672	0	1	1	1
RBBP4	ACTGCACAACGTCACACA	55	45	0	2	1	0
RBBP4	CATGGTGTACACGTACCA	116	124	0	0	0	0
RBBP4	TGTGGTGTACACGTACAC	9	1	0	0	0	0
RBBP4	ACTGGTGTACACGTACTG	180	387	0	0	0	0
RBBP4	ACTGGTGTACACGTTGCA	130	171	1	0	0	0
RBBP7	ACACCATGGTACGTGTTG	151	137	0	0	0	0
RBBP7	CAACCATGGTACGTCAGT	340	285	0	1	0	0
RBBP7	ACACCATGGTACGTCACA	10	25	0	0	0	0
RBBP7	GTACCATGGTACGTCAAC	11	12	0	0	0	0
RBBP7	TGACCATGGTACGTCATG	106	149	102	0	0	1

RBBP7	GTTGCACATGTGGTTGAC	34	13	0	0	0	0
RBBP8	GTCATGTGACACGTGTCA	376	369	0	0	1	0
RBBP8	TGCATGTGACCATGTGTG	932	911	1	0	0	6
RBBP8	TGCATGTGACACGTGTGT	168	169	0	0	0	0
RBBP8	ACCATGTGACACGTGTAC	54	60	0	0	0	0
RBBP8	CACATGTGACACGTGTTG	401	754	0	0	0	1
RBKS	ACTGGTTGGTGTACTGGT	340	419	0	1	1	0
RBKS	GTACTGTGACACCAACTG	708	846	1	0	0	0
RBKS	TGTGGTTGGTGTACACTG	120	486	0	1	0	0
RBKS	TGTGGTTGGTGTACTGCA	51	95	0	0	0	0
RBKS	CATGGTTGGTGTACTGAC	2	10	0	0	0	0
RBKS	ACTGCACATGACCAACGT	428	492	0	0	0	0
RBL1	CAACCATGCAACTGTGGT	737	2009	1	0	1	1
RBL1	ACACCATGCAACTGTGCA	562	595	0	1	1	0
RBL1	CAACCATGCAACTGACCA	486	258	0	0	0	0
RBL1	TGACCATGCAACTGACAC	214	38	0	0	0	0
RBL1	ACACCATGCAACTGACTG	112	141	0	0	0	0
RBL2	TGACCATGCAACGTTGAC	31	50	0	0	0	0
RBL2	ACACCATGCAACGTTGTG	501	162	0	0	0	1
RBL2	GTACCATGCAACCAGTGT	1186	1849	25	2	0	0
RBL2	CATGGTTGTGCATGCACA	36	23	0	0	0	0
RBL2	TGTGGTTGTGCATGCAAC	12	22	0	0	0	0
RBL2	ACTGCAACACGTCAGTCA	168	260	0	0	0	0
RBL2	TGTGTGACACACGTGTTG	241	266	1	0	0	0
RBL2	ACTGTGACACACGTCAGT	103	128	0	0	0	0
RBL2	TGTGTGACACACGTCACA	32	18	0	0	0	0
RBL2	TGTGTGACACACGTACGT	253	339	0	0	0	0
RBL2	GTTGTGACACACGTACCA	256	447	0	0	0	0
RBL2	ACTGTGACACACGTACAC	37	33	0	0	0	0
RBL2	CATGTGACACACGTTGCA	674	648	1	0	0	0
RBL2	TGTGTGACACACGTTGAC	30	43	0	0	0	0
RBL2	ACTGTGACACACGTTGTG	739	256	1	2	0	0
RBL2	TGTGTGACACTGACTGGT	127	105	0	0	0	0
RBL2	GTTGTGACACTGACTGCA	21	57	0	0	0	0
RBL2	ACTGTGACACTGACTGAC	12	16	0	0	0	0
RBL2	GTTGTGACACTGTGGTCA	618	444	1	1	0	1
RBL2	ACTGTGACACTGTGGTAC	59	32	0	0	0	0
RBL2	CATGTGACACTGTGGTTG	249	141	1	0	0	0
RBL2	TGTGTGACACTGTGCAAC	33	26	0	0	0	0
RBL2	ACTGTGACACTGTGCATG	248	194	1	1	0	0
RBL2	CATGTGACACTGTGACGT	235	295	1	0	0	2
RBL2	TGTGTGACACTGTGACTG	402	629	0	0	0	0
RBL2	ACTGTGACACTGTGTGGT	1202	297	0	4	0	1
RBL2	TGTGTGACACTGTGTGCA	427	355	1	0	0	0
RBM8A	ACACCATGGTACGTACGT	91	69	0	0	0	1
RBM8A	TGACCATGGTACGTACCA	131	186	0	1	0	0
RBM8A	CAACCATGGTACGTACAC	32	37	0	0	0	0
RBM8A	GTACCATGGTACGTACTG	304	957	0	1	0	1
RBM8A	CATGCAACGTACGTCAGT	291	53	0	1	0	0
RBM8A	ACTGCAACGTACGTCACA	155	191	0	0	0	0
RBP4	TGACTGCACAACCACATG	126	115	0	0	0	2
RBP4	ACACTGCACAACCAACGT	605	404	0	0	0	0
RBP4	TGACTGCACAACCAACCA	797	922	2	2	0	3
RBP4	CAACTGCACAACCAACAC	168	110	0	0	0	7
RBP4	GTACTGCACAACCAACTG	360	394	0	1	0	0
RBPJ	CAACGTACCATGGTCATG	77	77	0	0	0	0
RBPJ	GTACGTACCATGGTACGT	361	187	0	0	0	0
RBPJ	CAACGTACCATGGTACCA	87	186	0	0	0	1
RBPJ	TGACGTACCATGGTACAC	30	30	0	0	0	0
RBPJ	ACACGTACCATGGTACTG	168	130	0	0	0	0
RBPJL	GTACGTTGACTGACCAGT	149	447	1	0	0	0
RBPJL	CAACGTTGACTGACCACA	27	39	0	0	0	0
RBPJL	TGACGTTGACTGACCAAC	22	24	0	0	0	0
RBPJL	ACACGTTGACTGACCATG	80	94	0	0	0	0
RBPJL	CAACGTTGACTGACACGT	68	94	0	1	0	0
RBX1	CATGTGACACCAGTGTTG	895	1252	1	1	1	1
RBX1	TGACGTTGTGACGTGTGT	3	69	0	0	0	0
RBX1	GTACGTTGTGACGTGTCA	181	128	0	0	0	0
RBX1	ACACGTTGTGACGTGTAC	28	22	0	0	0	0
RBX1	CAACGTTGTGACGTGTTG	346	387	0	1	0	0
RBX1	GTACGTTGTGACGTCAGT	95	150	0	0	0	0
RBX1	ACTGCAACACACACCAGT	97	65	0	0	0	0
RBX1	GTTGTGACACGTACTGAC	2	9	0	0	0	0
RBX1	TGTGTGACACGTACTGTG	48	70	0	0	0	0
RBX1	CATGTGACACGTTGGTGT	244	143	0	0	0	0

RBX1	TGTGTGACACGTTGGTTG	263	243	2	1	0	0
RBX1	ACTGTGACACGTTGCAGT	30	36	0	0	0	1
RBX1	TGTGTGACACGTTGCACA	99	74	0	0	0	1
RBX1	TGTGTGACACGTTGACGT	235	335	0	0	0	0
RBX1	GTTGTGACACGTTGACCA	323	396	1	0	0	0
RBX1	ACTGTGACACGTTGACAC	90	80	0	0	0	0
RBX1	CATGTGACACGTTGTGCA	177	140	0	0	0	0
RBX1	TGTGTGACACGTTGTGAC	33	57	0	0	0	0
RBX1	ACTGTGACACGTTGTGTG	63	108	0	0	0	5
RBX1	ACTGTGACACCAGTGTAC	30	25	0	0	0	0
RBX1	GTTGTGACACCAGTCAGT	139	172	0	0	0	0
RBX1	TGTGTGACTGGTACGTTG	208	219	0	0	0	0
RBX1	ACTGTGACTGGTACCAGT	536	647	0	0	0	0
RBX1	TGTGTGACTGGTACCACA	148	167	0	0	0	0
RBX1	TGTGTGACTGGTACACGT	104	7	0	0	0	0
RBX1	GTTGTGACTGGTACACCA	24	36	0	0	0	0
RBX1	ACTGTGACTGGTACACAC	8	17	0	0	0	0
RCAN1	ACACGTTGGTACGTGTCA	12	33	0	0	0	0
RCAN1	GTACGTTGGTACGTGTAC	105	207	0	0	0	0
RCAN1	TGACGTTGGTACGTGTTG	194	141	0	1	0	0
RCAN1	ACACGTTGGTACGTCAGT	153	91	0	0	0	0
RCAN1	TGTGCAGTGTCATGTGAC	26	32	0	0	0	0
RCHY1	TGCATGTGGTTGACGTGT	591	596	2	0	0	4
RCHY1	GTCATGTGGTTGACGTCA	160	409	1	0	0	0
RCHY1	ACCATGTGGTTGACGTAC	21	4	0	0	0	0
RCHY1	CACATGTGGTTGACGTTG	1264	1163	0	2	0	7
RCHY1	GTTGCAGTCATGTGACCA	98	56	0	0	0	0
RDH11	TGACCAGTCAGTCATGGT	796	597	5	1	1	0
RDH11	CAACCAGTCAGTCAACAC	199	202	0	0	0	0
RDH11	GTACCAGTCAGTCAACTG	812	952	0	0	0	0
RDH11	GTACCAGTCAGTCATGCA	174	173	0	0	0	0
RDH11	ACACCAGTCAGTCATGAC	222	186	0	0	0	0
RDH11	ACTGCAACCAACGTACAC	97	55	0	0	0	0
RDH12	ACACCAGTGTCAACTGTG	187	242	0	0	1	0
RDH12	CAACCAGTGTCATGGTCA	682	558	0	0	1	2
RDH12	TGACCAGTGTCAACTGAC	73	69	0	0	0	0
RDH12	GTACCAGTGTCATGGTGT	677	617	0	1	0	0
RDH12	TGACCAGTGTCATGGTAC	65	62	0	0	0	0
RDH12	TGTGCAACACTGCATGCA	194	191	0	1	0	1
RDH13	ACACCAGTCAACTGCATG	371	529	1	0	1	63
RDH13	GTACCAGTCAACTGCAGT	740	492	0	2	0	0
RDH13	CAACCAGTCAACTGCACA	432	388	1	1	0	3
RDH13	TGACCAGTCAACTGCAAC	55	208	0	0	0	0
RDH13	CAACCAGTCAACTGACGT	73	94	0	0	0	0
RDH14	CAACCAGTGTTGCATGCA	382	389	25	0	0	0
RDH14	TGACCAGTGTTGCATGAC	46	46	0	0	0	0
RDH14	ACACCAGTGTTGCATGTG	417	890	0	0	0	19
RDH14	GTACCAGTGTTGACGTGT	244	117	0	0	0	0
RDH14	TGTGCAGTACTGGTACAC	57	115	0	0	0	0
RDH14	TGTGCACATGCACACAGT	61	68	0	0	0	0
RDH5	GTACCAGTGTCAGTCAGT	111	105	0	0	0	0
RDH5	CAACCAGTGTCAGTCACA	159	183	0	0	0	0
RDH5	TGACCAGTGTCAGTCAAC	59	24	0	0	0	0
RDH5	GTACCAGTGTCATGTGCA	68	126	0	0	0	0
RDH5	ACACCAGTGTCATGTGAC	51	57	0	0	0	0
RDM1	TGTGGTACACACTGACGT	242	497	0	0	0	1
RDM1	GTTGGTACACACTGACCA	255	237	0	0	0	0
RDM1	ACTGGTACACACTGACAC	161	135	0	2	0	0
RDM1	TGTGACCAACGTCAACTG	861	349	1	0	0	0
RDM1	ACTGACCAACGTCATGGT	1221	1227	4	0	0	0
RECK	TGACTGTGGTGTACACTG	233	382	0	0	0	1
RECK	ACACTGTGGTGTACTGGT	34	69	0	0	0	0
RECK	TGACTGTGGTGTACTGCA	109	162	0	0	0	0
RECK	CAACTGTGGTGTACTGAC	50	41	0	0	0	0
RECK	GTACTGTGGTGTACTGTG	184	191	0	0	0	0
RECK	GTTGCACATGACCATGCA	16	113	0	0	0	0
RECQL	CATGCAGTCAACCAACCA	427	590	4	4	2	4
RECQL	ACACACACACACGTACAC	35	16	0	0	0	0
RECQL	CAACACACACACGTACTG	569	242	2	0	0	0
RECQL	GTACACACACACGTTGGT	115	110	0	0	0	0
RECQL	CAACACACACACGTTGCA	82	117	0	0	0	0
RECQL	CATGCAACGTCAGTCATG	139	140	0	1	0	0
RECQL4	TGTGACACCACAACCATG	126	134	13	6	11	5
RECQL4	GTACACACCACACATGCA	328	384	3	3	2	6
RECQL4	GTACACACCACACAACTG	1352	1309	2	1	1	0

RECQL4	TGACACACCACACATGGT	206	278	0	0	0	0
RECQL4	ACACACACCACACATGAC	43	40	0	0	0	0
RECQL5	CAACACACCAACTGTGTG	568	781	1	0	0	1
RECQL5	GTACACACCATGGTGTGT	336	445	2	0	0	0
RECQL5	CAACACACCATGGTGTCA	367	260	0	0	0	0
RECQL5	GTTGGTTGTGCATGACAC	20	29	0	0	0	0
RECQL5	TGTGGTTGTGCATGACTG	301	341	1	0	0	0
REG1A	GTACTGCATGTGTGACTG	463	344	0	0	0	0
REG1A	TGACTGCATGTGTGTGGT	221	209	0	2	0	1
REG1A	GTACTGCATGTGTGTGCA	87	83	0	0	0	0
REG1A	ACACTGCATGTGTGTGAC	50	44	0	0	0	0
REG1A	TGTGCATGGTACTGGTCA	158	201	2	0	0	3
REG1A	CATGCATGGTACTGGTAC	155	84	0	0	0	0
REG3A	GTACTGCATGTGACACCA	259	309	0	6	221	1
REG3A	ACACTGCATGTGACACAC	48	69	0	0	1	0
REG3A	CAACTGCATGTGACACTG	448	421	0	0	0	0
REG3A	GTACTGCATGTGACTGGT	236	268	0	1	0	3
REG3A	CAACTGCATGTGACTGCA	95	106	1	1	0	0
REG3A	ACTGCAACCATGCAACAC	75	121	0	0	0	0
REL	TGACCATGCAACACTGCA	88	93	3	11	14	28
REL	GTACCATGCAACACACAC	48	247	0	0	0	0
REL	TGACCATGCAACACACTG	77	103	0	0	0	0
REL	ACACCATGCAACACTGGT	503	222	0	1	0	0
REL	CAACCATGCAACACTGAC	58	24	0	0	0	0
RELA	CAACGTACGTACGTGTCA	46	46	0	0	0	0
RELA	TGACGTACGTACGTGTAC	81	72	0	0	0	0
RELA	ACACGTACGTACGTGTTG	181	301	0	1	0	0
RELA	CAACGTACGTACGTCAGT	198	162	1	0	0	0
RELA	GTTGACGTCAGTGTCATG	617	372	0	0	0	1
RELB	CAACGTACGTACGTTGTG	160	95	0	0	0	0
RELB	TGACGTACGTACCAGTGT	233	124	0	0	0	0
RELB	GTACGTACGTACCAGTCA	73	45	1	0	0	0
RELB	ACACGTACGTACCAGTAC	4	27	0	0	0	0
RELB	TGTGCAGTCAACTGGTAC	172	69	1	0	0	0
RELN	TGACACACACCAACTGAC	57	42	0	0	0	0
RELN	ACACACACACCAACTGTG	335	360	2	0	0	0
RELN	GTACACACACCATGGTGT	191	237	0	0	0	0
RELN	CAACACACACCATGGTCA	137	142	0	0	0	0
RELN	TGACACACACCATGGTAC	274	323	0	0	0	1
RELN	GTTGCAACTGTGACACCA	65	239	0	0	0	0
REM2	ACACGTTGGTCAGTGTAC	77	73	1	0	0	0
REM2	CAACGTTGGTCAGTGTTG	352	380	1	0	0	3
REM2	GTACGTTGGTCAGTCAGT	188	185	0	0	0	0
REM2	CAACGTTGGTCAGTCACA	184	169	0	0	0	0
REM2	ACTGCAGTACACTGTGCA	75	138	1	0	0	0
REN	GTACACCATGCATGACGT	240	282	1	5	9	1
REN	TGACACCATGCATGCAGT	480	596	0	0	0	0
REN	GTACACCATGCATGCACA	67	99	0	0	0	0
REN	ACACACCATGCATGCAAC	22	12	0	0	0	0
REN	CAACACCATGCATGCATG	231	188	0	0	0	0
REN	ACTGCACATGACGTGTCA	553	789	1	0	0	1
RENBP	ACTGGTCAGTCAGTACTG	192	269	0	1	0	0
RENBP	CATGGTCAGTCAGTTGGT	324	163	0	0	0	0
RENBP	ACTGGTCAGTCAGTTGCA	250	305	0	1	0	0
RENBP	GTTGGTCAGTCAGTTGAC	82	106	0	2	0	0
RENBP	TGTGCACATGACCAGTAC	58	20	0	0	0	0
RENBP	ACTGCACATGACCAGTTG	292	257	0	1	0	4
REPS1	CAACACTGGTGTTGACGT	151	233	0	0	0	0
REPS1	ACACACTGGTGTTGACCA	290	181	1	0	0	1
REPS1	GTACACTGGTGTTGACAC	455	88	0	0	0	1
REPS1	TGACACTGGTGTTGACTG	508	634	0	0	0	0
REPS1	ACACACTGGTGTTGTGGT	412	657	0	0	0	2
REPS2	ACACACTGCACATGGTAC	192	70	0	0	0	0
REPS2	CAACACTGCACATGGTTG	1147	1137	3	3	0	1
REPS2	GTACACTGCACATGCAGT	95	51	0	0	0	0
REPS2	CAACACTGCACATGCACA	90	189	0	0	0	1
REPS2	TGACACTGCACATGCAAC	18	25	0	0	0	0
RERE	CAACGTCAACTGTGTGTG	1187	1453	0	0	1	1
RERE	TGACGTCAACTGTGTGGT	585	282	0	0	0	0
RERE	GTACGTCAACTGTGTGCA	102	126	1	0	0	0
RERE	ACACGTCAACTGTGTGAC	117	96	0	0	0	0
RERE	GTTGACACGTCATGACGT	192	193	0	0	0	0
RET	TGCATGCACAACTGTGGT	284	324	0	0	1	0
RET	CACATGCACAACTGACAC	56	68	0	0	0	0
RET	GTCATGCACAACTGACTG	6	14	0	0	0	0

RET	GTCATGCACAACTGTGCA	22	29	1	0	0	0
RET	GTACCATGCAACTGTGAC	178	89	0	0	0	0
RETN	GTTGGTTGACTGCAACCA	708	643	4	1	2	0
RETN	ACTGGTTGACTGCAACAC	6	43	0	0	0	0
RETN	CATGGTTGACTGCAACTG	402	656	0	1	0	0
RETN	GTTGGTTGACTGCATGGT	727	226	1	0	0	1
RETN	CATGGTTGACTGCATGCA	311	390	1	0	0	2
RETN	TGTGCACATGTGACTGGT	87	78	0	0	0	0
RETNLB	ACTGGTACACGTTGGTTG	596	691	1	0	1	0
RETNLB	ACTGGTACACGTACTGTG	72	54	0	0	0	0
RETNLB	GTTGGTACACGTTGGTGT	331	404	0	0	0	2
RETNLB	CATGGTACACGTTGGTCA	149	118	0	0	0	0
RETNLB	TGTGGTACACGTTGGTAC	67	64	0	0	0	0
RETNLB	ACTGCAACGTGTACTGTG	622	221	0	0	0	0
REV1	GTTGGTACACGTGTGTCA	321	247	3	0	0	0
REV1	ACTGGTACACGTGTGTAC	33	25	0	0	0	0
REV1	CATGGTACACGTGTGTTG	92	176	0	0	0	0
REV1	GTTGGTACACGTGTCAGT	119	149	0	0	0	0
REV1	CATGGTACACGTGTCACA	29	23	0	0	0	0
REV3L	ACACACACTGTGTGGTGT	616	1137	1	0	0	0
REV3L	TGACACACTGTGTGGTCA	225	340	0	0	0	1
REV3L	CAACACACTGTGTGGTAC	65	21	0	1	0	0
REV3L	GTACACACTGTGTGGTTG	774	358	0	0	0	1
REV3L	TGACACACTGTGTGCAGT	45	47	0	0	0	0
REV3L	ACTGCACACAGTACACAC	68	77	0	0	0	0
REXO2	ACACACCATGCACAACGT	1340	777	1	0	0	0
REXO2	TGACACCATGCACAACCA	141	203	0	0	0	0
REXO2	CAACACCATGCACAACAC	51	78	0	0	0	0
REXO2	GTACACCATGCACAACTG	337	452	1	3	0	0
REXO2	TGACACCATGCACATGGT	199	711	0	1	0	15
RFC1	GTACGTTGTGGTGTACGT	157	121	0	0	0	0
RFC1	CAACGTTGTGGTGTACCA	97	159	0	0	0	0
RFC1	TGACGTTGTGGTGTACAC	23	39	0	0	0	0
RFC1	ACACGTTGTGGTGTACTG	190	145	0	0	0	0
RFC1	CAACGTTGTGGTGTTGGT	319	833	1	1	0	0
RFC5	GTTGGTACACACACTGAC	38	36	0	0	0	12
RFC5	TGTGGTACACACACTGTG	127	147	0	0	0	0
RFC5	CATGGTACACACTGGTGT	595	145	1	1	0	0
RFC5	ACTGGTACACACTGGTCA	153	191	0	0	0	0
RFC5	GTTGGTACACACTGGTAC	42	43	0	0	0	0
RFC5	CATGCATGCAGTTGTGGT	661	990	2	1	0	6
RFK	TGACCAACACTGGTACTG	1878	844	1	1	1	0
RFK	ACACCAACACTGGTACCA	309	490	0	0	0	0
RFK	GTACCAACACTGGTACAC	6	6	0	0	0	0
RFK	ACACCAACACTGGTTGGT	232	260	0	0	0	0
RFK	TGACCAACACTGGTTGCA	50	108	1	0	0	0
RFNG	CATGGTACTGGTTGACGT	240	241	6	816	4	0
RFNG	ACTGGTACTGGTTGACCA	226	293	0	0	0	0
RFNG	GTTGGTACTGGTTGACAC	62	100	0	0	0	0
RFNG	TGTGGTACTGGTTGACTG	243	553	0	0	0	0
RFNG	ACTGGTTGACGTCAGTAC	47	40	0	0	0	0
RFWD2	TGACCAGTTGACCATGTG	810	416	1	0	0	0
RFWD2	CAACCAGTTGACACGTGT	889	648	0	0	0	0
RFWD2	ACACCAGTTGACACGTCA	34	46	0	0	0	0
RFWD2	GTACCAGTTGACACGTAC	85	80	0	0	0	1
RFWD2	TGACCAGTTGACACGTTG	504	265	0	2	0	0
RFX4	TGACGTACGTTGGTACGT	121	92	0	0	0	1
RFX4	GTACGTACGTTGGTACCA	128	172	0	0	0	0
RFX4	ACACGTACGTTGGTACAC	58	64	0	0	0	0
RFX4	TGACTGTGCACACATGGT	242	314	0	1	0	0
RFX4	TGTGACGTCAGTACTGCA	46	57	0	0	0	0
RFX5	ACCATGTGACTGCACATG	217	278	2	0	0	20
RFX5	CACATGTGACTGCAACGT	175	134	0	0	0	0
RFX5	ACCATGTGACTGCAACCA	912	510	1	0	0	1
RFX5	GTCATGTGACTGCAACAC	32	16	0	0	0	0
RFX5	TGCATGTGACTGCAACTG	243	256	3	0	0	12
RFXANK	ACACGTCAACACTGCATG	438	652	0	0	1	0
RFXANK	CAACGTCAACACTGGTTG	1222	1302	1	0	0	38
RFXANK	GTACGTCAACACTGCAGT	147	277	0	0	0	0
RFXANK	CAACGTCAACACTGCACA	491	217	1	0	0	0
RFXANK	TGACGTCAACACTGCAAC	15	22	0	0	0	0
RFXANK	TGTGCAACCACATGTGGT	163	420	1	1	0	0
RFXAP	TGACGTACGTTGACCAAC	38	27	0	0	0	0
RFXAP	ACACGTACGTTGACCATG	39	218	0	0	0	0
RFXAP	CAACGTACGTTGACACGT	2	2	0	0	0	0

RFXAP	GTTGCAGTACACACGTCA	92	79	0	0	0	0
RFXAP	GTTGCAACCACATGTGCA	156	382	0	0	0	0
RFXAP	ACTGCAACCACATGTGAC	47	44	0	0	0	1
RGS1	TGACCAACACACCACACA	211	215	2	78	37	5
RGS1	ACTGACGTTGCACAGTTG	492	527	0	2	1	2
RGS1	CAACCAACACACCACAAC	5	10	0	0	0	0
RGS1	GTACCAACACACCACATG	269	910	2	0	0	1
RGS1	TGACCAACACACCAACGT	256	185	0	0	0	1
RGS16	CAACGTACGTGTGTACGT	161	74	0	0	0	1
RGS16	ACACGTACGTGTGTACCA	174	127	0	0	0	0
RGS16	GTACGTACGTGTGTACAC	18	5	0	0	0	0
RGS16	TGTGCAGTACACCAACCA	455	354	0	0	0	0
RGS16	CATGCATGACCAACCAGT	348	352	0	0	0	0
RGS19	ACACCAACCATGTGACTG	267	243	0	0	0	0
RGS19	CAACCAACCATGTGTGGT	688	643	1	0	0	9
RGS19	ACACCAACCATGTGTGCA	644	738	1	0	0	1
RGS19	CATGCAGTCAACGTTGAC	77	11	0	0	0	0
RGS19	GTTGCAGTCAACGTTGTG	348	189	1	0	0	0
RGS4	CAACGTACGTGTGTTGAC	23	22	0	0	0	0
RGS4	GTACGTACGTGTGTTGTG	813	60	0	0	0	0
RGS4	ACACGTACGTGTCAGTGT	576	333	0	0	0	2
RGS4	TGACGTACGTGTCAGTCA	231	237	1	0	0	0
RGS4	ACACCACACACACACACA	200	151	0	0	0	0
RGS5	CAACGTACGTGTCAGTAC	50	49	0	0	1	4
RGS5	GTACGTACGTGTCAGTTG	127	115	0	0	0	0
RGS5	TGACGTACGTGTCACAGT	284	279	0	0	0	0
RGS5	GTACGTACGTGTCACACA	67	70	0	0	0	0
RGS5	ACACGTACGTGTCACAAC	16	27	0	0	0	0
RHEB	CAACCATGGTTGGTACTG	97	270	0	1	0	0
RHEB	GTACCATGGTTGGTTGGT	467	613	3	1	0	0
RHEB	CAACCATGGTTGGTTGCA	269	93	0	0	0	0
RHEB	TGACCATGGTTGGTTGAC	52	139	0	1	0	0
RHEB	GTACTGTGCACACATGCA	46	92	0	1	0	0
RHOA	TGACACCACAGTCAACCA	134	134	1	0	0	0
RHOA	CAACACCACAGTCAACAC	71	42	0	0	0	0
RHOA	GTACACCACAGTCAACTG	629	235	0	0	0	0
RHOA	TGACACCACAGTCATGGT	86	82	0	0	0	0
RHOA	GTACACCACAGTCATGCA	244	560	1	1	0	2
RHOQ	TGACACCAGTTGTGTGCA	385	217	2	0	0	0
RHOQ	CAACACCAGTTGTGTGAC	57	31	0	0	0	0
RHOQ	GTACACCAGTTGTGTGTG	495	488	0	0	0	0
RHOQ	CAACACCACAGTGTGTGT	709	383	1	0	0	0
RHOQ	ACACACCACAGTGTGTCA	128	171	0	0	0	0
RING1	TGACGTTGACTGTGTGTG	250	206	0	0	0	0
RING1	ACACGTTGTGGTGTGTGT	122	316	1	0	0	8
RING1	TGACGTTGTGGTGTGTCA	115	182	1	0	0	0
RING1	CAACGTTGTGGTGTGTAC	59	39	0	0	0	0
RING1	CATGACGTACGTTGACAC	30	24	0	0	0	0
RIPK1	CACATGCAACACTGACCA	83	109	0	0	0	1
RIPK1	TGCATGCAACACTGACAC	50	52	0	1	0	0
RIPK1	ACCATGCAACACTGACTG	327	280	0	1	0	0
RIPK1	CACATGCAACACTGTGGT	892	583	27	0	0	6
RIPK1	ACCATGCAACACTGTGCA	401	437	1	0	0	1
RIPK2	TGACGTGTGTCAACGTCA	78	94	0	0	0	0
RIPK2	CAACGTGTGTCAACGTAC	28	55	0	0	0	0
RIPK2	GTACGTGTGTCAACGTTG	234	293	1	0	0	0
RIPK2	TGACGTGTGTCAACCAGT	168	191	0	1	0	0
RIPK2	GTACGTGTGTCAACCACA	156	114	0	0	0	1
RIPK3	TGCATGACCAGTTGACCA	64	93	0	0	0	0
RIPK3	CACATGACCAGTTGACAC	81	89	0	0	0	0
RIPK3	GTCATGACCAGTTGACTG	257	546	0	1	0	0
RIPK3	TGCATGACCAGTTGTGGT	72	80	0	0	0	0
RIPK3	GTCATGACCAGTTGTGCA	20	25	0	0	0	0
RNASE1	TGACACCATGCAGTTGAC	14	26	0	0	0	0
RNASE1	ACACACCATGCAGTTGTG	1008	850	1	0	0	0
RNASE1	GTACACCATGCACAGTGT	364	488	1	0	0	0
RNASE1	CAACACCATGCACAGTCA	260	639	0	0	0	1
RNASE1	TGACACCATGCACAGTAC	67	52	0	0	0	0
RNASEL	GTCATGCATGCAGTCACA	54	72	0	0	0	4
RNASEL	ACCATGCATGCAGTCAAC	14	13	0	0	0	0
RNASEL	CACATGCATGCAGTCATG	633	273	1607	2	0	0
RNASEL	GTCATGCATGCAGTACGT	231	222	0	0	0	0
RNASEL	CACATGCATGCAGTACCA	740	455	4	1	0	0
RNASEL	CATGCATGGTTGGTTGGT	105	98	0	0	0	0
RNASEN	CAACGTTGTGCAGTACTG	97	225	0	0	0	0

RNASEN	GTACGTTGTGCAGTTGGT	57	63	0	0	0	0
RNASEN	CAACGTTGTGCAGTTGCA	177	559	0	0	0	0
RNASEN	TGACGTTGTGCAGTTGAC	52	28	0	0	0	0
RNASEN	CATGCAGTCAGTGTTGGT	835	821	1	2	0	1
RNF146	ACACCACACAGTACACGT	248	159	1	0	1	0
RNF146	TGACCACACAGTACACCA	468	210	0	0	0	0
RNF146	CAACCACACAGTACACAC	98	62	0	0	0	0
RNF146	GTACCACACAGTACACTG	528	374	1	0	0	3
RNF146	TGACCACACAGTACTGGT	193	389	0	0	0	0
RNF19A	GTCATGTGCACATGCAAC	17	142	0	0	0	0
RNF19A	TGCATGTGCACATGCATG	55	103	0	0	0	0
RNF19A	ACCATGTGCACATGACGT	45	80	0	0	0	0
RNF19A	TGCATGTGCACATGACCA	128	126	0	0	0	0
RNF19A	CACATGTGCACATGACAC	160	78	0	0	0	0
RNF2	ACACCACACAGTCACAGT	496	620	0	0	0	0
RNF2	TGACCACACAGTCACACA	144	141	2	0	0	0
RNF2	CAACCACACAGTCACAAC	24	18	0	0	0	3
RNF2	GTACCACACAGTCACATG	282	466	2	0	0	0
RNF2	TGACCACACAGTCAACGT	77	165	0	0	0	0
RNF216	CACATGACTGGTACCATG	33	32	0	0	0	3
RNF216	GTCATGACTGGTACACGT	102	68	0	3	0	1
RNF216	CACATGACTGGTACACCA	160	481	0	1	0	0
RNF216	CAACCATGACCACACAGT	122	95	0	0	0	0
RNF216	ACTGCAGTGTTGTGTGAC	20	26	0	0	0	0
RNF25	CACATGTGCACACACATG	31	36	0	0	0	0
RNF25	GTCATGTGCACACAACGT	313	223	1	0	0	0
RNF25	CACATGTGCACACAACCA	528	434	0	0	0	0
RNF25	TGCATGTGCACACAACAC	119	44	0	0	0	0
RNF25	ACCATGTGCACACAACTG	341	101	1	1	0	0
RNF4	GTACGTACACGTTGCAGT	151	152	0	0	1	9
RNF4	GTACGTACACGTTGGTCA	80	55	0	0	0	0
RNF4	ACACGTACACGTTGGTAC	52	56	0	0	0	0
RNF4	CAACGTACACGTTGGTTG	152	243	0	0	0	0
RNF4	CAACGTACACGTTGCACA	82	51	16	2	0	1
ROCK1	TGCATGACCAGTCACAGT	116	97	0	0	0	0
ROCK1	GTCATGACCAGTCACACA	43	61	0	0	0	0
ROCK1	ACCATGACCAGTCACAAC	5	11	0	0	0	0
ROCK1	CACATGACCAGTCACATG	224	276	14	0	0	0
ROCK1	GTCATGACCAGTCAACGT	234	426	0	3	0	0
ROCK2	ACCATGCATGCAACGTTG	970	652	2	0	2	0
ROCK2	TGCATGCATGCAACCATG	114	120	10	1	2	21
ROCK2	CACATGCATGCAACCAGT	1437	722	8	2	1	10
ROCK2	ACCATGCATGCAACCACA	123	91	0	0	0	0
ROCK2	GTCATGCATGCAACCAAC	30	39	0	0	0	0
ROR2	GTCATGACGTGTCAACCA	452	532	2	1	1	1
ROR2	CACATGACGTGTCACAAC	2	2	0	0	0	0
ROR2	GTCATGACGTGTCACATG	173	370	0	0	0	0
ROR2	TGCATGACGTGTCAACGT	225	195	1	0	0	0
ROR2	TGTGGTACTGACGTACTG	393	234	0	0	0	0
ROR2	TGTGCAACCAGTTGTGAC	167	42	0	0	0	0
RORC	ACACCACACAGTGTCAAC	30	20	0	0	0	0
RORC	CAACCACACAGTGTCATG	59	100	1	0	0	0
RORC	GTACCACACAGTGTACGT	410	359	0	0	0	0
RORC	CAACCACACAGTGTACCA	110	109	0	0	0	0
RORC	GTTGACGTCACATGACCA	142	216	0	0	0	0
RPA1	TGCATGTGTGCATGCACA	93	116	0	0	0	0
RPA1	CACATGTGTGCATGCAAC	77	61	0	0	0	0
RPA1	ACCATGTGTGACACTGAC	166	98	0	1	0	0
RPA1	CACATGTGTGACACTGTG	169	189	0	0	0	0
RPA1	ACTGCAGTACGTGTACGT	32	29	0	0	0	0
RPA1	TGTGCAACCAACGTCACA	70	73	0	0	0	0
RPA2	CACATGTGTGCATGACTG	222	183	1	1	1	0
RPA2	GTCATGTGTGCATGCATG	11	13	0	0	0	0
RPA2	TGCATGTGTGCATGACGT	75	43	0	0	0	0
RPA2	GTCATGTGTGCATGACCA	29	10	0	0	0	0
RPA2	ACCATGTGTGCATGACAC	19	28	0	0	0	0
RPA3	ACACGTACACTGACACTG	229	276	0	0	0	0
RPA3	CAACGTACACTGACTGGT	99	70	0	0	0	1
RPA3	ACACGTACACTGACTGCA	225	206	1	0	0	0
RPA3	GTACGTACACTGACTGAC	18	13	0	0	0	0
RPA3	TGACGTACACTGACTGTG	241	204	0	0	0	0
RPA4	TGTGGTTGGTACGTACAC	41	112	0	0	0	0
RPA4	ACTGGTTGGTACGTACTG	325	501	1	0	0	0
RPA4	CATGGTTGGTACGTTGGT	397	317	2	0	0	1
RPA4	ACTGGTTGGTACGTTGCA	84	78	0	0	0	0

RPA4	GTTGGTTGGTACGTTGAC	54	43	0	1	0	0
RPE	TGACACCAACTGACACTG	551	281	0	0	1	0
RPE	GTACACCAACTGACACAC	70	73	0	0	0	0
RPE	ACACACCAACTGACTGGT	1860	1290	2	0	0	0
RPE	TGACACCAACTGACTGCA	99	137	0	0	0	2
RPE	CAACACCAACTGACTGAC	27	33	0	0	0	0
RPE	CATGCAACTGCAGTCAGT	34	31	0	0	0	0
RPIA	GTACACCAACTGCATGCA	369	210	0	0	1	1
RPIA	GTACACCAACTGCAACTG	356	583	2	0	0	0
RPIA	TGACACCAACTGCATGGT	156	250	0	1	0	0
RPIA	ACACACCAACTGCATGAC	55	40	1	0	0	0
RPIA	CAACACCAACTGCATGTG	817	1379	4	2	0	0
RPL10	TGTGGTGTTGACTGGTCA	87	64	0	1	0	0
RPL10	CATGGTGTTGACTGGTAC	31	27	0	0	0	0
RPL10	GTTGGTGTTGACTGGTTG	429	420	1	0	0	0
RPL10	TGTGGTGTTGACTGCAGT	100	47	1	0	0	0
RPL10	GTTGGTGTTGACTGCACA	72	51	0	0	0	0
RPL10	ACTGCAACGTACGTGTTG	406	331	0	1	0	0
RPL11	CATGGTGTTGTGGTACCA	29	41	0	0	0	0
RPL11	TGTGGTGTTGTGGTACAC	3	9	0	0	0	0
RPL11	ACTGGTGTTGTGGTACTG	72	38	0	0	0	0
RPL11	CATGGTGTTGTGGTTGGT	38	15	0	0	0	0
RPL11	ACTGGTGTTGTGGTTGCA	152	137	0	0	0	0
RPL11	ACTGCAACGTCAACACGT	80	66	0	0	0	0
RPL12	TGTGGTGTCACACATGAC	39	27	0	0	0	0
RPL12	CATGGTGTTGACACGTTG	370	397	0	0	0	0
RPL12	GTTGGTGTTGACACCAGT	186	192	0	0	0	0
RPL12	CATGGTGTTGACACCACA	25	17	0	0	0	0
RPL12	TGTGGTGTTGACACCAAC	4	73	0	0	0	0
RPL12	TGTGCAACGTCAACACCA	126	98	0	0	0	0
RPL13	CATGGTGTTGACCATGTG	1621	622	2	2	0	1
RPL13	TGTGGTGTTGACACGTGT	91	120	0	0	0	1
RPL13	GTTGGTGTTGACACGTCA	153	143	1	0	0	0
RPL13	ACTGGTGTTGACACGTAC	14	17	0	0	0	0
RPL13	GTTGCAACGTCAACACTG	431	407	0	1	0	0
RPL13	TGTGCAACGTCAACTGGT	145	183	0	0	0	0
RPL13A	GTTGGTGTTGTGGTTGAC	7	10	0	0	0	0
RPL13A	TGTGGTGTTGTGGTTGTG	28	33	0	0	0	0
RPL13A	CATGGTGTTGTGCAGTGT	258	291	0	0	0	0
RPL13A	ACTGGTGTTGTGCAGTCA	172	180	1	0	0	0
RPL13A	GTTGGTGTTGTGCAGTAC	50	40	0	0	0	0
RPL13A	GTTGCAACGTCAGTACGT	279	525	0	0	0	1
RPL14	ACTGGTGTTGACACTGGT	75	102	0	0	0	0
RPL14	TGTGGTGTTGACACTGCA	29	36	0	0	0	0
RPL14	CATGGTGTTGACACTGAC	86	94	0	0	0	0
RPL14	TGTGCAGTCAACCATGTG	473	134	1	0	0	1
RPL14	CATGCAGTCAACACGTGT	173	182	0	0	0	0
RPL15	GTTGGTGTTGTGGTACGT	141	124	0	0	1	1
RPL15	TGTGGTGTTGTGGTCAGT	98	109	0	0	0	0
RPL15	GTTGGTGTTGTGGTCACA	166	195	1	0	0	0
RPL15	ACTGGTGTTGTGGTCAAC	30	36	1	0	0	0
RPL15	CATGGTGTTGTGGTCATG	13	23	0	0	0	0
RPL15	CATGCAACGTCAACACAC	95	66	0	0	0	1
RPL30	ACTGGTGTCACACATGTG	167	96	0	0	0	0
RPL30	GTTGGTGTCACAACGTGT	30	70	1	0	0	0
RPL30	CATGGTGTCACAACGTCA	89	250	1	0	0	0
RPL30	TGTGGTGTCACAACGTAC	9	5	0	0	0	0
RPL30	ACTGGTGTTGACTGGTGT	397	309	0	1	0	0
RPL30	GTTGCAACGTACGTCAAC	0	2	0	0	0	0
RPL4	GTTGGTGTTGACTGTGAC	17	16	0	0	0	0
RPL4	TGTGGTGTTGACTGTGTG	211	282	0	0	0	0
RPL4	ACTGGTGTTGTGGTGTGT	83	50	0	0	0	0
RPL4	TGTGGTGTTGTGGTGTCA	314	811	2	1	0	0
RPL4	GTTGCACAGTGTCATGGT	31	29	0	0	0	0
RPL4	GTTGCAACGTACACCACA	126	95	0	0	0	0
RPL6	CACATGTGCATGTGGTTG	215	119	0	0	0	0
RPL6	GTCATGTGCATGTGCAGT	67	46	0	0	0	0
RPL6	CACATGTGCATGTGCACA	28	48	0	0	0	0
RPL6	TGCATGTGCATGTGCAAC	7	14	0	0	0	0
RPL6	ACCATGTGCATGTGCATG	77	66	0	0	0	0
RPL8	ACTGGTGTTGACACCATG	204	215	0	0	0	0
RPL8	CATGGTGTTGACACACGT	31	60	0	0	0	0
RPL8	ACTGGTGTTGACACACCA	90	171	1	0	0	0
RPL8	GTTGGTGTTGACACACAC	13	19	0	0	0	0
RPL8	TGTGGTGTTGACACACTG	117	99	0	0	0	0

RPL8	GTTGCAACGTCAACTGCA	38	32	0	0	0	0
RPN1	TGACTGACTGTGGTTGGT	244	336	0	0	0	0
RPN1	GTACTGACTGTGGTTGCA	581	149	0	1	0	0
RPN1	ACACTGACTGTGGTTGAC	54	57	0	0	0	0
RPN1	CAACTGACTGTGGTTGTG	794	590	1	0	0	3
RPN1	TGACTGACTGTGCAGTGT	689	1280	0	0	0	3
RPN2	GTTGGTACCATGCAACGT	178	232	0	0	0	0
RPN2	CATGGTACCATGCAACCA	520	334	0	0	0	0
RPN2	TGTGGTACCATGCAACAC	15	164	0	0	0	0
RPN2	ACTGGTACCATGCAACTG	850	505	1	0	0	0
RPN2	CATGGTACCATGCATGGT	248	223	0	0	0	0
RPRM	ACTGGTTGACGTCACATG	377	447	1	3	1	0
RPRM	CATGGTTGACGTCAGTTG	343	276	0	0	0	0
RPRM	GTTGGTTGACGTCACAGT	268	351	1	0	0	0
RPRM	CATGGTTGACGTCACACA	237	111	0	0	0	0
RPRM	TGTGGTTGACGTCACAAC	56	25	0	0	0	0
RPS11	GTACTGTGGTTGTGACTG	64	49	0	0	0	0
RPS11	TGACTGTGGTTGTGTGGT	14	99	0	0	0	0
RPS11	GTACTGTGGTTGTGTGCA	205	120	1	17	0	1
RPS11	ACACTGTGGTTGTGTGAC	4	9	0	0	0	0
RPS11	CAACTGTGGTTGTGTGTG	1083	286	1	0	0	0
RPS13	GTACGTGTCAACACTGGT	387	192	0	1	0	4
RPS13	CAACGTGTCAACACTGCA	8	9	0	0	0	0
RPS13	TGACGTGTCAACACTGAC	71	78	0	0	0	0
RPS13	ACACGTGTCAACACTGTG	50	118	0	0	0	0
RPS13	GTACGTGTCAACTGGTGT	56	71	0	0	0	0
RPS13	CATGCAACGTCAGTACCA	15	10	0	0	0	0
RPS3A	ACACTGTGGTTGACGTCA	332	375	1	0	0	0
RPS3A	GTACTGTGGTTGACGTAC	37	148	0	0	0	0
RPS3A	TGACTGTGGTTGACGTTG	123	156	1	0	0	0
RPS3A	ACACTGTGGTTGACCAGT	27	37	0	0	0	0
RPS3A	GTTGGTGTTGACACTGTG	109	136	0	0	0	0
RPS6	TGACCATGCATGACGTAC	29	34	1	0	0	0
RPS6	ACACCATGCATGACGTTG	372	266	0	0	0	0
RPS6	CAACCATGCATGACCAGT	55	19	0	0	0	0
RPS6	ACACCATGCATGACCACA	13	15	0	0	0	0
RPS6	GTACCATGCATGACCAAC	7	3	0	0	0	0
RPS6	TGTGCAACGTTGGTACGT	45	40	0	0	0	0
RPS6KA1	GTCATGCATGTGCAACTG	334	437	2	1	0	0
RPS6KA1	TGCATGCATGTGCATGGT	225	260	1	0	0	0
RPS6KA1	GTCATGCATGTGCATGCA	35	34	0	0	0	0
RPS6KA1	ACCATGCATGTGCATGAC	39	64	0	0	0	0
RPS6KA1	CACATGCATGTGCATGTG	16	59	0	0	0	0
RPS6KA3	CACATGCATGTGACCACA	186	225	1	0	0	0
RPS6KA3	TGCATGCATGTGACCAAC	40	50	0	0	0	0
RPS6KA3	ACCATGCATGTGACCATG	34	67	0	0	0	0
RPS6KA3	CACATGCATGTGACACGT	83	103	0	0	0	0
RPS6KA3	ACCATGCATGTGACACCA	101	69	0	0	0	0
RPS6KA5	CACATGACGTGTCAACTG	436	387	0	0	1	0
RPS6KA5	ACCATGACGTGTCAACAC	31	39	0	0	0	0
RPS6KA5	GTCATGACGTGTCATGGT	806	377	0	0	0	0
RPS6KA5	CACATGACGTGTCATGCA	153	138	0	0	0	0
RPS6KA5	TGCATGACGTGTCATGAC	26	13	0	0	0	0
RPS6KB1	ACCATGACACACGTCAAC	31	34	0	0	0	0
RPS6KB1	CACATGACACACGTCATG	48	46	0	0	0	0
RPS6KB1	GTCATGACACACGTACGT	480	330	0	0	0	0
RPS6KB1	CACATGACACACGTACCA	156	165	3	0	0	0
RPS6KB1	TGACGTTGTGACGTCAAC	1	3	0	0	0	0
RPS6KB2	GTCATGCAACTGGTGTTG	328	312	0	1	0	1
RPS6KB2	TGCATGCAACTGGTCAGT	317	153	1	0	0	0
RPS6KB2	ACTGGTTGGTGTCATGAC	19	15	0	0	0	0
RPS6KB2	CATGGTTGGTGTCATGTG	470	618	1	0	0	18
RPS6KB2	TGTGGTTGGTGTACGTGT	205	207	1	0	0	0
RPSA	GTACCAGTTGGTGTACGT	17	31	0	0	0	0
RPSA	CAACCAGTTGGTGTACCA	90	44	0	0	0	0
RPSA	TGACCAGTTGGTGTACAC	61	30	0	0	0	0
RPSA	ACACCAGTTGGTGTACTG	111	120	0	0	0	0
RPSA	CAACCAGTTGGTGTTGGT	506	620	0	0	0	0
RRAD	CAACACCAGTTGACCAGT	167	166	0	0	0	0
RRAD	ACACACCAGTTGACCACA	206	413	0	0	0	0
RRAD	GTACACCAGTTGACCAAC	34	19	0	0	0	0
RRAD	TGACACCAGTTGACCATG	112	171	0	0	0	0
RRAD	ACACACCAGTTGACACGT	130	192	0	0	0	0
RRAD	CATGCACACATGCAACAC	156	175	0	1	0	0
RRM1	GTACCATGGTACCATGTG	25	10	0	0	0	0

RRM1	ACACCATGGTACACGTGT	249	1068	0	0	0	0
RRM1	TGACCATGGTACACGTCA	125	111	0	0	0	1
RRM1	CAACCATGGTACACGTAC	10	1	0	0	0	0
RRM1	CATGCAGTCAACCAGTAC	16	17	0	0	0	0
RRM1	TGTGCAACCATGACGTAC	9	13	0	0	0	0
RRM2	GTACCATGGTACCAACAC	55	27	0	0	0	0
RRM2	TGACCATGGTACCAACTG	513	740	0	0	0	0
RRM2	ACACCATGGTACCATGGT	45	126	0	0	0	0
RRM2	TGACCATGGTACCATGCA	347	530	0	1	0	0
RRM2	CAACCATGGTACCATGAC	50	64	1	0	0	0
RRM2	CATGCACACAACACCATG	96	55	0	0	0	0
RRM2B	ACACACGTTGTGCAACTG	1700	383	2	1	1	1
RRM2B	ACACCATGACCACATGAC	40	33	1	0	0	0
RRM2B	CAACACGTTGTGCATGGT	452	331	0	0	0	0
RRM2B	ACACACGTTGTGCATGCA	95	100	0	0	0	0
RRM2B	GTACACGTTGTGCATGAC	66	34	0	0	0	0
RSAD2	GTACACTGCATGTGCACA	15	40	0	0	0	0
RSAD2	ACACACTGCATGTGCAAC	23	24	0	0	0	0
RSAD2	CAACACTGCATGTGCATG	382	341	1	0	0	0
RSAD2	GTACACTGCATGTGACGT	112	60	0	0	0	0
RSAD2	CAACACTGCATGTGACCA	131	174	0	0	0	0
RTF1	TGACGTTGTGCAACTGCA	208	107	0	0	0	0
RTF1	CAACGTTGTGCAACTGAC	64	101	0	0	0	0
RTF1	GTACGTTGTGCAACTGTG	77	57	0	0	0	1
RTF1	ACACGTTGTGCATGGTGT	196	682	0	0	0	0
RTF1	TGACGTTGTGCATGGTCA	165	120	0	0	0	0
RTKN	TGACACCACATGGTCAAC	40	28	0	0	0	0
RTKN	ACACACCACATGGTCATG	123	70	0	0	0	0
RTKN	CAACACCACATGGTACGT	242	213	0	0	0	0
RTKN	ACACACCACATGGTACCA	766	445	2	0	0	0
RTKN	GTTGACCAGTGTTGACGT	181	181	1	0	0	0
RTN4	TGACTGACTGACCAGTCA	132	154	0	0	0	0
RTN4	CATGGTTGACGTCAACGT	121	153	1	0	0	0
RTN4	ACTGGTTGACGTCAACCA	88	82	0	0	0	2
RTN4	GTTGGTTGACGTCAACAC	15	11	0	0	0	0
RTN4	TGTGGTTGACGTCAACTG	263	359	0	0	0	1
RTN4R	GTACTGCATGGTCAGTGT	450	305	0	0	0	1
RTN4R	CAACTGCATGGTCAGTCA	103	133	0	0	0	0
RTN4R	TGACTGCATGGTCAGTAC	58	87	0	1	0	0
RTN4R	ACACTGCATGGTCAGTTG	747	289	0	0	0	0
RTN4R	CATGGTTGTGACCAACGT	56	98	1	0	0	37
RUNX1	ACACGTCATGGTACGTGT	42	66	0	0	0	0
RUNX1	TGACGTCATGGTACGTCA	30	78	0	0	0	1
RUNX1	CAACGTCATGGTACGTAC	35	209	0	0	0	0
RUNX1	CATGACGTCATGGTGTTG	248	347	0	0	0	0
RUNX1	GTTGACGTCATGGTCAGT	98	89	0	0	0	0
RUNX2	TGACGTCATGGTCAACTG	64	47	0	0	0	0
RUNX2	ACACGTCATGGTCATGGT	893	873	0	0	0	0
RUNX2	TGACGTCATGGTCATGCA	116	142	0	0	0	0
RUNX2	CAACGTCATGGTCATGAC	29	37	0	0	0	0
RUNX2	GTACGTCATGGTCATGTG	104	63	0	0	0	0
RUNX3	GTACGTCATGGTACCACA	136	148	3	2	7	5
RUNX3	GTACGTCATGGTACGTTG	349	185	3	0	0	0
RUNX3	TGACGTCATGGTACCAGT	99	134	0	0	0	0
RUNX3	ACACGTCATGGTACCAAC	24	40	0	0	0	0
RUNX3	ACTGGTGTGTTGCACATG	155	310	0	0	0	0
RUVBL1	TGACGTACTGGTGTACGT	314	187	0	0	0	1
RUVBL1	GTACGTACTGGTGTACCA	56	33	0	0	0	0
RUVBL1	ACACGTACTGGTGTACAC	2	2	0	0	0	0
RUVBL1	GTTGGTGTACCAACCACA	21	5	0	0	0	0
RUVBL1	ACTGACCAACTGCAGTCA	404	433	0	0	0	0
RUVBL2	CAACACACACGTGTGTCA	680	838	7	0	3	8
RUVBL2	ACACACACCATGTGTGTG	15	10	0	0	0	0
RUVBL2	GTACACACACGTGTGTGT	79	77	0	0	0	0
RUVBL2	TGACACACACGTGTGTAC	6	9	0	0	0	0
RUVBL2	ACACACACACGTGTGTTG	299	251	0	0	0	0
RUVBL2	CATGCACAACCACACACA	26	15	0	0	0	0
RXFP1	CAACGTCACATGACGTTG	49	87	0	0	0	0
RXFP1	GTACGTCACATGACCAGT	144	130	0	0	0	0
RXFP1	CAACGTCACATGACCACA	56	62	0	0	0	0
RXFP1	TGACGTCACATGACCAAC	50	69	0	0	0	0
RXFP1	ACACGTCACATGACCATG	139	89	2	0	0	0
RXRA	TGACGTTGACCATGTGCA	491	246	0	0	1	0
RXRA	ACACGTTGACCATGACCA	406	788	1	1	0	1
RXRA	GTACGTTGACCATGACAC	54	56	0	0	0	0

RXRA	TGACGTTGACCATGACTG	300	423	2	0	0	0
RXRA	ACACGTTGACCATGTGGT	772	545	1	0	0	1
RXRA	GTTGCAACACCAGTCATG	158	106	0	0	0	0
RXRB	ACACGTTGACACGTGTTG	371	471	1	0	0	3
RXRB	CAACGTTGACACGTCAGT	308	345	0	0	0	0
RXRB	ACACGTTGACACGTCACA	107	140	0	0	0	0
RXRB	GTACGTTGACACGTCAAC	8	15	0	0	0	0
RXRB	TGACGTTGACACGTCATG	32	91	0	0	0	0
RXRB	ACTGCAACTGTGACACAC	43	61	0	0	0	0
RXRG	ACTGACGTTGACCATGCA	501	591	0	0	0	1
RXRG	GTTGACGTTGACCATGAC	175	82	0	1	0	0
RXRG	TGTGACGTTGACCATGTG	695	47	2	0	0	1
RXRG	CATGACGTTGACACGTGT	643	623	6	0	0	0
RXRG	ACTGACGTTGACACGTCA	49	77	0	0	0	0
S100A12	TGACACTGGTACCAGTCA	1097	435	1	12	0	8
S100A12	CAACACTGGTACCAGTAC	49	41	0	0	0	0
S100A12	GTACACTGGTACCAGTTG	203	227	0	1	0	0
S100A12	TGACACTGGTACCACAGT	94	138	2	0	0	0
S100A12	ACTGCAGTTGCAACTGAC	25	24	0	0	0	0
S100A12	GTTGCACAACGTCACAAC	9	9	0	0	0	0
S100A4	ACACGTCAACCACAGTAC	122	80	0	0	0	0
S100A4	TGACACTGGTCACAGTAC	56	48	0	0	0	0
S100A4	ACACACTGGTCACAGTTG	679	688	0	0	0	0
S100A4	CAACACTGGTCACACAGT	340	90	1	0	0	1
S100A4	ACACACTGGTCACACACA	78	96	0	0	0	0
S100A6	GTACACGTCAGTGTCAAC	26	19	0	1	0	0
S100A6	TGACACGTCAGTGTCATG	24	36	0	1	0	0
S100A6	ACACACGTCAGTGTACGT	56	210	0	0	0	0
S100A6	TGACACGTCAGTGTACCA	46	214	0	1	0	0
S100A6	GTTGGTGTGTTGACACGT	99	101	0	0	0	0
S100A8	ACACACTGGTCATGGTGT	884	1153	1	1	2	3
S100A8	TGACACTGGTCAACTGCA	28	61	1	4	0	1
S100A8	CAACACTGGTCAACTGAC	78	92	0	1	0	0
S100A8	GTACACTGGTCAACTGTG	735	399	0	1	0	0
S100A8	TGACACTGGTCATGGTCA	288	465	0	1	0	0
S100A8	GTTGCAACACCAACCAGT	287	87	1	0	0	0
S100A9	TGACACTGGTCATGTGTG	356	456	0	0	1	0
S100A9	ACACACTGGTCATGACTG	245	267	0	0	0	0
S100A9	CAACACTGGTCATGTGGT	211	218	1	0	0	0
S100A9	ACACACTGGTCATGTGCA	152	137	0	0	0	0
S100A9	GTACACTGGTCATGTGAC	80	98	1	0	0	0
S100A9	ACTGCACATGGTTGACAC	89	97	1	0	0	0
S100B	ACACACTGGTACTGGTTG	1016	1257	2	0	0	0
S100B	CAACACTGGTACTGCAGT	152	214	0	0	0	0
S100B	ACACACTGGTACTGCACA	521	402	0	1	0	1
S100B	GTACACTGGTACTGCAAC	11	11	0	0	0	14
S100B	CATGCAGTTGCATGACGT	223	192	0	0	0	0
S100B	TGTGCACAACCAGTGTAC	55	66	0	0	0	0
SAA1	CAACTGTGTGGTTGGTCA	87	123	0	0	0	0
SAA1	TGACTGTGTGGTTGGTAC	74	62	0	0	0	0
SAA1	ACACTGTGTGGTTGGTTG	200	530	0	1	0	0
SAA1	CAACTGTGTGGTTGCAGT	105	92	0	0	0	0
SAA1	ACTGGTACCACATGACAC	82	84	0	0	0	0
SALL1	TGCATGTGGTACCATGCA	63	37	0	0	0	0
SALL1	CACATGTGGTACCATGAC	52	58	0	0	0	0
SALL1	GTCATGTGGTACCATGTG	705	927	2	0	0	0
SALL1	ACCATGTGGTACACGTGT	493	183	0	0	0	2
SALL1	TGCATGTGGTACACGTCA	283	372	0	2	0	1
SALL4	GTACGTTGACTGCATGCA	558	762	112	55	81	4080
SALL4	CAACGTTGACTGCAACAC	42	52	0	0	0	0
SALL4	GTACGTTGACTGCAACTG	573	475	2	0	0	0
SALL4	TGACGTTGACTGCATGGT	467	416	0	0	0	0
SALL4	ACACGTTGACTGCATGAC	60	56	0	0	0	0
SAP18	ACACGTTGACACCACATG	162	149	0	0	0	0
SAP18	CAACGTTGACACCAACGT	172	148	0	1	0	0
SAP18	TGTGCAGTCACAGTACGT	276	224	0	1	0	1
SAP18	GTTGCAGTCACAGTACCA	152	139	0	0	0	0
SAP18	ACTGCAGTCACAGTACAC	276	98	0	0	0	0
SAP18	TGTGCAACTGGTACACTG	141	331	0	1	0	0
SARM1	ACTGGTCATGGTACACCA	193	263	0	0	1	1
SARM1	CATGGTCATGGTACCACA	88	79	0	1	0	0
SARM1	TGTGGTCATGGTACCAAC	16	12	0	0	0	0
SARM1	ACTGGTCATGGTACCATG	119	396	0	0	0	0
SARM1	CATGGTCATGGTACACGT	241	163	1	0	0	0
SARS	ACACACGTCAACTGACTG	484	263	0	0	0	0

SARS	CAACACGTCAACTGTGGT	667	872	2	44	0	0
SARS	ACACACGTCAACTGTGCA	15	41	0	0	0	0
SARS	GTACACGTCAACTGTGAC	37	43	0	0	0	0
SARS	TGACACGTCAACTGTGTG	594	291	0	0	0	13
SARS	TGTGCAACGTACCAGTGT	136	126	0	0	0	1
SAT1	ACACCACATGGTGTGTAC	27	53	0	0	0	0
SAT1	CAACCACATGGTGTGTTG	41	65	0	0	0	0
SAT1	GTACCACATGGTGTCAGT	201	377	0	4	0	0
SAT1	CAACCACATGGTGTCACA	150	184	0	1	0	1
SAT1	TGACCACATGGTGTCAAC	24	14	0	0	0	0
SAT1	CATGCACACAGTACACTG	202	146	0	0	0	0
SC4MOL	TGTGGTTGTGGTGTGTGT	555	435	0	0	1	0
SC4MOL	TGACACGTTGCACACAAC	22	32	0	0	0	1
SC4MOL	ACACACGTTGCACACATG	93	20	0	0	0	0
SC4MOL	CAACACGTTGCACAACGT	64	75	0	0	0	0
SC4MOL	ACACACGTTGCACAACCA	391	337	0	0	0	1
SC5DL	TGACTGACACTGTGCAGT	199	341	0	4	1	0
SC5DL	CAACTGACACTGTGCATG	184	386	0	0	1	2
SC5DL	GTACTGACACTGTGCACA	179	54	0	0	0	0
SC5DL	ACACTGACACTGTGCAAC	9	14	0	0	0	0
SC5DL	GTACTGACACTGTGACGT	121	133	0	0	0	1
SCAP	ACACTGTGCAACCACAAC	14	22	0	0	0	0
SCAP	CAACTGTGCAACCACATG	218	233	0	1	0	6
SCAP	GTACTGTGCAACCAACGT	224	338	0	0	0	0
SCAP	CAACTGTGCAACCAACCA	75	87	0	0	0	0
SCAP	TGACTGTGCAACCAACAC	28	39	0	0	0	0
SCARB1	GTACACTGACACCACATG	685	483	0	3	1	0
SCARB1	ACACACTGACACCACAGT	330	709	0	3	0	0
SCARB1	TGACACTGACACCACACA	14	55	0	0	0	0
SCARB1	CAACACTGACACCACAAC	83	95	0	1	0	1
SCARB1	TGACACTGACACCAACGT	383	352	0	1	0	1
SCD	ACACACTGCATGACGTAC	287	46	0	0	0	0
SCD	CAACACTGCATGACGTTG	736	200	0	0	0	0
SCD	GTACACTGCATGACCAGT	246	371	0	0	0	0
SCD	CAACACTGCATGACCACA	34	84	0	0	0	0
SCD	CATGGTGTACCACAACGT	352	253	1	1	0	0
SCD5	GTACACTGCATGTGGTTG	1237	786	35	0	1	0
SCD5	TGACACTGCATGTGGTCA	154	240	0	0	0	0
SCD5	CAACACTGCATGTGGTAC	11	19	0	0	0	0
SCD5	TGACACTGCATGTGCAGT	144	156	0	0	0	0
SCD5	TGTGACACACTGACCACA	201	333	0	0	0	1
SCG2	CAACACTGCAGTGTGTTG	143	219	1	0	0	0
SCG2	GTACACTGCAGTGTCAGT	154	165	0	0	0	0
SCG2	CAACACTGCAGTGTCACA	54	45	0	0	0	0
SCG2	TGACACTGCAGTGTCAAC	3	3	0	0	0	0
SCG2	TGTGCAGTTGCACAACGT	171	129	0	0	0	0
SCLY	GTACTGTGCATGACCACA	171	37	0	0	1	0
SCLY	GTACTGTGCATGACGTTG	773	855	1	1	0	1
SCLY	TGACTGTGCATGACCAGT	76	87	0	0	0	0
SCLY	ACACTGTGCATGACCAAC	57	66	0	0	0	0
SCLY	CAACTGTGCATGACCATG	283	128	0	0	0	0
SCN10A	GTACACGTGTCAGTGTTG	478	531	1	0	0	0
SCN10A	TGACACGTGTCAGTCAGT	78	61	0	0	0	0
SCN10A	GTACACGTGTCAGTCACA	200	29	1	0	0	0
SCN10A	ACACACGTGTCAGTCAAC	56	62	0	0	0	0
SCN10A	CAACACGTGTCAGTCATG	351	133	0	0	0	0
SCN11A	GTACACGTGTCACATGGT	262	267	1	0	0	0
SCN11A	CAACACGTGTCACATGCA	96	95	0	0	0	0
SCN11A	TGACACGTGTCACATGAC	33	61	2	0	0	0
SCN11A	ACACACGTGTCACATGTG	162	163	0	0	0	0
SCN11A	GTACACGTGTCAACGTGT	161	164	0	0	0	1
SCN1A	TGACCATGTGTGACTGAC	79	53	0	0	0	0
SCN1A	ACACCATGTGTGACTGTG	123	70	0	0	0	0
SCN1A	GTACCATGTGTGTGGTGT	271	265	0	1	0	1
SCN1A	CAACCATGTGTGTGGTCA	383	246	1	1	0	0
SCN1A	ACTGGTACTGCAACACCA	235	130	0	0	0	0
SCN1B	CAACCATGTGTGTGTGTG	152	307	0	0	1	0
SCN1B	TGACCATGTGTGTGTGGT	298	537	0	0	0	1
SCN1B	GTACCATGTGTGTGTGCA	114	113	0	0	0	0
SCN1B	ACACCATGTGTGTGTGAC	38	21	0	0	0	0
SCN1B	TGACACGTGTGTGTGTGT	95	159	2	1	0	0
SCN2A	TGACACGTGTGTGTACTG	13	27	0	0	0	0
SCN2A	ACACACGTGTGTGTTGGT	38	47	0	0	0	0
SCN2A	TGACACGTGTGTGTTGCA	42	45	0	0	0	0
SCN2A	CAACACGTGTGTGTTGAC	209	17	0	0	0	3

SCN2A	GTACACGTGTGTGTTGTG	80	67	0	0	0	0
SCN2B	CATGCACAGTGTTGGTTG	123	108	0	0	1	0
SCN2B	TGACACGTGTGTCACAGT	82	128	0	0	0	0
SCN2B	GTACACGTGTGTCACACA	109	120	0	0	0	0
SCN2B	ACACACGTGTGTCACAAC	44	48	0	0	0	0
SCN2B	CATGCAGTTGGTGTTGAC	56	56	1	0	0	0
SCN3A	GTTGGTTGACGTCATGTG	175	206	0	0	1	0
SCN3A	ACTGGTTGACGTACGTGT	341	234	0	1	1	0
SCN3A	ACTGGTTGACGTCATGGT	524	365	1	1	0	0
SCN3A	TGTGGTTGACGTCATGCA	341	620	0	1	0	0
SCN3A	CATGGTTGACGTCATGAC	20	37	0	0	0	0
SCN3B	CAACACGTGTCAACGTCA	364	125	0	0	1	0
SCN3B	TGACACGTGTCAACGTAC	9	17	0	0	0	0
SCN3B	ACACACGTGTCAACGTTG	230	475	0	1	0	1
SCN3B	CAACACGTGTCAACCAGT	158	204	1	1	0	0
SCN3B	CATGACCATGACCAGTTG	1530	1289	0	2	0	0
SCN4A	ACACACGTGTGTCAACTG	174	257	0	0	0	0
SCN4A	CAACACGTGTGTCATGGT	524	516	0	4	0	1
SCN4A	ACACACGTGTGTCATGCA	431	377	1	0	0	4
SCN4A	GTACACGTGTGTCATGAC	40	59	0	0	0	0
SCN4A	CATGACCATGACCACACA	82	69	0	0	0	0
SCN4B	TGACACGTGTGTACGTTG	455	207	2	1	0	0
SCN4B	ACACACGTGTGTACCAGT	138	181	0	0	0	1
SCN4B	TGACACGTGTGTACCACA	185	34	0	0	0	0
SCN4B	CAACACGTGTGTACCAAC	8	14	0	0	0	0
SCN4B	GTACACGTGTGTACCATG	239	87	0	0	0	0
SCN5A	CAACCATGTGACCAGTGT	1964	1091	2	0	0	1
SCN5A	ACACCATGTGACCAGTCA	332	412	1	0	0	1
SCN5A	GTACCATGTGACCAGTAC	22	37	1	0	0	0
SCN5A	ACTGACCAACTGGTACTG	995	580	3	0	0	0
SCN5A	CATGACCAACTGGTTGGT	295	221	0	0	0	0
SCN7A	CAACACGTGTGTACTGCA	655	202	1	941	82	0
SCN7A	CAACACGTGTGTACACTG	701	648	0	0	0	0
SCN7A	GTACACGTGTGTACTGGT	313	194	0	0	0	0
SCN7A	TGACACGTGTGTACTGAC	76	39	0	1	0	0
SCN7A	ACACACGTGTGTACTGTG	91	102	2	0	0	0
SCN8A	GTACACGTGTGTTGGTGT	1199	775	0	0	0	1
SCN8A	CAACACGTGTGTTGGTCA	152	140	0	0	0	0
SCN8A	TGACACGTGTGTTGGTAC	24	17	0	0	0	0
SCN8A	ACACACGTGTGTTGGTTG	593	406	4	0	0	2
SCN8A	CAACACGTGTGTTGCAGT	185	188	1	0	0	0
SCN8A	GTTGCACAACTGGTACAC	37	25	0	0	0	1
SCN9A	CAACACGTGTGTTGACAC	25	18	0	0	0	0
SCN9A	GTACACGTGTGTTGACTG	92	86	0	0	0	0
SCN9A	TGACACGTGTGTTGTGGT	71	90	0	0	0	0
SCN9A	GTACACGTGTGTTGTGCA	87	152	0	0	0	0
SCN9A	GTTGGTACTGCAACCAGT	556	561	49	0	0	1
SCN9A	GTTGCACACAGTACTGGT	635	484	1	1	0	1
SCNN1A	ACACACGTGTCAGTTGCA	503	165	0	0	1	0
SCNN1A	CAACACGTGTCACAGTGT	882	882	3	0	1	0
SCNN1A	GTACACGTGTCAGTTGAC	11	81	0	0	0	0
SCNN1A	TGACACGTGTCAGTTGTG	70	190	0	0	0	0
SCNN1A	ACACACGTGTCACAGTCA	347	748	2	1	0	20
SCNN1B	CAACCATGTGACCATGCA	691	629	1	0	0	9
SCNN1B	TGACCATGTGACCATGAC	107	102	0	0	0	0
SCNN1B	ACACCATGTGACCATGTG	545	495	1	0	0	0
SCNN1B	GTACCATGTGACACGTGT	631	384	1	0	0	0
SCNN1B	CAACCATGTGACACGTCA	370	432	0	0	0	0
SCNN1D	TGACACGTGTCACAGTTG	677	790	1	1	1	1
SCNN1D	GTACACGTGTCACAGTAC	35	50	0	0	0	0
SCNN1D	ACACACGTGTCACACAGT	27	55	0	0	0	0
SCNN1D	TGACACGTGTCACACACA	63	102	0	1	0	0
SCNN1D	CAACACGTGTCACACAAC	49	43	0	0	0	0
SCP2	TGTGACGTTGGTCAGTTG	661	965	3	2	3	0
SCP2	ACTGGTACGTGTGTGTTG	236	183	0	0	0	14
SCP2	ACTGACGTTGGTCAGTCA	191	244	1	0	0	0
SCP2	GTTGACGTTGGTCAGTAC	47	36	0	0	0	0
SCP2	ACTGACGTTGGTCACAGT	231	370	1	1	0	1
SCTR	CAACGTACGTGTCACATG	38	32	0	0	0	0
SCTR	GTACGTACGTGTCAACGT	34	36	0	0	0	0
SCTR	CAACGTACGTGTCAACCA	469	198	2	1	0	6
SCTR	TGACGTACGTGTCAACAC	77	35	0	0	0	0
SCTR	ACACGTACGTGTCAACTG	84	92	0	0	0	0
SCYL1	GTACGTGTCAACACCATG	110	193	0	0	2	1
SCYL1	TGACGTGTCAACACGTTG	535	802	0	0	0	0

SCYL1	ACACGTGTCAACACCAGT	287	342	1	0	0	0
SCYL1	TGACGTGTCAACACCACA	272	188	1	0	0	0
SCYL1	CAACGTGTCAACACCAAC	96	150	0	0	0	0
SDC2	ACTGGTCACACACAGTGT	1082	777	2	1	0	7
SDC2	TGTGGTCACACACAGTCA	60	85	0	0	0	0
SDC2	CATGGTCACACACAGTAC	15	30	0	0	0	0
SDC2	GTTGGTCACACACAGTTG	461	460	0	0	0	0
SDC2	TGTGCACAGTGTTGGTGT	601	588	5	0	0	3
SDC3	GTTGGTCAGTGTTGGTGT	1229	1229	2	1	1	138
SDC3	TGTGACCAACACTGTGTG	1362	353	0	1	1	0
SDC3	ACTGGTCAGTGTACTGTG	435	370	0	0	0	0
SDC3	CATGGTCAGTGTTGGTCA	965	861	2	0	0	0
SDC3	ACTGACCAACTGGTGTGT	241	190	2	0	0	0
SDHA	CAACGTTGTGTGCACACA	181	215	0	0	0	0
SDHA	TGACCAGTGTGTGTTGGT	291	699	4	394	0	1
SDHA	GTACCAGTGTGTACTGAC	25	17	0	0	0	0
SDHA	TGACCAGTGTGTTGTGAC	51	62	0	0	0	1
SDHA	ACACCAGTGTGTTGTGTG	309	291	1	0	0	0
SDHA	TGTGCACACATGCATGGT	146	147	0	0	0	0
SDHB	TGACCAGTGTGTACTGTG	504	382	0	0	0	0
SDHB	CAACCAGTGTGTTGGTGT	520	295	3	1	0	3
SDHB	ACACCAGTGTGTTGGTCA	147	349	0	0	0	2
SDHB	GTACCAGTGTGTTGGTAC	112	51	0	0	0	0
SDHB	TGACCAGTGTGTTGGTTG	415	500	0	0	0	0
SDHC	CAACCAGTGTGTACCATG	557	267	14	19	8	29207
SDHC	GTACCAGTGTGTACACGT	39	20	0	0	0	0
SDHC	TGTGCAGTCATGCAGTGT	267	253	1	0	0	2
SDHC	GTTGCAGTCATGCAGTCA	130	135	0	2	0	0
SDHC	CATGACCAACGTACTGGT	644	599	0	0	0	100
SDHD	ACACCAACACGTTGCAAC	7	10	0	0	0	0
SDHD	CAACCAACACGTTGCATG	148	219	1	0	0	1
SDHD	GTACCAACACGTTGACGT	267	180	0	0	0	0
SDHD	CAACCAACACGTTGACCA	182	222	0	0	0	0
SDHD	TGACCAACACGTTGACAC	104	103	0	0	0	0
SDHD	CATGCACACAGTTGGTCA	207	157	1	0	0	0
SDS	CACATGTGACGTCAGTGT	427	649	0	0	0	0
SDS	ACCATGTGACGTCAGTCA	252	306	0	0	0	0
SDS	GTCATGTGACGTCAGTAC	41	39	1	0	0	0
SDS	TGCATGTGACGTCAGTTG	763	644	2	0	0	0
SDS	ACCATGTGACGTCACAGT	90	242	0	0	0	0
SEC22B	GTTGGTACACGTACGTAC	53	89	0	0	0	2
SEC22B	TGTGGTACACGTACGTTG	291	179	1	1	0	0
SEC22B	ACTGGTACACGTACCAGT	128	33	0	0	0	0
SEC22B	TGTGGTTGTGACCACAAC	2	4	0	0	0	0
SEC22B	ACTGGTTGTGACCACATG	83	117	2	0	0	0
SEC61A2	GTTGGTTGGTTGGTTGTG	988	1010	3	1	2	44
SEC61A2	TGTGCAACGTGTTGTGGT	467	392	2	0	1	0
SEC61A2	TGTGGTTGGTTGGTTGCA	509	380	3	1	0	0
SEC61A2	CATGGTTGGTTGGTTGAC	128	139	0	0	0	0
SEC61A2	ACTGGTTGGTTGCAGTGT	434	167	0	0	0	0
SEC61A2	TGTGGTTGGTTGCAGTCA	640	815	19	1	0	2
SEL1L	CATGGTTGGTTGGTACGT	100	120	0	0	0	0
SEL1L	ACTGGTTGGTTGGTACCA	165	98	1	0	0	0
SEL1L	GTTGGTTGGTTGGTACAC	40	35	0	0	0	0
SEL1L	TGTGGTTGGTTGGTACTG	56	168	0	0	0	0
SEL1L	ACTGGTTGGTTGGTTGGT	310	465	0	0	0	0
SEL1L	TGTGCAACCACAACCACA	44	43	0	0	0	0
SELE	CAACTGGTGTTGCAGTTG	205	185	2	0	0	0
SELE	GTACTGGTGTTGCACAGT	105	90	0	0	0	0
SELE	CAACTGGTGTTGCACACA	323	207	0	0	0	0
SELE	TGACTGGTGTTGCACAAC	3	10	0	0	0	0
SELE	ACACTGGTGTTGCACATG	240	151	0	1	0	0
SELE	TGTGCACACAGTTGGTAC	91	67	0	0	0	0
SELL	CAACTGGTGTTGCAACGT	100	116	0	0	0	0
SELL	ACACTGGTGTTGCAACCA	261	201	0	0	0	0
SELL	GTACTGGTGTTGCAACAC	58	78	0	0	0	0
SELL	TGACTGGTGTTGCAACTG	149	229	0	0	0	1
SELL	ACACTGGTGTTGCATGGT	401	507	0	0	0	1
SELP	TGACTGGTGTGTCAGTTG	116	91	0	0	0	0
SELP	ACACTGGTGTGTCACAGT	196	318	0	0	0	0
SELP	TGACTGGTGTGTCACACA	44	34	0	0	0	0
SELP	CAACTGGTGTGTCACAAC	5	11	0	0	0	0
SELP	GTACTGGTGTGTCACATG	690	247	1	1	0	0
SELP	GTTGCACAACCATGCATG	574	588	1	1	0	0
SELPLG	ACACACTGACACACTGAC	44	11	0	0	0	0

SELPLG	CAACACTGACACACTGTG	846	795	5	1	0	2
SELPLG	TGACACTGACACTGGTGT	1041	517	0	0	0	1
SELPLG	GTACACTGACACTGGTCA	472	244	2	0	0	0
SELPLG	ACACACTGACACTGGTAC	27	29	0	0	0	0
SEMA3A	GTACTGGTCAACTGACCA	119	145	1	1	0	0
SEMA3A	ACACTGGTCAACTGACAC	41	145	0	1	0	0
SEMA3A	CAACTGGTCAACTGACTG	911	682	2	0	0	6
SEMA3A	GTACTGGTCAACTGTGGT	876	297	0	0	0	0
SEMA3A	CAACTGACTGCAACTGAC	69	76	0	0	0	1
SEMA4A	ACACTGGTCAACTGCAGT	402	380	1	0	1	0
SEMA4A	TGACTGGTCAACTGCACA	398	240	0	0	0	1
SEMA4A	CAACTGGTCAACTGCAAC	35	32	0	0	0	0
SEMA4A	GTACTGGTCAACTGCATG	88	84	0	0	0	0
SEMA4A	TGACTGGTCAACTGACGT	534	126	0	0	0	0
SEMA4A	TGTGCAACACGTACCATG	488	723	0	0	0	1
SEMA7A	TGACTGGTCAACACTGTG	353	518	5	2810	0	0
SEMA7A	CAACTGGTCAACTGGTGT	958	1233	1	1	0	22
SEMA7A	ACACTGGTCAACTGGTCA	677	487	0	0	0	0
SEMA7A	GTACTGGTCAACTGGTAC	33	32	0	0	0	0
SEMA7A	TGACTGGTCAACTGGTTG	1063	364	1	0	0	1
SENP2	CACATGTGCACACAGTAC	30	58	0	0	0	0
SENP2	GTCATGTGCACACAGTTG	213	240	0	0	0	0
SENP2	TGCATGTGCACACACAGT	185	111	0	0	0	1
SENP2	GTCATGTGCACACACACA	342	276	1	0	0	0
SENP2	ACCATGTGCACACACAAC	18	38	0	0	0	0
SEPHS1	ACACACGTACACGTTGGT	149	190	0	0	0	0
SEPHS1	TGACACGTACACGTTGCA	138	164	0	0	0	0
SEPHS1	CAACACGTACACGTTGAC	42	35	0	0	0	0
SEPHS1	GTACACGTACACGTTGTG	111	65	0	0	0	0
SEPHS1	ACACACGTACACCAGTGT	331	278	0	0	0	18
SEPP1	ACTGGTACGTGTGTACGT	78	128	0	0	0	0
SEPP1	TGTGGTACGTGTGTACCA	140	310	0	1	0	2
SEPP1	CATGGTACGTGTGTACAC	43	19	0	0	0	0
SEPP1	GTTGGTACGTGTGTACTG	283	297	0	2	0	1
SEPP1	TGTGGTACGTGTGTTGGT	275	297	0	0	0	0
SERPINA1	GTACCAGTTGGTCATGGT	909	325	1	2	0	6
SERPINA1	CAACCAGTTGGTCATGCA	245	202	1	0	0	0
SERPINA1	TGACCAGTTGGTCATGAC	65	140	0	0	0	0
SERPINA1	ACACCAGTTGGTCATGTG	249	211	0	0	0	0
SERPINA1	GTACCAGTTGGTACGTGT	263	270	1	0	0	0
SERPINA10	ACACACACACTGCACAGT	664	307	1	0	1	0
SERPINA10	TGACACACACTGCAGTTG	118	126	0	0	0	0
SERPINA10	TGACACACACTGCACACA	46	34	0	0	0	0
SERPINA10	CAACACACACTGCACAAC	8	8	0	0	0	0
SERPINA10	GTACACACACTGCACATG	137	473	1	0	0	0
SERPINA3	TGACGTGTGTTGTGGTAC	395	94	0	0	0	0
SERPINA3	ACACGTGTGTTGTGGTTG	337	439	1	0	0	1
SERPINA3	CAACGTGTGTTGTGCAGT	527	263	2	0	0	1
SERPINA3	TGTGACCACAACTGACCA	733	624	1	1	0	0
SERPINA3	CATGACCACAACTGACAC	80	229	1	0	0	0
SERPINA4	CAACTGACACTGCACAGT	331	767	0	2	1	0
SERPINA4	ACACTGACACTGCAGTTG	502	392	1	1	0	0
SERPINA4	ACACTGACACTGCACACA	135	128	0	0	0	0
SERPINA4	GTACTGACACTGCACAAC	14	14	0	0	0	0
SERPINA4	TGACTGACACTGCACATG	273	767	0	0	0	12
SERPINA4	GTTGCAACACGTCAGTAC	130	78	0	0	0	0
SERPINA5	CAACACACACTGACGTCA	430	283	0	0	0	0
SERPINA5	TGACACACACTGACGTAC	53	49	0	0	0	0
SERPINA5	ACACACACACTGACGTTG	124	143	0	0	0	0
SERPINA5	CAACACACACTGACCAGT	183	129	0	0	0	0
SERPINA5	CATGCAGTTGCAACACAC	45	54	0	0	0	0
SERPINA6	GTCATGACTGACCAGTGT	343	280	0	0	0	0
SERPINA6	CACATGACTGACCAGTCA	313	171	0	0	0	0
SERPINA6	TGCATGACTGACCAGTAC	21	77	0	1	0	3
SERPINA6	ACCATGACTGACCAGTTG	1283	966	3	1	0	1
SERPINA6	ACTGCAGTACGTTGACAC	32	29	0	0	0	0
SERPINB2	GTACACACACTGACGTGT	232	213	1	0	1	1
SERPINB2	GTACACACACTGCATGGT	470	565	0	0	0	0
SERPINB2	CAACACACACTGCATGCA	639	238	0	2	0	0
SERPINB2	TGACACACACTGCATGAC	30	19	0	0	0	0
SERPINB2	ACACACACACTGCATGTG	510	527	0	0	0	0
SERPINB2	ACTGCACACAGTGTGTAC	21	123	1	0	0	0
SERPINB5	ACACACACACTGACACGT	381	145	0	0	1	0
SERPINB5	ACACACACACTGACCACA	95	119	0	0	0	1
SERPINB5	GTACACACACTGACCAAC	33	46	0	0	0	0

SERPINB5	TGACACACACTGACCATG	342	361	0	0	0	0
SERPINB5	TGACACACACTGACACCA	365	418	1	0	0	0
SERPINB9	CAACACACACTGACTGTG	63	59	0	0	0	0
SERPINB9	TGACACACACTGTGGTGT	319	232	0	0	0	1
SERPINB9	GTACACACACTGTGGTCA	976	855	0	1	0	1
SERPINB9	ACACACACACTGTGGTAC	49	31	0	0	0	0
SERPINB9	GTTGCAGTTGCAACTGCA	33	34	0	0	0	0
SERPINC1	TGACGTGTCAGTCAGTTG	435	646	8	6	0	5
SERPINC1	ACACGTGTCAGTCACAGT	427	265	0	1	0	1
SERPINC1	TGACGTGTCAGTCACACA	38	49	0	0	0	0
SERPINC1	CAACGTGTCAGTCACAAC	19	40	0	0	0	0
SERPINC1	GTACGTGTCAGTCACATG	292	335	1	0	0	0
SERPIND1	GTACACACACTGGTTGAC	19	27	0	0	0	0
SERPIND1	TGACACACACTGGTTGTG	348	283	0	0	0	0
SERPIND1	CAACACACACTGCAGTGT	639	671	5	0	0	1
SERPIND1	ACACACACACTGCAGTCA	447	302	0	0	0	0
SERPIND1	GTACACACACTGCAGTAC	83	80	0	0	0	1
SERPINE1	CAACACACACTGCAACTG	1106	1433	2	0	1	32
SERPINE1	TGACACACACTGCAACGT	492	510	1	0	0	1
SERPINE1	GTACACACACTGCAACCA	586	568	4	0	0	2
SERPINE1	ACACACACACTGCAACAC	56	78	0	0	0	0
SERPINE1	CATGCAGTTGCACATGCA	351	371	0	1	0	0
SERPINE1	TGTGCACATGCAACGTTG	1216	1096	2	0	0	1
SERPINE2	TGACACACACTGACTGGT	318	118	1	0	1	0
SERPINE2	CAACACACACTGACACAC	195	89	0	0	0	0
SERPINE2	GTACACACACTGACACTG	300	218	1	0	0	0
SERPINE2	GTACACACACTGACTGCA	120	112	0	0	0	0
SERPINE2	ACACACACACTGACTGAC	41	54	0	0	0	0
SERPINF2	CAACACACACTGTGACGT	207	213	36	0	0	2
SERPINF2	ACACACACACTGTGACCA	274	390	0	0	0	0
SERPINF2	GTACACACACTGTGACAC	26	27	0	0	0	0
SERPINF2	TGACACACACTGTGACTG	377	549	0	0	0	0
SERPINF2	ACACACACACTGTGTGGT	150	204	0	0	0	0
SERPING1	TGCATGACTGGTTGACGT	296	348	4	11	6	16
SERPING1	CACATGACTGGTTGCAAC	2	49	0	0	0	0
SERPING1	GTCATGACTGGTTGCATG	161	183	0	0	0	0
SERPING1	GTCATGACTGGTTGACCA	386	217	1	0	0	25
SERPING1	GTTGCAGTACGTTGGTAC	100	152	0	0	0	0
SERPINI1	CAACACACACTGTGGTTG	1089	759	0	21	0	0
SERPINI1	GTACACACACTGTGCAGT	73	88	0	0	0	0
SERPINI1	CAACACACACTGTGCACA	97	168	1	0	0	0
SERPINI1	TGACACACACTGTGCAAC	15	65	0	0	0	0
SERPINI1	ACACACACACTGTGCATG	167	334	0	0	0	2
SESN1	TGTGGTCATGGTTGCAGT	214	203	0	0	1	0
SESN1	GTTGGTCATGGTTGGTTG	408	587	1	0	0	400
SESN1	GTTGGTCATGGTTGCACA	109	110	1	0	0	0
SESN1	ACTGGTCATGGTTGCAAC	13	29	0	0	0	0
SESN1	CATGGTCATGGTTGCATG	224	350	0	1	0	0
SESN1	CATGCACAACTGGTACGT	108	274	1	0	0	0
SESN3	CATGGTGTGTTGACTGGT	354	255	0	0	0	0
SESN3	GTTGGTCATGGTACTGTG	230	301	0	0	0	0
SESN3	ACTGGTCATGGTTGGTGT	1023	1177	2	152	0	0
SESN3	TGTGGTCATGGTTGGTCA	318	349	0	0	0	3
SESN3	CATGGTCATGGTTGGTAC	101	104	0	0	0	0
SESN3	GTTGCATGGTTGTGGTCA	180	259	0	0	0	0
SET	TGACTGACCAGTTGACAC	73	20	0	0	0	0
SET	ACACTGACCAGTTGACTG	815	623	1	0	0	0
SET	CAACTGACCAGTTGTGGT	207	258	1	1	0	10
SET	ACACTGACCAGTTGTGCA	325	412	0	0	0	0
SET	GTACTGACCAGTTGTGAC	139	149	0	0	0	0
SETD2	ACCATGACACCAGTGTTG	834	450	3	0	0	0
SETD2	CACATGACACCAGTCAGT	100	73	0	1	0	1
SETD2	ACCATGACACCAGTCACA	24	36	0	0	0	6
SETD2	GTCATGACACCAGTCAAC	7	8	0	0	0	0
SETD2	TGCATGACACCAGTCATG	75	64	0	0	0	0
SETDB1	GTTGGTTGGTACGTGTTG	1072	701	5	1	1	0
SETDB1	GTTGGTGTCAGTGTTGTG	345	339	0	0	0	1
SETDB1	CATGGTTGGTACGTGTAC	110	113	1	0	0	0
SETDB1	TGTGGTTGGTACGTCAGT	73	115	0	0	0	0
SETDB1	GTTGGTTGGTACGTCACA	62	180	0	0	0	0
SF1	TGTGCAGTGTGTACCAAC	23	43	2	5	1	4
SF1	TGTGACGTGTCAGTACTG	202	380	0	0	0	0
SF1	ACTGACGTGTCAGTTGGT	114	149	0	0	0	0
SF1	TGTGACGTGTCAGTTGCA	117	72	0	0	0	0
SF1	CATGACGTGTCAGTTGAC	32	60	0	0	0	0

SFMBT2	CATGGTACGTCACATGGT	1258	1433	0	2	1	1
SFMBT2	ACTGGTACGTCACATGCA	829	427	1	1	1	0
SFMBT2	ACTGGTACGTCACAACTG	294	423	0	1	0	0
SFMBT2	GTTGGTACGTCACATGAC	87	81	0	0	0	0
SFMBT2	TGTGGTACGTCACATGTG	347	1530	5	1	0	0
SFN	GTACCATGACGTGTCACA	124	155	13	0	1	1
SFN	TGACCATGACGTGTCAGT	172	239	0	0	0	0
SFN	ACACCATGACGTGTCAAC	8	7	0	0	0	0
SFN	CAACCATGACGTGTCATG	14	17	1	0	0	0
SFN	CATGCACAGTGTTGCACA	59	53	0	0	0	1
SFRP1	CAACTGCATGTGGTGTTG	621	960	1	0	1	1
SFRP1	TGACCACACACACACATG	303	282	0	0	0	1
SFRP1	ACACTGCATGTGGTGTAC	15	16	0	0	0	0
SFRP1	GTACTGCATGTGGTCAGT	163	213	0	0	0	6
SFRP1	CAACTGCATGTGGTCACA	74	109	0	0	0	1
SFRP2	GTTGGTCATGACCACATG	316	384	1	0	2	0
SFRP2	GTACCACACACACACAAC	81	76	0	0	0	0
SFRP2	TGTGGTCATGACCAACGT	102	156	0	0	0	0
SFRP2	GTTGGTCATGACCAACCA	48	30	0	0	0	0
SFRP2	ACTGGTCATGACCAACAC	195	56	0	0	0	0
SFRP4	CAACGTACGTCAGTACCA	232	310	0	0	1	0
SFRP4	CAACGTACGTCAGTCATG	33	39	0	0	0	0
SFRP4	GTACGTACGTCAGTACGT	119	150	0	0	0	0
SFRP4	TGACGTACGTCAGTACAC	1	9	0	0	0	0
SFRP4	ACACGTACGTCAGTACTG	228	453	1	0	0	0
SFRP4	CATGCACATGGTGTTGTG	210	252	0	1	0	0
SFRS12	CACATGCATGTGGTGTGT	253	426	0	0	0	0
SFRS12	ACCATGCATGTGGTGTCA	326	607	0	0	0	0
SFRS12	GTCATGCATGTGGTGTAC	139	34	0	0	0	0
SFRS12	TGCATGCATGTGGTGTTG	409	943	1	1	0	1
SFRS12	ACCATGCATGTGGTCAGT	104	80	0	0	0	0
SFRS12IP1	ACTGGTCAACACCAACTG	701	586	0	0	1	1
SFRS12IP1	CATGGTCAACACCACATG	888	562	2	0	0	0
SFRS12IP1	GTTGGTCAACACCAACGT	194	318	0	1	0	0
SFRS12IP1	CATGGTCAACACCAACCA	136	242	0	0	0	0
SFRS12IP1	TGTGGTCAACACCAACAC	39	70	1	0	0	0
SFTPA2	GTACTGACGTGTCAGTCA	130	101	2	1	0	0
SFTPA2	ACACTGACGTGTCAGTAC	123	35	0	0	0	0
SFTPA2	TGTGGTCAACTGACACAC	89	40	0	0	0	0
SFTPA2	ACTGGTCAACTGACACTG	754	697	0	0	0	0
SFTPA2	CATGGTCAACTGACTGGT	248	316	0	0	0	0
SFTPA2	CATGCACATGTGCAACTG	83	97	0	0	0	0
SGK	ACTGCAACTGACACGTTG	329	225	1	488	42	1
SGK	GTACCATGACGTACGTGT	510	637	1	0	1	7
SGK	CAACCATGACGTCATGCA	96	168	0	0	0	0
SGK	TGACCATGACGTCATGAC	159	261	0	0	0	0
SGK	ACACCATGACGTCATGTG	1134	1105	2	2	0	4
SGK	GTTGGTACTGTGGTCAAC	16	21	0	0	0	0
SH2B2	ACTGACACGTGTTGACCA	263	264	0	1	1	0
SH2B2	ACTGGTTGACGTACCAAC	22	32	0	0	0	0
SH2B2	CATGGTTGACGTACCATG	36	105	0	0	0	1
SH2B2	GTTGGTTGACGTACACGT	57	31	0	0	0	0
SH2B2	CATGGTTGACGTACACCA	329	341	0	1	0	2
SH2D3C	TGACTGACTGTGACCAGT	732	619	1	0	0	0
SH2D3C	GTACTGACTGTGACCACA	110	120	0	0	0	0
SH2D3C	ACACTGACTGTGACCAAC	123	42	0	0	0	0
SH2D3C	CAACTGACTGTGACCATG	61	60	0	0	0	0
SH2D3C	GTACTGACTGTGACACGT	184	477	685	0	0	0
SH3BGRL	ACTGGTCACAACGTCAGT	350	437	1	0	1	1
SH3BGRL	GTTGGTCACAACGTGTAC	65	71	0	0	0	0
SH3BGRL	TGTGGTCACAACGTGTTG	619	557	1	0	0	0
SH3BGRL	TGTGGTCACAACGTCACA	103	99	0	0	0	0
SH3BGRL	TGTGGTCAACACCAGTCA	162	345	2	0	0	1
SH3BGRL	ACTGCACACAGTTGGTTG	1104	750	0	1	0	0
SH3GL2	CATGGTACTGTGTGACTG	607	1138	1	1	2	0
SH3GL2	CATGGTGTCAGTCACATG	99	126	0	0	0	1
SH3GL2	TGTGGTACTGTGTGACGT	117	194	0	0	0	0
SH3GL2	GTTGGTACTGTGTGACCA	351	213	1	0	0	2
SH3GL2	ACTGGTACTGTGTGACAC	20	6	0	0	0	0
SH3GL2	CATGCACACAGTTGCAGT	337	295	3	0	0	0
SH3GL3	CATGGTACACTGCACAGT	443	457	0	0	0	1
SH3GL3	ACTGGTACACTGCACACA	227	427	0	1	0	1
SH3GL3	GTTGGTACACTGCACAAC	27	33	0	0	0	0
SH3GL3	TGTGGTACACTGCACATG	599	763	0	2	0	0
SH3GL3	ACTGGTACACTGCAACGT	317	263	0	0	0	0

SH3GL3	ACTGCAACCAACGTCAGT	203	300	0	0	0	2
SH3GLB1	TGTGCAGTGTCAACTGTG	289	224	17	1	0	1
SH3GLB1	CATGCAGTGTCATGGTGT	134	101	0	0	0	0
SH3GLB1	ACTGCAGTGTCATGGTCA	170	250	2	1	0	1
SH3GLB1	GTTGCAACACGTGTCACA	177	139	0	0	0	0
SH3GLB1	ACTGCAACACGTGTCAAC	57	48	0	0	0	0
SH3GLB1	CATGCAACACGTGTCATG	12	15	0	0	0	0
SH3GLB2	CATGGTGTCAGTCAACCA	202	168	0	0	1	0
SH3GLB2	TGTGGTGTCAGTCAACAC	139	68	0	0	0	0
SH3GLB2	ACTGGTTGGTGTGTGTCA	758	486	2	1	0	2
SH3GLB2	GTTGGTTGGTGTGTGTAC	34	87	0	0	0	0
SH3GLB2	TGTGGTTGGTGTGTGTTG	849	485	3	1	0	0
SH3GLB2	TGTGCAACTGCAACACAC	7	15	0	0	0	0
SH3KBP1	GTACCAACTGACGTTGGT	585	150	0	0	1	1
SH3KBP1	CAACCAACTGACGTACTG	258	154	2	0	0	0
SH3KBP1	CAACCAACTGACGTTGCA	366	221	1	1	0	0
SH3KBP1	TGACCAACTGACGTTGAC	49	66	0	0	0	0
SH3KBP1	ACACCAACTGACGTTGTG	159	213	0	0	0	0
SH3KBP1	ACTGCAACGTGTCAACTG	250	272	0	1	0	0
SHB	TGTGCAGTGTTGGTGTGT	1570	1175	0	1	1	0
SHB	CATGGTCATGTGACACTG	387	588	0	1	0	0
SHB	GTTGGTCATGTGACTGGT	616	365	0	0	0	0
SHB	CATGGTCATGTGACTGCA	94	45	0	0	0	0
SHB	TGTGGTCATGTGACTGAC	168	27	1	0	0	0
SHBG	TGACTGTGTGACCAGTGT	752	556	0	1	0	1
SHBG	GTACTGTGTGACCAGTCA	335	321	0	0	0	0
SHBG	ACACTGTGTGACCAGTAC	55	79	0	0	0	0
SHBG	CAACTGTGTGACCAGTTG	544	281	2	0	0	0
SHBG	GTACTGTGTGACCACAGT	614	579	1	0	0	1
SHBG	TGTGCACAACACTGCAAC	56	43	0	1	0	0
SHC1	ACACCATGACGTTGACCA	185	237	0	0	0	0
SHC1	GTACCATGACGTTGACAC	29	134	0	0	0	0
SHC1	TGACCATGACGTTGACTG	246	199	0	0	0	0
SHC1	ACACCATGACGTTGTGGT	410	140	1	2	0	0
SHC1	CATGGTGTGTTGACACCA	5	4	0	0	0	0
SHC2	GTTGCAGTGTACCAACCA	999	762	3	1	1	6
SHC2	TGTGCAGTGTACCAACGT	166	104	1	0	0	0
SHC2	ACTGCAGTGTACCAACAC	82	60	0	0	0	0
SHC2	CATGCAGTGTACCAACTG	526	522	0	0	0	0
SHC2	GTTGCAGTGTACCATGGT	98	463	1	0	0	0
SHC3	TGTGGTGTACCACAACTG	247	279	0	0	0	0
SHC3	ACTGGTGTACACACCACA	54	20	0	0	0	0
SHC3	GTTGGTGTACACACCAAC	0	2	0	0	0	0
SHC3	TGTGGTGTACACACCATG	188	215	0	0	0	0
SHC3	ACTGGTGTACACACACGT	114	138	0	0	0	12
SHC4	TGTGGTGTCATGGTTGAC	26	29	0	0	0	4
SHC4	ACTGGTGTCATGGTTGTG	132	115	1	0	0	0
SHC4	GTTGGTGTCATGCAGTGT	933	548	0	0	0	0
SHC4	CATGGTGTCATGCAGTCA	293	207	0	2	0	0
SHC4	TGTGGTGTCATGCAGTAC	165	123	0	0	0	0
SHFM1	CATGGTGTGTGTACACAC	14	21	0	0	0	0
SHFM1	GTTGGTGTGTGTACACTG	29	46	1	0	0	0
SHFM1	TGTGGTGTGTGTACTGGT	359	171	0	1	0	0
SHFM1	GTTGGTGTGTGTACTGCA	93	87	0	0	0	0
SHFM1	ACTGGTGTGTGTACTGAC	20	21	0	0	0	0
SHFM1	CATGCACAACGTTGGTAC	0	6	0	0	0	0
SHH	GTACCAGTTGTGGTACTG	700	723	4	0	0	2
SHH	TGACCAGTTGTGGTTGGT	238	216	0	0	0	0
SHH	TGACCACAGTGTCATGGT	1356	456	1	1	0	2
SHH	GTACCACAGTGTCATGCA	83	94	1	0	0	0
SHH	ACACCACAGTGTCATGAC	107	111	0	0	0	0
SHH	TGTGCAACCATGCATGAC	101	312	0	0	0	0
SHISA5	TGTGCAACACCAACTGCA	236	140	0	0	1	0
SHISA5	TGTGGTTGACGTACGTCA	155	114	0	0	0	1
SHISA5	CATGGTTGACGTACGTAC	48	26	0	0	0	0
SHISA5	GTTGGTTGACGTACGTTG	306	222	3	0	0	0
SHISA5	TGTGGTTGACGTACCAGT	123	95	0	0	0	2
SHISA5	GTTGGTTGACGTACCACA	189	151	1	0	0	0
SHMT1	TGACCACACATGACGTCA	68	41	0	0	0	0
SHMT1	CAACCACACATGACGTAC	56	108	0	0	0	6
SHMT1	GTACCACACATGACGTTG	458	358	0	0	0	0
SHMT1	TGACCACACATGACCAGT	327	338	0	0	0	0
SHMT1	GTACCACACATGACCACA	232	182	0	0	0	0
SHMT1	ACTGCAACACTGCACATG	220	206	0	0	0	0
SHMT2	TGACCACACATGTGACGT	59	379	1	0	0	0

SHMT2	GTACCACACATGTGACCA	209	121	0	0	0	0
SHMT2	ACACCACACATGTGACAC	9	15	0	0	0	0
SHMT2	CAACCACACATGTGACTG	532	441	1	1	0	0
SHMT2	CATGGTTGTGACGTCAGT	32	72	1	0	0	0
SHOC2	ACACGTCAACCACATGGT	1389	1629	0	2	0	78
SHOC2	ACTGGTACCAGTACTGGT	190	176	0	1	0	0
SHOC2	TGTGGTACCAGTACTGCA	291	131	1	0	0	0
SHOC2	CATGGTACCAGTACTGAC	120	105	0	0	0	0
SHOC2	GTTGGTACCAGTACTGTG	817	911	1	0	0	1
SIGIRR	TGTGGTTGACGTACACAC	112	80	0	0	0	0
SIGIRR	ACTGGTTGACGTACACTG	218	172	1	0	0	0
SIGIRR	CATGGTTGACGTACTGGT	272	199	0	0	0	0
SIGIRR	ACTGGTTGACGTACTGCA	74	59	0	0	0	0
SIGIRR	TGTGCACATGACGTCACA	65	50	0	0	0	0
SIGIRR	CATGCACATGACGTCAAC	36	34	0	0	0	0
SIGLEC1	CATGGTACGTACCACAGT	75	48	3	0	1	0
SIGLEC1	GTTGGTACGTACCACAAC	9	16	0	0	1	0
SIGLEC1	ACTGGTACGTACCAGTTG	368	394	0	0	0	0
SIGLEC1	ACTGGTACGTACCACACA	92	289	0	5	0	7
SIGLEC1	TGTGGTACGTACCACATG	142	180	0	0	0	0
SIM1	TGACGTACCAGTGTCATG	348	277	1	1	0	0
SIM1	ACACGTACCAGTGTACGT	116	150	0	0	0	0
SIM1	TGACGTACCAGTGTACCA	197	133	0	0	0	0
SIM1	CAACGTACCAGTGTACAC	17	15	0	0	0	0
SIM1	GTACGTACCAGTGTACTG	218	360	0	0	0	0
SIM1	CATGCAACACCAACGTTG	1454	906	2	1	0	1
SIN3A	CAACGTTGACACTGCAAC	48	45	0	0	0	0
SIN3A	GTACGTTGACACTGCATG	141	358	0	0	0	0
SIN3A	TGACGTTGACACTGACGT	150	116	0	0	0	0
SIN3A	GTACGTTGACACTGACCA	13	34	0	0	0	0
SIN3A	ACACGTTGACACTGACAC	68	88	0	0	0	0
SIN3A	GTTGCAACTGGTACGTCA	32	49	0	0	0	0
SIN3B	GTTGGTGTCATGACACAC	55	65	0	0	0	0
SIN3B	TGTGGTGTCATGACACTG	264	227	0	1	0	0
SIN3B	ACTGGTGTCATGACTGGT	136	174	0	0	0	0
SIN3B	TGTGGTGTCATGACTGCA	175	203	0	0	0	0
SIN3B	CATGGTGTCATGACTGAC	40	49	0	0	0	0
SIPA1	ACTGGTACGTTGCAGTCA	226	368	3	0	0	0
SIPA1	GTTGGTACGTTGCAGTAC	265	292	0	0	0	1
SIPA1	TGTGGTACGTTGCAGTTG	1164	990	0	2	0	3
SIPA1	ACTGGTACGTTGCACAGT	391	338	0	0	0	0
SIPA1	GTTGGTTGACGTACTGAC	43	66	0	1	0	0
SIPA1	ACTGCAACTGACGTTGCA	107	94	0	0	0	0
SIRPA	GTCATGACCATGGTTGGT	257	260	0	0	0	0
SIRPA	CACATGACCATGGTTGCA	166	134	0	0	0	0
SIRPA	TGCATGACCATGGTTGAC	219	83	0	0	0	0
SIRPA	ACCATGACCATGGTTGTG	112	161	0	0	0	0
SIRPA	GTCATGACCATGCAGTGT	720	457	2	0	0	14
SIRT1	GTACGTACTGCAGTACTG	509	508	3	5	1	14
SIRT1	TGACGTCAACCACAACTG	909	1413	4	0	0	21
SIRT1	TGACGTACTGCAGTTGGT	452	350	0	0	0	0
SIRT1	GTACGTACTGCAGTTGCA	24	35	0	0	0	0
SIRT1	ACACGTACTGCAGTTGAC	15	16	0	0	0	0
SIRT1	CATGCAACGTGTGTACGT	239	159	1	0	0	0
SIRT2	CAACCATGACCAGTCAAC	98	43	0	0	1	1
SIRT2	GTACCATGACCAGTGTAC	109	125	0	0	0	0
SIRT2	TGACCATGACCAGTGTTG	471	468	0	0	0	17
SIRT2	ACACCATGACCAGTCAGT	181	183	1	0	0	0
SIRT2	TGACCATGACCAGTCACA	96	83	0	0	0	0
SIRT2	ACTGCAACGTGTGTACCA	148	176	0	0	0	0
SIRT4	GTACGTACTGGTTGACGT	193	273	0	8	0	0
SIRT4	CAACGTACTGGTTGACCA	139	196	0	0	0	0
SIRT4	TGACGTACTGGTTGACAC	23	35	0	0	0	0
SIRT4	ACACGTACTGGTTGACTG	105	130	0	0	0	0
SIRT4	CAACGTACTGGTTGTGGT	795	148	2	0	0	0
SIRT4	ACTGCACAACACACGTTG	310	322	1	0	0	0
SIRT5	TGACGTACTGGTACACTG	207	221	0	0	0	0
SIRT5	ACACGTACTGGTACTGGT	38	39	1	0	0	0
SIRT5	TGACGTACTGGTACTGCA	156	119	0	0	0	0
SIRT5	CAACGTACTGGTACTGAC	99	21	0	0	0	0
SIRT5	GTACGTACTGGTACTGTG	175	167	0	0	0	0
SIRT5	CATGCAACGTGTGTTGAC	42	67	0	0	0	0
SIRT6	ACACACACGTGTCATGAC	14	26	0	1	0	0
SIRT6	CAACACACGTGTCATGTG	266	383	1	0	0	0
SIRT6	TGACACACGTGTACGTGT	86	123	0	0	0	14

SIRT6	GTTGGTTGTGCAGTCACA	135	135	0	0	0	0
SIRT6	ACTGGTTGTGCAGTCAAC	40	46	0	0	0	0
SIRT6	ACTGCACAACTGTGACGT	323	386	0	0	0	1
SIRT7	TGACGTTGCAGTGTGTTG	974	1119	1	1	1	146
SIRT7	ACACGTTGCAGTGTCAGT	181	234	1	1	0	0
SIRT7	TGACGTTGCAGTGTCACA	105	126	0	0	0	0
SIRT7	CAACGTTGCAGTGTCAAC	50	23	0	0	0	0
SIRT7	ACTGCAGTACACTGACTG	319	487	0	1	0	1
SIRT7	TGTGCACAACTGTGACCA	1027	1060	2	0	0	2
SIX3	GTACGTACCAGTACGTTG	545	396	2	1	0	0
SIX3	TGACGTACCAGTACCAGT	95	73	0	0	0	5
SIX3	GTACGTACCAGTACCACA	61	122	0	1	0	0
SIX3	ACACGTACCAGTACCAAC	22	38	0	0	0	0
SIX3	CAACGTACCAGTACCATG	366	326	2	0	0	0
SIX3	GTTGCACACATGTGTGAC	10	39	0	0	0	0
SKI	GTACCATGCAGTGTCAAC	14	34	0	0	0	0
SKI	TGACCATGCAGTGTCATG	144	204	1	0	0	1
SKI	ACACCATGCAGTGTACGT	224	312	0	0	0	0
SKI	TGACCATGCAGTGTACCA	267	326	0	0	0	2
SKI	CAACCATGCAGTGTACAC	61	57	0	0	0	0
SKI	ACTGCACACAGTTGCACA	77	59	0	0	0	0
SKIL	CATGGTGTCAACCAGTTG	804	1171	3	0	0	0
SKIL	GTTGGTGTCAACCACAGT	247	288	0	0	0	0
SKIL	CATGGTGTCAACCACACA	148	124	0	0	0	3
SKIL	TGTGGTGTCAACCACAAC	0	0	0	0	0	0
SKIL	ACTGGTGTCAACCACATG	66	59	0	0	0	0
SKIP	TGACACACTGCATGACTG	940	1159	0	0	0	2
SKIP	ACACACACTGCATGTGGT	324	283	0	0	0	0
SKIP	TGACACACTGCATGTGCA	163	152	0	0	0	1
SKIP	CAACACACTGCATGTGAC	58	38	0	0	0	0
SKIP	TGTGCAGTTGCATGGTGT	333	221	1	0	0	0
SKIP	ACTGCAACCAGTCAGTAC	54	70	0	0	0	0
SKP1A	TGACGTGTGTACACGTAC	10	26	0	0	0	0
SKP1A	ACACGTGTGTACACGTTG	764	1222	0	0	0	28
SKP1A	CAACGTGTGTACACCAGT	73	244	0	0	0	0
SKP1A	ACTGCAGTCAGTGTGTGT	174	206	2	0	0	0
SKP1A	TGTGCAGTCAGTGTGTCA	211	194	2	0	0	0
SKP1A	TGTGCAACTGACTGGTGT	412	260	0	0	0	0
SKP2	ACACGTGTACCATGGTTG	1849	844	2	1	6	60
SKP2	CAACGTGTACCATGCAGT	207	332	0	0	1	0
SKP2	ACACGTGTACCATGCACA	309	392	17	0	0	0
SKP2	GTACGTGTACCATGCAAC	21	3	0	0	0	0
SKP2	TGACGTGTACCATGCATG	187	645	1	1	0	0
SKP2	GTTGCAACGTACCAGTCA	200	137	0	0	0	0
SLAIN1	ACTGCAGTGTGTTGCAAC	19	54	5	286	171	268
SLAIN1	CATGCAGTGTGTTGGTAC	38	28	0	0	0	2
SLAIN1	GTTGCAGTGTGTTGGTTG	544	553	3	0	0	0
SLAIN1	TGTGCAGTGTGTTGCAGT	303	198	0	0	0	0
SLAIN1	GTTGCAGTGTGTTGCACA	53	12	0	0	0	0
SLAMF1	ACACTGCATGGTACACCA	279	186	0	1	0	6
SLAMF1	GTACTGCATGGTACACAC	31	67	0	0	0	0
SLAMF1	TGACTGCATGGTACACTG	471	453	0	1	0	0
SLAMF1	ACACTGCATGGTACTGGT	535	228	2	0	0	0
SLAMF1	TGACTGCATGGTACTGCA	77	85	0	0	0	0
SLAMF1	GTTGCACACAGTTGCAAC	10	4	0	0	0	0
SLC10A1	ACACCAACTGTGACACGT	158	165	0	0	0	0
SLC10A1	TGACCAACTGTGACACCA	39	53	0	0	0	0
SLC10A1	CAACCAACTGTGACACAC	78	76	0	0	0	1
SLC10A1	GTACCAACTGTGACACTG	948	986	1	1	0	0
SLC10A1	TGACCAACTGTGACTGGT	607	331	0	1	0	1
SLC10A2	TGTGCACACAGTTGCATG	585	1137	2	1	3	2
SLC10A2	TGACCAACTGTGTGCAAC	57	12	0	0	0	0
SLC10A2	ACACCAACTGTGTGCATG	331	331	0	0	0	0
SLC10A2	CAACCAACTGTGTGACGT	370	296	1	0	0	1
SLC10A2	ACACCAACTGTGTGACCA	91	78	0	0	0	0
SLC10A2	GTACTGACTGCATGACGT	99	98	0	0	0	0
SLC11A1	TGACCATGACTGACTGTG	906	736	95369	146	23	70567
SLC11A1	CAACCATGACTGTGGTGT	1551	1810	1	0	2	0
SLC11A1	ACACCATGACTGTGGTCA	308	288	0	0	0	1
SLC11A1	GTACCATGACTGTGGTAC	113	98	0	0	0	0
SLC11A1	TGACCATGACTGTGGTTG	820	745	0	0	0	0
SLC12A1	TGACCATGACACGTTGCA	259	249	2	0	1	0
SLC12A1	ACACCATGACACGTACCA	205	299	1	0	0	0
SLC12A1	GTACCATGACACGTACAC	20	5	0	0	0	2
SLC12A1	TGACCATGACACGTACTG	279	528	0	0	0	0

SLC12A1	ACACCATGACACGTTGGT	382	341	0	0	0	0
SLC12A2	GTACCATGACACCAGTTG	1032	972	2	1	0	0
SLC12A2	TGACCATGACACCACAGT	221	209	1	0	0	0
SLC12A2	GTACCATGACACCACACA	187	273	1	0	0	2
SLC12A2	ACACCATGACACCACAAC	28	28	0	0	0	0
SLC12A2	CAACCATGACACCACATG	349	351	1	0	0	0
SLC12A3	CAACCATGACACGTTGAC	102	120	0	0	0	0
SLC12A3	GTACCATGACACGTTGTG	405	249	1	0	0	0
SLC12A3	ACACCATGACACCAGTGT	280	208	1	0	0	0
SLC12A3	TGACCATGACACCAGTCA	380	313	0	0	0	3
SLC12A3	CAACCATGACACCAGTAC	156	125	0	0	0	0
SLC12A3	ACTGCACACAGTTGACGT	142	155	0	0	0	0
SLC12A6	ACACCATGACACCATGCA	339	238	0	1	0	0
SLC12A6	GTACCATGACACCATGAC	46	26	39	1	0	0
SLC12A6	TGACCATGACACCATGTG	564	543	37	2	0	0
SLC12A6	CAACCATGACACACGTGT	373	182	0	1	0	0
SLC12A6	ACACCATGACACACGTCA	343	297	1	0	0	3
SLC13A5	TGACCATGTGCATGGTTG	465	508	2	0	1	1
SLC13A5	CAACCATGTGCATGGTGT	546	861	3	0	0	0
SLC13A5	ACACCATGTGCATGGTCA	292	348	2	0	0	0
SLC13A5	GTACCATGTGCATGGTAC	56	33	0	0	0	0
SLC13A5	TGTGCAGTTGCAACCATG	140	117	0	0	0	0
SLC13A5	TGTGCATGGTTGTGGTGT	799	366	0	0	0	0
SLC15A1	GTACCATGACTGTGACCA	52	57	1	0	0	0
SLC15A1	ACACCATGACTGTGACAC	32	18	0	0	0	0
SLC15A1	CAACCATGACTGTGACTG	194	164	0	0	0	0
SLC15A1	GTACCATGACTGTGTGGT	196	141	0	0	0	0
SLC15A1	CAACCATGACTGTGTGCA	231	249	1	1	0	0
SLC16A3	CAACCATGGTGTCATGGT	1150	719	1	0	1	3
SLC16A3	CAACCATGGTGTCAACCA	132	248	0	0	0	0
SLC16A3	TGACCATGGTGTCAACAC	16	30	0	0	0	0
SLC16A3	ACACCATGGTGTCAACTG	732	742	0	0	0	0
SLC16A3	ACACCATGGTGTCATGCA	600	196	0	0	0	1
SLC18A1	TGACACGTGTACGTCATG	18	37	0	0	0	0
SLC18A1	ACACACGTGTACGTACGT	150	288	0	0	0	24
SLC18A1	TGACACGTGTACGTACCA	212	192	0	0	0	4
SLC18A1	CAACACGTGTACGTACAC	213	99	0	0	0	0
SLC18A1	GTACACGTGTACGTACTG	189	201	0	0	0	7
SLC18A1	TGTGCACACAGTTGACCA	255	221	0	1	0	0
SLC18A2	TGACACGTGTACGTTGGT	127	174	0	0	0	5
SLC18A2	GTACACGTGTACGTTGCA	333	364	0	0	0	0
SLC18A2	ACACACGTGTACGTTGAC	10	11	1	0	0	0
SLC18A2	CAACACGTGTACGTTGTG	223	496	0	0	0	0
SLC18A2	TGACTGACTGCATGACAC	77	112	0	1	0	0
SLC18A3	GTACACGTGTCACACATG	185	186	0	0	0	0
SLC18A3	TGACACGTGTCACAACGT	248	183	0	0	0	1
SLC18A3	GTACACGTGTCACAACCA	340	352	0	0	0	0
SLC18A3	ACACACGTGTCACAACAC	23	16	0	0	0	0
SLC18A3	CAACACGTGTCACAACTG	562	740	1	1	0	0
SLC1A1	TGACCATGTGGTGTTGAC	44	194	0	0	1	0
SLC1A1	ACACCATGTGGTGTACAC	143	111	0	0	0	0
SLC1A1	CAACCATGTGGTGTACTG	581	252	0	0	0	0
SLC1A1	GTACCATGTGGTGTTGGT	324	388	0	0	0	0
SLC1A1	CAACCATGTGGTGTTGCA	76	46	0	0	0	0
SLC1A2	ACACCATGACTGCAACCA	295	338	4	129	61	65
SLC1A2	CAACCATGACTGCAACGT	1171	629	1	2	1	0
SLC1A2	TGACCATGACTGCACAAC	6	7	0	0	0	0
SLC1A2	ACACCATGACTGCACATG	232	201	0	0	0	0
SLC1A2	GTACCATGACTGCAACAC	49	55	0	0	0	0
SLC1A3	TGACCATGACTGCAACTG	488	459	1	0	1	0
SLC1A3	ACACCATGACTGCATGGT	427	382	1	1	0	7
SLC1A3	TGACCATGACTGCATGCA	266	295	1	0	0	0
SLC1A3	CAACCATGACTGCATGAC	14	23	0	0	0	0
SLC1A3	GTTGCAGTACTGACCAAC	53	31	0	0	0	0
SLC1A4	GTACCATGGTCAGTCACA	211	379	3	2	3	5
SLC1A4	GTACCATGGTCAGTGTTG	919	1036	1	1	0	11
SLC1A4	TGACCATGGTCAGTCAGT	567	314	0	2	0	1
SLC1A4	ACACCATGGTCAGTCAAC	43	35	0	0	0	0
SLC1A4	CAACCATGGTCAGTCATG	101	106	0	0	0	0
SLC1A4	ACTGCAACCATGTGGTAC	117	205	0	0	0	5
SLC1A6	TGACCATGTGCACAACTG	960	874	1	5	2	0
SLC1A6	ACACCATGTGCACACATG	377	269	0	0	0	0
SLC1A6	CAACCATGTGCACAACGT	179	222	0	0	0	0
SLC1A6	ACACCATGTGCACAACCA	321	364	0	0	0	0
SLC1A6	GTACCATGTGCACAACAC	61	91	0	0	0	0

SLC1A6	GTTGCACACATGACTGTG	1047	499	0	0	0	0
SLC1A7	TGACCATGTGGTACGTGT	316	293	0	0	0	0
SLC1A7	GTACCATGTGGTACGTCA	89	68	0	0	0	0
SLC1A7	ACACCATGTGGTACGTAC	20	56	0	0	0	0
SLC1A7	CAACCATGTGGTACGTTG	404	328	0	0	0	1
SLC1A7	GTACCATGTGGTACCAGT	181	197	0	0	0	0
SLC22A12	CAACCATGTGGTACTGAC	59	71	0	0	0	0
SLC22A12	GTACCATGTGGTACTGTG	56	85	1	0	0	0
SLC22A12	ACACCATGTGGTTGGTGT	328	518	0	0	0	2
SLC22A12	GTTGCAACTGCATGACCA	289	276	1	0	0	1
SLC22A12	ACTGCAACTGCATGACAC	86	79	0	0	0	0
SLC22A12	CATGCAACTGCATGACTG	175	161	0	0	0	1
SLC22A18	TGACCATGGTCAACGTAC	15	50	0	0	0	0
SLC22A18	ACACCATGGTCAACGTTG	643	514	0	0	0	6
SLC22A18	CAACCATGGTCAACCAGT	110	310	0	0	0	0
SLC22A18	ACACCATGGTCAACCACA	211	246	0	0	0	0
SLC22A18	GTACCATGGTCAACCAAC	42	40	0	0	0	0
SLC22A6	CAACCATGACTGACCATG	707	354	2	1	0	0
SLC22A6	GTACCATGACTGACACGT	14	24	0	0	0	0
SLC22A6	CAACCATGACTGACACCA	176	193	4	0	0	0
SLC22A6	TGACCATGACTGACACAC	246	176	0	0	0	2
SLC22A6	GTTGCAACTGCATGCATG	420	494	0	0	0	1
SLC22A6	TGTGCAACTGCATGACGT	200	177	0	0	0	2
SLC22A7	GTACCATGTGGTCAGTGT	1069	804	8	6	15	38
SLC22A7	TGACCATGTGGTCAGTAC	42	187	0	1	1	0
SLC22A7	ACACCATGTGGTGTTGTG	156	181	0	0	0	0
SLC22A7	CAACCATGTGGTCAGTCA	566	387	0	0	0	0
SLC22A7	ACACCATGTGGTCAGTTG	320	363	1	0	0	0
SLC23A1	TGACCAACTGTGGTACAC	30	48	0	0	0	0
SLC23A1	ACACCAACTGTGGTACTG	1206	888	0	1	0	2
SLC23A1	CAACCAACTGTGGTTGGT	323	219	0	0	0	0
SLC23A1	ACACCAACTGTGGTTGCA	48	23	0	0	0	0
SLC23A1	GTACCAACTGTGGTTGAC	68	32	0	0	0	0
SLC23A2	ACACCAACTGACTGACTG	793	895	1	1	0	0
SLC23A2	CAACCAACTGACTGTGGT	272	472	0	0	0	1
SLC23A2	ACACCAACTGACTGTGCA	120	121	0	0	0	0
SLC23A2	GTACCAACTGACTGTGAC	147	227	0	0	0	0
SLC23A2	TGACCAACTGACTGTGTG	592	543	3	1	0	15
SLC25A1	GTACCATGACACACCATG	244	230	0	0	0	0
SLC25A1	TGACCATGACACACACGT	68	86	0	0	0	1
SLC25A1	GTACCATGACACACACCA	206	243	0	0	0	0
SLC25A1	ACACCATGACACACACAC	87	53	0	0	0	0
SLC25A1	CAACCATGACACACACTG	1053	825	4	2	0	1
SLC25A1	TGTGCAACACGTACACCA	150	146	0	0	0	0
SLC25A11	CAACACGTGTGTCACATG	177	271	3	0	0	0
SLC25A11	GTACACGTGTGTCAACGT	307	164	1	0	0	0
SLC25A11	CAACACGTGTGTCAACCA	159	141	0	0	0	0
SLC25A11	TGACACGTGTGTCAACAC	40	29	0	0	0	0
SLC25A11	ACTGCAGTACGTCACATG	64	94	1	0	0	0
SLC25A20	GTACACGTGTCAGTACGT	263	283	0	0	0	0
SLC25A20	CAACACGTGTCAGTACCA	129	111	0	0	0	0
SLC25A20	TGACACGTGTCAGTACAC	22	28	0	0	0	0
SLC25A20	ACACACGTGTCAGTACTG	70	30	0	0	0	0
SLC25A20	CAACACGTGTCAGTTGGT	217	205	0	0	0	0
SLC25A4	CAACACGTGTACACTGGT	336	400	0	0	1	0
SLC25A4	ACACACGTGTACACTGCA	134	355	0	0	0	2
SLC25A4	GTACACGTGTACACTGAC	91	20	0	1	0	0
SLC25A4	TGACACGTGTACACTGTG	9	54	0	0	0	0
SLC25A4	CAACACGTGTACTGGTGT	541	824	0	2	0	0
SLC26A2	GTACGTCAGTTGGTTGCA	385	84	0	1	0	0
SLC26A2	ACACGTCAGTTGGTTGAC	27	28	0	0	0	0
SLC26A2	CAACGTCAGTTGGTTGTG	208	293	0	0	0	0
SLC26A2	TGACGTCAGTTGCAGTGT	960	704	8	1	0	1
SLC26A2	GTACGTCAGTTGCAGTCA	124	94	0	0	0	0
SLC27A1	ACACCAACTGACTGCAAC	115	81	1	0	0	0
SLC27A1	CAACCAACTGACTGCATG	178	200	0	0	0	0
SLC27A1	GTACCAACTGACTGACGT	92	168	0	0	0	0
SLC27A1	CAACCAACTGACTGACCA	248	289	1	0	0	22
SLC27A1	TGACCAACTGACTGACAC	35	166	0	0	0	0
SLC27A4	TGTGCATGTGTGGTTGGT	405	294	0	2	1	0
SLC27A4	ACACCATGTGGTCAACGT	239	283	0	0	0	0
SLC27A4	TGACCATGTGGTCAACCA	355	119	0	1	0	0
SLC27A4	CAACCATGTGGTCAACAC	240	401	0	0	0	0
SLC27A4	ACTGCAGTTGGTCAGTGT	1154	1369	1	1	0	4
SLC2A1	GTACCATGTGCACATGTG	193	146	1	0	1	0

SLC2A1	ACACCATGTGCAACGTGT	92	411	3	0	0	0
SLC2A1	TGACCATGTGCAACGTCA	183	131	0	0	0	0
SLC2A1	CAACCATGTGCAACGTAC	129	141	0	0	0	0
SLC2A1	GTACCATGTGCAACGTTG	699	770	0	1	0	0
SLC2A1	TGTGCACAACCACACAAC	19	9	0	0	0	0
SLC2A3	TGACCATGTGCAACCAGT	254	187	0	0	1	0
SLC2A3	GTACCATGACCAACACAC	64	62	0	0	0	11
SLC2A3	TGACCATGACCAACACTG	437	375	2	0	0	0
SLC2A3	GTACCATGTGCAACCACA	141	77	0	0	0	0
SLC2A3	ACACCATGTGCAACCAAC	31	35	0	0	0	0
SLC2A3	ACTGCACAACCAACGTGT	888	709	1	1	0	0
SLC2A4	CAACCATGTGCAACCATG	83	101	0	0	0	0
SLC2A4	GTACCATGTGCAACACGT	240	85	0	0	0	0
SLC2A4	CAACCATGTGCAACACCA	203	336	0	0	0	5
SLC2A4	TGACCATGTGCAACACAC	15	15	0	0	0	0
SLC2A4	TGTGCAGTGTGTACGTGT	801	340	1	0	0	0
SLC30A1	GTACCAACTGTGCAACCA	402	471	1	0	1	0
SLC30A1	CAACCAACTGTGCACAAC	40	27	0	0	0	0
SLC30A1	GTACCAACTGTGCACATG	228	174	0	0	0	0
SLC30A1	TGACCAACTGTGCAACGT	184	237	0	0	0	0
SLC30A1	ACACCAACTGTGCAACAC	21	35	0	0	0	1
SLC30A3	CAACCAACTGTGTGCACA	57	94	0	0	1	1
SLC30A3	GTACCAACTGTGTGGTCA	579	459	1	1	0	0
SLC30A3	ACACCAACTGTGTGGTAC	27	21	0	0	0	0
SLC30A3	CAACCAACTGTGTGGTTG	244	245	3	0	0	0
SLC30A3	GTACCAACTGTGTGCAGT	382	93	0	0	0	0
SLC30A4	CAACCAACTGTGTGTGAC	130	99	0	0	0	0
SLC30A4	GTACCAACTGTGTGTGTG	68	124	0	0	0	0
SLC30A4	TGACCATGGTGTGTGTGT	347	537	1	0	0	0
SLC30A4	GTACCATGGTGTGTGTCA	595	830	0	0	0	0
SLC30A4	GTTGCAGTACTGTGTGTG	445	482	2	0	0	0
SLC30A6	ACACCATGTGCAACACTG	210	238	1	0	0	0
SLC30A6	CAACCATGTGCAACTGGT	402	238	0	0	0	0
SLC30A6	ACACCATGTGCAACTGCA	256	151	0	0	0	0
SLC30A6	GTACCATGTGCAACTGAC	42	63	0	0	0	0
SLC30A6	TGACCATGTGCAACTGTG	206	294	0	0	0	7
SLC30A8	TGACCATGACCAACTGCA	253	295	0	0	1	78
SLC30A8	ACACCATGACCAACTGGT	136	223	1	0	0	0
SLC30A8	CAACCATGACCAACTGAC	26	23	0	0	0	0
SLC30A8	GTACCATGACCAACTGTG	178	187	1	1	0	0
SLC30A8	ACACCATGACCATGGTGT	438	441	2	0	0	1
SLC32A1	TGACCATGACTGGTCATG	130	158	1	0	0	1
SLC32A1	ACACCATGACTGGTACGT	95	424	0	1	0	1
SLC32A1	TGACCATGACTGGTACCA	424	432	0	0	0	43
SLC32A1	TGTGCAGTACTGTGGTGT	663	945	1	2	0	0
SLC32A1	GTTGCAGTACTGTGGTCA	943	1107	1	1	0	1
SLC33A1	GTACCATGACACGTCAGT	100	129	0	0	0	0
SLC33A1	CAACCATGACACGTCACA	222	148	0	0	0	1
SLC33A1	TGACCATGACACGTCAAC	23	22	25	0	0	0
SLC33A1	ACACCATGACACGTCATG	136	121	0	0	0	0
SLC33A1	CAACCATGACACGTACGT	231	339	0	0	0	0
SLC33A1	CATGCACAACCATGACTG	273	304	0	0	0	0
SLC34A1	CAACACGTGTACTGCAAC	21	14	0	0	0	0
SLC34A1	GTACACGTGTACTGCATG	70	94	0	0	0	0
SLC34A1	TGACACGTGTACTGACGT	214	425	0	0	0	7
SLC34A1	GTACACGTGTACTGACCA	38	39	0	0	0	0
SLC34A1	ACACACGTGTACTGACAC	16	24	0	0	0	0
SLC34A1	TGTGCATGCAGTACGTGT	144	155	0	0	0	0
SLC38A1	TGACCATGTGACCAACGT	209	212	1	0	0	0
SLC38A1	GTACCATGTGACCAACCA	795	788	3	0	0	0
SLC38A1	ACACCATGTGACCAACAC	40	88	0	0	0	0
SLC38A1	CAACCATGTGACCAACTG	580	249	2	1	0	0
SLC38A1	GTACCATGTGACCATGGT	889	448	0	1	0	0
SLC5A3	TGACCATGACCATGACAC	35	103	0	0	0	0
SLC5A3	ACACCATGACCATGACTG	140	464	3	1	0	0
SLC5A3	CAACCATGACCATGTGGT	172	190	0	0	0	0
SLC5A3	ACACCATGACCATGTGCA	139	269	0	0	0	0
SLC5A3	GTACCATGACCATGTGAC	54	52	0	0	0	0
SLC6A1	ACACCATGACCAACCATG	541	582	1699	0	1	6
SLC6A1	CAACCATGACCAACCACA	21	201	0	0	0	6
SLC6A1	TGACCATGACCAACCAAC	48	73	0	0	0	0
SLC6A1	CAACCATGACCAACACGT	13	16	0	0	0	2
SLC6A1	ACACCATGACCAACACCA	156	171	0	0	0	0
SLC6A11	GTACCATGACCATGCACA	77	58	0	0	0	0
SLC6A11	ACACCATGACCATGCAAC	28	3	0	0	0	0

SLC6A11	CAACCATGACCATGCATG	58	52	0	0	0	0
SLC6A11	GTACCATGACCATGACGT	122	188	0	0	0	1
SLC6A11	CAACCATGACCATGACCA	208	168	0	0	0	16
SLC6A11	TGTGCACAACTGGTACTG	627	968	2	1	0	2
SLC6A12	CAACCAACTGTGACCAGT	502	471	5	3030	5	0
SLC6A12	ACACCAACTGTGACGTTG	968	503	2	0	2	0
SLC6A12	GTACCAACTGTGACGTGT	713	732	1	0	0	0
SLC6A12	CAACCAACTGTGACGTCA	105	65	0	0	0	0
SLC6A12	TGACCAACTGTGACGTAC	91	57	0	0	0	0
SLC6A12	GTTGCAACCACAGTTGTG	440	441	0	1	0	0
SLC6A13	TGACCATGACCATGTGTG	495	902	1	0	0	0
SLC6A13	TGACCATGACACGTGTGT	226	194	0	0	0	0
SLC6A13	GTACCATGACACGTGTCA	122	126	0	0	0	0
SLC6A13	ACACCATGACACGTGTAC	196	269	2	0	0	0
SLC6A13	CAACCATGACACGTGTTG	837	514	0	1	0	0
SLC6A13	GTTGCAACACGTCATGGT	163	181	1	0	0	0
SLC6A2	ACACCATGTGCACATGGT	1039	344	3	2	0	0
SLC6A2	TGACCATGTGCACATGCA	136	141	0	1	0	0
SLC6A2	CAACCATGTGCACATGAC	127	125	0	1	0	0
SLC6A2	ACTGCAGTTGGTGTACCA	316	337	0	0	0	6
SLC6A2	GTTGCAGTTGGTGTACAC	67	51	0	0	0	0
SLC6A3	CAACCAACTGTGCAACTG	343	764	1	0	0	0
SLC6A3	GTACCAACTGTGCATGGT	1042	618	2	1	0	0
SLC6A3	CAACCAACTGTGCATGCA	710	561	0	0	0	0
SLC6A3	TGACCAACTGTGCATGAC	57	45	0	0	0	0
SLC6A3	ACACCAACTGTGCATGTG	2105	1046	6	2	0	10
SLC6A4	TGACCATGACCATGGTCA	199	398	0	0	1	0
SLC6A4	CAACCATGACCATGGTAC	30	37	0	0	0	0
SLC6A4	GTACCATGACCATGGTTG	272	337	0	0	0	0
SLC6A4	TGACCATGACCATGCAGT	386	476	0	0	0	0
SLC6A4	CATGCAGTTGGTGTCACA	206	254	0	0	0	1
SLC9A1	ACACACGTGTCAACCACA	53	78	0	0	0	0
SLC9A1	GTACACGTGTCAACCAAC	4	2	0	0	0	0
SLC9A1	TGACACGTGTCAACCATG	142	126	0	1	0	0
SLC9A1	ACACACGTGTCAACACGT	36	36	0	0	0	0
SLC9A1	TGACACGTGTCAACACCA	197	141	0	0	0	0
SLC9A1	GTTGCATGGTGTCAACAC	38	44	0	0	0	0
SLC9A3R1	GTACCATGTGCATGACCA	247	152	0	0	0	0
SLC9A3R1	ACACCATGTGCATGACAC	93	70	0	0	0	0
SLC9A3R1	CAACCATGTGCATGACTG	425	410	0	0	0	0
SLC9A3R1	GTACCATGTGCATGTGGT	400	363	0	0	0	0
SLC9A3R1	TGTGCAGTTGGTGTTGCA	45	91	0	0	0	0
SLCO1A2	CAACCATGACACTGACAC	63	57	0	0	0	0
SLCO1A2	GTACCATGACACTGACTG	227	322	0	1	0	0
SLCO1A2	TGACCATGACACTGTGGT	749	388	1	0	0	22
SLCO1A2	GTACCATGACACTGTGCA	196	399	1	0	0	0
SLCO1A2	TGTGACACCATGTGCAGT	218	228	1	0	0	0
SLCO1C1	ACACCATGACTGTGCAGT	429	164	0	0	0	0
SLCO1C1	TGACCATGACTGTGCACA	210	89	1	0	0	0
SLCO1C1	CAACCATGACTGTGCAAC	51	70	0	0	0	1
SLCO1C1	GTACCATGACTGTGCATG	41	37	0	0	0	0
SLCO1C1	TGACCATGACTGTGACGT	57	59	0	0	0	0
SLK	TGCATGCATGGTTGCATG	166	152	0	0	0	0
SLK	ACCATGCATGGTTGACGT	134	120	0	0	0	0
SLK	TGCATGCATGGTTGACCA	79	74	0	0	0	0
SLK	CACATGCATGGTTGACAC	29	7	0	0	0	0
SLK	GTCATGCATGGTTGACTG	117	122	1	0	0	0
SLPI	CAACACACTGGTGTGTTG	35	23	0	0	0	0
SLPI	GTACACACTGGTGTCAGT	29	36	0	0	0	0
SLPI	CAACACACTGGTGTCACA	184	99	1	0	0	0
SLPI	TGACACACTGGTGTCAAC	7	4	0	0	0	0
SLPI	ACACACACTGGTGTCATG	91	143	0	0	0	0
SLPI	TGTGCAACGTACGTCATG	372	350	0	1	0	3
SMAD1	CAACGTTGACACTGACTG	410	663	2	1	0	7
SMAD1	GTACGTTGACACTGTGGT	1136	511	2	1	0	0
SMAD1	CAACGTTGACACTGTGCA	102	187	0	1	0	0
SMAD1	TGACGTTGACACTGTGAC	45	39	0	0	0	0
SMAD1	ACACGTTGACACTGTGTG	813	717	1	2	0	0
SMAD2	GTACCATGCATGGTGTTG	380	984	2	0	0	0
SMAD2	TGACCATGCATGGTCAGT	356	270	16	0	0	28
SMAD2	GTACCATGCATGGTCACA	12	28	0	0	0	0
SMAD2	ACACCATGCATGGTCAAC	70	180	0	0	0	1
SMAD2	CAACCATGCATGGTCATG	362	130	0	0	0	0
SMAD3	GTACGTTGGTACACCAGT	43	34	1	0	0	0
SMAD3	CAACGTTGGTACACCACA	27	26	1	0	0	0

SMAD3	TGACGTTGGTACACCAAC	36	37	0	0	0	0
SMAD3	ACACGTTGGTACACCATG	236	164	0	0	0	0
SMAD3	CAACGTTGGTACACACGT	76	126	0	0	0	0
SMAD4	CAACGTTGTGTGGTGTCA	136	287	0	0	0	0
SMAD4	TGACCATGCAACTGTGTG	221	280	0	0	0	0
SMAD4	ACACCATGCATGGTGTGT	202	288	0	1	0	1
SMAD4	TGACCATGCATGGTGTCA	263	165	0	2	0	0
SMAD4	CAACCATGCATGGTGTAC	21	11	0	1	0	0
SMAD5	ACACACTGACTGGTCATG	231	93	0	0	0	0
SMAD5	CAACACTGACTGGTACGT	142	221	1	0	0	0
SMAD5	ACACACTGACTGGTACCA	584	88	0	0	0	1
SMAD5	GTACACTGACTGGTACAC	35	34	0	0	0	0
SMAD5	TGACACTGACTGGTACTG	769	538	0	0	0	0
SMAD6	GTACGTACTGACTGTGTG	680	338	0	0	0	0
SMAD6	TGACGTACTGTGGTGTGT	744	324	0	0	0	0
SMAD6	GTACGTACTGTGGTGTCA	234	366	0	2	0	0
SMAD6	ACACGTACTGTGGTGTAC	242	95	0	0	0	0
SMAD6	CAACGTACTGTGGTGTTG	1313	1005	16	1	0	16
SMAD7	CAACGTACTGTGCATGGT	193	161	0	0	0	0
SMAD7	ACACGTACTGTGCATGCA	294	376	0	2	0	1
SMAD7	GTACGTACTGTGCATGAC	17	22	0	0	0	0
SMAD7	ACACGTACTGTGACGTCA	144	288	1	1	0	0
SMAD7	GTACGTACTGTGACGTAC	9	11	0	0	0	0
SMARCA4	ACACGTACCACAGTGTAC	85	113	0	0	0	0
SMARCA4	CAACGTACCACAGTGTTG	696	724	3	0	0	1
SMARCA4	GTACGTACCACAGTCAGT	167	387	1	0	0	0
SMARCA4	CAACGTACCACAGTCACA	59	64	0	0	0	0
SMARCA4	TGACGTACCACAGTCAAC	14	80	0	0	0	0
SMARCA4	ACTGCAACCATGCATGTG	453	459	1	1	0	0
SMARCB1	GTACCATGGTTGGTGTAC	38	27	0	0	0	0
SMARCB1	TGACCATGGTTGGTGTTG	263	333	1	0	0	0
SMARCB1	ACACCATGGTTGGTCAGT	131	94	1	0	0	1
SMARCB1	TGACCATGGTTGGTCACA	34	65	0	0	0	0
SMARCB1	CAACCATGGTTGGTCAAC	15	12	0	0	0	0
SMARCC1	ACACGTACCACACAGTGT	43	80	0	0	0	0
SMARCC1	TGACGTACCACACAGTCA	97	158	2	0	0	0
SMARCC1	CAACGTACCACACAGTAC	7	10	0	0	0	0
SMARCC1	GTACGTACCACACAGTTG	356	275	1	0	0	1
SMARCC1	TGACGTACCACACACAGT	271	151	1	0	0	0
SMARCC1	ACTGCAACCATGACACGT	172	107	0	0	0	0
SMARCC2	TGACGTACCACACATGTG	80	91	0	0	0	0
SMARCC2	CAACGTACCACAACGTGT	916	310	0	0	0	0
SMARCC2	ACACGTACCACAACGTCA	321	330	0	0	0	0
SMARCC2	GTACGTACCACAACGTAC	3	4	0	0	0	0
SMARCC2	TGTGCAACCATGACACCA	341	428	1	0	0	2
SMARCC2	CATGCAACCATGACACAC	221	266	0	0	0	0
SMARCD1	ACTGGTACCAGTGTTGTG	280	655	0	0	0	0
SMARCD1	GTTGGTACCAGTCAGTGT	625	420	3	0	0	1
SMARCD1	CATGGTACCAGTCAGTCA	1611	1364	4	2	0	3
SMARCD1	TGTGGTACCAGTCAGTAC	135	173	0	1	0	0
SMARCD1	ACTGGTACCAGTCAGTTG	846	805	110	1	0	23
SMARCE1	CAACGTACCACATGGTCA	310	373	38	0	0	1
SMARCE1	TGACGTACCACATGGTAC	27	19	0	0	0	0
SMARCE1	ACACGTACCACATGGTTG	532	532	1	0	0	0
SMARCE1	CAACGTACCACATGCAGT	290	208	2	0	0	0
SMARCE1	ACACGTACCACATGCACA	181	377	1	0	0	0
SMC4	GTTGGTACCAACCACATG	322	210	1	0	1	0
SMC4	TGTGGTACCAACCAACGT	146	134	1	0	1	0
SMC4	CATGGTGTACGTACTGAC	26	39	0	0	0	0
SMC4	TGTGGTACCAACCACACA	28	23	0	0	0	0
SMC4	CATGGTACCAACCACAAC	16	24	0	0	0	0
SMC6	TGTGGTTGACTGCATGAC	46	44	0	1	0	0
SMC6	ACTGGTTGACTGCATGTG	450	302	0	0	0	21
SMC6	GTTGGTTGACTGACGTGT	288	178	1	1	0	0
SMC6	CATGGTTGACTGACGTCA	182	431	1	0	0	0
SMC6	TGTGGTTGACTGACGTAC	104	177	0	0	0	0
SMCHD1	ACTGCAGTGTCAACGTGT	382	336	2	0	1	0
SMCHD1	CATGCAGTGTGTACGTTG	1210	864	0	3	0	1
SMCHD1	TGTGCAGTGTCACATGCA	46	49	0	0	0	0
SMCHD1	CATGCAGTGTCACATGAC	69	94	0	0	0	0
SMCHD1	GTTGCAGTGTCACATGTG	990	564	2	2	0	0
SMN1	ACTGGTCAGTACGTGTTG	177	177	0	0	1	0
SMN1	CATGGTCAGTACGTGTCA	280	554	1	0	0	0
SMN1	TGTGGTCAGTACGTGTAC	165	120	0	0	0	6
SMN1	CATGGTCAGTACGTCAGT	645	683	0	1	0	0

SMN1	ACTGGTCAGTACGTCACA	34	45	0	0	0	0
SMN1	CATGCACATGTGCAGTGT	240	144	1	0	0	0
SMO	CAACGTACGTGTCATGGT	198	235	0	0	0	0
SMO	ACACGTACGTGTCATGCA	80	57	0	0	0	0
SMO	GTACGTACGTGTCATGAC	43	24	0	0	0	0
SMO	TGACGTACGTGTCATGTG	418	285	0	0	0	8
SMO	CAACGTACGTGTACGTGT	401	500	0	2	0	0
SMPD1	ACACACCAACCAACACGT	31	18	0	0	0	0
SMPD1	TGACACCAACCAACACCA	217	251	1	1	0	2
SMPD1	CAACACCAACCAACACAC	49	28	0	0	0	0
SMPD1	GTACACCAACCAACACTG	267	256	1	1	0	0
SMPD1	CATGGTGTCACAGTGTAC	24	31	0	0	0	0
SMPD2	TGACACCAACCAACTGGT	73	93	0	0	0	0
SMPD2	GTACACCAACCAACTGCA	460	267	0	0	0	0
SMPD2	ACACACCAACCAACTGAC	63	59	1	0	0	0
SMPD2	CAACACCAACCAACTGTG	685	281	1	0	0	0
SMPD2	TGACACCAACCATGGTGT	390	401	0	0	0	0
SMS	GTACACGTACCACACAAC	17	113	0	0	0	0
SMS	TGACACGTACCACACATG	307	167	0	0	0	1
SMS	ACACACGTACCACAACGT	218	566	1	0	0	4
SMS	TGACACGTACCACAACCA	94	70	1	0	0	0
SMS	CAACACGTACCACAACAC	62	57	0	0	0	0
SMS	GTTGCAACCATGACACTG	863	570	0	0	0	10
SMUG1	TGTGGTACGTTGCACACA	95	105	0	0	0	0
SMUG1	CATGGTACGTTGCACAAC	31	37	0	0	0	0
SMUG1	GTTGGTACGTTGCACATG	795	138	0	0	0	0
SMUG1	TGTGGTACGTTGCAACGT	604	497	0	0	0	0
SMUG1	GTTGGTACGTTGCAACCA	193	157	0	0	0	0
SMUG1	ACTGCACAACTGGTTGGT	873	404	1	2	0	1
SMURF1	GTCATGACTGGTACTGAC	7	13	0	0	0	0
SMURF1	TGCATGACTGGTACTGTG	325	224	1	0	0	0
SMURF1	CACATGACTGGTTGGTGT	266	603	0	0	0	0
SMURF1	ACCATGACTGGTTGGTCA	123	132	0	0	0	0
SMURF1	ACACCATGACCACACACA	231	239	0	0	0	1
SMURF1	GTTGCATGGTTGACCAAC	33	72	0	0	0	0
SMURF2	ACCATGACTGGTTGCAGT	250	161	0	0	1	39
SMURF2	TGCATGACTGGTTGCACA	115	121	0	1	1	0
SMURF2	GTCATGACTGGTTGGTAC	32	59	0	0	0	0
SMURF2	TGCATGACTGGTTGGTTG	148	231	0	0	0	0
SMURF2	TGACGTTGTGACTGTGGT	238	491	0	0	0	0
SNAI1	ACACTGACCACACAGTAC	61	80	0	0	0	0
SNAI1	CAACTGACCACACAGTTG	559	722	1	0	0	26
SNAI1	GTACTGACCACACACAGT	928	871	0	1	0	0
SNAI1	CAACTGACCACACACACA	98	109	4430	5	0	1
SNAI1	TGACTGACCACACACAAC	22	7	0	0	0	0
SNAI1	GTTGCAACCAGTCAACAC	14	5	0	0	0	0
SNAP23	TGTGGTCATGCAGTCATG	34	88	0	0	0	0
SNAP23	ACTGGTCATGCAGTACGT	118	137	0	0	0	0
SNAP23	TGTGGTCATGCAGTACCA	598	669	0	0	0	6
SNAP23	CATGGTCATGCAGTACAC	40	41	0	0	0	2
SNAP23	GTTGGTCATGCAGTACTG	254	1122	0	1	0	2
SNAP23	TGTGCAACCAGTCACAAC	23	45	0	0	0	0
SNAP25	ACTGGTACACGTCAGTGT	1070	668	1	1	3	2
SNAP25	GTTGGTACACGTCAGTTG	1649	932	0	2	1	1
SNAP25	CATGCAACCAGTCAACGT	157	277	2	0	1	2
SNAP25	TGTGGTACACGTCAGTCA	808	642	6	0	0	0
SNAP25	CATGGTACACGTCAGTAC	23	20	0	0	0	0
SNAP25	ACTGCAACCAGTCACATG	216	409	0	0	0	0
SNAP29	ACTGCAACCAGTCAACCA	521	674	2	1	2	1
SNAP29	CATGGTGTGTGTCAACTG	254	153	0	0	1	0
SNAP29	GTTGGTGTGTGTCAACCA	368	363	0	13	0	0
SNAP29	ACTGGTGTGTGTCAACAC	60	48	0	0	0	0
SNAP29	GTTGGTGTGTGTCATGGT	615	795	2	1	0	0
SNAP29	CATGGTGTGTGTCATGCA	1152	792	2	0	0	0
SNCA	TGTGCAGTACGTTGGTTG	320	630	0	0	1	1
SNCA	ACCATGACTGTGACCACA	158	138	0	0	0	0
SNCA	TGTGCACAACACCAGTTG	417	396	0	0	0	2
SNCA	ACTGCACAACACCACAGT	1440	829	2	0	0	1
SNCA	TGTGCACAACACCACACA	199	90	3	0	0	0
SNCA	CATGCACAACACCACAAC	16	36	0	0	0	0
SNCAIP	ACTGGTGTGTTGCAACCA	174	208	0	0	0	0
SNCAIP	TGTGGTACGTACGTCACA	14	88	0	0	0	0
SNCAIP	CATGGTACGTACGTCAAC	39	44	0	0	0	0
SNCAIP	GTTGGTACGTACGTCATG	52	83	0	0	0	0
SNCAIP	TGTGGTACGTACGTACGT	85	150	0	0	0	0

SNCB	GTTGGTTGACGTTGGTAC	60	53	0	0	1	0
SNCB	TGTGGTTGACGTACTGTG	282	102	0	0	0	0
SNCB	CATGGTTGACGTTGGTGT	478	637	1	0	0	0
SNCB	ACTGGTTGACGTTGGTCA	99	121	0	0	0	0
SNCB	TGTGGTTGACGTTGGTTG	506	514	0	1	0	0
SND1	ACACACCATGGTTGCATG	237	340	0	0	0	2
SND1	CAACACCATGGTTGACGT	38	49	0	0	0	0
SND1	ACACACCATGGTTGACCA	204	169	1	0	0	0
SND1	GTACACCATGGTTGACAC	48	93	0	0	0	1
SND1	TGACACCATGGTTGACTG	284	541	1	0	0	0
SNIP1	ACACTGTGCAGTACACGT	35	35	0	0	0	0
SNIP1	TGACTGTGCAGTACACCA	103	166	0	0	0	1
SNIP1	CAACTGTGCAGTACACAC	114	177	0	0	0	0
SNIP1	GTACTGTGCAGTACACTG	448	327	1	0	0	0
SNIP1	TGACTGTGCAGTACTGGT	256	302	0	0	0	0
SNIP1	GTTGCAACCATGTGACAC	29	54	0	0	0	0
SNRPA1	ACACTGACTGACTGCACA	18	12	0	0	0	0
SNRPA1	GTACTGACTGACTGCAAC	86	102	0	0	0	0
SNRPA1	TGACTGACTGACTGCATG	133	97	1	0	0	0
SNRPA1	ACACTGACTGACTGACGT	594	124	1	0	0	0
SNRPA1	TGACTGACTGACTGACCA	481	593	1	1	0	0
SNRPD2	CAACTGTGGTACTGTGAC	196	114	0	2	0	0
SNRPD2	GTACTGTGGTACTGTGTG	189	318	0	0	0	0
SNRPD2	TGACTGTGGTTGGTGTGT	60	82	0	0	0	0
SNRPD2	GTACTGTGGTTGGTGTCA	624	553	0	0	0	1
SNRPD2	TGTGGTTGTGGTGTACTG	68	53	0	0	0	0
SNRPD2	TGTGCATGGTACGTTGAC	22	60	0	0	0	0
SNRPN	TGACTGTGCAGTCATGAC	72	86	1	0	0	0
SNRPN	ACACTGTGCAGTCATGTG	549	605	2	0	0	1
SNRPN	GTACTGTGCAGTACGTGT	533	157	1	0	0	0
SNRPN	CAACTGTGCAGTACGTCA	201	135	1	0	0	1
SNRPN	TGACTGTGCAGTACGTAC	22	74	0	0	0	0
SNRPN	CATGCATGGTACCACAGT	115	91	0	0	0	0
SNW1	GTCATGCATGACACGTTG	193	318	0	0	0	0
SNW1	TGCATGCATGACACCAGT	142	212	0	0	0	0
SNW1	GTCATGCATGACACCACA	145	301	0	0	0	0
SNW1	ACCATGCATGACACCAAC	199	30	1	1426	0	0
SNW1	CACATGCATGACACCATG	96	153	0	0	0	0
SNW1	CATGCAACCAGTACGTAC	126	125	0	0	0	1
SNX1	CACATGTGGTACACGTAC	59	40	0	0	0	0
SNX1	GTCATGTGGTACACGTTG	545	642	5	36	0	0
SNX1	TGCATGTGGTACACCAGT	110	115	0	1	0	0
SNX1	GTCATGTGGTACACCACA	259	160	0	0	0	0
SNX1	ACCATGTGGTACACCAAC	41	65	0	0	0	0
SNX13	CATGGTACCAGTCACAGT	840	571	1	0	2	0
SNX13	ACTGGTACCAGTCACACA	101	118	0	0	0	0
SNX13	GTTGGTACCAGTCACAAC	30	40	0	0	0	0
SNX13	TGTGGTACCAGTCACATG	224	230	7	4	0	3
SNX13	ACTGGTACCAGTCAACGT	504	393	0	1	0	1
SNX2	GTACTGACTGGTGTTGAC	121	165	0	1	0	0
SNX2	TGACTGACTGGTGTTGTG	281	454	0	0	0	0
SNX2	CAACTGACTGGTCAGTGT	279	301	1	0	0	0
SNX2	ACACTGACTGGTCAGTCA	135	410	0	0	0	0
SNX2	GTTGCAGTCAACACACCA	74	92	0	0	0	0
SNX2	ACTGCAACTGGTGTCAGT	49	71	0	0	0	0
SNX4	ACTGGTCATGTGCAGTGT	998	657	1	1	1	0
SNX4	GTTGGTGTCAGTCAGTTG	77	217	0	0	0	19
SNX4	TGTGGTCATGTGGTTGCA	313	352	1	0	0	1
SNX4	CATGGTCATGTGGTTGAC	37	35	0	0	0	0
SNX4	GTTGGTCATGTGGTTGTG	137	175	0	0	0	0
SNX4	TGTGCAACTGGTGTCACA	51	58	1	0	0	0
SNX6	TGACTGACTGCAGTGTTG	969	722	0	2	0	0
SNX6	ACACTGACTGCAGTCAGT	47	36	0	0	0	0
SNX6	TGACTGACTGCAGTCACA	18	22	0	0	0	0
SNX6	CAACTGACTGCAGTCAAC	24	16	0	0	0	0
SNX6	GTTGGTGTCAGTCACACA	150	77	0	0	0	0
SOAT1	TGACCAACCATGCAACCA	506	460	1	1	0	0
SOAT1	CAACCAACCATGCAACAC	44	87	0	0	0	0
SOAT1	GTACCAACCATGCAACTG	344	444	0	1	0	12
SOAT1	TGACCAACCATGCATGGT	240	150	1	0	0	0
SOAT1	GTACCAACCATGCATGCA	226	179	0	0	0	0
SOCS1	ACACACTGACTGCACAAC	20	38	0	0	0	0
SOCS1	CAACACTGACTGCACATG	191	243	0	0	0	0
SOCS1	GTACACTGACTGCAACGT	289	225	0	0	0	0
SOCS1	CAACACTGACTGCAACCA	571	449	1	1	0	1

SOCS1	TGACACTGACTGCAACAC	83	99	2	0	0	0
SOCS1	GTTGCACACAGTTGTGCA	86	116	0	0	0	0
SOCS2	TGACACTGACTGCAGTCA	186	150	1	0	0	0
SOCS2	CAACACTGACTGCAGTAC	55	63	0	0	0	0
SOCS2	GTACACTGACTGCAGTTG	739	1015	1	0	0	8
SOCS2	TGACACTGACTGCACAGT	337	458	0	1	0	0
SOCS2	GTACACTGACTGCACACA	108	105	0	0	0	0
SOCS2	TGTGCAACACCAGTCACA	53	32	0	0	0	0
SOCS3	ACACACTGACTGCAACTG	154	278	1	0	0	0
SOCS3	CAACACTGACTGCATGGT	441	511	0	0	0	1
SOCS3	ACACACTGACTGCATGCA	566	477	0	1	0	1
SOCS3	GTACACTGACTGCATGAC	22	17	0	0	0	0
SOCS3	TGACACTGACTGCATGTG	814	1168	1	0	0	1
SOCS5	GTCATGACGTACACACCA	599	655	1	0	0	0
SOCS5	ACCATGACGTACACACAC	41	26	0	0	0	0
SOCS5	CACATGACGTACACACTG	422	418	0	0	0	2
SOCS5	GTCATGACGTACACTGGT	125	150	0	0	0	0
SOCS5	CACATGACGTACACTGCA	68	80	0	0	0	0
SOCS6	CACATGTGACACCATGGT	339	724	0	0	1	0
SOCS6	ACCATGTGACACCATGCA	296	237	1	1	0	0
SOCS6	GTCATGTGACACCATGAC	19	11	0	1	0	0
SOCS6	TGCATGTGACACCATGTG	649	621	0	0	0	1
SOCS6	CACATGTGACACACGTGT	315	752	1	1	0	0
SOCS7	TGTGGTGTCACAACCATG	113	49	0	0	0	0
SOCS7	ACTGGTGTCACAACACGT	59	22	0	0	0	0
SOCS7	GTTGGTACGTCACAACGT	136	94	0	0	0	0
SOCS7	CATGGTACGTCACAACCA	597	388	0	0	0	0
SOCS7	TGTGGTACGTCACAACAC	38	28	0	0	0	0
SOD1	CAACCATGCACAGTGTTG	2619	1035	6	0	1	4
SOD1	GTACCATGCACAGTCAGT	588	602	0	1	1	0
SOD1	ACACCATGCACAGTGTAC	47	76	0	10	0	0
SOD1	CAACCATGCACAGTCACA	60	152	0	0	0	0
SOD1	TGACCATGCACAGTCAAC	13	7	0	0	0	0
SOD2	TGCATGTGTGCACATGCA	115	77	0	1	0	0
SOD2	CACATGTGTGCACATGAC	25	36	0	0	0	0
SOD2	GTCATGTGTGCACATGTG	219	224	0	0	0	9
SOD2	ACCATGTGTGCAACGTGT	86	189	0	0	0	0
SOD2	CATGACCAGTTGACACTG	349	752	1	1	0	0
SOD3	TGACACCAACACGTACCA	97	388	0	0	0	0
SOD3	CAACACCAACACGTACAC	79	157	0	1	0	0
SOD3	GTACACCAACACGTACTG	296	284	0	0	0	0
SOD3	TGACACCAACACGTTGGT	435	967	0	0	0	0
SOD3	GTACACCAACACGTTGCA	274	340	0	0	0	0
SORBS1	CAACTGCACACAGTACGT	185	108	0	0	1	0
SORBS1	CAACTGCACACAGTCACA	436	282	2	0	0	3
SORBS1	TGACTGCACACAGTCAAC	15	8	0	0	0	0
SORBS1	ACACTGCACACAGTCATG	83	104	0	0	0	4
SORBS1	GTTGACCAACGTCATGTG	997	472	2	1	0	12
SORL1	TGACTGACGTCATGACCA	281	355	0	1	1	0
SORL1	CAACTGACGTCATGACAC	24	16	0	0	0	0
SORL1	GTACTGACGTCATGACTG	170	447	0	0	0	2
SORL1	TGACTGACGTCATGTGGT	350	792	2	3	0	0
SORL1	GTACTGACGTCATGTGCA	386	298	13	1	0	0
SORL1	ACTGCAACCAGTGTCACA	87	102	0	0	0	1
SOS1	CAACACCACACATGCAAC	26	27	0	0	1	0
SOS1	TGACACCACACATGACGT	753	807	1	1	1	1
SOS1	ACACACCACACATGCAGT	447	431	0	0	0	2
SOS1	TGACACCACACATGCACA	53	64	0	0	0	0
SOS1	GTACACCACACATGCATG	285	200	2	0	0	0
SOS1	GTTGCAACGTCATGACAC	119	154	0	0	0	1
SOS2	TGACACCACACATGTGAC	11	20	0	0	0	0
SOS2	ACACACCACACATGTGTG	160	200	0	0	0	0
SOS2	ACACACCACAACGTGTGT	106	149	0	0	0	8
SOS2	TGACACCACAACGTGTCA	85	73	0	0	0	0
SOS2	CAACACCACAACGTGTAC	22	5	0	0	0	0
SOST	ACTGGTGTGTCAACTGGT	344	733	0	1	1	0
SOST	TGTGGTGTGTCAACACTG	657	311	0	0	0	0
SOST	TGTGGTGTGTCAACTGCA	95	107	0	0	0	0
SOST	CATGGTGTGTCAACTGAC	47	38	0	0	0	0
SOST	GTTGGTGTGTCAACTGTG	536	1053	1	1	0	0
SOX1	ACACGTACCAACCAGTTG	1001	701	0	0	1	1
SOX1	CAACGTACCAACCAGTCA	170	286	0	0	0	0
SOX1	TGACGTACCAACCAGTAC	20	118	0	0	0	0
SOX1	CAACGTACCAACCACAGT	120	93	0	0	0	0
SOX1	TGTGCAGTACACACCAAC	51	60	0	0	0	0

SOX1	GTTGCATGGTACACTGTG	222	328	0	51	0	0
SOX10	CAACGTACTGCAGTTGTG	998	662	405	1	1	2
SOX10	TGACGTACTGCACAGTGT	748	544	1	1	0	0
SOX10	GTACGTACTGCACAGTCA	715	649	2	17	0	0
SOX10	ACACGTACTGCACAGTAC	121	45	0	0	0	0
SOX10	CAACGTACTGCACAGTTG	230	262	0	0	0	0
SOX10	CATGCATGGTACACTGAC	20	9	0	0	0	0
SOX17	TGACGTACTGGTTGGTCA	473	536	0	0	1	3
SOX17	ACTGCAGTACACTGGTGT	566	1235	3	0	1	19
SOX17	ACACGTACTGGTTGGTGT	398	943	0	0	0	1
SOX17	CAACGTACTGGTTGGTAC	74	60	0	0	0	0
SOX17	GTTGCAGTACACACTGTG	141	173	0	1	0	0
SOX2	GTTGGTGTGTTGCATGTG	382	377	0	2	1	0
SOX2	CACATGACACACTGGTTG	514	521	0	0	0	0
SOX2	GTCATGACACACTGCAGT	77	66	0	0	0	0
SOX2	TGTGGTGTGTTGCATGCA	252	243	0	0	0	0
SOX2	CATGGTGTGTTGCATGAC	18	14	0	1	0	0
SOX2	CATGCATGGTACACCACA	235	235	2	0	0	0
SOX4	ACACGTCAACCACAACCA	318	416	0	0	0	0
SOX4	GTACGTACACTGGTTGCA	202	228	1	0	0	0
SOX4	ACACGTACACTGGTTGAC	34	42	1	0	0	0
SOX4	CAACGTACACTGGTTGTG	342	335	0	0	0	0
SOX4	TGACGTACACTGCAGTGT	860	719	1	3	0	4
SOX4	ACTGCATGGTACTGGTGT	166	326	1	0	0	0
SOX6	TGACGTACACACTGCATG	856	908	2	2	1	2
SOX6	ACACGTACACACTGCACA	50	69	0	0	0	0
SOX6	GTACGTACACACTGCAAC	41	39	0	0	0	0
SOX6	ACACGTACACACTGACGT	97	142	1	0	0	9
SOX6	TGACGTACACACTGACCA	63	70	0	0	0	0
SOX9	ACACGTTGCAGTTGCAAC	59	53	0	0	1	1
SOX9	GTACGTTGCAGTTGCACA	119	158	0	0	0	0
SOX9	CAACGTTGCAGTTGCATG	416	296	1	0	0	1
SOX9	GTACGTTGCAGTTGACGT	89	122	0	0	0	0
SOX9	CAACGTTGCAGTTGACCA	330	503	3	2	0	0
SP1	TGACGTTGCACAGTTGGT	530	508	0	0	0	3
SP1	GTACGTTGCACAGTTGCA	35	78	1	0	0	0
SP1	ACACGTTGCACAGTTGAC	64	33	0	0	0	0
SP1	CAACGTTGCACAGTTGTG	10	5	0	0	0	0
SP1	CAACTGACTGACGTTGAC	46	21	0	0	0	0
SP100	ACACGTACTGACCACAGT	166	187	1	1	0	0
SP100	TGACGTACTGACCACACA	208	274	0	0	0	0
SP100	CAACGTACTGACCACAAC	62	85	0	0	0	0
SP100	GTACGTACTGACCACATG	46	58	0	0	0	0
SP100	TGACGTACTGACCAACGT	244	283	0	0	0	0
SP3	TGACGTTGCACAACCAGT	138	65	0	0	0	0
SP3	GTACGTTGCACAACCACA	213	189	0	1	0	0
SP3	ACACGTTGCACAACCAAC	10	11	0	0	0	0
SP3	CAACGTTGCACAACCATG	64	104	1	0	0	0
SP3	GTACGTTGCACAACACGT	20	9	0	0	0	0
SPAM1	ACTGGTTGACGTTGCAGT	81	315	0	0	0	1
SPAM1	TGTGGTTGACGTTGCACA	101	95	0	0	0	0
SPAM1	CATGGTTGACGTTGCAAC	8	19	0	0	0	0
SPAM1	GTTGGTTGACGTTGCATG	75	38	1	0	0	0
SPAM1	TGTGGTTGACGTTGACGT	83	66	0	0	0	0
SPAM1	ACTGCAACTGGTACTGGT	85	89	0	0	0	0
SPCS1	TGTGGTCAGTCAGTGTCA	650	463	0	0	0	0
SPCS1	CATGGTCAGTCAGTGTAC	225	111	0	0	0	0
SPCS1	GTTGGTCAGTCAGTGTTG	1014	623	1	0	0	0
SPCS1	TGTGGTCAGTCAGTCAGT	638	600	1	1	0	7
SPCS1	GTTGGTCAGTCAGTCACA	98	43	0	0	0	0
SPCS3	GTTGGTGTGTTGACCACA	63	67	0	0	0	0
SPCS3	ACTGGTCAGTGTTGCACA	61	34	0	0	0	0
SPCS3	GTTGGTCAGTGTTGCAAC	16	31	0	0	0	0
SPCS3	TGTGGTCAGTGTTGCATG	82	43	0	0	0	1
SPCS3	ACTGGTCAGTGTTGACGT	639	252	0	0	0	1
SPCS3	CATGCACATGACGTTGCA	418	149	0	2	0	0
SPDEF	CAACGTACCATGTGTGAC	63	61	0	0	0	0
SPDEF	GTACGTACCATGTGTGTG	296	226	1	0	0	0
SPDEF	ACACGTACACGTGTGTGT	104	218	1	0	0	0
SPDEF	TGACGTACACGTGTGTCA	212	151	0	0	0	0
SPDEF	CAACGTACACGTGTGTAC	46	39	0	0	0	0
SPDEF	TGTGCACAACACACGTAC	96	98	0	0	0	3
SPI1	GTACGTCAACCAGTTGCA	275	287	0	1	0	1
SPI1	ACACGTTGCACAACTGCA	110	248	0	0	0	0
SPI1	GTACGTTGCACAACTGAC	100	87	0	1	0	0

SPI1	TGACGTTGCACAACTGTG	103	111	0	0	0	0
SPI1	CAACGTTGCACATGGTGT	1139	1238	3	2	0	0
SPIB	CAACGTTGCACATGACTG	539	706	55767	40	4	9
SPIB	ACACGTTGCACATGACAC	118	146	0	0	0	0
SPIB	GTTGACCAGTGTGTACCA	160	163	0	0	0	0
SPIB	ACTGACCAGTGTGTACAC	39	49	0	0	0	0
SPIB	CATGACCAGTGTGTACTG	449	316	0	0	0	0
SPN	CATGGTTGGTTGGTGTTG	309	202	0	0	0	0
SPN	GTTGGTTGGTTGGTCAGT	84	71	0	0	0	0
SPN	CATGGTTGGTTGGTCACA	27	33	0	0	0	0
SPN	TGTGGTTGGTTGGTCAAC	14	6	0	0	0	0
SPN	ACTGGTTGGTTGGTCATG	72	160	0	0	0	0
SPO11	ACACACCAACTGGTTGTG	102	136	1	0	0	4
SPO11	GTACACCAACTGCAGTGT	906	901	2	0	0	6
SPO11	CAACACCAACTGCAGTCA	711	609	0	0	0	0
SPO11	TGACACCAACTGCAGTAC	186	129	0	0	0	0
SPO11	TGTGCAGTTGGTTGCATG	86	63	0	0	0	0
SPP1	TGCATGTGCAACTGCAGT	74	125	0	0	1	0
SPP1	TGCATGTGCAACTGGTCA	401	377	0	0	0	2
SPP1	CACATGTGCAACTGGTAC	48	40	0	0	0	0
SPP1	GTCATGTGCAACTGGTTG	235	249	1	0	0	0
SPP1	GTCATGTGCAACTGCACA	31	23	0	0	0	0
SPR	CAACACGTTGTGACGTGT	88	105	0	0	1	0
SPR	TGACACGTTGTGACGTTG	244	481	4	2	1	3
SPR	TGACACGTTGTGCATGTG	71	90	3	2	0	5
SPR	ACACACGTTGTGACGTCA	54	66	0	1	0	0
SPR	GTACACGTTGTGACGTAC	39	47	0	0	0	21
SPRED1	CAACACTGACGTCAGTCA	200	311	1	0	0	1
SPRED1	TGACACTGACGTCAGTAC	60	155	1	0	0	0
SPRED1	ACACACTGACGTCAGTTG	155	214	0	0	0	0
SPRED1	CAACACTGACGTCACAGT	389	353	0	1	0	0
SPRED1	ACACTGACTGCAACCATG	280	204	0	0	0	0
SPRED1	TGTGCAACACTGACGTCA	383	311	0	0	0	0
SPRED2	TGACACTGACCAGTACCA	582	346	0	0	1	3
SPRED2	GTTGCATGCACAACGTTG	531	558	0	0	1	0
SPRED2	CAACACTGACCAGTACAC	54	52	0	0	0	0
SPRED2	GTACACTGACCAGTACTG	291	174	1	0	0	0
SPRED2	TGACACTGACCAGTTGGT	633	784	1	1	0	3
SPRED2	TGTGCATGCACAACCAGT	44	205	0	0	0	50
SPRY1	CAACACTGACACGTCATG	130	232	0	0	0	0
SPRY1	GTACACTGACACGTACGT	64	55	0	0	0	0
SPRY1	CAACACTGACACGTACCA	157	219	0	0	0	2
SPRY1	TGACACTGACACGTACAC	16	23	0	0	0	0
SPRY1	ACACACTGACACGTACTG	116	440	0	1	0	0
SPRY2	GTACGTGTACCAACTGGT	615	441	7	1	3	8
SPRY2	GTACGTGTACCAACACCA	42	69	0	0	0	0
SPRY2	ACACGTGTACCAACACAC	37	33	0	0	0	0
SPRY2	CAACGTGTACCAACACTG	372	444	2	1	0	0
SPRY2	CAACGTGTACCAACTGCA	400	278	0	0	0	0
SPRY2	CATGCAACACACTGCAGT	410	179	1	0	0	0
SPRY3	ACACACTGACGTTGTGCA	87	79	0	0	0	1
SPRY3	GTACACTGACGTTGTGAC	45	49	0	0	0	0
SPRY3	TGACACTGACGTTGTGTG	655	484	1	1	0	0
SPRY3	GTACACTGACCAGTGTGT	267	83	0	0	0	0
SPRY3	TGTGCATGGTGTGTCATG	192	204	0	0	0	0
SPRY3	ACTGCATGGTGTGTACGT	331	177	0	1	0	0
SPRY4	GTACACTGACGTGTCATG	61	38	0	0	0	0
SPRY4	TGACACTGACGTGTACGT	89	130	0	0	0	0
SPRY4	GTACACTGACGTGTACCA	34	95	1	0	0	0
SPRY4	ACACACTGACGTGTACAC	12	13	0	0	0	0
SPRY4	CAACACTGACGTGTACTG	253	212	0	1	0	0
SPRY4	ACTGCAACTGGTCAACGT	1282	963	0	2	0	0
SPTAN1	TGACACTGGTCAACGTGT	262	459	1	0	0	5
SPTAN1	GTACACTGGTCAACGTCA	275	372	1	0	0	0
SPTAN1	ACACACTGGTCAACGTAC	60	84	1	0	0	0
SPTAN1	CAACACTGGTCAACGTTG	401	455	2	0	0	5
SPTAN1	CATGCAGTCAACACCAAC	36	126	0	0	0	1
SPTAN1	CATGCACACAGTTGACAC	40	26	0	0	0	0
SPTBN4	CATGGTGTCAGTGTACGT	35	56	0	0	0	0
SPTBN4	GTTGGTGTACCACAGTCA	357	628	1	0	0	0
SPTBN4	ACTGGTGTACCACAGTAC	95	124	1	0	0	2
SPTBN4	CATGGTGTACCACAGTTG	230	358	0	0	0	1
SPTBN4	GTTGGTGTACCACACAGT	250	184	4	0	0	0
SPTLC1	ACTGCATGGTACTGACTG	412	489	13	2	25	13
SPTLC1	TGACCACAACCATGTGGT	181	205	0	0	0	0

SPTLC1	CATGCAGTCACACATGTG	356	409	2	0	0	0
SPTLC1	TGTGCATGGTACTGACAC	27	14	0	0	0	0
SPTLC1	CATGCATGGTACTGTGGT	428	800	0	2	0	0
SPTLC1	ACTGCATGGTACTGTGCA	127	80	0	0	0	0
SPTLC2	TGACGTGTCAGTACGTAC	16	21	0	0	0	0
SPTLC2	TGACCACAACCACATGTG	503	454	0	0	0	0
SPTLC2	CAACCACAACCAACGTGT	397	405	2	0	0	0
SPTLC2	ACACCACAACCAACGTCA	231	149	0	0	0	0
SPTLC2	GTACCACAACCAACGTAC	45	58	0	0	0	0
SPTLC2	TGTGCATGCACAGTGTAC	125	150	0	0	0	0
SQLE	TGACACGTTGGTACACTG	1003	681	2	0	2	0
SQLE	GTACACGTTGGTACACAC	53	69	0	0	0	0
SQLE	ACACACGTTGGTACTGGT	207	450	0	0	0	0
SQLE	TGACACGTTGGTACTGCA	109	249	1	0	0	5
SQLE	CAACACGTTGGTACTGAC	57	63	0	0	0	0
SQSTM1	ACACGTGTCATGCAACCA	608	672	0	1	1	2
SQSTM1	CAACGTGTCATGCAACGT	222	535	0	0	0	0
SQSTM1	GTACGTGTCATGCAACAC	69	39	1	0	0	0
SQSTM1	TGACGTGTCATGCAACTG	867	1209	2	0	0	9
SQSTM1	TGTGACGTGTTGACACCA	152	135	0	0	0	1
SRC	GTACCAACTGACACCAGT	187	320	0	0	0	0
SRC	CAACCAACTGACACCACA	291	261	1	0	0	0
SRC	TGACCAACTGACACCAAC	57	78	0	0	0	1
SRC	ACACCAACTGACACCATG	136	59	0	0	0	0
SRC	ACTGGTTGGTGTTGCAAC	12	23	0	0	0	1
SRD5A1	CAACCAGTCATGACACTG	243	271	0	0	1	4
SRD5A1	ACACCAGTCATGACACAC	40	79	0	0	0	0
SRD5A1	GTACCAGTCATGACTGGT	112	95	0	0	0	0
SRD5A1	CAACCAGTCATGACTGCA	535	367	1	0	0	0
SRD5A1	CATGGTTGTGGTGTTGAC	242	48	1	1	0	0
SRD5A2	TGACCAGTCATGACTGAC	93	130	0	1	0	0
SRD5A2	ACACCAGTCATGACTGTG	249	316	0	0	0	0
SRD5A2	GTACCAGTCATGTGGTGT	1261	663	0	0	0	0
SRD5A2	CAACCAGTCATGTGGTCA	493	418	1	0	0	0
SRD5A2	TGACCAGTCATGTGGTAC	28	29	0	0	0	0
SREBF1	ACACGTTGCAACGTGTGT	388	570	1	0	0	0
SREBF1	TGACGTTGCAACGTGTCA	184	178	0	0	0	0
SREBF1	CAACGTTGCAACGTGTAC	98	226	0	0	0	0
SREBF1	GTACGTTGCAACGTGTTG	392	572	1	0	0	0
SREBF1	TGACGTTGCAACGTCAGT	218	116	0	1	0	0
SREBF2	ACACGTTGCAACCACAGT	175	195	1	0	0	4
SREBF2	TGACGTTGCAACCACACA	37	66	0	0	0	0
SREBF2	CAACGTTGCAACCACAAC	10	19	0	0	0	0
SREBF2	GTACGTTGCAACCACATG	108	101	0	0	0	1
SREBF2	TGACGTTGCAACCAACGT	384	445	1	0	0	0
SREBF2	CATGCATGGTGTGTCAGT	121	111	1	0	0	0
SRF	CACATGTGGTACACCATG	134	68	0	0	0	0
SRF	GTCATGTGGTACACACGT	11	11	0	0	0	0
SRF	CAACACTGGTTGACGTAC	79	55	0	0	0	0
SRF	GTACACTGGTTGACGTTG	656	605	3	1	0	0
SRF	TGACACTGGTTGACCAGT	160	190	1	0	0	0
SRM	TGTGCAGTCAGTACTGGT	474	416	0	0	1	0
SRM	CAACACGTACGTACGTCA	110	154	0	0	0	0
SRM	TGACACGTACGTACGTAC	65	69	0	0	0	0
SRM	ACACACGTACGTACGTTG	630	962	4	1	0	4
SRM	GTTGCAGTCAGTACACTG	344	211	0	0	0	0
SRP19	ACACTGTGCAGTCAGTCA	1202	766	0	6	1	0
SRP19	TGACTGTGCAGTGTTGTG	13	28	0	0	0	0
SRP19	CAACTGTGCAGTCAGTGT	544	382	3	0	0	0
SRP19	GTACTGTGCAGTCAGTAC	72	12	0	0	0	0
SRP19	TGACTGTGCAGTCAGTTG	864	467	0	1	0	8
SRP19	GTTGCACACAGTTGACTG	129	179	3	0	0	0
SRP54	TGTGGTGTCACACAGTTG	646	1116	1	2	0	14
SRP54	ACTGGTGTCACACACAGT	91	347	1	0	0	0
SRP54	GTTGGTCATGTGCACACA	218	141	1	0	0	2
SRP54	ACTGGTCATGTGCACAAC	16	31	0	2	0	0
SRP54	CATGGTCATGTGCACATG	72	77	0	0	0	0
SRP54	GTTGCAACCAACCATGCA	142	185	0	0	0	0
SRP68	ACACTGTGCAGTCACAGT	13	14	0	0	0	0
SRP68	TGACTGTGCAGTCACACA	182	66	0	1	0	0
SRP68	CAACTGTGCAGTCACAAC	15	34	0	0	0	0
SRP68	GTACTGTGCAGTCACATG	231	493	2	0	0	0
SRP68	TGACTGTGCAGTCAACGT	156	69	0	0	0	0
SRP68	ACTGCAACTGACGTACTG	327	273	0	1	0	0
SRP72	TGTGGTACCATGTGTGGT	353	247	2	0	0	0

SRP72	GTTGGTACCATGTGTGCA	158	123	0	0	0	13
SRP72	ACTGGTACCATGTGTGAC	87	52	0	0	0	0
SRP72	CATGGTACCATGTGTGTG	223	134	0	0	0	0
SRP72	TGTGGTACACGTGTGTGT	699	715	0	0	0	0
SRP9	TGACTGTGCAGTGTACAC	10	12	0	0	0	0
SRP9	ACACTGTGCAGTGTACTG	1005	251	0	1	0	0
SRP9	CAACTGTGCAGTGTTGGT	140	107	1	1	0	0
SRP9	ACACTGTGCAGTGTTGCA	108	186	0	0	0	3
SRP9	GTACTGTGCAGTGTTGAC	130	62	0	0	0	0
SRPR	TGACTGACGTACGTCACA	15	28	0	0	0	1
SRPR	CAACTGACGTACGTCAAC	6	7	0	0	0	0
SRPR	GTACTGACGTACGTCATG	67	232	0	0	0	0
SRPR	TGACTGACGTACGTACGT	56	47	0	0	0	0
SRPR	GTTGGTGTCACACAGTAC	49	30	0	0	0	0
SRPR	TGTGCAACTGGTCAACCA	61	44	1	0	0	0
SRXN1	GTACGTACACGTTGTGTG	59	60	0	0	0	0
SRXN1	CAACGTACACCAGTGTGT	631	523	1	2	0	1
SRXN1	ACACGTACACCAGTGTCA	360	316	0	0	0	0
SRXN1	GTACGTACACCAGTGTAC	57	52	0	1	0	0
SRXN1	CATGCAGTACACACACGT	57	107	0	0	0	0
SRXN1	GTTGCAACACACACACCA	522	538	0	1	0	0
SRY	ACACGTTGCAACTGCATG	447	485	0	0	1	0
SRY	TGACGTTGCAACTGCAAC	11	11	0	0	0	0
SRY	CAACGTTGCAACTGACGT	170	181	0	0	0	7
SRY	ACACGTTGCAACTGACCA	307	350	1	0	0	0
SRY	GTTGCAGTACACTGTGAC	52	35	0	0	0	0
SRY	TGTGCACACAGTTGTGGT	108	177	0	0	0	0
SSR4	GTACACTGGTCATGGTTG	446	566	1	0	1	0
SSR4	CAACACTGGTCATGGTAC	95	82	0	0	0	0
SSR4	TGACACTGGTCATGCAGT	19	16	0	0	0	0
SSR4	GTACACTGGTCATGCACA	549	285	0	1	0	0
SSR4	CATGCACAACCAGTCAGT	245	351	1	1	0	1
SSR4	ACTGCACAACCAGTCACA	137	120	0	0	0	38
SSRP1	TGACGTACTGACACCATG	65	36	0	0	0	0
SSRP1	ACACGTACTGACACACGT	108	178	0	0	0	2
SSRP1	TGACGTACTGACACACCA	202	400	1	0	0	0
SSRP1	CAACGTACTGACACACAC	49	56	1	0	0	0
SSRP1	CATGCATGGTCAGTTGAC	40	58	0	0	0	0
SSRP1	GTTGCATGGTCAGTTGTG	218	387	1	1	0	0
SST	TGACTGTGCAACGTACTG	212	421	0	1	1	1
SST	CAACTGTGCAACGTACGT	138	156	0	1	0	0
SST	ACACTGTGCAACGTACCA	300	174	0	0	0	0
SST	GTACTGTGCAACGTACAC	13	34	0	0	0	0
SST	TGTGGTGTCACAACTGGT	295	407	0	0	0	0
SSTR1	ACACGTACGTGTACGTCA	32	17	0	0	0	0
SSTR1	GTACGTACGTGTACGTAC	9	13	0	0	0	0
SSTR1	TGACGTACGTGTACGTTG	254	306	1	0	0	0
SSTR1	ACACGTACGTGTACCAGT	249	227	0	1	0	0
SSTR1	TGACGTACGTGTACCACA	29	47	0	0	0	0
SSTR2	CAACGTACGTGTACCAAC	44	48	0	0	0	0
SSTR2	GTACGTACGTGTACCATG	31	31	0	0	0	0
SSTR2	TGACGTACGTGTACACGT	9	8	0	0	0	0
SSTR2	GTACGTACGTGTACACCA	175	263	1	0	0	0
SSTR2	ACACGTACGTGTACACAC	34	30	0	0	0	0
SSTR3	CAACGTACGTGTACACTG	277	127	1	1	0	0
SSTR3	GTACGTACGTGTACTGGT	424	167	1	0	0	0
SSTR3	CAACGTACGTGTACTGCA	103	75	0	0	0	0
SSTR3	TGACGTACGTGTACTGAC	26	41	0	0	0	0
SSTR3	CATGCAGTACACCAGTCA	460	474	1	0	0	0
SSTR4	ACTGCAGTACACCAGTTG	1400	1074	2	1	1	3
SSTR4	ACACGTACGTGTACTGTG	179	193	0	0	0	0
SSTR4	GTACGTACGTGTTGGTGT	929	759	3	1	0	1
SSTR4	TGTGCAGTACACCAGTAC	60	71	0	0	0	6
SSTR4	GTTGCACACAACACACGT	41	22	0	0	0	0
SSTR5	CAACGTACGTGTTGGTCA	216	468	1	0	0	0
SSTR5	TGACGTACGTGTTGGTAC	28	40	0	0	0	0
SSTR5	ACACGTACGTGTTGGTTG	692	326	0	1	0	0
SSTR5	CATGCAGTACACCACAGT	203	131	1	0	0	0
SSTR5	TGTGCACACAACACACAC	118	120	0	0	0	0
SSTR5	ACTGCACACAACACACTG	995	893	1	1	0	1
ST3GAL1	CAACCACATGTGACACGT	136	111	0	1	0	0
ST3GAL1	ACACCACATGTGACACCA	204	355	0	1	0	0
ST3GAL1	GTACCACATGTGACACAC	15	16	0	0	0	0
ST3GAL1	TGACCACATGTGACACTG	95	47	0	0	0	0
ST3GAL1	ACACCACATGTGACTGGT	101	156	0	0	0	0

ST3GAL1	ACTGCATGGTGTCAACCA	140	120	0	0	0	0
ST3GAL2	TGACCAACGTGTACGTAC	3	8	0	0	0	0
ST3GAL2	ACACCAACGTGTACGTTG	504	828	0	0	0	0
ST3GAL2	CAACCAACGTGTACCAGT	181	474	4	0	0	0
ST3GAL2	ACACCAACGTGTACCACA	131	254	0	0	0	0
ST3GAL2	TGTGGTTGTGCATGGTGT	705	555	2	0	0	1
ST3GAL3	TGACCAACGTCAGTGTTG	264	309	6	2	2	12331
ST3GAL3	ACACCAACGTCAGTCAGT	370	265	0	0	1	0
ST3GAL3	ACTGGTTGTGGTTGCACA	58	63	0	0	0	0
ST3GAL3	GTTGGTTGTGGTTGCAAC	11	33	0	0	0	0
ST3GAL3	TGTGGTTGTGGTTGCATG	177	166	0	0	0	0
ST3GAL3	ACTGCATGGTCAGTGTAC	26	25	0	0	0	0
ST3GAL4	TGCATGTGACACTGTGGT	427	372	2	46	1	1
ST3GAL4	ACTGCACAACCAGTGTTG	240	269	3	0	1	0
ST3GAL4	ACCATGTGACACTGACGT	167	173	0	0	0	0
ST3GAL4	TGCATGTGACACTGACCA	118	285	0	0	0	0
ST3GAL4	CACATGTGACACTGACAC	20	48	0	0	0	0
ST3GAL4	GTCATGTGACACTGACTG	379	390	0	0	0	0
ST3GAL5	ACACCAACCAGTTGGTTG	1252	640	2	0	1	2
ST3GAL5	CAACCAACCAGTTGCAGT	210	250	0	0	0	0
ST3GAL5	ACACCAACCAGTTGCACA	282	171	0	0	0	0
ST3GAL5	GTTGCAGTACGTCACAGT	97	92	0	0	0	0
ST3GAL5	CATGCAGTACGTCACACA	241	204	0	0	0	0
ST3GAL6	ACACCACATGTGGTGTCA	396	484	0	1	0	0
ST3GAL6	GTACCACATGTGGTGTAC	4	25	0	0	0	0
ST3GAL6	TGACCACATGTGGTGTTG	836	730	1	0	0	2
ST3GAL6	ACACCACATGTGGTCAGT	101	110	0	0	0	0
ST3GAL6	TGACCACATGTGGTCACA	46	25	0	0	0	0
ST3GAL6	CATGCATGGTTGTGACGT	104	72	1	0	0	16
ST6GAL1	TGACCACATGACGTTGGT	205	227	0	1	1	0
ST6GAL1	TGACCACATGACGTACCA	114	156	0	0	0	0
ST6GAL1	CAACCACATGACGTACAC	54	124	0	0	0	0
ST6GAL1	GTACCACATGACGTACTG	324	183	0	0	0	0
ST6GAL1	GTACCACATGACGTTGCA	66	109	0	0	0	1
ST6GAL1	CATGCATGGTGTCAACGT	147	450	0	1	0	0
ST6GALNAC2	GTACCAACGTTGGTACTG	562	879	1	1	0	2
ST6GALNAC2	TGACCAACGTTGGTTGGT	130	119	0	0	0	0
ST6GALNAC2	GTACCAACGTTGGTTGCA	86	110	0	0	0	0
ST6GALNAC2	ACACCAACGTTGGTTGAC	96	95	0	0	0	0
ST6GALNAC2	CAACCAACGTTGGTTGTG	418	849	0	0	0	0
ST6GALNAC2	GTTGCACAACCAGTCAAC	36	45	11	1	0	0
ST6GALNAC3	TGACCACATGCACACACA	68	39	0	0	0	0
ST6GALNAC3	CAACCACATGCACACAAC	56	122	0	0	0	0
ST6GALNAC3	GTACCACATGCACACATG	302	397	0	0	0	0
ST6GALNAC3	TGACCACATGCACAACGT	43	49	0	0	0	0
ST6GALNAC3	CATGCAGTACTGACGTCA	130	186	0	0	0	0
ST6GALNAC3	CATGCAACTGGTCAACAC	35	46	0	0	0	0
ST6GALNAC4	CAACCAACGTTGCAGTTG	1787	1371	1	369	1	0
ST6GALNAC4	TGACCAACGTTGCAGTGT	589	594	0	0	0	1
ST6GALNAC4	GTACCAACGTTGCAGTCA	506	324	1	0	0	2
ST6GALNAC4	ACACCAACGTTGCAGTAC	17	20	0	0	0	0
ST6GALNAC4	GTTGGTGTACGTTGGTTG	254	260	1	1	0	0
ST6GALNAC5	GTACCACAACACACTGTG	269	178	0	0	0	0
ST6GALNAC5	ACACCACAACACTGGTGT	239	350	1	0	0	0
ST6GALNAC5	TGACCACAACACTGGTCA	431	238	3	5	0	0
ST6GALNAC5	CAACCACAACACTGGTAC	71	60	0	0	0	0
ST6GALNAC5	GTACCACAACACTGGTTG	468	521	0	0	0	0
ST6GALNAC5	GTTGCACATGTGTGACAC	52	34	0	0	0	0
ST6GALNAC6	ACACCACATGGTTGGTTG	440	384	0	0	0	0
ST6GALNAC6	CAACCACATGGTTGCAGT	127	164	0	0	0	0
ST6GALNAC6	ACACCACATGGTTGCACA	15	8	0	0	0	0
ST6GALNAC6	GTACCACATGGTTGCAAC	21	14	0	0	0	0
ST6GALNAC6	TGACCACATGGTTGCATG	59	88	0	0	0	0
ST8SIA1	TGACCAACGTTGTGGTTG	937	1768	2	0	1	0
ST8SIA1	ACACCAACGTTGTGCAGT	1048	653	26	1	0	27
ST8SIA1	TGACCAACGTTGTGCACA	89	262	0	0	0	0
ST8SIA1	CAACCAACGTTGTGCAAC	67	56	0	0	0	0
ST8SIA1	GTACCAACGTTGTGCATG	278	322	0	1	0	39
ST8SIA1	CATGCATGGTCAGTGTTG	1751	1343	0	1	0	0
ST8SIA2	ACACCACATGGTGTCATG	159	141	0	0	0	0
ST8SIA2	CAACCACATGGTGTACGT	98	84	0	0	0	0
ST8SIA2	ACACCACATGGTGTACCA	151	204	1	0	0	0
ST8SIA2	GTACCACATGGTGTACAC	93	23	0	0	0	0
ST8SIA2	TGACCACATGGTGTACTG	139	108	0	0	0	0
ST8SIA5	CAACCACATGGTACCAAC	46	85	0	0	0	0

ST8SIA5	GTACCACATGGTACCATG	75	70	0	0	0	0
ST8SIA5	TGACCACATGGTACACGT	172	456	0	0	0	0
ST8SIA5	GTACCACATGGTACACCA	57	245	0	0	0	0
ST8SIA5	ACACCACATGGTACACAC	40	61	0	0	0	7
STAG2	ACTGGTACGTCATGTGAC	70	72	5	0	3	0
STAG2	GTTGGTACGTCATGTGCA	157	259	0	0	2	0
STAG2	GTTGGTACGTCATGACTG	56	28	0	0	0	0
STAG2	TGTGGTACGTCATGTGGT	1081	806	1	3	0	0
STAG2	CATGGTACGTCATGTGTG	324	323	2	0	0	0
STAMBPL1	TGACTGTGGTACGTGTCA	208	223	0	1	1	0
STAMBPL1	ACACTGTGGTACGTGTGT	138	146	0	0	0	0
STAMBPL1	CAACTGTGGTACGTGTAC	75	110	0	0	0	0
STAMBPL1	GTACTGTGGTACGTGTTG	820	268	0	0	0	0
STAMBPL1	TGACTGTGGTACGTCAGT	212	201	0	0	0	0
STAR	GTACCATGACCAGTCATG	91	66	0	0	0	0
STAR	TGACCATGACCAGTACGT	426	503	0	0	0	1
STAR	GTACCATGACCAGTACCA	116	153	0	0	0	0
STAR	ACACCATGACCAGTACAC	57	68	0	0	0	0
STAR	CAACCATGACCAGTACTG	497	1056	2	0	0	1
STAT1	GTCATGTGCAGTGTACTG	320	322	0	1	1	0
STAT1	TGCATGTGCAGTGTACCA	36	75	15	0	0	0
STAT1	CACATGTGCAGTGTACAC	7	23	0	0	0	0
STAT1	TGCATGTGCAGTGTTGGT	278	286	2	1	0	0
STAT1	GTCATGTGCAGTGTTGCA	182	146	0	0	0	0
STAT1	ACTGCAACACCAGTCAGT	89	117	0	0	0	0
STAT2	ACACGTGTACCACACAAC	4	7	0	0	0	0
STAT2	CAACGTGTACCACACATG	46	50	0	0	0	0
STAT2	GTACGTGTACCACAACGT	529	423	0	1	0	0
STAT2	CAACGTGTACCACAACCA	127	124	1	0	0	0
STAT2	TGACGTGTACCACAACAC	97	67	0	0	0	0
STAT3	GTACGTTGCATGCAGTTG	909	963	0	0	0	1
STAT3	TGACGTTGCATGCACAGT	128	326	0	0	0	0
STAT3	GTACGTTGCATGCACACA	83	84	0	0	0	1
STAT3	ACACGTTGCATGCACAAC	28	31	0	0	0	2
STAT3	CAACGTTGCATGCACATG	288	170	0	0	0	0
STAT4	TGACGTTGCATGCATGTG	362	351	0	0	1	1
STAT4	CAACGTTGCATGACGTGT	356	430	0	0	0	1
STAT4	ACACGTTGCATGACGTCA	127	124	0	0	0	1
STAT4	GTACGTTGCATGACGTAC	42	65	0	0	0	0
STAT4	TGACGTTGCATGACGTTG	338	611	0	0	0	0
STAT4	ACTGCAACACCAACCATG	227	317	0	1	0	0
STAT5A	GTACGTCAACCAACTGAC	47	40	0	1	0	1
STAT5A	ACACGTACTGACACTGAC	31	36	0	0	0	0
STAT5A	CAACGTACTGACACTGTG	118	90	0	0	0	0
STAT5A	TGACGTACTGACTGGTGT	326	289	0	0	0	0
STAT5A	GTACGTACTGACTGGTCA	224	274	2	0	0	0
STAT5A	ACTGCAACACCAACACCA	412	354	0	0	0	0
STAT5B	TGACGTCAACCAACTGTG	233	280	0	0	0	0
STAT5B	ACACGTACTGTGGTACCA	203	326	0	0	0	0
STAT5B	GTACGTACTGTGGTACAC	21	23	0	0	0	0
STAT5B	TGACGTACTGTGGTACTG	99	90	0	0	0	0
STAT5B	ACACGTACTGTGGTTGGT	913	453	0	0	0	0
STAT6	CAACGTACTGTGACACTG	150	184	3	0	1	246
STAT6	TGACGTACTGTGACACGT	36	57	0	0	0	0
STAT6	GTACGTACTGTGACACCA	112	95	0	1	0	0
STAT6	ACACGTACTGTGACACAC	57	29	0	0	0	0
STAT6	GTACGTACTGTGACTGGT	328	405	0	0	0	2
STAT6	CATGCAACTGGTACGTTG	392	228	0	0	0	0
STEAP3	CAACTGACACACCAGTTG	139	124	0	0	0	0
STEAP3	GTACTGACACACCACAGT	158	128	1	0	0	0
STEAP3	CAACTGACACACCACACA	211	248	18	0	0	0
STEAP3	TGACTGACACACCACAAC	20	10	0	0	0	0
STEAP3	ACACTGACACACCACATG	199	220	0	0	0	0
STH	TGTGCAGTGTACACGTAC	16	16	0	0	0	0
STH	ACTGCAGTGTACACGTTG	563	361	2	1	0	0
STH	CATGCAGTGTACACCAGT	88	105	1	0	0	0
STH	ACTGCAGTGTACACCACA	115	54	0	0	0	0
STH	GTTGCAGTGTACACCAAC	14	42	0	0	0	0
STK11	ACCATGCACAACTGTGAC	51	35	0	0	0	0
STK11	CACATGCACAACTGTGTG	100	401	0	1	0	4
STK11	GTCATGCACATGGTGTGT	152	367	0	0	0	0
STK11	CACATGCACATGGTGTCA	498	697	0	0	0	13
STK11	TGCATGCACATGGTGTAC	84	77	0	1	0	0
STK36	ACACGTGTCACATGCAAC	33	21	0	0	1	0
STK36	GTACGTGTCACATGCACA	176	73	0	0	0	0

STK36	CAACGTGTCACATGCATG	10	35	0	0	0	0
STK36	GTACGTGTCACATGACGT	125	104	0	0	0	0
STK36	CAACGTGTCACATGACCA	582	218	0	0	0	1
STMN1	ACACTGACTGACCAGTGT	591	457	6	2	1	20
STMN1	GTACTGACTGACGTTGTG	227	279	1	2	0	0
STMN1	TGTGGTTGGTTGGTGTGT	368	802	0	0	0	1
STMN1	GTTGGTTGGTTGGTGTCA	273	189	0	0	0	0
STMN1	ACTGGTTGGTTGGTGTAC	45	44	0	1	0	0
STMN2	TGTGGTCATGCAACACAC	30	42	0	0	0	0
STMN2	ACTGGTCATGCAACACTG	361	403	0	2	0	2
STMN2	CATGGTCATGCAACTGGT	304	474	1	3	0	14
STMN2	ACTGGTCATGCAACTGCA	347	360	0	0	0	0
STMN2	GTTGGTCATGCAACTGAC	49	51	0	0	0	0
STON1	CAACGTACACACTGTGTG	463	660	1	1	0	0
STON1	GTACGTACACTGGTGTGT	48	137	1	0	0	0
STON1	CAACGTACACTGGTGTCA	284	224	0	0	0	1
STON1	CATGGTGTACGTTGACCA	221	385	0	0	0	0
STON1	CATGGTGTACACTGACGT	213	233	0	0	0	0
STON1	GTTGCAACTGTGCATGAC	57	51	0	0	0	0
STRA6	ACTGCAACCATGTGACCA	143	189	0	0	2	10
STRA6	CATGCAACCATGTGACGT	182	187	0	0	1	0
STRA6	CATGGTCAACGTTGACGT	110	114	0	0	0	0
STRA6	ACTGGTCAACGTTGACCA	164	14	0	1	0	0
STRA6	GTTGGTCAACGTTGACAC	73	37	1	0	0	0
STRA6	TGTGGTCAACGTTGACTG	377	524	5	0	0	0
STRAP	TGACTGCAACGTCAGTTG	539	354	0	1	1	0
STRAP	ACACTGCAACGTCAGTCA	47	37	0	0	0	0
STRAP	GTACTGCAACGTCAGTAC	24	20	0	1	0	0
STRAP	ACACTGCAACGTCACAGT	150	210	0	0	0	0
STRAP	TGACTGCAACGTCACACA	55	66	0	0	0	0
STS	TGTGACACTGGTTGGTTG	662	595	0	0	2	3
STS	ACACACACGTACTGGTAC	40	133	0	0	0	0
STS	CAACACACGTACTGGTTG	489	571	3	0	0	1
STS	GTACACACGTACTGCAGT	325	361	0	0	0	0
STS	CAACACACGTACTGCACA	109	101	0	0	0	0
STUB1	TGACGTGTACCAACTGAC	170	258	0	0	0	0
STUB1	ACACGTGTACCAACTGTG	621	1367	1	2	0	0
STUB1	GTACGTGTACCATGGTGT	367	955	2	0	0	0
STUB1	CAACGTGTACCATGGTCA	43	57	0	0	0	0
STUB1	TGACGTGTACCATGGTAC	90	185	0	0	0	0
STX10	TGTGGTTGACACTGCAGT	391	332	0	0	0	0
STX10	GTTGGTTGACACTGCACA	86	102	0	0	0	0
STX10	ACTGGTTGACACTGCAAC	6	8	0	0	0	0
STX10	CATGGTTGACACTGCATG	161	210	0	0	0	0
STX10	GTTGGTTGACACTGACGT	326	343	1	0	0	7
STX10	ACTGCACACAGTTGTGAC	55	39	0	0	0	0
STX11	TGTGCAGTTGTGGTGTTG	1129	824	0	2	1	1
STX11	CAACTGACTGGTTGCACA	91	93	1	0	0	0
STX11	TGACTGACTGGTTGCAAC	119	85	0	0	0	0
STX11	ACACTGACTGGTTGCATG	247	240	0	0	0	1
STX11	CAACTGACTGGTTGACGT	99	124	0	0	0	0
STX12	TGACTGACTGGTACCATG	118	116	0	0	0	0
STX12	ACACTGACTGGTACACGT	276	195	1	0	0	0
STX12	TGACTGACTGGTACACCA	34	25	0	0	0	5
STX12	CAACTGACTGGTACACAC	89	85	0	0	0	0
STX12	GTACTGACTGGTACACTG	85	90	4	0	0	2
STX16	TGTGGTTGGTACCAACGT	552	709	1	1	1	0
STX16	CATGGTTGGTACCACAAC	35	23	1	0	0	0
STX16	GTTGGTTGGTACCACATG	177	273	0	0	0	0
STX16	GTTGGTTGGTACCAACCA	6	2	0	0	0	0
STX16	ACTGGTTGGTACCAACAC	66	37	0	0	0	0
STX17	TGACTGCACAACACCAAC	9	16	0	0	0	0
STX17	ACACTGCACAACACCATG	158	156	0	0	0	0
STX17	CAACTGCACAACACACGT	84	90	1	0	0	0
STX17	TGTGCACATGGTACGTCA	160	334	0	0	0	0
STX17	CATGCACATGGTACGTAC	30	72	0	0	0	0
STX17	GTTGCACATGGTACGTTG	694	298	1	0	0	0
STX18	ACTGGTCACATGCACAGT	154	377	1	1	1	0
STX18	TGTGGTCACATGCACACA	62	43	0	1	0	0
STX18	CATGGTCACATGCACAAC	145	10	0	2	0	0
STX18	GTTGGTCACATGCACATG	849	744	4	0	0	0
STX18	CATGCAGTACGTCAGTTG	359	272	1	2	0	0
STX19	CAACTGACTGGTGTACCA	186	193	1	0	0	0
STX19	TGACTGACTGGTGTACAC	53	25	0	0	0	16
STX19	ACACTGACTGGTGTACTG	538	404	1	0	0	0

STX19	CAACTGACTGGTGTTGGT	268	316	0	0	0	0
STX19	ACACTGACTGGTGTTGCA	141	163	0	0	0	1
STX1A	GTACTGACTGGTCAGTAC	29	96	0	0	0	1
STX1A	TGACTGACTGGTCAGTTG	10	49	0	0	0	1
STX1A	ACACTGACTGGTCACAGT	326	222	1	0	0	10
STX1A	GTTGGTGTACGTTGCACA	44	89	1	0	0	0
STX1A	CATGGTTGTGCACACAAC	10	19	0	0	0	0
STX1A	GTTGCACACATGCATGCA	179	181	0	12	0	0
STX2	CAACTGACTGCACAGTCA	442	384	0	0	0	0
STX2	TGACTGACTGCACAGTAC	84	176	0	0	0	2
STX2	ACACTGACTGCACAGTTG	93	105	1	0	0	0
STX2	CAACTGACTGCACACAGT	100	128	0	0	0	2
STX2	ACACTGACTGCACACACA	135	103	0	0	0	4
STX3	TGACTGACTGGTCACACA	322	264	1	0	0	0
STX3	CAACTGACTGGTCACAAC	7	20	0	0	0	0
STX3	GTACTGACTGGTCACATG	109	136	0	0	0	12
STX3	TGACTGACTGGTCAACGT	227	279	1	1	0	0
STX3	GTTGCAGTCAACACCATG	679	493	1	1	0	0
STX4	TGACTGACTGGTCATGAC	95	76	0	0	0	0
STX4	ACACTGACTGGTCATGTG	78	74	0	0	0	0
STX4	GTACTGACTGGTACGTGT	289	370	1	1	0	1
STX4	CAACTGACTGGTACGTCA	128	135	0	0	0	1
STX4	TGACTGACTGGTACGTAC	33	241	0	0	0	0
STX5	CAACTGCACACAACTGCA	235	210	0	0	0	28
STX5	TGACTGCACACAACTGAC	18	34	0	0	0	0
STX5	ACACTGCACACAACTGTG	573	306	0	0	0	0
STX5	GTACTGCACACATGGTGT	299	279	0	0	0	0
STX5	CAACTGCACACATGGTCA	302	638	0	0	0	0
STX6	CAACTGCACAGTGTGTCA	159	233	0	0	1	0
STX6	GTACTGCAGTTGTGTGAC	20	9	0	0	0	0
STX6	TGACTGCAGTTGTGTGTG	799	609	2	1	0	0
STX6	GTACTGCACAGTGTGTGT	317	266	1	0	0	0
STX6	TGACTGCACAGTGTGTAC	38	25	0	0	0	0
STX8	ACACTGCACACATGGTTG	982	656	2	0	1	1
STX8	CAACTGCACACATGCAGT	43	98	0	0	1	0
STX8	TGACTGCACACATGGTAC	132	92	1	0	0	0
STX8	ACACTGCACACATGCACA	41	53	0	0	0	0
STX8	GTACTGCACACATGCAAC	51	28	0	0	0	0
STX8	ACTGCACACATGCATGAC	28	31	0	0	0	0
STXBP1	GTTGGTACGTGTCAGTCA	872	760	2	0	1	0
STXBP1	ACTGGTACGTGTCAGTAC	159	99	0	0	0	0
STXBP1	CATGGTACGTGTCAGTTG	353	295	2	0	0	3
STXBP1	GTTGGTACGTGTCACAGT	83	79	20	0	0	0
STXBP1	CATGGTACGTGTCACACA	32	82	1	0	0	0
STXBP4	CATGGTGTTGTGCATGCA	399	518	3	0	1	0
STXBP4	GTTGGTGTTGTGCATGGT	158	166	0	1	0	0
STXBP4	TGTGGTGTTGTGCATGAC	29	10	0	0	0	0
STXBP4	ACTGGTGTTGTGCATGTG	697	534	1	1	0	3
STXBP4	CATGCACAGTGTACTGTG	60	409	0	0	0	0
SUCLA2	TGACACCACAACTGTGCA	122	149	0	0	0	0
SUCLA2	CAACACCACAACTGTGAC	36	49	0	0	0	0
SUCLA2	GTACACCACAACTGTGTG	258	526	0	0	0	0
SUCLA2	TGACACCACATGGTGTGT	393	626	0	1	0	0
SUCLA2	GTACACCACATGGTGTCA	136	162	0	0	0	0
SUCLA2	CATGCACATGACGTACTG	198	226	0	1	0	0
SUCLG1	CAACACCACAACTGACGT	131	193	0	0	0	0
SUCLG1	ACACACCACAACTGACCA	330	330	0	0	0	4
SUCLG1	GTACACCACAACTGACAC	112	56	0	0	0	0
SUCLG1	TGACACCACAACTGACTG	443	511	0	0	0	0
SUCLG1	ACACACCACAACTGTGGT	107	153	1	0	0	0
SUCLG2	GTACACCACAACTGCAGT	455	280	0	0	0	2
SUCLG2	CAACACCACAACTGCACA	164	345	0	0	0	0
SUCLG2	TGACACCACAACTGCAAC	36	96	8	0	0	0
SUCLG2	ACACACCACAACTGCATG	461	409	0	0	0	0
SUCLG2	CAACACCACATGGTCACA	143	132	0	0	0	1
SUCLG2	CATGCATGGTACCATGTG	107	81	0	0	0	0
SUFU	GTACGTACTGTGTGCAAC	8	13	0	0	0	0
SUFU	TGACGTACTGTGTGCATG	108	128	0	0	0	0
SUFU	ACACGTACTGTGTGACGT	207	145	0	0	0	0
SUFU	TGACGTACTGTGTGACCA	221	193	0	0	0	0
SUFU	CAACGTACTGTGTGACAC	62	72	0	0	0	7
SUFU	GTTGCAACCACAACGTAC	70	51	0	0	0	0
SULT1A1	CAACCACAACTGACCAGT	139	139	0	0	0	0
SULT1A1	ACACCACAACTGACCACA	419	177	0	0	0	0
SULT1A1	ACACCACAACTGTGACCA	161	198	2	0	0	0

SULT1A1	GTACCACAACTGTGACAC	65	60	0	0	0	0
SULT1A1	TGACCACAACTGTGACTG	199	181	2	0	0	5
SULT1A1	CATGCATGGTACCAGTCA	160	144	1	0	0	1
SULT1A2	TGACCACAACTGACGTAC	84	123	0	0	0	0
SULT1A2	ACACCACAACTGACGTTG	302	390	0	0	0	0
SULT1A2	ACACCACAACTGTGTGGT	576	580	1	0	0	1
SULT1A2	TGTGCAGTGTGTACACTG	264	219	0	0	0	8
SULT1A2	GTTGCAGTGTGTACTGTG	663	1411	1	0	0	3
SULT1A2	GTTGCATGGTACCACAAC	108	45	0	0	0	0
SULT1A3	CAACCACATGGTACACTG	141	162	0	0	1	0
SULT1A3	GTACCACATGGTACTGGT	197	112	0	0	0	0
SULT1A3	CAACCACATGGTACTGCA	121	141	1	0	0	0
SULT1A3	TGTGGTACCATGTGACCA	505	536	0	0	0	0
SULT1A3	CATGGTACCATGTGACAC	22	15	0	0	0	0
SULT1A4	ACTGGTTGACACGTCAGT	116	161	0	1	0	0
SULT1A4	ACTGCAGTGTGTACTGGT	115	93	0	0	0	1
SULT1A4	TGTGCAGTGTGTACTGCA	62	65	0	0	0	0
SULT1A4	CATGCAGTGTGTACTGAC	19	24	0	0	0	0
SULT1A4	TGTGCAGTGTACACCATG	211	228	0	0	0	0
SULT1B1	ACACCACAACCAACTGTG	194	1124	0	0	2	0
SULT1B1	CAACCACAACCAACTGCA	216	232	0	0	0	0
SULT1B1	TGACCACAACCAACTGAC	27	32	0	0	0	0
SULT1B1	GTACCACAACCATGGTGT	104	67	0	0	0	0
SULT1B1	CAACCACAACCATGGTCA	828	592	0	0	0	2
SULT1B1	TGTGCATGGTACCACATG	334	485	1	0	0	0
SULT1C2	GTACCACATGGTCACACA	310	261	0	0	0	1
SULT1C2	ACACCACATGGTCACAAC	43	30	1	0	0	0
SULT1C2	CAACCACATGGTCACATG	56	87	0	0	0	0
SULT1C2	GTACCACATGGTCAACGT	330	314	0	0	0	0
SULT1C2	CAACCACATGGTCAACCA	776	639	4	3	0	1
SULT1C4	CAACCACAACGTCATGAC	164	89	0	0	0	0
SULT1C4	GTACCACAACGTCATGTG	318	201	1	0	0	0
SULT1C4	ACACCACAACGTACGTGT	822	854	0	0	0	0
SULT1C4	TGACCACAACGTACGTCA	564	545	4	0	0	0
SULT1C4	CAACCACAACGTACGTAC	12	25	0	0	0	0
SULT1C4	GTTGCATGGTCAACCATG	304	297	0	1	0	0
SULT1E1	CAACCAACGTACCACATG	275	195	0	0	0	17
SULT1E1	GTACCAACGTACCAACGT	230	228	0	0	0	0
SULT1E1	CAACCAACGTACCAACCA	89	102	0	0	0	0
SULT1E1	TGACCAACGTACCAACAC	59	38	0	0	0	0
SULT1E1	ACTGCATGGTACCAACGT	230	272	2	0	0	0
SULT1E1	TGTGCATGGTACCAACCA	191	204	1	1	0	0
SULT2A1	GTACCAACGTGTTGACAC	15	23	0	0	0	0
SULT2A1	TGACCAACGTGTTGACTG	485	989	2	1	0	0
SULT2A1	ACACCAACGTGTTGTGGT	228	198	0	0	0	1
SULT2A1	TGACCAACGTGTTGTGCA	386	450	1	1	0	0
SULT2A1	TGTGCATGGTACCAGTAC	13	32	0	0	0	0
SULT2A1	ACTGCATGGTACCAGTTG	323	493	0	2	0	0
SULT2B1	CAACCACATGCAGTCATG	272	497	30	1	1	0
SULT2B1	GTACCACATGCAGTACGT	89	41	0	0	0	0
SULT2B1	CAACCACATGCAGTACCA	252	147	1	0	0	4
SULT2B1	TGACCACATGCAGTACAC	24	36	0	0	0	0
SULT2B1	ACACCACATGCAGTACTG	159	148	0	0	0	0
SULT2B1	GTTGCATGCAGTACACAC	1	12	0	0	0	0
SULT4A1	CAACCACAACCACAACCA	339	471	0	0	1	0
SULT4A1	GTACCACAACCACACACA	63	53	0	0	0	0
SULT4A1	ACACCACAACCACACAAC	17	40	0	0	0	0
SULT4A1	CAACCACAACCACACATG	534	348	0	0	0	0
SULT4A1	GTACCACAACCACAACGT	542	498	1	0	0	0
SULT4A1	TGTGCATGGTCATGCATG	118	107	1	0	0	0
SUMO1	TGACGTCAACCATGCACA	396	327	1	0	0	0
SUMO1	GTTGGTCAACGTACGTGT	372	320	1	0	0	0
SUMO1	CATGGTCAACGTACGTCA	94	94	0	0	0	0
SUMO1	TGTGGTCAACGTACGTAC	156	93	1	0	0	0
SUMO1	ACTGGTCAACGTACGTTG	273	364	1	1	0	0
SUMO2	CATGGTGTCAGTTGTGTG	91	100	0	0	0	0
SUMO2	ACTGGTGTCACAGTGTGT	1252	1005	1	1	0	0
SUMO2	GTTGACGTACTGGTACCA	92	239	0	1	0	0
SUMO2	ACTGACGTACTGGTACAC	2	4	0	0	0	0
SUMO2	CATGACGTACTGGTACTG	238	189	0	1	0	0
SUMO3	CAACGTGTACACCAACAC	42	44	0	0	0	0
SUMO3	GTACGTGTACACCAACTG	222	286	0	0	0	0
SUMO3	TGACGTGTACACCATGGT	132	136	2	0	0	0
SUMO3	GTACGTGTACACCATGCA	387	209	0	0	0	0
SUMO3	TGTGCAGTACGTGTTGGT	210	322	0	0	0	0

SUOX	CAACACGTTGCAACGTAC	186	201	0	0	1	0
SUOX	GTACACGTTGCACATGTG	332	280	1	1	0	0
SUOX	ACACACGTTGCAACGTGT	122	108	0	1	0	0
SUOX	TGACACGTTGCAACGTCA	75	123	0	0	0	0
SUOX	GTACACGTTGCAACGTTG	104	179	2	0	0	0
SUPT6H	CAACGTTGGTCACAACCA	236	329	1	0	2	6
SUPT6H	GTACGTTGGTCACAACGT	40	54	1	0	0	0
SUPT6H	TGACGTTGGTCACAACAC	93	12	0	0	0	0
SUPT6H	ACACGTTGGTCACAACTG	653	552	0	0	0	16
SUPT6H	CATGCAGTGTTGTGACAC	73	57	0	1	0	0
SUV39H1	GTTGGTACCACAGTGTGT	1789	1292	3	1	1	1
SUV39H1	CATGGTACCACAGTGTCA	530	672	1	1	1	0
SUV39H1	GTTGGTACCAGTTGTGAC	18	19	0	0	0	3
SUV39H1	TGTGGTACCAGTTGTGTG	103	144	1	0	0	0
SUV39H1	CATGCAGTGTACTGACGT	112	111	0	0	0	0
SUZ12	TGACCATGGTCAACTGGT	236	183	0	0	0	0
SUZ12	GTACCATGGTCAACTGCA	250	489	1	0	0	0
SUZ12	ACACCATGGTCAACTGAC	176	152	0	0	0	0
SUZ12	CAACCATGGTCAACTGTG	116	182	0	0	0	0
SUZ12	TGACCATGGTCATGGTGT	293	221	0	0	0	9
SUZ12	CATGCAACGTCAACGTCA	447	488	1	0	0	2
SVIL	CATGGTGTTGGTGTTGTG	186	176	0	0	0	0
SVIL	TGTGGTGTTGGTCAGTGT	210	265	0	0	0	0
SVIL	GTTGGTGTTGGTCAGTCA	53	44	7	0	0	0
SVIL	ACTGGTGTTGGTCAGTAC	44	90	1	0	0	0
SVIL	CATGGTGTTGGTCAGTTG	490	929	1	1	0	2
SYK	TGCATGACACACGTACAC	57	149	0	0	0	0
SYK	ACCATGACACACGTACTG	430	389	4	0	0	0
SYK	CACATGACACACGTTGGT	661	98	0	0	0	0
SYK	ACCATGACACACGTTGCA	152	153	1	1	0	0
SYK	GTCATGACACACGTTGAC	40	28	0	0	0	0
SYN3	ACTGGTACCATGCATGCA	71	88	0	0	0	1
SYN3	GTTGGTACCATGCATGAC	39	66	0	1	0	0
SYN3	TGTGGTACCATGCATGTG	362	334	0	0	0	34
SYN3	CATGGTACCATGACGTGT	74	34	1	0	0	0
SYN3	ACTGGTACCATGACGTCA	128	148	0	0	0	0
SYN3	TGTGCAACCACATGGTAC	101	88	0	0	0	0
SYNE1	GTTGGTTGACGTTGACCA	139	48	0	0	0	0
SYNE1	ACTGGTTGACGTTGACAC	67	56	0	0	0	0
SYNE1	CATGGTTGACGTTGACTG	86	76	0	0	0	0
SYNE1	GTTGACACTGCACAGTTG	120	92	0	1	0	0
SYNE1	TGTGACACTGCACACAGT	197	224	0	0	0	0
SYNGR1	CAACGTCAACCACAGTTG	758	678	0	0	1	0
SYNGR1	ACTGCAGTGTTGTGCACA	32	78	1	0	0	0
SYNGR1	GTTGCAGTGTTGTGCAAC	26	20	0	0	0	0
SYNGR1	TGTGCAGTGTTGTGCATG	108	133	0	0	0	0
SYNGR1	ACTGCAGTGTTGTGACGT	315	329	0	1	0	1
SYNJ1	CAACACCATGCATGACCA	841	398	2	1	0	0
SYNJ1	TGACACCATGCATGACAC	21	7	0	0	0	0
SYNJ1	ACACACCATGCATGACTG	393	472	2	0	0	0
SYNJ1	CAACACCATGCATGTGGT	221	410	0	0	0	0
SYNJ1	ACACACCATGCATGTGCA	452	159	0	0	0	1
SYNJ2	ACACACCATGTGTGACAC	37	114	0	0	0	0
SYNJ2	CAACACCATGTGTGACTG	369	617	0	1	0	0
SYNJ2	GTACACCATGTGTGTGGT	455	524	429	1	0	0
SYNJ2	CAACACCATGTGTGTGCA	367	395	0	0	0	0
SYNJ2	TGACACCATGTGTGTGAC	73	29	0	0	0	0
SYP	TGACACTGGTACGTGTGT	2267	1462	0	0	2	6
SYP	GTACACTGGTACGTGTCA	542	586	1	0	0	0
SYP	ACACACTGGTACGTGTAC	191	36	0	0	0	0
SYP	CAACACTGGTACGTGTTG	196	157	0	0	0	0
SYP	GTACACTGGTACGTCAGT	376	305	0	1	0	4
SYT1	TGTGCACAACGTCACATG	99	222	3	0	1	8
SYT1	GTACACTGGTACGTACAC	23	22	0	0	0	0
SYT1	TGACACTGGTACGTACTG	219	274	0	0	0	0
SYT1	ACACACTGGTACGTTGGT	155	180	2	0	0	0
SYT1	TGACACTGGTACGTTGCA	67	37	0	0	0	0
SYT1	TGTGGTGTCAGTCACAGT	141	402	0	0	0	0
SYT2	GTTGCAGTACGTACCACA	48	36	1	0	1	11
SYT2	CACATGACGTACCAGTAC	77	204	0	0	0	0
SYT2	GTCATGACGTACCAGTTG	778	1038	1	1	0	0
SYT2	TGCATGACGTACACTGAC	12	14	0	0	0	0
SYT2	ACCATGACGTACACTGTG	538	191	0	0	0	0
SYT2	GTTGCATGCAGTACCAGT	254	212	0	0	0	0
T	ACCATGTGACACCAACTG	156	204	0	0	1	2

T	CACATGTGACACCACATG	54	72	0	0	0	0
T	GTCATGTGACACCAACGT	544	666	0	0	0	0
T	CACATGTGACACCAACCA	140	117	0	0	0	0
T	TGCATGTGACACCAACAC	72	42	0	0	0	0
T	ACTGCAACCAGTTGACAC	41	37	0	0	0	0
TAAR6	ACACGTCATGACACCATG	123	307	0	0	0	0
TAAR6	CAACGTCATGACACACGT	9	107	0	0	0	0
TAAR6	ACACGTCATGACACACCA	445	341	0	0	0	0
TAAR6	GTACGTCATGACACACAC	75	81	0	0	0	0
TAAR6	CATGCAGTACACCATGTG	346	658	0	1	0	0
TAAR6	GTTGCATGGTGTTGACCA	131	137	1	1	0	0
TACC3	CAACTGCATGACGTCATG	300	267	0	0	0	1
TACC3	GTACTGCATGACGTACGT	107	88	0	0	0	0
TACC3	CAACTGCATGACGTACCA	233	219	1	0	0	0
TACC3	TGACTGCATGACGTACAC	19	24	0	0	0	0
TACC3	CATGCAGTCATGCATGAC	115	34	0	0	0	0
TACC3	TGTGCACAACCAGTCATG	134	119	0	0	0	0
TACR1	CAACGTCATGACACTGAC	83	146	4	0	1	0
TACR1	TGACGTCATGACACACTG	411	692	6491	9	0	0
TACR1	ACACGTCATGACACTGGT	724	326	0	0	0	0
TACR1	TGACGTCATGACACTGCA	136	148	0	1	0	0
TACR1	GTACGTCATGACACTGTG	182	215	1	914	0	0
TACR1	GTTGCACAACTGCATGAC	13	28	0	0	0	0
TACR2	GTACGTCATGACTGGTTG	276	482	0	0	1	1
TACR2	ACACGTCATGACTGGTGT	346	344	0	0	0	0
TACR2	TGACGTCATGACTGGTCA	226	188	1	1	0	0
TACR2	CAACGTCATGACTGGTAC	263	189	0	0	0	0
TACR2	TGACGTCATGACTGCAGT	135	371	1	0	0	0
TACR2	CATGCACACAGTTGTGTG	192	462	2	1	0	0
TACR3	GTACGTCATGACTGCACA	141	526	3	0	1	0
TACR3	ACACGTCATGACTGCAAC	56	141	0	0	0	0
TACR3	CAACGTCATGACTGCATG	61	294	2	0	0	0
TACR3	GTACGTCATGACTGACGT	342	186	1	0	0	0
TACR3	CAACGTCATGACTGACCA	319	291	1	1	0	0
TACR3	TGTGCACAACTGCATGTG	561	474	0	0	0	1
TAF1	TGACGTGTGTCAGTCATG	124	171	0	0	0	0
TAF1	ACACGTGTGTCAGTACGT	78	76	0	0	0	0
TAF1	TGACGTGTGTCAGTACCA	74	128	0	0	0	0
TAF1	CAACGTGTGTCAGTACAC	91	115	1	0	0	0
TAF1	GTACGTGTGTCAGTACTG	97	49	1	0	0	0
TAF1	GTTGCAACCATGGTGTTG	435	471	1	2	0	0
TAF10	CAACGTACGTCAACTGTG	713	648	0	0	2	4
TAF10	ACACGTACGTCAACTGAC	24	20	0	0	0	0
TAF10	TGACGTACGTCATGGTGT	457	384	0	0	0	0
TAF10	GTACGTACGTCATGGTCA	206	112	0	0	0	0
TAF10	ACACGTACGTCATGGTAC	245	51	0	0	0	0
TAF10	ACTGCAACCATGACGTTG	352	615	0	1	0	0
TAF12	ACACGTACGTCATGACCA	205	221	0	1	1	0
TAF12	CAACGTACGTCATGACGT	405	102	1	0	0	0
TAF12	GTACGTACGTCATGACAC	20	29	0	0	0	0
TAF12	TGACGTACGTCATGACTG	77	222	1	0	0	0
TAF12	ACACGTACGTCATGTGGT	718	830	1	97	0	1
TAF12	CATGCACATGGTTGTGCA	166	183	1	0	0	0
TAF13	CATGACCAGTCATGACTG	159	201	0	0	1	0
TAF13	GTACACTGGTGTGTACGT	88	150	0	0	0	0
TAF13	CAACACTGGTGTGTACCA	68	267	0	0	0	1
TAF13	TGACACTGGTGTGTACAC	51	38	0	0	0	0
TAF13	ACTGACCAGTCATGACAC	24	33	0	0	0	0
TAF1L	CAACCAACACACACACAC	18	16	0	0	0	0
TAF1L	GTACCAACACACACACTG	318	585	2	0	0	0
TAF1L	TGACCAACACACACTGGT	1388	1089	0	0	0	2
TAF1L	GTACCAACACACACTGCA	190	177	0	0	0	0
TAF1L	GTACTGTGACACCATGCA	155	204	0	1	0	0
TAF2	GTACACACTGTGTGTGAC	39	27	0	0	0	0
TAF2	TGACACACTGTGTGTGTG	85	107	0	0	0	0
TAF2	ACACACTGGTGTGTGTGT	243	255	1	0	0	1
TAF2	TGACACTGGTGTGTGTCA	459	277	2	1	0	0
TAF2	CAACACTGGTGTGTGTAC	59	69	0	0	0	0
TAF4	ACACGTTGCAGTGTTGTG	918	687	533862	553	41	54
TAF4	CAACGTTGCAGTGTTGCA	150	253	0	0	1	0
TAF4	CAACGTTGCAGTGTACTG	241	265	0	1	0	1
TAF4	GTACGTTGCAGTGTTGGT	427	155	1	0	0	0
TAF4	TGACGTTGCAGTGTTGAC	67	38	0	0	0	0
TAGLN	GTACTGACTGTGCAACAC	35	31	0	0	1	0
TAGLN	CAACTGACTGTGCATGAC	55	60	2	0	1	0

TAGLN	TGACTGACTGTGCAACTG	955	727	9	12	0	0
TAGLN	ACACTGACTGTGCATGGT	340	201	0	0	0	0
TAGLN	TGACTGACTGTGCATGCA	371	138	3	1	0	0
TAGLN2	CATGGTACCATGGTGTTG	343	399	0	0	1	0
TAGLN2	GTTGGTACCAACTGTGTG	294	227	1	0	0	13
TAGLN2	TGTGGTACCATGGTGTGT	282	371	1	0	0	0
TAGLN2	GTTGGTACCATGGTGTCA	101	110	0	0	0	0
TAGLN2	ACTGGTACCATGGTGTAC	83	62	0	0	0	0
TAGLN2	ACTGCACACACAGTGTGT	404	610	1	0	0	0
TAL1	TGACGTACGTACGTCATG	67	150	0	0	1	0
TAL1	ACACGTACGTACGTCACA	44	36	0	0	0	0
TAL1	GTACGTACGTACGTCAAC	4	3	0	0	0	0
TAL1	ACACGTACGTACGTACGT	285	199	0	0	0	0
TAL1	TGACGTACGTACGTACCA	188	85	0	0	0	0
TAL1	TGTGCACACACAGTGTCA	220	141	0	0	0	2
TANK	ACACACTGGTTGACCAAC	25	106	0	0	0	0
TANK	CATGGTGTCATGGTTGCA	30	20	0	0	0	0
TANK	ACTGCAACCAGTTGCAGT	144	116	1	0	0	83
TANK	TGTGCAACCAGTTGCACA	16	15	0	0	0	0
TANK	CATGCAACCAGTTGCAAC	20	20	0	0	0	0
TANK	GTTGCAACCAGTTGCATG	70	66	0	1	0	0
TAP1	GTACCAACTGTGGTCACA	36	61	0	0	0	0
TAP1	ACACCAACTGTGGTCAAC	51	74	0	7	0	0
TAP1	CAACCAACTGTGGTCATG	405	408	0	1	0	0
TAP1	GTACCAACTGTGGTACGT	419	330	0	0	0	0
TAP1	CAACCAACTGTGGTACCA	446	263	1	1	0	1
TAP2	TGACCAACTGTGGTTGTG	663	432	0	0	0	6
TAP2	CAACCAACTGTGCAGTGT	711	1381	2	1	0	0
TAP2	ACACCAACTGTGCAGTCA	127	367	0	0	0	0
TAP2	GTACCAACTGTGCAGTAC	151	110	0	0	0	1
TAP2	TGACCAACTGTGCAGTTG	772	1208	2	1	0	1
TAPBP	CATGGTCAGTACCATGAC	15	2	0	0	0	0
TAPBP	GTTGGTCAGTACCATGTG	582	493	1	0	0	0
TAPBP	ACTGGTCAGTACACGTGT	247	192	0	0	0	0
TAPBP	CATGCAGTCACAACACGT	26	35	0	0	0	0
TAPBP	ACTGCAGTCACAACACCA	328	398	0	0	0	1
TAPBP	CATGCAACTGTGACCAAC	23	31	0	0	0	0
TARDBP	CAACGTACCAACCATGTG	511	243	2	0	0	2
TARDBP	TGACGTACCAACACGTGT	705	910	0	0	0	14
TARDBP	GTACGTACCAACACGTCA	229	207	0	0	0	0
TARDBP	ACACGTACCAACACGTAC	167	177	1	0	0	1
TARDBP	CAACGTACCAACACGTTG	334	470	0	0	0	0
TARS	CAACACGTCATGTGACGT	108	162	0	0	0	0
TARS	ACACACGTCATGTGACCA	173	243	0	0	0	0
TARS	GTACACGTCATGTGACAC	156	23	0	0	0	0
TARS	TGACACGTCATGTGACTG	293	416	1	4	0	1
TARS	ACACACGTCATGTGTGGT	277	386	0	0	0	5
TAT	CAACCACAACTGACACAC	68	76	0	0	0	0
TAT	GTACCACAACTGACACTG	232	1056	0	0	0	1
TAT	TGACCACAACTGACTGGT	260	185	0	1	0	0
TAT	GTACCACAACTGACTGCA	133	115	0	0	0	0
TAT	ACACCACAACTGACTGAC	41	50	1	0	0	0
TAT	CATGCACACACAGTGTAC	127	114	0	0	0	0
TBK1	CACATGACACACCATGCA	421	481	0	0	0	38
TBK1	TGCATGACACACCATGAC	28	48	0	0	0	0
TBK1	ACCATGACACACCATGTG	591	1089	0	2	0	1
TBK1	GTCATGACACACACGTGT	45	64	0	1	0	0
TBK1	CACATGACACACACGTCA	79	99	0	0	0	0
TBK1	GTTGCAACCAGTTGACCA	409	457	1	8	0	2
TBKBP1	GTTGGTCAACACTGACTG	299	741	0	0	1	0
TBKBP1	TGTGGTCAACACTGACCA	55	84	0	0	0	0
TBKBP1	CATGGTCAACACTGACAC	51	53	0	0	0	0
TBKBP1	TGTGGTCAACACTGTGGT	297	225	1	0	0	0
TBKBP1	GTTGGTCAACACTGTGCA	226	384	3	0	0	0
TBKBP1	TGTGCACAACTGCACAGT	123	141	0	2	0	0
TBL1X	ACTGGTCAGTCAACGTTG	209	365	0	0	0	10
TBL1X	CATGGTCAGTCAACCAGT	70	98	0	0	0	0
TBL1X	ACTGGTCAGTCAACCACA	157	206	0	0	0	0
TBL1X	GTTGGTCAGTCAACCAAC	7	12	0	0	0	0
TBL1X	TGTGGTCAGTCAACCATG	949	546	0	12	0	0
TBL1X	ACTGCAACCACAGTGTAC	64	48	0	0	0	0
TBL1XR1	GTACTGCAACCAGTTGGT	200	274	2	0	0	1
TBL1XR1	CAACTGCAACCAGTTGCA	127	226	0	0	0	0
TBL1XR1	TGACTGCAACCAGTTGAC	32	46	0	0	0	0
TBL1XR1	ACACTGCAACCAGTTGTG	246	124	0	0	0	0

TBL1XR1	GTACTGCAACCACAGTGT	560	533	2	0	0	0
TBP	TGACGTACGTACACCAGT	135	167	0	0	0	2
TBP	GTACGTACGTACACCACA	134	81	0	0	0	0
TBP	ACACGTACGTACACCAAC	68	62	0	0	0	0
TBP	CAACGTACGTACACCATG	551	516	275	1	0	6
TBP	TGACTGACTGACCACAGT	268	206	0	0	0	0
TBX1	ACACGTACGTACCATGGT	493	735	1	0	0	1
TBX1	TGACGTACGTACCATGCA	357	273	0	0	0	0
TBX1	CAACGTACGTACCATGAC	118	39	0	0	0	0
TBX1	ACTGCAGTGTCATGCAGT	239	273	0	0	0	0
TBX1	TGTGCAGTGTCATGCACA	81	88	0	0	0	0
TBX1	ACTGCAACGTTGACACCA	366	318	0	0	0	0
TBX2	ACACGTACGTACTGGTCA	680	942	3	0	0	1
TBX2	GTACGTACGTACTGGTAC	6	13	0	0	0	0
TBX2	TGACGTACGTACTGGTTG	388	312	0	0	0	1
TBX2	ACACGTACGTACTGCAGT	43	96	0	0	0	0
TBX2	TGACGTACGTACTGCACA	82	51	0	0	0	0
TBX21	CAACGTACACACTGGTCA	1129	991	1	11	1	0
TBX21	GTACGTACACACTGGTGT	298	536	0	0	0	0
TBX21	TGACGTACACACTGGTAC	32	46	0	0	0	0
TBX21	ACACGTACACACTGGTTG	323	676	0	1	0	0
TBX21	CAACGTACACACTGCAGT	102	158	0	0	0	0
TBX21	GTTGCACAACACACACTG	389	272	0	1	0	0
TBXA2R	GTACGTCATGTGACCAAC	28	27	0	0	0	0
TBXA2R	TGACGTCATGTGACCATG	113	107	0	0	0	0
TBXA2R	ACACGTCATGTGACACGT	643	241	0	0	0	0
TBXA2R	TGACGTCATGTGACACCA	291	234	0	0	0	0
TBXA2R	ACTGCAGTACACCACACA	54	58	0	0	0	0
TBXAS1	ACACACGTCAGTTGGTCA	841	785	1	0	0	1
TBXAS1	GTACACGTCAGTTGGTAC	54	45	0	0	0	0
TBXAS1	TGACACGTCAGTTGGTTG	490	346	0	0	0	12
TBXAS1	ACACACGTCAGTTGCAGT	56	118	0	0	0	0
TBXAS1	TGACACGTCAGTTGCACA	581	346	1	0	0	2
TBXAS1	CATGCAACGTGTGTCACA	107	245	0	0	0	0
TCEA3	ACACGTGTGTTGACGTCA	125	113	0	0	1	0
TCEA3	TGACGTGTGTTGCATGTG	275	308	1	2	0	0
TCEA3	CAACGTGTGTTGACGTGT	432	445	0	1	0	11
TCEA3	GTACGTGTGTTGACGTAC	9	4	0	0	0	0
TCEA3	TGACGTGTGTTGACGTTG	314	252	0	0	0	0
TCERG1	TGACGTACGTTGCAACCA	159	150	0	0	0	0
TCERG1	CAACGTACGTTGCAACAC	31	23	0	0	0	0
TCERG1	GTACGTACGTTGCAACTG	1115	268	0	1	0	0
TCERG1	TGACGTACGTTGCATGGT	199	163	0	0	0	0
TCERG1	GTACGTACGTTGCATGCA	119	225	0	0	0	0
TCF1	ACACGTACACCAGTCAGT	327	108	0	1	0	1
TCF1	TGACGTACACCAGTCACA	74	96	0	0	0	0
TCF1	CAACGTACACCAGTCAAC	36	39	0	0	0	0
TCF1	ACTGCAGTACACACGTAC	40	59	0	0	0	0
TCF1	CATGCATGACTGGTCAAC	26	43	0	0	0	0
TCF15	GTTGCAGTACACACCAGT	200	422	0	0	1	1
TCF15	TGACGTACCAGTACGTCA	86	85	0	0	0	0
TCF15	CAACGTACCAGTACGTAC	144	50	0	0	0	0
TCF15	CATGGTGTGTTGCAACGT	244	219	2	1	0	371
TCF15	CATGCAGTACACACGTTG	393	473	0	0	0	0
TCF3	CAACGTACACACGTCACA	133	148	1	0	1	0
TCF3	CAACGTACACACGTGTTG	465	431	14	1	0	2
TCF3	GTACGTACACACGTCAGT	115	351	0	1	0	0
TCF3	TGACGTACACACGTCAAC	9	7	0	0	0	0
TCF3	ACACGTACACACGTCATG	200	247	1	0	0	1
TCF3	TGTGCATGGTGTCAACTG	174	128	0	0	0	1
TCF4	TGACGTACGTTGTGTGTG	636	241	2	1	1	0
TCF4	GTACGTACCAGTGTGTGT	326	743	0	1	1	0
TCF4	GTACGTCAACCACAACAC	80	205	0	0	0	0
TCF4	CAACGTACCAGTGTGTCA	712	443	2	1	0	0
TCF4	TGACGTACCAGTGTGTAC	37	24	0	0	0	0
TCF7	TGACGTTGACACCATGCA	277	330	0	0	0	2
TCF7	CAACGTTGACACCATGAC	19	44	0	0	0	0
TCF7	CATGCAGTGTACCATGCA	89	146	0	0	0	0
TCF7	GTTGCAGTCATGGTACTG	110	174	0	0	0	0
TCF7	TGTGCAGTCATGGTTGGT	171	152	0	0	0	0
TCF7L1	GTACGTTGACACACGTTG	276	558	1	0	0	1
TCF7L1	TGACGTTGACACACCAGT	376	249	0	0	0	0
TCF7L1	GTACGTTGACACACCACA	96	120	0	0	0	1
TCF7L1	ACACGTTGACACACCAAC	34	106	0	1	0	1
TCF7L1	CAACGTTGACACACCATG	379	40	0	0	0	0

TCF7L1	GTTGCAACGTGTGTACAC	26	29	0	0	0	0
TCF7L2	TGACGTCAACCACATGCA	347	236	0	0	0	1
TCF7L2	ACACTGCATGCATGGTCA	163	173	0	0	0	0
TCF7L2	GTACTGCATGCATGGTAC	19	22	0	0	0	0
TCF7L2	TGACTGCATGCATGGTTG	675	509	0	0	0	0
TCF7L2	ACACTGCATGCATGCAGT	37	53	0	0	0	0
TCIRG1	GTTGCAACCACATGACTG	712	1163	2	3	1	1
TCIRG1	ACACCATGGTGTTGTGAC	68	90	0	0	0	0
TCIRG1	CAACCATGGTGTTGTGTG	314	207	0	0	0	0
TCIRG1	ACACCATGGTCAGTGTGT	272	372	0	0	0	2
TCIRG1	TGACCATGGTCAGTGTCA	391	274	1	0	0	0
TCIRG1	CAACCATGGTCAGTGTAC	154	113	1	0	0	0
TCL1A	ACTGGTGTCAACACGTGT	208	287	0	0	1	0
TCL1A	TGTGGTGTCAACACGTCA	437	390	0	0	0	0
TCL1A	CATGGTGTCAACACGTAC	3	6	0	0	0	0
TCL1A	GTTGGTGTCAACACGTTG	584	906	0	0	0	0
TCL1A	TGTGGTGTCAACACCAGT	317	323	1	0	0	0
TCOF1	ACACGTCAGTTGCACATG	228	235	3	0	0	1
TCOF1	CAACGTCAGTTGCAACGT	154	132	0	0	0	0
TCOF1	ACACGTCAGTTGCAACCA	152	200	0	0	0	0
TCOF1	GTACGTCAGTTGCAACAC	29	43	0	0	0	0
TCOF1	TGACGTCAGTTGCAACTG	198	178	0	0	0	0
TCP1	ACTGGTCACAGTGTGTTG	925	352	0	0	1	0
TCP1	GTACCAGTACCAGTGTTG	551	275	1	1	0	0
TCP1	TGACCAGTACCAGTCAGT	53	71	0	0	0	0
TCP1	GTACCAGTACCAGTCACA	38	43	0	0	0	0
TCP1	TGTGCAGTACTGGTTGTG	126	136	0	0	0	0
TDG	TGTGGTTGGTCAGTGTAC	76	117	0	1	0	0
TDG	ACTGGTTGGTCAGTGTTG	1102	1204	1	2	0	0
TDG	CATGGTTGGTCAGTCAGT	318	165	0	0	0	0
TDG	ACTGGTTGGTCAGTCACA	56	69	0	0	0	1
TDG	TGTGCAGTGTTGTGTGGT	909	174	0	1	0	0
TDGF1	ACCATGTGCATGGTGTGT	287	325	0	0	0	0
TDGF1	TGCATGTGCATGGTGTCA	413	439	0	0	0	0
TDGF1	CACATGTGCATGGTGTAC	47	66	0	0	0	0
TDGF1	GTCATGTGCATGGTGTTG	1195	259	4	1	0	5
TDGF1	TGCATGTGCATGGTCAGT	82	120	0	0	0	0
TDGF1	TGTGCACACACAGTCAGT	907	295	0	1	0	1
TDP1	TGACACCAACCACAGTTG	755	545	0	0	1	0
TDP1	ACACACCAACCACACAGT	498	521	1	1	1	0
TDP1	CAACACCAACCACAGTGT	908	438	2	2	0	3
TDP1	ACACACCAACCACAGTCA	364	333	0	2	0	0
TDP1	GTACACCAACCACAGTAC	95	104	0	0	0	0
TEAD2	ACACGTCAACACACGTTG	362	748	1	0	1	3
TEAD2	ACACGTCAACACCATGTG	1036	553	0	0	0	0
TEAD2	GTACGTCAACACACGTGT	947	1117	1	1	0	0
TEAD2	CAACGTCAACACACGTCA	138	220	0	0	0	0
TEAD2	TGACGTCAACACACGTAC	57	30	0	1	0	0
TEAD2	GTTGCAACCAACGTGTAC	67	27	0	0	0	0
TEC	ACTGGTACTGACCACAAC	4	12	0	0	0	0
TEC	CATGGTACTGACCACATG	87	99	0	0	0	0
TEC	GTTGGTACTGACCAACGT	143	124	0	0	0	0
TEC	CATGGTACTGACCAACCA	279	257	0	0	0	0
TEC	TGTGGTACTGACCAACAC	54	68	1	0	0	0
TEK	ACCATGCACATGGTGTTG	70	138	0	0	0	0
TEK	CACATGCACATGGTCAGT	111	40	0	0	0	0
TEK	ACCATGCACATGGTCACA	193	164	0	0	0	0
TEK	GTCATGCACATGGTCAAC	87	38	0	0	0	0
TEK	TGCATGCACATGGTCATG	135	248	0	0	0	0
TEP1	ACACCATGCACACAGTGT	817	792	1	1	3	0
TEP1	CATGCAACACCAGTACTG	321	354	0	0	1	0
TEP1	GTACCATGCACAGTTGTG	853	447	0	1	0	1
TEP1	TGACCATGCACACAGTCA	125	157	0	0	0	0
TEP1	CAACCATGCACACAGTAC	63	137	0	0	0	0
TEP1	GTACCATGCACACATGAC	59	77	1	2	0	2
TERF1	TGACCATGACGTCAGTTG	546	412	4	0	0	0
TERF1	ACACCATGACGTCACAGT	221	276	1	0	0	0
TERF1	TGACCATGACGTCACACA	136	188	0	0	0	2
TERF1	CAACCATGACGTCACAAC	26	51	0	0	0	0
TERF1	GTACCATGACGTCACATG	201	267	1	0	0	0
TERF1	CATGCACATGGTTGGTGT	128	192	1	0	0	0
TERF2IP	ACACCATGCACAGTTGGT	96	133	2	0	1	1
TERF2IP	TGACCATGCACAGTTGCA	106	125	0	0	0	0
TERF2IP	CAACCATGCACAGTTGAC	23	22	0	0	0	1
TERF2IP	CAACCATGCACACATGGT	147	239	0	0	0	0

TERF2IP	ACACCATGCACACATGCA	202	168	1	0	0	0
TERT	ACTGGTTGACTGCACAGT	35	31	0	0	0	1
TERT	TGTGGTTGACTGCACACA	185	454	0	1	0	0
TERT	CATGGTTGACTGCACAAC	56	103	0	0	0	0
TERT	GTTGGTTGACTGCACATG	77	102	1	0	0	0
TERT	TGTGGTTGACTGCAACGT	200	275	1	0	0	0
TF	ACTGCAACACCACAGTTG	2079	1719	133	1	1	12
TF	GTACACCAGTCATGCAAC	23	32	0	0	0	0
TF	TGACACCAGTCATGCATG	501	399	1	1	0	0
TF	ACACACCAGTCATGACGT	555	305	0	0	0	0
TF	TGACACCAGTCATGACCA	472	346	0	0	0	0
TF	CAACACCAGTCATGACAC	27	34	0	1	0	0
TFAM	GTTGCACACACAGTGTTG	1720	752	2	1	1	0
TFAM	ACACGTACCAACTGGTGT	702	808	6	0	0	0
TFAM	TGACGTACCAACTGGTCA	977	741	2	0	0	0
TFAM	CAACGTACCAACTGGTAC	11	17	0	0	0	0
TFAM	GTACGTACCAACTGGTTG	317	377	2	0	0	2
TFAM	TGACGTACCAACTGCAGT	70	97	0	1	0	0
TFAP2A	CACATGTGCATGACGTCA	237	334	1	0	0	1
TFAP2A	TGCATGTGCATGACGTAC	54	35	0	0	0	0
TFAP2A	ACCATGTGCATGACGTTG	303	521	0	1	0	0
TFAP2A	CACATGTGCATGACCAGT	333	380	0	1	0	0
TFAP2A	ACCATGTGCATGACCACA	242	223	0	0	0	0
TFAP2B	ACACGTTGGTGTTGGTCA	236	341	1	0	0	0
TFAP2B	GTACGTTGGTGTTGGTAC	80	72	0	0	0	0
TFAP2B	TGACGTTGGTGTTGGTTG	291	308	0	0	0	0
TFAP2B	ACACGTTGGTGTTGCAGT	165	100	0	0	0	0
TFAP2B	CATGACACCAACCATGAC	144	135	0	0	0	0
TFAP2C	CAACGTTGGTCAACGTGT	131	145	0	0	0	0
TFAP2C	ACACGTTGGTCAACGTCA	99	49	0	0	0	0
TFAP2C	GTACGTTGGTCAACGTAC	31	43	0	0	0	0
TFAP2C	TGACGTTGGTCAACGTTG	870	536	0	0	0	0
TFAP2C	ACACGTTGGTCAACCAGT	247	86	0	0	0	0
TFCP2	ACACGTTGGTCATGACGT	4	11	0	0	0	0
TFCP2	TGACGTTGGTCATGACCA	161	134	0	1	0	0
TFCP2	CAACGTTGGTCATGACAC	47	64	0	0	0	0
TFCP2	GTACGTTGGTCATGACTG	917	461	2	0	0	0
TFCP2	TGACGTTGGTCATGTGGT	638	544	1	0	0	0
TFDP1	GTACGTTGGTACGTACCA	98	64	0	0	0	0
TFDP1	ACACGTTGGTACGTACAC	29	81	0	0	0	0
TFDP1	CAACGTTGGTACGTACTG	487	456	1	0	0	0
TFDP1	TGTGCAGTGTTGTGGTAC	125	119	0	0	0	0
TFDP1	ACTGCAGTGTTGTGGTTG	532	430	2	0	0	0
TFDP2	CAACGTTGGTACCAGTCA	153	344	1	0	0	0
TFDP2	TGACGTTGGTACCAGTAC	81	235	0	0	0	0
TFDP2	ACACGTTGGTACCAGTTG	297	282	0	0	0	0
TFDP2	CAACGTTGGTACCACAGT	275	74	1	0	0	0
TFDP2	ACTGGTGTCAGTACCAGT	142	128	1	0	0	0
TFDP2	ACTGCACAACCAGTACGT	456	421	6	0	0	0
TFEB	ACACGTCAACACGTGTGT	188	200	0	0	0	0
TFEB	TGACGTCAACACGTGTCA	275	236	61	1	0	0
TFEB	CAACGTCAACACGTGTAC	32	45	0	0	0	0
TFEB	GTACGTCAACACGTGTTG	261	155	0	0	0	0
TFEB	TGACGTCAACACGTCAGT	79	47	0	0	0	0
TFEB	ACTGCAACTGCAGTCACA	16	37	0	0	0	1
TFPI	ACACTGTGGTGTTGACTG	199	515	0	0	1	0
TFPI	GTACTGTGGTGTTGACGT	284	90	0	0	0	0
TFPI	CAACTGTGGTGTTGACCA	142	203	0	0	0	0
TFPI	TGACTGTGGTGTTGACAC	90	83	0	0	0	0
TFPI	CAACTGTGGTGTTGTGGT	640	374	2	0	0	1
TFR2	ACACTGACCAGTACGTAC	15	42	0	0	0	0
TFR2	CAACTGACCAGTACGTTG	234	204	1	0	0	0
TFR2	GTACTGACCAGTACCAGT	35	41	0	0	0	0
TFR2	CAACTGACCAGTACCACA	65	69	0	0	0	1
TFR2	TGACTGACCAGTACCAAC	83	107	0	0	0	0
TFRC	ACACTGGTGTCATGACTG	503	376	1	0	0	9
TFRC	CAACTGGTGTCATGTGGT	44	143	0	0	0	0
TFRC	ACACTGGTGTCATGTGCA	268	214	0	0	0	0
TFRC	GTACTGGTGTCATGTGAC	51	99	0	0	0	0
TFRC	TGTGCAGTCACAACGTGT	142	189	0	0	0	0
TFRC	GTTGCACACACAGTCACA	90	188	0	1	0	0
TGDS	ACACACACTGGTACTGTG	915	319	11	0	6	26
TGDS	CAACACACTGGTACACTG	57	132	0	0	0	0
TGDS	GTACACACTGGTACTGGT	254	183	2	0	0	0
TGDS	CAACACACTGGTACTGCA	19	19	0	0	0	0

TGDS	TGACACACTGGTACTGAC	187	87	0	0	0	1
TGFA	ACACGTGTGTCAACCAAC	35	49	0	0	0	0
TGFA	CAACGTGTGTCAACCATG	220	237	0	0	0	6
TGFA	GTACGTGTGTCAACACGT	5	20	0	0	0	0
TGFA	CAACGTGTGTCAACACCA	520	204	0	0	0	0
TGFA	TGACGTGTGTCAACACAC	2	12	0	0	0	0
TGFB1	GTCATGACACTGGTTGTG	812	415	0	1	1	0
TGFB1	ACCATGACACTGGTTGGT	120	96	0	0	0	0
TGFB1	TGCATGACACTGGTTGCA	100	126	0	0	0	0
TGFB1	CACATGACACTGGTTGAC	41	43	0	0	0	0
TGFB1	ACCATGACACTGCAGTGT	165	445	1	0	0	1
TGFB1I1	CAACGTTGGTTGGTACCA	53	52	0	0	0	0
TGFB1I1	TGACGTTGGTTGGTACAC	42	49	0	0	0	1
TGFB1I1	ACACGTTGGTTGGTACTG	377	365	33	0	0	0
TGFB1I1	CAACGTTGGTTGGTTGGT	69	93	0	0	0	0
TGFB1I1	ACACGTTGGTTGGTTGCA	131	154	0	0	0	0
TGFB2	ACACCACAGTACGTGTGT	56	97	0	0	0	0
TGFB2	TGACCACAGTACGTGTCA	548	420	0	8	0	0
TGFB2	CAACCACAGTACGTGTAC	96	73	0	0	0	0
TGFB2	GTACCACAGTACGTGTTG	918	719	0	0	0	0
TGFB2	TGACCACAGTACGTCAGT	342	362	0	0	0	1
TGFB2	ACTGCACACACAGTCAAC	19	19	0	0	0	0
TGFB3	ACACTGCAGTGTCACAAC	23	41	0	0	0	0
TGFB3	CAACTGCAGTGTCACATG	358	548	0	0	0	2
TGFB3	GTACTGCAGTGTCAACGT	142	117	0	0	0	0
TGFB3	CAACTGCAGTGTCAACCA	353	474	0	0	0	0
TGFB3	ACTGCAGTTGACTGGTCA	296	473	0	0	0	1
TGFB3	CATGCACACACAGTCATG	106	115	0	0	0	5
TGFBI	TGACTGCATGTGGTCAAC	0	2	0	0	0	0
TGFBI	ACACTGCATGTGGTCATG	106	98	0	0	0	0
TGFBI	CAACTGCATGTGGTACGT	426	511	0	0	0	1
TGFBI	ACACTGCATGTGGTACCA	622	449	3	0	0	171
TGFBI	GTACTGCATGTGGTACAC	42	82	0	0	0	0
TGFBI	GTTGCACACACAGTACGT	295	255	0	0	0	2
TGFBR1	ACACCATGCAGTTGCAGT	698	359	1	1	4	1
TGFBR1	GTACCATGCAGTTGGTAC	23	29	2	3	1	5478
TGFBR1	CAACCATGCAGTTGGTGT	332	698	0	0	0	0
TGFBR1	ACACCATGCAGTTGGTCA	273	303	0	0	0	1
TGFBR1	TGACCATGCAGTTGGTTG	937	1591	5	2	0	0
TGFBR2	ACCATGCATGGTGTTGGT	71	62	0	0	0	0
TGFBR2	TGCATGCATGGTGTTGCA	22	23	0	0	0	24
TGFBR2	CACATGCATGGTGTTGAC	8	8	0	0	0	0
TGFBR2	GTCATGCATGGTGTTGTG	123	155	0	0	0	0
TGFBR2	ACCATGCATGGTCAGTGT	612	363	0	0	0	0
TGFBR3	CAACCAGTTGTGGTACAC	26	16	0	0	0	0
TGFBR3	GTACCACAGTACGTCACA	89	122	0	0	0	0
TGFBR3	ACACCACAGTACGTCAAC	20	35	0	0	0	0
TGFBR3	CAACCACAGTACGTCATG	287	236	11	0	0	0
TGFBR3	GTACCACAGTACGTACGT	60	105	0	0	0	0
TGFBRAP1	ACCATGTGACTGACACTG	24	81	0	0	1	0
TGFBRAP1	TGCATGTGACTGACACAC	42	25	0	0	0	0
TGFBRAP1	CACATGTGACTGACTGGT	717	489	1	0	0	0
TGFBRAP1	ACCATGTGACTGACTGCA	142	167	0	1	0	0
TGFBRAP1	GTCATGTGACTGACTGAC	47	40	0	0	0	0
TGIF1	CAACGTTGGTTGCAACTG	525	645	2	1	1	10
TGIF1	GTACGTTGGTTGCACATG	704	277	0	0	0	0
TGIF1	TGACGTTGGTTGCAACGT	93	128	0	0	0	0
TGIF1	GTACGTTGGTTGCAACCA	829	608	0	1	0	0
TGIF1	ACACGTTGGTTGCAACAC	47	36	0	0	0	0
TGIF1	ACTGCATGGTGTTGTGTG	92	79	1	0	0	0
TGIF2	ACACGTACACTGGTCACA	56	267	0	0	0	0
TGIF2	GTACGTACACTGGTCAAC	47	23	0	1	0	0
TGIF2	TGACGTACACTGGTCATG	70	173	0	0	0	0
TGIF2	ACACGTACACTGGTACGT	227	193	1	0	0	0
TGIF2	ACTGGTTGACTGTGACCA	259	106	0	0	0	1
TGM1	CAACCAACCAGTACGTGT	243	319	0	0	0	0
TGM1	ACACCAACCAGTACGTCA	34	58	0	0	0	0
TGM1	GTACCAACCAGTACGTAC	106	101	0	0	0	3
TGM1	TGACCAACCAGTACGTTG	520	136	1	0	0	0
TGM1	ACACCAACCAGTACCAGT	729	722	1	0	0	1
TGM2	TGCATGCACACAACCAAC	56	86	0	0	0	0
TGM2	GTTGCAGTGTTGACACCA	204	231	0	0	0	0
TGM2	ACTGCAGTGTTGACACAC	58	45	0	0	0	4
TGM2	CATGCAGTGTTGACACTG	431	683	0	0	0	0
TGM2	TGTGCATGTGCACAACCA	141	166	0	0	0	0

TGM3	ACACCAACCAGTACTGTG	866	626	0	0	0	0
TGM3	GTACCAACCAGTTGGTGT	279	337	0	0	0	0
TGM3	CAACCAACCAGTTGGTCA	645	788	1	2	0	1
TGM3	TGACCAACCAGTTGGTAC	37	57	0	0	0	0
TGM3	GTTGCATGTGCACAACTG	540	310	1	2	0	92
TH	TGACACTGCATGACCAAC	257	82	0	0	0	0
TH	ACACACTGCATGACCATG	165	237	0	0	0	1
TH	ACTGCAGTTGCATGGTAC	29	49	0	0	0	0
TH	ACTGACCAACACTGACTG	205	403	1	0	0	0
TH	CATGACCAACACTGTGGT	458	492	0	0	0	1
THBD	CAACACTGGTACCAACCA	298	429	1	0	0	0
THBD	TGACACTGGTACCAACAC	55	57	0	0	0	0
THBD	ACACACTGGTACCAACTG	513	411	0	1	0	0
THBD	CAACACTGGTACCATGGT	847	499	0	0	0	1
THBD	TGTGCAGTTGCAACACCA	185	156	0	0	0	0
THBS1	TGCATGCACACACACATG	452	688	0	1	0	0
THBS1	ACCATGCACACACAACGT	176	161	0	0	0	9
THBS1	TGCATGCACACACAACCA	524	483	0	1	0	0
THBS1	CACATGCACACACAACAC	90	88	0	0	0	0
THBS1	GTCATGCACACACAACTG	107	188	0	0	0	0
THBS2	GTACACTGGTACACCATG	92	178	0	0	0	2
THBS2	TGACACTGGTACACACGT	33	40	0	0	0	0
THBS2	GTACACTGGTACACACCA	90	279	1	0	0	2
THBS2	TGTGCAGTTGCAACTGGT	247	317	0	0	0	0
THBS2	TGTGCATGTGACCATGTG	269	171	0	0	0	4
THOC4	TGCATGCACAACCAGTCA	673	717	0	0	0	0
THOC4	CACATGCACAACCAGTAC	212	274	1	0	0	0
THOC4	GTCATGCACAACCAGTTG	228	246	1	0	0	0
THOC4	TGCATGCACAACCACAGT	223	203	0	0	0	0
THOC4	TGTGCAGTCATGACTGTG	797	439	0	1	0	0
THOP1	ACACACCAGTGTGTCACA	21	21	0	0	0	0
THOP1	GTACACCAGTGTGTCAAC	2	2	0	0	0	0
THOP1	TGACACCAGTGTGTCATG	602	177	0	0	0	0
THOP1	ACACACCAGTGTGTACGT	56	73	0	0	0	0
THOP1	TGACACCAGTGTGTACCA	124	201	0	0	0	0
THPO	ACACTGGTTGACCACACA	328	333	0	0	2	1
THPO	TGACTGGTTGACCAGTAC	285	131	0	0	0	0
THPO	ACACTGGTTGACCAGTTG	127	279	1	0	0	0
THPO	CAACTGGTTGACCACAGT	122	121	0	0	0	0
THPO	GTACTGGTTGACCACAAC	22	19	0	0	0	0
THRA	ACACGTTGCAGTCACACA	98	83	0	0	0	0
THRA	GTACGTTGCAGTCACAAC	41	66	0	0	0	0
THRA	TGACGTTGCAGTCACATG	151	175	1	0	0	1
THRA	ACACGTTGCAGTCAACGT	736	454	6	1	0	0
THRA	TGACGTTGCAGTCAACCA	129	134	0	0	0	0
THRB	GTACGTTGCAGTACTGTG	158	203	0	0	2	0
THRB	CAACGTTGCAGTACTGAC	72	185	0	0	0	0
THRB	ACACGTTGCAGTTGGTGT	605	791	0	0	0	0
THRB	TGACGTTGCAGTTGGTCA	137	173	0	0	0	0
THRB	CAACCAGTCAACACTGTG	304	284	2	0	0	0
THTPA	ACACACACCAGTTGGTAC	46	55	0	0	0	0
THTPA	CAACACACCAGTTGGTTG	1079	1643	2	1	0	1
THTPA	GTACACACCAGTTGCAGT	203	387	1	0	0	1
THTPA	CAACACACCAGTTGCACA	382	187	0	0	0	0
THTPA	TGTGGTACTGTGTGCACA	296	109	0	0	0	0
THY1	ACACACTGACACTGACCA	58	63	0	0	1	4
THY1	CAACACTGACACTGACGT	204	94	1	0	0	0
THY1	GTACACTGACACTGACAC	59	31	0	1	0	0
THY1	TGACACTGACACTGACTG	129	141	0	0	0	2
THY1	ACACACTGACACTGTGGT	278	325	1	0	0	0
THY1	ACTGCAACCACACATGCA	162	134	0	1	0	0
TIAM1	ACACCATGGTTGACTGGT	127	126	0	0	0	1
TIAM1	TGACCATGGTTGACTGCA	13	62	0	0	0	0
TIAM1	CAACCATGGTTGACTGAC	122	231	1	0	0	0
TIAM1	ACTGCAGTGTACACACGT	283	284	1	0	0	35
TIAM1	TGTGCAGTGTACACACCA	107	199	1	0	0	0
TICAM1	ACTGGTCACACATGACGT	374	275	0	0	1	0
TICAM1	ACTGGTCACACATGCACA	117	131	0	0	0	1
TICAM1	GTTGGTCACACATGCAAC	8	20	0	0	0	0
TICAM1	TGTGGTCACACATGCATG	60	89	0	0	0	0
TICAM1	TGTGCAGTCATGCAACTG	489	590	0	1	0	0
TICAM2	GTACACTGACGTACGTCA	241	281	0	1	0	0
TICAM2	ACACACTGACGTACGTAC	74	158	0	0	0	0
TICAM2	CAACACTGACGTACGTTG	112	76	0	0	0	0
TICAM2	GTACACTGACGTACCAGT	8	5	0	0	0	0

TICAM2	CAACACTGACGTACCACA	25	18	0	0	0	0
TIFA	ACTGGTCACATGGTGTGT	1542	1356	240	1	1	6
TIFA	TGTGGTCACAACTGTGTG	180	326	1	0	0	1
TIFA	TGTGGTCACATGGTGTCA	506	551	0	0	0	0
TIFA	CATGGTCACATGGTGTAC	20	48	0	0	0	0
TIFA	GTTGGTCACATGGTGTTG	810	1140	1	2	0	3
TIMELESS	ACTGGTACCAGTTGGTGT	535	556	3	1	0	0
TIMELESS	TGTGGTACCAGTTGGTCA	201	445	0	0	0	1
TIMELESS	CATGGTACCAGTTGGTAC	123	117	1	0	0	0
TIMELESS	GTTGGTACCAGTTGGTTG	389	269	1	0	0	34
TIMELESS	TGTGGTACCAGTTGCAGT	149	210	0	0	0	0
TIMP1	CAACACACTGGTGTACGT	481	132	0	0	0	0
TIMP1	ACACACACTGGTGTACCA	386	624	0	1	0	3
TIMP1	GTACACACTGGTGTACAC	7	0	0	0	0	0
TIMP1	TGACACACTGGTGTACTG	217	362	0	0	0	0
TIMP1	GTTGCAGTCATGTGGTAC	85	68	0	0	0	0
TIMP2	TGACACACTGGTGTTGCA	205	280	0	0	1	3
TIMP2	ACACACACTGGTGTTGGT	362	392	0	0	0	0
TIMP2	CAACACACTGGTGTTGAC	12	28	0	0	0	0
TIMP2	GTACACACTGGTGTTGTG	79	47	0	1	0	0
TIMP2	ACTGCAGTCATGTGGTCA	179	193	1	0	0	0
TIMP3	CAACACACACTGTGTGAC	130	59	1	0	2	2
TIMP3	GTACACACACTGTGTGTG	171	211	0	0	0	0
TIMP3	TGACACACTGGTGTGTGT	201	269	0	0	0	0
TIMP3	GTACACACTGGTGTGTCA	130	93	1	0	0	0
TIMP3	ACACACACTGGTGTGTAC	10	3	0	0	0	0
TIMP4	GTACACACTGGTCAGTTG	2183	2130	5	2	3	8
TIMP4	ACACACACTGGTCAGTGT	651	359	1	0	0	0
TIMP4	TGACACACTGGTCAGTCA	357	301	0	1	0	3
TIMP4	CAACACACTGGTCAGTAC	48	74	0	0	0	0
TIMP4	TGACACACTGGTCACAGT	140	159	1	0	0	1
TIRAP	CACATGTGACACTGCAGT	175	241	0	1	0	0
TIRAP	ACCATGTGACACTGCACA	192	382	0	0	0	0
TIRAP	GTCATGTGACACTGCAAC	79	52	0	0	0	1
TIRAP	TGCATGTGACACTGCATG	404	374	0	0	0	0
TIRAP	ACTGCAGTACCAGTTGGT	707	743	0	0	0	1
TIRAP	CATGCAACACTGCAACGT	214	233	0	0	0	0
TKT	CAACACTGGTTGGTCAGT	71	78	0	0	0	0
TKT	ACACACTGGTTGGTCACA	18	25	0	0	0	0
TKT	GTACACTGGTTGGTCAAC	59	59	0	1	0	0
TKT	TGACACTGGTTGGTCATG	747	486	0	0	0	0
TKT	GTTGCAGTTGCAGTGTTG	79	153	0	0	0	0
TKT	CATGCACACACAGTACCA	101	93	0	0	0	0
TKTL1	ACTGACGTTGACCAGTGT	379	330	0	0	1	1
TKTL1	ACACACTGCAGTCAGTGT	290	391	1	0	0	0
TKTL1	TGACACTGCAGTCAGTCA	33	68	0	0	0	0
TKTL1	CAACACTGCAGTCAGTAC	196	226	0	0	0	0
TKTL1	GTACACTGCAGTCAGTTG	1554	924	1	1	0	0
TKTL2	TGTGGTACACGTTGCATG	111	257	0	0	0	0
TKTL2	ACTGGTACACGTTGACGT	315	329	0	0	0	46
TKTL2	TGTGGTACACGTTGACCA	69	64	0	0	0	0
TKTL2	CATGGTACACGTTGACAC	193	95	1	0	0	0
TKTL2	GTTGGTACACGTTGACTG	98	128	1	0	0	0
TLE1	TGACGTTGGTGTCAACTG	227	207	0	1	0	0
TLE1	ACACGTTGGTGTCATGGT	78	97	0	0	0	0
TLE1	TGACGTTGGTGTCATGCA	31	64	0	0	0	0
TLE1	CAACGTTGGTGTCATGAC	26	39	1	0	0	2
TLE1	GTACGTTGGTGTCATGTG	96	93	0	0	0	0
TLK1	GTACGTGTCAACGTTGTG	34	63	1	0	0	0
TLK1	ACACGTGTCAACCAGTGT	383	440	2	1	0	0
TLK1	TGACGTGTCAACCAGTCA	63	108	1	0	0	0
TLK1	CAACGTGTCAACCAGTAC	141	176	0	0	0	0
TLK1	GTACGTGTCAACCAGTTG	665	412	2	0	0	2
TLN1	TGTGGTCACACACACAGT	399	234	1	1	0	0
TLN1	GTTGGTCACACACACACA	44	50	0	0	0	0
TLN1	ACTGGTCACACACACAAC	28	35	0	0	0	0
TLN1	CATGGTCACACACACATG	90	115	0	0	0	0
TLN1	GTTGGTCACACACAACGT	142	206	1	0	0	0
TLN1	CATGCAACGTACACACCA	121	57	0	0	0	0
TLR1	GTACACTGACGTCATGCA	1019	262	0	0	0	0
TLR1	ACACACTGACGTCATGAC	9	14	0	0	0	0
TLR1	CAACACTGACGTCATGTG	412	472	3	0	0	1
TLR1	TGACACTGACGTACGTGT	913	442	0	2	0	0
TLR1	TGTGCAGTTGACCAGTGT	336	386	3	0	0	0
TLR10	ACACACTGACCATGCAGT	311	77	1	1	0	1

TLR10	TGACACTGACCATGCACA	87	84	0	0	0	0
TLR10	CAACACTGACCATGCAAC	57	46	0	0	0	5
TLR10	GTACACTGACCATGCATG	101	89	0	0	0	0
TLR10	TGACACTGACCATGACGT	200	192	1	0	0	0
TLR2	CAACACTGACACTGGTTG	232	486	2	0	0	37
TLR2	GTACACTGACACTGCAGT	628	565	1	1	0	0
TLR2	CAACACTGACACTGCACA	129	89	0	0	0	0
TLR2	TGACACTGACACTGCAAC	17	27	0	0	0	0
TLR2	ACACACTGACACTGCATG	263	471	0	1	0	0
TLR3	TGACACTGACCACACAAC	32	15	0	0	0	0
TLR3	ACACACTGACCACACATG	234	175	0	0	0	0
TLR3	CAACACTGACCACAACGT	470	351	1	0	0	0
TLR3	ACACACTGACCACAACCA	513	407	2	0	0	0
TLR3	GTACACTGACCACAACAC	75	76	0	0	0	1
TLR3	TGTGCAACCAGTACACAC	106	231	0	0	0	0
TLR4	TGACACTGACCAACTGTG	186	281	0	0	0	0
TLR4	CAACACTGACCATGGTGT	89	113	0	0	0	0
TLR4	ACACACTGACCATGGTCA	90	241	0	0	0	0
TLR4	GTACACTGACCATGGTAC	210	98	0	0	0	0
TLR4	TGACACTGACCATGGTTG	457	367	1	0	0	0
TLR5	CAACACTGACACGTTGGT	508	798	1	0	0	0
TLR5	ACACACTGACACGTTGCA	255	215	0	0	0	1
TLR5	GTACACTGACACGTTGAC	41	45	0	0	0	0
TLR5	TGACACTGACACGTTGTG	127	447	1	0	0	0
TLR5	CAACACTGACACCAGTGT	291	438	1	0	0	0
TLR6	ACTGCAACCAACCAACGT	1587	1000	1	2	2	1
TLR6	GTACACTGACGTGTTGGT	85	100	0	0	0	0
TLR6	CAACACTGACGTGTTGCA	80	55	0	0	0	0
TLR6	TGACACTGACGTGTTGAC	69	48	0	0	0	0
TLR6	ACACACTGACGTGTTGTG	118	140	0	1	0	0
TLR6	GTACACTGACGTCAGTGT	248	223	0	0	0	0
TLR7	TGTGCAGTTGACCAACTG	738	812	2	0	3	6
TLR7	CAACACTGACACCATGCA	72	121	0	0	0	0
TLR7	TGACACTGACACCATGAC	133	111	4	0	0	0
TLR7	ACACACTGACACCATGTG	660	760	2	0	0	0
TLR7	ACTGCAGTTGACCATGGT	457	618	0	1	0	2
TLR8	ACACACTGACACCAGTCA	395	206	0	0	0	0
TLR8	GTACACTGACACCAGTAC	109	102	0	1	0	0
TLR8	TGACACTGACACCAGTTG	436	446	7	0	0	0
TLR8	TGACTGACTGCAACCAAC	140	113	1	0	0	0
TLR8	CATGCAACCAACACACGT	56	49	0	0	0	0
TLR8	ACTGCAACCAACACACCA	298	421	0	0	0	1
TLR9	TGACACTGACCAACACAC	74	63	0	0	0	0
TLR9	ACACACTGACCAACACTG	391	317	0	0	0	0
TLR9	CAACACTGACCAACTGGT	420	581	0	1	0	8
TLR9	ACACACTGACCAACTGCA	135	135	0	0	0	0
TLR9	GTACACTGACCAACTGAC	12	23	0	0	0	0
TLR9	GTTGCAACCAACACACAC	182	68	0	0	0	0
TLX1	ACACGTACGTTGTGCAAC	105	17	0	0	1	3
TLX1	CAACGTACGTTGTGCATG	116	123	0	0	0	0
TLX1	GTACGTACGTTGTGACGT	86	74	0	0	0	0
TLX1	CAACGTACGTTGTGACCA	406	166	0	0	0	0
TLX1	TGACGTACGTTGTGACAC	43	63	0	0	0	0
TLX1	CATGCAACCACACATGGT	155	174	0	0	0	1
TLX3	TGACGTACACTGGTGTAC	11	13	0	0	0	0
TLX3	ACACGTACACTGGTGTTG	611	978	0	0	0	0
TLX3	CAACGTACACTGGTCAGT	677	476	1	0	0	0
TLX3	GTACTGACTGACGTCAGT	271	138	0	1	0	0
TLX3	TGTGACGTCATGCAACAC	33	32	0	0	0	0
TM2D1	GTTGGTTGACGTTGTGGT	649	440	220	0	0	0
TM2D1	CATGGTTGACGTTGTGCA	379	359	0	0	0	0
TM2D1	TGTGGTTGACGTTGTGAC	19	28	0	0	0	0
TM2D1	ACTGGTTGACGTTGTGTG	388	309	0	0	0	0
TM2D1	TGTGGTTGACCAGTGTGT	222	295	0	1	0	1
TM2D1	TGTGCAACGTTGCAGTAC	49	79	0	0	0	0
TMED10	ACACCAGTACCAGTGTGT	611	905	1	0	1	2
TMED10	CAACCAGTACGTTGTGTG	656	686	0	0	0	1
TMED10	TGACCAGTACCAGTGTCA	73	67	0	0	0	0
TMED10	CAACCAGTACCAGTGTAC	145	116	0	0	0	0
TMED10	GTTGCAGTGTTGGTGTCA	352	394	0	0	0	0
TMEM166	ACTGGTCAACCACATGAC	76	332	0	0	1	0
TMEM166	CATGGTCAACCACAACAC	108	70	0	0	0	4
TMEM166	GTTGGTCAACCACAACTG	116	136	0	0	0	0
TMEM166	TGTGGTCAACCACATGGT	230	681	0	1	0	2
TMEM166	GTTGGTCAACCACATGCA	152	94	0	0	0	0

TMEM166	CATGCAACGTGTACCAAC	79	65	0	0	0	0
TNC	GTTGGTACCATGACGTAC	76	43	0	0	0	0
TNC	TGTGGTACCATGACGTTG	700	499	0	0	0	0
TNC	ACTGGTACCATGACCAGT	226	227	1	1	0	1
TNC	TGTGGTACCATGACCACA	192	168	1	26	0	0
TNC	CATGGTACCATGACCAAC	59	186	0	0	0	33
TNC	CATGCACAGTTGCAGTTG	318	407	0	0	0	0
TNF	CACATGACTGTGACTGTG	187	260	0	0	0	0
TNF	TGCATGACTGTGTGGTGT	258	275	2	0	0	0
TNF	GTCATGACTGTGTGGTCA	48	69	0	0	0	0
TNF	ACCATGACTGTGTGGTAC	66	63	0	0	0	1
TNF	TGTGCAGTACGTTGACGT	213	317	2	0	0	0
TNF	TGTGCAACTGACTGTGCA	70	88	0	0	0	0
TNFAIP1	GTTGGTACCATGCAGTTG	470	554	0	0	0	6
TNFAIP1	TGTGGTACCATGCACAGT	71	259	0	0	0	0
TNFAIP1	GTTGGTACCATGCACACA	152	158	0	0	0	1
TNFAIP1	ACTGGTACCATGCACAAC	17	29	0	0	0	1
TNFAIP1	CATGGTACCATGCACATG	258	292	1	0	0	0
TNFAIP1	CATGCAACTGTGGTCACA	53	72	0	0	0	0
TNFAIP3	ACACACACGTTGTGACGT	50	77	0	0	0	0
TNFAIP3	TGACACACGTTGTGACCA	489	248	0	0	0	0
TNFAIP3	CAACACACGTTGTGACAC	18	19	0	0	0	0
TNFAIP3	GTACACACGTTGTGACTG	137	30	0	1	0	0
TNFAIP3	TGACACACGTTGTGTGGT	269	241	0	0	0	0
TNFAIP3	TGTGCAACTGTGGTCAAC	37	47	0	0	0	0
TNFAIP6	CATGCACAGTGTACGTCA	255	305	0	1	1	3
TNFAIP6	ACTGCAACTGTGGTCATG	258	217	1	2	1	0
TNFAIP6	TGTGGTGTACACCAACGT	186	189	0	0	0	0
TNFAIP6	GTTGGTGTACACCAACCA	173	197	0	0	0	1
TNFAIP6	ACTGGTGTACACCAACAC	62	31	0	0	0	0
TNFAIP6	TGTGCACAGTGTACGTAC	26	20	0	0	0	0
TNFRSF10A	ACCATGTGTGCACAACCA	290	327	1	0	1	0
TNFRSF10A	CACATGTGTGCACAACGT	113	165	0	0	0	0
TNFRSF10A	GTCATGTGTGCACAACAC	7	8	1	0	0	0
TNFRSF10A	TGCATGTGTGCACAACTG	453	690	1	0	0	0
TNFRSF10A	ACCATGTGTGCACATGGT	130	142	0	0	0	0
TNFRSF10A	CATGCAACACGTGTTGGT	635	551	2	0	0	0
TNFRSF10B	CACATGTGTGCACAGTTG	611	818	2	2	0	0
TNFRSF10B	GTCATGTGTGCACACAGT	254	163	1	0	0	0
TNFRSF10B	CACATGTGTGCACACACA	273	208	0	0	0	0
TNFRSF10B	TGCATGTGTGCACACAAC	27	37	0	1	0	0
TNFRSF10B	ACCATGTGTGCACACATG	142	225	0	0	0	0
TNFRSF10B	GTTGCAACACTGCACAGT	146	189	0	0	0	0
TNFRSF10D	CAACTGGTTGTGACTGTG	44	98	0	1	0	0
TNFRSF10D	TGACTGGTTGTGTGGTGT	973	1817	1	1	0	0
TNFRSF10D	GTACTGGTTGTGTGGTCA	708	689	0	0	0	0
TNFRSF10D	ACACTGGTTGTGTGGTAC	157	170	1	0	0	0
TNFRSF10D	CAACTGGTTGTGTGGTTG	1188	503	1	3	0	0
TNFRSF11A	TGCATGACACTGACCACA	407	324	7	5	1	5
TNFRSF11A	CACATGACACTGACCAAC	13	10	0	0	0	0
TNFRSF11A	GTCATGACACTGACCATG	622	161	1	1	0	0
TNFRSF11A	TGCATGACACTGACACGT	21	12	0	0	0	0
TNFRSF11A	GTCATGACACTGACACCA	184	184	0	0	0	0
TNFRSF11A	CATGCAACACTGCAGTTG	801	650	2	0	0	53
TNFRSF11B	ACACCATGGTCATGCATG	209	128	0	1	1	0
TNFRSF11B	CAACCATGGTCATGCACA	322	192	0	0	0	1
TNFRSF11B	TGACCATGGTCATGCAAC	22	18	0	0	0	0
TNFRSF11B	CAACCATGGTCATGACGT	77	31	0	0	0	0
TNFRSF11B	CATGCAGTCATGTGTGCA	869	371	1	1	0	0
TNFRSF13B	CAACTGCAGTGTGTGTTG	688	886	1	0	1	0
TNFRSF13B	GTACTGCAGTGTGTGTCA	113	342	1	0	0	0
TNFRSF13B	ACACTGCAGTGTGTGTAC	10	20	0	0	0	0
TNFRSF13B	GTACTGCAGTGTGTCAGT	108	145	0	0	0	1
TNFRSF13B	CAACTGCAGTGTGTCACA	222	226	0	0	0	61
TNFRSF13B	GTTGCAACTGGTGTTGGT	822	951	1	0	0	0
TNFRSF13C	TGACTGGTTGTGGTTGTG	957	473	0	2	0	0
TNFRSF13C	CAACTGGTTGTGCAGTGT	1044	669	1	0	0	0
TNFRSF13C	ACACTGGTTGTGCAGTCA	180	261	0	0	0	0
TNFRSF13C	GTACTGGTTGTGCAGTAC	38	22	0	0	0	0
TNFRSF13C	TGACTGGTTGTGCAGTTG	185	811	1	2	0	11
TNFRSF13C	ACTGCAACGTTGCATGAC	37	37	0	0	0	0
TNFRSF17	TGACTGGTACTGTGTGGT	730	1771	2	3	0	0
TNFRSF17	GTACTGGTACTGTGTGCA	844	303	1	0	0	2
TNFRSF17	ACACTGGTACTGTGTGAC	12	3	0	0	0	0
TNFRSF17	CAACTGGTACTGTGTGTG	590	227	0	0	0	0

TNFRSF17	GTACTGGTTGGTGTGTGT	1284	1473	4	0	0	67
TNFRSF17	TGTGCAACTGGTGTACGT	222	219	0	0	0	0
TNFRSF18	ACACTGGTACCACATGGT	205	222	0	0	1	1
TNFRSF18	TGACTGGTACCACAACTG	147	344	0	1	0	0
TNFRSF18	TGACTGGTACCACATGCA	270	179	2	0	0	0
TNFRSF18	CAACTGGTACCACATGAC	8	37	0	0	0	0
TNFRSF18	ACTGCAACTGGTGTACAC	17	13	0	0	0	0
TNFRSF18	CATGCAACTGGTGTACTG	373	392	0	0	0	0
TNFRSF19	CAACTGGTTGTGGTTGGT	255	324	0	0	1	0
TNFRSF19	TGACTGGTTGTGGTACAC	9	12	0	0	0	0
TNFRSF19	ACACTGGTTGTGGTACTG	382	750	0	0	0	1
TNFRSF19	ACACTGGTTGTGGTTGCA	320	186	0	0	0	5
TNFRSF19	GTACTGGTTGTGGTTGAC	14	147	0	0	0	0
TNFRSF19	GTTGCATGCAGTACTGTG	150	108	0	0	0	0
TNFRSF1A	CAACTGGTTGCAGTGTTG	748	1163	0	0	1	1
TNFRSF1A	GTACTGGTTGCAGTCAGT	134	147	3	0	0	1
TNFRSF1A	CAACTGGTTGCAGTCACA	121	63	0	0	0	0
TNFRSF1A	TGACTGGTTGCAGTCAAC	15	21	0	0	0	0
TNFRSF1A	ACACTGGTTGCAGTCATG	98	77	0	0	0	1
TNFRSF1A	GTTGCAACTGGTCATGCA	76	151	1	0	0	0
TNFRSF1B	TGCATGACTGTGTGTGCA	98	110	0	0	0	0
TNFRSF1B	CACATGACTGTGTGTGAC	24	53	0	0	0	0
TNFRSF1B	GTCATGACTGTGTGTGTG	245	309	0	0	0	0
TNFRSF1B	TGCATGTGGTGTGTGTGT	741	1303	3	1	0	0
TNFRSF1B	GTTGCAGTGTACACTGCA	62	60	0	0	0	0
TNFRSF1B	GTTGCAACTGGTCAACTG	174	257	0	2	0	0
TNFRSF4	TGTGCAACTGGTCATGGT	464	580	0	0	1	0
TNFRSF4	GTACTGGTTGTGCACATG	277	260	0	0	0	0
TNFRSF4	TGACTGGTTGTGCAACGT	664	401	0	1	0	0
TNFRSF4	GTACTGGTTGTGCAACCA	199	181	0	0	0	4
TNFRSF4	ACACTGGTTGTGCAACAC	283	203	0	0	0	0
TNFRSF4	GTTGCAGTTGACTGGTAC	166	74	0	0	0	0
TNFRSF8	GTACTGGTTGACACACAC	175	99	0	0	0	6
TNFRSF8	TGACTGGTTGACACACTG	1690	1232	2	1	0	9
TNFRSF8	ACACTGGTTGACACTGGT	236	344	0	1	0	0
TNFRSF8	TGACTGGTTGACACTGCA	91	72	3	0	0	1
TNFRSF8	TGTGACCACAGTCAACGT	175	419	0	1	0	0
TNFRSF9	GTACTGGTTGGTACCACA	30	56	0	0	0	0
TNFRSF9	ACACTGGTTGGTACCAAC	11	19	0	0	0	0
TNFRSF9	CAACTGGTTGGTACCATG	476	212	0	0	0	0
TNFRSF9	GTACTGGTTGGTACACGT	32	25	0	0	0	0
TNFRSF9	CATGCAGTTGACGTCAGT	429	250	0	1	0	0
TNFSF10	ACCATGTGTGCAGTTGAC	39	75	0	0	0	0
TNFSF10	CACATGTGTGCAGTTGTG	162	63	0	0	0	0
TNFSF10	TGCATGTGTGCACAGTGT	224	318	0	0	0	2
TNFSF10	GTCATGTGTGCACAGTCA	414	379	0	0	0	1
TNFSF10	ACCATGTGTGCACAGTAC	116	113	0	1	0	0
TNFSF10	GTTGCAACTGGTACTGTG	300	290	0	0	0	0
TNFSF11	ACCATGACACTGTGCACA	383	488	4	5	4	672
TNFSF11	ACCATGACACTGTGGTTG	566	567	0	0	0	0
TNFSF11	CACATGACACTGTGCAGT	381	775	1	1	0	4
TNFSF11	GTCATGACACTGTGCAAC	24	45	0	0	0	0
TNFSF11	TGCATGACACTGTGCATG	102	144	0	0	0	0
TNFSF11	CATGCAACACCACACAGT	379	316	0	0	0	0
TNFSF13	GTACTGCAGTGTTGGTGT	891	845	1	0	1	0
TNFSF13	CAACTGCAGTGTACTGCA	20	46	0	0	0	0
TNFSF13	TGACTGCAGTGTACTGAC	125	120	0	0	0	0
TNFSF13	ACACTGCAGTGTACTGTG	153	190	1	0	0	0
TNFSF13	GTTGGTTGACCAGTGTCA	437	434	0	0	0	0
TNFSF13	CATGCAACTGTGGTACGT	117	204	0	0	0	0
TNFSF13B	ACACTGGTACTGCAACTG	617	369	1	0	1	1
TNFSF13B	ACTGCAACTGGTTGGTGT	439	547	0	0	1	0
TNFSF13B	CAACTGGTACTGCATGGT	569	438	0	0	0	0
TNFSF13B	ACACTGGTACTGCATGCA	273	304	0	0	0	0
TNFSF13B	GTACTGGTACTGCATGAC	28	21	0	0	0	0
TNFSF13B	TGACTGGTACTGCATGTG	395	442	0	0	0	0
TNFSF14	CAACTGGTTGCACATGGT	96	72	0	0	0	0
TNFSF14	ACACTGGTTGCACATGCA	189	256	0	0	0	0
TNFSF14	GTACTGGTTGCACATGAC	23	32	0	0	0	0
TNFSF14	TGACTGGTTGCACATGTG	1176	909	1	1	0	1
TNFSF14	CAACTGGTTGCAACGTGT	594	987	1	0	0	2
TNFSF18	GTACTGCAGTGTCATGAC	28	46	0	0	0	0
TNFSF18	TGACTGCAGTGTCATGTG	630	455	2	1	0	5
TNFSF18	CAACTGCAGTGTACGTGT	484	557	0	0	0	6
TNFSF18	ACACTGCAGTGTACGTCA	265	137	0	0	0	0

TNFSF18	GTACTGCAGTGTACGTAC	60	93	0	1	0	0
TNFSF4	TGACTGGTTGGTTGACGT	119	169	1	0	0	32
TNFSF4	GTACTGGTTGGTTGACCA	90	98	0	0	0	0
TNFSF4	ACACTGGTTGGTTGACAC	65	90	0	0	0	0
TNFSF4	CAACTGGTTGGTTGACTG	99	283	0	0	0	0
TNFSF4	TGTGCAACTGGTTGGTCA	72	199	0	0	0	0
TNFSF4	CATGCAACTGGTTGGTAC	61	46	0	0	0	0
TNFSF8	GTACTGGTCATGGTACCA	436	253	2	0	1	0
TNFSF8	GTACTGGTCATGGTCATG	125	148	0	1	0	0
TNFSF8	TGACTGGTCATGGTACGT	129	82	0	0	0	0
TNFSF8	ACACTGGTCATGGTACAC	46	68	0	0	0	1
TNFSF8	CAACTGGTCATGGTACTG	991	1055	1	1	0	1
TNFSF8	CATGCAACTGGTTGTGGT	734	272	0	1	0	0
TNFSF9	CAACTGGTTGTGCAACTG	418	250	0	1	0	1
TNFSF9	GTACTGGTTGTGCATGGT	571	179	1	0	0	0
TNFSF9	CAACTGGTTGTGCATGCA	196	397	1	1	0	0
TNFSF9	ACTGCAACTGGTTGTGCA	203	322	1	0	0	0
TNFSF9	GTTGCAACTGGTTGTGAC	17	9	0	0	0	0
TNFSF9	TGTGCAACTGGTTGTGTG	465	714	1	0	0	1
TNIP1	TGACGTCAGTTGACACAC	19	41	0	0	1	0
TNIP1	ACACGTCAGTTGACACTG	60	47	0	0	0	0
TNIP1	CAACGTCAGTTGACTGGT	46	73	0	0	0	0
TNIP1	ACACGTCAGTTGACTGCA	138	455	0	1	0	1
TNIP1	GTACGTCAGTTGACTGAC	30	28	0	0	0	0
TNIP2	ACTGGTCATGCAGTGTTG	718	847	0	1	1	0
TNIP2	CATGGTCATGCAGTCAGT	655	401	0	0	0	0
TNIP2	ACTGGTCATGCAGTCACA	131	97	0	0	0	0
TNIP2	GTTGGTCATGCAGTCAAC	47	49	0	0	0	0
TNIP2	ACTGGTTGACCAGTGTAC	78	68	0	0	0	0
TNIP3	TGTGGTCAACACACGTTG	1079	904	621931	682	40	49
TNIP3	ACTGGTCAACACACGTCA	182	331	1	0	2	0
TNIP3	GTTGGTCAACACACGTAC	168	27	0	1	0	0
TNIP3	ACTGGTCAACACACCAGT	119	127	0	0	0	0
TNIP3	TGTGGTCAACACACCACA	55	52	5	0	0	0
TNK2	ACCATGACGTTGACCAAC	66	43	0	0	0	0
TNK2	CACATGACGTTGACCATG	141	146	0	0	0	0
TNK2	GTCATGACGTTGACACGT	79	45	0	0	0	0
TNK2	CACATGACGTTGACACCA	252	349	0	0	0	0
TNK2	TGCATGACGTTGACACAC	20	15	0	0	0	0
TNKS	ACACCATGACGTACACGT	52	89	0	0	2	0
TNKS	TGACCATGACGTACCATG	212	241	0	0	1	0
TNKS	ACACCATGACGTACCACA	22	39	0	1	0	0
TNKS	GTACCATGACGTACCAAC	2	7	0	0	0	0
TNKS	TGACCATGACGTACACCA	404	169	1	0	0	0
TNKS1BP1	ACTGGTCAACTGGTACGT	176	104	0	3	102	1
TNKS1BP1	TGTGGTCAACTGGTACCA	439	189	0	0	0	0
TNKS1BP1	CATGGTCAACTGGTACAC	31	52	0	0	0	0
TNKS1BP1	GTTGGTCAACTGGTACTG	328	287	0	0	0	17
TNKS1BP1	TGTGGTCAACTGGTTGGT	133	43	0	1	0	0
TNKS2	CAACACTGGTGTCATGCA	188	556	0	0	1	7
TNKS2	TGACACTGGTGTCATGAC	27	22	0	0	0	0
TNKS2	ACACACTGGTGTCATGTG	452	520	0	0	0	1
TNKS2	GTACACTGGTGTACGTGT	214	179	2	0	0	0
TNKS2	CAACACTGGTGTACGTCA	111	94	0	0	0	0
TNKS2	ACTGCAACACGTACACGT	73	109	0	0	0	0
TNNT1	GTTGGTCAGTACTGGTAC	107	83	0	0	0	1
TNNT1	TGTGGTCAGTACTGGTTG	251	277	2	0	0	0
TNNT1	ACTGGTCAGTACTGCAGT	301	346	1	0	0	0
TNNT1	TGTGGTCAGTACTGCACA	22	36	0	1	0	0
TNNT1	CATGGTCAGTACTGCAAC	9	11	0	0	0	0
TNNT2	TGTGGTGTGTCAACGT	231	564	4	0	1	3
TNNT2	ACTGACGTCACATGGTCA	1114	1395	2	0	1	1
TNNT2	CATGGTGTGTGTCACAAC	21	23	0	0	0	1
TNNT2	GTTGGTGTGTGTCACATG	277	312	1	0	0	0
TNNT2	ACTGCAGTTGTGTGTGCA	212	225	0	0	0	0
TOB1	ACACGTGTACCAACCAGT	169	142	2	0	0	0
TOB1	TGACGTGTACCAACCACA	15	19	0	0	0	0
TOB1	CAACGTGTACCAACCAAC	40	46	0	0	0	0
TOB1	GTACGTGTACCAACCATG	178	235	0	0	0	0
TOB1	TGACGTGTACCAACACGT	9	18	0	0	0	0
TOB1	ACTGCAACACACCATGCA	323	206	1	0	0	0
TOB2	CACATGTGGTTGTGACCA	42	44	0	0	0	0
TOB2	TGCATGTGGTTGTGACAC	38	22	0	0	0	0
TOB2	ACCATGTGGTTGTGACTG	143	553	1	1	0	0
TOB2	CACATGTGGTTGTGTGGT	347	384	1	1	0	0

TOB2	GTTGCAGTGTACGTGTTG	639	770	0	1	0	0
TOLLIP	GTACTGACCAGTTGCACA	19	35	0	0	0	0
TOLLIP	ACACTGACCAGTTGCAAC	36	62	0	0	0	0
TOLLIP	CAACTGACCAGTTGCATG	79	118	2	0	0	0
TOLLIP	GTACTGACCAGTTGACGT	671	1143	2	0	0	1
TOLLIP	CAACTGACCAGTTGACCA	135	142	0	0	0	0
TOLLIP	CATGCAACACGTCATGCA	236	70	0	1	0	0
TOP1	ACCATGTGGTACACTGCA	49	80	0	0	0	0
TOP1	GTCATGTGGTACACTGAC	16	35	0	0	0	0
TOP1	TGCATGTGGTACACTGTG	240	235	2	0	0	0
TOP1	CACATGTGGTACTGGTGT	197	172	0	0	0	0
TOP1	ACCATGTGGTACTGGTCA	11	1	0	0	0	0
TOP1	GTTGCAACCACAGTCAGT	186	271	0	1	0	0
TOP2A	CAACACCAACTGGTGTGT	1052	792	2	0	1	1
TOP2A	GTACACCAACACTGTGGT	358	383	0	0	0	0
TOP2A	CAACACCAACACTGTGCA	44	68	1	0	0	0
TOP2A	TGACACCAACACTGTGAC	45	78	0	1	0	0
TOP2A	ACACACCAACACTGTGTG	167	451	0	0	0	0
TOP2A	CATGCAACCACAGTCACA	29	43	0	0	0	0
TOP2B	ACACACCAACTGGTGTCA	348	358	1	0	0	3
TOP2B	GTACACCAACTGGTGTAC	25	21	0	0	0	0
TOP2B	TGACACCAACTGGTGTTG	246	277	0	1	0	0
TOP2B	TGTGGTTGTGGTACGTTG	500	377	2	0	0	29
TOP2B	ACTGGTTGTGGTACCAGT	14	18	0	0	0	0
TOP2B	TGTGCAACCACAGTCAAC	46	47	0	0	0	0
TOP3A	ACACACCAACTGGTCAGT	132	100	1	0	0	0
TOP3A	TGACACCAACTGGTCACA	178	274	0	0	0	0
TOP3A	CAACACCAACTGGTCAAC	2	12	0	0	0	0
TOP3A	CATGCAGTTGGTACACTG	529	325	0	0	0	0
TOP3A	GTTGCAGTTGGTACTGGT	127	148	1	0	0	0
TOP3B	GTACACCAACTGGTCATG	171	218	0	0	0	0
TOP3B	TGACACCAACTGGTACGT	166	190	0	2	0	0
TOP3B	GTACACCAACTGGTACCA	133	140	0	0	0	0
TOP3B	ACACACCAACTGGTACAC	74	18	0	0	0	0
TOP3B	CAACACCAACTGGTACTG	939	1015	3	0	0	4
TOR1A	GTACTGTGACTGACACTG	421	571	0	0	0	0
TOR1A	TGACTGTGACTGACTGGT	494	330	2	1	0	32
TOR1A	GTACTGTGACTGACTGCA	273	255	1	1	0	0
TOR1A	ACACTGTGACTGACTGAC	28	42	0	0	0	0
TOR1A	CAACTGTGACTGACTGTG	784	564	0	1	0	2
TP53	TGCATGACTGTGACTGGT	644	551	1	0	1	86
TP53	GTCATGACTGTGACACTG	234	286	0	0	0	0
TP53	GTCATGACTGTGACTGCA	64	88	0	0	0	0
TP53	ACCATGACTGTGACTGAC	48	83	0	0	0	1
TP53	GTTGCAGTACGTTGCATG	113	121	0	0	0	0
TP53BP1	CAACGTACACTGTGGTGT	578	661	9	22	3	19
TP53BP1	ACACGTACACTGTGGTCA	482	523	0	0	1	0
TP53BP1	GTACGTACACTGTGGTAC	37	80	0	0	0	0
TP53BP1	TGACGTACACTGTGGTTG	802	721	730	1	0	20
TP53BP1	ACACGTACACTGTGCAGT	418	352	0	0	0	0
TP53BP1	TGTGCACAACGTCAACCA	296	178	0	0	0	6
TP53BP2	CAACTGCATGCACAGTTG	234	203	0	0	1	0
TP53BP2	GTACTGCATGCACAGTCA	237	354	1	0	0	0
TP53BP2	ACACTGCATGCACAGTAC	128	135	0	0	0	1
TP53BP2	GTACTGCATGCACACAGT	275	113	0	2	0	1
TP53BP2	CATGCAGTCATGACACCA	175	274	0	0	0	0
TP53INP1	GTTGGTCAACACCAGTTG	1734	1616	2	0	1	0
TP53INP1	CATGGTCAACACCAGTAC	309	134	0	0	0	0
TP53INP1	TGTGGTCAACACCACAGT	128	93	0	0	0	0
TP53INP1	GTTGGTCAACACCACACA	206	166	1	1	0	0
TP53INP1	ACTGGTCAACACCACAAC	24	31	0	0	0	0
TP53INP1	CATGCAACCAACCACAGT	171	187	0	0	0	0
TP73	TGACGTGTGTACACTGGT	203	638	0	0	1	0
TP73	CAACGTGTGTACACACAC	25	12	0	0	0	0
TP73	GTACGTGTGTACACACTG	486	986	2	0	0	0
TP73	GTACGTGTGTACACTGCA	111	73	0	0	0	0
TP73	ACACGTGTGTACACTGAC	55	52	0	0	0	0
TP73	TGTGCACACATGTGTGTG	433	384	0	2	0	0
TPH1	CAACACTGCATGACACGT	79	82	0	0	0	0
TPH1	ACACACTGCATGACACCA	310	300	0	0	0	0
TPH1	GTACACTGCATGACACAC	40	49	0	0	0	0
TPH1	TGACACTGCATGACACTG	168	55	0	0	0	0
TPH1	TGTGGTCACAGTGTGTAC	118	117	0	0	0	0
TPH1	CATGCACACATGCATGTG	159	218	0	0	0	0
TPH2	TGACACTGCATGTGACAC	19	14	0	0	0	0

TPH2	ACACACTGCATGTGACTG	170	186	0	0	0	0
TPH2	CAACACTGCATGTGTGGT	635	489	0	34	0	0
TPH2	ACACACTGCATGTGTGCA	52	66	1	0	0	0
TPH2	GTACACTGCATGTGTGAC	75	80	0	0	0	0
TPH2	GTTGCATGCAGTTGTGAC	36	33	0	0	0	0
TPI1	TGACACCAACACACGTCA	626	387	1	1	1	0
TPI1	CAACACCAACACACGTAC	69	52	0	0	0	0
TPI1	GTACACCAACACACGTTG	902	376	1	3	0	0
TPI1	TGACACCAACACACCAGT	368	340	0	0	0	1
TPI1	GTACACCAACACACCACA	203	45	0	0	0	0
TPK1	CAACCAACTGACGTCAAC	19	86	0	0	0	0
TPK1	GTACCAACTGACGTCATG	252	192	0	1	0	0
TPK1	TGACCAACTGACGTACGT	22	34	0	0	0	0
TPK1	GTACCAACTGACGTACCA	307	403	0	0	0	1
TPK1	ACACCAACTGACGTACAC	86	21	0	0	0	0
TPMT	TGACCAACCAACACTGTG	787	945	11	0	4	0
TPMT	CAACCAACCAACACTGGT	564	489	1	0	1	0
TPMT	ACACCAACCAACACTGCA	670	796	0	0	0	0
TPMT	GTACCAACCAACACTGAC	83	83	0	0	0	0
TPMT	TGTGCAGTACTGACGTAC	12	44	0	0	0	0
TPO	GTACACGTACTGGTACTG	349	402	7	14	5	27
TPO	TGACACGTACTGGTCATG	84	92	0	0	0	0
TPO	ACACACGTACTGGTACGT	208	255	0	0	0	0
TPO	TGACACGTACTGGTACCA	262	396	0	0	0	1
TPO	CAACACGTACTGGTACAC	88	66	0	0	0	1
TPO	CATGCATGGTCACAGTAC	101	288	0	0	0	8
TPP1	TGCATGACTGCATGTGGT	142	127	0	1	1	1
TPP1	GTCATGACTGCATGTGCA	89	97	1	0	0	0
TPP1	ACCATGACTGCATGTGAC	20	23	0	0	0	0
TPP1	ACTGCAGTACGTTGCAGT	267	314	0	0	0	0
TPP1	TGTGCAGTACGTTGCACA	223	108	0	0	0	0
TPP2	CAACCAGTTGTGACCATG	86	97	1	0	0	0
TPP2	TGACACACCAACTGCATG	89	80	2	0	0	0
TPP2	ACACACACCAACTGACGT	863	303	0	0	0	0
TPP2	TGACACACCAACTGACCA	117	144	0	0	0	0
TPP2	CAACACACCAACTGACAC	29	35	0	0	0	0
TPP2	TGTGCACACACAGTACAC	22	20	0	0	0	0
TRADD	ACACGTGTTGGTACGTCA	128	108	8	1	10	49
TRADD	TGACGTGTTGGTCATGTG	256	393	1	0	0	0
TRADD	CAACGTGTTGGTACGTGT	521	777	3	2	0	0
TRADD	GTACGTGTTGGTACGTAC	2	16	0	0	0	0
TRADD	CATGCAGTACCATGCAGT	146	135	1	0	0	35
TRAF1	CAACACTGACCAACACCA	355	291	30	1	2	7
TRAF1	GTACACTGACCAACCACA	20	25	0	0	0	0
TRAF1	ACACACTGACCAACCAAC	7	10	0	0	0	0
TRAF1	CAACACTGACCAACCATG	218	171	0	0	0	1
TRAF1	GTACACTGACCAACACGT	311	206	2	0	0	0
TRAF2	ACCATGTGCACACAGTGT	668	542	3	0	1	0
TRAF2	CACATGTGCACAGTTGAC	34	43	0	0	0	0
TRAF2	GTCATGTGCACAGTTGTG	65	188	2	0	0	0
TRAF2	TGCATGTGCACACAGTCA	522	574	0	2	0	0
TRAF2	TGTGCAGTACCAGTACTG	231	181	0	0	0	0
TRAF3	CAACCACACACAGTGTGT	742	883	2	1	2	116
TRAF3	TGACCACACACAGTGTTG	720	246	0	0	1	0
TRAF3	GTACCACACAGTTGTGTG	970	587	353	0	0	0
TRAF3	ACACCACACACAGTGTCA	762	400	0	1	0	0
TRAF3	GTACCACACACAGTGTAC	143	88	0	1	0	0
TRAF3	CATGCAACACACGTACGT	911	503	0	0	0	1
TRAF3IP1	TGACTGACCAGTCAACCA	275	139	0	0	0	0
TRAF3IP1	CAACTGACCAGTCAACAC	53	52	0	1	0	0
TRAF3IP1	GTACTGACCAGTCAACTG	325	716	0	0	0	1
TRAF3IP1	TGACTGACCAGTCATGGT	257	192	0	0	0	0
TRAF3IP1	GTACTGACCAGTCATGCA	360	222	0	0	0	1
TRAF3IP1	CATGCAACCAACGTCAAC	75	81	0	0	0	1
TRAF3IP2	CATGGTACTGCACATGTG	860	287	0	1	0	0
TRAF3IP2	TGTGGTACTGCAACGTGT	197	119	3	0	0	0
TRAF3IP2	GTTGGTACTGCAACGTCA	172	419	0	1	0	1
TRAF3IP2	ACTGGTACTGCAACGTAC	75	36	0	0	0	2
TRAF3IP2	CATGGTACTGCAACGTTG	36	26	2	0	0	0
TRAF3IP2	CATGCAACACTGGTGTCA	256	111	0	0	0	0
TRAF4	GTACCACACACAGTTGGT	822	492	2	0	0	0
TRAF4	CAACCACACACAGTTGCA	366	364	0	0	0	0
TRAF4	TGACCACACACAGTTGAC	102	43	0	0	0	0
TRAF4	ACACCACACACAGTTGTG	146	72	1	0	0	0
TRAF4	GTACCACACACACAGTGT	904	1788	3	2	0	0

TRAF5	CAACGTGTACGTCAACGT	278	212	0	0	0	0
TRAF5	ACACGTGTACGTCAACCA	181	318	0	0	0	0
TRAF5	GTACGTGTACGTCAACAC	13	19	0	0	0	0
TRAF5	TGACGTGTACGTCAACTG	41	64	0	0	0	0
TRAF5	ACACGTGTACGTCATGGT	288	661	0	1	0	1
TRAF6	ACTGCAACACACGTACCA	335	376	0	0	1	0
TRAF6	TGACGTGTACGTCATGCA	255	287	0	0	0	0
TRAF6	CAACGTGTACGTCATGAC	51	42	0	0	0	0
TRAF6	GTACGTGTACGTCATGTG	430	397	0	0	0	0
TRAF6	ACACGTGTACGTACGTGT	24	228	0	0	0	0
TRAF6	TGACGTGTACGTACGTCA	68	289	0	0	0	0
TRAFD1	TGCATGTGCATGTGGTGT	953	514	205	5	9	11057
TRAFD1	ACCATGTGCATGACTGAC	24	31	0	0	0	0
TRAFD1	CACATGTGCATGACTGTG	141	53	0	0	0	0
TRAFD1	GTCATGTGCATGTGGTCA	329	324	0	0	0	0
TRAFD1	ACCATGTGCATGTGGTAC	24	23	0	0	0	0
TRAFD1	TGTGCACAACCACAGTGT	41	66	0	0	0	0
TREH	ACTGGTCATGTGGTACCA	336	314	0	0	2	0
TREH	GTTGGTCATGTGGTACAC	62	89	0	0	0	0
TREH	TGTGGTCATGTGGTACTG	224	224	0	1	0	0
TREH	ACTGGTCATGTGGTTGGT	714	549	2	1	0	3
TREH	GTTGACACCACAGTGTTG	228	215	0	0	0	0
TREM1	TGACACTGACGTACACTG	209	55	0	0	0	0
TREM1	ACACACTGACGTACTGGT	133	144	0	0	0	0
TREM1	TGACACTGACGTACTGCA	199	240	1	0	0	0
TREM1	CAACACTGACGTACTGAC	71	30	0	0	0	0
TREM1	GTACACTGACGTACTGTG	1006	488	0	0	0	12
TREM1	GTTGCATGCAGTACGTCA	103	109	0	0	0	0
TREM2	ACACACTGACGTCACACA	270	241	0	0	0	0
TREM2	GTACACTGACGTCACAAC	11	16	0	0	0	0
TREM2	TGACACTGACGTCACATG	79	55	0	0	0	0
TREM2	ACACACTGACGTCAACGT	420	182	1	0	0	0
TREM2	ACTGCAGTTGACACGTGT	138	176	0	1	0	0
TREM2	GTTGCACATGGTTGGTAC	54	80	0	0	0	0
TREX1	GTACGTGTACTGCAGTAC	144	72	0	0	1	0
TREX1	TGACGTGTACTGCAGTTG	978	646	0	1	0	0
TREX1	CATGCAGTACCACATGGT	793	385	1	0	0	1
TREX1	ACTGCAGTACCACATGCA	178	194	0	0	0	4
TREX1	GTTGCAGTACCACATGAC	36	59	0	0	0	0
TREX2	ACACGTGTACTGCACAGT	274	386	0	0	0	0
TREX2	TGACGTGTACTGCACACA	109	87	0	0	0	0
TREX2	TGTGCAGTACCACATGTG	384	428	1	0	0	0
TREX2	TGTGACCAGTCATGTGAC	45	64	0	0	0	0
TREX2	ACTGACCAGTCATGTGTG	536	381	1	1	0	0
TRH	CAACTGTGTGTGTGCATG	98	219	1	0	0	0
TRH	GTACTGTGTGTGTGACGT	136	75	0	0	0	2
TRH	CAACTGTGTGTGTGACCA	195	124	0	1	0	0
TRH	TGACTGTGTGTGTGACAC	48	29	0	0	0	0
TRH	CATGCACAGTGTGTCATG	61	188	0	0	0	0
TRH	CATGCACAACCAACACCA	576	531	1	2	0	0
TRIB3	ACACCAACACACACCACA	208	384	0	0	0	0
TRIB3	GTACCAACACACACCAAC	108	93	0	0	0	0
TRIB3	TGACCAACACACACCATG	407	582	0	0	0	0
TRIB3	ACACCAACACACACACGT	81	40	0	0	0	0
TRIB3	TGACCAACACACACACCA	246	503	0	1	0	1
TRIM14	GTACTGCATGCACATGTG	39	58	0	0	0	0
TRIM14	ACACTGCATGCAACGTGT	533	699	1	1	0	1
TRIM14	ACTGCAGTTGTGGTACAC	40	80	0	0	0	0
TRIM14	CATGCAACGTCAACCAGT	163	117	0	1	0	0
TRIM14	ACTGCAACGTCAACCACA	98	77	0	0	0	0
TRIM14	GTTGCAACGTCAACCAAC	65	118	0	0	0	0
TRIM21	CACATGTGGTACACACCA	133	125	0	0	0	4
TRIM21	TGCATGTGGTACACACAC	91	63	0	0	0	0
TRIM21	ACCATGTGGTACACACTG	192	192	0	0	0	47
TRIM21	CACATGTGGTACACTGGT	93	51	0	0	0	0
TRIM21	ACTGGTTGTGCACAGTCA	196	128	1	0	0	0
TRIM25	ACCATGACTGGTCAACCA	610	744	0	10	12	0
TRIM25	TGCATGACTGGTCAACTG	1055	1431	0	0	2	0
TRIM25	CACATGACTGGTCAACGT	398	414	0	1	0	0
TRIM25	GTCATGACTGGTCAACAC	180	175	0	0	0	0
TRIM25	ACCATGACTGGTCATGGT	127	358	0	0	0	0
TRIM28	CAACGTACACACTGACAC	22	12	0	0	0	0
TRIM28	GTACGTACACACTGACTG	510	646	2	0	0	11
TRIM28	TGACGTACACACTGTGGT	602	752	0	0	0	0
TRIM28	GTACGTACACACTGTGCA	150	166	0	0	0	1

TRIM28	ACACGTACACACTGTGAC	18	13	0	0	0	0
TRIM28	TGTGCAACGTCACAGTTG	402	461	0	0	0	0
TRIO	GTCATGCATGGTACTGGT	313	307	36	27	21	101708
TRIO	TGTGGTACTGACACGTTG	275	452	1	0	1	0
TRIO	ACCATGCATGGTACACAC	75	53	0	0	0	0
TRIO	CACATGCATGGTACACTG	129	114	0	0	0	5
TRIO	CACATGCATGGTACTGCA	59	74	0	0	0	0
TRIP10	GTACTGACGTACTGGTTG	581	590	1	0	1	1
TRIP10	CAACTGACGTACTGGTAC	84	64	0	0	0	0
TRIP10	TGACTGACGTACTGCAGT	86	243	1	1	0	0
TRIP10	GTACTGACGTACTGCACA	43	78	0	0	0	0
TRIP10	ACACTGACGTACTGCAAC	45	48	0	0	0	0
TRIP12	ACACGTTGTGCATGTGCA	390	207	2	0	1	2
TRIP12	TGACGTTGTGCATGTGTG	1222	238	0	1	1	5
TRIP12	GTTGCACATGCAACACCA	127	91	0	0	1	0
TRIP12	ACACGTTGTGCATGACTG	361	605	1	0	0	0
TRIP12	CAACGTTGTGCATGTGGT	302	317	0	0	0	1
TRIP12	GTACGTTGTGCATGTGAC	29	24	0	0	0	0
TRO	CATGGTGTACCAACTGGT	539	238	0	0	0	0
TRO	ACTGGTGTACCAACTGCA	159	203	0	0	0	0
TRO	GTTGGTGTACCAACTGAC	8	8	0	0	0	0
TRO	TGTGGTGTACCAACTGTG	336	907	1	2	0	1
TRO	CATGGTGTACCATGGTGT	54	127	0	0	0	0
TRPC2	CATGACCAACCATGACAC	40	52	0	0	0	0
TRPC2	GTTGACCAACCATGACTG	313	304	0	0	0	0
TRPC2	TGTGACCAACCATGTGGT	404	452	0	0	0	4
TRPC2	GTTGACCAACCATGTGCA	275	286	1	0	0	13
TRPC2	ACTGACCAACCATGTGAC	58	42	0	0	0	0
TRPC7	ACACACGTGTACTGGTCA	161	170	119	1	0	0
TRPC7	GTACACGTGTACTGGTAC	15	44	0	0	0	0
TRPC7	TGACACGTGTACTGGTTG	1140	980	0	1	0	0
TRPC7	ACACACGTGTACTGCAGT	227	124	2	6	0	5
TRPC7	TGACACGTGTACTGCACA	79	81	0	0	0	0
TRPC7	TGTGCACATGCAGTTGCA	633	215	0	1	0	0
TRPM2	ACACCATGTGACTGTGGT	387	433	0	0	0	0
TRPM2	TGACCATGTGACTGTGCA	135	141	0	0	0	0
TRPM2	CAACCATGTGACTGTGAC	88	91	0	0	0	0
TRPM2	GTACCATGTGACTGTGTG	215	771	0	0	0	0
TRPM2	TGACCATGTGTGGTGTGT	301	427	1	1	0	0
TRPM4	CAACACGTGTACACGTAC	154	76	0	1	0	0
TRPM4	GTACACGTGTACACGTTG	167	142	1	0	0	0
TRPM4	TGACACGTGTACACCAGT	231	240	0	0	0	0
TRPM4	GTACACGTGTACACCACA	79	154	1	0	0	0
TRPM4	ACACACGTGTACACCAAC	13	12	0	0	0	0
TRPM4	TGTGCAACCATGTGCAAC	12	19	0	1	0	0
TRPM7	GTACGTTGACCAACGTGT	577	604	2	0	0	0
TRPM7	CAACGTTGACCAACGTCA	244	269	45	0	0	0
TRPM7	TGACGTTGACCAACGTAC	90	194	0	0	0	0
TRPM7	ACACGTTGACCAACGTTG	151	148	0	0	0	0
TRPM7	TGTGCAGTACGTGTACCA	141	170	0	0	0	0
TRPM7	ACTGCATGGTACACCATG	199	263	0	0	0	3
TRPV1	CATGCAGTTGGTCAGTAC	33	48	0	0	1	0
TRPV1	ACACCATGTGTGGTACCA	216	195	3	0	0	0
TRPV1	GTACCATGTGTGGTACAC	103	23	0	0	0	0
TRPV1	TGACCATGTGTGGTACTG	161	110	0	0	0	0
TRPV1	GTTGCAGTTGGTCAGTTG	2048	2149	4	1	0	14
TRPV6	GTCATGACGTACTGTGCA	195	81	0	0	0	0
TRPV6	ACCATGACGTACTGTGAC	21	34	0	1	0	0
TRPV6	CACATGACGTACTGTGTG	460	213	2	1	0	0
TRPV6	GTTGCACAGTGTTGACAC	37	25	0	0	0	0
TRPV6	TGTGCACAGTGTTGACTG	241	313	0	0	0	0
TRPV6	ACTGCAACCATGTGCATG	216	226	0	0	0	2
TRRAP	TGCATGTGACCAACTGCA	21	61	0	0	0	0
TRRAP	CACATGTGACCAACTGAC	62	43	0	0	0	0
TRRAP	GTCATGTGACCAACTGTG	410	142	0	1	0	0
TRRAP	ACCATGTGACCATGGTGT	104	105	0	0	0	0
TRRAP	TGCATGTGACCATGGTCA	543	212	0	0	0	0
TRRAP	CATGCACACACAGTTGGT	402	380	1	0	0	0
TSC1	GTACCATGCAGTCAACAC	173	221	1	0	0	0
TSC1	TGACCATGCAGTCAACTG	1270	538	1	1	0	0
TSC1	ACACCATGCAGTCATGGT	1303	367	1	0	0	3
TSC1	TGACCATGCAGTCATGCA	260	398	0	0	0	0
TSC1	CAACCATGCAGTCATGAC	231	129	0	1	0	0
TSC2	ACACCATGACGTACGTTG	290	583	1052	0	1	0
TSC2	GTTGCAGTCACAGTTGGT	444	407	1	0	1	0

TSC2	CAACCATGACGTACGTCA	175	185	0	0	0	0
TSC2	TGACCATGACGTACGTAC	25	192	0	0	0	0
TSC2	CAACCATGACGTACCAGT	182	158	1	0	0	0
TSG101	CATGCAACCAGTCAGTTG	301	359	0	0	1	0
TSG101	GTACGTGTACACGTGTAC	91	47	0	0	0	0
TSG101	TGACGTGTACACGTGTTG	608	177	1	0	0	0
TSG101	ACACGTGTACACGTCAGT	63	121	0	0	0	0
TSG101	TGACGTGTACACGTCACA	104	19	0	0	0	0
TSG101	CAACGTGTACACGTCAAC	12	5	0	0	0	0
TSHR	GTACGTCATGTGTGCAGT	76	71	0	1	0	0
TSHR	CAACGTCATGTGTGCACA	41	116	0	0	0	0
TSHR	TGTGACCAGTACGTACAC	200	113	0	0	0	0
TSHR	ACTGACCAGTACGTACTG	269	210	0	0	0	0
TSHR	CATGACCAGTACGTTGGT	314	333	0	1	0	0
TSLP	ACACCAGTTGCAACCATG	137	175	0	0	0	0
TSLP	CAACCAGTTGCAACACGT	375	141	2	1	0	0
TSLP	ACACCAGTTGCAACACCA	61	128	1	0	0	0
TSLP	GTTGACGTGTCACAACGT	88	74	0	0	0	0
TSLP	CATGACGTGTCACAACCA	221	225	0	0	0	0
TSNAX	CATGGTTGGTGTGTTGCA	205	219	0	0	0	0
TSNAX	TGTGGTTGGTGTGTTGAC	43	56	0	0	0	0
TSNAX	ACTGGTTGGTGTGTTGTG	347	426	1	0	0	0
TSNAX	GTTGGTTGGTGTCAGTGT	470	659	2	0	0	0
TSNAX	CATGGTTGGTGTCAGTCA	442	89	0	0	0	0
TSNAX	TGTGCAACCAACACCAAC	43	59	2	0	0	0
TSPAN7	GTTGGTCAGTACCAGTCA	647	433	1	0	1	0
TSPAN7	CATGGTCAGTACGTTGTG	249	447	0	0	0	0
TSPAN7	TGTGGTCAGTACCAGTGT	123	645	0	0	0	0
TSPAN7	ACTGGTCAGTACCAGTAC	27	12	0	0	0	0
TSPAN7	CATGGTCAGTACCAGTTG	476	730	0	1	0	1
TSPAN7	ACTGCAACCATGACTGAC	35	36	0	0	0	1
TSPO	CAACTGCAACGTGTACCA	424	701	0	0	0	1
TSPO	TGACTGCAACGTGTACAC	19	20	0	0	0	0
TSPO	ACACTGCAACGTGTACTG	646	505	0	2	0	0
TSPO	CAACTGCAACGTGTTGGT	295	339	0	0	0	0
TSPO	TGTGACCACATGCACAAC	16	7	0	0	0	0
TTF2	ACACGTTGTGCAGTCAGT	451	381	0	0	2	0
TTF2	GTACGTTGTGCAGTGTAC	43	30	0	0	0	0
TTF2	TGACGTTGTGCAGTGTTG	1047	705	0	1	0	1
TTF2	TGACGTTGTGCAGTCACA	145	124	0	0	0	0
TTF2	CAACGTTGTGCAGTCAAC	20	34	0	0	0	0
TTR	GTACTGCACAACACCAGT	477	385	2	1	1	0
TTR	GTACTGCACAACACGTCA	224	134	0	0	0	1
TTR	ACACTGCACAACACGTAC	198	60	1	0	0	0
TTR	CAACTGCACAACACGTTG	746	546	1	0	0	1
TTR	CAACTGCACAACACCACA	209	218	1	1	0	0
TTR	ACTGCACACACAGTTGCA	306	160	1	0	0	0
TUB	ACACCACACACACAACGT	1108	753	1	2	0	53
TUB	TGTGGTACGTGTTGCACA	58	39	0	0	0	0
TUB	CATGGTACGTGTTGCAAC	26	14	0	0	0	0
TUB	GTTGGTACGTGTTGCATG	310	400	9	0	0	0
TUB	TGTGGTACGTGTTGACGT	510	632	0	0	0	1
TUB	TGTGCATGGTACACGTGT	272	418	1	1	0	0
TUBA1A	TGACCAGTACGTTGTGGT	46	77	1	0	0	0
TUBA1A	GTACCAGTACGTTGTGCA	111	76	1	0	0	2
TUBA1A	ACACCAGTACGTTGTGAC	40	29	0	0	0	0
TUBA1A	TGTGGTGTCAGTGTCAAC	62	66	0	0	0	0
TUBA1A	ACTGGTGTACCAGTGTTG	248	207	1	0	0	1
TUBA1A	CATGCAACGTTGACCACA	55	65	1	0	0	0
TUBA1C	TGTGGTACACACGTTGGT	123	141	0	0	1	0
TUBA1C	TGTGCATGCAGTTGTGTG	59	77	0	0	0	0
TUBA1C	CAACCAGTACGTTGACAC	71	139	0	0	0	0
TUBA1C	GTTGGTCACAACGTTGGT	367	528	4	1	0	1
TUBA1C	CATGGTACACACGTACAC	24	2	0	0	0	0
TUBA1C	GTTGGTACACACGTACTG	383	232	1	0	0	0
TUBA3C	TGACTGACGTGTACACAC	37	19	0	0	0	0
TUBA3C	ACACTGACGTGTACACTG	369	655	1	0	0	0
TUBA3C	CAACTGACGTGTACTGGT	63	119	0	0	0	0
TUBA3C	ACACTGACGTGTACTGCA	193	351	0	1	0	3
TUBA3C	GTACTGACGTGTACTGAC	7	36	0	0	0	0
TUBA3C	ACTGCAACGTTGCAACGT	331	642	0	2	0	1
TUBA4	GTACCAGTACGTTGACTG	70	70	1	0	0	0
TUBA4	TGTGGTGTTGCAACCACA	111	39	0	0	0	0
TUBA4	CATGGTGTTGCAACCAAC	48	53	0	0	0	0
TUBA4	GTTGGTGTTGCAACCATG	79	70	0	0	0	0

TUBA4	TGTGGTGTTGCAACACGT	97	105	0	0	0	0
TUBA4	CATGCACATGTGACGTCA	162	156	0	0	0	0
TUBA4A	TGACTGACTGACACACGT	98	101	0	0	0	0
TUBA4A	GTACTGACTGACACACCA	315	661	1	2	0	0
TUBA4A	ACACTGACTGACACACAC	85	167	0	0	0	0
TUBA4A	CAACTGACTGACACACTG	584	859	1	2	0	0
TUBA4A	GTACTGACTGACACTGGT	132	135	0	0	0	0
TUBA4A	TGTGCAACGTTGCACATG	71	95	0	0	0	0
TUBA8	TGTGGTGTACGTCATGGT	894	792	2	2	1	0
TUBA8	GTTGGTGTACGTCAACTG	230	120	0	2	0	0
TUBA8	GTTGGTGTACGTCATGCA	93	81	1	0	0	1
TUBA8	ACTGGTGTACGTCATGAC	289	121	0	0	0	0
TUBA8	CATGGTGTACGTCATGTG	212	367	1	0	0	0
TUBA8	TGTGCACATGGTTGGTTG	464	625	0	2	0	0
TUBB2A	CATGGTGTACGTGTTGCA	198	260	0	1	0	0
TUBB2A	TGTGGTGTACGTCACATG	181	215	0	1	0	0
TUBB2A	ACTGGTGTACGTCAACGT	205	294	1	0	0	13
TUBB2A	TGTGGTGTACGTCAACCA	167	259	0	0	0	0
TUBB2A	CATGGTGTACGTCAACAC	55	94	0	0	0	0
TUBB2C	TGTGGTGTGTTGTGTGAC	339	242	1	0	0	4
TUBB2C	GTTGGTGTACCAGTTGCA	462	554	0	0	0	0
TUBB2C	ACTGGTGTACCAGTTGAC	15	5	0	0	0	0
TUBB2C	CATGGTGTACCAGTTGTG	1376	625	0	0	0	0
TUBB2C	TGTGGTGTACCACAGTGT	143	159	1	0	0	0
TUBB3	CAACGTCATGTGTGACGT	309	443	0	0	1	0
TUBB3	TGACGTCATGTGTGCAAC	19	24	0	0	0	0
TUBB3	ACACGTCATGTGTGCATG	77	97	0	0	0	0
TUBB3	ACACGTCATGTGTGACCA	161	231	1	0	0	0
TUBB3	CATGACGTCATGACACTG	483	347	2	1	0	4
TUBB4	ACACCAGTACGTTGACGT	174	128	0	1	1	0
TUBB4	ACACCAGTACGTTGCACA	189	88	0	0	0	0
TUBB4	GTACCAGTACGTTGCAAC	20	36	0	0	0	0
TUBB4	TGACCAGTACGTTGCATG	683	486	0	0	0	0
TUBB4	TGACCAGTACGTTGACCA	687	602	1	1	0	0
TUBB4	ACTGCAACACGTCACAGT	89	183	0	0	0	0
TUBD1	CATGGTGTACTGGTTGAC	18	30	0	0	1	0
TUBD1	GTTGGTGTGTTGTGTGGT	444	250	3	0	0	0
TUBD1	ACTGGTGTACTGGTTGGT	174	192	0	0	0	0
TUBD1	TGTGGTGTACTGGTTGCA	361	111	0	0	0	0
TUBD1	GTTGGTGTACTGGTTGTG	285	696	2	1	0	1
TUBE1	TGTGGTGTTGCACATGTG	522	449	2	0	1	0
TUBE1	ACTGGTGTTGCACAACTG	210	226	1	0	0	75
TUBE1	CATGGTGTTGCACATGGT	164	99	0	0	0	0
TUBE1	ACTGGTGTTGCACATGCA	291	444	0	0	0	0
TUBE1	GTTGGTGTTGCACATGAC	0	11	0	0	0	0
TUBG1	ACACTGACTGACACCAGT	77	108	0	0	0	0
TUBG1	TGACTGACTGACACCACA	146	135	0	0	0	1
TUBG1	CAACTGACTGACACCAAC	10	8	0	0	0	0
TUBG1	GTACTGACTGACACCATG	709	887	0	1	0	0
TUBG1	ACTGGTGTGTTGTGACAC	31	99	0	0	0	0
TUBG2	CATGGTGTTGCATGCAGT	475	234	1	1	0	0
TUBG2	ACTGGTGTTGCATGCACA	36	90	0	0	0	0
TUBG2	GTTGGTGTTGCATGCAAC	16	20	0	0	0	0
TUBG2	TGTGGTGTTGCATGCATG	279	240	0	0	0	0
TUBG2	ACTGGTGTTGCATGACGT	254	191	0	0	0	0
TUBG2	CATGCACAACTGTGACAC	103	98	0	0	0	0
TWIST1	TGACGTTGCACATGGTTG	542	670	2	1	1	0
TWIST1	ACACGTTGCACATGGTCA	563	511	0	1	0	7
TWIST1	GTACGTTGCACATGGTAC	11	15	0	0	0	0
TWIST1	ACACGTTGCACATGCAGT	427	448	2	0	0	1
TWIST1	TGACGTTGCACATGCACA	12	5	0	0	0	0
TXLNA	TGTGGTCAACCACACATG	584	815	12	21	17	26833
TXLNA	ACTGGTCAACCACACACA	304	270	0	0	1	0
TXLNA	GTTGGTCAACCACACAAC	30	33	0	0	0	0
TXLNA	ACTGGTCAACCACAACGT	434	181	0	0	0	0
TXLNA	TGTGGTCAACCACAACCA	230	54	0	0	0	1
TXN	GTACTGACCATGTGTGCA	417	63	0	0	1	1
TXN	TGACTGACCATGTGTGGT	614	297	0	1	0	1
TXN	ACACTGACCATGTGTGAC	44	34	0	0	0	0
TXN	CAACTGACCATGTGTGTG	1682	1344	2	2	0	6
TXN	GTTGACGTCATGACACCA	153	239	0	0	0	1
TXN2	ACACTGACCATGCAGTGT	672	590	2893	4	0	2
TXN2	TGACTGACCATGCAGTCA	305	589	1	0	0	1
TXN2	CAACTGACCATGCAGTAC	136	128	0	1	0	4
TXN2	GTACTGACCATGCAGTTG	516	353	1	1	0	0

TXN2	TGACTGACCATGCACAGT	151	186	0	0	0	0
TXN2	GTTGCAACTGTGACCATG	262	306	1	0	0	0
TXNDC1	ACTGGTGTACCACACATG	220	334	1	0	0	0
TXNDC1	GTTGGTTGACCAGTCAGT	361	303	1	1	0	0
TXNDC1	CATGGTTGACCAGTCACA	99	215	1	0	0	0
TXNDC1	TGTGGTTGACCAGTCAAC	37	15	0	0	0	0
TXNDC1	CATGACACTGGTACGTAC	129	93	0	0	0	0
TXNRD1	TGACACGTTGTGCAGTCA	293	316	0	0	0	0
TXNRD1	CAACACGTTGTGCAGTAC	37	43	0	0	0	0
TXNRD1	GTACACGTTGTGCAGTTG	555	1383	0	0	0	0
TXNRD1	TGACACGTTGTGCACAGT	118	110	0	0	0	0
TXNRD1	GTACACGTTGTGCACACA	26	43	0	0	0	0
TXNRD2	TGACACGTTGACTGGTGT	379	777	0	0	0	0
TXNRD2	GTACACGTTGACTGGTCA	154	118	0	0	0	0
TXNRD2	ACACACGTTGACTGGTAC	45	81	0	0	0	0
TXNRD2	CAACACGTTGACTGGTTG	609	1101	1	1	0	5
TXNRD2	GTACACGTTGACTGCAGT	225	892	0	0	0	0
TXNRD2	GTTGCAACACACCATGAC	133	120	0	0	0	0
TYK2	CACATGACACCATGGTGT	764	430	11	4239	3	1
TYK2	GTCATGACACCAACTGAC	192	146	2	2	0	0
TYK2	TGCATGACACCAACTGTG	483	721	5	0	0	8
TYK2	ACCATGACACCATGGTCA	107	126	0	0	0	0
TYK2	GTCATGACACCATGGTAC	18	19	0	0	0	0
TYMS	TGACACGTCATGACACCA	141	162	14	6	3	23
TYMS	TGACACGTCATGACTGGT	1028	497	1	0	1	0
TYMS	CAACACGTCATGACACAC	32	18	0	0	0	0
TYMS	GTACACGTCATGACACTG	736	147	0	0	0	1
TYMS	GTACACGTCATGACTGCA	179	105	1	0	0	0
TYMS	GTTGCACACACAGTTGAC	31	27	0	0	0	0
TYR	CAACTGTGTGTGTGGTAC	50	45	0	0	0	0
TYR	GTACTGTGTGTGTGGTTG	849	516	1	0	0	0
TYR	TGACTGTGTGTGTGCAGT	161	256	1	0	0	0
TYR	GTACTGTGTGTGTGCACA	36	33	0	1	0	1
TYR	ACACTGTGTGTGTGCAAC	117	13	0	0	0	0
TYROBP	GTACCAACTGGTTGGTAC	43	95	0	0	1	0
TYROBP	ACTGCAGTACTGTGACCA	125	122	0	0	1	0
TYROBP	TGACCAACTGGTTGGTTG	1163	707	2	1	0	3
TYROBP	ACACCAACTGGTTGCAGT	324	207	0	0	0	0
TYROBP	TGACCAACTGGTTGCACA	99	118	0	0	0	0
UAP1	TGACTGACTGACACGTTG	805	855	3	1	1	45
UAP1	TGACTGACTGACCATGTG	334	515	0	0	0	4
UAP1	CAACTGACTGACACGTGT	808	602	0	0	0	0
UAP1	ACACTGACTGACACGTCA	767	415	1	0	0	0
UAP1	GTACTGACTGACACGTAC	46	41	0	0	0	0
UBB	CAACGTGTCAACTGACAC	20	21	0	0	0	0
UBB	ACACGTGTACACCATGAC	83	99	0	0	0	0
UBB	CAACGTGTACACCATGTG	502	666	0	0	0	0
UBB	TGACGTGTACACACGTGT	331	303	1	0	0	0
UBB	TGTGGTGTGTTGACCAGT	203	186	0	0	0	0
UBB	CATGCAACACTGGTACAC	57	59	0	0	0	0
UBE1	GTCATGTGGTACTGGTAC	31	110	0	0	0	0
UBE1	TGCATGTGGTACTGGTTG	560	300	0	0	0	0
UBE1	ACCATGTGGTACTGCAGT	152	267	1	0	0	0
UBE1	TGCATGTGGTACTGCACA	79	42	0	0	0	0
UBE1	CACATGTGGTACTGCAAC	7	5	0	0	0	0
UBE1	TGTGCAACTGGTACCAAC	25	17	0	0	0	0
UBE2A	CATGCAGTGTGTACCACA	82	75	0	0	1	0
UBE2A	GTCATGTGGTACTGCATG	1084	464	0	1	0	15
UBE2A	TGCATGTGGTACTGACGT	17	39	0	0	0	0
UBE2A	GTCATGTGGTACTGACCA	193	279	0	0	0	0
UBE2A	ACCATGTGGTACTGACAC	9	12	0	0	0	0
UBE2B	CACATGTGGTACTGACTG	576	394	1	0	0	0
UBE2B	GTCATGTGGTACTGTGGT	274	315	0	0	0	0
UBE2B	CACATGTGGTACTGTGCA	166	222	0	0	0	0
UBE2B	TGCATGTGGTACTGTGAC	11	19	0	0	0	0
UBE2B	ACCATGTGGTACTGTGTG	332	394	0	0	0	3
UBE2C	TGCATGTGCAGTGTGTAC	36	44	0	0	0	0
UBE2C	ACCATGTGCAGTGTGTTG	147	168	0	0	0	0
UBE2C	ACTGCATGCAACCAGTCA	925	907	2	1	0	2
UBE2C	GTTGCATGCAACCAGTAC	72	79	0	0	0	0
UBE2C	TGTGCATGCAACCAGTTG	549	598	2	1	0	1
UBE2D1	TGCATGACTGGTGTGTAC	66	45	0	0	0	0
UBE2D1	ACCATGACTGGTGTGTTG	373	375	0	1	0	0
UBE2D1	CACATGACTGGTGTCAGT	97	135	0	0	0	0
UBE2D1	ACCATGACTGGTGTCACA	71	62	0	0	0	0

UBE2D1	GTCATGACTGGTGTCAAC	57	63	0	0	0	0
UBE2D2	GTCATGACTGGTGTACTG	137	151	1	0	1	0
UBE2D2	TGCATGACTGGTGTCATG	83	47	0	0	0	0
UBE2D2	ACCATGACTGGTGTACGT	316	265	0	0	0	1
UBE2D2	TGCATGACTGGTGTACCA	226	167	1	0	0	0
UBE2D2	CACATGACTGGTGTACAC	28	41	0	0	0	0
UBE2D3	TGACCATGGTCATGTGCA	265	495	4	779	267	2
UBE2D3	ACACCATGGTCATGACCA	140	80	0	0	0	0
UBE2D3	GTACCATGGTCATGACAC	33	187	0	0	0	0
UBE2D3	TGACCATGGTCATGACTG	34	75	0	0	0	0
UBE2D3	ACACCATGGTCATGTGGT	788	505	1	2	0	1
UBE2E1	CACATGTGGTTGGTGTGT	691	536	1	0	0	0
UBE2E1	ACCATGTGGTTGGTGTCA	210	168	0	0	0	0
UBE2E1	GTCATGTGGTTGGTGTAC	66	216	1	0	0	0
UBE2E1	TGCATGTGGTTGGTGTTG	309	343	0	0	0	1
UBE2E1	ACCATGTGGTTGGTCAGT	78	151	0	1	0	0
UBE2E2	GTACGTGTACACACGTCA	94	257	0	0	0	0
UBE2E2	ACACGTGTACACACGTAC	27	20	0	0	0	0
UBE2E2	CAACGTGTACACACGTTG	62	53	0	0	0	0
UBE2E2	TGACCATGACCACAACCA	66	79	0	0	0	0
UBE2E2	CAACCATGACCACAACAC	194	152	0	0	0	2
UBE2E3	CAACGTGTACACGTACTG	404	512	2	1	2	0
UBE2E3	GTACGTGTACACGTCATG	85	26	0	0	0	0
UBE2E3	TGACGTGTACACGTACGT	591	192	0	0	0	0
UBE2E3	GTACGTGTACACGTACCA	116	167	0	0	0	0
UBE2E3	ACACGTGTACACGTACAC	49	78	0	0	0	0
UBE2F	GTACCACACAGTTGGTCA	226	207	1	1	1	0
UBE2F	ACACCACACAGTTGGTAC	99	170	0	0	0	0
UBE2F	CAACCACACAGTTGGTTG	313	540	2	0	0	0
UBE2F	GTACCACACAGTTGCAGT	299	321	0	0	0	0
UBE2F	CAACCACACAGTTGCACA	198	142	0	0	0	0
UBE2F	CATGCAACGTTGACACGT	79	80	0	0	0	0
UBE2G1	TGACGTGTCAACTGTGGT	253	265	0	0	0	3
UBE2G1	GTACGTGTCAACTGTGCA	157	137	0	0	0	0
UBE2G1	ACACGTGTCAACTGTGAC	48	51	0	0	0	0
UBE2G1	CAACGTGTCAACTGTGTG	121	119	2	0	0	0
UBE2G1	GTACGTGTCATGGTGTGT	578	554	0	1	0	0
UBE2G2	TGCATGTGGTTGGTCACA	134	125	5	3	0	7491
UBE2G2	CACATGTGGTTGGTCAAC	38	120	0	0	0	0
UBE2G2	GTCATGTGGTTGGTCATG	756	611	0	0	0	1
UBE2G2	TGCATGTGGTTGGTACGT	392	91	0	0	0	0
UBE2G2	GTCATGTGGTTGGTACCA	148	163	0	0	0	1
UBE2H	GTCATGTGGTTGGTTGGT	742	452	0	0	4	1
UBE2H	ACCATGTGGTTGGTACAC	51	107	0	0	0	0
UBE2H	CACATGTGGTTGGTACTG	525	282	1	0	0	0
UBE2H	CACATGTGGTTGGTTGCA	27	16	0	0	0	0
UBE2H	TGCATGTGGTTGGTTGAC	2	2	0	0	0	0
UBE2I	CAACGTGTCATGGTGTCA	183	253	0	0	0	2
UBE2I	TGACGTGTCATGGTGTAC	13	14	0	0	0	0
UBE2I	ACACGTGTCATGGTGTTG	669	355	0	0	0	3
UBE2I	ACTGCAGTACCACACAAC	32	39	1	0	0	0
UBE2I	CATGCAGTACCACACATG	402	501	2	4	0	1
UBE2J1	ACCATGTGGTTGACACCA	429	361	0	0	0	0
UBE2J1	GTCATGTGGTTGACACAC	77	37	0	0	0	0
UBE2J1	TGCATGTGGTTGACACTG	202	218	0	0	0	0
UBE2J1	ACCATGTGGTTGACTGGT	464	375	2	0	0	0
UBE2J1	TGCATGTGGTTGACTGCA	221	130	1	0	0	38
UBE2J2	GTACCACACAGTACTGCA	155	183	1	0	0	1
UBE2J2	ACACCACACAGTACTGAC	21	33	0	1	0	0
UBE2J2	CAACCACACAGTACTGTG	669	1071	0	2	0	11
UBE2J2	TGACCACACAGTTGGTGT	602	439	2	1	0	1
UBE2J2	GTACCATGACCACACAAC	73	130	411	1	0	0
UBE2L3	CAACGTGTCATGGTCAGT	594	838	44	0	0	2
UBE2L3	ACACGTGTCATGGTCACA	16	20	0	0	0	0
UBE2L3	GTACGTGTCATGGTCAAC	34	40	0	0	0	1
UBE2L3	TGACGTGTCATGGTCATG	138	205	1	0	0	3
UBE2L3	ACACGTGTCATGGTACGT	150	206	0	0	0	0
UBE2L6	TGACGTGTCATGTGGTTG	1041	174	1	0	1	0
UBE2L6	ACACGTGTCATGTGCAGT	81	75	0	0	0	1
UBE2L6	TGACGTGTCATGTGCACA	101	65	0	0	0	0
UBE2L6	CAACGTGTCATGTGCAAC	21	31	0	0	0	0
UBE2L6	GTTGCATGACTGCATGCA	128	383	0	0	0	0
UBE2M	TGACGTGTCATGACGTCA	180	152	0	0	0	1
UBE2M	CAACGTGTCATGACGTAC	20	17	0	0	0	0
UBE2M	GTACGTGTCATGACGTTG	155	232	2	0	0	0

UBE2M	TGACGTGTCATGACCAGT	346	221	3	0	0	0
UBE2M	GTACGTGTCATGACCACA	155	54	0	1	0	0
UBE2N	TGACGTGTCATGGTACCA	117	303	0	0	0	8
UBE2N	CAACGTGTCATGGTACAC	15	149	1	1	0	0
UBE2N	GTACGTGTCATGGTACTG	569	443	1	0	0	0
UBE2N	TGACGTGTCATGGTTGGT	189	203	0	0	0	0
UBE2N	GTACGTGTCATGGTTGCA	218	105	0	0	0	0
UBE2V1	CAACCACACAGTCAACTG	895	934	0	3	1	2
UBE2V1	GTACCACACAGTCAACCA	474	356	1	0	0	1
UBE2V1	ACACCACACAGTCAACAC	51	51	0	0	0	0
UBE2V1	GTACCACACAGTCATGGT	236	252	1	0	0	1
UBE2V1	CAACCACACAGTCATGCA	228	217	1	0	0	1
UBE2V2	ACCATGTGGTTGGTTGTG	47	16	0	0	0	0
UBE2V2	GTCATGTGGTTGCAGTGT	162	189	0	0	0	0
UBE2V2	CACATGTGGTTGCAGTCA	355	334	0	0	0	0
UBE2V2	TGCATGTGGTTGCAGTAC	12	15	0	0	0	0
UBE2V2	ACACCATGACCACAACGT	683	609	0	3	0	0
UBE2V2	GTTGCACATGACACGTCA	191	202	0	0	0	0
UBE3A	ACCATGACACTGTGACGT	456	483	411	527	574	1653856
UBE3A	TGCATGACACTGTGACCA	625	186	0	0	0	0
UBE3A	CACATGACACTGTGACAC	19	38	0	0	0	0
UBE3A	GTCATGACACTGTGACTG	215	116	0	0	0	0
UBE3A	TGCATGACACTGTGTGGT	737	986	3	0	0	0
UBE3A	GTTGCAACCAGTACTGAC	28	42	0	0	0	0
UBQLN1	TGCATGTGCAGTACCAGT	330	188	0	0	0	0
UBQLN1	GTCATGTGCAGTACCACA	76	88	0	0	0	0
UBQLN1	ACCATGTGCAGTACCAAC	5	5	0	0	0	0
UBQLN1	CACATGTGCAGTACCATG	230	313	0	0	0	0
UBQLN1	GTCATGTGCAGTACACGT	125	33	0	0	0	0
UBTF	GTACGTTGCAACGTCACA	114	94	3	4	2	1
UBTF	GTACGTTGCAACGTACGT	126	218	1	1	1	0
UBTF	ACACGTTGCAACGTCAAC	23	25	0	0	0	0
UBTF	CAACGTTGCAACGTCATG	263	152	1	0	0	1
UBTF	CAACGTTGCAACGTACCA	664	194	1	0	0	0
UCHL1	TGACGTGTCATGACTGTG	350	276	0	0	0	0
UCHL1	CAACGTGTCATGTGGTGT	1102	1044	1	0	0	3
UCHL1	ACACGTGTCATGTGGTCA	221	254	1	0	0	4
UCHL1	GTACGTGTCATGTGGTAC	35	72	0	0	0	0
UCHL1	GTTGCAGTACCACAACGT	330	256	0	0	0	0
UCHL3	ACACGTGTACCATGACGT	76	87	0	0	0	0
UCHL3	TGACGTGTACCATGACCA	91	94	0	0	0	0
UCHL3	CAACGTGTACCATGACAC	6	9	0	0	0	0
UCHL3	GTACGTGTACCATGACTG	150	200	1	0	0	0
UCHL3	TGACGTGTACCATGTGGT	489	1072	1	0	0	0
UCN	ACACTGTGCAACGTTGGT	278	447	0	0	0	0
UCN	TGACTGTGCAACGTTGCA	450	168	1	0	0	2
UCN	CAACTGTGCAACGTTGAC	57	66	0	0	0	0
UCN	GTACTGTGCAACGTTGTG	358	387	1	0	0	0
UCN	ACACTGTGCAACCAGTGT	552	478	3	2	0	0
UCN	TGTGCACATGACACCAAC	134	25	2	0	0	0
UCP1	ACACTGCACAACACACCA	443	449	0	0	0	5
UCP1	GTACTGCACAACACACAC	67	38	0	0	0	0
UCP1	TGACTGCACAACACACTG	272	218	1	0	0	0
UCP1	ACACTGCACAACACTGGT	696	806	1	0	0	10
UCP1	TGACTGCACAACACTGCA	447	276	0	0	0	0
UCP1	TGTGCAACACCAACCAAC	22	28	0	0	0	0
UCP2	GTACTGCACAACACTGTG	654	228	0	0	1	0
UCP2	CAACTGCACAACACTGAC	124	274	0	0	0	0
UCP2	ACACTGCACAACTGGTGT	200	212	0	0	0	1
UCP2	TGACTGCACAACTGGTCA	347	548	0	0	0	2
UCP2	CAACTGCACAACTGGTAC	39	70	1	0	0	0
UCP2	GTTGCACATGTGCAGTAC	37	44	0	0	0	0
UCP3	ACTGGTTGTGGTGTGTAC	239	49	0	0	1	0
UCP3	GTACTGCACAACTGGTTG	1505	867	2	0	0	1
UCP3	TGACTGCACAACTGCAGT	187	130	0	1	0	0
UCP3	GTACTGCACAACTGCACA	384	320	0	2	0	0
UCP3	GTTGGTTGTGGTGTGTCA	627	710	16	0	0	1
UCP3	TGTGCACATGTGCAGTTG	163	120	0	0	0	0
UGCG	CAACCAACCAGTACACTG	286	184	0	0	2	0
UGCG	ACACCAACCAGTACACAC	13	11	0	0	0	0
UGCG	GTACCAACCAGTACTGGT	383	611	0	0	0	0
UGCG	CAACCAACCAGTACTGCA	331	340	0	0	0	0
UGCG	TGACCAACCAGTACTGAC	279	40	0	0	0	0
UGCG	TGTGCACACACAGTTGTG	311	141	0	0	0	0
UGDH	ACACCAGTGTGTCATGGT	360	376	0	0	0	2

UGDH	TGACCAGTGTGTCATGCA	108	116	0	0	0	1
UGDH	CAACCAGTGTGTCATGAC	123	27	0	0	0	0
UGDH	GTACCAGTGTGTCATGTG	686	783	1	2	0	0
UGDH	ACTGCAGTCAACTGCACA	44	55	0	0	0	0
UGP2	CAACCAACTGGTTGCAAC	26	30	0	0	0	0
UGP2	GTACCAACTGGTTGCATG	131	160	0	0	0	0
UGP2	TGACCAACTGGTTGACGT	342	251	0	0	0	0
UGP2	GTACCAACTGGTTGACCA	148	109	0	0	0	0
UGP2	GTTGACGTCAACGTGTTG	151	146	0	0	0	0
UGT1A1	GTACCAGTTGGTCACATG	477	823	0	0	0	0
UGT1A1	TGACCAGTTGGTCAACGT	317	321	0	0	0	0
UGT1A1	ACACCAGTTGGTCAACAC	52	40	0	0	0	0
UGT1A1	CAACCAGTTGGTCAACTG	144	205	0	1	0	0
UGT1A1	TGTGACGTGTTGGTTGTG	329	316	0	0	0	1
UGT1A10	ACACCAACCATGGTCAGT	124	433	0	0	1	0
UGT1A10	GTACCACACAACACACCA	237	307	0	0	0	0
UGT1A10	GTACCAACCATGGTGTAC	26	111	0	0	0	0
UGT1A10	TGACCAACCATGGTGTTG	508	504	0	0	0	0
UGT1A10	GTTGCAGTACTGACTGCA	146	51	0	0	0	0
UGT1A3	ACACCACACAACCAGTGT	370	695	2	0	1	0
UGT1A3	TGACCACACAACGTTGCA	238	377	2	0	0	1
UGT1A3	CAACCACACAACGTTGAC	63	83	0	0	0	0
UGT1A3	GTACCACACAACGTTGTG	400	366	0	0	0	0
UGT1A3	TGACCAACCATGGTCACA	51	50	0	0	0	0
UGT1A4	CAACCAACCAACTGGTGT	1666	1807	6	0	4	0
UGT1A4	ACACCAACCAACTGGTCA	401	377	0	0	1	0
UGT1A4	GTACCAACCAACTGGTAC	35	49	0	0	0	0
UGT1A4	TGACCAACCAACTGGTTG	745	941	2	0	0	0
UGT1A4	TGTGCAGTACTGACACCA	339	365	0	0	0	0
UGT1A5	GTACCACACACATGGTTG	503	689	3	0	0	0
UGT1A5	TGACCACACACATGCAGT	225	174	0	0	0	0
UGT1A5	GTACCACACACATGCACA	138	109	0	0	0	1
UGT1A5	ACACCACACACATGCAAC	58	126	0	1	0	0
UGT1A5	CAACCACACACATGCATG	427	804	1	0	0	0
UGT1A6	ACACCACACATGACCAAC	17	13	0	0	0	0
UGT1A6	CAACCACACATGACCATG	502	419	1	0	0	11
UGT1A6	TGTGACACACGTACTGCA	231	233	0	1	0	0
UGT1A6	CATGACACACGTACTGAC	67	51	0	1	0	0
UGT1A6	GTTGACACACGTACTGTG	553	88	0	0	0	36
UGT1A7	GTACCAGTTGGTCAACCA	32	53	0	0	0	0
UGT1A7	TGACCACACACAACCAAC	20	17	0	0	0	0
UGT1A7	GTACCACACATGACACGT	43	54	0	0	0	0
UGT1A7	CAACCACACATGACACCA	371	197	0	0	0	0
UGT1A7	TGACCACACATGACACAC	13	16	0	0	0	0
UGT1A8	ACACCAACCATGCACACA	202	193	0	1	0	0
UGT1A8	GTACCAACCATGCACAAC	37	52	0	2	0	0
UGT1A8	TGACCAACCATGCACATG	264	339	1	1	0	1
UGT1A8	ACACCAACCATGCAACGT	1259	873	1	0	0	1
UGT1A8	GTTGACGTGTTGCAGTAC	118	79	0	0	0	2
UGT1A9	ACACCACACAACACACAC	73	74	2	0	0	0
UGT1A9	CAACCACACAACACACTG	224	252	0	0	0	0
UGT1A9	GTACCACACAACACTGGT	469	727	146	0	0	0
UGT1A9	CAACCACACAACACTGCA	269	437	1	0	0	0
UGT1A9	ACTGACGTGTTGCACAGT	166	157	0	39	0	0
UGT2A1	GTACCAACCAACACGTTG	1119	706	3	1	1	0
UGT2A1	ACACCAACCAACACGTGT	385	342	0	0	0	0
UGT2A1	TGACCAACCAACACGTCA	342	195	0	0	0	0
UGT2A1	CAACCAACCAACACGTAC	312	191	0	0	0	0
UGT2A1	TGACCAACCAACACCAGT	179	479	0	0	0	0
UGT2A3	TGTGGTCATGTGTGCATG	79	73	0	0	0	0
UGT2A3	ACTGGTCATGTGTGACGT	160	248	1	0	0	0
UGT2A3	TGTGGTCATGTGTGACCA	291	493	0	0	0	0
UGT2A3	CATGGTCATGTGTGACAC	53	29	0	0	0	0
UGT2A3	CATGACGTGTGTTGACTG	142	257	1	0	0	0
UGT2B10	GTACCAACCACAACCAAC	25	21	0	0	0	0
UGT2B10	TGACCAACCACAACCATG	733	678	2	0	0	0
UGT2B10	ACACCAACCACAACACGT	38	23	0	0	0	0
UGT2B10	TGACCAACCACAACACCA	298	297	1	0	0	4
UGT2B10	CAACCAACCACAACACAC	24	27	0	0	0	0
UGT2B11	ACACCAACCACAGTACTG	256	431	3	0	0	0
UGT2B11	CAACCAACCACAGTTGGT	246	224	0	0	0	0
UGT2B11	ACACCAACCACAGTTGCA	147	281	0	0	0	0
UGT2B11	GTACCAACCACAGTTGAC	68	26	0	0	0	0
UGT2B11	TGACCAACCACAGTTGTG	1063	568	0	2	0	0
UGT2B15	GTACCAACCACATGCAGT	208	329	0	0	0	0

UGT2B15	GTACCAACCAACGTTGCA	155	197	0	0	0	0
UGT2B15	ACACCAACCAACGTTGAC	30	42	0	0	0	0
UGT2B15	ACTGCAGTACTGACGTTG	235	258	0	0	0	0
UGT2B15	TGTGACACCACAGTACAC	39	58	0	0	0	0
UGT2B17	CAACCAACCACACACAAC	4	2	0	0	0	0
UGT2B17	CAACCAACCACATGCACA	182	157	1	0	0	0
UGT2B17	TGACCAACCACATGCAAC	53	51	0	0	0	0
UGT2B17	GTACCAACCAACGTACTG	369	353	1	0	0	1
UGT2B17	TGACCAACCAACGTTGGT	173	257	0	0	0	15
UGT2B28	GTACCACACATGGTTGCA	246	120	2	0	2	1
UGT2B28	ACACCACACATGGTTGAC	99	255	0	0	0	2
UGT2B28	CAACCACACATGGTTGTG	129	125	0	0	0	0
UGT2B28	TGACCACACATGCAGTGT	655	276	3	1	0	18
UGT2B28	TGACCAACCACACACACA	76	68	0	0	0	0
UGT2B4	CAACCACACAACTGACAC	87	55	0	1	0	0
UGT2B4	GTACCACACAACTGACTG	94	134	0	0	0	0
UGT2B4	TGACCACACAACTGTGGT	92	106	0	0	0	0
UGT2B4	GTACCACACAACTGTGCA	116	121	0	1	0	0
UGT2B4	ACACCACACAACTGTGAC	113	125	0	0	0	0
UGT2B4	CATGCACATGCAACACTG	445	401	0	1	0	3
UGT8	CAACCAACGTGTTGTGAC	279	88	0	0	0	1
UGT8	GTACCAACGTGTTGTGTG	163	165	0	0	0	0
UGT8	CAACCAACGTCAGTGTGT	612	362	2	2	0	0
UGT8	ACACCAACGTCAGTGTCA	1064	741	803	0	0	0
UGT8	GTACCAACGTCAGTGTAC	30	22	0	0	0	0
UHRF1	TGCATGTGCAGTCACAAC	8	22	0	0	0	0
UHRF1	ACCATGTGCAGTCACATG	89	63	5	0	0	0
UHRF1	CACATGTGCAGTCAACGT	379	543	0	1	0	1
UHRF1	ACCATGTGCAGTCAACCA	140	174	1	0	0	0
UHRF1	GTCATGTGCAGTCAACAC	27	46	0	0	0	1
ULK1	TGCATGCATGGTCAGTCA	388	237	2	0	3	0
ULK1	CACATGCATGGTCAGTAC	33	35	0	0	0	0
ULK1	GTCATGCATGGTCAGTTG	1013	1087	4	3	0	6
ULK1	GTTGGTACTGTGCACAGT	83	62	0	0	0	1
ULK1	CATGGTACTGTGCACACA	351	302	0	0	0	0
UNC13A	TGTGCAGTGTCAGTTGGT	478	550	7	9	2	5
UNC13A	CATGCAGTGTCAGTACAC	30	13	0	0	0	0
UNC13A	GTTGCAGTGTCAGTACTG	527	266	3	0	0	0
UNC13A	GTTGCAGTGTCAGTTGCA	78	116	0	0	0	0
UNC13A	CATGACACGTTGACGTAC	21	27	0	0	0	0
UNC13B	ACCATGACCACAACCACA	166	67	0	0	0	0
UNC13B	GTCATGACCACAACCAAC	15	29	0	0	0	0
UNC13B	TGCATGACCACAACCATG	160	194	0	0	0	0
UNC13B	ACCATGACCACAACACGT	14	21	0	0	0	0
UNC13B	TGCATGACCACAACACCA	138	197	0	0	0	0
UNC5B	GTACTGCATGCACAACAC	60	83	1	0	0	0
UNC5B	TGACTGCATGCACAACTG	835	652	0	1	0	0
UNC5B	ACACTGCATGCACATGGT	1272	1511	1	2	0	1
UNC5B	TGACTGCATGCACATGCA	52	47	0	0	0	1
UNC5B	CAACTGCATGCACATGAC	28	29	0	0	0	0
UNC5B	GTTGCATGCACACAGTCA	212	199	0	0	0	0
UNC5CL	CATGGTCACATGCATGCA	161	278	0	0	0	0
UNC5CL	TGTGGTCACATGCATGAC	24	32	0	0	0	0
UNC5CL	ACTGGTCACATGCATGTG	892	1630	4	2	0	1
UNC5CL	GTTGGTCACATGACGTGT	212	170	2	0	0	0
UNC5CL	CATGGTCACATGACGTCA	193	198	0	1	0	1
UNC5CL	TGTGCATGGTGTCAGTGT	119	220	0	0	0	33
UNC93B1	CATGGTCAACGTGTACCA	195	177	0	0	0	0
UNC93B1	TGTGGTCAACGTGTACAC	29	31	0	0	0	0
UNC93B1	ACTGGTCAACGTGTACTG	184	142	0	0	0	12
UNC93B1	CATGGTCAACGTGTTGGT	402	351	1	0	0	1
UNC93B1	ACTGGTCAACGTGTTGCA	11	13	0	0	0	0
UNG	GTACTGTGACTGTGGTCA	434	209	0	0	0	0
UNG	ACACTGTGACTGTGGTAC	212	25	0	0	0	0
UNG	CAACTGTGACTGTGGTTG	730	861	1	4	0	1
UNG	GTACTGTGACTGTGCAGT	166	97	0	0	0	7
UNG	CAACTGTGACTGTGCACA	287	173	0	0	0	0
UNG	CATGCAACCAGTACTGGT	353	382	0	0	0	7
UPB1	GTTGGTCAGTTGCACAAC	17	5	0	0	0	0
UPB1	TGTGGTCAGTTGCACATG	315	209	1	1	0	0
UPB1	ACTGGTCAGTTGCAACGT	592	396	0	0	0	0
UPB1	TGTGGTCAGTTGCAACCA	183	263	2	0	0	0
UPB1	ACTGCACAGTGTACTGAC	39	27	0	0	0	0
UQCRC1	GTACACGTTGACTGACAC	22	68	0	0	0	0
UQCRC1	TGACACGTTGACTGACTG	29	25	0	0	0	0

UQCRC1	ACACACGTTGACTGTGGT	667	603	2	0	0	1
UQCRC1	TGACACGTTGACTGTGCA	132	225	1	0	0	0
UQCRC1	CAACACGTTGACTGTGAC	144	100	0	0	0	0
UQCRC2	CAACACGTTGACTGCACA	34	40	0	0	0	0
UQCRC2	TGACACGTTGACTGCAAC	8	5	0	0	0	0
UQCRC2	ACACACGTTGACTGCATG	132	448	0	0	0	0
UQCRC2	CAACACGTTGACTGACGT	75	69	0	0	0	2
UQCRC2	ACACACGTTGACTGACCA	239	261	1	0	0	0
UQCRH	GTACACGTTGTGGTTGTG	204	178	1	0	1	1
UQCRH	CAACACGTTGTGGTTGAC	37	103	0	0	0	0
UQCRH	ACACACGTTGTGCAGTGT	769	957	1	0	0	2
UQCRH	ACTGCAGTCATGGTTGAC	53	38	0	0	0	0
UQCRH	CATGCAGTCATGGTTGTG	151	175	0	1	0	0
UROD	GTACTGTGACGTACTGAC	91	54	0	0	0	0
UROD	TGACTGTGACGTACTGTG	129	313	1	0	0	0
UROD	CAACTGTGACGTTGGTGT	918	1248	1	3	0	1
UROD	ACACTGTGACGTTGGTCA	321	270	1	0	0	0
UROD	GTACTGTGACGTTGGTAC	27	28	0	0	0	0
UROS	CAACACGTACCAGTCAAC	76	106	4	0	1	0
UROS	GTACACGTACCAGTGTAC	49	51	0	0	0	0
UROS	TGACACGTACCAGTGTTG	477	787	0	0	0	2
UROS	ACACACGTACCAGTCAGT	117	203	0	0	0	0
UROS	TGACACGTACCAGTCACA	164	111	0	0	0	0
UROS	CATGCACACAACACTGGT	136	102	0	2	0	0
USH1C	GTTGGTGTGTTGCAGTCA	219	613	28	7	20	39
USH1C	TGTGGTGTGTTGCAGTGT	343	379	0	2	0	3
USH1C	ACTGGTGTGTTGCAGTAC	8	39	0	0	0	0
USH1C	CATGGTGTGTTGCAGTTG	1072	1043	0	1	0	0
USH1C	GTTGGTGTGTTGCACAGT	36	137	1	0	0	0
USP11	CAACGTGTACGTACGTAC	45	67	0	1	0	0
USP11	GTACGTGTACGTACGTTG	247	177	1	0	0	2
USP11	TGACGTGTACGTACCAGT	118	29	0	0	0	0
USP11	TGTGCAGTCATGACCAGT	511	233	0	1	0	0
USP11	GTTGCAGTCATGACCACA	51	54	0	0	0	0
USP9X	GTACGTGTACGTACCACA	55	17	0	0	0	0
USP9X	ACACGTGTACGTACCAAC	13	9	0	0	0	0
USP9X	CAACGTGTACGTACCATG	70	66	0	0	0	0
USP9X	GTACGTGTACGTACACGT	6	4	0	0	0	0
USP9X	ACTGGTTGTGCATGTGGT	1107	1070	2	2	0	0
UST	TGACCACACATGTGGTTG	1004	2079	1	36	0	0
UST	ACACCACACATGTGCAGT	243	257	1	0	0	0
UST	TGACCACACATGTGCACA	174	182	0	0	0	0
UST	CAACCACACATGTGCAAC	56	62	0	0	0	0
UST	GTACCACACATGTGCATG	87	72	0	0	0	0
UST	CATGCACAACGTCAACAC	23	45	0	0	0	0
UTF1	TGACGTCAACACCACACA	157	262	0	1	1	0
UTF1	GTTGACCACACACAGTGT	954	317	0	0	1	1
UTF1	CAACGTCAACACCACAAC	101	72	0	0	0	0
UTF1	CATGCAGTACACGTTGCA	187	181	2	0	0	0
UTF1	ACTGACCACACAGTTGTG	737	197	3	0	0	4
UVRAG	ACCATGTGACGTTGGTAC	24	46	0	0	0	0
UVRAG	CACATGTGACGTTGGTTG	903	1028	3	0	0	1
UVRAG	GTCATGTGACGTTGCAGT	174	155	1	0	0	0
UVRAG	CACATGTGACGTTGCACA	44	60	0	0	0	0
UVRAG	TGCATGTGACGTTGCAAC	33	35	0	0	0	0
UXS1	CAACTGTGACCACAGTAC	38	70	0	0	0	0
UXS1	GTACTGTGACCACAGTTG	311	343	1	2	0	0
UXS1	TGACTGTGACCACACAGT	506	478	1	0	0	0
UXS1	GTACTGTGACCACACACA	30	25	0	0	0	0
UXS1	ACACTGTGACCACACAAC	8	30	0	0	0	0
UXT	GTTGGTGTCACAGTCACA	268	241	2	0	0	0
UXT	ACTGGTACCAGTGTGTCA	467	412	1	0	0	0
UXT	GTTGGTACCAGTGTGTAC	56	137	0	0	0	0
UXT	TGTGGTACCAGTGTGTTG	505	243	0	1	0	0
UXT	ACTGGTACCAGTGTCAGT	369	272	0	0	0	3
UXT	GTTGCAACTGGTTGACGT	607	1005	1	1	0	1
VAMP1	GTTGGTTGACACTGGTTG	784	1256	0	0	1	0
VAMP1	GTTGGTTGACACACTGTG	301	452	0	0	0	0
VAMP1	ACTGGTTGACACTGGTGT	4	52	0	0	0	0
VAMP1	TGTGGTTGACACTGGTCA	14	23	0	0	0	0
VAMP1	CATGGTTGACACTGGTAC	74	57	1	0	0	0
VAMP2	CATGCACAACTGGTTGAC	256	183	0	1	1	1
VAMP2	TGTGGTGTACGTTGCAGT	202	187	2	0	0	0
VAMP2	GTTGGTACGTGTCAACAC	96	100	0	0	0	2
VAMP2	TGTGGTACGTGTCAACTG	265	250	1	0	0	1

VAMP2	ACTGGTACGTGTCATGGT	562	403	1	2	0	0
VAMP2	TGTGGTACGTGTCATGCA	116	200	0	1	0	0
VAMP5	ACTGGTACCATGGTTGGT	876	969	653446	1067	668	1149
VAMP5	ACTGGTACCATGGTACCA	96	46	0	0	0	0
VAMP5	GTTGGTACCATGGTACAC	55	15	0	0	0	0
VAMP5	TGTGGTACCATGGTACTG	514	518	0	0	0	2
VAMP5	TGTGGTACCATGGTTGCA	59	47	0	0	0	0
VAMP5	ACTGCATGCAGTACGTAC	83	48	0	0	0	0
VAMP7	CAACTGCACAACGTACTG	209	223	1	0	1	0
VAMP7	ACACTGCACAACGTACAC	67	58	0	0	0	0
VAMP7	GTACTGCACAACGTTGGT	189	90	0	1	0	0
VAMP7	CAACTGCACAACGTTGCA	246	214	0	0	0	0
VAMP7	TGACTGCACAACGTTGAC	13	28	0	0	0	0
VAMP7	GTTGCATGGTGTGTTGCA	100	205	0	0	0	0
VANGL1	ACACTGCATGACACGTTG	1419	1065	27	39	3	55
VANGL1	ACACTGCATGACCATGTG	1294	1255	2	1	0	2
VANGL1	GTACTGCATGACACGTGT	440	565	0	0	0	0
VANGL1	CAACTGCATGACACGTCA	69	67	0	0	0	0
VANGL1	TGACTGCATGACACGTAC	37	50	0	0	0	0
VANGL2	ACTGGTTGACCAGTCATG	167	200	0	0	0	0
VANGL2	CATGGTTGACCAGTACGT	250	285	1	0	0	7
VANGL2	ACTGGTTGACCAGTACCA	41	38	0	0	0	0
VANGL2	GTTGGTTGACCAGTACAC	21	22	0	0	0	0
VANGL2	ACTGACCAGTCAACACTG	203	157	0	0	0	0
VARS	CAACACGTACCATGTGGT	582	316	1	0	0	0
VARS	ACACACGTACCATGTGCA	87	42	0	0	0	0
VARS	GTACACGTACCATGTGAC	9	8	0	0	0	0
VARS	ACTGCAGTCAGTCAACAC	140	141	0	0	0	0
VARS	CATGCAGTCAGTCAACTG	565	542	0	1	0	1
VARS2	CATGGTTGGTACACGTCA	193	141	0	0	0	0
VARS2	TGTGGTTGGTACACGTAC	14	24	0	0	0	0
VARS2	ACTGGTTGGTACACGTTG	323	420	0	1	0	3
VARS2	CATGGTTGGTACACCAGT	149	152	1	0	0	0
VARS2	ACTGGTTGGTACACCACA	120	165	0	0	0	0
VAV1	GTACCATGCACAACCATG	158	121	0	0	0	1
VAV1	TGACCATGCACAACACGT	151	109	0	0	0	0
VAV1	GTACCATGCACAACACCA	226	341	1	0	0	0
VAV1	ACACCATGCACAACACAC	78	137	0	0	0	0
VAV1	CAACCATGCACAACACTG	549	270	0	1	0	2
VAV1	CATGCACAACACACTGTG	430	758	1	0	0	4
VAV2	CAACACCACAACGTTGGT	328	437	0	0	0	2
VAV2	ACACACCACAACGTTGCA	106	124	0	0	0	0
VAV2	GTACACCACAACGTTGAC	33	62	0	0	0	0
VAV2	TGACACCACAACGTTGTG	266	286	0	0	0	0
VAV2	CAACACCACAACCAGTGT	207	240	1	0	0	0
VAV3	CAACACCACAGTCACAGT	401	352	1	0	0	1
VAV3	ACACACCACAGTCACACA	144	159	0	0	0	0
VAV3	GTACACCACAGTCACAAC	143	34	0	0	0	0
VAV3	TGACACCACAGTCACATG	459	305	1	0	0	4
VAV3	ACACACCACAGTCAACGT	788	937	2	548	0	3
VAX2	GTACGTACACGTGTACGT	187	108	3	0	0	0
VAX2	CAACGTACACGTGTACCA	44	51	0	0	0	0
VAX2	TGACGTACACGTGTACAC	20	20	0	0	0	0
VAX2	ACACGTACACGTGTACTG	145	215	0	0	0	1
VAX2	CAACGTACACGTGTTGGT	373	286	0	0	0	0
VAX2	GTTGCACAACACTGGTCA	322	769	0	3	0	0
VCAM1	ACTGGTCACACAACCAGT	124	96	253	0	0	0
VCAM1	TGTGGTCACACAACCACA	81	147	0	1	0	0
VCAM1	CATGGTCACACAACCAAC	36	33	0	0	0	0
VCAM1	GTTGGTCACACAACCATG	177	184	0	0	0	0
VCAM1	ACTGCAACGTTGTGCAAC	30	18	0	0	0	0
VCAM1	CATGCAACGTTGTGCATG	66	49	0	0	0	0
VCAN	CAACCACAGTTGTGTGTG	226	241	295195	316	21	34
VCAN	TGACCACAGTTGTGTGGT	113	82	0	0	0	0
VCAN	GTACCACAGTTGTGTGCA	109	286	0	0	0	0
VCAN	ACACCACAGTTGTGTGAC	41	10	0	0	0	0
VCAN	ACACCACACAGTGTGTGT	395	257	0	1	0	0
VCAN	GTTGCAACACGTGTTGAC	23	22	0	1	0	0
VCL	GTTGGTGTTGGTACGTTG	667	362	1	0	2	0
VCL	TGTGGTGTTGGTACGTCA	137	139	0	0	0	1
VCL	CATGGTGTTGGTACGTAC	100	111	0	0	0	0
VCL	TGTGGTGTTGGTACCAGT	342	232	1	0	0	0
VCL	GTTGGTGTTGGTACCACA	65	33	0	0	0	0
VDR	ACACGTTGGTGTGTGTTG	722	641	0	1	0	0
VDR	CAACGTTGGTGTGTCAGT	149	209	0	0	0	1

VDR	ACACGTTGGTGTGTCACA	17	17	0	0	0	0
VDR	GTACGTTGGTGTGTCAAC	17	109	0	0	0	0
VDR	TGACGTTGGTGTGTCATG	22	40	0	0	0	0
VEGFA	ACCATGACACTGACGTCA	101	170	0	0	0	0
VEGFA	GTCATGACACTGACGTAC	23	18	0	2	0	0
VEGFA	TGCATGACACTGACGTTG	520	601	0	1	0	0
VEGFA	ACCATGACACTGACCAGT	126	110	0	0	0	1
VEGFA	TGACTGACTGCAACACTG	143	155	0	0	0	0
VEGFC	TGACTGGTACACTGCAAC	31	24	0	0	0	0
VEGFC	ACACTGGTACACTGCATG	195	236	0	0	0	0
VEGFC	CAACTGGTACACTGACGT	249	412	0	0	0	0
VEGFC	ACACTGGTACACTGACCA	113	121	0	0	0	0
VEGFC	GTACTGGTACACTGACAC	88	43	0	0	0	0
VEGFC	ACTGCAACCAACGTGTCA	420	289	47	0	0	2
VHL	TGACCATGGTTGCATGGT	135	410	0	0	1	6
VHL	GTACCATGGTTGCAACTG	730	746	0	1	0	12
VHL	GTACCATGGTTGCATGCA	95	160	0	0	0	0
VHL	ACTGGTTGGTACACTGAC	82	26	0	0	0	0
VHL	ACTGCACAGTGTTGACCA	111	108	0	0	0	0
VIL2	GTTGCAACACCACAACTG	340	1093	0	0	1	1
VIL2	ACACTGACGTGTCATGGT	521	370	0	0	0	0
VIL2	TGACTGACGTGTCATGCA	342	314	1	0	0	0
VIL2	CAACTGACGTGTCATGAC	32	30	0	0	0	0
VIL2	GTACTGACGTGTCATGTG	263	302	1	0	0	0
VIL2	ACACTGACGTGTACGTGT	300	278	1	0	0	0
VIM	ACACCAGTACGTTGGTTG	1313	1728	0	2	1	0
VIM	GTACCAGTACGTTGGTGT	327	657	1	1	0	0
VIM	CAACCAGTACGTTGGTCA	192	118	0	0	0	1
VIM	TGACCAGTACGTTGGTAC	11	9	0	0	0	0
VIM	CAACCAGTACGTTGCAGT	310	240	1	0	0	0
VIP	TGACTGTGCAACCAGTCA	406	147	0	0	0	0
VIP	CAACTGTGCAACCAGTAC	232	120	0	1	0	0
VIP	GTACTGTGCAACCAGTTG	751	594	3	0	0	0
VIP	TGACTGTGCAACCACAGT	133	189	0	2	0	0
VIP	GTACTGTGCAACCACACA	219	315	3	0	0	4
VIPR1	CAACGTCATGTGTGTGAC	40	28	0	0	0	0
VIPR1	GTACGTCATGTGTGTGTG	315	276	0	1	0	0
VIPR1	TGACGTACGTGTGTGTGT	379	365	0	1	0	0
VIPR1	GTACGTACGTGTGTGTCA	157	392	1	0	0	0
VIPR1	ACACGTACGTGTGTGTAC	15	9	0	0	0	0
VIPR1	ACTGCAACGTCATGTGGT	451	525	0	1	0	16
VISA	CATGGTCATGACTGCACA	226	261	1	0	0	0
VISA	TGTGGTCATGACTGCAAC	15	32	0	0	0	0
VISA	ACTGGTCATGACTGCATG	188	138	2	0	0	0
VISA	ACTGCAGTGTCAACCAAC	78	98	0	0	0	0
VISA	CATGCAGTGTCAACCATG	148	104	0	0	0	2
VKORC1	GTACCATGGTACACGTTG	731	176	0	0	0	0
VKORC1	TGACCATGGTACACCAGT	116	133	0	0	0	1
VKORC1	GTACCATGGTACACCACA	64	96	1	0	0	1
VKORC1	GTTGCAGTCAACCAGTTG	204	277	0	0	0	0
VKORC1	TGTGCAGTCAACCACAGT	219	196	0	0	0	1
VLDLR	GTACACTGGTTGCAACAC	195	92	0	0	1	1
VLDLR	ACACACTGGTTGCATGGT	481	103	0	0	1	0
VLDLR	ACACACTGGTTGCAACCA	365	604	0	0	0	0
VLDLR	TGACACTGGTTGCAACTG	76	91	1	1	0	0
VLDLR	GTTGACCAGTACCAGTAC	35	43	0	0	0	6
VNN1	ACTGGTTGACCAGTTGGT	164	145	0	0	1	0
VNN1	TGTGCACACATGACGTGT	131	174	0	0	1	0
VNN1	TGTGGTTGACCAGTACTG	104	101	0	0	0	0
VNN1	TGTGGTTGACCAGTTGCA	61	58	0	0	0	0
VNN1	CATGGTTGACCAGTTGAC	39	61	0	0	0	0
VNN1	GTTGGTTGACCAGTTGTG	593	387	2	0	0	0
VNN2	ACTGGTTGACCACAGTGT	245	366	0	0	0	0
VNN2	TGTGGTTGACCACAGTCA	162	201	0	0	0	0
VNN2	CATGGTTGACCACAGTAC	101	137	1	0	0	0
VNN2	GTTGGTTGACCACAGTTG	294	305	0	0	0	0
VNN2	TGTGGTTGACCACACAGT	72	124	0	0	0	0
VNN2	ACTGCAACGTTGCACACA	48	45	0	1	0	0
VNN3	GTTGGTTGGTACTGTGTG	296	319	2	1	2	3
VNN3	ACTGGTGTCAGTACGTCA	177	144	0	0	0	23
VNN3	ACTGGTTGGTACTGTGGT	106	598	2	0	0	5
VNN3	TGTGGTTGGTACTGTGCA	143	100	0	1	0	0
VNN3	CATGGTTGGTACTGTGAC	62	47	0	0	0	0
VPREB1	TGACTGTGACTGCAGTTG	630	1139	2	0	1	0
VPREB1	ACACTGTGACTGCAGTCA	314	710	2	0	0	0

VPREB1	GTACTGTGACTGCAGTAC	8	12	0	0	0	0
VPREB1	ACACTGTGACTGCACAGT	192	337	2	0	0	0
VPREB1	TGACTGTGACTGCACACA	121	164	0	0	0	0
VPREB1	GTTGCAACCAGTGTCAAC	4	114	0	0	0	0
VSIG4	TGTGGTCACATGCAACGT	360	516	0	1	1	2
VSIG4	GTTGGTCACATGCAACCA	673	351	1	156	0	0
VSIG4	ACTGGTCACATGCAACAC	118	183	1	0	0	1
VSIG4	CATGGTCACATGCAACTG	409	251	2	0	0	8
VSIG4	GTTGGTCACATGCATGGT	271	635	2	0	0	100
VSNL1	GTACACTGGTTGACTGAC	14	38	0	0	0	0
VSNL1	TGACACTGGTTGACTGTG	376	296	1	0	0	0
VSNL1	CAACACTGGTTGTGGTGT	243	138	0	0	0	0
VSNL1	ACACACTGGTTGTGGTCA	218	274	0	0	0	0
VSNL1	GTACACTGGTTGTGGTAC	46	70	0	0	0	0
VTN	ACTGGTCAGTTGTGTGCA	210	239	41	17	41	59
VTN	ACTGGTCAGTTGTGACTG	906	934	2505	3	1	1
VTN	CATGGTCAGTTGTGTGGT	693	497	0	0	0	1
VTN	GTTGGTCAGTTGTGTGAC	20	8	0	0	0	0
VTN	TGTGGTCAGTTGTGTGTG	306	199	3	0	0	0
VWF	ACACTGTGGTGTTGGTGT	363	209	2	0	0	0
VWF	TGACTGTGGTGTTGGTCA	319	342	2	0	0	4
VWF	CAACTGTGGTGTTGGTAC	16	35	0	0	0	0
VWF	GTACTGTGGTGTTGGTTG	753	601	1	1	0	0
VWF	CATGACCATGGTTGCAGT	106	165	0	0	0	0
WARS	GTACACGTCATGGTACGT	372	830	1	0	1	0
WARS	GTACACGTCATGGTCACA	106	105	0	0	0	0
WARS	ACACACGTCATGGTCAAC	4	7	0	0	0	0
WARS	CAACACGTCATGGTCATG	464	220	0	0	0	0
WARS	CAACACGTCATGGTACCA	37	192	0	0	0	1
WARS2	CAACACGTCAACTGCATG	394	242	0	1	0	8
WARS2	GTACACGTCAACTGACGT	6	22	0	0	0	0
WARS2	CAACACGTCAACTGACCA	256	135	0	0	0	0
WARS2	TGACACGTCAACTGACAC	31	31	0	0	0	4
WARS2	TGTGCAGTCATGACGTCA	37	30	0	0	0	0
WAS	GTACCAGTTGTGGTGTGT	856	548	4	2	2	5
WAS	GTACCAGTTGACTGTGCA	460	141	1	0	0	0
WAS	ACACCAGTTGACTGTGAC	175	290	1	0	0	0
WAS	CAACCAGTTGACTGTGTG	444	564	0	0	0	0
WAS	CAACCAGTTGTGGTGTCA	335	367	0	0	0	0
WAS	ACTGCACACACACAGTCA	108	98	0	0	0	0
WASF1	GTTGGTCACAGTACACGT	338	199	0	0	1	0
WASF1	CATGGTCACAGTACACCA	383	355	0	0	0	0
WASF1	TGTGGTCACAGTACACAC	49	44	0	0	0	0
WASF1	ACTGGTCACAGTACACTG	130	112	0	0	0	3
WASF1	CATGGTCACAGTACTGGT	236	871	2	0	0	0
WASF2	ACTGGTCACACAGTACCA	177	233	0	0	0	0
WASF2	GTTGGTCACACAGTACAC	61	56	1	0	0	0
WASF2	TGTGGTCACACAGTACTG	52	49	0	0	0	0
WASF2	GTTGCACAGTGTTGGTCA	120	139	0	0	0	0
WASF2	ACTGCACAGTGTTGGTAC	42	43	0	0	0	0
WASF3	TGTGGTCACAGTACGTCA	252	231	0	0	1	0
WASF3	TGTGGTCACAGTACCAGT	211	441	0	0	1	1
WASF3	CATGGTCACAGTACGTAC	275	198	0	1	0	2
WASF3	GTTGGTCACAGTACGTTG	1318	1082	1	1	0	0
WASF3	GTTGACCAGTCAGTTGCA	331	352	0	0	0	4
WASL	CATGGTCACACAGTCACA	134	51	0	0	0	1
WASL	TGTGGTCACACAGTCAAC	136	71	0	1	0	0
WASL	ACTGGTCACACAGTCATG	405	533	0	0	0	2
WASL	CATGGTCACACAGTACGT	390	613	0	0	0	0
WASL	CATGCAGTCACATGGTAC	91	42	0	0	0	0
WBSCR17	TGTGGTACACCATGGTGT	1229	1091	6	4	3	15
WBSCR17	ACTGGTACACCAACTGAC	30	10	0	0	0	0
WBSCR17	CATGGTACACCAACTGTG	219	211	0	1	0	0
WBSCR17	GTTGGTACACCATGGTCA	116	92	0	0	0	0
WBSCR17	ACTGGTACACCATGGTAC	106	111	0	0	0	0
WBSCR17	TGTGCAACACTGGTTGGT	730	549	0	0	0	0
WBSCR22	GTTGGTCAACTGACCACA	50	109	0	0	0	0
WBSCR22	ACTGGTCAACTGACCAAC	11	14	0	0	0	0
WBSCR22	CATGGTCAACTGACCATG	65	75	0	0	0	0
WBSCR22	GTTGGTCAACTGACACGT	46	96	0	0	0	0
WBSCR22	CATGGTCAACTGACACCA	329	489	0	1	0	0
WBSCR22	GTTGCAACTGGTGTCATG	59	83	0	0	0	0
WDFY3	TGTGGTTGTGACCAGTGT	147	140	11	2	8	5
WDFY3	GTTGGTTGTGACCAGTCA	957	870	0	1	1	0
WDFY3	CATGGTTGTGACGTTGTG	396	443	1	1	0	0

WDFY3	ACTGGTTGTGACCAGTAC	20	51	0	0	0	0
WDFY3	CATGGTTGTGACCAGTTG	117	172	1	0	0	0
WDFY3	ACTGCATGGTTGACCACA	188	386	0	0	0	35
WDR1	GTTGGTTGACCACACACA	200	93	1	0	0	1
WDR1	ACTGGTTGACCACACAAC	13	16	0	0	0	0
WDR1	CATGGTTGACCACACATG	512	494	2	0	0	2
WDR1	GTTGGTTGACCACAACGT	369	346	0	0	0	0
WDR1	CATGGTTGACCACAACCA	176	164	1	0	0	0
WDR61	CATGGTACGTACTGGTAC	78	74	0	0	0	0
WDR61	GTTGGTACGTACTGGTTG	764	747	1	0	0	0
WDR61	TGTGGTACGTACTGCAGT	182	192	0	0	0	0
WDR61	GTTGGTACGTACTGCACA	132	331	0	0	0	0
WDR61	TGTGCACATGTGACGTAC	99	64	0	0	0	0
WDR61	ACTGCACATGTGACGTTG	628	746	1	3	0	1
WDR79	ACCATGCACAACGTCATG	697	667	1	634	0	2
WDR79	CACATGCACAACGTACGT	122	168	0	0	0	2
WDR79	ACCATGCACAACGTACCA	181	179	0	1	0	0
WDR79	GTCATGCACAACGTACAC	27	48	0	0	0	0
WDR79	TGCATGCACAACGTACTG	50	78	0	0	0	0
WDR79	GTTGCACATGGTCACAGT	119	156	0	0	0	0
WEE1	ACCATGACGTCAACTGGT	329	348	2	1	0	0
WEE1	TGCATGACGTCAACTGCA	165	111	0	0	0	0
WEE1	CACATGACGTCAACTGAC	7	19	0	0	0	0
WEE1	GTCATGACGTCAACTGTG	304	317	0	0	0	0
WEE1	ACCATGACGTCATGGTGT	368	240	1	1	0	0
WEE1	CATGCAACCAGTTGGTGT	507	575	0	1	0	0
WFS1	ACACTGACCACACACATG	782	419	0	0	0	79
WFS1	CAACTGACCACACAACGT	1644	1965	7	0	0	0
WFS1	ACACTGACCACACAACCA	315	397	0	0	0	0
WFS1	GTACTGACCACACAACAC	43	56	0	0	0	0
WFS1	ACTGCAGTGTCACATGGT	274	382	0	0	0	0
WFS1	CATGCACATGTGACCAGT	127	142	0	0	0	1
WIF1	GTACCACAGTGTACGTCA	29	24	5	4	5	17
WIF1	CAACCACAGTGTCATGTG	255	194	0	0	1	1
WIF1	TGACCACAGTGTACGTGT	306	314	0	0	0	0
WIF1	ACACCACAGTGTACGTAC	67	64	0	0	0	0
WIF1	CAACCACAGTGTACGTTG	187	222	1	0	0	0
WIF1	TGTGCAACCAGTGTCATG	15	28	0	0	0	0
WIPI1	TGTGGTACGTACACTGCA	39	58	0	0	0	0
WIPI1	CATGGTACGTACACTGAC	52	49	0	0	0	0
WIPI1	GTTGGTACGTACACTGTG	57	59	0	0	0	0
WIPI1	ACTGGTACGTACTGGTGT	371	656	0	0	0	0
WIPI1	TGTGGTACGTACTGGTCA	84	71	0	1	0	1
WIPI2	TGTGGTACACACCAGTGT	807	1048	1	0	3	0
WIPI2	ACTGGTACACACGTTGAC	35	137	0	0	0	0
WIPI2	CATGGTACACACGTTGTG	100	120	0	0	0	0
WIPI2	GTTGGTACACACCAGTCA	289	293	0	0	0	1
WIPI2	TGTGGTTGACCACAACAC	61	74	0	0	0	0
WISP1	GTTGCAACCAGTGTACTG	235	363	1	0	1	0
WISP1	TGACCACAGTCAGTGTAC	172	122	0	0	0	0
WISP1	ACACCACAGTCAGTGTTG	172	213	0	0	0	1
WISP1	CAACCACAGTCAGTCAGT	141	150	0	0	0	0
WISP1	ACACCACAGTCAGTCACA	107	150	0	0	0	0
WISP1	CATGCAGTACTGCACAAC	53	10	0	0	0	0
WISP2	GTACCACAGTCAGTCAAC	79	26	0	0	0	0
WISP2	TGACCACAGTCAGTCATG	81	347	0	1	0	0
WISP2	ACACCACAGTCAGTACGT	165	218	0	1	0	0
WISP2	TGACCACAGTCAGTACCA	111	120	0	0	0	0
WISP2	CAACCACAGTCAGTACAC	266	43	0	0	0	0
WISP2	TGTGCAACCAGTGTTGGT	295	413	2	0	0	0
WNK1	TGCATGCATGCAGTGTCA	212	238	1	0	0	0
WNK1	CACATGCATGCAGTGTAC	73	75	0	0	0	0
WNK1	GTCATGCATGCAGTGTTG	88	72	0	1	0	0
WNK1	TGCATGCATGCAGTCAGT	68	52	0	0	0	0
WNK1	GTTGGTTGACACACACAC	78	51	0	0	0	0
WNT1	ACACCATGCACACAACTG	526	568	3	1	2	18
WNT1	TGACCATGCAGTTGTGAC	298	27	0	0	0	0
WNT1	ACACCATGCAGTTGTGTG	343	323	0	0	0	0
WNT1	TGACCATGCACAGTGTGT	541	727	1	1	0	0
WNT1	GTACCATGCACAGTGTCA	494	317	0	1	0	1
WNT1	GTTGCAACGTACTGGTAC	30	34	0	0	0	1
WNT10B	CAACTGCATGACCAACTG	1848	2029	381	4	2	2
WNT10B	ACACTGCATGACCAACAC	95	144	0	0	0	0
WNT10B	GTACTGCATGACCATGGT	231	318	1	0	0	4
WNT10B	CAACTGCATGACCATGCA	115	157	2	0	0	0

WNT10B	TGACTGCATGACCATGAC	49	84	0	0	0	0
WNT10B	ACTGCAACGTACTGGTCA	359	599	0	4	0	1
WNT11	ACCATGTGACGTTGCATG	308	368	1	1	0	0
WNT11	CACATGTGACGTTGACGT	151	196	0	0	0	0
WNT11	ACCATGTGACGTTGACCA	38	99	0	0	0	1
WNT11	CATGGTGTCAGTTGACAC	32	82	0	0	0	0
WNT11	CATGCAGTACCAGTACGT	147	261	0	0	0	0
WNT11	GTTGCAACGTACTGACCA	119	155	0	0	0	0
WNT2	GTACCACAGTCACAGTCA	291	244	2	0	1	0
WNT2	CAACCACAGTCAGTTGTG	456	612	0	0	0	0
WNT2	TGACCACAGTCACAGTGT	1294	1789	1	2	0	2
WNT2	ACACCACAGTCACAGTAC	118	53	0	0	0	0
WNT2	CAACCACAGTCACAGTTG	366	358	0	0	0	0
WNT2	GTTGCACACACACAGTAC	101	82	1	558	0	0
WNT3	GTACCACAGTCACACAGT	434	210	0	1	2	0
WNT3	CAACCACAGTCACACACA	80	77	0	0	0	0
WNT3	TGACCACAGTCACACAAC	33	83	4	0	0	0
WNT3	ACACCACAGTCACACATG	92	173	0	0	0	0
WNT3	CAACCACAGTCACAACGT	485	1061	0	0	0	10
WNT3	TGTGCAACACCACAGTAC	41	47	0	0	0	0
WNT3A	ACTGCAGTGTGTTGTGCA	227	430	0	1	1	2
WNT3A	GTTGCAACGTACTGTGGT	546	635	0	0	1	4
WNT3A	CATGCAGTGTGTTGTGGT	500	430	0	0	0	0
WNT3A	GTTGCAGTGTGTTGTGAC	111	97	0	0	0	0
WNT3A	TGTGCAGTGTGTTGTGTG	106	130	0	1	0	0
WNT3A	CATGCAACGTACTGACTG	267	234	1	1	0	0
WNT4	GTACTGTGTGACGTGTGT	534	256	1	0	1	0
WNT4	CAACTGTGTGCATGTGAC	69	79	0	0	0	0
WNT4	GTACTGTGTGCATGTGTG	240	196	0	0	0	0
WNT4	CAACTGTGTGACGTGTCA	98	161	0	0	0	0
WNT4	ACTGACGTGTACTGGTGT	804	1022	5	0	0	0
WNT5A	CAACTGACGTCACAACCA	149	158	0	0	0	0
WNT5A	TGACTGACGTCACAACAC	21	30	0	0	0	0
WNT5A	ACACTGACGTCACAACTG	502	468	0	0	0	12
WNT5A	CAACTGACGTCACATGGT	880	979	4	1	0	0
WNT5A	GTACTGACTGACCAGTTG	893	2061	3	0	0	1
WNT5B	ACTGCAACGTTGGTTGTG	911	227	0	0	1	1
WNT5B	GTTGGTCACACATGGTGT	460	348	2	0	0	1
WNT5B	CATGGTCACACATGGTCA	180	359	1	0	0	1
WNT5B	TGTGGTCACACATGGTAC	185	177	0	0	0	0
WNT5B	ACTGGTCACACATGGTTG	196	224	0	0	0	0
WNT5B	CATGGTCACACATGCAGT	149	435	2	0	0	0
WNT6	GTACTGCATGACTGGTCA	105	158	3	0	0	0
WNT6	ACACTGCATGACTGGTAC	75	59	0	0	0	0
WNT6	CAACTGCATGACTGGTTG	465	512	0	0	0	0
WNT6	CATGCAGTTGTGGTCAAC	42	41	0	0	0	0
WNT6	GTTGCAGTTGTGGTCATG	288	387	2	0	0	1
WNT7A	TGACTGCATGACACACCA	85	125	0	0	1	0
WNT7A	CAACTGCATGACACACAC	43	207	1	0	1	0
WNT7A	ACACTGCATGACACACGT	23	2	0	0	0	0
WNT7A	GTACTGCATGACACACTG	267	219	1	0	0	0
WNT7A	ACTGCAGTTGTGGTCAGT	211	216	0	0	0	0
WNT7B	CAACGTGTCATGCAGTTG	703	130	0	0	0	0
WNT7B	ACACTGCATGCAACTGCA	628	700	0	0	0	0
WNT7B	GTACTGCATGCAACTGAC	157	299	0	0	0	0
WNT7B	TGACTGCATGCAACTGTG	18	40	0	0	0	0
WNT7B	CAACTGCATGCATGGTGT	150	127	0	0	0	0
WNT7B	GTTGCAACGTTGCAGTGT	493	878	0	0	0	0
WNT8A	CATGCAGTGTCAGTCAGT	76	118	0	0	0	0
WNT8A	ACTGCAGTGTCAGTCACA	5	5	0	1	0	0
WNT8A	GTTGCAGTGTCAGTCAAC	9	15	0	0	0	0
WNT8A	TGTGCAGTGTCAGTCATG	31	24	0	0	0	0
WNT8A	ACTGCAGTGTCAGTACGT	131	164	0	0	0	0
WNT9A	TGACTGCATGACCAACGT	185	319	1	0	1	0
WNT9A	TGACTGCATGACCACACA	257	160	0	0	0	6
WNT9A	CAACTGCATGACCACAAC	31	44	0	0	0	0
WNT9A	GTACTGCATGACCACATG	315	216	0	0	0	0
WNT9A	GTACTGCATGACCAACCA	108	183	0	0	0	0
WNT9A	GTTGCAACGTCACAACCA	199	322	1	0	0	1
WNT9B	CAACTGCATGACCAGTGT	435	504	2	0	0	0
WNT9B	ACACTGCATGACCAGTCA	330	144	0	1	0	0
WNT9B	GTACTGCATGACCAGTAC	23	42	0	0	0	0
WNT9B	TGACTGCATGACCAGTTG	834	836	1	0	0	0
WNT9B	ACACTGCATGACCACAGT	189	202	0	1	0	0
WNT9B	ACTGCAACGTACTGACAC	29	42	0	0	0	0

WRN	ACCATGTGCATGGTACTG	201	95	2	0	1	0
WRN	TGCATGTGCATGGTACAC	15	17	0	0	0	0
WRN	CACATGTGCATGGTTGGT	1031	424	1	0	0	0
WRN	ACCATGTGCATGGTTGCA	87	94	0	0	0	0
WRN	GTCATGTGCATGGTTGAC	16	54	0	0	0	0
WT1	GTCATGCATGACACACGT	12	11	0	0	0	0
WT1	CACATGCATGACACACCA	287	201	0	0	0	0
WT1	TGCATGCATGACACACAC	44	33	0	1	0	0
WT1	ACCATGCATGACACACTG	491	497	0	0	0	0
WT1	CAACCATGCATGCAACTG	231	415	2	0	0	0
WWP1	TGCATGACTGGTCAGTGT	649	197	0	1	0	1
WWP1	GTCATGACTGGTCAGTCA	177	329	0	0	0	3
WWP1	ACCATGACTGGTCAGTAC	60	53	0	0	0	0
WWP1	CACATGACTGGTCAGTTG	735	822	1	0	0	30
WWP1	CATGCAGTGTTGACTGCA	121	112	0	0	0	0
WWP2	ACCATGACGTGTACACGT	25	42	0	0	0	0
WWP2	GTTGCATGTGTGGTCAAC	27	23	0	0	0	0
WWP2	TGTGCATGTGTGGTCATG	147	131	1	1	0	0
WWP2	ACTGCATGTGTGGTACGT	704	330	0	0	0	1
WWP2	TGTGCATGTGTGGTACCA	363	358	1	3	0	7
WWTR1	GTACGTACTGTGTGACTG	810	75	0	0	0	1
WWTR1	TGACGTACTGTGTGTGGT	164	138	3	0	0	0
WWTR1	GTACGTACTGTGTGTGCA	41	159	0	1	0	5
WWTR1	ACACGTACTGTGTGTGAC	78	68	0	0	0	0
WWTR1	CAACGTACTGTGTGTGTG	388	404	0	0	0	7
XAB2	ACACTGTGGTTGGTCATG	173	236	1	0	0	0
XAB2	CAACTGTGGTTGGTACGT	168	242	0	0	0	51
XAB2	ACACTGTGGTTGGTACCA	783	481	0	0	0	2
XAB2	GTACTGTGGTTGGTACAC	19	42	0	0	0	0
XAB2	ACTGGTTGTGCACACAGT	24	56	0	0	0	0
XBP1	ACACGTTGGTCATGGTTG	695	893	2	0	0	105
XBP1	CAACGTTGGTCATGCAGT	174	220	0	0	0	0
XBP1	ACACGTTGGTCATGCACA	32	53	0	0	0	0
XBP1	GTACGTTGGTCATGCAAC	103	25	0	0	0	0
XBP1	TGACGTTGGTCATGCATG	163	116	0	0	0	0
XBP1	TGTGCAACGTGTCATGTG	391	493	1	0	0	0
XCL1	GTACTGGTCAGTTGCAAC	79	38	0	0	0	0
XCL1	TGACTGGTCAGTTGCATG	61	105	0	0	0	0
XCL1	ACACTGGTCAGTTGACGT	69	62	0	0	0	0
XCL1	TGACTGGTCAGTTGACCA	213	77	0	0	0	0
XCL1	CAACTGGTCAGTTGACAC	4	8	0	0	0	0
XCL1	GTTGCACACAGTTGGTGT	533	545	0	0	0	0
XCR1	TGACGTACGTGTGTACTG	184	101	0	0	1	0
XCR1	ACACGTACGTGTGTTGGT	735	265	0	0	0	1
XCR1	TGACGTACGTGTGTTGCA	95	43	0	0	0	0
XCR1	ACTGCAGTACACCATGAC	102	74	0	0	0	0
XCR1	TGTGACCAGTTGGTTGCA	262	46	1	0	0	1
XDH	CAACCAGTGTGTCAGTTG	631	488	2	10	0	0
XDH	GTACCAGTGTGTCACAGT	825	288	1	1	0	1
XDH	CAACCAGTGTGTCACACA	76	63	0	0	0	0
XDH	TGACCAGTGTGTCACAAC	25	59	0	0	0	0
XDH	TGTGCAGTACTGGTCAGT	112	144	0	0	0	0
XIST	CATGCATGACACTGTGCA	132	73	0	0	0	0
XIST	TGTGCATGACACTGTGAC	30	49	0	0	0	0
XIST	ACTGCATGACACTGTGTG	101	126	1	0	0	0
XIST	CATGCATGACTGGTGTGT	396	983	0	0	0	12
XIST	ACTGCATGACTGGTGTCA	122	203	0	0	0	0
XK	TGACTGCACAACTGACAC	242	132	0	1	0	0
XK	ACACTGCACAACTGACTG	396	890	2	0	0	0
XK	CAACTGCACAACTGTGGT	284	470	0	1	0	0
XK	ACACTGCACAACTGTGCA	327	308	1	0	0	1
XK	GTACTGCACAACTGTGAC	81	99	0	0	0	6
XK	GTTGCAACCAGTACGTTG	42	93	0	0	0	0
XPA	TGACTGTGTGCATGCAAC	36	42	0	0	0	0
XPA	ACACTGTGTGCATGCATG	191	119	0	0	0	0
XPA	CAACTGTGTGCATGACGT	186	150	1	0	0	0
XPA	ACACTGTGTGCATGACCA	333	185	1	0	0	0
XPA	GTACTGTGTGCATGACAC	25	23	0	0	0	0
XPA	CATGCATGCAGTACGTTG	497	341	1	0	0	0
XPC	CAACTGTGTGCACAACTG	338	460	1	0	0	0
XPC	GTACTGTGTGCACATGGT	209	921	0	1	0	1
XPC	CAACTGTGTGCACATGCA	346	111	0	0	0	0
XPC	TGACTGTGTGCACATGAC	50	36	0	0	0	0
XPC	ACACTGTGTGCACATGTG	783	268	0	0	0	0
XPO1	GTTGGTACGTCATGCAAC	11	23	0	0	0	0

XPO1	TGTGGTACGTCATGCATG	497	386	1	0	0	0
XPO1	ACTGGTACGTCATGACGT	97	142	0	0	0	0
XPO1	TGTGGTACGTCATGACCA	53	57	0	0	0	0
XPO1	CATGGTACGTCATGACAC	70	38	0	0	0	0
XRCC1	CAACGTGTACTGCAACTG	322	384	0	0	0	0
XRCC1	GTACGTGTACTGCATGGT	75	99	0	0	0	0
XRCC1	CAACGTGTACTGCATGCA	540	330	1	1	0	0
XRCC1	CATGCAGTCATGCAGTTG	550	618	3	0	0	4
XRCC1	GTTGCAGTCATGCACAGT	141	226	0	1	0	0
XRCC1	TGTGCACAACCAGTACCA	120	96	0	0	0	1
XRCC2	CATGGTTGACACGTTGCA	352	329	2	2	3	3507
XRCC2	ACACTGTGTGGTTGCACA	49	54	0	0	1	0
XRCC2	GTACTGTGTGGTTGCAAC	27	29	1	0	0	0
XRCC2	TGACTGTGTGGTTGCATG	110	186	0	0	0	0
XRCC2	ACACTGTGTGGTTGACGT	143	149	0	0	0	0
XRCC2	CATGCACAACGTGTGTGT	186	126	0	0	0	0
XRCC3	GTTGACACTGCAGTTGTG	190	248	0	0	1	0
XRCC3	CAACTGTGTGGTACTGCA	400	297	1	3	0	0
XRCC3	TGACTGTGTGGTACTGAC	33	27	0	0	0	0
XRCC3	ACACTGTGTGGTACTGTG	184	462	0	1	0	0
XRCC3	GTACTGTGTGGTTGGTGT	253	154	0	0	0	0
XRCC4	ACACCATGCATGTGTGGT	394	252	0	0	1	0
XRCC4	TGTGCACATGACACACTG	757	438	1	0	1	0
XRCC4	GTACCATGCATGTGACAC	47	42	0	0	0	0
XRCC4	TGACCATGCATGTGACTG	591	588	3443	4	0	0
XRCC4	TGACCATGCATGTGTGCA	242	186	0	1	0	0
XRCC4	CAACCATGCATGTGTGAC	76	83	0	0	0	0
XRCC5	CAACCATGCACACACATG	401	507	1	1	1	0
XRCC5	ACACCATGCACACACAAC	19	7	0	0	0	0
XRCC5	GTACCATGCACACAACGT	304	220	0	0	0	0
XRCC5	CAACCATGCACACAACCA	107	142	0	0	0	0
XRCC5	ACACCATGCACAACGTCA	157	239	0	0	0	0
XRCC5	ACTGCACATGACACTGGT	224	382	0	0	0	0
XRCC6	ACACCATGGTTGCAGTTG	2000	1112	4	0	1	17
XRCC6	ACACCATGGTTGCACACA	183	211	0	0	1	2
XRCC6	CAACCATGGTTGCACAGT	215	150	0	0	0	0
XRCC6	GTACCATGGTTGCACAAC	17	15	0	0	0	0
XRCC6	TGACCATGGTTGCACATG	167	173	0	0	0	0
XYLT1	ACACCACAACGTACACTG	485	573	0	0	0	0
XYLT1	CAACCACAACGTACTGGT	174	143	0	0	0	0
XYLT1	ACACCACAACGTACTGCA	160	269	1	0	0	0
XYLT1	GTACCACAACGTACTGAC	63	168	0	1	0	0
XYLT1	CATGCAGTACTGCATGCA	522	82	0	0	0	1
XYLT2	GTACCACAACGTTGCATG	102	151	0	0	0	0
XYLT2	TGACCACAACGTTGACGT	258	210	0	0	0	1
XYLT2	GTACCACAACGTTGACCA	362	131	0	0	0	3
XYLT2	ACACCACAACGTTGACAC	29	63	0	0	0	0
XYLT2	CAACCACAACGTTGACTG	272	117	0	0	0	0
YAP1	ACTGGTGTCATGCACACA	70	52	3	7	5	16885
YAP1	ACTGGTGTCATGCAGTTG	183	243	2	1654	1	3
YAP1	CATGGTGTCATGCACAGT	212	185	0	0	1	0
YAP1	TGTGGTGTCAGTACCACA	92	172	0	0	0	2
YAP1	GTTGGTGTCATGCACAAC	16	51	0	0	0	0
YAP1	GTTGCAACCATGGTTGAC	46	18	0	0	0	0
YARS	GTACACGTCATGACGTGT	264	226	0	0	1	0
YARS	GTACACGTCATGCATGGT	747	563	0	1	0	1
YARS	CAACACGTCATGCATGCA	224	356	1	1	0	0
YARS	TGACACGTCATGCATGAC	25	22	0	0	0	0
YARS	ACACACGTCATGCATGTG	230	632	1	1	0	0
YARS2	GTTGGTCATGTGTGACTG	504	590	0	1	0	0
YARS2	TGTGGTCATGTGTGTGGT	477	830	0	0	0	1
YARS2	GTTGGTCATGTGTGTGCA	118	138	0	0	0	0
YARS2	ACTGGTCATGTGTGTGAC	76	26	0	0	0	0
YARS2	CATGACCATGACACTGGT	144	187	0	0	0	0
YES1	TGCATGACGTGTTGTGCA	110	155	0	0	1	0
YES1	GTCATGACGTGTTGACAC	26	20	0	0	0	0
YES1	TGCATGACGTGTTGACTG	392	349	0	0	0	0
YES1	ACCATGACGTGTTGTGGT	394	274	1	1	0	4
YES1	CACATGACGTGTTGTGAC	11	22	0	0	0	0
YWHAB	TGACTGTGCAACACTGAC	44	77	0	0	0	5
YWHAB	ACACTGTGCAACACTGTG	650	590	0	0	0	0
YWHAB	GTACTGTGCAACTGGTGT	332	587	2	1	0	0
YWHAB	CAACTGTGCAACTGGTCA	785	812	4	0	0	0
YWHAB	TGACTGTGCAACTGGTAC	69	68	0	0	0	0
YWHAB	GTTGCATGCAGTTGACGT	142	241	1	0	0	0

YWHAG	ACACTGTGCAACTGGTTG	527	742	0	1	0	0
YWHAG	CAACTGTGCAACTGCAGT	120	118	0	0	0	0
YWHAG	ACACTGTGCAACTGCACA	111	122	0	0	0	0
YWHAG	GTACTGTGCAACTGCAAC	2	15	0	0	0	0
YWHAG	TGTGCAACACGTTGGTGT	463	473	1	0	0	1
YWHAG	GTTGCAACACGTTGGTCA	137	180	0	0	0	0
YWHAH	CAACTGTGCACAACCACA	248	310	0	0	0	0
YWHAH	TGACTGTGCAACTGCATG	300	370	0	0	0	0
YWHAH	ACACTGTGCAACTGACGT	950	726	1	0	0	0
YWHAH	TGACTGTGCAACTGACCA	383	285	0	0	0	0
YWHAH	CAACTGTGCAACTGACAC	104	101	0	0	0	0
YWHAQ	GTACTGTGCAACTGACTG	83	159	0	0	0	0
YWHAQ	TGACTGTGCAACTGTGGT	338	317	2	0	0	0
YWHAQ	GTACTGTGCAACTGTGCA	525	387	2	0	0	0
YWHAQ	ACACTGTGCAACTGTGAC	50	42	0	0	0	0
YWHAQ	CAACTGTGCAACTGTGTG	288	364	0	0	0	0
YWHAQ	ACTGCAACACGTTGGTAC	97	112	0	0	0	0
YWHAZ	GTACCAGTACTGCACAAC	18	16	0	0	0	0
YWHAZ	TGACCAGTACTGCACATG	248	119	1	0	0	0
YWHAZ	ACACCAGTACTGCAACGT	238	259	0	0	0	0
YWHAZ	TGACCAGTACTGCAACCA	485	365	4	2745	0	0
YWHAZ	CAACTGACTGACGTCACA	354	150	0	0	0	0
YWHAZ	TGTGCAACACACGTGTGT	393	613	4	2	0	2
YY1	CATGCAACACGTTGGTTG	542	399	1	1	1	0
YY1	GTACGTTGGTACGTTGGT	167	128	3	1	0	0
YY1	CAACGTTGGTACGTTGCA	47	95	0	0	0	0
YY1	TGACGTTGGTACGTTGAC	191	108	0	0	0	0
YY1	ACACGTTGGTACGTTGTG	170	182	0	0	0	0
YY1	GTACGTTGGTACCAGTGT	517	413	1	0	0	0
ZAP70	TGCATGCACATGCAACTG	524	356	0	1	1	0
ZAP70	GTCATGCACATGCAACAC	43	22	0	0	0	0
ZAP70	ACCATGCACATGCATGGT	62	107	0	0	0	0
ZAP70	TGTGGTACTGACACACGT	39	51	0	0	0	0
ZAP70	GTTGGTACTGACACACCA	188	155	0	0	0	0
ZBTB16	GTACGTCAACCATGCATG	191	182	0	0	1	1
ZBTB16	CAACGTCAACCATGCAAC	59	50	0	0	0	0
ZBTB16	TGACGTCAACCATGACGT	128	172	0	0	0	1
ZBTB16	GTACGTCAACCATGACCA	24	17	0	0	0	0
ZBTB16	ACACGTCAACCATGACAC	71	75	0	0	0	0
ZBTB20	CAACGTACTGTGCACATG	207	158	0	1	0	0
ZBTB20	GTACGTACTGTGCAACGT	504	611	0	0	0	1
ZBTB20	CAACGTACTGTGCAACCA	136	165	0	0	0	0
ZBTB20	TGACGTACTGTGCAACAC	45	41	0	0	0	0
ZBTB20	ACACGTACTGTGCAACTG	521	153	0	1	0	0
ZBTB32	ACACGTACCACAGTCATG	71	79	2	0	0	0
ZBTB32	CAACGTACCACAGTACGT	60	64	0	0	0	0
ZBTB32	ACACGTACCACAGTACCA	333	299	0	0	0	0
ZBTB32	GTACGTACCACAGTACAC	51	69	4	0	0	0
ZBTB32	TGACGTACCACAGTACTG	53	115	0	0	0	0
ZBTB32	TGTGCACAACTGGTTGCA	812	487	3	0	0	3
ZBTB7A	CATGGTCACAACCAACAC	51	61	0	0	0	0
ZBTB7A	GTTGGTCACAACCAACTG	262	592	0	1	0	0
ZBTB7A	TGTGGTCACAACCATGGT	151	223	0	0	0	0
ZBTB7A	GTTGGTCACAACCATGCA	372	381	1	2	0	0
ZBTB7A	ACTGGTCACAACCATGAC	25	22	0	0	0	0
ZCCHC11	GTTGGTACGTCAGTACAC	5	2	0	0	0	0
ZCCHC11	TGTGGTACGTCAGTACTG	155	41	0	0	0	1
ZCCHC11	ACTGGTACGTCAGTTGGT	93	48	0	0	0	0
ZCCHC11	TGTGGTACGTCAGTTGCA	122	100	0	0	0	0
ZCCHC11	CATGGTACGTCAGTTGAC	48	37	0	1	0	0
ZDHHC8	ACTGGTTGACCACAACTG	1053	1427	1	0	0	2
ZDHHC8	CATGGTTGACCACATGGT	235	279	0	0	0	0
ZDHHC8	ACTGGTTGACCACATGCA	825	820	1	0	0	0
ZDHHC8	GTTGGTTGACCACATGAC	124	108	1	0	0	0
ZDHHC8	TGTGGTTGACCACATGTG	249	153	0	0	0	0
ZEB2	CAACTGACTGACGTGTTG	668	605	0	1	1	0
ZEB2	ACACGTCAACTGACACAC	48	79	0	0	0	0
ZEB2	CAACGTCAACTGACACTG	278	339	1	0	0	0
ZEB2	GTACGTCAACTGACTGGT	235	297	0	0	0	0
ZEB2	CAACGTCAACTGACTGCA	424	542	0	1	0	0
ZFP36	ACCATGTGACACCAGTGT	1301	1171	1	9	1	1
ZFP36	TGCATGTGACACGTTGCA	386	116	1	0	0	0
ZFP36	CACATGTGACACGTTGAC	62	38	0	0	0	0
ZFP36	GTCATGTGACACGTTGTG	311	292	0	0	0	0
ZFP36	TGCATGTGACACCAGTCA	86	121	0	0	0	0

ZFP42	ACTGGTGTCATGTGGTGT	831	757	0	0	1	1
ZFP42	GTTGGTGTCATGACTGTG	218	215	0	1	0	4
ZFP42	TGTGGTGTCATGTGGTCA	151	348	0	0	0	0
ZFP42	CATGGTGTCATGTGGTAC	47	49	0	0	0	0
ZFP42	CATGACCATGGTTGGTCA	111	175	0	0	0	0
ZFX	GTACGTACACCAACGTCA	392	175	0	0	1	0
ZFX	GTACGTACACCACATGCA	124	115	0	0	0	0
ZFX	ACACGTACACCACATGAC	96	83	0	0	0	0
ZFX	CAACGTACACCACATGTG	128	182	0	0	0	0
ZFX	TGACGTACACCAACGTGT	54	273	0	0	0	0
ZFYVE16	ACACACTGACTGGTTGGT	494	532	0	0	0	1
ZFYVE16	TGACACTGACTGGTTGCA	247	162	0	0	0	0
ZFYVE16	CAACACTGACTGGTTGAC	36	30	0	0	0	0
ZFYVE16	GTACACTGACTGGTTGTG	441	598	3	0	0	0
ZFYVE16	ACACACTGACTGCAGTGT	331	289	0	0	0	0
ZFYVE9	ACACACTGACTGGTGTAC	104	162	0	0	0	0
ZFYVE9	CAACACTGACTGGTGTTG	117	183	1	0	0	0
ZFYVE9	GTACACTGACTGGTCAGT	644	361	0	1	0	0
ZFYVE9	CAACACTGACTGGTCACA	103	108	0	0	0	0
ZFYVE9	TGACACTGACTGGTCAAC	18	37	0	0	0	0
ZIC1	ACTGGTGTACGTGTGTCA	107	105	0	0	1	0
ZIC1	CAACTGTGGTACACACAC	42	21	0	0	0	5
ZIC1	GTTGGTGTACGTGTGTAC	105	90	0	1	0	0
ZIC1	TGTGGTGTACGTGTGTTG	742	396	1	1	0	0
ZIC1	ACTGCACAGTGTCACAGT	210	317	1	0	0	0
ZMAT3	CATGGTCAACCACACAGT	887	1159	1	1	1	1
ZMAT3	GTTGGTCAACCACAGTGT	229	296	0	0	0	0
ZMAT3	CATGGTCAACCACAGTCA	163	243	0	0	0	0
ZMAT3	TGTGGTCAACCACAGTAC	22	37	0	0	0	0
ZMAT3	ACTGGTCAACCACAGTTG	257	348	0	1	0	0
ZMAT3	CATGCAACTGGTGTCAAC	38	19	0	0	0	0
ZMYM2	GTACTGACTGACGTACAC	49	51	0	0	0	0
ZMYM2	TGACTGACTGACGTACTG	305	155	0	0	0	0
ZMYM2	ACACTGACTGACGTTGGT	170	166	0	1	0	0
ZMYM2	TGTGGTCAGTACACGTCA	13	13	0	0	0	0
ZMYM2	CATGGTCAGTACACGTAC	14	13	0	1	0	0
ZMYND17	CATGGTTGACCAGTGTTG	892	910	0	1	1	0
ZMYND17	GTTGGTTGTGCAGTACGT	140	600	0	0	1	0
ZMYND17	CATGGTTGTGCAGTACCA	425	420	1	0	0	4
ZMYND17	TGTGGTTGTGCAGTACAC	15	36	0	0	0	0
ZMYND17	ACTGGTTGTGCAGTACTG	130	314	0	1	0	0
ZNF259	CATGGTTGACCAACGTGT	551	420	2	0	0	25
ZNF259	ACTGGTTGACCAACGTCA	150	139	0	0	0	3
ZNF259	GTTGGTTGACCAACGTAC	0	3	0	0	0	0
ZNF259	TGTGGTTGACCAACGTTG	1343	914	6	3	0	43
ZNF259	ACTGGTTGACCAACCAGT	87	113	0	0	0	0
ZNF395	TGTGGTTGGTACCAGTTG	745	959	2	0	2	0
ZNF395	CATGGTTGGTACCAGTGT	1695	1583	0	2	0	1
ZNF395	ACTGGTTGGTACCAGTCA	237	261	0	1	0	0
ZNF395	GTTGGTTGGTACCAGTAC	138	147	0	0	0	0
ZNF395	ACTGGTTGGTACCACAGT	75	98	0	0	0	0
