# Supplementary material for: Functional screening identifies aryl hydrocarbon receptor as suppressor of lung cancer metastasis
Source: Oncogenesis. 2020 Nov 19;9(11):102. doi: 10.1038/s41389-020-00286-8 (PMC7677369; doi:10.1038/s41389-020-00286-8)
Supplement: Supplementary file 6 — Legends to Supplementary Figures and Tables [file 41389_2020_286_MOESM6_ESM.docx]

**Supplementary information**

***Functional screening identifies aryl hydrocarbon receptor as suppressor of lung cancer metastasis***

*Nothdurft et al.*

**Supplementary Table 1 | Raw counts from barcode sequencing to identify candidate metastasis suppressors in a functional *in vivo* shRNA screen.**

Displayed are genes targeted by shRNAs in the DECIPHER shRNA Library Human Modul 1 (Cellecta Inc, column A), barcode sequences to identify the shRNAs in A (column B) and the raw read counts for the barcode representation at time point t= 0 prior to injection of shRNA-library transduced H1975 cells into mice (column C and D). Additionally, raw read counts are presented for barcodes identified in two primary tumors induced by shRNA-library transduced H1975 cells (columns E, F) and two metastatic tumors from the same mice (columns G, H). *See separate excel file.*

**Supplementary Table 2 | List of shRNAs and siRNAs used for RNA interference.** List of RNAs and their sequences. All RNAs were purchased form Sigma-Aldrich.

| **Type** | **Gene target** | **Sequence (5‘ – 3‘)** |
| --- | --- | --- |
| shRNA | Non-targeting | CCGGCAACAAGATGAAGAGCACCAACTCGAGTTGGTGC  TCTTCATCTTGTTGTTTTT |
| shRNA | *AHR* | CCGGCGGCATAGAGACCGACTTAATCTCGAGATTAAGTCG  GTCTCTATGCCGTTTTT |
| siRNA | Negative control | n/a |
| siRNA | *ATF4* | n/a, heterogeneous mixture of siRNAs |

**Supplementary Table 3 | List of human primers for SYBR Green-based qRT-PCR.** List of primers and their sequences. If not stated otherwise, oligonucleotides were purchased from IDT. For ATF4, QuantiTect® Primer Assay (#QT00074466) was purchased from Qiagen.

| **Gene** | **Sequence (5‘ – 3‘)** | **Comment** |
| --- | --- | --- |
| *ACTB* | For - GGATTCCTATGTGGGCG |  |
|  | Rev - GGCGTACAGGGATAGC |  |
| *AHR* | For - TACCGAAGACCGAGCTGAAT |  |
|  | Rev - GGAGACCAGTGGCTTCTTCA |  |
| *ASNS* | For - TCACTTCCAATATGATCTGCCA | IDT RTU mix |
|  | Rev - AGTACAGTATCCTCTCCAGACA |  |
| *ATF4* | For - n/a | Qiagen QuantiTect® Primer Assay |
|  | Rev - n/a |  |
| *CYP1A1* | For - CCCAGCTCAGCTCAGTACCT |  |
|  | Rev - AGGCCCTGATTACCCAGAAT |  |
| *GAPDH* | For - ATTGCCCTCAACGACCACT |  |
|  | Rev - TCTTCCTCTTGTGCTCTTGCT |  |
| *HPRT1* | For - GCGATGTCAATAGGACTCCAG | IDT RTU mix |
|  | Rev - TTGTTGTAGGATATGCCCTTGA |  |
| *MMP9* | For - GCACGACGTCTTCCAGTACC | Safranek et al. 2009 [1] |
|  | Rev - CAGGATGTCATAGGTCACGTAGC |  |
| *MMP24* | For - GGGGCGAGATGTTTGTCTTT |  |
|  | Rev - TCCCATCGGCCCTTTCATAG |  |

Supplementary Figure 1 | shRNA-mediated suppression of endogenous *AHR* alters metastatic potential of H1975 lung cancer cells. (a) Kaplan-Meier plot displaying overall survival (OS) of patients with stage I lung adenocarcinomas stratified by high or low *AHR* expression (20820_at) [2], when “high AHR” and “low AHR” depict that expression was higher or lower than the median, respectively. (b) AHR protein expression in H1975 shScr control and shAHR cell clones (K1-K3) was assessed by immunoblotting. (c) *AHR* mRNA levels were analysed by qRT-PCR. Target gene expression was normalised to two (*ACTB*, *GAPDH*) house keeping genes (HKG) relative to the shScr control. (d) Cell proliferation was analysed using MTT assay after 72 h. Data are shown as mean ± SD relative to the shScr control. n = 3 for all experiments. Significance was assessed using one-way ANOVA. (e) Representative images of explanted lung lobes from primary H1975 tumours and metastases with (shAHR-K2) and without (shScr) knockdown of *AHR* as visualised by *ex vivo* bioluminescent imaging.

**Supplementary Figure 2 | Reconstitution of *AHR* expression in H1975 with *AHR* knockdown partially rescued the metastatic phenotype observed *in vitro*. (a)** Retroviral vector encoding a shRNA-resistant *AHR* (CDS-AHR, 5 silent point mutations) cDNA or empty vector (EV) control was transduced into three independent clones of H1975 expressing shAHR (K1-K3) and H1975 shScr control cells (shScr). **(b)** qRT-PCR analysis of *AHR* expression. (**c)** Quantification of a combined migration / invasion assay evaluating invasive capacity of cells with reconstituted *AHR* expression (AHR) compared to controls (EV) and **(d)** representative images thereof. Scale bar, 200 µm. Relative invasion and migration was calculated by dividing the mean number of invading cells by the mean number of migrating cells. **(e)** Numbers of migrating and invading cells for H1975 shAHR clones and shScr controls with reconstituted *AHR* expression (AHR) compared to empty vector control (EV). n = 3 for experiments shown in (c) and (e). Data are shown as mean ± SD. Significance was assessed using one-way ANOVA.

**Supplementary Figure 3 | RNA sequencing analysis of AHR-regulated constitutive and ligand-activated transcription patterns. (a)** AHR activator-mediated induction of target gene expression is attenuated in H1975 shAHR cells. Left panel: Target gene expression of three independent clones of H1975 expressing shAHR (K1-K3) and control cells (shScr) was analysed after 24 h of treatment with kynurenic acid (KynA, 100 µM), 6-formylindolo[3,2b]carbazole (FICZ, 200 nM), biochanin A (BioA, 20 µM) or DMSO by qRT-PCR. Target gene expression (CYP1A1) was normalised to GAPDH. Data are shown as mean±SD, n=2. Right panel: qRT-PCR analysis of *CYP1A1* expression in the presence or absence of omeprazole (omep, 200 µM for 24 and 48 h, respectively). Expression was normalised to *GAPDH*. Data are shown as mean ± SD normalised to control. **(b)** Principal component analysis recovered clustering of experimental groups: H1975 cells with (shAHR-K2) and without knockdown of *AHR* (shScr), treated (+) or untreated (-) with omeprazole (omep, 200 µM) for 48 hours. **(c)** Proliferation and metabolic viability of H1975 expressing shAHR (K2) and H1975 shScr control cells upon omeprazole treatment (100-200 µM, 48 h) was studied using MTT assay. n = 3. Data are shown as mean ± SD normalised to respective DMSO control. For (b) and (c) significance was assessed using one-way ANOVA. **(d)** Gene set enrichment analysis (GSEA) revealed gene sets differentially represented in H1975 shScr omep compared to H1975 shScr DMSO. **(e)**Gene sets differentially represented in H1975 shAHR-K2 DMSO compared to H1975 shScr DMSO. n = 3. **(f)** GSEA plot indicating genes annotated with ‘TGF-beta signalling’ were enriched in H1975 cells with *AHR* knockdown compared to the H1975 shScr control cells. **(g)**Protein expression of regulators of EMT in H1975 cell clones expressing shAHR (K1-K3) and shShr control after treatment with TGF-β1 (10 ng/µl, 48 h) as compared to non-treated controls. n=3. **(h)** Combined migration / invasion assay evaluating invasive capacity of H1975 cells with *AHR* knockdown and H1975 shScr control cells after TGF-β1 treatment (10 ng/µl, 48 h) as compared to non-treated controls. n=4. Data are shown as mean ± SD normalised to control and significance was assessed using one-way ANOVA. **(i)** Representative images of migrated / invaded cells. Scale bar, 200 µm.

**Supplementary Figure 4 | A*HR* activation and modulation of *AHR* expression in H1975 cells impacts target gene expression and activity in a time- and dose dependent manner. (a)** AHR activation upregulates ATF4 expression in a luciferase reporter system, in which luciferase expression is under the control of the human ATF4 promoter. H1975 cells with (K1-K3) and without AHR k.o. were treated with omeprazole for 48 h. Luciferase activity was quantified and normalised to control treated cells, n=3. **(b-d)** Kinetics of expression of AHR target genes, ATF4, MMP24 and ASNS. H1975 cells with (K1-K2) and without AHR ko were treated for indicated time points with omeprazole (100 or 200 µM). Significance of target gene regulation was calculated by two-way ANOVA. **(e)** Invasion of H1975 cells with (shAHR-K2) or without AHR-knock-down (shScr) was assessed in the presence of absence of an the broad-range MMP inhibitor, BB94. Significance was calculated by one-way ANOVA. **(f)** Semiquantitative analysis of MMP9 activity in gelatinolytic assays upon expression of an shRNA-resistant AHR-cDNA (“AHR”) in H1975 cells without (shScr) or with shRNA-mediated knock-down of AHR (shAHR K2) using ImageJ. EV= empty vector control. **(g)** Gelatine zymography of MMP2 and MMP9 activity for results summarized in (f). n = 3. Data are shown as mean ± SD. Significance was assessed using one-way ANOVA.

Supplementary References

[1] Safranek J, Pesta M, Holubec L, Kulda V, Dreslerova J, Vrzalova J*, et al.* Expression of MMP-7, MMP-9, TIMP-1 and TIMP-2 mRNA in lung tissue of patients with non-small cell lung cancer (NSCLC) and benign pulmonary disease. Anticancer Res 2009; 29(7): 2513–2517.

[2] Győrffy B, Surowiak P, Budczies J, Lánczky A. Online survival analysis software to assess the prognostic value of biomarkers using transcriptomic data in non-small-cell lung cancer. PLoS ONE 2013; 8(12): e82241.
