# Supplementary material for: Functional screening identifies aryl hydrocarbon receptor as suppressor of lung cancer metastasis
Source: Oncogenesis. 2020 Nov 19;9(11):102. doi: 10.1038/s41389-020-00286-8 (PMC7677369; doi:10.1038/s41389-020-00286-8)
Supplement: Supplementary file 7 — Author list changes approval [file 41389_2020_286_MOESM7_ESM.pdf]

Innere Klinik (Tumorforschung)
Direktor: Univ.-Prof. Dr. M. Schuler

Tel. +49 (0) 201 723 20 00
Fax +49 (0) 201 723 59 24
martin.schuler@uk-essen.de

10. Juli 2020

Universitätsmedizin Essen
Westdeutsches Tumorzentrum Essen

Universitätsklinikum Essen | Hufelandstraße 55 | 45147 Essen

Editorial Office
Oncogenesis

westdeutsches tumorzentrum

Prof. Dr. Alexander Schramm
Molekulare Onkologie
Innere Klinik (Tumorforschung)
Universitätsklinikum Essen
Hufelandstraße 55
45147 Essen

Tel. +49 (0) 201 723 1630
Fax +49 (0) 201 723 5616
alexander.schramm@uk-essen.de
www.uk-essen.de

Confirmation of changed author list for ONCSIS-20-0064-R, "Functional screening identifies aryl hydrocarbon receptor as suppressor of lung metastasis" by Silke Nothdurft et al.

Dear Editorial Office Team,

please find attached the collection of pdfs signed by all authors approving the changed authors list of the above referenced manuscript.

Kind regards,

Prof. Dr. Alexander Schramm

SPRINGER NATURE

In accordance with Springer Nature Authorship Policy we agree to change the authors of the manuscript as indicated below.

NAME OF JOURNAL: Oncogenesis

TITLE OF MANUSCRIPT: Functional screening identifies aryl hydrocarbon receptor as suppressor of lung cancer metastasis

MANUSCRIPT NUMBER: ONCSIS-20-0064-R

CORRESPONDING AUTHORS NAME: Alexander Schramm

PREVIOUS AUTHOR NAMES:

Silke Nothdurft, Frank Breitenbücher, Ross A. Okimoto, Madeleine Dorsch, Christiane A. Opitz, Ahmed Sadik, Charlotte Esser, Michael Hölzel, Saurabh Asthana, Jan Forster, Sophie Kalmbach, Barbara M. Grüner, Trever G. Bivona, Alexander Schramm, Martin Schuler

UPDATED AUTHOR NAMES:

Silke Nothdurft, Clotilde Thumser-Henner, Frank Breitenbücher, Ross A. Okimoto, Madeleine Dorsch, Christiane A. Opitz, Ahmed Sadik, Charlotte Esser, Michael Hölzel, Saurabh Asthana, Jan Forster, Daniela Beisser, Sophie Kalmbach, Barbara M. Grüner, Trever G. Bivona, Alexander Schramm, Martin Schuler

CHANGE TO AUTHOR LIST:

Clotilde Thumser-Henner, Daniela Beisser

Print Name	Signature	Date
**Charlotte Esser**	[signature]	7.7.2020
Ahmed Sadik	Ahmed Sadik	9.7.2020

SPRINGER NATURE

In accordance with Springer Nature Authorship Policy we agree to change the authors of the manuscript as indicated below.

NAME OF JOURNAL: Oncogenesis

TITLE OF MANUSCRIPT: Functional screening identifies aryl hydrocarbon receptor as suppressor of lung cancer metastasis

MANUSCRIPT NUMBER: ONCSIS-20-0064-R

CORRESPONDING AUTHORS NAME: Alexander Schramm

PREVIOUS AUTHOR NAMES:

Silke Nothdurft, Frank Breitenbücher, Ross A. Okimoto, Madeleine Dorsch, Christiane A. Opitz, Ahmed Sadik, Charlotte Esser, Michael Hölzel, Saurabh Asthana, Jan Forster, Sophie Kalmbach, Barbara M. Grüner, Trever G. Bivona, Alexander Schramm, Martin Schuler

UPDATED AUTHOR NAMES:

Silke Nothdurft, Clotilde Thumser-Henner, Frank Breitenbücher, Ross A. Okimoto, Madeleine Dorsch, Christiane A. Opitz, Ahmed Sadik, Charlotte Esser, Michael Hölzel, Saurabh Asthana, Jan Forster, Daniela Beisser, Sophie Kalmbach, Barbara M. Grüner, Trever G. Bivona, Alexander Schramm, Martin Schuler

CHANGE TO AUTHOR LIST:

Clotilde Thumser-Henner, Daniela Beisser

Print Name	Signature	Date
Ross A. Okimoto	[illegible]	7/8/2020

SPRINGER NATURE

In accordance with Springer Nature Authorship Policy we agree to change the authors of the manuscript as indicated below.

NAME OF JOURNAL: Oncogenesis

TITLE OF MANUSCRIPT: Functional screening identifies aryl hydrocarbon receptor as suppressor of lung cancer metastasis

MANUSCRIPT NUMBER: ONCSIS-20-0064-R

CORRESPONDING AUTHORS NAME: Alexander Schramm

PREVIOUS AUTHOR NAMES:

Silke Nothdurft, Frank Breitenbücher, Ross A. Okimoto, Madeleine Dorsch, Christiane A. Opitz, Ahmed Sadik, Charlotte Esser, Michael Hölzel, Saurabh Asthana, Jan Forster, Sophie Kalmbach, Barbara M. Grüner, Trever G. Bivona, Alexander Schramm, Martin Schuler

UPDATED AUTHOR NAMES:

Silke Nothdurft, Clotilde Thumser-Henner, Frank Breitenbücher, Ross A. Okimoto, Madeleine Dorsch, Christiane A. Opitz, Ahmed Sadik, Charlotte Esser, Michael Hölzel, Saurabh Asthana, Jan Forster, Daniela Beisser, Sophie Kalmbach, Barbara M. Grüner, Trever G. Bivona, Alexander Schramm, Martin Schuler

CHANGE TO AUTHOR LIST:

Clotilde Thumser-Henner, Daniela Beisser

Print Name	Signature	Date
Clotilde Thumser-Henner	CTH	10.07.2020

SPRINGER NATURE

In accordance with Springer Nature Authorship Policy we agree to change the authors of the manuscript as indicated below.

NAME OF JOURNAL: Oncogenesis

TITLE OF MANUSCRIPT: Functional screening identifies aryl hydrocarbon receptor as suppressor of lung cancer metastasis

MANUSCRIPT NUMBER: ONCSIS-20-0064-R

CORRESPONDING AUTHORS NAME: Alexander Schramm

PREVIOUS AUTHOR NAMES:

Silke Nothdurft, Frank Breitenbücher, Ross A. Okimoto, Madeleine Dorsch, Christiane A. Opitz, Ahmed Sadik, Charlotte Esser, Michael Hölzel, Saurabh Asthana, Jan Forster, Sophie Kalmbach, Barbara M. Grüner, Trever G. Bivona, Alexander Schramm, Martin Schuler

UPDATED AUTHOR NAMES:

Silke Nothdurft, Clotilde Thumser-Henner, Frank Breitenbücher, Ross A. Okimoto, Madeleine Dorsch, Christiane A. Opitz, Ahmed Sadik, Charlotte Esser, Michael Hölzel, Saurabh Asthana, Jan Forster, Daniela Beisser, Sophie Kalmbach, Barbara M. Grüner, Trever G. Bivona, Alexander Schramm, Martin Schuler

CHANGE TO AUTHOR LIST:

Clotilde Thumser-Henner, Daniela Beisser

Print Name	Signature	Date
Alexander Schramm	A.S.	07/10/20
and on behalf of former co-worker Dr. Daniela Beisser	A.S.	07/10/20

SPRINGER NATURE

In accordance with Springer Nature Authorship Policy we agree to change the authors of the manuscript as indicated below.

NAME OF JOURNAL: Oncogenesis

TITLE OF MANUSCRIPT: Functional screening identifies aryl hydrocarbon receptor as suppressor of lung cancer metastasis

MANUSCRIPT NUMBER: ONCSIS-20-0064-R

CORRESPONDING AUTHORS NAME: Alexander Schramm

PREVIOUS AUTHOR NAMES:

Silke Nothdurft, Frank Breitenbücher, Ross A. Okimoto, Madeleine Dorsch, Christiane A. Opitz, Ahmed Sadik, Charlotte Esser, Michael Hölzel, Saurabh Asthana, Jan Forster, Sophie Kalmbach, Barbara M. Grüner, Trever G. Bivona, Alexander Schramm, Martin Schuler

UPDATED AUTHOR NAMES:

Silke Nothdurft, Clotilde Thumser-Henner, Frank Breitenbücher, Ross A. Okimoto, Madeleine Dorsch, Christiane A. Opitz, Ahmed Sadik, Charlotte Esser, Michael Hölzel, Saurabh Asthana, Jan Forster, Daniela Beisser, Sophie Kalmbach, Barbara M. Grüner, Trever G. Bivona, Alexander Schramm, Martin Schuler

CHANGE TO AUTHOR LIST:

Clotilde Thumser-Henner, Daniela Beisser

Print Name	Signature	Date
Dorsch, Madeleine	[signature]	9.07.20

SPRINGER NATURE

In accordance with Springer Nature Authorship Policy we agree to change the authors of the manuscript as indicated below.

NAME OF JOURNAL: Oncogenesis

TITLE OF MANUSCRIPT: Functional screening identifies aryl hydrocarbon receptor as suppressor of lung cancer metastasis

MANUSCRIPT NUMBER: ONCSIS-20-0064-R

CORRESPONDING AUTHORS NAME: Alexander Schramm

PREVIOUS AUTHOR NAMES:

Silke Nothdurft, Frank Breitenbücher, Ross A. Okimoto, Madeleine Dorsch, Christiane A. Opitz, Ahmed Sadik, Charlotte Esser, Michael Hölzel, Saurabh Asthana, Jan Forster, Sophie Kalmbach, Barbara M. Grüner, Trever G. Bivona, Alexander Schramm, Martin Schuler

UPDATED AUTHOR NAMES:

Silke Nothdurft, Clotilde Thumser-Henner, Frank Breitenbücher, Ross A. Okimoto, Madeleine Dorsch, Christiane A. Opitz, Ahmed Sadik, Charlotte Esser, Michael Hölzel, Saurabh Asthana, Jan Forster, Daniela Beisser, Sophie Kalmbach, Barbara M. Grüner, Trever G. Bivona, Alexander Schramm, Martin Schuler

CHANGE TO AUTHOR LIST:

Clotilde Thumser-Henner, Daniela Beisser

Print Name	Signature	Date
Silke Nothdurft	S. Nothdurft	08.07.2020

SPRINGER NATURE

In accordance with Springer Nature Authorship Policy we agree to change the authors of the manuscript as indicated below.

NAME OF JOURNAL: Oncogenesis

TITLE OF MANUSCRIPT: Functional screening identifies aryl hydrocarbon receptor as suppressor of lung cancer metastasis

MANUSCRIPT NUMBER: ONCSIS-20-0064-R

CORRESPONDING AUTHORS NAME: Alexander Schramm

PREVIOUS AUTHOR NAMES:

Silke Nothdurft, Frank Breitenbücher, Ross A. Okimoto, Madeleine Dorsch, Christiane A. Opitz, Ahmed Sadik, Charlotte Esser, Michael Hölzel, Saurabh Asthana, Jan Forster, Sophie Kalmbach, Barbara M. Grüner, Trever G. Bivona, Alexander Schramm, Martin Schuler

UPDATED AUTHOR NAMES:

Silke Nothdurft, Clotilde Thumser-Henner, Frank Breitenbücher, Ross A. Okimoto, Madeleine Dorsch, Christiane A. Opitz, Ahmed Sadik, Charlotte Esser, Michael Hölzel, Saurabh Asthana, Jan Forster, Daniela Beisser, Sophie Kalmbach, Barbara M. Grüner, Trever G. Bivona, Alexander Schramm, Martin Schuler

CHANGE TO AUTHOR LIST:

Clotilde Thumser-Henner, Daniela Beisser

Print Name	Signature	Date
Barbara M. Grüner		08.07.2020

SPRINGER NATURE

In accordance with Springer Nature Authorship Policy we agree to change the authors of the manuscript as indicated below.

NAME OF JOURNAL: Oncogenesis

TITLE OF MANUSCRIPT: Functional screening identifies aryl hydrocarbon receptor as suppressor of lung cancer metastasis

MANUSCRIPT NUMBER: ONCSIS-20-0064-R

CORRESPONDING AUTHORS NAME: Alexander Schramm

PREVIOUS AUTHOR NAMES:

Silke Nothdurft, Frank Breitenbücher, Ross A. Okimoto, Madeleine Dorsch, Christiane A. Opitz, Ahmed Sadik, Charlotte Esser, Michael Hölzel, Saurabh Asthana, Jan Forster, Sophie Kalmbach, Barbara M. Grüner, Trever G. Bivona, Alexander Schramm, Martin Schuler

UPDATED AUTHOR NAMES:

Silke Nothdurft, Clotilde Thumser-Henner, Frank Breitenbücher, Ross A. Okimoto, Madeleine Dorsch, Christiane A. Opitz, Ahmed Sadik, Charlotte Esser, Michael Hölzel, Saurabh Asthana, Jan Forster, Daniela Beisser, Sophie Kalmbach, Barbara M. Grüner, Trever G. Bivona, Alexander Schramm, Martin Schuler

CHANGE TO AUTHOR LIST:

Clotilde Thumser-Henner, Daniela Beisser

Print Name	Signature	Date
Michael Hölzel	M. Hölzel	July 8th, 2020

SPRINGER NATURE

In accordance with Springer Nature Authorship Policy we agree to change the authors of the manuscript as indicated below.

NAME OF JOURNAL: Oncogenesis

TITLE OF MANUSCRIPT: Functional screening identifies aryl hydrocarbon receptor as suppressor of lung cancer metastasis

MANUSCRIPT NUMBER: ONCSIS-20-0064-R

CORRESPONDING AUTHORS NAME: Alexander Schramm

PREVIOUS AUTHOR NAMES:

Silke Nothdurft, Frank Breitenbücher, Ross A. Okimoto, Madeleine Dorsch, Christiane A. Opitz, Ahmed Sadik, Charlotte Esser, Michael Hölzel, Saurabh Asthana, Jan Forster, Sophie Kalmbach, Barbara M. Grüner, Trever G. Bivona, Alexander Schramm, Martin Schuler

UPDATED AUTHOR NAMES:

Silke Nothdurft, Clotilde Thumser-Henner, Frank Breitenbücher, Ross A. Okimoto, Madeleine Dorsch, Christiane A. Opitz, Ahmed Sadik, Charlotte Esser, Michael Hölzel, Saurabh Asthana, Jan Forster, Daniela Beisser, Sophie Kalmbach, Barbara M. Grüner, Trever G. Bivona, Alexander Schramm, Martin Schuler

CHANGE TO AUTHOR LIST:

Clotilde Thumser-Henner, Daniela Beisser

Print Name	Signature	Date
Trever Bivona	Trever G Bivona	7/8/2020
Saurabh Asthana	Saurabh Asthana	7/8/2020

SPRINGER NATURE

In accordance with Springer Nature Authorship Policy we agree to change the authors of the manuscript as indicated below.

NAME OF JOURNAL: Oncogenesis

TITLE OF MANUSCRIPT: Functional screening identifies aryl hydrocarbon receptor as suppressor of lung cancer metastasis

MANUSCRIPT NUMBER: ONCSIS-20-0064-R

CORRESPONDING AUTHORS NAME: Alexander Schramm

PREVIOUS AUTHOR NAMES:

Silke Nothdurft, Frank Breitenbücher, Ross A. Okimoto, Madeleine Dorsch, Christiane A. Opitz, Ahmed Sadik, Charlotte Esser, Michael Hölzel, Saurabh Asthana, Jan Forster, Sophie Kalmbach, Barbara M. Grüner, Trever G. Bivona, Alexander Schramm, Martin Schuler

UPDATED AUTHOR NAMES:

Silke Nothdurft, Clotilde Thumser-Henner, Frank Breitenbücher, Ross A. Okimoto, Madeleine Dorsch, Christiane A. Opitz, Ahmed Sadik, Charlotte Esser, Michael Hölzel, Saurabh Asthana, Jan Forster, Daniela Beisser, Sophie Kalmbach, Barbara M. Grüner, Trever G. Bivona, Alexander Schramm, Martin Schuler

CHANGE TO AUTHOR LIST:

Clotilde Thumser-Henner, Daniela Beisser

Print Name	Signature	Date
Frank Breitenbücher	[signature]	08/07/2020

SPRINGER NATURE

In accordance with Springer Nature Authorship Policy we agree to change the authors of the manuscript as indicated below.

NAME OF JOURNAL: Oncogenesis

TITLE OF MANUSCRIPT: Functional screening identifies aryl hydrocarbon receptor as suppressor of lung cancer metastasis

MANUSCRIPT NUMBER: ONCSIS-20-0064-R

CORRESPONDING AUTHORS NAME: Alexander Schramm

PREVIOUS AUTHOR NAMES:

Silke Nothdurft, Frank Breitenbücher, Ross A. Okimoto, Madeleine Dorsch, Christiane A. Opitz, Ahmed Sadik, Charlotte Esser, Michael Hölzel, Saurabh Asthana, Jan Forster, Sophie Kalmbach, Barbara M. Grüner, Trever G. Bivona, Alexander Schramm, Martin Schuler

UPDATED AUTHOR NAMES:

Silke Nothdurft, Clotilde Thumser-Henner, Frank Breitenbücher, Ross A. Okimoto, Madeleine Dorsch, Christiane A. Opitz, Ahmed Sadik, Charlotte Esser, Michael Hölzel, Saurabh Asthana, Jan Forster, Daniela Beisser, Sophie Kalmbach, Barbara M. Grüner, Trever G. Bivona, Alexander Schramm, Martin Schuler

CHANGE TO AUTHOR LIST:

Clotilde Thumser-Henner, Daniela Beisser

Print Name	Signature	Date
MARTIN SCHULER	[signature]	08-07-2020
also on behalf of former lab members Dr. Sophie Kalmbach	[signature]	08-07-2020

SPRINGER NATURE

In accordance with Springer Nature Authorship Policy we agree to change the authors of the manuscript as indicated below.

NAME OF JOURNAL: Oncogenesis

TITLE OF MANUSCRIPT: Functional screening identif es aryl hydrocarbon receptor as suppressor of lung cancer metastasis

MANUSCRIPT NUMBER: ONCSIS-20-0064-R

CORRESPONDING AUTHORS NAME: Alexander Schramm

PREVIOUS AUTHOR NAMES:

Silke Nothdurft, Frank Breitenbücher, Ross A. Okimoto, Madeleine Dorsch, Christiane A. Opitz, Ahmed Sadik, Charlotte Esser, Michael Hölzel, Saurabh Asthana, Jan Forster, Sophie Kalmbach, Barbara M. Grüner, Trever G. Bivona, Alexander Schramm, Martin Schuler

UPDATED AUTHOR NAMES:

Silke Nothdurft, Clotilde Thumser-Henner, Frank Breitenbücher, Ross A. Okimoto, Madeleine Dorsch, Christiane A. Opitz, Ahmed Sadik, Charlotte Esser, Michael Hölzel, Saurabh Asthana, Jan Forster, Daniela Beisser, Sophie Kalmbach, Barbara M. Grüner, Trever G. Bivona, Alexander Schramm, Martin Schuler

CHANGE TO AUTHOR LIST:

Clotilde Thumser-Henner, Daniela Beisser

Print Name	Signature	Date
Jan Forster	Jan Forster	08.07.2020
